# Comparative efficacy analysis of anti-microbial peptides, LL-37 and indolicidin upon conjugation with CNT, in human monocytes

**DOI:** 10.1186/s12951-017-0278-1

**Published:** 2017-06-12

**Authors:** Biswaranjan Pradhan, Dipanjan Guha, Krushna Chandra Murmu, Abhinav Sur, Pratikshya Ray, Debashmita Das, Palok Aich

**Affiliations:** School of Biological Sciences, National Institute of Science Education and Research (NISER), HBNI, P.O. Bhimpur-Padanpur, Khurdha, Jatni, Odisha 752050 India

**Keywords:** Carbon nanotube, Antimicrobial peptides, Cationic peptides, Host defense peptides, Innate immunity

## Abstract

**Background:**

Antimicrobial peptides (AMPs) have the potential to serve as an alternative to antibiotic. AMPs usually exert bactericidal activity via direct killing of microbial pathogens. Reports have proposed that by harnessing innate immune activation, AMPs can regulate pathogen invasion and may control infection. It has been reported that AMPs could be utilized to activate the innate mucosal immune response in order to eliminate pathogenic infections. This way of controlling pathogen infection, by activating host immunity, confers the potential to the select AMPs to alleviate the problem of antibiotic resistance. Among various AMPs tested LL-37 and indolicidin, showed promise to be potential candidates for eliciting enhanced host innate immune responses. LL-37 and indolicidin had exhibited substantial innate immune activation in both human and murine macrophages. Dosage for each of the AMPs, however, was high with adverse side effects.

**Results:**

In this study, we reported that upon conjugation with carbon nanotubes (CNT), each AMP remained biologically functional at a concentration that was 1000-fold less than the dosage required for free AMP to remain active in the cells.

**Conclusions:**

Current study also revealed that while indolicidin induced signalling events mediated through the TNFRSF1A pathway in THP1 cells, followed by activation of NFκB and c-JUN pathways, treatment of cells with LL-37 induced signalling events by activating IL1R, with subsequent activation of NFκB and NFAT2. Thp1 cells, primed with CNT conjugated LL-37 or indolicidin, are protected against *Salmonella typhimurium* infection at 16 h post challenge.

**Electronic supplementary material:**

The online version of this article (doi:10.1186/s12951-017-0278-1) contains supplementary material, which is available to authorized users.

## Background

The antimicrobial activity of cationic peptides is mostly elicited via direct interaction with microbes [1, 2]. However, direct attack on microbes to attain anti-microbial effects is not a good strategy because microbes tend to develop resistance against antimicrobial agents over time. An alternative paradigm for prophylactic or therapeutic success would involve activating the innate immune system of the host through treatment with a sub optimal dosage of antimicrobial agents, rather than a direct attack on the microbes. This methodology could alleviate the possibility of microbes developing a resistance against antimicrobial agents. Keeping this logic in mind, we have compared the antimicrobial activities of two therapeutically potential antimicrobial peptides (AMPs), with potential and proven medicinal properties, LL-37 and indolicidin, in vitro.

LL-37 is a proven and potent AMP. LL-37 is of human origin [3]. LL-37 was first detected in leukocytes and in the testis of humans [4]. Subsequently, it was also found inside a large variety of cells, tissues and body fluids. LL-37 was initially recognized for its antimicrobial properties [5–7] against bacteria, fungi and viral pathogens [8, 9]. LL-37 neutralizes lipopolysaccharides [10, 11] because of its high affinity towards LPS [10]. LL-37 also plays a significant role in wound healing, angiogenesis and apoptosis [12]. Most importantly, recent studies suggest that it is also involved in the regulation of cancer [13].

Indolicidin, the other AMP used in the current study, belongs to the cathelicidin class of AMPs. Indolicidin purified from the cytoplasmic granules of bovine neutrophils. Indolicidin is capable of killing gram-negative bacteria by crossing the outer membrane and causing disruption of the cytoplasmic membrane by channel formation [14]. Indolicidin is also active against gram-positive bacteria, fungi, protozoa and enveloped viruses such as HIV-1 [15, 16]. Apart from direct neutralization of microbes, another important function of indolicidin is its ability to modulate the host innate immune system against infectious agents [17, 18]. Indolicidin exerts many immunomodulatory roles, including—but not limited to—chemotaxis, modulation of cytokine and chemokine expression, and leukocyte activation [19, 20]. Instead of utilizing the direct antimicrobial effects of indolicidin, its immunomodulatory properties could be exploited to facilitate pathogen clearance in the host. Interestingly, the concentration of indolicidin required to stimulate the innate immune system is comparable to its antimicrobial concentration of 10–20 µg/ml. This equivalence of concentration, for innate mucosal immunity activation and for antimicrobial activity, is a major concern to develop antimicrobial resistance. It is, therefore, urgently required to have a methodology to reduce the dosage required to modulate host innate immunity.

Both natural and synthetic AMPs have shown promise as ‘next generation antibiotics’ due to their unique mode of membranolytic action, which minimizes the development of microbial resistance. However, bacteria have evolved the following mechanisms to counteract AMPs: (i) by a transient induction of bacterial signalling systems that help the bacteria to cope with AMPs, and (ii) constitutive resistance as a result of genetic changes. Currently, there are several putative mechanisms known for bacterial resistance to AMPs [2, 21–24]. When AMPs are present at higher concentrations, bacteria modulate their cell surface by making it less negatively charged and less permeable [25–27].

Despite the apparent medical potential of AMPs, their activity is not clinically practical because of weak activity, nonspecific cytotoxicity and proteolytic effect on some host membrane proteins [28]. For example, indolicidin is cytotoxic for rat and human T-lymphocytes [29]. Also, in vivo studies have confirmed that indolicidin is toxic to erythrocytes [15] at a high concentration (10 µg/ml). Indolicidin’s immune modulatory efficacy with respect to concentration needs to be increased in order to avoid damage to the host and development of indolicidin resistance in bacteria. Previous studies have demonstrated that immune modulatory efficacy as well as delivery of CpG is enhanced when conjugated with nanoparticles [30–32]. Additionally, we have recently reported that conjugation of indolicidin with short multi-walled carbon nano-tubes (SM-CNT) enhanced the efficacy of indolicidin by increasing its ability to protect host cells from *Salmonella typhimurium serovar enterica* (ST) MTCC 3232 challenge [1].

In the present study, we have demonstrated that the comparative efficacy and in vitro functioning of LL-37 and indolicidin conjugated with SM-CNTs. We have studied the effects of free and nano-conjugated indolicidin treatment on the human monocyte cell line THP-1 through transcriptomics. We have also selected LL-37 for our current study as it has already been tested for various immune modulatory effects [33]. Our results revealed that following conjugation of LL-37 and indolicidin with SM-CNTs, the immune modulatory efficacy of LL-37 and indolicidin was significantly increased in vitro. Our results revealed that an effective level of activity for the peptides is maintained following CNT–conjugation even at a 1000-fold less dosage than free peptide.

## Methods

### Synthesis of CNT–indolicidin and CNT–LL-37

LL-37 was obtained from Prof. Bob Hancock, UBC, Canada as a gift and indolicidin was purchased from BR Biochem Lifesciences, India. Both AMPs were obtained as lyophilized powder. LL-37 and indolicidin were conjugated with CNT using EDC-NHS conjugation protocol as described elsewhere [34], which was described in our previous work reported with indolicidin [1]. LL-37 was conjugated using the same protocol 5 mg of LL-37 was suspended in 25 μl of DMSO. The resulting solution was mixed properly followed by further addition of 975 μl of PBS to make a 5 mg/ml peptide solution. This solution was used as the stock peptide solution for our experiment. 400 μl of the 1 mg/ml CNT solution, prepared earlier was put in a clean and sterile microfuge tube. To the above solution, 600 μl of MES buffer (pH = 5.0) used as the appropriate activation buffer was added. This is because activation of the carboxyl groups on the nanotubes using EDC and NHS is most efficient at pH = 4.5–7.2. 5 μl of 0.4 M EDC and 50 μl of 0.1 M NHS was added respectively and the solution was incubated in dark for 45 min at room temperature. Once the activation reaction is complete, 1.4 μl of 2-mercaptoethanol was added to quench the effect of EDC. 960 μl of PB (pH = 7.2) was added to 1 ml of the activated solution. The solution was mixed by gentle pipetting. PBS is used as the conjugation buffer. Therefore, after adding 40 μl of the stock 5 mg/ml peptide solution, the resulting solution was mixed thoroughly and incubated in dark for 2 h at room temperature. In addition, free LL-37 was diluted to the similar extent for proper comparison to the conjugates. Spike was prepared by adding same concentrations of LL-37 to a solution of non-activated CNTs. Free peptides were removed from the conjugate mixture using molecular weight cut-off spin columns (3 MWCO, Millipore, USA). Short multiwalled CNTs were purchased from Cheap tubes with outer diameter 8 nm and inner diameter of 2–5 nm and length between 500 and 2000 nm. Molecular weight of CNTs were calculated based on protocols mentioned before and on the homepage of Hipco [35, 36] assuming standard 0.14 nm of distance between C–C covalent bonds for the circumference and a hexagonal pack distance of 0.283 nm for weak long range interactions. Using these parameters and the value of total surface area as provided by the manufacturer, the average molecular weight determined was 2 × 10^6^. Stock concentration of CNT calculated was 50 μM.

### Physical characterization through Fourier transformed infrared (FTIR) spectroscopy

FTIR spectrum of free and conjugated AMPs along with positive controls was collected using Perkin Elmer FTIR model Spectrum RX1 equipment. Purified samples were lyophilized and prepared for FTIR measurement. FTIR measurements were performed at room temperature in the absorbance range from 4000 to 400 cm^−1^ by accumulating 20 scans with a spectral resolution of 1 cm^−1^. The data was normalized against potassium bromide spectrum, obtained from the same instrument under the same instrumental settings.

### Physical characterization through isothermal calorimetry

Isothermal calorimetry (GE Healthcare MicroCalTMiTC200) was used to investigate the potentiality of peptides to interact with free activated nanoparticles. 1 μl sample was injected at each time point (injection time 5 s) with a gap of 300 s between each injection, 40 such injections were carried out. The baseline setting was at 10 μcal. To get a steady baseline, a 2000 s delay was applied to the system. The resulting thermodynamic parameters related to the binding of the peptide to the carboxyl groups on the nanomaterials were obtained from the signal.

### Physical characterization through UV–Visible spectroscopy and isothermal calorimetry (ITC)

UV–Vis spectrophotometry was conducted using NanoDrop 2000 (Thermo-Scientific, USA) in the wavelength range of 200–550 nm. Concentration of peptide was evaluated spectrophotometrically and the stoichiometry of peptide–CNT conjugation was determined by Scatchard plot [1]. Thermodynamic parameters such as changes in free energy, enthalpy and entropy of peptide and CNT binding was determined by titrating the activated CNTs by the peptide using ITC as described before [1].

### Physical characterization through binding isotherm

Following activation of the carboxyl groups on free CNTs, carboxylated CNTs were titrated against increasing concentrations of the peptide. Change in absorbance at 260 nm was monitored till saturation of binding was observed. Concentration of CNT used was 5 μM for the titration. Binding isotherm for CNT–LL37 conjugation was determined using Scatchard plot to obtain the association constant and the stoichiometry of binding as described elsewhere for binding of CNT and indolicidin [1]. In addition, free non-activated nanoparticles were also titrated against increasing concentrations of LL-37 to ensure specificity of binding. Dissociation binding constant (K_d_) and Stoichiometry (B_max_) was determined using one, two and multiple site binding isotherm models [37]. All analyses were done using GraphPad Prism 5.01 software, CA, USA.

### Peptide and CNT conjugated peptide uptake assay through confocal microscopy

Peptide was labeled with Cy3 (GE HealthScience, USA). Cells were treated with free and conjugated labeled peptide at a concentration of 0.02 μg/ml in terms of peptide for 2 h following fixing of the cells with 2% paraformaldehyde. DAPI (Himedia, India) and cell mask red (Invitrogen, USA) were for nuclear and cell membrane staining. Cells were then mounted using fluoromount G (Southern Biotech, USA) and images were taken by a LSM confocal microscope (Carl Zeiss LSM 780, Germany).

### Animal cell culture

The Raw 264.7 murine macrophage and THP-1 human primary monocyte cell lines were obtained from ATCC (Manassas, VA). RPMI-1640 (Himedia, India) supplemented with 4.5 g/l d-glucose, 25 mM HEPES, 0.11 g/l sodium pyruvate, 1.5 g/l sodium bicarbonate, 2 mM l-glutamine and 10% FBS along with 100 units/ml Gentamycin and 100 pg/ml Amphotericin-B was used to maintain both the cell lines. PMA (phorbol-12-myristate-13-acetate) of 100 nM was used for THP-1 cell differentiation into adherent macrophages. Both cell lines were routinely cultured in our laboratory at 37 °C in a humidified atmosphere containing 5% CO_2_. The cells were sub-cultured twice a week to maintain an exponential growth state.

### Cell treatment

One million macrophage cells per well in 2 ml cell culture media were grown in 6 well plates overnight. Free AMP was administered to the cells at a final concentration of either 0.02 or 20 μg/ml. CNT conjugated AMPs were used at 0.02 μg/ml to treat the cells. Control treatment used free CNT and CNT spiked with AMP. Following treatment, the cells were incubated for 6 h in a CO_2_ incubator. Following 6 h of incubation, culture medium was aspirated off and the cells were washed 3 times with PBS. Cells were trypsinized and suspended in cell culture medium and centrifuged at approximately 300*g* for 5 min at 37 °C. The cell pellet was collected to execute further experiments.

### RNA isolation

Total RNA was extracted from treated and un-treated THP-1 cells using RNeasy mini kit (Qiagen #74106, Germany) following manufacturers protocol. In brief, cells were gently lysed with 350 µl RLT buffer by gentle pipetting and then equal volume of 70% ethanol was added to the lysate, mixed by pipetting and passed through RNeasy mini column, which retains the RNA in its silica matrix. The column was then washed once with 750 µl RW1 buffer and twice with 500 µl of RPE buffer to remove unwanted lipid, protein and DNA from the matrix. RNA was then eluted from the matrix with 30 µl nuclease free water and kept in ice. The concentration of extracted RNA was measured using NanoDrop 2000 instrument (Thermo Scientific, USA). RNA integrity was checked in bioanalyzer 2100 (Agilent, USA) using an RNA 6000 Nano kit (Agilent, USA) as per the manufacturer’s instruction. RNA integrity number 8.5 or more was considered for downstream experiments, which included quantitative real time polymerase chain reaction (qRT-PCR) and whole genome gene expression microarray.

### Complementary DNA (cDNA) synthesis

cDNA was synthesized from total RNA using reverse transcription methodology as described here briefly and detailed protocoled can be obtained from reports published before [38]. 5 µg of total RNA was mixed with the buffer containing affinity script reverse transcriptase and polyT primer. The mixture was kept in the thermo cycler at 45 °C for 45 min to synthesize c-DNA. Next, the temperature was raised to 92 °C for 1 min in order to deactivate the enzyme.

### Quantitative real time (qRT-) PCR assay

The qRT-PCR was performed using GoTaq qPCR Kit (Promega #A6002, USA) using the manufacturer protocol and the expression profile of select innate immune genes in terms of fold changes for the treatments with respect to untreated samples was checked. The reaction mixture was 25 μl in each well of a 96-well plate. According to the protocol, 9.4 μl of 2× GoTaq qPCR Master Mix, 12.6 μl of nuclease free water, 100 ng of template cDNA and 1 μM of each of forward and reverse primers (primer details are given in Additional file 1: Table S1) were added in each well. qRT-PCR amplification was performed in a programmable thermos-cycler (Stratagene 3500Mxp, USA) with the following settings: 2 min at 92 °C to activate DNA polymerase for 1 cycle, 15 s at 92 °C for melting and 1 min at 60 °C for primer annealing along with extension of the chain and detection of the florescence for 40 cycles. Cycle threshold (C_t_) values were noted, and fold changes of the desired genes were calculated with respect to the control after normalizing with the housekeeping gene, β-actin.

### Bacterial protection assay

PMA treated 0.5 × 10^6^ differentiated THP-1 cells were seeded into each well of 12-well plate and incubated at 37 °C for 24 h. Cells were treated with LL-37 and indolicidin separately at a final concentration of 20 and 0.02 μg/ml; whereas conjugated peptides were administered at a lower dose of 0.02 μg/ml. After treatment, plates were incubated for 6 h at 37 °C. Thereafter, THP-1 cells were challenged with ST, a pathogenic bacterium, at a multiplicity of infection (MOI) of 10. Cell viability counts at different time points of 6, 12 and 18 h were determined through trypan blue dye exclusion method.

### Microarray and data analysis

Genome wide gene expression study was performed using Agilent Quick-Amp labeling Kit (p/n 5190-0444 Agilent, USA). 500 ng of each RNA samples from the control and treated cells were incubated with reverse transcription mix at 40 °C and converted to cDNA primed by oligodT with a T7 polymerase promoter. cDNA synthesized was used as a template for cRNA generation. cRNA was synthesized by in vitro transcription and the dyes used were Cy3 CTP (to label control sample) and Cy5 CTP (to label test samples). The cDNA synthesis and in vitro transcription steps were carried out at 40 °C. Labeled cRNA was cleaned up and quality assessed for the yields and specific activity. 825 ng each of Cy3 and Cy5 labeled samples were fragmented and hybridized to 4 × 44 k microarray slides. Fragmentation of labeled cRNA and hybridization were conducted using the Gene Expression Hybridization kit of Agilent (Part Number 5188–5242, Agilent, USA). Hybridization was carried out in Agilent’s Surehyb Chambers at 65 °C for 17 h. The hybridized slides were washed using Agilent Gene Expression wash buffers (Part Number 5188–5327, Agilent, USA). Slides were scanned using Agilent scan control software and data extraction from images was done using feature extraction software Version 10.7 (Agilent, USA). We further obtained normalized fold change values for all genes present on the microarray slides using Arraypipe (v2.0). Differentially regulated genes at various conditions were functionally clustered using WEB-based GEneSeT AnaLysis Toolkit (Webgestalt).

### Graphs and statistical analysis

All graphs and statistical analysis were carried out using GraphPad Prism (V5.04, Prism, USA). Statistical analysis was performed using 2-way ANOVA to calculate levels of significance. One standard deviation was calculated and shown in the graphs.

## Results

### Characterization of CNT conjugates

**Fig. 1:**
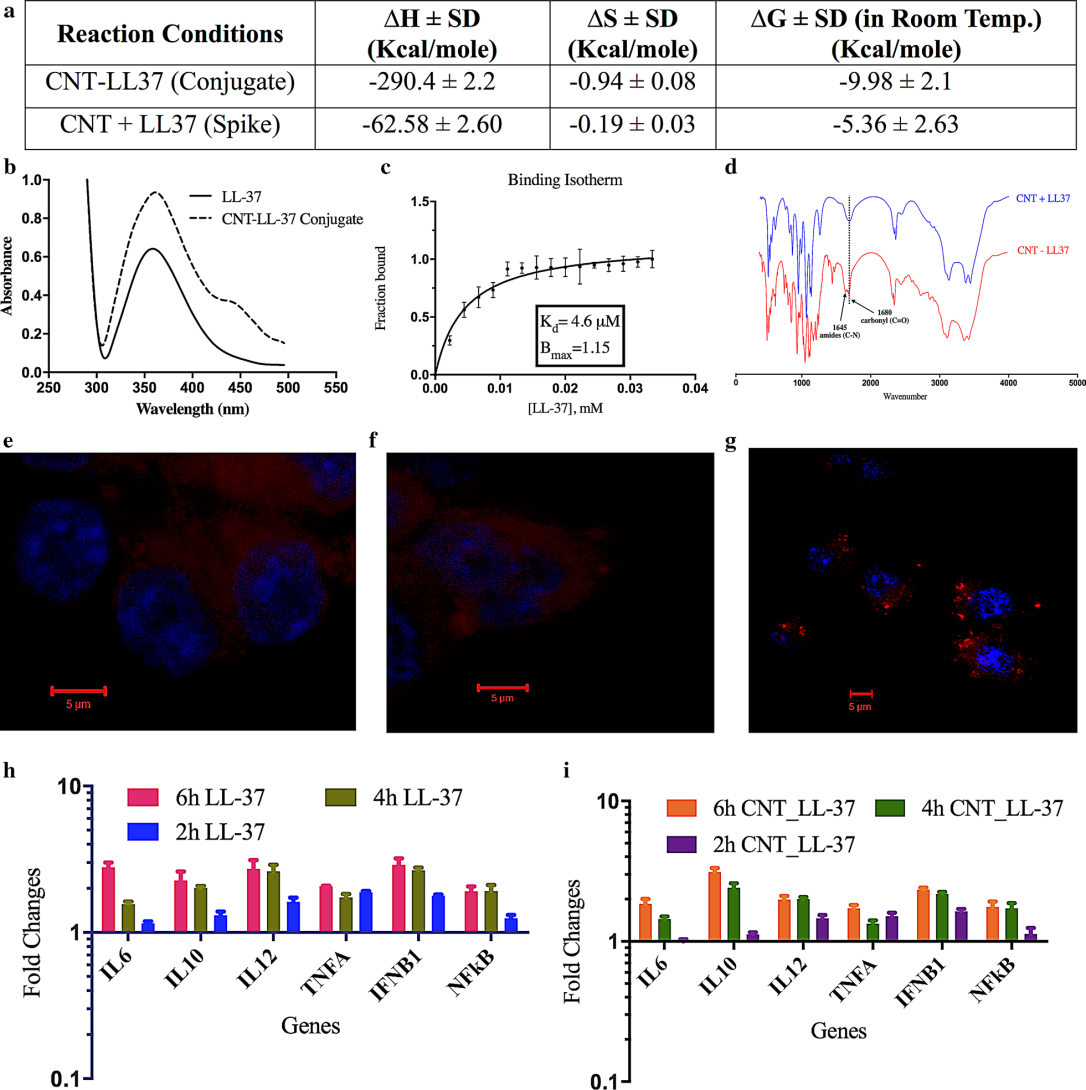
Characterization of CNT–peptide conjugate and its uptake by the macrophage cellls. Characterization of conjugation of CNT and LL-37 using **a** isothermal calorimetry, **b** UV–visible spectroscopy, **c** binding isotherm plot, **d** FT-IR spectrum. Uptake studies of Cy3 labeled free (**f**) and CNT–conjugated peptide (**g**) by THP-1 cells using confocal microscopy following 2 h of treatment. Untreated unlabelled control image is shown in **e**. Kinetics of gene expression of a few innate immune genes in THP-1 cells following treatment with free LL-37 at 20 μg/ml (**h**) and CNT–LL37 at 0.02 μg/ml (**i**). *Error bars* shown are representative of ±1 SD

The conjugation process and characterization for SM-CNT and indolicidin was already reported elsewhere by the current group [1]. Similar methodologies for conjugation were utilized for LL-37 followed by biophysical characterization using UV–Vis spectroscopy, Binding isotherm, isothermal calorimetry and FT-IR spectroscopy. Conclusive characterization was achieved using FT-IR analysis. Peaks, associated with carbonyl (C=O) stretching at 1680 cm^−1^ and amide (C–N) bending at 1645/cm appeared in CNT–LL-37 conjugated AMPs but not in free AMPs, confirmed formation of a peptide bond between SM-CNT and LL-37 (Fig. 1d). Free AMP when spiked with SM-CNT did not result into any such peak.

Isothermal calorimetry results revealed that SM-CNT and LL-37 conjugation process was exothermic with enthalpy (∆H) at −290.4 ± 2.2 kcal/mol and free energy change (∆G) −9.98 ± 2.1 kcal/mol. Free LL-37 when spiked with SM-CNT leads to enthalpy (∆H) at −62.58 ± 2.6 kcal/mol and free energy change (∆G) −5.36 ± 2.63 kcal/mol (Fig. 1a). We further established the strength and stoichiometry of binding. Fraction bound with increasing concentration of the peptides was determined by spectrophotometric titration for association of LL-37 with CNT (Fig. 1b, c). Dissociation constant (K_d_) for the LL-37 and CNT binding was 4.6 μM with a stoichiometry of binding (B_max_) value of 1.15 results from binding isotherm analysis (Fig. 1c). The binding constants determined also corroborated with the concentrations used in the experiment. There was no significant association observed when non-activated CNT was mixed with LL-37 (spiked samples). This observation was further validated by thermodynamic parameters described above. The CNT conjugated peptide was internalised by the macrophages (Fig. 1g) whereas the free peptide was diffused all over the macrophage cell membrane (Fig. 1f).

### Cell viability of THP-1 cells treated with free and conjugated LL-37 and indolicidin

**Fig. 2:**
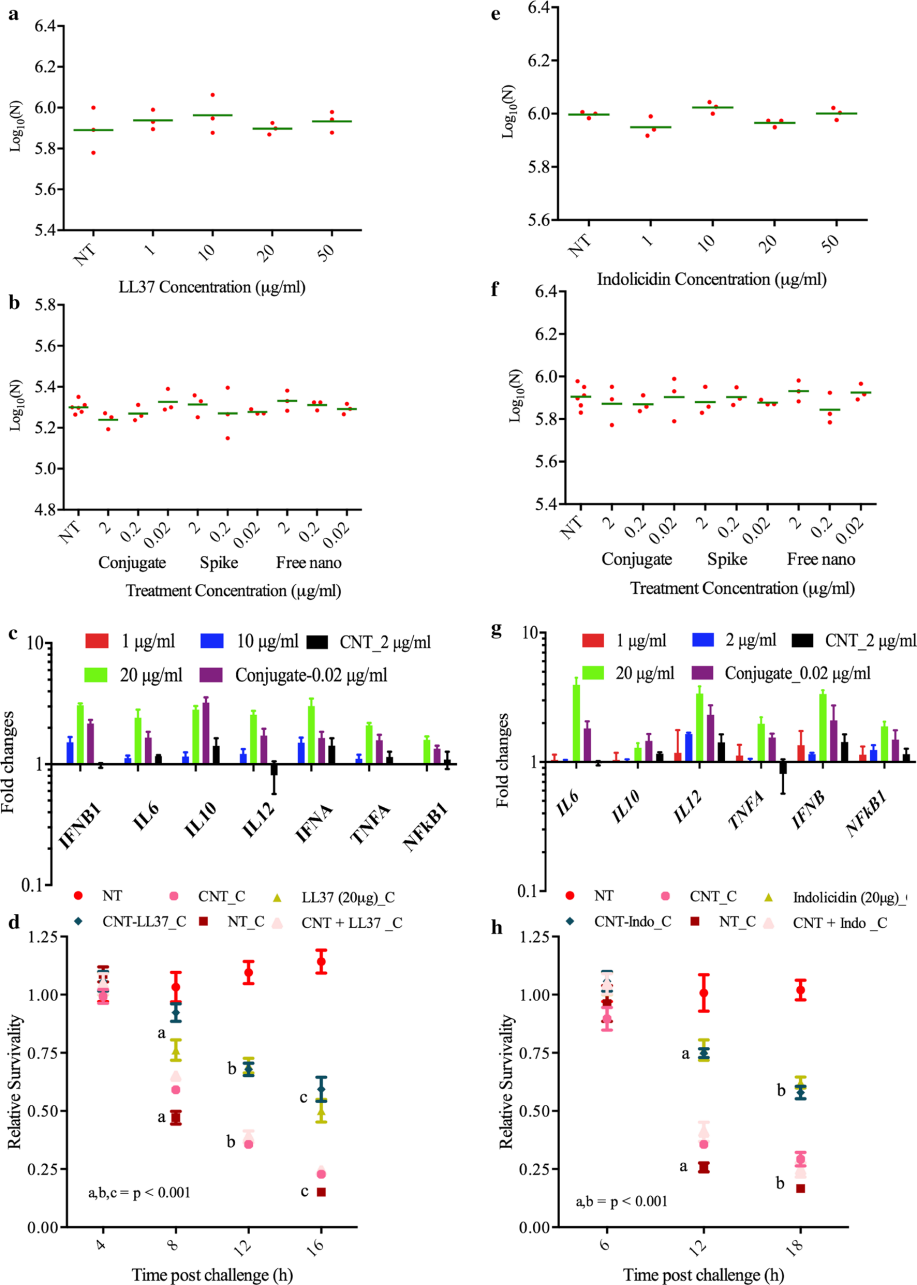
Viability of nano conjugated HDPs followed by gene expression modulation and protection of cells against *Salmonella* challenge. Viability of THP_1 cells following treatment with various concentrations of **a** LL-37, **b** CNT, CNT conjugated LL-37 and LL-37 spiked CNT, **e** indolicidin and **f** CNT, CNT conjugated indolicidin and indolicidin spiked CNT. Expression of select innate immune genes in Thp1 cells following treatment with **c** LL-37 and CNT conjugated LL-37 and **g** indolicidin and CNT–conjugated indolicidin. Relative survivability of THP-1 cells being challenged by *Salmonella* in the absence or presence of **d** free LL-37 at 20 µg/ml or CNT conjugated LL-37 at 0.02 µg/ml and **h** free indolicidin at 20 μg/ml or CNT conjugated indolicidin at 0.02 μg/ml. Significant changes with p ≤ 0.001 at each time point is shown in *letters*

Before establishing the efficacy of CNT conjugated AMPs, it is important to check viability of the cells following treatment with nano-conjugated peptides. Viability of the human macrophage cell line THP-1 and mice macrophage cell line Raw 264.7 were determined up to 6 h following treatment with free and CNT conjugated AMPS (LL-37 and indolicidin). Macrophage cells were viable for entire 6 h following treatment with free and conjugated AMPs (Fig. 2a, e). Longer than 6 h time point was not chosen since, (a) our goal is to prime early immune response and (b) 4 h was shown as sufficient to exhibit immune modulation in vitro [39]. For free AMPS highest concentration reported was 50 μg/ml while that for conjugated AMP was 2 μg/ml. Effects of untreated as well as free AMPS spiked with equivalent amount of CNTs as control groups of treatment were also evaluated. Experimental data revealed that none of the above treatments were toxic to the macrophage cell lines THP-1 (Fig. 2b, f). Similar results were also observed in Raw 264.7 (data not shown).

### Expression of selected innate immune genes

Objective, of the current work, is to understand potential of LL-37 and indolicidin in modulating expression of innate immune genes. Following the establishment of AMP toxicity to cell lines, we, therefore, evaluated the effects of AMP treatment on select innate immune gene expression to understand AMPs potential as an immune stimulant that facilitates antimicrobial activity. Expression kinetics of few select innate immune genes in THP-1 cells following peptide treatment at 20 μg/ml and CNT conjugated peptide at 0.02 μg/ml revealed that, optimal expression of genes occurred at 6 h (Fig. 1h, i). Expression values for a few innate immune genes at the transcriptional level in THP-1 cells following 6 h treatment with free LL-37 at concentrations of 1, 10 and 20 µg/ml were determined by qRT-PCR. Experimental data revealed that with increasing concentration of LL-37, the expression at a transcriptional level for the genes *IFNB1*, *IFNA*, *IL6*, *IL10*, *IL12*, *TNFA* and *NFκB1* increased with respect to time matched untreated controls (Fig. 2c). When the expression of the above-mentioned genes were calculated in THP-1 cells at 6 h following treatment with 0.02 µg/ml of conjugated LL-37, it was observed that the expression of these genes was similar (1-way ANOVA, p ≥ 0.05, n = 3) in comparison to free LL-37 which was treated with a higher dose of 20 µg/ml (Fig. 2c). Similarly, with increasing dose of free indolicidin, the above mentioned innate immune gene expressions increased with respect to time matched untreated control (Fig. 2g). Statistical analysis using 1-way ANOVA (p ≥ 0.05, n = 3) confirms that the gene expression pattern was almost similar in 20 µg/ml indolicidin treated cells with respect to 0.02 µg/ml indolicidin conjugated cells (Fig. 2g). It is clear, that, both of the conjugated peptides can induce controlled up-regulation of select innate immune genes at a lower dosage (1000 fold less) than that of the free peptides.

### Protection against ST challenge

When innate immune genes are moderately (fold changes up to 5) up-regulated in macrophages, we defined the macrophages as primed [38]. Primed macrophages have the ability to combat bacterial pathogens more efficiently than naive macrophages. To prove this hypothesis, free LL-37 and indolicidin, as well as conjugated AMPs primed THP-1 cells, were exposed to ST at MOI of 10. Results revealed that macrophage cells primed with free LL-37 at 20 µg/ml and CNT–LL-37 at 0.02 µg/ml were significantly (2-way ANOVA, p ≤ 0.001, n = 12) protected against ST challenge with respect to the unprimed cells (Fig. 2d). Primed cell survivability was found to be 80, 65 and 55%, whereas, unprimed cell survival was 48, 36 and 14% at 8, 12 and 16 h post challenge. Similarly, THP-1 cells primed with free indolicidin at 20 µg/ml and CNT–indolicidin at 0.02 µg/ml were significantly (2-way ANOVA, p ≤ 0.001, n = 12) protected against ST infection. The survivability of primed cells was found to be 75 and 55%, whereas, the survival of unprimed cells was 28 and 17% at 12 h and 18 h post challenge (Fig. 2h). The results from our pathogenic challenge study revealed that conjugating LL-37 and indolicidin with SM-CNT increased their immune modulatory efficacy by 1000 folds. However, the exact mechanism through which priming occurs is yet to be elucidated. Therefore, we conducted genome wide transcriptional gene expression microarray studies to understand the plethora of genes that could be responsible for priming.

### Genome wide gene expression to elucidate transcriptional pathway biology in vitro

**Fig. 3:**
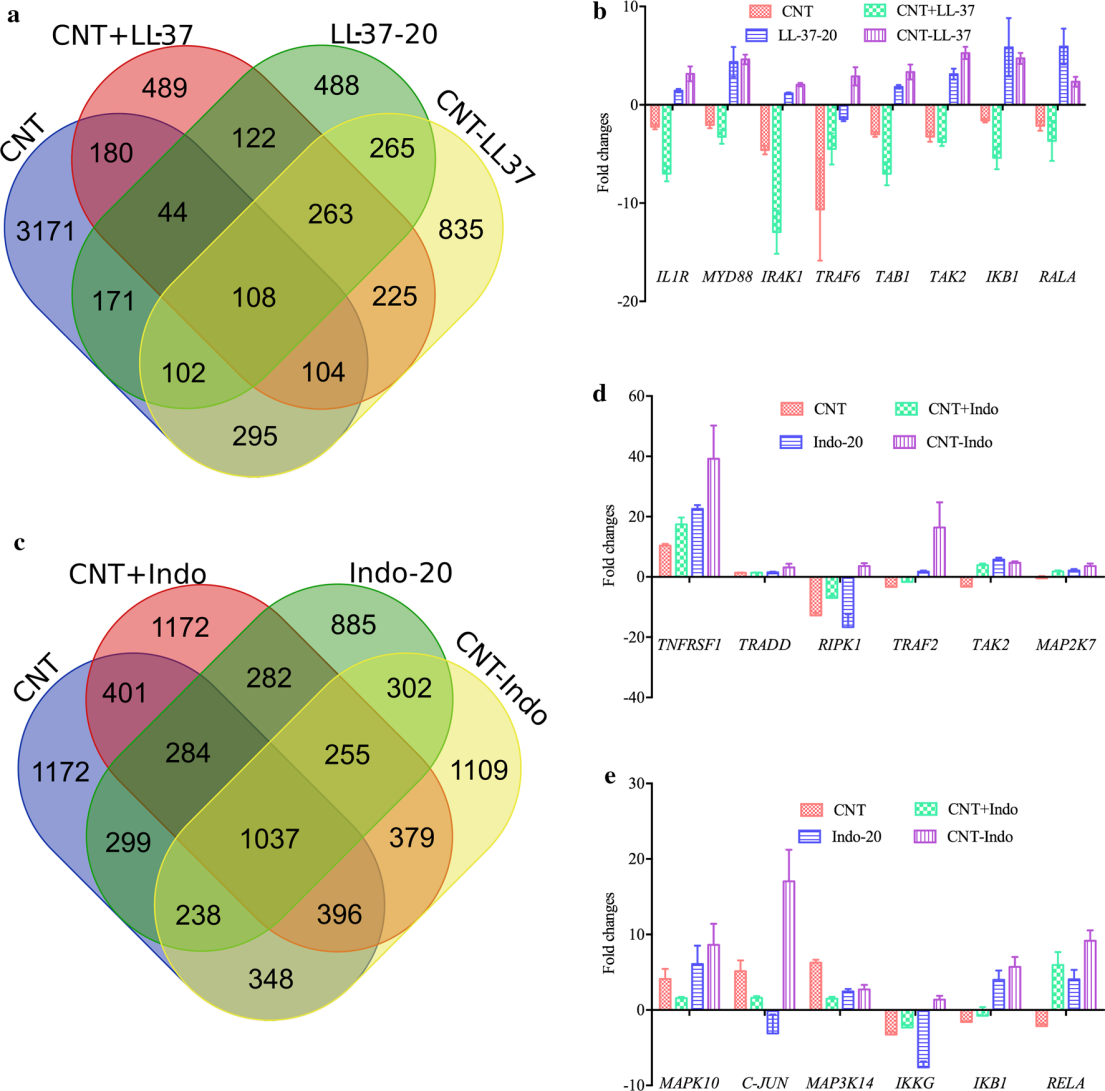
Venn diagram of genome wide gene expression at various treatments conditions and expression profile of select genes. Venn diagram is showing number of differentially expressed (up- or down-regulated) genes following 6 h treatment of Thp1 with **a** LL-37, CNT–LL-37, CNT and CNT + LL37 and **c** indolicidin, CNT–indolicidin, CNT and CNT + indolicidin. Validation microarray data by qRT of a few important and select genes of **b** IL1 signaling pathway in THP-1 cells following treatment with free and conjugated or spiked LL-37 and, **d**, **e** TNF signaling pathway in THP-1 cells following treatment with free, nano-conjugated or spiked indolicidin

We performed experiments with a view to understanding genome wide transcriptomic profiling by studying gene expression changes in THP-1 cells following treatment with either unconjugated LL-37 or indolicidin at 20 µg/ml or CNT conjugates at 0.02 µg/ml of either conjugate. For control studies, cells were also treated with free CNT or free AMP or AMPs spiked with SM-CNT at their respective conjugate concentrations. The genes were considered to be differentially expressed and statistically significant, if fold changes were ≥1.5 with p ≤ 0.05. There were total of 3784, 1535, 2197, 1563 genes that were differentially expressed in THP-1 cells following 6 h treatment with CNT, CNT + LL-37, CNT–LL-37 and LL-37-20 respectively (Fig. 3a). Out of which 3171, 489, 488, 835 genes were unique in THP-1 cells at 6 h following treatment with CNT, CNT + LL-37, CNT–LL-37 and LL-37-20. Similarly, a total of 3784, 2446, 2221 and 2015 genes were differentially expressed in THP-1 cells at 6 h following treatment with CNT, CNT + indolicidin, CNT–indolicidin and indolicidin-20 respectively (Fig. 3c). Out of these differentially expressed genes, 1172, 1172, 885 and 1109 were uniquely expressed at 6 h following treatment with CNT, CNT + indolicidin, CNT–indolicidin and indolicidin-20.

The genes were clustered using GeneAnalytics to find out the biological pathways that are populated most. The top 5 pathways related to innate immune signalling and cell cycle regulation are listed in Table 1. Innate immune signalling was enriched with 90 genes in THP-1 cells treated with free indolicidin as well as CNT conjugated indolicidin. There are 46 genes enriched with infectious disease signalling in the THP-1 cells treated with free LL-37 as well as conjugated LL-37. LL-37 also activates TGFβ signaling in THP-1 cells, which indicates feedback suppression of inflammatory pathways. The expression levels of these enriched genes are comparable in free peptide as well as CNT conjugated peptide treatments, but at 1000 fold less concentration (Additional file 2: Table S2, Additional file 3: Table S3). Important genes with their expression level and function are listed in Table 2 (for LL-37) and in Table 3 (for indolicidin). The gene expression profile revealed that conjugated AMPs show similar effects as free AMPs, but at a 1000-fold lower concentration (Tables 2, 3). It was also observed that when indolicidin spiked with CNT, the gene expression profile was better than free peptide at 0.02 μg/ml but we did not find this phenomenon in case of LL-37. This may be due to indolicidin stacking over the CNT surface via π electron cloud overlap of both the substances; increasing the effectiveness of indolicidin delivery into the cell through the added hydrophobicity from CNT. However, more study needs to be done to confirm this phenomenon.

**Table 1:** Top 5 enriched pathways in THP-1 following 6 h treatment with LL-37 and indolicidin

LL-37		Indolicidin	
Pathways	Enriched gene number	Pathways	Enriched gene number
AKT signaling	63	Innate immune system	90
Infectious diseases	46	MAPK signaling	54
IGF1R signaling	20	AKT signaling	55
TGFB signaling	20	TNF signaling	21
Cell cycle	22	Chemokine signaling	26

**Table 2:** Important genes differentially expressed in THP-1 following 6 h treatments with LL-37

Gene symbol	Fold changes WRT NT				Entrez ID	Function
	CNT	LL37-20	CNT–LL37	CNT + LL37		
TSG101	2.1	1.0	6.8	1.0	7251	Acts as a negative growth regulator
GRB2	1.5	1.0	5.2	1.0	2885	Links cell surface GFRs and the Ras signaling pathway
IL9R	1.0	2.6	2.6	2.5	3581	Interleukin-3, 5 and GM-CSF signaling
MAP4K3	1.0	1.7	6.2	1.0	8491	MAPK signaling pathway and TNF signaling
CFLAR	1.0	1.0	6.3	1.0	8837	Acts as an inhibitor of TNFRSF6 mediated apoptosis
ENPP1	3.0	1.0	7.0	1.0	5167	Appears to modulate insulin sensitivity and function
RALBP1	1.0	3.1	4.5	−2.6	10928	Can catalyze transport of glutathione and xenobiotics
SUCLA2	−1.8	4.0	5.6	4.8	8803	Catalyzes succinyl-CoA production
ALOX5	1.0	1.0	6.0	1.0	240	Catalyzes leukotriene biosynthesis and inflammation
MAOB	1.0	4.6	4.3	1.0	4129	Oxidative deamination of biogenic and xenobiotic amines
CCL20	5.5	9.2	10.1	1.0	6364	Chemotactic to lymphocytes and neutrophils. Possesses antibacterial activity *E. coli* and *S. aureus*
IL33	1.0	1.0	3.5	1.0	90865	Activates NF-kappa-B and MAPK signaling pathways
IL36G	1.0	2.9	3.8	2.7	56300	Activates NF-kappa-B and MAPK signaling pathways
SLC2A14	1.0	1.0	4.1	3.3	144195	Facilitative glucose transporter
DEFB105B	1.0	1.0	2.8	1.0	504180	Has antibacterial activity
DEFA5	1.0	1.0	5.0	1.0	1670	Antimicrobial activity against broad spectrum bacteria
EFR3A	−25.4	1.0	7.3	1.0	23167	Signaling through PIP3K and G protein couples receptors
PLA1A	1.0	6.2	5.2	1.0	51365	Stimulate histamine production
INO80B	1.0	1.0	2.8	1.0	83444	Cell cycle arrests at the G1 phase of the cell cycle
TLE1	1.0	3.8	5.8	1.0	7088	Inhibits NF-kappa-B-regulated gene expression
SKP2	1.0	2.3	7.1	1.0	6502	involved in regulation of G1/S transition
NLRC4	1.0	1.0	2.9	1.0	58484	Senses specific proteins from pathogenic bacteria and fungi and responds by assembling an inflammasome complex
BTG3	1.0	5.7	6.1	1.0	10950	Blocks cell cycle at G0/G1 to S phase
SLC22A15	5.6	1.0	9.5	9.0	55356	Probably transports organic cations
IL11RA	1.0	3.6	3.2	1.0	3590	Involved in macrophage proliferation and differentiation
OGFOD2	1.0	4.9	6.9	5.4	79676	Iron ion binding and oxidoreductase activity
IL17RE	1.0	1.0	3.9	3.8	132014	crucial regulator in innate immunity to bacterial pathogens
CCNE2	1.0	4.7	4.5	3.2	9134	Blocks cell cycle at the G1-S phase
IRF1	1.0	1.0	4.7	1.0	3659	Regulation of IFNs against viral and bacterial infections

**Table 3:** Important genes differentially expressed in THP-1 following 6 h treatments with indolicidin

Gene symbol	Fold changes WRT NT				Entrez Gene ID	Gene functions
	CNT	CNT + Indo	CNT–Indo	Indo-20		
TNFRSF1A	6.5	42.3	29.0	33.2	7132	Activate NFkB, mediated regulator of inflammation
RBCK1	3.4	10.9	6.9	1.0	10616	Activation of canonical NFkB and the JNK signaling
SLC11A1 & SLC5A5	4.4	6.1	6.9	4.3	6556	Transport of glucose and other sugars, bile salts and organic acids, metal ions and amine compounds
	2.2	5.0	8.7	1.8	6528	
ENPP7	5.1	12.1	16.3	8.5	339221	Converts sphingomyelin to ceramide
SOSTDC1	2.8	2.5	5.0	2.5	25928	Enhances Wnt and inhibits TGF-beta signaling
XPR1	1.0	1.6	47.6	−1.9	9213	G-protein coupled receptor activity
S100A5	69.2	67.4	150.4	113.5	6276	Helps in cell cycle progression and differentiation
NAPEPLD	8.0	25.7	22.6	17.7	222236	Responsible for the generation of anandamide, the ligand of cannabinoid and vanilloid receptors
RGS11	2.1	3.0	5.9	1.9	8786	Inhibits signal transduction by G Protein
RGS6	1.0	−1.6	5.9	1.5	9628	Inhibits signal transduction by G protein
RUSC1	1.1	−1.0	5.7	1.6	23623	Activation of the NFkB pathway
IBA57	2.5	4.0	4.9	1.0	200205	Activates iron-sulfur cluster assembly pathway
MYO5B	1.0	−1.3	13.7	−1.0	4645	Vesicular trafficking with the CART complex
AOX1	2.8	2.7	4.7	2.6	316	Regulation of reactive oxygen species homeostasis
RIPK4	3.7	7.3	6.9	1.4	54101	Plays a role in NF-kappa-B activation
FILIP1L	2.0	10.6	14.0	2.2	11259	Leads to inhibition of cell proliferation and migration
NCOA4	2.4	5.9	10.6	5.8	8031	Co-activator of the PPARG
CREB3L3	1.7	7.0	4.5	4.9	84699	Linked to acute inflammatory response
RASSF2	6.5	6.7	12.2	7.3	9770	May promote apoptosis and cell cycle arrest
DAP	2.8	1.7	7.0	2.7	1611	Negative regulator of autophagy
RELA	−1.2	1.7	8.4	−1.2	5970	NFkB pleiotropic transcription factor
CCL20	5.5	1.5	10.9	11.0	6364	Antibacterial activity against *E. coli* and *S. aureus*
LYRM4	3.3	3.4	6.2	2.2	57128	Nuclear and mitochondrial FE-S protein biosynthesis
ZDHHC22	17.0	41.5	44.6	44.4	283576	Feedback regulator of calcium mediated signaling
CYP3A5	3.7	3.6	6.9	3.7	1577	Oxidizes steroids, fatty acids, and xenobiotics

**Fig. 4:**
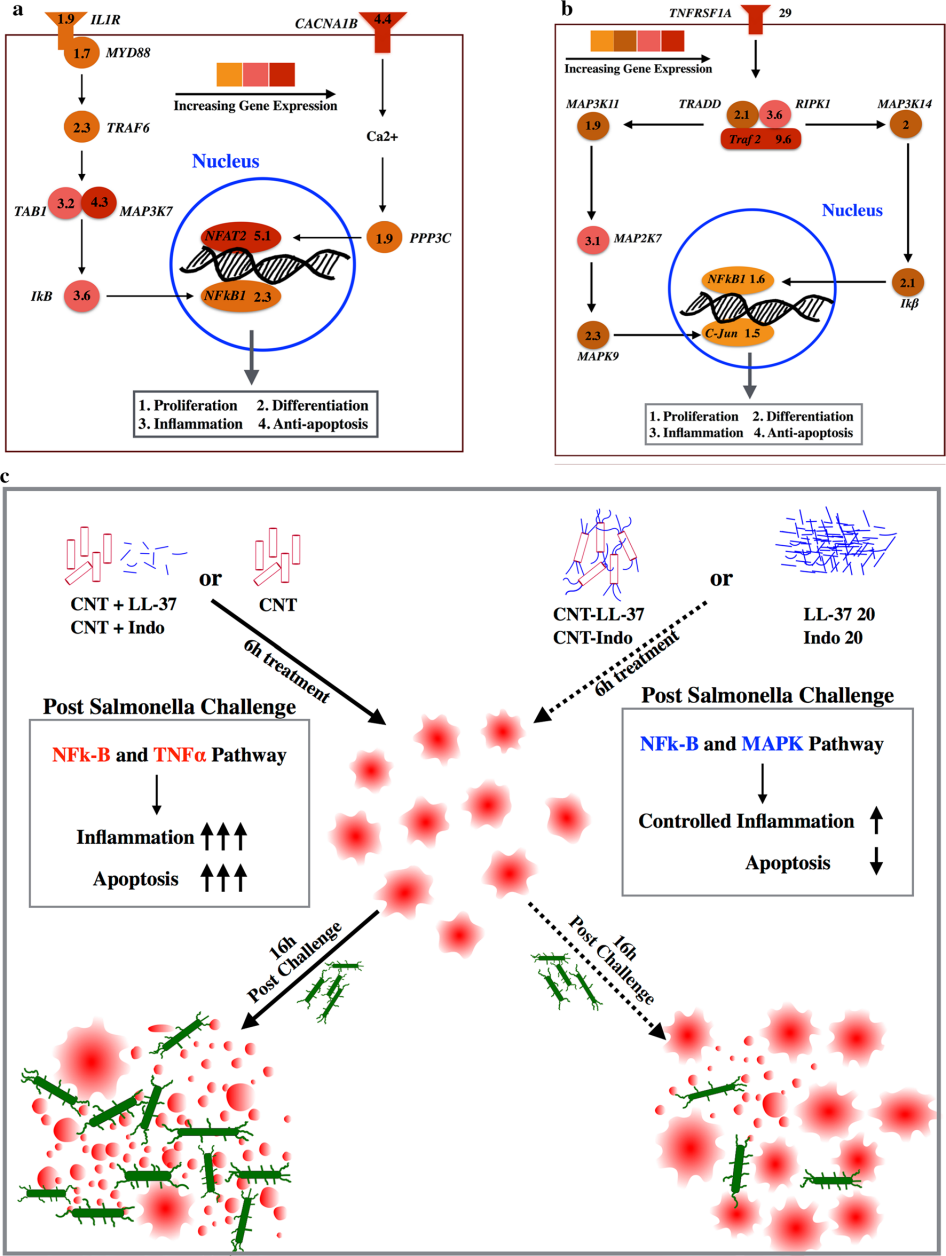
Pathways that were indicated by genome wide microarray data and validated by qRT-PCR results following treatment with nano-conjugated LL-37 and indolicidin. Activation of **a** IL1 pathway in THP-1 cells following treatment with conjugated LL-37 at 0.02 µg/ml, **b** TNF pathway via TNFRSF1A in THP-1 cells following treatment with conjugated indolicidin at 0.02 µg/ml. **c** Graphical summary of the consolidated schema of the current study depicting how LL-37 and indolicidin primed Thp1 cells to protect against *Salmonella* infection

We have tried to populate the pathways with important innate immune genes which were differentially expressed following treatment with both of the conjugates. From the list of differential gene expression, we searched for receptors, adaptors, kinases and transcription factors which are related to immune signaling and match them with the KEGG pathways. The genes of NFκB1 pathway and its downstream genes are up-regulated in THP-1 cells following LL-37 and CNT–LL-37 treatment. It was also observed that interleukin 1 receptor and its subsequent adaptors and kinases such as Myd88, Traf6 and Map3k7 was up-regulated in THP-1 cells following LL-37 and its CNT conjugate treatment. From this it may be inferred that LL-37 signals through the interleukin 1 receptor (IL1R) followed by nuclear factor kappa B1 (NFκB1) translocation to nucleus with subsequent transcription of pro-inflammatory cytokines, chemokines and defensins. We also observed the expression of genes related to cell proliferation and differentiation along with up-regulation of calcium transporter (CACNA1B), protein phosphatase 3 catalytic subunit alpha (PPP3CA) and NFAT2. It may be inferred that calcium release to cytoplasm through CACNA1B activated PPP3CA which in turn dephosphorylate the transcription factor NFAT2 which is subsequently translocated to the nucleus to transcribe genes related to cell proliferation and differentiation. The above pathway is represented in Fig. 4a. The genes involved in this pathway were also validated through qRT-PCR and represented with their fold changes with respect to untreated time matched controls in Fig. 3b.

It was also observed that many genes related to pro-inflammation, cell proliferation and cell differentiation were significantly up-regulated in THP-1 cells following treatment with indolicidin and CNT conjugated indolicidin. Tumor necrosis factor receptor 1a (TNFSF1A), MAP3K11, MAP3K14, NFκB and c-JUN are up-regulated in THP-1 cells following treatment with indolicidin. From this gene expression data, it may be inferred that TNFRSF1A signals through MAP3K11 and MAP3K14 followed by activation of transcription factors NFκB1 and c-JUN. Eventually the genes related to cell proliferation, differentiation and pro-inflammation get activated in THP-1 cells. This pathway was represented schematically in Fig. 4b and the genes shown were validated through qRT-PCR and plotted with their fold changes with respect to untreated time matched controls in Fig. 3d, e.

## Discussion

Cationic AMPs are non-toxic to cells up to a concentration of 50 μg/ml [40]. Our findings corroborate with this pre-established level of AMP toxicity. Our results revealed that following LL-37 and indolicidin treatment up to 50 μg/ml, human and mice macrophage cells show no toxic effects (Fig. 2a, e). The immune modulatory properties of AMPs include modulating pro- and anti-inflammatory responses through [41] various signaling pathways [40] directly [42] or indirectly [43] recruiting effector cells including phagocytes to the site of infection, enhancing intracellular [44] and extracellular [45] bactericidal activity. AMPs also mediate macrophage differentiation [46] which is required for effective clearance of pathogens from the host. AMPs also induce apoptosis [47] and pyroptosis [48] in the infected cells as a means of clearing pathogens. Despite their effectiveness in pathogen clearance, host defense peptides (HDPs) were not popular for clinical usage because of their high synthesis cost [49]. There is a need to improve the efficacy of AMPs and we tried to accomplish this by conjugating LL-37 and indolicidin to carboxylated CNT. We compared immune modulatory properties of these two peptides in treated macrophages, both in free and CNT conjugated state.

### Role of LL-37, indolicidin and their CNT conjugates in modulating pro- and anti-inflammation in THP-1 human macrophage cells

LL-37 activates the canonical NFkB pathway responsible for modulating the expression of various genes involved in the innate immune system [19]. Our results revealed that the expression of pro-inflammatory genes that play a critical role in regulating the NFκB pathway [RELA, TNFRSF1A, TRAF6, ATM and BTRC (Additional file 2: Table S2)] are up-regulated upon CNT–LL-37 treatment; notably, LL-37 conjugate treated cells also show almost the same expression pattern, but at 1000 fold less concentration than free LL-37. The expression profile of TNFRSF1A, LELA, RIPK4, RUSC1 and RBCK1 genes in CNT–indolicidin as well as free indolicidin treated cells confirms pro-inflammation mediated by the NFκB pathway. LL-37 induced signaling through the P^38^ MAPK pathway, followed by activation of genes responsible for macrophage differentiation, pro-inflammation and proliferation [50]. Activation of 36 genes (Additional file 2: Table S2) related to the P38 MAPK pathway following LL-37 treatment, and up-regulation of several genes (Additional file 3: Table S3) following indolicidin treatment confirmed that the conjugates stimulate signaling similar to that of the free peptide, but at a 1000-fold lower dose. Through the interaction of phosphoinositide 3-kinase (PI3 K), NFκB and MAPK pathways, LL-37 induced IL-1B, followed by pro-inflammation in monocytes and macrophages [51]. Expression of IL1B (Additional file 2: Table S2, Additional file 3: Table S3) in THP-1 cells following LL-37 and indolicidin conjugate treatment indicates that conjugated AMPs can induce pro-inflammation in macrophages at the same levels stimulated by free peptides, but at a 1000-fold lower concentration. AMPs can also induce the production of IL17 and reactive oxygen species (ROS), enhancing the phagocytic activity of macrophages and capacity for clearing pathogens within the phagosome [52]. Expression of IL17RE (Table 2) in THP-1 cells following treatment with LL-37 as well CNT conjugated LL-37 indicates a similar effect of the conjugate on macrophages. This is a controlled inflammation, indicated by the moderate expression of anti-inflammatory cytokine IL10 along with pro-inflammatory cytokines IL6, IL12, IL1a, IFNa and IFNb (Fig. 2c, g) in the conjugate treatments as well as free LL-37 and indolicidin treatments at 20 μg/ml. This data indicated that LL-37 and indolicidin conjugates are similarly effective in modulating pro-inflammatory pathways as their free peptide forms, but at 1000 fold less concentration.

### Role of LL-37, indolicidin and their CNT conjugates in modulating chemokine expression in the THP-1 human macrophage cell line

AMPs are chemo attractants for monocytes, neutrophils, macrophages and T-cells. These molecules can induce various chemokines and chemokine receptors in macrophages to attract these immune cells to the site of infection [43, 53]. Expression of CCL20, CCL4 and CCL19 (Table 2) in CNT–LL-37 and CCL20, CCL19, CCL7, CCL4 (Table 3) in indolicidin conjugate treated THP-1 cells indicates that both conjugates are able to stimulate similar signaling pathways involved in macrophage chemotaxis. However, this conclusion needs to be verified in vivo.

### Additional functions of free and conjugated AMPs in THP-1 cells

AMPs induce autophagy in infected macrophages to facilitate the clearance of intercellular debris, which is controlled through ATG5 gene [54]. Expression of ATG5 in LL-37 and its CNT conjugate treated cells might result in similar autophagic activity. However, CNT itself is a very efficient autophagic inducer, as CNT treatment shows 16 fold up-regulation of ATG5 in THP-1 cells. Apart from enhancing the efficacy of AMPs, CNT itself has this added effect. LL-37 and beta-defensin, induce epidermal growth factor receptor (EGFR) signaling, followed by activation of the PI3 K-AKT and MAPK pathways responsible for cell proliferation during wound healing [55]. THP-1 cells treated with LL-37 and its conjugate also show activation of the PI3 K-AKT and MAPK signaling pathways (Table 1), as well as up-regulation of PDGFRA gene (Additional file 2: Table S2).

It is reported that LL-37 delays apoptosis in monocytes and neutrophils by activating G protein coupled receptor (GPCR) mediated signaling [47]. THP-1 cells treated with LL-37 or its conjugate also appear to exhibit active GPCR signaling as we have recorded the expression of GPR180, GPRC5C, GPR174 and GPR3 (Additional file 2: Table S2); similarly, THP-1 cells treated with indolicidin or its conjugate resulted in GPCR signaling as shown by the expression of GPR135, GPR176, GPR112, GPR110, GPR173 and GPR3 (Additional file 3: Table S3). Compared to LL-37, indolicidin imparts more pro-inflammatory effects in THP-1 cells; however, the regulation of inflammation through TGFβ and IL10 pathway was stronger in LL-37 treated cells, indicated by the genes listed in Table 4. The overall mechanism through which LL-37, indolicidin and their CNT conjugates protect macrophages from *salmonella* induced cytotoxicity could be graphically summarized, as depicted in Fig. 4c.

**Table 4:** LL-37 and indolicidin modulates immune genes and pro-apoptotic genes differently

Gene symbol	CNT	CNT + Indo	CNT–Indo	Indo-20	CNT + LL37	CNT–LL37	LL37-20	Gene description
ANAPC11	−2.4	1.2	1.7	−1.4	1	1	1	Anaphase promoting complex subunit 11
CCL20	5.5	1.5	10.9	11	1	10.1	9.2	Chemokine (C–C motif) ligand 20
DEFA3	1.1	1	1.6	1.1	1	1	1	Defensin, alpha 3
IL31RA	1.4	−6.7	1.6	−1	1	4.4	1	Interleukin 31 receptor A
NFATC2	3.1	2.1	4.3	1.9	1.7	4.9	12.4	Nuclear factor of activated T-cells, cytoplasmic, calcineurin-dependent 2
NFATC2IP	1.3	−1.8	2	1.1	1	1	1	Nuclear factor of activated T-cells, cytoplasmic, calcineurin-dependent 2 interacting protein
PKMYT1	−1	−1.4	1.6	−1.3	1	1	1	Protein kinase, membrane associated tyrosine/threonine 1 (PKMYT1)
RB1	−3.3	−4.1	1.6	1.6	1	1	1	Retinoblastoma 1
SMAD3	−1.3	−2.3	2.5	1.2	1	2.5	1	Mad protein homolog
TGFBR1	1	−1	1.5	−1.1	1	2.5	1.7	Transforming growth factor, beta receptor 1
CHEK1	−3	−1.1	−6.5	1.6	1	−3.9	1	CHK1 checkpoint kinase
CDKN2A	1.5	−1.2	−4.2	−2	1	4.6	4.3	Cyclin-dependent kinase inhibitor 2A
TNFSF4	1.1	1	−3.3	5.5	1	−3.2	−2.9	Tumor necrosis factor (ligand) superfamily, member 4
CDC25C	−1	−1	−2.9	1	−3.6	1	1	Cell division cycle 25 homolog C
CDC14B	−1	−1.3	−2.4	−1.7	−3.8	−7.4	1	CDC14 cell division cycle 14 homolog B
GADD45B	1.8	−1.8	−2.1	−2	1	2.2	1.6	Growth arrest and DNA-damage-inducible, beta
TLR1	−4.4	−1.2	−1.4	−1.8	1	1	1	Toll-like receptor 1
TLR3	1	1	2.3	1	1	2.8	1	Toll-like receptor 3
TNFAIP3	−2.5	−7	−9	1.6	−4.4	1	1	Tumor necrosis factor, alpha-induced protein 3
CCL14	4.3	−1.3	3.9	−1.2	1	1	1	Chemokine (C–C motif) ligand 14
BCL2L2	1.4	2.7	2.5	1.1	1.1	3.2	1.3	BCL2-like 2
Apaf1	4.3	1.1	1.1	1.0	1.2	1.1	1.1	Apoptotic peptidase activating factor 1

Data from *Salmonella* challenge studies revealed that, LL-37 or indolicidin primed THP-1 cells can efficiently protect themselves against ST induced cytotoxicity for 16 h post challenge. The genome wide gene expression study shows that pro-inflammatory and anti-apoptotic signaling in THP-1 cells treated with indolicidin may be mediated through *TNFRSF1A,* followed by activation of *NFκB* and *c*-*JUN.* However, pro-inflammation, cell proliferation and cell differentiation in THP-1 following LL-37 treatment may mediated through *IL1R,* followed by activation *of NFκB* and *NFAT2*. Though immune modulation by LL-37 and indolicidin was partly known before, our data established the complete gene expression and signaling mechanism. The conjugation strategy enhanced the immune modulating efficacy of these two peptides by 1000 fold, which will reduce the cost of these peptides for antimicrobial treatment, thereby increasing treatment access to a wider population of developing countries. Although LL-37 and indolicidin conjugation with CNT shows promise with regards to resisting ST infection in vitro, further trials need to be conducted in vivo for better understanding of its working mechanism.

## Conclusions

Present study established an important fact for the usage of nanomaterials in biomedicine is that efficacy of a drug or a bio-agent can be enhanced by conjugating it with a suitable nanomaterial. Our results confirmed that at 1000-fold less concentration of either of the peptide, LL-37 or indolicidin, can be equally effective at 0.02 μg/ml while the same is observed at 20 μg/ml with free peptides. The current report also established the efficacy of the conjugated peptide in protecting macrophage cells against *salmonella* challenge as well as mechanism by which the peptides are protecting is also reported.

## Additional files

**Additional file 1: Table S1.** List of primers used to validate microarray through q-RTPCR.

**Additional file 2: Table S2.** Gene expression in terms of fold changes following treatment with free LL37, conjugates and other relevant controls.

**Additional file 3: Table S3.** Expression of genes in terms of fold changes following treatment free and conjugated Indolicidin and other conditions.

## Abbreviations

AMPs: antimicrobial peptides
CNT: carbon nanotubes
SM-CNT: short multi-walled carbon nano-tubes
ST: *Salmonella typhimurium serovar enterica*
PMA: phorbol-12-myristate-13-acetate
qRT-PCR: quantitative real time polymerase chain reaction
MOI: multiplicity of infection
cDNA: complementary DNA
Webgestalt: WEB-based GEneSeT AnaLysis Toolkit
