# Supplementary material for: Comparative efficacy analysis of anti-microbial peptides, LL-37 and indolicidin upon conjugation with CNT, in human monocytes
Source: J Nanobiotechnology. 2017 Jun 12;15:44. doi: 10.1186/s12951-017-0278-1 (PMC5469186; doi:10.1186/s12951-017-0278-1)
Supplement: Supplementary file 1 — Additional file 1: Table S1. List of primers used to validate microarray through q-RTPCR. [file 12951_2017_278_MOESM1_ESM.pdf]

Table S1: List of primers used to validate microarray through q-RTPCR.

GENE NAME	FORWARD PRIMER	REVERSE PRIMER	NCBI SEQUENCE ACCESSION NUMBER
C-JUN	TTGGACTGGGTTGCGTCCTG	CGAAAAGTCCAACGTTCCGTTC	NM_002228.3
IKB1	GCTGCTGCTTCAGGCAATTCAGAG	GTGCTTCAGCCACCAGTTCTTCAC	NM_001556.2
IKBKG	ACCGTGCAGTCTGCGCTTTC	GGCCGCATCTACCCCAAAAG	NM_003639.4
IL1R	ATGCCACCGATTGCAGGACA	CCCAGGAGAAAGCAGGTGGAA	M27492.1
IRAK1	GAAGCCCCTGGAAGGCAGAA	CGTCACCAATGCCCAGCTTC	NM_001569.3
MAP2K7	ACTGGGAAGGACCGGGTGAG	TTCTGCGCCTTTGGTGTTGG	NM_001297555.1
MAP3K14	CCCTGGCCAGAGGGTACTGC	AGGCTAAGCTGGGGCAATGG	NM_003954.4
MAPK10	CATCCCAACTTTTCCGGTAGGC	CCAACTGCCCCAAAGGAAGC	NM_138980.3
MYD88	TCTGTCTGCCTGTCCATGTACTTC	CCCAGAGCTATGCTTCACCATTTC	NM_001172567.1
RELA	ATGCAGTTGCGGAGACCTTCTGAC	GGTGCCATTGAGGCATGATGTGAC	NM_002908.2
RIPK2	GATATACCTCACCGAGCACGTATG	GGTGCTATCCCAACTGTGATTTCC	NM_003821.5
TAB1	AGCCTCTGGGGTGCTTGGTC	CGAGGACCCTGGGCTGAGAC	U49928.1
TAK1	AAGCTAGGATCGCCGCAACC	AGGGGTCCATGGATGACTTCG	AF218074.1
TNFRSF1	TCATGCCCGTTTTGGGTGTC	GCTGAAGGCCCCATTGTTCC	NM_001065.3
TRADD	TGGTGGAGGCACTCGAGGAG	CGTGGATGGACAGGGGTTCA	NM_003789.3
TRAF2	TCTGGCCCCTGGAGAGAAGG	TTACCCGCAGGCTGTGCTGT	NM_021138.3
TRAF6	ACCATCAAATCCGGGAGCTGACTG	CCAAGGGAGGTGGCTGTCATATTC	NM_145803.1
IL6	GAACAAGCCAGAGCTGTGCAGATG	TAAGTTCTGTGCCCAGTGGACAGG	NM_000600.3
IL10	GGCATCTACAAAGCCATGAGTGAG	TCAACAGCTAGAAAGCGTGGTCAG	NM_000572.2
IL12	TCTTGGAGCGAATGGGCATCTGTG	TGAAGGCCCATGGCAACTTGAGAG	NM_002187.2
IFNA	CGTGCTGGTACTCAGCTACAAATC	GGCACAAGGGCTGTATTTCTTCTC	NM_021057.2
IFNB1	TAGTAGGCGACACTGTTCGTGTTG	TGGCCTTCAGGTAATGCAGAATCC	NM_002176.2
TNFA	CTGTAGCCCATGTTGTAGCAAACC	CAGGGCAATGATCCCAAAGTAGAC	NM_000594.2
NFKB1	TGGAGGCGGAGGCATGTTTGGTAG	TTCACGTCTCCTGTCACCGAGTAG	NM_003998.3
