# Supplementary material for: Comparative efficacy analysis of anti-microbial peptides, LL-37 and indolicidin upon conjugation with CNT, in human monocytes
Source: J Nanobiotechnology. 2017 Jun 12;15:44. doi: 10.1186/s12951-017-0278-1 (PMC5469186; doi:10.1186/s12951-017-0278-1)
Supplement: Supplementary file 2 — Additional file 2: Table S2. Gene expression in terms of fold changes following treatment with free LL37, conjugates and other relevant controls. [file 12951_2017_278_MOESM2_ESM.pdf]

Table S2: Gene expression in terms of fold changes following treatment with free LL37, conjugates and other releavnt controls

ENTREZ ID	Fold Change Values				Gene Symbol
	**CNT**	**LL37-20**	**CNT-LL37**	**CNT+LL37**	
503538	1.0	1.0	1.0	1.0	A1BG-AS1
29974	1.0	-2.5	-2.6	-2.3	A1CF
87769	1.0	1.0	1.0	1.0	A2LD1
144568	-1.6	1.0	1.0	-5.6	A2ML1
53947	1.0	1.0	-2.6	1.0	A4GALT
51146	1.0	3.6	1.0	1.0	A4GNT
79719	1.0	1.0	1.0	1.0	AAGAB
57505	1.0	1.0	5.8	5.7	AARS2
10349	-18.0	-5.0	-4.4	-3.4	ABCA10
20	1.0	1.0	1.0	2.5	ABCA2
21	1.0	1.0	1.0	2.2	ABCA3
24	1.0	-3.7	-3.8	1.0	ABCA4
23460	1.0	1.0	1.0	1.0	ABCA6
10350	1.0	1.0	1.0	1.0	ABCA9
23456	-13.4	1.0	-5.2	1.0	ABCB10
340273	1.0	1.0	1.0	-4.6	ABCB5
22	23.7	1.0	1.0	1.0	ABCB7
150000	3.5	-3.2	-3.6	-4.6	ABCC13
1244	1.0	1.0	1.0	4.8	ABCC2
8714	1.0	1.0	1.0	1.0	ABCC3
10057	1.0	1.0	1.0	1.0	ABCC5
10060	-28.7	1.0	1.0	1.0	ABCC9
215	1.0	1.0	3.1	4.2	ABCD1
5825	4.3	-3.1	-2.7	-2.9	ABCD3
23	1.0	1.0	1.0	1.0	ABCF1
9619	4.8	1.0	1.0	7.1	ABCG1
9429	5.8	1.0	1.0	7.2	ABCG2
84696	1.0	1.0	1.0	1.0	ABHD1
140701	1.0	1.0	1.0	1.7	ABHD16B
57406	1.0	1.0	1.0	1.0	ABHD6
25890	1.0	-4.6	-6.6	-4.2	ABI3BP
25	1.0	1.0	1.0	1.0	ABL1
27	1.9	-7.6	-11.8	-14.0	ABL2
3983	1.0	1.0	1.0	1.0	ABLIM1
22885	1.0	1.0	1.0	1.0	ABLIM3
28	1.0	-1.5	-1.9	1.0	ABO
29	1.0	1.0	-2.6	-2.7	ABR
137735	2.1	-2.7	1.0	1.0	ABRA
80325	1.0	1.0	1.0	1.0	ABTB1
10449	1.0	1.0	1.0	-4.1	ACAA2
27034	1.0	1.0	2.1	1.0	ACAD8
28976	3.3	1.0	1.0	1.0	ACAD9
33	-2.2	1.0	1.0	1.0	ACADL
37	-1.7	1.0	1.0	1.0	ACADVL

176	1.0	1.0	2.1	1.0	ACAN
9744	1.0	3.2	2.1	1.0	ACAP1
38	1.0	1.0	-2.9	1.0	ACAT1
79777	1.0	1.0	1.0	1.0	ACBD4
91452	1.0	1.0	1.0	1.0	ACBD5
414149	1.0	1.0	1.0	1.0	ACBD7
84680	-5.0	1.0	2.8	1.0	ACCS
65057	1.0	1.0	1.0	1.0	ACD
22985	-3.3	1.0	1.0	-5.4	ACIN1
641371	1.0	1.0	1.0	1.0	ACOT1
26027	1.0	1.6	1.0	1.0	ACOT11
55856	1.0	-2.5	-2.7	1.0	ACOT13
10005	2.8	1.0	1.0	1.0	ACOT8
23597	1.0	1.0	2.0	1.0	ACOT9
8310	-12.7	1.0	1.0	-4.7	ACOX3
55289	1.0	1.0	-10.3	1.0	ACOXL
52	1.0	1.0	1.0	1.0	ACP1
53	-2.0	1.0	-1.6	1.0	ACP2
92370	1.0	1.0	1.0	1.0	ACPL2
55	2.1	-4.9	-3.6	1.0	ACPP
80221	1.0	1.0	-3.2	1.0	ACSF2
197322	1.7	1.0	1.9	1.0	ACSF3
2182	1.0	1.0	6.4	1.0	ACSL4
51703	1.0	1.0	1.0	3.2	ACSL5
116285	1.0	1.0	-2.3	-2.4	ACSM1
54988	1.0	1.0	-4.9	-4.2	ACSM5
84532	6.8	1.0	1.0	1.0	ACSS1
55902	1.0	1.0	1.9	1.0	ACSS2
79611	1.0	1.0	1.0	1.0	ACSS3
58	-5.1	2.8	2.7	3.1	ACTA1
60	1.0	-3.2	-3.3	-3.2	ACTB
86	1.0	1.0	1.0	1.0	ACTL6A
88	1.0	3.4	1.0	1.0	ACTN2
81	-6.8	1.0	3.3	1.0	ACTN4
10121	2.0	1.0	1.0	1.0	ACTR1A
10120	1.0	1.0	1.0	1.0	ACTR1B
57180	-1.6	1.0	-7.7	1.0	ACTR3B
140625	1.0	1.0	1.0	1.0	ACTRT2
90	-2.1	1.0	1.0	-5.7	ACVR1
130399	2.6	1.0	1.0	1.0	ACVR1C
93	-2.1	1.0	1.0	1.0	ACVR2B
95	2.0	1.0	1.0	1.0	ACY1
132612	1.0	1.0	1.0	1.0	ADAD1
161931	1.0	2.8	1.0	1.0	ADAD2
161823	1.0	2.7	1.0	1.0	ADAL
4185	1.0	1.0	1.0	1.0	ADAM11
8728	1.0	1.0	1.0	3.5	ADAM19
8747	1.0	6.0	6.5	6.7	ADAM21

53616	1.0	1.0	1.0	2.6	ADAM22
8745	1.0	1.0	1.0	1.0	ADAM23
11086	1.0	1.0	-3.1	1.0	ADAM29
11085	1.0	-6.7	-4.5	1.0	ADAM30
203102	6.3	1.6	1.0	1.0	ADAM32
80332	1.0	1.0	1.0	-4.9	ADAM33
8756	1.0	1.0	1.0	2.1	ADAM7
8754	1.0	1.0	-5.7	1.0	ADAM9
81792	1.0	1.0	-3.8	1.0	ADAMTS12
170689	1.0	1.0	1.0	1.0	ADAMTS15
170690	5.8	1.0	1.0	1.0	ADAMTS16
170691	4.2	8.8	1.0	1.0	ADAMTS17
9508	1.0	1.0	1.0	-5.4	ADAMTS3
92949	1.0	1.0	1.0	1.0	ADAMTSL1
9719	1.0	1.0	1.0	1.0	ADAMTSL2
57188	1.0	1.0	1.0	2.8	ADAMTSL3
54507	1.0	1.5	1.0	1.0	ADAMTSL4
339366	2.1	1.0	1.0	1.0	ADAMTSL5
11033	1.0	1.0	1.0	1.0	ADAP1
55803	1.0	1.0	-3.5	1.0	ADAP2
104	1.0	-6.3	1.0	1.0	ADARB1
105	1.0	4.8	3.0	3.2	ADARB2
642394	1.0	1.0	1.0	1.0	ADARB2-AS1
113451	1.0	-4.1	-4.1	1.0	ADC
108	-4.5	1.0	1.0	2.9	ADCY2
117	1.0	1.0	1.0	1.0	ADCYAP1R1
119	-2.2	1.0	-5.0	1.0	ADD2
120	-2.3	1.0	1.0	1.0	ADD3
127	1.0	1.0	1.0	1.0	ADH4
55256	1.0	1.0	-2.2	-1.6	ADI1
149685	1.0	4.8	3.7	1.0	ADIG
9370	1.0	1.0	2.5	1.0	ADIPOQ
51094	-1.8	1.0	-2.4	-2.4	ADIPOR1
79602	1.0	3.8	1.0	1.0	ADIPOR2
132	2.3	1.0	1.0	1.0	ADK
133	1.0	1.0	1.0	1.0	ADM
84890	15.8	1.0	1.0	1.0	ADO
134	1.0	1.0	2.4	1.0	ADORA1
136	1.0	1.0	5.7	5.0	ADORA2B
140	2.0	1.0	1.0	1.0	ADORA3
83440	1.0	1.0	1.0	1.0	ADPGK
54936	1.0	-4.3	1.0	1.0	ADPRHL2
153	1.0	1.0	1.0	1.0	ADRB1
157	1.0	1.0	4.3	1.0	ADRBK2
134265	1.0	-3.4	1.0	-5.8	AFAP1L1
4299	1.0	1.0	1.0	-3.8	AFF1
2334	1.0	3.6	2.9	6.0	AFF2
3899	1.0	1.0	1.0	1.0	AFF3

27125	5.6	1.0	2.4	1.0	AFF4
172	-2.9	1.0	-6.4	1.0	AFG3L1P
125061	1.0	1.0	1.0	1.0	AFMID
116988	2.6	2.8	1.0	1.0	AGAP3
653268	1.0	1.0	1.0	1.0	AGAP7
340351	1.0	1.0	1.0	1.0	AGBL3
3268	1.0	1.0	4.7	4.2	AGFG2
392636	1.0	1.0	1.0	1.0	AGMO
10555	1.0	2.9	1.0	1.0	AGPAT2
56895	1.0	6.1	4.1	1.0	AGPAT4
137964	1.0	1.0	1.0	1.0	AGPAT6
8540	3.3	1.0	1.5	1.0	AGPS
10551	1.0	1.0	1.0	1.0	AGR2
181	1.0	2.7	1.0	1.0	AGRP
186	1.0	1.0	1.0	1.0	AGTR2
57085	1.0	1.0	1.0	1.0	AGTRAP
85007	1.0	1.0	-5.8	1.0	AGXT2L2
25909	1.0	1.0	1.0	1.0	AHCTF1
10768	1.0	1.0	1.0	1.0	AHCYL1
23382	1.5	1.0	1.0	1.0	AHCYL2
54806	1.8	1.0	1.0	1.0	AHI1
79026	1.0	-2.5	1.0	-2.6	AHNAK
113146	1.0	1.0	3.2	1.0	AHNAK2
57379	1.0	1.0	1.0	1.0	AICDA
9255	-2.7	1.0	1.0	1.0	AIMP1
203	1.0	1.0	1.0	-6.1	AK1
204	1.0	1.0	-4.8	1.0	AK2
50808	-17.6	1.0	1.0	1.0	AK3
26289	1.0	1.0	1.0	1.0	AK5
8165	1.0	1.0	1.0	1.0	AKAP1
11215	1.0	-7.7	-6.4	1.0	AKAP11
9590	-2.2	-3.6	-3.4	-3.1	AKAP12
11214	-3.3	1.0	1.0	1.0	AKAP13
10566	1.0	6.7	3.1	1.0	AKAP3
8852	1.0	1.0	1.0	1.0	AKAP4
9495	1.0	1.0	1.0	1.0	AKAP5
9472	1.0	1.0	-2.8	1.0	AKAP6
9465	1.0	1.0	1.0	1.0	AKAP7
10142	1.0	1.0	1.0	1.0	AKAP9
221264	1.0	1.0	1.0	1.0	AKD1
80709	1.0	-13.0	-14.8	-7.3	AKNA
254268	1.0	1.0	-7.3	1.0	AKNAD1
231	1.0	2.6	2.6	2.7	AKR1B1
1645	-1.8	-4.0	-1.7	1.0	AKR1C1
83592	1.0	1.0	1.0	1.0	AKR1E2
208	2.2	-3.1	1.0	1.0	AKT2
10000	1.0	1.0	1.0	1.0	AKT3
211	3.4	1.0	-3.4	-4.9	ALAS1

212	1.0	1.0	1.0	1.0	ALAS2
219	1.7	1.0	1.0	1.0	ALDH1B1
10840	1.0	1.0	5.0	1.6	ALDH1L1
224	1.0	1.0	-4.0	1.0	ALDH3A2
7915	1.0	1.0	1.0	1.0	ALDH5A1
4329	1.0	-2.9	1.0	1.0	ALDH6A1
223	1.0	1.0	-4.3	1.0	ALDH9A1
228	1.0	-3.4	1.0	-3.0	ALDOAP2
229	1.0	1.0	1.0	1.9	ALDOB
56052	12.3	7.8	1.0	1.0	ALG1
79868	1.5	1.0	-2.3	-2.3	ALG13
29880	1.0	1.0	1.0	1.0	ALG5
29929	1.0	1.0	3.0	1.0	ALG6
221120	1.0	1.0	1.0	1.0	ALKBH3
54784	1.0	1.0	1.0	1.0	ALKBH4
54890	1.0	1.0	5.8	1.0	ALKBH5
84964	1.0	-2.4	-2.4	1.0	ALKBH6
91801	1.5	1.0	-3.0	1.0	ALKBH8
245	1.0	1.0	1.0	1.0	ALOX12P2
240	1.0	1.0	6.0	1.0	ALOX5
80216	1.0	1.0	2.5	1.0	ALPK1
115701	5.9	1.0	7.0	5.1	ALPK2
57538	1.0	1.0	-4.2	1.0	ALPK3
249	-1.6	5.3	1.0	1.0	ALPL
57679	1.8	1.0	5.2	1.0	ALS2
130540	1.0	1.0	1.0	1.0	ALS2CR12
60529	1.5	2.0	1.8	1.0	ALX4
23600	-1.6	-4.3	1.0	1.0	AMACR
262	1.8	1.0	1.0	1.0	AMD1
267	1.0	1.0	1.0	1.0	AMFR
268	1.0	1.0	1.0	1.0	AMH
120425	1.0	1.0	1.0	1.0	AMICA1
57463	1.0	-2.7	1.0	1.0	AMIGO1
83607	1.0	1.0	1.0	1.0	AMMECR1L
154796	1.0	3.5	-4.9	1.0	AMOT
51421	1.0	-1.8	1.0	1.0	AMOTL2
271	1.6	1.0	1.0	1.0	AMPD2
275	1.0	1.0	1.0	2.7	AMT
51321	1.0	1.0	3.8	1.0	AMZ2
201283	1.0	1.0	1.0	1.0	AMZ2P1
64682	1.0	1.0	-2.4	-2.9	ANAPC1
51529	-2.4	1.0	1.0	1.0	ANAPC11
25847	1.0	1.0	-3.1	1.0	ANAPC13
29945	1.0	1.0	1.0	1.5	ANAPC4
51434	1.0	-5.5	1.0	1.0	ANAPC7
23357	1.0	1.0	1.0	1.0	ANGEL1
285	1.0	1.0	1.0	1.0	ANGPT2
23452	1.6	1.0	-1.6	1.0	ANGPTL2

253935	1.0	1.0	1.0	1.0	ANGPTL5
288	10.1	-3.3	3.5	2.6	ANK3
150709	1.0	1.0	-7.6	-6.7	ANKAR
162282	1.0	1.0	1.0	1.0	ANKFN1
51479	1.0	2.6	3.3	1.0	ANKFY1
126549	1.0	1.0	1.0	1.0	ANKLE1
23141	1.0	-4.3	-5.6	-5.3	ANKLE2
51281	1.0	2.6	2.6	1.0	ANKMY1
55608	1.0	-2.5	1.0	1.0	ANKRD10
29123	1.0	1.0	1.0	1.0	ANKRD11
23253	-2.1	1.0	1.0	-4.8	ANKRD12
81573	1.0	1.0	-6.6	1.0	ANKRD13C
54522	4.1	1.0	1.0	1.0	ANKRD16
26057	1.0	1.0	1.0	1.0	ANKRD17
253650	12.5	1.0	1.0	1.0	ANKRD18A
441459	1.0	-5.5	1.0	1.0	ANKRD18B
138649	1.0	1.0	-3.5	1.0	ANKRD19P
26287	1.0	1.0	-3.5	1.0	ANKRD2
441430	-3.5	1.0	1.0	1.0	ANKRD20A2
440482	-2.6	2.5	-5.6	1.0	ANKRD20A5P
284232	-21.3	-7.8	-8.2	-9.0	ANKRD20A9P
118932	1.0	1.0	1.0	-24.7	ANKRD22
200539	1.0	-3.2	-3.4	1.0	ANKRD23
170961	1.0	1.0	1.0	1.0	ANKRD24
23243	4.1	-7.4	-7.4	1.0	ANKRD28
147463	1.0	1.0	1.0	2.7	ANKRD29
91074	1.0	1.0	1.0	1.0	ANKRD30A
374860	1.0	-2.9	1.0	1.0	ANKRD30B
149992	1.0	1.0	1.0	1.0	ANKRD30BP2
256006	1.0	1.0	-7.2	1.0	ANKRD31
148741	2.0	1.0	1.0	1.0	ANKRD35
57730	2.2	-7.0	-2.4	-3.2	ANKRD36B
84832	1.0	1.0	-4.4	1.0	ANKRD36BP1
157567	1.6	1.0	4.9	1.0	ANKRD46
79998	1.0	1.0	1.0	1.0	ANKRD53
129138	1.0	1.0	3.3	3.4	ANKRD54
56311	1.0	1.0	1.0	1.0	ANKRD7
56899	1.0	-2.7	-4.9	-3.1	ANKS1B
124401	1.0	1.0	1.0	1.0	ANKS3
55139	5.3	1.0	1.0	1.0	ANKZF1
57101	1.0	1.0	1.0	1.0	ANO2
50636	1.0	3.4	1.0	-4.8	ANO7
338440	1.0	1.0	1.0	-2.8	ANO9
8125	1.0	1.0	1.0	-6.4	ANP32A
23519	1.0	-2.0	1.0	1.0	ANP32D
302	1.0	2.5	2.5	1.0	ANXA2
308	-1.7	1.0	1.0	1.0	ANXA5
314	1.0	1.0	1.0	1.0	AOC2

90586	1.0	1.0	1.0	1.0	AOC4
316	1.0	4.4	1.0	1.0	AOX1
164	1.0	1.0	3.0	1.0	AP1G1
8907	1.0	3.0	1.0	1.0	AP1M1
1174	1.0	2.1	1.0	1.0	AP1S1
8905	1.0	1.0	-6.0	1.0	AP1S2
160	-17.0	1.0	1.0	1.0	AP2A1
161	1.0	-4.0	1.0	1.0	AP2A2
8120	1.0	1.0	4.4	5.2	AP3B2
26985	1.0	-4.4	1.0	1.0	AP3M1
1176	1.0	1.0	1.0	1.0	AP3S1
10239	1.0	-5.8	1.0	1.0	AP3S2
10717	1.0	1.0	1.0	-1.7	AP4B1
23431	1.0	1.0	1.0	1.0	AP4E1
9179	1.0	-4.4	1.0	1.0	AP4M1
9546	1.0	1.0	4.3	1.0	APBA3
10307	1.0	1.0	-2.5	1.0	APBB3
328	1.0	1.0	1.0	1.0	APEX1
51107	1.5	1.0	1.0	1.0	APH1A
378708	1.0	1.0	1.0	1.0	APITD1
200558	1.0	1.0	-7.4	1.0	APLF
187	1.0	1.0	-3.8	1.0	APLNR
334	1.0	2.0	2.4	1.0	APLP2
338	1.0	-1.8	-2.1	-2.2	APOB
9582	1.0	1.0	1.0	1.0	APOBEC3B
27350	1.0	1.0	1.0	1.0	APOBEC3C
200316	1.0	1.0	-7.6	-4.4	APOBEC3F
346	1.0	1.0	1.0	2.7	APOC4
80831	1.0	1.0	1.0	1.0	APOL5
80830	1.0	1.0	1.0	5.1	APOL6
81575	1.0	1.0	1.0	1.0	APOLD1
84334	-2.7	5.3	1.0	1.0	APOPT1
351	1.0	1.0	1.0	1.0	APP
353	1.0	-5.3	-8.0	-7.1	APRT
54840	1.0	1.0	1.0	1.0	APTX
358	1.0	1.0	1.0	1.0	AQP1
359	1.6	1.0	-2.6	1.0	AQP2
363	3.9	1.0	1.0	1.0	AQP6
364	1.0	2.4	1.0	1.0	AQP7
9716	1.0	1.0	1.0	1.0	AQR
369	1.0	1.0	-3.0	-2.9	ARAF
116985	1.0	2.0	2.3	1.0	ARAP1
23237	1.0	1.0	1.0	1.0	ARC
372	-2.1	1.0	1.0	2.7	ARCN1
374	1.0	1.0	1.0	1.0	AREG
377	1.0	1.0	1.0	1.0	ARF3
378	1.0	-3.1	1.0	1.0	ARF4
381	-1.5	1.0	1.0	1.0	ARF5

55738	2.0	1.0	1.0	1.0	ARFGAP1
10564	1.0	1.0	-3.0	1.0	ARFGEF2
27236	1.0	3.8	3.8	1.0	ARFIP1
10139	1.0	1.0	1.0	1.0	ARFRP1
384	2.6	2.9	1.0	1.0	ARG2
84986	-3.5	1.0	5.4	1.0	ARHGAP19
57569	1.0	2.5	1.0	1.0	ARHGAP20
57584	1.7	1.0	1.0	1.0	ARHGAP21
58504	1.0	1.0	1.0	1.0	ARHGAP22
201176	2.9	1.0	-4.5	1.0	ARHGAP27
79822	1.0	1.0	4.9	1.0	ARHGAP28
9411	1.0	-6.7	-6.5	1.0	ARHGAP29
57514	1.0	1.0	1.0	1.0	ARHGAP31
9743	1.0	1.0	1.0	1.0	ARHGAP32
115703	1.0	1.0	1.0	1.0	ARHGAP33
2909	1.0	-3.6	-6.3	-5.1	ARHGAP35
80728	1.0	1.0	1.0	1.0	ARHGAP39
9912	1.0	1.0	3.9	1.0	ARHGAP44
394	1.0	1.0	1.0	1.0	ARHGAP5
64333	1.0	1.0	1.0	1.0	ARHGAP9
397	1.0	2.1	1.0	1.0	ARHGDIB
9639	-18.5	1.0	2.9	1.0	ARHGEF10
55160	1.0	-5.1	-6.2	-5.5	ARHGEF10L
9828	1.0	6.1	1.0	5.1	ARHGEF17
23370	1.0	1.0	1.0	1.0	ARHGEF18
115557	4.3	1.0	1.0	1.0	ARHGEF25
26084	1.0	1.0	1.0	3.3	ARHGEF26
389337	1.0	1.0	1.6	1.0	ARHGEF37
50649	-5.1	5.5	1.0	1.0	ARHGEF4
55701	1.0	1.0	-8.3	1.0	ARHGEF40
8874	2.7	1.0	-3.1	1.0	ARHGEF7
8289	2.0	2.6	2.8	2.9	ARID1A
57492	1.7	1.0	1.0	-3.9	ARID1B
196528	1.0	1.0	1.0	1.0	ARID2
5926	1.0	1.0	-2.3	1.0	ARID4A
10865	3.0	-2.0	1.0	1.0	ARID5A
84159	1.0	1.0	1.0	1.0	ARID5B
285598	1.0	-2.2	-4.5	1.0	ARL10
200894	3.1	1.0	2.3	1.0	ARL13B
100506084	1.0	1.0	1.0	2.4	ARL17B
403	13.3	1.0	1.0	1.0	ARL3
10124	1.0	1.0	-5.8	1.0	ARL4A
10123	1.0	1.0	2.2	1.0	ARL4C
84100	1.9	1.0	1.0	-3.6	ARL6
23204	-2.1	1.0	1.0	1.0	ARL6IP1
55207	1.0	1.0	1.0	1.0	ARL8B
84071	1.0	1.0	1.0	-3.3	ARMC2
219681	1.0	1.0	1.0	-2.5	ARMC3

93436	1.0	1.0	1.0	-2.1	ARMC6
100131755	1.0	1.0	1.0	1.0	ARMCX4
9915	1.0	1.0	1.0	1.0	ARNT2
56938	1.0	1.0	5.0	1.0	ARNTL2
10095	1.0	1.0	1.0	1.0	ARPC1B
100526693	-1.8	1.0	1.0	1.0	ARPC4-TTLL3
10092	-1.8	1.0	-7.0	1.0	ARPC5
84517	1.0	1.0	1.0	1.0	ARPM1
10777	1.0	7.7	1.0	1.0	ARPP21
57561	1.0	4.3	1.0	1.0	ARRDC3
91947	1.0	1.0	1.0	1.0	ARRDC4
645432	1.0	5.6	4.9	1.0	ARRDC5
411	1.0	1.0	1.0	1.0	ARSB
414	-2.6	1.0	-4.9	-4.9	ARSD
340075	1.0	1.0	2.4	1.0	ARSI
79642	1.0	1.0	1.0	-3.1	ARSJ
420	1.0	3.4	2.6	2.9	ART4
116969	1.0	1.0	1.0	1.0	ART5
64801	-9.4	-1.9	-6.3	-2.8	ARV1
421	1.6	1.0	1.0	-3.8	ARVCF
442092	1.0	1.0	3.3	1.0	ARVP6125
57412	1.0	-2.9	-3.1	1.0	AS3MT
56624	1.0	1.0	3.1	1.0	ASAH2
55616	1.0	1.0	-1.5	1.0	ASAP3
51665	-1.8	1.0	1.0	1.0	ASB1
136371	1.0	1.0	1.0	1.0	ASB10
140456	1.0	1.0	1.0	1.0	ASB11
92591	1.6	1.0	1.0	1.0	ASB16
140461	-11.7	-3.4	-4.1	1.0	ASB8
84164	1.8	1.0	1.0	1.0	ASCC2
10973	1.0	1.0	1.0	-2.6	ASCC3
430	1.0	1.0	1.0	1.0	ASCL2
55723	52.5	1.0	1.0	1.0	ASF1B
432	1.0	1.0	1.0	1.0	ASGR1
55870	1.0	1.0	1.0	1.0	ASH1L
54529	1.0	1.0	1.0	1.0	ASNSD1
444	1.0	-2.5	-2.6	1.0	ASPH
253982	1.0	1.0	-4.4	1.0	ASPHD1
57168	1.0	1.0	1.0	3.2	ASPHD2
54829	1.0	-4.2	-4.6	1.0	ASPN
151516	1.0	1.0	1.0	1.0	ASPRV1
79058	1.0	1.0	1.7	1.0	ASPSCR1
445	1.0	1.0	-1.9	1.0	ASS1
431705	4.1	1.0	1.0	1.0	ASTL
23245	1.0	4.0	1.0	1.0	ASTN2
55252	-13.0	1.0	1.0	2.8	ASXL2
80816	1.0	1.0	1.0	1.0	ASXL3
54454	1.0	5.6	7.0	1.0	ATAD2B

83858	1.0	1.0	2.8	2.9	ATAD3B
219293	1.0	1.0	1.0	1.0	ATAD3C
11101	1.0	1.0	1.0	-2.1	ATE1
466	1.0	1.0	1.0	1.0	ATF1
1386	1.0	1.0	1.0	1.0	ATF2
467	1.0	1.0	1.0	1.0	ATF3
80063	1.0	-1.8	1.0	1.0	ATF7IP2
9140	1.0	1.0	-5.0	1.0	ATG12
9776	2.4	2.5	1.0	2.1	ATG13
22863	1.0	1.0	1.0	1.0	ATG14
55054	1.0	1.0	1.0	1.0	ATG16L1
84938	-3.5	1.0	1.0	1.0	ATG4C
9474	16.1	4.3	3.7	3.3	ATG5
51062	2.1	1.0	1.0	1.0	ATL1
64225	1.0	3.2	1.0	5.5	ATL2
472	1.0	-2.9	2.3	1.0	ATM
23300	1.0	1.0	-5.6	-3.4	ATMIN
84913	1.0	3.3	1.0	4.2	ATOH8
23120	1.0	1.0	-5.6	1.0	ATP10B
57205	1.0	1.0	1.0	1.0	ATP10D
23200	1.0	1.0	4.9	1.0	ATP11B
84239	1.0	1.0	1.0	1.0	ATP13A4
84852	1.0	1.0	1.0	1.0	ATP1A1OS
477	1.0	1.0	1.0	1.0	ATP1A2
478	-4.5	1.0	1.0	1.0	ATP1A3
480	1.0	1.0	1.0	1.8	ATP1A4
487	1.0	1.0	-3.4	2.5	ATP2A1
488	1.0	1.0	-7.2	1.0	ATP2A2
491	1.0	-1.9	-9.2	-4.0	ATP2B2
492	-4.7	-3.6	1.0	1.0	ATP2B3
493	1.0	-2.3	1.0	1.0	ATP2B4
9914	1.0	1.0	1.0	1.0	ATP2C2
495	1.0	1.0	1.0	3.4	ATP4A
496	1.0	1.0	1.0	1.0	ATP4B
521	3.0	1.0	1.0	1.0	ATP5I
55101	3.3	4.2	1.0	4.2	ATP5SL
9114	2.5	1.0	1.0	1.0	ATP6V0D1
155066	1.0	1.0	1.0	1.0	ATP6V0E2
525	1.0	1.0	1.0	1.0	ATP6V1B1
538	1.0	1.0	1.0	1.0	ATP7A
540	1.0	3.2	1.9	1.0	ATP7B
5205	1.0	1.0	1.0	1.0	ATP8B1
148229	1.0	1.0	-7.1	1.0	ATP8B3
158381	1.0	1.0	1.0	-2.5	ATP8B5P
374868	1.0	1.0	1.0	1.0	ATP9B
64756	1.0	1.0	6.1	4.8	ATPAF1
374973	1.0	1.0	1.0	1.0	ATPAF1-AS1
91647	1.0	1.0	-2.8	1.0	ATPAF2

93974	1.0	4.5	3.7	1.0	ATPIF1
84126	1.0	6.4	1.0	1.0	ATRIP
26033	1.0	3.0	1.0	1.0	ATRNL1
546	-4.1	-4.1	1.0	-4.0	ATRX
6310	1.0	1.0	1.0	1.0	ATXN1
6311	1.0	-2.9	1.0	1.0	ATXN2
11273	1.0	1.0	-5.1	1.0	ATXN2L
92552	1.0	1.0	1.0	-2.2	ATXN3L
6314	1.0	3.6	6.5	1.0	ATXN7
127002	3.8	1.0	1.0	1.0	ATXN7L2
550	1.0	1.0	1.0	1.0	AUP1
6790	1.0	1.0	1.0	1.0	AURKA
26053	1.0	1.0	1.0	1.0	AUTS2
57099	1.0	1.0	-2.4	1.0	AVEN
23080	1.0	-2.3	-2.5	1.0	AVL9
551	1.0	1.0	1.0	1.0	AVP
158833	1.0	1.0	1.0	1.0	AWAT1
8313	1.0	5.0	4.0	1.0	AXIN2
563	1.0	1.0	1.0	1.0	AZGP1
22994	1.0	1.0	1.0	1.0	AZI1
64343	1.0	1.0	-4.3	1.0	AZI2
567	1.0	1.0	1.0	1.0	B2M
8706	1.0	1.0	1.0	1.0	B3GALNT1
8707	1.0	1.0	-2.4	1.0	B3GALT2
8705	1.0	1.0	1.0	1.0	B3GALT4
126792	1.0	-3.9	-2.6	-3.6	B3GALT6
11041	1.0	1.0	1.0	1.0	B3GNT1
10331	1.0	1.0	1.0	4.9	B3GNT3
79369	1.0	1.0	5.3	3.5	B3GNT4
192134	1.0	1.0	1.0	1.0	B3GNT6
84752	1.0	6.5	6.0	5.6	B3GNT9
2583	1.7	1.0	2.3	1.0	B4GALNT1
338707	1.0	1.0	-5.8	1.0	B4GALNT4
8704	1.0	1.0	1.0	1.0	B4GALT2
27077	2.1	-4.8	-5.4	-2.5	B9D1
80776	1.0	1.0	-4.4	1.0	B9D2
79870	1.0	1.0	1.0	1.0	BAALC
29086	1.0	1.0	1.0	3.9	BABAM1
23621	1.0	1.0	1.0	1.0	BACE1
60468	1.0	1.9	1.0	1.0	BACH2
572	1.0	2.4	1.0	1.0	BAD
9530	2.3	-3.6	-3.9	-6.3	BAG4
574	1.9	1.0	4.1	1.0	BAGE
85317	1.0	1.0	1.0	1.0	BAGE4
575	1.6	1.0	1.0	4.2	BAI1
576	2.3	1.0	1.0	1.0	BAI2
10458	1.0	1.0	1.0	5.1	BAIAP2
55971	1.0	-3.5	-5.0	1.0	BAIAP2L1

8815	1.0	2.5	1.0	1.0	BANF1
54971	1.6	-4.7	1.0	-4.4	BANP
56751	1.0	2.4	1.0	1.0	BARHL1
343472	1.5	1.0	-4.9	1.0	BARHL2
8538	1.0	1.0	1.0	1.0	BARX2
55509	1.0	1.8	1.0	1.0	BATF3
581	1.0	1.0	1.0	-2.9	BAX
27113	-1.6	1.0	1.0	-3.2	BBC3
92482	1.0	1.0	1.0	1.0	BBIP1
582	1.0	1.0	-2.3	1.0	BBS1
585	1.5	1.0	1.0	1.0	BBS4
129880	1.0	1.0	-2.5	-2.8	BBS5
10134	1.0	1.0	1.0	1.0	BCAP31
10286	1.0	1.0	1.0	1.0	BCAS2
586	-1.9	1.0	1.0	3.2	BCAT1
587	1.0	1.0	1.0	1.0	BCAT2
144233	1.0	1.0	3.5	1.0	BCDIN3D
590	1.0	1.0	1.0	1.0	BCHE
594	1.0	1.0	1.0	1.0	BCKDHB
53335	1.0	1.0	1.0	1.0	BCL11A
64919	1.0	1.0	-2.5	-2.5	BCL11B
596	1.0	1.0	1.0	-2.5	BCL2
83596	1.9	1.0	1.0	1.0	BCL2L12
23786	1.0	1.0	3.6	3.3	BCL2L13
79370	1.0	-2.5	-3.4	1.0	BCL2L14
440603	1.0	2.4	3.2	1.0	BCL2L15
9274	1.7	1.0	1.0	1.0	BCL7C
283149	1.0	1.0	1.0	1.0	BCL9L
9774	1.0	1.0	1.0	1.0	BCLAF1
54880	2.2	1.0	-11.7	1.0	BCOR
613	1.7	1.0	1.0	1.0	BCR
400892	1.0	1.0	1.0	-2.6	BCRP2
8678	-2.6	1.0	1.0	1.0	BECN1
57596	1.0	1.0	1.0	1.0	BEGAIN
57673	1.0	1.0	-2.4	-2.4	BEND3
222389	1.0	1.0	1.0	1.0	BEND7
7439	1.5	1.0	-2.0	-1.5	BEST1
144453	1.0	1.0	1.0	1.0	BEST3
266675	1.7	2.3	1.0	2.2	BEST4
10282	-1.6	-2.6	-2.5	1.0	BET1
51272	1.5	1.0	-3.8	1.0	BET1L
631	1.0	1.0	1.0	1.0	BFSP1
8419	1.0	1.0	1.0	1.0	BFSP2
632	3.2	-2.9	1.0	1.0	BGLAP
633	1.0	1.0	1.0	2.0	BGN
8553	1.0	1.0	1.0	1.0	BHLHE40
636	1.0	1.0	1.0	1.0	BICD1
23299	1.0	1.9	1.0	1.0	BICD2

51411	1.0	-5.1	-7.4	-3.4	BIN2
329	1.0	-5.4	1.0	-5.0	BIRC2
57448	1.0	1.0	1.0	1.0	BIRC6
54841	6.9	1.0	9.9	1.0	BIVM
641	1.0	1.0	1.0	1.0	BLM
642	1.0	1.0	3.0	1.0	BLMH
90427	1.0	1.0	2.6	1.0	BMF
649	1.0	1.0	1.0	1.0	BMP1
27302	1.0	1.0	-1.7	1.0	BMP10
650	1.0	1.0	1.0	1.0	BMP2
653	1.0	1.0	1.0	1.0	BMP5
655	1.0	1.0	1.0	-6.4	BMP7
353500	1.0	1.0	1.0	1.0	BMP8A
168667	1.0	1.0	1.0	1.0	BMPER
9790	-2.5	-3.9	1.0	1.0	BMS1
54796	1.0	-2.9	1.0	1.0	BNC2
662	1.0	5.4	1.0	1.0	BNIP1
665	1.0	2.5	-1.7	-4.0	BNIP3L
91653	1.0	1.0	1.0	1.0	BOC
284257	1.0	1.0	1.0	1.0	BOD1P
388962	1.0	1.0	1.0	1.0	BOLA3
23246	5.5	1.0	1.0	1.0	BOP1
669	1.0	1.0	1.0	1.0	BPGM
670	1.0	1.0	1.0	1.0	BPHL
51297	1.0	1.0	1.0	4.6	BPIFA1
317716	1.0	1.0	1.0	1.0	BPIFA4P
80341	1.0	6.4	1.0	1.0	BPIFB2
10380	1.0	1.0	-3.5	-3.6	BPNT1
673	1.0	1.0	1.0	1.0	BRAF
23774	1.9	1.0	1.0	-2.5	BRD1
8019	-3.4	1.0	4.7	1.0	BRD3
29117	1.0	2.1	1.0	1.0	BRD7
23629	-16.3	1.0	1.0	-5.9	BRD7P3
10902	1.0	1.0	1.0	1.0	BRD8
65980	1.0	2.1	1.0	1.0	BRD9
9577	-3.7	1.0	1.0	1.0	BRE
2972	3.3	1.0	1.0	1.0	BRF1
55290	1.0	1.0	1.0	-4.7	BRF2
140707	1.0	1.0	3.2	1.0	BRI3BP
55845	1.0	1.0	1.0	1.0	BRK1
148362	-3.7	-2.9	-1.8	1.0	BROX
51660	1.0	2.2	1.0	1.0	BRP44L
680	1.0	1.0	1.0	1.0	BRS3
84446	1.0	1.0	1.0	1.0	BRSK1
9024	1.0	2.5	3.9	3.6	BRSK2
54014	1.7	1.0	1.0	1.6	BRWD1
682	1.0	1.0	1.0	1.0	BSG
8927	1.0	1.9	1.0	1.0	BSN

7809	1.0	1.0	1.0	1.0	BSND
684	1.0	1.0	4.9	3.7	BST2
90135	1.0	1.0	1.0	1.0	BTBD6
55727	1.0	1.0	1.0	1.0	BTBD7
114781	1.0	4.4	1.0	1.0	BTBD9
10950	1.0	5.7	6.1	1.0	BTG3
54766	1.0	1.0	1.0	1.0	BTG4
11118	1.0	1.0	1.0	1.0	BTN3A2
79908	1.0	1.0	1.0	1.0	BTNL8
8945	2.2	1.0	5.5	4.4	BTRC
84811	1.0	2.6	2.8	2.5	BUD13
705	1.0	1.0	1.0	1.0	BYSL
770	1.0	3.3	1.0	1.0	CA11
760	1.0	1.0	3.0	1.0	CA2
762	1.0	1.0	1.0	1.0	CA4
340591	1.0	1.0	1.0	1.0	CA5BP1
765	9.4	6.2	1.0	6.4	CA6
767	1.0	1.0	1.0	1.0	CA8
23523	3.3	1.0	3.0	1.0	CABIN1
57010	1.0	1.0	1.0	1.0	CABP4
26256	1.0	1.0	-6.5	1.0	CABYR
57685	5.1	1.0	2.2	1.0	CACHD1
774	1.0	3.5	4.4	1.0	CACNA1B
775	1.0	-7.9	-14.2	-9.3	CACNA1C
777	1.8	1.0	-3.1	-3.2	CACNA1E
778	1.0	1.8	1.0	1.0	CACNA1F
781	1.0	1.0	1.0	-2.5	CACNA2D1
784	1.0	1.0	1.0	1.0	CACNB3
786	1.0	1.0	1.0	1.0	CACNG1
59285	1.0	2.2	1.0	1.0	CACNG6
59283	1.0	-7.7	1.0	1.0	CACNG8
27101	1.0	1.0	-7.8	1.0	CACYBP
23705	1.0	1.0	1.0	-3.6	CADM1
57863	-2.1	1.0	4.0	1.0	CADM3
8618	1.0	1.0	1.0	1.0	CADPS
285782	1.0	-3.5	-3.7	1.0	CAGE1
796	1.0	2.0	1.0	1.0	CALCA
10203	1.0	1.0	-1.5	-4.7	CALCRL
255022	1.0	5.9	5.7	3.2	CALHM1
808	-1.6	1.0	1.0	-3.5	CALM3
91860	2.6	1.0	1.0	1.0	CALML4
163688	1.0	1.0	1.0	1.0	CALML6
811	1.0	1.0	1.0	1.0	CALR
813	6.9	8.6	8.1	8.0	CALU
8536	1.0	1.0	1.0	1.0	CAMK1
57118	1.0	1.0	2.6	1.0	CAMK1D
57172	1.0	1.0	1.0	2.3	CAMK1G
815	1.0	1.0	1.0	5.2	CAMK2A

818	1.0	1.0	1.0	1.0	CAMK2G
79823	2.7	1.0	-5.8	1.0	CAMKMT
79012	1.0	1.0	1.6	1.0	CAMKV
157922	1.0	1.0	5.6	1.0	CAMSAP1
23271	2.2	-4.9	-4.8	1.0	CAMSAP2
57662	1.0	1.0	1.0	1.0	CAMSAP3
23261	-2.3	1.0	1.0	4.4	CAMTA1
55832	1.5	1.0	3.3	2.7	CAND1
124583	2.2	1.0	2.2	1.0	CANT1
821	1.0	-2.5	1.0	1.0	CANX
10487	-1.6	-2.8	-2.8	1.0	CAP1
822	3.9	-3.3	1.0	1.0	CAPG
11132	-2.5	1.0	-44.2	1.0	CAPN10
147968	-2.1	1.0	1.0	1.0	CAPN12
824	1.0	1.0	2.3	1.0	CAPN2
726	-2.1	-5.9	-6.1	-4.6	CAPN5
10753	1.0	1.0	1.0	1.0	CAPN9
826	1.0	1.0	1.0	1.0	CAPNS1
84290	1.0	1.0	1.0	1.0	CAPNS2
65981	1.0	1.0	1.0	1.0	CAPRIN2
828	1.0	1.0	1.0	1.0	CAPS
829	-2.3	1.0	1.0	2.5	CAPZA1
830	1.0	1.0	1.0	1.0	CAPZA2
93661	4.0	1.0	1.0	1.0	CAPZA3
29775	1.0	4.3	1.0	4.5	CARD10
84433	1.0	1.0	-3.0	1.0	CARD11
114769	1.0	1.0	1.0	1.0	CARD16
440068	1.0	1.0	-3.4	1.0	CARD17
64170	1.0	1.0	1.0	1.0	CARD9
833	-4.1	1.0	1.0	-3.0	CARS
255082	1.0	1.0	1.0	1.0	CASC2
113201	1.0	1.0	-1.8	1.0	CASC4
57082	1.0	1.0	1.0	1.0	CASC5
57524	1.0	1.0	1.0	1.0	CASKIN1
834	1.0	1.0	1.0	1.0	CASP1
841	1.0	1.0	-7.0	1.0	CASP8
846	1.0	1.0	1.0	1.0	CASR
57091	1.0	1.0	1.0	1.0	CASS4
54897	1.0	1.0	-2.7	-2.7	CASZ1
847	1.0	1.0	1.0	1.0	CAT
117155	1.0	1.0	1.0	1.0	CATSPER2
378807	-1.8	1.0	1.0	1.0	CATSPER4
858	6.6	1.0	1.0	3.6	CAV2
9139	1.0	2.2	1.0	1.0	CBFA2T2
863	1.0	1.0	1.0	1.0	CBFA2T3
867	1.0	1.0	1.0	1.0	CBL
868	-28.7	1.0	1.0	1.0	CBLB
140689	-26.2	1.0	1.0	2.4	CBLN4

873	1.0	3.5	1.0	1.0	CBR1
875	1.0	1.0	5.9	5.0	CBS
220869	1.0	1.0	-2.5	-2.5	CBWD5
10951	1.7	1.0	-2.4	-2.3	CBX1
8535	1.9	1.0	1.0	3.9	CBX4
23468	1.0	1.0	1.0	1.0	CBX5
23466	5.7	1.0	1.0	1.0	CBX6
25776	1.0	1.0	1.0	1.0	CBY1
54862	7.0	1.0	-5.9	-2.8	CC2D1A
200014	2.0	1.0	1.0	1.0	CC2D1B
57545	1.0	1.0	1.0	1.0	CC2D2A
387707	1.0	1.0	4.0	1.0	CC2D2B
55749	1.0	1.0	1.0	1.0	CCAR1
92922	54.0	1.0	1.0	1.0	CCDC102A
79839	1.0	2.9	1.0	1.0	CCDC102B
112942	1.0	1.0	1.0	-3.8	CCDC104
255101	-2.1	-4.4	-3.7	-3.9	CCDC108
220136	1.0	-3.1	1.0	-5.7	CCDC11
256309	1.0	1.0	-3.2	1.0	CCDC110
84317	5.2	1.0	6.1	1.0	CCDC115
164592	1.0	1.0	-3.1	1.0	CCDC116
151903	1.0	1.0	-1.6	1.0	CCDC12
79635	1.0	1.0	1.0	1.0	CCDC121
202243	-5.5	1.0	1.0	1.0	CCDC125
90693	1.0	1.0	1.0	-2.4	CCDC126
81576	1.0	4.3	3.9	3.7	CCDC130
55610	1.0	1.0	1.0	1.0	CCDC132
84229	1.0	1.0	1.0	1.0	CCDC135
64770	1.0	1.0	1.0	1.0	CCDC14
285025	1.0	1.0	1.0	-3.1	CCDC141
9720	1.0	-6.4	1.0	1.0	CCDC144A
57639	1.0	1.0	-2.7	-3.1	CCDC146
91050	2.6	1.0	1.0	1.0	CCDC149
284992	1.0	1.0	1.0	1.0	CCDC150
283152	1.0	-4.1	-5.2	1.0	CCDC153
339965	1.0	1.0	1.0	1.0	CCDC158
643677	1.0	1.0	1.0	1.0	CCDC168
149483	1.0	1.0	3.0	1.0	CCDC17
343099	1.0	1.0	6.3	5.1	CCDC18
25790	1.7	1.0	1.0	1.0	CCDC19
149473	1.0	1.0	1.0	1.0	CCDC24
148870	1.0	-2.2	-1.7	1.0	CCDC27
79140	1.0	1.0	1.0	-3.9	CCDC28B
83643	1.0	2.3	1.0	1.0	CCDC3
728621	1.0	1.0	1.0	-2.8	CCDC30
51134	-2.0	-2.7	-3.0	1.0	CCDC41
79825	1.0	-6.2	1.0	1.0	CCDC48
152137	-4.4	1.0	2.4	1.0	CCDC50

28958	1.0	-2.5	1.0	1.0	CCDC56
284001	-14.9	1.0	4.6	4.2	CCDC57
8030	1.0	3.6	3.7	1.0	CCDC6
160777	1.0	1.0	3.4	1.0	CCDC60
85478	1.0	1.0	1.0	1.0	CCDC65
26112	3.8	1.0	2.4	1.0	CCDC69
51372	1.0	1.0	5.2	1.0	CCDC72
493860	1.0	-2.0	1.0	1.0	CCDC73
91409	1.0	-5.0	-5.6	1.0	CCDC74B
253635	1.0	1.0	1.0	1.0	CCDC75
84318	1.0	5.2	1.0	5.0	CCDC77
83987	1.0	3.1	1.0	1.0	CCDC8
60494	1.0	31.0	1.0	1.0	CCDC81
338657	1.0	-3.7	-7.5	-4.8	CCDC84
11007	1.0	-2.9	-2.6	-2.6	CCDC85B
55231	1.0	3.1	3.5	1.0	CCDC87
55704	-1.6	1.0	1.0	1.0	CCDC88A
283234	3.6	1.0	4.9	-2.7	CCDC88B
440193	1.9	1.0	24.4	1.0	CCDC88C
26093	-1.6	1.0	2.5	1.0	CCDC9
63933	1.0	1.0	-2.5	1.0	CCDC90A
90324	-2.1	1.0	1.0	1.0	CCDC97
54908	1.0	1.0	1.0	1.0	CCDC99
54535	1.0	1.0	-2.0	-2.0	CCHCR1
885	1.0	1.0	1.0	1.0	CCK
887	1.0	1.0	1.0	-3.1	CCKBR
6363	3.2	1.0	2.4	1.0	CCL19
6347	1.0	1.8	1.0	1.0	CCL2
6364	5.5	9.2	10.1	1.0	CCL20
56477	-2.3	-3.3	-4.6	1.0	CCL28
6348	1.0	1.0	1.0	1.0	CCL3
6351	4.3	1.0	4.8	1.0	CCL4
6355	1.0	1.0	-5.2	1.0	CCL8
891	1.0	-2.4	-2.2	-2.2	CCNB1
9133	1.7	1.0	1.0	1.0	CCNB2
85417	1.0	1.0	1.0	1.0	CCNB3
595	1.0	2.8	1.0	1.0	CCND1
894	1.0	1.0	1.0	1.0	CCND2
898	-4.3	1.0	-2.3	-2.3	CCNE1
9134	1.0	4.7	4.5	3.2	CCNE2
902	1.0	1.0	-5.0	1.0	CCNH
57018	1.0	1.0	-3.5	1.0	CCNL1
10309	1.0	1.0	1.0	1.0	CCNO
904	1.0	-3.6	-4.6	-3.5	CCNT1
2826	1.0	1.0	1.0	-5.3	CCR10
1232	1.0	1.0	1.0	1.0	CCR3
1234	1.0	1.0	1.0	-2.2	CCR5
25819	1.0	1.0	1.0	1.0	CCRN4L

10575	2.3	1.0	1.0	-2.7	CCT4
908	1.0	1.0	1.0	1.0	CCT6A
10693	1.0	1.0	1.0	1.0	CCT6B
10574	4.6	1.0	1.0	1.0	CCT7
10694	1.0	1.0	1.0	-2.4	CCT8
51622	1.0	1.0	1.0	1.0	CCZ1
11126	1.0	1.0	1.0	1.0	CD160
9332	1.0	1.0	1.0	2.0	CD163
8763	1.0	-5.5	1.0	1.0	CD164
57126	5.8	1.0	1.0	1.0	CD177
930	1.0	1.0	1.0	1.0	CD19
910	1.0	1.0	1.0	1.0	CD1B
912	-6.2	1.0	1.0	1.0	CD1D
913	-5.7	1.0	4.7	1.0	CD1E
914	1.0	1.0	1.0	1.0	CD2
4345	1.0	1.0	1.0	1.0	CD200
131450	1.0	-4.5	-7.1	-5.6	CD200R1
50489	1.0	3.2	1.0	1.0	CD207
30835	1.0	1.0	1.0	1.0	CD209
51744	1.0	1.0	1.0	1.0	CD244
939	1.0	1.0	-5.2	-5.2	CD27
29126	1.0	1.0	1.0	1.0	CD274
940	5.5	1.0	1.0	1.0	CD28
10421	15.1	1.0	1.0	1.0	CD2BP2
11314	1.5	1.0	1.0	1.0	CD300A
342510	1.0	5.5	3.7	5.4	CD300E
124599	1.0	1.0	1.0	1.0	CD300LB
146894	1.0	1.0	3.2	1.0	CD300LG
51293	1.0	1.0	1.0	1.0	CD320
945	1.0	1.0	1.0	1.0	CD33
947	1.0	3.3	1.0	1.0	CD34
948	1.0	-2.3	1.0	1.0	CD36
951	2.2	-3.3	1.0	2.6	CD37
959	1.0	1.0	3.1	1.0	CD40LG
961	1.0	2.9	1.0	1.0	CD47
921	1.0	1.0	1.0	-5.3	CD5
1604	1.0	-1.7	-6.6	1.0	CD55
923	-1.8	1.0	2.4	1.0	CD6
971	1.0	1.0	1.0	1.0	CD72
974	1.0	5.3	1.0	1.0	CD79B
975	1.0	1.0	1.0	5.6	CD81
8832	1.0	2.8	1.0	2.7	CD84
942	1.0	-2.7	-3.3	-2.9	CD86
926	1.0	1.0	1.0	1.0	CD8B
928	1.0	1.0	1.0	1.0	CD9
10225	1.0	1.0	-5.6	1.0	CD96
976	1.0	1.0	1.0	1.0	CD97
4267	-6.1	1.0	1.0	1.0	CD99

83692	1.0	2.6	1.8	3.1	CD99L2
81602	-25.9	-2.9	-3.0	1.0	CDADC1
8555	1.0	1.0	-7.4	-3.8	CDC14B
8697	1.0	1.0	1.0	1.0	CDC23
993	1.0	1.0	1.0	2.2	CDC25A
994	1.0	1.0	1.0	1.0	CDC25B
995	1.0	1.0	1.0	-3.4	CDC25C
11140	1.0	1.0	2.8	1.0	CDC37
51362	2.4	1.0	1.0	1.0	CDC40
8476	1.0	1.0	1.0	1.0	CDC42BPA
11135	1.8	1.0	1.0	1.0	CDC42EP1
10435	1.0	1.0	1.0	5.7	CDC42EP2
990	1.0	3.4	1.0	3.0	CDC6
157313	1.0	1.0	1.0	1.0	CDCA2
113130	1.0	1.0	2.2	2.3	CDCA5
55143	1.0	1.0	1.0	1.0	CDCA8
64866	1.0	1.0	2.0	1.0	CDCP1
1010	2.7	1.0	7.1	1.0	CDH12
1016	1.0	1.0	1.0	1.0	CDH18
1000	1.0	1.0	1.0	1.0	CDH2
64072	1.0	1.0	1.0	1.0	CDH23
64403	1.0	1.0	1.0	3.3	CDH24
1001	2.1	2.8	1.0	1.0	CDH3
1002	1.0	1.0	1.0	1.0	CDH4
1004	-3.3	1.0	6.8	6.2	CDH6
1005	1.0	1.0	1.0	1.0	CDH7
92211	1.0	1.0	1.0	7.5	CDHR1
222256	1.0	1.0	1.0	1.0	CDHR3
8558	2.8	-2.7	1.0	2.7	CDK10
51755	1.0	1.0	1.0	1.0	CDK12
8621	1.0	1.0	1.0	1.0	CDK13
5127	1.0	1.0	1.0	-5.6	CDK16
5128	3.6	1.0	1.0	1.0	CDK17
5129	1.0	5.1	1.0	1.0	CDK18
8099	1.0	1.7	1.0	2.9	CDK2AP1
10263	1.0	1.6	1.0	1.0	CDK2AP2
1018	1.0	1.0	1.0	1.0	CDK3
1019	3.1	1.0	3.7	2.4	CDK4
51654	1.0	2.8	3.5	1.0	CDK5RAP1
55755	-5.6	1.0	1.0	1.0	CDK5RAP2
1022	1.0	1.0	1.0	-4.8	CDK7
8814	1.0	1.0	1.0	1.0	CDKL1
8999	1.0	1.0	1.0	-3.4	CDKL2
51265	2.5	2.6	2.7	2.5	CDKL3
344387	1.0	1.0	1.0	1.0	CDKL4
6792	1.0	-3.6	1.0	1.0	CDKL5
1026	1.0	1.0	1.0	1.0	CDKN1A
1027	1.0	1.0	1.0	1.0	CDKN1B

1031	1.8	1.0	1.0	1.0	CDKN2C
1033	1.0	1.0	-2.6	1.0	CDKN3
30850	1.0	1.0	1.0	1.0	CDR2L
374286	1.0	1.0	1.0	1.0	CDRT1
1040	1.0	1.0	1.0	1.0	CDS1
8760	1.0	1.0	1.0	1.0	CDS2
1041	1.0	-1.6	-4.1	1.0	CDSN
81620	1.0	1.0	1.0	1.0	CDT1
1044	1.0	1.0	1.0	1.0	CDX1
1046	1.0	-2.9	-2.8	-2.8	CDX4
634	14.8	1.0	2.7	1.0	CEACAM1
125931	1.0	1.0	1.0	1.0	CEACAM20
1087	1.0	1.0	2.7	1.0	CEACAM7
1088	1.0	1.0	1.0	1.0	CEACAM8
1054	1.0	5.3	5.0	1.0	CEBPG
27440	1.0	1.0	1.0	1.0	CECR5
100130717	1.0	1.0	1.0	1.0	CECR5-AS1
27439	1.0	1.0	1.0	3.6	CECR6
1056	1.0	-3.2	-3.1	-3.1	CEL
1990	1.0	1.0	-2.9	-3.4	CELA1
10659	1.0	1.0	2.4	1.0	CELF2
9620	1.0	1.0	1.0	1.0	CELSR1
51286	2.3	1.0	1.0	1.0	CEND1
1059	1.0	-3.5	1.0	1.0	CENPB
92806	1.0	1.0	1.0	1.0	CENPBD1
1062	1.0	2.4	1.0	1.0	CENPE
1063	1.0	1.0	1.0	1.0	CENPF
64946	1.0	1.0	1.0	1.0	CENPH
2491	-4.5	1.0	-7.6	4.9	CENPI
79172	1.0	1.8	1.0	1.0	CENPO
401541	-12.0	1.0	1.0	1.0	CENPP
80152	1.0	1.0	1.0	1.0	CENPT
9731	1.0	1.0	1.0	2.5	CEP104
153241	1.0	1.0	5.7	1.0	CEP120
145508	1.0	1.0	1.0	1.0	CEP128
9662	1.0	1.0	1.0	5.6	CEP135
22897	1.0	1.0	3.1	1.0	CEP164
9859	1.0	1.0	1.0	1.0	CEP170
84984	1.0	1.0	1.0	1.0	CEP19
55125	1.0	1.0	5.8	4.7	CEP192
11190	1.9	1.0	1.0	1.0	CEP250
55165	12.2	1.0	1.0	1.0	CEP55
9702	1.0	1.0	1.0	1.0	CEP57
285753	1.0	-2.9	1.0	-2.6	CEP57L1
80321	1.0	1.0	1.0	1.0	CEP70
55722	1.0	4.5	3.3	1.0	CEP72
79959	1.0	1.0	3.7	3.6	CEP76
64793	-2.4	1.0	-3.7	1.0	CEP85

10390	1.0	1.0	1.0	1.0	CEPT1
64781	1.0	1.0	3.1	1.0	CERK
204219	28.9	1.0	1.0	1.0	CERS3
1066	-6.7	1.0	1.0	2.8	CES1
51716	1.0	-6.9	1.0	1.0	CES1P1
55997	1.0	1.0	1.0	1.0	CFC1
1675	-1.6	2.2	1.6	1.0	CFD
10428	-4.0	1.0	-5.4	1.0	CFDP1
10878	1.0	1.0	-2.2	1.0	CFHR3
142913	3.0	-5.2	1.0	1.0	CFL1P1
8837	1.0	1.0	6.3	1.0	CFLAR
5199	2.5	1.0	1.0	1.0	CFP
79094	1.0	1.0	3.9	1.0	CHAC1
494143	1.0	1.0	1.0	1.0	CHAC2
1101	-2.1	1.0	1.0	1.0	CHAD
150356	1.0	1.7	1.0	1.0	CHADL
283489	1.0	1.0	1.0	2.7	CHAMP1
1103	-2.1	1.0	1.0	1.0	CHAT
84269	2.8	-3.1	1.0	1.0	CHCHD5
84303	1.0	-6.9	1.0	1.0	CHCHD6
79145	1.0	1.0	1.0	1.0	CHCHD7
9557	1.0	1.0	4.2	1.0	CHD1L
1106	4.8	-3.3	5.2	6.3	CHD2
84181	1.0	1.0	1.0	1.0	CHD6
55636	1.0	1.0	1.0	1.0	CHD7
57680	1.0	1.0	1.0	1.0	CHD8
11200	1.0	1.0	-3.9	1.0	CHEK2
10523	1.0	1.0	1.0	1.0	CHERP
1116	1.0	-6.3	1.0	1.0	CHI3L1
66005	2.2	-2.5	1.0	1.0	CHID1
5119	1.0	1.0	4.6	2.1	CHMP1A
51652	1.0	-3.7	-4.0	1.0	CHMP3
51510	1.0	4.6	1.0	1.0	CHMP5
79643	1.0	1.0	1.0	1.0	CHMP6
1124	1.0	-7.1	-7.5	1.0	CHN2
26973	1.0	1.0	1.0	1.0	CHORDC1
63928	1.0	1.0	2.5	1.0	CHP2
54480	-4.8	1.0	1.0	1.0	CHPF2
56994	1.0	2.0	2.5	1.6	CHPT1
8646	1.7	1.0	1.0	1.0	CHRD
25884	1.0	1.0	2.6	1.6	CHRDL2
1129	1.0	1.0	1.0	1.0	CHRM2
1131	1.6	1.0	-3.2	1.0	CHRM3
1133	1.0	1.0	2.7	2.4	CHRM5
1135	1.0	2.7	1.0	1.0	CHRNA2
1136	1.0	-3.8	-3.4	1.0	CHRNA3
1138	1.0	1.0	1.0	2.9	CHRNA5
55584	1.0	1.0	1.0	1.0	CHRNA9

1142	1.0	3.3	3.1	3.2	CHRNB3
166012	-2.5	1.0	1.0	1.0	CHST13
113189	9.2	1.0	3.8	1.0	CHST14
10164	1.0	1.0	1.0	-6.2	CHST4
26097	1.0	1.0	1.0	1.0	CHTOP
10518	1.8	1.0	3.7	1.0	CIB2
63924	1.0	1.0	1.0	1.7	CIDEC
152302	1.0	1.0	1.0	-4.1	CIDECP
4261	1.0	1.0	1.0	1.0	CIITA
1153	1.6	1.0	1.0	1.0	CIRBP
55847	1.0	-5.2	-6.4	-5.3	CISD1
4435	1.0	1.0	1.0	1.0	CITED1
25792	1.0	-6.5	1.0	1.0	CIZ1
150468	1.0	1.0	1.0	1.0	CKAP2L
548596	1.0	1.0	1.0	1.0	CKMT1A
1163	1.0	1.0	1.0	1.0	CKS1B
1164	1.0	1.0	1.0	1.0	CKS2
23332	6.8	1.0	1.0	1.0	CLASP1
23122	1.0	1.0	1.0	1.0	CLASP2
1178	1.0	-2.1	-2.6	-2.2	CLC
9629	1.0	1.0	1.0	1.0	CLCA3P
22802	1.0	1.0	1.0	1.0	CLCA4
23529	1.0	1.0	6.2	1.0	CLCF1
1180	1.0	1.0	1.0	1.0	CLCN1
1184	1.0	6.4	1.0	1.0	CLCN5
1185	1.0	1.0	1.0	1.0	CLCN6
1188	1.0	1.0	1.0	1.0	CLCNKB
9071	1.0	1.0	1.0	1.0	CLDN10
23562	1.0	1.0	1.0	3.4	CLDN14
10686	1.0	5.4	6.1	1.0	CLDN16
26285	1.0	-3.4	-3.1	1.0	CLDN17
53842	1.0	-4.3	1.0	-7.5	CLDN22
1364	1.0	1.0	1.0	1.0	CLDN4
9080	1.7	1.0	3.4	1.0	CLDN9
56650	1.0	3.3	1.0	3.8	CLDND1
125875	1.0	-3.5	1.0	-3.6	CLDND2
10462	2.0	1.0	1.0	1.0	CLEC10A
160364	1.0	1.0	-8.1	-5.8	CLEC12A
387837	1.0	1.0	1.0	2.9	CLEC12B
161198	1.0	1.0	1.0	1.0	CLEC14A
497190	1.0	1.0	1.0	4.1	CLEC18B
51267	1.0	1.0	1.0	2.8	CLEC1A
9976	1.0	1.0	1.0	1.0	CLEC2B
29121	-3.4	-4.5	1.0	1.0	CLEC2D
7123	1.0	5.4	1.0	6.4	CLEC3B
339390	1.0	1.0	1.0	1.0	CLEC4G
10332	1.0	-5.2	1.0	1.0	CLEC4M
1192	1.6	-2.5	-2.5	1.0	CLIC1

54102	1.0	-3.9	1.0	1.0	CLIC6
7461	1.0	1.0	1.0	1.0	CLIP2
1195	1.0	1.0	-4.5	1.0	CLK1
1207	1.6	1.0	4.2	1.0	CLNS1A
81037	1.0	1.0	1.6	1.0	CLPTM1L
7401	1.0	1.0	1.0	1.0	CLRN1
9746	1.0	1.0	1.0	1.0	CLSTN3
23059	1.0	1.0	-5.2	1.0	CLUAP1
27098	1.0	1.0	-5.2	-5.5	CLUL1
171425	1.0	1.0	1.0	4.1	CLYBL
1215	1.0	1.0	1.0	1.0	CMA1
8418	1.0	1.0	1.0	-3.2	CMAHP
55907	-3.9	1.0	1.0	1.0	CMAS
1240	-4.5	-2.9	-2.9	-2.5	CMKLR1
113540	1.0	1.0	-2.6	1.0	CMTM1
123920	1.0	-21.6	4.0	1.0	CMTM3
116173	1.0	1.5	1.0	1.0	CMTM5
168975	1.0	1.0	1.0	1.0	CNBD1
7555	1.0	1.0	1.0	1.0	CNBP
1261	1.0	1.0	1.0	1.0	CNGA3
254263	-1.9	1.0	1.0	-4.8	CNIH2
29097	1.0	1.0	-5.9	-5.4	CNIH4
22866	1.0	-3.2	1.0	-5.6	CNKSR2
54805	1.0	1.0	-5.2	-7.7	CNNM2
23019	1.0	1.0	1.0	1.0	CNOT1
4848	1.0	1.0	2.5	1.0	CNOT2
4850	1.0	1.0	1.0	1.0	CNOT4
246175	1.0	2.4	1.0	1.0	CNOT6L
29883	5.2	1.0	1.0	4.7	CNOT7
9337	1.0	1.0	1.0	1.0	CNOT8
1267	1.0	-5.7	1.0	1.0	CNP
163882	1.0	1.0	1.0	1.0	CNST
1271	1.0	1.0	1.0	1.0	CNTFR
54875	1.0	1.0	-6.6	1.0	CNTLN
6900	-3.2	6.0	4.6	4.3	CNTN2
8506	3.8	1.0	1.0	1.0	CNTNAP1
79937	1.0	1.0	1.0	1.0	CNTNAP3
728577	1.0	1.0	1.0	2.1	CNTNAP3B
11064	1.0	1.0	-5.1	1.0	CNTRL
493753	-24.2	1.0	1.0	1.0	COA5
80347	-6.3	1.0	1.0	1.0	COASY
22837	4.1	8.1	6.3	1.0	COBLL1
25920	1.0	-3.0	1.0	1.0	COBRA1
22796	-9.8	1.0	-3.6	1.0	COG2
57511	1.0	1.0	1.0	1.0	COG6
91949	1.0	1.0	1.0	1.0	COG7
7373	1.0	1.0	1.0	1.0	COL14A1
1307	1.0	-2.5	-2.6	-2.8	COL16A1

1310	1.0	1.0	1.0	1.0	COL19A1
1277	1.0	1.0	1.0	1.0	COL1A1
1278	1.0	1.0	1.0	1.0	COL1A2
57642	-3.7	1.0	3.7	5.9	COL20A1
169044	1.0	-5.7	-6.3	1.0	COL22A1
85301	-1.8	1.0	1.0	1.0	COL27A1
10087	1.0	1.0	1.0	1.0	COL4A3BP
1286	1.0	1.0	1.0	1.0	COL4A4
1287	1.0	1.0	-3.7	1.0	COL4A5
1288	1.6	1.0	1.0	1.0	COL4A6
1289	1.0	1.0	1.0	2.9	COL5A1
1290	1.0	1.0	1.0	1.0	COL5A2
50509	1.0	4.7	4.8	1.0	COL5A3
1294	1.0	1.0	1.0	1.0	COL7A1
150684	1.0	1.0	-2.4	-2.4	COMMD1
23412	1.0	1.0	1.0	1.0	COMMD3
54939	1.0	1.0	1.0	1.0	COMMD4
149951	1.0	1.0	-3.8	-3.8	COMMD7
9276	1.0	1.0	3.2	1.0	COPB2
8533	1.0	1.0	1.0	3.8	COPS3
64708	1.0	1.0	1.0	1.0	COPS7B
10920	1.0	1.0	1.0	-2.5	COPS8
80219	1.0	1.0	2.6	1.0	COQ10B
27235	1.0	1.0	1.0	1.0	COQ2
51805	5.7	1.0	6.8	7.5	COQ3
51117	1.0	1.0	1.0	1.0	COQ4
84274	1.0	2.7	2.3	1.0	COQ5
10229	-5.3	1.0	1.0	1.0	COQ7
57017	2.0	1.0	1.0	1.0	COQ9
57175	-3.2	1.0	1.0	-4.1	CORO1B
7464	1.0	1.0	1.0	1.0	CORO2A
10391	1.0	1.0	1.0	1.0	CORO2B
23406	1.0	1.0	1.0	1.0	COTL1
4512	1.8	1.0	1.0	2.1	COX1
1352	1.0	1.0	1.0	1.0	COX10
1355	1.0	1.0	1.0	1.0	COX15
51241	1.0	1.0	1.0	1.0	COX16
1327	1.0	1.0	1.0	1.0	COX4I1
1347	1.0	1.0	1.0	1.0	COX7A2
9167	1.0	1.0	-4.7	1.0	COX7A2L
1349	1.0	3.0	4.7	1.9	COX7B
170712	-10.9	-5.1	-3.8	-3.7	COX7B2
1350	13.3	1.0	10.8	1.0	COX7C
1356	1.0	1.0	1.0	-5.8	CP
1359	1.0	-2.5	1.0	1.0	CPA3
93979	1.0	1.0	1.0	1.0	CPA5
27151	1.0	1.0	1.0	1.0	CPAMD8
1361	1.0	1.0	-7.1	1.0	CPB2

1363	1.0	1.0	1.0	1.0	CPE
80315	1.0	2.6	1.0	1.0	CPEB4
10814	1.0	4.4	1.0	1.0	CPLX2
1370	1.0	1.0	1.0	3.3	CPN2
57699	1.0	1.0	1.0	1.0	CPNE5
144402	1.0	-7.6	-6.4	1.0	CPNE8
55313	1.0	1.0	1.0	1.0	CPPED1
53981	1.0	1.0	1.0	1.0	CPSF2
10898	1.0	3.8	1.8	2.9	CPSF4
1376	1.0	1.0	-2.1	1.0	CPT2
119587	1.0	1.0	1.0	1.5	CPXM2
1379	1.0	25.1	1.0	1.0	CR1L
8738	1.0	2.2	1.0	1.0	CRADD
23418	1.0	1.0	-2.8	1.0	CRB1
286204	1.0	2.6	1.0	1.0	CRB2
51185	-3.6	1.0	1.0	1.0	CRBN
90993	-1.9	1.0	1.0	1.0	CREB3L1
64764	1.0	1.0	1.0	1.0	CREB3L2
84699	1.7	1.0	1.6	1.8	CREB3L3
9586	1.0	1.0	2.5	1.0	CREB5
1387	1.0	3.0	1.0	1.0	CREBBP
1392	1.0	1.0	1.0	1.0	CRH
1394	1.9	1.0	1.0	1.0	CRHR1
1396	1.0	3.0	4.2	5.1	CRIP1
1397	1.0	1.0	1.0	1.0	CRIP2
9419	1.0	-4.0	-5.7	1.0	CRIPT
7180	2.7	1.0	1.0	1.0	CRISP2
51340	1.0	1.0	1.0	1.0	CRNKL1
9696	1.0	1.0	1.0	1.0	CROCC
84809	1.0	-7.7	1.0	1.0	CROCCP2
54677	1.0	1.0	-5.8	1.0	CROT
1401	1.0	1.0	2.6	1.0	CRP
10491	-9.3	1.0	1.0	1.0	CRTAP
23373	1.0	1.0	1.0	1.0	CRTC1
1406	1.0	1.0	1.0	1.0	CRX
1409	1.0	1.0	1.0	1.0	CRYAA
1413	1.0	1.0	1.0	1.0	CRYBA4
1415	1.0	-2.7	1.0	1.0	CRYBB2
155051	1.0	1.0	1.0	1.0	CRYGN
9946	1.0	-7.7	-10.3	-8.6	CRYZL1
158511	1.0	1.0	1.0	-3.1	CSAG1
8531	1.0	1.0	1.0	-1.6	CSDA
7812	-1.7	-2.4	1.0	1.0	CSDE1
1434	-2.9	1.0	1.0	2.4	CSE1L
1436	1.0	1.0	-2.6	-2.4	CSF1R
55790	1.0	1.0	1.0	1.0	CSGALNACT1
55454	1.0	1.0	1.9	1.0	CSGALNACT2
64478	1.0	1.0	1.0	1.0	CSMD1

114784	1.0	1.0	1.0	1.0	CSMD2
1447	1.0	1.0	1.0	1.0	CSN2
1448	1.0	-2.7	-11.2	-2.2	CSN3
122011	1.0	4.1	1.0	1.0	CSNK1A1L
1455	6.1	1.0	1.0	1.0	CSNK1G2
1457	1.0	1.0	5.4	1.0	CSNK2A1
64651	1.8	1.0	1.0	1.0	CSRNP1
1469	1.0	1.0	1.6	1.0	CST1
1470	1.0	1.0	1.0	6.7	CST2
1471	3.0	1.0	1.0	-2.5	CST3
1476	-5.6	1.0	1.0	-3.4	CSTB
1479	1.0	4.7	1.0	1.0	CSTF3
128817	1.0	1.0	-3.1	1.0	CSTL1
164380	1.0	1.0	-6.8	1.0	CSTT
541466	1.0	1.0	1.0	1.0	CT45A1
64693	1.0	1.0	2.6	2.7	CTAGE1
441294	1.0	1.0	1.0	1.0	CTAGE15P
100128553	1.0	1.0	1.0	1.0	CTAGE4
4253	1.0	1.0	-7.1	1.0	CTAGE5
1488	2.8	1.0	1.0	-3.0	CTBP2
10664	13.6	1.0	3.1	1.0	CTCF
9150	1.0	1.0	1.0	1.0	CTDP1
58190	-5.1	1.0	-6.7	1.0	CTDSP1
10106	1.0	1.0	1.0	-19.6	CTDSP2
51496	1.0	1.0	1.0	-3.0	CTDSPL2
1489	1.0	1.0	1.0	1.0	CTF1
8727	1.0	1.0	1.0	1.0	CTNNAL1
1499	1.5	1.0	1.0	1.0	CTNNB1
1497	1.0	1.0	1.0	1.0	CTNS
56474	-2.0	1.0	-3.5	-3.5	CTPS2
1075	4.8	1.0	-2.8	-2.8	CTSC
1509	2.0	1.0	1.0	1.8	CTSD
1510	1.0	1.0	1.0	1.0	CTSE
8722	1.0	1.0	1.0	4.4	CTSF
1512	1.5	1.0	1.0	1.0	CTSH
1514	1.0	1.0	1.0	1.0	CTSL1
55917	1.0	1.0	1.0	1.0	CTTNBP2NL
8029	1.0	1.0	1.0	1.0	CUBN
8454	1.0	1.0	-3.0	1.0	CUL1
8451	1.0	2.9	3.4	1.0	CUL4A
8065	1.0	3.7	1.0	1.0	CUL5
9820	1.0	3.3	1.0	1.0	CUL7
1523	-13.9	1.0	1.0	1.0	CUX1
54883	1.0	1.0	1.0	1.0	CWC25
55280	2.1	1.0	1.0	1.0	CWF19L1
1525	-9.4	1.0	1.0	1.0	CXADR
6387	1.0	2.0	1.0	1.0	CXCL12
10563	1.0	-2.9	-2.3	-2.6	CXCL13

58191	1.0	1.0	1.0	1.0	CXCL16
284340	1.0	1.0	1.0	1.0	CXCL17
4283	1.0	-3.4	1.0	1.0	CXCL9
3577	-2.0	1.0	1.0	-3.4	CXCR1
3580	1.0	1.0	1.0	1.0	CXCR2P1
2833	1.0	-2.5	-2.4	1.0	CXCR3
9142	1.0	1.0	1.0	1.0	CXorf1
170063	1.0	1.0	1.0	-3.0	CXorf22
256643	1.0	1.0	1.0	1.0	CXorf23
79742	1.0	1.0	1.0	1.0	CXorf36
159013	1.0	1.0	2.5	1.0	CXorf38
541578	1.0	1.0	1.0	1.0	CXorf40B
100129239	1.0	1.0	-5.6	1.0	CXorf51A
63932	1.0	-4.5	3.2	1.0	CXorf56
158830	1.0	1.0	1.0	1.0	CXorf65
1528	5.1	1.0	1.0	1.0	CYB5A
124637	1.0	1.0	1.0	1.0	CYB5D1
51700	1.0	1.0	3.1	1.0	CYB5R2
1727	1.0	5.4	1.0	3.1	CYB5R3
51167	1.0	-2.5	1.0	1.0	CYB5R4
54205	-7.3	1.0	1.0	-7.0	CYCS
26999	1.0	1.0	1.0	1.0	CYFIP2
50626	1.7	1.0	1.0	1.0	CYHR1
1584	2.1	4.4	1.0	1.0	CYP11B1
1588	1.0	1.0	1.0	-6.2	CYP19A1
1543	1.0	2.7	2.6	1.0	CYP1A1
56603	1.0	1.0	1.0	1.0	CYP26B1
1593	1.0	1.0	1.0	1.0	CYP27A1
1594	1.0	1.0	-3.9	1.0	CYP27B1
1553	1.0	1.0	1.0	-2.5	CYP2A13
1549	-7.0	1.0	1.0	1.0	CYP2A7
1562	1.0	1.0	1.0	1.0	CYP2C18
1565	1.0	1.0	1.0	1.0	CYP2D6
1572	1.8	1.0	1.0	3.9	CYP2F1
1573	-21.4	1.0	3.2	1.0	CYP2J2
120227	1.0	1.0	1.0	1.0	CYP2R1
54905	1.0	1.0	1.0	1.0	CYP2W1
1576	1.0	1.0	1.0	1.0	CYP3A4
64816	-2.0	1.0	1.0	-5.7	CYP3A43
1577	-2.1	1.0	1.0	-3.0	CYP3A5
10858	1.0	1.0	1.0	1.0	CYP46A1
1579	2.2	1.0	3.0	1.0	CYP4A11
57834	1.0	1.0	1.0	1.0	CYP4F11
8529	1.0	1.0	1.0	1.0	CYP4F2
1581	1.0	1.0	2.6	1.0	CYP7A1
9420	1.0	1.0	1.0	-3.8	CYP7B1
9267	1.0	1.0	1.0	1.0	CYTH1
9595	1.0	1.0	1.0	1.0	CYTIP

116159	1.0	1.0	-6.7	-6.4	CYYR1
153090	1.0	3.3	3.4	3.1	DAB2IP
1602	1.0	-3.8	1.0	1.0	DACH1
168002	-7.8	1.0	-7.6	1.0	DACT2
147906	-3.4	1.0	1.0	1.0	DACT3
1605	1.0	1.0	-2.7	1.0	DAG1
26007	1.0	1.0	1.0	1.0	DAK
55152	1.0	1.0	-3.5	-3.5	DALRD3
1611	2.8	1.0	2.7	6.8	DAP
1612	1.0	1.0	1.0	1.0	DAPK1
92196	1.0	1.0	1.0	1.0	DAPL1
27071	1.0	1.0	1.0	1.0	DAPP1
1615	-1.6	1.0	1.0	4.7	DARS
1616	1.0	1.0	1.0	2.9	DAXX
26528	1.0	1.0	1.0	1.0	DAZAP1
1622	1.0	1.0	-2.7	1.0	DBI
100131454	1.0	-3.7	-4.1	-2.1	DBIL5P
100169989	1.0	1.0	1.0	-3.5	DBIL5P2
28988	1.0	-5.6	-3.8	1.0	DBNL
1628	1.0	-3.8	1.0	-3.4	DBP
51163	1.0	1.0	1.0	1.0	DBR1
79269	90.5	1.0	81.4	1.0	DCAF10
25853	1.0	1.0	1.0	1.0	DCAF12
139170	1.0	-3.6	1.0	1.0	DCAF12L1
25879	1.0	-3.4	1.0	-3.5	DCAF13
54876	1.0	1.0	1.0	1.0	DCAF16
80067	1.0	1.9	1.0	1.0	DCAF17
138009	1.0	-3.4	1.0	-5.5	DCAF4L2
10238	1.6	3.6	1.0	1.0	DCAF7
50717	1.0	1.0	1.0	1.0	DCAF8
285761	1.0	1.0	1.0	-2.3	DCBLD1
54798	-1.8	1.0	-5.5	1.0	DCHS2
64421	1.0	1.0	3.6	1.0	DCLRE1C
55802	1.0	1.0	1.0	1.0	DCP1A
84516	1.0	1.0	1.0	5.7	DCTN5
55208	1.0	-7.2	-5.6	1.0	DCUN1D2
79016	6.0	1.0	1.0	1.0	DDA1
23564	1.0	1.0	1.0	1.0	DDAH2
1642	1.0	1.0	5.7	1.0	DDB1
1643	-2.5	-4.4	-4.2	1.0	DDB2
23259	4.5	1.0	1.0	1.0	DDHD2
84301	1.0	1.0	1.0	1.0	DDI2
54541	1.0	1.0	2.0	1.0	DDIT4
115265	1.0	1.0	1.0	1.0	DDIT4L
8528	1.0	1.0	1.0	1.0	DDO
4921	1.0	1.0	1.0	1.0	DDR2
1662	1.0	1.0	5.7	1.0	DDX10
10521	1.0	1.0	1.0	1.0	DDX17

8886	1.0	1.0	1.0	1.0	DDX18
55308	1.0	1.0	-3.4	1.0	DDX19A
11218	1.0	1.0	3.3	2.4	DDX20
57062	1.0	1.0	1.0	1.0	DDX24
29118	1.0	1.0	1.0	1.0	DDX25
203522	1.0	1.0	1.0	1.0	DDX26B
55661	1.0	1.0	1.7	1.0	DDX27
7919	1.0	1.0	1.0	-3.3	DDX39B
8653	1.0	1.0	1.0	1.0	DDX3Y
51202	-2.0	1.0	1.0	1.0	DDX47
1655	1.0	1.0	1.0	1.0	DDX5
1656	-2.9	3.7	3.2	2.2	DDX6
10522	1.0	-2.6	1.0	1.0	DEAF1
26063	1.0	1.0	1.0	1.0	DECR2
9191	3.5	1.0	1.0	1.0	DEDD
50619	1.0	1.0	1.0	1.0	DEF6
1669	1.0	1.0	1.0	4.4	DEFA4
1670	1.0	1.0	5.0	1.0	DEFA5
503618	1.0	1.0	1.0	1.0	DEFB104B
504180	1.0	1.0	2.8	1.0	DEFB105B
503841	1.0	1.0	-3.3	1.0	DEFB106B
245928	1.0	1.0	-3.6	1.0	DEFB114
245936	1.8	1.0	1.7	1.0	DEFB123
140881	8.4	9.1	9.4	9.0	DEFB129
8560	1.0	-1.6	1.0	1.0	DEGS1
57706	1.0	-3.8	1.0	1.0	DENND1A
27147	2.9	-2.5	1.0	-2.6	DENND2A
55667	1.0	1.0	1.0	1.0	DENND4C
160518	1.0	1.0	1.0	1.0	DENND5B
55789	1.0	2.6	2.9	1.0	DEPDC1B
9681	1.0	-3.0	1.0	1.0	DEPDC5
64798	1.0	1.0	1.0	1.0	DEPTOR
8694	1.0	1.0	1.0	1.0	DGAT1
8220	1.0	1.0	1.0	1.0	DGCR14
1607	1.0	1.0	1.0	1.0	DGKB
8526	1.0	1.0	1.0	-2.8	DGKE
160851	1.0	1.0	1.0	1.0	DGKH
1716	1.0	1.0	-3.9	1.0	DGUOK
79947	1.0	-4.8	1.0	1.0	DHDDS
1719	-17.4	1.6	1.0	1.0	DHFR
1725	1.0	-4.0	1.0	1.0	DHPS
10202	1.0	1.0	2.6	1.0	DHRS2
51635	-2.9	-4.0	-3.5	-3.4	DHRS7
25979	-1.9	1.0	1.0	1.0	DHRS7B
201140	1.0	1.0	2.5	1.0	DHRS7C
10170	1.0	1.0	1.0	3.7	DHRS9
9704	1.0	1.0	-3.3	1.0	DHX34
57647	7.6	1.0	4.0	1.0	DHX37

90957	1.0	-3.6	1.0	1.0	DHX57
1660	1.0	1.0	1.0	1.0	DHX9
1734	1.0	1.0	5.3	1.0	DIO2
1735	1.0	1.0	-3.0	1.0	DIO3
22982	1.0	1.0	2.8	4.5	DIP2C
148252	1.0	3.4	1.0	1.0	DIRAS1
84925	1.0	1.0	1.0	1.0	DIRC2
129563	1.6	1.0	-6.5	1.0	DIS3L2
27185	1.0	4.4	1.0	1.0	DISC1
84976	1.0	1.0	1.0	1.0	DISP1
1736	-6.0	2.9	3.8	1.0	DKC1
26082	1.0	1.0	1.0	1.0	DKFZP434L187
222029	1.0	1.0	1.0	1.0	DKFZp434L192
84237	1.0	1.0	1.0	1.0	DKFZp547J222
26120	1.0	1.0	1.0	-4.0	DKFZP564C152
401232	1.0	-1.6	1.0	-1.9	DKFZP686I15217
27121	1.0	-6.2	-8.9	-4.6	DKK4
10395	1.0	1.0	2.9	1.0	DLC1
10301	1.0	1.0	4.5	1.0	DLEU1
220107	1.0	1.0	1.0	1.0	DLEU7
1740	1.0	1.0	5.3	5.1	DLG2
1741	1.0	1.0	1.0	-4.7	DLG3
58512	1.0	1.0	1.0	1.0	DLGAP3
54567	1.0	1.0	1.0	3.1	DLL4
1743	1.0	1.0	4.2	1.0	DLST
1745	1.0	-4.6	-4.8	-5.4	DLX1
1747	2.1	1.0	1.0	1.0	DLX3
55929	2.2	1.0	-3.0	1.0	DMAP1
1755	1.0	1.0	-3.7	1.0	DMBT1
1756	1.0	1.0	-3.1	-3.3	DMD
29958	1.0	1.0	1.0	1.0	DMGDH
93099	1.0	1.0	1.0	1.0	DMKN
10655	2.4	2.6	4.1	1.0	DMRT2
58524	1.0	1.0	1.0	1.0	DMRT3
23312	1.0	1.0	1.0	1.0	DMXL2
123872	1.0	1.0	1.0	1.0	DNAAF1
196385	1.0	1.0	1.9	1.0	DNAH10
8701	1.0	1.0	1.0	1.0	DNAH11
201625	1.0	1.0	1.0	5.7	DNAH12
146754	3.7	4.1	1.0	1.0	DNAH2
55567	4.2	1.0	1.0	1.0	DNAH3
1768	1.0	1.0	-4.7	1.0	DNAH6
56171	-14.4	2.4	3.0	1.0	DNAH7
27019	1.0	1.0	-6.7	1.0	DNAI1
9093	1.0	4.1	5.0	3.9	DNAJA3
55466	1.0	1.0	1.0	1.0	DNAJA4
51726	1.0	1.0	-3.1	1.0	DNAJB11
10049	-2.4	1.0	2.7	5.0	DNAJB6

165721	1.0	1.0	-3.9	1.0	DNAJB8
64215	1.0	1.0	-1.9	1.0	DNAJC1
85406	1.0	1.0	1.0	1.0	DNAJC14
23341	1.0	1.0	1.0	1.0	DNAJC16
202052	5.8	1.0	1.0	2.4	DNAJC18
134218	1.0	-3.8	-3.8	1.0	DNAJC21
84277	-1.6	5.2	1.0	1.0	DNAJC30
3338	2.8	1.0	1.0	-3.4	DNAJC4
80331	1.0	-3.2	-4.0	-3.1	DNAJC5
85479	1.0	-3.0	1.0	1.0	DNAJC5B
23234	1.0	1.0	3.0	1.0	DNAJC9
1775	1.0	1.0	1.0	1.0	DNASE1L2
373863	1.0	1.0	4.8	4.8	DND1
1759	1.0	1.0	1.0	1.0	DNM1
10059	1.0	1.0	1.0	4.0	DNM1L
23268	1.0	1.0	1.0	1.0	DNMBP
1788	1.0	-3.2	1.0	1.0	DNMT3A
23549	1.0	4.1	5.0	4.7	DNPEP
116092	1.0	1.0	1.0	1.0	DNTTIP1
8448	1.0	1.0	5.3	1.0	DOC2A
8447	1.0	1.0	1.0	1.0	DOC2B
1794	1.0	1.0	1.0	1.0	DOCK2
1795	6.7	1.0	1.0	1.0	DOCK3
23348	3.9	1.0	1.0	1.0	DOCK9
83475	1.0	1.0	1.0	1.0	DOHH
9046	-2.0	-6.9	-6.9	1.0	DOK2
285489	1.0	1.0	2.5	1.0	DOK7
29980	1.0	1.0	1.0	1.0	DONSON
23033	1.0	1.0	1.0	1.0	DOPEY1
84444	1.0	1.0	1.0	1.0	DOT1L
1798	1.0	1.0	1.0	1.0	DPAGT1
1800	1.0	1.0	1.0	1.0	DPEP1
64174	1.0	1.0	-2.7	1.0	DPEP2
8110	-2.2	1.0	1.0	1.0	DPF3
285381	1.0	1.0	-6.3	1.0	DPH3
100132911	1.0	1.0	1.0	1.0	DPH3P1
54344	1.0	1.0	1.0	1.0	DPM3
1804	-2.3	1.0	-5.9	1.0	DPP6
29952	1.6	1.0	1.0	1.0	DPP7
54878	-4.8	1.0	-8.6	1.0	DPP8
91039	1.0	1.0	1.0	1.0	DPP9
151871	1.0	1.0	1.0	1.0	DPPA2
1806	1.0	1.0	1.0	1.0	DPYD
1809	1.0	5.1	7.1	2.9	DPYSL3
10570	1.7	1.0	1.0	1.0	DPYSL4
56896	1.0	1.0	1.0	1.0	DPYSL5
55332	4.1	1.0	1.0	1.0	DRAM1
1814	1.0	1.0	3.2	1.0	DRD3

1815	5.1	1.0	1.0	-1.9	DRD4
1816	1.0	1.0	1.0	1.0	DRD5
4733	-1.6	1.0	1.0	1.0	DRG1
1823	1.0	-3.8	1.0	1.0	DSC1
1824	1.0	3.3	2.7	2.7	DSC2
1825	6.9	1.0	4.6	1.0	DSC3
10281	4.9	1.0	1.0	1.0	DSCR4
53820	-2.2	1.0	1.0	1.0	DSCR6
147409	1.0	1.0	1.0	1.0	DSG4
1834	1.0	1.0	1.0	-3.9	DSPP
667	4.7	1.0	-4.7	-4.4	DST
11034	1.0	1.0	1.0	1.0	DSTN
51514	1.0	1.0	1.0	1.0	DTL
1837	1.0	1.0	1.0	1.0	DTNA
1838	1.0	1.0	1.0	1.0	DTNB
196403	2.1	1.0	1.0	1.0	DTX3
23220	1.0	-3.8	1.0	1.0	DTX4
53905	1.0	1.0	1.0	1.0	DUOX1
50506	1.0	1.0	1.0	1.0	DUOX2
90527	1.0	1.0	1.0	1.0	DUOXA1
405753	1.0	1.0	1.0	1.0	DUOXA2
64118	1.0	1.0	1.0	3.2	DUS1L
54920	1.0	1.0	1.0	1.0	DUS2L
1843	1.0	1.0	1.0	1.0	DUSP1
51207	1.0	1.0	2.7	1.0	DUSP13
11072	1.0	3.1	1.0	1.0	DUSP14
574029	-8.4	5.3	1.0	1.0	DUSP5P
1848	1.0	1.0	1.0	1.0	DUSP6
1850	1.0	1.0	1.0	1.0	DUSP8
1852	1.0	1.0	1.0	-2.4	DUSP9
1855	-2.0	1.0	-1.6	1.0	DVL1
1857	-2.6	1.0	1.0	1.0	DVL3
1780	1.0	1.0	1.0	1.0	DYNC1I1
51626	1.0	1.0	1.0	1.0	DYNC2LI1
8655	1.0	1.0	1.0	1.0	DYNLL1
140735	1.0	1.0	2.6	1.0	DYNLL2
83658	1.0	1.0	1.0	1.0	DYNLRB1
83657	1.0	1.0	-4.1	-3.6	DYNLRB2
9149	1.0	1.0	1.0	3.4	DYRK1B
8445	1.6	2.5	1.0	1.0	DYRK2
8444	1.0	1.0	-4.7	1.0	DYRK3
55184	1.7	5.7	1.0	1.0	DZANK1
1871	1.0	1.0	1.0	3.3	E2F3
1874	2.0	1.0	-2.9	-2.9	E2F4
9166	1.0	1.0	1.0	1.0	EBAG9
1879	1.9	1.0	1.0	1.0	EBF1
64641	1.0	1.0	1.0	1.0	EBF2
253738	-7.1	1.0	1.0	1.0	EBF3

10969	1.0	2.8	2.9	2.9	EBNA1BP2
84650	1.0	1.0	-2.5	-2.5	EBPL
11319	1.0	1.0	-3.0	-2.6	ECD
1889	1.0	1.0	1.0	1.0	ECE1
9718	10.0	1.8	1.0	1.0	ECE2
9427	1.0	1.0	1.0	1.0	ECEL1
347694	1.0	1.0	1.0	1.0	ECEL1P2
55862	-12.0	1.0	1.0	1.0	ECHDC1
345930	1.0	1.0	2.8	1.0	ECT2L
1896	1.0	1.0	1.0	1.0	EDA
10913	1.0	1.0	-2.2	1.0	EDAR
10876	1.0	1.0	1.0	1.0	EDDM3A
64184	6.2	1.0	1.0	1.0	EDDM3B
55741	1.0	1.0	1.0	-4.5	EDEM2
80267	1.0	1.0	1.0	1.0	EDEM3
1906	1.0	1.0	-2.4	1.0	EDN1
1907	1.0	3.0	1.0	1.0	EDN2
1909	1.0	1.5	1.0	1.0	EDNRA
1910	2.2	-7.4	-5.2	-1.7	EDNRB
9521	2.3	-2.7	1.0	1.0	EEF1E1
60678	1.0	6.1	1.0	5.4	EEFSEC
80820	1.0	5.4	5.8	5.2	EEPD1
79645	1.0	1.0	3.2	1.0	EFCAB1
90141	1.0	-6.4	-5.7	1.0	EFCAB11
84455	1.0	1.0	1.0	-3.2	EFCAB7
1942	1.0	1.0	-3.8	-3.8	EFNA1
1943	1.0	1.0	1.0	-3.4	EFNA2
1944	1.0	1.0	1.0	1.0	EFNA3
1947	1.6	1.0	1.0	1.0	EFNB1
1948	1.0	4.4	1.0	1.0	EFNB2
23167	-25.4	1.0	7.3	1.0	EFR3A
22979	-3.9	1.0	2.2	1.0	EFR3B
93556	1.0	1.0	1.0	-3.7	EGFEM1P
51162	1.0	1.0	-2.9	1.0	EGFL7
80864	-1.7	-5.0	-6.4	-4.3	EGFL8
133584	1.0	1.0	1.0	1.0	EGFLAM
1956	1.0	1.0	-2.2	1.0	EGFR
112398	1.0	-2.8	-2.6	1.0	EGLN2
1958	1.0	4.2	1.0	1.0	EGR1
23301	-2.1	1.0	1.0	1.0	EHBP1
10938	1.0	2.8	1.0	1.0	EHD1
163126	1.0	1.0	1.0	1.0	EID2
10209	1.0	2.4	1.0	1.0	EIF1
84285	1.7	9.0	6.6	8.9	EIF1AD
9086	1.0	1.0	-3.4	-3.4	EIF1AY
1967	-5.5	1.0	1.0	-3.6	EIF2B1
8893	1.0	1.0	2.8	1.0	EIF2B5
26523	1.0	3.4	3.4	1.0	EIF2C1

1939	1.0	-2.6	-2.4	1.0	EIF2D
8894	1.0	1.0	1.0	1.0	EIF2S2
8664	1.0	1.0	1.0	1.0	EIF3D
8666	1.0	-2.7	1.0	1.0	EIF3G
8669	1.0	1.0	1.0	1.0	EIF3J
51386	1.0	1.0	1.0	1.0	EIF3L
10480	2.9	1.0	-3.3	-2.8	EIF3M
1973	-3.0	-2.6	1.0	1.0	EIF4A1
9470	1.0	1.0	1.0	1.0	EIF4E2
56478	1.0	-2.8	-2.7	1.0	EIF4ENIF1
8672	1.0	1.0	1.0	1.0	EIF4G3
1984	1.0	-3.1	1.0	1.0	EIF5A
9669	1.0	1.0	1.0	1.0	EIF5B
3692	-2.1	-1.9	1.0	1.0	EIF6
60528	1.0	1.0	1.0	1.0	ELAC2
1994	1.0	1.0	4.9	1.0	ELAVL1
1997	-5.8	1.0	1.0	1.0	ELF1
1998	1.0	1.0	1.0	1.0	ELF2
2002	1.0	1.0	2.4	1.0	ELK1
2005	1.0	1.0	4.8	1.0	ELK4
80237	-4.3	1.0	1.0	1.0	ELL3
63916	1.0	1.0	1.0	1.0	ELMO2
64834	1.6	1.0	1.0	2.4	ELOVL1
83401	8.2	1.0	10.0	10.9	ELOVL3
79071	1.0	1.0	1.0	1.0	ELOVL6
51705	1.0	1.0	1.0	1.0	EMCN
2010	1.0	1.0	2.5	3.0	EMD
84034	14.4	1.0	11.6	1.0	EMILIN2
90187	1.0	1.0	1.0	1.0	EMILIN3
2009	1.0	1.0	5.7	1.0	EML1
27436	1.9	1.0	-5.7	1.0	EML4
2012	1.0	2.4	1.0	3.7	EMP1
326342	18.2	1.0	20.8	1.0	EMR4P
2016	1.0	1.0	1.0	-5.8	EMX1
2019	-1.9	1.0	1.0	1.0	EN1
55740	1.0	1.0	30.6	1.0	ENAH
8507	1.0	1.0	1.7	1.0	ENC1
23052	1.0	1.0	1.0	1.0	ENDOD1
284131	1.0	1.0	1.0	1.0	ENDOV
2022	1.8	1.0	1.0	1.0	ENG
64772	1.0	-1.6	-1.6	1.0	ENGASE
375704	1.0	-4.5	-4.3	1.0	ENHO
219670	1.0	-5.3	1.0	1.0	ENKUR
55556	1.0	1.0	1.0	1.0	ENOSF1
10495	1.0	-4.0	1.0	1.0	ENOX2
5167	3.0	1.0	7.0	1.0	ENPP1
955	1.0	1.0	1.0	1.0	ENTPD6
2036	1.0	1.0	2.9	3.0	EPB41L1

114915	1.0	1.0	1.0	1.0	EPB41L4A-AS1
54566	1.0	4.8	4.7	3.0	EPB41L4B
2039	1.0	1.0	1.0	3.3	EPB49
26122	-13.0	1.0	1.0	1.0	EPC2
284656	1.0	-7.3	1.0	1.0	EPHA10
1969	1.0	1.0	1.0	1.0	EPHA2
2045	1.0	1.0	1.0	1.0	EPHA7
2046	1.0	1.0	3.6	1.9	EPHA8
2047	1.9	1.0	-2.5	1.0	EPHB1
2051	1.0	1.0	1.0	-2.3	EPHB6
2052	-4.3	1.0	1.0	1.0	EPHX1
253152	1.0	1.0	1.0	1.0	EPHX4
55040	1.0	-5.2	1.0	1.0	EPN3
2057	3.7	1.0	1.0	1.0	EPOR
2060	1.0	1.0	1.0	1.0	EPS15
58513	2.3	4.4	4.0	1.0	EPS15L1
54869	1.0	-2.7	1.0	1.0	EPS8L1
94240	1.0	-2.9	1.0	-3.4	EPSTI1
26284	1.0	1.0	1.0	-2.9	ERAL1
2064	1.9	-1.6	2.2	1.0	ERBB2
55914	1.0	1.0	1.0	1.0	ERBB2IP
2066	1.0	2.9	1.0	2.6	ERBB4
2067	1.0	1.0	1.0	1.0	ERCC1
2073	1.0	3.1	3.2	2.6	ERCC5
51614	1.0	1.0	1.0	1.0	ERGIC3
2079	1.0	1.0	1.0	2.5	ERH
90459	1.0	1.0	1.0	1.0	ERI1
79033	2.8	1.0	1.0	-2.3	ERI3
10613	1.0	-3.3	-3.0	1.0	ERLIN1
11160	1.0	-2.4	1.0	1.0	ERLIN2
57471	-7.5	1.0	-2.2	1.0	ERMN
79956	1.0	1.0	1.0	1.0	ERMP1
10961	-2.8	-4.7	-4.7	-4.7	ERP29
54206	1.0	5.0	1.0	1.0	ERRFI1
90952	-7.8	1.0	1.0	1.0	ESAM
2098	1.0	1.0	1.0	1.0	ESD
9700	1.0	1.0	2.4	1.0	ESPL1
83715	1.0	1.0	1.0	1.0	ESPN
2099	2.0	1.0	1.0	2.1	ESR1
2100	1.0	1.0	1.0	1.0	ESR2
54845	1.0	1.0	1.0	1.0	ESRP1
80004	1.8	1.0	1.0	1.0	ESRP2
2101	1.0	-2.7	-2.5	-2.5	ESRRA
57488	1.0	1.0	1.0	1.0	ESYT2
83850	1.0	-2.6	1.0	1.0	ESYT3
54465	4.0	2.7	1.0	-2.0	ETAA1
2109	1.7	-3.4	-3.4	-3.5	ETFB
23474	1.0	1.0	1.0	-3.8	ETHE1

55500	-8.3	1.0	1.0	1.0	ETNK1
2115	1.0	1.0	-5.1	1.0	ETV1
2117	1.0	1.0	1.0	1.0	ETV3
645027	-1.9	1.0	1.0	1.0	EVPLL
2130	1.0	-2.9	1.0	1.0	EWSR1
161829	1.0	1.0	1.0	3.8	EXD1
54932	1.0	1.0	1.0	1.0	EXD3
9156	1.0	1.0	1.0	1.0	EXO1
55763	1.0	1.0	-3.4	-3.5	EXOC1
283849	1.0	1.0	1.0	1.0	EXOC3L1
91828	1.0	1.0	3.6	1.0	EXOC3L4
54536	1.0	2.0	-6.4	-7.6	EXOC6
23233	1.0	1.0	1.0	1.0	EXOC6B
23265	1.0	1.0	1.0	1.0	EXOC7
9941	1.0	1.0	1.0	1.0	EXOG
23404	1.0	-3.1	1.0	1.0	EXOSC2
54512	4.0	6.0	1.0	1.0	EXOSC4
56915	1.0	-3.5	1.0	1.0	EXOSC5
2131	1.9	1.0	1.0	-3.1	EXT1
2134	-2.1	4.7	5.2	6.0	EXTL1
2137	1.0	1.0	2.7	2.7	EXTL3
2138	1.0	1.0	1.0	2.6	EYA1
2139	1.0	1.0	5.5	1.0	EYA2
7430	1.0	1.0	1.0	4.8	EZR
2160	1.0	1.0	1.0	1.0	F11
2149	1.0	1.0	1.0	-3.3	F2R
2157	1.0	-4.2	-3.9	-3.9	F8
8263	6.3	8.2	8.4	1.0	F8A1
2158	1.0	1.0	1.0	7.9	F9
2166	1.0	1.0	1.0	1.0	FAAH
646486	1.0	1.0	-7.2	1.0	FABP12
8772	1.0	2.4	1.6	1.0	FADD
3992	1.0	1.0	1.0	1.0	FADS1
283985	1.0	1.0	1.0	1.0	FADS6
23197	-1.6	1.0	1.7	1.0	FAF2
81889	1.0	1.0	1.0	3.9	FAHD1
51011	1.0	1.0	1.0	1.0	FAHD2A
283991	1.0	-2.8	1.0	1.0	FAM100B
284611	1.0	1.0	1.0	1.0	FAM102B
83641	1.0	1.0	1.0	-5.9	FAM107B
83541	1.0	1.0	-1.8	1.0	FAM110A
91523	1.0	4.1	1.0	1.7	FAM113B
9747	1.0	1.0	1.0	1.0	FAM115A
285966	1.0	2.8	2.6	1.0	FAM115C
201627	1.0	1.0	1.0	1.0	FAM116A
150864	1.0	1.0	1.0	1.0	FAM117B
55007	1.0	1.0	-2.1	1.0	FAM118A
79607	1.0	-7.1	-5.3	-6.2	FAM118B

84498	1.0	27.3	1.0	1.0	FAM120B
54954	1.0	1.0	1.0	2.5	FAM120C
159090	1.0	1.0	1.0	1.0	FAM122B
159091	1.0	1.0	1.0	1.0	FAM122C
139285	1.0	1.0	1.0	1.0	FAM123B
220108	1.0	1.0	-5.8	1.0	FAM124A
79843	1.0	1.0	-3.5	1.0	FAM124B
26071	1.0	1.0	1.0	1.0	FAM127B
116496	1.0	-3.9	-3.4	1.0	FAM129A
199786	1.0	1.0	1.0	1.0	FAM129C
131408	1.0	1.0	1.6	1.0	FAM131A
388581	1.0	1.0	5.6	1.0	FAM132A
257415	1.0	1.0	1.0	1.0	FAM133B
79137	1.0	1.0	1.0	1.0	FAM134A
54463	1.0	1.0	1.0	3.1	FAM134B
57579	1.0	1.0	1.0	1.0	FAM135A
84908	1.0	2.2	1.0	2.2	FAM136A
317662	1.0	1.0	1.0	1.0	FAM149B1
338094	1.0	1.0	1.0	1.0	FAM151A
158297	1.0	1.0	1.0	1.0	FAM154A
27112	1.0	1.8	1.0	1.0	FAM155B
29057	1.0	1.0	1.0	1.0	FAM156A
51016	1.0	1.0	1.0	1.0	FAM158A
57700	1.0	1.0	5.0	1.0	FAM160B1
221303	1.0	1.0	1.0	1.0	FAM162B
148753	1.0	1.0	1.0	1.0	FAM163A
51101	1.0	1.0	1.0	1.0	FAM164A
23201	1.0	1.0	1.0	1.0	FAM168A
340069	1.0	1.0	4.6	1.0	FAM170A
221061	1.0	1.0	1.0	-2.8	FAM171A1
65990	1.0	1.0	1.0	6.9	FAM173A
134145	1.0	1.0	1.0	1.0	FAM173B
84141	1.0	4.0	1.0	1.0	FAM176A
55719	1.0	1.0	2.5	1.0	FAM178A
90050	1.0	1.0	1.0	-1.6	FAM181A
220382	1.0	1.0	3.8	5.0	FAM181B
440585	1.0	-1.8	-1.9	1.0	FAM183A
340286	1.0	-5.6	1.0	-8.7	FAM183B
79632	1.0	1.0	-3.8	1.0	FAM184A
148109	1.0	2.9	1.0	1.0	FAM187B
10712	1.0	1.0	1.6	1.0	FAM189B
201158	1.0	1.0	3.2	1.0	FAM18B2
100533496	1.0	1.0	1.0	1.0	FAM18B2-CDRT4
54462	1.0	4.7	6.5	1.0	FAM190B
80011	1.0	1.0	1.0	1.0	FAM192A
8603	1.0	1.0	2.3	1.0	FAM193A
54540	1.0	-3.0	1.0	1.0	FAM193B
131831	1.0	1.0	1.0	1.0	FAM194A

84331	1.0	-7.2	1.0	1.0	FAM195A
63877	1.0	1.0	1.8	2.0	FAM204A
54942	1.0	-7.1	1.0	1.0	FAM206A
54757	1.0	1.0	1.0	1.0	FAM20A
9917	1.0	1.0	1.0	1.0	FAM20B
56975	1.0	1.0	1.0	1.0	FAM20C
253725	1.0	2.4	1.0	1.0	FAM21C
728130	1.0	1.0	1.0	1.0	FAM22D
221301	1.0	1.0	-3.2	1.0	FAM26D
441168	1.0	1.0	1.0	1.0	FAM26F
284123	1.0	1.0	1.0	1.0	FAM27L
26017	1.0	1.0	3.6	1.0	FAM32A
54537	1.0	4.0	1.0	1.0	FAM35A
116228	1.0	1.0	-6.1	1.0	FAM36A
60343	1.0	1.0	1.0	2.9	FAM3A
54097	1.0	1.0	1.8	1.0	FAM3B
85369	1.0	1.0	1.0	1.0	FAM40A
163933	1.0	1.0	1.0	1.0	FAM43B
55603	1.0	1.0	1.0	1.0	FAM46A
115572	1.0	2.1	1.0	2.0	FAM46B
158724	1.0	1.0	1.0	1.0	FAM47A
55578	1.0	1.0	2.4	2.5	FAM48A
51571	1.0	3.8	4.2	3.4	FAM49B
26240	1.0	2.6	2.6	1.0	FAM50B
120400	1.0	1.0	-5.7	1.0	FAM55A
120406	1.0	1.0	1.0	1.0	FAM55B
54827	1.0	8.3	1.0	5.6	FAM55D
54629	1.0	-7.5	-8.0	-7.7	FAM63B
348013	1.0	1.0	1.6	1.0	FAM70B
149647	1.0	2.3	1.0	1.0	FAM71A
161142	1.0	1.0	-7.7	-6.0	FAM71D
112703	1.0	1.0	1.0	1.0	FAM71E1
84691	1.0	1.0	1.0	-4.0	FAM71F1
346653	1.0	1.0	5.3	1.0	FAM71F2
401507	1.0	1.0	1.0	1.0	FAM74A1
645961	1.0	-3.0	1.0	1.0	FAM75C2
149297	1.0	1.0	-2.5	1.0	FAM78B
151393	1.0	2.4	1.8	1.0	FAM82A1
51115	1.0	1.0	2.3	1.0	FAM82B
84985	1.0	1.0	1.0	2.6	FAM83A
128876	1.0	1.0	-4.6	1.0	FAM83C
54854	1.0	1.0	1.0	1.0	FAM83E
113828	1.0	1.0	1.0	1.0	FAM83F
286077	1.0	1.0	1.0	1.0	FAM83H
645332	1.0	1.0	1.0	1.0	FAM86C2P
692099	1.0	1.0	1.0	1.0	FAM86DP
23625	1.0	3.2	3.1	1.0	FAM89B
55138	1.0	1.0	1.0	1.0	FAM90A1

441317	1.0	1.0	1.0	1.0	FAM90A7
100133036	1.0	1.0	1.0	1.0	FAM95B1
51647	1.0	1.0	1.0	1.0	FAM96B
147965	1.0	1.0	1.0	1.0	FAM98C
171483	1.0	1.0	1.0	1.0	FAM9B
171484	1.0	1.0	-5.3	1.0	FAM9C
2175	1.0	1.0	1.0	1.0	FANCA
2177	-4.6	5.7	1.0	4.8	FANCD2
2178	1.0	1.0	1.0	1.0	FANCE
57697	1.0	1.0	-3.5	1.0	FANCM
92565	1.0	1.0	1.0	1.0	FANK1
84188	1.0	-5.7	-5.5	-5.1	FAR1
55711	1.0	-7.0	-6.2	-7.0	FAR2
10160	1.0	1.0	1.0	1.0	FARP1
9855	1.0	1.0	1.0	1.0	FARP2
10667	1.5	-4.9	1.0	1.0	FARS2
355	1.0	2.2	1.0	1.0	FAS
356	1.0	1.0	1.0	-2.2	FASLG
2194	1.0	1.0	1.0	1.0	FASN
10922	1.7	1.0	1.0	1.0	FASTK
22868	8.5	1.0	1.0	1.0	FASTKD2
79072	1.0	1.0	1.0	1.0	FASTKD3
2195	1.0	5.6	1.0	1.0	FAT1
79633	1.0	-4.2	-8.4	-4.0	FAT4
54751	1.0	1.0	1.0	1.0	FBLIM1
345630	1.0	2.5	3.8	1.0	FBLL1
2192	1.0	1.0	1.0	1.0	FBLN1
129804	1.9	1.0	2.8	1.0	FBLN7
2201	1.0	1.0	1.0	3.1	FBN2
222235	1.0	1.0	3.8	1.0	FBXL13
64839	1.0	1.0	1.0	-4.5	FBXL17
26224	1.0	1.0	1.0	1.0	FBXL3
23194	23.3	15.0	1.0	15.0	FBXL7
55336	1.0	1.0	1.0	1.0	FBXL8
80204	1.0	-2.0	1.0	1.0	FBXO11
201456	1.0	1.0	1.0	1.0	FBXO15
115290	1.0	1.0	1.0	1.0	FBXO17
84893	1.0	1.0	1.0	1.0	FBXO18
26232	-4.4	1.0	1.0	1.0	FBXO2
126433	1.0	1.0	1.0	1.0	FBXO27
254170	1.0	-4.0	1.0	1.0	FBXO33
55030	1.0	1.0	1.0	1.0	FBXO34
130888	1.0	1.0	1.0	1.0	FBXO36
93611	1.0	1.0	4.3	1.0	FBXO44
25793	1.0	1.0	1.0	1.0	FBXO7
26268	1.6	-4.4	1.0	1.0	FBXO9
23291	1.0	1.0	-2.8	1.0	FBXW11
6468	1.0	1.0	-2.0	1.0	FBXW4

84261	1.0	1.0	1.0	2.2	FBXW9
83953	2.3	1.0	-1.8	1.0	FCAMR
2204	-3.2	1.0	1.0	1.0	FCAR
2208	1.0	1.0	1.0	1.0	FCER2
2212	1.0	-3.4	-3.3	-3.8	FCGR2A
2213	1.0	1.0	-6.1	-6.9	FCGR2B
2214	1.0	1.8	1.0	2.5	FCGR3A
2217	4.3	1.0	1.0	1.0	FCGRT
115548	1.0	1.0	1.0	1.0	FCHO2
9873	1.0	1.0	2.7	2.6	FCHSD2
8547	-5.1	1.0	1.0	1.0	FCN3
115350	1.0	1.0	1.0	-4.0	FCRL1
79368	1.0	1.0	-3.5	3.5	FCRL2
83417	1.0	1.0	-2.3	-2.3	FCRL4
83416	1.0	1.0	1.0	3.8	FCRL5
84824	1.0	1.0	1.0	1.0	FCRLA
127943	7.5	1.0	1.0	1.0	FCRLB
2222	1.0	1.0	1.0	1.0	FDFT1
2230	1.0	1.0	1.0	1.0	FDX1
91893	-9.1	1.0	1.7	1.0	FDXACB1
2232	1.0	1.0	1.0	1.0	FDXR
2235	1.0	3.4	1.0	5.0	FECH
10116	-2.2	1.0	-4.0	-4.0	FEM1B
439941	1.0	1.0	1.0	1.0	FER1L6-AS1
55612	-5.0	1.0	1.0	1.0	FERMT1
10979	1.0	-6.5	1.0	1.0	FERMT2
9637	1.0	1.0	1.0	1.0	FEZ2
55079	1.0	1.0	1.0	1.0	FEZF2
2245	1.0	5.1	1.0	1.0	FGD1
221472	1.0	1.0	1.0	1.0	FGD2
121512	1.0	-3.0	-2.1	1.0	FGD4
2246	1.0	1.0	1.0	2.5	FGF1
2257	1.0	-7.5	1.0	1.0	FGF12
2258	-2.4	-3.0	1.0	1.0	FGF13
9965	1.0	1.0	2.5	1.0	FGF19
26281	1.0	1.0	1.0	1.0	FGF20
26291	1.0	1.0	1.0	1.0	FGF21
27006	1.0	1.0	1.0	1.0	FGF22
2248	1.8	1.0	1.0	1.0	FGF3
2251	1.0	-2.3	1.0	1.0	FGF6
9982	1.0	3.7	1.0	1.0	FGFBP1
83888	1.0	1.0	4.9	1.0	FGFBP2
2260	1.0	1.0	1.0	1.0	FGFR1
26127	1.0	1.0	1.0	1.0	FGFR1OP2
2263	-2.1	-6.0	1.0	1.0	FGFR2
2261	1.0	1.0	1.0	1.0	FGFR3
2266	-2.8	1.0	-2.4	-2.2	FGG
2267	1.0	1.0	1.0	2.6	FGL1

2268	1.0	1.8	1.8	1.0	FGR
114827	1.0	1.0	1.0	1.0	FHAD1
2273	-18.7	1.0	1.0	1.0	FHL1
2275	1.0	1.0	1.0	1.0	FHL3
55137	-4.9	1.0	1.0	1.0	FIGN
27145	1.0	1.0	4.8	4.7	FILIP1
11259	2.0	4.1	3.7	3.7	FILIP1L
161247	1.0	2.3	2.5	1.0	FITM1
84922	1.0	1.0	1.0	1.6	FIZ1
51303	1.0	1.0	1.0	1.0	FKBP11
2280	1.0	1.0	1.0	1.0	FKBP1A
2286	1.0	1.0	1.0	1.0	FKBP2
2218	1.0	1.0	1.0	-2.8	FKTN
201163	1.0	5.6	3.2	1.0	FLCN
2312	1.0	1.0	1.0	1.0	FLG
2316	2.6	1.0	1.0	4.3	FLNA
2318	-1.6	1.0	1.0	1.0	FLNC
2319	1.0	1.0	1.0	1.0	FLOT2
23768	-2.5	1.0	1.0	-3.0	FLRT2
2321	1.0	1.0	1.0	1.0	FLT1
2324	1.7	1.0	1.0	1.0	FLT4
28982	1.0	1.0	2.4	1.0	FLVCR1
84256	1.0	-5.4	-6.6	-7.5	FLYWCH1
56776	1.0	1.0	1.0	-3.4	FMN2
2326	1.0	1.0	-2.1	-1.8	FMO1
2330	1.0	1.0	1.0	1.0	FMO5
2331	1.0	1.0	1.0	1.0	FMOD
252995	1.0	1.0	1.0	1.0	FNDC5
2339	1.0	1.0	1.0	1.0	FNTA
2342	8.5	1.0	1.0	1.0	FNTB
219595	1.0	1.0	1.0	1.0	FOLH1B
8061	1.0	1.0	3.0	1.0	FOSL1
3169	1.0	1.0	1.0	1.0	FOXA1
3170	1.5	1.0	1.0	3.6	FOXA2
3171	1.0	2.8	1.0	1.0	FOXA3
2298	-7.2	1.0	1.0	-6.7	FOXD4
2304	-3.8	1.0	1.0	1.0	FOXE1
2295	1.0	1.0	1.0	1.0	FOXF2
2299	1.0	1.0	2.9	1.0	FOXI1
3607	1.0	1.0	1.0	1.0	FOXK2
668	1.0	1.0	1.0	1.0	FOXL2
2305	1.0	1.0	1.0	1.0	FOXM1
1112	1.0	1.0	1.0	4.3	FOXN3
2308	3.2	5.8	3.2	1.0	FOXO1
27086	1.0	1.0	1.0	2.6	FOXP1
116113	1.0	1.0	1.0	1.0	FOXP4
94234	1.0	1.0	1.0	1.0	FOXQ1
283150	2.0	1.0	1.0	1.0	FOXR1

80020	-3.9	1.0	1.0	1.0	FOXRED2
2307	1.5	1.0	1.0	1.0	FOXS1
2356	1.0	1.0	2.5	1.0	FPGS
2357	1.5	1.0	1.0	1.0	FPR1
2358	1.0	1.0	3.5	1.0	FPR2
118924	1.0	-3.8	1.0	-3.5	FRA10AC1
10023	1.0	1.0	-4.9	1.0	FRAT1
158326	1.0	1.0	5.7	1.0	FREM1
448831	1.6	4.8	1.0	1.0	FRG2
257019	1.0	1.0	1.0	1.0	FRMD3
9758	1.0	1.0	-6.1	1.0	FRMPD4
10129	1.0	1.0	-7.0	1.0	FRY
6624	1.0	1.0	1.0	1.0	FSCN1
25794	1.0	1.0	1.0	1.0	FSCN2
29999	1.0	3.4	1.0	1.0	FSCN3
2492	1.0	1.0	1.0	1.0	FSHR
161835	1.0	1.0	-8.4	-3.5	FSIP1
10272	1.0	1.0	5.4	1.0	FSTL3
23105	1.0	-2.7	1.0	1.0	FSTL4
10841	1.9	1.0	1.0	1.0	FTCD
2512	-1.8	-2.0	1.0	1.0	FTL
94033	1.0	1.0	1.0	1.0	FTMT
23070	1.0	2.8	4.3	3.0	FTSJD2
8880	-2.3	-2.1	1.0	1.0	FUBP1
2519	1.0	1.0	1.0	1.0	FUCA2
84750	1.0	1.0	1.0	1.0	FUT10
170384	-1.5	1.0	1.0	-6.3	FUT11
2527	-1.6	1.0	1.0	1.0	FUT5
2528	1.0	1.0	1.0	1.0	FUT6
2529	1.0	1.0	1.0	1.0	FUT7
10690	-6.1	1.0	1.0	1.0	FUT9
2395	1.0	1.0	2.7	1.8	FXN
9513	8.9	1.0	1.0	1.0	FXR2
5349	1.0	1.0	5.2	1.0	FXYD3
53828	1.0	3.0	1.0	1.0	FXYD4
53827	-17.7	-3.7	-6.5	-5.0	FXYD5
53822	1.0	1.0	1.0	2.2	FXYD7
2533	1.0	1.0	-5.9	1.0	FYB
2534	1.0	2.6	2.7	2.7	FYN
8322	1.0	1.0	1.0	1.0	FZD4
10146	1.0	-2.5	2.5	1.0	G3BP1
9908	1.0	2.3	2.3	1.0	G3BP2
2548	1.0	1.0	1.0	1.0	GAA
9846	3.0	1.0	6.7	1.0	GAB2
11345	1.0	1.0	1.0	1.0	GABARAPL2
2550	1.0	1.0	1.0	1.0	GABBR1
2553	1.0	1.0	1.0	1.0	GABPB1
126626	1.0	1.0	1.0	-4.5	GABPB2

2554	-1.6	-5.2	1.0	1.0	GABRA1
2556	1.0	1.0	1.0	1.0	GABRA3
2558	-3.5	8.4	8.6	1.0	GABRA5
2560	1.0	1.0	1.0	1.0	GABRB1
2562	-5.0	1.0	5.2	1.0	GABRB3
2563	1.0	1.0	1.0	1.0	GABRD
2564	1.0	1.8	1.0	1.0	GABRE
2565	1.0	1.0	1.0	1.0	GABRG1
55879	1.0	1.0	2.4	2.7	GABRQ
2569	1.0	1.0	1.0	2.2	GABRR1
2571	1.0	1.0	4.2	3.3	GAD1
2572	-1.8	1.0	1.0	1.0	GAD2
10912	1.0	1.6	1.0	1.0	GADD45G
90480	1.0	1.0	2.0	1.0	GADD45GIP1
2543	1.0	1.0	1.0	1.0	GAGE1
2579	1.0	1.0	1.0	1.0	GAGE7
2580	1.0	1.0	1.0	1.0	GAK
51083	1.0	1.0	1.0	1.0	GAL
2581	1.0	1.0	1.0	1.0	GALC
130589	1.0	1.0	1.0	1.0	GALM
2588	1.0	1.0	1.0	1.0	GALNS
2590	1.0	1.0	1.0	1.0	GALNT2
11227	1.0	1.0	1.0	1.0	GALNT5
51809	-3.6	1.0	1.0	1.0	GALNT7
57452	1.0	1.0	1.0	1.6	GALNTL1
374378	1.0	2.3	1.0	1.0	GALNTL4
8139	1.0	6.6	1.0	5.9	GAN
2596	1.0	1.0	1.0	1.0	GAP43
26130	1.0	1.0	3.3	3.4	GAPVD1
2620	1.0	1.0	1.0	1.0	GAS2
246176	2.3	6.4	5.0	5.1	GAS2L2
8522	1.0	1.0	4.5	1.0	GAS7
2626	-2.5	2.7	1.0	-10.7	GATA4
352954	3.3	1.0	-2.2	-5.4	GATS
57733	1.0	1.0	1.0	1.0	GBA3
2631	1.0	1.0	1.0	1.0	GBAS
2632	1.0	1.0	1.0	1.0	GBE1
26301	-5.2	1.0	1.0	1.0	GBGT1
115362	1.0	1.0	1.0	1.0	GBP5
163351	1.0	1.0	1.0	1.0	GBP6
9648	1.0	-5.7	-8.0	-7.1	GCC2
94104	-1.6	2.9	1.0	1.0	GCFC1
2641	1.0	1.0	-6.9	1.0	GCG
2646	2.7	1.0	1.0	1.0	GCKR
2650	1.0	1.0	-7.3	1.0	GCNT1
9245	1.0	1.0	-7.0	1.0	GCNT3
51301	1.0	1.0	1.0	1.0	GCNT4
9615	1.0	1.0	3.8	1.0	GDA

54332	1.0	1.0	-6.1	1.0	GDAP1
78997	1.0	1.0	1.0	1.0	GDAP1L1
2657	3.4	7.3	4.5	1.0	GDF1
2662	1.0	1.0	1.0	1.0	GDF10
2658	1.0	1.0	1.0	1.0	GDF2
392255	1.7	-2.8	1.0	1.0	GDF6
2665	1.0	1.0	1.0	1.0	GDI2
2668	1.0	1.0	1.0	2.1	GDNF
220032	1.0	1.0	1.0	1.0	GDPD4
2669	1.0	1.0	1.0	1.0	GEM
25929	1.0	1.0	1.0	1.0	GEMIN5
79833	1.0	3.6	1.0	1.0	GEMIN6
492303	-5.8	1.0	1.0	1.0	GEMIN8P4
348654	1.0	-7.5	-4.7	-8.1	GEN1
2671	-3.3	1.0	1.0	1.0	GFER
2672	1.0	1.0	1.0	1.0	GFI1
8328	1.6	1.0	1.0	2.5	GFI1B
85476	1.0	2.5	2.8	1.0	GFM1
84340	1.0	1.0	1.0	1.0	GFM2
81577	1.0	1.0	2.3	1.0	GFOD2
2675	1.0	1.0	1.0	-2.7	GFRA2
26088	1.0	3.6	3.7	3.1	GGA1
79017	1.0	1.0	1.0	1.0	GGCT
199720	-7.1	-3.6	1.0	1.0	GGN
79893	1.0	5.1	8.3	5.9	GGNBP2
2679	4.3	1.0	1.0	1.0	GGT3P
124975	3.4	1.0	1.0	1.0	GGT6
2686	9.6	1.0	1.0	1.0	GGT7
2681	1.0	1.0	3.1	1.0	GGTA1P
91227	1.0	1.0	1.0	1.0	GGTLC2
84514	-12.2	1.0	1.0	1.0	GHDC
2691	1.0	1.0	1.0	1.0	GHRH
2692	1.9	1.0	1.0	1.0	GHRHR
51738	1.0	1.0	2.2	2.6	GHRL
84657	1.0	1.0	1.0	1.0	GHRLOS2
64599	1.8	1.0	1.0	1.0	GIGYF1
26058	-1.7	-3.6	-3.6	-3.7	GIGYF2
54826	1.0	1.0	1.0	1.0	GIN1
51659	1.0	1.0	1.0	1.0	GINS2
84296	-1.7	1.0	1.0	1.0	GINS4
2695	1.0	1.0	1.0	1.0	GIP
54810	1.0	-8.1	-7.9	-5.9	GIPC2
28964	1.0	1.0	1.0	1.0	GIT1
9815	1.0	1.0	4.8	1.0	GIT2
84694	1.0	-5.3	-6.3	-8.3	GJA10
349149	1.0	2.6	1.0	1.8	GJC3
2710	1.0	1.0	1.0	1.0	GK
56287	1.0	1.0	1.0	1.0	GKN1

200504	1.8	1.0	1.0	1.0	GKN2
2717	1.0	1.0	1.0	1.0	GLA
79411	1.0	1.0	1.0	1.0	GLB1L
89944	1.0	1.0	1.0	1.0	GLB1L2
112937	1.0	1.0	1.0	1.0	GLB1L3
2731	1.0	4.4	4.8	4.3	GLDC
2736	1.0	1.0	2.6	1.0	GLI2
2738	1.0	1.0	1.0	1.0	GLI4
152007	1.0	1.0	1.0	1.0	GLIPR2
169792	-1.9	1.0	1.0	1.0	GLIS3
84850	1.0	1.0	1.0	1.0	GLIS3-AS1
11146	1.0	1.0	1.0	1.0	GLMN
392465	1.0	1.0	1.0	1.0	GLOD5
9340	1.0	1.9	2.2	1.0	GLP2R
2741	1.6	1.0	1.0	1.0	GLRA1
51022	1.0	1.0	1.0	1.0	GLRX2
51218	-4.3	-2.5	-2.3	1.0	GLRX5
144423	1.0	1.0	-3.9	1.0	GLT1D1
79709	1.0	1.0	1.0	1.0	GLT25D1
23127	1.0	1.0	1.0	1.0	GLT25D2
83468	1.0	1.0	1.0	1.0	GLT8D2
80772	1.0	1.0	1.0	1.0	GLTPD1
29998	1.7	1.0	1.0	1.0	GLTSCR1
10249	1.0	-1.7	-4.8	-2.9	GLYAT
644076	2.4	-3.3	-2.0	-2.6	GLYCAM1
2762	1.0	1.0	1.0	1.0	GMDS
51291	1.0	1.0	1.0	1.0	GMIP
2765	1.0	3.6	3.0	1.0	GML
29926	-2.1	1.0	1.0	1.0	GMPPA
2766	1.0	-1.9	-1.7	1.0	GMPR
8833	-5.3	1.0	1.0	1.0	GMPS
10672	1.0	1.0	1.0	1.0	GNA13
2770	1.0	1.0	1.0	1.0	GNAI1
2774	-4.2	-3.4	1.0	1.0	GNAL
2778	1.0	1.0	1.0	1.0	GNAS
2782	1.0	1.0	1.0	-3.3	GNB1
2783	1.0	1.0	1.0	1.0	GNB2
10399	1.0	1.0	1.0	1.0	GNB2L1
2784	1.0	2.6	1.0	1.0	GNB3
2790	3.3	1.0	1.0	1.0	GNG10
2791	1.0	1.0	1.0	1.0	GNG11
54331	1.0	1.0	2.3	1.0	GNG2
2786	1.0	1.0	1.0	1.6	GNG4
2787	1.0	1.0	1.0	1.0	GNG5
2793	1.0	1.0	-7.5	1.0	GNGT2
54552	1.0	1.0	1.0	-2.6	GNL3L
2796	1.0	1.0	1.0	1.0	GNRH1
2797	1.0	1.0	3.5	1.0	GNRH2

2798	1.0	1.0	1.0	1.0	GNRHR
2801	1.0	1.0	1.0	-4.1	GOLGA2
55592	1.0	1.0	-3.9	1.0	GOLGA2P5
9950	1.0	1.0	1.0	1.0	GOLGA5
342096	-2.3	1.0	1.0	1.0	GOLGA6A
727832	1.0	1.0	1.0	1.0	GOLGA6L6
51125	-1.7	1.0	1.0	1.0	GOLGA7
390535	1.0	-4.7	1.0	1.0	GOLGA8E
283796	1.0	1.0	-2.1	1.0	GOLGA8IP
127845	1.0	1.0	1.0	1.0	GOLT1A
54856	5.4	1.0	1.0	1.0	GON4L
57120	1.0	1.0	-5.6	1.0	GOPC
92344	-2.3	-3.2	1.0	1.0	GORAB
64689	1.7	-2.8	-4.1	1.0	GORASP1
9570	1.0	1.0	1.0	1.0	GOSR2
2811	1.0	1.0	1.0	1.0	GP1BA
2813	-6.9	1.0	1.0	1.0	GP2
2815	1.0	-5.6	1.0	1.0	GP9
150763	1.0	1.0	1.0	1.0	GPAT2
63906	1.0	1.0	4.5	1.0	GPATCH3
54865	1.0	1.0	1.0	1.0	GPATCH4
65056	1.0	3.3	1.0	1.0	GPBP1
2262	-5.4	-2.8	-3.0	1.0	GPC5
2819	2.6	1.0	1.0	-4.2	GPD1
2820	1.0	1.0	1.0	-4.1	GPD2
2852	1.0	1.0	1.0	-2.5	GPER
27238	1.0	1.0	2.4	1.0	GPKOW
2823	7.5	1.0	9.1	1.0	GPM6A
2824	1.0	4.9	1.0	1.0	GPM6B
11321	1.0	1.0	1.0	1.0	GPN1
221188	-5.7	1.0	-2.5	1.0	GPR114
221393	3.3	1.0	1.0	1.0	GPR115
84435	1.0	1.0	1.0	1.0	GPR123
64582	9.1	1.0	1.0	1.0	GPR135
4935	1.0	1.0	-2.5	1.0	GPR143
115330	1.0	1.0	1.0	1.0	GPR146
344758	1.0	-4.2	1.0	-6.1	GPR149
387509	1.7	1.0	-7.2	1.0	GPR153
151556	1.0	1.0	-2.6	-2.5	GPR155
165829	1.0	1.0	1.0	1.0	GPR156
27239	1.0	1.0	1.0	1.0	GPR162
2840	1.0	-4.0	1.0	2.8	GPR17
29909	1.0	1.0	1.0	1.0	GPR171
79581	1.0	1.0	-2.6	1.0	GPR172A
84636	1.0	1.0	2.5	1.0	GPR174
11245	3.3	1.0	1.0	1.0	GPR176
160897	1.0	3.1	2.8	5.1	GPR180
1880	1.0	1.0	1.0	1.0	GPR183

2849	1.0	1.0	1.0	1.0	GPR26
2827	1.0	1.0	2.0	1.0	GPR3
2854	1.0	1.0	1.0	1.0	GPR32
2857	1.0	1.0	1.0	-2.2	GPR34
2859	1.6	-2.5	1.0	1.0	GPR35
2863	1.0	1.0	1.0	1.0	GPR39
9290	1.0	1.0	1.0	2.0	GPR55
2830	1.0	1.0	-2.2	1.0	GPR6
8477	1.0	1.0	1.0	1.0	GPR65
10936	1.0	1.0	1.0	1.0	GPR75
27202	1.5	1.0	1.0	1.0	GPR77
27201	1.0	1.0	1.0	1.0	GPR78
27197	1.0	1.0	1.0	1.0	GPR82
53831	1.0	1.0	1.0	1.0	GPR84
53836	1.0	-3.4	-5.4	-3.4	GPR87
54112	1.0	1.0	1.0	1.0	GPR88
51463	1.0	1.0	-7.0	-7.6	GPR89B
84059	1.0	1.0	1.0	1.0	GPR98
9052	1.0	1.0	1.0	1.0	GPRC5A
55890	1.0	1.0	3.2	1.0	GPRC5C
285513	1.0	1.0	1.0	1.0	GPRIN3
2874	1.0	1.0	2.5	2.5	GPS2
26086	1.0	3.6	3.1	6.0	GPSM1
2878	1.0	3.4	3.7	2.5	GPX3
2880	1.0	1.0	1.0	1.0	GPX5
257202	1.0	1.0	1.0	1.0	GPX6
2882	-4.4	1.0	1.0	1.0	GPX7
57476	1.0	1.0	1.0	1.0	GRAMD1B
65983	1.0	2.3	1.0	3.0	GRAMD3
10750	4.4	-3.4	-3.4	2.9	GRAP
400581	1.0	1.0	3.6	1.0	GRAPL
2887	1.0	-2.2	1.0	-2.6	GRB10
2885	1.5	1.0	5.2	1.0	GRB2
80000	1.0	1.0	-3.2	1.0	GREB1L
29841	-1.9	1.0	1.0	1.0	GRHL1
79977	1.0	1.0	1.0	1.0	GRHL2
57822	3.8	3.5	1.0	1.0	GRHL3
2890	1.0	1.0	1.0	1.0	GRIA1
2894	1.0	1.0	2.8	1.0	GRID1
2898	-6.3	1.0	1.0	1.0	GRIK2
2902	1.0	1.0	1.0	1.0	GRIN1
2903	1.0	-3.9	1.0	1.0	GRIN2A
116443	1.0	1.0	1.0	1.0	GRIN3A
23426	1.0	-2.8	-3.1	-3.1	GRIP1
80852	1.0	-7.3	-7.4	-8.4	GRIP2
56850	1.0	1.0	-5.4	1.0	GRIPAP1
131890	1.0	1.0	5.9	1.0	GRK7
2911	1.0	1.0	1.0	1.0	GRM1

2913	1.0	1.0	5.3	1.0	GRM3
2914	2.1	1.0	1.0	1.0	GRM4
2915	1.0	1.0	1.0	1.0	GRM5
2917	1.0	1.0	1.0	1.0	GRM7
2918	1.9	1.0	1.0	1.0	GRM8
2922	1.0	1.0	2.3	2.3	GRP
2925	1.0	1.0	1.0	1.0	GRPR
56169	1.0	1.0	-2.7	1.0	GSDMC
79792	1.0	1.0	1.0	1.0	GSDMD
83445	1.0	1.0	1.0	1.0	GSG1
23708	1.0	2.0	4.8	1.0	GSPT2
2936	1.0	1.0	-1.7	1.0	GSR
2941	1.0	3.8	3.3	3.9	GSTA4
79807	1.0	-3.1	1.0	1.0	GSTCD
2944	1.0	1.0	2.2	1.0	GSTM1
2952	1.0	1.0	1.0	1.0	GSTT1
2953	-17.3	1.0	1.0	1.0	GSTT2
170825	1.0	1.0	1.0	1.0	GSX2
79712	1.0	1.0	1.0	1.0	GTDC1
2958	-3.3	1.0	1.0	-3.5	GTF2A2
2965	1.0	1.0	1.0	-5.2	GTF2H1
730394	2.2	-2.6	1.0	1.0	GTF2H2D
9569	1.0	2.5	1.0	1.0	GTF2IRD1
389524	1.0	-4.6	1.0	1.0	GTF2IRD2B
2971	1.0	1.0	1.0	1.0	GTF3A
2975	1.0	1.0	1.0	2.2	GTF3C1
2976	1.0	1.0	1.0	1.0	GTF3C2
9330	1.0	1.0	1.0	1.0	GTF3C3
9329	4.6	1.0	-3.2	1.0	GTF3C4
9328	1.0	1.0	1.0	-3.6	GTF3C5
85865	1.0	-6.5	-1.8	1.0	GTPBP10
54676	1.0	1.0	1.0	1.0	GTPBP2
26164	2.9	1.0	1.0	1.0	GTPBP5
29083	1.0	1.0	1.0	1.0	GTPBP8
51512	1.0	1.0	-5.0	-3.2	GTSE1
121355	1.0	1.0	1.0	1.0	GTSF1
2978	1.0	1.0	-4.4	1.0	GUCA1A
9626	1.0	1.0	1.0	-6.3	GUCA1C
2980	1.0	1.0	1.0	1.0	GUCA2A
2982	1.0	1.0	-3.4	1.0	GUCY1A3
2974	1.0	1.0	1.0	1.0	GUCY1B2
2983	1.0	-8.0	-10.0	1.0	GUCY1B3
2984	1.0	1.0	1.0	-3.6	GUCY2C
3000	1.0	1.0	1.0	5.7	GUCY2D
2987	1.0	1.0	2.7	1.7	GUK1
51454	1.0	-6.0	-8.3	1.0	GULP1
728411	1.0	1.0	1.0	1.0	GUSBP1
283464	1.0	1.0	1.0	1.0	GXYLT1

727936	1.0	1.0	-6.2	1.0	GXYLT2
2992	1.0	1.0	-6.3	1.0	GYG1
8908	1.0	1.0	-3.2	1.0	GYG2
2994	1.0	1.0	2.2	1.0	GYPB
2996	1.0	1.0	4.8	5.4	GYPE
3001	1.0	1.0	1.0	1.0	GZMA
3002	1.0	-3.0	-2.9	-3.0	GZMB
2999	1.0	2.5	1.0	1.0	GZMH
283120	1.0	1.0	1.0	1.0	H19
341567	1.0	1.0	1.0	1.0	H1FNT
132243	13.8	1.0	1.0	1.0	H1FOO
8971	1.7	1.0	1.0	-2.5	H1FX
55766	2.1	-5.0	-5.0	-8.3	H2AFJ
767811	1.0	-4.4	1.0	1.0	H2BFXP
23498	1.0	1.0	5.6	1.0	HAAO
57531	1.0	3.1	3.1	5.7	HACE1
9421	1.0	4.7	6.3	1.0	HAND1
54363	1.0	-2.8	1.0	1.0	HAO1
1404	1.0	1.0	2.3	1.0	HAPLN1
60484	1.0	-3.2	1.0	1.0	HAPLN2
145864	1.0	1.0	1.0	3.9	HAPLN3
3036	1.0	1.0	1.0	1.0	HAS1
115106	1.0	1.0	5.1	1.0	HAUS1
55142	1.0	1.0	1.0	1.0	HAUS2
3043	1.0	1.0	1.0	2.4	HBB
3044	1.0	2.3	1.0	1.0	HBBP1
1839	1.0	1.0	1.0	1.0	HBEGF
3047	1.0	2.1	2.3	1.0	HBG1
26959	-1.9	2.7	2.6	3.0	HBP1
10767	-1.6	1.0	1.0	1.0	HBS1L
3050	1.0	-4.1	1.0	1.0	HBZ
8843	1.0	4.4	3.5	1.0	HCAR3
3052	-2.1	4.3	4.4	1.0	HCCS
54985	1.0	-3.2	1.0	1.0	HCFC1R1
29915	1.0	-4.2	6.0	1.0	HCFC2
285834	1.0	-8.4	1.0	-8.3	HCG22
80868	-13.3	1.0	1.0	2.6	HCG4B
610	1.0	-2.1	1.0	1.0	HCN2
57657	1.0	2.8	3.9	2.4	HCN3
10866	1.0	4.2	3.3	1.0	HCP5
10870	1.0	1.0	1.0	-2.2	HCST
83933	1.0	1.0	1.0	1.0	HDAC10
3066	1.0	-2.5	-2.5	-2.4	HDAC2
9759	1.0	4.6	2.1	1.0	HDAC4
10013	1.0	1.0	1.0	1.0	HDAC6
51564	1.0	1.0	-4.3	1.0	HDAC7
55869	1.0	-2.6	-2.5	-2.4	HDAC8
9734	1.0	1.0	1.0	1.0	HDAC9

51020	1.0	1.0	-5.7	-2.6	HDDC2
3068	1.0	1.0	1.0	1.0	HDGF
84717	1.0	1.0	1.0	4.1	HDGFRP2
3069	-11.9	1.0	-4.0	1.0	HDLBP
55127	1.0	1.8	1.0	1.0	HEATR1
54919	1.0	1.0	1.0	1.0	HEATR2
399671	1.0	1.0	1.0	1.0	HEATR4
339766	1.0	1.0	1.0	4.6	HEATR7B1
23593	-2.0	1.0	1.0	-7.2	HEBP2
25831	1.0	1.0	1.0	1.0	HECTD1
143279	1.0	1.0	-5.9	1.0	HECTD2
79654	1.0	1.0	1.0	1.0	HECTD3
3070	-1.8	-2.9	1.0	1.0	HELLS
55363	1.0	1.0	1.0	-4.8	HEMGN
113802	1.0	2.4	1.0	1.0	HENMT1
220296	1.0	6.8	1.0	1.0	HEPACAM
253012	1.0	1.0	2.7	1.0	HEPACAM2
26091	1.0	1.0	1.0	1.0	HERC4
64224	1.5	1.0	1.0	1.0	HERPUD2
54626	1.0	1.0	1.0	1.0	HES2
57801	5.9	1.0	1.0	8.1	HES4
55502	1.0	-3.2	-3.3	-3.3	HES6
84667	1.0	1.0	-4.7	-2.4	HES7
124790	1.0	1.0	3.0	1.0	HEXIM2
26508	1.0	2.4	1.0	1.0	HEYL
3077	1.0	1.0	1.0	1.0	HFE
148738	1.0	1.0	1.0	1.0	HFE2
3081	1.0	1.0	1.0	1.0	HGD
3082	1.0	1.0	1.0	1.0	HGF
9146	1.8	1.0	1.0	1.0	HGS
138050	2.5	4.6	1.0	1.0	HGSNAT
84439	1.0	1.0	1.0	1.0	HHIPL1
64645	19.3	1.0	2.3	1.0	HIAT1
26275	1.0	1.0	1.0	1.0	HIBCH
3090	2.0	1.0	8.3	1.0	HIC1
55662	1.0	1.7	1.0	2.7	HIF1AN
64344	1.0	1.0	1.0	1.0	HIF3A
51751	1.0	1.0	1.0	1.0	HIGD1B
192286	1.0	1.0	1.0	1.0	HIGD2A
29923	1.0	1.0	1.0	1.0	HILPDA
373861	1.0	1.0	-3.8	1.0	HILS1
3092	-1.5	3.9	1.0	1.0	HIP1
9026	1.0	1.0	1.0	1.0	HIP1R
204851	2.6	1.0	-2.4	1.0	HIPK1
28996	-6.7	-2.0	1.0	-2.4	HIPK2
147746	1.0	1.0	1.0	-3.9	HIPK4
7290	1.0	1.0	1.9	1.0	HIRA
8479	1.0	1.0	-3.6	1.0	HIRIP3

3009	1.0	1.0	-6.5	-3.1	HIST1H1B
3008	1.0	1.0	1.0	4.6	HIST1H1E
8334	1.0	4.6	7.5	2.6	HIST1H2AC
3012	1.0	1.0	1.0	1.0	HIST1H2AE
8330	1.0	1.0	1.0	2.6	HIST1H2AK
8332	1.0	-4.1	-4.0	1.0	HIST1H2AL
8347	1.0	1.0	1.0	1.0	HIST1H2BC
8343	1.0	1.0	-1.8	1.0	HIST1H2BF
8339	1.0	1.0	1.0	1.0	HIST1H2BG
8341	1.0	1.0	-2.5	1.0	HIST1H2BN
8350	1.0	1.0	1.0	5.6	HIST1H3A
8351	1.0	1.0	1.0	1.0	HIST1H3D
8357	-1.5	-2.7	1.0	1.0	HIST1H3H
8354	1.0	1.0	1.0	1.0	HIST1H3I
8356	2.0	-4.5	1.0	1.0	HIST1H3J
8359	1.0	1.0	1.0	1.9	HIST1H4A
8366	1.0	1.0	1.0	1.0	HIST1H4B
8361	-2.0	-3.0	-2.8	1.0	HIST1H4F
8362	1.0	-2.5	1.0	-2.4	HIST1H4K
8368	1.8	-6.3	-6.3	1.0	HIST1H4L
723790	1.0	1.0	4.7	1.0	HIST2H2AA4
8338	1.0	-2.9	1.0	-3.6	HIST2H2AC
8349	1.0	-3.8	1.0	1.0	HIST2H2BE
92815	1.0	1.0	1.0	1.0	HIST3H2A
3096	-37.5	1.0	5.5	1.0	HIVEP1
59269	1.0	1.0	1.0	1.0	HIVEP3
3101	1.6	-5.2	1.0	1.0	HK3
284459	1.0	1.0	1.0	1.0	HKR1
3105	2.1	1.0	1.0	1.0	HLA-A
3106	1.0	-3.2	1.0	-5.9	HLA-B
3107	1.6	1.0	-5.7	1.0	HLA-C
3111	1.0	1.0	1.0	1.0	HLA-DOA
3112	1.9	1.0	1.0	1.0	HLA-DOB
3115	1.0	4.7	1.0	1.0	HLA-DPB1
3116	1.0	1.0	1.0	1.0	HLA-DPB2
3119	1.0	1.0	1.0	3.4	HLA-DQB1
3120	-7.1	1.0	-1.5	1.0	HLA-DQB2
3123	1.9	1.0	-1.8	1.0	HLA-DRB1
3127	-7.9	1.0	2.7	1.6	HLA-DRB5
3137	1.0	2.8	3.0	2.8	HLA-J
3139	1.7	1.0	1.0	1.0	HLA-L
81502	1.0	5.4	7.0	1.0	HM13
79618	1.0	1.0	1.0	1.0	HMBOX1
3145	1.0	-2.6	1.0	1.0	HMBS
10362	1.0	1.0	1.0	1.0	HMG20B
8091	1.0	1.0	1.0	1.0	HMGA2
54511	1.0	1.0	1.0	1.0	HMGCLL1
3156	1.0	3.3	5.8	4.4	HMGCR

3157	1.0	1.0	1.0	1.0	HMGCS1
3158	1.0	1.0	1.0	1.0	HMGCS2
3150	1.0	1.0	1.0	-1.5	HMGN1
3151	1.0	1.0	2.4	1.0	HMGN2
9324	-8.0	-3.8	1.0	1.0	HMGN3
10042	1.0	1.0	2.8	1.0	HMGXB4
57824	-1.6	1.0	1.0	1.0	HMHB1
3161	1.0	1.0	1.0	2.9	HMMR
3162	1.0	1.0	1.0	-2.1	HMOX1
90861	1.0	1.0	-5.3	1.0	HN1L
283460	1.0	4.5	4.5	1.0	HNF1A-AS1
3174	1.0	1.0	1.0	1.0	HNF4G
3176	1.7	-3.7	-5.8	-6.0	HNMT
10949	1.0	1.0	1.0	1.0	HNRNPA0
3178	1.0	-2.6	1.0	1.0	HNRNPA1
144983	1.0	-3.4	1.0	1.0	HNRNPA1L2
3181	-2.0	1.0	-3.0	1.0	HNRNPA2B1
220988	1.0	1.0	1.0	1.0	HNRNPA3
3183	1.0	-2.5	-2.4	-2.4	HNRNPC
3188	1.0	2.6	2.5	2.4	HNRNPH2
3189	2.1	4.1	3.1	2.8	HNRNPH3
3191	1.0	1.0	4.9	1.0	HNRNPL
221092	1.0	-3.7	-3.6	-4.2	HNRNPUL2
9987	2.3	1.0	-3.2	-3.2	HNRPDL
112817	1.0	1.0	1.0	1.0	HOGA1
9456	1.0	1.0	1.0	1.0	HOMER1
57594	1.0	1.0	1.0	1.0	HOMEZ
51361	1.0	1.0	1.0	1.0	HOOK1
84376	1.0	-5.0	1.0	1.0	HOOK3
84072	1.0	1.0	4.1	3.2	HORMAD1
3198	1.0	-1.6	-1.6	1.0	HOXA1
221883	1.0	1.0	1.0	1.0	HOXA11-AS1
3209	-2.9	1.0	1.0	1.0	HOXA13
3201	1.0	1.0	1.0	1.0	HOXA4
3212	1.0	1.0	1.0	-2.8	HOXB2
3213	1.0	1.0	1.0	1.0	HOXB3
3214	1.0	1.0	1.0	-3.5	HOXB4
3215	-1.9	1.0	1.0	1.0	HOXB5
3219	1.0	1.0	3.4	2.3	HOXB9
3223	1.0	1.0	1.0	1.0	HOXC6
3231	1.0	1.0	4.1	3.8	HOXD1
3236	1.0	1.0	1.0	1.7	HOXD10
3237	1.5	1.0	-1.8	-2.0	HOXD11
3238	1.0	1.0	1.0	1.0	HOXD12
3232	-10.4	7.4	5.5	1.0	HOXD3
3235	1.9	4.5	1.0	1.0	HOXD9
51440	1.0	1.0	-3.1	1.0	HPCAL4
84842	1.0	1.0	1.0	1.0	HPDL

27306	3.2	1.0	1.0	1.0	HPGDS
3250	1.0	1.0	2.4	1.0	HPR
11234	1.0	1.0	1.0	-2.8	HPS5
79803	1.0	1.0	1.0	1.0	HPS6
10855	1.0	1.0	1.0	1.0	HPSE
60495	1.0	1.0	2.3	1.0	HPSE2
652764	1.0	1.0	2.9	1.0	HPX-2
55806	1.0	1.0	1.0	1.0	HR
117245	-4.5	1.0	1.0	1.0	HRASLS5
3270	1.0	1.0	2.8	1.0	HRC
3273	1.0	1.0	2.2	1.0	HRG
11255	-1.9	1.0	1.0	1.0	HRH3
8739	1.0	1.0	1.0	1.0	HRK
9953	1.0	1.0	2.7	1.0	HS3ST3B1
9951	1.0	1.0	-3.1	1.0	HS3ST4
90161	1.0	1.0	-3.0	1.0	HS6ST2
266722	-1.5	1.0	1.0	-6.1	HS6ST3
3281	1.0	-5.1	-4.8	-4.4	HSBP1
150274	1.0	1.0	1.0	-3.4	HSCB
3295	1.0	1.0	1.0	1.0	HSD17B4
8630	1.0	1.0	1.0	1.0	HSD17B6
51478	1.0	1.0	1.0	-2.5	HSD17B7
80270	-2.7	1.0	3.0	1.0	HSD3B7
84263	1.0	-3.6	-4.0	1.0	HSDL2
3298	1.0	4.4	5.7	1.0	HSF2
84941	1.0	1.0	1.0	1.0	HSH2D
259217	1.9	1.0	1.0	1.0	HSPA12A
51182	1.0	1.0	1.0	1.0	HSPA14
3303	1.0	1.0	1.0	2.6	HSPA1A
3305	1.0	1.0	-6.7	1.0	HSPA1L
3315	1.0	1.0	-7.6	1.0	HSPB1
8988	1.0	1.0	1.0	1.0	HSPB3
27129	1.0	1.0	1.0	1.0	HSPB7
79663	1.0	1.0	1.0	1.0	HSPBAP1
23640	1.6	1.0	1.0	1.0	HSPBP1
3329	1.0	1.0	1.0	1.0	HSPD1
3336	1.0	2.6	2.6	1.0	HSPE1
3339	1.0	1.0	1.0	1.0	HSPG2
10808	1.0	1.0	-3.1	1.0	HSPH1
27336	1.0	1.0	1.0	1.0	HTATSF1
401233	1.0	1.0	2.8	1.0	HTATSF1P2
3350	1.0	1.0	-3.5	1.0	HTR1A
3352	1.0	1.0	1.0	1.0	HTR1D
3359	1.0	1.0	1.0	3.8	HTR3A
9177	1.0	1.0	1.0	1.0	HTR3B
27429	1.0	1.0	5.1	1.0	HTRA2
94031	1.0	1.0	1.0	1.0	HTRA3
3064	1.0	1.0	1.0	-1.6	HTT

10075	2.7	1.0	1.0	6.2	HUWE1
84329	1.0	1.0	1.0	1.0	HVCN1
54768	1.0	1.0	1.0	1.0	HYDIN
3376	1.0	3.3	-2.9	3.2	IARS
55699	1.0	1.0	1.0	1.0	IARS2
3382	1.0	1.0	1.0	1.0	ICA1
3386	1.0	1.0	-6.3	-3.9	ICAM4
7087	1.0	3.1	1.0	2.7	ICAM5
23463	-15.4	1.0	1.0	1.0	ICMT
29851	1.0	1.0	-3.1	-3.0	ICOS
23308	6.5	1.0	1.7	1.0	ICOSLG
3399	1.0	-3.2	1.0	1.0	ID3
3400	2.1	1.0	3.4	1.0	ID4
3420	1.0	-2.5	1.0	1.0	IDH3B
3422	1.0	1.0	2.2	1.0	IDI1
91734	1.0	1.0	1.0	1.0	IDI2
3620	1.0	1.0	1.0	1.0	IDO1
3423	-2.3	1.0	4.8	1.0	IDS
3425	1.0	1.0	1.0	1.0	IDUA
9592	1.0	1.0	2.5	4.3	IER2
8870	1.0	1.0	1.0	-1.7	IER3
51278	1.0	1.0	1.0	1.0	IER5
25900	1.0	1.0	1.0	1.0	IFFO1
3428	1.0	-3.4	-6.8	-5.0	IFI16
122509	3.0	1.0	1.0	1.0	IFI27L1
83982	3.4	1.0	6.2	6.6	IFI27L2
10437	1.0	1.0	1.0	1.0	IFI30
10964	1.0	1.0	1.0	4.5	IFI44L
3433	1.0	1.0	1.0	1.0	IFIT2
8519	1.0	1.0	-4.1	1.0	IFITM1
10581	1.0	1.0	1.0	1.0	IFITM2
387733	1.0	5.5	1.0	1.0	IFITM5
3452	1.0	1.0	-3.0	1.0	IFNA21
3441	1.0	1.0	1.0	1.0	IFNA4
3443	1.0	1.0	1.0	-3.9	IFNA6
3454	1.6	1.0	-2.4	1.0	IFNAR1
3456	1.0	1.0	1.0	1.0	IFNB1
338376	1.0	-2.9	1.0	1.0	IFNE
3459	1.0	1.0	1.0	1.0	IFNGR1
3475	-2.4	-3.4	1.0	-3.2	IFRD1
55081	1.0	1.0	1.0	1.0	IFT57
80173	1.0	1.0	-3.1	-3.1	IFT74
57560	1.0	1.0	1.0	1.0	IFT80
3479	1.0	1.0	1.0	1.0	IGF1
3481	1.0	1.0	1.0	1.0	IGF2
10642	1.5	1.0	-5.5	1.0	IGF2BP1
10644	-2.2	1.0	-4.7	1.0	IGF2BP2
10643	1.0	1.0	1.0	-3.1	IGF2BP3

3482	1.0	1.0	-3.4	1.0	IGF2R
3485	2.1	1.0	2.2	2.1	IGFBP2
3486	1.0	1.0	1.0	1.0	IGFBP3
91156	1.0	1.0	1.0	1.0	IGFN1
3508	1.0	1.0	4.7	1.0	IGHMBP2
3512	1.0	1.0	-2.5	1.0	IGJ
3543	1.0	1.0	1.0	1.0	IGLL1
402665	-10.4	1.0	1.0	1.0	IGLON5
285313	1.0	1.0	1.0	-4.8	IGSF10
84966	1.0	1.0	1.0	1.0	IGSF21
3321	1.5	1.0	1.0	1.0	IGSF3
93185	1.0	1.0	1.0	1.0	IGSF8
57549	1.0	1.7	1.0	1.0	IGSF9
10320	1.0	1.0	1.0	1.0	IKZF1
22807	1.0	2.6	1.0	1.0	IKZF2
3586	1.0	1.0	1.0	1.0	IL10
3590	1.0	3.6	3.2	1.0	IL11RA
3600	1.0	1.0	1.8	1.6	IL15
3603	1.0	-6.9	-5.8	1.0	IL16
27189	1.8	1.0	1.0	1.0	IL17C
23765	1.0	1.0	1.0	1.0	IL17RA
55540	1.0	1.0	-2.3	1.0	IL17RB
132014	1.0	1.0	3.9	3.8	IL17RE
10068	1.7	3.1	1.0	1.0	IL18BP
8807	1.0	1.0	1.0	1.0	IL18RAP
84639	-2.0	1.0	1.0	1.0	IL1F10
3554	-1.7	1.0	1.0	1.0	IL1R1
7850	1.0	1.0	1.0	1.0	IL1R2
26280	1.0	1.8	1.9	1.0	IL1RAPL2
8808	1.0	1.0	1.0	1.0	IL1RL2
3558	1.0	-3.0	-3.3	1.0	IL2
50604	1.0	1.0	-5.2	1.0	IL20
9466	2.0	1.0	1.0	1.0	IL27RA
282617	1.0	1.0	1.0	1.0	IL28B
163702	1.0	1.0	1.0	1.0	IL28RA
386653	1.0	1.0	1.0	1.0	IL31
133396	1.0	1.0	1.0	1.0	IL31RA
90865	1.0	1.0	3.5	1.0	IL33
27177	1.0	1.0	1.0	2.7	IL36B
56300	1.0	2.9	3.8	2.7	IL36G
3565	1.0	1.0	1.0	-2.2	IL4
3566	1.0	1.0	1.0	1.0	IL4R
3567	1.0	1.0	-6.8	1.0	IL5
3569	1.0	1.0	1.0	-6.5	IL6
3572	3.0	-8.0	-9.3	-6.2	IL6ST
3574	1.0	-6.9	-7.1	-9.8	IL7
3581	1.0	2.6	2.6	2.5	IL9R
286676	1.0	1.0	1.0	1.0	ILDR1

3609	1.0	1.0	3.6	1.0	ILF3
92856	4.0	2.4	1.0	1.0	IMP4
162540	1.0	1.0	1.0	1.0	IMP5
3612	1.0	1.0	5.0	1.0	IMPA1
3613	1.0	1.8	3.6	3.6	IMPA2
3614	1.6	-5.2	1.0	1.0	IMPDH1
50939	1.0	1.0	1.0	1.0	IMPG2
10207	1.0	1.0	1.0	1.0	INADL
64423	1.7	3.9	1.0	1.0	INF2
3621	1.0	1.0	1.0	1.0	ING1
54556	1.0	1.0	2.5	1.8	ING3
3624	1.0	1.0	2.2	1.0	INHBA
83729	1.0	1.0	3.7	1.0	INHBE
11185	1.0	1.0	1.0	3.0	INMT
83444	1.0	1.0	2.8	1.0	INO80B
54891	1.0	-2.2	1.0	-2.2	INO80D
283899	1.0	1.0	1.0	-3.0	INO80E
3631	1.0	5.7	5.0	6.3	INPP4A
3632	1.0	1.0	1.0	1.0	INPP5A
3633	1.0	1.0	1.0	1.0	INPP5B
56623	1.0	1.0	1.0	4.0	INPP5E
22876	1.0	3.2	1.0	4.0	INPP5F
51141	1.6	1.0	1.0	-2.4	INSIG2
3643	1.0	1.0	1.0	1.0	INSR
3645	1.0	1.0	1.0	1.0	INSRR
26173	4.2	1.0	1.0	6.4	INTS1
55174	1.0	1.0	1.0	2.3	INTS10
57117	1.0	1.0	1.0	1.0	INTS12
26512	-14.8	1.0	1.0	1.0	INTS6
55656	1.0	1.0	1.0	1.0	INTS8
27152	1.0	1.0	1.0	3.1	INTU
27130	1.0	1.0	1.0	-4.2	INVS
26034	1.0	1.0	1.0	1.0	IPCEF1
3843	5.4	1.0	1.0	1.0	IPO5
10527	-1.5	-2.6	1.0	1.0	IPO7
64768	1.0	1.0	2.3	1.0	IPPK
3653	1.0	1.0	7.7	1.0	IPW
389124	1.0	1.0	-6.7	1.0	IQCF5
64799	1.0	-2.3	1.0	1.0	IQCH
8826	-1.7	1.0	1.0	1.0	IQGAP1
23096	-2.0	1.0	1.0	1.0	IQSEC2
134728	1.0	1.0	-5.2	-6.2	IRAK1BP1
3659	1.0	1.0	4.7	1.0	IRF1
3660	1.0	1.0	1.0	1.0	IRF2
3665	1.0	1.0	1.0	1.0	IRF7
10379	1.0	1.0	1.0	1.0	IRF9
8660	1.0	-5.0	1.0	1.0	IRS2
8471	1.0	1.0	1.0	-13.8	IRS4

79192	2.9	1.0	1.0	1.0	IRX1
50805	-2.9	1.0	1.0	1.0	IRX4
10265	1.0	1.0	1.0	1.0	IRX5
81689	1.0	3.4	5.0	1.0	ISCA1
81875	-3.5	1.0	-1.8	1.0	ISG20L2
3670	5.9	1.0	-2.4	-2.0	ISL1
140862	2.8	1.0	1.0	1.0	ISM1
145501	1.0	1.0	1.0	1.0	ISM2
91464	1.0	1.0	1.0	-5.8	ISX
51477	-1.7	1.0	-4.0	1.0	ISYNA1
81533	1.0	1.0	1.0	1.0	ITFG1
3676	1.0	1.0	2.6	1.0	ITGA4
3655	1.0	4.9	3.7	4.9	ITGA6
3679	2.4	1.0	1.0	1.0	ITGA7
3682	1.0	1.0	1.0	1.0	ITGAE
3684	1.0	3.2	3.6	2.7	ITGAM
3687	1.0	1.0	1.0	1.0	ITGAX
3688	-1.7	1.0	1.0	1.0	ITGB1
3689	1.0	1.0	-2.9	1.0	ITGB2
3691	-2.1	1.0	1.0	1.0	ITGB4
3694	1.0	1.0	-2.2	1.0	ITGB6
3700	1.0	1.0	-2.6	1.0	ITIH4
142683	1.0	2.1	1.0	1.0	ITLN2
9445	1.0	1.0	2.9	4.5	ITM2B
81618	1.0	1.0	1.0	1.0	ITM2C
3704	1.0	3.5	1.0	4.3	ITPA
3713	1.0	1.0	-4.0	1.0	IVL
55677	1.0	1.0	-4.3	1.0	IWS1
389434	1.0	1.0	1.0	1.5	IYD
284359	1.0	1.0	1.0	1.0	IZUMO1
3714	1.0	1.0	3.8	3.5	JAG2
3716	-1.8	-3.9	-3.6	1.0	JAK1
3717	1.0	1.0	1.0	-7.1	JAK2
58494	5.1	1.0	1.0	1.0	JAM2
80853	-3.6	3.1	2.8	1.0	JHDM1D
65094	-10.8	1.0	2.4	1.0	JMJD4
126119	-1.7	-3.8	1.0	1.0	JOSD2
57158	1.0	1.0	3.6	1.0	JPH2
57338	1.0	1.0	1.0	1.0	JPH3
8690	1.0	1.0	1.0	1.0	JRKL
10899	1.0	2.2	1.0	1.0	JTB
3725	-1.7	1.0	1.0	1.0	JUN
3727	-1.5	1.0	3.2	1.0	JUND
353219	-3.2	1.0	1.0	1.0	KAAG1
8997	1.0	-3.3	1.0	1.0	KALRN
256949	1.0	1.0	1.0	1.0	KANK3
163782	1.0	1.0	1.0	1.0	KANK4
54934	1.0	1.0	1.0	1.0	KANSL2

2648	1.0	1.0	1.0	1.0	KAT2A
10524	-2.6	1.0	1.0	-3.8	KAT5
7994	1.0	2.7	1.0	1.8	KAT6A
23522	-10.4	-2.8	-2.8	1.0	KAT6B
84056	1.0	5.5	7.7	3.7	KATNAL1
83473	1.0	1.0	1.0	1.0	KATNAL2
55709	1.0	1.0	1.0	1.0	KBTBD4
131377	1.0	1.0	1.0	2.2	KBTBD5
84078	1.0	1.0	1.0	1.0	KBTBD7
84541	-22.1	1.0	1.0	1.0	KBTBD8
56888	1.0	1.0	3.3	1.0	KCMF1
3744	-2.0	1.0	-3.5	1.0	KCNA10
3741	1.0	1.0	3.2	1.0	KCNA5
8514	1.0	1.0	1.0	1.0	KCNAB2
9196	1.0	-6.2	-7.2	-5.2	KCNAB3
3745	1.0	1.0	-6.0	1.0	KCNB1
23630	1.0	1.0	1.0	1.0	KCNE1L
23704	1.0	1.0	1.0	2.0	KCNE4
3754	1.0	1.0	1.0	1.0	KCNF1
3755	-1.5	1.0	1.0	2.4	KCNG1
170850	1.0	1.0	1.0	1.0	KCNG3
27133	-2.7	1.0	1.0	5.6	KCNH5
3769	1.0	1.0	1.0	1.0	KCNJ13
3772	-26.2	1.0	1.0	1.0	KCNJ15
3759	1.0	1.0	1.0	3.5	KCNJ2
3762	1.0	-5.2	1.0	-3.9	KCNJ5
3764	1.0	1.0	-1.5	1.0	KCNJ8
3765	1.0	1.0	1.0	1.0	KCNJ9
54207	1.0	1.0	1.0	1.0	KCNK10
83795	1.0	1.0	1.0	1.0	KCNK16
3777	1.0	-3.1	1.0	1.0	KCNK3
8645	1.0	1.0	1.0	1.0	KCNK5
51305	1.0	1.0	1.0	1.0	KCNK9
3778	-10.1	1.0	1.0	-2.8	KCNMA1
3783	1.0	1.0	1.0	2.4	KCNN4
3785	1.5	1.0	1.0	2.6	KCNQ2
3786	-2.3	1.0	1.0	-2.4	KCNQ3
56479	1.0	1.0	1.0	1.0	KCNQ5
3790	1.0	1.0	-3.8	-3.0	KCNS3
27012	1.0	1.0	1.0	-6.0	KCNV1
169522	1.0	3.3	1.0	1.0	KCNV2
147040	1.7	1.0	1.9	2.7	KCTD11
115207	1.0	1.0	1.0	1.0	KCTD12
253980	-2.4	1.0	1.0	1.0	KCTD13
79047	1.0	1.0	1.0	1.0	KCTD15
51133	1.0	1.0	5.6	3.4	KCTD3
54442	1.0	1.0	4.6	6.0	KCTD5
79070	1.0	1.0	1.0	1.0	KDELC1

10945	1.0	1.0	1.0	1.0	KDELR1
221656	1.6	-3.3	-4.9	-2.9	KDM1B
84678	1.0	1.0	1.0	1.0	KDM2B
9682	1.0	1.0	1.0	1.0	KDM4A
10765	2.9	1.0	6.6	5.1	KDM5B
7403	-1.7	1.0	1.0	2.7	KDM6A
2531	1.0	1.0	1.0	1.0	KDSR
387628	1.0	1.0	1.0	1.0	KGFLP1
80759	1.0	3.2	1.0	1.0	KHDC1
10656	1.0	4.6	1.0	1.0	KHDRBS3
3795	1.0	-6.9	1.0	1.0	KHK
23351	1.0	1.0	3.0	1.0	KHNYN
124602	1.6	3.2	1.0	1.0	KIF19
10112	1.0	2.7	2.7	2.4	KIF20A
23046	2.3	1.0	1.0	1.0	KIF21B
9493	1.0	1.0	1.0	1.0	KIF23
55083	1.0	1.0	1.0	1.0	KIF26B
3796	1.0	1.0	1.0	1.0	KIF2A
84643	1.0	-3.0	-3.7	-3.5	KIF2B
11004	1.0	1.0	1.0	3.0	KIF2C
3797	1.0	2.2	1.0	2.9	KIF3C
3798	1.0	1.0	2.8	1.0	KIF5A
221458	1.0	-3.3	1.0	1.0	KIF6
22920	1.0	5.4	4.9	1.0	KIFAP3
90990	1.0	4.0	1.0	1.0	KIFC2
3801	1.0	1.0	1.0	1.0	KIFC3
100132285	1.0	-3.4	1.0	1.0	KIR2DS2
3809	1.0	1.0	1.0	1.0	KIR2DS4
115653	1.0	2.5	1.0	1.0	KIR3DL3
55243	1.0	1.0	1.0	1.0	KIRREL
283165	1.0	2.8	3.1	1.0	KIRREL3-AS3
3815	1.0	1.0	1.0	1.0	KIT
4254	1.0	1.0	1.0	1.0	KITLG
152831	1.0	1.0	1.0	4.7	KLB
64837	1.0	-3.7	-1.7	1.0	KLC2
10661	1.0	2.6	2.5	1.0	KLF1
8462	1.0	-8.1	-6.4	-6.4	KLF11
28999	1.0	1.0	1.0	1.0	KLF15
11279	1.0	1.0	2.0	1.0	KLF8
113730	2.7	1.0	1.0	2.3	KLHDC7B
59349	1.0	1.0	1.0	-4.7	KLHL12
90293	1.0	5.3	1.0	5.1	KLHL13
9903	1.0	1.0	1.0	1.0	KLHL21
151230	1.0	1.0	-7.8	1.0	KLHL23
64410	1.0	1.0	1.0	1.0	KLHL25
283212	1.0	1.0	-3.3	1.0	KLHL35
79786	1.0	1.0	1.0	1.0	KLHL36
57563	1.0	1.0	1.0	1.0	KLHL8

55958	1.0	4.0	3.2	5.2	KLHL9
5655	1.0	1.0	1.0	6.4	KLK10
26085	1.0	1.0	1.0	1.0	KLK13
3817	1.0	1.0	1.0	1.0	KLK2
5653	1.0	1.0	1.0	1.0	KLK6
3821	1.0	1.0	3.7	1.0	KLRC1
8302	1.0	2.8	5.6	1.0	KLRC4
51348	1.0	1.0	1.0	1.0	KLRF1
10219	1.0	1.0	1.0	1.0	KLRG1
346689	1.0	1.0	1.0	1.0	KLRG2
8564	1.0	1.0	1.0	1.0	KMO
9735	1.0	2.2	1.0	2.6	KNTC1
3838	1.0	1.0	1.0	1.0	KPNA2
3837	-4.1	-4.0	1.0	1.0	KPNB1
83999	1.0	1.0	-2.4	1.0	KREMEN1
79412	1.0	1.0	1.0	-6.5	KREMEN2
65095	1.0	1.0	4.8	1.0	KRI1
889	1.0	1.0	1.0	1.0	KRIT1
11103	1.0	1.0	-3.9	1.0	KRR1
3859	1.0	1.0	1.0	1.0	KRT12
3860	1.0	1.0	6.7	1.0	KRT13
3872	3.0	1.0	1.0	6.5	KRT17
3880	1.6	6.2	5.7	3.6	KRT19
162605	3.0	1.0	1.0	1.7	KRT28
3882	1.0	1.0	1.0	1.0	KRT32
3883	1.0	1.0	1.0	1.0	KRT33A
3886	1.0	1.0	1.0	1.0	KRT35
8688	1.0	1.0	2.9	1.0	KRT37
125115	-1.9	1.0	1.0	1.0	KRT40
3852	1.0	1.0	1.0	1.0	KRT5
140807	1.0	1.7	2.9	1.0	KRT72
121391	1.0	1.0	1.0	1.0	KRT74
3889	1.0	1.0	1.0	2.0	KRT83
3891	-1.8	1.0	1.0	1.0	KRT85
353332	1.0	1.0	-4.2	1.0	KRTAP12-1
140258	1.0	1.0	1.0	2.8	KRTAP13-1
337882	-3.6	1.0	1.0	1.0	KRTAP19-1
85294	3.1	1.0	3.1	1.0	KRTAP2-4
83896	1.0	-1.8	-1.9	-1.8	KRTAP3-1
83897	1.0	2.3	1.9	1.0	KRTAP3-2
85285	-2.3	1.0	1.0	1.0	KRTAP4-1
84616	1.0	1.0	1.0	1.0	KRTAP4-4
81871	1.0	1.0	1.0	4.8	KRTAP4-6
100132476	1.8	1.0	1.0	1.0	KRTAP4-7
728224	1.0	1.0	1.0	1.0	KRTAP4-8
100132386	1.0	1.0	3.3	1.0	KRTAP4-9
83900	1.0	1.0	1.0	1.0	KRTAP9-3
83901	1.0	1.0	-4.2	1.0	KRTAP9-8

200185	1.0	1.0	2.4	2.4	KRTCAP2
283455	1.0	1.0	1.0	4.6	KSR2
8942	1.0	1.0	1.0	1.0	KYNU
79944	-1.7	1.0	1.0	1.0	L2HGDH
144811	1.8	1.0	1.0	1.0	LACC1
3902	-2.7	1.0	2.6	1.0	LAG3
3903	1.0	1.0	1.0	1.0	LAIR1
3904	1.0	1.0	1.0	-3.0	LAIR2
3906	1.0	2.7	3.1	1.0	LALBA
3910	1.0	1.0	1.0	1.5	LAMA4
3911	-3.3	1.0	1.0	1.0	LAMA5
3912	1.0	2.7	3.1	1.0	LAMB1
22798	1.0	1.0	-6.9	-6.9	LAMB4
3915	1.0	1.0	1.0	1.0	LAMC1
10319	2.6	1.0	1.0	1.0	LAMC3
3920	1.0	1.0	-5.8	1.0	LAMP2
27074	1.0	1.0	1.0	1.0	LAMP3
55353	1.0	4.7	3.4	1.0	LAPTM4B
7805	1.0	-4.0	1.0	1.0	LAPTM5
23367	1.0	1.0	1.0	1.0	LARP1
55132	1.0	1.0	1.0	1.0	LARP1B
23185	1.0	1.8	1.9	1.5	LARP4B
55323	1.0	1.0	4.8	4.5	LARP6
51574	1.0	1.0	1.0	1.0	LARP7
81887	1.0	-3.2	1.0	1.0	LAS1L
27040	1.0	2.6	1.0	3.7	LAT
7462	-2.2	1.0	1.0	1.0	LAT2
54900	1.0	1.0	-6.7	1.0	LAX1
81606	1.0	-2.6	-1.8	-2.6	LBH
10660	-3.6	-2.7	-2.7	1.0	LBX1
85474	2.7	1.0	1.0	1.0	LBX2
150082	1.0	1.0	1.0	1.0	LCA5L
353139	2.7	1.0	1.0	1.0	LCE2A
84648	3.4	1.0	2.7	1.0	LCE3D
353145	1.0	1.0	1.0	1.0	LCE3E
3933	1.0	1.0	2.4	1.0	LCN1
158062	1.0	1.0	1.0	1.0	LCN6
138307	-2.5	1.0	1.0	1.0	LCN8
254251	1.0	1.0	2.3	1.0	LCORL
3936	1.0	1.0	1.0	1.0	LCP1
3938	1.0	1.0	1.0	2.2	LCT
9079	1.0	-2.4	-4.5	-1.6	LDB2
11155	1.0	1.0	1.0	1.0	LDB3
3945	1.0	1.0	1.0	1.0	LDHB
3948	1.0	1.0	1.0	2.2	LDHC
197257	1.0	1.0	2.4	1.0	LDHD
3949	2.7	2.4	1.0	1.0	LDLR
23641	1.0	1.0	1.0	-3.1	LDOC1

116842	1.0	-2.6	1.0	1.0	LEAP2
11061	1.0	1.0	1.0	1.0	LECT1
3950	1.0	1.0	1.0	1.0	LECT2
7044	1.0	1.0	2.8	1.0	LEFTY2
221496	1.0	1.0	1.0	1.0	LEMD2
79165	-5.5	1.0	1.0	1.0	LENG1
94059	-2.9	1.0	-5.0	-4.8	LENG9
123169	1.0	1.0	-2.8	-2.7	LEO1
3952	1.0	1.0	1.0	1.0	LEP
3953	1.0	1.0	-8.5	1.0	LEPR
3954	2.4	1.0	1.0	1.0	LETM1
25875	1.0	2.8	2.8	3.5	LETMD1
3955	1.0	-5.7	1.0	1.0	LFNG
3957	1.0	-2.2	-2.4	1.0	LGALS2
3964	1.0	1.0	1.0	1.0	LGALS8
3965	1.0	3.0	1.0	1.0	LGALS9
55203	1.0	1.0	1.0	1.0	LGI2
163175	1.0	1.0	1.0	1.0	LGI4
5641	1.0	-4.0	1.0	1.0	LGMN
3973	1.0	1.0	1.0	1.0	LHCGR
3975	1.0	1.0	1.0	1.0	LHX1
9355	1.0	1.0	1.0	1.0	LHX2
56956	1.0	-2.8	1.0	1.0	LHX9
11019	1.0	1.0	1.0	1.0	LIAS
3977	-4.0	1.0	1.0	1.0	LIFR
3978	1.0	1.0	6.4	1.0	LIG1
3981	1.0	1.0	-3.1	1.0	LIG4
11026	1.0	1.0	1.0	1.7	LILRA3
23547	1.0	1.0	1.0	2.7	LILRA4
10859	1.6	1.0	1.0	1.0	LILRB1
10288	1.0	1.0	1.0	1.0	LILRB2
11006	1.0	-7.3	1.0	1.0	LILRB4
3982	1.0	1.0	1.0	1.0	LIM2
80774	2.2	1.0	1.0	1.0	LIMD2
3987	1.0	15.7	1.0	1.0	LIMS1
55679	1.0	1.0	1.0	1.0	LIMS2
100271835	1.0	1.0	1.0	1.0	LIMS3-LOC440895
79727	1.0	1.0	1.0	1.0	LIN28A
389421	1.0	1.0	1.0	1.0	LIN28B
286826	1.0	1.0	1.0	1.0	LIN9
145978	1.0	1.0	1.0	-6.6	LINC00052
147650	1.0	1.0	1.0	1.0	LINC00085
644596	1.0	1.0	1.0	4.2	LINC00087
79854	1.0	1.0	6.0	1.0	LINC00115
112597	1.0	1.0	-5.0	1.0	LINC00152
54072	1.0	-6.2	1.0	-6.0	LINC00158
440072	1.0	1.0	1.0	1.0	LINC00167
285908	1.0	7.5	3.6	6.9	LINC00174

387644	1.0	-3.6	1.0	1.0	LINC00202
83655	1.0	1.0	1.0	1.0	LINC00208
338005	1.0	1.0	1.0	1.0	LINC00221
145200	1.0	1.0	1.0	1.0	LINC00239
203429	1.0	1.0	-5.5	-4.6	LINC00246A
286333	1.0	1.0	1.0	1.0	LINC00256A
100128385	1.0	1.0	2.3	1.0	LINC00256B
140828	1.0	1.0	1.0	1.0	LINC00261
90271	1.0	-5.1	-8.0	-8.7	LINC00263
645528	1.0	1.0	1.0	1.0	LINC00264
140849	1.0	-2.6	-10.6	1.0	LINC00266-1
266919	1.0	-5.0	-7.5	-5.3	LINC00307
114036	1.0	1.0	1.0	-6.8	LINC00310
114038	1.0	1.0	1.0	1.0	LINC00313
387486	1.0	-1.9	-1.7	1.0	LINC00320
29092	1.0	1.0	1.0	2.3	LINC00339
401237	1.0	4.7	5.0	1.0	LINC00340
84791	1.0	1.0	1.0	-2.3	LINC00467
90632	1.0	1.0	1.0	1.0	LINC00473
158314	1.0	1.0	-2.4	1.0	LINC00475
100128782	1.0	1.0	1.0	1.0	LINC00476
388815	1.0	1.0	-4.4	1.0	LINC00478
84894	1.0	1.0	-16.0	1.0	LINGO1
158038	1.0	1.0	2.7	1.0	LINGO2
3990	1.0	1.0	1.0	1.0	LIPC
8513	1.0	1.0	1.0	1.0	LIPF
3996	1.0	1.0	1.0	-7.2	LLGL1
3993	1.0	1.0	1.0	1.0	LLGL2
79748	1.0	1.0	1.0	1.0	LMAN1L
81562	1.0	1.0	1.0	1.0	LMAN2L
29995	1.0	1.0	5.0	1.0	LMCD1
64788	-2.3	-6.3	1.0	1.0	LMF1
91289	1.0	1.0	1.0	1.0	LMF2
4000	1.0	-2.6	-2.9	-3.2	LMNA
4004	1.0	1.0	1.0	1.0	LMO1
8543	1.6	1.0	1.0	1.0	LMO4
56203	16.0	1.0	7.2	1.0	LMOD3
114783	1.0	1.0	-5.0	1.0	LMTK3
4012	1.0	1.0	2.7	3.2	LNPEP
222484	1.0	1.0	4.8	1.0	LNX2
503693	1.0	1.0	1.0	1.0	LOH12CR2
164832	1.0	1.0	1.0	1.0	LONRF2
79836	2.1	1.0	1.0	1.0	LONRF3
4015	1.0	1.0	1.0	1.0	LOX
125336	1.0	1.0	1.0	1.0	LOXHD1
4016	1.0	1.0	1.0	1.0	LOXL1
4017	1.0	1.0	2.0	1.0	LOXL2
4018	1.0	-3.1	1.0	1.0	LPA

1902	1.0	1.0	1.0	1.0	LPAR1
10161	-2.6	-6.8	1.0	1.0	LPAR6
23266	1.0	1.0	1.0	4.7	LPHN2
23284	1.0	1.0	-2.6	1.0	LPHN3
23175	1.0	1.0	1.0	1.0	LPIN1
9227	1.0	-1.8	1.0	1.0	LRAT
57622	1.0	-4.5	1.0	1.0	LRFN1
57497	-3.6	1.0	1.0	1.0	LRFN2
79414	1.6	1.0	1.0	1.0	LRFN3
136332	6.0	1.0	1.0	1.0	LRGUK
26018	1.0	2.6	2.6	1.0	LRIG1
121227	1.0	1.0	-6.3	-5.7	LRIG3
26103	1.0	1.0	1.0	1.0	LRIT1
340745	1.0	1.0	4.1	1.0	LRIT2
4033	2.5	1.0	1.0	1.0	LRMP
4035	1.0	2.7	2.7	2.6	LRP1
4036	1.0	1.0	1.0	1.0	LRP2
4037	1.0	-7.0	-5.0	1.0	LRP3
91355	1.0	1.0	1.0	1.0	LRP5L
4043	1.0	1.0	1.0	1.0	LRPAP1
55227	1.0	-5.8	4.4	1.0	LRRC1
390205	1.0	1.0	1.0	1.0	LRRC10B
90668	1.0	-3.0	1.0	-2.8	LRRC16B
10234	1.0	1.0	1.0	1.0	LRRC17
474354	2.2	1.0	1.0	-4.4	LRRC18
79442	1.0	1.0	-7.4	1.0	LRRC2
55222	-8.8	1.0	1.0	1.0	LRRC20
123355	-2.2	-4.8	-4.8	1.0	LRRC28
81543	1.0	1.0	1.0	1.0	LRRC3
79782	1.0	1.6	1.0	1.0	LRRC31
2615	1.8	1.0	1.0	1.0	LRRC32
151827	1.0	1.0	-4.9	1.0	LRRC34
55282	1.0	1.0	1.0	1.0	LRRC36
374819	1.0	1.0	1.0	1.0	LRRC37A3
55073	1.0	5.0	1.0	1.0	LRRC37A4
114659	1.0	1.0	-4.6	1.0	LRRC37B
147172	1.0	-3.1	1.0	1.0	LRRC37BP1
100505591	1.0	1.0	1.0	1.0	LRRC3C
115353	1.0	1.0	3.4	2.5	LRRC42
254050	1.0	-5.7	-5.6	1.0	LRRC43
201255	-3.0	-3.1	1.0	1.0	LRRC45
83450	-2.4	1.0	3.5	1.0	LRRC48
54839	1.0	1.0	3.2	1.0	LRRC49
219527	-9.3	1.0	-5.7	1.0	LRRC55
255252	1.0	1.0	1.0	1.0	LRRC57
55379	1.0	-2.5	1.0	1.0	LRRC59
23639	1.0	-3.8	-4.4	-4.3	LRRC6
149499	1.0	1.0	1.0	1.0	LRRC71

23507	2.1	1.5	1.0	1.0	LRRC8B
84230	1.0	1.0	1.0	2.0	LRRC8C
341883	1.0	-3.3	1.0	1.0	LRRC9
85444	1.0	1.0	-2.6	1.0	LRRCC1
9208	1.0	1.0	-6.1	1.0	LRRFIP1
127255	1.0	-3.0	1.0	1.0	LRRIQ3
347730	1.0	1.0	1.0	1.0	LRRTM1
90678	1.0	1.0	1.0	1.0	LRSAM1
57408	1.0	1.0	4.4	1.0	LRTM1
654429	1.0	1.0	1.0	1.0	LRTM2
220074	4.2	1.0	1.6	1.0	LRTOMT
4045	1.0	1.0	-3.3	1.0	LSAMP
149986	1.0	-4.1	-4.3	-7.7	LSM14B
57819	-2.1	1.0	1.0	1.0	LSM2
27258	-1.6	1.0	1.0	1.0	LSM3
51690	1.0	4.5	1.5	1.0	LSM7
4048	1.0	-2.6	-2.5	-2.5	LTA4H
4052	1.0	1.0	1.0	1.0	LTBP1
4053	-2.1	1.0	1.0	5.1	LTBP2
26046	1.0	1.0	1.0	1.0	LTN1
55692	1.0	-3.8	-3.5	-3.4	LUC7L
51747	1.0	1.0	1.0	1.0	LUC7L3
7798	1.0	1.0	1.0	1.0	LUZP1
8581	-3.3	1.0	1.0	1.0	LY6D
58496	1.0	1.0	1.0	1.0	LY6G5B
80740	1.0	1.0	-3.0	1.0	LY6G6C
54742	1.0	1.0	1.0	1.0	LY6K
9450	2.7	1.0	1.0	1.0	LY86
4063	1.0	1.0	1.0	1.0	LY9
129530	1.0	-2.3	1.0	1.0	LYG1
254773	1.0	1.0	1.0	1.0	LYG2
4066	1.0	1.0	1.0	1.0	LYL1
66004	1.0	1.0	1.0	1.0	LYNX1
137797	1.0	1.0	1.0	2.4	LYPD2
10434	1.0	1.8	2.0	1.0	LYPLA1
11313	1.0	1.0	1.0	1.0	LYPLA2
127018	1.0	1.0	1.0	1.0	LYPLAL1
57149	1.0	1.0	1.0	3.6	LYRM1
57128	1.0	2.8	1.0	1.0	LYRM4
90624	66.4	1.0	1.0	1.0	LYRM7
10894	1.0	1.0	1.0	1.0	LYVE1
84328	1.0	1.0	1.0	1.0	LZIC
11178	1.0	7.1	5.9	3.9	LZTS1
4074	1.0	1.0	1.0	1.0	M6PR
4081	1.9	1.0	1.0	1.0	MAB21L1
23499	-3.6	-5.0	-5.0	1.0	MACF1
140733	-6.4	1.0	1.0	1.0	MACROD2
8379	1.5	1.0	1.0	1.0	MAD1L1

10459	1.9	1.0	1.0	1.0	MAD2L2
8567	3.0	-2.9	1.0	1.0	MADD
84944	1.0	1.0	2.3	2.3	MAEL
389692	-3.0	1.0	1.0	1.0	MAFA
9935	1.0	-7.6	1.0	1.0	MAFB
23764	1.0	1.0	1.0	1.0	MAFF
727764	1.6	1.0	1.0	1.0	MAFIP
7975	1.0	1.0	1.0	1.0	MAFK
4110	-3.5	-2.3	-1.8	1.0	MAGEA11
4111	1.0	1.0	1.0	1.0	MAGEA12
266740	8.3	1.0	16.5	1.0	MAGEA2B
4107	1.0	1.0	2.9	1.0	MAGEA8
4112	1.0	1.0	-3.0	1.0	MAGEB1
9947	1.0	1.0	1.0	1.0	MAGEC1
51438	1.0	1.0	1.0	1.0	MAGEC2
9500	3.3	1.0	1.0	1.0	MAGED1
57692	3.5	1.0	1.0	1.0	MAGEE1
139599	1.0	1.0	1.0	1.0	MAGEE2
54551	1.0	1.0	5.4	7.9	MAGEL2
9863	1.0	1.0	1.0	1.0	MAGI2
260425	1.0	1.0	1.0	1.0	MAGI3
79917	1.0	1.0	1.0	1.0	MAGIX
84061	-1.5	-5.4	-5.5	-5.3	MAGT1
4117	1.0	1.0	4.8	1.0	MAK
4118	1.0	4.1	1.0	1.9	MAL
7851	1.0	-3.9	-3.8	1.0	MALL
10892	-13.3	1.0	1.0	1.0	MALT1
9794	1.0	1.0	1.0	1.0	MAML1
55534	1.0	1.0	-4.3	1.0	MAML3
10046	1.0	1.0	2.6	1.0	MAMLD1
284358	1.0	1.0	1.0	1.0	MAMSTR
10905	1.6	1.0	4.8	1.0	MAN1A2
11253	1.0	1.0	1.0	1.0	MAN1B1
57134	1.0	1.0	1.0	1.0	MAN1C1
4122	-4.3	1.0	1.0	1.0	MAN2A2
23324	1.0	-5.8	1.0	1.0	MAN2B2
4126	-14.1	1.0	1.0	1.0	MANBA
63905	1.0	1.0	1.0	1.0	MANBAL
149175	1.0	1.0	1.0	1.0	MANEAL
4129	1.0	4.6	4.3	1.0	MAOB
81631	1.0	1.0	1.0	1.0	MAP1LC3B
5605	7.6	1.0	1.0	1.0	MAP2K2
5608	1.0	1.0	-2.5	1.0	MAP2K6
4216	-3.5	1.0	1.0	1.0	MAP3K4
4217	1.0	1.0	1.0	1.0	MAP3K5
9064	2.8	1.0	5.5	1.0	MAP3K6
1326	1.0	1.0	1.0	1.0	MAP3K8
4293	1.5	1.0	-5.3	1.0	MAP3K9

4134	1.0	1.0	3.7	1.0	MAP4
8491	1.0	1.7	6.2	1.0	MAP4K3
79929	3.1	4.5	6.2	4.4	MAP6D1
256714	-2.4	1.0	1.0	1.0	MAP7D2
79649	1.0	4.4	-3.6	4.5	MAP7D3
5600	1.0	1.0	-5.5	1.0	MAPK11
225689	1.7	1.0	1.0	1.0	MAPK15
5595	1.0	1.0	-2.3	-2.2	MAPK3
5599	1.0	1.0	1.0	1.0	MAPK8
57787	1.0	1.0	1.0	-3.1	MARK4
4141	1.0	1.0	1.0	1.0	MARS
153562	1.0	1.0	1.0	1.0	MARVELD2
23139	1.0	1.0	1.0	1.0	MAST2
84930	1.0	1.0	1.0	1.0	MASTL
4143	1.0	2.1	1.0	1.0	MAT1A
4144	1.0	1.0	1.0	1.0	MAT2A
27430	1.0	-5.7	1.0	1.0	MAT2B
4146	1.7	1.0	1.0	1.0	MATN1
4148	1.0	-3.8	-6.3	1.0	MATN3
8785	1.0	3.3	1.0	1.0	MATN4
23383	1.0	1.0	1.0	5.1	MAU2
4149	1.0	3.0	3.0	1.0	MAX
4152	1.0	2.5	1.0	-1.7	MBD1
8932	1.0	1.0	1.0	1.0	MBD2
85509	1.0	1.0	1.0	1.0	MBD3L1
55777	20.0	19.6	1.0	1.0	MBD5
114785	1.6	1.0	1.0	1.0	MBD6
51562	1.0	1.0	1.5	1.0	MBIP
4154	1.0	-2.7	1.0	1.0	MBNL1
10150	1.0	1.0	1.0	1.0	MBNL2
129642	1.0	1.0	1.0	1.0	MBOAT2
79143	2.1	1.0	1.0	1.0	MBOAT7
4155	1.0	1.0	-4.3	1.0	MBP
8720	1.0	1.0	-5.2	1.0	MBTPS1
4157	-3.4	-3.0	1.0	1.0	MC1R
4159	1.0	1.0	1.0	1.0	MC3R
4162	6.2	1.0	1.0	1.0	MCAM
64087	1.0	1.0	1.0	1.0	MCCC2
4168	1.0	1.0	1.0	1.0	MCF2
23263	1.0	-3.2	-3.2	1.0	MCF2L
100289410	1.0	1.0	1.0	1.0	MCF2L-AS1
90411	1.0	1.6	1.0	1.0	MCFD2
2847	1.0	1.0	4.3	4.6	MCHR1
84539	-1.6	1.0	-3.8	1.0	MCHR2
4170	1.0	1.0	-6.0	1.0	MCL1
4171	1.0	1.0	3.2	6.6	MCM2
114044	-1.7	1.0	1.0	4.3	MCM3AP-AS1
4174	1.0	1.0	1.0	1.0	MCM5

4175	1.0	1.0	1.0	1.0	MCM6
55283	1.0	1.0	-3.1	1.0	MCOLN3
79772	1.0	1.0	1.0	-6.3	MCTP1
55784	1.0	1.0	1.0	1.0	MCTP2
28985	1.0	1.0	-5.6	1.0	MCTS1
4190	1.0	1.0	1.0	1.0	MDH1
130752	1.0	3.7	5.0	1.0	MDH1B
4193	1.0	1.0	1.0	2.0	MDM2
4194	1.0	1.0	1.0	1.0	MDM4
259283	1.0	1.0	1.0	1.0	MDS2
4200	1.0	1.0	-3.7	1.0	ME2
10873	1.0	1.0	-2.7	1.0	ME3
64769	1.0	-3.0	1.0	1.0	MEAF6
2122	-4.6	1.0	1.0	1.0	MECOM
4204	1.0	1.0	1.0	1.0	MECP2
400569	1.0	1.0	1.0	1.0	MED11
9968	1.0	1.0	1.0	-3.8	MED12
116931	1.0	-6.2	-5.9	1.0	MED12L
9969	-2.1	1.0	1.0	1.0	MED13
23389	1.0	1.0	1.0	1.0	MED13L
9440	1.0	1.0	-9.9	1.0	MED17
219541	1.0	1.0	1.0	1.0	MED19
9439	1.0	1.0	1.0	1.0	MED23
81857	-2.8	1.0	-2.3	1.0	MED25
55588	1.0	1.0	1.0	1.0	MED29
90390	1.0	1.0	-3.4	-2.8	MED30
29079	1.0	1.0	3.0	1.0	MED4
10001	1.0	1.0	1.0	1.0	MED6
9443	1.0	1.0	-6.6	1.0	MED7
112950	1.0	1.0	1.0	1.0	MED8
4205	1.8	1.0	1.0	1.0	MEF2A
729991	1.7	1.0	3.3	1.0	MEF2BNB
55384	1.0	1.0	1.0	-1.9	MEG3
84465	1.0	1.0	1.0	1.0	MEGF11
4211	-2.8	-6.7	1.0	-7.1	MEIS1
51072	1.0	1.0	2.4	1.0	MEMO1
56257	1.0	1.0	-5.0	1.0	MEPCE
56955	-1.9	-2.2	1.0	1.0	MEPE
10461	1.0	1.0	-6.8	1.0	MERTK
4233	1.0	1.0	1.0	1.0	MET
284207	1.0	1.0	-4.3	1.0	METRNL
4234	1.0	1.0	-1.7	1.0	METTL1
399818	1.0	1.0	1.0	-2.8	METTL10
51603	1.0	5.0	4.7	3.2	METTL13
57721	1.0	3.1	3.4	1.0	METTL14
196074	1.0	-4.5	1.0	1.0	METTL15
64745	1.0	1.0	1.0	4.7	METTL17
254013	1.0	1.0	2.2	1.0	METTL20

151194	1.0	-3.5	1.0	1.0	METTL21A
196541	1.0	1.0	-1.6	1.0	METTL21C
124512	1.0	1.0	4.6	1.0	METTL23
339175	1.0	1.0	1.0	1.0	METTL2A
29081	3.6	1.0	1.0	1.0	METTL5
131965	1.0	1.0	1.0	1.0	METTL6
196410	1.0	2.8	1.0	1.0	METTL7B
79828	1.0	1.0	4.4	1.0	METTL8
51108	1.5	-5.2	-3.5	-6.0	METTL9
51320	1.0	-5.1	1.0	1.0	MEX3C
4238	1.0	2.2	1.0	1.0	MFAP3
9848	1.0	1.0	4.5	1.0	MFAP3L
9258	1.0	1.0	1.0	1.0	MFHAS1
4242	1.0	-1.9	-1.8	-1.8	MFNG
64747	1.0	2.8	1.9	1.0	MFSD1
113655	1.0	1.0	1.0	-3.3	MFSD3
23269	1.0	1.0	-3.6	-3.3	MGA
8972	1.0	1.0	1.0	1.0	MGAM
4245	1.0	1.0	-3.3	1.0	MGAT1
25834	1.0	1.0	1.0	1.0	MGAT4C
4249	1.0	1.0	1.0	1.0	MGAT5
84757	1.0	1.0	1.0	1.0	MGC10814
84793	1.0	1.0	1.0	2.4	MGC12982
113691	1.0	-2.9	-2.6	1.0	MGC16703
389741	1.0	2.3	1.0	1.0	MGC21881
196872	1.0	1.0	1.0	1.0	MGC23270
197187	1.0	1.0	1.0	1.0	MGC23284
158295	1.0	1.0	1.0	1.0	MGC24103
157247	1.0	1.0	1.0	1.0	MGC27345
154089	1.0	1.0	1.0	1.0	MGC34034
414927	1.0	1.0	1.0	1.0	MGC34796
403312	1.0	1.0	4.3	1.0	MGC39545
79100	1.0	1.0	1.0	-3.2	MGC4473
79150	1.0	6.4	6.2	7.7	MGC4859
399693	1.0	1.0	1.0	1.0	MGC50722
401884	1.0	-1.6	1.0	1.0	MGC57346
11343	1.0	1.0	1.0	5.2	MGLL
23295	-4.5	1.0	1.0	1.0	MGRN1
4259	1.0	-2.7	1.0	1.0	MGST3
8190	1.0	1.0	1.0	1.0	MIA
117153	1.0	1.0	1.0	1.0	MIA2
375056	1.0	1.0	1.0	1.0	MIA3
57534	1.0	1.0	1.0	1.0	MIB1
142678	2.7	1.0	1.0	1.0	MIB2
64780	1.0	-6.0	1.0	1.0	MICAL1
84953	1.0	1.0	1.0	1.0	MICALCL
85377	1.0	1.0	1.0	2.7	MICALL1
4277	36.6	1.0	1.0	1.0	MICB

10367	1.0	1.0	-6.4	1.0	MICU1
58526	3.0	1.0	1.0	1.0	MID1IP1
11043	1.0	1.0	1.0	1.0	MID2
90007	1.0	3.2	1.0	1.0	MIDN
57708	-2.4	-4.4	-4.8	-4.8	MIER1
166968	1.0	1.0	1.0	1.0	MIER3
4282	1.0	1.0	1.0	1.0	MIF
84864	1.0	1.0	1.0	1.0	MINA
9562	1.6	1.0	3.5	3.6	MINPP1
55586	1.9	1.0	1.0	1.0	MIOX
145282	1.0	1.0	-3.1	1.0	MIPOL1
399959	1.0	1.0	1.0	2.7	MIR100HG
114614	1.0	1.0	1.0	1.0	MIR155HG
407975	1.0	1.0	1.0	1.0	MIR17HG
54069	1.0	1.0	1.0	1.0	MIS18A
4286	-4.0	1.0	-2.8	3.3	MITF
83881	1.0	1.0	2.3	1.0	MIXL1
57496	1.0	1.0	1.0	1.0	MKL2
4289	1.0	-7.6	-7.2	-8.4	MKLN1
8569	1.0	3.8	1.0	3.4	MKNK1
23608	1.8	1.0	1.0	1.0	MKRN1
400058	1.0	1.0	1.0	1.0	MKRN9P
54903	1.0	1.0	1.0	1.0	MKS1
283078	3.2	1.0	1.0	1.0	MKX
2315	1.0	1.0	6.4	1.0	MLANA
8079	1.0	6.7	4.7	1.0	MLF2
4292	1.0	1.0	1.0	1.0	MLH1
27030	1.0	1.0	1.0	4.7	MLH3
197259	1.0	1.0	1.0	1.0	MLKL
4297	1.0	1.0	1.0	1.0	MLL
55904	1.0	1.0	1.0	1.0	MLL5
8028	-1.8	1.0	1.0	1.0	MLLT10
4301	1.0	1.0	1.0	-3.7	MLLT4
4302	-2.1	1.0	2.5	1.0	MLLT6
6945	1.0	1.0	1.0	1.0	MLX
326625	1.0	1.0	1.0	1.0	MMAB
23531	1.6	-2.9	1.0	1.0	MMD
221938	1.0	1.0	1.0	1.0	MMD2
4312	1.0	1.0	-2.4	1.0	MMP1
4322	1.0	1.0	-4.3	1.0	MMP13
4323	1.0	1.0	3.5	1.0	MMP14
4318	1.0	1.0	1.0	1.0	MMP9
79812	1.0	1.0	1.5	1.0	MMRN2
55329	1.0	1.0	1.0	1.0	MNS1
4335	1.0	1.0	1.0	1.0	MNT
55233	1.0	4.3	4.8	4.0	MOB1A
92597	-1.6	-2.8	-3.4	-3.3	MOB1B
4336	1.6	4.1	3.0	3.5	MOBP

55034	1.0	-3.0	1.0	1.0	MOCOS
4337	1.0	1.0	1.0	1.0	MOCS1
4340	1.0	1.0	4.6	1.0	MOG
80168	1.0	1.0	-2.0	1.0	MOGAT2
7841	1.8	1.0	1.0	1.0	MOGS
5891	1.0	-2.7	2.8	1.0	MOK
22879	-4.3	1.0	1.0	2.9	MON1B
23041	1.0	1.0	1.0	1.0	MON2
79710	2.4	1.0	-3.2	1.0	MORC4
10933	-1.9	1.0	1.0	1.0	MORF4L1
9643	1.0	1.0	2.4	2.4	MORF4L2
283385	1.0	2.7	1.0	2.7	MORN3
4343	1.0	1.0	2.2	1.0	MOV10
9526	1.0	3.4	4.8	5.8	MPDU1
54737	1.0	7.1	3.3	3.1	MPHOSPH8
4352	1.0	1.0	1.0	1.0	MPL
4353	1.0	1.0	1.0	1.0	MPO
4354	1.0	1.0	1.0	1.0	MPP1
4355	1.0	1.0	1.0	1.0	MPP2
4356	1.0	5.4	1.0	1.0	MPP3
64398	1.0	1.0	1.0	2.5	MPP5
65258	1.0	1.0	-2.9	1.0	MPPE1
23164	1.0	1.0	1.0	2.3	MPRIP
4357	1.0	1.0	1.0	1.0	MPST
4358	1.0	1.0	1.0	1.0	MPV17
255027	1.0	1.0	2.4	1.0	MPV17L
4359	-8.9	-3.8	-10.5	-4.9	MPZ
9019	-1.8	1.0	1.0	1.0	MPZL1
196264	1.0	1.0	1.0	-3.1	MPZL3
4360	1.0	6.1	1.0	5.9	MRC1
4361	1.0	1.0	4.6	1.0	MRE11A
116535	1.0	1.0	1.0	1.0	MRGPRF
259249	1.0	2.6	1.0	1.0	MRGPRX1
117195	1.0	2.7	1.0	1.0	MRGPRX3
117196	1.0	1.0	1.0	1.0	MRGPRX4
79922	-2.8	3.1	1.0	4.1	MRM1
78988	1.0	1.0	1.0	1.0	MRP63
124995	1.0	3.1	3.7	2.5	MRPL10
6182	2.6	-2.9	-2.8	1.0	MRPL12
54948	4.3	1.0	2.6	1.0	MRPL16
63875	1.0	-2.7	1.0	1.0	MRPL17
9801	-48.0	1.0	1.0	1.0	MRPL19
219927	1.0	-3.6	-3.5	-3.4	MRPL21
6150	1.0	1.0	1.0	1.0	MRPL23
79590	1.0	1.0	-1.7	1.0	MRPL24
51264	1.0	1.0	1.0	2.0	MRPL27
64983	1.0	-2.1	-2.2	1.0	MRPL32
9553	1.0	1.0	1.0	1.0	MRPL33

64981	1.0	2.6	1.0	1.0	MRPL34
51073	1.0	3.0	2.6	1.0	MRPL4
64976	1.0	1.0	1.0	1.0	MRPL40
28977	-1.5	1.0	1.0	1.0	MRPL42
84545	1.0	1.0	1.0	2.2	MRPL43
26589	1.0	1.0	1.0	2.2	MRPL46
122704	1.0	-2.6	1.0	1.0	MRPL52
116540	1.9	1.0	1.0	1.0	MRPL53
65005	1.0	3.0	1.0	1.0	MRPL9
64963	3.6	1.0	1.0	1.0	MRPS11
6183	-1.6	1.0	1.0	1.0	MRPS12
51021	1.0	2.5	2.5	6.3	MRPS16
28973	1.0	2.3	1.0	1.0	MRPS18B
56945	2.4	1.0	-4.4	-4.2	MRPS22
64951	1.0	1.0	1.0	1.0	MRPS24
64432	1.0	1.0	-6.0	1.0	MRPS25
23107	1.0	4.7	4.1	1.0	MRPS27
28957	1.0	-4.5	1.0	1.0	MRPS28
10884	1.0	1.0	-6.5	1.0	MRPS30
10240	1.9	1.0	1.0	1.0	MRPS31
65993	1.0	1.0	1.0	1.0	MRPS34
92259	1.0	-4.3	-4.6	-6.2	MRPS36
64969	1.0	1.0	3.2	1.0	MRPS5
51081	1.0	1.0	-3.7	1.0	MRPS7
92399	1.0	1.0	1.0	1.0	MRRF
10335	1.0	1.0	1.0	-4.0	MRVI1
931	1.0	1.0	1.0	1.0	MS4A1
54860	1.0	5.6	3.4	1.0	MS4A12
84689	1.0	1.0	1.0	1.0	MS4A14
2206	-3.1	1.0	-5.2	1.0	MS4A2
51338	1.0	-3.6	-3.4	-4.1	MS4A4A
64231	1.0	1.0	4.4	1.0	MS4A6A
58475	1.0	1.0	1.0	1.0	MS4A7
4436	1.0	1.0	-5.6	1.0	MSH2
4437	1.9	1.0	-3.0	-2.6	MSH3
124540	1.0	2.6	5.0	3.0	MSI2
339287	1.0	2.4	1.0	1.0	MSL1
10232	1.0	1.0	1.0	1.0	MSLN
6307	1.0	1.0	1.0	-2.3	MSMO1
4478	1.0	1.0	3.1	1.0	MSN
55154	1.0	-9.7	-4.8	-8.1	MSTO1
4487	1.0	1.0	1.0	-2.4	MSX1
4488	1.0	1.0	1.0	1.0	MSX2
4493	1.9	-1.6	1.0	1.0	MT1E
4502	1.0	1.0	1.0	1.0	MT2A
84560	1.0	1.0	-8.0	1.0	MT4
9219	1.0	1.0	1.0	1.0	MTA2
57504	1.0	1.0	1.0	1.0	MTA3

4507	1.0	-2.6	1.0	1.0	MTAP
23788	1.0	-3.0	1.0	1.0	MTCH2
4515	1.5	1.0	1.0	1.0	MTCP1
92140	1.0	1.0	1.0	1.0	MTDH
130916	1.0	1.0	1.0	1.0	MTERFD2
92170	1.0	1.0	1.0	5.1	MTG1
4522	1.0	-3.2	-3.2	-3.2	MTHFD1
25902	1.0	1.0	2.5	1.0	MTHFD1L
441024	1.0	1.0	1.0	1.0	MTHFD2L
54893	1.0	2.4	2.3	1.0	MTMR10
64419	1.0	-3.4	-2.8	1.0	MTMR14
8898	1.0	1.0	1.0	1.0	MTMR2
9107	5.1	-4.2	1.0	1.0	MTMR6
9108	1.0	1.0	1.0	1.0	MTMR7
2475	1.0	1.0	1.0	1.0	MTOR
10651	2.0	1.0	1.0	1.0	MTX2
4582	-3.9	1.0	1.0	1.0	MUC1
10071	-1.6	1.0	-3.2	1.0	MUC12
94025	1.0	1.0	1.0	1.0	MUC16
140453	1.0	-2.8	-3.0	-3.0	MUC17
100133790	1.0	1.0	4.6	1.0	MUC3
4584	1.0	1.6	-3.9	2.8	MUC3A
4585	2.0	1.0	1.0	1.0	MUC4
727897	1.0	1.0	1.0	1.0	MUC5B
4588	1.0	1.0	-3.2	1.0	MUC6
4589	1.0	1.0	1.0	-2.1	MUC7
118430	1.0	1.0	1.0	1.0	MUCL1
55745	1.0	1.0	-6.5	1.0	MUDENG
4597	-5.0	1.0	1.0	1.0	MVD
9961	1.7	1.0	1.0	1.0	MVP
4599	1.0	1.0	1.0	1.0	MX1
4084	1.0	1.0	1.0	1.0	MXD1
83463	2.4	1.0	-4.3	1.0	MXD3
10608	-1.6	1.0	1.0	-2.6	MXD4
4601	1.0	14.7	5.1	-4.5	MXI1
439921	1.7	1.0	1.0	1.0	MXRA7
91663	1.0	2.6	1.0	1.0	MYADM
4602	1.0	1.0	1.0	1.0	MYB
4604	1.0	1.0	-6.2	1.0	MYBPC1
4607	1.0	1.0	1.0	1.0	MYBPC3
4609	-4.1	-2.6	1.0	1.0	MYC
23077	1.0	-3.6	-3.3	1.0	MYCBP2
84073	1.0	1.0	1.0	1.0	MYCBPAP
4610	1.0	2.8	1.0	1.0	MYCL1
10408	1.0	1.0	-2.9	1.0	MYCNOS
4615	1.0	1.0	1.7	1.0	MYD88
4618	1.0	1.0	5.1	1.0	MYF6
4628	-5.7	-6.8	1.0	1.0	MYH10

79784	-2.5	1.0	-2.0	1.0	MYH14
4622	1.0	1.0	-3.6	1.0	MYH4
57644	1.0	1.0	-3.8	1.0	MYH7B
4627	1.0	1.0	1.0	1.0	MYH9
4635	1.0	-2.7	-2.8	1.0	MYL4
140465	3.1	3.8	1.0	1.0	MYL6B
58498	1.0	1.0	1.0	1.0	MYL7
10398	1.0	1.0	1.0	1.0	MYL9
29116	1.0	-2.7	1.0	1.0	MYLIP
4638	1.0	1.0	-5.5	1.0	MYLK
91807	-1.6	1.0	5.7	1.0	MYLK3
55892	1.0	1.0	-3.2	-3.2	MYNN
4651	1.0	1.0	1.0	3.3	MYO10
399687	1.0	1.0	1.0	1.0	MYO18A
4640	-4.4	1.0	3.4	4.3	MYO1A
4430	-8.0	1.0	-2.8	1.0	MYO1B
4641	-2.1	-2.5	1.0	1.0	MYO1C
4542	1.0	1.0	1.0	1.0	MYO1F
53904	1.0	2.3	2.2	1.0	MYO3A
4644	1.0	1.0	4.6	1.0	MYO5A
4645	1.0	1.0	1.0	1.0	MYO5B
55930	1.0	1.0	4.1	1.0	MYO5C
4647	1.0	1.0	1.0	1.0	MYO7A
4648	1.0	1.0	1.0	1.0	MYO7B
4650	1.0	2.7	2.6	1.0	MYO9B
4654	-1.8	1.0	1.0	1.0	MYOD1
26509	1.0	1.0	1.0	-4.5	MYOF
8736	-1.5	1.0	1.0	1.0	MYOM1
84665	1.0	-1.9	-3.5	-1.9	MYPN
339344	1.7	1.0	-6.3	1.0	MYPOP
25924	1.0	1.0	1.0	1.0	MYRIP
4661	1.0	1.0	1.0	1.0	MYT1
100820829	1.0	1.0	1.0	1.0	MYZAP
51237	1.0	1.0	1.0	1.0	MZB1
80097	1.5	1.0	1.0	1.0	MZT2B
9683	1.0	1.0	-3.2	1.0	N4BP1
90634	1.0	1.0	1.0	1.0	N4BP2L1
29104	-3.3	1.0	1.0	1.0	N6AMT1
8260	1.0	-2.9	1.0	1.0	NAA10
51126	1.0	1.0	1.0	1.0	NAA20
122830	1.0	4.5	5.0	4.7	NAA30
79829	-3.3	1.0	-4.2	1.0	NAA40
80218	1.0	1.0	1.0	-2.6	NAA50
10003	1.0	1.0	-5.1	1.0	NAALAD2
4665	1.5	1.0	1.0	1.0	NAB2
23148	1.0	1.0	1.0	1.0	NACAD
138151	1.0	1.0	-2.8	1.0	NACC2
4668	1.0	1.0	1.0	1.0	NAGA

55577	1.0	-3.3	-2.9	-2.8	NAGK
51172	6.4	1.0	1.0	1.0	NAGPA
259232	1.0	1.0	1.0	1.0	NALCN
10135	1.0	1.0	1.0	1.0	NAMPT
342977	1.0	1.0	1.7	1.0	NANOS3
4673	1.0	1.0	3.2	1.0	NAP1L1
63908	1.0	1.0	1.0	-4.1	NAPB
222236	8.0	1.0	1.0	1.0	NAPEPLD
8774	1.0	1.0	1.0	1.0	NAPG
9476	1.0	1.0	1.0	1.0	NAPSA
4678	1.0	1.0	1.0	2.0	NASP
24142	1.0	1.0	-3.8	1.0	NAT6
9027	1.0	1.0	5.5	1.0	NAT8
51471	1.0	1.0	1.0	1.0	NAT8B
89796	1.0	1.0	-6.2	1.0	NAV1
89797	-1.6	1.0	1.8	1.0	NAV2
89795	1.0	1.0	1.0	2.8	NAV3
65065	1.0	1.0	1.0	1.0	NBEAL1
23218	1.0	1.0	1.0	1.0	NBEAL2
100132406	1.0	1.0	1.0	4.4	NBPF10
25832	1.0	3.2	8.1	1.0	NBPF14
4077	1.0	1.0	1.0	3.4	NBR1
10230	1.0	1.0	-3.3	1.0	NBR2
4685	1.0	1.0	1.0	1.0	NCAM2
1463	1.0	1.9	1.0	1.0	NCAN
9918	1.0	1.0	1.0	1.0	NCAPD2
23397	1.0	1.0	1.0	1.0	NCAPH
29781	5.0	1.0	-3.0	1.0	NCAPH2
653361	1.7	1.0	1.0	1.0	NCF1
4690	1.0	1.0	1.0	1.0	NCK1
8440	1.0	-4.9	-5.4	1.0	NCK2
3071	1.0	-4.2	1.0	1.0	NCKAP1L
57701	1.0	1.0	1.0	1.0	NCKAP5L
56926	1.0	3.8	1.0	1.8	NCLN
8648	1.0	1.0	-7.5	1.0	NCOA1
10499	1.0	-4.4	1.0	1.0	NCOA2
8202	1.0	1.0	3.0	1.0	NCOA3
57727	1.0	1.0	2.8	1.0	NCOA5
23054	1.0	1.0	1.0	1.0	NCOA6
9437	1.0	1.0	1.0	1.0	NCR1
9436	1.0	1.0	1.0	-3.5	NCR2
23413	1.0	1.0	1.0	1.0	NCS1
10403	1.0	1.0	1.0	1.0	NDC80
81565	1.0	1.0	1.0	1.0	NDEL1
27158	2.0	-5.6	1.0	1.0	NDOR1
10397	2.0	5.8	1.0	1.0	NDRG1
57447	2.8	3.4	1.0	1.0	NDRG2
8509	1.0	3.3	5.1	1.0	NDST2

64579	1.0	2.4	1.0	1.0	NDST4
126328	1.0	1.0	1.0	1.0	NDUFA11
4695	1.0	1.0	-2.7	1.0	NDUFA2
51103	1.0	1.0	1.0	1.0	NDUFAF1
4707	1.0	1.0	1.0	1.0	NDUFB1
4718	1.0	1.0	2.7	1.0	NDUFC2
4724	1.7	1.0	1.0	1.0	NDUFS4
4726	1.0	1.0	-2.8	1.0	NDUFS6
374291	1.0	1.0	1.0	-2.6	NDUFS7
4703	1.0	1.0	-1.8	1.0	NEB
25977	1.6	2.6	2.1	1.0	NECAP1
121441	1.0	1.0	3.1	1.0	NEDD1
4734	1.0	1.0	1.0	1.0	NEDD4
4744	1.0	-4.5	-5.9	-4.0	NEFH
4750	-4.3	1.0	1.0	1.0	NEK1
6787	1.0	1.0	1.0	1.0	NEK4
140609	1.0	3.3	1.0	3.9	NEK7
26012	1.0	2.3	1.0	1.0	NELF
4753	1.0	-2.4	1.0	-2.4	NELL2
4756	1.0	2.8	2.3	1.0	NEO1
81832	-56.0	1.0	1.0	1.0	NETO1
81831	1.0	3.1	3.1	2.9	NETO2
4759	1.0	1.0	6.1	1.0	NEU2
10825	1.0	1.0	1.0	1.0	NEU3
54492	1.0	3.8	1.0	4.7	NEURL1B
93082	1.0	1.0	1.0	1.0	NEURL3
4763	1.7	-7.3	1.0	1.0	NF1
4771	1.0	-6.8	1.0	1.0	NF2
23114	-5.3	1.0	4.9	1.0	NFASC
4772	1.0	1.6	1.0	1.0	NFATC1
4773	3.1	12.5	1.0	1.0	NFATC2
4776	3.9	1.0	-3.8	-2.6	NFATC4
4780	1.0	1.0	1.0	-3.2	NFE2L2
58160	1.0	1.0	-2.7	-2.8	NFE4
4781	1.0	-4.7	-6.9	-3.7	NFIB
4792	1.0	1.0	1.0	1.0	NFKBIA
27247	1.0	1.0	1.0	1.0	NFU1
4800	1.0	1.0	-3.7	1.0	NFYA
25791	1.0	3.4	3.2	3.1	NGEF
27018	1.0	1.0	1.0	1.0	NGFRAP1
55768	1.0	1.0	1.0	1.0	NGLY1
4808	1.0	1.0	1.0	1.0	NHLH2
374354	3.1	1.0	-3.0	1.0	NHLRC2
283948	1.0	1.0	1.0	1.0	NHLRC4
57224	1.0	1.0	-2.8	1.0	NHSL1
84276	1.0	1.0	1.0	1.0	NICN1
4811	1.0	1.0	1.0	1.0	NID1
22795	1.7	1.0	1.0	3.8	NID2

60491	1.0	1.0	1.0	4.7	NIF3L1
167359	1.0	1.0	1.0	1.0	NIM1
22981	1.0	1.0	1.0	1.0	NINL
8508	1.0	1.0	1.0	1.0	NIPSNAP1
55335	1.0	1.0	-4.0	1.0	NIPSNAP3B
11188	1.0	1.0	2.4	2.4	NISCH
4817	1.0	1.0	1.0	1.0	NIT1
79570	-1.8	1.0	1.0	1.0	NKAIN1
154215	1.0	1.0	1.0	1.0	NKAIN2
286183	1.0	1.0	1.0	1.0	NKAIN3
158801	1.0	1.0	1.0	4.8	NKAPP1
85409	1.0	4.5	3.9	1.0	NKD2
7080	1.0	1.0	2.6	1.0	NKX2-1
26257	4.6	1.0	1.0	1.0	NKX2-8
4824	1.0	1.0	1.0	1.0	NKX3-1
157848	1.0	1.0	1.0	1.0	NKX6-3
54475	1.0	-3.2	1.0	1.0	NLE1
57502	1.0	1.0	1.0	1.0	NLGN4X
197358	1.0	1.0	1.0	1.0	NLRC3
58484	1.0	1.0	2.9	1.0	NLRC4
84166	1.0	5.1	1.0	1.0	NLRC5
22861	1.0	1.0	3.2	1.0	NLRP1
338323	1.0	1.0	1.0	1.0	NLRP14
55655	1.0	1.0	1.0	1.0	NLRP2
126206	1.0	1.0	1.0	1.0	NLRP5
4828	1.0	1.0	1.0	1.0	NMB
4829	1.0	1.0	1.0	1.0	NMBR
347736	1.5	1.0	1.0	2.8	NME9
9111	1.0	1.0	1.0	1.0	NMI
64802	1.0	1.0	-8.8	-7.4	NMNAT1
23530	2.7	1.0	-6.0	1.0	NNT
28987	1.0	1.0	1.0	1.0	NOB1
26155	1.0	1.0	1.0	2.4	NOC2L
4838	1.0	1.0	1.0	1.0	NODAL
79954	1.0	1.0	1.0	-3.5	NOL10
25926	1.8	1.0	1.0	1.0	NOL11
8715	-9.0	1.0	1.0	1.0	NOL4
51406	1.0	1.0	1.0	2.9	NOL7
55035	1.0	1.0	1.0	1.0	NOL8
64434	1.0	-4.0	-4.4	1.0	NOM1
8602	1.0	4.2	1.0	3.6	NOP14
51491	2.6	1.0	1.0	1.0	NOP16
10528	1.0	1.0	3.1	1.0	NOP56
4842	1.0	1.0	1.0	1.0	NOS1
9722	1.0	1.0	1.0	1.0	NOS1AP
51070	1.0	1.0	1.0	1.0	NOSIP
115677	1.0	1.0	1.0	2.4	NOSTRIN
4855	1.0	1.0	1.0	1.0	NOTCH4

4856	2.0	1.0	-3.0	1.0	NOV
27035	-10.2	1.0	1.0	1.0	NOX1
10811	2.8	1.0	1.0	1.0	NOXA1
124056	1.0	1.0	4.3	2.2	NOXO1
122945	-3.2	1.0	-3.3	1.0	NOXRED1
64067	3.7	1.0	-5.6	-6.0	NPAS3
10577	1.0	2.4	1.0	1.0	NPC2
64106	5.0	1.0	1.0	2.7	NPFFR1
10886	1.0	1.0	2.3	1.0	NPFFR2
27031	1.0	1.0	4.8	1.0	NPHP3
7827	1.0	1.9	1.0	1.0	NPHS2
23117	1.6	-11.9	-19.3	-9.1	NPIPL3
80896	1.0	-2.7	-2.8	-2.8	NPL
4869	1.6	1.0	1.0	-2.4	NPM1
4878	1.0	1.0	2.7	1.0	NPPA
4881	1.0	1.0	4.9	1.0	NPR1
4882	1.0	1.0	1.0	1.0	NPR2
8131	-2.9	1.0	1.0	1.0	NPRL3
387129	1.0	1.0	6.2	6.3	NPSR1
23467	1.0	1.0	1.0	-1.7	NPTXR
64111	1.0	1.0	1.0	1.0	NPVF
4852	1.0	1.0	1.0	8.4	NPY
4886	-30.4	1.0	1.0	1.0	NPY1R
4887	1.0	1.0	1.0	-2.7	NPY2R
4835	1.0	1.0	1.0	1.0	NQO2
9572	1.0	-4.9	1.0	-4.7	NR1D1
9975	1.0	1.0	1.0	1.0	NR1D2
9971	3.7	6.4	1.0	1.0	NR1H4
9970	1.0	1.0	1.0	1.0	NR1I3
7181	1.0	1.0	1.0	1.0	NR2C1
7182	1.0	1.0	1.0	1.0	NR2C2
10002	1.0	1.0	1.0	1.0	NR2E3
2063	1.0	1.0	1.0	1.0	NR2F6
2908	1.0	1.0	-5.7	-5.4	NR3C1
4306	1.0	1.0	1.0	1.0	NR3C2
8013	1.0	1.0	1.0	1.0	NR4A3
2516	1.0	3.3	1.0	1.0	NR5A1
2649	1.0	1.0	5.4	6.1	NR6A1
4893	1.5	1.0	1.0	1.0	NRAS
29959	1.7	1.0	1.0	-4.5	NRBP1
4897	1.0	1.0	4.1	1.0	NRCAM
4898	4.9	1.0	1.0	1.0	NRD1
9542	2.3	1.0	1.0	2.8	NRG2
4900	1.0	1.0	1.0	1.0	NRGN
8204	1.0	1.0	1.0	1.0	NRIP1
56675	1.0	1.0	5.1	1.0	NRIP3
11270	1.0	1.0	1.0	3.0	NRM
51299	2.6	-3.9	-6.7	-9.7	NRN1

123904	1.0	1.0	5.1	1.0	NRN1L
8829	1.0	1.0	-3.2	1.0	NRP1
8828	1.0	1.0	4.9	1.0	NRP2
80023	1.0	1.0	1.0	1.0	NRSN2
9378	1.7	-1.7	1.0	5.3	NRXN1
10412	-11.1	-5.6	1.0	-8.4	NSA2
64324	1.0	1.0	1.0	1.0	NSD1
4905	1.0	1.0	1.0	1.0	NSF
84081	1.0	1.0	-1.7	1.0	NSRP1
54888	1.0	1.0	1.0	1.0	NSUN2
63899	2.5	1.0	1.0	-3.2	NSUN3
79730	1.0	1.0	1.0	1.0	NSUN7
115024	17.4	3.3	1.0	3.4	NT5C3L
221294	1.0	1.0	1.0	1.0	NT5DC1
64943	1.5	1.0	-3.1	1.0	NT5DC2
4907	1.0	1.0	1.0	1.0	NT5E
126147	1.0	1.0	1.0	1.0	NTN5
22854	1.0	1.0	1.0	1.0	NTNG1
84628	1.0	1.0	5.3	1.0	NTNG2
84284	1.0	1.0	4.8	1.0	NTPCR
4915	1.0	1.0	1.0	1.0	NTRK2
4923	2.7	1.0	1.0	3.3	NTSR1
9891	1.0	1.0	1.9	1.7	NUAK1
4682	1.0	1.0	1.0	1.0	NUBP1
80224	1.0	1.0	1.0	1.0	NUBPL
4924	1.0	1.0	1.0	1.0	NUCB1
64710	1.0	-8.3	1.0	1.0	NUCKS1
23386	1.0	-4.0	5.1	5.2	NUDCD3
25961	-3.1	1.0	1.0	1.0	NUDT13
256281	2.8	1.0	1.0	2.8	NUDT14
55270	1.0	1.0	1.0	1.0	NUDT15
131870	1.0	1.0	1.0	1.0	NUDT16
84309	1.0	1.0	-3.6	1.0	NUDT16L1
390916	1.0	2.8	5.7	1.0	NUDT19
318	-2.0	-3.8	-3.7	-3.6	NUDT2
11163	1.0	1.0	1.0	1.0	NUDT4
11164	1.0	-2.5	1.0	-2.4	NUDT5
254552	1.0	1.0	1.0	2.4	NUDT8
4926	1.0	1.0	1.0	-3.2	NUMA1
8650	1.0	1.0	1.0	1.0	NUMB
57122	1.0	1.0	1.0	1.0	NUP107
9631	1.0	1.0	1.0	1.0	NUP155
23279	1.0	1.0	3.7	1.0	NUP160
23165	1.0	2.5	2.3	1.0	NUP205
23636	1.0	1.0	4.4	4.8	NUP62
11097	1.0	1.0	2.5	1.0	NUPL2
116150	1.0	1.0	2.5	1.0	NUS1
55998	1.5	3.0	4.0	3.2	NXF5

158046	1.0	1.0	1.0	1.0	NXNL2
11247	1.0	1.0	1.0	1.0	NXPH4
55916	1.0	1.0	1.0	2.2	NXT2
57523	1.0	1.0	1.0	1.0	NYNRIN
4939	1.0	3.2	1.0	1.0	OAS2
4940	2.4	1.0	1.0	4.5	OAS3
8638	1.0	1.0	1.0	1.0	OASL
4947	1.0	1.0	-2.5	1.0	OAZ2
51686	1.0	1.0	1.0	1.0	OAZ3
64859	-1.8	1.0	2.5	1.0	OBFC2A
29991	1.0	1.0	1.0	1.0	OBP2A
84033	1.0	1.0	1.0	1.0	OBSCN
23363	1.0	1.0	3.9	1.0	OBSL1
79629	-42.9	2.7	1.0	1.0	OCEL1
132299	1.0	2.8	2.6	1.0	OCIAD2
4953	2.8	1.0	1.0	1.0	ODC1
57489	1.0	-10.0	-8.6	-5.8	ODF2L
113746	1.0	1.0	1.0	1.0	ODF3
284451	1.0	1.0	1.0	1.0	ODF3L2
146852	1.0	1.0	1.0	1.0	ODF4
10178	1.0	1.0	1.0	4.8	ODZ1
55714	1.0	1.0	2.9	1.0	ODZ3
79676	1.0	4.9	6.9	5.4	OGFOD2
4968	1.8	1.0	1.0	-2.7	OGG1
4969	1.0	1.0	4.9	1.0	OGN
8473	-1.8	1.0	1.0	1.0	OGT
729082	1.0	-5.7	1.0	1.0	OIP5-AS1
10439	1.0	1.0	3.4	-3.5	OLFM1
169611	1.0	1.0	4.9	2.5	OLFML2A
56944	-1.8	1.0	1.0	1.0	OLFML3
116448	1.0	1.0	1.0	1.0	OLIG1
4958	1.0	1.0	1.0	1.0	OMD
4974	1.6	1.0	1.0	1.0	OMG
9480	1.0	1.0	1.0	3.6	ONECUT2
441161	1.0	5.0	6.2	1.0	OOEP
4976	1.0	1.0	1.0	3.3	OPA1
93377	1.0	1.0	1.0	3.3	OPALIN
221391	1.0	-2.9	1.0	1.0	OPN5
4985	1.8	1.0	1.6	1.0	OPRD1
4987	1.0	1.0	1.0	1.0	OPRL1
341276	17.5	1.0	1.0	1.0	OR10A2
283297	1.0	1.0	1.0	2.8	OR10A4
442194	1.0	1.0	1.0	1.0	OR10C1
219869	1.0	4.0	1.0	1.0	OR10G8
26539	1.0	1.0	1.0	6.5	OR10H1
26538	1.7	1.0	1.0	-1.6	OR10H2
128360	1.0	1.0	1.0	2.5	OR10T2
440153	1.0	1.0	1.0	1.0	OR11H12

81797	1.0	1.0	1.0	1.0	OR12D3
138804	1.0	1.0	1.0	1.0	OR13C4
286365	1.0	1.0	1.0	1.0	OR13D1
26189	1.0	1.0	3.0	1.0	OR1A2
26188	1.0	1.0	1.0	1.0	OR1C1
8386	3.0	1.0	1.0	1.0	OR1D5
8387	1.0	1.0	2.8	1.0	OR1E1
4992	1.0	1.0	3.6	1.0	OR1F1
26184	1.0	2.7	1.0	1.0	OR1F2P
26735	1.0	1.0	1.0	1.0	OR1L3
401428	1.0	4.4	1.0	3.4	OR2A20P
392138	-10.5	1.0	1.0	1.0	OR2A25
401427	1.0	1.0	1.0	1.0	OR2A7
81697	1.0	-2.9	1.0	1.0	OR2B2
26212	1.0	1.0	1.0	1.0	OR2B6
4993	1.0	1.0	1.0	1.0	OR2C1
81472	1.0	1.0	1.0	1.0	OR2C3
26211	1.0	1.0	1.9	2.3	OR2F1
26716	1.0	-5.6	-6.4	-5.6	OR2H1
7932	9.5	2.5	1.0	1.0	OR2H2
26707	1.0	1.5	1.0	1.0	OR2J2
391194	1.0	1.0	1.0	1.0	OR2M2
56656	1.0	1.0	1.0	1.0	OR2S2
401993	1.0	1.0	1.0	6.8	OR2T5
343172	1.0	1.0	1.0	1.0	OR2T8
285659	1.0	1.0	1.0	1.0	OR2V2
343171	1.0	1.0	2.3	1.0	OR2W3
4995	1.0	1.0	3.4	1.0	OR3A2
81328	1.0	1.0	1.0	1.0	OR4A15
124538	1.0	1.0	1.0	1.0	OR4D2
79544	1.5	4.8	1.0	1.0	OR4K1
390436	1.0	1.0	1.0	1.0	OR4K17
79345	1.0	1.0	1.0	1.0	OR51B2
79339	1.0	1.0	1.0	1.0	OR51B4
119774	1.0	2.7	1.0	1.0	OR52K2
387748	1.0	1.0	1.0	5.6	OR56B1
338675	1.0	2.8	2.5	1.0	OR5AP2
338674	1.0	10.5	1.0	1.0	OR5F1
10798	1.0	1.0	1.0	1.0	OR5I1
120065	1.0	3.1	4.1	2.6	OR5P2
120066	1.0	1.0	1.0	1.0	OR5P3
8590	1.0	1.0	1.0	1.0	OR6A2
128372	1.0	1.0	3.1	3.3	OR6N1
391112	1.0	1.0	1.0	1.0	OR6Y1
390892	1.0	1.0	1.0	1.0	OR7A10
26333	1.0	1.0	1.0	4.5	OR7A17
125958	1.0	1.0	1.0	1.0	OR7D4
10819	1.0	1.0	1.0	1.0	OR7E14P

219445	1.0	3.1	3.1	1.0	OR7E5P
79315	1.0	1.0	1.0	1.0	OR7E91P
390883	1.0	1.0	2.6	1.0	OR7G3
26493	-1.8	1.0	1.0	1.0	OR8B8
283160	1.0	1.0	1.5	1.0	OR8D2
26494	1.0	1.0	1.0	1.0	OR8G1
219477	1.0	1.0	1.0	1.0	OR8J1
80228	1.0	1.0	1.0	1.0	ORAI2
93129	1.0	1.0	1.0	1.0	ORAI3
220064	1.0	1.0	1.0	1.0	ORAOV1
4999	1.0	1.0	1.0	1.0	ORC2
23595	4.0	29.4	1.0	1.0	ORC3
5000	1.0	1.0	1.0	1.0	ORC4
5001	1.0	2.6	2.7	1.0	ORC5
5004	1.0	1.0	1.0	1.0	ORM1
5005	1.0	1.0	1.0	1.0	ORM2
94101	-4.2	1.0	1.0	1.0	ORMDL1
29095	1.0	1.0	3.0	1.0	ORMDL2
10956	1.0	1.0	1.0	1.0	OS9
5007	1.0	1.0	4.2	4.0	OSBP
23762	1.0	1.0	2.9	1.0	OSBP2
114876	1.0	1.0	1.0	1.0	OSBPL1A
114879	-5.3	1.0	4.7	1.0	OSBPL5
55644	1.0	1.0	1.0	1.0	OSGEP
5008	1.0	6.1	1.0	4.8	OSM
26578	1.0	1.0	1.0	1.0	OSTF1
55611	1.0	-4.3	1.0	1.0	OTUB1
78990	1.0	1.0	1.0	1.0	OTUB2
220213	1.0	1.0	1.0	5.7	OTUD1
139562	2.7	4.4	1.0	1.0	OTUD6A
51633	1.0	-2.1	-2.1	1.0	OTUD6B
161725	1.9	2.8	1.0	1.0	OTUD7A
5015	1.0	1.0	2.9	2.6	OTX2
22953	1.0	1.0	1.0	1.0	P2RX2
9127	1.0	1.0	1.0	2.3	P2RX6
5027	1.0	1.0	1.0	1.0	P2RX7
27334	16.6	1.0	1.0	1.0	P2RY10
53829	1.0	1.0	2.4	1.0	P2RY13
5030	1.0	1.0	1.0	-1.7	P2RY4
5034	82.3	78.2	78.7	78.5	P4HB
54681	-14.5	1.0	1.0	1.0	P4HTM
80336	1.0	1.0	1.0	1.0	PABPC1L
8761	1.0	1.0	5.0	1.0	PABPC4
132430	1.0	1.0	1.0	1.0	PABPC4L
140886	1.0	4.6	1.0	1.0	PABPC5
29993	1.0	2.6	3.3	1.0	PACSIN1
11252	1.0	1.0	1.0	-3.1	PACSIN2
29943	1.0	1.0	1.0	1.0	PADI1

11240	1.0	1.0	3.8	1.0	PADI2
51702	1.0	1.9	1.0	-4.1	PADI3
54623	-1.5	1.0	1.0	1.0	PAF1
5048	1.0	1.0	1.0	1.0	PAFAH1B1
5050	1.0	1.0	1.0	1.0	PAFAH1B3
9506	1.0	1.0	1.0	-6.1	PAGE4
51247	1.0	1.0	1.0	5.9	PAIP2
55003	1.0	-2.6	-3.6	1.0	PAK1IP1
56924	1.0	1.0	3.1	1.0	PAK6
23022	1.0	1.0	1.0	2.8	PALLD
342979	1.0	2.8	1.6	2.8	PALM3
25891	1.0	1.0	1.0	3.5	PAMR1
80025	1.0	1.0	1.0	1.0	PANK2
79646	1.0	-2.6	1.0	1.0	PANK3
196743	1.0	1.0	1.0	1.0	PAOX
390928	1.0	1.0	1.0	1.0	PAPL
89932	1.0	1.0	2.6	1.0	PAPLN
10914	1.0	1.0	1.0	1.0	PAPOLA
9061	1.0	1.0	1.0	1.0	PAPSS1
124222	1.0	1.0	1.0	1.0	PAQR4
164091	1.0	1.0	1.0	1.0	PAQR7
85315	1.0	1.0	1.0	1.0	PAQR8
56288	1.0	6.6	7.1	1.0	PARD3
8505	1.0	1.0	1.0	1.0	PARG
55486	1.0	-2.5	1.0	1.0	PARL
5073	1.0	1.0	1.0	1.0	PARN
64761	1.0	1.0	1.0	1.0	PARP12
10038	1.0	1.0	1.0	1.0	PARP2
10039	1.0	1.0	-1.6	1.0	PARP3
143	-2.9	1.0	1.0	1.0	PARP4
79668	1.0	1.0	1.0	2.5	PARP8
25859	1.0	1.0	1.0	1.0	PART1
29780	1.0	1.0	2.2	1.0	PARVB
23178	1.0	1.0	1.0	5.9	PASK
23598	1.0	1.0	5.9	3.6	PATZ1
5074	1.0	1.0	1.0	1.0	PAWR
5078	-2.6	1.0	4.2	4.9	PAX4
5080	1.0	1.0	1.0	1.0	PAX6
5083	1.0	-2.6	1.0	1.0	PAX9
59351	1.0	1.0	1.0	1.0	PBOV1
5087	1.0	1.0	1.0	1.0	PBX1
54039	1.0	1.0	1.0	1.0	PCBP3
57060	1.0	1.0	1.0	1.0	PCBP4
5097	1.9	4.0	1.0	1.0	PCDH1
51294	1.0	6.7	1.0	1.0	PCDH12
65217	1.0	-3.3	-6.0	-3.2	PCDH15
57526	1.0	4.5	1.0	1.0	PCDH19
5099	1.0	1.0	1.0	1.0	PCDH7

5101	-9.1	1.0	1.0	1.0	PCDH9
56138	1.0	3.7	1.0	2.4	PCDHA11
56123	1.0	2.9	3.0	1.0	PCDHB13
56131	1.0	2.4	1.0	1.0	PCDHB4
56128	1.0	5.8	1.0	5.7	PCDHB8
56107	1.0	1.0	1.0	2.2	PCDHGA9
56103	1.0	1.0	2.0	1.0	PCDHGB2
8641	1.0	2.5	1.0	1.0	PCDHGB4
56099	1.0	1.0	-4.5	1.0	PCDHGB7
56097	1.0	1.0	1.0	1.0	PCDHGC5
84759	1.0	1.0	1.0	1.0	PCGF1
84108	1.0	1.0	1.0	1.0	PCGF6
55795	1.0	1.0	1.0	1.0	PCID2
5106	-1.5	1.0	1.0	-8.7	PCK2
5110	-2.0	-2.5	-2.5	1.0	PCMT1
5111	1.0	-2.5	-2.5	-2.5	PCNA
57092	1.0	1.0	6.3	1.0	PCNP
22990	2.4	1.0	-2.0	1.0	PCNX
5121	-2.7	1.0	-2.6	-2.5	PCP4
5125	1.0	29.7	1.0	1.0	PCSK5
5046	1.0	1.0	2.8	1.0	PCSK6
9159	1.9	1.0	1.0	1.9	PCSK7
255738	1.0	1.0	3.6	3.4	PCSK9
58488	1.0	1.0	1.0	1.0	PCTP
78991	1.0	1.0	4.6	1.0	PCYOX1L
5130	1.0	1.0	-2.6	1.0	PCYT1A
9468	1.0	1.0	1.0	1.0	PCYT1B
5833	1.5	1.0	1.0	2.4	PCYT2
5132	1.0	1.0	1.0	1.0	PDC
22984	1.0	1.0	1.0	1.0	PDCD11
80380	1.0	1.0	1.0	1.0	PDCD1LG2
5134	1.0	1.0	1.0	-2.6	PDCD2
10016	1.0	1.0	1.0	1.0	PDCD6
347862	-4.5	1.0	1.0	1.6	PDDC1
5136	1.0	1.0	1.0	1.0	PDE1A
5137	1.0	1.0	1.0	1.0	PDE1C
5141	1.0	1.0	1.0	1.0	PDE4A
9659	1.0	-4.2	-4.3	1.0	PDE4DIP
5158	1.0	1.0	1.0	1.0	PDE6B
5148	1.0	1.0	1.0	1.0	PDE6G
5150	2.0	1.0	1.0	1.0	PDE7A
5152	1.0	1.0	1.0	1.0	PDE9A
5154	1.0	5.1	5.1	4.7	PDGFA
80310	1.8	1.0	1.0	1.0	PDGFD
5156	-8.5	4.1	3.2	1.0	PDGFRA
8050	1.0	1.0	1.0	1.0	PDHX
9601	-5.9	-3.2	1.0	1.0	PDIA4
10954	1.0	1.0	1.0	1.0	PDIA5

149420	1.0	1.0	1.0	1.0	PDIK1L
5164	1.0	1.0	1.0	1.0	PDK2
5165	1.0	1.0	1.0	1.0	PDK3
5166	1.0	8.0	8.0	1.0	PDK4
27295	1.0	1.0	1.0	1.0	PDLIM3
8572	1.0	1.0	1.0	1.0	PDLIM4
10611	1.0	2.3	-4.1	-4.4	PDLIM5
5170	1.5	1.0	1.0	1.0	PDPK1
81572	1.0	1.0	1.0	1.9	PDRG1
23590	-4.9	-3.2	1.0	1.0	PDSS1
283970	1.0	1.0	2.4	1.0	PDXDC2P
8566	1.0	1.0	3.1	3.4	PDXK
57026	-3.3	1.0	1.0	1.0	PDXP
79849	1.0	1.0	1.0	1.0	PDZD3
79955	-5.5	1.0	1.0	1.0	PDZD7
10158	1.0	-2.3	1.0	1.0	PDZK1IP1
23024	1.0	1.0	1.0	1.0	PDZRN3
29951	2.4	2.2	1.0	1.0	PDZRN4
8682	1.0	1.8	1.0	1.0	PEA15
157310	1.0	1.0	6.1	1.0	PEBP4
5175	1.0	-6.7	1.0	1.0	PECAM1
553115	-2.0	1.0	1.0	1.0	PEF1
5178	1.0	-3.1	-2.6	1.0	PEG3
5184	1.0	1.0	1.7	1.0	PEPD
8863	1.0	1.0	1.0	1.0	PER3
5189	-5.9	-6.8	-6.5	1.0	PEX1
8800	1.0	1.0	2.3	1.0	PEX11A
9409	1.0	1.0	1.0	1.0	PEX16
55670	1.0	1.0	1.0	1.0	PEX26
5830	1.0	1.0	1.0	1.0	PEX5
5191	1.0	1.0	6.6	1.0	PEX7
5207	9.1	1.0	2.6	1.0	PFKFB1
5208	1.0	2.4	1.8	1.0	PFKFB2
5209	-1.8	1.0	1.0	1.0	PFKFB3
345456	1.0	1.0	1.0	1.0	PFN3
643834	9.1	8.9	9.2	11.4	PGA3
5223	1.0	1.0	1.0	1.0	PGAM1
192111	1.0	1.0	1.0	1.0	PGAM5
80055	1.0	1.0	-5.5	1.0	PGAP1
27315	1.0	1.0	1.0	1.0	PGAP2
93210	-1.9	1.0	1.0	1.7	PGAP3
267002	1.0	1.0	1.0	2.5	PGBD2
5225	2.3	1.0	1.0	1.0	PGC
10404	1.0	3.5	1.0	3.6	PGCP
5228	-3.0	1.0	1.0	1.0	PGF
114770	1.0	1.0	1.0	1.0	PGLYRP2
57115	1.6	1.0	1.0	1.0	PGLYRP4
55276	1.0	1.0	1.0	1.0	PGM2

5239	1.0	1.0	1.0	1.0	PGM5
54858	1.0	1.0	1.0	1.0	PGPEP1
10857	1.0	1.0	1.0	1.0	PGRMC1
9489	-4.2	1.0	1.0	1.0	PGS1
221692	1.0	1.0	1.0	4.5	PHACTR1
9749	-1.5	1.0	-3.1	1.0	PHACTR2
116154	1.0	7.2	1.0	1.0	PHACTR3
65979	2.4	1.0	-4.2	1.0	PHACTR4
5245	2.5	-2.8	-2.6	-2.6	PHB
1911	1.0	1.0	1.0	-6.0	PHC1
1912	1.0	1.0	1.0	-2.6	PHC2
55274	7.3	-2.5	-3.0	-3.2	PHF10
57649	1.0	1.0	1.0	1.0	PHF12
9678	1.0	1.0	1.0	1.0	PHF14
23338	-17.4	1.0	1.0	1.0	PHF15
79960	1.0	1.0	2.5	1.0	PHF17
51105	-1.6	1.0	-4.0	-3.5	PHF20L1
51317	1.0	1.0	1.0	1.0	PHF21A
79142	-1.8	1.0	1.0	1.0	PHF23
84295	1.0	-1.8	1.0	1.0	PHF6
23133	-5.6	1.0	-6.0	-6.2	PHF8
55023	1.0	1.0	-3.7	1.0	PHIP
5255	1.0	9.9	1.0	1.0	PHKA1
5257	1.0	1.0	1.0	1.0	PHKB
22822	1.0	1.0	1.0	1.0	PHLDA1
90102	-53.1	1.0	2.6	3.2	PHLDB2
653583	1.0	1.0	1.0	-2.8	PHLDB3
23239	1.0	1.0	1.8	4.3	PHLPP1
162466	1.0	2.7	2.5	2.5	PHOSPHO1
51050	1.0	1.0	1.0	1.0	PI15
5266	1.5	1.0	1.0	1.0	PI3
5298	1.0	5.5	1.0	1.0	PI4KB
51588	-1.7	-6.7	-5.7	-5.4	PIAS4
10464	-4.7	-4.4	-3.8	-3.9	PIBF1
8301	1.0	1.0	1.0	1.0	PICALM
55022	1.0	1.0	1.0	1.0	PID1
55367	2.0	1.0	1.0	1.0	PIDD
80119	3.2	1.0	1.0	1.0	PIF1
5281	1.0	1.0	1.0	1.0	PIGF
10026	1.0	1.0	1.0	1.0	PIGK
9487	1.0	1.0	4.8	1.0	PIGL
84720	1.0	1.0	1.0	1.0	PIGO
9091	1.0	1.0	1.0	1.0	PIGQ
94005	1.0	1.7	4.3	1.0	PIGS
128869	1.0	1.0	1.0	1.0	PIGU
80235	1.0	1.0	1.0	1.0	PIGZ
55011	1.0	1.0	1.0	1.0	PIH1D1
5286	-3.1	1.0	1.0	1.0	PIK3C2A

5289	1.0	6.7	4.3	1.0	PIK3C3
5293	1.0	1.0	1.0	1.0	PIK3CD
23533	1.8	1.0	1.0	1.0	PIK3R5
146850	1.0	1.0	1.0	1.0	PIK3R6
29992	-17.0	1.0	-2.7	1.0	PILRA
5303	1.0	1.0	-2.5	1.0	PIN4
54984	1.0	1.0	1.0	3.7	PINX1
54103	1.0	1.0	1.0	1.0	PION
5304	-18.9	1.0	3.2	1.0	PIP
8396	1.0	1.0	1.0	1.0	PIP4K2B
8394	1.6	1.0	1.0	1.0	PIP5K1A
138429	1.0	1.0	-3.9	1.0	PIP5KL1
57095	1.0	1.0	1.0	1.0	PITHD1
5306	1.0	1.0	4.1	1.0	PITPNA
26207	1.0	2.2	1.0	1.0	PITPNC1
10531	1.0	-2.5	1.0	1.0	PITRM1
64219	1.0	1.0	5.3	-4.2	PJA1
114780	1.0	1.0	1.0	1.0	PKD1L2
342372	1.0	1.0	1.0	1.0	PKD1L3
91461	1.0	1.0	-2.7	-3.2	PKDCC
10343	1.0	1.0	-2.2	1.0	PKDREJ
150967	1.0	1.0	1.0	1.0	PKI55
5569	-2.5	1.0	1.0	1.0	PKIA
5570	1.0	1.0	1.0	1.0	PKIB
9088	1.0	1.0	1.0	1.0	PKMYT1
5585	-1.8	1.0	1.0	1.0	PKN1
5586	1.0	1.0	1.0	1.0	PKN2
5317	1.0	1.0	1.0	1.0	PKP1
51365	1.0	6.2	5.2	1.0	PLA1A
84647	1.0	1.0	1.0	1.0	PLA2G12B
11145	1.0	5.0	4.7	4.1	PLA2G16
26279	-16.7	1.0	1.0	1.0	PLA2G2D
64600	6.0	1.0	-3.0	-3.2	PLA2G2F
8605	2.3	-2.6	1.0	1.0	PLA2G4C
255189	1.0	1.0	1.0	1.0	PLA2G4F
10761	1.0	1.0	1.0	1.0	PLAC1
51316	1.0	-5.8	1.0	-3.4	PLAC8
5324	1.0	1.0	1.0	-3.9	PLAG1
5329	1.0	1.0	1.0	1.0	PLAUR
151056	1.0	-3.7	-7.8	-3.6	PLB1
196463	1.0	1.0	1.0	1.0	PLBD2
23236	1.7	1.0	3.5	6.2	PLCB1
5333	1.0	1.0	-4.2	1.0	PLCD1
84812	1.0	1.0	-1.5	1.0	PLCD4
51196	1.0	1.0	1.0	1.0	PLCE1
5335	1.0	-3.2	-3.4	-3.3	PLCG1
23007	-1.7	1.0	1.0	1.0	PLCH1
23228	-15.6	1.0	-35.4	1.0	PLCL2

55344	1.0	1.0	1.0	4.5	PLCXD1
257068	1.0	1.0	1.0	1.0	PLCXD2
122618	-2.9	1.0	1.0	-9.1	PLD4
201164	1.0	1.0	3.3	1.0	PLD6
84725	1.0	1.0	-5.3	1.0	PLEKHA8
55041	1.0	1.0	1.0	1.0	PLEKHB2
79156	-4.7	1.0	3.6	1.0	PLEKHF1
57480	1.0	1.0	1.0	1.0	PLEKHG1
26030	1.0	1.0	1.0	1.0	PLEKHG3
153478	1.6	1.0	-3.9	1.0	PLEKHG4B
9842	6.7	7.4	6.4	1.0	PLEKHM1
23207	1.0	1.0	1.0	-3.7	PLEKHM2
84069	1.0	1.0	1.0	1.0	PLEKHN1
51177	1.0	-2.6	1.0	1.0	PLEKHO1
5340	5.5	1.0	1.0	1.0	PLG
10226	1.0	1.0	1.0	1.0	PLIN3
729359	1.0	1.0	1.0	1.0	PLIN4
440503	1.0	3.6	9.9	4.8	PLIN5
126520	1.0	1.0	1.0	1.0	PLK5
51090	-3.2	1.0	1.0	1.0	PLLP
5351	1.7	1.0	1.0	1.0	PLOD1
5352	1.0	1.0	2.6	1.0	PLOD2
5354	1.0	-7.9	-4.5	-5.0	PLP1
83483	-1.6	-1.7	-2.6	1.0	PLVAP
57125	10.4	1.0	2.7	2.4	PLXDC1
91584	1.0	-4.8	1.0	1.0	PLXNA4
23654	1.0	1.0	-4.9	-4.3	PLXNB2
10154	-2.6	-3.5	1.0	1.0	PLXNC1
148811	1.0	-1.7	-2.8	1.0	PM20D1
5366	1.0	1.0	1.0	1.0	PMAIP1
5367	1.0	1.0	1.0	1.0	PMCH
56937	1.0	1.0	1.0	1.0	PMEPA1
83449	1.0	3.1	3.4	3.4	PMFBP1
23203	1.0	1.0	3.1	1.0	PMPCA
5378	1.0	1.0	-1.9	1.0	PMS1
5395	1.0	1.0	1.0	1.0	PMS2
5380	-3.2	1.0	2.3	1.0	PMS2L2
10654	1.0	1.0	1.0	1.0	PMVK
139728	5.5	1.0	1.0	1.0	PNCK
11284	1.8	1.0	1.0	1.0	PNKP
5407	1.0	1.0	1.0	1.0	PNLIPRP1
9240	1.0	1.0	4.4	1.0	PNMA1
114824	1.5	1.0	-2.4	1.0	PNMA5
5409	1.0	-4.4	1.0	1.0	PNMT
285848	1.0	-3.8	1.0	1.0	PNPLA1
80339	1.0	1.0	-2.7	1.0	PNPLA3
375775	1.0	1.0	1.0	1.0	PNPLA7
87178	4.5	1.0	-9.1	1.0	PNPT1

10957	1.0	1.0	1.0	1.0	PNRC1
55629	1.0	2.3	5.1	3.3	PNRC2
25886	-4.3	1.0	1.0	1.0	POC1A
282809	1.0	1.0	1.0	1.0	POC1B
50512	1.0	1.0	1.0	1.0	PODXL2
79983	1.0	1.0	-1.8	1.0	POF1B
23509	1.0	1.0	1.0	-3.2	POFUT1
23275	1.0	3.3	1.0	2.3	POFUT2
23126	1.0	1.0	6.3	1.0	POGZ
5423	1.0	1.0	-9.8	1.0	POLB
5425	1.0	1.0	1.0	1.0	POLD2
10714	1.0	1.0	1.0	1.0	POLD3
5426	1.0	1.0	-2.7	1.0	POLE
5429	1.0	1.0	1.0	1.0	POLH
27434	1.0	1.0	-7.0	1.0	POLM
10721	1.0	1.0	1.0	3.0	POLQ
25885	1.0	1.0	1.0	1.0	POLR1A
9533	-14.9	-3.1	1.0	1.0	POLR1C
51082	1.0	1.0	1.0	1.0	POLR1D
5430	1.8	1.0	5.4	5.6	POLR2A
5433	1.0	1.0	1.0	1.0	POLR2D
5434	-1.9	1.0	1.0	1.0	POLR2E
5435	1.0	17.0	1.0	1.0	POLR2F
5437	1.0	1.0	3.7	1.0	POLR2H
5439	4.6	1.7	-9.1	1.0	POLR2J
246721	1.8	1.0	1.0	5.8	POLR2J2
84820	1.0	1.0	1.0	1.0	POLR2J4
5440	-2.9	1.0	1.0	1.0	POLR2K
5441	-2.0	1.0	1.0	1.0	POLR2L
55703	1.0	1.0	1.0	1.0	POLR3B
661	1.0	1.0	1.0	1.0	POLR3D
10621	1.0	1.0	1.0	3.0	POLR3F
10622	1.0	1.0	1.0	1.0	POLR3G
84265	2.0	4.8	1.0	1.0	POLR3GL
171568	1.0	-2.9	-2.5	1.0	POLR3H
646074	1.0	-3.3	-5.3	-1.9	POM121L10P
25812	1.0	1.0	1.0	1.0	POM121L1P
266697	1.0	1.0	1.0	1.0	POM121L4P
55624	-2.6	-10.7	1.0	1.0	POMGNT1
22932	2.1	1.0	-6.2	1.0	POMZP3
5444	1.0	1.0	1.0	1.0	PON1
64091	1.0	1.0	-3.2	1.0	POPDC2
5447	2.7	1.0	1.8	1.0	POR
10631	-2.8	1.0	1.0	1.0	POSTN
25913	1.0	1.0	1.0	-3.1	POT1
317754	1.0	1.0	1.0	1.0	POTED
5452	2.2	-3.7	1.0	1.0	POU2F2
5453	1.0	1.0	-2.3	1.0	POU3F1

5458	2.6	1.0	4.2	1.0	POU4F2
134187	-7.5	1.0	3.3	4.6	POU5F2
100192379	1.0	4.1	1.0	1.0	PP12613
100130449	2.0	1.0	1.0	1.0	PP14571
56342	1.0	-2.9	1.0	1.0	PPAN
8611	1.0	1.0	2.6	1.0	PPAP2A
8613	3.9	1.0	1.8	7.7	PPAP2B
84513	1.0	1.0	1.0	1.0	PPAPDC1B
84814	1.0	1.0	1.0	-3.0	PPAPDC3
5465	1.0	1.0	5.5	1.0	PPARA
5468	1.0	1.0	-5.8	1.0	PPARG
133522	-2.0	1.0	1.0	-5.6	PPARGC1B
10895	1.0	1.0	1.0	1.0	PPBPL2
60490	1.0	1.0	3.7	1.0	PPCDC
79717	1.0	1.0	1.0	1.0	PPCS
79144	1.0	-3.2	-2.8	-3.2	PPDPF
8541	1.0	1.0	2.8	1.0	PPFIA3
51535	1.0	-4.4	1.0	1.0	PPHLN1
5479	1.0	-2.7	-2.7	-2.6	PPIB
9360	1.0	1.0	1.0	1.0	PPIG
10465	1.0	3.9	1.0	4.5	PPIH
53938	1.0	1.0	4.7	1.0	PPIL3
85313	1.0	1.0	5.5	6.0	PPIL4
9677	1.0	1.0	1.0	1.0	PPIP5K1
5495	1.0	1.0	-3.6	-3.4	PPM1B
8493	1.0	1.0	1.9	1.0	PPM1D
22843	31.7	1.0	-3.1	1.0	PPM1E
9647	1.0	-2.6	1.0	1.0	PPM1F
152926	1.0	1.0	1.0	1.0	PPM1K
147699	1.0	1.0	1.0	1.0	PPM1N
5498	1.6	1.0	1.0	1.0	PPOX
5499	-4.7	1.0	1.0	1.0	PPP1CA
6992	1.7	1.0	1.0	-3.0	PPP1R11
4660	1.0	1.0	1.0	1.0	PPP1R12B
54776	1.0	-2.8	1.0	1.0	PPP1R12C
23368	1.7	-7.6	1.0	1.0	PPP1R13B
23645	-6.2	1.0	1.0	1.0	PPP1R15A
26051	-2.4	1.0	1.0	1.0	PPP1R16B
10842	1.0	1.0	4.5	1.0	PPP1R17
151242	1.0	1.0	1.0	1.0	PPP1R1C
129285	-3.6	-5.6	1.0	-2.0	PPP1R21
9858	1.0	1.0	3.7	1.0	PPP1R26
79660	1.0	1.0	1.0	1.0	PPP1R3B
5507	1.0	3.6	5.1	1.0	PPP1R3C
5516	1.0	1.0	1.0	1.0	PPP2CB
5518	3.1	1.0	1.0	1.0	PPP2R1A
5520	-3.4	1.0	1.0	-2.0	PPP2R2A
55844	3.2	1.0	1.0	2.4	PPP2R2D

5525	1.7	1.0	1.0	2.4	PPP2R5A
5530	1.0	1.0	1.0	1.0	PPP3CA
5534	1.0	1.0	1.0	1.0	PPP3R1
55370	1.0	5.7	1.0	1.0	PPP4R1L
151987	1.0	1.0	-6.8	1.0	PPP4R2
9701	-3.0	1.0	1.0	1.0	PPP6R2
55291	1.0	1.0	5.2	5.1	PPP6R3
51029	1.0	4.1	1.0	1.0	PPPDE1
23082	1.0	1.0	1.0	1.0	PPRC1
5538	1.0	1.0	1.0	1.0	PPT1
160760	1.0	1.0	1.7	1.0	PPTC7
23614	1.0	1.0	1.0	2.4	PPY2
10084	1.0	1.0	3.1	1.0	PQBP1
80148	1.0	1.0	1.0	1.0	PQLC1
54896	1.0	1.0	1.0	1.0	PQLC2
84366	1.0	1.0	1.0	1.0	PRAC
84279	1.0	1.0	1.0	1.0	PRADC1
23532	1.0	1.0	1.0	2.6	PRAME
65121	1.0	1.0	1.0	1.0	PRAMEF1
5542	4.0	6.8	6.7	1.0	PRB1
5545	2.0	-3.4	-7.8	1.0	PRB4
56980	-4.5	-4.8	-7.6	1.0	PRDM10
56981	1.6	1.0	1.6	1.0	PRDM11
63976	1.0	1.0	-3.0	-3.3	PRDM16
7799	1.0	4.8	6.3	1.0	PRDM2
11108	1.0	1.0	-3.0	1.0	PRDM4
56978	1.0	1.0	1.0	1.0	PRDM8
56979	2.1	1.0	-3.9	1.0	PRDM9
7001	1.0	-5.5	-5.6	1.0	PRDX2
10549	1.0	1.0	6.0	1.0	PRDX4
25824	21.1	1.0	4.4	1.0	PRDX5
5549	1.0	1.0	1.0	1.0	PRELP
5550	1.0	1.0	1.0	1.0	PREP
9581	2.1	1.0	1.0	1.0	PREPL
80243	2.6	1.0	1.0	1.0	PREX2
10394	1.0	1.0	1.0	3.5	PRG3
85441	2.4	1.0	3.6	1.0	PRIC285
4007	1.0	1.0	1.0	1.0	PRICKLE3
5557	1.0	3.0	3.1	2.7	PRIM1
100169750	1.0	1.0	1.0	1.0	PRINS
5562	1.0	1.0	1.0	1.0	PRKAA1
5567	-2.0	1.0	1.0	6.7	PRKACB
5568	1.0	1.0	1.0	1.0	PRKACG
5571	1.0	1.0	1.0	1.0	PRKAG1
5573	1.0	1.0	1.0	1.0	PRKAR1A
5575	-1.6	1.0	4.9	1.0	PRKAR1B
5576	1.0	1.0	1.0	1.0	PRKAR2A
5583	8.7	1.0	1.0	8.5	PRKCH

5587	1.0	4.7	4.8	4.7	PRKD1
23683	1.0	1.0	1.0	-5.9	PRKD3
79706	1.7	1.0	1.0	1.0	PRKRIP1
5612	1.0	1.0	1.0	-3.4	PRKRIR
5616	1.0	1.0	2.8	3.0	PRKY
5617	1.0	1.0	2.2	1.0	PRL
5618	1.0	1.0	1.0	-2.7	PRLR
5619	1.0	-3.6	-3.5	-3.5	PRM1
5620	1.0	1.0	-4.3	1.0	PRM2
58531	-4.0	1.0	1.0	1.0	PRM3
90826	1.0	1.0	5.9	4.1	PRMT10
54496	4.8	-6.0	-5.4	1.0	PRMT7
5621	1.0	1.0	1.0	1.0	PRNP
29018	1.0	-3.6	-5.1	-4.6	PRO1768
55478	1.0	1.0	2.7	1.0	PRO2012
114224	1.0	1.0	4.4	1.0	PRO2852
5625	-7.0	1.0	1.0	1.0	PRODH
58510	1.0	2.5	1.8	1.0	PRODH2
60675	1.0	1.0	9.9	1.0	PROK2
150696	2.0	1.0	-6.5	1.0	PROM2
5626	2.3	1.0	1.0	1.0	PROP1
344405	1.0	1.0	-4.5	1.0	PRORSD1P
5627	1.6	1.0	-6.1	1.0	PROS1
80209	1.0	1.0	1.0	1.0	PROSER1
8858	1.0	1.0	-5.9	1.0	PROZ
27339	1.0	1.0	1.0	-2.6	PRPF19
26121	-2.1	-5.8	-5.2	1.0	PRPF31
55660	1.0	1.0	4.6	4.2	PRPF40A
8899	1.0	1.0	1.0	2.5	PRPF4B
10594	-1.9	1.0	-5.6	-5.8	PRPF8
5631	1.0	-6.1	-7.8	1.0	PRPS1
54458	1.0	-4.3	1.0	1.0	PRR13
78994	1.7	1.0	1.0	1.0	PRR14
222171	2.2	4.1	3.0	1.0	PRR15
285800	1.0	1.0	1.0	2.3	PRR18
80758	-3.6	1.0	-3.8	1.0	PRR7
133619	1.0	1.0	1.0	1.0	PRRC1
5639	1.0	1.0	1.0	1.0	PRRG2
79057	1.0	1.0	1.0	-4.4	PRRG3
112476	1.0	1.7	1.0	1.0	PRRT2
51450	1.0	1.0	1.0	-1.7	PRRX2
5644	-8.6	-3.1	-3.0	-3.1	PRSS1
10279	1.0	2.0	3.2	1.0	PRSS16
5645	1.0	1.0	1.0	1.0	PRSS2
83886	1.0	1.0	1.0	1.0	PRSS27
260429	1.0	1.0	1.0	1.0	PRSS33
167681	1.0	7.2	1.0	1.0	PRSS35
339501	1.0	1.0	2.8	1.0	PRSS38

646960	1.0	1.0	-2.1	1.0	PRSS56
400668	1.0	1.0	1.0	-1.6	PRSS57
56952	1.0	1.0	5.7	-5.5	PRTFDC1
5660	-17.3	1.0	1.0	1.0	PSAP
5663	1.0	1.0	1.0	2.5	PSEN1
5664	1.0	1.8	2.0	1.9	PSEN2
55851	1.0	-2.7	1.0	1.0	PSENEN
5680	1.0	1.0	1.0	1.0	PSG11
5673	-14.6	1.0	1.0	1.0	PSG5
5678	-2.0	-7.6	1.0	-6.0	PSG9
11168	-1.6	-6.3	-4.8	-6.3	PSIP1
5687	1.0	1.0	1.0	1.0	PSMA6
5699	1.0	1.0	1.0	1.0	PSMB10
5690	-4.6	1.0	1.0	1.0	PSMB2
5700	1.0	1.0	2.6	1.0	PSMC1
5702	1.0	1.0	1.0	1.0	PSMC3
29893	1.0	1.0	-3.4	1.0	PSMC3IP
5705	1.0	1.0	1.0	1.0	PSMC5
5706	1.0	1.0	1.0	1.0	PSMC6
5707	1.0	1.0	1.0	1.0	PSMD1
5716	1.0	1.0	-3.5	-3.4	PSMD10
5719	1.0	-2.5	1.0	-2.7	PSMD13
5708	-2.7	-5.2	1.0	1.0	PSMD2
5711	1.0	1.0	2.1	1.0	PSMD5
23198	1.0	1.8	1.0	1.0	PSME4
9491	2.0	1.0	3.3	1.0	PSMF1
56984	1.0	1.0	1.0	1.0	PSMG2
170679	1.0	1.0	1.0	1.0	PSORS1C1
55269	1.0	-4.5	-5.3	1.0	PSPC1
5623	1.0	1.0	1.0	-1.6	PSPN
84722	1.0	1.0	1.0	1.0	PSRC1
118672	1.0	1.0	-1.7	1.0	PSTK
9051	1.0	1.0	1.0	1.0	PSTPIP1
9050	1.0	1.0	1.0	1.0	PSTPIP2
9991	1.0	1.0	4.7	1.0	PTBP3
55037	1.0	1.0	-3.7	1.0	PTCD3
5727	1.0	1.0	1.0	1.0	PTCH1
8643	1.0	1.0	1.0	1.0	PTCH2
57540	1.7	1.0	1.0	1.0	PTCHD2
171558	3.2	-5.8	-7.4	1.0	PTCRA
9791	1.0	1.0	1.0	1.0	PTDSS1
256297	-1.9	-3.2	-6.3	-5.2	PTF1A
5731	1.0	1.0	2.9	1.0	PTGER1
5737	1.0	5.8	3.5	1.0	PTGFR
22949	1.0	1.0	1.0	-2.9	PTGR1
145482	1.0	2.7	1.0	2.7	PTGR2
5744	1.0	1.0	1.0	1.0	PTHLH
2185	1.0	1.0	1.0	1.0	PTK2B

5754	1.0	1.0	1.8	1.0	PTK7
5757	1.0	-6.2	-2.4	1.0	PTMA
7803	1.0	1.0	1.0	-2.6	PTP4A1
201562	1.0	1.0	1.0	-3.6	PTPLB
5781	1.0	1.0	1.0	-4.0	PTPN11
5784	1.5	1.0	1.0	1.0	PTPN14
26469	1.0	1.0	1.0	1.0	PTPN18
26095	1.6	1.0	1.0	1.0	PTPN20B
5775	-2.0	-6.3	1.0	1.0	PTPN4
5788	1.0	-2.6	4.8	9.0	PTPRC
5792	1.0	5.9	4.0	4.4	PTPRF
5793	1.0	7.0	3.7	3.1	PTPRG
5796	1.0	6.5	1.0	1.0	PTPRK
5799	1.0	1.0	-1.6	1.0	PTPRN2
5800	2.2	1.0	1.0	1.0	PTPRO
5802	-3.3	1.0	1.0	1.0	PTPRS
10076	-18.8	1.0	4.2	1.0	PTPRU
284119	1.7	-4.3	-4.4	1.0	PTRF
138428	1.0	1.0	1.0	1.0	PTRH1
51651	1.0	1.0	1.0	1.0	PTRH2
26255	1.0	5.0	1.0	1.0	PTTG3P
5806	1.0	4.7	3.5	2.6	PTX3
22827	1.0	6.6	1.0	1.0	PUF60
23369	4.1	1.0	1.0	1.0	PUM2
29942	1.0	2.1	2.4	2.4	PURG
150962	-2.1	-2.1	-5.0	1.0	PUS10
83480	1.0	1.0	1.0	1.0	PUS3
5816	1.0	1.0	1.0	1.0	PVALB
5817	1.0	1.0	1.0	1.0	PVR
79037	2.6	1.0	1.0	1.0	PVRIG
5818	1.0	1.0	1.0	-3.5	PVRL1
25945	1.0	-7.7	1.0	1.0	PVRL3
81607	1.0	1.0	3.3	2.8	PVRL4
5822	1.0	1.0	1.0	1.0	PWP2
5827	1.0	1.0	3.7	1.0	PXMP2
11264	1.0	1.0	-3.4	-3.7	PXMP4
5829	2.1	1.0	1.0	1.0	PXN
29108	1.0	1.0	1.0	1.0	PYCARD
5831	-29.1	1.0	1.0	1.0	PYCR1
149628	1.0	1.0	1.0	1.0	PYHIN1
84795	5.8	1.0	1.0	1.0	PYROXD2
23615	2.1	1.0	1.0	1.0	PYY2
9444	-2.1	4.1	1.0	-2.9	QKI
25797	1.0	1.0	1.0	1.0	QPCT
55278	-2.7	1.0	1.0	1.0	QRSL1
5768	-3.5	1.0	1.0	1.0	QSOX1
23518	1.0	-4.0	1.0	1.0	R3HDM1
22864	2.6	1.0	1.0	1.0	R3HDM2

140902	1.0	1.0	1.0	1.0	R3HDML
10890	1.0	2.7	1.0	1.0	RAB10
80223	1.0	3.2	1.0	1.0	RAB11FIP1
201475	1.0	1.0	1.0	1.0	RAB12
376267	-3.2	-1.9	1.0	1.0	RAB15
81876	1.0	1.0	1.0	1.0	RAB1B
55647	1.0	1.0	1.0	1.0	RAB20
51715	1.0	1.0	1.0	1.0	RAB23
9364	4.4	-5.6	-4.8	1.0	RAB28
27314	1.0	5.3	1.0	5.5	RAB30
11031	1.0	1.0	1.0	1.0	RAB31
83452	-2.3	1.0	-4.3	1.0	RAB33B
326624	1.0	1.0	1.0	1.0	RAB37
115827	1.0	-3.4	1.0	1.0	RAB3C
9545	1.0	1.0	1.0	1.0	RAB3D
53916	1.0	1.0	1.0	1.0	RAB4B
5868	3.0	2.5	1.0	1.0	RAB5A
7879	2.5	1.0	1.0	1.0	RAB7A
51762	-13.6	1.0	-9.4	1.0	RAB8B
79874	1.6	1.0	1.0	1.0	RABEP2
9910	1.0	1.0	-2.6	4.7	RABGAP1L
11159	-2.2	2.9	2.6	2.6	RABL2A
5880	2.8	1.0	1.0	3.7	RAC2
5810	1.0	1.0	1.0	1.0	RAD1
10111	1.7	-3.0	1.0	1.0	RAD50
5890	1.0	1.0	1.0	1.0	RAD51B
5893	1.0	-4.1	1.0	1.0	RAD52
25788	1.0	2.2	2.3	1.0	RAD54B
5883	1.5	4.6	5.4	4.3	RAD9A
144715	1.0	1.0	1.0	1.0	RAD9B
8480	1.0	3.6	1.0	2.7	RAE1
135250	-4.3	1.0	1.0	-7.1	RAET1E
5894	1.0	1.0	2.5	2.8	RAF1
5897	1.0	1.0	6.7	1.0	RAG2
10743	1.0	1.0	1.0	2.7	RAI1
26064	1.0	1.0	1.0	1.0	RAI14
10742	1.0	1.0	2.5	1.0	RAI2
10928	1.0	3.1	4.5	-2.6	RALBP1
253959	-2.3	1.0	1.0	4.4	RALGAPA1
57186	1.0	1.0	1.0	1.0	RALGAPA2
5900	1.0	-5.7	-5.4	1.0	RALGDS
9649	1.0	2.8	3.0	3.4	RALGPS1
10268	1.0	2.3	1.7	1.0	RAMP3
5901	1.0	1.0	1.0	1.0	RAN
26953	1.0	1.0	4.6	1.0	RANBP6
29098	1.0	2.3	1.0	1.0	RANGRF
5906	-2.3	1.0	1.0	1.0	RAP1A
5908	1.0	1.0	1.0	1.0	RAP1B

5910	1.0	1.0	2.8	1.0	RAP1GDS1
5912	1.0	1.0	1.0	2.8	RAP2B
2889	1.0	3.7	7.4	1.0	RAPGEF1
9693	24.1	1.0	-7.5	1.0	RAPGEF2
5914	1.0	1.0	1.0	1.0	RARA
5916	-1.7	1.0	-3.2	1.0	RARG
5920	1.0	1.0	1.0	1.0	RARRES3
57038	1.0	-2.5	1.0	-2.4	RARS2
22821	1.7	1.0	1.0	1.0	RASA3
9462	1.0	1.0	1.0	1.0	RASAL2
64926	1.0	3.4	1.0	1.0	RASAL3
51655	1.0	6.4	4.5	7.0	RASD1
221002	1.0	1.0	1.0	1.0	RASGEF1A
5923	1.0	1.0	1.0	-4.0	RASGRF1
10235	1.0	-2.8	2.8	1.0	RASGRP2
25780	2.4	1.0	1.0	1.0	RASGRP3
115727	-4.0	1.0	1.0	1.0	RASGRP4
91608	1.0	1.0	1.0	-3.2	RASL10B
387496	1.0	1.0	1.0	1.0	RASL11A
83593	1.0	1.0	1.0	1.0	RASSF5
166824	1.0	-3.6	1.0	1.0	RASSF6
11228	1.0	1.0	1.0	-3.4	RASSF8
55225	1.0	-5.4	-7.7	1.0	RAVER2
30062	-1.6	1.0	-4.3	1.0	RAX
5925	-3.3	1.0	1.0	-2.2	RB1
57786	-1.9	1.0	-5.1	-3.7	RBAK
5928	-3.5	1.0	1.0	1.0	RBBP4
5929	1.0	1.0	-5.6	1.0	RBBP5
5931	1.0	1.0	1.0	1.0	RBBP7
5932	1.0	1.0	1.0	1.0	RBBP8
10741	-2.2	1.0	1.0	1.0	RBBP9
10616	3.4	3.9	1.0	1.0	RBCK1
54715	2.3	5.2	1.0	1.0	RBFOX1
23543	1.0	3.0	2.5	2.4	RBFOX2
8241	13.3	1.0	1.0	1.0	RBM10
10432	1.0	1.0	-2.4	-2.4	RBM14
29890	-6.0	1.0	3.6	1.0	RBM15B
9904	1.0	2.2	1.0	1.0	RBM19
282996	1.0	-2.8	1.0	1.0	RBM20
58517	1.0	1.0	1.0	1.0	RBM25
55131	-4.7	6.2	1.0	1.0	RBM28
155435	1.0	1.0	1.0	1.0	RBM33
55285	2.8	1.0	1.0	-2.1	RBM41
79171	1.0	1.0	1.0	1.0	RBM42
9939	1.0	1.0	1.0	1.0	RBM8A
27303	1.0	-3.0	1.0	1.0	RBMS3
27316	1.0	1.0	-4.2	1.0	RBMX
11317	1.0	1.0	-4.4	1.0	RBPJL

11030	1.0	4.5	1.8	1.0	RBPMS
9978	1.0	1.0	5.8	1.0	RBX1
149041	1.0	3.1	1.0	1.0	RC3H1
1102	1.0	1.0	1.0	1.0	RCBTB2
91433	1.0	1.0	1.9	2.4	RCCD1
9986	1.0	-2.7	1.0	1.0	RCE1
10171	1.0	1.0	1.0	1.0	RCL1
23186	1.0	2.4	1.0	1.0	RCOR1
5957	1.0	-3.0	1.0	-2.9	RCVRN
51109	1.0	1.0	-4.5	-6.0	RDH11
8608	1.0	1.0	1.0	1.0	RDH16
5959	1.0	1.0	1.0	1.0	RDH5
50700	1.0	1.0	1.0	4.3	RDH8
9985	1.0	1.0	1.0	1.0	REC8
9401	1.0	1.0	1.0	1.0	RECQL4
9400	1.0	1.0	6.4	1.0	RECQL5
65055	1.0	1.0	1.0	1.0	REEP1
92840	-12.0	-3.6	1.0	-3.7	REEP6
5068	1.0	1.0	1.0	1.0	REG3A
83998	1.0	1.0	1.0	1.0	REG4
5970	1.0	1.6	1.6	1.9	RELA
5971	1.0	1.0	1.0	3.8	RELB
768211	1.0	1.0	1.0	1.0	RELL1
5972	4.8	1.0	-2.7	-2.8	REN
5973	1.0	1.0	3.2	1.0	RENBP
29803	5.5	1.0	1.0	1.0	REPIN1
85021	3.0	3.1	1.0	1.0	REPS1
11079	1.0	-3.0	1.0	1.0	RER1
473	1.0	1.0	1.0	1.0	RERE
56729	1.0	-2.9	1.0	1.0	RETN
54884	1.0	1.0	1.0	1.0	RETSAT
5980	1.0	1.0	-3.1	-3.0	REV3L
254958	1.0	1.0	1.0	1.0	REXO1L1
5981	-1.6	1.7	1.0	1.0	RFC1
5984	1.0	1.0	-4.8	1.0	RFC4
5986	-1.7	1.0	1.0	1.0	RFNG
91869	1.7	-2.8	1.0	1.0	RFT1
54931	1.0	1.0	1.0	1.0	RG9MTD1
23179	1.0	-2.8	-2.6	-2.4	RGL1
9104	-5.5	1.0	-2.8	-2.8	RGN
5995	1.0	1.0	1.0	1.0	RGR
6001	1.0	1.0	1.0	1.0	RGS10
6002	1.0	2.6	1.0	1.0	RGS12
10636	-4.2	1.0	2.5	1.0	RGS14
6004	1.0	1.0	2.7	1.0	RGS16
10287	4.1	1.0	1.0	1.0	RGS19
9628	1.0	1.0	1.0	1.0	RGS6
64285	1.0	-4.0	1.0	1.0	RHBDF1

9028	1.0	1.0	1.0	-3.1	RHBDL1
6007	1.0	1.0	-6.1	1.0	RHD
6009	1.0	1.0	1.0	1.0	RHEB
6010	-1.8	1.0	1.0	4.7	RHO
387	1.0	4.0	1.0	1.0	RHOA
388	1.0	1.0	1.0	1.0	RHOB
23221	1.0	1.0	2.0	1.0	RHOBTB2
389	1.0	1.0	-2.7	1.0	RHOC
399	1.0	1.5	1.0	1.0	RHOH
23433	1.0	2.5	1.0	1.0	RHOQ
58480	1.0	1.0	-7.3	1.0	RHOU
171177	1.0	3.2	1.0	1.0	RHOV
85415	1.0	1.0	1.0	1.0	RHPN2
60626	-8.6	1.0	1.0	1.0	RIC8A
55188	1.0	1.0	1.0	1.0	RIC8B
83547	1.0	1.0	-5.6	1.0	RILP
23504	1.0	1.0	-3.4	1.0	RIMBP2
85376	1.0	1.0	2.2	1.0	RIMBP3
284716	1.0	1.0	1.0	1.0	RIMKLA
9783	1.0	1.0	1.0	1.0	RIMS3
60561	1.0	1.0	-1.8	1.0	RINT1
55781	1.0	1.0	1.0	1.0	RIOK2
8780	-1.8	2.1	3.6	2.8	RIOK3
54101	3.7	1.0	1.0	4.2	RIPK4
6016	-1.7	1.0	1.0	-9.0	RIT1
6017	1.0	1.0	1.7	2.9	RLBP1
6018	1.0	4.9	5.9	3.2	RLF
51132	1.0	1.0	1.0	1.0	RLIM
80010	1.0	1.0	1.0	1.0	RMI1
116028	-9.8	1.0	1.0	1.0	RMI2
6036	1.6	-2.8	1.0	-4.1	RNASE2
84659	-12.3	1.0	1.0	1.0	RNASE7
8635	1.0	1.0	1.0	1.0	RNASET2
390	1.0	3.2	3.5	3.1	RND3
7732	1.0	1.0	1.0	1.0	RNF112
27246	1.0	1.0	1.0	1.0	RNF115
55658	2.3	1.0	-2.5	1.0	RNF126
9604	-9.5	1.0	1.0	1.0	RNF14
153830	-2.3	2.5	1.0	1.0	RNF145
81847	1.0	1.0	1.0	-2.9	RNF146
284996	1.0	2.0	1.0	1.0	RNF149
26001	1.0	1.0	1.0	4.8	RNF167
254225	1.0	1.0	1.0	1.0	RNF169
81790	1.0	1.0	1.0	1.0	RNF170
149603	1.0	1.0	1.0	1.0	RNF187
25897	1.7	2.8	2.6	1.0	RNF19A
285498	1.0	1.0	2.0	1.0	RNF212
57674	1.0	1.0	1.0	1.0	RNF213

200312	1.0	1.0	-4.1	1.0	RNF215
54476	1.0	1.0	1.0	1.0	RNF216
441191	4.9	5.3	5.9	4.8	RNF216P1
79596	1.0	1.0	2.6	1.0	RNF219
64320	-3.4	1.0	-3.6	-4.0	RNF25
140545	-1.9	1.0	1.0	-2.5	RNF32
80196	1.0	2.2	1.0	1.0	RNF34
152006	2.4	3.5	1.0	1.0	RNF38
9810	1.0	1.0	1.0	1.0	RNF40
6048	1.0	16.8	1.0	1.0	RNF5
9616	1.0	-2.5	-2.5	-2.5	RNF7
9025	1.0	1.0	2.2	1.0	RNF8
55178	1.0	1.0	1.0	1.5	RNMTL1
6051	1.9	2.0	1.0	1.0	RNPEP
267010	1.0	1.7	1.0	1.0	RNU12
6092	1.0	1.0	1.0	1.0	ROBO2
79641	1.0	-4.3	1.0	1.0	ROGDI
6094	1.0	1.0	-2.5	1.0	ROM1
54763	1.0	1.0	1.0	1.0	ROPN1
83853	1.0	1.0	-2.2	1.0	ROPN1L
4919	1.0	1.0	1.0	1.0	ROR1
4920	1.0	1.0	1.0	3.2	ROR2
6101	1.0	1.0	1.0	1.0	RP1
94137	1.0	1.0	1.0	-7.3	RP1L1
6102	1.0	1.0	1.0	2.6	RP2
441212	-2.0	1.0	1.0	-3.9	RP9P
6117	1.0	1.0	5.7	1.0	RPA1
6119	1.0	1.0	6.0	1.0	RPA3
6103	1.0	-5.0	-4.0	1.0	RPGR
23322	1.0	-2.6	1.0	1.0	RPGRIP1L
9501	1.0	1.0	1.0	1.0	RPH3AL
22934	1.0	1.0	2.9	1.0	RPIA
6137	1.0	1.0	1.0	1.0	RPL13
645683	4.0	1.0	1.0	1.0	RPL13AP3
9045	1.6	1.0	-3.8	1.0	RPL14
6138	1.6	1.0	-3.3	1.0	RPL15
6144	1.0	1.0	-5.2	1.0	RPL21
402176	1.0	1.0	1.0	-10.9	RPL21P44
6146	1.0	8.6	2.1	2.8	RPL22
9349	1.0	3.0	2.7	2.6	RPL23
56969	1.0	1.0	1.0	1.0	RPL23AP32
644128	1.0	1.0	1.0	1.0	RPL23AP53
118433	1.0	1.0	2.9	1.0	RPL23AP7
6152	1.0	-3.5	-2.9	-3.9	RPL24
6154	1.0	1.0	-6.1	1.0	RPL26
6159	2.0	1.0	-4.9	1.0	RPL29
6160	2.0	1.0	-2.7	1.0	RPL31
132241	1.0	1.0	1.0	1.0	RPL32P3

6170	1.0	1.0	1.0	1.0	RPL39
6125	5.0	1.0	1.0	1.0	RPL5
285855	1.0	-2.6	1.0	1.0	RPL7L1
10556	1.0	1.0	1.0	1.0	RPP30
10557	9.8	1.0	1.0	1.0	RPP38
55197	-2.1	1.0	-3.3	1.0	RPRD1A
58490	1.0	1.0	2.6	1.0	RPRD1B
6206	4.1	1.0	2.8	2.7	RPS12
6208	1.0	-2.6	-2.6	-6.1	RPS14
6210	1.0	1.0	1.0	1.0	RPS15A
6218	1.0	1.0	1.0	1.0	RPS17
6222	1.0	-2.5	-2.6	-2.6	RPS18
91582	1.0	1.0	2.2	1.0	RPS19BP1
6227	1.0	1.0	1.0	1.9	RPS21
6228	1.0	1.0	-3.5	1.0	RPS23
6231	1.0	-4.0	-4.4	1.0	RPS26
6232	1.9	2.7	2.6	2.6	RPS27
6191	1.0	1.0	1.0	1.0	RPS4X
6192	1.0	1.0	1.0	1.0	RPS4Y1
140032	1.0	1.0	1.0	1.0	RPS4Y2
6194	2.0	-3.1	-3.2	-3.2	RPS6
6195	1.0	1.0	1.0	1.0	RPS6KA1
6196	-1.6	-5.4	1.0	1.0	RPS6KA2
8986	1.0	1.0	1.0	1.0	RPS6KA4
9252	1.0	1.0	1.0	1.0	RPS6KA5
6198	2.4	-2.7	-2.5	-2.7	RPS6KB1
6199	1.0	-2.4	1.0	-2.9	RPS6KB2
83694	-3.0	1.0	1.0	1.0	RPS6KL1
645884	1.8	1.0	-3.6	1.0	RPS7P5
6202	1.0	1.0	1.0	1.0	RPS8
6203	1.0	1.0	1.0	1.0	RPS9
27079	1.0	1.0	1.0	1.0	RPUSD2
285367	-1.6	1.0	1.0	1.0	RPUSD3
22800	1.0	1.0	1.0	1.0	RRAS2
6238	1.0	-4.1	1.0	1.0	RRBP1
6240	1.0	1.0	4.6	1.0	RRM1
6241	1.0	1.0	1.0	1.0	RRM2
50484	1.0	1.0	1.0	-5.9	RRM2B
54700	1.0	1.0	1.0	1.0	RRN3
23223	1.0	1.0	1.0	1.0	RRP12
51018	-2.3	1.0	1.0	1.0	RRP15
91543	1.0	1.0	1.0	1.0	RSAD2
79363	1.0	1.0	-2.8	2.6	RSG1
51187	1.0	1.0	1.0	1.0	RSL24D1
83861	1.0	1.0	5.7	5.0	RSPH3
221421	1.0	1.0	1.0	3.1	RSPH9
6251	-18.0	1.0	1.0	1.0	RSU1
83546	2.8	1.0	5.0	1.0	RTBDN

6242	1.0	1.0	1.0	1.0	RTKN
6253	-4.9	-3.2	1.0	-3.1	RTN2
146760	1.0	1.0	1.0	1.0	RTN4RL1
132112	-3.5	1.0	5.2	1.0	RTP1
80230	1.0	1.0	2.4	1.0	RUFY1
55680	1.0	-3.6	1.0	1.0	RUFY2
10900	-3.5	1.0	-3.0	1.0	RUNDC3A
861	1.0	2.8	-2.4	-2.4	RUNX1
860	1.0	1.0	-7.9	-5.2	RUNX2
864	1.0	1.0	1.0	1.0	RUNX3
51389	1.0	1.0	1.0	1.0	RWDD1
122042	1.0	1.0	2.4	1.0	RXFP2
23429	1.0	1.0	1.0	1.0	RYBP
6282	1.0	1.0	1.0	1.0	S100A11
6283	1.0	1.0	-2.5	1.0	S100A12
6273	1.0	1.0	1.0	6.0	S100A2
6275	1.0	1.0	1.0	1.0	S100A4
338324	1.0	1.0	1.0	1.0	S100A7A
795	1.0	1.0	-6.6	1.0	S100G
9294	-1.8	1.0	1.0	1.0	S1PR2
1903	2.2	1.0	1.0	1.0	S1PR3
8698	1.0	1.0	6.7	1.0	S1PR4
6290	1.0	1.0	1.0	1.0	SAA3P
113174	-1.5	1.0	1.0	1.0	SAAL1
26278	2.2	1.0	1.0	3.4	SACS
6294	1.0	1.0	7.8	6.2	SAFB
6297	1.0	3.8	2.1	1.0	SALL2
57167	1.0	1.0	1.0	1.0	SALL4
148418	1.0	1.0	1.0	1.0	SAMD13
201191	1.0	-3.3	1.0	1.0	SAMD14
23034	1.9	1.0	-2.6	-3.3	SAMD4A
55095	1.9	1.0	1.0	1.0	SAMD4B
389432	1.0	1.0	1.0	1.0	SAMD5
142891	1.0	1.0	1.0	1.0	SAMD8
54809	1.0	1.0	1.0	-5.7	SAMD9
79595	1.0	1.0	1.0	1.0	SAP130
8819	1.0	1.0	1.0	1.0	SAP30
29115	1.0	1.0	1.0	1.0	SAP30BP
51128	1.0	1.0	1.0	1.0	SAR1B
1757	1.7	1.0	2.5	1.0	SARDH
23098	-2.5	1.0	5.5	1.0	SARM1
54938	1.0	1.0	1.0	1.0	SARS2
9733	1.0	1.0	1.0	1.0	SART3
23328	1.0	1.0	1.0	1.0	SASH1
6303	1.0	1.0	1.0	-2.6	SAT1
6304	1.0	2.1	3.5	1.0	SATB1
6305	1.0	1.0	1.0	1.0	SBF1
6309	-3.6	-5.0	1.0	-5.3	SC5DL

57466	-2.9	-4.2	1.0	1.0	SCAF4
286205	2.3	1.0	1.0	1.0	SCAI
9522	1.0	3.0	3.5	4.1	SCAMP1
10067	1.0	1.0	1.0	1.0	SCAMP3
54581	-18.5	3.4	1.0	1.0	SCAND2
114821	-4.9	1.0	1.0	1.0	SCAND3
49855	1.0	1.0	1.0	1.0	SCAPER
51435	1.9	-3.2	1.0	1.0	SCARA3
949	2.3	1.0	1.0	1.0	SCARB1
91179	1.0	1.0	5.4	1.0	SCARF2
51097	4.7	1.0	1.0	1.0	SCCPDH
79966	1.0	1.0	2.9	1.8	SCD5
23256	1.0	1.0	1.0	1.0	SCFD1
29106	1.0	1.0	7.0	1.0	SCG3
7356	-2.5	1.0	1.0	1.0	SCGB1A1
4246	1.0	1.0	1.0	-3.3	SCGB2A1
10590	1.0	1.0	1.0	1.0	SCGN
132320	1.6	1.0	2.9	1.0	SCLT1
51540	1.0	1.0	1.0	1.0	SCLY
6323	1.0	1.0	1.0	1.0	SCN1A
6326	-4.0	1.0	-4.5	1.0	SCN2A
6329	1.0	4.0	4.7	6.6	SCN4A
6330	1.0	1.0	1.0	1.0	SCN4B
6331	1.0	1.0	1.0	1.0	SCN5A
6332	-3.9	1.0	1.0	1.0	SCN7A
6334	1.0	-3.9	-4.3	1.0	SCN8A
6335	2.0	-2.7	1.0	-3.7	SCN9A
6337	2.3	-2.8	1.0	-3.8	SCNN1A
6338	1.0	-4.3	-8.5	1.0	SCNN1B
6341	1.0	1.0	2.3	1.0	SCO1
9997	-2.0	1.0	1.0	-3.8	SCO2
59342	1.0	1.0	1.0	1.0	SCPEP1
6344	1.0	1.0	1.0	1.0	SCTR
222663	1.0	1.0	1.0	1.0	SCUBE3
55153	-2.0	1.0	-4.5	-8.0	SDAD1
6382	1.0	2.5	1.0	1.0	SDC1
6383	1.0	1.0	-2.1	1.0	SDC2
9672	1.0	1.0	1.0	1.6	SDC3
6386	-5.9	1.0	-3.8	1.0	SDCBP
27111	1.0	1.0	3.2	1.0	SDCBP2
10807	1.0	1.0	1.0	1.0	SDCCAG3
23753	1.7	1.0	1.0	1.0	SDF2L1
51150	1.0	1.0	-6.6	1.0	SDF4
6389	1.0	1.0	1.0	1.0	SDHA
54949	1.0	3.4	1.0	1.0	SDHAF2
6390	23.4	1.0	1.0	5.5	SDHB
6391	1.0	1.0	1.0	1.0	SDHC
113675	1.0	3.9	1.0	1.0	SDSL

6396	1.0	-1.9	-3.1	1.0	SEC13
6397	1.0	1.0	1.0	1.0	SEC14L1
266629	-5.5	-5.2	-3.5	-5.8	SEC14L3
284904	1.0	1.0	1.0	2.1	SEC14L4
89866	4.6	1.0	-1.6	1.0	SEC16B
10802	1.0	5.1	1.0	1.0	SEC24A
25956	1.0	1.0	1.0	1.0	SEC31B
55176	1.0	1.0	1.0	3.2	SEC61A2
10952	1.0	1.0	1.0	-2.8	SEC61B
7095	1.0	1.0	-2.8	1.0	SEC62
9728	4.7	1.0	2.0	1.0	SECISBP2L
6398	1.0	1.0	1.0	-6.7	SECTM1
6400	1.0	1.0	-11.8	1.0	SEL1L
23231	1.0	1.0	1.0	1.0	SEL1L3
6403	1.0	1.0	1.0	1.0	SELP
65260	1.0	1.0	2.4	1.0	SELRC1
51714	1.0	-2.5	1.0	1.0	SELT
348303	1.0	1.7	1.0	1.0	SELV
6405	1.0	1.0	1.5	1.0	SEMA3F
64218	1.6	1.0	1.0	-1.8	SEMA4A
54910	1.6	1.0	1.0	1.0	SEMA4C
10507	1.5	1.0	-2.0	1.0	SEMA4D
54437	1.0	1.0	1.0	1.0	SEMA5B
10501	2.2	1.0	-2.1	1.0	SEMA6B
8482	1.0	1.0	1.0	1.0	SEMA7A
6407	1.0	-3.8	1.0	1.0	SEMG2
26054	2.4	-2.5	1.0	1.0	SENP6
57337	1.0	1.0	2.4	1.0	SENP7
22929	1.0	1.0	1.0	1.0	SEPHS1
285961	1.0	1.0	1.0	1.0	SEPT7L
51734	1.0	1.0	1.0	1.0	SEPX1
84947	1.0	1.0	4.5	1.0	SERAC1
26135	1.0	1.0	1.0	-9.0	SERBP1
728492	1.0	1.0	1.0	1.0	SERF1B
10169	-4.7	1.0	1.0	1.0	SERF2
26297	1.0	1.0	-8.4	-3.3	SERGEF
253190	1.0	2.0	1.0	1.8	SERHL2
10955	1.0	1.0	1.0	-1.7	SERINC3
619189	1.0	1.0	1.0	1.0	SERINC4
256987	1.0	1.0	1.0	1.0	SERINC5
387923	1.0	1.0	-8.5	-3.8	SERP2
5265	1.0	1.0	2.4	1.0	SERPINA1
256394	1.0	1.0	-2.4	1.0	SERPINA11
866	1.0	-3.9	-3.8	1.0	SERPINA6
1992	1.6	1.0	1.0	1.0	SERPINB1
5273	1.0	-3.4	-2.3	1.0	SERPINB10
89778	1.0	1.0	-2.9	1.0	SERPINB11
89777	1.0	-2.4	-2.1	1.0	SERPINB12

5275	1.0	3.3	4.6	1.0	SERPINB13
6317	1.0	1.0	1.8	1.0	SERPINB3
5268	1.6	4.0	1.0	5.9	SERPINB5
5272	1.0	1.0	1.0	3.6	SERPINB9
5054	1.0	1.0	1.0	2.4	SERPINE1
5270	1.0	1.0	4.7	6.1	SERPINE2
647174	1.0	1.0	1.0	1.0	SERPINE3
710	1.0	-1.7	-2.3	-2.4	SERPING1
29950	1.0	1.0	1.0	1.0	SERTAD1
26040	1.0	1.0	2.3	1.0	SETBP1
29072	-1.6	2.7	3.0	1.0	SETD2
84193	1.0	-3.6	-20.3	1.0	SETD3
9869	1.0	4.9	2.5	1.0	SETDB1
83852	-15.8	1.0	3.4	1.0	SETDB2
124925	1.0	1.0	1.0	1.0	SEZ6
23544	1.0	1.0	1.0	1.0	SEZ6L
10946	1.0	1.0	1.0	1.0	SF3A3
23451	1.0	1.0	1.0	-3.0	SF3B1
10262	14.6	1.0	1.0	6.5	SF3B4
9814	3.5	1.0	6.6	1.0	SFI1
57713	1.0	1.0	-3.7	1.0	SFMBT2
6422	1.0	1.0	3.2	2.9	SFRP1
6423	1.0	1.0	-2.7	1.0	SFRP2
113402	1.0	3.5	3.0	1.0	SFT2D1
6439	3.9	1.0	-2.4	-2.9	SFTPB
6441	1.6	1.0	1.0	1.0	SFTPD
81855	15.6	1.0	1.0	1.0	SFXN3
6444	-4.6	2.8	-4.0	2.7	SGCD
8910	1.0	1.0	1.0	-4.9	SGCE
84251	-5.0	1.0	1.0	1.0	SGIP1
84197	1.0	1.0	1.0	1.0	SGK196
124923	1.0	1.0	2.8	1.0	SGK494
151648	1.0	1.7	1.0	1.0	SGOL1
81537	-2.5	1.0	-1.9	1.0	SGPP1
130367	1.0	1.0	4.1	1.0	SGPP2
9905	1.0	2.6	1.0	1.0	SGSM2
10603	2.9	-2.7	1.0	1.0	SH2B2
117157	1.0	1.0	1.0	1.0	SH2D1B
387694	1.0	1.0	1.0	1.0	SH2D4B
6452	1.8	1.0	1.0	-4.2	SH3BP2
23677	-2.0	2.8	1.8	3.0	SH3BP4
80851	1.0	1.0	1.0	1.0	SH3BP5L
6457	1.0	1.0	1.0	1.0	SH3GL3
56904	-1.5	1.0	1.0	-1.9	SH3GLB2
30011	1.0	-4.5	4.6	-6.2	SH3KBP1
9644	1.0	1.0	-1.7	1.0	SH3PXD2A
285590	1.0	1.0	1.0	1.0	SH3PXD2B
153769	1.0	1.0	-3.4	1.0	SH3RF2

344558	1.0	1.0	9.0	1.0	SH3RF3
54436	1.0	1.0	1.8	1.0	SH3TC1
79628	1.0	1.0	1.0	1.0	SH3TC2
50944	1.0	4.8	1.0	2.9	SHANK1
22941	1.0	1.0	1.0	5.5	SHANK2
81858	1.0	1.0	1.0	1.0	SHARPIN
6461	1.0	1.0	1.0	-3.6	SHB
6462	-5.5	1.0	1.0	1.0	SHBG
81626	1.0	1.0	1.0	1.0	SHCBP1L
149345	1.7	1.0	1.0	-2.8	SHISA4
51246	1.0	1.0	1.0	1.0	SHISA5
388336	1.0	1.0	1.0	1.0	SHISA6
6470	2.1	1.0	1.0	1.0	SHMT1
6472	1.0	1.0	5.0	1.0	SHMT2
8036	1.0	1.0	1.0	1.0	SHOC2
6474	1.0	-4.0	-3.8	-3.8	SHOX2
23729	1.0	1.0	1.0	1.0	SHPK
134549	1.0	1.0	-2.8	3.4	SHROOM1
57619	1.0	1.0	-5.8	-2.7	SHROOM3
57477	-1.7	1.0	1.0	1.0	SHROOM4
6476	-12.8	1.0	1.0	1.0	SI
6477	-3.9	1.0	1.0	1.0	SIAH1
6478	1.9	1.0	1.8	1.0	SIAH2
54847	1.0	5.0	1.0	1.0	SIDT1
51092	1.0	1.0	4.6	5.1	SIDT2
946	1.0	1.0	1.0	1.0	SIGLEC6
27181	1.0	1.9	1.0	1.0	SIGLEC8
284367	1.0	1.0	1.0	1.0	SIGLECP3
80143	1.0	15.2	1.0	1.0	SIKE1
64374	1.0	1.0	1.0	1.0	SIL1
57568	1.0	1.0	1.0	1.0	SIPA1L2
10326	1.0	1.0	-4.3	-7.8	SIRPB1
284759	2.1	1.0	1.0	3.8	SIRPB2
128646	12.4	-4.3	1.0	-5.4	SIRPD
55423	1.0	1.0	1.0	-2.1	SIRPG
23408	1.0	1.0	1.0	1.0	SIRT5
10572	1.0	1.0	1.0	4.0	SIVA1
6496	1.0	1.0	1.0	1.0	SIX3
4990	1.0	2.9	2.7	1.0	SIX6
348235	1.0	1.0	1.0	1.0	SKA2
8631	1.0	1.0	1.0	1.0	SKAP1
6499	2.2	1.0	1.0	1.0	SKIV2L
6500	1.0	-2.4	-2.5	1.0	SKP1
6502	1.0	2.3	7.1	1.0	SKP2
6503	1.0	2.7	1.6	1.0	SLA
6504	1.0	1.0	2.5	1.0	SLAMF1
114836	1.0	1.0	1.0	1.0	SLAMF6
7884	1.0	1.0	1.0	1.0	SLBP

6560	1.0	1.0	3.0	1.0	SLC12A4
57468	1.0	1.0	1.0	1.0	SLC12A5
10723	1.0	3.6	3.1	1.0	SLC12A7
56996	2.1	1.0	1.0	1.0	SLC12A9
9058	-2.7	-3.4	-3.2	1.0	SLC13A2
284111	1.0	5.6	5.9	1.0	SLC13A5
6563	1.0	1.0	-10.8	1.0	SLC14A1
117247	1.0	-2.4	1.0	1.0	SLC16A10
9121	1.0	1.0	2.8	1.0	SLC16A5
9194	-1.9	1.0	1.0	-6.2	SLC16A7
10246	1.0	1.0	1.0	1.0	SLC17A2
10786	1.0	1.0	1.0	9.9	SLC17A3
26503	1.0	3.4	-3.4	1.0	SLC17A5
63910	1.0	1.0	-14.5	-9.7	SLC17A9
6570	1.0	3.8	1.0	1.0	SLC18A1
6506	1.0	1.0	-7.9	1.0	SLC1A2
6580	1.0	1.0	1.0	1.0	SLC22A1
387775	1.0	4.8	3.7	2.9	SLC22A10
116085	1.0	1.0	1.0	-4.5	SLC22A12
55356	5.6	1.0	9.5	9.0	SLC22A15
5003	1.0	1.0	1.0	1.0	SLC22A18AS
6582	1.0	1.0	-5.5	1.0	SLC22A2
283238	1.0	1.0	4.6	4.7	SLC22A24
6583	1.0	1.0	1.0	-5.4	SLC22A4
114571	1.0	1.0	1.0	1.0	SLC22A9
9963	1.0	1.0	1.0	1.0	SLC23A1
9187	1.0	1.0	2.5	1.0	SLC24A1
25769	1.0	1.0	-5.9	-6.2	SLC24A2
123041	1.0	1.0	1.0	1.0	SLC24A4
8402	1.0	1.0	1.0	1.0	SLC25A11
10166	1.0	1.0	1.0	1.0	SLC25A15
10478	1.0	1.0	1.0	1.0	SLC25A17
788	1.0	1.9	1.0	1.0	SLC25A20
115286	1.0	1.0	1.0	1.0	SLC25A26
81034	4.8	1.0	-5.6	1.0	SLC25A32
51312	1.0	1.0	3.2	1.0	SLC25A37
284427	1.0	1.8	1.0	2.9	SLC25A41
284439	1.0	1.0	1.0	1.0	SLC25A42
284129	1.0	1.0	2.7	1.0	SLC26A11
1811	1.0	4.3	4.1	4.5	SLC26A3
376497	1.0	1.8	1.0	4.5	SLC27A1
11001	1.0	1.0	1.0	1.0	SLC27A2
11000	1.0	-4.8	1.0	1.0	SLC27A3
9154	1.0	-3.7	-3.8	1.0	SLC28A1
64078	-1.7	1.0	-6.4	1.0	SLC28A3
55315	1.5	1.0	3.0	3.6	SLC29A3
222962	1.6	3.0	1.0	1.0	SLC29A4
66035	1.0	1.0	1.0	1.0	SLC2A11

154091	1.0	1.0	2.3	1.0	SLC2A12
144195	1.0	1.0	4.1	3.3	SLC2A14
6518	1.0	1.0	1.0	1.0	SLC2A5
11182	1.0	1.0	1.0	1.0	SLC2A6
56606	1.0	1.0	1.0	1.9	SLC2A9
7779	1.0	1.0	1.0	1.0	SLC30A1
64924	1.0	-3.9	-5.0	1.0	SLC30A5
169026	1.0	1.0	1.0	1.0	SLC30A8
1317	1.0	1.0	-2.4	-2.5	SLC31A1
1318	1.0	1.0	2.9	1.0	SLC31A2
9197	1.0	1.0	1.0	1.0	SLC33A1
10568	1.0	1.0	4.8	1.0	SLC34A2
10559	5.7	1.0	1.0	3.8	SLC35A1
23443	-1.7	1.0	1.0	1.0	SLC35A3
347734	1.0	1.0	1.0	1.0	SLC35B2
55343	1.0	1.0	1.0	1.0	SLC35C1
340146	1.0	1.0	1.0	1.0	SLC35D3
9906	1.0	1.0	1.0	2.4	SLC35E2
55508	1.0	5.0	1.0	1.0	SLC35E3
339665	-1.5	-2.9	1.0	1.0	SLC35E4
222553	1.0	1.0	1.0	1.0	SLC35F1
80255	1.0	1.0	1.0	2.2	SLC35F5
285641	1.0	1.0	1.0	1.0	SLC36A3
219855	1.0	3.1	2.7	1.0	SLC37A2
124565	1.0	1.0	1.0	1.0	SLC38A10
54407	1.0	-2.7	1.0	1.0	SLC38A2
57181	1.0	1.0	1.0	-2.1	SLC39A10
283375	1.0	-4.3	-4.2	1.0	SLC39A5
64116	1.0	-3.5	-5.1	-7.4	SLC39A8
6519	1.0	1.0	1.0	1.0	SLC3A1
54946	1.6	1.0	1.0	1.0	SLC41A3
23446	2.9	1.0	1.0	-2.5	SLC44A1
57153	1.0	1.0	3.8	1.0	SLC44A2
126969	1.0	-2.9	1.0	1.0	SLC44A3
204962	1.0	1.0	1.0	1.0	SLC44A5
51151	1.0	1.0	1.0	1.0	SLC45A2
283537	1.0	1.0	1.0	1.0	SLC46A3
146802	1.0	1.0	-5.7	-6.7	SLC47A2
6521	1.0	1.0	1.0	-5.5	SLC4A1
83959	1.0	1.0	1.0	1.0	SLC4A11
22950	1.0	1.0	1.0	1.0	SLC4A1AP
6522	1.0	1.6	1.0	1.0	SLC4A2
6508	-8.4	1.0	-3.5	1.0	SLC4A3
57835	1.0	-3.5	-4.5	-5.0	SLC4A5
9497	1.0	3.0	4.7	3.4	SLC4A7
9498	1.0	3.5	1.0	1.0	SLC4A8
55974	-2.2	-4.8	1.0	1.0	SLC50A1
6523	1.0	1.0	1.0	1.9	SLC5A1

159963	1.8	1.0	1.0	1.0	SLC5A12
6526	1.0	2.5	1.0	1.0	SLC5A3
6527	1.0	1.0	1.0	1.0	SLC5A4
60482	1.0	1.0	1.0	1.0	SLC5A7
6540	-5.2	1.0	2.9	1.0	SLC6A13
55117	-2.8	1.0	1.0	1.0	SLC6A15
6532	1.6	1.0	-2.7	-2.6	SLC6A4
6533	1.6	-2.6	1.0	1.0	SLC6A6
6535	1.0	1.0	1.0	1.0	SLC6A8
84889	1.0	1.0	1.0	-2.7	SLC7A3
6545	1.0	2.8	1.0	1.0	SLC7A4
23428	1.0	2.7	5.0	4.1	SLC7A8
6546	1.6	-2.9	-3.4	-2.8	SLC8A1
6543	1.0	1.0	1.0	1.0	SLC8A2
6548	1.0	1.0	1.0	1.0	SLC9A1
285335	1.0	-2.1	-1.8	1.0	SLC9A10
9351	-1.9	1.0	1.0	1.0	SLC9A3R2
84679	1.0	1.0	1.0	1.0	SLC9A7
53919	1.0	-2.7	-3.1	1.0	SLCO1C1
28232	1.0	5.4	1.0	1.0	SLCO3A1
353189	1.0	3.8	1.0	3.9	SLCO4C1
81796	1.0	1.9	1.0	2.7	SLCO5A1
162394	1.0	-3.9	-4.4	-4.5	SLFN5
114798	1.0	-3.4	-3.6	1.0	SLITRK1
84631	1.0	-3.8	1.0	-6.3	SLITRK2
139065	1.0	1.0	-3.2	1.0	SLITRK4
26050	1.0	1.0	3.0	1.0	SLITRK5
84189	1.0	1.0	1.0	1.0	SLITRK6
9748	1.0	1.0	1.0	1.0	SLK
7871	1.0	1.0	1.0	1.0	SLMAP
51012	1.0	1.0	2.5	1.0	SLMO2
84464	1.8	1.0	1.0	1.0	SLX4
4088	1.0	1.0	2.5	1.0	SMAD3
9597	1.0	1.0	-1.5	1.0	SMAD5-AS1
4091	1.0	1.0	1.0	1.0	SMAD6
4092	1.0	1.0	1.0	5.9	SMAD7
4093	5.7	1.0	3.1	1.0	SMAD9
6594	1.0	2.7	3.1	1.0	SMARCA1
6595	1.0	1.0	1.0	1.0	SMARCA2
56916	1.0	2.3	1.0	2.1	SMARCAD1
6602	-14.6	3.3	1.0	2.6	SMARCD1
6603	1.0	1.0	4.4	1.0	SMARCD2
6605	1.0	1.0	-3.0	1.0	SMARCE1
8243	1.0	1.0	1.0	-6.2	SMC1A
10592	-2.6	1.0	-2.4	-1.6	SMC2
23137	-7.2	1.0	1.0	1.0	SMC5
23347	1.0	1.0	1.0	1.0	SMCHD1
140771	1.0	1.0	-7.5	1.0	SMCR5

54471	1.0	1.0	1.0	1.0	SMCR7L
140775	1.0	1.0	1.0	1.0	SMCR8
23049	1.0	1.0	1.0	2.5	SMG1
23381	1.0	1.0	-4.9	1.0	SMG5
56006	1.0	1.0	1.0	1.0	SMG9
6606	1.0	1.0	1.0	1.0	SMN1
6607	1.0	-5.7	-5.4	1.0	SMN2
6608	1.0	1.0	1.0	1.0	SMO
54498	1.0	1.0	1.0	1.0	SMOX
55627	1.0	2.2	1.0	1.0	SMPD4
10879	1.0	-5.4	1.0	1.0	SMR3B
6525	-2.0	-10.1	1.0	-10.6	SMTN
55234	1.0	1.0	1.0	1.0	SMU1
10322	-3.1	1.0	1.0	-6.7	SMYD5
6591	1.0	1.0	4.2	1.0	SNAI2
333929	1.0	1.0	1.0	1.0	SNAI3
8773	1.7	1.0	-3.6	1.0	SNAP23
9342	1.0	1.0	1.0	-7.6	SNAP29
116841	1.0	1.0	4.7	1.0	SNAP47
9892	2.0	1.0	1.5	1.0	SNAP91
6617	2.1	1.0	1.0	1.0	SNAPC1
6621	1.0	1.0	4.7	1.0	SNAPC4
6622	1.0	2.5	2.2	2.8	SNCA
9627	1.0	-3.9	1.0	1.0	SNCAIP
25992	1.0	1.0	1.0	1.0	SNED1
23642	1.0	1.0	1.0	1.0	SNHG1
283596	1.0	1.0	1.0	1.0	SNHG10
100093630	1.0	1.0	1.0	1.0	SNHG8
79622	2.0	-4.5	-4.5	-4.5	SNRNP25
11066	1.0	1.0	1.0	1.0	SNRNP35
9410	1.0	-3.0	1.0	1.0	SNRNP40
154007	1.0	1.0	1.0	1.0	SNRNP48
6628	1.0	-2.7	1.0	1.0	SNRPB
6631	1.0	2.7	1.0	3.2	SNRPC
6632	1.0	-2.3	1.0	1.0	SNRPD1
6634	1.0	1.0	1.0	1.0	SNRPD3
6635	1.0	1.0	3.0	1.0	SNRPE
6637	1.0	2.4	1.0	1.0	SNRPG
6638	1.0	1.0	1.0	1.0	SNRPN
6640	1.0	1.0	1.0	1.0	SNTA1
6641	1.0	1.0	1.0	1.0	SNTB1
54212	1.0	-4.2	-4.2	-5.3	SNTG1
29887	1.0	1.0	-3.8	1.0	SNX10
23161	1.6	1.0	-1.7	1.0	SNX13
57231	1.0	1.0	1.0	1.0	SNX14
399979	1.6	1.0	1.0	3.5	SNX19
90203	1.0	1.0	1.0	1.0	SNX21
83891	1.0	1.0	2.8	1.0	SNX25

92017	1.0	1.0	3.0	3.0	SNX29
8724	1.0	1.0	1.0	1.0	SNX3
58533	1.0	1.0	1.0	1.0	SNX6
8435	1.0	1.0	3.9	1.0	SOAT2
55084	1.0	1.0	1.0	1.0	SOBP
54937	1.8	1.0	-1.8	1.0	SOHLH2
6650	2.1	1.0	1.0	1.0	SOLH
6651	3.2	1.0	1.0	1.0	SON
10580	1.0	1.0	1.0	1.0	SORBS1
114815	1.0	1.0	-5.2	1.0	SORCS1
22986	1.0	1.0	1.0	1.0	SORCS3
6654	4.9	1.0	1.0	3.3	SOS1
50964	1.0	1.0	-3.3	1.0	SOST
6666	1.0	1.9	1.0	1.0	SOX12
9580	1.0	1.0	1.0	-3.0	SOX13
8403	4.5	8.8	1.0	1.0	SOX14
64321	1.0	1.0	1.0	1.0	SOX17
54345	20.2	3.2	1.0	-2.8	SOX18
347689	1.0	1.0	1.0	1.0	SOX2-OT
6658	2.2	1.0	2.6	1.0	SOX3
11063	1.0	1.0	1.0	1.0	SOX30
6659	2.6	1.0	1.0	1.0	SOX4
6660	2.3	1.0	-6.6	1.0	SOX5
83595	1.0	1.0	-5.2	1.0	SOX7
3431	1.0	1.0	1.0	1.0	SP110
11262	-1.7	1.0	-3.3	1.0	SP140
6670	116.7	1.0	1.0	1.0	SP3
124912	1.0	1.0	1.0	1.0	SPACA3
389852	5.9	2.1	1.8	2.0	SPACA5
79582	1.0	1.0	1.0	1.0	SPAG16
6676	1.0	1.0	4.6	5.7	SPAG4
26206	1.0	1.0	1.0	1.0	SPAG8
6677	1.0	1.0	1.0	1.0	SPAM1
30014	1.0	1.0	-5.9	-3.9	SPANXA1
139067	3.9	-4.6	-6.4	-1.9	SPANXN3
6678	1.0	3.7	3.9	1.0	SPARC
221178	1.0	4.8	1.0	1.0	SPATA13
132671	-5.6	1.0	1.0	-2.4	SPATA18
124044	1.0	1.0	-3.4	1.0	SPATA2L
132851	1.0	1.0	1.0	1.0	SPATA4
55812	1.0	2.8	1.0	1.0	SPATA7
145946	-19.6	1.0	1.0	2.0	SPATA8
83890	-33.8	1.0	1.0	1.0	SPATA9
221409	-1.7	1.0	1.0	-2.6	SPATS1
65244	1.0	1.0	1.0	3.9	SPATS2
441273	2.1	-4.4	1.0	1.0	SPDYE2
441272	-5.1	1.0	-7.2	1.0	SPDYE3
92521	-7.1	2.8	3.8	1.0	SPECC1

23384	1.0	1.0	1.0	1.0	SPECC1L
374768	2.4	1.0	1.0	1.0	SPEM1
56848	1.0	1.0	1.0	1.0	SPHK2
6688	1.0	1.0	5.0	1.0	SPI1
6689	-1.7	6.2	1.0	1.0	SPIB
10927	1.0	-7.2	-5.7	1.0	SPIN1
139886	1.0	1.0	1.0	1.0	SPIN4
11005	-11.0	1.0	1.0	1.0	SPINK5
10653	1.0	1.0	1.0	1.0	SPINT2
56907	1.0	1.0	1.0	1.0	SPIRE1
6693	1.0	1.0	1.0	1.0	SPN
83985	1.6	1.0	1.0	1.0	SPNS1
23626	1.7	1.0	1.0	1.0	SPO11
9806	4.6	1.0	1.0	-6.4	SPOCK2
10417	1.0	1.0	1.0	1.0	SPON2
6694	1.0	1.0	1.0	1.0	SPP2
84888	1.0	1.0	1.0	1.0	SPPL2A
56928	1.0	1.0	-8.9	1.0	SPPL2B
121665	5.8	3.7	1.0	1.0	SPPL3
503542	2.0	1.0	4.6	1.0	SPRN
6698	1.9	5.2	1.0	1.0	SPRR1A
6699	3.0	1.0	1.0	1.0	SPRR1B
6704	8.7	1.0	1.0	1.0	SPRR2E
163778	-1.8	-1.8	1.0	-3.3	SPRR4
90864	-4.2	1.0	1.0	1.0	SPSB3
6710	1.0	1.0	1.0	4.4	SPTB
6711	1.0	1.0	1.0	1.0	SPTBN1
10558	1.0	1.0	1.0	1.0	SPTLC1
9517	1.0	1.0	-1.8	-3.3	SPTLC2
55304	1.6	1.0	1.0	1.0	SPTLC3
58472	1.0	1.0	1.0	1.0	SQRDL
10847	1.0	1.0	4.3	1.0	SRCAP
80725	3.9	1.0	1.0	1.0	SRCIN1
6716	1.0	1.0	1.0	2.5	SRD5A2
285672	-2.4	1.0	1.0	1.0	SREK1IP1
6722	-2.1	-3.9	-3.7	-3.2	SRF
23380	-2.9	1.0	1.0	1.0	SRGAP2
6728	-1.7	1.0	-2.4	1.0	SRP19
6730	1.0	1.0	1.0	1.0	SRP68
26576	1.0	1.0	1.0	1.0	SRPK3
8406	1.0	1.0	4.8	1.0	SRPX
23524	1.0	-5.7	1.0	1.0	SRRM2
222183	1.0	1.0	1.0	-3.8	SRRM3
9295	1.0	1.0	1.6	1.7	SRSF11
6428	1.5	-3.3	-3.3	-3.3	SRSF3
6429	1.0	1.0	1.0	1.0	SRSF4
6431	1.0	-2.6	1.0	-2.6	SRSF6
8683	-1.9	-2.7	-2.4	1.0	SRSF9

140809	1.0	1.0	2.7	1.0	SRXN1
6760	1.0	1.0	1.0	1.0	SS18
51188	1.0	1.0	-5.0	1.0	SS18L2
6741	1.6	1.0	1.0	1.6	SSB
6742	1.0	-2.6	-3.0	-3.2	SSBP1
23635	-5.8	1.0	1.0	1.0	SSBP2
284297	2.0	1.0	-3.3	1.0	SSC5D
23145	2.4	-4.5	-6.9	-9.6	SSPO
10534	1.7	1.0	1.0	1.0	SSSCA1
6752	1.0	1.0	1.0	1.0	SSTR2
6754	1.0	1.0	3.5	1.0	SSTR4
29101	2.1	1.0	1.0	1.0	SSU72
10214	1.0	1.0	1.0	1.0	SSX3
280658	1.0	1.0	1.0	1.0	SSX7
6484	1.0	1.0	1.0	1.0	ST3GAL4
8869	1.0	1.0	1.0	1.0	ST3GAL5
6764	1.0	4.6	4.6	1.0	ST5
55808	-3.2	1.0	1.0	1.0	ST6GALNAC1
10610	1.0	1.0	1.0	1.0	ST6GALNAC2
256435	1.0	1.0	1.0	1.0	ST6GALNAC3
81849	-46.3	1.0	1.0	1.0	ST6GALNAC5
30815	1.0	1.0	1.0	1.0	ST6GALNAC6
7982	1.0	1.0	1.0	1.0	ST7
93653	1.0	1.0	1.0	1.0	ST7-AS1
54879	-6.4	1.0	1.0	1.0	ST7L
8128	1.0	1.0	1.0	1.0	ST8SIA2
246329	1.0	1.0	1.0	-5.0	STAC3
10735	1.0	1.0	1.0	1.0	STAG2
10734	-1.6	1.0	2.0	1.0	STAG3
442582	1.5	1.0	1.0	-1.6	STAG3L2
8027	1.0	1.0	3.5	1.0	STAM
10617	31.5	1.0	1.0	1.0	STAMBP
57559	2.3	1.0	1.0	1.0	STAMBPL1
26228	1.0	1.0	1.0	1.0	STAP1
6770	1.0	1.0	1.0	1.0	STAR
90627	1.0	1.0	1.0	1.0	STARD13
56910	1.0	3.7	4.0	3.9	STARD7
57519	1.0	1.0	-6.2	-2.7	STARD9
6772	1.0	-2.1	1.0	1.0	STAT1
8987	5.1	1.0	8.2	1.0	STBD1
6781	4.9	1.0	8.2	1.0	STC1
8614	1.0	1.0	-7.8	-7.5	STC2
26872	1.0	1.0	-6.3	1.0	STEAP1
55240	1.0	1.0	6.9	4.1	STEAP3
474171	1.0	4.1	1.0	1.0	STGC3
246744	1.0	1.0	1.0	1.0	STH
6793	1.0	1.0	1.0	1.0	STK10
6794	-2.5	2.5	4.8	3.9	STK11

114790	1.0	1.0	1.0	1.0	STK11IP
9262	1.0	1.0	1.0	1.0	STK17B
8428	1.0	1.0	-1.6	1.0	STK24
56164	1.0	1.0	1.0	1.0	STK31
282974	1.0	1.8	1.0	1.0	STK32C
65975	1.0	5.2	1.0	1.0	STK33
27148	2.1	1.0	-4.6	1.0	STK36
27347	1.0	1.0	-3.4	-3.6	STK39
6789	1.0	1.0	1.0	2.3	STK4
50861	1.0	3.1	3.6	1.0	STMN3
30968	-5.3	1.0	1.0	1.0	STOML2
161003	1.0	1.0	1.0	1.0	STOML3
85439	1.0	1.0	1.0	1.0	STON2
64220	1.0	-5.5	-4.7	1.0	STRA6
55437	-9.9	5.2	1.0	3.3	STRADB
55342	1.0	1.0	2.4	2.4	STRBP
3703	1.0	-2.7	1.0	-2.5	STT3A
201595	-2.3	1.0	1.0	-5.9	STT3B
55014	1.0	1.0	-2.4	-2.4	STX17
112755	1.0	1.0	1.0	1.0	STX1B
10228	1.0	1.0	1.0	1.0	STX6
8417	1.0	1.0	1.8	1.0	STX7
9482	-2.2	1.0	1.0	-4.1	STX8
252983	1.0	1.0	-1.5	1.0	STXBP4
9515	1.0	1.0	2.8	1.0	STXBP5L
29091	1.0	1.0	1.0	1.0	STXBP6
55359	1.0	1.0	1.0	1.0	STYK1
51657	1.0	1.0	1.0	1.0	STYXL1
10923	1.0	1.0	1.0	1.0	SUB1
8803	-1.8	4.0	5.6	4.8	SUCLA2
51684	2.6	1.0	1.0	2.4	SUFU
57794	1.0	1.0	2.8	2.5	SUGP1
6799	1.0	1.0	1.0	-2.7	SULT1A2
445329	1.0	1.0	-3.8	1.0	SULT1A4
6819	1.7	1.0	1.0	-3.4	SULT1C2
27233	1.0	1.0	6.2	1.0	SULT1C4
6820	1.0	1.0	1.0	1.0	SULT2B1
25830	1.0	1.0	1.0	1.0	SULT4A1
285362	1.0	1.0	3.7	1.0	SUMF1
7341	1.0	1.0	-2.8	-2.2	SUMO1
474338	1.0	-2.9	1.0	-2.6	SUMO1P3
6612	1.0	-2.4	1.0	1.0	SUMO3
23353	1.0	1.0	6.2	1.0	SUN1
256979	1.0	-1.8	1.0	1.0	SUN3
140732	-3.7	-2.8	-2.7	1.0	SUN5
8464	-30.0	1.5	1.0	1.0	SUPT3H
6832	1.0	-2.4	-7.1	-2.9	SUPV3L1
203328	1.0	1.0	1.0	1.0	SUSD3

6839	1.9	1.0	-3.8	1.0	SUV39H1
79723	1.0	1.0	1.6	1.0	SUV39H2
6840	1.0	1.0	4.3	1.0	SVIL
258010	1.9	1.0	-2.6	1.0	SVIP
55530	1.0	1.0	2.6	1.0	SVOP
55638	1.0	1.0	1.0	1.0	SYBU
644186	1.0	1.0	1.0	1.0	SYCE3
342898	1.0	1.0	1.0	1.0	SYCN
84144	1.0	1.0	1.0	5.6	SYDE2
6853	1.0	1.0	1.0	1.0	SYN1
81493	1.0	1.0	-3.1	1.0	SYNC
23345	1.0	3.0	1.0	1.0	SYNE1
8831	1.0	1.0	1.0	1.0	SYNGAP1
9145	1.0	-4.1	1.0	1.0	SYNGR1
9144	-5.6	1.0	1.0	1.0	SYNGR2
8871	1.0	1.0	-2.6	1.0	SYNJ2
11346	2.5	1.0	2.5	1.0	SYNPO
171024	1.0	-5.8	-8.1	1.0	SYNPO2
79933	1.0	1.0	1.0	-1.5	SYNPO2L
132204	1.0	1.0	1.0	1.0	SYNPR
6856	1.0	1.0	4.0	4.2	SYPL1
6857	1.0	3.0	1.0	1.0	SYT1
51760	1.0	1.0	1.0	1.0	SYT17
148281	1.0	1.0	-4.0	1.0	SYT6
90019	1.0	-7.4	1.0	1.0	SYT8
84958	1.0	4.3	3.7	1.0	SYTL1
94121	1.0	1.0	1.0	1.0	SYTL4
84447	1.0	1.0	10.4	1.0	SYVN1
23334	-2.3	1.0	1.0	1.0	SZT2
9287	1.0	1.0	1.0	1.0	TAAR2
10454	1.0	1.0	1.0	1.0	TAB1
6863	1.0	-6.3	1.0	-7.9	TAC1
117143	1.0	1.0	1.0	1.0	TADA1
6871	1.0	-2.6	1.0	1.0	TADA2A
9015	1.0	1.0	1.0	1.0	TAF1A
79101	-13.8	1.0	1.0	1.0	TAF1D
138474	1.0	1.0	5.3	4.3	TAF1L
83860	-5.5	1.0	1.0	1.0	TAF3
6874	1.0	-5.4	-5.4	1.0	TAF4
6875	1.0	1.0	-4.8	1.0	TAF4B
27097	-3.8	1.0	1.0	1.0	TAF5L
6878	1.0	1.0	1.0	1.0	TAF6
6880	1.0	1.0	1.0	1.0	TAF9
117289	1.0	-4.8	1.0	1.0	TAGAP
6876	1.0	1.0	1.0	1.0	TAGLN
6886	1.0	-4.4	-2.7	-5.4	TAL1
6887	1.0	1.0	1.0	1.0	TAL2
26115	1.0	4.1	3.0	1.0	TANC2

10010	1.0	1.0	1.0	1.0	TANK
57551	1.0	1.0	1.0	1.0	TAOK1
9344	3.3	2.4	1.0	-2.8	TAOK2
6890	1.0	4.6	1.0	1.0	TAP1
6891	3.8	1.0	4.6	3.1	TAP2
6892	-16.4	1.0	1.0	1.0	TAPBP
55080	1.0	1.0	1.0	1.0	TAPBPL
202018	-12.1	-5.6	-5.0	-3.7	TAPT1
445347	1.0	-5.7	-5.8	1.0	TARP
80222	1.0	1.0	1.0	1.0	TARS2
80835	-2.6	1.0	1.0	-7.6	TAS1R1
50839	1.0	1.0	-2.7	-3.0	TAS2R10
50838	1.0	1.0	1.0	1.0	TAS2R13
259290	1.0	1.0	1.0	2.8	TAS2R31
259285	1.0	1.0	1.0	1.0	TAS2R39
50832	1.0	1.0	1.0	1.0	TAS2R4
259286	1.0	1.0	2.7	1.0	TAS2R40
54429	1.0	1.0	1.0	1.0	TAS2R5
338398	-2.3	1.0	1.0	-5.2	TAS2R60
6898	1.0	1.0	1.0	1.0	TAT
83940	1.0	1.0	-3.5	1.0	TATDN1
6901	1.0	1.0	2.7	1.0	TAZ
54662	1.0	1.0	1.0	1.0	TBC1D13
64786	1.0	1.0	1.0	1.0	TBC1D15
125058	1.0	1.0	2.4	1.0	TBC1D16
25771	1.0	1.0	1.0	1.0	TBC1D22A
4943	1.0	-4.4	1.0	1.0	TBC1D25
353149	1.0	1.0	1.0	1.0	TBC1D26
26083	1.0	1.0	1.0	1.0	TBC1D29
23329	1.0	1.0	1.0	1.0	TBC1D30
9779	1.0	1.0	5.1	1.0	TBC1D5
1155	1.0	1.0	1.0	-3.7	TBCB
29110	1.0	1.0	1.0	1.0	TBK1
90665	1.0	1.0	1.7	1.0	TBL1Y
6908	1.0	1.0	2.3	2.4	TBP
10716	1.0	4.8	1.0	1.0	TBR1
6899	1.8	1.0	1.0	1.0	TBX1
6913	1.0	1.0	-2.8	1.0	TBX15
6926	1.0	1.0	-9.5	-8.6	TBX3
6911	1.0	1.0	1.0	1.0	TBX6
6915	1.0	1.0	-3.2	-2.5	TBXA2R
6916	2.2	3.7	1.0	1.0	TBXAS1
9338	1.0	3.0	3.1	1.0	TCEAL1
140597	1.0	1.0	1.0	1.0	TCEAL2
79921	1.0	3.1	2.3	1.0	TCEAL4
56849	1.0	-6.1	-8.3	-6.3	TCEAL7
90843	1.0	1.0	1.0	1.0	TCEAL8
162699	1.0	1.0	1.0	1.0	TCEB3C

6941	1.0	1.0	1.0	1.0	TCF19
6943	1.0	1.0	-3.0	1.0	TCF21
150921	1.0	1.0	1.0	1.0	TCF23
22980	1.7	1.0	4.6	2.9	TCF25
6929	2.6	1.0	1.0	1.0	TCF3
6925	-8.8	1.0	1.0	1.0	TCF4
6932	1.0	1.0	1.0	1.0	TCF7
83439	1.0	1.0	1.0	1.0	TCF7L1
6934	1.0	1.0	1.0	-1.6	TCF7L2
10732	1.0	2.0	1.0	1.0	TCFL5
8115	1.0	2.8	1.0	1.0	TCL1A
9623	1.7	1.0	5.6	1.0	TCL1B
6949	2.1	1.0	-2.4	1.0	TCOF1
6950	-1.8	-6.1	-4.1	1.0	TCP1
6953	1.0	1.0	1.0	1.0	TCP10
140290	1.0	-2.7	-2.6	1.0	TCP10L
401285	1.6	1.0	6.0	1.0	TCP10L2
6954	1.0	1.0	1.0	1.0	TCP11
55346	1.0	1.0	-2.3	1.0	TCP11L1
202500	1.0	1.0	-3.3	1.0	TCTE1
6991	5.5	1.0	1.0	1.0	TCTE3
255758	1.0	1.0	-6.0	1.0	TCTEX1D2
79867	1.0	4.7	4.1	5.8	TCTN2
26123	1.0	-7.1	1.0	-7.5	TCTN3
6996	1.0	1.0	1.0	1.0	TDG
55775	1.0	1.0	1.0	1.0	TDP1
56165	-7.9	1.0	4.7	1.0	TDRD1
163589	1.0	-2.6	1.0	-2.2	TDRD5
221400	1.0	1.0	1.0	1.0	TDRD6
23424	-2.7	1.0	-5.0	-5.0	TDRD7
732253	1.0	5.4	5.1	3.8	TDRG1
11022	1.0	1.0	1.0	1.0	TDRKH
7004	1.0	-3.1	1.0	1.0	TEAD4
25851	1.0	1.0	1.8	1.0	TECPR1
9895	1.0	1.0	1.0	1.0	TECPR2
7007	1.0	1.0	1.0	1.0	TECTA
6975	-1.8	1.0	1.0	-4.4	TECTB
83659	1.0	1.0	-3.5	1.0	TEKT1
100132288	-3.7	1.0	1.0	1.0	TEKT4P2
9894	1.7	-3.9	1.0	1.0	TELO2
7013	4.2	-6.0	4.6	4.5	TERF1
54386	1.0	1.0	1.8	1.0	TERF2IP
26136	1.0	2.4	2.9	1.0	TES
54997	1.0	1.0	1.0	1.0	TESC
10420	1.0	1.0	3.3	3.2	TESK2
80312	-6.0	1.0	1.0	1.0	TET1
54881	-1.5	1.0	1.0	1.0	TEX10
56157	1.0	-3.4	-3.9	1.0	TEX13A

56156	1.0	1.0	1.0	1.0	TEX13B
56154	1.0	-4.1	-5.6	-3.6	TEX15
1527	2.3	1.0	1.0	1.0	TEX28
7018	1.0	1.0	1.0	1.0	TF
7019	1.0	1.0	-5.4	1.0	TFAM
7020	1.0	1.0	1.0	1.0	TFAP2A
7022	1.0	1.0	4.1	4.7	TFAP2C
83741	1.0	-7.2	-9.0	1.0	TFAP2D
51106	1.0	1.0	4.4	1.0	TFB1M
7027	1.9	1.0	1.0	1.0	TFDP1
51270	1.0	1.0	1.0	1.0	TFDP3
7030	1.0	1.0	3.2	1.0	TFE3
7942	1.0	1.0	-2.4	1.0	TFEB
7033	-1.7	1.0	1.0	1.0	TFF3
10342	1.0	1.0	1.0	1.0	TFG
7035	1.0	1.0	2.4	1.0	TFPI
7037	1.0	2.7	3.3	1.0	TFRC
7040	1.0	-1.7	-1.5	1.0	TGFB1
7042	1.0	1.0	-5.8	1.0	TGFB2
7043	1.0	1.0	-4.6	1.0	TGFB3
7045	1.0	1.0	2.5	1.0	TGFBI
7048	1.0	1.0	1.0	1.0	TGFBR2
9392	1.0	1.5	1.0	1.0	TGFBRAP1
60436	1.0	1.0	1.0	1.0	TGIF2
9333	1.0	1.0	-4.2	1.0	TGM5
116179	1.0	1.0	3.4	1.0	TGM7
10618	1.0	1.0	1.0	2.5	TGOLN2
51497	1.0	1.0	3.5	1.0	TH1L
63892	1.0	1.0	1.0	1.0	THADA
439931	1.0	1.0	-5.6	1.0	THAP7-AS1
199745	1.0	1.0	1.0	1.0	THAP8
7056	1.0	1.0	6.8	1.0	THBD
7058	1.0	1.0	1.0	1.0	THBS2
51298	1.0	1.0	1.0	1.0	THEG
117145	1.6	1.0	1.0	1.0	THEM4
284486	4.4	1.0	-6.5	1.0	THEM5
54974	1.9	-2.3	1.0	1.0	THG1L
57187	1.0	-2.9	2.7	-3.3	THOC2
84321	1.0	-4.3	-4.3	-4.1	THOC3
8563	-7.2	1.0	1.0	-3.8	THOC5
79228	1.0	1.0	1.0	-2.4	THOC6
7066	1.0	1.0	-2.3	1.0	THPO
7067	3.2	1.0	1.0	1.0	THRA
9967	1.0	1.0	1.0	1.0	THRAP3
79875	1.0	1.0	-16.8	1.0	THSD4
80731	1.5	1.0	1.0	1.0	THSD7B
80745	1.0	1.0	2.5	1.0	THUMPD2
7072	2.0	1.0	1.0	1.0	TIA1

7073	1.0	1.0	1.0	1.0	TIAL1
200765	2.0	1.0	-2.9	1.0	TIGD1
220359	1.0	1.0	1.0	1.0	TIGD3
201633	1.0	1.0	1.7	1.0	TIGIT
26519	1.0	1.0	1.0	1.0	TIMM10
26517	1.0	1.0	1.0	1.0	TIMM13
10440	-3.0	1.0	1.0	-7.5	TIMM17A
29928	1.0	1.9	5.5	2.1	TIMM22
100287932	1.9	1.0	1.0	1.0	TIMM23
10469	1.0	1.0	1.0	1.0	TIMM44
26521	1.0	1.0	1.0	1.0	TIMM8B
26520	1.0	1.0	1.0	1.0	TIMM9
7079	1.0	1.0	2.9	1.0	TIMP4
64129	1.0	1.0	1.0	1.0	TINAGL1
26277	1.0	1.0	3.1	3.4	TINF2
25976	1.0	-2.3	1.0	-5.1	TIPARP
54962	1.0	-3.2	-3.3	-3.2	TIPIN
261726	1.0	1.0	1.0	1.0	TIPRL
114609	1.0	1.0	1.0	1.0	TIRAP
93643	1.0	1.0	1.0	1.0	TJAP1
9414	1.0	1.0	4.0	3.4	TJP2
8277	1.0	1.0	2.5	1.0	TKTL1
7088	1.0	3.8	5.8	1.0	TLE1
7091	1.0	1.0	1.0	1.0	TLE4
79816	1.0	-2.6	1.0	1.0	TLE6
9874	-1.6	1.0	1.0	1.0	TLK1
11011	1.8	1.0	-4.7	1.0	TLK2
7092	-4.5	1.0	1.0	1.0	TLL1
7098	1.0	1.0	1.0	1.0	TLR3
7099	1.0	1.0	1.0	1.0	TLR4
349408	1.0	1.0	2.6	1.0	TLR8-AS1
83941	1.0	1.0	-2.4	1.0	TM2D1
4071	1.0	1.0	-6.9	1.0	TM4SF1
7104	1.0	1.0	1.0	1.0	TM4SF4
9032	1.0	5.2	3.6	1.0	TM4SF5
53346	1.8	-2.5	-2.8	1.0	TM6SF1
53345	1.0	-3.5	1.0	1.0	TM6SF2
9375	1.0	1.0	1.0	1.0	TM9SF2
51643	1.0	1.0	1.0	1.0	TMBIM4
117531	1.0	1.0	1.0	1.0	TMC1
342125	1.0	1.0	5.9	1.0	TMC3
147138	1.0	1.0	5.1	1.0	TMC8
23023	-2.8	5.2	4.8	3.5	TMCC1
9911	1.0	1.0	-3.5	-5.6	TMCC2
127391	1.0	1.0	1.0	1.0	TMCO2
55002	1.0	1.0	1.0	1.0	TMCO3
255104	1.0	1.0	2.1	1.0	TMCO4
145942	1.5	1.0	1.0	5.3	TMCO5A

79613	1.0	1.0	1.0	1.0	TMCO7
11018	1.0	-2.8	1.0	-2.5	TMED1
10972	1.0	1.0	4.5	1.0	TMED10
146456	1.0	1.0	1.0	1.0	TMED6
283578	1.0	1.0	4.5	1.0	TMED8
284114	1.0	1.0	1.0	-3.3	TMEM102
54868	1.0	1.0	1.0	1.0	TMEM104
113277	1.0	1.0	1.0	4.4	TMEM106A
79022	1.0	1.0	4.6	3.3	TMEM106C
66000	1.0	1.0	1.0	1.0	TMEM108
8834	1.0	-2.6	1.0	1.0	TMEM11
55831	1.0	1.0	1.0	1.0	TMEM111
283953	1.0	1.0	-4.7	1.0	TMEM114
89894	1.0	1.0	1.0	1.0	TMEM116
338773	1.0	1.0	-2.9	1.0	TMEM119
144404	1.0	1.0	-5.5	-4.4	TMEM120B
114908	1.0	2.4	1.7	1.0	TMEM123
128218	1.0	1.0	1.0	1.0	TMEM125
55863	1.0	1.0	1.0	1.0	TMEM126B
51524	1.0	1.0	1.0	1.0	TMEM138
135932	1.0	1.0	1.0	1.0	TMEM139
55281	1.0	1.0	1.0	3.2	TMEM140
257062	1.0	5.6	3.5	4.7	TMEM146
81853	1.0	1.0	1.0	2.4	TMEM14B
51522	1.0	1.0	1.0	1.0	TMEM14C
129303	-1.6	1.7	1.5	2.1	TMEM150A
201799	1.7	-1.9	-4.3	1.0	TMEM154
153396	3.1	1.0	6.4	1.0	TMEM161B
84187	1.0	1.0	2.2	1.0	TMEM164
55858	1.0	1.0	1.0	1.0	TMEM165
56900	1.0	1.0	1.0	1.0	TMEM167B
124491	1.0	2.0	1.0	1.0	TMEM170A
100113407	1.0	1.0	1.0	15.2	TMEM170B
84286	1.0	1.0	2.7	1.0	TMEM175
28959	1.0	1.0	1.0	1.0	TMEM176B
80775	1.0	1.0	1.9	1.0	TMEM177
130733	1.0	1.0	-4.9	-6.3	TMEM178
388021	1.0	1.0	1.0	1.0	TMEM179
653659	1.0	1.0	2.3	1.0	TMEM183B
202915	1.0	1.0	1.0	1.0	TMEM184A
147744	1.0	1.9	1.0	1.0	TMEM190
84222	1.0	1.0	1.0	1.0	TMEM191A
147007	1.0	1.0	1.0	1.0	TMEM199
23670	1.0	1.0	1.0	1.0	TMEM2
645369	1.0	1.0	1.0	3.5	TMEM200C
199953	-1.6	1.0	1.0	1.0	TMEM201
374882	1.0	1.0	1.0	-4.4	TMEM205
84928	1.0	3.1	2.9	2.9	TMEM209

221468	1.0	1.0	3.0	3.2	TMEM217
84065	1.0	-2.4	1.0	1.0	TMEM222
338661	1.0	1.0	1.0	-6.4	TMEM225
161145	1.0	1.0	1.0	1.0	TMEM229B
79583	4.1	1.0	1.0	5.1	TMEM231
65062	-2.7	1.0	-12.5	1.0	TMEM237
100288797	1.0	1.0	1.0	1.0	TMEM239
84866	1.0	1.0	-3.5	1.0	TMEM25
203562	1.0	1.0	3.8	2.9	TMEM31
140738	1.0	2.7	1.0	2.4	TMEM37
79041	1.0	1.0	1.0	-2.1	TMEM38A
90407	-2.1	-7.0	-3.7	-6.8	TMEM41A
131616	1.0	3.8	1.0	1.0	TMEM42
79188	1.0	3.0	2.6	2.8	TMEM43
93109	-2.8	1.0	1.0	1.0	TMEM44
55706	1.6	-4.6	1.0	1.0	TMEM48
10329	1.0	1.0	1.0	1.0	TMEM5
757	1.0	1.0	1.0	1.0	TMEM50B
339456	1.0	1.0	2.4	2.0	TMEM52
79639	1.0	1.0	1.0	1.0	TMEM53
113452	1.0	1.0	-2.6	1.0	TMEM54
55529	1.0	1.0	1.0	1.0	TMEM55A
90809	1.0	1.0	6.0	1.0	TMEM55B
55219	1.0	1.0	-3.3	-1.9	TMEM57
9528	1.0	1.0	-2.5	-2.2	TMEM59
169200	1.0	1.0	1.0	1.0	TMEM64
137695	1.0	2.0	4.1	1.0	TMEM68
51249	1.0	1.0	1.0	1.0	TMEM69
137835	1.0	2.6	-4.3	1.0	TMEM71
643236	1.0	3.5	1.0	2.6	TMEM72
157753	1.0	26.6	1.0	1.0	TMEM74
283232	1.0	1.0	2.5	4.2	TMEM80
51234	-5.3	1.0	1.0	-4.1	TMEM85
144110	-3.6	1.0	-1.5	1.0	TMEM86A
84910	1.0	1.0	1.0	1.0	TMEM87B
440955	1.0	-3.4	-3.4	1.0	TMEM89
51754	1.0	1.0	-2.6	-2.5	TMEM8B
26022	1.0	1.0	1.0	1.0	TMEM98
29765	1.0	-3.6	-4.2	-3.5	TMOD4
339967	1.0	1.0	1.0	1.0	TMPRSS11A
132724	1.0	1.0	-1.6	1.0	TMPRSS11B
84000	1.7	1.0	6.1	1.0	TMPRSS13
56649	1.6	1.0	1.0	1.0	TMPRSS4
80975	1.0	1.0	1.0	1.0	TMPRSS5
164656	1.0	1.0	3.6	1.0	TMPRSS6
360200	1.0	1.0	1.0	1.0	TMPRSS9
286527	1.0	1.0	1.0	4.6	TMSB15B
83857	1.0	1.0	4.4	5.4	TMTC1

81542	1.0	1.0	2.7	1.0	TMX1
51075	1.0	3.6	5.2	4.4	TMX2
54495	1.0	1.0	1.0	1.0	TMX3
7126	1.0	1.0	1.0	-4.4	TNFAIP1
25816	1.9	1.0	1.0	1.0	TNFAIP8
8794	1.0	1.0	5.3	1.0	TNFRSF10C
4982	1.0	-2.8	-3.8	-2.3	TNFRSF11B
51330	1.0	1.0	1.8	1.0	TNFRSF12A
23495	1.0	1.0	1.0	1.0	TNFRSF13B
8764	1.0	2.8	1.0	1.0	TNFRSF14
7132	6.5	1.0	5.6	4.1	TNFRSF1A
7293	1.0	-3.5	1.0	1.0	TNFRSF4
8743	1.0	1.0	1.0	2.5	TNFSF10
8741	1.0	1.0	1.0	1.0	TNFSF13
10673	1.0	1.0	-3.2	1.0	TNFSF13B
8740	1.0	1.0	1.0	1.0	TNFSF14
9966	-3.0	1.0	1.0	1.0	TNFSF15
8995	2.0	1.0	-2.9	1.0	TNFSF18
8658	1.0	1.0	1.0	-4.8	TNKS
80351	1.0	1.0	1.0	2.8	TNKS2
7138	1.0	-3.3	-3.3	1.0	TNNT1
7140	1.0	1.0	1.0	1.0	TNNT3
7141	1.0	1.0	1.0	1.0	TNP1
7142	-5.3	1.0	-3.1	-3.2	TNP2
23534	1.0	1.0	-4.4	-3.8	TNPO3
7143	1.0	1.0	-4.4	1.0	TNR
84629	1.0	1.0	1.0	1.0	TNRC18
7145	-2.8	1.0	1.0	1.0	TNS1
84951	1.0	1.0	1.0	1.0	TNS4
7148	1.0	1.0	1.0	1.0	TNXB
222699	1.0	1.0	1.0	1.0	TOB2P1
146691	1.0	1.0	1.0	1.0	TOM1L2
9804	-2.7	-2.4	-2.5	-2.5	TOMM20
56993	1.0	1.0	1.0	1.0	TOMM22
10452	-18.9	1.0	1.0	-4.5	TOMM40
100188893	1.6	2.4	2.6	1.0	TOMM6
7150	1.0	1.0	1.0	-2.9	TOP1
26092	1.7	1.0	1.0	1.0	TOR1AIP1
84969	1.0	1.6	1.0	1.0	TOX2
9878	1.0	1.0	1.0	1.0	TOX4
7157	1.0	1.0	1.0	3.0	TP53
63970	1.0	1.0	1.0	1.0	TP53AIP1
94241	1.0	1.0	4.7	1.0	TP53INP1
112858	1.0	1.0	1.0	1.0	TP53RK
11257	1.0	1.0	1.0	1.0	TP53TG1
24150	2.9	2.3	1.0	1.5	TP53TG3
27296	1.0	-2.8	-2.8	-3.0	TP53TG5
7161	1.0	1.0	1.0	1.0	TP73

7162	1.0	1.0	1.0	2.3	TPBG
53373	1.0	1.0	1.0	1.0	TPCN1
7163	1.0	1.0	1.0	1.0	TPD52
7164	1.0	1.0	1.0	1.0	TPD52L1
89882	1.0	-5.0	1.0	-5.3	TPD52L3
7166	2.0	1.0	1.0	1.0	TPH1
7169	1.0	-2.3	1.0	1.0	TPM2
7170	1.0	-5.8	4.0	2.9	TPM3
7172	1.0	1.0	1.0	1.0	TPMT
7173	1.0	13.4	1.0	1.0	TPO
122664	1.0	1.0	1.0	1.6	TPPP2
7175	1.6	1.0	-2.6	1.0	TPR
348825	1.0	3.0	1.0	1.0	TPRXL
7177	1.0	1.0	1.0	-4.8	TPSAB1
23430	1.0	4.1	3.3	1.0	TPSD1
7178	-1.8	1.0	-5.4	-4.2	TPT1
93492	1.0	1.0	1.0	1.0	TPTE2
646405	1.0	1.0	-3.7	1.0	TPTE2P1
220115	1.0	-3.4	1.0	1.0	TPTE2P3
22974	1.0	1.0	1.0	1.0	TPX2
6434	-1.9	1.0	-2.6	1.0	TRA2B
8717	1.0	1.0	1.0	1.0	TRADD
80342	-8.9	-4.2	-3.1	-3.2	TRAF3IP3
7189	1.6	1.0	2.3	1.0	TRAF6
133022	1.0	1.0	1.0	3.8	TRAM1L1
7109	1.0	1.0	-3.6	-3.3	TRAPPC10
6399	1.0	1.0	-7.6	1.0	TRAPPC2
27095	1.0	1.0	1.0	1.0	TRAPPC3
79090	1.0	1.0	3.0	1.0	TRAPPC6A
22878	1.0	1.0	-7.1	1.0	TRAPPC8
11181	1.0	1.0	1.0	1.0	TREH
11277	1.0	1.0	1.0	-3.8	TREX1
29953	1.0	-3.2	-4.0	1.0	TRHDE
57761	1.0	1.0	1.0	1.0	TRIB3
9865	9.3	1.0	1.0	1.0	TRIL
81559	1.0	-3.3	-3.2	1.0	TRIM11
9830	1.0	1.0	2.7	3.7	TRIM14
89870	-13.5	-12.7	-8.5	-4.9	TRIM15
23321	-4.7	1.0	1.0	1.0	TRIM2
10346	1.0	-2.7	1.0	1.0	TRIM22
8805	1.0	1.0	5.4	1.0	TRIM24
7706	-1.6	1.0	1.0	1.0	TRIM25
5987	5.1	1.0	1.0	1.0	TRIM27
10612	1.0	1.0	1.0	1.0	TRIM3
53840	1.0	2.7	1.7	1.0	TRIM34
10475	1.0	-4.1	-6.3	-4.2	TRIM38
89122	1.0	2.8	1.0	1.0	TRIM4
135644	1.0	1.0	-7.1	-5.5	TRIM40

80128	1.0	1.0	-2.6	1.0	TRIM46
57093	1.0	1.0	3.0	1.0	TRIM49
642569	1.0	1.0	1.0	1.0	TRIM53P
84675	1.0	1.0	1.0	1.0	TRIM55
81844	1.0	1.0	1.0	1.0	TRIM56
55223	1.8	-3.3	1.0	1.0	TRIM62
9866	1.0	1.0	1.0	1.0	TRIM66
205860	1.0	2.7	1.0	1.0	TRIML2
7204	1.0	2.7	1.0	3.6	TRIO
11078	6.9	-10.6	-8.8	-7.8	TRIOBP
9321	1.0	1.0	1.0	1.0	TRIP11
9320	1.0	1.0	-6.4	1.0	TRIP12
9319	1.7	5.1	-1.6	1.0	TRIP13
51504	1.0	1.0	5.2	1.0	TRMT112
55039	1.0	1.0	1.0	3.3	TRMT12
27037	1.0	1.0	1.0	3.6	TRMT2A
79979	-3.2	1.0	1.6	3.2	TRMT2B
57570	1.0	-2.6	-2.4	1.7	TRMT5
51605	1.0	1.0	1.0	1.0	TRMT6
115708	1.5	1.0	1.0	2.5	TRMT61A
54952	-1.9	1.0	-6.9	1.0	TRNAU1AP
51095	1.0	-7.1	1.0	1.0	TRNT1
7216	1.0	1.0	1.0	1.0	TRO
8989	1.0	1.0	1.0	1.0	TRPA1
7221	1.0	5.3	1.0	1.0	TRPC2
7222	1.0	1.0	1.0	1.0	TRPC3
7223	1.0	2.1	1.0	1.9	TRPC4
26133	-1.8	1.0	1.0	1.0	TRPC4AP
57113	1.0	-5.9	1.0	1.0	TRPC7
4308	-8.4	1.0	-3.3	1.0	TRPM1
80036	-1.7	1.0	1.0	1.0	TRPM3
29850	1.0	1.0	-3.5	1.0	TRPM5
140803	-1.8	1.0	-2.0	1.0	TRPM6
54822	1.0	3.8	1.0	1.0	TRPM7
7227	1.0	-3.0	-4.8	-3.3	TRPS1
7442	1.0	1.0	1.0	1.0	TRPV1
162514	-21.8	1.0	1.0	1.0	TRPV3
55503	1.0	1.0	1.0	1.0	TRPV6
7249	1.8	1.0	1.0	1.0	TSC2
8848	1.0	1.0	1.0	1.0	TSC22D1
9819	2.6	1.0	1.0	1.0	TSC22D2
80746	1.0	1.0	1.0	4.6	TSEN2
10102	1.0	1.0	-5.5	1.0	TSFM
7251	2.1	1.0	6.8	1.0	TSG101
254187	1.0	1.0	1.0	-3.7	TSGA10IP
10194	2.8	1.0	2.6	1.0	TSHZ1
128553	1.0	-3.6	-5.1	1.0	TSHZ2
25987	1.0	3.1	2.9	1.0	TSKU

203062	-1.6	1.0	1.0	1.0	TSNARE1
441631	2.1	1.0	1.0	1.0	TSPAN11
23555	1.0	1.0	1.0	1.7	TSPAN15
10077	1.0	1.0	2.5	1.0	TSPAN32
7102	1.0	1.0	-2.0	1.0	TSPAN7
7103	1.0	1.0	1.0	1.0	TSPAN8
706	1.0	1.0	1.0	1.0	TSPO
728137	1.0	1.0	1.0	1.0	TSPY3
83942	1.0	1.0	1.0	1.0	TSSK1B
81629	1.0	1.0	1.0	1.0	TSSK3
83983	2.9	1.0	1.0	1.0	TSSK6
7263	4.6	7.2	1.0	1.0	TST
158427	1.0	1.0	1.0	1.0	TSTD2
84630	1.0	-1.6	1.0	1.0	TTBK1
146057	1.0	1.0	4.1	1.0	TTBK2
7265	1.0	1.0	-5.5	1.0	TTC1
79573	1.0	1.0	1.0	-2.2	TTC13
158248	1.0	1.0	1.0	-3.2	TTC16
55761	1.0	1.0	1.0	1.0	TTC17
118491	1.0	1.0	1.0	1.0	TTC18
79809	1.0	-3.0	3.3	1.0	TTC21B
64927	1.0	1.0	1.0	1.0	TTC23
79989	1.0	1.0	-7.6	1.0	TTC26
23331	1.0	2.4	1.0	1.0	TTC28
284900	1.0	1.0	1.0	1.0	TTC28-AS1
83894	1.0	1.0	-4.0	1.0	TTC29
92104	-1.8	1.0	1.0	1.0	TTC30A
9694	1.0	1.0	1.0	1.0	TTC35
9652	1.0	-4.2	-4.8	1.0	TTC37
55020	1.0	2.8	1.0	2.2	TTC38
22996	1.0	25.6	1.0	2.1	TTC39A
125488	2.0	1.0	1.0	-7.2	TTC39C
7268	1.0	1.0	1.0	3.8	TTC4
91875	1.0	1.0	1.0	1.0	TTC5
23508	-4.6	-6.5	-5.5	-11.8	TTC9
148014	-4.4	1.0	1.0	1.0	TTC9B
9675	1.0	1.0	1.0	1.0	TTI1
80185	1.0	-1.8	1.0	-2.9	TTI2
25809	1.0	1.0	-2.2	1.0	TTLL1
254173	1.0	-2.8	1.0	1.0	TTLL10
23093	1.0	3.0	1.0	1.0	TTLL5
79739	-27.2	1.0	1.0	1.0	TTLL7
7273	1.0	-3.5	1.0	1.0	TTN
83869	1.0	2.2	1.0	1.0	TTTY14
252953	1.0	1.0	-3.9	-6.8	TTTY21
114760	2.2	-4.4	-2.2	1.0	TTTY3
83863	1.0	1.0	1.0	1.0	TTTY5
84672	1.0	1.0	1.0	-3.4	TTTY6

94015	1.0	1.0	1.0	1.0	TTYH2
7846	1.0	1.0	1.0	1.0	TUBA1A
7277	1.0	1.0	1.0	2.8	TUBA4A
203068	1.0	1.0	1.0	1.0	TUBB
81027	1.9	-1.8	-5.0	-3.6	TUBB1
10381	1.0	1.0	-8.7	1.0	TUBB3
84617	1.0	1.0	1.0	1.0	TUBB6
347688	1.0	-3.8	-4.9	-5.3	TUBB8
27175	1.0	1.0	4.5	1.0	TUBG2
114791	1.0	1.0	1.0	1.0	TUBGCP5
85378	1.0	-3.8	1.0	1.0	TUBGCP6
7286	1.0	2.3	4.8	1.0	TUFT1
7289	1.0	1.0	4.8	1.0	TULP3
64852	1.0	1.0	1.0	1.0	TUT1
117581	1.0	1.0	1.0	1.0	TWIST2
57045	1.0	-5.9	-5.9	1.0	TWSG1
7294	1.0	1.0	1.0	1.0	TXK
55787	2.1	-2.0	1.0	1.0	TXLNG
7295	2.6	1.0	1.0	1.0	TXN
51061	1.5	1.0	1.0	1.0	TXNDC11
84817	1.0	1.0	1.6	1.0	TXNDC17
81567	1.0	1.0	1.0	1.0	TXNDC5
10190	1.0	1.0	2.6	1.0	TXNDC9
9352	1.0	1.0	1.0	1.0	TXNL1
10907	1.0	1.0	-5.6	1.0	TXNL4A
7296	1.0	1.0	1.0	1.0	TXNRD1
7305	1.0	-2.7	-2.7	1.0	TYROBP
10054	1.0	1.0	1.0	1.0	UBA2
79876	1.0	1.0	1.0	1.0	UBA5
7311	1.0	1.0	1.0	1.0	UBA52
55236	1.0	1.0	1.0	1.0	UBA6
7318	1.0	1.0	1.0	1.0	UBA7
337867	1.0	-2.9	1.0	1.0	UBAC2
51271	1.0	3.2	2.6	2.7	UBAP1
390595	1.0	1.0	1.0	1.0	UBAP1L
9898	1.0	1.0	1.0	2.3	UBAP2L
7319	1.0	1.0	1.0	1.0	UBE2A
7321	7.1	1.0	3.2	3.5	UBE2D1
7323	1.0	8.6	-6.8	1.0	UBE2D3
7328	-3.1	1.0	1.0	1.0	UBE2H
389898	1.0	1.0	1.0	1.0	UBE2NL
29089	1.0	1.0	1.5	1.0	UBE2T
7335	-2.0	1.0	1.0	1.0	UBE2V1
65264	1.0	1.0	1.0	-3.0	UBE2Z
89910	1.0	1.0	2.5	1.0	UBE3B
9690	1.0	3.5	6.8	1.0	UBE3C
10277	1.0	1.0	-7.3	1.0	UBE4B
56061	1.0	1.0	1.0	1.0	UBFD1

5412	1.0	2.5	2.5	1.6	UBL3
59286	1.0	1.0	1.0	1.0	UBL5
84993	1.0	1.0	1.0	-2.0	UBL7
7343	1.0	1.0	1.0	1.0	UBTF
26043	1.0	3.9	1.0	1.0	UBXN7
7993	1.0	1.0	1.0	5.0	UBXN8
221044	1.0	1.0	1.0	1.0	UCMA
90226	1.0	1.0	-4.3	-3.8	UCN2
51569	1.0	1.0	1.0	1.0	UFM1
643763	1.0	1.0	4.6	1.0	UG0898H09
7358	1.0	-6.5	1.0	1.0	UGDH
54576	2.1	-2.2	1.0	1.0	UGT1A8
79799	1.0	1.0	1.0	1.0	UGT2A3
7365	1.0	1.0	-4.8	1.0	UGT2B10
10720	1.0	1.0	2.4	1.0	UGT2B11
7366	1.0	1.0	1.0	1.0	UGT2B15
7364	1.0	1.0	1.0	1.0	UGT2B7
133688	1.0	1.0	1.0	1.0	UGT3A1
7368	12.0	1.0	3.4	1.0	UGT8
127933	1.0	1.0	3.4	1.0	UHMK1
8408	1.0	1.0	1.0	1.0	ULK1
9706	1.0	-4.1	-5.9	-5.0	ULK2
54986	-6.0	1.0	1.0	2.6	ULK4
89766	1.0	5.1	4.0	5.3	UMODL1
9094	1.0	1.0	1.0	1.0	UNC119
10497	1.0	1.0	4.5	1.0	UNC13B
55898	1.8	1.0	2.6	1.0	UNC45A
219699	1.0	1.0	-6.2	1.0	UNC5B
285175	3.0	-2.7	-4.5	1.0	UNC80
81622	1.0	1.0	-4.0	1.0	UNC93B1
85451	1.0	1.0	1.0	1.0	UNK
64718	1.8	1.0	-3.3	1.0	UNKL
51733	1.0	3.1	3.4	3.2	UPB1
26019	1.0	1.0	4.0	1.8	UPF2
11045	1.0	5.7	6.7	6.6	UPK1A
7380	1.0	1.0	4.7	1.0	UPK3A
7378	-1.8	1.0	1.0	1.0	UPP1
55245	1.0	1.0	1.0	3.4	UQCC
29796	1.0	1.0	5.4	4.8	UQCR10
7381	1.0	1.0	-3.9	1.0	UQCRB
7384	1.0	1.0	1.0	1.0	UQCRC1
7385	1.0	1.0	1.0	-2.6	UQCRC2
7388	1.0	1.0	1.0	1.0	UQCRH
27089	1.0	1.0	-2.6	1.0	UQCRQ
9875	2.7	2.3	1.0	1.0	URB1
9816	1.0	1.9	1.0	1.0	URB2
55665	-3.4	1.0	1.0	1.0	URGCP
131669	1.0	1.0	1.0	1.0	UROC1

7390	1.0	1.0	-3.5	1.0	UROS
7392	1.0	1.0	1.0	1.0	USF2
7399	1.7	-8.6	-4.3	-4.9	USH2A
83878	1.0	1.0	1.0	1.0	USHBP1
9100	1.0	1.0	-2.2	1.0	USP10
8237	1.0	1.0	3.6	4.1	USP11
9958	1.7	1.0	1.0	1.0	USP15
10600	1.0	1.0	-6.1	1.0	USP16
10869	-3.7	1.0	1.0	1.0	USP19
9099	2.8	1.0	2.9	2.6	USP2
27005	-1.7	-2.6	-2.1	-2.3	USP21
29761	1.0	1.0	1.0	1.0	USP25
57663	1.0	1.0	1.0	1.0	USP29
57602	1.0	1.0	1.0	1.0	USP36
373856	1.0	1.0	2.1	3.0	USP41
84132	1.0	1.0	-7.0	1.0	USP42
85015	-3.3	1.0	-7.8	-9.1	USP45
84196	1.0	1.0	1.0	1.0	USP48
25862	1.0	1.0	1.0	1.0	USP49
8078	1.0	-5.1	1.0	1.0	USP5
158880	1.0	1.0	-6.0	1.0	USP51
54532	1.0	1.0	1.0	1.0	USP53
9098	1.0	1.0	1.0	-6.4	USP6
51118	1.0	1.0	1.0	1.0	UTP11L
10813	1.7	1.0	1.0	-2.5	UTP14A
84135	1.0	1.0	1.0	1.0	UTP15
27340	1.0	1.0	1.0	1.0	UTP20
84294	1.0	1.0	1.0	1.0	UTP23
7402	1.0	1.0	1.0	1.0	UTRN
7404	-1.8	1.0	-3.5	1.0	UTY
7405	1.0	1.0	1.0	1.0	UVRAG
55697	-2.3	1.0	1.0	1.0	VAC14
8674	-1.5	-7.3	-9.5	1.0	VAMP4
10791	1.0	1.0	1.0	1.0	VAMP5
7407	2.9	-3.3	-3.1	1.0	VARS
57176	1.5	1.0	1.0	3.7	VARS2
22846	1.6	1.0	1.0	1.0	VASH1
79805	1.0	2.4	2.6	2.3	VASH2
7408	1.0	-2.2	1.0	1.0	VASP
57687	1.0	-5.7	1.0	1.0	VAT1L
10451	1.0	-3.0	1.0	1.0	VAV3
25806	-1.5	1.0	1.0	1.0	VAX2
7412	1.0	1.0	1.0	1.0	VCAM1
7415	1.0	1.0	1.0	1.0	VCP
51480	2.2	-2.5	1.0	1.0	VCX2
7416	1.0	-4.4	1.0	1.0	VDAC1
7417	1.5	1.0	-3.3	1.0	VDAC2
7421	1.0	1.0	1.0	1.0	VDR

7422	1.0	1.0	1.0	1.0	VEGFA
79674	1.0	-7.4	1.0	1.0	VEPH1
55591	1.0	1.0	1.0	1.0	VEZT
51442	1.0	-3.6	-5.1	1.0	VGLL1
389136	1.0	1.0	1.0	1.0	VGLL3
9686	-5.4	1.0	-4.6	1.0	VGLL4
7431	-2.5	1.0	1.0	1.0	VIM
7432	1.0	1.0	-5.2	1.0	VIP
7434	1.0	1.0	2.3	1.0	VIPR2
79001	1.0	3.8	1.0	1.0	VKORC1
8875	1.0	1.0	-4.1	1.0	VNN2
9730	2.1	1.0	-4.7	1.0	VPRBP
7441	1.0	1.0	1.0	1.0	VPREB1
55187	1.0	-5.0	-2.8	-7.3	VPS13D
57617	2.7	1.0	1.0	1.0	VPS18
26276	1.0	1.0	1.0	1.0	VPS33B
137492	-1.5	2.5	1.0	1.0	VPS37A
79720	1.0	1.0	3.6	1.0	VPS37B
155382	1.0	2.6	1.0	2.5	VPS37D
11311	2.6	6.7	1.0	2.7	VPS45
27183	1.0	1.0	1.0	1.0	VPS4A
6293	1.6	1.0	1.0	-3.6	VPS52
55275	1.0	4.9	5.5	6.1	VPS53
6944	1.0	-2.3	1.0	1.0	VPS72
7443	1.0	5.7	1.0	5.3	VRK1
7444	1.0	1.0	-6.1	1.0	VRK2
51231	1.0	1.0	1.0	2.7	VRK3
340547	1.0	1.0	1.0	4.9	VSIG1
54621	4.5	1.0	1.0	1.0	VSIG10
147645	3.3	-2.9	-3.3	-2.4	VSIG10L
23584	1.0	1.0	1.0	1.0	VSIG2
11326	-6.1	4.2	3.8	1.0	VSIG4
222008	1.0	1.0	-4.1	1.0	VSTM2A
128434	1.0	1.0	1.0	1.0	VSTM2L
196740	10.8	1.0	1.0	1.0	VSTM4
30813	2.1	1.0	1.0	1.0	VSX1
51534	-1.9	1.0	-2.3	1.0	VTA1
143187	1.0	1.0	1.0	3.0	VTI1A
200403	1.0	-6.3	1.0	1.0	VWA3B
4013	2.0	1.0	1.0	1.0	VWA5A
127731	1.0	1.0	1.0	-3.5	VWA5B1
220001	1.0	1.0	1.0	1.0	VWCE
221806	1.0	1.0	1.0	1.0	VWDE
51322	-2.4	1.0	1.0	1.0	WAC
8936	1.0	1.0	1.0	-2.6	WASF1
10163	1.0	1.0	1.8	1.0	WASF2
10810	1.0	1.0	1.0	1.0	WASF3
23559	1.0	1.0	1.0	1.0	WBP1

164684	2.2	-2.2	-4.6	-2.8	WBP2NL
114049	1.8	1.0	1.0	3.6	WBSCR22
155368	1.0	1.0	1.0	1.0	WBSCR27
57705	1.0	1.0	1.0	1.0	WDFY4
11169	1.0	1.0	2.4	3.0	WDHD1
9948	1.0	-2.2	-3.9	1.0	WDR1
55717	1.0	1.0	1.0	1.0	WDR11
116966	1.0	1.0	-4.7	1.0	WDR17
57728	1.0	1.0	1.0	-3.3	WDR19
84219	1.0	1.0	1.0	1.0	WDR24
79446	1.0	1.0	1.0	1.7	WDR25
114987	1.0	-2.1	1.0	1.0	WDR31
55339	-4.5	-2.4	-9.0	1.0	WDR33
57539	1.0	1.0	1.0	-4.8	WDR35
10785	1.0	1.0	1.0	1.0	WDR4
23160	1.0	1.0	1.0	-5.8	WDR43
9277	-1.6	-3.7	1.0	1.0	WDR46
11091	1.0	1.0	2.3	1.0	WDR5
55112	1.0	2.6	1.0	1.0	WDR60
126820	1.0	1.0	1.0	3.0	WDR63
144406	1.0	1.0	-3.6	1.0	WDR66
84942	1.0	-2.4	1.0	1.0	WDR73
79968	1.0	1.0	1.0	1.0	WDR76
79084	1.0	1.0	1.5	1.0	WDR77
79819	1.0	1.0	1.0	1.0	WDR78
80335	1.0	1.0	1.0	1.0	WDR82
84292	2.1	-2.4	1.0	1.0	WDR83
83889	1.0	1.0	1.0	-2.6	WDR87
197335	1.0	1.0	1.0	1.0	WDR90
29062	1.0	1.0	1.0	1.0	WDR91
56964	1.0	1.0	1.0	1.0	WDR93
23038	1.8	1.0	1.0	-3.0	WDTC1
7465	1.0	1.0	3.4	3.4	WEE1
140832	1.0	1.0	1.0	1.0	WFDC10A
280664	4.1	1.0	1.0	1.0	WFDC10B
259239	1.0	1.0	5.1	1.0	WFDC11
140870	1.0	1.0	-4.2	1.0	WFDC6
124857	1.0	1.0	1.0	3.1	WFIKKN2
7466	1.0	1.0	3.2	2.2	WFS1
123720	1.0	1.0	1.0	-6.0	WHAMM
7468	1.0	1.0	1.0	1.0	WHSC1
54904	1.0	1.0	1.0	1.0	WHSC1L1
84305	-1.5	1.0	1.0	1.0	WIBG
644150	1.0	-7.8	1.0	1.0	WIPF3
55062	1.0	1.0	1.0	1.0	WIPI1
26100	1.0	1.0	1.0	1.0	WIPI2
8839	1.0	1.0	1.0	1.0	WISP2
79971	1.0	2.6	1.0	1.0	WLS

65125	1.0	1.0	1.0	1.0	WNK1
65268	1.0	1.0	1.0	1.0	WNK2
65267	1.0	-6.3	-8.0	-7.6	WNK3
7473	1.0	1.0	1.0	1.0	WNT3
7476	1.0	1.0	1.0	1.0	WNT7A
7477	1.0	2.7	4.7	3.1	WNT7B
55135	3.1	1.0	1.0	1.0	WRAP53
49856	1.0	1.0	1.0	1.0	WRAP73
7485	1.0	-2.4	1.0	1.0	WRB
7486	1.0	1.0	1.0	1.0	WRN
26118	1.0	2.7	2.6	1.0	WSB1
9671	1.0	1.0	1.0	1.0	WSCD2
9589	1.6	1.0	1.0	1.0	WTAP
23286	1.0	1.0	1.0	1.0	WWC1
51741	1.0	1.0	-3.2	1.0	WWOX
11059	1.0	1.0	1.0	1.0	WWP1
11060	1.0	1.0	1.0	1.0	WWP2
25937	1.6	1.0	-8.1	1.0	WWTR1
54739	6.5	1.0	-5.0	1.0	XAF1
170626	1.0	1.0	1.0	1.0	XAGE3
170627	1.0	1.0	-2.4	-1.8	XAGE5
331	1.0	1.0	1.0	1.0	XIAP
150165	1.0	1.0	2.6	1.0	XKR3
114786	-2.1	1.0	-4.7	1.0	XKR4
389610	1.0	1.0	-3.5	1.0	XKR5
55113	1.0	5.8	6.7	3.4	XKR8
7507	1.0	3.1	1.0	1.0	XPA
7511	1.0	3.5	1.0	1.0	XPNPEP1
64328	1.7	1.0	1.0	1.0	XPO4
9213	1.9	1.0	1.0	1.0	XPR1
7516	1.0	2.6	1.0	1.0	XRCC2
7517	1.6	-4.2	-4.9	-5.1	XRCC3
2547	1.0	-5.3	-5.2	1.0	XRCC6
22803	1.5	1.0	1.0	1.0	XRN2
152002	1.0	1.0	3.7	1.0	XXYLT1
64132	-3.0	1.0	1.0	1.0	XYLT2
8565	-5.4	1.0	-6.9	1.0	YARS
54059	1.0	-3.4	1.0	-3.2	YBEY
4904	1.0	2.4	2.4	1.0	YBX1
51087	3.9	1.0	1.0	1.0	YBX2
150223	1.0	1.0	1.0	1.0	YDJC
8089	1.0	1.0	1.0	1.0	YEATS4
54432	1.0	1.0	1.0	1.0	YIPF1
25844	1.0	1.0	1.0	1.0	YIPF3
286451	1.0	3.2	3.9	3.3	YIPF6
56252	1.0	1.0	1.8	1.0	YLPM1
10730	1.0	1.0	1.0	-3.3	YME1L1
55432	-10.5	1.0	1.0	1.0	YOD1

29799	4.2	-2.8	1.0	1.0	YPEL1
388403	-1.7	1.0	-3.4	1.0	YPEL2
51646	1.0	1.0	1.6	1.0	YPEL5
91746	1.0	4.7	1.0	1.0	YTHDC1
54915	1.0	1.0	1.0	1.0	YTHDF1
51441	1.0	1.0	1.0	1.0	YTHDF2
253943	1.0	1.0	1.0	-3.1	YTHDF3
7531	1.0	1.0	1.0	1.0	YWHAE
7532	1.0	1.0	1.0	1.0	YWHAG
7533	1.0	1.0	2.2	2.3	YWHAH
10971	1.0	4.3	5.2	4.9	YWHAQ
7534	1.0	1.0	-6.1	1.0	YWHAZ
353174	1.0	1.0	1.0	1.0	ZACN
284273	1.0	2.4	2.0	1.0	ZADH2
7455	1.0	1.0	1.0	1.0	ZAN
7535	1.0	1.0	1.0	1.0	ZAP70
326340	1.0	1.0	-1.9	1.0	ZAR1
79413	1.0	1.0	1.0	1.0	ZBED2
58486	1.0	1.0	1.0	1.0	ZBED5
27107	1.0	1.0	1.0	1.0	ZBTB11
7704	1.0	-3.2	1.0	1.0	ZBTB16
57621	1.0	1.0	1.0	1.0	ZBTB2
9278	1.8	3.0	2.9	3.0	ZBTB22
9841	1.0	1.0	1.0	1.0	ZBTB24
79842	1.0	1.0	1.0	1.0	ZBTB3
27033	1.0	1.0	-6.9	-5.0	ZBTB32
84614	1.0	1.0	1.0	1.0	ZBTB37
57659	1.0	1.0	1.0	1.0	ZBTB4
23099	-2.0	1.0	-7.3	-7.0	ZBTB43
84878	1.5	1.0	-4.3	1.0	ZBTB45
92999	1.0	2.4	1.0	3.3	ZBTB47
3104	1.0	1.0	1.0	1.0	ZBTB48
9925	1.0	5.0	1.0	1.0	ZBTB5
51341	1.0	2.6	1.0	1.0	ZBTB7A
201501	2.7	-2.9	1.0	-2.7	ZBTB7C
9877	1.0	1.0	1.0	1.0	ZC3H11A
340152	1.0	1.0	2.6	1.0	ZC3H12D
124245	1.0	1.0	1.0	1.0	ZC3H18
92092	-8.6	1.0	-2.5	1.0	ZC3HAV1L
51530	-2.5	1.0	4.6	1.0	ZC3HC1
55906	1.0	1.0	1.0	2.3	ZC4H2
54819	1.0	1.0	1.0	-7.1	ZCCHC10
23318	1.0	5.2	4.7	6.6	ZCCHC11
23174	1.6	1.0	-2.1	1.0	ZCCHC14
84240	1.0	1.0	1.0	1.0	ZCCHC9
85437	1.0	1.0	-7.7	1.0	ZCRB1
55063	1.0	1.0	1.0	2.6	ZCWPW1
29800	-2.5	1.0	1.0	-4.8	ZDHHC1

54503	1.0	1.0	3.3	1.0	ZDHHC13
79683	1.0	1.0	1.0	1.0	ZDHHC14
158866	1.0	-6.2	1.0	1.0	ZDHHC15
84287	1.0	1.0	1.0	-3.5	ZDHHC16
131540	1.0	1.0	1.0	1.0	ZDHHC19
253832	-2.7	1.0	3.7	5.0	ZDHHC20
340481	1.0	1.0	1.0	1.0	ZDHHC21
254887	1.0	1.0	1.0	1.0	ZDHHC23
29801	1.0	3.1	1.0	5.3	ZDHHC8
150244	1.0	1.0	1.0	1.0	ZDHHC8P1
79752	1.0	1.0	1.0	1.0	ZFAND1
90637	1.0	1.0	1.0	1.0	ZFAND2A
130617	1.0	-2.5	1.0	1.0	ZFAND2B
93550	-2.4	-7.1	1.0	1.0	ZFAND4
57623	-12.5	4.3	-7.2	-4.5	ZFAT
196441	1.0	-4.8	1.0	-5.8	ZFC3H1
85446	1.6	-7.7	-6.9	-7.9	ZFHX2
162239	1.0	1.0	-5.1	1.0	ZFP1
57677	1.0	1.0	1.0	1.0	ZFP14
140612	1.0	1.0	1.0	1.0	ZFP28
677	1.0	3.2	1.0	1.0	ZFP36L1
678	1.0	-2.8	1.0	1.0	ZFP36L2
286128	3.2	1.0	1.0	4.5	ZFP41
643836	-1.8	1.0	1.0	1.0	ZFP62
55734	1.0	4.0	4.6	3.2	ZFP64
80829	1.0	1.0	1.0	3.7	ZFP91
51663	1.0	1.0	1.0	1.0	ZFR
23217	6.2	5.7	1.0	2.7	ZFR2
7543	1.8	1.0	1.0	1.0	ZFX
9765	-1.5	1.0	1.0	1.0	ZFYVE16
118813	1.0	1.0	1.0	1.0	ZFYVE27
57732	1.0	1.0	6.4	1.0	ZFYVE28
11244	1.0	1.0	1.0	1.0	ZHX1
22882	1.0	1.0	1.0	1.0	ZHX2
7545	1.0	1.0	1.0	-2.7	ZIC1
7547	1.0	1.0	1.0	1.0	ZIC3
284307	1.0	1.0	1.0	1.0	ZIK1
342357	1.0	1.0	1.0	1.0	ZKSCAN2
23660	1.0	1.0	1.0	1.0	ZKSCAN5
10269	1.0	5.3	1.0	4.5	ZMPSTE24
79830	1.0	1.0	1.0	1.0	ZMYM1
7750	1.0	2.5	1.0	1.0	ZMYM2
9202	1.0	1.0	-3.1	1.0	ZMYM4
9205	-3.7	1.0	1.0	1.0	ZMYM5
9204	1.0	1.0	1.0	1.0	ZMYM6
118490	1.0	1.0	1.0	1.0	ZMYND17
7556	1.0	1.0	1.0	1.0	ZNF10
163227	2.2	1.0	1.0	-5.4	ZNF100

51351	-1.8	1.0	1.0	1.0	ZNF117
7678	1.0	-5.2	1.0	-3.7	ZNF124
7693	1.0	1.0	1.0	1.0	ZNF134
7695	1.0	1.0	1.0	1.0	ZNF136
7561	1.0	1.0	1.0	-2.2	ZNF14
7700	2.2	1.0	5.9	1.0	ZNF141
7702	1.0	-2.4	-5.0	-3.7	ZNF143
7705	5.2	1.0	1.0	1.0	ZNF146
7707	5.8	1.0	1.0	1.5	ZNF148
90338	1.9	-3.1	1.0	1.0	ZNF160
7718	1.0	1.0	1.0	1.0	ZNF165
55888	2.9	-4.5	-2.2	1.0	ZNF167
169841	1.0	-3.2	1.0	1.0	ZNF169
7727	1.0	1.0	-2.8	1.0	ZNF174
7741	1.0	1.0	1.0	1.0	ZNF187
7567	-2.7	-2.7	1.0	1.0	ZNF19
7745	-1.6	1.0	1.0	1.0	ZNF192
7753	1.0	1.0	1.0	1.0	ZNF202
7988	1.0	1.0	3.6	3.0	ZNF212
7760	1.7	1.0	1.0	1.0	ZNF213
51222	1.0	1.0	1.0	1.0	ZNF219
7768	1.0	1.0	1.0	-6.8	ZNF225
7769	1.0	1.0	1.0	1.0	ZNF226
353355	1.0	-7.6	-11.8	-7.6	ZNF233
10780	1.0	1.0	-3.9	1.0	ZNF234
7776	1.0	1.0	1.0	2.8	ZNF236
7572	1.0	2.6	1.0	1.0	ZNF24
219749	1.0	1.0	1.0	1.0	ZNF25
56242	1.0	1.0	-6.3	1.0	ZNF253
8882	1.0	1.0	2.9	1.0	ZNF259
10127	1.0	1.0	1.0	1.0	ZNF263
9422	-1.7	1.0	3.0	1.0	ZNF264
10795	1.0	1.0	1.0	1.0	ZNF268
10838	1.0	3.3	1.0	1.0	ZNF275
92822	1.0	1.0	1.0	1.0	ZNF276
7576	-15.3	1.0	1.0	2.2	ZNF28
129025	1.0	1.0	2.5	1.0	ZNF280A
140883	1.0	1.0	1.0	1.0	ZNF280B
54816	1.0	-10.3	1.0	1.0	ZNF280D
342909	1.0	1.0	-5.2	-5.5	ZNF284
26974	1.0	1.0	1.0	1.0	ZNF285
49854	1.0	-3.5	-3.8	1.0	ZNF295
7551	1.0	2.3	3.3	2.6	ZNF3
91975	-2.7	-6.1	-7.4	-6.7	ZNF300
55900	1.0	1.0	1.0	4.2	ZNF302
162967	1.0	1.0	1.0	5.4	ZNF320
79692	1.0	1.0	1.0	1.0	ZNF322
25799	1.0	3.3	1.8	1.0	ZNF324

27309	1.0	1.0	1.0	-2.5	ZNF330
55422	1.0	3.6	1.0	2.6	ZNF331
84449	3.0	1.0	2.9	1.0	ZNF333
55713	-7.8	1.0	1.0	1.0	ZNF334
80778	1.0	1.0	1.0	1.0	ZNF34
79175	-1.8	1.0	-4.2	1.0	ZNF343
30832	1.0	-5.2	1.0	1.0	ZNF354C
22891	1.0	1.0	1.0	1.0	ZNF365
100129482	1.0	1.0	1.0	1.0	ZNF37BP
171017	1.0	1.0	3.5	1.0	ZNF384
25946	-2.7	1.0	1.0	1.0	ZNF385A
79750	1.0	6.5	3.5	3.7	ZNF385D
84124	1.0	-2.8	-6.1	1.0	ZNF394
252884	-2.2	-4.3	-4.8	-4.8	ZNF396
55628	2.3	5.9	1.0	1.0	ZNF407
84330	1.0	1.0	-3.1	1.0	ZNF414
79744	2.0	1.0	1.0	1.0	ZNF419
79088	-2.0	1.0	-3.0	1.0	ZNF426
126299	1.0	2.7	2.3	1.0	ZNF428
353088	1.0	1.0	3.8	1.0	ZNF429
54925	1.0	1.0	2.5	1.0	ZNF434
90594	1.0	1.0	1.0	1.0	ZNF439
126068	1.0	1.0	-7.2	1.0	ZNF441
79973	1.0	-3.1	-2.7	-3.2	ZNF442
7596	1.0	1.0	1.0	1.0	ZNF45
26036	5.0	1.0	-2.5	-2.7	ZNF451
92283	1.0	1.0	1.0	1.0	ZNF461
90333	1.0	1.0	1.0	3.9	ZNF468
84627	-1.7	1.0	1.0	1.0	ZNF469
83744	1.0	1.0	1.0	1.0	ZNF484
90649	1.0	1.0	1.0	-4.5	ZNF486
57474	7.0	1.0	1.0	1.0	ZNF490
284443	1.0	-2.7	-3.1	1.0	ZNF493
84838	1.0	1.0	1.0	1.0	ZNF496
162968	1.0	1.0	1.0	3.3	ZNF497
221785	1.0	1.0	1.0	1.0	ZNF498
84858	1.0	1.0	1.0	1.0	ZNF503
253264	1.0	1.0	1.0	1.0	ZNF503-AS1
22847	1.0	1.0	1.0	1.0	ZNF507
118472	1.0	-4.2	1.0	-4.1	ZNF511
57473	1.0	3.1	3.1	1.0	ZNF512B
162655	1.0	1.0	-5.6	1.0	ZNF519
147807	1.0	1.0	1.0	1.0	ZNF524
116115	1.0	1.0	1.0	1.0	ZNF526
84436	1.0	1.0	1.0	1.0	ZNF528
55205	1.0	1.0	1.0	1.0	ZNF532
84215	1.0	3.2	1.0	2.4	ZNF541
147947	1.0	1.0	1.0	1.0	ZNF542

284306	1.0	-3.9	-4.4	-6.3	ZNF547
115196	1.0	-5.4	1.0	1.0	ZNF554
147741	1.0	1.0	1.0	1.0	ZNF560
147837	1.0	-2.6	1.0	1.0	ZNF563
148266	1.0	1.0	-7.3	-6.0	ZNF569
51276	1.0	1.0	-2.8	1.0	ZNF571
137209	1.0	1.0	1.0	1.0	ZNF572
284346	1.0	4.6	4.4	1.0	ZNF575
84765	1.0	1.0	-3.3	1.0	ZNF577
147660	1.0	1.0	1.0	1.0	ZNF578
147948	1.0	-3.4	1.0	-3.5	ZNF582
147949	1.0	1.0	1.0	-3.2	ZNF583
201514	1.0	3.9	5.9	1.0	ZNF584
199704	1.0	1.0	1.0	1.0	ZNF585A
54807	1.0	2.3	2.5	2.5	ZNF586
84914	-6.2	1.0	1.0	1.0	ZNF587
9640	1.0	1.0	4.5	4.4	ZNF592
152687	1.0	-3.7	1.0	1.0	ZNF595
90850	1.0	2.8	2.3	2.4	ZNF598
148103	1.0	1.0	1.0	-3.1	ZNF599
162966	1.0	1.0	6.8	1.0	ZNF600
57507	1.0	-7.9	1.0	1.0	ZNF608
79898	1.0	-4.5	1.0	1.0	ZNF613
284370	1.0	1.0	1.0	1.0	ZNF615
285267	1.0	1.0	1.0	2.1	ZNF619
253639	1.0	1.0	-4.6	-5.8	ZNF620
90441	1.9	1.0	1.0	1.0	ZNF622
9831	1.0	1.0	1.0	1.0	ZNF623
57547	1.0	1.0	1.0	1.0	ZNF624
90589	1.9	-2.4	-2.4	-2.4	ZNF625
199777	1.0	1.0	1.0	1.0	ZNF626
199692	1.0	1.0	1.0	1.0	ZNF627
57232	1.0	1.0	1.0	1.0	ZNF630
27332	-10.7	1.0	-9.5	-6.4	ZNF638
158506	1.0	1.0	2.7	1.0	ZNF645
9726	1.0	1.0	1.0	1.0	ZNF646
79027	1.0	-3.0	1.0	1.0	ZNF655
26149	-15.3	1.0	1.0	1.0	ZNF658
144348	1.0	3.3	1.0	1.0	ZNF664
79788	2.6	1.0	4.1	1.0	ZNF665
63934	1.0	1.0	1.0	1.0	ZNF667
641339	1.0	1.0	1.0	1.0	ZNF674
163223	-1.6	1.0	-3.5	1.0	ZNF676
340252	2.6	-2.5	1.0	1.0	ZNF680
148213	1.0	1.0	1.0	1.0	ZNF681
257101	1.0	1.0	1.0	1.0	ZNF683
7620	1.0	-2.5	1.0	1.0	ZNF69
55657	1.0	3.8	3.2	1.0	ZNF692

90874	1.0	1.0	4.1	1.0	ZNF697
80139	1.0	1.0	1.0	5.1	ZNF703
619279	1.0	1.0	1.8	1.0	ZNF704
286075	1.0	1.0	1.0	1.0	ZNF707
163051	1.0	1.0	1.0	1.0	ZNF709
58491	1.0	1.0	1.0	1.0	ZNF71
349075	5.4	5.8	7.2	1.0	ZNF713
100131827	1.0	1.0	1.0	1.0	ZNF717
124411	1.0	1.0	1.0	1.0	ZNF720
100129842	1.0	1.0	1.0	-2.7	ZNF737
155061	-1.5	1.0	1.0	1.0	ZNF746
65988	5.0	1.0	9.0	1.0	ZNF747
7627	1.0	1.0	1.0	1.0	ZNF75A
7626	1.0	1.0	1.0	1.0	ZNF75D
7629	1.0	1.0	1.0	4.8	ZNF76
388561	2.2	3.6	1.0	1.0	ZNF761
92595	1.0	1.7	1.0	1.0	ZNF764
79724	1.0	1.0	1.0	1.0	ZNF768
54989	1.0	2.1	1.0	1.0	ZNF770
51333	1.0	1.0	1.0	-2.6	ZNF771
374928	1.0	1.0	-5.9	1.0	ZNF773
27153	1.0	1.0	1.0	1.0	ZNF777
163131	1.0	1.0	-7.8	1.0	ZNF780B
100289678	1.0	1.0	1.0	7.0	ZNF783
388507	1.0	1.0	1.0	1.0	ZNF788
126375	1.0	1.0	1.0	1.0	ZNF792
7554	1.0	1.0	3.2	3.3	ZNF8
168850	1.6	1.0	-2.7	-2.2	ZNF800
347344	1.0	1.0	1.0	1.0	ZNF81
729648	1.0	1.0	1.0	1.0	ZNF812
374899	1.0	1.0	1.0	1.0	ZNF829
128611	1.0	1.0	1.0	1.0	ZNF831
55778	1.0	1.0	1.0	1.0	ZNF839
284371	1.0	1.0	1.0	1.0	ZNF841
91664	1.0	2.0	4.4	1.0	ZNF845
7652	1.0	1.0	1.0	1.0	ZNF99
57169	2.1	1.0	1.0	1.0	ZNFX1
10467	1.0	-3.2	-2.7	1.0	ZNHIT1
84133	1.0	1.0	1.0	1.0	ZNRF3
148066	1.0	1.0	1.0	-4.1	ZNRF4
22917	1.0	1.0	1.0	1.0	ZP1
7783	1.0	1.0	-4.4	1.0	ZP2
7784	1.0	1.0	1.0	1.0	ZP3
11055	1.0	1.0	1.0	1.0	ZPBP
84083	-9.5	1.0	1.0	1.0	ZRANB3
8233	1.0	1.0	1.0	1.0	ZRSR2
9753	1.0	1.0	6.4	1.0	ZSCAN12
80345	1.0	-4.1	-2.6	1.0	ZSCAN16

100101467	1.0	1.0	-4.2	-4.1	ZSCAN30
79149	-1.9	1.0	1.0	-5.9	ZSCAN5A
221302	12.1	1.0	1.0	7.9	ZUFSP
9183	1.0	1.0	2.3	1.0	ZW10
