# Supplementary material for: Comparative efficacy analysis of anti-microbial peptides, LL-37 and indolicidin upon conjugation with CNT, in human monocytes
Source: J Nanobiotechnology. 2017 Jun 12;15:44. doi: 10.1186/s12951-017-0278-1 (PMC5469186; doi:10.1186/s12951-017-0278-1)
Supplement: Supplementary file 3 — Additional file 3: Table S3. Expression of genes in terms of fold changes following treatment free and conjugated Indolicidin and other conditions. [file 12951_2017_278_MOESM3_ESM.pdf]

Table S3: Expression of genes in terms of fold changes following treatment free and conjugated Indolicidin and other conditions

ProbeName	**Fold Change Values**				GeneSymbol	n	EntrezGeneID
	CNT	CNT + Indo	CNT-Indo	Indo-20			
A_23_P38816	2.3	2.0	1.3	1.1	A1BG	Homo sapiens alpha-1-B glycoprotein (A1BG), mRNA [NM_130786]	1
A_24_P721699	-1.3	2.0	-1.5	1.5	A1BG-AS1	Homo sapiens A1BG antisense RNA 1 (non-protein coding) (A1BG-AS1), non-coding RNA [NR_015380]	503538
A_23_P104224	3.4	2.1	2.5	2.6	A1CF	Homo sapiens APOBEC1 complementation factor (A1CF), transcript variant 3, mRNA [NM_138933]	29974

A_23_P57570	-1.0	-8.4	-1.0	-1.0	A4GALT	Homo sapiens alpha 1,4-galactosyltransferase (A4GALT), mRNA [NM_017436]	53947
A_23_P58046	1.0	-3.1	1.2	1.1	A4GNT	Homo sapiens alpha-1,4-N-acetylglucosaminyltransferase (A4GNT), mRNA [NM_016161]	51146
A_24_P299271	-1.5	-2.4	-1.7	-1.4	AAK1	Homo sapiens AP2 associated kinase 1 (AAK1), mRNA [NM_014911]	22848
A_23_P152527	2.2	2.1	-1.2	-2.4	AANAT	Homo sapiens aralkylamine N-acetyltransferase (AANAT), transcript variant 2, mRNA [NM_001088]	15

A_24_P172990	-1.7	-1.9	-2.1	-1.3	AARS	Homo sapiens alanyl-tRNA synthetase (AARS), mRNA [NM_001605]	16
A_23_P207414	-8.7	1.9	1.6	-1.7	AARSD1	Homo sapiens alanyl-tRNA synthetase domain containing 1 (AARSD1), transcript variant 2, mRNA [NM_025267]	80755
A_24_P307195	-2.4	-1.0	-16.8	-1.3	AASDH	Homo sapiens aminoadipate-semialdehyde dehydrogenase (AASDH), mRNA [NM_181806]	132949

A_24_P184230	-2.2	2.1	1.8	1.3	AASDHPPT	Homo sapiens aminoadipate-semialdehyde dehydrogenase-phosphopantetheinyl transferase (AASDHPPT), mRNA [NM_015423]	60496
A_23_P159169	4.3	4.3	3.2	1.5	AATK	Homo sapiens apoptosis-associated tyrosine kinase (AATK), transcript variant 1, mRNA [NM_001080395]	9625
A_23_P152505	2.1	2.8	2.5	1.4	ABAT	Homo sapiens 4-aminobutyrate aminotransferase (ABAT), nuclear gene encoding mitochondrial protein, transcript variant 2, mRNA [NM_000663]	18

A_24_P235429	1.9	2.1	2.0	2.0	ABCA1	Homo sapiens ATP-binding cassette, sub-family A (ABC1), member 1 (ABCA1), mRNA [NM_005502]	19
A_23_P89422	-18.0	-1.6	-1.9	-2.0	ABCA10	Homo sapiens ATP-binding cassette, sub-family A (ABC1), member 10 (ABCA10), mRNA [NM_080282]	10349
A_23_P160940	-2.4	-2.0	1.6	1.0	ABCA4	Homo sapiens ATP-binding cassette, sub-family A (ABC1), member 4 (ABCA4), mRNA [NM_000350]	24

A_23_P39481	1.6	2.5	2.6	1.3	ABCA7	Homo sapiens ATP-binding cassette, sub-family A (ABC1), member 7 (ABCA7), mRNA [NM_019112]	10347
A_23_P118615	-9.0	1.0	1.3	1.1	ABCA8	Homo sapiens ATP-binding cassette, sub-family A (ABC1), member 8 (ABCA8), mRNA [NM_007168]	10351
A_24_P324449	-13.4	1.0	-1.0	-1.7	ABCB10	Homo sapiens ATP-binding cassette, sub-family B (MDR/TAP), member 10 (ABCB10), nuclear gene encoding mitochondrial protein, mRNA [NM_012089]	23456

A_32_P23624	3.0	1.3	1.0	2.8	ABCB10	Homo sapiens ATP-binding cassette, sub-family B (MDR/TAP), member 10 (ABCB10), nuclear gene encoding mitochondrial protein, mRNA [NM_012089]	23456
A_23_P111662	1.1	-2.4	-1.0	1.0	ABCB5	Homo sapiens ATP-binding cassette, sub-family B (MDR/TAP), member 5 (ABCB5), transcript variant 2, mRNA [NM_178559]	340273

A_23_P171258	23.7	10.7	9.5	2.2	ABCB7	Homo sapiens ATP-binding cassette, sub-family B (MDR/TAP), member 7 (ABCB7), nuclear gene encoding mitochondrial protein, mRNA [NM_004299]	22
A_23_P128094	1.0	1.0	-1.0	2.6	ABCB9	Homo sapiens ATP-binding cassette, sub-family B (MDR/TAP), member 9 (ABCB9), transcript variant 1, mRNA [NM_019625]	23457

A_23_P166207	3.5	3.7	2.6	-1.2	ABCC13	Homo sapiens ATP-binding cassette, sub-family C (CFTR/MRP), member 13, pseudogene (ABCC13), transcript variant A, non-coding RNA [NR_003087]	150000
A_23_P307570	-1.0	-1.0	-3.7	1.0	ABCC13	Homo sapiens ATP-binding cassette, sub-family C (CFTR/MRP), member 13, pseudogene (ABCC13), transcript variant A, non-coding RNA [NR_003087]	150000

A_23_P25559	-2.2	1.5	1.4	1.0	ABCC4	Homo sapiens ATP-binding cassette, sub-family C (CFTR/MRP), member 4 (ABCC4), transcript variant 1, mRNA [NM_005845]	10257
A_23_P212665	-1.4	-3.0	-1.0	1.0	ABCC5	Homo sapiens ATP-binding cassette, sub-family C (CFTR/MRP), member 5 (ABCC5), transcript variant 1, mRNA [NM_005688]	10057

A_24_P178503	-28.7	-2.0	-2.1	-1.9	ABCC9	Homo sapiens ATP-binding cassette, sub-family C (CFTR/MRP), member 9 (ABCC9), transcript variant SUR2A, mRNA [NM_005691]	10060
A_23_P388124	-1.0	-4.4	-1.1	-1.1	ABCD2	Homo sapiens ATP-binding cassette, sub-family D (ALD), member 2 (ABCD2), mRNA [NM_005164]	225
A_24_P221154	4.3	1.0	1.0	1.0	ABCD3	Homo sapiens ATP-binding cassette, sub-family D (ALD), member 3 (ABCD3), transcript variant 1, mRNA [NM_002858]	5825

A_23_P37327	-8.7	-1.1	-1.1	-1.1	ABCD4	Homo sapiens ATP-binding cassette, sub-family D (ALD), member 4 (ABCD4), transcript variant 1, mRNA [NM_005050]	5826
A_23_P41380	2.5	-1.0	-2.0	1.0	ABCE1	Homo sapiens ATP-binding cassette, sub-family E (OABP), member 1 (ABCE1), transcript variant 1, mRNA [NM_002940]	6059
A_32_P108474	-3.3	-2.5	1.1	1.0	ABCE1	Homo sapiens ATP-binding cassette, sub-family E (OABP), member 1 (ABCE1), transcript variant 1, mRNA [NM_002940]	6059

A_24_P251688	2.3	-2.1	2.0	-1.4	ABCF3	Homo sapiens ATP-binding cassette, sub-family F (GCN20), member 3 (ABCF3), mRNA [NM_018358]	55324
A_23_P166297	4.8	2.4	5.6	7.4	ABCG1	Homo sapiens ATP-binding cassette, sub-family G (WHITE), member 1 (ABCG1), transcript variant 5, mRNA [NM_207627]	9619
A_23_P18713	5.8	6.5	7.0	1.4	ABCG2	Homo sapiens ATP-binding cassette, sub-family G (WHITE), member 2 (ABCG2), mRNA [NM_004827]	9429

A_24_P355626	1.8	3.1	2.6	2.6	ABCG4	Homo sapiens ATP-binding cassette, sub-family G (WHITE), member 4 (ABCG4), transcript variant 1, mRNA [NM_022169]	64137
A_23_P17242	-2.8	1.1	1.0	-1.6	ABHD1	Homo sapiens abhydrolase domain containing 1 (ABHD1), mRNA [NM_032604]	84696
A_24_P918739	-1.1	-1.3	-1.8	-3.0	ABHD1	Homo sapiens abhydrolase domain containing 1, mRNA (cDNA clone IMAGE:4812401), with apparent retained intron. [BC028378]	84696

A_24_P308590	2.4	2.3	2.2	2.3	ABHD10	Homo sapiens abhydrolase domain containing 10 (ABHD10), mRNA [NM_018394]	55347
A_24_P268662	-1.0	-1.0	-1.0	-4.1	ABHD10	Homo sapiens abhydrolase domain containing 10 (ABHD10), mRNA [NM_018394]	55347
A_23_P92213	-1.4	-2.7	-1.4	1.3	ABHD10	Homo sapiens abhydrolase domain containing 10 (ABHD10), mRNA [NM_018394]	55347
A_23_P8558	-1.3	-1.6	-1.3	-2.6	ABHD11	Homo sapiens abhydrolase domain containing 11 (ABHD11), nuclear gene encoding mitochondrial protein, transcript variant 1, mRNA [NM_148912]	83451

A_24_P61464	-4.1	-1.1	1.2	-1.2	ABHD13	Homo sapiens abhydrolase domain containing 13 (ABHD13), mRNA [NM_032859]	84945
A_23_P69267	1.1	2.9	-1.5	2.1	ABHD14A	Homo sapiens abhydrolase domain containing 14A (ABHD14A), mRNA [NM_015407]	25864
A_23_P155417	-2.4	1.0	1.4	1.1	ABHD14B	Homo sapiens abhydrolase domain containing 14B (ABHD14B), transcript variant 1, mRNA [NM_032750]	84836
A_32_P465065	1.4	1.1	-2.3	1.5	ABHD16B	Homo sapiens abhydrolase domain containing 16B (ABHD16B), mRNA [NM_080622]	140701

A_24_P924915	-1.0	3.8	1.4	-1.7	ABHD2	Homo sapiens abhydrolase domain containing 2 (ABHD2), transcript variant 1, mRNA [NM_007011]	11057
A_24_P924920	1.3	1.3	2.3	-1.0	ABHD2	Homo sapiens abhydrolase domain containing 2 (ABHD2), transcript variant 1, mRNA [NM_007011]	11057
A_23_P140591	1.8	-2.0	-1.2	-1.3	ABHD2	Homo sapiens abhydrolase domain containing 2 (ABHD2), transcript variant 1, mRNA [NM_007011]	11057
A_23_P305759	3.5	-1.7	1.1	1.0	ABHD3	Homo sapiens abhydrolase domain containing 3 (ABHD3), mRNA [NM_138340]	171586

A_23_P50857	4.9	6.6	7.3	3.8	ABHD8	Homo sapiens abhydrolase domain containing 8 (ABHD8), mRNA [NM_024527]	79575
A_23_P79692	2.1	4.7	2.6	-1.3	ABI2	Homo sapiens abl-interactor 2 (ABI2), mRNA [NM_005759]	10152
A_23_P141429	-1.4	2.0	2.2	1.8	ABI3	Homo sapiens ABI family, member 3 (ABI3), transcript variant 1, mRNA [NM_016428]	51225
A_24_P282416	2.6	2.5	1.8	4.3	ABL1	Homo sapiens c-abl oncogene 1, non-receptor tyrosine kinase (ABL1), transcript variant b, mRNA [NM_007313]	25

A_32_P139894	3.0	2.8	2.2	3.0	ABL2	Homo sapiens v-abl Abelson murine leukemia viral oncogene homolog 2 (ABL2), transcript variant b, mRNA [NM_007314]	27
A_23_P112646	1.5	2.1	1.3	-1.1	ABO	Homo sapiens ABO blood group (transferase A, alpha 1-3-N-acetylgalactosaminyltransferase; transferase B, alpha 1-3-galactosyltransferase) (ABO), mRNA [NM_020469]	28
A_23_P503182	1.5	2.4	1.0	1.0	ABR	Homo sapiens active BCR-related gene (ABR), transcript variant 1, mRNA [NM_021962]	29

A_23_P313482	2.1	2.9	2.2	2.1	ABRA	Homo sapiens actin-binding Rho activating protein (ABRA), mRNA [NM_139166]	137735
A_24_P200297	1.5	3.0	1.9	-1.0	ABTB1	Homo sapiens ankyrin repeat and BTB (POZ) domain containing 1 (ABTB1), transcript variant 2, mRNA [NM_172027]	80325
A_23_P89799	-1.3	1.3	1.1	2.3	ACAA2	Homo sapiens acetyl-CoA acyltransferase 2 (ACAA2), nuclear gene encoding mitochondrial protein, mRNA [NM_006111]	10449

A_23_P141434	-3.7	-1.1	-4.0	-1.5	ACACA	Homo sapiens acetyl-CoA carboxylase alpha (ACACA), transcript variant 2, mRNA [NM_198839]	31
A_23_P25348	-1.3	1.1	-3.9	-7.6	ACAD10	Homo sapiens acyl-CoA dehydrogenase family, member 10 (ACAD10), transcript variant 2, mRNA [NM_025247]	80724
A_24_P227450	2.5	-1.2	-1.2	2.9	ACAD10	Homo sapiens acyl-CoA dehydrogenase family, member 10 (ACAD10), transcript variant 2, mRNA [NM_025247]	80724

A_24_P8284	2.6	-1.2	2.4	2.4	ACAD10	Homo sapiens acyl-CoA dehydrogenase family, member 10 (ACAD10), transcript variant 2, mRNA [NM_025247]	80724
A_23_P47426	-1.3	-2.1	1.1	-1.9	ACAD8	Homo sapiens acyl-CoA dehydrogenase family, member 8 (ACAD8), nuclear gene encoding mitochondrial protein, mRNA [NM_014384]	27034

A_23_P132405	-2.3	1.0	-1.1	-1.0	ACAD9	Homo sapiens acyl-CoA dehydrogenase family, member 9 (ACAD9), nuclear gene encoding mitochondrial protein, transcript variant 1, mRNA [NM_014049]	28976
A_24_P191656	3.3	5.3	-1.1	1.8	ACAD9	Homo sapiens acyl-CoA dehydrogenase family, member 9 (ACAD9), nuclear gene encoding mitochondrial protein, transcript variant 1, mRNA [NM_014049]	28976

A_24_P234242	-2.2	-1.5	-1.6	1.4	ACADL	Homo sapiens acyl-CoA dehydrogenase, long chain (ACADL), nuclear gene encoding mitochondrial protein, mRNA [NM_001608]	33
A_23_P96761	1.1	-2.0	1.1	1.1	ACADM	Homo sapiens acyl-CoA dehydrogenase, C-4 to C-12 straight chain (ACADM), nuclear gene encoding mitochondrial protein, transcript variant 1, mRNA [NM_000016]	34

A_23_P65022	1.9	3.7	3.7	3.7	ACADS	Homo sapiens acyl-CoA dehydrogenase, C-2 to C-3 short chain (ACADS), nuclear gene encoding mitochondrial protein, mRNA [NM_000017]	35
A_24_P189516	-3.2	1.0	-1.0	-1.6	ACADSB	Homo sapiens acyl-CoA dehydrogenase, short/branched chain (ACADSB), nuclear gene encoding mitochondrial protein, mRNA [NM_001609]	36

A_32_P31945	-1.0	-1.0	-1.1	2.3	ACADSB	Homo sapiens acyl-CoA dehydrogenase, short/branched chain (ACADSB), nuclear gene encoding mitochondrial protein, mRNA [NM_001609]	36
A_24_P942002	-6.7	1.0	-1.8	1.1	ACAP2	Homo sapiens ArfGAP with coiled-coil, ankyrin repeat and PH domains 2 (ACAP2), mRNA [NM_012287]	23527
A_23_P23630	1.4	2.5	3.5	2.0	ACAP3	Homo sapiens ArfGAP with coiled-coil, ankyrin repeat and PH domains 3 (ACAP3), mRNA [NM_030649]	116983

A_23_P12231	-1.2	-2.7	-1.0	-3.5	ACAP3	Homo sapiens ArfGAP with coiled-coil, ankyrin repeat and PH domains 3 (ACAP3), mRNA [NM_030649]	116983
A_24_P203678	1.2	1.5	2.2	1.1	ACAT1	Homo sapiens acetyl-CoA acetyltransferase 1 (ACAT1), nuclear gene encoding mitochondrial protein, mRNA [NM_000019]	38
A_24_P171182	2.9	2.8	-1.5	2.8	ACBD3	Homo sapiens acyl-CoA binding domain containing 3 (ACBD3), mRNA [NM_022735]	64746

A_24_P366122	2.8	1.8	-1.6	-1.0	ACBD4	Homo sapiens acyl-CoA binding domain containing 4 (ACBD4), transcript variant 2, mRNA [NM_024722]	79777
A_24_P175563	1.1	1.0	-6.6	1.0	ACBD5	Homo sapiens acyl-CoA binding domain containing 5 (ACBD5), transcript variant 1, mRNA [NM_145698]	91452
A_23_P52127	1.0	-1.0	-1.0	-5.0	ACBD6	Homo sapiens acyl-CoA binding domain containing 6 (ACBD6), mRNA [NM_032360]	84320

A_32_P159612	-1.3	-1.1	1.3	-2.8	ACCN1	Homo sapiens amiloride-sensitive cation channel 1, neuronal (ACCN1), transcript variant MDEG2, mRNA [NM_183377]	40
A_23_P93658	-3.1	-3.2	-3.2	-3.3	ACCN3	Homo sapiens amiloride-sensitive cation channel 3 (ACCN3), transcript variant 3, mRNA [NM_020322]	9311
A_24_P405375	-1.1	1.3	2.1	1.1	ACCN4	Homo sapiens amiloride-sensitive cation channel 4, pituitary (ACCN4), transcript variant 1, mRNA [NM_018674]	55515

A_23_P24586	-5.0	1.0	-1.0	-1.0	ACCS	Homo sapiens 1-aminocyclopropane-1-carboxylate synthase homolog (Arabidopsis)(non-functional) (ACCS), transcript variant 1, mRNA [NM_032592]	84680
A_23_P26375	1.4	4.0	-1.4	-1.8	ACD	Homo sapiens adrenocortical dysplasia homolog (mouse) (ACD), transcript variant 2, mRNA [NM_022914]	65057
A_23_P38235	-1.0	-1.4	-1.1	-2.2	ACE	Homo sapiens angiotensin I converting enzyme (peptidyl-dipeptidase A) 1 (ACE), transcript variant 1, mRNA [NM_000789]	1636

A_23_P14389	-3.3	-2.5	-5.9	-3.3	ACIN1	Homo sapiens apoptotic chromatin condensation inducer 1 (ACIN1), transcript variant 1, mRNA [NM_014977]	22985
A_23_P376727	5.0	4.5	4.5	4.6	ACMSD	Homo sapiens aminocarboxymuconate semialdehyde decarboxylase (ACMSD), mRNA [NM_138326]	130013
A_23_P9416	-1.1	-2.5	-1.3	-1.4	ACO1	Homo sapiens aconitase 1, soluble (ACO1), mRNA [NM_002197]	48
A_24_P945165	-1.1	-3.6	-1.2	-1.1	ACOT11	acyl-CoA thioesterase 11 [Source:HGNC Symbol;Acc:18156] [ENST00000371316]	26027

A_23_P31116	2.4	-1.5	-1.4	1.4	ACOT13	Homo sapiens acyl-CoA thioesterase 13 (ACOT13), transcript variant 1, mRNA [NM_018473]	55856
A_23_P143218	2.8	1.7	1.1	-1.6	ACOT8	Homo sapiens acyl-CoA thioesterase 8 (ACOT8), mRNA [NM_005469]	10005
A_23_P125624	2.5	1.5	1.5	1.7	ACOT9	Homo sapiens acyl-CoA thioesterase 9 (ACOT9), nuclear gene encoding mitochondrial protein, transcript variant 1, mRNA [NM_001037171]	23597
A_23_P10182	-2.9	1.3	1.2	-5.7	ACOX2	Homo sapiens acyl-CoA oxidase 2, branched chain (ACOX2), mRNA [NM_003500]	8309

A_24_P354451	-12.7	-1.2	-1.4	-1.4	ACOX3	Homo sapiens acyl-CoA oxidase 3, pristanoyl (ACOX3), transcript variant 1, mRNA [NM_003501]	8310
A_23_P316381	1.6	1.1	-2.2	-1.0	ACOX3	Homo sapiens acyl-CoA oxidase 3, pristanoyl (ACOX3), transcript variant 1, mRNA [NM_003501]	8310
A_23_P138776	-2.0	1.1	1.5	-3.2	ACP2	Homo sapiens acid phosphatase 2, lysosomal (ACP2), transcript variant 1, mRNA [NM_001610]	53
A_23_P138782	-1.2	2.5	1.8	1.0	ACP2	Homo sapiens acid phosphatase 2, lysosomal (ACP2), transcript variant 1, mRNA [NM_001610]	53

A_23_P142075	-7.5	-1.5	-1.6	-2.5	ACP5	Homo sapiens acid phosphatase 5, tartrate resistant (ACP5), transcript variant 4, mRNA [NM_001611]	54
A_23_P160240	1.0	-1.0	-1.1	-2.3	ACP6	Homo sapiens acid phosphatase 6, lysophosphatidic (ACP6), mRNA [NM_016361]	51205
A_24_P37589	2.1	-1.6	1.0	1.2	ACPP	Homo sapiens acid phosphatase, prostate (ACPP), transcript variant 1, mRNA [NM_001099]	55
A_23_P330419	5.3	1.9	1.1	-1.8	ACPT	Homo sapiens acid phosphatase, testicular (ACPT), mRNA [NM_033068]	93650

A_32_P191262	1.4	2.0	-1.6	1.2	ACR	Homo sapiens acrosin (ACR), mRNA [NM_001097]	49
A_23_P40734	1.2	1.1	-2.1	1.2	ACR	Homo sapiens acrosin (ACR), mRNA [NM_001097]	49
A_23_P151120	1.0	-5.3	-1.1	2.0	ACRBP	Homo sapiens acrosin binding protein (ACRBP), mRNA [NM_032489]	84519
A_23_P171237	1.0	-1.0	-3.1	1.7	ACRC	Homo sapiens acidic repeat containing (ACRC), mRNA [NM_052957]	93953
A_23_P54488	1.3	-6.4	1.0	2.0	ACSBG1	Homo sapiens acyl-CoA synthetase bubblegum family member 1 (ACSBG1), transcript variant 1, mRNA [NM_015162]	23205

A_23_P131050	1.3	1.8	-1.0	2.3	ACSBG2	Homo sapiens acyl-CoA synthetase bubblegum family member 2 (ACSBG2), mRNA [NM_030924]	81616
A_23_P4190	3.4	-1.6	-1.3	1.1	ACSF2	Homo sapiens acyl-CoA synthetase family member 2 (ACSF2), mRNA [NM_025149]	80221
A_23_P110212	2.2	2.1	2.9	1.7	ACSL1	Homo sapiens acyl-CoA synthetase long-chain family member 1 (ACSL1), mRNA [NM_001995]	2180
A_23_P217564	1.3	2.4	1.3	1.0	ACSL4	Homo sapiens acyl-CoA synthetase long-chain family member 4 (ACSL4), transcript variant 1, mRNA [NM_004458]	2182

A_24_P941526	-1.2	-1.3	-2.0	-2.7	ACSL6	Homo sapiens acyl-CoA synthetase long-chain family member 6 (ACSL6), transcript variant 2, mRNA [NM_001009185]	23305
A_23_P415806	1.1	-3.6	-1.3	1.1	ACSL6	acyl-CoA synthetase long-chain family member 6 [Source:HGNC Symbol;Acc:16496] [ENST00000489047]	23305
A_23_P66328	-7.9	1.7	-2.0	-4.7	ACSM2B	Homo sapiens acyl-CoA synthetase medium-chain family member 2B (ACSM2B), nuclear gene encoding mitochondrial protein, transcript variant 1, mRNA [NM_182617]	348158

A_24_P367776	-1.4	-1.4	-1.4	-13.2	ACSM5	Homo sapiens acyl-CoA synthetase medium-chain family member 5, mRNA (cDNA clone IMAGE:4069865), complete cds. [BC016703]	54988
A_23_P120594	6.8	1.5	1.5	2.1	ACSS1	Homo sapiens acyl-CoA synthetase short-chain family member 1 (ACSS1), nuclear gene encoding mitochondrial protein, mRNA [NM_032501]	84532
A_24_P940831	2.6	2.6	2.5	2.6	ACSS1	Homo sapiens mRNA for FLJ00013 protein, partial cds. [AK024424]	84532

A_24_P156295	-4.2	1.0	-1.0	1.1	ACSS2	Homo sapiens acyl-CoA synthetase short-chain family member 2 (ACSS2), transcript variant 1, mRNA [NM_018677]	55902
A_23_P339119	1.1	-3.0	1.5	1.0	ACSS3	Homo sapiens acyl-CoA synthetase short-chain family member 3 (ACSS3), mRNA [NM_024560]	79611
A_23_P1102	-5.1	2.0	-15.3	-1.1	ACTA1	Homo sapiens actin, alpha 1, skeletal muscle (ACTA1), mRNA [NM_001100]	58

A_23_P150053	1.6	2.0	-1.2	1.1	ACTA2	Homo sapiens actin, alpha 2, smooth muscle, aorta (ACTA2), transcript variant 2, mRNA [NM_001613]	59
A_24_P6903	-1.4	1.1	-2.2	-1.0	ACTBL2	Homo sapiens actin, beta-like 2 (ACTBL2), mRNA [NM_001017992]	345651
A_23_P205894	-1.0	-2.6	-1.2	-1.2	ACTC1	Homo sapiens actin, alpha, cardiac muscle 1 (ACTC1), mRNA [NM_005159]	70
A_24_P121535	1.8	2.1	1.4	1.4	ACTC1	Homo sapiens actin, alpha, cardiac muscle 1 (ACTC1), mRNA [NM_005159]	70

A_23_P39955	-3.0	-1.6	-2.1	-1.7	ACTG2	Homo sapiens actin, gamma 2, smooth muscle, enteric (ACTG2), transcript variant 1, mRNA [NM_001615]	72
A_23_P31984	-1.0	-1.2	1.7	2.1	ACTL7A	Homo sapiens actin-like 7A (ACTL7A), mRNA [NM_006687]	10881
A_23_P9186	3.5	1.9	2.0	1.4	ACTL7B	Homo sapiens actin-like 7B (ACTL7B), mRNA [NM_006686]	10880
A_24_P107695	2.6	2.1	1.5	2.8	ACTN1	Homo sapiens actinin, alpha 1 (ACTN1), transcript variant 2, mRNA [NM_001102]	87

A_23_P105957	-1.1	2.4	1.2	1.6	ACTN1	Homo sapiens actinin, alpha 1 (ACTN1), transcript variant 2, mRNA [NM_001102]	87
A_23_P315241	-6.8	1.2	1.6	1.3	ACTN4	Homo sapiens actinin, alpha 4 (ACTN4), mRNA [NM_004924]	81
A_24_P261326	2.0	2.6	2.1	2.1	ACTR1A	Homo sapiens ARP1 actin-related protein 1 homolog A, centractin alpha (yeast) (ACTR1A), mRNA [NM_005736]	10121
A_23_P28279	2.3	1.1	-1.1	1.3	ACTR1B	Homo sapiens ARP1 actin-related protein 1 homolog B, centractin beta (yeast) (ACTR1B), mRNA [NM_005735]	10120

A_24_P276947	2.3	2.2	1.7	-1.4	ACTR2	Homo sapiens ARP2 actin-related protein 2 homolog (yeast) (ACTR2), transcript variant 1, mRNA [NM_001005386]	10097
A_24_P105771	-3.0	-1.0	-1.8	1.3	ACTR3	Homo sapiens ARP3 actin-related protein 3 homolog (yeast) (ACTR3), mRNA [NM_005721]	10096
A_23_P108785	-1.1	-4.7	-2.5	1.5	ACTR3	Homo sapiens ARP3 actin-related protein 3 homolog (yeast) (ACTR3), mRNA [NM_005721]	10096

A_24_P303594	-1.6	-9.8	-1.5	-1.8	ACTR3B	Homo sapiens ARP3 actin-related protein 3 homolog B (yeast) (ACTR3B), transcript variant 2, mRNA [NM_001040135]	57180
A_23_P123193	1.7	1.7	2.1	1.7	ACTR3B	Homo sapiens ARP3 actin-related protein 3 homolog B (yeast) (ACTR3B), transcript variant 1, mRNA [NM_020445]	57180
A_24_P76635	-2.0	-3.7	-4.3	-1.0	ACTR3C	Homo sapiens ARP3 actin-related protein 3 homolog C (yeast) (ACTR3C), transcript variant 1, mRNA [NM_001164458]	653857

A_23_P165952	1.1	-2.8	-1.0	1.0	ACTR5	Homo sapiens ARP5 actin-related protein 5 homolog (yeast) (ACTR5), mRNA [NM_024855]	79913
A_24_P131697	-5.6	1.2	-2.6	-6.7	ACTR8	Homo sapiens ARP8 actin-related protein 8 homolog (yeast) (ACTR8), mRNA [NM_022899]	93973
A_23_P147311	3.0	1.4	3.1	-40.2	ACTRT1	Homo sapiens actin-related protein T1 (ACTRT1), mRNA [NM_138289]	139741
A_23_P79221	-2.1	-2.1	1.4	1.4	ACVR1	Homo sapiens activin A receptor, type I (ACVR1), transcript variant 1, mRNA [NM_001105]	90

A_23_P99036	1.3	1.6	1.5	-7.7	ACVR1B	Homo sapiens activin A receptor, type IB (ACVR1B), transcript variant 1, mRNA [NM_004302]	91
A_24_P341897	-1.3	2.1	-2.0	1.5	ACVR1B	Homo sapiens activin A receptor, type IB (ACVR1B), transcript variant 1, mRNA [NM_004302]	91
A_32_P190944	2.6	-1.1	4.0	2.8	ACVR1C	Homo sapiens activin A receptor, type IC (ACVR1C), transcript variant 1, mRNA [NM_145259]	130399
A_23_P109950	-2.1	-1.6	-1.9	1.4	ACVR2B	Homo sapiens activin A receptor, type IIB (ACVR2B), mRNA [NM_001106]	93

A_24_P324783	1.1	-2.6	-1.1	1.0	ACVRL1	Homo sapiens activin A receptor type II-like 1 (ACVRL1), transcript variant 1, mRNA [NM_000020]	94
A_23_P163143	1.2	-1.5	-3.3	1.1	ACYP1	Homo sapiens acylphosphatase 1, erythrocyte (common) type (ACYP1), transcript variant 2, mRNA [NM_203488]	97
A_23_P159424	1.4	2.7	1.3	-1.0	ACYP2	H.sapiens mRNA for acylphosphatase, muscle type (MT) isoenzyme. [X84195]	98
A_23_P256988	1.1	-2.8	1.0	1.1	ACYP2	Homo sapiens acylphosphatase 2, muscle type (ACYP2), mRNA [NM_138448]	98

A_23_P210482	1.5	-1.1	-3.5	-1.5	ADA	Homo sapiens adenosine deaminase (ADA), mRNA [NM_000022]	100
A_32_P221694	1.0	-4.3	-1.1	-1.0	ADAL	Homo sapiens adenosine deaminase-like (ADAL), transcript variant 1, mRNA [NM_001159280]	161823
A_23_P207345	1.1	-2.3	-2.2	1.1	ADAM11	Homo sapiens ADAM metallopeptidase domain 11 (ADAM11), mRNA [NM_002390]	4185
A_23_P350512	-1.0	-2.1	-1.0	-1.0	ADAM12	Homo sapiens ADAM metallopeptidase domain 12 (ADAM12), transcript variant 2, mRNA [NM_021641]	8038

A_32_P129621	1.0	1.5	-2.9	1.0	ADAM17	Homo sapiens ADAM metallopeptidase domain 17 (ADAM17), mRNA [NM_003183]	6868
A_24_P157053	2.0	2.0	2.0	1.1	ADAM17	ADAM metallopeptidase domain 17 [Source:HGNC Symbol;Acc:195] [ENST00000310823]	6868
A_23_P72527	7.1	9.2	9.0	8.8	ADAM18	Homo sapiens ADAM metallopeptidase domain 18 (ADAM18), transcript variant 1, mRNA [NM_014237]	8749
A_23_P136832	1.1	-2.3	1.1	1.0	ADAM2	Homo sapiens ADAM metallopeptidase domain 2 (ADAM2), mRNA [NM_001464]	2515

A_23_P215625	1.0	-6.3	1.1	-1.6	ADAM22	Homo sapiens ADAM metallopeptidase domain 22 (ADAM22), transcript variant 1, mRNA [NM_021723]	53616
A_24_P243741	-1.0	-1.1	-5.2	-1.0	ADAM22	Homo sapiens ADAM metallopeptidase domain 22 (ADAM22), transcript variant 5, mRNA [NM_021721]	53616
A_23_P21758	-4.9	1.7	1.2	1.0	ADAM28	Homo sapiens ADAM metallopeptidase domain 28 (ADAM28), transcript variant 1, mRNA [NM_014265]	10863

A_23_P44665	6.3	6.1	2.8	5.3	ADAM32	Homo sapiens ADAM metallopeptidase domain 32 (ADAM32), mRNA [NM_145004]	203102
A_23_P502343	5.9	-1.2	2.7	1.7	ADAM33	Homo sapiens ADAM metallopeptidase domain 33 (ADAM33), transcript variant 1, mRNA [NM_025220]	80332
A_24_P230026	-3.0	-1.8	-2.0	-1.9	ADAM5P	Homo sapiens ADAM metallopeptidase domain 5, pseudogene (ADAM5P), non-coding RNA [NR_001448]	255926

A_24_P592260	3.7	1.6	-1.6	3.2	ADAM6	Homo sapiens ADAM metallopeptidase domain 6 (pseudogene) (ADAM6), non-coding RNA [NR_002224]	8755
A_23_P256425	-2.2	1.3	1.3	-2.7	ADAMDEC1	Homo sapiens ADAM-like, decysin 1 (ADAMDEC1), transcript variant 1, mRNA [NM_014479]	27299
A_23_P211039	1.1	2.0	1.9	1.1	ADAMTS1	Homo sapiens ADAM metallopeptidase with thrombospondin type 1 motif, 1 (ADAMTS1), mRNA [NM_006988]	9510

A_23_P44648	-2.8	1.1	3.4	1.1	ADAMTS12	Homo sapiens ADAM metallopeptidase with thrombospondin type 1 motif, 12 (ADAMTS12), mRNA [NM_030955]	81792
A_23_P393645	-1.3	-1.5	-1.4	-2.0	ADAMTS13	Homo sapiens ADAM metallopeptidase with thrombospondin type 1 motif, 13 (ADAMTS13), transcript variant 1, mRNA [NM_139025]	11093

A_24_P172304	-2.2	-2.2	-1.8	-2.1	ADAMTS13	Homo sapiens ADAM metallopeptidase with thrombospondin type 1 motif, 13 (ADAMTS13), transcript variant 2, mRNA [NM_139027]	11093
A_24_P923832	1.8	-1.7	-1.7	-3.1	ADAMTS13	Homo sapiens ADAM metallopeptidase with thrombospondin type 1 motif, 13 (ADAMTS13), transcript variant 2, mRNA [NM_139027]	11093

A_23_P202558	1.1	1.2	-5.7	-1.5	ADAMTS14	Homo sapiens ADAM metallopeptidase with thrombospondin type 1 motif, 14 (ADAMTS14), transcript variant 1, mRNA [NM_139155]	140766
A_24_P275073	-7.4	1.2	1.1	1.8	ADAMTS14	Homo sapiens ADAM metallopeptidase with thrombospondin type 1 motif, 14 (ADAMTS14), transcript variant 1, mRNA [NM_139155]	140766

A_23_P52749	1.0	1.1	-11.3	-1.2	ADAMTS15	Homo sapiens ADAM metallopeptidase with thrombospondin type 1 motif, 15 (ADAMTS15), mRNA [NM_139055]	170689
A_24_P69084	5.8	2.7	5.1	4.1	ADAMTS16	Homo sapiens ADAM metallopeptidase with thrombospondin type 1 motif, 16 (ADAMTS16), mRNA [NM_139056]	170690
A_23_P112916	4.2	3.0	1.4	2.0	ADAMTS17	Homo sapiens ADAM metallopeptidase with thrombospondin type 1 motif, 17 (ADAMTS17), mRNA [NM_139057]	170691

A_23_P15004	-1.0	-1.1	1.1	-2.7	ADAMTS18	Homo sapiens ADAM metallopeptidase with thrombospondin type 1 motif, 18 (ADAMTS18), mRNA [NM_199355]	170692
A_23_P321307	1.0	-1.5	2.3	2.0	ADAMTS2	Homo sapiens ADAM metallopeptidase with thrombospondin type 1 motif, 2 (ADAMTS2), transcript variant 2, mRNA [NM_021599]	9509
A_23_P144521	-3.3	-1.5	1.2	-1.7	ADAMTS3	Homo sapiens ADAM metallopeptidase with thrombospondin type 1 motif, 3 (ADAMTS3), mRNA [NM_014243]	9508

A_23_P40415	1.0	1.1	-8.0	-1.1	ADAMTS5	Homo sapiens ADAM metallopeptidase with thrombospondin type 1 motif, 5 (ADAMTS5), mRNA [NM_007038]	11096
A_23_P37632	1.1	2.0	1.0	1.0	ADAMTS7	Homo sapiens zinc metalloprotease ADAMTS7 (ADAMTS7) mRNA, complete cds. [AF140675]	11173
A_24_P756494	1.0	-2.3	-4.5	-4.0	ADAMTS9-AS	Homo sapiens cDNA FLJ25194 fis, clone REC04095. [AK057923]	100507098
A_23_P348304	-1.1	-2.0	-4.4	-1.2	ADAMTSL1	ADAMTS-like 1 [Source:HGNC Symbol;Acc:14632] [ENST00000380566]	92949

A_23_P20697	1.5	2.2	1.6	1.9	ADAMTSL2	Homo sapiens ADAMTS-like 2 (ADAMTSL2), transcript variant 1, mRNA [NM_014694]	9719
A_23_P115011	-1.0	-1.0	-2.8	-1.0	ADAMTSL4	Homo sapiens ADAMTS-like 4 (ADAMTSL4), transcript variant 1, mRNA [NM_019032]	54507
A_24_P315821	-1.4	-1.1	-1.3	-3.0	ADAMTSL5	Homo sapiens ADAMTS-like 5 (ADAMTSL5), mRNA [NM_213604]	339366
A_24_P22939	2.1	-2.9	-3.0	-3.8	ADAMTSL5	Homo sapiens ADAMTS-like 5 (ADAMTSL5), mRNA [NM_213604]	339366
A_23_P145865	1.4	-6.6	-1.1	-2.0	ADAP1	Homo sapiens ArfGAP with dual PH domains 1 (ADAP1), mRNA [NM_006869]	11033

A_23_P49816	1.2	-19.0	-3.9	-1.2	ADAP2	Homo sapiens ArfGAP with dual PH domains 2 (ADAP2), mRNA [NM_018404]	55803
A_23_P361336	4.2	4.1	4.1	4.2	ADARB1	Homo sapiens adenosine deaminase, RNA-specific, B1 (ADARB1), transcript variant 2, mRNA [NM_015833]	104
A_24_P936556	1.3	-3.7	-1.2	-1.1	ADARB1	adenosine deaminase, RNA-specific, B1 [Source:HGNC Symbol;Acc:226] [ENST00000460734]	104
A_24_P48139	1.1	-1.2	-4.4	-2.3	ADAT1	Homo sapiens adenosine deaminase, tRNA-specific 1 (ADAT1), transcript variant 1, mRNA [NM_012091]	23536

A_23_P134014	-1.0	-1.1	-5.6	-1.1	ADAT2	Homo sapiens adenosine deaminase, tRNA-specific 2 (ADAT2), mRNA [NM_182503]	134637
A_24_P186204	1.0	-1.0	-2.2	-1.1	ADAT2	Homo sapiens adenosine deaminase, tRNA-specific 2 (ADAT2), mRNA [NM_182503]	134637
A_24_P82848	1.7	-1.3	1.5	-33.1	ADAT3	Homo sapiens adenosine deaminase, tRNA-specific 3 (ADAT3), mRNA [NM_138422]	113179
A_23_P25945	1.2	1.3	3.7	2.3	ADCK1	Homo sapiens aarF domain containing kinase 1 (ADCK1), transcript variant 1, mRNA [NM_020421]	57143

A_32_P405973	-4.5	1.1	-1.2	-1.1	ADCY2	Homo sapiens adenylate cyclase 2 (brain) (ADCY2), mRNA [NM_020546]	108
A_23_P381261	-1.2	-1.4	-1.2	-5.1	ADCY4	Homo sapiens adenylate cyclase 4 (ADCY4), transcript variant 2, mRNA [NM_139247]	196883
A_23_P169993	1.3	1.3	1.2	-2.5	ADCY8	Homo sapiens adenylate cyclase 8 (brain) (ADCY8), mRNA [NM_001115]	114
A_23_P49443	2.5	3.4	2.8	4.3	ADCY9	Homo sapiens adenylate cyclase 9 (ADCY9), mRNA [NM_001116]	115
A_24_P935103	-1.0	-1.1	-4.5	-1.1	ADCY9	Homo sapiens adenylate cyclase 9 (ADCY9), mRNA [NM_001116]	115

A_24_P335781	1.5	2.8	-1.1	-1.6	ADCYAP1	Homo sapiens adenylate cyclase activating polypeptide 1 (pituitary) (ADCYAP1), transcript variant 2, mRNA [NM_001117]	116
A_24_P109272	1.0	-2.3	-1.0	-1.0	ADD1	adducin 1 (alpha) [Source:HGNC Symbol;Acc:243] [ENST00000513762]	118
A_24_P117866	-2.7	1.1	-4.7	-1.7	ADD1	Homo sapiens adducin 1 (alpha) (ADD1), transcript variant 1, mRNA [NM_001119]	118
A_23_P209856	-2.2	-1.9	-1.2	-2.7	ADD2	Homo sapiens adducin 2 (beta) (ADD2), transcript variant 4, mRNA [NM_017488]	119

A_23_P202435	-2.3	-1.4	-6.4	1.5	ADD3	Homo sapiens adducin 3 (gamma) (ADD3), transcript variant 1, mRNA [NM_016824]	120
A_24_P940469	-3.0	2.1	2.1	2.0	ADH1B	alcohol dehydrogenase 1B (class I), beta polypeptide [Source:HGNC Symbol;Acc:250] [ENST00000305046]	125
A_23_P81158	-1.0	1.6	-1.6	2.5	ADH1C	Homo sapiens alcohol dehydrogenase 1C (class I), gamma polypeptide (ADH1C), mRNA [NM_000669]	126

A_32_P198399	-1.2	2.2	-2.2	1.9	ADH5	Homo sapiens alcohol dehydrogenase 5 (class III), chi polypeptide (ADH5), mRNA [NM_000671]	128
A_24_P25550	-1.5	1.6	-2.4	1.5	ADH6	Homo sapiens alcohol dehydrogenase 6 (class V) (ADH6), transcript variant 1, mRNA [NM_001102470]	130
A_23_P92562	1.9	-2.3	-1.0	-1.0	ADH7	Homo sapiens alcohol dehydrogenase 7 (class IV), mu or sigma polypeptide (ADH7), transcript variant 2, mRNA [NM_000673]	131

A_32_P52911	-2.1	1.1	-1.7	-1.0	ADI1	Homo sapiens acireductone dioxygenase 1 (ADI1), mRNA [NM_018269]	55256
A_32_P423691	1.1	1.1	-2.4	1.1	ADIG	Homo sapiens adipogenin (ADIG), mRNA [NM_001018082]	149685
A_23_P46627	-1.8	-1.0	-2.1	-1.1	ADIPOR1	Homo sapiens adiponectin receptor 1 (ADIPOR1), transcript variant 1, mRNA [NM_015999]	51094
A_24_P13376	1.4	1.6	1.2	2.4	ADIPOR2	Homo sapiens adiponectin receptor 2 (ADIPOR2), mRNA [NM_024551]	79602
A_23_P112596	2.3	-1.1	-1.8	2.2	ADK	Homo sapiens adenosine kinase (ADK), transcript variant 1, mRNA [NM_001123]	132

A_23_P254179	1.4	-2.2	1.9	-1.0	ADNP	Homo sapiens activity-dependent neuroprotector homeobox (ADNP), transcript variant 1, mRNA [NM_015339]	23394
A_23_P202345	15.8	15.8	14.9	2.8	ADO	Homo sapiens 2-aminoethanethiol (cysteamine) dioxygenase (ADO), mRNA [NM_032804]	84890
A_23_P74299	-3.4	-1.3	-1.2	-1.3	ADORA1	Homo sapiens adenosine A1 receptor (ADORA1), transcript variant 1, mRNA [NM_000674]	134
A_23_P109436	2.2	1.7	-1.1	1.1	ADORA2A	Homo sapiens adenosine A2a receptor (ADORA2A), mRNA [NM_000675]	135

A_24_P237270	1.8	1.8	2.8	1.0	ADORA2A	Homo sapiens adenosine A2a receptor (ADORA2A), mRNA [NM_000675]	135
A_23_P126540	2.0	2.1	2.1	1.1	ADORA3	Homo sapiens adenosine A3 receptor (ADORA3), transcript variant 1, mRNA [NM_020683]	140
A_23_P14716	1.9	-1.2	2.1	-1.1	ADPGK	Homo sapiens ADP-dependent glucokinase (ADPGK), transcript variant 1, mRNA [NM_031284]	83440
A_23_P371145	-3.8	-3.0	-1.7	-2.1	ADPRHL1	Homo sapiens ADP-ribosylhydrolase like 1 (ADPRHL1), transcript variant 1, mRNA [NM_138430]	113622

A_23_P34568	-1.3	-2.8	-1.9	-2.1	ADPRHL2	Homo sapiens ADP-ribosylhydrolase like 2 (ADPRHL2), nuclear gene encoding mitochondrial protein, mRNA [NM_017825]	54936
A_23_P8938	1.8	1.7	3.0	-1.1	ADRA1A	Homo sapiens adrenergic, alpha-1A-, receptor (ADRA1A), transcript variant 4, mRNA [NM_033304]	148
A_23_P378926	1.8	1.8	3.6	1.0	ADRA2B	Homo sapiens adrenergic, alpha-2B-, receptor (ADRA2B), mRNA [NM_000682]	151
A_23_P168993	-4.6	-1.3	-1.5	-2.6	ADRB3	Homo sapiens adrenergic, beta-3-, receptor (ADRB3), mRNA [NM_000025]	155

A_23_P68665	2.2	1.8	1.1	1.6	ADRM1	Homo sapiens adhesion regulating molecule 1 (ADRM1), transcript variant 1, mRNA [NM_007002]	11047
A_23_P859	-1.0	1.4	2.2	1.3	ADSS	Homo sapiens adenylosuccinate synthase (ADSS), mRNA [NM_001126]	159
A_23_P157299	-1.1	-1.1	-3.8	-1.1	AEBP1	Homo sapiens AE binding protein 1 (AEBP1), mRNA [NM_001129]	165
A_23_P145916	1.8	-1.3	1.3	-3.1	AEBP1	Homo sapiens AE binding protein 1 (AEBP1), mRNA [NM_001129]	165

A_23_P116913	-2.6	4.1	-1.7	1.1	AEBP2	Homo sapiens AE binding protein 2 (AEBP2), transcript variant 1, mRNA [NM_153207]	121536
A_23_P206077	1.0	-6.3	1.0	1.1	AEN	Homo sapiens apoptosis enhancing nuclease (AEN), mRNA [NM_022767]	64782
A_23_P341312	1.4	1.6	-30.8	1.4	AES	Homo sapiens amino-terminal enhancer of split (AES), transcript variant 1, mRNA [NM_198969]	166
A_23_P72387	1.3	-1.1	1.1	2.4	AFAP1	Homo sapiens actin filament associated protein 1 (AFAP1), transcript variant a, mRNA [NM_001134647]	60312

A_32_P178092	-3.3	1.0	-1.0	1.0	AFAP1-AS1	Homo sapiens AFAP1 antisense RNA 1 (non-protein coding) (AFAP1-AS1), antisense RNA [NR_026892]	84740
A_24_P33770	-1.2	-1.2	-2.6	-1.2	AFAP1L2	Homo sapiens actin filament associated protein 1-like 2 (AFAP1L2), transcript variant 2, mRNA [NM_032550]	84632
A_23_P29897	-2.6	1.3	-1.2	-1.0	AFF1	Homo sapiens AF4/FMR2 family, member 1 (AFF1), transcript variant 2, mRNA [NM_005935]	4299

A_24_P414332	1.0	-1.7	-2.6	-1.1	AFF1	Homo sapiens AF4/FMR2 family, member 1 (AFF1), transcript variant 2, mRNA [NM_005935]	4299
A_24_P942636	5.6	1.4	5.6	5.7	AFF4	Homo sapiens AF4/FMR2 family, member 4 (AFF4), mRNA [NM_014423]	27125
A_24_P8318	5.0	-2.2	1.5	3.5	AFF4	Homo sapiens AF4/FMR2 family, member 4 (AFF4), mRNA [NM_014423]	27125

A_23_P355289	-2.9	-2.4	-3.9	-6.6	AFG3L1P	Homo sapiens AFG3 ATPase family gene 3-like 1 (S. cerevisiae), pseudogene (AFG3L1P), transcript variant 1, non-coding RNA [NR_003226]	172
A_32_P117338	-2.1	1.0	-5.7	1.4	AFG3L2	Homo sapiens AFG3 ATPase family gene 3-like 2 (S. cerevisiae) (AFG3L2), nuclear gene encoding mitochondrial protein, mRNA [NM_006796]	10939

A_24_P867342	1.3	2.0	1.3	1.6	AFMID	Homo sapiens arylformamidase (AFMID), transcript variant 3, non-coding RNA [NR_027083]	125061
A_24_P122862	-3.0	1.0	-1.0	1.1	AFTPH	Homo sapiens aftiphilin (AFTPH), transcript variant 2, mRNA [NM_017657]	54812
A_23_P428382	-1.5	1.3	1.6	2.6	AFTPH	Homo sapiens aftiphilin (AFTPH), transcript variant 1, mRNA [NM_203437]	54812
A_23_P7099	5.3	-1.0	-1.1	-1.1	AGA	Homo sapiens aspartylglucosaminidase (AGA), transcript variant 1, mRNA [NM_000027]	175

A_32_P55161	2.4	1.9	-1.2	2.6	AGAP1	Homo sapiens ArfGAP with GTPase domain, ankyrin repeat and PH domain 1 (AGAP1), transcript variant 1, mRNA [NM_001037131]	116987
A_32_P124493	-2.6	-3.7	-6.2	-1.3	AGAP10	PREDICTED: Homo sapiens ArfGAP with GTPase domain, ankyrin repeat and PH domain 10 (AGAP10), mRNA [XM_001714786]	728127
A_24_P244495	-2.8	-35.5	-1.0	1.0	AGAP2	Homo sapiens ArfGAP with GTPase domain, ankyrin repeat and PH domain 2 (AGAP2), transcript variant 1, mRNA [NM_001122772]	116986

A_24_P333364	2.6	1.5	1.2	1.0	AGAP3	Homo sapiens ArfGAP with GTPase domain, ankyrin repeat and PH domain 3 (AGAP3), transcript variant 2, mRNA [NM_001042535]	116988
A_32_P211363	1.1	1.0	-1.0	-2.9	AGAP8	Homo sapiens ArfGAP with GTPase domain, ankyrin repeat and PH domain 8 (AGAP8), mRNA [NM_001077686]	728404
A_32_P8881	-1.3	1.1	-1.5	-3.6	AGAP8	Homo sapiens ArfGAP with GTPase domain, ankyrin repeat and PH domain 8 (AGAP8), mRNA [NM_001077686]	728404

A_32_P167705	1.0	-2.4	-1.0	-1.0	AGBL2	Homo sapiens ATP/GTP binding protein-like 2 (AGBL2), mRNA [NM_024783]	79841
A_32_P82623	-1.3	-2.3	-3.3	-2.3	AGBL3	Homo sapiens ATP/GTP binding protein-like 3 (AGBL3), mRNA [NM_178563]	340351
A_23_P154466	1.6	2.5	2.3	1.4	AGBL5	Homo sapiens ATP/GTP binding protein-like 5 (AGBL5), transcript variant 3, mRNA [NM_001035507]	60509
A_23_P311640	-1.0	-1.1	1.0	-23.7	AGFG2	Homo sapiens ArfGAP with FG repeats 2 (AGFG2), mRNA [NM_006076]	3268

A_23_P103720	-1.2	-1.2	-7.1	-1.3	AGMAT	Homo sapiens agmatine ureohydrolase (agmatinase) (AGMAT), mRNA [NM_024758]	79814
A_23_P218743	-2.1	1.0	1.0	1.2	AGPAT3	Homo sapiens 1-acylglycerol-3-phosphate O-acyltransferase 3 (AGPAT3), transcript variant 1, mRNA [NM_020132]	56894
A_23_P329452	-2.4	1.2	1.2	-2.3	AGPAT4	1-acylglycerol-3-phosphate O-acyltransferase 4 (lysophosphatidic acid acyltransferase, delta) [Source:HGNC Symbol;Acc:20885] [ENST00000436279]	56895

A_24_P29876	-2.6	-3.7	-1.8	-3.2	AGPAT5	Homo sapiens 1-acylglycerol-3-phosphate O-acyltransferase 5 (lysophosphatidic acid acyltransferase, epsilon) (AGPAT5), mRNA [NM_018361]	55326
A_23_P82839	1.2	-2.7	-1.8	-1.0	AGPAT6	Homo sapiens 1-acylglycerol-3-phosphate O-acyltransferase 6 (lysophosphatidic acid acyltransferase, zeta) (AGPAT6), mRNA [NM_178819]	137964
A_23_P69810	-1.4	-1.4	-1.5	-2.2	AGPAT9	Homo sapiens 1-acylglycerol-3-phosphate O-acyltransferase 9 (AGPAT9), mRNA [NM_032717]	84803

A_24_P902100	3.3	1.5	2.9	2.3	AGPS	Homo sapiens alkylglycerone phosphate synthase (AGPS), mRNA [NM_003659]	8540
A_23_P42811	1.0	-6.6	-1.0	-1.4	AGR3	Homo sapiens anterior gradient homolog 3 (Xenopus laevis) (AGR3), mRNA [NM_176813]	155465
A_24_P358462	3.1	2.9	1.4	2.0	AGRN	Homo sapiens agrin (AGRN), mRNA [NM_198576]	375790
A_23_P502320	-1.1	3.4	-1.3	1.4	AGRP	Homo sapiens agouti related protein homolog (mouse) (AGRP), mRNA [NM_001138]	181

A_23_P169278	1.1	-2.1	-1.1	-1.0	AGTPBP1	Homo sapiens ATP/GTP binding protein 1 (AGTPBP1), mRNA [NM_015239]	23287
A_24_P350832	9.0	9.5	8.8	5.1	AGTPBP1	Homo sapiens mRNA for hypothetical protein, clone C91-V61. [AJ437018]	23287
A_23_P158596	1.0	1.0	-1.1	-2.6	AGTRAP	Homo sapiens angiotensin II receptor-associated protein (AGTRAP), transcript variant 4, mRNA [NM_001040196]	57085
A_23_P83590	-1.5	1.3	2.3	-2.5	AGXT	Homo sapiens alanine-glyoxylate aminotransferase (AGXT), mRNA [NM_000030]	189

A_23_P257231	-1.2	-14.0	1.1	-1.5	AGXT2L1	Homo sapiens alanine-glyoxylate aminotransferase 2-like 1 (AGXT2L1), transcript variant 1, mRNA [NM_031279]	64850
A_23_P41888	-1.0	-2.2	-1.1	-1.0	AGXT2L2	Homo sapiens alanine-glyoxylate aminotransferase 2-like 2 (AGXT2L2), mRNA [NM_153373]	85007
A_23_P63584	-3.0	-2.5	1.8	2.2	AHCTF1	Homo sapiens AT hook containing transcription factor 1 (AHCTF1), mRNA [NM_015446]	25909
A_23_P17575	-3.3	-1.0	-1.1	1.0	AHCY	Homo sapiens adenosylhomocysteinase (AHCY), transcript variant 1, mRNA [NM_000687]	191

A_24_P57367	2.0	3.2	3.0	3.2	AHCY	Homo sapiens adenosylhomocysteinase (AHCY), transcript variant 1, mRNA [NM_000687]	191
A_24_P213710	1.8	4.4	6.5	2.8	AHI1	Homo sapiens Abelson helper integration site 1 (AHI1), transcript variant 2, mRNA [NM_017651]	54806
A_24_P38143	-1.5	-1.7	-1.8	-8.7	AHI1	Homo sapiens Abelson helper integration site 1 (AHI1), transcript variant 2, mRNA [NM_017651]	54806
A_24_P912684	-2.6	-1.3	-1.3	-1.4	AHNAK	Homo sapiens AHNAK nucleoprotein (AHNAK), transcript variant 1, mRNA [NM_001620]	79026

A_23_P426636	1.4	2.3	-1.8	-1.3	AHNAK	Homo sapiens AHNAK nucleoprotein (AHNAK), transcript variant 1, mRNA [NM_001620]	79026
A_24_P178618	1.1	-1.2	-2.1	1.1	AHNAK2	Homo sapiens AHNAK nucleoprotein 2 (AHNAK2), mRNA [NM_138420]	113146
A_23_P215566	-4.1	1.5	1.5	1.0	AHR	Homo sapiens aryl hydrocarbon receptor (AHR), mRNA [NM_001621]	196
A_23_P344400	-2.7	1.0	-1.0	1.0	AHRR	Homo sapiens aryl-hydrocarbon receptor repressor (AHRR), transcript variant 1, mRNA [NM_020731]	57491

A_23_P372467	3.4	1.0	-4.1	1.6	AHSA2	Homo sapiens AHA1, activator of heat shock 90kDa protein ATPase homolog 2 (yeast) (AHSA2), mRNA [NM_152392]	130872
A_23_P135486	19.1	20.9	18.6	12.4	AHSP	Homo sapiens alpha hemoglobin stabilizing protein (AHSP), mRNA [NM_016633]	51327
A_23_P392384	1.0	-6.7	-6.7	1.0	AIF1L	Homo sapiens allograft inflammatory factor 1-like (AIF1L), transcript variant 3, mRNA [NM_001185095]	83543

A_23_P138567	-1.0	2.0	1.1	1.3	AIFM2	Homo sapiens apoptosis-inducing factor, mitochondrion-associated, 2 (AIFM2), transcript variant 2, mRNA [NM_032797]	84883
A_23_P93431	-6.4	1.1	1.3	1.0	AIG1	Homo sapiens androgen-induced 1 (AIG1), mRNA [NM_016108]	51390
A_23_P360329	1.5	-1.2	-2.0	-2.2	AIM1L	Homo sapiens absent in melanoma 1-like (AIM1L), mRNA [NM_001039775]	55057

A_24_P354523	-2.7	-5.4	-1.8	-6.5	AIMP1	Homo sapiens aminoacyl tRNA synthetase complex-interacting multifunctional protein 1 (AIMP1), transcript variant 1, mRNA [NM_004757]	9255
A_23_P121686	-1.1	-1.1	-1.0	-2.3	AIMP1	Homo sapiens aminoacyl tRNA synthetase complex-interacting multifunctional protein 1 (AIMP1), transcript variant 1, mRNA [NM_004757]	9255
A_23_P75380	4.2	4.5	1.6	3.6	AIP	Homo sapiens aryl hydrocarbon receptor interacting protein (AIP), mRNA [NM_003977]	9049

A_23_P100935	-2.9	-1.0	-1.6	-1.0	AIPL1	Homo sapiens aryl hydrocarbon receptor interacting protein-like 1 (AIPL1), transcript variant 1, mRNA [NM_014336]	23746
A_24_P254355	-1.0	2.0	1.8	1.3	AIRE	Homo sapiens autoimmune regulator (AIRE), transcript variant AIRE-2, mRNA [NM_000658]	326
A_23_P68740	3.3	2.9	-3.2	1.6	AIRE	Homo sapiens autoimmune regulator (AIRE), transcript variant AIRE-2, mRNA [NM_000658]	326
A_24_P278192	1.1	-1.0	-2.8	-1.7	AK1	Homo sapiens adenylate kinase 1 (AK1), mRNA [NM_000476]	203

A_23_P217088	2.4	-3.0	1.2	1.4	AK1	Homo sapiens adenylate kinase 1 (AK1), mRNA [NM_000476]	203
A_32_P207360	1.1	-3.2	-1.3	-2.0	AK2	Homo sapiens adenylate kinase 2 (AK2), nuclear gene encoding mitochondrial protein, transcript variant 2, mRNA [NM_013411]	204
A_24_P500891	1.0	1.4	-1.1	-2.3	AK2	Homo sapiens adenylate kinase 2 (AK2), nuclear gene encoding mitochondrial protein, transcript variant 2, mRNA [NM_013411]	204

A_24_P247106	-17.6	-2.3	-2.6	-5.2	AK3	Homo sapiens adenylate kinase 3 (AK3), nuclear gene encoding mitochondrial protein, transcript variant 1, mRNA [NM_016282]	50808
A_32_P108655	-1.1	1.0	-2.0	1.1	AK4	Homo sapiens adenylate kinase 4 (AK4), nuclear gene encoding mitochondrial protein, transcript variant 5, mRNA [NM_001005353]	205
A_23_P200015	1.3	-1.0	1.4	-2.3	AK5	Homo sapiens adenylate kinase 5 (AK5), transcript variant 1, mRNA [NM_174858]	26289

A_24_P931097	-6.0	1.1	1.0	-5.9	AK8	Homo sapiens cDNA FLJ36014 fis, clone TESTI2016101. [AK093333]	158067
A_23_P83200	1.7	2.8	2.9	3.1	AK8	Homo sapiens adenylate kinase 8 (AK8), mRNA [NM_152572]	158067
A_23_P111311	1.0	-2.5	-1.4	1.0	AKAP12	Homo sapiens A kinase (PRKA) anchor protein 12 (AKAP12), transcript variant 2, mRNA [NM_144497]	9590
A_32_P152437	-2.2	4.3	2.3	3.1	AKAP12	Homo sapiens A kinase (PRKA) anchor protein 12 (AKAP12), transcript variant 1, mRNA [NM_005100]	9590

A_24_P155761	-3.3	-3.4	-3.2	-1.2	AKAP13	Homo sapiens A kinase (PRKA) anchor protein 13 (AKAP13), transcript variant 1, mRNA [NM_006738]	11214
A_23_P359043	-2.9	-1.1	-1.1	-1.1	AKAP2	Homo sapiens A kinase (PRKA) anchor protein 2 (AKAP2), transcript variant 1, mRNA [NM_001004065]	11217
A_23_P14035	1.6	2.6	2.5	2.6	AKAP3	Homo sapiens A kinase (PRKA) anchor protein 3 (AKAP3), mRNA [NM_006422]	10566
A_23_P106103	2.3	-1.1	-1.3	-1.2	AKAP5	Homo sapiens A kinase (PRKA) anchor protein 5 (AKAP5), mRNA [NM_004857]	9495

A_23_P14343	2.0	2.0	3.6	1.2	AKAP6	Homo sapiens A kinase (PRKA) anchor protein 6 (AKAP6), mRNA [NM_004274]	9472
A_24_P934594	-5.4	1.3	-2.4	-2.3	AKAP8L	Homo sapiens mRNA; cDNA DKFZp434L0650 (from clone DKFZp434L0650). [AL133576]	26993
A_23_P153767	3.8	3.7	3.8	4.2	AKAP8L	Homo sapiens A kinase (PRKA) anchor protein 8-like (AKAP8L), mRNA [NM_014371]	26993
A_24_P357809	-1.3	-1.4	-1.5	-3.0	AKIP1	Homo sapiens A kinase (PRKA) interacting protein 1 (AKIP1), transcript variant 1, mRNA [NM_020642]	56672

A_23_P337292	-1.3	-1.3	-1.9	-2.3	AKNAD1	AKNA domain containing 1 [Source:HGNC Symbol;Acc:28398] [ENST00000472781]	254268
A_24_P129341	-7.7	1.1	1.0	-1.0	AKR1B10	Homo sapiens aldo-keto reductase family 1, member B10 (aldose reductase) (AKR1B10), mRNA [NM_020299]	57016
A_24_P152968	-2.3	1.1	-1.0	-1.0	AKR1C1	Homo sapiens aldo-keto reductase family 1, member C1 (dihydrodiol dehydrogenase 1; 20-alpha (3-alpha)-hydroxysteroid dehydrogenase) (AKR1C1), mRNA [NM_001353]	1645

A_24_P312578	-1.8	-1.3	1.4	-14.1	AKR1C1	aldo-keto reductase family 1, member C1 (dihydrodiol dehydrogenase 1; 20-alpha (3-alpha)-hydroxysteroid dehydrogenase) [Source:HGNC Symbol;Acc:384] [ENST00000434459]	1645
A_23_P149926	1.8	1.9	2.5	1.0	AKR1C4	Homo sapiens aldo-keto reductase family 1, member C4 (chlordecone reductase; 3-alpha hydroxysteroid dehydrogenase, type I; dihydrodiol dehydrogenase 4) (AKR1C4), mRNA [NM_001818]	1109

A_23_P316812	3.0	3.9	-1.1	1.0	AKR7A2P1	Homo sapiens aldo-keto reductase family 7, member A2 pseudogene 1 (AKR7A2P1), non-coding RNA [NR_002796]	246182
A_23_P103968	2.2	-3.9	-1.0	1.6	AKR7A3	Homo sapiens aldo-keto reductase family 7, member A3 (aflatoxin aldehyde reductase) (AKR7A3), mRNA [NM_012067]	22977
A_23_P320304	5.8	5.3	1.4	2.5	AKR7L	Homo sapiens aldo-keto reductase family 7-like (AKR7L), transcript variant 1, non-coding RNA [NR_040288]	246181

A_23_P153502	1.8	2.4	1.7	1.6	AKT1S1	Homo sapiens AKT1 substrate 1 (proline-rich) (AKT1S1), transcript variant 1, mRNA [NM_032375]	84335
A_23_P208870	2.2	2.3	1.6	1.4	AKT2	Homo sapiens v-akt murine thymoma viral oncogene homolog 2 (AKT2), transcript variant 1, mRNA [NM_001626]	208
A_24_P359267	1.1	-1.0	-2.8	-1.8	AKT2	Homo sapiens v-akt murine thymoma viral oncogene homolog 2 (AKT2), transcript variant 1, mRNA [NM_001626]	208

A_23_P160354	1.5	1.4	-2.1	-1.0	AKT3	Homo sapiens v-akt murine thymoma viral oncogene homolog 3 (protein kinase B, gamma) (AKT3), transcript variant 2, mRNA [NM_181690]	10000
A_23_P163622	1.1	1.1	1.1	-2.8	AKTIP	Homo sapiens AKT interacting protein (AKTIP), transcript variant 1, mRNA [NM_001012398]	64400
A_23_P324278	2.0	1.4	-1.6	2.5	ALAD	Homo sapiens aminolevulinate dehydratase (ALAD), mRNA [NM_000031]	210

A_24_P191588	3.4	1.5	1.9	1.6	ALAS1	Homo sapiens aminolevulinate, delta-, synthase 1 (ALAS1), transcript variant 1, mRNA [NM_000688]	211
A_32_P74643	-2.1	-2.6	-6.0	-1.8	ALCAM	Homo sapiens activated leukocyte cell adhesion molecule (ALCAM), transcript variant 1, mRNA [NM_001627]	214
A_24_P5743	-1.7	1.0	1.3	-3.4	ALDH16A1	Homo sapiens aldehyde dehydrogenase 16 family, member A1 (ALDH16A1), transcript variant 1, mRNA [NM_153329]	126133

A_23_P99956	-5.0	1.1	1.0	-1.0	ALDH1A2	Homo sapiens aldehyde dehydrogenase 1 family, member A2 (ALDH1A2), transcript variant 3, mRNA [NM_170697]	8854
A_24_P64233	-2.4	-1.3	-3.4	1.5	ALDH1B1	Homo sapiens aldehyde dehydrogenase 1 family, member B1 (ALDH1B1), nuclear gene encoding mitochondrial protein, mRNA [NM_000692]	219
A_24_P941870	-1.0	-1.5	-1.7	-3.2	ALDH1L1	aldehyde dehydrogenase 1 family, member L1 [Source:HGNC Symbol;Acc:3978] [ENST00000484724]	10840

A_32_P117016	-1.2	-2.8	-1.3	-1.0	ALDH1L2	Homo sapiens aldehyde dehydrogenase 1 family, member L2 (ALDH1L2), nuclear gene encoding mitochondrial protein, transcript variant 1, mRNA [NM_001034173]	160428
A_24_P218688	1.3	1.6	1.6	-2.9	ALDH3B1	Homo sapiens aldehyde dehydrogenase 3 family, member B1 (ALDH3B1), transcript variant 1, mRNA [NM_000694]	221

A_23_P170337	-1.6	-1.8	-1.5	-2.2	ALDH4A1	Homo sapiens aldehyde dehydrogenase 4 family, member A1 (ALDH4A1), nuclear gene encoding mitochondrial protein, transcript variant P5CDhL, mRNA [NM_003748]	8659
A_24_P263036	1.5	-2.3	1.8	1.0	ALDH4A1	Homo sapiens aldehyde dehydrogenase 4 family, member A1 (ALDH4A1), nuclear gene encoding mitochondrial protein, transcript variant P5CDhL, mRNA [NM_003748]	8659

A_24_P3761	-2.9	1.4	-1.1	1.8	ALDH5A1	Homo sapiens aldehyde dehydrogenase 5 family, member A1 (ALDH5A1), nuclear gene encoding mitochondrial protein, transcript variant 1, mRNA [NM_170740]	7915
A_23_P128967	1.2	1.0	-1.6	-4.3	ALDH6A1	Homo sapiens aldehyde dehydrogenase 6 family, member A1 (ALDH6A1), nuclear gene encoding mitochondrial protein, mRNA [NM_005589]	4329

A_24_P921584	2.1	1.3	-1.1	1.9	ALDOA	Homo sapiens aldolase A, fructose-bisphosphate (ALDOA), transcript variant 2, mRNA [NM_184041]	226
A_23_P32143	1.5	1.2	1.8	2.6	ALDOB	Homo sapiens aldolase B, fructose-bisphosphate (ALDOB), mRNA [NM_000035]	229
A_23_P78108	1.8	2.1	2.4	2.0	ALDOC	Homo sapiens aldolase C, fructose-bisphosphate (ALDOC), mRNA [NM_005165]	230

A_23_P66306	12.3	13.5	13.4	3.8	ALG1	Homo sapiens asparagine-linked glycosylation 1, beta-1,4-mannosyltransferase homolog (S. cerevisiae) (ALG1), mRNA [NM_019109]	56052
A_32_P169131	-6.1	-1.3	-1.0	1.1	ALG1	Homo sapiens asparagine-linked glycosylation 1, beta-1,4-mannosyltransferase homolog (S. cerevisiae) (ALG1), mRNA [NM_019109]	56052
A_24_P565556	1.9	2.0	1.9	-19.8	ALG1	Homo sapiens asparagine-linked glycosylation 1, beta-1,4-mannosyltransferase homolog (S. cerevisiae) (ALG1), mRNA [NM_019109]	56052

A_23_P60718	3.7	-1.4	3.7	1.8	ALG12	Homo sapiens asparagine-linked glycosylation 12, alpha-1,6-mannosyltransferase homolog (S. cerevisiae) (ALG12), mRNA [NM_024105]	79087
A_24_P583350	4.8	2.2	4.0	2.8	ALG1L	Homo sapiens asparagine-linked glycosylation 1-like (ALG1L), transcript variant 2, mRNA [NM_001015050]	200810
A_24_P414269	-2.6	-1.7	1.0	1.0	ALG3	Homo sapiens asparagine-linked glycosylation 3, alpha-1,3-mannosyltransferase homolog (S. cerevisiae) (ALG3), transcript variant 1, mRNA [NM_005787]	10195

A_23_P151436	-1.4	-1.2	-2.1	-1.8	ALG5	Homo sapiens asparagine-linked glycosylation 5, dolichyl-phosphate beta-glucosyltransferase homolog (S. cerevisiae) (ALG5), transcript variant 1, mRNA [NM_013338]	29880
A_23_P35168	1.3	1.2	2.1	1.3	ALG6	Homo sapiens asparagine-linked glycosylation 6, alpha-1,3-glucosyltransferase homolog (S. cerevisiae) (ALG6), mRNA [NM_013339]	29929

A_24_P795594	-5.0	-1.0	-1.7	-1.3	ALG9	Homo sapiens asparagine-linked glycosylation 9, alpha-1,2-mannosyltransferase homolog (S. cerevisiae) (ALG9), transcript variant 3, mRNA [NM_001077691]	79796
A_23_P64193	-2.1	-2.2	1.0	1.4	ALG9	Homo sapiens asparagine-linked glycosylation 9, alpha-1,2-mannosyltransferase homolog (S. cerevisiae) (ALG9), transcript variant 3, mRNA [NM_001077691]	79796
A_23_P14543	-2.0	-6.6	4.1	-1.0	ALKBH1	Homo sapiens alkB, alkylation repair homolog 1 (E. coli) (ALKBH1), mRNA [NM_006020]	8846

A_24_P628384	-1.3	-1.2	3.5	2.0	ALKBH2	Homo sapiens alkB, alkylation repair homolog 2 (E. coli) (ALKBH2), transcript variant 2, mRNA [NM_001001655]	121642
A_32_P28939	2.6	2.8	-1.0	-1.1	ALKBH2	Homo sapiens alkB, alkylation repair homolog 2 (E. coli) (ALKBH2), transcript variant 2, mRNA [NM_001001655]	121642
A_23_P255523	1.1	1.1	-1.0	-6.0	ALKBH4	Homo sapiens alkB, alkylation repair homolog 4 (E. coli) (ALKBH4), mRNA [NM_017621]	54784
A_23_P312179	-4.0	1.8	1.6	-2.1	ALMS1	Homo sapiens Alstrom syndrome 1 (ALMS1), mRNA [NM_015120]	7840

A_23_P435657	-1.0	-2.4	-1.0	-1.0	ALMS1P	Homo sapiens Alstrom syndrome 1 pseudogene (ALMS1P), non-coding RNA [NR_003683]	200420
A_23_P152909	1.7	4.3	4.1	-1.7	ALOX12	Homo sapiens arachidonate 12-lipoxygenase (ALOX12), mRNA [NM_000697]	239
A_23_P83634	1.2	1.3	1.3	-3.0	ALOX12B	Homo sapiens arachidonate 12-lipoxygenase, 12R type (ALOX12B), mRNA [NM_001139]	242
A_32_P440768	-1.1	-24.6	-1.3	-1.7	ALOX12P2	Homo sapiens arachidonate 12-lipoxygenase pseudogene 2 (ALOX12P2), non-coding RNA [NR_002710]	245

A_23_P55373	2.6	5.0	4.9	6.0	ALOX15	Homo sapiens arachidonate 15-lipoxygenase (ALOX15), mRNA [NM_001140]	246
A_23_P2793	-1.4	-1.4	1.1	-3.5	ALOX5AP	Homo sapiens arachidonate 5-lipoxygenase-activating protein (ALOX5AP), transcript variant 1, mRNA [NM_001629]	241
A_24_P347378	1.7	-1.1	-1.5	-2.1	ALOX5AP	Homo sapiens arachidonate 5-lipoxygenase-activating protein (ALOX5AP), transcript variant 1, mRNA [NM_001629]	241

A_24_P347880	2.3	1.3	1.6	1.0	ALOXE3	Homo sapiens arachidonate lipoxygenase 3 (ALOXE3), transcript variant 2, mRNA [NM_021628]	59344
A_23_P337658	1.1	-2.8	1.6	-1.0	ALPI	Homo sapiens alkaline phosphatase, intestinal (ALPI), mRNA [NM_001631]	248
A_23_P144531	1.1	-1.0	2.1	1.0	ALPK1	alpha-kinase 1 [Source:HGNC Symbol;Acc:20917] [ENST00000309610]	80216
A_23_P133142	-1.0	-2.0	1.2	-1.1	ALPK1	Homo sapiens alpha-kinase 1 (ALPK1), transcript variant 1, mRNA [NM_025144]	80216

A_23_P15876	5.9	5.6	5.8	3.6	ALPK2	Homo sapiens alpha-kinase 2 (ALPK2), mRNA [NM_052947]	115701
A_23_P97046	-1.6	-1.4	-3.1	-1.8	ALPL	Homo sapiens alkaline phosphatase, liver/bone/kidney (ALPL), transcript variant 1, mRNA [NM_000478]	249
A_23_P79587	1.3	2.8	1.3	-1.6	ALPP	Homo sapiens alkaline phosphatase, placental (ALPP), mRNA [NM_001632]	250
A_24_P131580	1.0	3.1	1.1	2.5	ALPPL2	Homo sapiens alkaline phosphatase, placental-like 2 (ALPPL2), mRNA [NM_031313]	251

A_24_P222410	1.8	1.3	-2.1	-1.9	ALS2	Homo sapiens amyotrophic lateral sclerosis 2 (juvenile) (ALS2), transcript variant 1, mRNA [NM_020919]	57679
A_23_P416705	-2.4	-1.2	-1.7	-1.2	ALS2CL	Homo sapiens ALS2 C-terminal like (ALS2CL), transcript variant 1, mRNA [NM_147129]	259173
A_32_P420009	1.1	-2.2	1.3	-1.8	ALS2CL	Homo sapiens ALS2 C-terminal like (ALS2CL), transcript variant 1, mRNA [NM_147129]	259173
A_23_P85941	-2.0	-2.2	-2.5	-4.7	ALX3	Homo sapiens ALX homeobox 3 (ALX3), mRNA [NM_006492]	257

A_23_P161792	1.5	1.5	1.0	-3.9	ALX4	Homo sapiens ALX homeobox 4 (ALX4), mRNA [NM_021926]	60529
A_24_P106297	-1.6	-39.6	-1.6	-1.6	AMACR	Homo sapiens alpha-methylacyl-CoA racemase (AMACR), nuclear gene encoding mitochondrial protein, transcript variant 1, mRNA [NM_014324]	23600
A_23_P44867	-1.1	1.4	2.1	-1.2	AMBN	Homo sapiens ameloblastin (enamel matrix protein) (AMBN), mRNA [NM_016519]	258

A_23_P256504	-1.0	-2.6	-1.8	-1.1	AMBP	Homo sapiens alpha-1-microglobulin/bikunin precursor (AMBP), mRNA [NM_001633]	259
A_24_P371962	1.8	6.2	-1.4	-1.3	AMD1	Homo sapiens adenosylmethionine decarboxylase 1 (AMD1), transcript variant 1, mRNA [NM_001634]	262
A_23_P49351	2.7	2.3	2.6	2.2	AMDHD2	Homo sapiens amidohydrolase domain containing 2 (AMDHD2), transcript variant 1, mRNA [NM_015944]	51005

A_23_P96478	-1.5	-1.6	-3.8	-1.6	AMELX	Homo sapiens amelogenin, X-linked (AMELX), transcript variant 2, mRNA [NM_182681]	265
A_23_P78944	1.6	1.1	-2.3	1.6	AMH	Homo sapiens anti-Mullerian hormone (AMH), mRNA [NM_000479]	268
A_24_P115990	2.3	2.3	1.4	9.1	AMHR2	Homo sapiens anti-Mullerian hormone receptor, type II (AMHR2), transcript variant 1, mRNA [NM_020547]	269
A_24_P192914	-2.6	-2.7	-2.9	-2.8	AMICA1	Homo sapiens adhesion molecule, interacts with CXADR antigen 1 (AMICA1), transcript variant 2, mRNA [NM_153206]	120425

A_23_P97598	1.1	1.1	1.6	-2.2	AMIGO1	Homo sapiens adhesion molecule with Ig-like domain 1 (AMIGO1), mRNA [NM_020703]	57463
A_23_P14083	1.2	1.5	-1.0	-2.1	AMIGO2	Homo sapiens adhesion molecule with Ig-like domain 2 (AMIGO2), transcript variant 2, mRNA [NM_181847]	347902
A_23_P306655	1.4	-2.4	1.2	-1.6	AMIGO3	Homo sapiens adhesion molecule with Ig-like domain 3 (AMIGO3), mRNA [NM_198722]	386724

A_23_P137116	-1.2	-6.1	-1.0	-1.0	AMMECR1	Homo sapiens Alport syndrome, mental retardation, midface hypoplasia and elliptocytosis chromosomal region gene 1 (AMMECR1), transcript variant 1, mRNA [NM_015365]	9949
A_32_P201773	-2.8	1.0	-1.0	1.0	AMMECR1	Homo sapiens Alport syndrome, mental retardation, midface hypoplasia and elliptocytosis chromosomal region gene 1 (AMMECR1), transcript variant 1, mRNA [NM_015365]	9949

A_23_P138796	1.5	-4.8	1.4	1.3	AMOTL1	Homo sapiens angiomotin like 1 (AMOTL1), mRNA [NM_130847]	154810
A_24_P329815	2.7	1.4	4.3	2.2	AMOTL1	Homo sapiens angiomotin like 1 (AMOTL1), mRNA [NM_130847]	154810
A_23_P166686	1.2	-1.5	1.0	-3.3	AMOTL2	Homo sapiens angiomotin like 2 (AMOTL2), mRNA [NM_016201]	51421
A_23_P51787	-1.4	-1.4	-1.4	-24.4	AMPD1	Homo sapiens adenosine monophosphate deaminase 1 (AMPD1), transcript variant 1, mRNA [NM_000036]	270

A_24_P174503	1.0	-1.0	-3.9	-1.8	AMT	Homo sapiens aminomethyltransferase (AMT), nuclear gene encoding mitochondrial protein, transcript variant 1, mRNA [NM_000481]	275
A_24_P383649	-3.7	-1.0	-12.1	-1.0	AMZ1	Homo sapiens archaelysin family metallopeptidase 1 (AMZ1), mRNA [NM_133463]	155185
A_24_P410587	1.0	1.0	2.2	1.0	AMZ2P1	Homo sapiens archaelysin family metallopeptidase 2 pseudogene 1 (AMZ2P1), non-coding RNA [NR_026903]	201283

A_24_P186346	1.0	1.5	-3.1	-1.3	ANAPC1	Homo sapiens anaphase promoting complex subunit 1 (ANAPC1), mRNA [NM_022662]	64682
A_32_P12104	1.9	1.9	-6.1	1.9	ANAPC1	Homo sapiens anaphase promoting complex subunit 1 (ANAPC1), mRNA [NM_022662]	64682
A_23_P27147	-2.4	1.2	1.7	-1.4	ANAPC11	Homo sapiens anaphase promoting complex subunit 11 (ANAPC11), transcript variant 1, mRNA [NM_001002244]	51529
A_23_P317800	1.0	-3.2	1.0	1.0	ANAPC4	Homo sapiens anaphase promoting complex subunit 4 (ANAPC4), mRNA [NM_013367]	29945

A_23_P377830	1.1	-8.9	-1.0	1.0	ANAPC5	Homo sapiens anaphase promoting complex subunit 5 (ANAPC5), transcript variant 1, mRNA [NM_016237]	51433
A_23_P44636	-1.0	-2.2	-1.1	1.0	ANAPC7	Homo sapiens anaphase promoting complex subunit 7 (ANAPC7), transcript variant 1, mRNA [NM_016238]	51434
A_23_P363406	1.0	-3.1	-1.0	-4.2	ANGEL1	Homo sapiens angel homolog 1 (Drosophila) (ANGEL1), mRNA [NM_015305]	23357
A_23_P343237	1.1	-12.3	1.1	1.0	ANGEL2	Homo sapiens angel homolog 2 (Drosophila) (ANGEL2), mRNA [NM_144567]	90806

A_24_P60887	-3.1	1.0	1.0	1.1	ANGPT1	Homo sapiens angiopoietin 1 (ANGPT1), transcript variant 1, mRNA [NM_001146]	284
A_23_P216023	-1.3	2.1	1.5	1.0	ANGPT1	Homo sapiens angiopoietin 1 (ANGPT1), transcript variant 1, mRNA [NM_001146]	284
A_24_P367900	-1.0	-4.9	-1.0	-1.0	ANGPT2	angiopoietin 2 [Source:HGNC Symbol;Acc:485] [ENST00000325203]	285
A_23_P68511	1.2	1.3	-1.9	-2.0	ANGPT4	Homo sapiens angiopoietin 4 (ANGPT4), mRNA [NM_015985]	51378
A_23_P126706	-2.5	1.0	1.3	1.3	ANGPTL1	Homo sapiens angiopoietin-like 1 (ANGPTL1), mRNA [NM_004673]	9068

A_24_P199484	2.4	2.5	2.5	2.7	ANGPTL1	Homo sapiens angiopoietin-like 1 (ANGPTL1), mRNA [NM_004673]	9068
A_23_P50276	1.9	1.6	2.3	1.0	ANGPTL6	Homo sapiens angiopoietin-like 6 (ANGPTL6), mRNA [NM_031917]	83854
A_23_P216108	-1.5	-3.2	-1.0	1.2	ANK1	Homo sapiens ankyrin 1, erythrocytic (ANK1), transcript variant 3, mRNA [NM_000037]	286
A_23_P133068	1.0	1.0	-1.0	-2.2	ANK2	Homo sapiens ankyrin 2, neuronal (ANK2), transcript variant 1, mRNA [NM_001148]	287
A_32_P33304	10.1	9.9	9.5	9.2	ANK3	Homo sapiens ankyrin 3, node of Ranvier (ankyrin G) (ANK3), transcript variant 4, mRNA [NM_001204404]	288

A_23_P350698	-2.4	-2.3	-1.9	-2.1	ANKFN1	Homo sapiens ankyrin-repeat and fibronectin type III domain containing 1 (ANKFN1), mRNA [NM_153228]	162282
A_23_P92642	1.8	-7.1	1.2	-1.1	ANKHD1	Homo sapiens ankyrin repeat and KH domain containing 1 (ANKHD1), transcript variant 3, mRNA [NM_024668]	54882
A_24_P18020	1.0	1.0	-2.6	-1.1	ANKIB1	Homo sapiens ankyrin repeat and IBR domain containing 1 (ANKIB1), mRNA [NM_019004]	54467

A_23_P93912	2.9	1.0	1.9	1.0	ANKIB1	Homo sapiens ankyrin repeat and IBR domain containing 1 (ANKIB1), mRNA [NM_019004]	54467
A_23_P142497	3.1	2.4	2.0	3.1	ANKLE1	Homo sapiens ankyrin repeat and LEM domain containing 1 (ANKLE1), mRNA [NM_152363]	126549
A_23_P159012	-3.0	1.8	1.7	1.0	ANKRA2	Homo sapiens ankyrin repeat, family A (RFXANK-like), 2 (ANKRA2), mRNA [NM_023039]	57763
A_23_P163639	1.0	-1.8	-1.0	2.0	ANKRD11	Homo sapiens ankyrin repeat domain 11 (ANKRD11), mRNA [NM_013275]	29123

A_32_P82403	1.2	-1.0	-2.3	-1.1	ANKRD11	Homo sapiens ankyrin repeat domain 11 (ANKRD11), mRNA [NM_013275]	29123
A_23_P130293	-2.1	4.7	5.1	1.7	ANKRD12	Homo sapiens ankyrin repeat domain 12 (ANKRD12), transcript variant 1, mRNA [NM_015208]	23253
A_24_P76105	2.1	-1.3	2.2	1.2	ANKRD13D	Homo sapiens ankyrin repeat domain 13 family, member D (ANKRD13D), transcript variant 1, mRNA [NM_207354]	338692

A_24_P349356	-1.0	-2.6	-4.4	-3.1	ANKRD13D	Homo sapiens ankyrin repeat domain 13 family, member D (ANKRD13D), transcript variant 1, mRNA [NM_207354]	338692
A_23_P150069	4.1	1.6	-1.1	1.2	ANKRD16	Homo sapiens ankyrin repeat domain 16 (ANKRD16), transcript variant 4, mRNA [NM_001009943]	54522
A_23_P144378	-1.1	1.4	1.5	4.2	ANKRD17	Homo sapiens ankyrin repeat domain 17 (ANKRD17), transcript variant 1, mRNA [NM_032217]	26057

A_24_P220771	-2.5	2.3	2.3	2.5	ANKRD17	Homo sapiens ankyrin repeat domain 17 (ANKRD17), transcript variant 1, mRNA [NM_0322 17]	26057
A_24_P172293	12.5	14.1	6.7	9.7	ANKRD18A	ankyrin repeat domain 18A [Source:H GNC Symbol;Ac c:23643] [ENST0000 0313339]	253650
A_24_P188116	1.4	1.3	2.7	2.8	ANKRD2	Homo sapiens ankyrin repeat domain 2 (stretch responsive muscle) (ANKRD2), transcript variant 1, mRNA [NM_0203 49]	26287

A_24_P64241	-3.5	1.1	1.0	1.0	ANKRD20A2	Homo sapiens ankyrin repeat domain 20 family, member A2 (ANKRD20A2), mRNA [NM_001012421]	441430
A_32_P168326	1.9	-1.0	-2.6	-1.0	ANKRD20A2	Homo sapiens ankyrin repeat domain 20 family, member A2 (ANKRD20A2), mRNA [NM_001012421]	441430
A_24_P868583	-2.9	1.1	1.1	1.1	ANKRD20A2	Homo sapiens ankyrin repeat domain 20 family, member A2 (ANKRD20A2), mRNA [NM_001012421]	441430

A_32_P150086	-2.6	1.0	1.0	-50.8	ANKRD20A5P	Homo sapiens ankyrin repeat domain 20 family, member A5, pseudogene (ANKRD20A5P), non-coding RNA [NR_040113]	440482
A_24_P84220	-21.3	-1.0	-1.1	-1.0	ANKRD20A9P	Homo sapiens ankyrin repeat domain 20 family, member A9, pseudogene (ANKRD20A9P), non-coding RNA [NR_027995]	284232
A_32_P13151	4.7	9.9	2.9	4.3	ANKRD20A9P	Homo sapiens ankyrin repeat domain 20 family, member A9, pseudogene (ANKRD20A9P), non-coding RNA [NR_027995]	284232

A_23_P161428	-1.0	-1.1	-7.7	-1.7	ANKRD22	Homo sapiens ankyrin repeat domain 22 (ANKRD22), mRNA [NM_144590]	118932
A_32_P451874	3.8	1.8	2.9	1.8	ANKRD24	Homo sapiens ankyrin repeat domain 24 (ANKRD24), mRNA [NM_133475]	170961
A_23_P153640	2.0	2.3	1.5	-1.1	ANKRD27	Homo sapiens ankyrin repeat domain 27 (VPS9 domain) (ANKRD27), mRNA [NM_032139]	84079
A_23_P109794	4.1	1.2	2.8	2.6	ANKRD28	Homo sapiens ankyrin repeat domain 28 (ANKRD28), transcript variant 1, mRNA [NM_015199]	23243

A_24_P217365	3.2	-1.9	1.5	1.4	ANKRD28	Homo sapiens ankyrin repeat domain 28 (ANKRD28), transcript variant 1, mRNA [NM_0151 99]	23243
A_23_P12533	-1.5	-1.0	-9.5	-1.1	ANKRD30A	Homo sapiens ankyrin repeat domain 30A (ANKRD30 A), mRNA [NM_0529 97]	91074
A_24_P357647	1.0	-1.0	-2.0	-2.9	ANKRD31	Homo sapiens ankyrin repeat domain 31 (ANKRD31), mRNA [NM_0011 64443]	256006
A_24_P212072	4.0	3.9	1.1	2.0	ANKRD32	Homo sapiens ankyrin repeat domain 32 (ANKRD32), mRNA [NM_0322 90]	84250

A_32_P516342	5.3	-2.5	2.2	3.9	ANKRD33	Homo sapiens ankyrin repeat domain 33 (ANKRD33), transcript variant 2, mRNA [NM_182608]	341405
A_24_P83799	1.5	4.1	4.0	2.5	ANKRD33B	Homo sapiens ankyrin repeat domain 33B (ANKRD33B), mRNA [NM_001164440]	651746
A_32_P137604	2.1	4.5	-1.1	5.8	ANKRD33B	Homo sapiens ankyrin repeat domain 33B (ANKRD33B), mRNA [NM_001164440]	651746
A_23_P325690	2.0	2.0	1.8	-1.3	ANKRD35	Homo sapiens ankyrin repeat domain 35 (ANKRD35), mRNA [NM_144698]	148741

A_23_P253622	2.2	1.5	-1.5	1.1	ANKRD36B	Homo sapiens cDNA: FLJ21281 fis, clone COL01904. [AK024934]	57730
A_24_P324749	1.2	1.2	1.1	-2.5	ANKRD36B	Homo sapiens CLL-associated antigen KW-1 splice variant 2 mRNA, partial cds; alternatively spliced. [AF432209]	57730
A_23_P97123	1.0	1.3	-3.0	1.3	ANKRD36BP1	Homo sapiens ankyrin repeat domain 36B pseudogene 1 (ANKRD36BP1), non-coding RNA [NR_026844]	84832

A_23_P95027	3.6	3.2	1.1	2.3	ANKRD39	Homo sapiens ankyrin repeat domain 39 (ANKRD39), mRNA [NM_016466]	51239
A_23_P400406	1.1	-2.6	-1.0	-1.8	ANKRD43	Homo sapiens ankyrin repeat domain 43 (ANKRD43), mRNA [NM_175873]	134548
A_24_P307498	1.1	1.0	1.0	-2.5	ANKRD45	Homo sapiens ankyrin repeat domain 45 (ANKRD45), mRNA [NM_198493]	339416
A_24_P662086	1.6	1.3	2.4	-1.0	ANKRD46	ankyrin repeat domain 46 [Source:HGNC Symbol;Acc:27229] [ENST00000524319]	157567

A_23_P256047	-8.8	-1.0	-2.8	-1.3	ANKRD5	Homo sapiens ankyrin repeat domain 5 (ANKRD5), transcript variant 1, mRNA [NM_022096]	63926
A_23_P81017	-1.3	-1.4	-2.7	-3.4	ANKRD50	Homo sapiens ankyrin repeat domain 50 (ANKRD50), transcript variant 1, mRNA [NM_020337]	57182
A_24_P371184	1.1	-2.1	-1.2	-1.4	ANKRD53	Homo sapiens ankyrin repeat domain 53 (ANKRD53), transcript variant 2, mRNA [NM_024933]	79998

A_23_P120921	1.3	-2.6	1.2	-1.0	ANKRD54	Homo sapiens ankyrin repeat domain 54 (ANKRD54), transcript variant 1, mRNA [NM_138797]	129138
A_24_P146188	1.1	-2.6	-1.0	1.8	ANKRD6	Homo sapiens ankyrin repeat domain 6 (ANKRD6), transcript variant 3, mRNA [NM_014942]	22881
A_23_P250231	-1.1	-2.3	-1.1	-1.1	ANKRD7	Homo sapiens ankyrin repeat domain 7 (ANKRD7), mRNA [NM_019644]	56311
A_23_P307525	-8.2	1.8	1.6	1.3	ANKRD9	Homo sapiens ankyrin repeat domain 9 (ANKRD9), mRNA [NM_152326]	122416

A_32_P16975	-1.1	-1.6	-1.7	-3.9	ANKS3	Homo sapiens ankyrin repeat and sterile alpha motif domain containing 3 (ANKS3), transcript variant 1, mRNA [NM_133450]	124401
A_24_P358400	-2.2	1.0	1.1	1.8	ANKS6	Homo sapiens cDNA clone IMAGE:3620129. [BC012981]	203286
A_23_P362183	-5.6	-1.1	-1.2	-1.1	ANKS6	Homo sapiens ankyrin repeat and sterile alpha motif domain containing 6 (ANKS6), mRNA [NM_173551]	203286

A_24_P340954	-2.0	1.3	1.2	-1.0	ANKUB1	Homo sapiens ankyrin repeat and ubiquitin domain containing 1 (ANKUB1), mRNA [NM_001144960]	389161
A_23_P119907	5.3	5.3	5.3	5.4	ANKZF1	Homo sapiens ankyrin repeat and zinc finger domain containing 1 (ANKZF1), transcript variant 1, mRNA [NM_018089]	55139
A_24_P87036	1.5	1.5	-2.0	-1.1	ANO1	Homo sapiens anoctamin 1, calcium activated chloride channel (ANO1), transcript variant 1, mRNA [NM_018043]	55107

A_32_P141238	-3.8	-1.4	-1.1	-1.7	ANO2	Homo sapiens anoctamin 2 (ANO2), mRNA [NM_020373]	57101
A_23_P53641	-5.2	-1.0	-1.0	-1.0	ANO6	Homo sapiens anoctamin 6 (ANO6), transcript variant 1, mRNA [NM_001025356]	196527
A_32_P73452	-2.2	-1.1	-1.1	-1.1	ANO8	Homo sapiens anoctamin 8 (ANO8), mRNA [NM_020959]	57719
A_24_P8109	1.1	2.0	-2.8	-1.1	ANO9	Homo sapiens anoctamin 9 (ANO9), mRNA [NM_001012302]	338440
A_32_P133670	-1.1	-1.2	-2.8	-1.1	ANP32A	Homo sapiens acidic (leucine-rich) nuclear phosphoprotein 32 family, member A (ANP32A), mRNA [NM_006305]	8125

A_24_P377144	-1.4	-4.0	-2.0	-2.3	ANTXR2	Homo sapiens anthrax toxin receptor 2 (ANTXR2), transcript variant 1, mRNA [NM_058172]	118429
A_23_P94501	-22.7	1.1	-1.0	-1.0	ANXA1	Homo sapiens annexin A1 (ANXA1), mRNA [NM_000700]	301
A_32_P150632	-1.5	3.3	2.0	3.5	ANXA11	annexin A11 [Source:HGNC Symbol;Acc:535] [ENST00000372234]	311
A_24_P107142	-1.7	1.6	-2.1	-4.1	ANXA11	Homo sapiens annexin A11 (ANXA11), transcript variant c, mRNA [NM_145869]	311
A_32_P148345	1.1	-3.9	-1.0	-1.0	ANXA2	Homo sapiens annexin A2 (ANXA2), transcript variant 2, mRNA [NM_001002857]	302

A_32_P94798	-3.5	-3.2	1.5	-7.7	ANXA2	Homo sapiens annexin A2 (ANXA2), transcript variant 2, mRNA [NM_001002857]	302
A_24_P204244	-3.1	-1.0	-1.6	1.0	ANXA2P1	Homo sapiens annexin A2 pseudogene 1 (ANXA2P1), non-coding RNA [NR_001562]	303
A_23_P121716	1.7	2.5	1.7	1.7	ANXA3	Homo sapiens annexin A3 (ANXA3), mRNA [NM_005139]	306
A_23_P16976	-2.1	1.8	1.4	1.3	ANXA4	Homo sapiens annexin A4 (ANXA4), mRNA [NM_001153]	307
A_23_P69720	-1.7	2.0	-1.6	1.2	ANXA5	Homo sapiens annexin A5 (ANXA5), mRNA [NM_001154]	308

A_23_P357104	1.7	2.3	-1.0	-2.7	ANXA6	Homo sapiens annexin A6 (ANXA6), transcript variant 1, mRNA [NM_001155]	309
A_23_P395054	-1.6	1.2	-6.0	-1.9	ANXA8L2	annexin A8-like 2 [Source:HGNC Symbol;Acc:23335] [ENST00000340243]	244
A_23_P103617	1.0	-2.4	-1.1	-1.1	ANXA9	Homo sapiens annexin A9 (ANXA9), mRNA [NM_003568]	8416
A_23_P4133	1.2	-3.7	-1.3	-1.3	AOC2	Homo sapiens amine oxidase, copper containing 2 (retina-specific) (AOC2), transcript variant 1, mRNA [NM_001158]	314

A_23_P154037	2.8	2.7	4.7	2.6	AOX1	Homo sapiens aldehyde oxidase 1 (AOX1), mRNA [NM_001159]	316
A_23_P69791	-28.0	-1.1	-1.1	-1.2	AP1AR	Homo sapiens adaptor-related protein complex 1 associated regulatory protein (AP1AR), transcript variant 1, mRNA [NM_018569]	55435
A_23_P206788	1.1	-2.9	-1.8	1.1	AP1G1	Homo sapiens adaptor-related protein complex 1, gamma 1 subunit (AP1G1), transcript variant 1, mRNA [NM_001030007]	164

A_23_P2967	-1.2	2.2	1.8	-1.9	AP1G2	Homo sapiens adaptor-related protein complex 1, gamma 2 subunit (AP1G2), mRNA [NM_003917]	8906
A_23_P78543	1.9	2.1	1.5	2.8	AP1M2	Homo sapiens adaptor-related protein complex 1, mu 2 subunit (AP1M2), mRNA [NM_005498]	10053
A_24_P937306	-1.0	-2.4	-1.1	-1.1	AP1S2	adaptor-related protein complex 1, sigma 2 subunit [Source:HGNC Symbol;Acc:560] [ENST00000380291]	8905

A_23_P217384	-2.4	1.1	-1.0	1.0	AP1S2	Homo sapiens adaptor-related protein complex 1, sigma 2 subunit (AP1S2), mRNA [NM_003916]	8905
A_24_P105747	1.0	1.0	1.0	-4.0	AP1S3	Homo sapiens adaptor-related protein complex 1, sigma 3 subunit (AP1S3), mRNA [NM_001039569]	130340
A_23_P4885	-17.0	-1.8	1.9	1.4	AP2A1	Homo sapiens adaptor-related protein complex 2, alpha 1 subunit (AP2A1), transcript variant 1, mRNA [NM_014203]	160

A_23_P136635	1.9	1.6	2.5	1.4	AP3B1	Homo sapiens adaptor-related protein complex 3, beta 1 subunit (AP3B1), mRNA [NM_003664]	8546
A_32_P47510	-2.1	1.5	1.4	1.0	AP3B1	adaptor-related protein complex 3, beta 1 subunit [Source:HGNC Symbol;Acc:566] [ENST00000255194]	8546
A_23_P77304	1.3	1.8	2.3	1.5	AP3B2	Homo sapiens adaptor-related protein complex 3, beta 2 subunit (AP3B2), mRNA [NM_004644]	8120

A_23_P158938	-1.0	-6.6	-1.9	-1.0	AP3M1	Homo sapiens adaptor-related protein complex 3, mu 1 subunit (AP3M1), transcript variant 1, mRNA [NM_207012]	26985
A_24_P167030	1.4	2.0	1.6	-1.0	AP3M1	Homo sapiens adaptor-related protein complex 3, mu 1 subunit (AP3M1), transcript variant 1, mRNA [NM_207012]	26985
A_32_P195853	-1.0	2.0	1.1	-1.1	AP3M1	Homo sapiens adaptor-related protein complex 3, mu 1 subunit (AP3M1), transcript variant 1, mRNA [NM_207012]	26985

A_23_P170331	1.7	2.0	-2.1	-1.1	AP3M2	Homo sapiens adaptor-related protein complex 3, mu 2 subunit (AP3M2), transcript variant 2, mRNA [NM_006803]	10947
A_24_P401645	35.6	19.6	24.6	29.5	AP4S1	Homo sapiens adaptor-related protein complex 4, sigma 1 subunit (AP4S1), transcript variant 2, mRNA [NM_001128126]	11154
A_23_P36611	4.3	1.1	1.1	1.0	APAF1	Homo sapiens apoptotic peptidase activating factor 1 (APAF1), transcript variant 3, mRNA [NM_181861]	317

A_32_P24857	2.1	1.0	1.9	1.7	APBA1	ij42f09.y1 Human insulinoma Homo sapiens cDNA clone IMAGE:5633800 5', mRNA sequence [BM511013]	320
A_23_P1998	-1.2	-1.4	-2.9	-5.7	APBB1	Homo sapiens amyloid beta (A4) precursor protein-binding, family B, member 1 (Fe65) (APBB1), transcript variant 1, mRNA [NM_001164]	322
A_24_P372613	3.5	3.8	3.1	1.1	APBB1	Homo sapiens amyloid beta (A4) precursor protein-binding, family B, member 1 (Fe65) (APBB1), transcript variant 1, mRNA [NM_001164]	322

A_23_P401700	-4.6	-1.6	-1.8	-2.8	APBB1IP	Homo sapiens amyloid beta (A4) precursor protein-binding, family B, member 1 interacting protein (APBB1IP), mRNA [NM_019043]	54518
A_23_P12549	1.0	-3.9	-1.1	-1.1	APBB1IP	Homo sapiens amyloid beta (A4) precursor protein-binding, family B, member 1 interacting protein (APBB1IP), mRNA [NM_019043]	54518
A_24_P234701	-1.2	-1.2	-2.5	-1.3	APBB2	Homo sapiens amyloid beta (A4) precursor protein-binding, family B, member 2 (APBB2), transcript variant 1, mRNA [NM_004307]	323

A_23_P10701	1.0	1.4	-1.1	-4.1	APBB2	Homo sapiens amyloid beta (A4) precursor protein-binding, family B, member 2 (APBB2), transcript variant 3, mRNA [NM_173075]	323
A_24_P918740	-1.5	-2.0	-2.7	-2.9	APC	Homo sapiens adenomatous polyposis coli (APC), transcript variant 3, mRNA [NM_000038]	324
A_24_P254133	1.6	2.4	4.8	2.2	APC2	Homo sapiens adenomatosis polyposis coli 2 (APC2), mRNA [NM_005883]	10297
A_24_P76844	1.1	1.0	-2.7	-1.0	APC2	Homo sapiens adenomatosis polyposis coli 2 (APC2), mRNA [NM_005883]	10297

A_23_P101905	1.1	-1.3	-5.7	-1.2	APC2	Homo sapiens adenomatosis polyposis coli 2 (APC2), mRNA [NM_005883]	10297
A_23_P101908	1.4	-5.0	1.1	1.7	APC2	Homo sapiens adenomatosis polyposis coli 2 (APC2), mRNA [NM_005883]	10297
A_24_P208794	2.6	3.0	1.1	-1.1	APEH	Homo sapiens N-acylaminoacyl-peptide hydrolase (APEH), mRNA [NM_001640]	327
A_24_P404822	1.5	2.1	2.2	1.2	APLN	Homo sapiens apelin (APLN), mRNA [NM_017413]	8862
A_23_P147755	-1.3	-1.0	1.1	4.5	APLN	Homo sapiens apelin (APLN), mRNA [NM_017413]	8862

A_23_P318860	-1.2	-2.2	-2.5	-1.2	APLNR	Homo sapiens apelin receptor (APLNR), transcript variant 1, mRNA [NM_005161]	187
A_23_P27983	-30.3	1.7	-1.3	1.2	APLP1	Homo sapiens amyloid beta (A4) precursor-like protein 1 (APLP1), transcript variant 2, mRNA [NM_005166]	333
A_23_P126448	-1.7	-2.6	-3.5	-1.1	APOA1BP	Homo sapiens apolipoprotein A-I binding protein (APOA1BP), mRNA [NM_144772]	128240
A_23_P87036	-2.2	1.7	-2.3	1.5	APOA4	Homo sapiens apolipoprotein A-IV (APOA4), mRNA [NM_000482]	337

A_23_P75630	-1.0	-1.1	-1.7	-4.3	APOA5	Homo sapiens apolipoprotein A-V (APOA5), transcript variant 1, mRNA [NM_052968]	116519
A_32_P9543	1.1	-2.4	-3.7	1.4	APOBEC3A	Homo sapiens apolipoprotein B mRNA editing enzyme, catalytic polypeptide-like 3A (APOBEC3A), transcript variant 1, mRNA [NM_145699]	200315
A_24_P66027	1.0	-1.6	1.7	-3.4	APOBEC3B	Homo sapiens apolipoprotein B mRNA editing enzyme, catalytic polypeptide-like 3B (APOBEC3B), mRNA [NM_004900]	9582

A_23_P132316	-1.2	2.6	1.1	1.0	APOBEC3D	Homo sapiens apolipoprotein B mRNA editing enzyme, catalytic polypeptide-like 3D (APOBEC3D), mRNA [NM_152426]	140564
A_23_P357101	-1.0	2.2	-1.0	1.0	APOBEC3F	Homo sapiens apolipoprotein B mRNA editing enzyme, catalytic polypeptide-like 3F (APOBEC3F), transcript variant 1, mRNA [NM_145298]	200316
A_23_P143713	1.1	-3.4	1.1	1.1	APOBEC3G	Homo sapiens apolipoprotein B mRNA editing enzyme, catalytic polypeptide-like 3G (APOBEC3G), mRNA [NM_021822]	60489

A_23_P4649	-1.0	-1.0	-12.7	-1.1	APOC1	Homo sapiens apolipoprotein C-I (APOC1), mRNA [NM_001645]	341
A_23_P38244	1.1	1.0	-1.2	-2.0	APOH	Homo sapiens apolipoprotein H (beta-2-glycoprotein I) (APOH), mRNA [NM_000042]	350
A_24_P48898	1.1	1.5	2.1	1.5	APOL2	Homo sapiens apolipoprotein L, 2 (APOL2), transcript variant beta, mRNA [NM_145637]	23780
A_23_P29237	2.0	6.3	1.1	2.6	APOL3	Homo sapiens apolipoprotein L, 3 (APOL3), transcript variant beta/a, mRNA [NM_145641]	80833

A_24_P416997	-1.2	-3.1	-1.2	-1.3	APOL3	Homo sapiens apolipoprotein L, 3 (APOL3), transcript variant beta/a, mRNA [NM_145641]	80833
A_23_P380857	-1.0	-3.1	-1.0	-1.0	APOL4	Homo sapiens apolipoprotein L, 4 (APOL4), transcript variant a, mRNA [NM_030643]	80832
A_24_P374163	3.5	3.5	4.2	2.5	APOL4	apolipoprotein L, 4 [Source:HGNC Symbol;Acc:14867] [ENST00000328429]	80832
A_23_P155049	-1.0	-1.0	-1.1	-7.6	APOL6	Homo sapiens apolipoprotein L, 6 (APOL6), mRNA [NM_030641]	80830
A_24_P941167	-1.0	-1.0	-1.1	-3.3	APOL6	Homo sapiens apolipoprotein L, 6 (APOL6), mRNA [NM_030641]	80830

A_23_P48217	1.1	-2.7	-1.1	-1.0	APOLD1	Homo sapiens apolipoprotein L domain containing 1 (APOLD1), transcript variant 2, mRNA [NM_030817]	81575
A_24_P89426	5.4	1.1	-1.0	-1.0	APOM	Homo sapiens apolipoprotein M (APOM), mRNA [NM_019101]	55937
A_23_P61127	1.4	2.8	-1.1	1.2	APOO	Homo sapiens apolipoprotein O (APOO), transcript variant 1, mRNA [NM_024122]	79135
A_23_P162879	-2.7	2.7	3.4	1.8	APOPT1	Homo sapiens apoptogenic 1 (APOPT1), mRNA [NM_032374]	84334

A_24_P302833	-1.7	-2.3	-1.6	-1.2	APPL1	Homo sapiens adaptor protein, phosphotyrosine interaction, PH domain and leucine zipper containing 1 (APPL1), mRNA [NM_012096]	26060
A_23_P117955	1.1	-1.2	-1.7	-3.8	APRT	Homo sapiens adenine phosphoribosyltransferase (APRT), transcript variant 1, mRNA [NM_000485]	353
A_23_P252775	1.1	2.1	1.9	1.1	APTX	Homo sapiens aprataxin (APTX), transcript variant 1, mRNA [NM_175073]	54840

A_23_P19894	1.4	-3.5	-1.2	-1.2	AQP1	Homo sapiens aquaporin 1 (Colton blood group) (AQP1), transcript variant 1, mRNA [NM_198098]	358
A_23_P126613	1.9	2.2	4.3	1.5	AQP10	Homo sapiens aquaporin 10 (AQP10), mRNA [NM_080429]	89872
A_23_P149398	2.1	-1.1	1.6	-1.6	AQP10	aquaporin 10 [Source:HGNC Symbol;Acc:16029] [ENST00000484864]	89872
A_23_P204721	1.6	3.7	1.6	1.6	AQP2	Homo sapiens aquaporin 2 (collecting duct) (AQP2), mRNA [NM_000486]	359

A_23_P112482	-1.0	-1.1	-4.1	-1.1	AQP3	Homo sapiens aquaporin 3 (Gill blood group) (AQP3), mRNA [NM_004925]	360
A_23_P366812	2.8	-1.9	1.0	2.1	AQP5	Homo sapiens aquaporin 5 (AQP5), mRNA [NM_001651]	362
A_23_P502060	3.9	3.9	3.7	3.9	AQP6	Homo sapiens aquaporin 6, kidney specific (AQP6), mRNA [NM_001652]	363
A_24_P280378	1.3	-1.1	1.4	-4.3	AQR	Homo sapiens aquarius homolog (mouse) (AQR), mRNA [NM_014691]	9716

A_24_P398432	1.6	2.2	2.2	1.2	ARAP1	Homo sapiens ArfGAP with RhoGAP domain, ankyrin repeat and PH domain 1 (ARAP1), transcript variant 3, mRNA [NM_001040118]	116985
A_23_P167389	-5.8	1.0	1.0	1.1	ARAP3	Homo sapiens ArfGAP with RhoGAP domain, ankyrin repeat and PH domain 3 (ARAP3), mRNA [NM_022481]	64411
A_23_P203103	-2.1	-3.1	-2.1	1.8	ARCN1	Homo sapiens archain 1 (ARCN1), transcript variant 1, mRNA [NM_001655]	372

A_24_P190894	-1.0	-1.5	2.6	2.6	ARF1	Homo sapiens ADP-ribosylation factor 1 (ARF1), transcript variant 1, mRNA [NM_001024227]	375
A_23_P36521	1.7	2.3	-1.3	-1.5	ARF3	Homo sapiens ADP-ribosylation factor 3 (ARF3), mRNA [NM_001659]	377
A_24_P187750	-1.1	-6.6	-1.4	-1.4	ARF3	Homo sapiens ADP-ribosylation factor 3 (ARF3), mRNA [NM_001659]	377
A_23_P254601	2.1	-1.0	-1.1	1.3	ARF5	Homo sapiens ADP-ribosylation factor 5 (ARF5), mRNA [NM_001662]	381
A_23_P48691	-2.4	-1.0	-3.7	1.6	ARF6	Homo sapiens ADP-ribosylation factor 6 (ARF6), mRNA [NM_001663]	382

A_24_P271296	2.0	2.2	1.8	-1.1	ARFGAP1	Homo sapiens ADP-ribosylation factor GTPase activating protein 1 (ARFGAP1), transcript variant 2, mRNA [NM_175609]	55738
A_23_P210379	-1.6	-1.1	-2.3	-2.1	ARFGAP1	Homo sapiens ADP-ribosylation factor GTPase activating protein 1 (ARFGAP1), transcript variant 2, mRNA [NM_175609]	55738
A_23_P161552	-1.2	-3.7	-1.9	1.2	ARFGAP2	Homo sapiens ADP-ribosylation factor GTPase activating protein 2 (ARFGAP2), transcript variant 1, mRNA [NM_032389]	84364

A_23_P5995	3.9	3.8	3.7	1.1	ARFGEF2	Homo sapiens ADP-ribosylation factor guanine nucleotide-exchange factor 2 (brefeldin A-inhibited) (ARFGEF2), mRNA [NM_006420]	10564
A_23_P95050	1.2	1.1	-1.0	-3.3	ARFIP1	Homo sapiens ADP-ribosylation factor interacting protein 1 (ARFIP1), transcript variant 1, mRNA [NM_001025595]	27236
A_24_P166094	2.0	4.5	2.8	3.3	ARFIP1	Homo sapiens ADP-ribosylation factor interacting protein 1 (ARFIP1), transcript variant 1, mRNA [NM_001025595]	27236

A_23_P139228	-1.1	1.2	1.3	-2.8	ARFIP2	Homo sapiens ADP-ribosylation factor interacting protein 2 (ARFIP2), transcript variant 2, mRNA [NM_012402]	23647
A_23_P111321	-3.2	1.4	1.4	1.5	ARG1	Homo sapiens arginase, liver (ARG1), transcript variant 2, mRNA [NM_000045]	383
A_23_P128728	2.6	2.8	1.2	-1.7	ARG2	Homo sapiens arginase, type II (ARG2), nuclear gene encoding mitochondrial protein, mRNA [NM_001172]	384
A_23_P314070	1.3	1.0	2.6	2.3	ARHGAP1	Homo sapiens Rho GTPase activating protein 1 (ARHGAP1), mRNA [NM_004308]	392

A_23_P44057	-1.7	1.4	-1.2	2.7	ARHGAP1	Homo sapiens Rho GTPase activating protein 1 (ARHGAP1), mRNA [NM_004308]	392
A_23_P15113	5.5	6.9	5.9	1.0	ARHGAP17	Homo sapiens Rho GTPase activating protein 17 (ARHGAP17), transcript variant 1, mRNA [NM_001006634]	55114
A_23_P334845	-3.5	-4.5	1.0	-1.0	ARHGAP19	Homo sapiens Rho GTPase activating protein 19 (ARHGAP19), transcript variant 1, mRNA [NM_032900]	84986
A_23_P75310	-1.1	-6.8	1.0	-1.2	ARHGAP22	Homo sapiens Rho GTPase activating protein 22 (ARHGAP22), mRNA [NM_021226]	58504

A_24_P388659	-2.8	1.2	1.1	1.1	ARHGAP24	Homo sapiens Rho GTPase activating protein 24 (ARHGAP24), transcript variant 1, mRNA [NM_001025616]	83478
A_23_P214066	-1.0	1.6	-1.6	-2.2	ARHGAP26	Homo sapiens Rho GTPase activating protein 26 (ARHGAP26), transcript variant 1, mRNA [NM_015071]	23092
A_23_P401626	2.9	-1.1	1.7	2.3	ARHGAP27	Homo sapiens Rho GTPase activating protein 27 (ARHGAP27), transcript variant 3, mRNA [NM_174919]	201176

A_23_P141342	2.9	3.1	-1.2	-1.1	ARHGAP27	Homo sapiens Rho GTPase activating protein 27 (ARHGAP27), transcript variant 1, mRNA [NM_199282]	201176
A_23_P130359	-1.1	1.0	-3.4	1.8	ARHGAP28	Homo sapiens Rho GTPase activating protein 28 (ARHGAP28), mRNA [NM_001010000]	79822
A_23_P35045	-113.7	-1.2	-1.2	-1.3	ARHGAP30	Homo sapiens Rho GTPase activating protein 30 (ARHGAP30), transcript variant 1, mRNA [NM_001025598]	257106

A_24_P911231	1.1	-1.5	-3.4	-2.8	ARHGAP30	Homo sapiens Rho GTPase activating protein 30 (ARHGAP30), transcript variant 2, mRNA [NM_181720]	257106
A_24_P349039	2.4	2.4	-1.8	3.5	ARHGAP31	Homo sapiens Rho GTPase activating protein 31 (ARHGAP31), mRNA [NM_020754]	57514
A_32_P226620	1.1	-2.6	1.0	1.0	ARHGAP31	Rho GTPase activating protein 31 [Source:HGNC Symbol;Acc:29216] [ENST00000264245]	57514
A_24_P42436	2.0	1.0	1.6	1.1	ARHGAP32	Homo sapiens Rho GTPase activating protein 32 (ARHGAP32), transcript variant 2, mRNA [NM_014715]	9743

A_24_P36299	1.1	2.1	-1.1	1.1	ARHGAP35	Homo sapiens Rho GTPase activating protein 35 (ARHGAP35), mRNA [NM_004491]	2909
A_24_P41864	-1.0	-2.2	-1.0	-1.3	ARHGAP6	Homo sapiens Rho GTPase activating protein 6 (ARHGAP6), transcript variant 3, mRNA [NM_006125]	395
A_23_P64661	1.0	1.3	-5.5	1.0	ARHGAP9	Homo sapiens Rho GTPase activating protein 9 (ARHGAP9), transcript variant 1, mRNA [NM_032496]	64333

A_24_P133933	-1.5	-1.1	-5.3	-3.0	ARHGDIA	Homo sapiens Rho GDP dissociation inhibitor (GDI) alpha (ARHGDIA), transcript variant 2, mRNA [NM_004309]	396
A_23_P151075	1.5	2.2	2.7	1.1	ARHGDIB	Homo sapiens Rho GDP dissociation inhibitor (GDI) beta (ARHGDIB), mRNA [NM_001175]	397
A_24_P215882	3.7	5.6	2.8	2.7	ARHGDIG	Homo sapiens Rho GDP dissociation inhibitor (GDI) gamma (ARHGDIG), mRNA [NM_001176]	398

A_24_P202581	1.3	2.8	1.7	1.6	ARHGEF1	Homo sapiens Rho guanine nucleotide exchange factor (GEF) 1 (ARHGEF1), transcript variant 1, mRNA [NM_199002]	9138
A_24_P188085	-18.5	-1.1	-1.2	-1.2	ARHGEF10	Rho guanine nucleotide exchange factor (GEF) 10 [Source:HGNC Symbol;Acc:14103] [ENST00000523711]	9639
A_23_P392024	-1.1	1.0	-1.0	-2.0	ARHGEF10	Rho guanine nucleotide exchange factor (GEF) 10 [Source:HGNC Symbol;Acc:14103] [ENST00000522969]	9639

A_24_P283535	1.6	1.7	2.2	1.0	ARHGEF10	Homo sapiens Rho guanine nucleotide exchange factor (GEF) 10 (ARHGEF10), mRNA [NM_014629]	9639
A_24_P175783	-2.8	-3.8	-1.3	-1.4	ARHGEF12	Homo sapiens Rho guanine nucleotide exchange factor (GEF) 12 (ARHGEF12), transcript variant 1, mRNA [NM_015313]	23365
A_24_P359007	-1.5	-1.6	-1.2	-2.5	ARHGEF15	Homo sapiens cDNA FLJ13791 fis, clone THYRO1000070. [AK023853]	22899

A_23_P49674	1.9	2.5	2.4	1.2	ARHGEF15	Homo sapiens Rho guanine nucleotide exchange factor (GEF) 15 (ARHGEF15), transcript variant 1, mRNA [NM_173728]	22899
A_23_P114670	-1.3	1.6	-1.0	2.2	ARHGEF16	Homo sapiens Rho guanine nucleotide exchange factor (GEF) 16 (ARHGEF16), mRNA [NM_014448]	27237
A_23_P50357	-1.1	-1.2	-2.8	-1.3	ARHGEF18	Homo sapiens Rho/Rac guanine nucleotide exchange factor (GEF) 18 (ARHGEF18), transcript variant 1, mRNA [NM_015318]	23370

A_23_P332326	1.8	-8.5	-1.0	-1.1	ARHGEF19	Homo sapiens Rho guanine nucleotide exchange factor (GEF) 19 (ARHGEF19), mRNA [NM_153213]	128272
A_23_P98844	4.3	2.2	2.3	2.2	ARHGEF25	Homo sapiens Rho guanine nucleotide exchange factor (GEF) 25 (ARHGEF25), transcript variant 1, mRNA [NM_182947]	115557
A_24_P740549	1.5	2.9	1.4	-2.3	ARHGEF26-AS	Homo sapiens ARHGEF26 antisense RNA 1 (non-protein coding) (ARHGEF26-AS1), non-coding RNA [NR_037901]	100507524

A_23_P253221	-5.1	1.7	1.6	-3.9	ARHGEF4	Homo sapiens Rho guanine nucleotide exchange factor (GEF) 4 (ARHGEF4), transcript variant 2, mRNA [NM_032995]	50649
A_24_P302685	16.0	14.8	15.4	5.3	ARHGEF4	Homo sapiens Rho guanine nucleotide exchange factor (GEF) 4 (ARHGEF4), transcript variant 1, mRNA [NM_015320]	50649
A_23_P85024	1.6	2.9	1.1	-1.2	ARHGEF6	Homo sapiens Rac/Cdc42 guanine nucleotide exchange factor (GEF) 6 (ARHGEF6), mRNA [NM_004840]	9459

A_23_P417891	2.7	1.9	2.2	1.3	ARHGEF7	Homo sapiens Rho guanine nucleotide exchange factor (GEF) 7 (ARHGEF7), transcript variant 2, mRNA [NM_145735]	8874
A_23_P321855	1.3	1.1	2.1	1.5	ARHGEF7	Homo sapiens Rho guanine nucleotide exchange factor (GEF) 7 (ARHGEF7), transcript variant 1, mRNA [NM_003899]	8874
A_24_P254551	2.3	1.6	1.6	1.8	ARHGEF9	Homo sapiens Cdc42 guanine nucleotide exchange factor (GEF) 9 (ARHGEF9), transcript variant 1, mRNA [NM_015185]	23229

A_24_P92952	2.0	4.9	-1.1	1.4	ARID1A	Homo sapiens AT rich interactive domain 1A (SWI-like) (ARID1A), transcript variant 1, mRNA [NM_006015]	8289
A_23_P88580	-1.7	-7.9	-2.0	-2.8	ARID3B	Homo sapiens AT rich interactive domain 3B (BRIGHT-like) (ARID3B), mRNA [NM_006465]	10620
A_23_P143016	3.0	2.1	2.0	2.5	ARID5A	Homo sapiens AT rich interactive domain 5A (MRF1-like) (ARID5A), mRNA [NM_212481]	10865
A_24_P929441	-2.2	-1.0	1.5	1.2	ARID5A	Homo sapiens clone DNA128322 RFVG5814 (UNQ5814) mRNA, complete cds. [AY358167]	10865

A_32_P18440	2.4	2.7	1.8	1.3	ARID5B	Homo sapiens AT rich interactive domain 5B (MRF1-like) (ARID5B), transcript variant 1, mRNA [NM_032199]	84159
A_32_P68586	-3.8	-1.1	-1.4	1.6	ARL1	Homo sapiens ADP-ribosylation factor-like 1 (ARL1), mRNA [NM_001177]	400
A_32_P36101	-5.0	-2.2	-3.6	-2.0	ARL1	Homo sapiens ADP-ribosylation factor-like 1 (ARL1), mRNA [NM_001177]	400
A_24_P378202	1.4	1.4	-1.4	-3.3	ARL11	Homo sapiens ADP-ribosylation factor-like 11 (ARL11), mRNA [NM_138450]	115761

A_24_P328668	3.1	-1.1	2.9	3.3	ARL13B	Homo sapiens ADP-ribosylation factor-like 13B (ARL13B), transcript variant 1, mRNA [NM_182896]	200894
A_23_P92161	-5.0	1.0	1.0	1.0	ARL14	Homo sapiens ADP-ribosylation factor-like 14 (ARL14), mRNA [NM_025047]	80117
A_24_P286935	13.3	11.9	9.1	8.1	ARL3	Homo sapiens ADP-ribosylation factor-like 3 (ARL3), mRNA [NM_004311]	403
A_23_P145761	2.3	-1.1	-1.3	-1.5	ARL4A	Homo sapiens ADP-ribosylation factor-like 4A (ARL4A), transcript variant 1, mRNA [NM_005738]	10124

A_24_P356218	-2.1	-2.7	-3.1	-1.2	ARL6IP1	Homo sapiens ADP-ribosylation factor-like 6 interacting protein 1 (ARL6IP1), mRNA [NM_015161]	23204
A_24_P287233	1.5	1.8	-4.1	1.6	ARL6IP4	Homo sapiens ADP-ribosylation-like factor 6 interacting protein 4 (ARL6IP4), transcript variant 4, mRNA [NM_001002252]	51329
A_23_P415033	1.8	5.0	1.9	3.6	ARL8A	Homo sapiens ADP-ribosylation factor-like 8A (ARL8A), mRNA [NM_138795]	127829
A_24_P377065	-1.4	-2.6	1.3	1.1	ARL8B	Homo sapiens ADP-ribosylation factor-like 8B (ARL8B), mRNA [NM_018184]	55207

A_32_P234827	1.0	-1.0	-1.1	-2.3	ARMC1	Homo sapiens armadillo repeat containing 1 (ARMC1), mRNA [NM_018120]	55156
A_32_P141418	2.9	-1.0	-1.1	1.0	ARMC4	Homo sapiens armadillo repeat containing 4 (ARMC4), mRNA [NM_018076]	55130
A_23_P425750	1.5	-18.3	1.5	1.3	ARMC6	Homo sapiens armadillo repeat containing 6 (ARMC6), transcript variant 2, mRNA [NM_033415]	93436
A_24_P23411	-13.4	-1.0	-1.1	-1.0	ARMCX3	Homo sapiens armadillo repeat containing, X-linked 3 (ARMCX3), transcript variant 1, mRNA [NM_016607]	51566

A_24_P374375	-2.3	1.1	1.0	1.1	ARMCX4	Homo sapiens armadillo repeat containing, X-linked 4 (ARMCX4), non-coding RNA [NR_028407]	100131755
A_23_P550	1.1	-6.7	1.0	1.1	ARNT	Homo sapiens aryl hydrocarbon receptor nuclear translocator (ARNT), transcript variant 1, mRNA [NM_001668]	405
A_23_P83579	-1.2	-1.5	-2.2	-1.2	ARNT2	Homo sapiens aryl-hydrocarbon receptor nuclear translocator 2 (ARNT2), mRNA [NM_014862]	9915

A_32_P399546	-3.9	1.1	-1.0	-1.0	ARNTL2	Homo sapiens aryl hydrocarbon receptor nuclear translocator-like 2 (ARNTL2), transcript variant 1, mRNA [NM_020183]	56938
A_23_P53345	-1.7	-1.7	-1.7	-3.0	ARNTL2	Homo sapiens aryl hydrocarbon receptor nuclear translocator-like 2 (ARNTL2), transcript variant 1, mRNA [NM_020183]	56938
A_23_P20196	2.0	1.1	-2.5	1.1	ARPC1B	Homo sapiens actin related protein 2/3 complex, subunit 1B, 41kDa (ARPC1B), mRNA [NM_005720]	10095

A_23_P102122	1.4	1.1	1.8	4.8	ARPC2	Homo sapiens actin related protein 2/3 complex, subunit 2, 34kDa (ARPC2), transcript variant 1, mRNA [NM_152862]	10109
A_24_P167473	1.3	-3.6	1.2	-1.0	ARPC3	Homo sapiens actin related protein 2/3 complex, subunit 3, 21kDa (ARPC3), mRNA [NM_005719]	10094
A_24_P20383	1.8	2.6	2.2	5.6	ARPC4	Homo sapiens actin related protein 2/3 complex, subunit 4, 20kDa (ARPC4), transcript variant 1, mRNA [NM_005718]	10093

A_23_P391382	-1.8	1.4	-2.0	1.0	ARPC4-TTLL3	Homo sapiens cDNA FLJ14048 fis, clone HEMBA1006650, weakly similar to ARP2/3 COMPLEX 20 KD SUBUNIT. [AK024110]	100526693
A_23_P126803	-1.8	-2.1	-1.5	-1.0	ARPC5	Homo sapiens actin related protein 2/3 complex, subunit 5, 16kDa (ARPC5), mRNA [NM_005717]	10092
A_23_P121447	1.1	1.1	-3.1	1.0	ARPM1	Homo sapiens actin related protein M1 (ARPM1), mRNA [NM_032487]	84517
A_24_P344976	1.0	-1.0	-1.1	-15.5	ARR3	Homo sapiens arrestin 3, retinal (X-arrestin) (ARR3), mRNA [NM_004312]	407

A_24_P386622	-1.1	2.2	-1.1	-1.1	ARRB1	Homo sapiens arrestin, beta 1 (ARRB1), transcript variant 1, mRNA [NM_004041]	408
A_23_P203702	1.1	-4.5	1.2	-1.1	ARRB1	Homo sapiens arrestin, beta 1 (ARRB1), transcript variant 1, mRNA [NM_004041]	408
A_24_P22976	-4.1	1.2	-1.1	-1.0	ARRDC2	Homo sapiens arrestin domain containing 2 (ARRDC2), transcript variant 2, mRNA [NM_001025604]	27106
A_24_P274615	1.9	2.0	4.3	1.0	ARRDC3	Homo sapiens arrestin domain containing 3 (ARRDC3), mRNA [NM_020801]	57561

A_24_P414553	1.1	-1.0	1.1	-9.9	ARRDC3	Homo sapiens arrestin domain containing 3 (ARRDC3), mRNA [NM_020801]	57561
A_23_P339818	1.1	-2.7	-1.1	-1.0	ARRDC4	Homo sapiens arrestin domain containing 4 (ARRDC4), mRNA [NM_183376]	91947
A_32_P30271	1.5	1.5	2.4	1.5	ARRDC5	Homo sapiens arrestin domain containing 5 (ARRDC5), mRNA [NM_001080523]	645432
A_23_P255436	1.7	2.5	-1.0	1.2	ARSA	Homo sapiens arylsulfatase A (ARSA), transcript variant 1, mRNA [NM_000487]	410
A_23_P217712	-2.6	-1.2	-1.1	-2.0	ARSD	Homo sapiens arylsulfatase D (ARSD), mRNA [NM_001669]	414

A_23_P304304	5.2	3.0	2.6	5.7	ARSF	Homo sapiens arylsulfatase F (ARSF), transcript variant 1, mRNA [NM_004042]	416
A_24_P339416	1.6	1.9	-1.6	-2.5	ARSG	Homo sapiens arylsulfatase G (ARSG), mRNA [NM_014960]	22901
A_23_P87363	1.2	-2.4	1.8	1.6	ART1	Homo sapiens ADP-ribosyltransferase 1 (ART1), mRNA [NM_004314]	417
A_24_P251040	3.0	2.4	-1.2	1.3	ARTN	Homo sapiens artemin (ARTN), transcript variant 2, mRNA [NM_057091]	9048
A_24_P144527	-9.4	1.1	1.1	-1.0	ARV1	Homo sapiens ARV1 homolog (S. cerevisiae) (ARV1), mRNA [NM_022786]	64801

A_23_P135995	1.7	1.7	-1.1	-2.5	ARV1	Homo sapiens ARV1 homolog (S. cerevisiae) (ARV1), mRNA [NM_022786]	64801
A_23_P109393	2.8	-1.1	2.1	1.0	ARVCF	Homo sapiens armadillo repeat gene deleted in velocardiofacial syndrome (ARVCF), mRNA [NM_001670]	421
A_23_P44831	-1.2	-1.2	-1.7	-3.6	ASAP1	Homo sapiens ArfGAP with SH3 domain, ankyrin repeat and PH domain 1 (ASAP1), transcript variant 1, mRNA [NM_018482]	50807

A_23_P114689	-2.1	-2.8	-1.7	-1.3	ASAP3	Homo sapiens ArfGAP with SH3 domain, ankyrin repeat and PH domain 3 (ASAP3), transcript variant 1, mRNA [NM_017707]	55616
A_23_P165360	-1.8	2.3	-1.4	-1.2	ASB1	Homo sapiens ankyrin repeat and SOCS box containing 1 (ASB1), mRNA [NM_001040445]	51665
A_23_P427634	-1.1	3.0	3.2	1.4	ASB10	Homo sapiens ankyrin repeat and SOCS box containing 10 (ASB10), transcript variant 3, mRNA [NM_080871]	136371

A_23_P114259	-1.0	-3.6	-1.0	-1.0	ASB11	Homo sapiens ankyrin repeat and SOCS box containing 11 (ASB11), transcript variant 1, mRNA [NM_080873]	140456
A_23_P202510	1.2	2.9	1.2	-1.1	ASB13	Homo sapiens ankyrin repeat and SOCS box containing 13 (ASB13), transcript variant 1, mRNA [NM_024701]	79754
A_23_P155265	-4.9	1.9	1.5	1.0	ASB14	Homo sapiens ankyrin repeat and SOCS box containing 14 (ASB14), transcript variant 2, mRNA [NM_130387]	142686

A_24_P56837	1.4	2.6	1.1	-1.4	ASB16	Homo sapiens ankyrin repeat and SOCS box containing 16 (ASB16), mRNA [NM_080863]	92591
A_23_P205370	1.1	-25.7	1.6	1.1	ASB2	Homo sapiens ankyrin repeat and SOCS box containing 2 (ASB2), transcript variant 2, mRNA [NM_016150]	51676
A_23_P56833	2.2	1.5	1.9	1.9	ASB3	Homo sapiens ankyrin repeat and SOCS box containing 3 (ASB3), transcript variant 1, mRNA [NM_016115]	51130

A_23_P144326	1.1	1.0	1.0	-5.7	ASB5	Homo sapiens ankyrin repeat and SOCS box containing 5 (ASB5), mRNA [NM_080874]	140458
A_24_P155791	-1.7	-1.0	-2.4	-1.0	ASB7	Homo sapiens ankyrin repeat and SOCS box containing 7 (ASB7), transcript variant 1, mRNA [NM_024708]	140460
A_23_P117743	-19.5	1.1	1.0	1.1	ASB7	Homo sapiens ankyrin repeat and SOCS box containing 7 (ASB7), transcript variant 1, mRNA [NM_024708]	140460
A_24_P643587	-11.7	-1.6	-1.0	-2.5	ASB8	Homo sapiens ankyrin repeat and SOCS box containing 8 (ASB8), mRNA [NM_024095]	140461

A_23_P22200	2.5	1.2	1.4	1.3	ASB8	Homo sapiens ankyrin repeat and SOCS box containing 8 (ASB8), mRNA [NM_024095]	140461
A_23_P1280	1.1	-2.8	1.0	1.0	ASCC1	Homo sapiens activating signal cointegrator 1 complex subunit 1 (ASCC1), transcript variant 3, mRNA [NM_015947]	51008
A_23_P143643	1.8	2.1	1.9	1.2	ASCC2	Homo sapiens activating signal cointegrator 1 complex subunit 2 (ASCC2), transcript variant 1, mRNA [NM_032204]	84164

A_23_P30972	-2.1	-1.5	-1.8	-1.5	ASCC3	Homo sapiens activating signal cointegrator 1 complex subunit 3 (ASCC3), transcript variant 2, mRNA [NM_022091]	10973
A_23_P25305	1.5	2.0	2.5	1.4	ASCL1	Homo sapiens achaete-scute complex homolog 1 (Drosophila) (ASCL1), mRNA [NM_004316]	429
A_23_P2083	2.4	2.4	-1.1	-2.3	ASCL3	Homo sapiens achaete-scute complex homolog 3 (Drosophila) (ASCL3), mRNA [NM_020646]	56676

A_23_P119254	52.5	36.6	14.0	7.6	ASF1B	Homo sapiens ASF1 anti-silencing function 1 homolog B (S. cerevisiae) (ASF1B), mRNA [NM_018154]	55723
A_24_P367666	1.0	-1.6	-4.1	-4.1	ASH1L	Homo sapiens ash1 (absent, small, or homeotic)-like (Drosophila) (ASH1L), mRNA [NM_018489]	55870
A_23_P120629	2.4	1.6	2.9	2.0	ASIP	Homo sapiens agouti signaling protein (ASIP), mRNA [NM_001672]	434
A_23_P26223	-1.5	2.1	-1.1	1.0	ASL	Homo sapiens argininosuccinate lyase (ASL), transcript variant 1, mRNA [NM_001024943]	435

A_23_P255535	2.2	2.2	2.1	2.4	ASMT	Homo sapiens acetylserotonin O-methyltransferase (ASMT), transcript variant 2, mRNA [NM_004043]	438
A_23_P159544	1.4	1.9	-2.3	1.8	ASMTL	Homo sapiens acetylserotonin O-methyltransferase-like (ASMTL), transcript variant 1, mRNA [NM_004192]	8623
A_24_P602013	3.3	1.8	2.4	3.1	ASPA	aspartoacylase [Source:HGNC Symbol;Acc:756] [ENST00000263080]	443
A_24_P690811	-1.2	-3.6	-1.3	-2.4	ASPDH	Homo sapiens aspartate dehydrogenase domain containing (ASPDH), transcript variant 2, mRNA [NM_001024656]	554235

A_23_P76743	-1.8	-1.1	-2.1	-2.5	ASPG	Homo sapiens asparaginase homolog (S. cerevisiae) (ASPG), mRNA [NM_001080464]	374569
A_23_P216094	2.1	-1.0	-1.1	-1.0	ASPH	Homo sapiens aspartate beta-hydroxylase (ASPH), transcript variant 1, mRNA [NM_004318]	444
A_24_P18105	-2.4	-7.7	-2.4	-2.4	ASPH	Homo sapiens aspartate beta-hydroxylase (ASPH), transcript variant 3, mRNA [NM_032466]	444
A_23_P170667	-4.3	-3.3	-1.2	-1.8	ASPHD1	Homo sapiens aspartate beta-hydroxylase domain containing 1 (ASPHD1), mRNA [NM_181718]	253982

A_23_P216429	-2.1	1.0	1.1	1.0	ASPN	Homo sapiens asporin (ASPN), transcript variant 1, mRNA [NM_017680]	54829
A_23_P379864	-3.3	-1.1	-1.2	-1.1	ASRGL1	Homo sapiens asparaginase like 1 (ASRGL1), transcript variant 1, mRNA [NM_001083926]	80150
A_23_P1936	-3.4	-1.7	-1.1	1.0	ASRGL1	Homo sapiens asparaginase like 1 (ASRGL1), transcript variant 2, mRNA [NM_025080]	80150
A_23_P102439	4.1	1.9	1.3	-2.8	ASTL	Homo sapiens astacin-like metallo-endopeptidase (M12 family) (ASTL), mRNA [NM_001002036]	431705

A_24_P83738	-1.1	1.1	-10.9	-1.4	ASTN2	Homo sapiens astrotactin 2 (ASTN2), transcript variant 4, mRNA [NM_198188]	23245
A_32_P41021	-10.2	1.0	1.0	1.0	ASXL1	Homo sapiens additional sex combs like 1 (Drosophila) (ASXL1), transcript variant 1, mRNA [NM_015338]	171023
A_24_P359771	-13.0	1.5	1.3	1.1	ASXL2	additional sex combs like 2 (Drosophila) [Source:HGNC Symbol;Acc:23805] [ENST00000336112]	55252
A_23_P210300	-94.1	-16.3	-18.2	-13.6	ASXL2	Homo sapiens additional sex combs like 2 (Drosophila) (ASXL2), mRNA [NM_018263]	55252

A_24_P329777	2.4	5.1	1.3	2.3	ATAD1	Homo sapiens ATPase family, AAA domain containing 1 (ATAD1), mRNA [NM_032810]	84896
A_23_P74688	2.4	2.8	-1.0	1.1	ATAD3B	Homo sapiens ATPase family, AAA domain containing 3B (ATAD3B), nuclear gene encoding mitochondrial protein, mRNA [NM_031921]	83858
A_32_P353677	1.0	-1.0	-1.1	-5.5	ATAD3B	Homo sapiens TOB3 mRNA, complete cds. [AF343078]	83858

A_23_P302750	1.3	2.0	1.2	2.0	ATAD3B	ATPase family, AAA domain containing 3B [Source:HGNC Symbol;Acc:24007] [ENST00000472194]	83858
A_24_P113144	1.1	1.1	-2.4	1.0	ATAD5	Homo sapiens ATPase family, AAA domain containing 5 (ATAD5), mRNA [NM_024857]	79915
A_23_P255750	-1.1	-3.1	-2.2	-1.1	ATAD5	Homo sapiens ATPase family, AAA domain containing 5 (ATAD5), mRNA [NM_024857]	79915

A_23_P70509	2.8	2.6	2.8	-1.1	ATAT1	Homo sapiens alpha tubulin acetyltransferase 1 (ATAT1), transcript variant 2, mRNA [NM_024909]	79969
A_24_P40094	-1.3	-1.3	1.1	-2.4	ATCAY	Homo sapiens ataxia, cerebellar, Cayman type (ATCAY), mRNA [NM_033064]	85300
A_23_P433063	3.2	5.5	-1.2	-3.7	ATCAY	Homo sapiens ataxia, cerebellar, Cayman type (ATCAY), mRNA [NM_033064]	85300
A_23_P209879	-1.1	-5.4	-1.3	-1.3	ATF2	activating transcription factor 2 [Source:HGNC Symbol;Acc:784] [ENST00000542046]	1386

A_24_P128145	-2.5	-1.3	-5.1	-2.9	ATF2	Homo sapiens activating transcription factor 2 (ATF2), mRNA [NM_001880]	1386
A_24_P33895	-2.8	1.0	1.0	1.1	ATF3	Homo sapiens activating transcription factor 3 (ATF3), transcript variant 4, mRNA [NM_001040619]	467
A_23_P62907	1.1	1.9	-1.2	-2.0	ATF6	Homo sapiens activating transcription factor 6 (ATF6), mRNA [NM_007348]	22926
A_23_P59163	1.8	2.1	2.1	3.4	ATF6B	Homo sapiens activating transcription factor 6 beta (ATF6B), transcript variant 1, mRNA [NM_004381]	1388

A_24_P419109	2.1	-1.1	1.1	1.4	ATF6B	Homo sapiens activating transcription factor 6 beta (ATF6B), transcript variant 1, mRNA [NM_004381]	1388
A_23_P112950	5.4	6.9	4.4	2.5	ATF7IP	Homo sapiens activating transcription factor 7 interacting protein (ATF7IP), mRNA [NM_018179]	55729
A_23_P92824	1.2	-1.1	-2.1	1.3	ATG10	Homo sapiens ATG10 autophagy related 10 homolog (S. cerevisiae) (ATG10), transcript variant 2, mRNA [NM_031482]	83734

A_23_P95292	2.4	-1.7	3.3	2.2	ATG13	Homo sapiens ATG13 autophagy related 13 homolog (S. cerevisiae) (ATG13), transcript variant 2, mRNA [NM_014741]	9776
A_23_P309989	1.5	4.2	2.2	2.4	ATG14	Homo sapiens ATG14 autophagy related 14 homolog (S. cerevisiae) (ATG14), mRNA [NM_014924]	22863
A_23_P315378	-2.6	1.0	-1.0	1.0	ATG16L1	ATG16 autophagy related 16-like 1 (S. cerevisiae) [Source:HGNC Symbol;Acc:21498] [ENST00000472242]	55054

A_23_P36305	-1.2	-2.0	-2.0	-1.3	ATG16L2	Homo sapiens ATG16 autophagy related 16-like 2 (S. cerevisiae) (ATG16L2), mRNA [NM_033388]	89849
A_24_P196382	1.2	-2.1	1.4	1.5	ATG3	Homo sapiens ATG3 autophagy related 3 homolog (S. cerevisiae), mRNA (cDNA clone MGC:3478 IMAGE:3633903), complete cds. [BC002830]	64422
A_23_P212706	1.1	-2.6	-1.4	1.5	ATG3	Homo sapiens ATG3 autophagy related 3 homolog (S. cerevisiae) (ATG3), mRNA [NM_022488]	64422

A_23_P217367	-1.9	-17.8	1.2	1.1	ATG4A	Homo sapiens ATG4 autophagy related 4 homolog A (S. cerevisiae) (ATG4A), transcript variant 1, mRNA [NM_052936]	115201
A_24_P182764	1.0	-2.2	1.0	1.0	ATG4B	Homo sapiens ATG4 autophagy related 4 homolog B (S. cerevisiae) (ATG4B), transcript variant 2, mRNA [NM_178326]	23192
A_23_P9903	-1.0	-1.0	-1.1	-7.1	ATG4B	Homo sapiens ATG4 autophagy related 4 homolog B (S. cerevisiae) (ATG4B), transcript variant 2, mRNA [NM_178326]	23192

A_24_P182539	-3.5	1.3	1.2	1.0	ATG4C	Homo sapiens ATG4 autophagy related 4 homolog C (S. cerevisiae) (ATG4C), transcript variant 1, mRNA [NM_032852]	84938
A_24_P175059	16.1	16.0	15.2	14.1	ATG5	Homo sapiens ATG5 autophagy related 5 homolog (S. cerevisiae) (ATG5), mRNA [NM_004849]	9474
A_23_P143987	-1.6	1.5	2.3	1.4	ATG7	Homo sapiens ATG7 autophagy related 7 homolog (S. cerevisiae) (ATG7), transcript variant 1, mRNA [NM_006395]	10533

A_24_P944827	-1.0	1.7	-1.4	-2.8	ATG7	Homo sapiens ATG7 autophagy related 7 homolog (S. cerevisiae) (ATG7), transcript variant 1, mRNA [NM_006395]	10533
A_32_P379379	-1.5	-1.5	-2.3	-1.9	ATG9B	Homo sapiens ATG9 autophagy related 9 homolog B (S. cerevisiae) (ATG9B), mRNA [NM_173681]	285973
A_23_P98686	2.6	4.8	2.8	-1.1	ATHL1	Homo sapiens ATH1, acid trehalase-like 1 (yeast) (ATHL1), mRNA [NM_025092]	80162

A_23_P68087	-3.3	-2.3	-1.2	-1.2	ATIC	Homo sapiens 5-aminoimidazole-4-carboxamide ribonucleotide formyltransferase/IMP cyclohydrolase (ATIC), mRNA [NM_004044]	471
A_23_P88351	2.1	2.1	2.2	-4.4	ATL1	Homo sapiens atlastin GTPase 1 (ATL1), transcript variant 2, mRNA [NM_181598]	51062
A_23_P105028	1.4	-3.4	1.0	1.1	ATL3	Homo sapiens atlastin GTPase 3 (ATL3), mRNA [NM_015459]	25923
A_23_P13885	-1.9	-1.6	-2.1	-2.1	ATN1	Homo sapiens atrophin 1 (ATN1), transcript variant 1, mRNA [NM_001007026]	1822

A_23_P332246	-4.9	-1.0	-1.1	-1.1	ATOH1	Homo sapiens atonal homolog 1 (Drosophila) (ATOH1), mRNA [NM_005172]	474
A_23_P378514	-1.0	-1.3	-3.9	-2.3	ATOH7	Homo sapiens atonal homolog 7 (Drosophila) (ATOH7), mRNA [NM_145178]	220202
A_23_P144877	-1.0	4.9	-1.5	-1.1	ATOX1	Homo sapiens ATX1 antioxidant protein 1 homolog (yeast) (ATOX1), mRNA [NM_004045]	475
A_23_P65659	3.7	2.0	2.2	1.8	ATP10A	Homo sapiens ATPase, class V, type 10A (ATP10A), mRNA [NM_024490]	57194

A_23_P311901	1.1	-1.2	-1.1	-2.1	ATP10B	Homo sapiens ATPase, class V, type 10B (ATP10B), mRNA [NM_025153]	23120
A_24_P161973	-1.3	1.0	1.0	-2.9	ATP11A	Homo sapiens ATPase, class VI, type 11A (ATP11A), transcript variant 1, mRNA [NM_015205]	23250
A_24_P59403	1.0	-3.4	-1.0	1.1	ATP11A	Homo sapiens ATPase, class VI, type 11A (ATP11A), transcript variant 1, mRNA [NM_015205]	23250
A_23_P212522	-1.1	-1.2	-3.1	-4.1	ATP11B	Homo sapiens ATPase, class VI, type 11B (ATP11B), mRNA [NM_014616]	23200

A_24_P157415	-3.3	-1.2	1.4	-1.3	ATP11B	Homo sapiens ATPase, class VI, type 11B (ATP11B), mRNA [NM_014616]	23200
A_24_P340428	1.1	1.1	1.0	-6.7	ATP11B	Homo sapiens ATPase, class VI, type 11B (ATP11B), mRNA [NM_014616]	23200
A_24_P152310	-7.0	-1.3	1.1	-1.1	ATP11B	Homo sapiens ATPase, class VI, type 11B (ATP11B), mRNA [NM_014616]	23200
A_24_P399942	1.5	2.4	1.2	4.0	ATP11C	Homo sapiens ATPase, class VI, type 11C (ATP11C), transcript variant 1, mRNA [NM_173694]	286410

A_23_P251730	1.5	1.9	-2.4	1.4	ATP11C	Homo sapiens ATPase, class VI, type 11C (ATP11C), transcript variant 2, mRNA [NM_0010 10986]	286410
A_23_P87982	3.0	3.4	2.3	2.9	ATP12A	Homo sapiens ATPase, H+/K+ transporti ng, nongastric , alpha polypeptid e (ATP12A), transcript variant 2, mRNA [NM_0016 76]	479
A_23_P12423	-1.5	-1.7	-1.5	-2.5	ATP13A2	Homo sapiens ATPase type 13A2 (ATP13A2) , transcript variant 1, mRNA [NM_0220 89]	23400
A_23_P29394	-1.1	-11.6	-1.2	-1.7	ATP13A4	Homo sapiens ATPase type 13A4 (ATP13A4) , mRNA [NM_0322 79]	84239

A_23_P149695	-1.3	-1.0	-1.6	-4.5	ATP1A1OS	Homo sapiens ATP1A1 opposite strand (ATP1A1OS), transcript variant 3, non-coding RNA [NR_024126]	84852
A_23_P27472	-4.5	-3.7	-2.9	-2.6	ATP1A3	Homo sapiens ATPase, Na+/K+ transporting, alpha 3 polypeptide (ATP1A3), mRNA [NM_152296]	478
A_23_P160177	2.3	2.7	-1.0	1.3	ATP1A4	Homo sapiens ATPase, Na+/K+ transporting, alpha 4 polypeptide (ATP1A4), transcript variant 1, mRNA [NM_144699]	480

A_24_P31275	2.5	-1.1	1.8	1.7	ATP1B2	Homo sapiens ATPase, Na+/K+ transporting, beta 2 polypeptide (ATP1B2), mRNA [NM_001678]	482
A_23_P217430	1.0	-1.0	-3.5	-1.1	ATP1B4	Homo sapiens ATPase, Na+/K+ transporting, beta 4 polypeptide (ATP1B4), transcript variant 1, mRNA [NM_001142447]	23439
A_23_P72462	-2.8	1.2	1.1	1.4	ATP2A1	Homo sapiens ATPase, Ca++ transporting, cardiac muscle, fast twitch 1 (ATP2A1), transcript variant b, mRNA [NM_173201]	487

A_24_P202319	-1.3	-1.8	-1.2	-2.6	ATP2A3	Homo sapiens ATPase, Ca++ transporting, ubiquitous (ATP2A3), transcript variant 7, mRNA [NM_174958]	489
A_23_P207632	-6.7	-1.0	-1.0	-1.0	ATP2A3	Homo sapiens ATPase, Ca++ transporting, ubiquitous (ATP2A3), transcript variant 5, mRNA [NM_174953]	489
A_23_P128319	-1.4	1.1	-2.0	1.5	ATP2B1	Homo sapiens ATPase, Ca++ transporting, plasma membrane 1 (ATP2B1), transcript variant 2, mRNA [NM_001682]	490

A_23_P337642	-4.7	-2.1	-1.2	-1.3	ATP2B3	Homo sapiens ATPase, Ca++ transporting, plasma membrane 3 (ATP2B3), transcript variant 1, mRNA [NM_021949]	492
A_23_P11841	-1.3	-1.3	-2.1	1.3	ATP2B4	Homo sapiens ATPase, Ca++ transporting, plasma membrane 4 (ATP2B4), transcript variant 2, mRNA [NM_001684]	493
A_23_P117992	-4.3	-1.8	-1.1	-2.3	ATP2C2	Homo sapiens ATPase, Ca++ transporting, type 2C, member 2 (ATP2C2), mRNA [NM_014861]	9914

A_24_P245358	1.3	-2.7	-1.3	1.6	ATP5A1	Homo sapiens ATP synthase, H+ transporting, mitochondrial F1 complex, alpha subunit 1, cardiac muscle (ATP5A1), nuclear gene encoding mitochondrial protein, transcript variant 1, mRNA [NM_001001937]	498

A_23_P33216	3.3	3.2	5.2	5.0	ATP5B	Homo sapiens ATP synthase, H+ transporting, mitochondrial F1 complex, beta polypeptide (ATP5B), nuclear gene encoding mitochondrial protein, mRNA [NM_001686]	506
A_23_P63655	-11.9	1.1	1.1	1.0	ATP5C1	Homo sapiens ATP synthase, H+ transporting, mitochondrial F1 complex, gamma polypeptide 1 (ATP5C1), nuclear gene encoding mitochondrial protein, transcript variant 2, mRNA [NM_005174]	509

A_24_P168456	1.1	1.5	2.2	1.0	ATP5D	Homo sapiens ATP synthase, H+ transporting, mitochondrial F1 complex, delta subunit (ATP5D), nuclear gene encoding mitochondrial protein, transcript variant 2, mRNA [NM_001001975]	513
A_32_P123771	1.2	1.2	1.1	-2.5	ATP5E	Homo sapiens ATP synthase, H+ transporting, mitochondrial F1 complex, epsilon subunit (ATP5E), nuclear gene encoding mitochondrial protein, mRNA [NM_006886]	514

A_24_P659099	1.1	1.1	1.0	3.0	ATP5F1	ATP synthase, H+ transporting, mitochondrial Fo complex, subunit B1 [Source:HGNC Symbol;Acc:840] [ENST00000369722]	515
A_24_P315326	-5.9	-1.2	1.1	1.1	ATP5G2	ATP synthase, H+ transporting, mitochondrial Fo complex, subunit C2 (subunit 9) [Source:HGNC Symbol;Acc:842] [ENST00000550241]	517

A_24_P174346	1.1	1.2	-2.1	1.1	ATP5G3	Homo sapiens ATP synthase, H+ transporting, mitochondrial Fo complex, subunit C3 (subunit 9) (ATP5G3), nuclear gene encoding mitochondrial protein, transcript variant 3, mRNA [NM_001002258]	518

A_23_P77818	2.5	-1.0	2.6	2.5	ATP5H	Homo sapiens ATP synthase, H+ transporting, mitochondrial Fo complex, subunit d (ATP5H), nuclear gene encoding mitochondrial protein, transcript variant 1, mRNA [NM_006356]	10476
A_24_P302998	3.0	2.6	2.4	1.8	ATP5I	Homo sapiens ATP synthase, H+ transporting, mitochondrial Fo complex, subunit E (ATP5I), nuclear gene encoding mitochondrial protein, transcript variant 1, mRNA [NM_007100]	521

A_23_P215832	-1.0	-1.1	-2.6	-1.0	ATP5J2	Homo sapiens ATP synthase, H+ transporting, mitochondrial Fo complex, subunit F2 (ATP5J2), nuclear gene encoding mitochondrial protein, transcript variant 1, mRNA [NM_004889]	9551
A_24_P118231	-2.0	-2.0	-2.0	-2.1	ATP5S	Homo sapiens ATP synthase, H+ transporting, mitochondrial Fo complex, subunit s (factor B) (ATP5S), nuclear gene encoding mitochondrial protein, transcript variant 1, mRNA [NM_001003803]	27109

A_23_P107795	3.3	3.1	2.8	2.9	ATP5SL	Homo sapiens ATP5S-like (ATP5SL), transcript variant 4, mRNA [NM_018035]	55101
A_24_P74753	-2.0	-4.9	-2.0	-2.0	ATP6AP1	Homo sapiens ATPase, H+ transporting, lysosomal accessory protein 1 (ATP6AP1), mRNA [NM_001183]	537
A_23_P54636	2.5	2.9	2.6	-1.2	ATP6V0D1	Homo sapiens ATPase, H+ transporting, lysosomal 38kDa, V0 subunit d1 (ATP6V0D1), mRNA [NM_004691]	9114

A_23_P146146	1.9	1.9	2.2	-1.0	ATP6V0D2	Homo sapiens ATPase, H+ transporting, lysosomal 38kDa, V0 subunit d2 (ATP6V0D2), mRNA [NM_152565]	245972
A_23_P142815	1.0	1.0	1.0	3.8	ATP6V1B1	Homo sapiens ATPase, H+ transporting, lysosomal 56/58kDa, V1 subunit B1 (ATP6V1B1), mRNA [NM_001692]	525
A_23_P146058	1.0	1.0	-3.5	-2.5	ATP6V1C1	Homo sapiens ATPase, H+ transporting, lysosomal 42kDa, V1 subunit C1 (ATP6V1C1), mRNA [NM_001695]	528

A_23_P250914	-1.1	1.1	1.6	2.6	ATP6V1C2	Homo sapiens ATPase, H+ transporting, lysosomal 42kDa, V1 subunit C2 (ATP6V1C2), transcript variant 1, mRNA [NM_001039362]	245973
A_23_P143047	1.0	-1.0	-1.1	2.1	ATP6V1E2	Homo sapiens ATPase, H+ transporting, lysosomal 31kDa, V1 subunit E2 (ATP6V1E2), mRNA [NM_080653]	90423
A_23_P500410	1.1	-1.2	-2.8	-2.6	ATP6V1G2	Homo sapiens ATPase, H+ transporting, lysosomal 13kDa, V1 subunit G2 (ATP6V1G2), transcript variant 1, mRNA [NM_130463]	534

A_23_P23797	-3.0	1.0	-1.4	1.0	ATP6V1G3	Homo sapiens ATPase, H+ transporting, lysosomal 13kDa, V1 subunit G3 (ATP6V1G3), transcript variant 2, mRNA [NM_133326]	127124
A_24_P363679	1.5	2.7	2.4	2.6	ATP6V1H	Homo sapiens ATPase, H+ transporting, lysosomal 50/57kDa, V1 subunit H (ATP6V1H), transcript variant 1, mRNA [NM_015941]	51606
A_23_P205228	1.0	1.0	2.4	-1.0	ATP7B	Homo sapiens ATPase, Cu++ transporting, beta polypeptide (ATP7B), transcript variant 1, mRNA [NM_000053]	540

A_23_P258612	-1.0	-1.1	-1.1	2.6	ATP8A2	Homo sapiens ATPase, aminophospholipid transporter, class I, type 8A, member 2 (ATP8A2), mRNA [NM_016529]	51761
A_32_P185229	-1.0	-1.0	3.3	1.3	ATP8B2	Homo sapiens ATPase, class I, type 8B, member 2 (ATP8B2), transcript variant 1, mRNA [NM_020452]	57198
A_24_P366749	-1.1	-1.1	-5.8	-2.6	ATP8B2	Homo sapiens ATPase, class I, type 8B, member 2 (ATP8B2), transcript variant 2, mRNA [NM_001005855]	57198

A_23_P79108	1.8	-1.9	1.8	2.5	ATP8B3	Homo sapiens ATPase, aminophospholipid transporter, class I, type 8B, member 3 (ATP8B3), transcript variant 1, mRNA [NM_138813]	148229
A_24_P358245	2.0	-3.5	-1.5	1.0	ATP8B5P	Homo sapiens ATPase, class I, type 8B, member 5, pseudogene (ATP8B5P), transcript variant 2, non-coding RNA [NR_003582]	158381
A_23_P380614	-4.3	-4.4	-10.3	-5.6	ATP9A	Homo sapiens ATPase, class II, type 9A (ATP9A), mRNA [NM_006045]	10079

A_23_P10497	-1.3	-2.2	-1.1	-1.1	ATP9B	Homo sapiens ATPase, class II, type 9B (ATP9B), mRNA [NM_198531]	374868
A_24_P229199	2.0	1.5	2.0	-1.0	ATPAF1	Homo sapiens ATP synthase mitochondrial F1 complex assembly factor 1 (ATPAF1), nuclear gene encoding mitochondrial protein, transcript variant 1, mRNA [NM_022745]	64756
A_32_P97862	-1.3	-1.3	-1.6	-6.9	ATPAF1-AS1	Homo sapiens ATPAF1 antisense RNA 1 (non-protein coding) (ATPAF1-AS1), mRNA [NM_001145474]	374973

A_24_P56557	1.0	1.0	2.2	-1.0	ATPBD4	Homo sapiens ATP binding domain 4 (ATPBD4), transcript variant 1, mRNA [NM_080650]	89978
A_23_P77135	-3.1	-2.5	-1.4	-1.4	ATPBD4	Homo sapiens ATP binding domain 4 (ATPBD4), transcript variant 1, mRNA [NM_080650]	89978
A_23_P501770	1.1	1.1	-2.2	1.0	ATRIP	Homo sapiens ATR interacting protein (ATRIP), transcript variant 2, mRNA [NM_032166]	84126
A_23_P388379	1.0	-1.0	-2.1	1.0	ATRNL1	Homo sapiens attractin-like 1 (ATRNL1), mRNA [NM_207303]	26033

A_23_P136874	-4.1	1.6	-1.5	-3.6	ATRX	Homo sapiens alpha thalassemia/mental retardation syndrome X-linked (ATRX), transcript variant 1, mRNA [NM_000489]	546
A_24_P128041	1.6	1.5	-4.0	1.5	ATRX	Homo sapiens alpha thalassemia/mental retardation syndrome X-linked (RAD54 homolog, S. cerevisiae) mRNA, complete cds. [BT007188]	546
A_24_P348660	3.4	3.2	2.2	-1.2	ATRX	Homo sapiens alpha thalassemia/mental retardation syndrome X-linked (ATRX), transcript variant 1, mRNA [NM_000489]	546

A_32_P179740	-13.4	1.1	1.4	1.1	ATXN7L1	Homo sapiens ataxin 7-like 1 (ATXN7L1), transcript variant 1, mRNA [NM_020725]	222255
A_24_P6790	1.8	4.1	1.0	6.6	ATXN7L1	Homo sapiens ataxin 7-like 1 (ATXN7L1), transcript variant 1, mRNA [NM_020725]	222255
A_23_P317207	3.8	4.4	1.0	3.9	ATXN7L2	Homo sapiens ataxin 7-like 2 (ATXN7L2), mRNA [NM_153340]	127002
A_24_P263956	-1.0	-1.0	-1.1	2.0	ATXN7L3B	Homo sapiens ataxin 7-like 3B (ATXN7L3B), mRNA [NM_001136262]	552889
A_23_P131866	2.9	-1.5	2.3	2.3	AURKA	Homo sapiens aurora kinase A (AURKA), transcript variant 1, mRNA [NM_198433]	6790

A_23_P130182	1.1	1.0	-80.8	1.5	AURKB	Homo sapiens aurora kinase B (AURKB), mRNA [NM_004217]	9212
A_24_P419087	-3.8	1.2	-1.6	-1.0	AVIL	Homo sapiens advillin (AVIL), mRNA [NM_006576]	10677
A_24_P55356	1.0	-2.7	-1.0	-1.0	AVL9	Homo sapiens mRNA for KIAA0241 gene, partial cds. [D87682]	23080
A_24_P272967	-2.9	-1.1	-1.2	-1.3	AVL9	Homo sapiens AVL9 homolog (S. cerevisiase) (AVL9), mRNA [NM_015060]	23080
A_23_P208389	-1.9	2.7	1.5	1.8	AXL	Homo sapiens AXL receptor tyrosine kinase (AXL), transcript variant 1, mRNA [NM_021913]	558

A_24_P66522	-2.2	-2.2	-2.3	-1.7	AZI1	Homo sapiens 5-azacytidine induced 1 (AZI1), transcript variant 1, mRNA [NM_014984]	22994
A_23_P319270	1.7	1.4	-1.1	3.3	AZI1	Homo sapiens 5-azacytidine induced 1 (AZI1), transcript variant 1, mRNA [NM_014984]	22994
A_24_P51805	-1.3	-1.6	-2.1	-1.7	AZI2	Homo sapiens 5-azacytidine induced 2 (AZI2), transcript variant 1, mRNA [NM_022461]	64343
A_23_P153741	-2.0	1.0	1.0	-1.5	AZU1	Homo sapiens azurocidin 1 (AZU1), mRNA [NM_001700]	566

A_24_P88870	-1.4	-2.2	-1.5	-2.3	B3GALNT1	Homo sapiens beta-1,3-N-acetylgalactosaminyltransferase 1 (globoside blood group) (B3GALNT1), transcript variant 5, mRNA [NM_001038628]	8706
A_23_P41166	1.1	1.8	-96.7	-1.0	B3GALNT1	Homo sapiens beta-1,3-N-acetylgalactosaminyltransferase 1 (globoside blood group) (B3GALNT1), transcript variant 5, mRNA [NM_001038628]	8706
A_23_P334751	-1.0	-1.0	-2.4	-8.0	B3GALNT2	Homo sapiens beta-1,3-N-acetylgalactosaminyltransferase 2 (B3GALNT2), mRNA [NM_152490]	148789

A_23_P493	2.2	-1.7	1.9	1.3	B3GALT2	Homo sapiens UDP-Gal:betaGlcNAc beta 1,3-galactosyltransferase, polypeptide 2 (B3GALT2), mRNA [NM_003783]	8707
A_23_P499	-3.5	1.0	1.5	-1.1	B3GALT2	Homo sapiens UDP-Gal:betaGlcNAc beta 1,3-galactosyltransferase, polypeptide 2 (B3GALT2), mRNA [NM_003783]	8707
A_23_P422071	-1.4	2.4	1.3	1.2	B3GALT4	Homo sapiens UDP-Gal:betaGlcNAc beta 1,3-galactosyltransferase, polypeptide 4 (B3GALT4), mRNA [NM_003782]	8705

A_24_P832426	-2.8	-2.3	1.0	1.1	B3GALTL	Homo sapiens beta 1,3-galactosyltransferase-like (B3GALTL), mRNA [NM_194318]	145173
A_23_P36140	1.4	2.1	1.3	3.4	B3GAT3	Homo sapiens beta-1,3-glucuronyltransferase 3 (glucuronosyltransferase I) (B3GAT3), mRNA [NM_012200]	26229
A_23_P18372	-2.8	1.0	-1.0	-1.1	B3GNT5	Homo sapiens UDP-GlcNAc:betaGal beta-1,3-N-acetylglucosaminyltransferase 5 (B3GNT5), mRNA [NM_032047]	84002

A_23_P366895	-1.4	-2.5	-2.0	-2.5	B3GNT6	Homo sapiens UDP-GlcNAc:betaGal beta-1,3-N-acetylglucosaminyltransferase 6 (core 3 synthase) (B3GNT6), mRNA [NM_138706]	192134
A_24_P20292	2.4	2.9	3.2	-1.3	B3GNT7	Homo sapiens UDP-GlcNAc:betaGal beta-1,3-N-acetylglucosaminyltransferase 7 (B3GNT7), mRNA [NM_145236]	93010
A_23_P101380	-1.3	1.0	-1.4	-2.1	B3GNT8	Homo sapiens UDP-GlcNAc:betaGal beta-1,3-N-acetylglucosaminyltransferase 8 (B3GNT8), mRNA [NM_198540]	374907

A_24_P279172	1.3	6.5	5.4	2.6	B3GNT9	Homo sapiens UDP-GlcNAc:betaGal beta-1,3-N-acetylglucosaminyltransferase 9 (B3GNT9), mRNA [NM_033309]	84752
A_23_P66050	-2.0	-1.9	-1.2	-2.0	B3GNT9	Homo sapiens UDP-GlcNAc:betaGal beta-1,3-N-acetylglucosaminyltransferase 9 (B3GNT9), mRNA [NM_033309]	84752
A_23_P379736	-1.3	1.5	-2.2	1.1	B3GNT9	Homo sapiens UDP-GlcNAc:betaGal beta-1,3-N-acetylglucosaminyltransferase 9 (B3GNT9), mRNA [NM_033309]	84752

A_24_P181672	2.2	-1.1	1.4	1.2	B3GNTL1	Homo sapiens UDP-GlcNAc:betaGal beta-1,3-N-acetylglucosaminyltransferase-like 1 (B3GNTL1), mRNA [NM_001009905]	146712
A_23_P87461	1.7	3.1	1.4	2.6	B4GALNT1	Homo sapiens beta-1,4-N-acetyl-galactosaminyl transferase 1 (B4GALNT1), mRNA [NM_001478]	2583
A_24_P359291	3.1	5.4	-1.2	2.7	B4GALNT1	Homo sapiens beta-1,4-N-acetyl-galactosaminyl transferase 1 (B4GALNT1), mRNA [NM_001478]	2583

A_32_P49748	1.1	-6.7	1.6	1.1	B4GALNT3	beta-1,4-N-acetyl-galactosaminyl transferase 3 [Source:HGNC Symbol;Acc:24137] [ENST00000535402]	283358
A_23_P135271	1.5	2.5	7.1	1.3	B4GALT1	Homo sapiens UDP-Gal:betaGlcNAc beta 1,4-galactosyltransferase, polypeptide 1 (B4GALT1), mRNA [NM_001497]	2683
A_24_P242440	1.6	2.7	-3.1	1.0	B4GALT2	Homo sapiens UDP-Gal:betaGlcNAc beta 1,4-galactosyltransferase, polypeptide 2 (B4GALT2), transcript variant 2, mRNA [NM_003780]	8704

A_23_P103919	2.8	1.7	2.1	2.5	B4GALT3	Homo sapiens UDP-Gal:betaGlcNAc beta 1,4-galactosyltransferase, polypeptide 3 (B4GALT3), transcript variant 2, mRNA [NM_003779]	8703
A_32_P103945	-3.8	4.0	2.6	-1.6	B4GALT4	Homo sapiens UDP-Gal:betaGlcNAc beta 1,4-galactosyltransferase, polypeptide 4 (B4GALT4), transcript variant 1, mRNA [NM_212543]	8702

A_23_P118946	1.6	1.6	-2.1	1.3	B4GALT6	Homo sapiens UDP-Gal:betaGlcNAc beta 1,4-galactosyltransferase, polypeptide 6 (B4GALT6), mRNA [NM_004775]	9331
A_23_P41872	-5.0	2.0	1.9	-1.0	B4GALT7	Homo sapiens xylosylprotein beta 1,4-galactosyltransferase, polypeptide 7 (galactosyltransferase I) (B4GALT7), mRNA [NM_007255]	11285
A_24_P176714	2.1	2.1	3.0	1.5	B9D1	Homo sapiens B9 protein domain 1 (B9D1), transcript variant 2, mRNA [NM_015681]	27077

A_23_P154875	-1.2	-3.8	1.3	-1.4	BACE2	Homo sapiens beta-site APP-cleaving enzyme 2 (BACE2), transcript variant a, mRNA [NM_012105]	25825
A_24_P14584	1.5	1.0	2.2	1.0	BACE2	Homo sapiens beta-site APP-cleaving enzyme 2 (BACE2), transcript variant a, mRNA [NM_012105]	25825
A_23_P150207	-1.1	1.5	1.4	-2.1	BAD	Homo sapiens BCL2-associated agonist of cell death (BAD), transcript variant 1, mRNA [NM_004322]	572
A_23_P356554	1.5	1.0	-7.1	1.0	BAG2	Homo sapiens BCL2-associated athanogene 2 (BAG2), mRNA [NM_004282]	9532

A_23_P52552	-2.0	-3.0	-1.1	-1.0	BAG3	Homo sapiens BCL2-associated athanogene 3 (BAG3), mRNA [NM_004281]	9531
A_23_P146217	2.3	2.3	2.3	2.3	BAG4	Homo sapiens BCL2-associated athanogene 4 (BAG4), transcript variant 1, mRNA [NM_004874]	9530
A_24_P608411	1.8	-1.1	-1.4	3.1	BAG6	BCL2-associated athanogene 6 [Source:HGNC Symbol;Acc:13919] [ENST00000383448]	7917

A_32_P103771	1.9	2.0	-7.6	1.6	BAGE	Homo sapiens B melanoma antigen variant d (BAGE1) mRNA, complete cds, alternatively spliced. [AF527552]	574
A_24_P126262	1.1	1.0	-2.2	-1.0	BAGE4	Homo sapiens B melanoma antigen family, member 4 (BAGE4), mRNA [NM_181704]	85317
A_23_P125056	1.6	6.7	-1.3	1.7	BAI1	Homo sapiens brain-specific angiogenesis inhibitor 1 (BAI1), mRNA [NM_001702]	575
A_23_P149019	2.3	2.7	2.1	1.2	BAI2	Homo sapiens brain-specific angiogenesis inhibitor 2 (BAI2), mRNA [NM_001703]	576

A_23_P61810	-1.0	-9.5	-1.0	-1.0	BAIAP2	Homo sapiens BAI1-associated protein 2 (BAIAP2), transcript variant 1, mRNA [NM_017450]	10458
A_24_P159648	2.7	2.6	-1.4	1.9	BAIAP2	Homo sapiens BAI1-associated protein 2 (BAIAP2), transcript variant 3, mRNA [NM_006340]	10458
A_23_P168470	-1.0	-1.0	-1.0	-3.2	BAIAP2L1	Homo sapiens BAI1-associated protein 2-like 1 (BAIAP2L1), mRNA [NM_018842]	55971
A_23_P47208	-1.1	-2.2	2.1	-2.7	BANF1	Homo sapiens barrier to autointegration factor 1 (BANF1), transcript variant 1, mRNA [NM_003860]	8815

A_23_P251767	3.1	1.9	2.2	-5.5	BANP	Homo sapiens BTG3 associated nuclear protein (BANP), transcript variant 1, mRNA [NM_017869]	54971
A_23_P251765	6.5	2.0	4.8	2.7	BANP	Homo sapiens BTG3 associated nuclear protein (BANP), transcript variant 2, mRNA [NM_079837]	54971
A_23_P259012	-1.0	-1.3	-1.1	-2.4	BAP1	Homo sapiens BRCA1 associated protein-1 (ubiquitin carboxy-terminal hydrolase) (BAP1), mRNA [NM_004656]	8314

A_24_P302898	-8.7	1.0	2.2	-2.1	BAP1	Homo sapiens BRCA1 associated protein-1 (ubiquitin carboxy-terminal hydrolase) (BAP1), mRNA [NM_004656]	8314
A_23_P67771	1.6	2.5	1.6	-1.2	BARD1	Homo sapiens BRCA1 associated RING domain 1 (BARD1), mRNA [NM_000465]	580
A_23_P126349	1.5	-5.0	-1.2	-1.6	BARHL2	Homo sapiens BarH-like homeobox 2 (BARHL2), mRNA [NM_020063]	343472
A_23_P90189	-1.6	-1.5	-2.8	-1.6	BBC3	Homo sapiens BCL2 binding component 3 (BBC3), transcript variant 4, mRNA [NM_014417]	27113

A_24_P305312	4.6	1.5	1.9	1.9	BBC3	Homo sapiens BCL2 binding component 3 (BBC3), transcript variant 4, mRNA [NM_014417]	27113
A_24_P852082	1.1	-1.7	-2.2	1.1	BBIP1	Homo sapiens BBSome interacting protein 1 (BBIP1), transcript variant 1, mRNA [NM_001195304]	92482
A_23_P150267	2.0	2.3	1.8	2.4	BBS1	Homo sapiens Bardet-Biedl syndrome 1 (BBS1), mRNA [NM_024649]	582
A_24_P184305	1.2	2.4	2.3	2.5	BBS1	Homo sapiens Bardet-Biedl syndrome 1 (BBS1), mRNA [NM_024649]	582

A_23_P5785	1.3	-1.0	2.0	-1.0	BBS5	Homo sapiens Bardet-Biedl syndrome 5 (BBS5), mRNA [NM_152384]	129880
A_32_P22191	1.4	-1.1	-8.8	1.8	BBX	Homo sapiens bobby sox homolog (Drosophila) (BBX), transcript variant 2, mRNA [NM_020235]	56987
A_23_P160607	6.1	2.3	1.3	1.8	BCAN	Homo sapiens brevican (BCAN), transcript variant 1, mRNA [NM_021948]	63827
A_23_P412526	-1.9	-3.1	-1.8	-1.3	BCAP29	Homo sapiens B-cell receptor-associated protein BAP29 mRNA, complete cds. [AF126020]	55973

A_24_P134683	1.2	1.3	2.6	1.1	BCAP31	Homo sapiens B-cell receptor-associated protein 31 (BCAP31), transcript variant 2, mRNA [NM_005745]	10134
A_32_P4364	1.2	1.8	2.5	1.0	BCAP31	Homo sapiens B-cell receptor-associated protein 31 (BCAP31), transcript variant 2, mRNA [NM_005745]	10134
A_23_P66319	-1.4	1.1	1.1	-2.4	BCAR1	Homo sapiens breast cancer anti-estrogen resistance 1 (BCAR1), transcript variant 6, mRNA [NM_014567]	9564

A_32_P106933	-1.3	-1.3	-1.3	-2.4	BCAR4	Homo sapiens breast cancer anti-estrogen resistance 4 (non-protein coding) (BCAR4), non-coding RNA [NR_024049]	400500
A_23_P153070	-1.9	-1.9	1.2	-2.1	BCAS3	Homo sapiens breast carcinoma amplified sequence 3 (BCAS3), transcript variant 2, mRNA [NM_017679]	54828
A_24_P100761	1.3	-1.0	1.0	3.1	BCAS3	Homo sapiens breast carcinoma amplified sequence 3 (BCAS3), transcript variant 2, mRNA [NM_017679]	54828

A_32_P23659	1.5	-6.6	-1.4	-1.5	BCAS4	breast carcinoma amplified sequence 4 [Source:HGNC Symbol;Acc:14367] [ENST00000463943]	55653
A_24_P52921	-1.9	1.1	-6.3	-1.5	BCAT1	Homo sapiens branched chain amino-acid transaminase 1, cytosolic (BCAT1), transcript variant 1, mRNA [NM_005504]	586
A_24_P30206	1.1	1.1	-3.0	1.1	BCCIP	Homo sapiens BRCA2 and CDKN1A interacting protein (BCCIP), transcript variant B, mRNA [NM_078468]	56647

A_23_P67188	1.4	-4.6	1.0	1.7	BCKDHA	Homo sapiens branched chain keto acid dehydrogenase E1, alpha polypeptide (BCKDHA), nuclear gene encoding mitochondrial protein, transcript variant 1, mRNA [NM_000709]	593
A_23_P3823	-1.1	-7.1	-1.2	-1.1	BCKDK	Homo sapiens branched chain ketoacid dehydrogenase kinase (BCKDK), nuclear gene encoding mitochondrial protein, transcript variant 2, mRNA [NM_001122957]	10295

A_23_P218584	-1.1	-1.1	-1.2	2.3	BCL11A	Homo sapiens B-cell CLL/lymphoma 11A (zinc finger protein) (BCL11A), transcript variant 1, mRNA [NM_022893]	53335
A_23_P205738	1.0	-1.2	1.0	-2.4	BCL11B	Homo sapiens B-cell CLL/lymphoma 11B (zinc finger protein) (BCL11B), transcript variant 1, mRNA [NM_138576]	64919
A_23_P425954	1.1	-2.7	-1.1	1.0	BCL2L11	Homo sapiens BCL2-like 11 (apoptosis facilitator) (BCL2L11), transcript variant 1, mRNA [NM_138621]	10018

A_23_P50477	1.9	1.6	2.0	1.3	BCL2L12	Homo sapiens BCL2-like 12 (proline rich) (BCL2L12), transcript variant 1, mRNA [NM_138639]	83596
A_23_P418373	1.4	2.7	2.5	1.1	BCL2L2	Homo sapiens BCL2-like 2 (BCL2L2), transcript variant 1, mRNA [NM_004050]	599
A_23_P61826	2.5	2.2	2.0	1.8	BCL7A	Homo sapiens B-cell CLL/lymphoma 7A (BCL7A), transcript variant 1, mRNA [NM_020993]	605
A_24_P203056	2.5	1.3	1.4	2.0	BCL7A	Homo sapiens B-cell CLL/lymphoma 7A (BCL7A), transcript variant 1, mRNA [NM_020993]	605

A_23_P123010	-1.2	-1.7	-2.1	-1.2	BCL7B	Homo sapiens B-cell CLL/lymphoma 7B (BCL7B), transcript variant 1, mRNA [NM_001707]	9275
A_23_P118289	1.7	2.1	1.4	-2.4	BCL7C	Homo sapiens B-cell CLL/lymphoma 7C (BCL7C), mRNA [NM_004765]	9274
A_23_P382602	1.1	-2.1	1.8	2.0	BCL9	Homo sapiens B-cell CLL/lymphoma 9 (BCL9), mRNA [NM_004326]	607
A_24_P134816	1.0	-1.0	-2.2	1.6	BCL9L	Homo sapiens B-cell CLL/lymphoma 9-like (BCL9L), mRNA [NM_182557]	283149

A_23_P116187	-1.2	-1.2	-2.4	1.2	BCO2	Homo sapiens beta-carotene oxygenase 2 (BCO2), transcript variant 1, mRNA [NM_031938]	83875
A_23_P159741	2.2	1.6	1.4	1.1	BCOR	Homo sapiens BCL6 corepressor (BCOR), transcript variant 1, mRNA [NM_017745]	54880
A_24_P15270	1.7	1.5	2.2	-1.5	BCR	Homo sapiens breakpoint cluster region (BCR), transcript variant 1, mRNA [NM_004327]	613
A_23_P503115	-3.7	-1.1	1.4	1.4	BCR	Homo sapiens breakpoint cluster region (BCR), transcript variant 1, mRNA [NM_004327]	613

A_32_P232865	1.2	-1.1	-11.4	1.3	BCR	Homo sapiens breakpoint cluster region (BCR), transcript variant 1, mRNA [NM_004327]	613
A_24_P23951	-1.5	-19.5	-2.4	-1.2	BCRP2	Homo sapiens breakpoint cluster region pseudogene 2 (BCRP2), non-coding RNA [NR_037566]	400892
A_23_P154086	1.0	1.0	-2.0	1.0	BCS1L	Homo sapiens BCS1-like (S. cerevisiae) (BCS1L), transcript variant 1, mRNA [NM_004328]	617
A_24_P80500	17.3	10.3	7.6	1.9	BDH2	Homo sapiens 3-hydroxybutyrate dehydrogenase, type 2 (BDH2), mRNA [NM_020139]	56898

A_24_P919850	2.3	1.0	-2.0	-1.0	BDKRB1	Homo sapiens bradykinin receptor B1 (BDKRB1), mRNA [NM_000710]	623
A_32_P7316	-1.0	-1.1	-3.5	-1.1	BDNF	Homo sapiens brain-derived neurotrophic factor (BDNF), transcript variant 1, mRNA [NM_170735]	627
A_24_P247910	-1.2	3.9	3.5	2.7	BDP1	Homo sapiens mRNA for KIAA1241 protein, partial cds. [AB033067]	55814
A_24_P42316	1.5	1.0	-3.5	-1.0	BDP1	Homo sapiens B double prime 1, subunit of RNA polymerase III transcription initiation factor IIIB (BDP1), mRNA [NM_018429]	55814

A_24_P313597	-2.6	1.1	1.1	1.5	BECN1	Homo sapiens beclin 1, autophagy related (BECN1), mRNA [NM_003766]	8678
A_23_P433071	-5.5	-1.0	-1.0	1.0	BECN1	Homo sapiens beclin 1, autophagy related (BECN1), mRNA [NM_003766]	8678
A_23_P904	-1.0	-1.0	-1.1	-8.4	BEND5	Homo sapiens BEN domain containing 5 (BEND5), mRNA [NM_024603]	79656
A_23_P312358	1.0	-2.6	1.0	1.1	BEND7	Homo sapiens BEN domain containing 7 (BEND7), transcript variant 1, mRNA [NM_152751]	222389

A_23_P104996	1.5	-1.2	-1.1	11.2	BEST1	Homo sapiens bestrophin 1 (BEST1), transcript variant 1, mRNA [NM_004183]	7439
A_32_P98966	2.6	1.3	-1.2	1.1	BEST1	bestrophin 1 [Source:HGNC Symbol;Acc:12703] [ENST00000449131]	7439
A_23_P323801	1.0	1.0	-2.9	1.1	BEST3	Homo sapiens bestrophin 3 (BEST3), transcript variant 1, mRNA [NM_032735]	144453
A_23_P97242	1.7	4.6	-1.3	-1.2	BEST4	Homo sapiens bestrophin 4 (BEST4), mRNA [NM_153274]	266675

A_23_P59700	-2.1	-1.3	-1.4	-1.5	BET1	Homo sapiens blocked early in transport 1 homolog (S. cerevisiae) (BET1), mRNA [NM_005868]	10282
A_24_P269432	-1.6	-3.3	-3.6	-3.5	BET1	Homo sapiens blocked early in transport 1 homolog (S. cerevisiae) (BET1), mRNA [NM_005868]	10282
A_23_P127879	1.5	2.8	1.4	-1.9	BET1L	Homo sapiens blocked early in transport 1 homolog (S. cerevisiae)-like (BET1L), transcript variant 2, mRNA [NM_016526]	51272

A_24_P40551	2.4	1.0	1.1	1.0	BEX4	Homo sapiens brain expressed, X-linked 4 (BEX4), transcript variant 2, mRNA [NM_001080425]	56271
A_24_P184931	-3.4	-1.4	-1.5	-1.5	BFAR	Homo sapiens bifunctional apoptosis regulator (BFAR), mRNA [NM_016561]	51283
A_24_P336551	3.2	1.3	1.9	1.4	BGLAP	Homo sapiens bone gamma-carboxyglutamate (gla) protein (BGLAP), mRNA [NM_199173]	632
A_24_P57898	-1.0	1.4	-2.6	1.2	BHLHE23	Homo sapiens basic helix-loop-helix family, member e23 (BHLHE23), mRNA [NM_080606]	128408

A_24_P268676	-2.5	-1.0	1.1	-1.0	BHLHE40	Homo sapiens basic helix-loop-helix family, member e40 (BHLHE40), mRNA [NM_003670]	8553
A_24_P257511	1.7	-2.4	-2.2	1.6	BHMT	Homo sapiens betaine--homocysteine S-methyltransferase (BHMT), mRNA [NM_001713]	635
A_24_P231829	1.0	-9.8	-1.0	-2.8	BHMT2	Homo sapiens betaine--homocysteine S-methyltransferase 2 (BHMT2), transcript variant 1, mRNA [NM_017614]	23743
A_23_P154929	1.1	1.2	3.1	1.6	BID	Homo sapiens BH3 interacting domain death agonist (BID), transcript variant 1, mRNA [NM_197966]	637

A_24_P187948	2.2	2.2	1.0	1.0	BID	Homo sapiens BH3 interacting domain death agonist (BID), transcript variant 1, mRNA [NM_197966]	637
A_24_P156993	3.4	-21.4	3.1	2.7	BIN1	Homo sapiens bridging integrator 1 (BIN1), transcript variant 4, mRNA [NM_139346]	274
A_24_P115443	-2.2	-1.0	-1.3	1.2	BIN3	Homo sapiens bridging integrator 3 (BIN3), mRNA [NM_018688]	55909
A_23_P43086	1.3	1.3	1.3	-7.9	BIN3	Homo sapiens bridging integrator 3 (BIN3), mRNA [NM_018688]	55909

A_24_P115774	1.1	-7.2	1.1	1.0	BIRC2	Homo sapiens baculoviral IAP repeat containing 2 (BIRC2), mRNA [NM_001166]	329
A_23_P98350	1.0	-1.0	2.6	1.0	BIRC3	Homo sapiens baculoviral IAP repeat containing 3 (BIRC3), transcript variant 1, mRNA [NM_001165]	330
A_23_P142616	1.0	-1.6	-2.1	-3.9	BIRC6	Homo sapiens baculoviral IAP repeat containing 6 (BIRC6), mRNA [NM_016252]	57448
A_23_P79769	-1.1	2.2	1.2	-2.0	BIRC7	Homo sapiens baculoviral IAP repeat containing 7 (BIRC7), transcript variant 2, mRNA [NM_022161]	79444

A_23_P105833	6.9	6.4	6.2	6.5	BIVM	Homo sapiens basic, immunoglobulin-like variable motif containing (BIVM), transcript variant 1, mRNA [NM_017693]	54841
A_24_P50368	-1.0	-1.1	-3.0	-1.1	BLID	Homo sapiens BH3-like motif containing, cell death inducer (BLID), nuclear gene encoding mitochondrial protein, mRNA [NM_001001786]	414899
A_23_P88630	1.3	1.3	2.4	1.3	BLM	Homo sapiens Bloom syndrome, RecQ helicase-like (BLM), mRNA [NM_000057]	641

A_24_P60930	1.2	-1.0	2.7	-1.3	BMP1	Homo sapiens bone morphogenetic protein 1 (BMP1), transcript variant 3, mRNA [NM_006129]	649
A_23_P33277	1.5	3.1	1.5	1.1	BMP1	bone morphogenetic protein 1 [Source:HGNC Symbol;Acc:1067] [ENST00000397816]	649
A_23_P81058	-5.7	1.1	1.0	1.6	BMP3	Homo sapiens bone morphogenetic protein 3 (BMP3), mRNA [NM_001201]	651
A_23_P54144	2.9	2.2	2.9	2.8	BMP4	Homo sapiens bone morphogenetic protein 4 (BMP4), transcript variant 1, mRNA [NM_001202]	652

A_23_P68487	1.1	-15.3	1.0	1.0	BMP7	Homo sapiens bone morphogenetic protein 7 (BMP7), mRNA [NM_001719]	655
A_23_P380939	-1.0	-4.8	-1.1	-1.0	BMP8B	Homo sapiens bone morphogenetic protein 8b (BMP8B), mRNA [NM_001720]	656
A_24_P527404	-12.2	-1.0	-1.1	1.0	BMPR1A	Homo sapiens bone morphogenetic protein receptor, type IA (BMPR1A), mRNA [NM_004329]	657
A_24_P418717	2.3	-1.1	2.1	-1.0	BMPR1A	Homo sapiens bone morphogenetic protein receptor, type IA (BMPR1A), mRNA [NM_004329]	657

A_24_P753161	7.7	12.8	12.5	13.7	BMPR2	Homo sapiens bone morphogenetic protein receptor, type II (serine/threonine kinase) (BMPR2), mRNA [NM_001204]	659
A_24_P649747	-2.5	2.5	-1.5	-1.3	BMS1	Homo sapiens BMS1 homolog, ribosome assembly protein (yeast) (BMS1), mRNA [NM_014753]	9790
A_23_P24068	2.0	2.0	1.4	1.8	BMS1	Homo sapiens BMS1 homolog, ribosome assembly protein (yeast) (BMS1), mRNA [NM_014753]	9790

A_24_P263672	1.4	2.1	2.1	1.0	BMS1	Homo sapiens BMS1 homolog, ribosome assembly protein (yeast) (BMS1), mRNA [NM_014753]	9790
A_24_P615822	1.1	-1.6	-3.7	-2.4	BMS1	Homo sapiens BMS1 homolog, ribosome assembly protein (yeast) (BMS1), mRNA [NM_014753]	9790
A_24_P263144	20.4	19.6	18.1	18.7	BMX	Homo sapiens BMX non-receptor tyrosine kinase (BMX), transcript variant 2, mRNA [NM_001721]	660
A_23_P43690	1.1	1.0	-2.9	1.6	BNC2	Homo sapiens basonuclin 2 (BNC2), mRNA [NM_017637]	54796

A_23_P138635	-1.1	-1.1	-1.1	-4.9	BNIP3	Homo sapiens BCL2/adenovirus E1B 19kDa interacting protein 3 (BNIP3), nuclear gene encoding mitochondrial protein, mRNA [NM_004052]	664
A_23_P257763	-1.8	-1.1	-2.8	-1.5	BOC	Homo sapiens Boc homolog (mouse) (BOC), mRNA [NM_033254]	91653
A_23_P345212	1.4	-3.1	1.0	-1.0	BOD1P	Homo sapiens biorientation of chromosomes in cell division 1 pseudogene (BOD1P), non-coding RNA [NR_033372]	284257

A_23_P253029	-2.1	-1.3	-6.6	-2.0	BOK	Homo sapiens BCL2-related ovarian killer (BOK), mRNA [NM_032515]	666
A_23_P46507	3.4	1.6	-1.6	-1.1	BOLA1	Homo sapiens bolA homolog 1 (E. coli) (BOLA1), mRNA [NM_016074]	51027
A_24_P77364	1.1	-2.1	-2.9	-3.3	BOLA3	Homo sapiens bolA homolog 3 (E. coli) (BOLA3), transcript variant 1, mRNA [NM_212552]	388962
A_23_P131706	1.8	1.8	1.8	2.2	BOLA3	Homo sapiens bolA homolog 3 (E. coli) (BOLA3), transcript variant 1, mRNA [NM_212552]	388962

A_23_P43800	5.5	10.4	3.6	18.6	BOP1	Homo sapiens block of proliferation 1 (BOP1), mRNA [NM_015201]	23246
A_23_P25626	-5.9	-1.1	1.3	-1.0	BORA	Homo sapiens bora, aurora kinase A activator (BORA), mRNA [NM_024808]	79866
A_23_P154784	-6.1	-2.4	-1.9	-1.1	BPIFB1	Homo sapiens BPI fold containing family B, member 1 (BPIFB1), mRNA [NM_033197]	92747
A_32_P419502	3.2	1.3	3.1	3.7	BPIFC	Homo sapiens BPI fold containing family C (BPIFC), mRNA [NM_174932]	254240

A_24_P104538	1.3	-2.9	1.5	1.4	BPTF	bromodomain PHD finger transcription factor [Source:HGNC Symbol;Acc:3581] [ENST00000342579]	2186
A_23_P428548	4.9	3.0	3.8	1.6	BPTF	Homo sapiens bromodomain PHD finger transcription factor (BPTF), transcript variant 1, mRNA [NM_182641]	2186
A_23_P45598	-1.0	-5.8	-1.1	-1.0	BPY2B	Homo sapiens basic charge, Y-linked, 2B (BPY2B), mRNA [NM_001002760]	442867
A_23_P251898	-2.7	-3.8	-3.3	-1.4	BRAT1	Homo sapiens BRCA1-associated ATM activator 1 (BRAT1), mRNA [NM_152743]	221927

A_23_P166536	1.9	3.7	1.5	1.9	BRD1	Homo sapiens bromodomain containing 1 (BRD1), mRNA [NM_014577]	23774
A_24_P307014	3.0	1.6	-1.1	-1.0	BRD2	Homo sapiens bromodomain containing 2 (BRD2), transcript variant 1, mRNA [NM_005104]	6046
A_23_P256898	1.0	-1.0	1.1	-11.0	BRD2	Homo sapiens bromodomain containing 2 (BRD2), transcript variant 1, mRNA [NM_005104]	6046
A_24_P32887	-3.4	1.0	-2.6	-1.1	BRD3	Homo sapiens bromodomain containing 3 (BRD3), mRNA [NM_007371]	8019

A_23_P42020	-16.3	1.0	-1.0	1.0	BRD7P3	Homo sapiens bromodomain containing 7 pseudogene 3 (BRD7P3), non-coding RNA [NR_002730]	23629
A_23_P145016	-2.7	-3.5	-1.0	1.4	BRD8	Homo sapiens bromodomain containing 8 (BRD8), transcript variant 1, mRNA [NM_006696]	10902
A_32_P132796	-3.7	2.7	-1.4	1.2	BRE	Homo sapiens brain and reproductive organ-expressed (TNFRSF1A modulator) (BRE), transcript variant 1, mRNA [NM_004899]	9577

A_24_P102411	3.3	1.0	1.0	1.0	BRF1	Homo sapiens BRF1 homolog, subunit of RNA polymerase III transcription initiation factor IIIB (S. cerevisiae) (BRF1), transcript variant 8, mRNA [NM_001242790]	2972
A_23_P117416	1.9	1.8	2.4	1.2	BRF1	Homo sapiens BRF1 homolog, subunit of RNA polymerase III transcription initiation factor IIIB (S. cerevisiae) (BRF1), transcript variant 3, mRNA [NM_145685]	2972

A_23_P15844	-1.5	-1.5	-9.1	-2.9	BRIP1	Homo sapiens BRCA1 interacting protein C-terminal helicase 1 (BRIP1), mRNA [NM_032043]	83990
A_23_P252857	4.0	3.4	3.5	3.3	BRIX1	Homo sapiens BRX1, biogenesis of ribosomes, homolog (S. cerevisiae) (BRIX1), mRNA [NM_018321]	55299
A_23_P420361	1.0	1.2	-1.5	2.2	BRK1	Homo sapiens BRICK1, SCAR/WAVE actin-nucleating complex subunit (BRK1), mRNA [NM_018462]	55845

A_23_P75500	-1.0	-20.7	-1.1	-1.1	BRMS1	Homo sapiens breast cancer metastasis suppressor 1 (BRMS1), transcript variant 2, mRNA [NM_001024957]	25855
A_23_P205419	-1.7	-2.0	-1.5	-1.2	BRMS1L	Homo sapiens breast cancer metastasis-suppressor 1-like (BRMS1L), mRNA [NM_032352]	84312
A_23_P359636	-3.7	1.2	1.1	-1.0	BROX	Homo sapiens BRO1 domain and CAAX motif containing (BROX), mRNA [NM_144695]	148362
A_32_P179854	3.2	3.2	-8.2	2.1	BRP44	Homo sapiens brain protein 44 (BRP44), transcript variant 1, mRNA [NM_001143674]	25874

A_23_P110961	2.7	1.0	1.0	1.0	BRP44L	Homo sapiens brain protein 44-like (BRP44L), mRNA [NM_016098]	51660
A_23_P250948	2.6	3.4	3.2	2.4	BRPF1	Homo sapiens bromodomain and PHD finger containing, 1 (BRPF1), transcript variant 1, mRNA [NM_001003694]	7862
A_24_P326708	1.4	1.5	2.0	1.2	BRSK2	Homo sapiens BR serine/threonine kinase 2 (BRSK2), mRNA [NM_003957]	9024
A_24_P861009	-2.1	1.0	-1.0	-1.0	BRWD1	Homo sapiens bromodomain and WD repeat domain containing 1 (BRWD1), transcript variant 3, mRNA [NM_001007246]	54014

A_23_P379159	-1.1	1.9	-2.7	-2.3	BRWD3	Homo sapiens bromodomain and WD repeat domain containing 3 (BRWD3), mRNA [NM_153252]	254065
A_32_P148380	1.1	-1.1	-2.1	-1.1	BRWD3	Homo sapiens bromodomain and WD repeat domain containing 3 (BRWD3), mRNA [NM_153252]	254065
A_32_P489130	1.3	-1.3	-2.0	-1.3	BRWD3	Homo sapiens bromodomain and WD repeat domain containing 3 (BRWD3), mRNA [NM_153252]	254065

A_24_P127602	-2.0	-1.5	-4.2	1.3	BSN-AS2	Homo sapiens BSN antisense RNA 2 (non-protein coding) (BSN-AS2), non-coding RNA [NR_038866]	100132677
A_24_P180849	-3.6	1.3	-2.6	-1.2	BSPRY	Homo sapiens B-box and SPRY domain containing (BSPRY), mRNA [NM_017688]	54836
A_23_P39465	1.2	-2.0	1.4	1.1	BST2	Homo sapiens bone marrow stromal cell antigen 2 (BST2), mRNA [NM_004335]	684
A_23_P419714	-1.6	-1.6	-1.8	-2.2	BTBD11	Homo sapiens BTB (POZ) domain containing 11 (BTBD11), transcript variant a, mRNA [NM_001018072]	121551

A_23_P383915	3.4	2.7	1.4	1.9	BTBD16	Homo sapiens BTB (POZ) domain containing 16 (BTBD16), mRNA [NM_144587]	118663
A_23_P153698	1.6	1.6	-2.3	-1.4	BTBD2	Homo sapiens BTB (POZ) domain containing 2 (BTBD2), mRNA [NM_017797]	55643
A_23_P99614	1.4	1.5	1.9	2.0	BTBD6	Homo sapiens BTB (POZ) domain containing 6 (BTBD6), mRNA [NM_033271]	90135
A_23_P88184	11.7	11.1	10.7	10.7	BTBD7	Homo sapiens BTB (POZ) domain containing 7 (BTBD7), transcript variant 2, mRNA [NM_018167]	55727

A_24_P564030	1.1	-2.6	-1.0	-1.0	BTBD8	Homo sapiens BTB (POZ) domain containing 8 (BTBD8), mRNA [NM_183242]	284697
A_32_P94722	-2.7	1.1	1.0	-1.3	BTBD9	Homo sapiens BTB (POZ) domain containing 9 (BTBD9), transcript variant 1, mRNA [NM_052893]	114781
A_32_P217401	-1.0	-1.0	-1.0	-7.3	BTBD9	Homo sapiens BTB (POZ) domain containing 9 (BTBD9), transcript variant 1, mRNA [NM_052893]	114781
A_32_P108505	-2.1	4.2	1.1	-7.4	BTF3	Homo sapiens basic transcription factor 3 (BTF3), transcript variant 1, mRNA [NM_001037637]	689

A_32_P33723	1.1	-3.5	-1.6	-1.4	BTG3	Homo sapiens BTG family, member 3, mRNA (cDNA clone IMAGE:5240818), with apparent retained intron. [BC028229]	10950
A_23_P80068	-2.7	1.2	1.1	1.0	BTG3	Homo sapiens BTG family, member 3 (BTG3), transcript variant 2, mRNA [NM_006806]	10950
A_24_P136470	-2.1	-1.5	-1.3	-3.3	BTN2A3P	Homo sapiens butyrophilin, subfamily 2, member A3, pseudogene (BTN2A3P), non-coding RNA [NR_027795]	54718

A_24_P126282	-1.0	-1.3	-3.4	-1.0	BTN3A1	Homo sapiens butyrophilin, subfamily 3, member A1 (BTN3A1), transcript variant 1, mRNA [NM_007048]	11119
A_23_P214499	-1.0	-29.7	-1.0	1.1	BTN3A1	Homo sapiens butyrophilin, subfamily 3, member A1 (BTN3A1), transcript variant 1, mRNA [NM_007048]	11119
A_23_P376686	-2.1	1.4	2.5	-1.1	BTNL2	Homo sapiens butyrophilin-like 2 (MHC class II associated) (BTNL2), mRNA [NM_019602]	56244

A_23_P35427	2.2	-1.5	-1.2	1.4	BTRC	Homo sapiens beta-transducin repeat containing (BTRC), transcript variant 1, mRNA [NM_033637]	8945
A_23_P163481	3.0	-4.4	2.4	1.2	BUB1B	Homo sapiens budding uninhibited by benzimidazoles 1 homolog beta (yeast) (BUB1B), mRNA [NM_001211]	701
A_32_P181638	-2.2	-2.2	1.2	2.9	BVES	Homo sapiens blood vessel epicardial substance (BVES), transcript variant B, mRNA [NM_147147]	11149

A_23_P502783	-1.0	-2.4	-2.2	-1.1	BVES	Homo sapiens blood vessel epicardial substance (BVES), transcript variant B, mRNA [NM_147147]	11149
A_23_P145197	1.3	1.1	-1.0	-2.2	BYSL	Homo sapiens bystin-like (BYSL), mRNA [NM_004053]	705
A_24_P113131	2.8	-1.1	-1.1	-1.1	BZRAP1	Homo sapiens benzodiazapine receptor (peripheral) associated protein 1 (BZRAP1), transcript variant 1, mRNA [NM_004758]	9256
A_23_P152559	2.0	3.6	4.8	8.1	BZRAP1	Homo sapiens peripheral benzodiazepine receptor interacting protein mRNA, complete cds. [AF039571]	9256

A_24_P924389	-2.4	-3.6	-2.3	-1.3	BZW1	Homo sapiens basic leucine zipper and W2 domains 1 (BZW1), transcript variant 4, mRNA [NM_014670]	9689
A_23_P168916	-2.0	-3.1	-2.2	-4.7	CA1	Homo sapiens carbonic anhydrase I (CA1), transcript variant 2, mRNA [NM_001738]	759
A_23_P207461	-1.3	-3.2	-1.3	-1.3	CA10	Homo sapiens carbonic anhydrase X (CA10), transcript variant 2, mRNA [NM_020178]	56934
A_23_P163338	1.0	1.1	-9.2	-1.4	CA12	carbonic anhydrase XII [Source:HGNC Symbol;Acc:1371] [ENST00000178638]	771

A_23_P63379	1.5	3.1	2.2	3.4	CA14	Homo sapiens carbonic anhydrase XIV (CA14), mRNA [NM_012113]	23632
A_23_P95683	1.6	1.4	-3.2	1.3	CA5A	Homo sapiens carbonic anhydrase VA, mitochondrial (CA5A), nuclear gene encoding mitochondrial protein, mRNA [NM_001739]	763
A_24_P941268	1.1	-19.9	-1.0	-1.0	CA5B	Homo sapiens carbonic anhydrase VB, mitochondrial (CA5B), nuclear gene encoding mitochondrial protein, mRNA [NM_007220]	11238

A_24_P376760	9.4	4.5	4.4	4.3	CA6	Homo sapiens carbonic anhydrase VI (CA6), mRNA [NM_001215]	765
A_23_P106656	3.0	2.6	-1.5	1.5	CA7	Homo sapiens carbonic anhydrase VII (CA7), transcript variant 2, mRNA [NM_001014435]	766
A_23_P157793	2.4	-1.0	2.4	3.0	CA9	Homo sapiens carbonic anhydrase IX (CA9), mRNA [NM_001216]	768
A_23_P204879	4.7	2.8	4.7	2.7	CAB39L	Homo sapiens calcium binding protein 39-like (CAB39L), transcript variant 1, mRNA [NM_030925]	81617

A_24_P336113	3.3	-3.5	1.6	2.3	CABIN1	Homo sapiens calcineurin binding protein 1 (CABIN1), transcript variant 2, mRNA [NM_012295]	23523
A_23_P307761	2.2	1.2	2.5	1.8	CABLES2	Homo sapiens Cdk5 and Abl enzyme substrate 2 (CABLES2), mRNA [NM_031215]	81928
A_23_P308021	1.0	-2.9	-1.1	1.0	CABP4	Homo sapiens calcium binding protein 4 (CABP4), mRNA [NM_145200]	57010
A_24_P388662	-2.6	-1.8	-1.9	-60.8	CABS1	Homo sapiens calcium-binding protein, spermatid-specific 1 (CABS1), mRNA [NM_033122]	85438

A_24_P943922	5.1	5.1	5.0	3.9	CACHD1	Homo sapiens cache domain containing 1 (CACHD1), mRNA [NM_020925]	57685
A_24_P130559	1.4	-2.1	2.5	-1.1	CACNA1A	Homo sapiens calcium channel, voltage-dependent, P/Q type, alpha 1A subunit (CACNA1A), transcript variant 1, mRNA [NM_000068]	773
A_23_P159622	1.2	3.7	1.0	1.1	CACNA1B	Homo sapiens calcium channel, voltage-dependent, N type, alpha 1B subunit (CACNA1B), transcript variant 1, mRNA [NM_000718]	774

A_24_P160716	-1.3	-1.3	-2.8	1.0	CACNA1B	calcium channel, voltage-dependent, N type, alpha 1B subunit [Source:HGNC Symbol;Acc:1389] [ENST00000277550]	774
A_32_P153773	-2.4	1.0	-1.1	1.3	CACNA1B	Homo sapiens calcium channel, voltage-dependent, N type, alpha 1B subunit (CACNA1B), transcript variant 1, mRNA [NM_000718]	774
A_23_P84849	6.5	5.9	2.2	7.1	CACNA1B	Homo sapiens calcium channel, voltage-dependent, N type, alpha 1B subunit (CACNA1B), transcript variant 2, mRNA [NM_001243812]	774

A_23_P34554	1.8	1.1	-1.1	-4.5	CACNA1E	Homo sapiens calcium channel, voltage-dependent, R type, alpha 1E subunit (CACNA1E), transcript variant 3, mRNA [NM_000721]	777
A_24_P911621	-3.1	1.3	2.0	-3.0	CACNA1E	calcium channel, voltage-dependent, R type, alpha 1E subunit [Source:HGNC Symbol;Acc:1392] [ENST00000524607]	777
A_24_P401686	1.5	2.2	2.5	-1.0	CACNA1G	Homo sapiens calcium channel, voltage-dependent, T type, alpha 1G subunit (CACNA1G), transcript variant 15, mRNA [NM_198397]	8913

A_23_P107247	1.7	1.6	3.4	1.0	CACNA1G	Homo sapiens calcium channel, voltage-dependent, T type, alpha 1G subunit (CACNA1G), transcript variant 1, mRNA [NM_018896]	8913
A_23_P3483	4.4	1.6	1.4	1.1	CACNA1H	Homo sapiens calcium channel, voltage-dependent, T type, alpha 1H subunit (CACNA1H), transcript variant 1, mRNA [NM_021098]	8912
A_24_P399871	-1.7	-1.4	-3.6	-1.8	CACNA1I	Homo sapiens calcium channel, voltage-dependent, T type, alpha 1I subunit (CACNA1I), transcript variant 1, mRNA [NM_021096]	8911

A_23_P346900	1.0	1.1	1.8	-4.5	CACNA2D2	Homo sapiens calcium channel, voltage-dependent, alpha 2/delta subunit 2 (CACNA2D2), transcript variant 1, mRNA [NM_001005505]	9254
A_24_P402825	2.0	2.0	2.1	1.0	CACNA2D3	Homo sapiens calcium channel, voltage-dependent, alpha 2/delta subunit 3 (CACNA2D3), mRNA [NM_018398]	55799
A_23_P353014	1.0	-5.7	-1.1	-1.1	CACNA2D4	Homo sapiens calcium channel, voltage-dependent, alpha 2/delta subunit 4 (CACNA2D4), mRNA [NM_172364]	93589

A_24_P91165	2.2	-2.1	-1.6	2.2	CACNB1	Homo sapiens calcium channel, voltage-dependent, beta 1 subunit (CACNB1), transcript variant 3, mRNA [NM_199248]	782
A_23_P371794	1.3	2.6	2.4	4.4	CACNB3	Homo sapiens calcium channel, voltage-dependent, beta 3 subunit (CACNB3), transcript variant 1, mRNA [NM_000725]	784
A_23_P204016	1.1	2.0	-1.1	-1.0	CACNB3	Homo sapiens calcium channel, voltage-dependent, beta 3 subunit (CACNB3), transcript variant 1, mRNA [NM_000725]	784

A_23_P66180	2.2	2.5	-1.1	1.2	CACNG3	Homo sapiens calcium channel, voltage-dependent, gamma subunit 3 (CACNG3), mRNA [NM_006539]	10368
A_24_P70303	1.5	2.8	2.2	1.7	CACNG4	Homo sapiens calcium channel, voltage-dependent, gamma subunit 4 (CACNG4), mRNA [NM_014405]	27092
A_23_P414328	-1.5	-2.3	-1.0	-1.5	CACNG5	Homo sapiens calcium channel, voltage-dependent, gamma subunit 5 (CACNG5), mRNA [NM_145811]	27091
A_23_P4782	-1.4	-5.1	1.0	-1.3	CACNG7	Homo sapiens calcium channel, voltage-dependent, gamma subunit 7 (CACNG7), mRNA [NM_031896]	59284

A_23_P420218	-1.4	-1.4	-5.7	-1.8	CACNG8	Homo sapiens calcium channel, voltage-dependent, gamma subunit 8 (CACNG8), mRNA [NM_031895]	59283
A_32_P114574	-1.5	-2.0	-2.6	-1.3	CACYBP	Homo sapiens calcyclin binding protein (CACYBP), transcript variant 1, mRNA [NM_014412]	27101
A_24_P84428	-1.8	-1.9	-2.0	-1.9	CACYBP	Homo sapiens calcyclin binding protein (CACYBP), transcript variant 1, mRNA [NM_014412]	27101
A_32_P129200	-1.0	-2.6	-1.1	-1.1	CADM2	Homo sapiens cell adhesion molecule 2 (CADM2), transcript variant 3, mRNA [NM_153184]	253559

A_23_P201156	-2.1	1.9	1.9	-1.4	CADM3	Homo sapiens cell adhesion molecule 3 (CADM3), transcript variant 1, mRNA [NM_021189]	57863
A_23_P5064	2.1	4.0	1.9	1.2	CADM4	Homo sapiens cell adhesion molecule 4 (CADM4), mRNA [NM_145296]	199731
A_24_P246710	2.2	3.1	3.4	2.8	CADPS2	Homo sapiens Ca++-dependent secretion activator 2 (CADPS2), transcript variant 1, mRNA [NM_017954]	93664
A_23_P43197	5.3	2.6	2.8	3.0	CALB1	Homo sapiens calbindin 1, 28kDa (CALB1), mRNA [NM_004929]	793

A_23_P21092	2.8	1.3	2.4	2.4	CALB2	Homo sapiens calbindin 2 (CALB2), transcript variant CALB2, mRNA [NM_001740]	794
A_24_P936884	-1.0	-1.0	-2.3	-2.3	CALD1	Homo sapiens cDNA FLJ12160 fis, clone MAMMA1000567. [AK022222]	800
A_23_P163178	1.1	1.5	-2.0	1.0	CALM1	Homo sapiens calmodulin 1 (phosphorylase kinase, delta) (CALM1), transcript variant 1, mRNA [NM_006888]	801
A_24_P219785	-1.6	-4.0	-3.1	-1.3	CALM3	Homo sapiens calmodulin 3 (phosphorylase kinase, delta) (CALM3), mRNA [NM_005184]	808

A_23_P4944	-1.4	-1.2	-1.6	-2.4	CALM3	Homo sapiens calmodulin 3 (phosphorylase kinase, delta) (CALM3), mRNA [NM_005184]	808
A_23_P393080	-1.2	1.0	-2.1	-1.3	CALML3	Homo sapiens calmodulin-like 3 (CALML3), mRNA [NM_005185]	810
A_23_P129188	2.6	1.9	2.0	2.8	CALML4	Homo sapiens calmodulin-like 4 (CALML4), transcript variant 1, mRNA [NM_033429]	91860
A_23_P124095	-1.1	1.1	1.0	2.6	CALML5	Homo sapiens calmodulin-like 5 (CALML5), mRNA [NM_017422]	51806
A_23_P62588	-1.0	-1.0	-3.0	-1.0	CALML6	Homo sapiens calmodulin-like 6 (CALML6), mRNA [NM_138705]	163688

A_24_P380567	2.8	1.3	1.0	1.7	CALN1	Homo sapiens calneuron 1 (CALN1), transcript variant 1, mRNA [NM_031468]	83698
A_23_P27538	-3.7	1.5	-5.7	-2.1	CALR3	Homo sapiens calreticulin 3 (CALR3), mRNA [NM_145046]	125972
A_23_P122815	6.9	1.5	10.1	16.4	CALU	Homo sapiens calumenin (CALU), transcript variant 1, mRNA [NM_001219]	813
A_23_P86561	2.4	-1.0	1.3	2.1	CALY	Homo sapiens calcyon neuron-specific vesicular protein (CALY), mRNA [NM_015722]	50632

A_23_P121215	1.2	1.7	-2.7	1.7	CAMK1	Homo sapiens calcium/calmodulin-dependent protein kinase I (CAMK1), mRNA [NM_003656]	8536
A_24_P114739	1.9	-2.0	1.6	2.4	CAMK2D	Homo sapiens calcium/calmodulin-dependent protein kinase II delta (CAMK2D), transcript variant 1, mRNA [NM_172127]	817
A_23_P144458	-1.3	1.7	2.5	1.6	CAMK2D	Homo sapiens calcium/calmodulin-dependent protein kinase II delta (CAMK2D), transcript variant 3, mRNA [NM_001221]	817

A_23_P46903	1.0	-1.4	-30.6	1.4	CAMK2G	Homo sapiens calcium/calmodulin-dependent protein kinase II gamma (CAMK2G), transcript variant 1, mRNA [NM_172171]	818
A_24_P141332	-1.1	-1.1	-2.3	1.0	CAMK2G	Homo sapiens calcium/calmodulin-dependent protein kinase II gamma (CAMK2G), transcript variant 1, mRNA [NM_172171]	818
A_24_P117620	-1.5	1.0	-1.6	-2.4	CAMK2N1	Homo sapiens calcium/calmodulin-dependent protein kinase II inhibitor 1 (CAMK2N1), mRNA [NM_018584]	55450

A_32_P57146	1.3	2.5	1.2	1.8	CAMK2N1	Homo sapiens PRO1489 mRNA, complete cds. [AF116637]	55450
A_23_P250347	1.1	-9.0	-1.4	1.3	CAMK4	Homo sapiens calcium/calmodulin-dependent protein kinase IV (CAMK4), mRNA [NM_001744]	814
A_23_P207648	1.0	2.3	-1.2	1.3	CAMKK1	Homo sapiens calcium/calmodulin-dependent protein kinase kinase 1, alpha (CAMKK1), transcript variant 1, mRNA [NM_032294]	84254

A_23_P408830	1.7	2.6	1.9	1.0	CAMKK2	Homo sapiens calcium/calmodulin-dependent protein kinase kinase 2, beta (CAMKK2), transcript variant 6, mRNA [NM_172215]	10645
A_23_P153941	2.7	1.1	-1.4	1.1	CAMKMT	Homo sapiens calmodulin-lysine N-methyltransferase (CAMKMT), mRNA [NM_024766]	79823
A_24_P926285	-1.6	-1.6	-1.6	-3.4	CAMKMT	Homo sapiens chromosome 2 open reading frame 34, mRNA (cDNA clone IMAGE:4673016), complete cds. [BC029359]	79823

A_23_P29680	1.0	-28.1	-1.1	-1.1	CAMKV	Homo sapiens CaM kinase-like vesicle-associated (CAMKV), mRNA [NM_024046]	79012
A_24_P234856	5.0	4.9	4.7	4.1	CAMLG	Homo sapiens calcium modulating ligand (CAMLG), mRNA [NM_001745]	819
A_23_P213728	2.2	2.7	2.9	2.2	CAMLG	Homo sapiens calcium modulating ligand (CAMLG), mRNA [NM_001745]	819
A_23_P253791	-1.6	1.0	-1.0	2.6	CAMP	Homo sapiens cathelicidin antimicrobial peptide (CAMP), mRNA [NM_004345]	820

A_24_P175460	-1.2	-8.4	1.4	1.5	CAMSAP1	Homo sapiens calmodulin regulated spectrin-associated protein 1 (CAMSAP1), mRNA [NM_015447]	157922
A_23_P406702	2.2	3.4	2.5	2.1	CAMSAP2	Homo sapiens calmodulin regulated spectrin-associated protein family, member 2 (CAMSAP2), mRNA [NM_203459]	23271
A_23_P394043	1.3	3.8	4.9	1.1	CAMSAP3	Homo sapiens calmodulin regulated spectrin-associated protein family, member 3 (CAMSAP3), transcript variant 1, mRNA [NM_001080429]	57662

A_24_P302406	-2.3	-1.0	1.4	1.4	CAMTA1	Homo sapiens calmodulin binding transcription activator 1 (CAMTA1), transcript variant 3, mRNA [NM_001242701]	23261
A_24_P220921	1.2	-3.4	-1.2	-1.1	CAMTA1	Homo sapiens calmodulin binding transcription activator 1 (CAMTA1), transcript variant 1, mRNA [NM_015215]	23261
A_23_P365189	2.5	2.4	1.6	-1.1	CAMTA2	Homo sapiens calmodulin binding transcription activator 2 (CAMTA2), transcript variant 1, mRNA [NM_015099]	23125

A_23_P25137	1.5	6.1	1.9	3.0	CAND1	Homo sapiens cullin-associated and neddylation-dissociated 1 (CAND1), mRNA [NM_018448]	55832
A_23_P72180	1.1	2.0	1.0	1.1	CAND1	Homo sapiens TIP120 protein (TIP120) mRNA, complete cds. [AF157326]	55832
A_23_P25141	1.9	2.0	-2.0	2.2	CAND1	Homo sapiens cullin-associated and neddylation-dissociated 1 (CAND1), mRNA [NM_018448]	55832
A_24_P116669	2.2	3.2	1.3	2.3	CANT1	Homo sapiens calcium activated nucleotidase 1 (CANT1), transcript variant 1, mRNA [NM_138793]	124583

A_23_P147185	1.5	-1.0	-2.0	-1.0	CANX	Homo sapiens calnexin (CANX), transcript variant 1, mRNA [NM_001746]	821
A_24_P62615	-1.6	-1.4	-2.1	1.2	CAP1	Homo sapiens CAP, adenylate cyclase-associated protein 1 (yeast) (CAP1), transcript variant 1, mRNA [NM_006367]	10487
A_23_P421664	1.5	-6.5	1.3	1.3	CAP2	Homo sapiens CAP, adenylate cyclase-associated protein, 2 (yeast) (CAP2), mRNA [NM_006366]	10486
A_23_P165636	3.9	-1.9	1.7	2.7	CAPG	Homo sapiens capping protein (actin filament), gelsolin-like (CAPG), mRNA [NM_001747]	822

A_23_P312217	-2.5	-3.5	-2.3	-1.6	CAPN10	Homo sapiens calpain 10 (CAPN10), transcript variant 1, mRNA [NM_023083]	11132
A_23_P7853	-1.2	-11.2	-1.3	-1.4	CAPN11	Homo sapiens calpain 11 (CAPN11), mRNA [NM_007058]	11131
A_23_P16409	-2.1	-1.6	-8.6	-2.0	CAPN12	Homo sapiens calpain 12 (CAPN12), mRNA [NM_144691]	147968
A_23_P101972	2.2	-1.1	2.6	1.5	CAPN13	Homo sapiens calpain 13 (CAPN13), mRNA [NM_144575]	92291
A_32_P167076	-1.4	-4.6	-1.1	-1.5	CAPN14	Homo sapiens calpain 14 (CAPN14), mRNA [NM_001145122]	440854
A_23_P218190	2.1	1.2	2.8	2.1	CAPN3	Homo sapiens calpain 3, (p94) (CAPN3), transcript variant 1, mRNA [NM_000070]	825

A_23_P105197	-2.1	-1.0	1.3	-3.0	CAPN5	Homo sapiens calpain 5 (CAPN5), mRNA [NM_004055]	726
A_23_P83381	1.0	1.0	2.3	-1.9	CAPN8	Homo sapiens calpain 8 (CAPN8), mRNA [NM_001143962]	388743
A_24_P107208	-4.6	-1.2	-4.3	-1.3	CAPRIN1	Homo sapiens cell cycle associated protein 1 (CAPRIN1), transcript variant 2, mRNA [NM_203364]	4076
A_32_P235872	-9.0	-1.1	1.0	-1.0	CAPRIN1	Homo sapiens cell cycle associated protein 1 (CAPRIN1), transcript variant 1, mRNA [NM_005898]	4076

A_23_P98722	2.7	3.0	3.0	2.6	CAPRIN1	Homo sapiens cell cycle associated protein 1 (CAPRIN1), transcript variant 1, mRNA [NM_005898]	4076
A_24_P400675	-1.0	2.5	1.4	1.2	CAPRIN2	Homo sapiens caprin family member 2 (CAPRIN2), transcript variant 2, mRNA [NM_023925]	65981
A_23_P87532	-1.4	-1.3	-1.1	-2.6	CAPRIN2	Homo sapiens caprin family member 2 (CAPRIN2), transcript variant 1, mRNA [NM_001002259]	65981
A_23_P78958	1.1	-1.9	-2.0	-8.7	CAPS	Homo sapiens calcyphosine (CAPS), transcript variant 1, mRNA [NM_004058]	828

A_23_P380379	1.0	-2.7	-1.7	-1.0	CAPS2	calcyphosine 2 [Source:HGNC Symbol;Acc:16471] [ENST00000328705]	84698
A_24_P379379	-2.3	-5.1	-2.7	-1.1	CAPZA1	Homo sapiens capping protein (actin filament) muscle Z-line, alpha 1 (CAPZA1), mRNA [NM_006135]	829
A_23_P307940	-1.1	-7.0	-1.3	-2.0	CAPZA2	Homo sapiens capping protein (actin filament) muscle Z-line, alpha 2 (CAPZA2), mRNA [NM_006136]	830
A_23_P87727	4.0	3.8	3.4	2.9	CAPZA3	Homo sapiens capping protein (actin filament) muscle Z-line, alpha 3 (CAPZA3), mRNA [NM_033328]	93661

A_23_P82324	-1.5	-8.2	-5.4	-2.8	CARD11	Homo sapiens caspase recruitment domain family, member 11 (CARD11), mRNA [NM_032415]	84433
A_23_P320070	1.2	1.1	-4.1	-1.2	CARD14	Homo sapiens caspase recruitment domain family, member 14 (CARD14), transcript variant 2, mRNA [NM_052819]	79092
A_23_P207879	11.9	7.9	9.6	1.2	CARD14	Homo sapiens caspase recruitment domain family, member 14 (CARD14), transcript variant 1, mRNA [NM_024110]	79092

A_23_P64173	1.1	1.0	-3.4	-1.0	CARD16	Homo sapiens caspase recruitment domain family, member 16 (CARD16), transcript variant 1, mRNA [NM_001017534]	114769
A_24_P192805	-1.1	1.1	1.0	-3.0	CARD17	Homo sapiens caspase recruitment domain family, member 17 (CARD17), mRNA [NM_001007232]	440068
A_23_P86975	-4.3	-1.2	-1.3	-1.7	CARD18	Homo sapiens caspase recruitment domain family, member 18 (CARD18), mRNA [NM_021571]	59082

A_24_P180830	1.1	1.4	1.8	5.4	CARD9	caspase recruitment domain family, member 9 [Source:HGNC Symbol;Acc:16391] [ENST00000481053]	64170
A_23_P38085	3.7	5.4	3.4	1.9	CARHSP1	Homo sapiens calcium regulated heat stable protein 1, 24kDa (CARHSP1), transcript variant 2, mRNA [NM_001042476]	23589
A_24_P401709	-1.4	4.1	1.6	-3.4	CARKD	Homo sapiens clone 664 unknown mRNA, partial sequence. [AF091088]	55739
A_24_P95439	-4.1	-6.7	-1.7	-2.0	CARS	Homo sapiens cysteinyl-tRNA synthetase (CARS), transcript variant 3, mRNA [NM_001014437]	833

A_23_P128624	1.1	2.1	1.4	1.1	CARS2	Homo sapiens cysteinyl-tRNA synthetase 2, mitochondrial (putative) (CARS2), nuclear gene encoding mitochondrial protein, mRNA [NM_024537]	79587
A_23_P167493	1.1	2.1	1.0	1.1	CARTPT	Homo sapiens CART prepropeptide (CARTPT), mRNA [NM_004291]	9607
A_24_P125353	2.3	-2.0	-2.0	-1.3	CASC3	Homo sapiens cancer susceptibility candidate 3 (CASC3), mRNA [NM_007359]	22794
A_23_P374288	1.2	2.3	1.1	1.6	CASC3	Homo sapiens cancer susceptibility candidate 3 (CASC3), mRNA [NM_007359]	22794

A_24_P284213	2.5	2.5	2.9	2.5	CASC4	Homo sapiens cancer susceptibility candidate 4 (CASC4), transcript variant 2, mRNA [NM_177974]	113201
A_23_P140469	1.0	1.0	1.0	-2.4	CASC4	Homo sapiens cancer susceptibility candidate 4 (CASC4), transcript variant 1, mRNA [NM_138423]	113201
A_24_P940678	1.1	-2.6	1.0	1.1	CASC5	cancer susceptibility candidate 5 [Source:HGNC Symbol;Acc:24054] [ENST00000526913]	57082
A_24_P69379	1.5	7.8	3.2	3.4	CASD1	Homo sapiens CAS1 domain containing 1 (CASD1), mRNA [NM_022900]	64921

A_23_P202978	1.1	-4.0	-1.0	-1.0	CASP1	Homo sapiens caspase 1, apoptosis-related cysteine peptidase (interleukin 1, beta, convertase) (CASP1), transcript variant alpha, mRNA [NM_033292]	834
A_24_P139993	-4.2	-6.2	-1.0	1.1	CASP10	Homo sapiens caspase 10, apoptosis-related cysteine peptidase (CASP10), transcript variant 1, mRNA [NM_032977]	843
A_23_P90407	2.6	7.5	5.9	3.9	CASP14	Homo sapiens caspase 14, apoptosis-related cysteine peptidase (CASP14), mRNA [NM_012114]	23581

A_23_P215701	-1.0	-1.0	-5.7	-1.0	CASP2	Homo sapiens caspase 2, apoptosis-related cysteine peptidase (CASP2), transcript variant 1, mRNA [NM_032982]	835
A_23_P35906	1.5	1.8	1.3	3.6	CASP4	Homo sapiens caspase 4, apoptosis-related cysteine peptidase (CASP4), transcript variant gamma, mRNA [NM_033306]	837
A_23_P35912	-2.3	2.2	-1.8	-2.0	CASP4	Homo sapiens caspase 4, apoptosis-related cysteine peptidase (CASP4), transcript variant gamma, mRNA [NM_033306]	837

A_24_P157087	-2.2	1.0	1.0	-1.3	CASP8	Homo sapiens caspase 8, apoptosis-related cysteine peptidase (CASP8), transcript variant B, mRNA [NM_033355]	841
A_24_P148499	-5.8	1.0	-1.0	1.1	CASP8	Homo sapiens caspase 8, apoptosis-related cysteine peptidase (CASP8), transcript variant E, mRNA [NM_033358]	841
A_23_P97309	-2.2	-1.5	-1.6	-4.0	CASP9	Homo sapiens caspase 9, apoptosis-related cysteine peptidase (CASP9), transcript variant alpha, mRNA [NM_001229]	842

A_24_P935951	-1.4	-2.4	-2.9	-1.6	CASZ1	Homo sapiens castor zinc finger 1 (CASZ1), transcript variant 1, mRNA [NM_001079843]	54897
A_23_P201295	1.2	1.1	-4.1	1.1	CASZ1	Homo sapiens castor zinc finger 1 (CASZ1), transcript variant 2, mRNA [NM_017766]	54897
A_24_P229084	6.1	5.9	2.0	5.4	CATSPERG	Homo sapiens cation channel, sperm-associated, gamma (CATSPERG), mRNA [NM_021185]	57828
A_24_P238412	1.8	1.8	1.8	-5.4	CATX-1	Homo sapiens CATX-1 mRNA, partial cds. [AF083117]	100506513
A_23_P123071	6.6	3.4	5.9	1.9	CAV2	Homo sapiens caveolin 2 (CAV2), transcript variant 1, mRNA [NM_001233]	858

A_24_P925040	-6.6	-3.0	-1.0	-1.0	CAV2	Homo sapiens caveolin 2 (CAV2), transcript variant 1, mRNA [NM_001233]	858
A_24_P251599	-1.2	1.4	1.3	2.5	CAV3	Homo sapiens caveolin 3 (CAV3), transcript variant 2, mRNA [NM_001234]	859
A_24_P107918	-1.9	-2.2	-1.7	1.7	CBFB	Homo sapiens core-binding factor, beta subunit (CBFB), transcript variant 1, mRNA [NM_022845]	865
A_23_P339191	1.3	2.7	1.7	2.0	CBL	Homo sapiens Cas-Br-M (murine) ecotropic retroviral transforming sequence (CBL), mRNA [NM_005188]	867

A_23_P29830	-28.7	-1.2	-2.4	-1.9	CBLB	Homo sapiens Cas-Br-M (murine) ecotropic retroviral transforming sequence b (CBLB), mRNA [NM_170662]	868
A_23_P3159	2.0	-1.6	-1.6	-1.9	CBLN3	Homo sapiens cerebellin 3 precursor (CBLN3), mRNA [NM_001039771]	643866
A_23_P313428	-26.2	-4.3	-1.1	-1.1	CBLN4	Homo sapiens cerebellin 4 precursor (CBLN4), mRNA [NM_080617]	140689
A_23_P29046	-1.6	-1.0	-3.0	-1.0	CBR1	Homo sapiens carbonyl reductase 1 (CBR1), mRNA [NM_001757]	873

A_24_P48856	2.1	1.1	3.7	2.7	CBS	Homo sapiens cystathionine-beta-synthase (CBS), transcript variant 1, mRNA [NM_000071]	875
A_23_P166306	-2.2	-1.0	1.2	-1.0	CBS	Homo sapiens cystathionine-beta-synthase (CBS), transcript variant 1, mRNA [NM_000071]	875
A_32_P161432	2.2	1.1	2.2	-1.3	CBWD2	Homo sapiens COBW domain containing 2 (CBWD2), mRNA [NM_172003]	150472
A_24_P170593	1.1	1.1	-3.2	1.0	CBWD6	Homo sapiens clone 1659351 unknown mRNA. [AF293368]	644019

A_23_P107507	1.7	-1.3	-1.2	2.6	CBX1	Homo sapiens chromobox homolog 1 (CBX1), transcript variant 1, mRNA [NM_006807]	10951
A_23_P353567	-1.3	-1.4	-1.3	-2.8	CBX2	Homo sapiens chromobox homolog 2 (CBX2), transcript variant 2, mRNA [NM_032647]	84733
A_23_P89621	1.9	2.1	4.0	1.0	CBX4	Homo sapiens chromobox homolog 4 (CBX4), mRNA [NM_003655]	8535
A_24_P664995	1.4	-1.8	1.1	2.7	CBX5	Homo sapiens chromobox homolog 5 (CBX5), transcript variant 1, mRNA [NM_001127322]	23468
A_24_P254437	5.7	6.7	4.6	6.2	CBX6	chromobox homolog 6 [Source:HGNC Symbol;Acc:1556] [ENST00000407418]	23466

A_23_P250735	2.3	2.1	1.0	1.5	CBX7	Homo sapiens chromobox homolog 7 (CBX7), mRNA [NM_175709]	23492
A_23_P55873	7.0	2.4	1.5	2.0	CC2D1A	Homo sapiens coiled-coil and C2 domain containing 1A (CC2D1A), mRNA [NM_017721]	54862
A_24_P41371	1.7	1.8	2.7	-1.0	CC2D1A	Homo sapiens coiled-coil and C2 domain containing 1A (CC2D1A), mRNA [NM_017721]	54862
A_24_P76740	2.0	2.1	-1.4	-1.0	CC2D1B	Homo sapiens coiled-coil and C2 domain containing 1B (CC2D1B), mRNA [NM_032449]	200014

A_23_P55544	-2.3	-2.4	-3.0	-6.4	CCBE1	Homo sapiens collagen and calcium binding EGF domains 1 (CCBE1), mRNA [NM_133459]	147372
A_23_P46017	1.5	2.0	1.6	2.7	CCBL2	Homo sapiens cysteine conjugate-beta lyase 2 (CCBL2), transcript variant 1, mRNA [NM_001008661]	56267
A_23_P388914	1.6	1.1	-2.6	1.0	CCDC101	Homo sapiens coiled-coil domain containing 101 (CCDC101), mRNA [NM_138414]	112869
A_23_P65983	54.0	52.7	58.1	53.9	CCDC102A	Homo sapiens coiled-coil domain containing 102A (CCDC102A), mRNA [NM_033212]	92922

A_32_P176701	-2.1	1.7	-2.0	1.4	CCDC108	Homo sapiens coiled-coil domain containing 108 (CCDC108), transcript variant 1, mRNA [NM_194302]	255101
A_23_P255376	-4.9	-1.1	-1.8	-1.1	CCDC109B	Homo sapiens coiled-coil domain containing 109B (CCDC109B), mRNA [NM_017918]	55013
A_23_P384532	1.0	-1.3	-4.7	-1.0	CCDC11	Homo sapiens coiled-coil domain containing 11 (CCDC11), mRNA [NM_145020]	220136
A_24_P73730	1.0	-14.9	-1.1	1.0	CCDC113	Homo sapiens coiled-coil domain containing 113 (CCDC113), transcript variant 1, mRNA [NM_014157]	29070

A_24_P264154	1.3	2.1	-1.5	1.7	CCDC113	Homo sapiens coiled-coil domain containing 113 (CCDC113), transcript variant 1, mRNA [NM_014157]	29070
A_32_P24125	2.5	3.2	13.8	2.2	CCDC114	Homo sapiens coiled-coil domain containing 114 (CCDC114), transcript variant 2, mRNA [NM_144577]	93233
A_24_P54485	5.2	2.2	5.2	3.5	CCDC115	Homo sapiens coiled-coil domain containing 115 (CCDC115), mRNA [NM_032357]	84317
A_23_P372334	1.1	1.1	-2.4	-2.9	CCDC12	Homo sapiens coiled-coil domain containing 12 (CCDC12), mRNA [NM_144716]	151903

A_24_P48983	4.5	2.7	4.8	1.5	CCDC120	Homo sapiens coiled-coil domain containing 120 (CCDC120), transcript variant 3, mRNA [NM_033626]	90060
A_24_P319684	1.3	-3.4	1.6	-2.9	CCDC121	Homo sapiens coiled-coil domain containing 121 (CCDC121), transcript variant 3, mRNA [NM_024584]	79635
A_23_P302078	1.2	1.6	-4.5	-1.6	CCDC122	coiled-coil domain containing 122 [Source:HGNC Symbol;Acc:26478] [ENST00000476570]	160857
A_32_P213661	-1.0	-2.3	-1.1	1.3	CCDC122	Homo sapiens coiled-coil domain containing 122 (CCDC122), mRNA [NM_144974]	160857

A_23_P342118	1.4	1.9	-2.1	1.3	CCDC124	Homo sapiens coiled-coil domain containing 124 (CCDC124), transcript variant 1, mRNA [NM_138442]	115098
A_23_P432591	-5.5	-1.0	-1.0	1.0	CCDC125	Homo sapiens coiled-coil domain containing 125 (CCDC125), mRNA [NM_176816]	202243
A_23_P101461	2.2	2.1	2.1	2.2	CCDC130	Homo sapiens coiled-coil domain containing 130 (CCDC130), mRNA [NM_030818]	81576
A_24_P409219	1.1	-6.0	1.0	1.3	CCDC132	Homo sapiens coiled-coil domain containing 132 (CCDC132), transcript variant 1, mRNA [NM_017667]	55610

A_23_P215614	1.0	-1.0	-1.1	-2.1	CCDC132	Homo sapiens coiled-coil domain containing 132 (CCDC132), transcript variant 1, mRNA [NM_017667]	55610
A_23_P129367	1.0	-1.0	-1.1	2.2	CCDC135	Homo sapiens coiled-coil domain containing 135 (CCDC135), mRNA [NM_032269]	84229
A_23_P429478	-8.3	1.1	1.0	1.1	CCDC140	Homo sapiens coiled-coil domain containing 140 (CCDC140), mRNA [NM_153038]	151278
A_32_P216369	1.0	-10.8	1.1	1.0	CCDC144A	Homo sapiens coiled-coil domain containing 144A (CCDC144A), mRNA [NM_014695]	9720

A_23_P168771	-1.1	1.0	-2.5	-2.5	CCDC146	Homo sapiens coiled-coil domain containing 146 (CCDC146), mRNA [NM_020879]	57639
A_23_P419764	2.6	-1.5	3.0	1.7	CCDC149	coiled-coil domain containing 149 [Source:HGNC Symbol;Acc:25405] [ENST00000503881]	91050
A_32_P108738	-3.4	1.0	1.0	1.1	CCDC149	Homo sapiens coiled-coil domain containing 149 (CCDC149), transcript variant 1, mRNA [NM_173463]	91050
A_23_P44942	1.0	1.0	-2.2	-1.7	CCDC15	Homo sapiens coiled-coil domain containing 15 (CCDC15), mRNA [NM_025004]	80071

A_23_P39574	-1.0	-2.0	-1.0	-1.0	CCDC150	Homo sapiens coiled-coil domain containing 150 (CCDC150), mRNA [NM_001080539]	284992
A_23_P326893	-2.4	-2.5	-1.7	-2.0	CCDC151	Homo sapiens coiled-coil domain containing 151 (CCDC151), mRNA [NM_145045]	115948
A_24_P75920	2.1	4.3	4.0	1.1	CCDC159	Homo sapiens coiled-coil domain containing 159 (CCDC159), mRNA [NM_001080503]	126075
A_23_P360605	1.7	1.0	-7.5	-1.1	CCDC165	Homo sapiens coiled-coil domain containing 165 (CCDC165), mRNA [NM_015210]	23255

A_23_P14216	1.6	4.2	1.6	1.1	CCDC169	Homo sapiens coiled-coil domain containing 169 (CCDC169), transcript variant 7, mRNA [NM_001198908]	728591
A_24_P310864	-1.4	-1.6	-2.7	-1.1	CCDC24	Homo sapiens coiled-coil domain containing 24 (CCDC24), mRNA [NM_152499]	149473
A_24_P379353	1.5	1.4	2.2	1.0	CCDC24	Homo sapiens coiled-coil domain containing 24 (CCDC24), mRNA [NM_152499]	149473
A_24_P158142	-3.2	1.6	-6.6	-1.7	CCDC25	Homo sapiens coiled-coil domain containing 25 (CCDC25), mRNA [NM_018246]	55246

A_23_P71300	-1.4	-5.8	-1.1	-1.1	CCDC25	Homo sapiens coiled-coil domain containing 25 (CCDC25), mRNA [NM_018246]	55246
A_23_P62764	-1.5	-3.7	-2.6	-2.6	CCDC28B	Homo sapiens coiled-coil domain containing 28B (CCDC28B), mRNA [NM_024296]	79140
A_24_P349196	1.0	1.3	-1.7	-2.1	CCDC30	Homo sapiens coiled-coil domain containing 30 (CCDC30), mRNA [NM_001080850]	728621
A_24_P195831	3.4	2.2	2.8	1.6	CCDC34	Homo sapiens coiled-coil domain containing 34 (CCDC34), transcript variant 1, mRNA [NM_030771]	91057

A_24_P134765	2.7	2.7	2.6	2.6	CCDC36	Homo sapiens coiled-coil domain containing 36 (CCDC36), transcript variant 1, mRNA [NM_178173]	339834
A_32_P54037	1.3	1.2	2.3	-1.3	CCDC38	Homo sapiens coiled-coil domain containing 38 (CCDC38), mRNA [NM_182496]	120935
A_32_P404549	1.0	-1.0	-1.0	-2.7	CCDC39	Homo sapiens coiled-coil domain containing 39 (CCDC39), mRNA [NM_181426]	339829
A_23_P162378	-2.0	1.7	1.5	-1.7	CCDC41	Homo sapiens coiled-coil domain containing 41 (CCDC41), transcript variant 1, mRNA [NM_016122]	51134

A_23_P49616	-1.3	-11.1	2.0	1.1	CCDC47	Homo sapiens coiled-coil domain containing 47 (CCDC47), mRNA [NM_020198]	57003
A_24_P176733	3.8	3.7	3.7	3.6	CCDC47	Homo sapiens coiled-coil domain containing 47 (CCDC47), mRNA [NM_020198]	57003
A_24_P51777	-4.4	1.1	-1.1	1.2	CCDC50	Homo sapiens coiled-coil domain containing 50 (CCDC50), transcript variant 2, mRNA [NM_178335]	152137
A_24_P910733	1.3	2.9	2.3	-3.3	CCDC50	Homo sapiens coiled-coil domain containing 50 (CCDC50), transcript variant 2, mRNA [NM_178335]	152137

A_23_P384024	1.0	-1.0	-1.1	-4.9	CCDC50	Homo sapiens coiled-coil domain containing 50 (CCDC50), transcript variant 2, mRNA [NM_178335]	152137
A_23_P69362	2.4	1.0	1.9	-1.0	CCDC51	Homo sapiens coiled-coil domain containing 51 (CCDC51), mRNA [NM_024661]	79714
A_23_P356965	-14.9	1.1	-1.0	-1.0	CCDC57	Homo sapiens cDNA FLJ43953 fis, clone TESTI4015477. [AK125941]	284001
A_23_P337327	1.1	-1.4	-2.9	-2.0	CCDC57	Homo sapiens mRNA; cDNA DKFZp434C2019 (from clone DKFZp434C2019); partial cds. [AL137530]	284001

A_23_P365119	2.0	2.5	1.7	-1.1	CCDC57	coiled-coil domain containing 57 [Source:HGNC Symbol;Acc:27564] [ENST00000324808]	284001
A_24_P896381	2.5	1.1	1.6	1.3	CCDC57	Homo sapiens coiled-coil domain containing 57 (CCDC57), mRNA [NM_198082]	284001
A_32_P215113	2.1	-1.0	2.0	-1.2	CCDC58	Homo sapiens coiled-coil domain containing 58 (CCDC58), mRNA [NM_001017928]	131076
A_23_P400147	4.6	-1.7	1.8	3.3	CCDC61	Homo sapiens coiled-coil domain containing 61 (CCDC61), mRNA [NM_001080402]	729440

A_24_P273647	4.6	5.7	5.3	2.7	CCDC64B	Homo sapiens mRNA; cDNA DKFZp666 L166 (from clone DKFZp666 L166). [AL833749]	146439
A_32_P153954	1.6	1.6	3.2	1.0	CCDC67	Homo sapiens coiled-coil domain containing 67 (CCDC67), mRNA [NM_1816 45]	159989
A_23_P213810	-1.1	-1.1	-1.2	-3.0	CCDC69	Homo sapiens coiled-coil domain containing 69 (CCDC69), mRNA [NM_0156 21]	26112
A_24_P97825	3.8	3.5	3.4	-1.9	CCDC69	Homo sapiens coiled-coil domain containing 69 (CCDC69), mRNA [NM_0156 21]	26112

A_23_P374294	1.3	1.5	1.9	-3.0	CCDC7	Homo sapiens coiled-coil domain containing 7 (CCDC7), transcript variant 1, mRNA [NM_145023]	221016
A_23_P2857	-3.7	-1.0	1.0	1.0	CCDC70	Homo sapiens coiled-coil domain containing 70 (CCDC70), mRNA [NM_031290]	83446
A_23_P61524	-35.6	1.0	-1.1	-1.3	CCDC71	Homo sapiens coiled-coil domain containing 71 (CCDC71), mRNA [NM_022903]	64925
A_24_P358425	3.7	2.0	1.9	1.4	CCDC75	Homo sapiens coiled-coil domain containing 75 (CCDC75), mRNA [NM_174931]	253635

A_24_P396557	-2.2	-2.4	-1.0	-2.0	CCDC76	Homo sapiens coiled-coil domain containing 76 (CCDC76), mRNA [NM_019083]	54482
A_23_P354864	-2.1	-2.2	-2.5	-1.3	CCDC78	Homo sapiens coiled-coil domain containing 78 (CCDC78), mRNA [NM_001031737]	124093
A_23_P164958	1.1	2.2	1.1	-1.1	CCDC8	Homo sapiens coiled-coil domain containing 8 (CCDC8), mRNA [NM_032040]	83987
A_23_P58082	-3.2	1.1	1.0	1.0	CCDC80	Homo sapiens coiled-coil domain containing 80 (CCDC80), transcript variant 1, mRNA [NM_199511]	151887

A_23_P128008	1.1	1.1	1.1	-2.6	CCDC81	Homo sapiens coiled-coil domain containing 81 (CCDC81), transcript variant 2, mRNA [NM_021827]	60494
A_24_P372580	1.1	-3.4	1.1	1.1	CCDC82	Homo sapiens coiled-coil domain containing 82 (CCDC82), mRNA [NM_024725]	79780
A_23_P217972	4.8	5.6	3.6	8.3	CCDC82	Homo sapiens HSPC048 mRNA, complete cds. [AF161533]	79780
A_24_P636332	2.0	1.2	-1.0	1.0	CCDC84	Homo sapiens coiled-coil domain containing 84 (CCDC84), mRNA [NM_198489]	338657

A_23_P37391	-1.0	-1.0	-2.6	-1.0	CCDC85C	Homo sapiens coiled-coil domain containing 85C (CCDC85C), mRNA [NM_001144995]	317762
A_24_P263259	-1.0	-1.0	1.9	-2.2	CCDC85C	Homo sapiens coiled-coil domain containing 85C (CCDC85C), mRNA [NM_001144995]	317762
A_24_P38944	2.2	3.1	1.5	7.1	CCDC86	Homo sapiens coiled-coil domain containing 86 (CCDC86), mRNA [NM_024098]	79080
A_23_P17269	-1.6	1.1	-2.1	1.1	CCDC88A	Homo sapiens coiled-coil domain containing 88A (CCDC88A), transcript variant 2, mRNA [NM_018084]	55704

A_23_P28664	-1.4	1.4	-2.7	1.4	CCDC88A	Homo sapiens coiled-coil domain containing 88A (CCDC88A), transcript variant 2, mRNA [NM_018084]	55704
A_24_P20120	2.3	2.2	-1.1	-1.1	CCDC88A	Homo sapiens coiled-coil domain containing 88A (CCDC88A), transcript variant 2, mRNA [NM_018084]	55704
A_23_P24389	3.6	7.9	1.5	2.4	CCDC88B	Homo sapiens coiled-coil domain containing 88B (CCDC88B), mRNA [NM_032251]	283234
A_23_P24384	-1.0	-1.1	-3.0	-1.1	CCDC88B	Homo sapiens coiled-coil domain containing 88B (CCDC88B), mRNA [NM_032251]	283234

A_24_P289170	-2.1	-1.2	-1.6	-1.4	CCDC88C	Homo sapiens coiled-coil domain containing 88C (CCDC88C), mRNA [NM_001080414]	440193
A_32_P170811	-1.4	-2.2	-1.3	-1.4	CCDC88C	Homo sapiens coiled-coil domain containing 88C (CCDC88C), mRNA [NM_001080414]	440193
A_24_P136094	1.9	2.1	-1.2	4.8	CCDC88C	Homo sapiens coiled-coil domain containing 88C (CCDC88C), mRNA [NM_001080414]	440193
A_23_P379945	1.0	-14.5	-1.1	-1.1	CCDC88C	Homo sapiens coiled-coil domain containing 88C (CCDC88C), mRNA [NM_001080414]	440193

A_24_P192988	5.8	6.5	11.2	6.5	CCDC89	Homo sapiens coiled-coil domain containing 89 (CCDC89), mRNA [NM_152723]	220388
A_23_P417965	-1.6	2.4	-1.3	-1.3	CCDC9	Homo sapiens coiled-coil domain containing 9 (CCDC9), mRNA [NM_015603]	26093
A_24_P133085	-1.2	-2.2	-2.2	1.1	CCDC90B	Homo sapiens coiled-coil domain containing 90B (CCDC90B), mRNA [NM_021825]	60492
A_32_P184003	1.1	-4.8	-1.0	1.0	CCDC91	Homo sapiens coiled-coil domain containing 91 (CCDC91), mRNA [NM_018318]	55297

A_24_P372959	-1.4	-3.5	1.0	1.0	CCDC91	Homo sapiens coiled-coil domain containing 91 (CCDC91), mRNA [NM_018318]	55297
A_23_P91062	-1.1	-2.8	-3.2	-2.4	CCDC93	Homo sapiens coiled-coil domain containing 93 (CCDC93), mRNA [NM_019044]	54520
A_23_P398044	-2.1	1.1	1.1	1.2	CCDC97	Homo sapiens coiled-coil domain containing 97 (CCDC97), mRNA [NM_052848]	90324
A_23_P60227	2.1	1.6	2.0	1.4	CCIN	Homo sapiens calicin (CCIN), mRNA [NM_005893]	881
A_23_P66635	-8.8	1.0	1.0	-1.3	CCL11	Homo sapiens chemokine (C-C motif) ligand 11 (CCL11), mRNA [NM_002986]	6356

A_23_P26325	1.7	1.3	-3.5	-1.1	CCL17	Homo sapiens chemokine (C-C motif) ligand 17 (CCL17), mRNA [NM_002987]	6361
A_23_P123853	3.2	2.7	1.0	3.2	CCL19	Homo sapiens chemokine (C-C motif) ligand 19 (CCL19), mRNA [NM_006274]	6363
A_23_P17065	5.5	1.5	10.9	11.0	CCL20	Homo sapiens chemokine (C-C motif) ligand 20 (CCL20), transcript variant 1, mRNA [NM_004591]	6364
A_23_P112470	4.7	7.2	1.2	1.4	CCL21	Homo sapiens chemokine (C-C motif) ligand 21 (CCL21), mRNA [NM_002989]	6366

A_24_P319088	1.6	-2.1	1.0	-1.4	CCL23	Homo sapiens chemokine (C-C motif) ligand 23 (CCL23), transcript variant CKbeta8-1, mRNA [NM_005064]	6368
A_23_P55828	1.5	-2.3	-1.1	-1.0	CCL25	Homo sapiens chemokine (C-C motif) ligand 25 (CCL25), transcript variant 1, mRNA [NM_005624]	6370
A_23_P503072	-2.3	-3.0	-2.9	-2.9	CCL28	Homo sapiens chemokine (C-C motif) ligand 28 (CCL28), mRNA [NM_148672]	56477
A_23_P373017	1.0	-1.0	-6.3	-1.1	CCL3	Homo sapiens mRNA for pLD78 peptide, complete cds. [D00044]	6348

A_23_P207564	4.3	-1.3	3.9	-1.2	CCL4	Homo sapiens chemokine (C-C motif) ligand 4 (CCL4), transcript variant 1, mRNA [NM_002984]	6351
A_23_P78037	2.0	2.5	2.4	2.4	CCL7	Homo sapiens chemokine (C-C motif) ligand 7 (CCL7), mRNA [NM_006273]	6354
A_23_P65757	1.7	-3.4	2.4	1.6	CCNB2	Homo sapiens cyclin B2 (CCNB2), mRNA [NM_004701]	9133
A_32_P72822	-1.6	-1.9	-2.1	-1.8	CCNB2	Homo sapiens cDNA FLJ13342 fis, clone OVARC1001950. [AK023404]	9133
A_23_P171107	2.3	2.2	2.2	2.2	CCNB3	Homo sapiens cyclin B3 (CCNB3), transcript variant 3, mRNA [NM_033031]	85417

A_24_P278747	-2.5	1.0	1.0	1.1	CCND2	Homo sapiens cyclin D2 (CCND2), mRNA [NM_001759]	894
A_23_P139881	2.3	-1.3	2.3	2.2	CCND2	Homo sapiens cyclin D2 (CCND2), mRNA [NM_001759]	894
A_23_P361773	-1.1	2.4	-1.8	2.5	CCND3	Homo sapiens cyclin D3 (CCND3), transcript variant 2, mRNA [NM_001760]	896
A_23_P214464	2.1	6.1	2.9	1.4	CCND3	Homo sapiens cyclin D3 (CCND3), transcript variant 2, mRNA [NM_001760]	896
A_23_P209200	-4.3	-1.4	-1.0	-4.4	CCNE1	Homo sapiens cyclin E1 (CCNE1), mRNA [NM_001238]	898
A_23_P215976	1.1	1.1	1.0	2.1	CCNE2	Homo sapiens cyclin E2 (CCNE2), mRNA [NM_057749]	9134

A_23_P58606	2.1	-2.3	1.2	1.1	CCNG1	Homo sapiens cyclin G1 (CCNG1), transcript variant 1, mRNA [NM_004060]	900
A_24_P80532	1.0	-4.9	1.4	2.3	CCNG2	Homo sapiens cyclin G2 (CCNG2), mRNA [NM_004354]	901
A_24_P12065	2.7	2.6	2.5	2.4	CCNG2	Homo sapiens cyclin G2 (CCNG2), mRNA [NM_004354]	901
A_24_P140621	1.0	-1.4	1.4	-2.3	CCNJL	Homo sapiens cyclin J-like (CCNJL), mRNA [NM_024565]	79616
A_24_P348925	1.0	-1.0	-1.0	-4.6	CCNK	Homo sapiens cyclin K (CCNK), mRNA [NM_001099402]	8812
A_23_P92860	-1.4	-1.5	-1.6	-4.7	CCNO	Homo sapiens cyclin O (CCNO), mRNA [NM_021147]	10309

A_24_P246397	1.9	1.4	2.7	-1.2	CCNO	Homo sapiens cyclin O (CCNO), mRNA [NM_021147]	10309
A_23_P87575	1.2	2.9	4.0	-1.1	CCNT1	Homo sapiens cyclin T1 (CCNT1), mRNA [NM_001240]	904
A_24_P174341	-2.0	-2.1	-2.1	-1.8	CCNT2	Homo sapiens cyclin T2 (CCNT2), transcript variant b, mRNA [NM_058241]	905
A_24_P659113	1.1	1.0	1.0	-12.2	CCNYL1	Homo sapiens cyclin Y-like 1 (CCNYL1), transcript variant 2, mRNA [NM_152523]	151195
A_23_P26501	1.1	-18.7	-1.1	-1.0	CCP110	Homo sapiens centriolar coiled coil protein 110kDa (CCP110), transcript variant 2, mRNA [NM_014711]	9738

A_23_P48897	1.0	-1.2	-1.1	-2.6	CCPG1	Homo sapiens cell cycle progression restoration 8 protein (CPR8) mRNA, complete cds. [AF011794]	9236
A_23_P6849	-1.0	-41.0	-1.1	-1.1	CCR1	Homo sapiens chemokine (C-C motif) receptor 1 (CCR1), mRNA [NM_001295]	1230
A_23_P141367	1.1	-1.9	-4.3	-1.4	CCR10	Homo sapiens chemokine (C-C motif) receptor 10 (CCR10), mRNA [NM_016602]	2826
A_23_P250302	-1.3	-3.2	-2.3	-1.5	CCR3	Homo sapiens chemokine (C-C motif) receptor 3 (CCR3), transcript variant 1, mRNA [NM_001837]	1232

A_23_P72989	-2.8	1.0	1.0	-1.0	CCR4	Homo sapiens chemokine (C-C motif) receptor 4 (CCR4), mRNA [NM_005508]	1233
A_23_P343398	-1.0	4.0	1.3	1.0	CCR7	Homo sapiens chemokine (C-C motif) receptor 7 (CCR7), mRNA [NM_001838]	1236
A_23_P69012	-5.5	-1.0	-1.0	1.0	CCR8	Homo sapiens chemokine (C-C motif) receptor 8 (CCR8), mRNA [NM_005201]	1237
A_23_P69310	-1.2	-1.3	-1.3	-2.1	CCRL2	Homo sapiens chemokine (C-C motif) receptor-like 2 (CCRL2), transcript variant 1, mRNA [NM_003965]	9034

A_24_P97405	1.0	1.0	-2.5	1.0	CCRL2	Homo sapiens chemokine (C-C motif) receptor-like 2 (CCRL2), transcript variant 1, mRNA [NM_003965]	9034
A_23_P127475	1.6	2.3	1.8	3.2	CCS	Homo sapiens copper chaperone for superoxide dismutase (CCS), mRNA [NM_005125]	9973
A_23_P102420	2.3	1.6	2.2	1.3	CCT4	Homo sapiens chaperonin containing TCP1, subunit 4 (delta) (CCT4), mRNA [NM_006430]	10575

A_24_P328872	-1.6	-1.6	-1.7	-2.8	CCT5	Homo sapiens chaperonin containing TCP1, subunit 5 (epsilon) (CCT5), mRNA [NM_012073]	22948
A_23_P102404	4.6	4.6	8.0	6.9	CCT7	Homo sapiens chaperonin containing TCP1, subunit 7 (eta) (CCT7), transcript variant 1, mRNA [NM_006429]	10574
A_24_P936671	1.1	1.0	-68.4	-1.0	CCT8	Homo sapiens chaperonin containing TCP1, subunit 8 (theta), mRNA (cDNA clone IMAGE:3930332). [BC005220]	10694

A_24_P131066	2.9	1.0	-1.0	1.1	CD101	Homo sapiens CD101 molecule (CD101), mRNA [NM_004258]	9398
A_23_P354341	1.4	1.3	-3.4	-1.0	CD160	Homo sapiens CD160 molecule (CD160), mRNA [NM_007053]	11126
A_23_P61466	1.0	-2.7	-1.6	-1.0	CD163L1	Homo sapiens CD163 molecule-like 1 (CD163L1), mRNA [NM_174941]	283316
A_23_P254756	2.0	1.9	-1.0	1.9	CD164	Homo sapiens CD164 molecule, sialomucin (CD164), transcript variant 1, mRNA [NM_006016]	8763
A_23_P259863	5.8	-1.3	1.1	1.1	CD177	Homo sapiens CD177 molecule (CD177), mRNA [NM_020406]	57126

A_23_P259868	1.0	1.3	1.2	7.5	CD177	Homo sapiens CD177 molecule (CD177), mRNA [NM_020406]	57126
A_23_P113572	1.1	1.0	2.7	-5.1	CD19	Homo sapiens CD19 molecule (CD19), transcript variant 2, mRNA [NM_001770]	930
A_24_P11208	-6.2	-1.0	1.4	-1.1	CD1D	Homo sapiens CD1d molecule (CD1D), mRNA [NM_001766]	912
A_23_P74575	-1.0	-1.1	-31.9	-1.3	CD1D	Homo sapiens CD1d molecule (CD1D), mRNA [NM_001766]	912
A_23_P201160	-5.7	1.0	-1.1	-1.1	CD1E	Homo sapiens CD1e molecule (CD1E), transcript variant 1, mRNA [NM_030893]	913

A_23_P161076	2.2	1.0	1.2	-1.0	CD2	Homo sapiens CD2 molecule (CD2), mRNA [NM_001767]	914
A_23_P41217	1.4	1.0	-2.0	2.4	CD200R1	Homo sapiens CD200 receptor 1 (CD200R1), transcript variant 1, mRNA [NM_138806]	131450
A_23_P351286	-6.0	-3.7	-2.7	-4.9	CD22	Homo sapiens cDNA: FLJ22814 fis, clone KAIA3004. [AK026467]	933
A_23_P85453	-1.0	-1.0	-1.1	-29.3	CD244	Homo sapiens CD244 molecule, natural killer cell receptor 2B4 (CD244), transcript variant 1, mRNA [NM_016382]	51744

A_24_P105332	1.9	1.0	3.2	1.1	CD244	Homo sapiens CD244 molecule, natural killer cell receptor 2B4 (CD244), transcript variant 1, mRNA [NM_016382]	51744
A_23_P52697	-2.8	1.1	1.2	-2.1	CD248	Homo sapiens CD248 molecule, endosialin (CD248), mRNA [NM_020404]	57124
A_23_P91095	5.5	5.3	5.1	5.3	CD28	Homo sapiens CD28 molecule (CD28), transcript variant 1, mRNA [NM_006139]	940
A_24_P329152	3.7	1.3	1.5	-3.3	CD2AP	Homo sapiens CD2-associated protein (CD2AP), mRNA [NM_012120]	23607

A_32_P155811	1.1	-2.3	-1.0	-1.0	CD2AP	Homo sapiens CD2-associated protein (CD2AP), mRNA [NM_012120]	23607
A_24_P236522	-3.4	1.1	1.0	1.0	CD2BP2	Homo sapiens CD2 (cytoplasmic tail) binding protein 2 (CD2BP2), transcript variant 2, mRNA [NM_001243646]	10421
A_23_P61551	15.1	7.2	7.7	12.8	CD2BP2	Homo sapiens CD2 (cytoplasmic tail) binding protein 2 (CD2BP2), transcript variant 1, mRNA [NM_006110]	10421
A_23_P26771	1.6	1.8	3.3	1.9	CD300C	Homo sapiens CD300c molecule (CD300C), mRNA [NM_006678]	10871

A_32_P201564	1.1	1.1	-2.2	-1.0	CD300E	Homo sapiens CD300e molecule (CD300E), mRNA [NM_181449]	342510
A_23_P15369	1.5	2.9	1.8	1.2	CD300LB	Homo sapiens CD300 molecule-like family member b (CD300LB), mRNA [NM_174892]	124599
A_24_P279307	1.0	-2.2	-1.1	-1.5	CD300LF	Homo sapiens CD300 molecule-like family member f (CD300LF), mRNA [NM_139018]	146722
A_23_P38304	1.7	3.3	1.3	1.5	CD300LG	Homo sapiens CD300 molecule-like family member g (CD300LG), transcript variant 1, mRNA [NM_145273]	146894

A_24_P301655	-1.0	-1.0	-2.3	-1.0	CD33	Homo sapiens CD33 molecule (CD33), transcript variant 1, mRNA [NM_001772]	945
A_23_P111583	-1.7	2.0	1.7	-1.2	CD36	Homo sapiens CD36 molecule (thrombospondin receptor) (CD36), transcript variant 2, mRNA [NM_001001547]	948
A_24_P82749	2.2	-3.7	2.5	-1.0	CD37	Homo sapiens CD37 molecule (CD37), transcript variant 1, mRNA [NM_001774]	951
A_23_P416747	1.3	1.1	2.7	2.2	CD3E	Homo sapiens CD3e molecule, epsilon (CD3-TCR complex) (CD3E), mRNA [NM_000733]	916

A_24_P86968	1.4	2.0	1.4	2.2	CD3E	Homo sapiens CD3e molecule, epsilon (CD3-TCR complex) (CD3E), mRNA [NM_000733]	916
A_24_P267452	-5.6	-1.3	-1.1	-2.3	CD3EAP	Homo sapiens CD3e molecule, epsilon associated protein (CD3EAP), mRNA [NM_012099]	10849
A_24_P295999	2.8	2.3	1.0	1.3	CD4	Homo sapiens CD4 molecule (CD4), transcript variant 1, mRNA [NM_000616]	920
A_23_P62220	-1.0	-1.1	-6.7	-1.3	CD40LG	Homo sapiens CD40 ligand (CD40LG), mRNA [NM_000074]	959
A_32_P175934	1.1	1.0	-1.0	-5.0	CD48	Homo sapiens CD48 molecule (CD48), mRNA [NM_001778]	962

A_23_P85800	1.7	2.8	2.6	-1.2	CD52	Homo sapiens CD52 molecule (CD52), mRNA [NM_001803]	1043
A_23_P312008	2.0	1.3	-2.9	1.2	CD55	CD55 molecule, decay accelerating factor for complement (Cromer blood group) [Source:HGNC Symbol;Acc:2665] [ENST00000465534]	1604
A_24_P188377	1.0	-1.0	-2.8	-2.6	CD55	Homo sapiens CD55 molecule, decay accelerating factor for complement (Cromer blood group) (CD55), transcript variant 1, mRNA [NM_000574]	1604

A_23_P138308	1.8	4.7	2.0	2.7	CD58	Homo sapiens CD58 molecule (CD58), transcript variant 1, mRNA [NM_001779]	965
A_24_P141481	1.0	-1.0	-1.1	2.6	CD59	Homo sapiens CD59 molecule, complement regulatory protein (CD59), transcript variant 1, mRNA [NM_203330]	966
A_23_P139162	-1.8	1.1	-2.6	1.0	CD6	Homo sapiens CD6 molecule (CD6), mRNA [NM_006725]	923
A_24_P270144	2.0	2.1	1.4	-1.8	CD63	Homo sapiens CD63 molecule (CD63), transcript variant 2, mRNA [NM_001040034]	967

A_23_P15394	-5.2	1.0	-1.0	-2.2	CD68	Homo sapiens CD68 molecule (CD68), transcript variant 1, mRNA [NM_001251]	968
A_23_P70095	-1.2	-2.2	1.2	1.0	CD74	Homo sapiens CD74 molecule, major histocompatibility complex, class II invariant chain (CD74), transcript variant 3, mRNA [NM_001025158]	972
A_23_P155632	-6.9	-1.6	-1.7	-1.7	CD80	Homo sapiens CD80 molecule (CD80), mRNA [NM_005191]	941
A_23_P13425	1.1	1.1	2.5	2.2	CD81	Homo sapiens CD81 molecule (CD81), mRNA [NM_004356]	975

A_23_P1782	3.3	2.5	-10.7	1.8	CD82	Homo sapiens CD82 molecule (CD82), transcript variant 1, mRNA [NM_002231]	3732
A_32_P58215	1.1	2.2	2.2	1.1	CD84	Homo sapiens CD84 molecule (CD84), transcript variant 1, mRNA [NM_001184879]	8832
A_23_P159335	-3.6	1.9	-2.5	-1.5	CD8B	Homo sapiens CD8b molecule (CD8B), transcript variant 5, mRNA [NM_004931]	926
A_23_P502312	1.4	-4.4	2.0	1.1	CD97	Homo sapiens CD97 molecule (CD97), transcript variant 1, mRNA [NM_078481]	976

A_23_P502314	2.3	2.9	-1.1	1.6	CD97	Homo sapiens CD97 molecule (CD97), transcript variant 1, mRNA [NM_078481]	976
A_24_P70993	-6.1	1.0	-1.1	-1.1	CD99	Homo sapiens CD99 molecule (CD99), transcript variant 1, mRNA [NM_002414]	4267
A_24_P68222	1.1	1.1	-2.0	-1.0	CD99P1	Homo sapiens CD99 molecule pseudogene 1 (CD99P1), transcript variant 2, non-coding RNA [NR_033381]	401577
A_23_P34597	5.0	4.8	1.4	2.6	CDA	Homo sapiens cytidine deaminase (CDA), mRNA [NM_001785]	978

A_24_P177763	-25.9	-1.5	-2.4	-1.7	CDADC1	Homo sapiens cytidine and dCMP deaminase domain containing 1 (CDADC1), transcript variant 1, mRNA [NM_030911]	81602
A_23_P20622	-1.0	-1.3	-2.4	-1.7	CDC14B	Homo sapiens CDC14 cell division cycle 14 homolog B (S. cerevisiae) (CDC14B), transcript variant 2, mRNA [NM_033331]	8555
A_23_P367043	-3.8	-1.6	-1.7	-2.5	CDC14C	Homo sapiens CDC14 cell division cycle 14 homolog C (S. cerevisiae) (CDC14C), non-coding RNA [NR_003595]	168448

A_23_P149200	1.0	2.6	2.3	2.2	CDC20	Homo sapiens cell division cycle 20 homolog (S. cerevisiae) (CDC20), mRNA [NM_001255]	991
A_24_P913227	-1.0	-2.7	-1.1	-2.4	CDC23	cell division cycle 23 homolog (S. cerevisiae) [Source:HGNC Symbol;Acc:1724] [ENST00000394884]	8697
A_23_P70249	-1.0	-1.0	-2.9	1.0	CDC25C	Homo sapiens cell division cycle 25 homolog C (S. pombe) (CDC25C), transcript variant 1, mRNA [NM_001790]	995

A_32_P228501	-2.1	1.2	-1.0	1.1	CDC26	Homo sapiens cell division cycle 26 homolog (S. cerevisiae) (CDC26), mRNA [NM_139286]	246184
A_24_P177025	2.0	2.7	4.2	1.7	CDC34	Homo sapiens cell division cycle 34 homolog (S. cerevisiae) (CDC34), mRNA [NM_004359]	997
A_24_P269624	1.9	1.1	-1.0	-6.5	CDC37L1	cell division cycle 37 homolog (S. cerevisiae)-like 1 [Source:HGNC Symbol;Acc:17179] [ENST00000381854]	55664

A_23_P351320	2.4	1.2	1.9	3.0	CDC40	Homo sapiens cell division cycle 40 homolog (S. cerevisiae) (CDC40), mRNA [NM_015891]	51362
A_23_P200560	-1.1	-1.2	-2.7	-1.4	CDC42	Homo sapiens cell division cycle 42 (GTP binding protein, 25kDa) (CDC42), transcript variant 3, mRNA [NM_001039802]	998
A_32_P115015	-2.4	-2.1	-4.4	-2.9	CDC42	Homo sapiens cell division cycle 42 (GTP binding protein, 25kDa) (CDC42), transcript variant 3, mRNA [NM_001039802]	998

A_24_P249626	-4.0	-1.4	1.5	-1.4	CDC42BPG	Homo sapiens CDC42 binding protein kinase gamma (DMPK-like) (CDC42BPG), mRNA [NM_017525]	55561
A_24_P143032	1.8	-3.7	-1.5	-1.5	CDC42EP1	Homo sapiens CDC42 effector protein (Rho GTPase binding) 1 (CDC42EP1), mRNA [NM_152243]	11135
A_23_P166453	1.1	2.4	3.6	1.6	CDC42EP1	Homo sapiens CDC42 effector protein (Rho GTPase binding) 1 (CDC42EP1), mRNA [NM_152243]	11135

A_23_P1602	1.9	1.9	2.7	1.9	CDC42EP2	Homo sapiens CDC42 effector protein (Rho GTPase binding) 2 (CDC42EP2), mRNA [NM_006779]	10435
A_24_P924697	-1.0	-3.0	-2.0	-14.0	CDC42EP3	CDC42 effector protein (Rho GTPase binding) 3 [Source:HGNC Symbol;Acc:16943] [ENST00000295324]	10602
A_23_P209636	1.0	1.0	-1.0	2.3	CDC42EP3	Homo sapiens CDC42 effector protein (Rho GTPase binding) 3 (CDC42EP3), mRNA [NM_006449]	10602

A_24_P19054	2.0	1.7	1.8	1.5	CDC42EP4	Homo sapiens CDC42 effector protein (Rho GTPase binding) 4 (CDC42EP4), mRNA [NM_012121]	23580
A_24_P44916	1.1	2.2	-1.6	2.0	CDC42EP5	Homo sapiens CDC42 effector protein (Rho GTPase binding) 5 (CDC42EP5), mRNA [NM_145057]	148170
A_24_P409042	1.1	1.4	-3.0	1.2	CDC42SE2	Homo sapiens CDC42 small effector 2 (CDC42SE2), transcript variant 1, mRNA [NM_020240]	56990
A_23_P162476	-2.5	-1.0	1.0	-1.6	CDCA3	Homo sapiens cell division cycle associated 3 (CDCA3), mRNA [NM_031299]	83461

A_24_P218979	-1.9	-2.2	-1.5	1.1	CDCA3	Homo sapiens cell division cycle associated 3 (CDCA3), mRNA [NM_031299]	83461
A_24_P133488	1.0	-4.8	-1.0	1.0	CDCA4	Homo sapiens cell division cycle associated 4 (CDCA4), transcript variant 1, mRNA [NM_017955]	55038
A_24_P171549	1.1	-3.1	-5.0	-2.3	CDCA7	Homo sapiens cell division cycle associated 7 (CDCA7), transcript variant 1, mRNA [NM_031942]	83879

A_24_P274795	-1.7	-1.9	-1.6	-10.3	CDCA7L	Homo sapiens cell division cycle associated 7-like (CDCA7L), transcript variant 1, mRNA [NM_018719]	55536
A_23_P32474	1.1	1.1	1.0	-6.8	CDCP1	Homo sapiens CUB domain containing protein 1 (CDCP1), transcript variant 1, mRNA [NM_022842]	64866
A_24_P357997	1.1	-2.1	-1.1	-1.0	CDCP2	Homo sapiens CUB domain containing protein 2 (CDCP2), mRNA [NM_201546]	200008
A_23_P152305	2.1	2.0	1.9	1.9	CDH11	Homo sapiens cadherin 11, type 2, OB-cadherin (osteoblast) (CDH11), mRNA [NM_001797]	1009

A_32_P10936	2.7	2.3	1.4	-3.7	CDH12	Homo sapiens cadherin 12, type 2 (N-cadherin 2) (CDH12), mRNA [NM_004061]	1010
A_32_P25357	2.7	-1.1	1.8	-1.1	CDH15	Homo sapiens cadherin 15, type 1, M-cadherin (myotubule) (CDH15), mRNA [NM_004933]	1013
A_23_P100240	1.0	-2.8	1.5	-1.1	CDH16	Homo sapiens cadherin 16, KSP-cadherin (CDH16), transcript variant 1, mRNA [NM_004062]	1014
A_23_P38735	-3.9	1.1	-3.5	-1.2	CDH19	Homo sapiens cadherin 19, type 2 (CDH19), mRNA [NM_021153]	28513

A_23_P55586	1.2	1.2	1.2	-3.0	CDH20	Homo sapiens cadherin 20, type 2 (CDH20), mRNA [NM_031891]	28316
A_23_P40192	-1.3	1.5	-5.3	1.2	CDH22	Homo sapiens cadherin 22, type 2 (CDH22), mRNA [NM_021248]	64405
A_23_P307968	1.4	1.1	-1.0	-2.8	CDH23	Homo sapiens cadherin-related 23 (CDH23), transcript variant 2, mRNA [NM_052836]	64072
A_23_P405267	-3.0	1.0	1.0	1.0	CDH24	Homo sapiens cDNA FLJ25193 fis, clone JTH00761. [AK057922]	64403
A_24_P113458	1.1	-6.0	-2.7	1.0	CDH26	cadherin 26 [Source:HGNC Symbol;Acc:15902] [ENST00000244049]	60437

A_23_P49155	2.1	1.3	1.9	1.3	CDH3	Homo sapiens cadherin 3, type 1, P-cadherin (placental) (CDH3), mRNA [NM_001793]	1001
A_24_P940656	-2.6	1.0	-1.0	-2.2	CDH4	GB	1002
A_23_P28999	1.1	1.2	-1.3	2.1	CDH4	Homo sapiens cadherin 4, type 1, R-cadherin (retinal) (CDH4), transcript variant 1, mRNA [NM_001794]	1002
A_23_P214011	-3.3	1.4	2.5	1.0	CDH6	Homo sapiens cadherin 6, type 2, K-cadherin (fetal kidney) (CDH6), mRNA [NM_004932]	1004
A_24_P382287	2.4	2.8	4.0	1.4	CDH7	Homo sapiens cadherin 7, type 2 (CDH7), transcript variant b, mRNA [NM_004361]	1005

A_23_P140797	1.4	1.4	2.9	1.1	CDH8	Homo sapiens cadherin 8, type 2 (CDH8), mRNA [NM_001796]	1006
A_23_P92999	2.9	2.9	1.3	2.4	CDH9	Homo sapiens cadherin 9, type 2 (T1-cadherin) (CDH9), mRNA [NM_016279]	1007
A_24_P358805	4.3	4.3	4.3	2.2	CDHR1	Homo sapiens cadherin-related family member 1 (CDHR1), transcript variant 2, mRNA [NM_001171971]	92211
A_23_P149946	-1.0	-5.7	1.4	-1.0	CDHR1	Homo sapiens cadherin-related family member 1 (CDHR1), transcript variant 1, mRNA [NM_033100]	92211

A_24_P16143	-1.5	-1.9	-2.2	-2.2	CDHR4	Homo sapiens cadherin-related family member 4 (CDHR4), mRNA [NM_001007540]	389118
A_24_P65199	2.8	1.1	-1.2	1.2	CDK10	Homo sapiens cyclin-dependent kinase 10 (CDK10), transcript variant b, mRNA [NM_052987]	8558
A_23_P152245	2.5	1.9	1.4	2.3	CDK10	Homo sapiens cyclin-dependent kinase 10 (CDK10), transcript variant b, mRNA [NM_052987]	8558
A_24_P312915	-1.5	-1.4	-4.6	-1.4	CDK11B	Homo sapiens cyclin-dependent kinase 11B (CDK11B), transcript variant 5, mRNA [NM_033489]	984

A_24_P355772	-1.0	-1.0	-2.9	-1.0	CDK11B	Homo sapiens cyclin-dependent kinase 11B (CDK11B), transcript variant 5, mRNA [NM_033489]	984
A_23_P217804	-2.4	-1.9	-2.5	-1.9	CDK11B	Homo sapiens cyclin-dependent kinase 11B (CDK11B), transcript variant 3, mRNA [NM_033487]	984
A_24_P198820	1.0	-1.7	-2.6	1.0	CDK12	Homo sapiens cyclin-dependent kinase 12 (CDK12), transcript variant 1, mRNA [NM_016507]	51755
A_23_P159119	-2.3	1.7	1.2	1.3	CDK12	Homo sapiens cyclin-dependent kinase 12 (CDK12), transcript variant 1, mRNA [NM_016507]	51755

A_23_P154050	1.3	1.3	1.3	-3.8	CDK15	Homo sapiens cyclin-dependent kinase 15 (CDK15), mRNA [NM_139158]	65061
A_24_P233663	-1.2	-1.4	-1.4	-23.2	CDK16	Homo sapiens cyclin-dependent kinase 16 (CDK16), transcript variant 2, mRNA [NM_033018]	5127
A_23_P33376	3.6	5.2	2.3	7.6	CDK17	Homo sapiens cyclin-dependent kinase 17 (CDK17), transcript variant 1, mRNA [NM_002595]	5128
A_24_P699896	1.0	-4.3	-1.7	-1.1	CDK19	cyclin-dependent kinase 19 [Source:HGNC Symbol;Acc:19338] [ENST00000497709]	23097

A_24_P343289	1.7	1.5	-1.5	2.9	CDK19	Homo sapiens cyclin-dependent kinase 19 (CDK19), mRNA [NM_015076]	23097
A_23_P104579	1.4	1.3	-1.4	2.8	CDK2AP2	Homo sapiens cyclin-dependent kinase 2 associated protein 2 (CDK2AP2), mRNA [NM_005851]	10263
A_23_P24997	3.1	3.0	1.1	8.4	CDK4	Homo sapiens cyclin-dependent kinase 4 (CDK4), mRNA [NM_000075]	1019
A_23_P141467	-1.3	2.3	1.2	1.9	CDK5R1	Homo sapiens cyclin-dependent kinase 5, regulatory subunit 1 (p35) (CDK5R1), mRNA [NM_003885]	8851

A_23_P79323	2.8	-2.8	1.1	2.2	CDK5R2	Homo sapiens cyclin-dependent kinase 5, regulatory subunit 2 (p39) (CDK5R2), mRNA [NM_003936]	8941
A_24_P51683	-1.1	1.1	8.6	1.1	CDK5R2	Homo sapiens cyclin-dependent kinase 5, regulatory subunit 2 (p39) (CDK5R2), mRNA [NM_003936]	8941
A_23_P83110	-5.6	-5.1	-5.2	-1.5	CDK5RAP2	Homo sapiens CDK5 regulatory subunit associated protein 2 (CDK5RAP2), transcript variant 1, mRNA [NM_018249]	55755

A_24_P292964	2.2	2.2	2.0	-1.1	CDK5RAP3	Homo sapiens CDK5 regulatory subunit associated protein 3 (CDK5RAP3), mRNA [NM_176096]	80279
A_23_P44781	-1.7	-2.7	-1.4	1.3	CDKAL1	Homo sapiens CDK5 regulatory subunit associated protein 1-like 1 (CDKAL1), mRNA [NM_017774]	54901
A_23_P110643	2.5	1.5	1.3	3.0	CDKL3	Homo sapiens cyclin-dependent kinase-like 3 (CDKL3), transcript variant 2, mRNA [NM_016508]	51265
A_23_P204696	1.0	-4.8	-1.0	-67.0	CDKN1B	Homo sapiens cyclin-dependent kinase inhibitor 1B (p27, Kip1) (CDKN1B), mRNA [NM_004064]	1027

A_23_P43490	1.5	-1.2	-4.2	-2.0	CDKN2A	Homo sapiens cyclin-dependent kinase inhibitor 2A (melanoma, p16, inhibits CDK4) (CDKN2A), transcript variant 3, mRNA [NM_058197]	1029
A_23_P258002	1.1	-2.7	-2.9	-1.0	CDKN2AIP	Homo sapiens CDKN2A interacting protein (CDKN2AIP), mRNA [NM_017632]	55602
A_24_P360674	-2.5	1.3	1.3	-1.0	CDKN2B	Homo sapiens cyclin-dependent kinase inhibitor 2B (p15, inhibits CDK4) (CDKN2B), transcript variant 2, mRNA [NM_078487]	1030

A_23_P14957	1.5	2.3	1.3	1.5	CDR2	Homo sapiens cerebellar degeneration-related protein 2, 62kDa (CDR2), mRNA [NM_001802]	1039
A_24_P339071	1.2	-2.7	1.2	1.2	CDR2	Homo sapiens cerebellar degeneration-related protein 2, 62kDa (CDR2), mRNA [NM_001802]	1039
A_23_P352426	1.2	1.1	-3.2	-1.0	CDRT1	Homo sapiens CMT1A duplicated region transcript 1 (CDRT1), mRNA [NM_006382]	374286
A_24_P756766	-153.4	-1.7	-1.8	-1.7	CDRT15	Homo sapiens CMT1A duplicated region transcript 15 (CDRT15), mRNA [NM_001007530]	146822

A_23_P154698	1.6	2.3	-2.8	1.5	CDS2	Homo sapiens CDP-diacylglycerol synthase (phosphatidate cytidylyltransferase) 2 (CDS2), mRNA [NM_003818]	8760
A_23_P70520	1.6	2.4	-1.4	1.5	CDSN	Homo sapiens corneodesmosin (CDSN), mRNA [NM_001264]	1041
A_23_P259413	2.9	1.7	1.7	2.1	CDV3	Homo sapiens CDV3 homolog (mouse) (CDV3), transcript variant 2, mRNA [NM_017548]	55573

A_23_P55738	14.8	25.4	15.6	2.5	CEACAM1	Homo sapiens carcinoembryonic antigen-related cell adhesion molecule 1 (biliary glycoprotein) (CEACAM1), transcript variant 2, mRNA [NM_001024912]	634
A_32_P53234	1.5	-1.1	-1.1	-2.5	CEACAM21	carcinoembryonic antigen-related cell adhesion molecule 21 [Source:HGNC Symbol;Acc:28834] [ENST00000487420]	90273

A_23_P78595	-1.0	-4.3	-1.0	1.4	CEACAM21	Homo sapiens carcinoembryonic antigen-related cell adhesion molecule 21 (CEACAM21), transcript variant 2, mRNA [NM_033543]	90273
A_23_P130515	2.6	1.4	2.5	2.4	CEACAM3	Homo sapiens carcinoembryonic antigen-related cell adhesion molecule 3 (CEACAM3), mRNA [NM_001815]	1084
A_23_P153301	6.1	6.0	1.2	6.0	CEACAM5	Homo sapiens carcinoembryonic antigen-related cell adhesion molecule 5 (CEACAM5), mRNA [NM_004363]	1048

A_23_P421483	1.0	-1.0	1.3	-10.8	CEACAM6	Homo sapiens carcinoembryonic antigen-related cell adhesion molecule 6 (non-specific cross reacting antigen), mRNA (cDNA clone MGC:10467 IMAGE:3640231), complete cds. [BC005008]	4680
A_24_P228302	-1.2	1.4	2.2	1.4	CEACAM7	Homo sapiens carcinoembryonic antigen-related cell adhesion molecule 7 (CEACAM7), mRNA [NM_006890]	1087

A_23_P380240	1.3	-1.9	-2.2	-1.3	CEACAM8	Homo sapiens carcinoembryonic antigen-related cell adhesion molecule 8 (CEACAM8), mRNA [NM_001816]	1088
A_23_P375494	1.8	1.7	1.6	3.6	CEBPA	Homo sapiens CCAAT/enhancer binding protein (C/EBP), alpha (CEBPA), mRNA [NM_004364]	1050
A_24_P224727	-1.6	-2.9	-1.0	-1.0	CEBPA	Homo sapiens CCAAT/enhancer binding protein (C/EBP), alpha (CEBPA), mRNA [NM_004364]	1050

A_24_P216165	1.8	1.5	-1.8	2.4	CEBPA	Homo sapiens CCAAT/enhancer binding protein (C/EBP), alpha (CEBPA), mRNA [NM_004364]	1050
A_23_P2990	4.2	7.2	6.8	1.1	CEBPE	Homo sapiens CCAAT/enhancer binding protein (C/EBP), epsilon (CEBPE), mRNA [NM_001805]	1053
A_23_P68773	-1.3	6.2	1.3	-1.4	CECR1	Homo sapiens cat eye syndrome chromosome region, candidate 1 (CECR1), transcript variant 1, mRNA [NM_017424]	51816

A_23_P211326	1.8	-2.7	1.2	1.6	CECR2	Homo sapiens cat eye syndrome chromosome region, candidate 2 (CECR2), mRNA [NM_031413]	27443
A_24_P403501	1.4	-10.3	1.8	1.5	CECR7	Homo sapiens cat eye syndrome chromosome region, candidate 7, mRNA (cDNA clone IMAGE:5289002). [BC043198]	100130418
A_23_P169503	-3.1	1.2	-2.0	-3.5	CEL	Homo sapiens carboxyl ester lipase (bile salt-stimulated lipase) (CEL), mRNA [NM_001807]	1056

A_23_P123794	1.0	1.6	-3.2	1.1	CEL	Homo sapiens carboxyl ester lipase (bile salt-stimulated lipase) (CEL), mRNA [NM_001807]	1056
A_23_P46238	2.1	1.1	2.0	1.0	CELA2A	Homo sapiens chymotrypsin-like elastase family, member 2A (CELA2A), mRNA [NM_033440]	63036
A_23_P200579	-1.0	-2.8	-1.1	-1.0	CELA3B	Homo sapiens chymotrypsin-like elastase family, member 3B (CELA3B), mRNA [NM_007352]	23436
A_23_P318396	-1.9	-1.4	-1.7	-2.4	CELF1	Homo sapiens CUGBP, Elav-like family member 1 (CELF1), transcript variant 2, mRNA [NM_198700]	10658

A_23_P337849	1.1	-1.2	-6.7	-1.0	CELF3	Homo sapiens CUGBP, Elav-like family member 3 (CELF3), transcript variant 1, mRNA [NM_007185]	11189
A_24_P933572	-1.5	-6.9	1.7	1.0	CELF4	Homo sapiens cDNA FLJ34375 fis, clone FEBRA2017533. [AK091694]	56853
A_24_P182620	1.1	1.1	-4.4	1.1	CELSR2	Homo sapiens cadherin, EGF LAG seven-pass G-type receptor 2 (flamingo homolog, Drosophila) (CELSR2), mRNA [NM_001408]	1952

A_23_P201570	1.9	1.4	3.2	1.6	CELSR2	Homo sapiens cadherin, EGF LAG seven-pass G-type receptor 2 (flamingo homolog, Drosophila) (CELSR2), mRNA [NM_001408]	1952
A_24_P312845	2.3	1.5	-1.2	1.3	CEND1	Homo sapiens cell cycle exit and neuronal differentiation 1 (CEND1), mRNA [NM_016564]	51286
A_23_P257905	2.0	1.6	-1.4	2.4	CENPB	Homo sapiens centromere protein B, 80kDa (CENPB), mRNA [NM_001810]	1059
A_23_P206454	1.8	2.7	3.4	2.0	CENPBD1	Homo sapiens CENPB DNA-binding domains containing 1 (CENPBD1), mRNA [NM_145039]	92806

A_23_P252292	-4.5	1.2	1.2	1.1	CENPI	Homo sapiens centromere protein I (CENPI), mRNA [NM_006733]	2491
A_24_P298996	-3.1	-1.2	-2.8	-1.2	CENPJ	Homo sapiens centromere protein J (CENPJ), mRNA [NM_018451]	55835
A_24_P930100	-1.0	-2.8	-1.1	-1.0	CENPL	centromere protein L [Source:HGNC Symbol;Acc:17879] [ENST00000495275]	91687
A_24_P392109	1.0	-1.0	-4.9	-1.1	CENPN	Homo sapiens centromere protein N (CENPN), transcript variant 3, mRNA [NM_018455]	55839
A_32_P119174	-12.0	1.0	-1.0	-1.0	CENPP	Homo sapiens centromere protein P (CENPP), mRNA [NM_001012267]	401541

A_23_P313828	-2.0	-1.6	-1.1	2.0	CENPV	Homo sapiens centromere protein V (CENPV), mRNA [NM_181716]	201161
A_23_P200047	2.0	2.0	2.0	-1.7	CEP104	Homo sapiens centrosomal protein 104kDa (CEP104), mRNA [NM_014704]	9731
A_23_P320829	-2.7	-2.8	-1.0	-1.1	CEP128	Homo sapiens centrosomal protein 128kDa (CEP128), mRNA [NM_152446]	145508
A_23_P18479	1.5	1.5	3.1	1.1	CEP135	centrosomal protein 135kDa [Source:HGNC Symbol;Acc:29086] [ENST00000422247]	9662

A_32_P220519	-1.0	-1.0	-2.9	-1.0	CEP152	Homo sapiens centrosomal protein 152kDa (CEP152), transcript variant 2, mRNA [NM_014985]	22995
A_24_P652700	-2.2	-1.0	-1.1	1.0	CEP152	Homo sapiens centrosomal protein 152kDa (CEP152), transcript variant 2, mRNA [NM_014985]	22995
A_32_P14978	1.0	-1.7	-1.1	-2.2	CEP152	Homo sapiens centrosomal protein 152kDa (CEP152), transcript variant 2, mRNA [NM_014985]	22995
A_24_P389843	-2.0	-1.0	-1.2	-2.0	CEP164	Homo sapiens centrosomal protein 164kDa (CEP164), mRNA [NM_014956]	22897

A_24_P36944	-2.1	-2.1	-2.2	-4.0	CEP170	Homo sapiens centrosomal protein 170kDa (CEP170), transcript variant alpha, mRNA [NM_014812]	9859
A_32_P212058	1.0	-2.9	-1.1	-1.1	CEP19	Homo sapiens centrosomal protein 19kDa (CEP19), mRNA [NM_032898]	84984
A_23_P102832	1.9	2.2	-1.7	3.1	CEP250	Homo sapiens centrosomal protein 250kDa (CEP250), mRNA [NM_007186]	11190
A_24_P51067	-2.6	-2.5	-2.5	-2.3	CEP290	Homo sapiens centrosomal protein 290kDa (CEP290), mRNA [NM_025114]	80184

A_23_P115872	12.2	11.7	11.0	11.0	CEP55	Homo sapiens centrosomal protein 55kDa (CEP55), transcript variant 1, mRNA [NM_018131]	55165
A_23_P86822	-1.5	-1.4	-2.9	-1.5	CEP57	Homo sapiens centrosomal protein 57kDa (CEP57), transcript variant 1, mRNA [NM_014679]	9702
A_24_P189112	-1.0	-1.2	-3.3	-1.0	CEP57L1	Homo sapiens centrosomal protein 57kDa-like 1 (CEP57L1), transcript variant 2, mRNA [NM_173830]	285753

A_23_P59358	1.2	-2.7	1.3	1.1	CEP57L1	Homo sapiens centrosomal protein 57kDa-like 1 (CEP57L1), transcript variant 2, mRNA [NM_173830]	285753
A_23_P21409	-2.4	-10.6	1.4	-1.1	CEP63	Homo sapiens centrosomal protein 63kDa (CEP63), transcript variant 4, mRNA [NM_001042384]	80254
A_23_P210323	1.0	-5.2	-1.1	1.0	CEP68	Homo sapiens centrosomal protein 68kDa (CEP68), mRNA [NM_015147]	23177
A_23_P353436	-1.2	-1.2	-1.8	-4.3	CEP78	centrosomal protein 78kDa [Source:HGNC Symbol;Acc:25740] [ENST00000447629]	84131

A_23_P200874	-2.4	-5.2	-5.3	-7.8	CEP85	Homo sapiens centrosomal protein 85kDa (CEP85), mRNA [NM_022778]	64793
A_24_P920355	4.5	4.5	4.5	4.4	CEP97	centrosomal protein 97kDa [Source:HGNC Symbol;Acc:26244] [ENST00000341893]	79598
A_24_P418294	1.5	1.5	1.4	2.3	CEP97	Homo sapiens centrosomal protein 97kDa (CEP97), mRNA [NM_024548]	79598
A_24_P62237	-1.0	-1.2	-1.1	-2.8	CERK	Homo sapiens ceramide kinase (CERK), mRNA [NM_022766]	64781
A_23_P211659	1.2	-3.6	1.1	-1.0	CERK	Homo sapiens ceramide kinase (CERK), mRNA [NM_022766]	64781

A_24_P943017	28.9	25.3	29.4	26.2	CERS3	Homo sapiens ceramide synthase 3 (CERS3), mRNA [NM_178842]	204219
A_23_P153867	1.7	1.4	-5.1	-1.0	CERS4	Homo sapiens ceramide synthase 4 (CERS4), mRNA [NM_024552]	79603
A_23_P206733	-6.7	1.2	1.0	1.1	CES1	Homo sapiens carboxylesterase 1 (CES1), transcript variant 3, mRNA [NM_001266]	1066
A_23_P49192	-5.2	-1.5	-7.9	-4.0	CES2	Homo sapiens carboxylesterase 2 (CES2), transcript variant 2, mRNA [NM_198061]	8824
A_24_P13790	-2.3	1.0	-1.0	1.0	CES2	Homo sapiens carboxylesterase 2 (CES2), transcript variant 1, mRNA [NM_003869]	8824

A_23_P361544	2.1	2.0	1.9	2.0	CES4A	Homo sapiens carboxylesterase 4A (CES4A), transcript variant 1, mRNA [NM_173815]	283848
A_24_P270674	-2.0	-1.5	-2.8	-3.5	CES5A	Homo sapiens carboxylesterase 5A (CES5A), transcript variant 2, mRNA [NM_145024]	221223
A_23_P118234	1.0	1.1	-1.1	-2.0	CES5A	Homo sapiens carboxylesterase 5A (CES5A), transcript variant 2, mRNA [NM_145024]	221223
A_23_P66967	-4.6	-2.0	-1.3	-1.3	CETN1	Homo sapiens centrin, EF-hand protein, 1 (CETN1), mRNA [NM_004066]	1068

A_23_P119562	-1.6	3.7	2.4	2.8	CFD	Homo sapiens compleme nt factor D (adipsin) (CFD), mRNA [NM_0019 28]	1675
A_23_P89123	-4.0	1.0	-1.5	1.2	CFDP1	Homo sapiens craniofacia l developm ent protein 1 (CFDP1), mRNA [NM_0063 24]	10428
A_23_P23759	1.0	-1.0	-1.0	-2.1	CFHR5	Homo sapiens compleme nt factor H-related 5 (CFHR5), mRNA [NM_0307 87]	81494
A_23_P7212	32.0	31.6	30.4	31.0	CFI	Homo sapiens compleme nt factor I (CFI), mRNA [NM_0002 04]	3426
A_24_P92472	3.6	3.4	3.0	1.1	CFI	Homo sapiens compleme nt factor I (CFI), mRNA [NM_0002 04]	3426

A_23_P395493	3.0	1.6	2.1	1.2	CFL1P1	Homo sapiens cofilin 1 (non-muscle) pseudogene 1 (CFL1P1), non-coding RNA [NR_028492]	142913
A_23_P65401	2.5	2.1	2.1	2.1	CFL2	Homo sapiens cofilin 2 (muscle) (CFL2), transcript variant 1, mRNA [NM_021914]	1073
A_23_P156807	6.0	3.7	3.5	1.4	CFLAR	Homo sapiens CASP8 and FADD-like apoptosis regulator (CFLAR), transcript variant 8, mRNA [NM_001202519]	8837
A_23_P22444	2.5	1.3	1.6	1.7	CFP	Homo sapiens complement factor properdin (CFP), transcript variant 1, mRNA [NM_002621]	5199

A_24_P371782	-3.5	1.3	-1.2	1.3	CGA	Homo sapiens glycoprotein hormones, alpha polypeptide (CGA), transcript variant 2, mRNA [NM_000735]	1081
A_32_P45462	-4.8	-1.6	1.0	-2.7	CGA	Homo sapiens mRNA expressed only in placental villi, clone SMAP84. [AB019569]	1081
A_24_P395814	2.0	1.6	2.1	-1.6	CGB	Homo sapiens chorionic gonadotropin, beta polypeptide (CGB), mRNA [NM_000737]	1082
A_23_P39095	-2.6	-1.8	-1.9	-1.9	CGB1	Homo sapiens chorionic gonadotropin, beta polypeptide 1 (CGB1), mRNA [NM_033377]	114335

A_23_P155276	3.1	2.9	1.7	3.1	CGGBP1	Homo sapiens CGG triplet repeat binding protein 1 (CGGBP1), transcript variant 1, mRNA [NM_001008390]	8545
A_23_P149390	-2.1	-1.2	-1.2	-1.6	CGN	Homo sapiens cingulin (CGN), mRNA [NM_020770]	57530
A_24_P135319	1.4	1.5	2.4	1.3	CGNL1	Homo sapiens cingulin-like 1 (CGNL1), transcript variant 2, mRNA [NM_032866]	84952
A_23_P37275	-1.1	-3.4	-1.2	-1.2	CGRRF1	Homo sapiens cell growth regulator with ring finger domain 1 (CGRRF1), mRNA [NM_006568]	10668

A_23_P37283	1.7	-1.1	2.1	5.0	CGRRF1	Homo sapiens cell growth regulator with ring finger domain 1 (CGRRF1), mRNA [NM_006568]	10668
A_23_P26976	-2.1	6.6	4.5	2.1	CHAD	Homo sapiens chondroadherin (CHAD), mRNA [NM_001267]	1101
A_24_P53519	1.0	1.0	-1.0	-2.3	CHAF1A	Homo sapiens chromatin assembly factor 1, subunit A (p150) (CHAF1A), mRNA [NM_005483]	10036
A_23_P164559	-1.4	-1.4	-34.7	-1.4	CHAMP1	Homo sapiens chromosome alignment maintaining phosphoprotein 1 (CHAMP1), transcript variant 1, mRNA [NM_032436]	283489

A_23_P46894	-2.1	-2.0	-3.0	1.0	CHAT	Homo sapiens choline O-acetyltransferase (CHAT), transcript variant M, mRNA [NM_020549]	1103
A_24_P109511	3.7	1.6	3.1	1.9	CHCHD2	Homo sapiens coiled-coil-helix-coiled-coil-helix domain containing 2 (CHCHD2), nuclear gene encoding mitochondrial protein, mRNA [NM_016139]	51142

A_23_P157405	1.3	1.3	5.4	1.6	CHCHD2	Homo sapiens coiled-coil-helix-coiled-coil-helix domain containing 2 (CHCHD2), nuclear gene encoding mitochondrial protein, mRNA [NM_016139]	51142
A_24_P400376	3.0	3.8	3.6	3.8	CHCHD2	Homo sapiens coiled-coil-helix-coiled-coil-helix domain containing 2 (CHCHD2), nuclear gene encoding mitochondrial protein, mRNA [NM_016139]	51142

A_23_P154282	2.8	1.9	1.5	2.3	CHCHD5	Homo sapiens coiled-coil-helix-coiled-coil-helix domain containing 5 (CHCHD5), mRNA [NM_032309]	84269
A_23_P91870	-1.1	-1.1	-2.4	-1.3	CHCHD6	Homo sapiens coiled-coil-helix-coiled-coil-helix domain containing 6 (CHCHD6), mRNA [NM_032343]	84303
A_23_P170626	1.0	-1.0	-1.1	-3.2	CHCHD7	Homo sapiens coiled-coil-helix-coiled-coil-helix domain containing 7 (CHCHD7), transcript variant 1, mRNA [NM_001011667]	79145

A_23_P13502	1.8	2.0	1.6	1.2	CHCHD8	Homo sapiens coiled-coil-helix-coiled-coil-helix domain containing 8 (CHCHD8), mRNA [NM_016565]	51287
A_24_P85317	-3.9	1.0	1.0	1.4	CHD2	Homo sapiens chromodomain helicase DNA binding protein 2 (CHD2), transcript variant 2, mRNA [NM_001042572]	1106
A_24_P264664	4.8	4.6	6.0	9.8	CHD2	Homo sapiens chromodomain helicase DNA binding protein 2 (CHD2), transcript variant 1, mRNA [NM_001271]	1106

A_23_P53541	2.1	1.6	1.3	1.5	CHD4	Homo sapiens chromodomain helicase DNA binding protein 4 (CHD4), mRNA [NM_001273]	1108
A_23_P91217	1.1	-6.3	1.1	1.1	CHD6	Homo sapiens chromodomain helicase DNA binding protein 6 (CHD6), mRNA [NM_032221]	84181
A_32_P150012	-1.1	1.2	2.8	-2.9	CHD7	Homo sapiens chromodomain helicase DNA binding protein 7 (CHD7), mRNA [NM_017780]	55636
A_23_P354929	4.0	1.5	16.5	16.4	CHD9	Homo sapiens chromodomain helicase DNA binding protein 9 (CHD9), mRNA [NM_025134]	80205

A_24_P942250	-2.3	1.6	2.3	1.2	CHD9	Homo sapiens chromodomain helicase DNA binding protein 9 (CHD9), mRNA [NM_025134]	80205
A_23_P372638	-2.0	1.8	-4.0	1.1	CHD9	Homo sapiens chromodomain helicase DNA binding protein 9 (CHD9), mRNA [NM_025134]	80205
A_23_P357185	-1.1	-1.8	1.2	-2.7	CHDH	Homo sapiens choline dehydrogenase (CHDH), nuclear gene encoding mitochondrial protein, mRNA [NM_018397]	55349

A_23_P116123	-3.0	-1.1	-6.5	1.6	CHEK1	Homo sapiens CHK1 checkpoint homolog (S. pombe) (CHEK1), transcript variant 3, mRNA [NM_001274]	1111
A_23_P109452	-1.5	-3.8	-2.7	-3.7	CHEK2	Homo sapiens CHK2 checkpoint homolog (S. pombe) (CHEK2), transcript variant 3, mRNA [NM_001005735]	11200
A_32_P27046	1.4	-1.1	3.4	-1.4	CHGA	Homo sapiens chromogranin A (parathyroid secretory protein 1) (CHGA), mRNA [NM_001275]	1113
A_23_P57155	2.0	2.0	1.8	1.8	CHGB	Homo sapiens chromogranin B (secretogranin 1) (CHGB), mRNA [NM_001819]	1114

A_24_P274111	1.0	-1.0	-5.6	-1.0	CHI3L1	Homo sapiens chitinase 3-like 1 (cartilage glycoprotein-39) (CHI3L1), mRNA [NM_001276]	1116
A_23_P34888	-17.8	1.1	1.1	1.1	CHIA	Homo sapiens chitinase, acidic (CHIA), transcript variant 2, mRNA [NM_021797]	27159
A_23_P147875	2.2	1.2	3.1	-1.3	CHID1	Homo sapiens chitinase domain containing 1 (CHID1), transcript variant 3, mRNA [NM_023947]	66005
A_23_P126278	1.2	1.2	-2.4	1.1	CHIT1	Homo sapiens chitinase 1 (chitotriosidase) (CHIT1), mRNA [NM_003465]	1118

A_23_P136135	3.6	3.5	3.4	3.5	CHKA	Homo sapiens choline kinase alpha (CHKA), transcript variant 1, mRNA [NM_001277]	1119
A_24_P63178	1.0	1.0	-3.3	-1.1	CHL1	Homo sapiens cell adhesion molecule with homology to L1CAM (close homolog of L1) (CHL1), mRNA [NM_006614]	10752
A_23_P22548	-14.5	-1.0	-1.0	-1.0	CHM	Homo sapiens choroideremia (Rab escort protein 1) (CHM), transcript variant 1, mRNA [NM_000390]	1121

A_24_P240065	2.3	1.9	2.6	-3.0	CHMP3	Homo sapiens charged multivesicular body protein 3 (CHMP3), transcript variant 1, mRNA [NM_016079]	51652
A_24_P142024	1.2	1.3	1.0	2.1	CHMP4A	Homo sapiens charged multivesicular body protein 4A (CHMP4A), mRNA [NM_014169]	29082
A_23_P301732	-2.2	-1.8	1.0	-1.3	CHMP4B	Homo sapiens charged multivesicular body protein 4B (CHMP4B), mRNA [NM_176812]	128866
A_23_P303203	2.8	2.2	1.6	1.2	CHMP7	Homo sapiens charged multivesicular body protein 7 (CHMP7), mRNA [NM_152272]	91782

A_23_P68669	1.3	-1.5	1.7	-2.7	CHODL	Homo sapiens chondrolectin (CHODL), transcript variant 1, mRNA [NM_024944]	140578
A_23_P100111	-1.3	1.0	2.2	1.8	CHP	Homo sapiens calcium binding protein P22 (CHP), mRNA [NM_007236]	11261
A_23_P131289	1.0	1.6	-4.2	1.1	CHPF	Homo sapiens chondroitin polymerizing factor (CHPF), transcript variant 1, mRNA [NM_024536]	79586
A_23_P397055	-4.8	1.2	1.0	2.5	CHPF2	Homo sapiens chondroitin polymerizing factor 2 (CHPF2), mRNA [NM_019015]	54480

A_32_P54442	-1.9	3.9	-1.3	-1.3	CHPF2	Homo sapiens chondroitin polymerizing factor 2 (CHPF2), mRNA [NM_019015]	54480
A_23_P123544	-1.0	1.3	2.4	1.1	CHRAC1	Homo sapiens chromatin accessibility complex 1 (CHRAC1), transcript variant 1, mRNA [NM_017444]	54108
A_24_P117725	1.7	-2.7	1.6	-1.3	CHRD	Homo sapiens chordin (CHRD), mRNA [NM_003741]	8646
A_23_P502047	-1.7	-1.9	-2.4	-1.7	CHRD	Homo sapiens chordin (CHRD), mRNA [NM_003741]	8646

A_24_P911939	2.4	1.4	2.3	1.1	CHRM2	Homo sapiens cholinergic receptor, muscarinic 2 (CHRM2), transcript variant 2, mRNA [NM_001006627]	1129
A_23_P401472	1.6	2.6	1.1	1.8	CHRM3	Homo sapiens cholinergic receptor, muscarinic 3 (CHRM3), mRNA [NM_000740]	1131
A_23_P210395	-1.3	1.6	2.1	1.1	CHRNA4	Homo sapiens cholinergic receptor, nicotinic, alpha 4 (CHRNA4), mRNA [NM_000744]	1137
A_24_P912720	1.1	-1.3	-2.6	1.2	CHRNA4	H.sapiens mRNA for neuronal acetylcholine receptor alpha-4 subunit, exon 1. [X89741]	1137

A_32_P35969	1.0	-1.2	-4.4	-1.0	CHRNA7	Homo sapiens cholinergic receptor, nicotinic, alpha 7 (CHRNA7), transcript variant 2, mRNA [NM_001190455]	1139
A_24_P27627	-11.3	-1.2	1.0	-2.1	CHRNB1	Homo sapiens cholinergic receptor, nicotinic, beta 1 (muscle) (CHRNB1), mRNA [NM_000747]	1140
A_23_P207106	-1.0	-1.0	-3.2	-1.0	CHRNB1	Homo sapiens cholinergic receptor, nicotinic, beta 1 (muscle) (CHRNB1), mRNA [NM_000747]	1140

A_23_P123424	2.1	2.0	1.9	1.9	CHRNB3	Homo sapiens cholinergic receptor, nicotinic, beta 3 (CHRNB3), mRNA [NM_000749]	1142
A_23_P304279	-1.3	2.6	2.9	2.0	CHRNE	Homo sapiens cholinergic receptor, nicotinic, epsilon (CHRNE), mRNA [NM_000080]	1145
A_23_P102351	1.6	1.6	-2.4	1.0	CHST10	Homo sapiens carbohydrate sulfotransferase 10 (CHST10), mRNA [NM_004854]	9486
A_23_P139919	-3.9	1.1	1.1	-3.2	CHST11	Homo sapiens carbohydrate (chondroitin 4) sulfotransferase 11 (CHST11), transcript variant 1, mRNA [NM_018413]	50515

A_32_P99171	1.7	2.3	-1.4	1.3	CHST11	Homo sapiens carbohydrate (chondroitin 4) sulfotransferase 11 (CHST11), transcript variant 1, mRNA [NM_018413]	50515
A_23_P80473	-2.5	-1.3	-2.0	-1.4	CHST13	Homo sapiens carbohydrate (chondroitin 4) sulfotransferase 13 (CHST13), mRNA [NM_152889]	166012
A_24_P210637	9.2	1.8	2.7	8.7	CHST14	Homo sapiens carbohydrate (N-acetylgalactosamine 4-0) sulfotransferase 14 (CHST14), mRNA [NM_130468]	113189

A_23_P141035	-2.1	-2.0	-1.0	1.7	CHST4	Homo sapiens carbohydrate (N-acetylglucosamine 6-O) sulfotransferase 4 (CHST4), transcript variant 1, mRNA [NM_005769]	10164
A_23_P106922	-1.0	1.2	-3.8	-1.0	CHST6	Homo sapiens carbohydrate (N-acetylglucosamine 6-O) sulfotransferase 6 (CHST6), mRNA [NM_021615]	4166
A_23_P319617	-9.1	-1.0	-1.1	1.0	CHST7	Homo sapiens carbohydrate (N-acetylglucosamine 6-O) sulfotransferase 7 (CHST7), mRNA [NM_019886]	56548

A_23_P435649	-7.4	1.0	1.0	1.1	CHST9-AS1	Homo sapiens CHST9 antisense RNA 1 (non-protein coding) (CHST9-AS1), non-coding RNA [NR_026908]	147429
A_32_P44274	1.2	-1.4	1.4	-2.6	CHTF18	Homo sapiens CTF18, chromosome transmission fidelity factor 18 homolog (S. cerevisiae) (CHTF18), mRNA [NM_022092]	63922
A_23_P354297	-2.2	-2.1	-3.1	-3.6	CHTF18	Homo sapiens CTF18, chromosome transmission fidelity factor 18 homolog (S. cerevisiae) (CHTF18), mRNA [NM_022092]	63922

A_23_P201726	-1.2	-2.2	-1.2	-1.2	CHTOP	Homo sapiens chromatin target of PRMT1 (CHTOP), transcript variant 2, mRNA [NM_001206612]	26097
A_23_P77360	1.6	2.0	3.2	2.8	CIAO1	Homo sapiens cytosolic iron-sulfur protein assembly 1 (CIAO1), mRNA [NM_004804]	9391
A_23_P410059	-3.9	-3.2	-5.5	-2.0	CIAO1	Homo sapiens cytosolic iron-sulfur protein assembly 1 (CIAO1), mRNA [NM_004804]	9391
A_23_P257638	1.8	1.5	1.1	2.5	CIB1	Homo sapiens calcium and integrin binding 1 (calmyrin) (CIB1), mRNA [NM_006384]	10519

A_23_P65890	1.8	3.9	3.9	1.5	CIB2	Homo sapiens calcium and integrin binding family member 2 (CIB2), mRNA [NM_006383]	10518
A_23_P130585	1.2	1.3	-2.3	-1.5	CIB3	Homo sapiens calcium and integrin binding family member 3 (CIB3), mRNA [NM_054113]	117286
A_23_P55731	1.6	2.4	2.0	1.8	CIC	Homo sapiens capicua homolog (Drosophila) (CIC), mRNA [NM_015125]	23152
A_23_P37359	-1.3	-1.3	-2.0	-1.4	CIDEB	Homo sapiens cell death-inducing DFFA-like effector b (CIDEB), mRNA [NM_014430]	27141

A_23_P92025	-1.4	-1.5	-1.1	-2.1	CIDEC	Homo sapiens cell death-inducing DFFA-like effector c (CIDEC), transcript variant 3, mRNA [NM_022094]	63924
A_23_P33384	1.1	2.2	1.0	-1.0	CIITA	Homo sapiens class II, major histocompatibility complex, transactivator (CIITA), mRNA [NM_000246]	4261
A_23_P377318	2.3	2.0	1.5	2.0	CILP2	Homo sapiens cartilage intermediate layer protein 2 (CILP2), mRNA [NM_153221]	148113
A_23_P39814	-1.7	-1.1	-2.2	-1.2	CIR1	Homo sapiens corepressor interacting with RBPJ, 1 (CIR1), mRNA [NM_004882]	9541

A_23_P413456	1.2	1.7	2.6	1.3	CIRBP	Homo sapiens cold inducible RNA binding protein (CIRBP), transcript variant 2, non-coding RNA [NR_023312]	1153
A_23_P35467	1.0	-2.4	1.7	1.2	CISD1	Homo sapiens CDGSH iron sulfur domain 1 (CISD1), mRNA [NM_018464]	55847
A_32_P162357	1.5	1.5	-2.4	1.1	CISD2	Homo sapiens CDGSH iron sulfur domain 2 (CISD2), mRNA [NM_001008388]	493856
A_24_P97465	1.5	-1.4	-1.5	-3.5	CISH	Homo sapiens cytokine inducible SH2-containing protein (CISH), transcript variant 2, mRNA [NM_145071]	1154

A_23_P144096	2.5	11.1	5.3	9.0	CISH	Homo sapiens cytokine inducible SH2-containing protein (CISH), transcript variant 2, mRNA [NM_145071]	1154
A_32_P209230	-1.2	1.4	-1.3	-2.4	CITED4	Homo sapiens Cbp/p300-interacting transactivator, with Glu/Asp-rich carboxy-terminal domain, 4 (CITED4), mRNA [NM_133467]	163732
A_23_P32229	-1.5	2.5	-2.0	-1.3	CIZ1	Homo sapiens CDKN1A interacting zinc finger protein 1 (CIZ1), transcript variant 1, mRNA [NM_012127]	25792

A_23_P388812	-1.0	-1.0	1.4	-7.2	CKAP2L	Homo sapiens cytoskeleton associated protein 2-like (CKAP2L), mRNA [NM_152515]	150468
A_24_P61537	-1.6	2.2	-1.1	1.3	CKB	Homo sapiens creatine kinase, brain (CKB), mRNA [NM_001823]	1152
A_24_P215804	-4.1	-1.2	-1.0	-1.7	CKLF	Homo sapiens chemokine-like factor (CKLF), transcript variant 1, mRNA [NM_016951]	51192
A_23_P106663	10.9	8.8	8.8	8.9	CKLF-CMTM1	Homo sapiens CKLF-CMTM1 readthrough (CKLF-CMTM1), transcript variant 1, mRNA [NM_001204099]	100529251

A_23_P71727	-1.1	-3.5	-1.2	-1.2	CKS2	Homo sapiens CDC28 protein kinase regulatory subunit 2 (CKS2), mRNA [NM_001827]	1164
A_23_P311232	6.8	8.4	2.6	6.4	CLASP1	Homo sapiens cytoplasmic linker associated protein 1 (CLASP1), transcript variant 1, mRNA [NM_015282]	23332
A_24_P127748	-4.1	-1.0	-2.1	-1.6	CLASP2	Homo sapiens cytoplasmic linker associated protein 2 (CLASP2), transcript variant 1, mRNA [NM_015097]	23122

A_23_P44295	1.8	2.4	2.0	2.2	CLASP2	Homo sapiens cytoplasmic linker associated protein 2 (CLASP2), transcript variant 1, mRNA [NM_015097]	23122
A_23_P78517	-2.2	-1.4	-1.1	-1.0	CLASRP	Homo sapiens CLK4-associating serine/arginine rich protein (CLASRP), mRNA [NM_007056]	11129
A_23_P51217	1.0	-1.0	-2.4	-1.1	CLCA1	Homo sapiens chloride channel accessory 1 (CLCA1), mRNA [NM_001285]	1179
A_23_P45751	1.0	-1.0	-2.7	-1.1	CLCA4	Homo sapiens chloride channel accessory 4 (CLCA4), transcript variant 1, mRNA [NM_012128]	22802

A_24_P402847	1.1	-43.5	1.0	-1.0	CLCN3	Homo sapiens chloride channel 3 (CLCN3), transcript variant b, mRNA [NM_001829]	1182
A_23_P124476	3.2	3.3	4.0	-1.1	CLCN3	Homo sapiens chloride channel 3 (CLCN3), transcript variant e, mRNA [NM_173872]	1182
A_23_P369210	1.9	2.2	1.9	1.1	CLCN5	chloride channel 5 [Source:HGNC Symbol;Acc:2023] [ENST00000376108]	1184
A_23_P200361	1.1	1.6	1.5	-4.8	CLCN6	Homo sapiens chloride channel 6 (CLCN6), transcript variant ClC-6a, mRNA [NM_001286]	1185

A_24_P2361	1.6	1.5	2.6	2.7	CLCNKA	Homo sapiens chloride channel Ka (CLCNKA), transcript variant 1, mRNA [NM_004070]	1187
A_23_P405088	1.2	3.8	1.3	-1.8	CLDN11	Homo sapiens claudin 11 (CLDN11), transcript variant 1, mRNA [NM_005602]	5010
A_32_P23272	1.9	1.9	1.9	2.0	CLDN11	Homo sapiens full length insert cDNA clone YO11A01. [AF085871]	5010
A_23_P29800	1.3	-1.2	-1.6	2.3	CLDN11	Homo sapiens claudin 11 (CLDN11), transcript variant 1, mRNA [NM_005602]	5010
A_23_P157268	1.2	-1.0	-1.0	-2.0	CLDN12	Homo sapiens claudin 12 (CLDN12), transcript variant 3, mRNA [NM_012129]	9069

A_23_P500886	2.0	2.0	3.9	3.5	CLDN15	Homo sapiens claudin 15 (CLDN15), transcript variant 2, mRNA [NM_014343]	24146
A_23_P379054	1.1	1.1	-2.0	-1.0	CLDN19	Homo sapiens claudin 19 (CLDN19), transcript variant 1, mRNA [NM_148960]	149461
A_23_P394947	1.5	3.6	1.5	1.9	CLDN2	Homo sapiens claudin 2 (CLDN2), transcript variant 1, mRNA [NM_020384]	9075
A_23_P134854	17.6	3.8	4.7	4.8	CLDN23	Homo sapiens claudin 23 (CLDN23), mRNA [NM_194284]	137075
A_24_P115183	-2.6	-1.1	-2.2	-2.2	CLDN4	Homo sapiens claudin 4 (CLDN4), mRNA [NM_001305]	1364

A_24_P79300	4.9	2.8	6.3	4.0	CLDN5	Homo sapiens claudin 5 (CLDN5), transcript variant 2, mRNA [NM_003277]	7122
A_23_P37942	3.0	2.1	3.1	1.1	CLDN6	Homo sapiens claudin 6 (CLDN6), mRNA [NM_021195]	9074
A_23_P141505	1.1	1.0	2.2	1.0	CLEC10A	Homo sapiens C-type lectin domain family 10, member A (CLEC10A), transcript variant 1, mRNA [NM_182906]	10462
A_23_P141508	2.0	1.6	-2.2	-1.9	CLEC10A	Homo sapiens C-type lectin domain family 10, member A (CLEC10A), transcript variant 2, mRNA [NM_006344]	10462

A_23_P204671	-1.3	-1.3	-3.1	-1.3	CLEC12B	Homo sapiens C-type lectin domain family 12, member B (CLEC12B), transcript variant 1, mRNA [NM_001129998]	387837
A_24_P215653	1.1	-1.0	1.3	-2.0	CLEC14A	Homo sapiens C-type lectin domain family 14, member A (CLEC14A), mRNA [NM_175060]	161198
A_23_P206501	1.3	1.1	1.1	-2.0	CLEC18B	Homo sapiens C-type lectin domain family 18, member B (CLEC18B), mRNA [NM_001011880]	497190
A_24_P201702	1.0	-2.5	-5.0	-3.0	CLEC2B	Homo sapiens C-type lectin domain family 2, member B (CLEC2B), mRNA [NM_005127]	9976

A_32_P95960	-1.3	-4.2	-1.7	-1.4	CLEC2D	Homo sapiens C-type lectin domain family 2, member D (CLEC2D), transcript variant 2, mRNA [NM_001004419]	29121
A_24_P364335	-3.4	1.3	1.1	-9.1	CLEC2D	Homo sapiens C-type lectin domain family 2, member D (CLEC2D), transcript variant 2, mRNA [NM_001004419]	29121
A_32_P80068	1.0	-1.8	-1.0	-2.1	CLEC2D	Homo sapiens C-type lectin domain family 2, member D (CLEC2D), transcript variant 2, mRNA [NM_001004419]	29121

A_24_P241183	-1.0	-1.0	-1.1	2.6	CLEC2D	Homo sapiens C-type lectin domain family 2, member D (CLEC2D), transcript variant 1, mRNA [NM_013269]	29121
A_23_P380990	-1.1	1.7	-3.0	-1.5	CLEC4F	Homo sapiens C-type lectin domain family 4, member F (CLEC4F), mRNA [NM_173535]	165530
A_23_P208482	-1.2	1.2	1.0	-2.1	CLEC4M	Homo sapiens C-type lectin domain family 4, member M (CLEC4M), transcript variant 2, mRNA [NM_001144904]	10332

A_24_P227415	-1.0	-4.0	-1.5	-2.7	CLEC7A	Homo sapiens C-type lectin domain family 7, member A (CLEC7A), transcript variant 6, mRNA [NM_197954]	64581
A_23_P18684	1.1	1.0	-2.5	-3.3	CLGN	Homo sapiens calmegin (CLGN), transcript variant 1, mRNA [NM_004362]	1047
A_23_P30884	1.6	-1.9	-1.4	2.3	CLIC1	Homo sapiens chloride intracellular channel 1 (CLIC1), mRNA [NM_001288]	1192
A_23_P45456	-3.5	1.3	1.3	1.0	CLIC2	Homo sapiens chloride intracellular channel 2 (CLIC2), mRNA [NM_001289]	1193

A_23_P259189	-3.6	-4.1	1.1	1.1	CLIC4	Homo sapiens chloride intracellular channel 4 (CLIC4), nuclear gene encoding mitochondrial protein, mRNA [NM_013943]	25932
A_23_P416774	-1.1	-5.2	-1.0	1.1	CLIC5	Homo sapiens chloride intracellular channel 5 (CLIC5), nuclear gene encoding mitochondrial protein, transcript variant 2, mRNA [NM_016929]	53405
A_23_P25003	-5.4	-1.7	-1.2	1.0	CLIP1	Homo sapiens CAP-GLY domain containing linker protein 1 (CLIP1), transcript variant 1, mRNA [NM_002956]	6249

A_24_P279718	-3.8	-1.9	-2.5	-2.9	CLIP3	Homo sapiens CAP-GLY domain containing linker protein 3 (CLIP3), transcript variant 2, mRNA [NM_015526]	25999
A_23_P50786	-1.0	-4.3	-2.1	-1.5	CLIP3	Homo sapiens CAP-GLY domain containing linker protein 3 (CLIP3), transcript variant 2, mRNA [NM_015526]	25999
A_23_P209232	1.0	-1.0	-11.6	-1.1	CLIP4	Homo sapiens CAP-GLY domain containing linker protein family, member 4 (CLIP4), mRNA [NM_024692]	79745

A_23_P417363	2.5	2.3	2.2	2.7	CLIP4	CAP-GLY domain containing linker protein family, member 4 [Source:HGNC Symbol;Acc:26108] [ENST00000415891]	79745
A_23_P16817	1.2	2.3	1.9	1.8	CLK1	Homo sapiens CDC-like kinase 1 (CLK1), transcript variant 1, mRNA [NM_004071]	1195
A_24_P257151	1.0	1.2	1.2	-3.9	CLK1	Homo sapiens CDC-like kinase 1 (CLK1), transcript variant 1, mRNA [NM_004071]	1195
A_23_P85888	1.2	2.2	1.5	2.6	CLK2	Homo sapiens CDC-like kinase 2 (CLK2), mRNA [NM_003993]	1196

A_24_P67027	-1.3	4.4	1.2	-1.7	CLK2	Homo sapiens CDC-like kinase 2 (CLK2), mRNA [NM_003993]	1196
A_23_P99974	-1.8	-2.1	-2.4	-1.1	CLK3	Homo sapiens CDC-like kinase 3 (CLK3), transcript variant 2, mRNA [NM_003992]	1198
A_32_P20691	1.0	-1.4	-1.0	-3.0	CLK3	Homo sapiens CDC-like kinase 3 (CLK3), transcript variant 2, mRNA [NM_003992]	1198
A_23_P61674	2.6	4.5	1.3	2.5	CLK4	Homo sapiens CDC-like kinase 4 (CLK4), mRNA [NM_020666]	57396
A_23_P35995	-1.3	-1.4	-1.2	-4.7	CLMP	Homo sapiens CXADR-like membrane protein (CLMP), mRNA [NM_024769]	79827

A_23_P88971	-2.8	-1.8	-4.3	-4.1	CLN3	Homo sapiens ceroid-lipofuscinosis, neuronal 3 (CLN3), transcript variant 1, mRNA [NM_001042432]	1201
A_24_P296424	-14.6	-2.3	-2.6	-2.7	CLN3	Homo sapiens ceroid-lipofuscinosis, neuronal 3 (CLN3), transcript variant 1, mRNA [NM_001042432]	1201
A_24_P179071	-3.1	1.7	1.1	1.8	CLN3	Homo sapiens ceroid-lipofuscinosis, neuronal 3 (CLN3), transcript variant 1, mRNA [NM_001042432]	1201

A_24_P914649	1.6	-2.2	-3.0	-1.5	CLNS1A	chloride channel, nucleotide-sensitive, 1A [Source:HGNC Symbol;Acc:2080] [ENST00000533957]	1207
A_23_P419038	1.1	1.1	-7.5	1.0	CLOCK	Homo sapiens clock homolog (mouse) (CLOCK), mRNA [NM_004898]	9575
A_23_P87141	-5.4	-1.0	-1.0	-1.0	CLP1	Homo sapiens CLP1, cleavage and polyadenylation factor I subunit, homolog (S. cerevisiae) (CLP1), transcript variant 1, mRNA [NM_006831]	10978

A_24_P252973	-1.1	-1.1	-1.2	-53.5	CLP1	Homo sapiens CLP1, cleavage and polyadenylation factor I subunit, homolog (S. cerevisiae) (CLP1), transcript variant 1, mRNA [NM_006831]	10978
A_23_P75978	1.1	-3.5	-1.6	1.0	CLPB	Homo sapiens ClpB caseinolytic peptidase B homolog (E. coli) (CLPB), mRNA [NM_030813]	81570

A_23_P4754	2.1	1.7	2.4	1.3	CLPP	Homo sapiens ClpP caseinolytic peptidase, ATP-dependent, proteolytic subunit homolog (E. coli) (CLPP), nuclear gene encoding mitochondrial protein, mRNA [NM_006012]	8192
A_23_P8142	-1.0	-1.4	-2.4	-3.4	CLPS	Homo sapiens colipase, pancreatic (CLPS), mRNA [NM_001832]	1208
A_24_P191067	1.0	1.0	1.0	-2.0	CLSTN1	Homo sapiens calsyntenin 1 (CLSTN1), transcript variant 1, mRNA [NM_001009566]	22883

A_23_P252671	-8.2	1.1	-1.3	-1.3	CLTB	Homo sapiens clathrin, light chain B (CLTB), transcript variant 2, mRNA [NM_007097]	1212
A_23_P143559	-4.1	-1.5	1.2	2.1	CLTCL1	Homo sapiens clathrin, heavy chain-like 1 (CLTCL1), transcript variant 1, mRNA [NM_007098]	8218
A_23_P77714	1.4	1.0	-4.6	1.1	CLUAP1	Homo sapiens clusterin associated protein 1 (CLUAP1), transcript variant 2, mRNA [NM_024793]	23059
A_23_P55572	1.1	-5.7	1.0	1.1	CLUL1	Homo sapiens clusterin-like 1 (retinal) (CLUL1), transcript variant 1, mRNA [NM_014410]	27098

A_24_P23482	2.4	3.1	2.3	2.1	CLYBL	Homo sapiens cDNA FLJ38187 fis, clone FCBBF1000182. [AK095506]	171425
A_23_P151778	1.2	3.7	4.0	1.1	CMA1	Homo sapiens chymase 1, mast cell (CMA1), mRNA [NM_001836]	1215
A_32_P73045	-3.9	1.0	1.0	1.0	CMAS	Homo sapiens cytidine monophosphate N-acetylneuraminic acid synthetase (CMAS), mRNA [NM_018686]	55907

A_24_P408740	1.1	1.1	-1.6	-6.6	CMC1	Homo sapiens COX assembly mitochondrial protein homolog (S. cerevisiae) (CMC1), nuclear gene encoding mitochondrial protein, mRNA [NM_182523]	152100
A_23_P105465	-2.3	1.8	-1.2	-1.1	CMKLR1	Homo sapiens chemokine-like receptor 1 (CMKLR1), transcript variant 2, mRNA [NM_004072]	1240
A_23_P105461	1.1	2.0	1.2	1.0	CMKLR1	Homo sapiens chemokine-like receptor 1 (CMKLR1), transcript variant 2, mRNA [NM_004072]	1240

A_24_P766716	-4.5	1.0	1.0	-2.0	CMKLR1	Homo sapiens chemokine-like receptor 1 (CMKLR1), transcript variant 1, mRNA [NM_001142343]	1240
A_24_P39508	2.4	2.7	2.4	2.3	CMTM3	Homo sapiens CKLF-like MARVEL transmembrane domain containing 3 (CMTM3), transcript variant 1, mRNA [NM_144601]	123920
A_23_P391980	-1.2	-2.3	-1.3	-1.5	CMTM4	Homo sapiens CKLF-like MARVEL transmembrane domain containing 4 (CMTM4), transcript variant 1, mRNA [NM_178818]	146223

A_23_P256413	1.0	-4.9	1.3	-1.0	CMTM7	Homo sapiens CKLF-like MARVEL transmembrane domain containing 7 (CMTM7), transcript variant 1, mRNA [NM_138410]	112616
A_24_P937546	2.8	3.4	2.8	2.6	CMTM7	Homo sapiens mRNA; cDNA DKFZp434I2129 (from clone DKFZp434I2129). [AL832450]	112616
A_23_P40880	-8.2	-1.0	-1.1	-1.0	CMTM8	Homo sapiens CKLF-like MARVEL transmembrane domain containing 8 (CMTM8), mRNA [NM_178868]	152189

A_24_P151084	2.5	2.9	4.0	2.9	CNDP2	Homo sapiens CNDP dipeptidase 2 (metallopeptidase M20 family) (CNDP2), transcript variant 1, mRNA [NM_018235]	55748
A_23_P27473	2.9	1.5	1.5	1.4	CNFN	Homo sapiens cornifelin (CNFN), mRNA [NM_032488]	84518
A_24_P256722	2.7	2.7	1.6	2.5	CNGA1	Homo sapiens cyclic nucleotide gated channel alpha 1 (CNGA1), transcript variant 2, mRNA [NM_000087]	1259
A_23_P2006	1.4	2.1	1.4	1.4	CNGA4	Homo sapiens cyclic nucleotide gated channel alpha 4 (CNGA4), mRNA [NM_001037329]	1262

A_24_P229588	1.6	-2.3	1.3	-4.7	CNGB1	Homo sapiens cyclic nucleotide gated channel beta 1 (CNGB1), transcript variant 1, mRNA [NM_001297]	1258
A_24_P203315	-1.9	-2.2	-3.1	-1.8	CNIH2	Homo sapiens cornichon homolog 2 (Drosophila) (CNIH2), mRNA [NM_182553]	254263
A_23_P23341	-1.1	2.2	2.2	1.3	CNKSR1	Homo sapiens connector enhancer of kinase suppressor of Ras 1 (CNKSR1), transcript variant 1, mRNA [NM_006314]	10256
A_23_P46947	-1.2	-3.6	-1.4	-1.3	CNNM1	Homo sapiens cyclin M1 (CNNM1), mRNA [NM_020348]	26507

A_23_P24044	-1.6	-1.1	-1.4	-2.0	CNNM2	Homo sapiens cyclin M2 (CNNM2), transcript variant 1, mRNA [NM_017649]	54805
A_24_P184154	1.6	3.1	1.5	1.0	CNNM2	Homo sapiens cyclin M2 (CNNM2), transcript variant 1, mRNA [NM_017649]	54805
A_24_P93896	4.6	2.4	3.1	2.0	CNNM2	Homo sapiens cyclin M2 (CNNM2), transcript variant 3, mRNA [NM_199077]	54805
A_23_P69670	1.0	-1.0	-1.0	-10.9	CNO	Homo sapiens cappuccino homolog (mouse) (CNO), mRNA [NM_018366]	55330

A_23_P77371	-1.1	2.6	1.1	1.1	CNOT1	Homo sapiens CCR4-NOT transcription complex, subunit 1 (CNOT1), transcript variant 1, mRNA [NM_016284]	23019
A_24_P101114	1.5	-1.0	1.6	2.6	CNOT1	Homo sapiens CCR4-NOT transcription complex, subunit 1 (CNOT1), transcript variant 2, mRNA [NM_206999]	23019
A_23_P421147	-1.3	1.7	4.0	-1.9	CNOT2	Homo sapiens CCR4-NOT transcription complex, subunit 2 (CNOT2), transcript variant 2, mRNA [NM_014515]	4848

A_24_P8049	-1.0	11.6	1.2	1.2	CNOT2	Homo sapiens CCR4-NOT transcription complex, subunit 2 (CNOT2), transcript variant 2, mRNA [NM_014515]	4848
A_23_P38987	1.9	1.8	1.0	6.4	CNOT3	Homo sapiens CCR4-NOT transcription complex, subunit 3 (CNOT3), mRNA [NM_014516]	4849
A_24_P941922	1.3	-1.8	1.2	-2.1	CNOT4	CCR4-NOT transcription complex, subunit 4 [Source:HGNC Symbol;Acc:7880] [ENST00000315544]	4850
A_23_P167417	2.2	1.4	2.1	2.1	CNOT6	Homo sapiens CCR4-NOT transcription complex, subunit 6 (CNOT6), mRNA [NM_015455]	57472

A_23_P18818	1.1	1.1	-1.1	-5.6	CNOT6	Homo sapiens CCR4-NOT transcription complex, subunit 6 (CNOT6), mRNA [NM_015455]	57472
A_23_P110304	-1.0	-5.9	-1.1	-2.2	CNOT6L	Homo sapiens CCR4-NOT transcription complex, subunit 6-like (CNOT6L), mRNA [NM_144571]	246175
A_23_P216312	5.2	3.2	1.5	2.2	CNOT7	Homo sapiens CCR4-NOT transcription complex, subunit 7 (CNOT7), transcript variant 2, mRNA [NM_054026]	29883
A_23_P10798	1.0	-2.5	-1.1	-1.7	CNP	Homo sapiens 2',3'-cyclic nucleotide 3' phosphodiesterase (CNP), mRNA [NM_033133]	1267

A_24_P128683	-1.3	-2.0	1.1	-2.0	CNPPD1	Homo sapiens cyclin Pas1/PHO80 domain containing 1 (CNPPD1), mRNA [NM_015680]	27013
A_24_P16503	-1.2	-1.5	-3.7	-1.9	CNPY3	Homo sapiens HSPC084 mRNA, partial cds. [AF161347]	10695
A_24_P363259	2.6	-1.5	-1.1	-1.7	CNR1	Homo sapiens cannabinoid receptor 1 (brain) (CNR1), transcript variant 2, mRNA [NM_033181]	1268
A_32_P48054	-2.1	-2.0	-1.0	1.1	CNR2	cannabinoid receptor 2 (macrophage) [Source:HGNC Symbol;Acc:2160] [ENST00000374472]	1269

A_23_P157963	-2.1	-1.0	-1.1	-1.9	CNTLN	Homo sapiens centlein, centrosomal protein (CNTLN), transcript variant 1, mRNA [NM_017738]	54875
A_32_P95082	1.5	2.4	1.4	1.0	CNTLN	Homo sapiens centlein, centrosomal protein (CNTLN), transcript variant 1, mRNA [NM_017738]	54875
A_24_P924217	-2.4	-1.0	-1.1	1.0	CNTLN	centlein, centrosomal protein [Source:HGNC Symbol;Acc:23432] [ENST00000484374]	54875
A_24_P128200	1.2	1.2	1.1	-2.6	CNTN1	contactin 1 [Source:HGNC Symbol;Acc:2171] [ENST00000547849]	1272

A_23_P204541	2.1	2.1	3.0	1.1	CNTN1	contactin 1 [Source:HGNC Symbol;Acc:2171] [ENST00000551295]	1272
A_24_P2901	-3.2	1.3	1.1	1.4	CNTN2	Homo sapiens contactin 2 (axonal) (CNTN2), mRNA [NM_005076]	6900
A_32_P92188	-2.3	1.1	1.0	1.0	CNTN5	Homo sapiens contactin 5 (CNTN5), transcript variant 1, mRNA [NM_014361]	53942
A_23_P503059	-2.0	3.4	1.4	1.8	CNTN5	Homo sapiens contactin 5 (CNTN5), transcript variant 1, mRNA [NM_014361]	53942
A_23_P411113	3.8	3.7	2.6	3.6	CNTNAP1	Homo sapiens contactin associated protein 1 (CNTNAP1), mRNA [NM_003632]	8506

A_24_P418203	-3.1	-1.0	-1.0	1.1	CNTNAP3	Homo sapiens contactin associated protein-like 3 (CNTNAP3), mRNA [NM_033655]	79937
A_24_P652609	-4.1	-1.7	-2.5	-1.3	CNTNAP3B	Homo sapiens contactin associated protein-like 3B (CNTNAP3B), mRNA [NM_001201380]	728577
A_23_P9768	1.1	1.4	-3.7	-1.1	CNTROB	Homo sapiens centrobin, centrosomal BRCA2 interacting protein (CNTROB), transcript variant 1, mRNA [NM_053051]	116840
A_23_P39910	-24.2	-1.0	-1.0	-1.0	COA5	Homo sapiens cytochrome C oxidase assembly factor 5 (COA5), mRNA [NM_001008215]	493753

A_23_P4144	-6.3	3.5	1.9	1.9	COASY	Homo sapiens CoA synthase (COASY), nuclear gene encoding mitochondrial protein, transcript variant 1, mRNA [NM_025233]	80347
A_32_P228804	4.1	-1.7	1.6	2.8	COBLL1	COBL-like 1 [Source:HGNC Symbol;Acc:23571] [ENST00000375458]	22837
A_24_P915926	1.5	1.5	2.0	-2.9	COBLL1	COBL-like 1 [Source:HGNC Symbol;Acc:23571] [ENST00000496396]	22837
A_23_P148150	1.2	-2.3	1.6	1.2	COBRA1	Homo sapiens cofactor of BRCA1 (COBRA1), mRNA [NM_015456]	25920

A_24_P184803	-1.3	-1.0	-1.1	-2.0	COCH	Homo sapiens coagulation factor C homolog, cochlin (Limulus polyphemus) (COCH), transcript variant 2, mRNA [NM_004086]	1690
A_23_P50020	-5.0	-1.2	-1.0	1.1	COG1	Homo sapiens component of oligomeric golgi complex 1 (COG1), mRNA [NM_018714]	9382
A_23_P402437	-9.8	1.0	-6.8	1.1	COG2	component of oligomeric golgi complex 2 [Source:HGNC Symbol;Acc:6546] [ENST00000494371]	22796

A_24_P310946	1.1	1.0	-4.0	1.1	COG2	Homo sapiens component of oligomeric golgi complex 2 (COG2), transcript variant 1, mRNA [NM_007357]	22796
A_23_P2692	-1.7	-2.4	-2.3	-2.4	COG3	Homo sapiens component of oligomeric golgi complex 3 (COG3), mRNA [NM_031431]	83548
A_24_P296070	1.0	1.0	-1.0	-11.8	COG3	Homo sapiens component of oligomeric golgi complex 3 (COG3), mRNA [NM_031431]	83548
A_23_P320717	4.8	4.0	2.1	4.5	COG4	Homo sapiens component of oligomeric golgi complex 4 (COG4), transcript variant 1, mRNA [NM_015386]	25839

A_23_P329361	1.2	-1.1	-1.2	-2.1	COG7	Homo sapiens component of oligomeric golgi complex 7 (COG7), mRNA [NM_153603]	91949
A_24_P155815	1.9	-6.7	-1.3	-1.0	COG8	Homo sapiens component of oligomeric golgi complex 8 (COG8), mRNA [NM_032382]	84342
A_23_P26729	2.4	1.3	1.9	2.2	COIL	Homo sapiens coilin (COIL), mRNA [NM_004645]	8161
A_23_P214144	-1.4	1.2	-1.1	-2.1	COL10A1	Homo sapiens collagen, type X, alpha 1 (COL10A1), mRNA [NM_000493]	1300

A_23_P42322	1.0	-9.3	-2.6	1.0	COL11A2	Homo sapiens collagen, type XI, alpha 2 (COL11A2), transcript variant 1, mRNA [NM_080680]	1302
A_23_P214168	1.0	-1.0	1.1	-3.4	COL12A1	Homo sapiens collagen, type XII, alpha 1 (COL12A1), transcript variant long, mRNA [NM_004370]	1303
A_32_P80850	1.2	1.6	-1.1	3.2	COL14A1	Homo sapiens collagen, type XIV, alpha 1 (COL14A1), mRNA [NM_021110]	7373
A_24_P254789	-11.5	1.2	1.1	-1.9	COL14A1	Homo sapiens collagen, type XIV, alpha 1 (COL14A1), mRNA [NM_021110]	7373

A_23_P501010	-1.0	-1.0	-2.7	-1.5	COL17A1	Homo sapiens collagen, type XVII, alpha 1 (COL17A1), mRNA [NM_000494]	1308
A_24_P57426	-1.9	-1.3	-4.5	-4.0	COL18A1	Homo sapiens collagen, type XVIII, alpha 1 (COL18A1), transcript variant 1, mRNA [NM_030582]	80781
A_23_P211212	-2.3	-2.3	-2.4	-2.4	COL18A1	Homo sapiens collagen, type XVIII, alpha 1 (COL18A1), transcript variant 1, mRNA [NM_030582]	80781
A_24_P265274	4.2	4.2	4.0	3.8	COL1A2	Homo sapiens collagen, type I, alpha 2 (COL1A2), mRNA [NM_000089]	1278

A_32_P185637	-3.7	1.1	-1.3	-1.0	COL20A1	Homo sapiens collagen, type XX, alpha 1 (COL20A1), mRNA [NM_020882]	57642
A_23_P387031	-1.2	2.2	-1.3	-1.8	COL23A1	Homo sapiens collagen, type XXIII, alpha 1 (COL23A1), mRNA [NM_173465]	91522
A_23_P58427	1.8	-1.3	-2.4	1.3	COL25A1	Homo sapiens collagen, type XXV, alpha 1 (COL25A1), transcript variant 2, mRNA [NM_032518]	84570
A_24_P257478	11.4	7.9	2.6	9.5	COL25A1	Homo sapiens collagen, type XXV, alpha 1 (COL25A1), transcript variant 1, mRNA [NM_198721]	84570

A_24_P363896	-1.8	-1.1	-2.8	-2.0	COL27A1	Homo sapiens collagen, type XXVII, alpha 1 (COL27A1), mRNA [NM_032888]	85301
A_24_P935491	-3.0	-1.0	-1.1	-2.1	COL3A1	Homo sapiens collagen, type III, alpha 1 (COL3A1), mRNA [NM_000090]	1281
A_24_P68342	-1.7	-2.9	-1.5	-1.2	COL4A1	Homo sapiens collagen, type IV, alpha 1 (COL4A1), mRNA [NM_001845]	1282
A_23_P205031	-1.3	-2.5	1.0	-1.1	COL4A2	Homo sapiens collagen, type IV, alpha 2 (COL4A2), mRNA [NM_001846]	1284
A_24_P915591	1.6	1.5	-3.7	-1.1	COL4A6	collagen, type IV, alpha 6 [Source:HGNC Symbol;Acc:2208] [ENST00000461897]	1288

A_23_P158593	-1.5	-3.3	-1.0	-1.6	COL5A1	Homo sapiens collagen, type V, alpha 1 (COL5A1), mRNA [NM_000093]	1289
A_32_P218734	-1.0	-22.1	-1.0	1.2	COL5A2	Homo sapiens collagen, type V, alpha 2 (COL5A2), mRNA [NM_000393]	1290
A_32_P32254	1.1	-1.0	-2.3	1.1	COL6A1	Homo sapiens collagen, type VI, alpha 1 (COL6A1), mRNA [NM_001848]	1291
A_24_P331918	-1.2	1.0	-1.3	-2.7	COL6A1	Homo sapiens collagen, type VI, alpha 1 (COL6A1), mRNA [NM_001848]	1291
A_23_P211233	-1.6	-2.5	-1.2	-1.9	COL6A2	Homo sapiens collagen, type VI, alpha 2 (COL6A2), transcript variant 2C2, mRNA [NM_001849]	1292

A_23_P131614	1.2	4.3	3.1	1.0	COL6A3	Homo sapiens collagen, type VI, alpha 3 (COL6A3), transcript variant 1, mRNA [NM_004369]	1293
A_32_P224525	-1.1	-1.1	-1.2	-20.0	COL6A6	Homo sapiens collagen, type VI, alpha 6 (COL6A6), mRNA [NM_001102608]	131873
A_23_P144071	-1.0	1.5	1.3	-2.3	COL7A1	Homo sapiens collagen, type VII, alpha 1 (COL7A1), mRNA [NM_000094]	1294
A_23_P69030	2.4	2.3	2.1	2.5	COL8A1	Homo sapiens collagen, type VIII, alpha 1 (COL8A1), transcript variant 1, mRNA [NM_001850]	1295

A_24_P365975	1.8	3.1	1.4	-1.1	COL8A2	Homo sapiens collagen, type VIII, alpha 2 (COL8A2), mRNA [NM_005202]	1296
A_24_P311856	-2.2	-1.9	-5.4	-1.9	COL9A1	Homo sapiens collagen, type IX, alpha 1 (COL9A1), transcript variant 1, mRNA [NM_001851]	1297
A_23_P40108	1.0	-2.7	-1.1	-1.0	COL9A3	Homo sapiens collagen, type IX, alpha 3 (COL9A3), mRNA [NM_001853]	1299
A_24_P26160	1.4	2.4	1.1	1.4	COLQ	Homo sapiens collagen-like tail subunit (single strand of homotrimer) of asymmetric acetylcholinesterase (COLQ), transcript variant II, mRNA [NM_080538]	8292

A_23_P212126	1.7	1.7	3.8	1.0	COLQ	Homo sapiens collagen-like tail subunit (single strand of homotrimer) of asymmetric acetylcholinesterase (COLQ), transcript variant II, mRNA [NM_080538]	8292
A_23_P40782	-1.4	-2.0	-1.6	-1.2	COMMD2	Homo sapiens COMM domain containing 2 (COMMD2), mRNA [NM_016094]	51122
A_24_P93371	-1.2	-1.2	-1.4	-29.3	COMMD4	Homo sapiens COMM domain containing 4 (COMMD4), mRNA [NM_017828]	54939

A_24_P331904	3.2	2.2	1.7	-1.0	COMMD4	Homo sapiens COMM domain containing 4 (COMMD4), mRNA [NM_017828]	54939
A_23_P420428	-2.5	-2.7	-6.5	-3.7	COMMD7	Homo sapiens COMM domain containing 7 (COMMD7), transcript variant 1, mRNA [NM_053041]	149951
A_24_P264943	1.1	-4.2	-1.5	-2.3	COMP	Homo sapiens cartilage oligomeric matrix protein (COMP), mRNA [NM_000095]	1311
A_23_P90436	1.3	-1.0	-1.1	-21.6	COMP	Homo sapiens cartilage oligomeric matrix protein (COMP), mRNA [NM_000095]	1311

A_23_P325932	1.6	1.6	2.9	1.6	COPA	Homo sapiens coatomer protein complex, subunit alpha (COPA), transcript variant 2, mRNA [NM_004371]	1314
A_23_P91891	1.0	1.0	-2.5	1.0	COPB2	Homo sapiens coatomer protein complex, subunit beta 2 (beta prime) (COPB2), transcript variant 1, mRNA [NM_004766]	9276
A_24_P389959	2.0	2.6	-1.0	1.4	COPZ1	Homo sapiens coatomer protein complex, subunit zeta 1 (COPZ1), mRNA [NM_016057]	22818

A_23_P111228	5.7	5.7	5.5	5.6	COQ3	Homo sapiens coenzyme Q3 homolog, methyltransferase (S. cerevisiae) (COQ3), mRNA [NM_017421]	51805
A_24_P389491	1.6	2.1	1.4	-1.3	COQ4	Homo sapiens coenzyme Q4 homolog (S. cerevisiae) (COQ4), nuclear gene encoding mitochondrial protein, mRNA [NM_016035]	51117

A_23_P65584	1.1	1.1	1.3	-2.4	COQ6	Homo sapiens coenzyme Q6 homolog, monooxygenase (S. cerevisiae) (COQ6), nuclear gene encoding mitochondrial protein, transcript variant 1, mRNA [NM_182476]	51004
A_23_P152322	-5.3	1.3	1.9	-1.2	COQ7	Homo sapiens coenzyme Q7 homolog, ubiquinone (yeast) (COQ7), transcript variant 1, mRNA [NM_016138]	10229
A_24_P398972	-2.1	1.1	-1.2	-3.6	COQ7	Homo sapiens coenzyme Q7 homolog, ubiquinone (yeast) (COQ7), transcript variant 1, mRNA [NM_016138]	10229

A_24_P932308	2.6	2.8	2.0	1.8	COQ9	Homo sapiens cDNA PSEC0129 fis, clone PLACE1004170. [AK075438]	57017
A_23_P14928	2.0	-5.6	1.9	1.3	COQ9	Homo sapiens coenzyme Q9 homolog (S. cerevisiae) (COQ9), nuclear gene encoding mitochondrial protein, mRNA [NM_020312]	57017
A_23_P81131	-1.3	1.0	-2.0	1.2	CORIN	Homo sapiens corin, serine peptidase (CORIN), mRNA [NM_006587]	10699

A_23_P104583	-3.2	-2.7	-2.0	-1.6	CORO1B	Homo sapiens coronin, actin binding protein, 1B (CORO1B), transcript variant 1, mRNA [NM_020441]	57175
A_23_P117694	1.6	-1.0	1.9	2.0	CORO2B	Homo sapiens coronin, actin binding protein, 2B (CORO2B), transcript variant 1, mRNA [NM_006091]	10391
A_23_P107322	4.4	1.0	1.5	-2.5	CORO6	Homo sapiens coronin 6 (CORO6), mRNA [NM_032854]	84940
A_23_P38046	3.1	4.3	1.2	2.9	CORO7	Homo sapiens coronin 7 (CORO7), transcript variant 1, mRNA [NM_024535]	79585

A_23_P258164	2.0	4.9	1.2	1.2	CORT	Homo sapiens cortistatin (CORT), mRNA [NM_001302]	1325
A_23_P83624	-1.0	-4.6	-1.0	-1.0	COTL1	Homo sapiens coactosin-like 1 (Dictyostelium) (COTL1), mRNA [NM_021149]	23406
A_32_P158385	-1.5	-1.5	-1.5	-4.5	COTL1	Human autonomously replicating sequence (ARS) mRNA. [L08436]	23406
A_23_P301925	1.8	2.4	2.0	1.2	COX1	mitochondrially encoded cytochrome c oxidase I [Source:HGNC Symbol;Acc:7419] [ENST00000361624]	4512

A_24_P391531	1.0	-1.0	-3.0	-1.7	COX11	Homo sapiens COX11 cytochrome c oxidase assembly homolog (yeast) (COX11), nuclear gene encoding mitochondrial protein, transcript variant 1, mRNA [NM_004375]	1353
A_23_P144244	1.0	-1.4	-1.0	-2.1	COX17	COX17 cytochrome c oxidase assembly homolog (S. cerevisiae) [Source:HGNC Symbol;Acc:2264] [ENST00000484810]	10063

A_24_P208897	1.1	1.1	-1.0	-4.0	COX18	Homo sapiens COX18 cytochrome c oxidase assembly homolog (S. cerevisiae) (COX18), nuclear gene encoding mitochondrial protein, mRNA [NM_173827]	285521
A_24_P350200	1.9	1.8	-1.2	2.0	COX3	mitochondrially encoded cytochrome c oxidase III [Source:HGNC Symbol;Acc:7422] [ENST00000362079]	4514

A_23_P210869	-1.9	1.7	1.1	-4.2	COX4I2	Homo sapiens cytochrome c oxidase subunit IV isoform 2 (lung) (COX4I2), nuclear gene encoding mitochondrial protein, mRNA [NM_032609]	84701
A_32_P98502	7.5	7.3	7.2	7.1	COX5A	Homo sapiens cytochrome c oxidase subunit Va (COX5A), nuclear gene encoding mitochondrial protein, mRNA [NM_004255]	9377

A_23_P401524	-2.7	-1.5	-6.2	-4.7	COX6A2	Homo sapiens cytochrome c oxidase subunit VIa polypeptide 2, mRNA (cDNA clone MGC:34466 IMAGE:5174639), complete cds. [BC029818]	1339
A_23_P108244	-2.1	-2.4	-1.7	-1.2	COX6B1	Homo sapiens cytochrome c oxidase subunit VIb polypeptide 1 (ubiquitous) (COX6B1), nuclear gene encoding mitochondrial protein, mRNA [NM_001863]	1340

A_23_P67661	-1.1	-1.1	-2.2	-1.2	COX7A1	Homo sapiens cytochrome c oxidase subunit VIIa polypeptide 1 (muscle) (COX7A1), nuclear gene encoding mitochondrial protein, mRNA [NM_001864]	1346
A_23_P81690	1.2	3.2	-1.7	-1.2	COX7A2	Homo sapiens cytochrome c oxidase subunit VIIa polypeptide 2 (liver) (COX7A2), nuclear gene encoding mitochondrial protein, transcript variant 1, mRNA [NM_001865]	1347

A_23_P136246	-10.9	1.0	-1.0	-1.7	COX7B2	Homo sapiens cytochrome c oxidase subunit VIIb2 (COX7B2), mRNA [NM_130902]	170712
A_23_P110811	13.3	13.0	12.6	13.5	COX7C	Homo sapiens cytochrome c oxidase subunit VIIc (COX7C), nuclear gene encoding mitochondrial protein, mRNA [NM_001867]	1350
A_23_P414793	1.5	2.3	-1.1	-1.0	CP	Homo sapiens ceruloplasmin (ferroxidase) (CP), mRNA [NM_000096]	1356
A_23_P31161	2.0	2.0	2.0	1.9	CPA2	Homo sapiens carboxypeptidase A2 (pancreatic) (CPA2), mRNA [NM_001869]	1358

A_23_P18017	2.6	1.0	1.4	-1.0	CPA3	Homo sapiens carboxypeptidase A3 (mast cell) (CPA3), mRNA [NM_001870]	1359
A_24_P4170	1.0	1.0	-1.0	2.4	CPA6	Homo sapiens carboxypeptidase A6 (CPA6), mRNA [NM_020361]	57094
A_32_P225355	2.7	-1.3	-1.2	1.3	CPEB2	Homo sapiens cytoplasmic polyadenylation element binding protein 2 (CPEB2), transcript variant B, mRNA [NM_182485]	132864

A_23_P46812	5.3	5.2	4.9	5.3	CPEB3	Homo sapiens cytoplasmic polyadenylation element binding protein 3 (CPEB3), transcript variant 1, mRNA [NM_014912]	22849
A_24_P58740	1.1	1.1	-2.3	-1.8	CPEB4	Homo sapiens cytoplasmic polyadenylation element binding protein 4 (CPEB4), mRNA [NM_030627]	80315
A_24_P391868	-1.2	-1.2	-1.0	-5.1	CPLX2	Homo sapiens complexin 2 (CPLX2), transcript variant 1, mRNA [NM_006650]	10814
A_23_P117851	-5.4	-1.0	-1.1	-1.1	CPLX3	Homo sapiens complexin 3 (CPLX3), mRNA [NM_001030005]	594855

A_24_P938352	2.2	2.3	-2.0	1.2	CPM	Homo sapiens carboxypeptidase M (CPM), transcript variant 1, mRNA [NM_001874]	1368
A_23_P98147	-1.2	-10.4	1.1	-2.1	CPN1	Homo sapiens carboxypeptidase N, polypeptide 1 (CPN1), mRNA [NM_001308]	1369
A_23_P37988	1.4	2.5	1.2	1.5	CPNE2	Homo sapiens copine II (CPNE2), mRNA [NM_152727]	221184
A_24_P64126	2.6	1.3	1.6	2.1	CPNE3	Homo sapiens copine III (CPNE3), mRNA [NM_003909]	8895
A_23_P94370	1.6	-1.0	1.0	-2.1	CPNE3	Homo sapiens copine III (CPNE3), mRNA [NM_003909]	8895

A_23_P151595	8.3	1.9	2.1	1.8	CPNE6	Homo sapiens copine VI (neuronal) (CPNE6), mRNA [NM_006032]	9362
A_23_P26426	2.1	2.5	-2.5	-1.8	CPNE7	Homo sapiens copine VII (CPNE7), transcript variant 2, mRNA [NM_014427]	27132
A_24_P56240	1.0	1.0	-2.4	-2.7	CPNE8	Homo sapiens copine VIII (CPNE8), mRNA [NM_153634]	144402
A_23_P144179	3.3	4.4	1.3	2.9	CPOX	Homo sapiens coproporphyrinogen oxidase (CPOX), mRNA [NM_000097]	1371
A_23_P169112	1.8	2.2	-3.2	1.4	CPSF1	Homo sapiens cleavage and polyadenylation specific factor 1, 160kDa (CPSF1), mRNA [NM_013291]	29894

A_23_P35030	1.0	-1.0	-2.4	-1.0	CPSF3L	Homo sapiens cleavage and polyadenylation specific factor 3-like (CPSF3L), mRNA [NM_017871]	54973
A_23_P42997	-1.3	2.2	-1.2	-1.2	CPSF4	Homo sapiens cleavage and polyadenylation specific factor 4, 30kDa (CPSF4), transcript variant 1, mRNA [NM_006693]	10898
A_24_P347065	2.2	2.7	4.6	1.4	CPT1A	Homo sapiens carnitine palmitoyltransferase 1A (liver) (CPT1A), nuclear gene encoding mitochondrial protein, transcript variant 1, mRNA [NM_001876]	1374

A_23_P104563	1.8	2.5	1.3	1.4	CPT1A	Homo sapiens carnitine palmitoyltransferase 1A (liver) (CPT1A), nuclear gene encoding mitochondrial protein, transcript variant 2, mRNA [NM_001031847]	1374
A_23_P16283	2.6	1.7	-1.5	-1.1	CPT1C	Homo sapiens carnitine palmitoyltransferase 1C (CPT1C), transcript variant 2, mRNA [NM_152359]	126129
A_23_P148919	1.8	-1.1	-1.1	-3.5	CPT2	Homo sapiens carnitine palmitoyltransferase 2 (CPT2), nuclear gene encoding mitochondrial protein, mRNA [NM_000098]	1376

A_23_P62336	-4.9	-1.0	-4.1	-1.0	CPXCR1	Homo sapiens CPX chromosome region, candidate 1 (CPXCR1), transcript variant 1, mRNA [NM_033048]	53336
A_23_P6066	1.2	1.4	2.4	1.1	CPXM1	Homo sapiens carboxypeptidase X (M14 family), member 1 (CPXM1), transcript variant 1, mRNA [NM_019609]	56265
A_23_P124542	1.2	1.0	-2.5	-1.4	CR2	Human CR2/CD21/C3d/Epstein-Barr virus receptor mRNA, complete cds. [M26004]	1380
A_23_P117882	2.6	1.7	2.1	1.2	CRABP1	Homo sapiens cellular retinoic acid binding protein 1 (CRABP1), mRNA [NM_004378]	1381

A_24_P324871	-1.0	1.3	1.2	3.6	CRABP1	Homo sapiens cDNA FLJ38687 fis, clone KIDNE200 1117. [AK096006]	1381
A_23_P115064	1.7	4.8	2.3	3.4	CRABP2	Homo sapiens cellular retinoic acid binding protein 2 (CRABP2), transcript variant 1, mRNA [NM_0018 78]	1382
A_23_P407992	-2.3	-1.0	-2.2	-1.0	CRAMP1L	Homo sapiens Crm, cramped-like (Drosophil a) (CRAMP1L), mRNA [NM_0208 25]	57585
A_24_P51279	2.0	-1.2	1.2	1.0	CRAMP1L	Crm, cramped-like (Drosophil a) [Source:H GNC Symbol;Ac c:14122] [ENST0000 0454337]	57585

A_32_P151437	-3.1	-2.3	-1.3	-1.4	CRAMP1L	Homo sapiens Crm, cramped-like (Drosophila) (CRAMP1L), mRNA [NM_020825]	57585
A_23_P32199	5.2	5.1	3.8	1.2	CRAT	Homo sapiens carnitine O-acetyltransferase (CRAT), nuclear gene encoding mitochondrial protein, transcript variant 1, mRNA [NM_000755]	1384
A_23_P12357	1.0	1.0	-1.7	-2.1	CRB1	Homo sapiens crumbs homolog 1 (Drosophila) (CRB1), transcript variant 1, mRNA [NM_201253]	23418

A_24_P351435	2.0	1.9	-1.4	1.9	CRBN	Homo sapiens cereblon (CRBN), transcript variant 1, mRNA [NM_016302]	51185
A_23_P250930	-3.6	-2.0	-2.9	1.0	CRBN	Homo sapiens cereblon (CRBN), transcript variant 1, mRNA [NM_016302]	51185
A_24_P286412	-3.4	-1.8	-2.8	1.0	CRCP	Homo sapiens CGRP receptor component (CRCP), transcript variant 1, mRNA [NM_014478]	27297
A_23_P134573	-2.2	-1.9	-1.9	-1.9	CRCP	Homo sapiens CGRP receptor component (CRCP), transcript variant 1, mRNA [NM_014478]	27297

A_24_P191047	-3.0	1.8	-1.0	-1.0	CRCT1	Homo sapiens cysteine-rich C-terminal 1 (CRCT1), mRNA [NM_019060]	54544
A_23_P12155	8.2	8.1	8.2	2.8	CRCT1	Homo sapiens cysteine-rich C-terminal 1 (CRCT1), mRNA [NM_019060]	54544
A_23_P423389	-1.7	-1.7	-1.7	-3.6	CREB3	Homo sapiens cAMP responsive element binding protein 3 (CREB3), mRNA [NM_006368]	10488
A_23_P150407	-1.9	-1.7	-4.4	-1.8	CREB3L1	Homo sapiens cAMP responsive element binding protein 3-like 1 (CREB3L1), mRNA [NM_052854]	90993

A_24_P180654	1.1	1.1	-4.0	-1.0	CREB3L2	cAMP responsive element binding protein 3-like 2 [Source:HGNC Symbol;Acc:23720] [ENST00000452463]	64764
A_23_P108082	1.7	7.0	4.5	4.9	CREB3L3	Homo sapiens cAMP responsive element binding protein 3-like 3 (CREB3L3), mRNA [NM_032607]	84699
A_24_P322025	2.3	1.5	3.1	2.0	CREBBP	Homo sapiens CREB binding protein (CREBBP), transcript variant 1, mRNA [NM_004380]	1387
A_23_P394986	2.1	1.1	-1.2	2.0	CREG2	Homo sapiens cellular repressor of E1A-stimulated genes 2 (CREG2), mRNA [NM_153836]	200407

A_23_P33465	3.7	1.5	1.2	-1.7	CRELD2	Homo sapiens cysteine-rich with EGF-like domains 2 (CRELD2), transcript variant 2, mRNA [NM_024324]	79174
A_24_P922139	-1.0	-1.0	-1.1	-14.6	CREM	Homo sapiens cAMP responsive element modulator (CREM), transcript variant 16, mRNA [NM_182772]	1390
A_24_P278393	13.9	21.4	13.9	13.5	CREM	Homo sapiens cAMP responsive element modulator (CREM), transcript variant 19, mRNA [NM_183013]	1390

A_23_P414884	1.9	10.3	2.2	5.4	CRHR1	Homo sapiens corticotropin releasing hormone receptor 1 (CRHR1), transcript variant 2, mRNA [NM_004382]	1394
A_23_P215331	1.3	-1.6	1.1	-2.5	CRHR2	Homo sapiens corticotropin releasing hormone receptor 2 (CRHR2), transcript variant 1, mRNA [NM_001883]	1395
A_24_P926058	2.5	2.2	2.1	1.0	CRHR2	H.sapiens mRNA for CRF2 receptor, beta isoform, aberrantly spliced, (94bp deletion). [Y10152]	1395

A_24_P323395	1.5	3.8	-1.1	-1.1	CRIM1	Homo sapiens cysteine rich transmembrane BMP regulator 1 (chordin-like) (CRIM1), mRNA [NM_016441]	51232
A_23_P112798	-1.2	-6.7	-1.7	-1.2	CRIP2	Homo sapiens cysteine-rich protein 2 (CRIP2), mRNA [NM_001312]	1397
A_23_P133722	-1.8	-5.0	-4.4	-1.3	CRISP1	Homo sapiens cysteine-rich secretory protein 1 (CRISP1), transcript variant 1, mRNA [NM_001131]	167
A_32_P173662	2.7	2.6	2.2	2.3	CRISP2	Homo sapiens cysteine-rich secretory protein 2 (CRISP2), transcript variant 1, mRNA [NM_003296]	7180

A_23_P59960	-1.4	1.0	-1.5	-2.3	CRISPLD1	Homo sapiens cysteine-rich secretory protein LCCL domain containing 1 (CRISPLD1), mRNA [NM_031461]	83690
A_24_P339059	1.0	-8.7	-1.0	1.5	CRISPLD2	Homo sapiens cysteine-rich secretory protein LCCL domain containing 2 (CRISPLD2), mRNA [NM_031476]	83716
A_23_P83556	-1.0	-1.0	2.3	-1.0	CRK	Homo sapiens v-crk sarcoma virus CT10 oncogene homolog (avian) (CRK), transcript variant II, mRNA [NM_016823]	1398

A_24_P270814	8.7	8.6	8.4	8.5	CRK	Homo sapiens v-crk sarcoma virus CT10 oncogene homolog (avian) (CRK), transcript variant II, mRNA [NM_016823]	1398
A_24_P56887	-2.2	1.1	-1.1	-1.0	CRLF3	Homo sapiens cytokine receptor-like factor 3 (CRLF3), mRNA [NM_015986]	51379
A_23_P400435	-1.0	-2.7	-1.0	-1.0	CRLS1	Homo sapiens cardiolipin synthase 1 (CRLS1), transcript variant 1, mRNA [NM_019095]	54675
A_24_P20506	-1.0	1.5	-3.2	1.2	CRMP1	Homo sapiens collapsin response mediator protein 1 (CRMP1), transcript variant 1, mRNA [NM_001014809]	1400

A_23_P131967	1.1	-4.1	1.1	1.1	CRNKL1	Homo sapiens crooked neck pre-mRNA splicing factor-like 1 (Drosophila) (CRNKL1), mRNA [NM_016652]	51340
A_23_P115202	-1.2	-1.7	-3.9	1.4	CRNN	Homo sapiens cornulin (CRNN), mRNA [NM_016190]	49860
A_23_P104086	1.5	1.9	2.0	1.0	CROCC	Homo sapiens ciliary rootlet coiled-coil, rootletin (CROCC), mRNA [NM_014675]	9696
A_32_P384562	4.0	4.0	1.4	3.1	CROCCP3	Homo sapiens ciliary rootlet coiled-coil, rootletin pseudogene 3 (CROCCP3), non-coding RNA [NR_023386]	114819

A_24_P342484	-1.3	-1.6	-1.7	-2.0	CRP	Homo sapiens C-reactive protein, pentraxin-related (CRP), mRNA [NM_000567]	1401
A_23_P431971	1.1	-2.8	1.0	1.1	CRTAC1	Homo sapiens cartilage acidic protein 1 (CRTAC1), transcript variant 1, mRNA [NM_018058]	55118
A_24_P71661	-9.3	-1.2	-1.2	-2.9	CRTAP	Homo sapiens cartilage associated protein (CRTAP), mRNA [NM_006371]	10491
A_24_P140171	1.0	-5.2	-1.0	-1.0	CRTAP	Homo sapiens cartilage associated protein (CRTAP), mRNA [NM_006371]	10491

A_24_P219952	-1.8	-3.2	1.1	-3.0	CRTC1	Homo sapiens CREB regulated transcription coactivator 1 (CRTC1), transcript variant 3, mRNA [NM_001098482]	23373
A_23_P12173	1.5	2.1	1.9	-1.0	CRTC2	Homo sapiens CREB regulated transcription coactivator 2 (CRTC2), mRNA [NM_181715]	200186
A_23_P388027	1.1	-2.0	-1.4	-1.4	CRY2	Homo sapiens cryptochrome 2 (photolyase-like) (CRY2), transcript variant 1, mRNA [NM_021117]	1408

A_24_P158587	4.4	2.8	2.9	1.6	CRY2	Homo sapiens cryptochrome 2 (photolyase-like) (CRY2), transcript variant 1, mRNA [NM_021117]	1408
A_23_P306755	-2.2	1.6	-1.9	2.5	CRYAA	Homo sapiens crystallin, alpha A (CRYAA), mRNA [NM_000394]	1409
A_24_P206776	-2.1	2.3	2.2	2.0	CRYAB	Homo sapiens crystallin, alpha B (CRYAB), mRNA [NM_001885]	1410
A_23_P165457	9.5	6.6	9.1	-5.3	CRYBA2	Homo sapiens crystallin, beta A2 (CRYBA2), transcript variant 1, mRNA [NM_005209]	1412
A_23_P425066	1.0	1.4	-1.4	-2.8	CRYBB2	Homo sapiens crystallin, beta B2 (CRYBB2), mRNA [NM_000496]	1415

A_24_P823219	4.9	3.2	2.4	3.7	CRYBB2P1	Homo sapiens crystallin, beta B2 pseudogene 1 (CRYBB2P1), transcript variant 1, non-coding RNA [NR_033733]	1416
A_23_P29179	3.0	3.3	-1.6	2.5	CRYBB3	Homo sapiens crystallin, beta B3 (CRYBB3), mRNA [NM_004076]	1417
A_23_P165380	-8.1	-1.3	-1.1	-1.8	CRYGA	Homo sapiens crystallin, gamma A (CRYGA), mRNA [NM_014617]	1418
A_23_P414842	3.2	3.1	2.9	11.1	CRYGB	Homo sapiens crystallin, gamma B (CRYGB), mRNA [NM_005210]	1419

A_23_P320705	-2.6	1.2	1.1	-2.0	CRYGD	Homo sapiens crystallin, gamma D, mRNA (cDNA clone MGC:150947 IMAGE:40125889), complete cds. [BC117338]	1421
A_23_P131215	1.1	1.3	-1.4	-2.6	CRYGD	Homo sapiens crystallin, gamma D (CRYGD), mRNA [NM_006891]	1421
A_23_P132738	1.1	1.1	-2.2	-1.2	CRYGS	Homo sapiens crystallin, gamma S (CRYGS), mRNA [NM_017541]	1427
A_23_P77731	-6.4	-1.7	-2.1	-2.0	CRYM	Homo sapiens crystallin, mu (CRYM), transcript variant 1, mRNA [NM_001888]	1428

A_23_P114662	1.0	-1.0	-1.6	-2.0	CRYZ	Homo sapiens crystallin, zeta (quinone reductase) (CRYZ), transcript variant 2, mRNA [NM_001889]	1429
A_24_P408341	2.0	1.7	1.3	2.0	CRYZL1	crystallin, zeta (quinone reductase)-like 1 [Source:HGNC Symbol;Acc:2420] [ENST00000452332]	9946
A_24_P79529	-2.5	1.4	-1.1	-5.5	CSAG2	Homo sapiens CSAG family, member 2 (CSAG2), transcript variant 1, mRNA [NM_001080848]	728461
A_23_P25224	1.2	2.3	1.4	-1.1	CSDA	Homo sapiens cold shock domain protein A (CSDA), transcript variant 1, mRNA [NM_003651]	8531

A_24_P625382	3.1	2.5	-1.2	1.2	CSDA	Homo sapiens cold shock domain protein A (CSDA), transcript variant 1, mRNA [NM_003651]	8531
A_24_P177279	1.8	2.2	1.4	1.3	CSDC2	Homo sapiens cold shock domain containing C2, RNA binding (CSDC2), mRNA [NM_014460]	27254
A_23_P74595	-1.0	2.3	1.1	1.4	CSDE1	Homo sapiens cold shock domain containing E1, RNA-binding (CSDE1), transcript variant 1, mRNA [NM_001007553]	7812
A_23_P17393	-2.9	-4.3	-1.1	-1.3	CSE1L	Homo sapiens CSE1 chromosome segregation 1-like (yeast) (CSE1L), mRNA [NM_001316]	1434

A_23_P407012	1.8	-4.6	1.1	-1.3	CSF1	Homo sapiens colony stimulating factor 1 (macrophage) (CSF1), transcript variant 2, mRNA [NM_172210]	1435
A_23_P133408	3.8	4.8	3.2	2.2	CSF2	Homo sapiens colony stimulating factor 2 (granulocyte-macrophage) (CSF2), mRNA [NM_000758]	1437
A_23_P501754	-2.7	-2.6	-2.9	-3.0	CSF3	Homo sapiens colony stimulating factor 3 (granulocyte) (CSF3), transcript variant 1, mRNA [NM_000759]	1440

A_23_P126218	1.1	1.3	-6.6	-2.2	CSF3R	Homo sapiens colony stimulating factor 3 receptor (granulocyte) (CSF3R), transcript variant 4, mRNA [NM_172313]	1441
A_23_P134835	-1.9	-1.8	-2.4	-3.8	CSGALNACT1	Homo sapiens chondroitin sulfate N-acetylgalactosaminyltransferase 1 (CSGALNACT1), transcript variant 2, mRNA [NM_018371]	55790
A_23_P161269	1.9	-1.1	1.9	2.6	CSGALNACT2	Homo sapiens chondroitin sulfate N-acetylgalactosaminyltransferase 2 (CSGALNACT2), mRNA [NM_018590]	55454

A_24_P211044	2.5	3.0	2.6	-1.0	CSH1	Homo sapiens chorionic somatomammotropin hormone 1 (placental lactogen) (CSH1), mRNA [NM_001317]	1442
A_23_P381351	1.0	-4.1	-1.0	-1.0	CSH1	Homo sapiens chorionic somatomammotropin hormone 1 (placental lactogen) (CSH1), mRNA [NM_001317]	1442
A_23_P207170	-1.6	2.1	1.2	1.1	CSH1	Homo sapiens chorionic somatomammotropin hormone 1 (placental lactogen) (CSH1), mRNA [NM_001317]	1442

A_23_P207154	1.5	1.5	1.9	3.8	CSH2	Homo sapiens chorionic somatomammotropin hormone 2 (CSH2), transcript variant 2, mRNA [NM_022644]	1443
A_23_P207180	2.8	2.3	2.1	2.1	CSHL1	Homo sapiens chorionic somatomammotropin hormone-like 1 (CSHL1), transcript variant 2, mRNA [NM_022581]	1444
A_23_P152024	1.9	2.2	2.0	2.4	CSK	Homo sapiens c-src tyrosine kinase (CSK), transcript variant 1, mRNA [NM_004383]	1445
A_24_P338788	1.4	2.2	1.3	1.0	CSNK1A1L	Homo sapiens casein kinase 1, alpha 1-like (CSNK1A1L), mRNA [NM_145203]	122011

A_32_P68408	1.1	-1.0	-2.6	1.3	CSNK1A1P1	Homo sapiens casein kinase 1, alpha 1 pseudogene 1 (CSNK1A1P1), non-coding RNA [NR_027320]	161635
A_23_P29263	-3.0	1.1	1.3	1.3	CSNK1E	Homo sapiens casein kinase 1, epsilon (CSNK1E), transcript variant 1, mRNA [NM_152221]	1454
A_23_P252636	1.2	2.0	2.8	1.9	CSNK1G1	Homo sapiens casein kinase 1, gamma 1 (CSNK1G1), mRNA [NM_022048]	53944
A_24_P99963	6.1	1.1	6.4	5.9	CSNK1G2	Homo sapiens casein kinase 1, gamma 2 (CSNK1G2), mRNA [NM_001319]	1455

A_23_P56140	9.2	10.4	6.0	6.3	CSNK1G2	Homo sapiens casein kinase 1, gamma 2 (CSNK1G2), mRNA [NM_001319]	1455
A_23_P14915	1.7	2.5	1.5	-1.4	CSNK2A2	Homo sapiens casein kinase 2, alpha prime polypeptide (CSNK2A2), mRNA [NM_001896]	1459
A_23_P73870	1.7	1.0	2.4	-1.0	CSPG4P1Y	Homo sapiens chondroitin sulfate proteoglycan 4 pseudogene 1, Y-linked (CSPG4P1Y), non-coding RNA [NR_001554]	114758

A_24_P342944	4.3	-1.3	1.5	4.0	CSPG5	Homo sapiens chondroitin sulfate proteoglycan 5 (neuroglycan C) (CSPG5), transcript variant 1, mRNA [NM_006574]	10675
A_23_P169558	-3.7	-1.0	-2.4	-1.0	CSPP1	Homo sapiens centrosome and spindle pole associated protein 1 (CSPP1), mRNA [NM_024790]	79848
A_23_P121011	1.8	1.1	-6.7	1.5	CSRNP1	Homo sapiens cysteine-serine-rich nuclear protein 1 (CSRNP1), mRNA [NM_033027]	64651

A_23_P151209	3.7	-1.3	1.2	2.9	CSRNP2	Homo sapiens cysteine-serine-rich nuclear protein 2 (CSRNP2), transcript variant 1, mRNA [NM_030809]	81566
A_24_P225448	1.0	-1.1	-1.2	2.0	CSRP1	Homo sapiens cysteine and glycine-rich protein 1 (CSRP1), transcript variant 1, mRNA [NM_004078]	1465
A_23_P44724	1.0	-2.3	-5.2	-1.3	CSRP2	Homo sapiens cysteine and glycine-rich protein 2 (CSRP2), mRNA [NM_001321]	1466
A_24_P237175	2.3	4.3	2.9	1.5	CST2	Homo sapiens cystatin SA (CST2), mRNA [NM_001322]	1470

A_24_P216294	3.0	-1.2	2.7	1.1	CST3	Homo sapiens cystatin C (CST3), mRNA [NM_000099]	1471
A_23_P170453	-3.4	1.2	-1.0	1.0	CST5	Homo sapiens cystatin D (CST5), mRNA [NM_001900]	1473
A_23_P146946	-1.0	1.5	-3.0	-1.1	CST6	Homo sapiens cystatin E/M (CST6), mRNA [NM_001323]	1474
A_32_P81879	1.0	-1.0	-7.3	-71.4	CST9	Homo sapiens cystatin 9 (testatin) (CST9), mRNA [NM_001008693]	128822
A_23_P154894	-5.6	-1.0	-1.1	1.5	CSTB	Homo sapiens cystatin B (stefin B) (CSTB), mRNA [NM_000100]	1476

A_24_P941051	-2.0	-1.7	-1.8	-2.1	CSTF2T	Homo sapiens cleavage stimulation factor, 3' pre-RNA, subunit 2, 64kDa, tau variant (CSTF2T), mRNA [NM_015235]	23283
A_32_P207124	2.7	-1.3	1.4	2.9	CT47A11	Homo sapiens cancer/testis antigen family 47, member A11 (CT47A11), mRNA [NM_173571]	255313
A_23_P22696	1.3	1.0	-2.2	1.1	CTAG2	Homo sapiens cancer/testis antigen 2 (CTAG2), transcript variant 2, mRNA [NM_020994]	30848

A_23_P128773	1.0	-2.3	-1.1	-1.1	CTAGE5	Homo sapiens CTAGE family, member 5 (CTAGE5), transcript variant 4, mRNA [NM_203356]	4253
A_32_P116840	1.0	1.0	1.0	-2.2	CTAGE5	Homo sapiens CTAGE family, member 5 (CTAGE5), transcript variant 4, mRNA [NM_203356]	4253
A_23_P348936	2.0	2.1	2.1	1.0	CTAGE7P	Homo sapiens CTAGE family, member 7, pseudogene (CTAGE7P), non-coding RNA [NR_044994]	119437
A_32_P167856	1.1	-1.1	1.4	-3.1	CTAGE9	Homo sapiens CTAGE family, member 9 (CTAGE9), mRNA [NM_001145659]	643854

A_32_P118655	1.1	-8.7	-1.3	-1.4	CTBP1	Homo sapiens mRNA; cDNA DKFZp434 B0914 (from clone DKFZp434 B0914). [AL137653]	1487
A_23_P41286	3.1	3.4	2.9	2.6	CTBP1	Homo sapiens C-terminal binding protein 1 (CTBP1), transcript variant 2, mRNA [NM_0010 12614]	1487
A_32_P144708	-1.3	-2.9	-1.5	-1.4	CTBP2	Homo sapiens C-terminal binding protein 2 (CTBP2), transcript variant 2, mRNA [NM_0228 02]	1488
A_24_P103922	2.8	4.6	2.1	2.0	CTBP2	Homo sapiens C-terminal binding protein 2 (CTBP2), transcript variant 1, mRNA [NM_0013 29]	1488

A_23_P78191	-1.0	-1.0	-2.4	-1.0	CTC1	Homo sapiens CTS telomere maintenance complex component 1 (CTC1), mRNA [NM_025099]	80169
A_24_P347704	-3.6	-1.2	-1.3	-1.3	CTCF	Homo sapiens CCCTC-binding factor (zinc finger protein) (CTCF), transcript variant 1, mRNA [NM_006565]	10664
A_24_P356254	13.6	21.6	7.9	11.0	CTCF	Homo sapiens CCCTC-binding factor (zinc finger protein) (CTCF), transcript variant 1, mRNA [NM_006565]	10664

A_23_P101111	2.1	1.5	1.9	1.0	CTDP1	Homo sapiens CTD (carboxy-terminal domain, RNA polymerase II, polypeptide A) phosphatase, subunit 1 (CTDP1), transcript variant 1, mRNA [NM_004715]	9150
A_24_P97197	-5.1	-2.6	-3.7	-4.6	CTDSP1	Homo sapiens CTD (carboxy-terminal domain, RNA polymerase II, polypeptide A) small phosphatase 1 (CTDSP1), transcript variant 2, mRNA [NM_182642]	58190

A_23_P28263	4.3	1.7	3.9	2.1	CTDSP1	Homo sapiens CTD (carboxy-terminal domain, RNA polymerase II, polypeptide A) small phosphatase 1 (CTDSP1), transcript variant 1, mRNA [NM_021198]	58190
A_23_P212436	-4.7	-4.9	-5.1	-4.8	CTDSPL	Homo sapiens CTD (carboxy-terminal domain, RNA polymerase II, polypeptide A) small phosphatase-like (CTDSPL), transcript variant 1, mRNA [NM_001008392]	10217

A_32_P76060	-1.2	1.5	-2.6	-2.3	CTDSPL2	Homo sapiens CTD (carboxy-terminal domain, RNA polymerase II, polypeptide A) small phosphatase like 2 (CTDSPL2), mRNA [NM_016396]	51496
A_23_P111888	1.0	-1.0	-4.7	-1.5	CTHRC1	Homo sapiens collagen triple helix repeat containing 1 (CTHRC1), mRNA [NM_138455]	115908
A_23_P102481	1.4	2.9	1.6	-1.2	CTLA4	Homo sapiens cytotoxic T-lymphocyte-associated protein 4 (CTLA4), transcript variant 1, mRNA [NM_005214]	1493

A_23_P58647	-2.9	2.5	-1.1	-1.2	CTNNA1	Homo sapiens catenin (cadherin-associated protein), alpha 1, 102kDa (CTNNA1), mRNA [NM_001903]	1495
A_23_P84736	-1.6	-1.6	-2.7	-1.8	CTNNA2	Homo sapiens catenin (cadherin-associated protein), alpha 2 (CTNNA2), transcript variant 1, mRNA [NM_004389]	1496
A_23_P95080	2.3	1.5	3.1	1.9	CTNND1	Homo sapiens catenin (cadherin-associated protein), delta 1 (CTNND1), transcript variant 16, mRNA [NM_001206885]	1500

A_23_P10305	-2.0	-2.3	-3.2	-1.8	CTPS2	Homo sapiens CTP synthase II (CTPS2), transcript variant 2, mRNA [NM_175859]	56474
A_32_P86150	2.3	1.5	2.0	2.6	CTRB2	Homo sapiens chymotrypsinogen B2 (CTRB2), mRNA [NM_001025200]	440387
A_23_P34852	1.5	1.2	-2.2	-1.0	CTRC	Homo sapiens chymotrypsin C (caldecrin) (CTRC), mRNA [NM_007272]	11330
A_24_P74374	-2.1	3.3	2.9	2.3	CTSA	Homo sapiens cathepsin A (CTSA), transcript variant 1, mRNA [NM_000308]	5476
A_24_P74371	3.5	2.9	2.2	2.1	CTSA	Homo sapiens cathepsin A (CTSA), transcript variant 1, mRNA [NM_000308]	5476

A_24_P303770	2.5	7.4	2.4	-2.0	CTSB	Homo sapiens cathepsin B (CTSB), transcript variant 2, mRNA [NM_147780]	1508
A_23_P215944	-1.3	-18.7	1.5	-10.2	CTSB	Homo sapiens cathepsin B (CTSB), transcript variant 2, mRNA [NM_147780]	1508
A_23_P1552	4.8	7.1	4.4	5.5	CTSC	Homo sapiens cathepsin C (CTSC), transcript variant 1, mRNA [NM_001814]	1075
A_23_P52556	2.0	1.7	1.1	1.3	CTSD	Homo sapiens cathepsin D (CTSD), mRNA [NM_001909]	1509
A_23_P10291	-1.1	2.7	2.6	2.8	CTSE	Homo sapiens cathepsin E (CTSE), transcript variant 1, mRNA [NM_001910]	1510

A_23_P34744	1.8	1.3	1.2	2.1	CTSK	Homo sapiens cathepsin K (CTSK), mRNA [NM_000396]	1513
A_23_P146456	-1.9	-2.0	-3.0	-2.0	CTSL2	Homo sapiens cathepsin L2 (CTSL2), transcript variant 1, mRNA [NM_001333]	1515
A_23_P13031	5.8	1.8	3.5	4.1	CTSW	Homo sapiens cathepsin W (CTSW), mRNA [NM_001335]	1521
A_24_P353740	1.3	2.5	-1.1	2.7	CTTNBP2NL	Homo sapiens CTTNBP2 N-terminal like (CTTNBP2NL), mRNA [NM_018704]	55917

A_24_P118247	-1.7	2.5	1.5	-1.6	CTU2	Homo sapiens cytosolic thiouridylase subunit 2 homolog (S. pombe) (CTU2), transcript variant 2, mRNA [NM_001012762]	348180
A_32_P155016	-1.7	1.4	-2.2	-1.0	CTXN1	Homo sapiens cortexin 1 (CTXN1), mRNA [NM_206833]	404217
A_24_P303589	3.4	6.3	-1.1	3.0	CUL1	Homo sapiens cullin 1 (CUL1), mRNA [NM_003592]	8454
A_24_P252794	1.0	1.0	-1.5	-2.9	CUL2	Homo sapiens cullin 2 (CUL2), transcript variant 3, mRNA [NM_003591]	8453
A_24_P140030	4.6	1.7	1.2	-3.4	CUL3	Homo sapiens cullin 3 (CUL3), mRNA [NM_003590]	8452

A_23_P151471	-2.3	-2.3	-1.4	-1.9	CUL4A	Homo sapiens cullin 4A (CUL4A), transcript variant 1, mRNA [NM_001008895]	8451
A_24_P61467	1.0	3.5	-1.1	-1.1	CUL4A	Homo sapiens cullin 4A (CUL4A), transcript variant 1, mRNA [NM_001008895]	8451
A_32_P134167	2.2	2.2	2.0	1.3	CUL5	602299712F1 NIH_MGC_87 Homo sapiens cDNA clone IMAGE:4394138 5', mRNA sequence [BG031574]	8065
A_23_P203009	-1.4	-2.5	-1.4	-1.4	CUL5	Homo sapiens cullin 5 (CUL5), mRNA [NM_003478]	8065
A_23_P311740	1.4	-1.5	2.1	-1.3	CUL9	Homo sapiens cullin 9 (CUL9), mRNA [NM_015089]	23113

A_23_P214678	2.5	2.0	2.1	1.1	CUTA	Homo sapiens cutA divalent cation tolerance homolog (E. coli) (CUTA), transcript variant 2, mRNA [NM_015921]	51596
A_23_P98015	-1.3	-2.3	-1.5	-1.2	CUTC	Homo sapiens cutC copper transporter homolog (E. coli) (CUTC), mRNA [NM_015960]	51076
A_24_P691604	-13.9	-1.0	1.0	-1.0	CUX1	Homo sapiens cut-like homeobox 1 (CUX1), transcript variant 4, mRNA [NM_001202543]	1523

A_23_P47058	2.0	1.2	-1.0	1.8	CUZD1	Homo sapiens CUB and zona pellucida-like domains 1 (CUZD1), transcript variant 1, mRNA [NM_022034]	50624
A_24_P163845	2.1	2.0	1.9	-2.8	CWF19L1	Homo sapiens CWF19-like 1, cell cycle control (S. pombe) (CWF19L1), mRNA [NM_018294]	55280
A_23_P35354	2.1	2.8	2.3	1.5	CWF19L1	Homo sapiens CWF19-like 1, cell cycle control (S. pombe) (CWF19L1), mRNA [NM_018294]	55280
A_23_P161719	-2.4	1.4	-3.4	-1.1	CWF19L2	Homo sapiens CWF19-like 2, cell cycle control (S. pombe) (CWF19L2), mRNA [NM_152434]	143884

A_24_P381901	-1.6	-1.2	-6.7	-1.1	CX3CL1	Homo sapiens chemokine (C-X3-C motif) ligand 1 (CX3CL1), mRNA [NM_002996]	6376
A_24_P374943	-9.4	1.0	1.1	1.0	CXADR	Homo sapiens coxsackie virus and adenovirus receptor (CXADR), transcript variant 1, mRNA [NM_001338]	1525
A_23_P7144	-1.2	-3.2	-1.2	-1.2	CXCL1	Homo sapiens chemokine (C-X-C motif) ligand 1 (melanoma growth stimulating activity, alpha) (CXCL1), mRNA [NM_001511]	2919
A_24_P944054	-1.0	1.9	2.6	1.5	CXCL12	Homo sapiens mRNA for FLJ00404 protein. [AK090482]	6387

A_24_P412156	1.0	-4.6	-1.0	-1.9	CXCL12	Homo sapiens chemokine (C-X-C motif) ligand 12 (CXCL12), transcript variant 2, mRNA [NM_000609]	6387
A_23_P121695	-1.1	-2.5	-1.1	-1.0	CXCL13	Homo sapiens chemokine (C-X-C motif) ligand 13 (CXCL13), mRNA [NM_006419]	10563
A_23_P38505	-1.1	1.8	-1.4	-3.4	CXCL16	Homo sapiens chemokine (C-X-C motif) ligand 16 (CXCL16), transcript variant 1, mRNA [NM_022059]	58191
A_24_P257416	1.6	2.3	2.3	1.1	CXCL2	Homo sapiens chemokine (C-X-C motif) ligand 2 (CXCL2), mRNA [NM_002089]	2920

A_24_P251764	1.5	2.4	2.6	-1.0	CXCL3	Homo sapiens chemokine (C-X-C motif) ligand 3 (CXCL3), mRNA [NM_002090]	2921
A_23_P18452	-1.4	-1.4	-2.0	-1.7	CXCL9	Homo sapiens chemokine (C-X-C motif) ligand 9 (CXCL9), mRNA [NM_002416]	4283
A_24_P252945	-1.0	-1.0	-25.4	-1.0	CXCR5	Homo sapiens chemokine (C-X-C motif) receptor 5 (CXCR5), transcript variant 2, mRNA [NM_032966]	643
A_23_P203215	4.8	1.0	-1.6	2.0	CXCR5	Homo sapiens chemokine (C-X-C motif) receptor 5 (CXCR5), transcript variant 2, mRNA [NM_032966]	643

A_23_P141779	2.4	1.7	1.5	2.1	CXXC1	Homo sapiens CXXC finger protein 1 (CXXC1), transcript variant 2, mRNA [NM_014593]	30827
A_24_P930062	-1.6	1.1	-1.1	-7.0	CXXC5	Homo sapiens CXXC finger protein 5 (CXXC5), mRNA [NM_016463]	51523
A_23_P399001	1.8	2.3	-2.1	-1.8	CXXC5	Homo sapiens CXXC finger protein 5 (CXXC5), mRNA [NM_016463]	51523
A_24_P348885	2.0	-2.2	1.7	-1.0	CYB561D1	Homo sapiens cytochrome b-561 domain containing 1 (CYB561D1), transcript variant 2, mRNA [NM_182580]	284613

A_23_P121326	-2.4	1.0	2.0	1.1	CYB561D2	Homo sapiens cytochrome b-561 domain containing 2 (CYB561D2), mRNA [NM_007022]	11068
A_23_P101208	5.1	1.3	3.5	-1.2	CYB5A	Homo sapiens cytochrome b5 type A (microsomal) (CYB5A), transcript variant 2, mRNA [NM_001914]	1528
A_23_P52101	1.1	-2.1	-1.7	1.5	CYB5R1	Homo sapiens cytochrome b5 reductase 1 (CYB5R1), mRNA [NM_016243]	51706
A_24_P100277	-1.1	1.4	-2.1	-19.7	CYB5R3	Homo sapiens cytochrome b5 reductase 3 (CYB5R3), transcript variant 2, mRNA [NM_007326]	1727

A_23_P163506	2.4	1.1	1.5	1.4	CYBA	Homo sapiens cytochrome b-245, alpha polypeptide (CYBA), mRNA [NM_000101]	1535
A_23_P209564	1.4	2.8	-1.0	-1.1	CYBRD1	Homo sapiens cytochrome b reductase 1 (CYBRD1), transcript variant 1, mRNA [NM_024843]	79901
A_23_P20980	1.1	-1.4	-2.8	-1.0	CYC1	Homo sapiens cytochrome c-1 (CYC1), nuclear gene encoding mitochondrial protein, mRNA [NM_001916]	1537

A_24_P29665	-7.3	-5.4	-10.4	-5.1	CYCS	Homo sapiens cytochrome c, somatic (CYCS), nuclear gene encoding mitochondrial protein, mRNA [NM_018947]	54205
A_23_P156117	5.7	2.9	1.7	2.8	CYFIP2	Homo sapiens cytoplasmic FMR1 interacting protein 2 (CYFIP2), transcript variant 2, mRNA [NM_001037332]	26999
A_23_P123555	-1.0	1.8	2.3	-1.1	CYHR1	Homo sapiens cysteine/histidine-rich 1 (CYHR1), transcript variant 2, mRNA [NM_032687]	50626

A_24_P89843	1.1	-2.3	1.3	1.1	CYHR1	Homo sapiens cysteine/histidine-rich 1 (CYHR1), transcript variant 2, mRNA [NM_032687]	50626
A_23_P398197	-4.9	-1.0	-1.0	-1.0	CYLC1	Homo sapiens cylicin, basic protein of sperm head cytoskeleton 1 (CYLC1), mRNA [NM_021118]	1538
A_24_P48078	-1.0	1.4	1.7	2.4	CYLD	Homo sapiens cylindromatosis (turban tumor syndrome) (CYLD), transcript variant 1, mRNA [NM_015247]	1540

A_32_P209325	2.8	4.1	-1.5	-1.3	CYP11A1	cytochrome P450, family 11, subfamily A, polypeptide 1 [Source:HGNC Symbol;Acc:2590] [ENST00000467407]	1583
A_23_P168928	2.1	2.1	1.0	-1.0	CYP11B1	Homo sapiens cytochrome P450, family 11, subfamily B, polypeptide 1 (CYP11B1), nuclear gene encoding mitochondrial protein, transcript variant 1, mRNA [NM_000497]	1584

A_24_P329424	-1.0	-2.1	-1.0	-1.1	CYP11B1	Homo sapiens cytochrome P450, family 11, subfamily B, polypeptide 1 (CYP11B1), nuclear gene encoding mitochondrial protein, transcript variant 1, mRNA [NM_000497]	1584
A_23_P206110	-5.5	1.0	1.4	1.2	CYP1A2	Homo sapiens cytochrome P450, family 1, subfamily A, polypeptide 2 (CYP1A2), mRNA [NM_000761]	1544
A_23_P206113	-1.3	-2.1	-1.0	-2.7	CYP1A2	Homo sapiens cytochrome P450, family 1, subfamily A, polypeptide 2 (CYP1A2), mRNA [NM_000761]	1544

A_32_P361884	-1.4	-1.6	-4.2	-1.8	CYP1B1-AS1	Homo sapiens CYP1B1 antisense RNA 1 (non-protein coding) (CYP1B1-AS1), non-coding RNA [NR_027252]	285154
A_32_P2442	-2.5	-1.0	-1.1	1.2	CYP20A1	Homo sapiens cytochrome P450, family 20, subfamily A, polypeptide 1 (CYP20A1), mRNA [NM_177538]	57404
A_32_P110433	-31.0	1.1	1.1	1.1	CYP20A1	Homo sapiens cytochrome P450, family 20, subfamily A, polypeptide 1 (CYP20A1), mRNA [NM_177538]	57404

A_23_P56894	-5.2	-1.7	-1.7	-1.5	CYP20A1	Homo sapiens cytochrome P450, family 20, subfamily A, polypeptide 1 (CYP20A1), mRNA [NM_177538]	57404
A_23_P257478	-3.7	2.3	2.0	-2.1	CYP21A2	Homo sapiens cytochrome P450, family 21, subfamily A, polypeptide 2 (CYP21A2), transcript variant 1, mRNA [NM_000500]	1589
A_23_P210109	-1.0	-2.8	-1.6	-1.0	CYP26B1	Homo sapiens cytochrome P450, family 26, subfamily B, polypeptide 1 (CYP26B1), mRNA [NM_019885]	56603

A_23_P131308	1.2	-1.4	-2.0	1.2	CYP27A1	Homo sapiens cytochrome P450, family 27, subfamily A, polypeptide 1 (CYP27A1), nuclear gene encoding mitochondrial protein, mRNA [NM_000784]	1593
A_23_P55779	1.4	3.0	-3.0	1.9	CYP2A13	Homo sapiens cytochrome P450, family 2, subfamily A, polypeptide 13 (CYP2A13), mRNA [NM_000766]	1553
A_23_P27528	-7.0	-1.3	-3.5	-2.9	CYP2A7	Homo sapiens cytochrome P450, family 2, subfamily A, polypeptide 7 (CYP2A7), transcript variant 1, mRNA [NM_000764]	1549

A_23_P158481	-1.5	1.0	-2.7	1.0	CYP2C19	Homo sapiens cytochrome P450, family 2, subfamily C, polypeptide 19 (CYP2C19), mRNA [NM_000769]	1557
A_23_P161368	1.9	2.2	-1.5	1.7	CYP2C8	Homo sapiens cytochrome P450, family 2, subfamily C, polypeptide 8 (CYP2C8), transcript variant 1, mRNA [NM_000770]	1558
A_23_P143734	1.1	-5.5	1.2	-1.9	CYP2D6	Homo sapiens cytochrome P450, family 2, subfamily D, polypeptide 6 (CYP2D6), transcript variant 1, mRNA [NM_000106]	1565

A_23_P155123	3.3	2.9	1.8	1.8	CYP2D6	Homo sapiens cytochrome P450, family 2, subfamily D, polypeptide 6 (CYP2D6), transcript variant 1, mRNA [NM_000106]	1565
A_24_P394940	-2.2	1.0	-2.1	-1.1	CYP2E1	Homo sapiens cytochrome P450, family 2, subfamily E, polypeptide 1 (CYP2E1), mRNA [NM_000773]	1571
A_23_P89981	1.8	2.3	4.0	2.3	CYP2F1	Homo sapiens cytochrome P450, family 2, subfamily F, polypeptide 1 (CYP2F1), mRNA [NM_000774]	1572

A_23_P103486	-21.4	-1.0	-1.2	-1.1	CYP2J2	Homo sapiens cytochrome P450, family 2, subfamily J, polypeptide 2 (CYP2J2), mRNA [NM_000775]	1573
A_24_P913156	-1.0	3.1	2.0	2.5	CYP2U1	cytochrome P450, family 2, subfamily U, polypeptide 1 [Source:HGNC Symbol;Acc:20582] [ENST00000513302]	113612
A_23_P155835	7.1	6.6	6.4	6.8	CYP2U1	Homo sapiens cytochrome P450, family 2, subfamily U, polypeptide 1 (CYP2U1), mRNA [NM_183075]	113612

A_23_P133712	-1.5	4.8	1.9	1.3	CYP39A1	Homo sapiens cytochrome P450, family 39, subfamily A, polypeptide 1 (CYP39A1), mRNA [NM_016593]	51302
A_23_P71258	-2.0	-4.5	-3.9	-2.5	CYP3A43	Homo sapiens cytochrome P450, family 3, subfamily A, polypeptide 43 (CYP3A43), transcript variant 3, mRNA [NM_057096]	64816
A_23_P215828	1.3	-1.4	-2.5	-2.2	CYP3A43	Homo sapiens cytochrome P450, family 3, subfamily A, polypeptide 43 (CYP3A43), transcript variant 1, mRNA [NM_022820]	64816

A_23_P8801	3.7	3.6	6.9	3.7	CYP3A5	Homo sapiens cytochrome P450, family 3, subfamily A, polypeptide 5 (CYP3A5), transcript variant 1, mRNA [NM_000777]	1577
A_24_P916916	-2.1	-1.8	-2.6	-2.5	CYP3A5	Homo sapiens cytochrome P450, family 3, subfamily A, polypeptide 5 (CYP3A5), transcript variant 2, mRNA [NM_001190484]	1577
A_23_P48784	1.3	2.4	1.2	1.1	CYP46A1	Homo sapiens cytochrome P450, family 46, subfamily A, polypeptide 1 (CYP46A1), mRNA [NM_006668]	10858

A_23_P97562	2.2	1.3	-1.2	1.4	CYP4A11	CYP4A11=fatty acid omega-hydroxylase [human, kidney, mRNA, 2470 nt]. [S67580]	1579
A_24_P191013	-1.3	-1.4	-7.2	1.1	CYP4A11	Homo sapiens cytochrome P450, family 4, subfamily A, polypeptide 11 (CYP4A11), mRNA [NM_000778]	1579
A_23_P114713	3.0	2.8	1.8	2.0	CYP4B1	Homo sapiens cytochrome P450, family 4, subfamily B, polypeptide 1 (CYP4B1), transcript variant 2, mRNA [NM_000779]	1580

A_24_P168495	1.4	1.6	-2.2	1.2	CYP4F2	Homo sapiens cytochrome P450, family 4, subfamily F, polypeptide 2 (CYP4F2), mRNA [NM_001082]	8529
A_24_P28811	1.5	1.6	1.8	-10.0	CYP4F30P	Homo sapiens cytochrome P450, family 4, subfamily F, polypeptide 30, pseudogene (CYP4F30P), non-coding RNA [NR_023391]	100132708
A_24_P945228	-1.0	-6.8	-1.2	-1.5	CYP4V2	Homo sapiens cytochrome P450, family 4, subfamily V, polypeptide 2 (CYP4V2), mRNA [NM_207352]	285440

A_23_P72411	1.4	1.0	-2.6	2.2	CYP4X1	Homo sapiens cytochrome P450, family 4, subfamily X, polypeptide 1 (CYP4X1), mRNA [NM_178033]	260293
A_23_P103971	5.4	1.3	1.7	1.3	CYP4Z1	Homo sapiens cytochrome P450, family 4, subfamily Z, polypeptide 1 (CYP4Z1), mRNA [NM_178134]	199974
A_24_P145529	2.1	2.1	2.0	1.4	CYP4Z2P	Homo sapiens cytochrome P450, family 4, subfamily Z, polypeptide 2 pseudogene (CYP4Z2P), non-coding RNA [NR_002788]	163720

A_23_P169092	-1.1	-1.0	-2.4	-1.7	CYP7B1	Homo sapiens cytochrome P450, family 7, subfamily B, polypeptide 1 (CYP7B1), mRNA [NM_004820]	9420
A_23_P46426	-1.0	-4.2	-1.4	-1.8	CYR61	Homo sapiens cysteine-rich, angiogenic inducer, 61 (CYR61), mRNA [NM_001554]	3491
A_23_P22660	3.6	4.3	2.3	2.2	CYSLTR1	Homo sapiens cysteinyl leukotriene receptor 1 (CYSLTR1), mRNA [NM_006639]	10800
A_23_P319466	-2.1	1.0	-1.6	1.3	CYSLTR2	Homo sapiens cysteinyl leukotriene receptor 2 (CYSLTR2), mRNA [NM_020377]	57105

A_24_P551842	1.5	1.6	1.4	-2.4	CYTB	mitochondrially encoded cytochrome b [Source:HGNC Symbol;Acc:7427] [ENST00000361789]	4519
A_24_P557534	-1.0	-4.3	-2.1	-2.3	CYTB	mitochondrially encoded cytochrome b [Source:HGNC Symbol;Acc:7427] [ENST00000361789]	4519
A_24_P374918	-1.4	-1.2	-1.3	-2.4	CYTH2	cytohesin 2 [Source:HGNC Symbol;Acc:9502] [ENST00000467412]	9266
A_23_P155057	1.0	-1.2	-2.7	-8.5	CYTH4	Homo sapiens cytohesin 4 (CYTH4), mRNA [NM_013385]	27128

A_32_P6769	2.0	-1.3	1.1	2.1	D2HGDH	D-2-hydroxyglutarate dehydrogenase [Source:HGNC Symbol;Acc:28358] [ENST00000496252]	728294
A_23_P54116	2.1	2.1	2.1	-1.2	DAAM1	Homo sapiens dishevelled associated activator of morphogenesis 1 (DAAM1), mRNA [NM_014992]	23002
A_23_P23850	1.9	2.1	1.8	1.3	DAB1	Homo sapiens disabled homolog 1 (Drosophila) (DAB1), mRNA [NM_021080]	1600
A_24_P149704	1.2	1.3	-5.6	-2.8	DAB2IP	Homo sapiens DAB2 interacting protein (DAB2IP), transcript variant 2, mRNA [NM_138709]	153090

A_32_P464135	-1.0	-5.5	-1.1	-1.0	DACH2	Homo sapiens dachshund homolog 2 (Drosophila) (DACH2), transcript variant 1, mRNA [NM_053281]	117154
A_23_P65518	-4.0	-1.1	1.5	-3.1	DACT1	Homo sapiens dapper, antagonist of beta-catenin, homolog 1 (Xenopus laevis) (DACT1), transcript variant 1, mRNA [NM_016651]	51339
A_24_P284093	4.1	1.3	1.8	2.0	DACT1	Homo sapiens dapper, antagonist of beta-catenin, homolog 1 (Xenopus laevis) (DACT1), transcript variant 1, mRNA [NM_016651]	51339

A_24_P289260	-7.8	-1.1	-1.0	-2.2	DACT2	Homo sapiens dapper, antagonist of beta-catenin, homolog 2 (Xenopus laevis) (DACT2), mRNA [NM_214462]	168002
A_23_P360964	-3.4	-1.9	-3.2	1.2	DACT3	Homo sapiens dapper, antagonist of beta-catenin, homolog 3 (Xenopus laevis) (DACT3), mRNA [NM_145056]	147906
A_24_P33014	8.6	3.5	6.6	6.2	DACT3	Homo sapiens dapper, antagonist of beta-catenin, homolog 3 (Xenopus laevis) (DACT3), mRNA [NM_145056]	147906

A_24_P47870	1.0	-1.0	-1.3	-2.8	DAD1	Homo sapiens defender against cell death 1 (DAD1), mRNA [NM_001344]	1603
A_24_P225961	2.4	1.9	2.6	1.1	DAG1	Homo sapiens dystroglycan 1 (dystrophin-associated glycoprotein 1) (DAG1), transcript variant 2, mRNA [NM_004393]	1605
A_23_P135611	1.1	2.6	-1.2	1.7	DALRD3	Homo sapiens DALR anticodon binding domain containing 3 (DALRD3), transcript variant 2, mRNA [NM_018114]	55152

A_23_P435636	-4.3	1.1	-1.1	1.0	DAND5	Homo sapiens DAN domain family, member 5 (DAND5), mRNA [NM_152654]	199699
A_23_P139635	1.1	7.5	-1.1	-1.0	DAO	Homo sapiens D-amino-acid oxidase (DAO), mRNA [NM_001917]	1610
A_24_P155617	2.4	2.2	2.6	-1.9	DAOA	Homo sapiens D-amino acid oxidase activator (DAOA), transcript variant 1, mRNA [NM_172370]	267012
A_23_P306240	-1.1	-1.1	-1.2	-2.0	DAOA-AS1	Homo sapiens DAOA antisense RNA 1 (non-protein coding) (DAOA-AS1), non-coding RNA [NR_040247]	282706

A_24_P243278	2.8	1.7	7.0	2.7	DAP	Homo sapiens death-associated protein (DAP), mRNA [NM_004394]	1611
A_24_P10233	1.8	-1.3	-1.7	3.3	DAPK2	Homo sapiens death-associated protein kinase 2 (DAPK2), mRNA [NM_014326]	23604
A_23_P165028	1.0	-4.2	1.5	-1.0	DAPK3	Homo sapiens death-associated protein kinase 3 (DAPK3), mRNA [NM_001348]	1613
A_23_P255444	1.3	1.3	-1.6	-3.6	DAPP1	Homo sapiens dual adaptor of phosphotyrosine and 3-phosphoinositides (DAPP1), mRNA [NM_014395]	27071

A_23_P148984	-27.7	1.0	1.0	1.2	DARS2	Homo sapiens aspartyl-tRNA synthetase 2, mitochondrial (DARS2), nuclear gene encoding mitochondrial protein, mRNA [NM_018122]	55157
A_32_P504673	-1.2	-1.3	-4.8	-1.2	DAZ2	Homo sapiens deleted in azoospermia 2 (DAZ2), transcript variant 3, mRNA [NM_001005786]	57055
A_24_P921906	-1.6	-1.4	-2.7	-1.7	DBIL5P2	Homo sapiens diazepam binding inhibitor-like 5 pseudogene 2 (DBIL5P2), non-coding RNA [NR_036635]	100169989

A_23_P156284	-1.4	-2.7	-1.5	1.1	DBN1	Homo sapiens drebrin 1 (DBN1), transcript variant 2, mRNA [NM_080881]	1627
A_23_P26439	-1.5	1.9	2.0	1.1	DBNDD1	Homo sapiens dysbindin (dystrobrevin binding protein 1) domain containing 1 (DBNDD1), transcript variant 1, mRNA [NM_001042610]	79007
A_23_P28772	-1.4	-1.5	-2.6	1.2	DBNDD2	Homo sapiens dysbindin (dystrobrevin binding protein 1) domain containing 2 (DBNDD2), transcript variant 6, mRNA [NM_001048226]	55861

A_23_P130753	1.1	-1.1	-1.2	-3.2	DBP	Homo sapiens D site of albumin promoter (albumin D-box) binding protein (DBP), mRNA [NM_001352]	1628
A_23_P130761	1.9	1.4	1.3	2.1	DBP	Human albumin D-box binding protein (DBP) mRNA, complete cds. [U06936]	1628
A_32_P50005	-1.3	-2.0	-1.4	-1.1	DBT	Homo sapiens dihydrolipoamide branched chain transacylase E2 (DBT), nuclear gene encoding mitochondrial protein, mRNA [NM_001918]	1629

A_24_P149766	90.5	90.6	89.4	29.0	DCAF10	Homo sapiens DDB1 and CUL4 associated factor 10 (DCAF10), mRNA [NM_024345]	79269
A_32_P133005	-1.0	-2.9	-1.0	-1.0	DCAF10	Homo sapiens DDB1 and CUL4 associated factor 10 (DCAF10), mRNA [NM_024345]	79269
A_23_P215875	1.1	1.1	-6.1	1.8	DCAF13	Homo sapiens DDB1 and CUL4 associated factor 13 (DCAF13), mRNA [NM_015420]	25879
A_23_P50399	2.1	2.0	2.9	1.5	DCAF15	Homo sapiens DDB1 and CUL4 associated factor 15 (DCAF15), mRNA [NM_138353]	90379

A_24_P248167	1.0	-2.5	-1.1	1.0	DCAF4	Homo sapiens mRNA; cDNA DKFZp434 K114 (from clone DKFZp434 K114). [AL080157]	26094
A_23_P352684	-3.3	-1.4	-1.8	-1.1	DCAF5	Homo sapiens DDB1 and CUL4 associated factor 5 (DCAF5), nuclear gene encoding mitochond rial protein, mRNA [NM_0038 61]	8816
A_23_P160481	-3.4	1.0	-1.0	1.1	DCAF6	Homo sapiens DDB1 and CUL4 associated factor 6 (DCAF6), transcript variant 1, mRNA [NM_0184 42]	55827

A_23_P422268	1.6	-1.5	-6.1	1.4	DCAF7	Homo sapiens DDB1 and CUL4 associated factor 7 (DCAF7), mRNA [NM_005828]	10238
A_24_P916141	1.3	2.5	-1.5	-1.0	DCAF7	Homo sapiens DDB1 and CUL4 associated factor 7 (DCAF7), mRNA [NM_005828]	10238
A_23_P200143	1.3	-1.7	-2.6	-2.2	DCAF8	Homo sapiens DDB1 and CUL4 associated factor 8 (DCAF8), transcript variant 1, mRNA [NM_015726]	50717
A_24_P76725	-2.4	1.0	1.4	1.6	DCAF8	Homo sapiens DDB1 and CUL4 associated factor 8 (DCAF8), transcript variant 4, non-coding RNA [NR_028105]	50717

A_24_P82493	1.1	1.5	2.2	1.4	DCAKD	Homo sapiens dephospho-CoA kinase domain containing (DCAKD), transcript variant 1, mRNA [NM_024819]	79877
A_24_P243633	1.1	-2.2	1.1	1.6	DCBLD1	Homo sapiens discoidin, CUB and LCCL domain containing 1 (DCBLD1), mRNA [NM_173674]	285761
A_23_P409462	-11.3	-1.0	-1.1	-1.0	DCBLD1	Homo sapiens discoidin, CUB and LCCL domain containing 1 (DCBLD1), mRNA [NM_173674]	285761

A_23_P258234	-11.4	2.4	1.7	-11.0	DCBLD1	discoidin, CUB and LCCL domain containing 1 [Source:HGNC Symbol;Acc:21479] [ENST00000338728]	285761
A_24_P51061	-7.9	-1.1	-1.6	-1.1	DCBLD2	Homo sapiens discoidin, CUB and LCCL domain containing 2 (DCBLD2), mRNA [NM_080927]	131566
A_24_P117663	1.0	-9.8	-1.1	-1.1	DCDC1	Homo sapiens doublecortin domain containing 1 (DCDC1), mRNA [NM_181807]	341019
A_23_P98645	-1.0	-1.1	-3.0	-1.1	DCHS1	Homo sapiens dachsous 1 (Drosophila) (DCHS1), mRNA [NM_003737]	8642

A_23_P144490	-1.8	-1.9	-12.1	-1.8	DCHS2	Homo sapiens dachsous 2 (Drosophila) (DCHS2), transcript variant 1, mRNA [NM_017639]	54798
A_32_P108889	-3.3	-8.1	-6.9	-2.5	DCLK1	Homo sapiens doublecortin-like kinase 1 (DCLK1), transcript variant 1, mRNA [NM_004734]	9201
A_23_P86632	2.2	2.2	2.0	2.2	DCLRE1C	Homo sapiens DNA cross-link repair 1C (DCLRE1C), transcript variant c, mRNA [NM_001033858]	64421
A_23_P64873	-4.6	-1.3	-1.5	1.0	DCN	Homo sapiens decorin (DCN), transcript variant A1, mRNA [NM_001920]	1634

A_23_P313512	-2.7	1.0	1.0	1.1	DCP1B	Homo sapiens DCP1 decapping enzyme homolog B (S. cerevisiae) (DCP1B), mRNA [NM_152640]	196513
A_23_P314798	1.4	1.7	-2.3	1.7	DCST2	Homo sapiens DC-STAMP domain containing 2 (DCST2), mRNA [NM_144622]	127579
A_23_P142835	1.8	-1.1	2.1	1.2	DCTN1	Homo sapiens dynactin 1 (DCTN1), transcript variant 1, mRNA [NM_004082]	1639
A_23_P360167	1.9	1.0	-1.7	2.4	DCTN2	Homo sapiens dynactin 2 (p50) (DCTN2), mRNA [NM_006400]	10540

A_24_P209765	1.3	2.9	2.2	1.6	DCTN3	Homo sapiens dynactin 3 (p22) (DCTN3), transcript variant 1, mRNA [NM_007234]	11258
A_24_P166012	-1.6	-3.0	-2.1	-1.2	DCUN1D1	Homo sapiens DCN1, defective in cullin neddylation 1, domain containing 1 (S. cerevisiae) (DCUN1D1), mRNA [NM_020640]	54165
A_24_P215628	1.0	1.0	-2.2	-1.3	DCUN1D2	Homo sapiens DCN1, defective in cullin neddylation 1, domain containing 2 (S. cerevisiae) (DCUN1D2), mRNA [NM_001014283]	55208

A_32_P43812	-1.0	-3.4	-1.1	-2.1	DCUN1D4	Homo sapiens DCN1, defective in cullin neddylation 1, domain containing 4 (S. cerevisiae) (DCUN1D4), transcript variant 1, mRNA [NM_001040402]	23142
A_24_P262738	6.0	3.2	2.3	3.8	DDA1	Homo sapiens DET1 and DDB1 associated 1 (DDA1), mRNA [NM_024050]	79016
A_23_P16671	5.1	8.5	4.2	-8.0	DDA1	Homo sapiens DET1 and DDB1 associated 1 (DDA1), mRNA [NM_024050]	79016

A_23_P52610	-2.5	-2.5	1.0	1.2	DDB2	Homo sapiens damage-specific DNA binding protein 2, 48kDa (DDB2), mRNA [NM_000107]	1643
A_24_P104278	-1.1	-4.8	-1.1	-1.1	DDHD1	Homo sapiens DDHD domain containing 1 (DDHD1), transcript variant 3, mRNA [NM_001160148]	80821
A_24_P911489	1.3	-3.9	-1.2	1.2	DDHD1	Homo sapiens DDHD domain containing 1 (DDHD1), transcript variant 1, mRNA [NM_030637]	80821
A_23_P205623	-30.0	1.5	1.5	-1.2	DDHD1	Homo sapiens DDHD domain containing 1 (DDHD1), transcript variant 1, mRNA [NM_030637]	80821

A_23_P401238	4.5	6.4	6.2	3.3	DDHD2	Homo sapiens DDHD domain containing 2 (DDHD2), transcript variant 1, mRNA [NM_015214]	23259
A_23_P116145	-1.0	-8.2	1.0	-1.0	DDI1	Homo sapiens DNA-damage inducible 1 homolog 1 (S. cerevisiae) (DDI1), mRNA [NM_001001711]	414301
A_23_P63114	1.6	1.0	1.0	2.7	DDI2	DNA-damage inducible 1 homolog 2 (S. cerevisiae) [Source:HGNC Symbol;Acc:24578] [ENST00000486680]	84301

A_32_P83547	2.3	2.3	1.9	1.4	DDI2	DNA-damage inducible 1 homolog 2 (S. cerevisiae) [Source:HGNC Symbol;Acc:24578] [ENST00000480945]	84301
A_24_P123601	-65.4	-1.6	-1.1	-1.0	DDR1	Homo sapiens discoidin domain receptor tyrosine kinase 1 (DDR1), transcript variant 2, mRNA [NM_013993]	780
A_23_P93311	-1.4	-4.9	1.2	1.0	DDR1	Homo sapiens discoidin domain receptor tyrosine kinase 1 (DDR1), transcript variant 2, mRNA [NM_013993]	780

A_23_P203949	3.8	2.3	1.5	2.3	DDX11	Homo sapiens DEAD/H (Asp-Glu-Ala-Asp/His) box polypeptide 11 (DDX11), transcript variant 1, mRNA [NM_030653]	1663
A_23_P154294	-2.8	-3.3	1.4	-1.2	DDX11L2	Homo sapiens DEAD/H (Asp-Glu-Ala-Asp/His) box polypeptide 11 like 2 (DDX11L2), transcript variant 2, non-coding RNA [NR_024005]	84771

A_32_P227317	1.0	-1.8	-2.6	1.0	DDX11L2	Homo sapiens DEAD/H (Asp-Glu-Ala-Asp/His) box polypeptide 11 like 2 (DDX11L2), transcript variant 2, non-coding RNA [NR_024005]	84771
A_24_P925611	1.4	-1.4	-1.3	-3.7	DDX17	Homo sapiens DEAD (Asp-Glu-Ala-Asp) box polypeptide 17 (DDX17), transcript variant 1, mRNA [NM_006386]	10521
A_23_P218784	2.6	2.8	-1.1	1.3	DDX17	Homo sapiens DEAD (Asp-Glu-Ala-Asp) box polypeptide 17 (DDX17), transcript variant 1, mRNA [NM_006386]	10521

A_23_P140994	2.4	1.6	1.7	1.6	DDX19B	Homo sapiens DEAD (Asp-Glu-Ala-As) box polypeptide 19B (DDX19B), transcript variant 1, mRNA [NM_007242]	11269
A_24_P146603	6.7	6.8	6.4	4.7	DDX21	Homo sapiens DEAD (Asp-Glu-Ala-Asp) box polypeptide 21 (DDX21), mRNA [NM_004728]	9188
A_23_P151544	1.2	2.2	1.0	-1.2	DDX24	Homo sapiens DEAD (Asp-Glu-Ala-Asp) box polypeptide 24 (DDX24), mRNA [NM_020414]	57062

A_23_P138910	-1.1	1.0	-2.8	-1.0	DDX25	Homo sapiens DEAD (Asp-Glu-Ala-Asp) box polypeptide 25 (DDX25), mRNA [NM_013264]	29118
A_23_P40194	-2.2	1.0	-1.1	1.0	DDX27	Homo sapiens DEAD (Asp-Glu-Ala-Asp) box polypeptide 27 (DDX27), mRNA [NM_017895]	55661
A_24_P112377	3.5	3.6	3.4	2.6	DDX31	Homo sapiens DEAD (Asp-Glu-Ala-Asp) box polypeptide 31 (DDX31), transcript variant 2, mRNA [NM_138620]	64794

A_23_P60405	4.0	2.4	1.6	1.3	DDX31	Homo sapiens DEAD (Asp-Glu-Ala-Asp) box polypeptide 31 (DDX31), transcript variant 1, mRNA [NM_022779]	64794
A_23_P78664	2.1	-1.0	1.1	1.4	DDX39A	Homo sapiens DEAD (Asp-Glu-Ala-Asp) box polypeptide 39A (DDX39A), transcript variant 1, mRNA [NM_005804]	10212
A_23_P317657	-2.0	-1.6	-2.1	1.0	DDX3X	Homo sapiens DEAD (Asp-Glu-Ala-Asp) box polypeptide 3, X-linked (DDX3X), transcript variant 1, mRNA [NM_001356]	1654

A_23_P122116	2.4	3.3	3.0	1.3	DDX41	Homo sapiens DEAD (Asp-Glu-Ala-Asp) box polypeptide 41 (DDX41), mRNA [NM_016222]	51428
A_23_P156445	-4.8	1.7	1.6	1.0	DDX43	Homo sapiens DEAD (Asp-Glu-Ala-Asp) box polypeptide 43 (DDX43), mRNA [NM_018665]	55510
A_23_P30377	3.8	3.7	-1.5	-1.5	DDX46	Homo sapiens DEAD (Asp-Glu-Ala-Asp) box polypeptide 46 (DDX46), mRNA [NM_014829]	9879

A_23_P65089	-2.0	-21.3	-1.2	-1.8	DDX47	Homo sapiens DEAD (Asp-Glu-Ala-Asp) box polypeptide 47 (DDX47), transcript variant 1, mRNA [NM_016355]	51202
A_23_P16573	-1.8	-1.2	-1.2	-4.4	DDX49	Homo sapiens DEAD (Asp-Glu-Ala-Asp) box polypeptide 49 (DDX49), transcript variant 1, mRNA [NM_019070]	54555
A_23_P104451	1.1	-4.4	1.0	1.0	DDX50	Homo sapiens DEAD (Asp-Glu-Ala-Asp) box polypeptide 50 (DDX50), mRNA [NM_024045]	79009

A_24_P512054	-1.0	-1.0	-3.9	1.1	DDX50	Homo sapiens DEAD (Asp-Glu-Ala-Asp) box polypeptide 50 (DDX50), mRNA [NM_024045]	79009
A_32_P430359	-1.0	2.1	-1.1	-1.1	DDX54	Homo sapiens DEAD (Asp-Glu-Ala-Asp) box polypeptide 54 (DDX54), transcript variant 2, mRNA [NM_024072]	79039
A_23_P47839	-1.0	-2.0	-1.1	-1.1	DDX55	Homo sapiens DEAD (Asp-Glu-Ala-Asp) box polypeptide 55 (DDX55), mRNA [NM_020936]	57696

A_24_P935622	1.4	-2.4	1.2	1.6	DDX56	DEAD (Asp-Glu-Ala-Asp) box polypeptide 56 [Source:HGNC Symbol;Acc:18193] [ENST00000421223]	54606
A_23_P20814	-3.7	-1.0	-1.2	1.1	DDX58	Homo sapiens DEAD (Asp-Glu-Ala-Asp) box polypeptide 58 (DDX58), mRNA [NM_014314]	23586
A_23_P203201	-2.9	1.4	1.5	1.4	DDX6	Homo sapiens DEAD (Asp-Glu-Ala-Asp) box polypeptide 6 (DDX6), mRNA [NM_004397]	1656

A_23_P41470	-3.5	-1.1	1.0	1.0	DDX60	Homo sapiens DEAD (Asp-Glu-Ala-Asp) box polypeptide 60 (DDX60), mRNA [NM_017631]	55601
A_32_P161292	-1.0	-3.9	-1.1	-1.0	DDX60L	Homo sapiens DEAD (Asp-Glu-Ala-Asp) box polypeptide 60-like (DDX60L), mRNA [NM_001012967]	91351
A_23_P158533	2.3	1.5	1.5	1.8	DEAF1	Homo sapiens deformed epidermal autoregulatory factor 1 (Drosophila) (DEAF1), mRNA [NM_021008]	10522

A_23_P124044	1.2	2.2	1.2	1.1	DEAF1	Homo sapiens deformed epidermal autoregulatory factor 1 (Drosophila) (DEAF1), mRNA [NM_021008]	10522
A_23_P74716	3.5	2.5	-1.7	2.7	DEDD	Homo sapiens death effector domain containing (DEDD), transcript variant 1, mRNA [NM_032998]	9191
A_24_P207479	1.2	-1.6	-2.1	1.0	DEDD2	Homo sapiens death effector domain containing 2 (DEDD2), mRNA [NM_133328]	162989

A_23_P88893	5.3	8.2	6.3	2.7	DEF8	Homo sapiens differentially expressed in FDCP 8 homolog (mouse) (DEF8), transcript variant 2, mRNA [NM_017702]	54849
A_24_P193582	-2.7	1.0	-1.0	1.1	DEF8	Homo sapiens differentially expressed in FDCP 8 homolog (mouse) (DEF8), transcript variant 2, mRNA [NM_017702]	54849
A_23_P169017	-4.8	-1.0	1.3	1.5	DEFB103B	Homo sapiens defensin, beta 103B (DEFB103B), mRNA [NM_018661]	55894
A_23_P380871	-1.1	5.7	-1.0	-1.0	DEFB105B	Homo sapiens defensin, beta 105B (DEFB105B), mRNA [NM_001040703]	504180

A_32_P71343	3.1	3.1	2.2	2.1	DEFB106B	Homo sapiens defensin, beta 106B (DEFB106 B), mRNA [NM_0010 40704]	503841
A_23_P312285	2.2	1.6	1.7	3.4	DEFB119	Homo sapiens defensin, beta 119 (DEFB119) , transcript variant 2, mRNA [NM_1734 60]	245932
A_32_P108107	-71.9	-2.1	1.0	1.9	DEFB119	Homo sapiens defensin, beta 119 (DEFB119) , transcript variant 3, mRNA [NM_1533 23]	245932
A_23_P356694	1.8	3.3	1.7	-1.8	DEFB123	Homo sapiens defensin, beta 123 (DEFB123) , mRNA [NM_1533 24]	245936
A_23_P320846	-1.2	-1.2	-4.7	-1.9	DEFB125	Homo sapiens defensin, beta 125 (DEFB125) , mRNA [NM_1533 25]	245938

A_23_P385843	-2.2	-1.0	1.0	-1.6	DEFB127	Homo sapiens defensin, beta 127 (DEFB127), mRNA [NM_139074]	140850
A_23_P102694	8.4	7.9	8.3	8.0	DEFB129	Homo sapiens defensin, beta 129 (DEFB129), mRNA [NM_080831]	140881
A_32_P23854	2.1	-2.9	-1.2	-1.1	DENND1A	Homo sapiens DENN/MADD domain containing 1A (DENND1A), transcript variant 1, mRNA [NM_020946]	57706
A_24_P272761	-1.3	-1.0	-1.1	-30.1	DENND1A	Homo sapiens DENN/MADD domain containing 1A (DENND1A), transcript variant 1, mRNA [NM_020946]	57706

A_23_P43557	1.2	1.6	1.1	-2.4	DENND1A	Homo sapiens DENN/MADD domain containing 1A (DENND1A), transcript variant 2, mRNA [NM_024820]	57706
A_24_P943283	-2.2	-2.3	-2.5	-3.1	DENND1B	DENN/MADD domain containing 1B [Source:HGNC Symbol;Acc:28404] [ENST00000496935]	163486
A_23_P78608	1.2	-1.7	-2.7	-3.3	DENND1C	Homo sapiens DENN/MADD domain containing 1C (DENND1C), mRNA [NM_024898]	79958
A_24_P334726	1.8	2.3	2.2	1.9	DENND2A	Homo sapiens DENN/MADD domain containing 2A (DENND2A), mRNA [NM_015689]	27147

A_23_P257583	2.9	2.7	3.0	1.5	DENND2A	Homo sapiens DENN/MADD domain containing 2A (DENND2A), mRNA [NM_015689]	27147
A_24_P6467	-1.8	-7.0	-1.8	-5.1	DENND2C	Homo sapiens DENN/MADD domain containing 2C (DENND2C), mRNA [NM_198459]	163259
A_23_P311346	-1.7	-1.4	-1.8	-5.2	DENND2D	DENN/MADD domain containing 2D [Source:HGNC Symbol;Acc:26192] [ENST00000473682]	79961
A_23_P384329	2.1	-1.1	-1.3	-1.2	DENND4A	Homo sapiens DENN/MADD domain containing 4A (DENND4A), transcript variant 1, mRNA [NM_001144823]	10260

A_24_P136497	-2.7	-1.9	-1.1	-2.1	DENND4A	Homo sapiens DENN/MADD domain containing 4A (DENND4A), transcript variant 2, mRNA [NM_005848]	10260
A_24_P409265	4.4	4.4	2.4	3.4	DENND4A	Homo sapiens DENN/MADD domain containing 4A (DENND4A), transcript variant 2, mRNA [NM_005848]	10260
A_23_P420092	2.7	2.6	2.6	2.6	DENND4A	Homo sapiens DENN/MADD domain containing 4A (DENND4A), transcript variant 1, mRNA [NM_001144823]	10260

A_23_P774	-1.2	-2.9	-1.4	-15.7	DENND4B	Homo sapiens DENN/MADD domain containing 4B (DENND4B), mRNA [NM_014856]	9909
A_24_P93633	3.4	2.9	2.9	3.0	DENND4C	DENN/MADD domain containing 4C [Source:HGNC Symbol;Acc:26079] [ENST00000380424]	55667
A_24_P541919	1.1	-1.2	-1.3	-6.0	DENND5B	Homo sapiens DENN/MADD domain containing 5B (DENND5B), mRNA [NM_144973]	160518
A_23_P361419	2.2	1.0	1.0	1.1	DEPDC1B	Homo sapiens DEP domain containing 1B (DEPDC1B), transcript variant 1, mRNA [NM_018369]	55789

A_23_P60166	-1.7	-1.0	-3.1	-3.0	DEPTOR	Homo sapiens DEP domain containing MTOR-interacting protein (DEPTOR), mRNA [NM_022783]	64798
A_24_P239343	9.1	3.7	3.5	3.8	DERL3	Homo sapiens Der1-like domain family, member 3 (DERL3), transcript variant 3, mRNA [NM_198440]	91319
A_23_P6362	1.3	2.2	1.4	2.8	DERL3	Homo sapiens Der1-like domain family, member 3 (DERL3), transcript variant 3, mRNA [NM_198440]	91319

A_23_P26184	-9.3	1.0	-1.0	1.1	DET1	Homo sapiens de-etiolated homolog 1 (Arabidopsis) (DET1), transcript variant 1, mRNA [NM_017996]	55070
A_23_P12189	2.0	2.3	-1.2	-1.3	DFFA	Homo sapiens DNA fragmentation factor, 45kDa, alpha polypeptide (DFFA), transcript variant 2, mRNA [NM_213566]	1676
A_23_P44546	1.0	-2.1	-2.5	-1.4	DFFB	Homo sapiens DNA fragmentation factor, 40kDa, beta polypeptide (caspase-activated DNase) (DFFB), mRNA [NM_004402]	1677

A_24_P370626	-1.3	-5.6	-2.4	-1.4	DFFB	Homo sapiens DNA fragmentation factor, 40kDa, beta polypeptide (caspase-activated DNase) (DFFB), mRNA [NM_004402]	1677
A_24_P376129	-6.0	1.0	-1.6	-1.0	DFNB31	deafness, autosomal recessive 31 [Source:HGNC Symbol;Acc:16361] [ENST00000374057]	25861
A_23_P112162	-1.0	-2.1	-2.9	-1.2	DGAT1	Homo sapiens diacylglycerol O-acyltransferase 1 (DGAT1), mRNA [NM_012079]	8694
A_23_P53198	-2.0	-1.1	-1.2	-1.2	DGAT2	Homo sapiens diacylglycerol O-acyltransferase 2 (DGAT2), mRNA [NM_032564]	84649

A_24_P125881	-3.5	-1.0	-3.0	-1.8	DGCR2	Homo sapiens DiGeorge syndrome critical region gene 2 (DGCR2), transcript variant 1, mRNA [NM_005137]	9993
A_24_P202948	-1.3	2.6	2.3	-1.1	DGCR6	Homo sapiens DiGeorge syndrome critical region gene 6 (DGCR6), mRNA [NM_005675]	8214
A_24_P113521	-1.2	-3.1	-1.4	1.0	DGCR8	Homo sapiens DiGeorge syndrome critical region gene 8 (DGCR8), transcript variant 1, mRNA [NM_022720]	54487

A_23_P211355	1.1	1.7	2.1	2.1	DGCR8	Homo sapiens DiGeorge syndrome critical region gene 8 (DGCR8), transcript variant 1, mRNA [NM_022720]	54487
A_23_P40926	-2.4	-1.1	-1.2	-20.3	DGKG	Homo sapiens diacylglycerol kinase, gamma 90kDa (DGKG), transcript variant 1, mRNA [NM_001346]	1608
A_23_P502980	-1.0	-9.6	-1.0	-1.1	DGKH	Homo sapiens diacylglycerol kinase, eta (DGKH), transcript variant 1, mRNA [NM_152910]	160851

A_24_P103952	-1.2	-1.1	-2.3	-1.6	DGKZ	Homo sapiens diacylglycerol kinase, zeta (DGKZ), transcript variant 2, mRNA [NM_003646]	8525
A_23_P127627	1.3	3.5	1.6	1.2	DGKZ	Homo sapiens diacylglycerol kinase, zeta (DGKZ), transcript variant 2, mRNA [NM_003646]	8525
A_23_P24444	-1.3	1.2	-1.4	-2.3	DHCR7	Homo sapiens 7-dehydrocholesterol reductase (DHCR7), transcript variant 1, mRNA [NM_001360]	1717
A_23_P201764	2.0	-1.3	-2.5	-2.5	DHDDS	Homo sapiens dehydrodolichyl diphosphate synthase (DHDDS), transcript variant 2, mRNA [NM_024887]	79947

A_32_P211045	-1.1	-1.0	-1.5	-2.0	DHFR	Homo sapiens dihydrofolate reductase (DHFR), mRNA [NM_000791]	1719
A_24_P343095	3.7	5.0	5.0	3.6	DHFR	Homo sapiens dihydrofolate reductase (DHFR), mRNA [NM_000791]	1719
A_24_P942328	-17.4	-1.1	-1.1	-1.0	DHFR	Homo sapiens dihydrofolate reductase (DHFR), mRNA [NM_000791]	1719
A_23_P139312	-2.0	-1.3	-3.1	-4.2	DHFRL1	Homo sapiens dihydrofolate reductase-like 1 (DHFRL1), transcript variant 2, mRNA [NM_176815]	200895
A_23_P162314	-1.4	1.0	2.2	1.6	DHH	Homo sapiens desert hedgehog (DHH), mRNA [NM_021044]	50846

A_23_P15202	1.2	1.2	-11.2	1.0	DHODH	Homo sapiens dihydroorotate dehydrogenase (quinone) (DHODH), nuclear gene encoding mitochondrial protein, mRNA [NM_001361]	1723
A_23_P48747	-2.0	2.1	1.4	1.1	DHRS1	Homo sapiens dehydrogenase/reductase (SDR family) member 1 (DHRS1), transcript variant 2, mRNA [NM_138452]	115817
A_24_P322215	-1.6	1.6	-2.3	-1.6	DHRS13	Homo sapiens dehydrogenase/reductase (SDR family) member 13 (DHRS13), mRNA [NM_144683]	147015

A_23_P66719	-10.2	1.2	1.2	1.0	DHRS13	Homo sapiens dehydrogenase/reductase (SDR family) member 13 (DHRS13), mRNA [NM_144683]	147015
A_23_P321501	-2.6	-1.5	-1.6	-1.5	DHRS2	Homo sapiens dehydrogenase/reductase (SDR family) member 2 (DHRS2), transcript variant 1, mRNA [NM_182908]	10202
A_23_P162982	-2.5	1.7	1.6	1.0	DHRS4	Homo sapiens dehydrogenase/reductase (SDR family) member 4 (DHRS4), mRNA [NM_021004]	10901

A_24_P903378	2.0	2.4	1.5	1.3	DHRS4L1	Homo sapiens isolate 1261292 dehydrogenase/reductase SDR family member 4-like 1 (DHRS4L1) mRNA, complete sequence, alternatively spliced. [GQ871922]	728635
A_23_P117506	-2.9	-4.3	-2.8	-4.5	DHRS7	Homo sapiens dehydrogenase/reductase (SDR family) member 7 (DHRS7), mRNA [NM_016029]	51635
A_24_P279520	3.4	3.3	3.1	1.0	DHRS7B	Homo sapiens dehydrogenase/reductase (SDR family) member 7B (DHRS7B), mRNA [NM_015510]	25979

A_24_P130352	-1.3	-1.7	-2.2	-1.7	DHRS7B	Homo sapiens dehydrogenase/reductase (SDR family) member 7B (DHRS7B), mRNA [NM_015510]	25979
A_23_P141208	-1.9	2.6	1.6	-1.3	DHRS7B	Homo sapiens dehydrogenase/reductase (SDR family) member 7B (DHRS7B), mRNA [NM_015510]	25979
A_23_P129916	1.2	2.1	1.3	1.2	DHRS7C	Homo sapiens dehydrogenase/reductase (SDR family) member 7C (DHRS7C), transcript variant 1, mRNA [NM_001220493]	201140

A_23_P12601	1.0	1.0	-1.0	-2.7	DHTKD1	Homo sapiens dehydrogenase E1 and transketolase domain containing 1 (DHTKD1), nuclear gene encoding mitochondrial protein, mRNA [NM_018706]	55526
A_23_P168062	2.2	1.3	1.7	2.9	DHX16	Homo sapiens DEAH (Asp-Glu-Ala-His) box polypeptide 16 (DHX16), transcript variant 1, mRNA [NM_003587]	8449
A_23_P81463	2.3	1.9	2.7	3.1	DHX29	Homo sapiens DEAH (Asp-Glu-Ala-His) box polypeptide 29 (DHX29), mRNA [NM_019030]	54505

A_23_P57697	1.4	2.4	1.1	-1.2	DHX36	Homo sapiens DEAH (Asp-Glu-Ala-His) box polypeptide 36 (DHX36), transcript variant 1, mRNA [NM_020865]	170506
A_23_P13914	7.6	4.5	1.1	4.1	DHX37	Homo sapiens DEAH (Asp-Glu-Ala-His) box polypeptide 37 (DHX37), mRNA [NM_032656]	57647
A_23_P38011	2.6	4.3	2.0	-1.4	DHX38	Homo sapiens DEAH (Asp-Glu-Ala-His) box polypeptide 38 (DHX38), mRNA [NM_014003]	9785

A_23_P390343	2.4	1.1	1.5	1.9	DHX40	Homo sapiens DEAH (Asp-Glu-Ala-His) box polypeptide 40 (DHX40), transcript variant 1, mRNA [NM_024612]	79665
A_23_P16908	1.1	-2.2	-2.3	-2.0	DHX57	Homo sapiens DEAH (Asp-Glu-Ala-Asp/His) box polypeptide 57 (DHX57), mRNA [NM_198963]	90957
A_24_P808534	1.7	-1.6	2.9	1.5	DHX9	Homo sapiens DEAH (Asp-Glu-Ala-His) box polypeptide 9 (DHX9), transcript variant 1, mRNA [NM_001357]	1660

A_23_P213344	1.1	-2.5	-1.8	-1.7	DIAPH1	Homo sapiens diaphanous homolog 1 (Drosophila) (DIAPH1), transcript variant 1, mRNA [NM_005219]	1729
A_23_P85004	-1.0	2.0	1.2	-1.1	DIAPH2	Homo sapiens diaphanous homolog 2 (Drosophila) (DIAPH2), transcript variant 12C, mRNA [NM_007309]	1730
A_23_P162719	1.1	-3.7	-2.8	1.4	DIAPH3	Homo sapiens diaphanous homolog 3 (Drosophila) (DIAPH3), transcript variant 2, mRNA [NM_030932]	81624

A_23_P419254	-1.9	1.1	-2.1	-1.3	DIAPH3	Homo sapiens diaphanous homolog 3 (Drosophila) (DIAPH3), transcript variant 2, mRNA [NM_030932]	81624
A_32_P64919	1.0	-1.0	-2.7	-7.4	DIAPH3	Homo sapiens diaphanous homolog 3 (Drosophila) (DIAPH3), transcript variant 1, mRNA [NM_001042517]	81624
A_32_P79313	-1.2	-1.3	-2.6	-1.8	DICER1-AS	Homo sapiens cDNA clone IMAGE:5270177, with apparent retained intron. [BC040596]	400242

A_24_P678418	3.5	1.2	1.2	1.0	DICER1-AS	Homo sapiens DICER1 antisense RNA (non-protein coding) (DICER1-AS), non-coding RNA [NR_015415]	400242
A_24_P944144	-1.0	-3.6	-1.1	-1.0	DIDO1	Homo sapiens death inducer-obliterator 1 (DIDO1), transcript variant 3, mRNA [NM_080797]	11083
A_24_P322611	-3.4	1.9	1.8	1.9	DIDO1	Homo sapiens death inducer-obliterator 1 (DIDO1), transcript variant 1, mRNA [NM_022105]	11083

A_23_P395426	2.5	-1.0	-1.1	-1.1	DIDO1	Homo sapiens death inducer-obliterator 1 (DIDO1), transcript variant 1, mRNA [NM_022105]	11083
A_23_P45811	2.5	3.0	1.8	4.3	DIO1	Homo sapiens deiodinase, iodothyronine, type I (DIO1), transcript variant 1, mRNA [NM_000792]	1733
A_23_P68752	1.7	2.0	1.8	-1.0	DIP2A	Homo sapiens DIP2 disco-interacting protein 2 homolog A (Drosophila) (DIP2A), transcript variant 1, mRNA [NM_015151]	23181

A_23_P131139	1.0	2.4	-1.2	1.9	DIRC1	Homo sapiens disrupted in renal carcinoma 1 (DIRC1), mRNA [NM_052952]	116093
A_23_P80778	1.1	-1.1	-5.6	-1.3	DIRC2	Homo sapiens disrupted in renal carcinoma 2 (DIRC2), mRNA [NM_032839]	84925
A_24_P395317	3.3	4.8	3.1	2.0	DIS3	Homo sapiens DIS3 mitotic control homolog (S. cerevisiae) (DIS3), transcript variant 1, mRNA [NM_014953]	22894
A_23_P65741	3.6	8.2	9.4	5.9	DIS3L	Homo sapiens DIS3 mitotic control homolog (S. cerevisiae)-like (DIS3L), transcript variant 2, mRNA [NM_133375]	115752

A_32_P232192	3.0	6.2	3.9	4.2	DIS3L2	DIS3 mitotic control homolog (S. cerevisiae)-like 2 [Source:HGNC Symbol;Acc:28648] [ENST00000273009]	129563
A_23_P201319	1.0	1.1	-2.7	-1.4	DISP1	Homo sapiens dispatched homolog 1 (Drosophila) (DISP1), mRNA [NM_032890]	84976
A_23_P324340	-1.1	1.6	2.3	1.1	DISP2	Homo sapiens dispatched homolog 2 (Drosophila) (DISP2), mRNA [NM_033510]	85455

A_23_P137143	-6.0	1.1	-1.3	-1.2	DKC1	Homo sapiens dyskeratosis congenita 1, dyskerin (DKC1), transcript variant 1, mRNA [NM_001363]	1736
A_23_P24129	1.1	1.0	-10.1	1.1	DKK1	Homo sapiens dickkopf homolog 1 (Xenopus laevis) (DKK1), mRNA [NM_012242]	22943
A_24_P311679	1.4	2.2	-1.9	-1.5	DKK2	Homo sapiens dickkopf homolog 2 (Xenopus laevis) (DKK2), mRNA [NM_014421]	27123
A_23_P162047	1.2	7.7	-1.1	1.9	DKK3	Homo sapiens dickkopf homolog 3 (Xenopus laevis) (DKK3), transcript variant 1, mRNA [NM_015881]	27122

A_23_P36248	-1.0	-3.0	-1.1	-1.2	DKK3	Homo sapiens RIG mRNA, complete cds. [U32331]	27122
A_23_P94275	-2.5	-1.1	1.2	-3.1	DKK4	Homo sapiens dickkopf homolog 4 (Xenopus laevis) (DKK4), mRNA [NM_014420]	27121
A_24_P372672	1.1	1.0	-2.6	1.1	DLAT	Homo sapiens dihydrolipoamide S-acetyltransferase (DLAT), nuclear gene encoding mitochondrial protein, mRNA [NM_001931]	1737
A_23_P22072	3.5	3.5	2.6	3.6	DLEU2	Homo sapiens BCMS-upstream neighbor (BCMSUN) mRNA, partial sequence. [AF264787]	8847

A_24_P131752	-16.8	1.0	-1.0	1.2	DLG1	Homo sapiens discs, large homolog 1 (Drosophila) (DLG1), transcript variant 2, mRNA [NM_004087]	1739
A_24_P945283	1.0	-2.5	-1.1	-1.3	DLG3	Homo sapiens discs, large homolog 3 (Drosophila) (DLG3), transcript variant 1, mRNA [NM_021120]	1741
A_23_P218398	1.4	5.6	7.0	4.5	DLG4	discs, large homolog 4 (Drosophila) [Source:HGNC Symbol;Acc:2903] [ENST00000485100]	1742

A_23_P411102	1.0	1.4	-2.5	1.3	DLG4	Homo sapiens discs, large homolog 4 (Drosophila) (DLG4), transcript variant 1, mRNA [NM_001365]	1742
A_24_P216253	-9.5	-1.1	-1.3	-1.7	DLGAP4	Homo sapiens discs, large (Drosophila) homolog-associated protein 4 (DLGAP4), transcript variant 1, mRNA [NM_014902]	22839
A_23_P210419	-1.1	-1.7	2.7	1.8	DLGAP4	Homo sapiens discs, large (Drosophila) homolog-associated protein 4 (DLGAP4), transcript variant 1, mRNA [NM_014902]	22839

A_23_P167920	1.0	-3.1	-1.1	-1.1	DLL1	Homo sapiens delta-like 1 (Drosophila) (DLL1), mRNA [NM_005618]	28514
A_24_P57047	-1.0	-4.2	-1.1	-1.0	DLL3	Homo sapiens delta-like 3 (Drosophila) (DLL3), transcript variant 2, mRNA [NM_203486]	10683
A_32_P142818	1.0	-2.2	-1.9	1.2	DLX1	Homo sapiens distal-less homeobox 1 (DLX1), transcript variant 1, mRNA [NM_178120]	1745
A_23_P28595	-1.2	1.1	-4.4	-1.9	DLX2	Homo sapiens distal-less homeobox 2 (DLX2), mRNA [NM_004405]	1746

A_23_P28598	1.2	-1.7	1.2	-5.2	DLX2	Homo sapiens distal-less homeobox 2 (DLX2), mRNA [NM_004405]	1746
A_23_P308552	2.1	2.3	-1.6	1.4	DLX3	Homo sapiens distal-less homeobox 3 (DLX3), mRNA [NM_005220]	1747
A_23_P388780	2.2	1.3	2.2	-2.0	DMAP1	Homo sapiens DNA methyltransferase 1 associated protein 1 (DMAP1), transcript variant 1, mRNA [NM_019100]	55929
A_23_P586	-8.4	-2.3	-1.8	-1.0	DMAP1	Homo sapiens DNA methyltransferase 1 associated protein 1 (DMAP1), transcript variant 1, mRNA [NM_019100]	55929

A_23_P500542	1.0	-5.4	1.0	1.2	DMBX1	Homo sapiens diencephalon/mesencephalon homeobox 1 (DMBX1), transcript variant 2, mRNA [NM_147192]	127343
A_24_P288612	-5.0	-2.0	-1.1	1.3	DMC1	Homo sapiens DMC1 dosage suppressor of mck1 homolog, meiosis-specific homologous recombination (yeast) (DMC1), mRNA [NM_007068]	11144
A_23_P321860	-1.0	-2.2	-1.0	-1.0	DMD	Homo sapiens dystrophin (DMD), transcript variant Dp40, mRNA [NM_004019]	1756

A_23_P257803	2.3	4.1	1.7	1.9	DMGDH	Homo sapiens dimethylglycine dehydrogenase (DMGDH), nuclear gene encoding mitochondrial protein, mRNA [NM_013391]	29958
A_32_P213459	2.4	10.1	6.2	6.6	DMRT2	Homo sapiens doublesex and mab-3 related transcription factor 2 (DMRT2), transcript variant 1, mRNA [NM_006557]	10655
A_23_P417821	-2.1	-1.0	-1.0	1.0	DMRTB1	Homo sapiens DMRT-like family B with proline-rich C-terminal, 1 (DMRTB1), mRNA [NM_033067]	63948

A_23_P8664	-1.0	-1.0	-3.5	-1.0	DMTF1	Homo sapiens cyclin D binding myb-like transcription factor 1 (DMTF1), transcript variant 1, mRNA [NM_021145]	9988
A_23_P56010	1.7	2.2	1.6	1.6	DMWD	Homo sapiens dystrophia myotonica, WD repeat containing (DMWD), mRNA [NM_004943]	1762
A_23_P250571	-1.5	1.0	-1.0	-3.9	DMXL1	Homo sapiens Dmx-like 1 (DMXL1), mRNA [NM_005509]	1657
A_24_P101128	1.0	1.0	-2.6	-2.4	DMXL1	Homo sapiens Dmx-like 1 (DMXL1), mRNA [NM_005509]	1657

A_24_P366107	-1.3	-1.3	-2.9	-1.3	DNA2	Homo sapiens DNA replication helicase 2 homolog (yeast) (DNA2), mRNA [NM_001080449]	1763
A_23_P18213	-2.1	-2.5	-1.6	-2.5	DNAH1	Homo sapiens dynein, axonemal, heavy chain 1 (DNAH1), mRNA [NM_015512]	25981
A_32_P224253	1.1	-2.9	-1.1	-1.0	DNAH11	Homo sapiens dynein, axonemal, heavy chain 11 (DNAH11), mRNA [NM_003777]	8701
A_23_P93866	1.1	1.2	-4.5	1.0	DNAH11	Homo sapiens dynein, axonemal, heavy chain 11 (DNAH11), mRNA [NM_003777]	8701

A_24_P169402	1.1	1.1	1.0	2.2	DNAH12	Homo sapiens dynein, axonemal, heavy chain 12 (DNAH12), transcript variant 1, mRNA [NM_178504]	201625
A_32_P14721	-1.0	-1.0	-1.0	-2.7	DNAH12	Homo sapiens dynein, axonemal, heavy chain 12 (DNAH12), transcript variant 1, mRNA [NM_178504]	201625
A_23_P333951	-3.6	-1.2	-1.1	-1.7	DNAH14	Homo sapiens dynein, axonemal, heavy chain 14 (DNAH14), transcript variant 3, mRNA [NM_144989]	127602

A_32_P87531	1.1	1.1	1.0	-5.5	DNAH14	Homo sapiens dynein, axonemal, heavy chain 14 (DNAH14), transcript variant 2, mRNA [NM_001145154]	127602
A_24_P127691	3.1	3.0	2.8	3.0	DNAH14	dynein, axonemal, heavy chain 14 [Source:HGNC Symbol;Acc:2945] [ENST00000495456]	127602
A_23_P359457	3.7	-1.3	-1.4	-1.4	DNAH2	dynein, axonemal, heavy chain 2 [Source:HGNC Symbol;Acc:2948] [ENST00000082259]	146754
A_24_P419286	4.2	2.8	2.3	4.0	DNAH3	Homo sapiens dynein, axonemal, heavy chain 3 (DNAH3), mRNA [NM_017539]	55567

A_24_P120462	-1.9	-1.0	-1.8	-2.3	DNAH5	Homo sapiens dynein, axonemal, heavy chain 5 (DNAH5), mRNA [NM_001369]	1767
A_32_P351037	-14.4	1.0	1.0	1.1	DNAH7	dynein, axonemal, heavy chain 7 [Source:HGNC Symbol;Acc:18661] [ENST00000410072]	56171
A_23_P169966	-1.4	1.8	2.7	1.4	DNAI2	Homo sapiens mRNA for intermediate dynein chain (DNAI2 gene). [AJ295276]	64446
A_23_P60479	-1.1	-2.0	-1.4	-2.0	DNAJA1	Homo sapiens DnaJ (Hsp40) homolog, subfamily A, member 1 (DNAJA1), mRNA [NM_001539]	3301

A_23_P90062	1.1	1.0	-2.3	-1.0	DNAJB1	Homo sapiens DnaJ (Hsp40) homolog, subfamily B, member 1 (DNAJB1), mRNA [NM_006145]	3337
A_23_P166899	2.1	-1.4	-1.0	1.8	DNAJB11	Homo sapiens DnaJ (Hsp40) homolog, subfamily B, member 11 (DNAJB11), mRNA [NM_016306]	51726
A_23_P52382	4.3	4.4	1.8	3.9	DNAJB12	Homo sapiens DnaJ (Hsp40) homolog, subfamily B, member 12 (DNAJB12), transcript variant 2, mRNA [NM_017626]	54788

A_24_P244096	-1.3	-1.2	-2.2	-1.0	DNAJB12	Homo sapiens DnaJ (Hsp40) homolog, subfamily B, member 12 (DNAJB12), transcript variant 1, mRNA [NM_001002762]	54788
A_24_P102981	-1.0	-7.9	-1.2	-1.0	DNAJB2	Homo sapiens DnaJ (Hsp40) homolog, subfamily B, member 2 (DNAJB2), transcript variant 2, mRNA [NM_006736]	3300
A_32_P2362	-1.1	1.5	1.2	4.1	DNAJB3	Homo sapiens DnaJ (Hsp40) homolog, subfamily B, member 3 (DNAJB3), mRNA [NM_001001394]	414061

A_23_P51339	-4.3	3.4	-2.3	-3.7	DNAJB4	Homo sapiens DnaJ (Hsp40) homolog, subfamily B, member 4 (DNAJB4), mRNA [NM_007034]	11080
A_32_P229746	1.2	2.2	1.8	-1.1	DNAJB6	Homo sapiens DnaJ (Hsp40) homolog, subfamily B, member 6 (DNAJB6), transcript variant 2, mRNA [NM_005494]	10049
A_23_P385063	2.7	2.6	2.3	1.9	DNAJB6	Homo sapiens DnaJ (Hsp40) homolog, subfamily B, member 6 (DNAJB6), transcript variant 1, mRNA [NM_058246]	10049

A_23_P215227	-2.4	1.5	-1.4	-1.5	DNAJB6	Homo sapiens DnaJ (Hsp40) homolog, subfamily B, member 6 (DNAJB6), transcript variant 2, mRNA [NM_005494]	10049
A_23_P166910	-2.5	1.0	-1.6	-1.1	DNAJC13	Homo sapiens DnaJ (Hsp40) homolog, subfamily C, member 13 (DNAJC13), mRNA [NM_015268]	23317
A_23_P140698	-1.4	-1.0	-2.0	-2.8	DNAJC17	Homo sapiens DnaJ (Hsp40) homolog, subfamily C, member 17 (DNAJC17), mRNA [NM_018163]	55192

A_32_P149251	5.8	1.2	2.7	2.2	DNAJC18	Homo sapiens DnaJ (Hsp40) homolog, subfamily C, member 18 (DNAJC18), mRNA [NM_152686]	202052
A_23_P121396	-3.2	1.1	-1.0	-1.0	DNAJC19	Homo sapiens DnaJ (Hsp40) homolog, subfamily C, member 19 (DNAJC19), transcript variant 1, mRNA [NM_145261]	131118
A_23_P431890	1.3	2.4	1.0	1.3	DNAJC19	Homo sapiens DnaJ (Hsp40) homolog, subfamily C, member 19 (DNAJC19), transcript variant 1, mRNA [NM_145261]	131118

A_23_P254573	-2.9	-3.0	-1.3	-1.6	DNAJC2	Homo sapiens DnaJ (Hsp40) homolog, subfamily C, member 2 (DNAJC2), transcript variant 1, mRNA [NM_014377]	27000
A_32_P407245	6.0	3.4	1.6	1.1	DNAJC22	Homo sapiens DnaJ (Hsp40) homolog, subfamily C, member 22 (DNAJC22), mRNA [NM_024902]	79962
A_23_P397019	1.1	1.1	-4.1	1.0	DNAJC25	Homo sapiens DnaJ (Hsp40) homolog, subfamily C , member 25 (DNAJC25), transcript variant 1, mRNA [NM_001015882]	548645

A_23_P17275	-1.5	-1.2	-5.3	-1.1	DNAJC27	Homo sapiens DnaJ (Hsp40) homolog, subfamily C, member 27 (DNAJC27), transcript variant 1, mRNA [NM_016544]	51277
A_23_P68700	1.3	1.3	-2.7	1.0	DNAJC28	Homo sapiens DnaJ (Hsp40) homolog, subfamily C, member 28 (DNAJC28), transcript variant 1, mRNA [NM_017833]	54943

A_24_P51184	3.0	3.0	2.6	1.1	DNAJC3	Homo sapiens DnaJ (Hsp40) homolog, subfamily C, member 3, mRNA (cDNA clone IMAGE:5218144), with apparent retained intron. [BC033823]	5611
A_24_P218006	-1.6	3.0	3.0	3.0	DNAJC30	Homo sapiens DnaJ (Hsp40) homolog, subfamily C, member 30 (DNAJC30), mRNA [NM_032317]	84277
A_32_P37461	1.3	1.3	-2.2	1.0	DNAJC3-AS1	PREDICTED: Homo sapiens DNAJC3 antisense RNA 1 (non-protein coding) (DNAJC3-AS1), miscRNA [XR_109147]	100289274

A_23_P202769	2.8	-1.1	1.1	2.3	DNAJC4	Homo sapiens DnaJ (Hsp40) homolog, subfamily C, member 4 (DNAJC4), mRNA [NM_005528]	3338
A_24_P355145	-2.2	1.0	1.0	1.0	DNAJC5B	Homo sapiens DnaJ (Hsp40) homolog, subfamily C, member 5 beta (DNAJC5B), mRNA [NM_033105]	85479
A_23_P95359	-15.5	1.0	-1.2	1.1	DNAJC6	Homo sapiens DnaJ (Hsp40) homolog, subfamily C, member 6 (DNAJC6), mRNA [NM_014787]	9829

A_23_P37137	2.5	2.5	2.2	-1.1	DNAJC8	Homo sapiens DnaJ (Hsp40) homolog, subfamily C, member 8 (DNAJC8), mRNA [NM_014280]	22826
A_23_P46455	1.2	-1.1	-1.7	3.5	DNAJC8	Homo sapiens DnaJ (Hsp40) homolog, subfamily C, member 8 (DNAJC8), mRNA [NM_014280]	22826
A_32_P157192	-2.2	-3.7	-3.8	-2.0	DNAJC8	Homo sapiens DnaJ (Hsp40) homolog, subfamily C, member 8 (DNAJC8), mRNA [NM_014280]	22826
A_23_P25913	1.1	-3.2	1.0	-1.4	DNAL1	Homo sapiens dynein, axonemal, light chain 1 (DNAL1), transcript variant 1, mRNA [NM_031427]	83544

A_23_P17880	6.0	-1.1	1.4	1.2	DNAL4	Homo sapiens dynein, axonemal, light chain 4 (DNAL4), mRNA [NM_005740]	10126
A_23_P85164	2.3	1.3	-1.6	1.4	DNASE1L1	Homo sapiens deoxyribonuclease I-like 1 (DNASE1L1), transcript variant 1, mRNA [NM_006730]	1774
A_23_P257993	1.0	-12.2	-1.2	-1.7	DNASE1L3	Homo sapiens deoxyribonuclease I-like 3 (DNASE1L3), mRNA [NM_004944]	1776
A_23_P126677	-1.0	-4.7	1.5	-1.0	DNASE2B	Homo sapiens deoxyribonuclease II beta (DNASE2B), transcript variant 1, mRNA [NM_021233]	58511

A_24_P943352	-15.7	1.3	-1.2	-1.2	DNHD1	Homo sapiens dynein heavy chain domain 1 (DNHD1), transcript variant 1, mRNA [NM_144666]	144132
A_32_P360193	5.7	8.9	3.4	6.9	DNHD1	Homo sapiens dynein heavy chain domain 1 (DNHD1), transcript variant 1, mRNA [NM_144666]	144132
A_23_P316472	1.0	1.2	-1.3	-2.2	DNHD1	Homo sapiens dynein heavy chain domain 1 (DNHD1), transcript variant 1, mRNA [NM_144666]	144132
A_23_P367586	11.1	10.9	8.5	9.2	DNHD1	Homo sapiens dynein heavy chain domain 1 (DNHD1), transcript variant 2, mRNA [NM_173589]	144132

A_23_P135273	2.2	-1.2	1.5	-1.6	DNM1	Homo sapiens dynamin 1 (DNM1), transcript variant 2, mRNA [NM_001005336]	1759
A_24_P50091	1.6	-1.7	-2.3	-1.1	DNM1P46	Homo sapiens DNM1 pseudogene 46 (DNM1P46), non-coding RNA [NR_003260]	196968
A_23_P328206	1.2	-1.9	-2.3	-1.6	DNMBP	Homo sapiens dynamin binding protein (DNMBP), mRNA [NM_015221]	23268
A_24_P408083	3.0	2.4	1.1	1.0	DNMT1	Homo sapiens DNA (cytosine-5-)-methyltransferase 1 (DNMT1), transcript variant 2, mRNA [NM_001379]	1786

A_23_P142676	-1.5	-2.2	-2.0	-1.6	DNPEP	Homo sapiens aspartyl aminopeptidase (DNPEP), mRNA [NM_012100]	23549
A_23_P409489	2.6	1.3	1.4	1.1	DNTT	Homo sapiens deoxynucleotidyltransferase, terminal (DNTT), transcript variant 1, mRNA [NM_004088]	1791
A_23_P16722	-1.8	1.1	1.1	-3.2	DOCK10	Homo sapiens dedicator of cytokinesis 10 (DOCK10), mRNA [NM_014689]	55619
A_24_P263653	1.2	1.3	-2.4	1.2	DOCK2	Homo sapiens dedicator of cytokinesis 2 (DOCK2), mRNA [NM_004946]	1794

A_24_P153643	6.7	6.5	5.9	5.0	DOCK3	Homo sapiens dedicator of cytokinesis 3 (DOCK3), mRNA [NM_004947]	1795
A_24_P272845	1.9	1.2	2.7	-1.4	DOCK3	Homo sapiens dedicator of cytokinesis 3 (DOCK3), mRNA [NM_004947]	1795
A_23_P364580	1.2	1.2	-13.4	-1.2	DOCK4	Homo sapiens dedicator of cytokinesis 4 (DOCK4), mRNA [NM_014705]	9732
A_24_P324886	2.1	2.2	2.1	1.4	DOCK4	Homo sapiens dedicator of cytokinesis 4 (DOCK4), mRNA [NM_014705]	9732

A_24_P350245	9.3	12.0	3.6	3.3	DOCK5	Homo sapiens dedicator of cytokinesis 5 (DOCK5), mRNA [NM_024940]	80005
A_23_P67299	-24.5	1.6	1.5	1.0	DOCK6	Homo sapiens dedicator of cytokinesis 6 (DOCK6), mRNA [NM_020812]	57572
A_23_P331348	-5.7	1.0	1.2	1.7	DOCK7	Homo sapiens dedicator of cytokinesis 7 (DOCK7), mRNA [NM_033407]	85440
A_32_P230547	3.0	2.8	-1.0	2.5	DOCK7	Homo sapiens dedicator of cytokinesis 7 (DOCK7), mRNA [NM_033407]	85440

A_24_P331998	1.1	1.1	2.4	1.0	DOCK8	Homo sapiens dedicator of cytokinesis 8 (DOCK8), transcript variant 1, mRNA [NM_203447]	81704
A_23_P353888	2.0	3.0	3.3	2.3	DOCK8	Homo sapiens dedicator of cytokinesis 8 (DOCK8), transcript variant 1, mRNA [NM_203447]	81704
A_23_P162766	3.9	3.9	4.0	3.9	DOCK9	Homo sapiens dedicator of cytokinesis 9 (DOCK9), transcript variant 1, mRNA [NM_015296]	23348

A_23_P5610	5.0	4.7	3.1	-4.3	DOK1	Homo sapiens docking protein 1, 62kDa (downstream of tyrosine kinase 1) (DOK1), transcript variant 1, mRNA [NM_001381]	1796
A_23_P134851	-2.0	-2.9	-4.7	1.4	DOK2	Homo sapiens docking protein 2, 56kDa (DOK2), mRNA [NM_003974]	9046
A_23_P133506	2.6	2.7	2.8	2.4	DOK3	Homo sapiens docking protein 3 (DOK3), transcript variant 1, mRNA [NM_024872]	79930
A_24_P48069	1.1	-2.1	1.3	-1.0	DOK4	Homo sapiens docking protein 4 (DOK4), mRNA [NM_018110]	55715

A_23_P77381	1.2	-1.8	-4.2	-5.5	DOK4	Homo sapiens docking protein 4 (DOK4), mRNA [NM_018110]	55715
A_23_P10870	3.0	2.4	2.5	1.7	DOLK	Homo sapiens dolichol kinase (DOLK), mRNA [NM_014908]	22845
A_23_P386764	1.1	-2.6	-1.0	-1.0	DOLPP1	Homo sapiens dolichyl pyrophosphate phosphatase 1 (DOLPP1), transcript variant 1, mRNA [NM_020438]	57171
A_23_P330017	1.1	1.0	-1.3	-2.1	DOLPP1	Homo sapiens dolichyl pyrophosphate phosphatase 1 (DOLPP1), transcript variant 1, mRNA [NM_020438]	57171

A_32_P181020	1.4	1.1	-2.2	-1.9	DOM3Z	Homo sapiens dom-3 homolog Z (C. elegans) (DOM3Z), mRNA [NM_005510]	1797
A_23_P434268	1.0	-2.1	-1.0	-1.0	DOPEY1	Homo sapiens dopey family member 1 (DOPEY1), transcript variant 2, mRNA [NM_001199942]	23033
A_23_P253586	9.8	9.5	8.8	9.4	DOPEY2	Homo sapiens dopey family member 2 (DOPEY2), mRNA [NM_005128]	9980
A_24_P936875	-1.6	-1.8	1.3	-3.6	DOT1L	Homo sapiens DOT1-like, histone H3 methyltransferase (S. cerevisiae) (DOT1L), mRNA [NM_032482]	84444

A_23_P129413	-22.4	1.5	-2.0	-2.9	DPEP3	Homo sapiens dipeptidase 3 (DPEP3), transcript variant 1, mRNA [NM_022357]	64180
A_23_P307392	-2.2	1.4	1.9	1.5	DPF3	Homo sapiens cDNA FLJ14079 fis, clone HEMBB1002134, weakly similar to ZINC-FINGER PROTEIN NEURO-D4. [AK024141]	8110
A_24_P225907	1.0	-1.0	-1.0	-3.8	DPH3	Homo sapiens DPH3, KTI11 homolog (S. cerevisiae) (DPH3), transcript variant 1, mRNA [NM_206831]	285381

A_23_P258814	1.3	1.6	2.4	-1.0	DPH3P1	Homo sapiens DPH3, KTI11 homolog (S. cerevisiae) pseudogene 1 (DPH3P1), mRNA [NM_080750]	100132911
A_23_P148821	-3.0	1.2	1.1	1.1	DPH5	Homo sapiens DPH5 homolog (S. cerevisiae) (DPH5), transcript variant 1, mRNA [NM_001077394]	51611
A_23_P217079	-2.0	1.4	2.1	-2.1	DPM2	Homo sapiens dolichyl-phosphate mannosyltransferase polypeptide 2, regulatory subunit (DPM2), mRNA [NM_003863]	8818

A_24_P179925	-1.4	-1.7	-3.3	-2.1	DPM3	Homo sapiens dolichyl-phosphate mannosyltransferase polypeptide 3 (DPM3), transcript variant 1, mRNA [NM_018973]	54344
A_23_P250506	3.3	7.1	8.0	1.5	DPM3	Homo sapiens dolichyl-phosphate mannosyltransferase polypeptide 3 (DPM3), transcript variant 1, mRNA [NM_018973]	54344
A_23_P102278	1.0	1.0	1.0	-8.7	DPP10	Homo sapiens dipeptidyl-peptidase 10 (non-functional) (DPP10), transcript variant 1, mRNA [NM_020868]	57628

A_24_P140057	3.9	3.5	2.5	4.0	DPP10	Homo sapiens dipeptidyl-peptidase 10 (non-functional) (DPP10), transcript variant 1, mRNA [NM_020868]	57628
A_23_P170888	-2.3	1.0	-1.0	-5.6	DPP6	Homo sapiens dipeptidyl-peptidase 6 (DPP6), transcript variant 3, mRNA [NM_001039350]	1804
A_24_P6552	2.0	3.1	2.0	1.0	DPP6	Homo sapiens dipeptidyl-peptidase 6 (DPP6), transcript variant 3, mRNA [NM_001039350]	1804
A_23_P32975	1.6	1.0	-3.7	1.2	DPP7	Homo sapiens dipeptidyl-peptidase 7 (DPP7), mRNA [NM_013379]	29952

A_24_P130066	-4.8	-4.7	-4.7	-5.0	DPP8	Homo sapiens dipeptidyl-peptidase 8 (DPP8), transcript variant 2, mRNA [NM_017743]	54878
A_23_P200741	3.3	1.3	-2.6	2.0	DPT	Homo sapiens dermatopontin (DPT), mRNA [NM_001937]	1805
A_24_P652033	2.1	2.0	1.8	-1.1	DPY19L1P1	Homo sapiens dpy-19-like 1 pseudogene 1 (C. elegans) (DPY19L1P1), non-coding RNA [NR_036680]	100129460
A_23_P374250	-3.1	-1.1	-1.7	-8.4	DPY19L2	Homo sapiens dpy-19-like 2 (C. elegans) (DPY19L2), mRNA [NM_173812]	283417

A_32_P195850	1.1	-1.5	2.1	-3.0	DPY19L2	Homo sapiens dpy-19-like 2 (C. elegans) (DPY19L2), mRNA [NM_173812]	283417
A_24_P186608	1.0	-5.1	-1.7	2.0	DPY19L2P2	Homo sapiens dpy-19-like 2 pseudogene 2 (C. elegans) (DPY19L2P2), transcript variant 2, non-coding RNA [NR_003561]	349152
A_32_P78385	1.6	-1.0	-2.2	1.4	DPY19L2P2	Homo sapiens dpy-19-like 2 pseudogene 2 (C. elegans) (DPY19L2P2), transcript variant 1, non-coding RNA [NR_027768]	349152

A_32_P23825	36.7	35.9	34.6	35.8	DPY19L2P2	Homo sapiens dpy-19-like 2 pseudogene 2 (C. elegans) (DPY19L2P2), transcript variant 2, non-coding RNA [NR_003561]	349152
A_24_P156153	1.1	3.0	1.4	2.0	DPY19L3	Homo sapiens dpy-19-like 3 (C. elegans), mRNA (cDNA clone IMAGE:5190889), complete cds. [BC029162]	147991
A_24_P254101	1.5	3.6	3.6	3.7	DPY19L3	Homo sapiens dpy-19-like 3 (C. elegans) (DPY19L3), transcript variant 1, mRNA [NM_207325]	147991

A_24_P481824	-2.5	-1.2	-10.8	-1.0	DPY19L4	Homo sapiens dpy-19-like 4 (C. elegans) (DPY19L4), mRNA [NM_181787]	286148
A_32_P196047	1.0	-1.0	3.1	-1.1	DPY19L4	Homo sapiens dpy-19-like 4 (C. elegans) (DPY19L4), mRNA [NM_181787]	286148
A_23_P20337	1.1	2.0	-2.1	1.8	DPYSL2	Homo sapiens dihydropyrimidinase-like 2 (DPYSL2), transcript variant 2, mRNA [NM_001386]	1808
A_23_P331049	1.7	2.8	1.6	-4.8	DPYSL4	Homo sapiens dihydropyrimidinase-like 4 (DPYSL4), mRNA [NM_006426]	10570

A_24_P381455	4.1	4.1	4.2	1.2	DRAM1	Homo sapiens DNA-damage regulated autophagy modulator 1 (DRAM1), mRNA [NM_018370]	55332
A_24_P107277	-2.0	3.4	3.1	1.3	DRAP1	Homo sapiens DR1-associated protein 1 (negative cofactor 2 alpha) (DRAP1), mRNA [NM_006442]	10589
A_23_P57733	-1.1	-10.3	-1.2	-1.2	DRD3	Homo sapiens dopamine receptor D3 (DRD3), transcript variant e, mRNA [NM_033663]	1814
A_24_P25668	5.1	-1.0	1.8	1.5	DRD4	Homo sapiens dopamine receptor D4 (DRD4), mRNA [NM_000797]	1815

A_23_P68884	-1.6	2.6	1.6	2.8	DRG1	Homo sapiens developmentally regulated GTP binding protein 1 (DRG1), mRNA [NM_004147]	4733
A_23_P141656	1.8	-2.8	1.2	-3.6	DRG2	Homo sapiens developmentally regulated GTP binding protein 2 (DRG2), mRNA [NM_001388]	1819
A_23_P38696	-2.5	1.0	-1.0	1.0	DSC1	Homo sapiens desmocollin 1 (DSC1), transcript variant Dsc1b, mRNA [NM_004948]	1823
A_24_P344416	6.9	10.0	6.5	9.0	DSC3	Homo sapiens desmocollin 3 (DSC3), transcript variant Dsc3b, mRNA [NM_024423]	1825

A_23_P47340	1.0	1.1	-2.2	1.6	DSCAML1	Homo sapiens Down syndrome cell adhesion molecule like 1 (DSCAML1), mRNA [NM_020693]	57453
A_23_P154874	-1.0	-7.5	1.0	-1.0	DSCR3	Homo sapiens Down syndrome critical region gene 3 (DSCR3), mRNA [NM_006052]	10311
A_24_P75963	4.9	5.0	7.7	3.4	DSCR4	Homo sapiens Down syndrome critical region gene 4 (DSCR4), mRNA [NM_005867]	10281
A_23_P109320	-2.2	-3.9	1.5	-1.2	DSCR6	Homo sapiens Down syndrome critical region gene 6 (DSCR6), mRNA [NM_018962]	53820

A_23_P430691	-9.1	-1.1	-1.1	1.2	DSCR9	Homo sapiens mRNA, complete cds. [AB066100]	257203
A_23_P27265	-1.0	-1.0	-1.1	-3.0	DSEL	Homo sapiens dermatan sulfate epimerase-like (DSEL), mRNA [NM_032160]	92126
A_23_P164504	-2.9	1.1	1.1	1.1	DSG1	Homo sapiens desmoglein 1 (DSG1), mRNA [NM_001942]	1828
A_32_P157945	-1.3	-1.2	-12.3	-1.3	DSP	Homo sapiens desmoplakin (DSP), transcript variant 1, mRNA [NM_004415]	1832
A_23_P373541	2.2	-2.1	1.9	1.6	DSPP	Homo sapiens dentin sialophosphoprotein (DSPP), mRNA [NM_014208]	1834

A_23_P59388	2.1	1.7	1.3	1.3	DST	Homo sapiens dystonin (DST), transcript variant 1e, mRNA [NM_001723]	667
A_23_P303718	4.7	2.3	2.8	5.9	DST	Homo sapiens dystonin (DST), transcript variant 1eA, mRNA [NM_015548]	667
A_23_P23894	1.1	-12.3	1.1	1.0	DSTYK	Homo sapiens dual serine/threonine and tyrosine protein kinase (DSTYK), transcript variant 1, mRNA [NM_015375]	25778

A_32_P71943	2.7	1.7	1.9	2.3	DSTYK	Homo sapiens dual serine/threonine and tyrosine protein kinase (DSTYK), transcript variant 1, mRNA [NM_015375]	25778
A_32_P787109	-2.3	-2.0	1.8	2.4	DTHD1	Homo sapiens death domain containing 1 (DTHD1), transcript variant 1, mRNA [NM_001170700]	401124
A_23_P394809	3.0	1.6	2.7	1.9	DTNA	Homo sapiens dystrobrevin, alpha (DTNA), transcript variant 1, mRNA [NM_001390]	1837
A_23_P210319	-1.1	-1.2	-2.0	-2.0	DTNB	Homo sapiens dystrobrevin, beta (DTNB), transcript variant 4, mRNA [NM_183360]	1838

A_24_P70117	1.1	-7.1	-1.0	-1.0	DTWD1	Homo sapiens DTW domain containing 1 (DTWD1), transcript variant 1, mRNA [NM_020234]	56986
A_32_P373557	1.1	1.0	-1.0	-3.9	DTWD2	Homo sapiens DTW domain containing 2 (DTWD2), mRNA [NM_173666]	285605
A_23_P36567	-1.9	-1.1	1.0	-3.5	DTX1	Homo sapiens deltex homolog 1 (Drosophila) (DTX1), mRNA [NM_004416]	1840
A_24_P290751	1.1	1.1	1.1	-23.8	DTX1	Homo sapiens deltex homolog 1 (Drosophila) (DTX1), mRNA [NM_004416]	1840

A_23_P395033	-2.9	1.0	1.0	1.1	DTX2	Homo sapiens deltex homolog 2 (Drosophila) (DTX2), transcript variant 1, mRNA [NM_020892]	113878
A_24_P6911	-4.6	-1.1	-1.1	-1.1	DTX2P1-UPK3	Homo sapiens DTX2P1-UPK3BP1-PMS2P11 readthrough (non-protein coding) (DTX2P1-UPK3BP1-PMS2P11), non-coding RNA [NR_023383]	441263
A_23_P336708	2.1	-2.5	1.3	1.3	DTX3	Homo sapiens deltex homolog 3 (Drosophila) (DTX3), mRNA [NM_178502]	196403

A_23_P53247	2.4	3.2	1.2	1.2	DTX3	Homo sapiens deltex homolog 3 (Drosophila) (DTX3), mRNA [NM_178502]	196403
A_24_P941912	2.7	4.1	1.2	2.6	DTX3L	Homo sapiens deltex 3-like (Drosophila) (DTX3L), mRNA [NM_138287]	151636
A_23_P255569	1.9	2.9	1.6	1.1	DUS1L	Homo sapiens dihydrouridine synthase 1-like (S. cerevisiae) (DUS1L), mRNA [NM_022156]	64118
A_23_P215778	-2.1	1.0	-2.3	-8.4	DUS4L	Homo sapiens dihydrouridine synthase 4-like (S. cerevisiae) (DUS4L), mRNA [NM_181581]	11062

A_23_P110712	3.7	3.6	3.5	3.2	DUSP1	Homo sapiens dual specificity phosphatase 1 (DUSP1), mRNA [NM_004417]	1843
A_24_P182494	-1.0	1.5	-1.1	-2.2	DUSP10	Homo sapiens dual specificity phosphatase 10 (DUSP10), transcript variant 1, mRNA [NM_007207]	11221
A_23_P51856	1.2	-2.4	-1.1	-1.1	DUSP10	Homo sapiens dual specificity phosphatase 10 (DUSP10), transcript variant 1, mRNA [NM_007207]	11221
A_23_P376759	1.1	1.1	-2.5	1.8	DUSP11	Homo sapiens dual specificity phosphatase 11 (RNA/RNP complex 1-interacting) (DUSP11), mRNA [NM_003584]	8446

A_23_P207537	1.3	1.0	1.2	-3.9	DUSP14	Homo sapiens dual specificity phosphatase 14 (DUSP14), mRNA [NM_007026]	11072
A_23_P154771	1.6	2.0	1.7	3.6	DUSP15	Homo sapiens dual specificity phosphatase 15 (DUSP15), transcript variant 1, mRNA [NM_080611]	128853
A_24_P73264	1.3	-1.6	1.0	-3.8	DUSP16	Homo sapiens dual specificity phosphatase 16 (DUSP16), mRNA [NM_030640]	80824
A_24_P189739	-2.2	1.5	1.5	1.0	DUSP16	Homo sapiens dual specificity phosphatase 16 (DUSP16), mRNA [NM_030640]	80824

A_24_P37409	1.2	1.6	-3.3	1.4	DUSP2	Homo sapiens dual specificity phosphatase 2 (DUSP2), mRNA [NM_004418]	1844
A_23_P108842	1.4	-4.3	1.3	1.4	DUSP2	Homo sapiens dual specificity phosphatase 2 (DUSP2), mRNA [NM_004418]	1844
A_23_P256077	1.1	1.2	1.0	4.2	DUSP21	Homo sapiens dual specificity phosphatase 21 (DUSP21), mRNA [NM_022076]	63904
A_23_P7896	1.6	1.6	2.5	1.3	DUSP22	Homo sapiens dual specificity phosphatase 22 (DUSP22), mRNA [NM_020185]	56940

A_23_P103232	-6.8	1.0	-1.0	1.1	DUSP23	Homo sapiens dual specificity phosphatase 23 (DUSP23), mRNA [NM_017823]	54935
A_23_P146134	-1.7	-1.9	-3.0	-1.9	DUSP26	Homo sapiens dual specificity phosphatase 26 (putative) (DUSP26), mRNA [NM_024025]	78986
A_23_P129956	1.0	-2.3	-1.0	1.1	DUSP3	Homo sapiens dual specificity phosphatase 3 (DUSP3), mRNA [NM_004090]	1845
A_24_P173790	1.1	2.0	1.3	1.5	DUSP5P	Homo sapiens cDNA FLJ31401 fis, clone NT2NE2000038. [AK055963]	574029

A_32_P157846	-8.4	-1.6	-1.8	-2.2	DUSP5P	Homo sapiens dual specificity phosphatase 5 pseudogene (DUSP5P), non-coding RNA [NR_002834]	574029
A_23_P139704	2.3	2.2	2.0	1.1	DUSP6	Homo sapiens dual specificity phosphatase 6 (DUSP6), transcript variant 1, mRNA [NM_001946]	1848
A_24_P51855	1.6	-1.0	-2.0	-1.8	DUSP7	Homo sapiens dual specificity phosphatase 7 (DUSP7), mRNA [NM_001947]	1849
A_23_P390528	-3.0	-2.7	-1.6	-1.6	DUSP8	Homo sapiens dual specificity phosphatase 8 (DUSP8), mRNA [NM_004420]	1850

A_23_P104413	-2.0	-2.0	-2.1	-1.3	DUX4	Homo sapiens double homeobox 4 (DUX4), mRNA [NM_033178]	22947
A_23_P333129	-2.4	-1.3	-4.8	1.9	DUX4L4	Homo sapiens double homeobox 4 like 4 (DUX4L4), mRNA [NM_001177376]	441056
A_23_P347432	-2.6	-1.0	-1.8	-4.5	DVL1	Homo sapiens dishevelled, dsh homolog 1 (Drosophila) (DVL1), mRNA [NM_004421]	1855
A_23_P201342	-2.0	1.3	1.5	-1.2	DVL1	Homo sapiens dishevelled, dsh homolog 1 (Drosophila) (DVL1), mRNA [NM_004421]	1855

A_24_P283000	-2.6	-1.8	-2.1	-3.3	DVL3	Homo sapiens dishevelled, dsh homolog 3 (Drosophila) (DVL3), mRNA [NM_004423]	1857
A_23_P218884	1.3	-1.2	-3.0	-1.1	DVL3	Homo sapiens dishevelled, dsh homolog 3 (Drosophila) (DVL3), mRNA [NM_004423]	1857
A_23_P360990	-4.3	1.4	1.3	1.2	DYDC1	Homo sapiens DPY30 domain containing 1 (DYDC1), mRNA [NM_138812]	143241
A_24_P221414	-1.0	-1.1	-1.1	-4.0	DYNC1I1	Homo sapiens dynein, cytoplasmic 1, intermediate chain 1 (DYNC1I1), transcript variant 1, mRNA [NM_004411]	1780

A_32_P30926	-2.4	-10.8	-4.2	-3.0	DYNC1I2	Homo sapiens dynein, cytoplasmic 1, intermediate chain 2 (DYNC1I2), mRNA [NM_001378]	1781
A_23_P147397	-1.2	2.3	-1.2	-1.3	DYNC2H1	Homo sapiens dynein, cytoplasmic 2, heavy chain 1 (DYNC2H1), transcript variant 2, mRNA [NM_001080463]	79659
A_24_P83846	4.6	16.2	15.6	31.2	DYNC2H1	Homo sapiens dynein, cytoplasmic 2, heavy chain 1 (DYNC2H1), transcript variant 1, mRNA [NM_001377]	79659

A_24_P143440	2.1	2.0	1.8	1.8	DYNLRB1	Homo sapiens dynein, light chain, roadblock-type 1 (DYNLRB1), mRNA [NM_014183]	83658
A_24_P100368	-4.2	1.0	-1.1	1.1	DYNLT3	Homo sapiens dynein, light chain, Tctex-type 3 (DYNLT3), mRNA [NM_006520]	6990
A_24_P62169	1.2	1.6	1.3	2.0	DYRK1A	Homo sapiens dual-specificity tyrosine-(Y)-phosphorylation regulated kinase 1A (DYRK1A), transcript variant 2, mRNA [NM_130436]	1859

A_32_P852607	1.5	1.2	-1.4	-2.1	DYRK1A	dual-specificity tyrosine-(Y)-phosphorylation regulated kinase 1A [Source:HGNC Symbol;Acc:3091] [ENST00000462274]	1859
A_24_P322753	-2.6	-2.6	-4.5	-2.4	DYRK1A	Homo sapiens dual-specificity tyrosine-(Y)-phosphorylation regulated kinase 1A (DYRK1A), transcript variant 5, mRNA [NM_130438]	1859
A_23_P211126	1.1	1.1	-2.1	1.1	DYRK1A	Homo sapiens dual-specificity tyrosine-(Y)-phosphorylation regulated kinase 1A (DYRK1A), transcript variant 2, mRNA [NM_130436]	1859

A_23_P90296	-1.1	2.4	-2.0	-1.0	DYRK1B	Homo sapiens dual-specificity tyrosine-(Y)-phosphorylation regulated kinase 1B (DYRK1B), transcript variant a, mRNA [NM_004714]	9149
A_23_P87902	1.1	-2.3	1.1	-1.1	DYRK4	Homo sapiens dual-specificity tyrosine-(Y)-phosphorylation regulated kinase 4 (DYRK4), mRNA [NM_003845]	8798
A_23_P39925	-4.6	-1.1	-1.8	-1.3	DYSF	Homo sapiens dysferlin, limb girdle muscular dystrophy 2B (autosomal recessive) (DYSF), transcript variant 8, mRNA [NM_003494]	8291

A_23_P39931	-1.2	4.1	5.2	4.5	DYSF	Homo sapiens dysferlin, limb girdle muscular dystrophy 2B (autosomal recessive) (DYSF), transcript variant 8, mRNA [NM_003494]	8291
A_24_P323425	1.7	1.8	-10.1	-1.3	DZANK1	Homo sapiens double zinc ribbon and ankyrin repeat domains 1 (DZANK1), mRNA [NM_001099407]	55184
A_24_P408297	1.9	-1.0	-4.2	1.1	DZANK1	Homo sapiens double zinc ribbon and ankyrin repeat domains 1 (DZANK1), mRNA [NM_001099407]	55184

A_23_P14157	1.1	1.1	1.1	-2.3	DZIP1	Homo sapiens DAZ interacting protein 1 (DZIP1), transcript variant 2, mRNA [NM_198968]	22873
A_23_P144165	-1.0	-2.2	-1.5	1.4	DZIP3	Homo sapiens DAZ interacting protein 3, zinc finger (DZIP3), mRNA [NM_014648]	9666
A_24_P102504	-1.3	-3.6	-6.8	-3.0	DZIP3	Homo sapiens DAZ interacting protein 3, zinc finger (DZIP3), mRNA [NM_014648]	9666
A_23_P408955	1.1	-28.2	1.0	-1.0	E2F2	Homo sapiens E2F transcription factor 2 (E2F2), mRNA [NM_004091]	1870

A_23_P125990	2.3	3.2	2.5	1.3	E2F2	Homo sapiens E2F transcription factor 2 (E2F2), mRNA [NM_004091]	1870
A_23_P152218	2.0	2.3	-2.9	-1.1	E2F4	Homo sapiens E2F transcription factor 4, p107/p130-binding (E2F4), mRNA [NM_001950]	1874
A_24_P318457	-1.0	-1.0	-1.1	-2.1	E2F4	Homo sapiens E2F transcription factor 4, p107/p130-binding (E2F4), mRNA [NM_001950]	1874
A_23_P15045	2.4	-3.3	-1.5	1.5	E4F1	Homo sapiens E4F transcription factor 1 (E4F1), mRNA [NM_004424]	1877

A_23_P252201	1.9	1.9	2.0	1.1	EAF2	Homo sapiens ELL associated factor 2 (EAF2), mRNA [NM_018456]	55840
A_24_P156501	1.0	-3.9	1.0	-1.0	EBF1	Homo sapiens early B-cell factor 1 (EBF1), mRNA [NM_024007]	1879
A_24_P43826	-2.8	1.0	-1.5	1.9	EBF2	Homo sapiens early B-cell factor 2 (EBF2), mRNA [NM_022659]	64641
A_23_P381645	-7.1	-1.7	1.0	1.8	EBF3	Homo sapiens early B-cell factor 3 (EBF3), mRNA [NM_001005463]	253738
A_32_P160127	2.2	2.2	-1.2	2.3	EBF3	Homo sapiens early B-cell factor 3 (EBF3), mRNA [NM_001005463]	253738

A_23_P109122	1.5	2.0	1.2	1.7	EBF4	Homo sapiens early B-cell factor 4 (EBF4), mRNA [NM_001110514]	57593
A_23_P119478	2.9	2.0	1.1	1.0	EBI3	Homo sapiens Epstein-Barr virus induced 3 (EBI3), mRNA [NM_005755]	10148
A_23_P115762	1.3	-2.4	-3.0	1.8	ECD	Homo sapiens ecdysoneless homolog (Drosophila) (ECD), transcript variant 1, mRNA [NM_007265]	11319
A_24_P154080	1.0	-12.2	1.0	1.0	ECE1	Homo sapiens endothelin converting enzyme 1 (ECE1), transcript variant 1, mRNA [NM_001397]	1889

A_23_P92261	-1.9	-2.1	-2.1	-2.2	ECE2	Homo sapiens endothelin converting enzyme 2 (ECE2), transcript variant 3, mRNA [NM_032331]	9718
A_23_P257786	10.0	5.3	13.3	12.6	ECE2	Homo sapiens endothelin converting enzyme 2 (ECE2), transcript variant 1, mRNA [NM_014693]	9718
A_23_P377299	1.1	-34.7	1.0	1.3	ECE2	Homo sapiens endothelin converting enzyme 2 (ECE2), transcript variant 1, mRNA [NM_014693]	9718
A_23_P153853	2.6	4.5	4.1	2.6	ECH1	Homo sapiens enoyl CoA hydratase 1, peroxisomal (ECH1), mRNA [NM_001398]	1891

A_23_P215051	-12.0	-1.0	-1.0	-1.0	ECHDC1	Homo sapiens enoyl CoA hydratase domain containing 1 (ECHDC1), transcript variant 2, mRNA [NM_018479]	55862
A_24_P225477	-1.0	-2.3	-1.2	-1.7	ECHDC2	Homo sapiens enoyl CoA hydratase domain containing 2 (ECHDC2), transcript variant 2, mRNA [NM_018281]	55268
A_23_P163647	-4.1	-1.4	-1.2	-2.6	ECI1	Homo sapiens enoyl-CoA delta isomerase 1 (ECI1), nuclear gene encoding mitochondrial protein, transcript variant 1, mRNA [NM_001919]	1632

A_23_P216434	-2.4	-2.6	-2.7	-2.6	ECM2	extracellular matrix protein 2, female organ and adipocyte specific [Source:HGNC Symbol;Acc:3154] [ENST00000375540]	1842
A_23_P44684	-7.1	1.2	1.1	-1.1	ECT2	Homo sapiens epithelial cell transforming sequence 2 oncogene (ECT2), mRNA [NM_018098]	1894
A_24_P100419	-1.4	-2.8	-1.0	-1.0	EDA	Homo sapiens ectodysplasin A (EDA), transcript variant 5, mRNA [NM_001005612]	1896
A_23_P171132	3.2	3.0	2.9	2.3	EDA2R	Homo sapiens ectodysplasin A2 receptor (EDA2R), transcript variant 2, mRNA [NM_021783]	60401

A_23_P503233	-1.0	-3.6	-1.1	-1.4	EDARADD	Homo sapiens EDAR-associated death domain (EDARADD), transcript variant B, mRNA [NM_080738]	128178
A_32_P223644	1.2	-2.2	1.3	1.3	EDC3	Homo sapiens enhancer of mRNA decapping 3 homolog (S. cerevisiae) (EDC3), transcript variant 3, mRNA [NM_025083]	80153
A_24_P399009	1.1	-2.2	-1.0	-1.0	EDC4	Homo sapiens enhancer of mRNA decapping 4 (EDC4), mRNA [NM_014329]	23644
A_23_P14414	6.2	6.2	1.2	6.1	EDDM3B	Homo sapiens epididymal protein 3B (EDDM3B), mRNA [NM_022360]	64184

A_24_P285768	8.6	1.8	9.7	7.1	EDEM1	Homo sapiens ER degradation enhancer, mannosidase alpha-like 1 (EDEM1), mRNA [NM_014674]	9695
A_23_P40975	-3.2	-1.1	-1.2	-1.0	EDEM1	Homo sapiens ER degradation enhancer, mannosidase alpha-like 1 (EDEM1), mRNA [NM_014674]	9695
A_23_P166193	2.8	2.7	1.9	2.2	EDEM2	Homo sapiens ER degradation enhancer, mannosidase alpha-like 2 (EDEM2), transcript variant 1, mRNA [NM_018217]	55741

A_23_P7337	-2.6	-2.7	-2.8	-2.7	EDNRA	Homo sapiens endothelin receptor type A (EDNRA), transcript variant 1, mRNA [NM_001957]	1909
A_24_P330263	2.2	-1.4	-1.1	1.2	EDNRB	Homo sapiens endothelin receptor type B (EDNRB), transcript variant 2, mRNA [NM_003991]	1910
A_23_P53217	-2.1	2.5	1.2	1.7	EED	Homo sapiens embryonic ectoderm development (EED), transcript variant 2, mRNA [NM_152991]	8726
A_32_P44316	1.3	-1.0	-28.5	1.2	EEF1A1	Homo sapiens eukaryotic translation elongation factor 1 alpha 1 (EEF1A1), mRNA [NM_001402]	1915

A_32_P47701	-1.8	-2.7	-2.5	-2.3	EEF1A1	Homo sapiens eukaryotic translation elongation factor 1 alpha 1 (EEF1A1), mRNA [NM_001402]	1915
A_23_P256033	-3.0	1.7	-1.1	1.1	EEF1A2	Homo sapiens eukaryotic translation elongation factor 1 alpha 2 (EEF1A2), mRNA [NM_001958]	1917
A_24_P734297	-1.4	2.0	1.7	1.3	EEF1D	Homo sapiens eukaryotic translation elongation factor 1 delta (guanine nucleotide exchange protein) (EEF1D), transcript variant 1, mRNA [NM_032378]	1936

A_23_P156842	2.3	1.1	-1.3	1.9	EEF1E1	Homo sapiens eukaryotic translation elongation factor 1 epsilon 1 (EEF1E1), transcript variant 1, mRNA [NM_004280]	9521
A_23_P333498	-1.1	-2.2	-1.2	-1.2	EEPD1	Homo sapiens endonuclease/exonuclease/phosphatase family domain containing 1 (EEPD1), mRNA [NM_030636]	80820
A_24_P415090	1.0	-4.0	-1.7	-1.0	EFCAB1	Homo sapiens EF-hand calcium binding domain 1 (EFCAB1), transcript variant 1, mRNA [NM_024593]	79645

A_23_P149476	2.4	1.3	1.4	1.1	EFCAB2	Homo sapiens EF-hand calcium binding domain 2 (EFCAB2), transcript variant 1, mRNA [NM_032328]	84288
A_32_P515971	-1.2	-3.1	-1.4	-1.1	EFCAB3	Homo sapiens EF-hand calcium binding domain 3 (EFCAB3), transcript variant 2, mRNA [NM_173503]	146779
A_32_P462013	-7.1	-1.9	-1.1	-2.8	EFCAB4B	EF-hand calcium binding domain 4B [Source:HGNC Symbol;Acc:28657] [ENST00000333750]	84766
A_23_P68978	-1.0	-3.3	-1.1	-1.1	EFCAB6	Homo sapiens EF-hand calcium binding domain 6 (EFCAB6), transcript variant 1, mRNA [NM_022785]	64800

A_24_P118608	1.4	2.0	1.4	-2.4	EFCAB7	Homo sapiens EF-hand calcium binding domain 7 (EFCAB7), mRNA [NM_032437]	84455
A_23_P24414	1.8	-2.5	1.2	1.2	EFEMP2	Homo sapiens EGF containing fibulin-like extracellular matrix protein 2 (EFEMP2), transcript variant 1, mRNA [NM_016938]	30008
A_23_P76598	-7.4	1.2	-3.1	1.1	EFHA1	Homo sapiens EF-hand domain family, member A1 (EFHA1), mRNA [NM_152726]	221154
A_23_P214291	-1.2	1.5	2.0	-2.4	EFHC1	Homo sapiens EF-hand domain (C-terminal) containing 1 (EFHC1), transcript variant A, mRNA [NM_018100]	114327

A_23_P154338	-1.3	-3.5	1.0	-1.8	EFHD1	Homo sapiens EF-hand domain family, member D1 (EFHD1), transcript variant 1, mRNA [NM_025202]	80303
A_24_P251053	-4.9	-5.3	-2.6	-3.4	EFHD2	Homo sapiens EF-hand domain family, member D2 (EFHD2), mRNA [NM_024329]	79180
A_23_P113005	-2.1	-1.6	-4.5	-1.6	EFNA1	Homo sapiens ephrin-A1 (EFNA1), transcript variant 1, mRNA [NM_004428]	1942
A_23_P119535	-1.5	1.5	1.0	-2.4	EFNA2	Homo sapiens ephrin-A2 (EFNA2), mRNA [NM_001405]	1943
A_24_P355944	-2.9	1.3	1.0	-1.2	EFNB2	Homo sapiens ephrin-B2 (EFNB2), mRNA [NM_004093]	1948

A_24_P940006	-1.5	-1.5	-1.5	-5.7	EFNB3	Homo sapiens ephrin-B3 (EFNB3), mRNA [NM_001406]	1949
A_23_P71513	-25.4	1.0	-1.1	1.4	EFR3A	Homo sapiens EFR3 homolog A (S. cerevisiae) (EFR3A), mRNA [NM_015137]	23167
A_23_P369654	-3.9	-1.2	-8.2	2.7	EFR3B	Homo sapiens mRNA for KIAA0953 protein, partial cds. [AB023170]	22979
A_24_P754817	-2.3	-1.2	-1.2	-1.3	EFTUD1	Homo sapiens elongation factor Tu GTP binding domain containing 1 (EFTUD1), transcript variant 1, mRNA [NM_024580]	79631

A_23_P155979	1.7	1.7	-2.1	1.5	EGF	Homo sapiens epidermal growth factor (EGF), transcript variant 1, mRNA [NM_001963]	1950
A_24_P68183	1.0	-1.7	-3.4	-1.0	EGFEM1P	Homo sapiens EGF-like and EMI domain containing 1, pseudogene (EGFEM1P), non-coding RNA [NR_021485]	93556
A_23_P168105	-1.7	2.1	-1.0	1.0	EGFL8	Homo sapiens EGF-like-domain, multiple 8 (EGFL8), transcript variant 1, mRNA [NM_030652]	80864
A_23_P424582	3.5	1.8	-1.1	1.5	EGFL8	Homo sapiens EGF-like-domain, multiple 8 (EGFL8), transcript variant 1, mRNA [NM_030652]	80864

A_24_P410363	1.3	1.5	-2.4	1.0	EGLN1	Homo sapiens egl nine homolog 1 (C. elegans) (EGLN1), mRNA [NM_022051]	54583
A_23_P34710	35.7	57.4	17.1	23.9	EGLN1	Homo sapiens egl nine homolog 1 (C. elegans) (EGLN1), mRNA [NM_022051]	54583
A_24_P322395	2.4	1.9	-1.4	1.2	EGLN2	Homo sapiens egl nine homolog 2 (C. elegans) (EGLN2), transcript variant 3, mRNA [NM_080732]	112398
A_23_P360379	2.3	2.3	2.0	1.4	EGLN3	Homo sapiens egl nine homolog 3 (C. elegans) (EGLN3), mRNA [NM_022073]	112399

A_32_P53908	-2.1	-1.1	1.4	-2.7	EHBP1	Homo sapiens EH domain binding protein 1 (EHBP1), transcript variant 1, mRNA [NM_015252]	23301
A_24_P194886	-1.5	2.7	1.2	2.7	EHBP1	Homo sapiens EH domain binding protein 1 (EHBP1), transcript variant 1, mRNA [NM_015252]	23301
A_23_P119390	-2.1	1.8	-1.8	1.8	EHD2	Homo sapiens EH-domain containing 2 (EHD2), mRNA [NM_014601]	30846
A_24_P64442	-2.1	-1.0	-1.0	-1.0	EHF	Homo sapiens ets homologous factor (EHF), transcript variant 1, mRNA [NM_001206616]	26298

A_24_P801451	-1.1	-1.3	-4.7	-4.6	EHMT2	Homo sapiens euchromatic histone-lysine N-methyltransferase 2 (EHMT2), transcript variant NG36/G9a, mRNA [NM_006709]	10919
A_24_P303390	-3.3	-1.4	1.0	1.1	EHMT2	Homo sapiens euchromatic histone-lysine N-methyltransferase 2 (EHMT2), transcript variant NG36/G9a-SPI, mRNA [NM_025256]	10919
A_24_P4661	-2.6	-3.8	-3.5	-1.5	EI24	Homo sapiens etoposide induced 2.4 mRNA (EI24), transcript variant 1, mRNA [NM_004879]	9538

A_23_P368259	1.6	1.6	1.5	-3.8	EID2B	Homo sapiens EP300 interacting inhibitor of differentiation 2B (EID2B), mRNA [NM_152361]	126272
A_23_P127467	1.7	4.1	1.8	2.6	EIF1AD	Homo sapiens eukaryotic translation initiation factor 1A domain containing (EIF1AD), transcript variant 3, mRNA [NM_032325]	84285
A_23_P148629	-1.4	1.8	-6.4	1.5	EIF1AY	Homo sapiens eukaryotic translation initiation factor 1A, Y-linked (EIF1AY), mRNA [NM_004681]	9086

A_24_P237511	1.0	-12.7	-1.0	-1.0	EIF1AY	Homo sapiens eukaryotic translation initiation factor 1A, Y-linked (EIF1AY), mRNA [NM_004681]	9086
A_23_P6891	1.0	-1.0	-1.1	-2.8	EIF1B	Homo sapiens eukaryotic translation initiation factor 1B (EIF1B), mRNA [NM_005875]	10289
A_32_P197524	1.1	1.1	-2.1	-1.0	EIF2A	Homo sapiens eukaryotic translation initiation factor 2A, 65kDa (EIF2A), mRNA [NM_032025]	83939

A_23_P142750	1.0	-2.5	1.0	1.0	EIF2AK2	Homo sapiens eukaryotic translation initiation factor 2-alpha kinase 2 (EIF2AK2), transcript variant 1, mRNA [NM_002759]	5610
A_23_P54540	1.8	2.0	1.6	-1.3	EIF2AK4	Homo sapiens eukaryotic translation initiation factor 2 alpha kinase 4 (EIF2AK4), mRNA [NM_001013703]	440275
A_23_P105313	-5.5	-1.3	1.4	-1.4	EIF2B1	Homo sapiens eukaryotic translation initiation factor 2B, subunit 1 alpha, 26kDa (EIF2B1), mRNA [NM_001414]	1967

A_23_P115046	-1.1	-2.0	-2.0	-2.1	EIF2B3	Homo sapiens eukaryotic translation initiation factor 2B, subunit 3 gamma, 58kDa (EIF2B3), transcript variant 1, mRNA [NM_020365]	8891
A_23_P154058	2.6	-1.0	-1.1	2.1	EIF2B4	Homo sapiens eukaryotic translation initiation factor 2B, subunit 4 delta, 67kDa (EIF2B4), transcript variant 2, mRNA [NM_001034116]	8890
A_24_P96749	5.0	5.2	4.6	4.2	EIF2C1	Homo sapiens eukaryotic translation initiation factor 2C, 1 (EIF2C1), mRNA [NM_012199]	26523

A_24_P941670	-5.0	1.0	-1.0	1.0	EIF2C4	Homo sapiens eukaryotic translation initiation factor 2C, 4 (EIF2C4), mRNA [NM_017629]	192670
A_24_P138556	-1.2	-3.9	-3.4	-4.4	EIF2S1	Homo sapiens eukaryotic translation initiation factor 2, subunit 1 alpha, 35kDa (EIF2S1), mRNA [NM_004094]	1965
A_23_P137031	3.4	3.2	2.0	1.1	EIF2S3	Homo sapiens eukaryotic translation initiation factor 2, subunit 3 gamma, 52kDa (EIF2S3), mRNA [NM_001415]	1968

A_23_P77572	-2.0	2.4	2.6	-1.9	EIF3C	Homo sapiens eukaryotic translation initiation factor 3, subunit C (EIF3C), transcript variant 2, mRNA [NM_001037808]	8663
A_23_P77568	-2.0	-2.0	-3.3	-2.0	EIF3C	Homo sapiens eukaryotic translation initiation factor 3, subunit C (EIF3C), transcript variant 2, mRNA [NM_001037808]	8663
A_23_P91699	-3.3	-1.0	-1.0	1.0	EIF3D	Homo sapiens eukaryotic translation initiation factor 3, subunit D (EIF3D), mRNA [NM_003753]	8664

A_32_P229760	-5.1	1.7	1.6	1.0	EIF3E	Homo sapiens eukaryotic translation initiation factor 3, subunit E (EIF3E), mRNA [NM_001568]	3646
A_23_P57521	3.1	1.1	1.8	1.9	EIF3L	Homo sapiens eukaryotic translation initiation factor 3, subunit L (EIF3L), transcript variant 1, mRNA [NM_016091]	51386
A_32_P68076	2.9	8.0	3.5	2.5	EIF3M	eukaryotic translation initiation factor 3, subunit M [Source:HGNC Symbol;Acc:24460] [ENST00000531120]	10480

A_32_P135902	-1.0	-1.5	-118.6	-1.0	EIF4A1	Homo sapiens eukaryotic translation initiation factor 4A1 (EIF4A1), transcript variant 1, mRNA [NM_001416]	1973
A_24_P390793	-3.0	-1.2	-2.6	-5.1	EIF4A1	Homo sapiens eukaryotic translation initiation factor 4A1 (EIF4A1), transcript variant 1, mRNA [NM_001416]	1973
A_23_P137103	-2.3	-1.6	-2.6	-1.4	EIF4A1	Homo sapiens eukaryotic translation initiation factor 4A1 (EIF4A1), transcript variant 1, mRNA [NM_001416]	1973

A_32_P110751	-4.3	-1.9	1.1	1.3	EIF4A2	Homo sapiens eukaryotic translation initiation factor 4A2 (EIF4A2), mRNA [NM_001967]	1974
A_23_P60286	-1.8	-2.1	-1.8	-1.0	EIF4B	Homo sapiens eukaryotic translation initiation factor 4B (EIF4B), mRNA [NM_001417]	1975
A_23_P162679	-3.5	-2.9	-2.0	-15.6	EIF4B	Homo sapiens eukaryotic translation initiation factor 4B (EIF4B), mRNA [NM_001417]	1975
A_32_P99648	-1.7	-2.2	-1.8	-3.8	EIF4E3	Homo sapiens eukaryotic translation initiation factor 4E family member 3 (EIF4E3), transcript variant 3, mRNA [NM_001134649]	317649

A_24_P943113	-1.2	-4.0	-1.3	-1.2	EIF4E3	Homo sapiens eukaryotic translation initiation factor 4E family member 3 (EIF4E3), transcript variant 2, mRNA [NM_173359]	317649
A_23_P22224	3.2	1.2	-1.0	1.1	EIF4EBP1	Homo sapiens eukaryotic translation initiation factor 4E binding protein 1 (EIF4EBP1), mRNA [NM_004095]	1978
A_23_P218892	2.6	2.5	-1.2	1.3	EIF4G1	Homo sapiens eukaryotic translation initiation factor 4 gamma, 1 (EIF4G1), transcript variant 1, mRNA [NM_182917]	1981

A_24_P383850	1.1	1.8	-1.1	3.4	EIF4G3	Homo sapiens eukaryotic translation initiation factor 4 gamma, 3 (EIF4G3), transcript variant 3, mRNA [NM_003760]	8672
A_23_P126241	2.4	1.6	2.1	-1.1	EIF4G3	Homo sapiens eukaryotic translation initiation factor 4 gamma, 3 (EIF4G3), transcript variant 3, mRNA [NM_003760]	8672
A_24_P149390	-1.1	-28.5	-1.1	-1.1	EIF4H	Homo sapiens eukaryotic translation initiation factor 4H (EIF4H), transcript variant 2, mRNA [NM_031992]	7458

A_32_P168886	-2.9	1.1	1.0	-1.1	EIF4H	Homo sapiens eukaryotic translation initiation factor 4H (EIF4H), transcript variant 1, mRNA [NM_022170]	7458
A_24_P720202	-5.3	-1.8	1.5	-1.1	EIF4H	Homo sapiens eukaryotic translation initiation factor 4H (EIF4H), transcript variant 1, mRNA [NM_022170]	7458
A_24_P415959	1.1	-3.4	1.0	-33.6	EIF5	Homo sapiens eukaryotic translation initiation factor 5 (EIF5), transcript variant 1, mRNA [NM_001969]	1983

A_24_P398810	-1.1	1.1	-3.1	-1.2	EIF5	Homo sapiens eukaryotic translation initiation factor 5 (EIF5), transcript variant 1, mRNA [NM_001969]	1983
A_23_P210939	-2.1	-1.4	-1.6	-1.8	EIF6	Homo sapiens eukaryotic translation initiation factor 6 (EIF6), transcript variant 2, mRNA [NM_181468]	3692
A_23_P208477	2.4	2.4	2.3	1.6	ELAVL1	Homo sapiens ELAV (embryonic lethal, abnormal vision, Drosophila)-like 1 (Hu antigen R) (ELAVL1), mRNA [NM_001419]	1994

A_24_P188447	-1.1	2.1	1.1	-1.1	ELAVL4	Homo sapiens ELAV (embryonic lethal, abnormal vision, Drosophila)-like 4 (Hu antigen D) (ELAVL4), transcript variant 1, mRNA [NM_021952]	1996
A_24_P944336	-2.7	2.0	2.3	1.3	ELAVL4	ELAV (embryonic lethal, abnormal vision, Drosophila)-like 4 (Hu antigen D) [Source:HGNC Symbol;Acc:3315] [ENST00000371827]	1996
A_23_P2801	-5.8	-1.1	-2.3	-3.4	ELF1	Homo sapiens E74-like factor 1 (ets domain transcription factor) (ELF1), transcript variant 1, mRNA [NM_172373]	1997

A_23_P132948	1.0	1.0	-7.0	-1.0	ELF2	Homo sapiens E74-like factor 2 (ets domain transcription factor) (ELF2), transcript variant 1, mRNA [NM_201999]	1998
A_23_P104188	-2.2	-3.3	-1.4	-1.7	ELF3	Homo sapiens E74-like factor 3 (ets domain transcription factor, epithelial-specific) (ELF3), transcript variant 1, mRNA [NM_004433]	1999
A_24_P7873	3.2	-1.7	1.3	-1.0	ELFN1	Homo sapiens extracellular leucine-rich repeat and fibronectin type III domain containing 1 (ELFN1), mRNA [NM_001128636]	392617

A_24_P133171	1.0	-1.1	-2.3	-3.4	ELK3	ELK3, ETS-domain protein (SRF accessory protein 2) [Source:HGNC Symbol;Acc:3325] [ENST00000228741]	2004
A_23_P161098	1.1	-3.5	-1.0	-1.0	ELK4	Homo sapiens ELK4, ETS-domain protein (SRF accessory protein 1) (ELK4), transcript variant a, mRNA [NM_001973]	2005
A_23_P27936	-2.2	1.2	-5.3	-1.9	ELL	Homo sapiens elongation factor RNA polymerase II (ELL), mRNA [NM_006532]	8178
A_23_P58506	5.0	4.5	5.5	3.3	ELL2	Homo sapiens elongation factor, RNA polymerase II, 2 (ELL2), mRNA [NM_012081]	22936

A_24_P47988	-4.3	-4.7	-3.1	-6.3	ELL3	Homo sapiens elongation factor RNA polymerase II-like 3 (ELL3), mRNA [NM_025165]	80237
A_24_P72420	-1.0	-2.5	-1.6	1.3	ELMO1	Homo sapiens engulfment and cell motility 1 (ELMO1), transcript variant 1, mRNA [NM_014800]	9844
A_23_P7282	-4.3	-1.1	1.1	-1.2	ELMOD2	Homo sapiens ELMO/CED-12 domain containing 2 (ELMOD2), mRNA [NM_153702]	255520
A_23_P305692	1.1	-7.0	-1.2	-1.0	ELMOD2	Homo sapiens ELMO/CED-12 domain containing 2 (ELMOD2), mRNA [NM_153702]	255520

A_24_P937435	-3.2	1.0	1.1	-1.2	ELMOD3	ELMO/CED-12 domain containing 3 [Source:HGNC Symbol;Acc:26158] [ENST00000488150]	84173
A_23_P154256	-1.2	1.2	2.2	-1.5	ELMOD3	Homo sapiens ELMO/CED-12 domain containing 3 (ELMOD3), transcript variant 1, mRNA [NM_032213]	84173
A_23_P215454	-3.6	-1.2	-1.1	-2.8	ELN	Homo sapiens elastin (ELN), transcript variant 1, mRNA [NM_000501]	2006
A_23_P130689	2.3	2.1	1.9	5.0	ELOF1	Homo sapiens elongation factor 1 homolog (S. cerevisiae) (ELOF1), mRNA [NM_032377]	84337

A_23_P149858	8.2	8.0	7.9	8.6	ELOVL3	Homo sapiens ELOVL fatty acid elongase 3 (ELOVL3), mRNA [NM_152310]	83401
A_23_P82065	-8.2	1.1	1.1	1.0	ELOVL4	Homo sapiens ELOVL fatty acid elongase 4 (ELOVL4), mRNA [NM_022726]	6785
A_32_P80597	1.1	1.1	-2.0	-1.5	ELOVL6	ELOVL fatty acid elongase 6 [Source:HGNC Symbol;Acc:15829] [ENST00000394607]	79071
A_23_P7361	-1.9	-2.1	-2.6	-3.7	ELOVL6	Homo sapiens ELOVL fatty acid elongase 6 (ELOVL6), transcript variant 1, mRNA [NM_024090]	79071

A_23_P359277	-7.8	-1.0	1.1	-1.0	ELOVL7	Homo sapiens ELOVL fatty acid elongase 7 (ELOVL7), transcript variant 1, mRNA [NM_024930]	79993
A_23_P78444	1.2	2.2	-10.3	1.3	ELP2	Homo sapiens elongation protein 2 homolog (S. cerevisiae) (ELP2), transcript variant 2, mRNA [NM_018255]	55250
A_24_P4110	3.5	3.5	3.5	1.5	ELP3	Homo sapiens elongation protein 3 homolog (S. cerevisiae) (ELP3), mRNA [NM_018091]	55140
A_32_P206541	-2.2	-1.1	-1.5	-2.0	EMB	Homo sapiens embigin (EMB), mRNA [NM_198449]	133418

A_24_P253215	1.1	4.5	4.7	1.1	EMG1	Homo sapiens EMG1 nucleolar protein homolog (S. cerevisiae) (EMG1), mRNA [NM_006331]	10436
A_24_P96449	-1.1	2.0	1.5	2.6	EMID1	Homo sapiens EMI domain containing 1 (EMID1), mRNA [NM_133455]	129080
A_23_P134627	-1.4	1.3	-5.4	-2.8	EMID2	Homo sapiens EMI domain containing 2 (EMID2), mRNA [NM_133457]	136227
A_23_P165848	-1.3	1.5	-2.6	1.3	EMILIN1	Homo sapiens elastin microfibril interfacer 1 (EMILIN1), mRNA [NM_007046]	11117

A_23_P27315	14.4	14.1	13.6	14.1	EMILIN2	Homo sapiens elastin microfibril interfacer 2 (EMILIN2), mRNA [NM_032048]	84034
A_32_P219845	-3.6	1.1	-2.2	1.1	EML1	echinoderm microtubule associated protein like 1 [Source:HGNC Symbol;Acc:3330] [ENST00000327921]	2009
A_23_P127793	1.6	2.0	1.0	-2.0	EML3	Homo sapiens echinoderm microtubule associated protein like 3 (EML3), mRNA [NM_153265]	256364

A_23_P143127	1.9	3.4	3.1	2.2	EML4	Homo sapiens echinoderm microtubule associated protein like 4 (EML4), transcript variant 1, mRNA [NM_019063]	27436
A_24_P863775	-2.9	-1.3	-1.4	-1.4	EML6	Homo sapiens echinoderm microtubule associated protein like 6 (EML6), mRNA [NM_001039753]	400954
A_23_P106682	-1.1	-1.1	-2.7	-1.4	EMP2	Homo sapiens epithelial membrane protein 2 (EMP2), mRNA [NM_001424]	2013
A_23_P304682	1.0	-1.0	-2.4	1.0	EMP2	Homo sapiens epithelial membrane protein 2 (EMP2), mRNA [NM_001424]	2013

A_24_P924611	18.2	17.0	22.0	17.8	EMR4P	Homo sapiens G protein-coupled receptor 127 (GPR127) mRNA, partial cds. [AY181245]	326342
A_23_P315122	-1.3	-1.8	-2.0	-3.4	EMX1	Homo sapiens empty spiracles homeobox 1 (EMX1), mRNA [NM_004097]	2016
A_23_P44264	-4.0	-1.2	-1.2	-1.5	EMX2	Homo sapiens empty spiracles homeobox 2 (EMX2), transcript variant 1, mRNA [NM_004098]	2018
A_23_P337767	-1.1	-2.4	-1.1	-1.1	ENAH	Homo sapiens enabled homolog (Drosophila) (ENAH), transcript variant 2, mRNA [NM_018212]	55740

A_23_P51397	-2.1	-1.0	-1.0	-1.0	ENAH	Homo sapiens enabled homolog (Drosophila) (ENAH), transcript variant 1, mRNA [NM_001008493]	55740
A_24_P347310	-2.0	-2.1	-2.2	-2.0	ENDOU	Homo sapiens endonuclease, polyU-specific (ENDOU), transcript variant 2, mRNA [NM_006025]	8909
A_23_P83328	1.8	2.1	1.6	1.4	ENG	Homo sapiens endoglin (ENG), transcript variant 2, mRNA [NM_000118]	2022
A_23_P152583	-1.6	-1.3	-1.9	-3.2	ENGASE	Homo sapiens endo-beta-N-acetylglucosaminidase (ENGASE), mRNA [NM_001042573]	64772

A_24_P115651	1.0	-1.0	-1.0	-2.9	ENKUR	Homo sapiens enkurin, TRPC channel interacting protein (ENKUR), mRNA [NM_145010]	219670
A_23_P137391	-1.9	1.5	2.0	-1.0	ENO1	Homo sapiens enolase 1, (alpha) (ENO1), transcript variant 1, mRNA [NM_001428]	2023
A_32_P145447	-2.6	1.0	-1.0	1.0	ENO1-AS1	Homo sapiens ENO1 antisense RNA 1 (non-protein coding) (ENO1-AS1), non-coding RNA [NR_038351]	100505975
A_23_P121806	-1.0	-3.9	-3.2	-1.0	ENOPH1	Homo sapiens enolase-phosphatase 1 (ENOPH1), mRNA [NM_021204]	58478

A_23_P156880	3.0	1.8	4.8	1.4	ENPP1	Homo sapiens ectonucleotide pyrophosphatase/phosphodiesterase 1 (ENPP1), mRNA [NM_006208]	5167
A_23_P10640	5.1	12.1	16.3	8.5	ENPP7	Homo sapiens ectonucleotide pyrophosphatase/phosphodiesterase 7 (ENPP7), mRNA [NM_178543]	339221
A_23_P410115	2.4	1.3	2.7	2.4	ENTHD1	Homo sapiens ENTH domain containing 1 (ENTHD1), mRNA [NM_152512]	150350

A_23_P24260	1.1	1.1	-4.2	1.1	ENTPD1	Homo sapiens ectonucleoside triphosphate diphosphohydrolase 1 (ENTPD1), transcript variant 1, mRNA [NM_001776]	953
A_24_P335025	-1.3	-1.1	-4.3	-1.5	ENTPD1	Homo sapiens ectonucleoside triphosphate diphosphohydrolase 1 (ENTPD1), transcript variant 1, mRNA [NM_001776]	953
A_23_P395365	7.8	6.0	4.2	5.6	ENTPD2	Homo sapiens ectonucleoside triphosphate diphosphohydrolase 2 (ENTPD2), transcript variant 2, mRNA [NM_001246]	954

A_24_P25452	-1.0	1.3	-5.7	-1.0	ENTPD5	Homo sapiens ectonucleoside triphosphate diphosphohydrolase 5, mRNA (cDNA clone IMAGE:3847878), with apparent retained intron. [BC020966]	957
A_24_P916585	1.6	1.6	2.7	1.0	ENTPD6	Homo sapiens IL-6SAG (IL-6SAG) mRNA, partial cds. [AF058296]	955
A_23_P44335	1.3	-1.0	-9.2	-1.1	ENTPD8	Homo sapiens ectonucleoside triphosphate diphosphohydrolase 8 (ENTPD8), transcript variant 1, mRNA [NM_001033113]	377841

A_24_P81149	1.7	2.7	1.9	1.9	ENY2	Homo sapiens enhancer of yellow 2 homolog (Drosophila) (ENY2), transcript variant 1, mRNA [NM_020189]	56943
A_23_P430120	1.3	-3.7	-1.4	-1.7	EPAS1	Homo sapiens endothelial PAS domain protein 1 (EPAS1), mRNA [NM_001430]	2034
A_23_P210210	1.5	1.4	2.1	1.2	EPAS1	Homo sapiens endothelial PAS domain protein 1 (EPAS1), mRNA [NM_001430]	2034

A_23_P160359	-2.7	1.0	1.3	-1.3	EPB41	Homo sapiens erythrocyte membrane protein band 4.1 (elliptocytosis 1, RH-linked) (EPB41), transcript variant 6, mRNA [NM_004437]	2035
A_23_P134109	1.0	-1.0	-1.1	-15.2	EPB41L2	Homo sapiens erythrocyte membrane protein band 4.1-like 2 (EPB41L2), transcript variant 1, mRNA [NM_001431]	2037
A_23_P4536	2.0	1.4	-1.5	1.4	EPB41L3	Homo sapiens erythrocyte membrane protein band 4.1-like 3 (EPB41L3), mRNA [NM_012307]	23136

A_24_P257579	-1.1	-11.3	-1.2	-1.2	EPB41L4A	Homo sapiens erythrocyte membrane protein band 4.1 like 4A (EPB41L4A), mRNA [NM_022140]	64097
A_23_P370514	1.1	2.2	1.0	1.0	EPB41L4B	Homo sapiens cDNA: FLJ21596 fis, clone COL07110. [AK025249]	54566
A_23_P416794	1.8	-2.8	1.7	-2.9	EPB41L5	Homo sapiens erythrocyte membrane protein band 4.1 like 5 (EPB41L5), transcript variant 1, mRNA [NM_020909]	57669

A_23_P140675	-2.4	4.5	3.4	1.8	EPB42	Homo sapiens erythrocyte membrane protein band 4.2 (EPB42), transcript variant 1, mRNA [NM_000119]	2038
A_24_P252718	-2.6	1.0	1.0	1.1	EPC1	enhancer of polycomb homolog 1 (Drosophila) [Source:HGNC Symbol;Acc:19876] [ENST00000319778]	80314
A_23_P359738	-13.0	-1.7	-1.9	1.0	EPC2	Homo sapiens enhancer of polycomb homolog 2 (Drosophila) (EPC2), mRNA [NM_015630]	26122

A_24_P341923	1.3	1.1	-1.9	-2.1	EPG5	Homo sapiens ectopic P-granules autophagy protein 5 homolog (C. elegans) (EPG5), mRNA [NM_020964]	57724
A_24_P392146	1.4	1.1	1.6	2.1	EPG5	Homo sapiens ectopic P-granules autophagy protein 5 homolog (C. elegans) (EPG5), mRNA [NM_020964]	57724
A_23_P385199	1.1	-1.1	-5.3	1.2	EPHA10	Homo sapiens EPH receptor A10 (EPHA10), transcript variant 2, mRNA [NM_173641]	284656

A_24_P930741	-1.3	1.0	-3.0	1.1	EPHA10	Homo sapiens EPH receptor A10 (EPHA10), transcript variant 3, mRNA [NM_001099439]	284656
A_23_P149281	-1.2	1.0	-2.8	-1.2	EPHA2	Homo sapiens EPH receptor A2 (EPHA2), mRNA [NM_004431]	1969
A_24_P254744	3.3	2.1	2.0	2.2	EPHA3	Homo sapiens EPH receptor A3 (EPHA3), transcript variant 2, mRNA [NM_182644]	2042
A_24_P274219	1.2	1.9	-19.5	2.1	EPHA4	Homo sapiens EPH receptor A4 (EPHA4), mRNA [NM_004438]	2043

A_23_P108501	2.1	-1.6	-1.0	1.0	EPHA4	Homo sapiens EPH receptor A4 (EPHA4), mRNA [NM_004438]	2043
A_24_P226332	1.4	-2.2	-1.4	1.5	EPHA7	Homo sapiens EPH receptor A7 (EPHA7), mRNA [NM_004440]	2045
A_23_P7835	1.1	-2.0	-1.3	-1.8	EPHA7	Homo sapiens EPH receptor A7 (EPHA7), mRNA [NM_004440]	2045
A_24_P34031	2.2	2.1	1.9	3.3	EPHA7	EPH receptor A7 [Source:HGNC Symbol;Acc:3390] [ENST00000369297]	2045
A_24_P419239	-2.3	1.2	-1.1	-1.0	EPHA8	Homo sapiens EPH receptor A8 (EPHA8), transcript variant 2, mRNA [NM_001006943]	2046

A_23_P318464	1.1	-3.2	2.1	1.0	EPHA8	Homo sapiens EPH receptor A8 (EPHA8), transcript variant 1, mRNA [NM_020526]	2046
A_23_P95060	-1.1	-1.9	-11.9	-2.0	EPHB3	Homo sapiens EPH receptor B3 (EPHB3), mRNA [NM_004443]	2049
A_23_P145935	1.5	1.7	-1.2	-2.0	EPHB6	Homo sapiens EPH receptor B6 (EPHB6), mRNA [NM_004445]	2051
A_23_P34537	-4.3	-1.0	-9.3	1.1	EPHX1	Homo sapiens epoxide hydrolase 1, microsomal (xenobiotic) (EPHX1), transcript variant 1, mRNA [NM_000120]	2052

A_23_P119593	5.0	4.5	1.2	-1.1	EPHX3	Homo sapiens epoxide hydrolase 3 (EPHX3), transcript variant 1, mRNA [NM_024794]	79852
A_23_P43898	2.0	1.1	-1.0	-1.0	EPHX4	Homo sapiens epoxide hydrolase 4 (EPHX4), mRNA [NM_173567]	253152
A_24_P239419	-2.3	1.2	-1.1	1.6	EPM2A	Homo sapiens epilepsy, progressive myoclonus type 2A, Lafora disease (laforin) (EPM2A), transcript variant 2, mRNA [NM_001018041]	7957

A_23_P170600	2.4	-1.0	1.0	2.2	EPM2A	Homo sapiens epilepsy, progressive myoclonus type 2A, Lafora disease (laforin) (EPM2A), transcript variant 1, mRNA [NM_005670]	7957
A_24_P627984	-1.0	-11.6	-1.0	-1.0	EPM2AIP1	Homo sapiens EPM2A (laforin) interacting protein 1 (EPM2AIP1), mRNA [NM_014805]	9852
A_24_P102343	1.2	-1.1	-1.0	-2.2	EPN3	Homo sapiens epsin 3 (EPN3), mRNA [NM_017957]	55040
A_23_P130027	-2.2	1.8	1.9	1.3	EPN3	Homo sapiens epsin 3 (EPN3), mRNA [NM_017957]	55040

A_23_P367899	1.1	1.0	-2.8	-2.1	EPOR	Homo sapiens erythropoietin receptor (EPOR), transcript variant 1, mRNA [NM_000121]	2057
A_24_P914376	3.7	3.2	6.3	4.5	EPOR	H.sapiens mRNA for soluble erythropoietin receptor. [X57282]	2057
A_24_P357169	2.2	-1.0	1.3	1.7	EPPK1	Homo sapiens epiplakin 1 (EPPK1), mRNA [NM_031308]	83481
A_23_P38941	2.3	-1.7	1.3	-1.7	EPS15L1	Homo sapiens epidermal growth factor receptor pathway substrate 15-like 1 (EPS15L1), mRNA [NM_021235]	58513

A_23_P136347	1.0	1.0	-1.0	2.6	EPS8	Homo sapiens epidermal growth factor receptor pathway substrate 8 (EPS8), mRNA [NM_004447]	2059
A_24_P940803	1.0	1.0	-2.9	-1.0	EPT1	Homo sapiens ethanolaminephosphotransferase 1 (CDP-ethanolamine-specific) (EPT1), mRNA [NM_033505]	85465
A_23_P68300	3.8	3.8	5.9	3.6	EPT1	Homo sapiens ethanolaminephosphotransferase 1 (CDP-ethanolamine-specific) (EPT1), mRNA [NM_033505]	85465

A_23_P30243	1.1	-1.4	5.6	1.1	ERAP2	Homo sapiens endoplasmic reticulum aminopeptidase 2 (ERAP2), transcript variant 1, mRNA [NM_022350]	64167
A_23_P89249	1.9	2.8	2.3	1.3	ERBB2	Homo sapiens v-erb-b2 erythroblastic leukemia viral oncogene homolog 2, neuro/glioblastoma derived oncogene homolog (avian) (ERBB2), transcript variant 2, mRNA [NM_001005862]	2064

A_23_P349416	1.2	-2.6	-1.1	-1.1	ERBB3	Homo sapiens v-erb-b2 erythroblastic leukemia viral oncogene homolog 3 (avian) (ERBB3), transcript variant 1, mRNA [NM_001982]	2065
A_24_P694760	-3.6	-1.0	1.0	1.0	ERC1	Homo sapiens ELKS/RAB6-interacting/CAST family member 1 (ERC1), transcript variant epsilon, mRNA [NM_178040]	23085
A_24_P249784	-2.0	-1.1	-1.1	-1.1	ERC1	Homo sapiens ELKS/RAB6-interacting/CAST family member 1 (ERC1), transcript variant epsilon, mRNA [NM_178040]	23085

A_23_P107701	1.1	-2.5	-4.2	-1.6	ERCC1	Homo sapiens excision repair cross-complementing rodent repair deficiency, complementation group 1 (includes overlapping antisense sequence) (ERCC1), transcript variant 2, mRNA [NM_001983]	2067
A_24_P401990	3.0	2.0	1.4	2.3	ERCC2	Homo sapiens excision repair cross-complementing rodent repair deficiency, complementation group 2 (ERCC2), transcript variant 1, mRNA [NM_000400]	2068

A_23_P117225	1.2	-8.8	1.1	1.1	ERCC5	Homo sapiens excision repair cross-complementing rodent repair deficiency, complementation group 5 (ERCC5), mRNA [NM_000123]	2073
A_23_P1292	2.8	2.7	2.6	2.8	ERCC6	Homo sapiens excision repair cross-complementing rodent repair deficiency, complementation group 6 (ERCC6), mRNA [NM_000124]	2074

A_24_P97770	1.4	1.5	1.6	-3.2	ERGIC1	Homo sapiens endoplasmic reticulum-golgi intermediate compartment (ERGIC) 1 (ERGIC1), mRNA [NM_001031711]	57222
A_23_P333227	1.3	-10.3	1.4	1.8	ERGIC1	Homo sapiens endoplasmic reticulum-golgi intermediate compartment (ERGIC) 1 (ERGIC1), mRNA [NM_001031711]	57222
A_24_P216313	-1.3	1.4	2.6	1.6	ERGIC3	Homo sapiens ERGIC and golgi 3 (ERGIC3), transcript variant 2, mRNA [NM_015966]	51614

A_23_P128734	-1.9	-2.2	1.3	-1.8	ERH	Homo sapiens enhancer of rudimentary homolog (Drosophila) (ERH), mRNA [NM_004450]	2079
A_24_P934800	-3.3	1.0	-1.1	-1.0	ERI2	Homo sapiens ERI1 exoribonuclease family member 2 (ERI2), transcript variant 1, mRNA [NM_001142725]	112479
A_32_P155416	2.8	1.0	1.2	1.9	ERI3	Homo sapiens ERI1 exoribonuclease family member 3 (ERI3), mRNA [NM_024066]	79033
A_23_P39971	1.9	2.1	2.1	2.1	ERLEC1	Homo sapiens endoplasmic reticulum lectin 1 (ERLEC1), transcript variant 1, mRNA [NM_015701]	27248

A_23_P52219	1.5	-3.2	1.2	5.7	ERLIN1	Homo sapiens ER lipid raft associated 1 (ERLIN1), mRNA [NM_006459]	10613
A_23_P123440	1.8	2.3	1.2	-5.6	ERLIN2	Homo sapiens ER lipid raft associated 2 (ERLIN2), transcript variant 1, mRNA [NM_007175]	11160
A_24_P276873	-7.5	-1.1	-1.2	-1.1	ERMN	Homo sapiens ermin, ERM-like protein (ERMN), transcript variant 2, mRNA [NM_020711]	57471
A_32_P387905	-2.0	-2.1	-1.1	-3.8	ERO1L	ERO1-like (S. cerevisiae) [Source:HGNC Symbol;Acc:13280] [ENST00000395686]	30001

A_23_P347623	1.1	-3.8	-1.0	-1.2	ERO1LB	Homo sapiens ERO1-like beta (S. cerevisiae) (ERO1LB), mRNA [NM_019891]	56605
A_23_P347618	-6.4	1.2	-10.1	-1.0	ERO1LB	Homo sapiens ERO1-like beta (S. cerevisiae) (ERO1LB), mRNA [NM_019891]	56605
A_23_P139687	2.2	2.2	1.1	2.1	ERP27	Homo sapiens endoplasmic reticulum protein 27 (ERP27), mRNA [NM_152321]	121506
A_23_P139929	-2.8	-2.5	-4.0	-3.3	ERP29	Homo sapiens endoplasmic reticulum protein 29 (ERP29), transcript variant 1, mRNA [NM_006817]	10961

A_23_P373927	-1.0	-7.8	1.0	-1.0	ERP44	Homo sapiens endoplasmic reticulum protein 44 (ERP44), mRNA [NM_015051]	23071
A_24_P11384	2.3	3.5	2.2	1.6	ERRFI1	Homo sapiens ERBB receptor feedback inhibitor 1 (ERRFI1), mRNA [NM_018948]	54206
A_24_P324577	-2.1	1.0	1.0	1.0	ERV3-2	Homo sapiens mRNA for KIAA1466 protein, partial cds. [AB040899]	57612
A_24_P324581	2.1	1.4	2.0	1.1	ERV3-2	Homo sapiens mRNA for KIAA1466 protein, partial cds. [AB040899]	57612
A_24_P248007	-2.0	-3.2	-1.8	-1.5	ERVH-3	Homo sapiens mRNA, endogenous retrovirus. [AB128832]	100289683

A_23_P95599	-1.0	2.4	1.9	1.3	ERVK13-1	Homo sapiens endogenous retrovirus group K13, member 1 (ERVK13-1), non-coding RNA [NR_040023]	100507321
A_32_P4466	1.3	-2.9	-2.3	-1.2	ERVV-2	Homo sapiens endogenous retrovirus group V, member 2 (ERVV-2), mRNA [NM_001191055]	100271846
A_23_P31443	-2.8	-3.8	-2.4	-1.3	ERVW-1	Homo sapiens endogenous retrovirus group W, member 1 (ERVW-1), transcript variant 1, mRNA [NM_014590]	30816
A_24_P13190	-7.8	1.6	1.4	-1.1	ESAM	Homo sapiens endothelial cell adhesion molecule (ESAM), mRNA [NM_138961]	90952

A_24_P37873	-5.7	1.3	-1.3	-1.2	ESM1	Homo sapiens endothelial cell-specific molecule 1 (ESM1), transcript variant 1, mRNA [NM_007036]	11082
A_32_P901770	1.9	2.4	2.3	2.4	ESPN	Homo sapiens espin (ESPN), mRNA [NM_031475]	83715
A_24_P383478	2.0	3.4	2.4	2.2	ESR1	Homo sapiens estrogen receptor 1 (ESR1), transcript variant 1, mRNA [NM_000125]	2099
A_23_P100220	1.8	1.6	-3.4	-1.0	ESRP2	Homo sapiens epithelial splicing regulatory protein 2 (ESRP2), mRNA [NM_024939]	80004

A_23_P391857	1.9	-1.2	2.6	1.2	ESRRB	Homo sapiens estrogen-related receptor beta (ESRRB), mRNA [NM_004452]	2103
A_24_P415928	1.3	1.3	-1.2	-2.3	ESRRB	Homo sapiens estrogen-related receptor beta (ESRRB), mRNA [NM_004452]	2103
A_24_P7965	4.1	6.4	-1.2	1.6	ESRRG	Homo sapiens estrogen-related receptor gamma (ESRRG), transcript variant 2, mRNA [NM_206594]	2104
A_23_P150852	1.5	2.5	1.4	-1.0	ESYT1	Homo sapiens extended synaptotagmin-like protein 1 (ESYT1), transcript variant 2, mRNA [NM_015292]	23344

A_24_P132470	1.5	1.4	-3.7	-1.0	ESYT2	Homo sapiens extended synaptotagmin-like protein 2 (ESYT2), mRNA [NM_020728]	57488
A_24_P697685	2.6	1.0	1.4	2.7	ESYT3	Homo sapiens extended synaptotagmin-like protein 3 (ESYT3), mRNA [NM_031913]	83850
A_23_P51117	4.0	3.9	30.9	3.7	ETAA1	Homo sapiens Ewing tumor-associated antigen 1 (ETAA1), mRNA [NM_019002]	54465

A_32_P119616	-1.3	-2.6	-1.4	-1.7	ETFA	Homo sapiens electron-transfer-flavoprotein, alpha polypeptide (ETFA), nuclear gene encoding mitochondrial protein, transcript variant 1, mRNA [NM_000126]	2108
A_23_P419239	-8.3	2.9	-1.5	1.9	ETNK1	Homo sapiens ethanolamine kinase 1 (ETNK1), transcript variant 1, mRNA [NM_018638]	55500
A_24_P751074	2.0	2.1	2.1	-7.8	ETS1	Homo sapiens v-ets erythroblastosis virus E26 oncogene homolog 1 (avian) (ETS1), transcript variant 2, mRNA [NM_005238]	2113

A_23_P400945	-1.0	-1.5	-2.0	-1.5	ETV3	Homo sapiens ets variant 3 (ETV3), transcript variant 2, mRNA [NM_005240]	2117
A_24_P416346	1.8	-1.3	2.1	1.5	ETV4	Homo sapiens ets variant 4 (ETV4), transcript variant 2, mRNA [NM_001079675]	2118
A_23_P9836	2.0	2.0	1.9	-3.1	ETV5	Homo sapiens ets variant 5 (ETV5), mRNA [NM_004454]	2119
A_24_P372862	1.9	2.0	2.1	1.0	ETV6	Homo sapiens ets variant 6 (ETV6), mRNA [NM_001987]	2120
A_23_P42353	4.2	-1.8	-2.1	-2.0	ETV7	Homo sapiens ets variant 7 (ETV7), transcript variant 1, mRNA [NM_016135]	51513

A_23_P167051	6.2	6.1	4.4	2.9	EVC	Ellis van Creveld syndrome [Source:HGNC Symbol;Acc:3497] [ENST00000382674]	2121
A_23_P78092	1.2	1.1	-1.1	-3.9	EVI2A	Homo sapiens ecotropic viral integration site 2A (EVI2A), transcript variant 1, mRNA [NM_001003927]	2123
A_23_P101427	-5.8	2.5	1.4	1.2	EVI5L	Homo sapiens ecotropic viral integration site 5-like (EVI5L), transcript variant 2, mRNA [NM_145245]	115704
A_24_P942648	-13.0	-1.0	-1.1	1.0	EVL	Homo sapiens mRNA; cDNA DKFZp586G1721 (from clone DKFZp586G1721). [AL133642]	51466

A_23_P140427	3.4	3.2	3.1	3.1	EVL	Homo sapiens Enah/Vasp-like (EVL), mRNA [NM_016337]	51466
A_32_P141682	-1.9	1.2	-3.1	-1.1	EVPLL	Homo sapiens envoplakin-like (EVPLL), mRNA [NM_001145127]	645027
A_24_P368943	-1.0	1.6	1.1	3.2	EVX1	Homo sapiens even-skipped homeobox 1 (EVX1), mRNA [NM_001989]	2128
A_23_P91657	1.3	1.6	-1.8	-3.0	EWSR1	Homo sapiens Ewing sarcoma breakpoint region 1 (EWSR1), transcript variant 1, mRNA [NM_013986]	2130

A_24_P384578	3.4	1.4	1.1	3.3	EWSR1	Homo sapiens Ewing sarcoma breakpoint region 1 (EWSR1), transcript variant 1, mRNA [NM_013986]	2130
A_24_P384573	5.8	4.2	-2.6	1.5	EWSR1	Homo sapiens Ewing sarcoma breakpoint region 1 (EWSR1), transcript variant 1, mRNA [NM_013986]	2130
A_23_P205296	6.6	4.1	2.4	4.3	EXD2	Homo sapiens exonuclease 3'-5' domain containing 2 (EXD2), transcript variant 5, mRNA [NM_018199]	55218

A_23_P170088	-1.0	-4.4	-1.2	-1.7	EXD3	Homo sapiens exonuclease 3'-5' domain containing 3, mRNA (cDNA clone IMAGE:6023681), complete cds. [BC065002]	54932
A_23_P214354	-4.0	1.1	1.1	-1.5	EXOC2	Homo sapiens exocyst complex component 2 (EXOC2), mRNA [NM_018303]	55770
A_23_P424734	1.2	1.4	1.5	-2.2	EXOC3L1	Homo sapiens exocyst complex component 3-like 1 (EXOC3L1), mRNA [NM_178516]	283849
A_23_P38860	2.4	1.6	1.4	-1.5	EXOC3L2	Homo sapiens exocyst complex component 3-like 2 (EXOC3L2), mRNA [NM_138568]	90332

A_24_P7946	-1.0	-2.1	-1.0	1.4	EXOC4	Homo sapiens exocyst complex component 4 (EXOC4), transcript variant 2, mRNA [NM_001037126]	60412
A_24_P392265	-3.6	1.7	1.6	1.0	EXOC5	Homo sapiens exocyst complex component 5 (EXOC5), mRNA [NM_006544]	10640
A_23_P14464	2.3	1.5	1.4	1.3	EXOC5	Homo sapiens exocyst complex component 5 (EXOC5), mRNA [NM_006544]	10640
A_32_P58280	-1.2	-2.2	-1.1	-1.8	EXOC6	Homo sapiens exocyst complex component 6 (EXOC6), transcript variant 1, mRNA [NM_019053]	54536

A_23_P415820	-1.0	-1.5	-1.3	-2.3	EXOC6B	Homo sapiens mRNA for KIAA0919 protein, partial cds. [AB023136]	23233
A_23_P32463	4.0	3.3	2.1	1.3	EXOSC4	Homo sapiens exosome component 4 (EXOSC4), mRNA [NM_019037]	54512
A_24_P211151	-1.0	-2.4	-10.9	-1.1	EXOSC5	Homo sapiens exosome component 5 (EXOSC5), mRNA [NM_020158]	56915
A_24_P39639	-6.1	-1.7	-2.0	-1.7	EXOSC6	Homo sapiens exosome component 6 (EXOSC6), mRNA [NM_058219]	118460
A_24_P937691	3.2	3.1	2.9	3.1	EXPH5	Homo sapiens exophilin 5 (EXPH5), mRNA [NM_015065]	23086

A_23_P13183	-14.8	1.1	-1.0	-1.0	EXT2	Homo sapiens exostosin 2 (EXT2), transcript variant 1, mRNA [NM_000401]	2132
A_23_P62999	-2.1	1.5	1.5	1.0	EXTL1	Homo sapiens exostoses (multiple)-like 1 (EXTL1), mRNA [NM_004455]	2134
A_24_P122658	-3.7	1.0	-1.0	1.0	EYA3	Homo sapiens eyes absent homolog 3 (Drosophila) (EYA3), mRNA [NM_001990]	2140
A_23_P167674	-1.2	-2.4	-2.4	-2.3	F12	Homo sapiens coagulation factor XII (Hageman factor) (F12), mRNA [NM_000505]	2161

A_32_P140139	1.4	1.0	1.4	-2.8	F13A1	Homo sapiens coagulation factor XIII, A1 polypeptide (F13A1), mRNA [NM_000129]	2162
A_23_P46549	1.1	-3.5	-1.9	-2.5	F13B	Homo sapiens coagulation factor XIII, B polypeptide (F13B), mRNA [NM_001994]	2165
A_32_P60065	-8.7	1.1	-1.0	1.0	F2RL2	Homo sapiens coagulation factor II (thrombin) receptor-like 2 (F2RL2), mRNA [NM_004101]	2151
A_23_P208422	-2.4	1.0	-1.0	1.0	F2RL3	Homo sapiens coagulation factor II (thrombin) receptor-like 3 (F2RL3), mRNA [NM_003950]	9002

A_23_P126782	1.0	-2.3	-1.0	-1.1	F3	Homo sapiens coagulation factor III (thromboplastin, tissue factor) (F3), transcript variant 1, mRNA [NM_001993]	2152
A_24_P172738	1.1	-1.3	2.1	1.1	F7	Homo sapiens coagulation factor VII (serum prothrombin conversion accelerator) (F7), transcript variant 1, mRNA [NM_000131]	2155
A_23_P117298	-1.4	5.4	1.4	1.9	F7	Homo sapiens coagulation factor VII (serum prothrombin conversion accelerator) (F7), transcript variant 1, mRNA [NM_000131]	2155

A_24_P314451	-2.7	-1.7	-3.7	-4.2	F8	Homo sapiens coagulation factor VIII, procoagulant component (F8), transcript variant 1, mRNA [NM_000132]	2157
A_23_P11262	6.3	9.2	5.9	5.0	F8A1	Homo sapiens coagulation factor VIII-associated 1 (F8A1), mRNA [NM_012151]	8263
A_23_P62406	-3.7	-1.0	-1.0	-1.0	F9	Homo sapiens coagulation factor IX (F9), mRNA [NM_000133]	2158
A_24_P558365	-1.9	-1.2	-2.2	-3.0	FABP12	Homo sapiens fatty acid binding protein 12 (FABP12), mRNA [NM_001105281]	646486

A_32_P5673	-1.0	3.2	-1.0	2.2	FABP3	602405126F1 NIH_MGC_21 Homo sapiens cDNA clone IMAGE:4542655 5', mRNA sequence [BG336702]	2170
A_23_P59877	2.0	2.0	2.0	1.0	FABP5	Homo sapiens fatty acid binding protein 5 (psoriasis-associated) (FABP5), mRNA [NM_001444]	2171
A_23_P43846	-2.2	1.0	-1.5	-1.5	FABP6	Homo sapiens fatty acid binding protein 6, ileal (FABP6), transcript variant 1, mRNA [NM_001040442]	2172

A_24_P278637	-1.3	-1.4	-2.3	-6.8	FADD	Homo sapiens Fas (TNFRSF6)-associated via death domain (FADD), mRNA [NM_003824]	8772
A_23_P98580	1.4	3.2	-1.1	-1.1	FADS2	Homo sapiens fatty acid desaturase 2 (FADS2), mRNA [NM_004265]	9415
A_23_P156109	-1.6	1.3	1.1	-2.9	FAF2	Homo sapiens Fas associated factor family member 2 (FAF2), mRNA [NM_014613]	23197

A_23_P118300	3.1	3.0	4.4	3.0	FAHD1	Homo sapiens fumarylacetoacetate hydrolase domain containing 1 (FAHD1), nuclear gene encoding mitochondrial protein, transcript variant 2, mRNA [NM_031208]	81889
A_23_P5731	-1.2	-1.2	-2.5	-3.8	FAHD2A	Homo sapiens fumarylacetoacetate hydrolase domain containing 2A (FAHD2A), mRNA [NM_016044]	51011
A_23_P139891	7.9	5.6	5.5	5.6	FAIM2	Homo sapiens Fas apoptotic inhibitory molecule 2 (FAIM2), mRNA [NM_012306]	23017

A_23_P138125	-1.3	-1.2	-2.7	-1.1	FAIM3	Homo sapiens Fas apoptotic inhibitory molecule 3 (FAIM3), transcript variant 1, mRNA [NM_005449]	9214
A_23_P356021	2.2	3.2	2.1	1.8	FANCB	Homo sapiens Fanconi anemia, complementation group B (FANCB), transcript variant 1, mRNA [NM_001018113]	2187
A_32_P24165	-4.6	1.1	1.1	1.0	FANCD2	Homo sapiens Fanconi anemia, complementation group D2 (FANCD2), transcript variant 2, mRNA [NM_001018115]	2177

A_23_P404211	-1.9	-2.0	-2.2	-4.4	FANCM	Homo sapiens Fanconi anemia, complementation group M (FANCM), mRNA [NM_020937]	57697
A_24_P163920	1.5	1.3	-4.1	-1.9	FAR1	Homo sapiens fatty acyl CoA reductase 1 (FAR1), mRNA [NM_032228]	84188
A_23_P150903	-1.0	-3.0	-4.5	-4.6	FAR2	Homo sapiens fatty acyl CoA reductase 2 (FAR2), mRNA [NM_018099]	55711
A_23_P90855	-1.9	1.1	-2.9	-1.2	FARP2	Homo sapiens FERM, RhoGEF and pleckstrin domain protein 2 (FARP2), mRNA [NM_014808]	9855

A_23_P78685	1.6	3.0	2.8	-1.1	FARSA	Homo sapiens phenylalanyl-tRNA synthetase, alpha subunit (FARSA), mRNA [NM_004461]	2193
A_23_P502930	2.2	1.5	1.8	1.6	FASTK	Homo sapiens Fas-activated serine/threonine kinase (FASTK), transcript variant 1, mRNA [NM_006712]	10922
A_23_P28590	-2.9	-1.2	-1.9	2.1	FASTKD1	Homo sapiens FAST kinase domains 1 (FASTKD1), mRNA [NM_024622]	79675
A_23_P209649	8.5	8.4	8.0	8.3	FASTKD2	Homo sapiens FAST kinase domains 2 (FASTKD2), transcript variant 3, mRNA [NM_014929]	22868

A_23_P58489	1.1	1.1	1.1	-2.2	FASTKD3	Homo sapiens FAST kinase domains 3 (FASTKD3) , transcript variant 1, mRNA [NM_024091]	79072
A_23_P380724	1.0	1.0	-8.2	1.0	FASTKD5	Homo sapiens FAST kinase domains 5 (FASTKD5) , mRNA [NM_021826]	60493
A_32_P206415	-1.0	2.2	-1.0	-1.0	FAT3	Homo sapiens FAT tumor suppressor homolog 3 (Drosophila) (FAT3), mRNA [NM_001008781]	120114
A_24_P73848	-1.2	-1.3	-2.5	-1.3	FBF1	Homo sapiens cDNA FLJ32699 fis, clone TESTI2000435, weakly similar to CALDESMON. [AK057261]	85302

A_23_P23457	1.1	-4.1	-1.5	1.2	FBLIM1	Homo sapiens filamin binding LIM protein 1 (FBLIM1), transcript variant 2, mRNA [NM_001024215]	54751
A_24_P94222	-1.0	1.1	-1.3	-11.9	FBLIM1	Homo sapiens filamin binding LIM protein 1 (FBLIM1), transcript variant 1, mRNA [NM_017556]	54751
A_23_P102462	1.9	-1.1	-1.1	4.1	FBLN7	Homo sapiens fibulin 7 (FBLN7), transcript variant 1, mRNA [NM_153214]	129804
A_23_P65678	-2.6	-1.1	-1.1	-1.1	FBN1	Homo sapiens fibrillin 1 (FBN1), mRNA [NM_000138]	2200
A_23_P101893	-1.3	-2.8	-3.0	-1.6	FBN3	Homo sapiens fibrillin 3 (FBN3), mRNA [NM_032447]	84467

A_23_P257111	-1.0	-1.1	-1.2	-2.2	FBP1	Homo sapiens fructose-1,6-bisphosphatase 1 (FBP1), transcript variant 1, mRNA [NM_000507]	2203
A_24_P279263	2.8	1.5	-1.0	1.9	FBRS	Homo sapiens fibrosin (FBRS), mRNA [NM_001105079]	64319
A_24_P280497	-2.6	1.3	1.2	1.3	FBRSL1	Homo sapiens fibrosin-like 1 (FBRSL1), mRNA [NM_001142641]	57666
A_23_P36724	1.9	-1.2	-2.6	-1.3	FBXL14	Homo sapiens F-box and leucine-rich repeat protein 14 (FBXL14), mRNA [NM_152441]	144699

A_23_P12635	-4.1	1.8	1.1	1.3	FBXL15	Homo sapiens F-box and leucine-rich repeat protein 15 (FBXL15), mRNA [NM_024326]	79176
A_23_P396322	3.1	3.4	3.4	2.9	FBXL17	F-box and leucine-rich repeat protein 17 [Source:HGNC Symbol;Acc:13615] [ENST00000496714]	64839
A_23_P423957	-3.6	1.1	-1.2	-1.4	FBXL18	Homo sapiens F-box and leucine-rich repeat protein 18 (FBXL18), mRNA [NM_024963]	80028
A_24_P307854	4.3	5.4	4.0	1.6	FBXL18	Homo sapiens F-box and leucine-rich repeat protein 18 (FBXL18), mRNA [NM_024963]	80028

A_23_P141146	1.6	1.6	2.0	1.4	FBXL20	Homo sapiens F-box and leucine-rich repeat protein 20 (FBXL20), transcript variant 1, mRNA [NM_032875]	84961
A_32_P334875	-1.5	-1.5	-1.9	-2.9	FBXL22	Homo sapiens F-box and leucine-rich repeat protein 22 (FBXL22), mRNA [NM_203373]	283807
A_23_P140069	1.1	1.0	1.1	-2.2	FBXL3	Homo sapiens F-box and leucine-rich repeat protein 3 (FBXL3), mRNA [NM_012158]	26224
A_32_P108748	1.1	-15.0	1.5	1.0	FBXL5	F-box and leucine-rich repeat protein 5 [Source:HGNC Symbol;Acc:13602] [ENST00000514541]	26234

A_23_P43296	-1.1	1.7	-1.1	2.6	FBXL6	Homo sapiens F-box and leucine-rich repeat protein 6 (FBXL6), transcript variant 1, mRNA [NM_012162]	26233
A_24_P7600	23.3	9.2	12.8	9.0	FBXL7	Homo sapiens F-box and leucine-rich repeat protein 7 (FBXL7), mRNA [NM_012304]	23194
A_32_P57453	-1.0	-1.4	-1.1	-3.6	FBXO10	Homo sapiens F-box protein 10 (FBXO10), mRNA [NM_012166]	26267
A_24_P357536	2.9	2.9	3.0	2.9	FBXO11	Homo sapiens F-box protein 11 (FBXO11), transcript variant 4, mRNA [NM_001190274]	80204

A_23_P356101	2.2	2.1	1.5	1.7	FBXO11	Homo sapiens F-box protein 11 (FBXO11), transcript variant 4, mRNA [NM_001190274]	80204
A_23_P342709	-1.0	-1.4	-2.1	-1.8	FBXO15	Homo sapiens F-box protein 15 (FBXO15), transcript variant 1, mRNA [NM_152676]	201456
A_23_P45999	-4.4	1.1	1.2	1.6	FBXO2	Homo sapiens F-box protein 2 (FBXO2), mRNA [NM_012168]	26232
A_24_P13715	1.6	2.1	2.1	2.1	FBXO22	Homo sapiens F-box protein 22 (FBXO22), transcript variant 1, mRNA [NM_147188]	26263
A_23_P65870	-1.6	-2.5	-1.5	1.3	FBXO22	Homo sapiens F-box protein 22 (FBXO22), transcript variant 2, mRNA [NM_012170]	26263

A_23_P70888	1.0	-1.3	-3.0	-1.7	FBXO24	Homo sapiens F-box protein 24 (FBXO24), transcript variant 1, mRNA [NM_033506]	26261
A_23_P363831	-2.6	1.2	-6.1	1.5	FBXO3	Homo sapiens F-box protein 3 (FBXO3), transcript variant 2, mRNA [NM_033406]	26273
A_23_P256231	-2.1	-1.0	-1.0	1.0	FBXO30	Homo sapiens F-box protein 30 (FBXO30), mRNA [NM_032145]	84085
A_24_P919493	2.3	2.3	2.3	2.4	FBXO34	Homo sapiens F-box protein 34 (FBXO34), transcript variant 1, mRNA [NM_017943]	55030
A_23_P117527	-1.0	-1.1	-5.3	-2.1	FBXO34	Homo sapiens F-box protein 34 (FBXO34), transcript variant 1, mRNA [NM_017943]	55030

A_23_P146811	-1.8	-1.8	-2.5	-2.9	FBXO38	Homo sapiens F-box protein 38 (FBXO38), transcript variant 2, mRNA [NM_205836]	81545
A_23_P121013	-3.5	-1.9	-1.6	-5.1	FBXO40	Homo sapiens F-box protein 40 (FBXO40), mRNA [NM_016298]	51725
A_23_P8241	-1.2	-1.2	-4.7	-1.3	FBXO5	Homo sapiens F-box protein 5 (FBXO5), transcript variant 1, mRNA [NM_012177]	26271
A_23_P97064	3.0	-1.8	-1.1	1.4	FBXO6	Homo sapiens F-box protein 6 (FBXO6), mRNA [NM_018438]	26270
A_24_P106522	-1.0	-3.0	-1.1	5.2	FBXO9	Homo sapiens F-box protein 9 (FBXO9), transcript variant 2, mRNA [NM_033480]	26268

A_23_P218358	-2.2	-2.2	-2.3	-2.3	FBXW10	Homo sapiens F-box and WD repeat domain containing 10 (FBXW10), mRNA [NM_031456]	10517
A_24_P257539	-1.2	-1.2	-7.7	1.0	FBXW11	Homo sapiens F-box and WD repeat domain containing 11 (FBXW11), transcript variant 3, mRNA [NM_012300]	23291
A_23_P254648	8.1	10.0	7.8	1.0	FBXW2	Homo sapiens F-box and WD repeat domain containing 2 (FBXW2), mRNA [NM_012164]	26190

A_23_P104295	-1.1	-2.4	-2.6	1.3	FBXW4	Homo sapiens F-box and WD repeat domain containing 4 (FBXW4), mRNA [NM_022039]	6468
A_24_P174613	-1.6	-1.5	-2.4	-1.7	FBXW7	Homo sapiens F-box and WD repeat domain containing 7 (FBXW7), transcript variant 1, mRNA [NM_033632]	55294
A_24_P44279	1.7	4.1	2.5	1.6	FBXW8	Homo sapiens F-box and WD repeat domain containing 8 (FBXW8), transcript variant 1, mRNA [NM_153348]	26259

A_23_P48266	-4.8	2.1	3.5	1.5	FBXW8	Homo sapiens F-box and WD repeat domain containing 8 (FBXW8), transcript variant 1, mRNA [NM_153348]	26259
A_23_P201510	2.3	5.3	4.1	-3.8	FCAMR	Homo sapiens Fc receptor, IgA, IgM, high affinity (FCAMR), transcript variant 3, mRNA [NM_001170631]	83953
A_23_P160902	-12.1	-1.1	-1.2	-1.1	FCAMR	Homo sapiens Fc receptor, IgA, IgM, high affinity (FCAMR), transcript variant 3, mRNA [NM_001170631]	83953

A_23_P208768	-3.2	-1.0	-1.1	-1.0	FCAR	Homo sapiens Fc fragment of IgA, receptor for (FCAR), transcript variant 1, mRNA [NM_002000]	2204
A_23_P85716	1.4	-1.2	-2.0	-2.6	FCGR2A	Homo sapiens Fc fragment of IgG, low affinity IIa, receptor (CD32) (FCGR2A), transcript variant 2, mRNA [NM_021642]	2212
A_23_P34644	-1.1	-2.2	-3.2	-4.8	FCGR2B	Homo sapiens Fc fragment of IgG, low affinity IIb, receptor (CD32) (FCGR2B), transcript variant 1, mRNA [NM_004001]	2213

A_23_P55936	4.3	10.5	2.4	9.5	FCGRT	Homo sapiens Fc fragment of IgG, receptor, transporter, alpha (FCGRT), transcript variant 2, mRNA [NM_004107]	2217
A_32_P459533	-11.2	-11.6	-12.2	-9.5	FCHO1	Homo sapiens FCH domain only 1 (FCHO1), transcript variant 2, mRNA [NM_015122]	23149
A_23_P343826	1.6	2.5	1.5	2.0	FCHSD1	Homo sapiens FCH and double SH3 domains 1 (FCHSD1), mRNA [NM_033449]	89848
A_23_P157879	-7.0	-1.5	-1.4	-2.3	FCN1	Homo sapiens ficolin (collagen/fibrinogen domain containing) 1 (FCN1), mRNA [NM_002003]	2219

A_23_P115246	-5.1	1.3	1.9	-2.3	FCN3	Homo sapiens ficolin (collagen/fibrinogen domain containing) 3 (Hakata antigen) (FCN3), transcript variant 1, mRNA [NM_003665]	8547
A_24_P319647	2.3	3.0	2.8	1.0	FCRL2	Homo sapiens Fc receptor-like 2 (FCRL2), mRNA [NM_030764]	79368
A_23_P358438	-3.3	-1.1	-1.4	-1.1	FCRL3	Homo sapiens Fc receptor-like 3 (FCRL3), mRNA [NM_052939]	115352
A_23_P115201	1.1	1.1	-1.0	-10.0	FCRL4	Homo sapiens Fc receptor-like 4 (FCRL4), mRNA [NM_031282]	83417

A_24_P285158	7.5	8.9	-1.1	5.3	FCRLB	Fc receptor-like B [Source:HGNC Symbol;Acc:26431] [ENST00000495397]	127943
A_23_P350574	20.7	19.5	18.9	19.2	FCRLB	Homo sapiens Fc receptor-like B (FCRLB), mRNA [NM_001002901]	127943
A_24_P246293	-9.1	1.0	-1.0	1.0	FDXACB1	Homo sapiens ferredoxin-fold anticodon binding domain containing 1 (FDXACB1), transcript variant 1, mRNA [NM_138378]	91893

A_32_P151933	1.1	-4.0	1.0	1.1	FECH	Homo sapiens ferrochelatase (FECH), nuclear gene encoding mitochondrial protein, transcript variant 1, mRNA [NM_001012515]	2235
A_23_P151970	-2.2	1.6	-3.1	-2.0	FEM1B	Homo sapiens fem-1 homolog b (C. elegans) (FEM1B), mRNA [NM_015322]	10116
A_24_P84898	-5.4	-1.0	-1.0	1.2	FEN1	Homo sapiens flap structure-specific endonuclease 1 (FEN1), mRNA [NM_004111]	2237

A_32_P104053	-1.0	-1.1	-3.4	1.1	FER	fer (fps/fes related) tyrosine kinase [Source:HGNC Symbol;Acc:3655] [ENST00000281092]	2241
A_23_P80048	2.5	1.4	1.5	1.4	FER1L4	Homo sapiens fer-1-like 4 (C. elegans) pseudogene (FER1L4), non-coding RNA [NR_024377]	80307
A_32_P366349	-5.7	3.0	2.7	1.7	FER1L5	Homo sapiens fer-1-like 5 (C. elegans) (FER1L5), mRNA [NM_001113382]	90342
A_23_P322013	2.3	2.0	2.6	1.8	FER1L6-AS1	Homo sapiens FER1L6 antisense RNA 1 (non-protein coding) (FER1L6-AS1), non-coding RNA [NR_040044]	439941

A_23_P422849	5.2	5.0	4.2	4.1	FERD3L	Homo sapiens Fer3-like (Drosophila) (FERD3L), mRNA [NM_152898]	222894
A_24_P365506	-5.0	1.2	-2.3	1.1	FERMT1	Homo sapiens fermitin family member 1 (FERMT1), mRNA [NM_017671]	55612
A_23_P64044	1.8	1.6	1.4	2.2	FERMT3	Homo sapiens fermitin family member 3 (FERMT3), transcript variant URP2LF, mRNA [NM_178443]	83706
A_24_P405631	1.2	2.3	-1.4	-1.5	FEZF2	Homo sapiens FEZ family zinc finger 2 (FEZF2), mRNA [NM_018008]	55079
A_23_P39356	2.5	1.7	2.5	1.8	FFAR1	Homo sapiens free fatty acid receptor 1 (FFAR1), mRNA [NM_005303]	2864

A_23_P397391	2.3	-1.1	-1.8	1.3	FFAR2	Homo sapiens free fatty acid receptor 2 (FFAR2), mRNA [NM_005306]	2867
A_23_P56239	9.4	7.3	8.3	3.5	FFAR3	Homo sapiens free fatty acid receptor 3 (FFAR3), mRNA [NM_005304]	2865
A_24_P282251	4.1	4.1	2.4	3.1	FGA	Homo sapiens fibrinogen alpha chain (FGA), transcript variant alpha, mRNA [NM_021871]	2243
A_24_P143138	3.5	2.5	1.9	2.5	FGD1	Homo sapiens FYVE, RhoGEF and PH domain containing 1 (FGD1), mRNA [NM_004463]	2245

A_23_P73559	-1.4	-2.9	1.0	-1.9	FGD1	Homo sapiens FYVE, RhoGEF and PH domain containing 1 (FGD1), mRNA [NM_004463]	2245
A_24_P140788	-2.5	-1.0	-1.0	1.0	FGD2	Homo sapiens FYVE, RhoGEF and PH domain containing 2 (FGD2), mRNA [NM_173558]	221472
A_23_P134980	3.8	1.5	1.1	2.5	FGD3	Homo sapiens FYVE, RhoGEF and PH domain containing 3 (FGD3), transcript variant 2, mRNA [NM_033086]	89846
A_32_P11579	-1.1	-1.1	-2.9	-2.8	FGF12	Homo sapiens fibroblast growth factor 12 (FGF12), transcript variant 1, mRNA [NM_021032]	2257

A_24_P85009	-2.4	-2.0	-1.9	-2.8	FGF13	Homo sapiens fibroblast growth factor 13 (FGF13), transcript variant 4, mRNA [NM_001139498]	2258
A_23_P88033	6.6	6.5	6.5	7.3	FGF14	Homo sapiens fibroblast growth factor 14 (FGF14), transcript variant 2, mRNA [NM_175929]	2259
A_23_P254896	-1.2	-1.2	-3.3	-2.3	FGF16	Homo sapiens fibroblast growth factor 16 (FGF16), mRNA [NM_003868]	8823
A_23_P101564	-1.0	-1.3	1.5	-3.2	FGF21	Homo sapiens fibroblast growth factor 21 (FGF21), mRNA [NM_019113]	26291

A_24_P337239	2.3	2.3	-1.2	-1.4	FGF5	Homo sapiens fibroblast growth factor 5 (FGF5), transcript variant 1, mRNA [NM_004464]	2250
A_23_P212800	-3.4	1.1	-1.3	1.1	FGF5	Homo sapiens fibroblast growth factor 5 (FGF5), transcript variant 1, mRNA [NM_004464]	2250
A_23_P105705	-1.0	-1.1	-1.4	-2.3	FGF6	Homo sapiens fibroblast growth factor 6 (FGF6), mRNA [NM_020996]	2251
A_23_P46829	2.3	2.2	2.2	2.5	FGF8	Homo sapiens fibroblast growth factor 8 (androgen-induced) (FGF8), transcript variant F, mRNA [NM_033163]	2253

A_24_P201381	-1.7	-1.8	-4.6	-5.3	FGFBP3	Homo sapiens fibroblast growth factor binding protein 3 (FGFBP3), mRNA [NM_152429]	143282
A_23_P219105	1.1	1.1	1.0	-4.5	FGFR1	Homo sapiens fibroblast growth factor receptor 1 (FGFR1), transcript variant 11, mRNA [NM_001174064]	2260
A_23_P372923	1.0	1.7	-3.9	1.0	FGFR1	Homo sapiens fibroblast growth factor receptor 1 (FGFR1), transcript variant 13, mRNA [NM_001174066]	2260
A_23_P315131	-19.0	-1.1	-1.1	-1.1	FGFR1	Homo sapiens cDNA FLJ14326 fis, clone PLACE4000247. [AK024388]	2260

A_23_P202334	-2.1	-1.7	-1.4	-7.8	FGFR2	Homo sapiens fibroblast growth factor receptor 2 (FGFR2), transcript variant 2, mRNA [NM_022970]	2263
A_23_P303149	-2.6	-1.1	-2.3	-3.1	FGFR2	Homo sapiens fibroblast growth factor receptor 2 (FGFR2), transcript variant 1, mRNA [NM_000141]	2263
A_24_P206624	3.3	-1.2	2.7	2.7	FGFR2	Homo sapiens fibroblast growth factor receptor 2 (FGFR2), transcript variant 2, mRNA [NM_022970]	2263
A_23_P500501	1.0	1.0	-1.1	-8.8	FGFR3	Homo sapiens fibroblast growth factor receptor 3 (FGFR3), transcript variant 1, mRNA [NM_000142]	2261

A_23_P92349	1.7	-3.2	1.6	1.8	FGFRL1	Homo sapiens fibroblast growth factor receptor-like 1 (FGFRL1), transcript variant 1, mRNA [NM_001004356]	53834
A_23_P148088	-2.8	2.2	1.0	1.2	FGG	Homo sapiens fibrinogen gamma chain (FGG), transcript variant gamma-A, mRNA [NM_000509]	2266
A_23_P42969	1.7	-2.2	2.2	-1.2	FGL2	Homo sapiens fibrinogen-like 2 (FGL2), mRNA [NM_006682]	10875

A_24_P316634	1.0	1.0	1.0	-2.5	FGR	Homo sapiens Gardner-Rasheed feline sarcoma viral (v-fgr) oncogene homolog (FGR), transcript variant 2, mRNA [NM_001042747]	2268
A_24_P391840	1.1	-1.2	-1.0	-3.3	FHAD1	Homo sapiens mRNA for KIAA1937 protein, partial cds. [AB067524]	114827
A_32_P61495	1.0	-1.0	-1.6	-2.6	FHAD1	Homo sapiens forkhead-associated (FHA) phosphopeptide binding domain 1 (FHAD1), mRNA [NM_052929]	114827

A_23_P125171	1.1	-2.1	1.2	-1.1	FHIT	Homo sapiens fragile histidine triad gene (FHIT), transcript variant 1, mRNA [NM_002012]	2272
A_23_P125164	4.6	1.4	1.7	1.8	FHIT	Homo sapiens fragile histidine triad gene (FHIT), transcript variant 1, mRNA [NM_002012]	2272
A_23_P217326	-18.7	-1.4	-1.4	-5.5	FHL1	Homo sapiens four and a half LIM domains 1 (FHL1), transcript variant 2, mRNA [NM_001449]	2273
A_23_P42358	-2.7	-1.7	-1.9	-1.9	FHL5	Homo sapiens four and a half LIM domains 5 (FHL5), transcript variant 1, mRNA [NM_020482]	9457

A_23_P37778	2.5	2.7	2.4	1.4	FHOD1	Homo sapiens formin homology 2 domain containing 1 (FHOD1), mRNA [NM_013241]	29109
A_32_P34444	-1.0	-8.9	-1.2	-1.1	FHOD3	Homo sapiens formin homology 2 domain containing 3 (FHOD3), mRNA [NM_025135]	80206
A_23_P1615	1.0	-2.0	-2.2	1.3	FIBP	Homo sapiens fibroblast growth factor (acidic) intracellular binding protein (FIBP), transcript variant 2, mRNA [NM_004214]	9158

A_23_P145541	1.0	-1.0	-1.0	-4.4	FIG4	Homo sapiens FIG4 homolog, SAC1 lipid phosphatase domain containing (S. cerevisiae) (FIG4), mRNA [NM_014845]	9896
A_32_P34003	-4.9	1.0	1.3	1.1	FIGN	fidgetin [Source:HGNC Symbol;Acc:13285] [ENST00000333129]	55137
A_23_P165909	-1.1	-3.0	-2.6	-3.3	FIGN	Homo sapiens fidgetin (FIGN), mRNA [NM_018086]	55137
A_23_P410233	-2.0	1.0	-1.1	-1.1	FILIP1	Homo sapiens mRNA; cDNA DKFZp451B134 (from clone DKFZp451B134); complete cds. [AL832009]	27145

A_23_P436369	-1.3	-9.2	-1.3	-1.3	FILIP1	Homo sapiens filamin A interacting protein 1 (FILIP1), mRNA [NM_015687]	27145
A_23_P252052	2.0	10.6	14.0	2.2	FILIP1L	Homo sapiens filamin A interacting protein 1-like (FILIP1L), transcript variant 1, mRNA [NM_182909]	11259
A_23_P20183	1.0	1.0	-5.5	1.1	FIS1	Homo sapiens fission 1 (mitochondrial outer membrane) homolog (S. cerevisiae) (FIS1), nuclear gene encoding mitochondrial protein, mRNA [NM_016068]	51024

A_24_P203407	-28.0	-1.4	1.2	-1.2	FITM2	Homo sapiens fat storage-inducing transmembrane protein 2 (FITM2), mRNA [NM_001080472]	128486
A_23_P154667	-2.0	-1.2	-1.1	-1.2	FKBP1A	Homo sapiens FK506 binding protein 1A, 12kDa (FKBP1A), transcript variant 1, mRNA [NM_000801]	2280
A_23_P142631	3.8	2.5	4.3	3.2	FKBP1B	Homo sapiens FK506 binding protein 1B, 12.6 kDa (FKBP1B), transcript variant 2, mRNA [NM_054033]	2281
A_24_P38081	-1.7	-1.0	-2.3	-1.0	FKBP5	Homo sapiens FK506 binding protein 5 (FKBP5), transcript variant 1, mRNA [NM_004117]	2289

A_23_P79622	1.1	1.1	-2.9	1.0	FKBP7	Homo sapiens FK506 binding protein 7 (FKBP7), transcript variant 1, mRNA [NM_181342]	51661
A_23_P376211	2.1	2.2	2.1	1.4	FKBP9	Homo sapiens FK506 binding protein 9, 63 kDa (FKBP9), mRNA [NM_007270]	11328
A_23_P70566	1.8	4.5	4.1	4.1	FKBPL	Homo sapiens FK506 binding protein like (FKBPL), mRNA [NM_022110]	63943
A_23_P255827	-5.9	1.0	-1.0	1.7	FKSG2	Homo sapiens apoptosis inhibitor (FKSG2) mRNA, complete cds. [AF300871]	59347

A_23_P33927	1.1	1.2	-7.4	1.1	FKSG43	Homo sapiens FERM domain-containing protein 8 pseudogene (FKSG43), non-coding RNA [NR_033742]	83957
A_23_P300087	2.4	2.8	4.2	1.8	FKSG83	Homo sapiens FKSG83 (FKSG83), mRNA [NM_032030]	83954
A_23_P34527	2.1	-1.4	1.2	1.4	FLAD1	Homo sapiens FAD1 flavin adenine dinucleotide synthetase homolog (S. cerevisiae) (FLAD1), transcript variant 1, mRNA [NM_025207]	80308
A_23_P311410	1.0	-1.0	-9.2	1.0	FLCN	folliculin [Source:HGNC Symbol;Acc:27310] [ENST00000466317]	201163

A_24_P51322	2.0	2.0	2.5	2.4	FLG	Homo sapiens filaggrin (FLG), mRNA [NM_002016]	2312
A_23_P96568	2.6	-7.7	2.2	1.9	FLNA	Homo sapiens filamin A, alpha (FLNA), transcript variant 1, mRNA [NM_001456]	2316
A_24_P683917	1.0	2.9	-1.1	1.0	FLNB	filamin B, beta [Source:HGNC Symbol;Acc:3755] [ENST00000477629]	2317
A_24_P77968	-1.6	-1.4	-1.8	2.1	FLNC	Homo sapiens filamin C, gamma (FLNC), transcript variant 1, mRNA [NM_001458]	2318
A_24_P794833	3.9	7.3	-1.1	3.1	FLOT1	flotillin 1 [Source:HGNC Symbol;Acc:3757] [ENST00000474138]	10211

A_23_P55319	1.9	3.0	2.6	1.5	FLOT2	Homo sapiens flotillin 2 (FLOT2), mRNA [NM_004475]	2319
A_24_P253818	-1.0	-1.1	-4.5	-1.0	FLOT2	Homo sapiens flotillin 2 (FLOT2), mRNA [NM_004475]	2319
A_24_P236235	-2.5	1.9	1.3	-1.2	FLRT2	Homo sapiens fibronectin leucine rich transmembrane protein 2 (FLRT2), mRNA [NM_013231]	23768
A_23_P99442	1.1	1.0	1.0	-2.8	FLT3	Homo sapiens fms-related tyrosine kinase 3 (FLT3), mRNA [NM_004119]	2322
A_23_P213394	1.7	2.5	-1.1	1.4	FLT4	Homo sapiens fms-related tyrosine kinase 4 (FLT4), transcript variant 1, mRNA [NM_182925]	2324

A_32_P197511	-1.1	-1.9	-3.3	-1.2	FLYWCH2	Homo sapiens FLYWCH family member 2 (FLYWCH2), transcript variant 1, mRNA [NM_138439]	114984
A_32_P180825	-12.4	-1.0	-1.3	-1.0	FMN1	Homo sapiens formin 1 (FMN1), mRNA [NM_001103184]	342184
A_32_P229132	-3.1	1.2	1.1	1.1	FMN2	Homo sapiens formin 2 (FMN2), mRNA [NM_020066]	56776
A_24_P390668	1.2	-2.2	-1.3	-1.7	FMNL1	Homo sapiens formin-like 1 (FMNL1), mRNA [NM_005892]	752
A_32_P193080	1.3	2.2	1.8	-1.2	FMNL2	Homo sapiens formin-like 2 (FMNL2), mRNA [NM_052905]	114793

A_23_P379200	3.5	3.4	1.0	3.5	FMNL3	Homo sapiens formin-like 3 (FMNL3), transcript variant 1, mRNA [NM_175736]	91010
A_24_P692566	2.4	1.0	-1.2	2.2	FMO1	flavin containing monooxygenase 1 [Source:HGNC Symbol;Acc:3769] [ENST00000469112]	2326
A_23_P155601	-4.3	1.3	-1.5	1.3	FMO3	Homo sapiens flavin containing monooxygenase 3 (FMO3), transcript variant 2, mRNA [NM_001002294]	2328
A_23_P89215	-1.3	-2.1	1.3	-1.8	FN3K	Homo sapiens fructosamine 3 kinase (FN3K), mRNA [NM_022158]	64122

A_24_P205130	-1.3	1.1	-1.1	-9.5	FNBP1	Homo sapiens formin binding protein 1 (FNBP1), mRNA [NM_015033]	23048
A_23_P253434	-4.2	1.4	-1.1	-1.0	FNDC3B	Homo sapiens fibronectin type III domain containing 3B (FNDC3B), transcript variant 1, mRNA [NM_022763]	64778
A_23_P66552	-5.9	1.0	1.0	1.0	FNDC8	Homo sapiens fibronectin type III domain containing 8 (FNDC8), mRNA [NM_017559]	54752
A_23_P306489	-1.1	-1.4	-2.2	-1.6	FNDC9	Homo sapiens fibronectin type III domain containing 9 (FNDC9), mRNA [NM_001001343]	408263

A_24_P222300	1.4	3.5	1.3	1.9	FNIP1	Homo sapiens folliculin interacting protein 1 (FNIP1), transcript variant 1, mRNA [NM_133372]	96459
A_32_P190737	-1.0	1.7	1.8	3.5	FNIP2	folliculin interacting protein 2 [Source:HGNC Symbol;Acc:29280] [ENST00000264433]	57600
A_24_P830025	1.4	-2.9	1.4	1.3	FNTA	farnesyltransferase, CAAX box, alpha [Source:HGNC Symbol;Acc:3782] [ENST00000525099]	2339
A_23_P24926	-5.2	1.2	2.4	1.1	FNTA	Homo sapiens farnesyltransferase, CAAX box, alpha (FNTA), transcript variant 1, mRNA [NM_002027]	2339

A_23_P25835	8.5	13.6	5.1	15.2	FNTB	Homo sapiens farnesyltransferase, CAAX box, beta (FNTB), mRNA [NM_002028]	2342
A_23_P47616	-1.2	-2.2	-1.4	-1.5	FOLH1	Homo sapiens folate hydrolase (prostate-specific membrane antigen) 1 (FOLH1), transcript variant 1, mRNA [NM_004476]	2346
A_23_P53176	1.9	1.9	2.4	1.2	FOLR1	Homo sapiens folate receptor 1 (adult) (FOLR1), transcript variant 1, mRNA [NM_016725]	2348
A_24_P32935	2.2	2.0	3.0	1.1	FOLR2	Homo sapiens folate receptor 2 (fetal) (FOLR2), transcript variant 1, mRNA [NM_000803]	2350

A_24_P206702	2.1	1.2	1.5	1.8	FOLR4	Homo sapiens folate receptor 4 (delta) homolog (mouse) (FOLR4), mRNA [NM_001199206]	390243
A_24_P247587	1.2	-2.7	1.2	-2.3	FONG	Homo sapiens uncharacterized LOC348751 (FONG), non-coding RNA [NR_034096]	348751
A_23_P161624	1.0	1.1	1.5	-2.6	FOSL1	Homo sapiens FOS-like antigen 1 (FOSL1), mRNA [NM_005438]	8061
A_24_P365523	-2.2	-1.9	1.2	-1.5	FOXA2	Homo sapiens forkhead box A2 (FOXA2), transcript variant 1, mRNA [NM_021784]	3170
A_23_P142174	1.4	-1.2	1.9	2.0	FOXA3	Homo sapiens forkhead box A3 (FOXA3), mRNA [NM_004497]	3171

A_23_P140527	-2.9	-2.3	-1.1	-1.4	FOXB1	Homo sapiens forkhead box B1 (FOXB1), mRNA [NM_012182]	27023
A_32_P205110	1.1	-4.7	2.4	-1.0	FOXC1	Homo sapiens forkhead box C1 (FOXC1), mRNA [NM_001453]	2296
A_24_P82358	3.9	4.1	3.8	5.2	FOXC2	Homo sapiens forkhead box C2 (MFH-1, mesenchyme forkhead 1) (FOXC2), mRNA [NM_005251]	2303
A_23_P86171	-2.2	1.2	-1.1	-1.1	FOXD2	Homo sapiens forkhead box D2 (FOXD2), mRNA [NM_004474]	2306
A_23_P413585	1.6	-1.4	-2.5	1.1	FOXD2	Homo sapiens forkhead box D2 (FOXD2), mRNA [NM_004474]	2306

A_32_P119248	-7.2	-1.2	-1.3	-1.2	FOXD4	Homo sapiens forkhead box D4 (FOXD4), mRNA [NM_207305]	2298
A_23_P358370	-3.8	1.3	-1.9	-2.4	FOXE1	H.sapiens HFKH4 mRNA for fork head like protein. [X94553]	2304
A_23_P216664	1.6	1.9	3.4	1.3	FOXE1	Homo sapiens forkhead box E1 (thyroid transcription factor 2) (FOXE1), mRNA [NM_004473]	2304
A_23_P112666	7.4	11.9	4.6	9.7	FOXE3	Homo sapiens forkhead box E3 (FOXE3), mRNA [NM_012186]	2301
A_23_P82959	2.9	3.2	-1.4	-1.6	FOXH1	Homo sapiens forkhead box H1 (FOXH1), mRNA [NM_003923]	8928

A_24_P168033	1.3	-1.8	1.7	-2.1	FOXJ1	Homo sapiens forkhead box J1 (FOXJ1), mRNA [NM_001454]	2302
A_23_P204238	-1.0	-4.9	-1.0	1.0	FOXJ2	Homo sapiens forkhead box J2 (FOXJ2), mRNA [NM_018416]	55810
A_24_P416301	1.1	-7.4	-1.2	-2.1	FOXK2	Homo sapiens forkhead box K2 (FOXK2), mRNA [NM_004514]	3607
A_23_P365081	-8.5	1.5	2.1	-1.1	FOXL1	Homo sapiens forkhead box L1 (FOXL1), mRNA [NM_005250]	2300
A_32_P140898	-1.0	1.1	-1.1	-3.4	FOXN2	Homo sapiens forkhead box N2 (FOXN2), mRNA [NM_002158]	3344
A_23_P151426	3.2	3.4	2.7	1.3	FOXO1	Homo sapiens forkhead box O1 (FOXO1), mRNA [NM_002015]	2308

A_23_P345575	2.7	3.7	1.5	3.0	FOXO3	Homo sapiens forkhead box O3 (FOXO3), transcript variant 1, mRNA [NM_001455]	2309
A_32_P102062	1.6	2.1	2.1	1.0	FOXO3	Homo sapiens forkhead box O3 (FOXO3), transcript variant 1, mRNA [NM_001455]	2309
A_23_P217487	-5.1	1.1	1.2	-1.5	FOXO4	Homo sapiens forkhead box O4 (FOXO4), transcript variant 1, mRNA [NM_005938]	4303
A_24_P38316	-31.2	-1.2	-1.1	-1.0	FOXP2	Homo sapiens forkhead box P2 (FOXP2), transcript variant 2, mRNA [NM_148898]	93986

A_23_P258703	-2.0	1.2	-2.0	-1.3	FOXP4	Homo sapiens forkhead box P4 (FOXP4), transcript variant 1, mRNA [NM_001012426]	116113
A_24_P204011	2.0	6.2	4.2	4.0	FOXR1	Homo sapiens forkhead box R1 (FOXR1), mRNA [NM_181721]	283150
A_24_P147765	-1.8	-1.9	-1.1	-2.0	FOXRED2	Homo sapiens FAD-dependent oxidoreductase domain containing 2 (FOXRED2), transcript variant 1, mRNA [NM_024955]	80020

A_23_P369948	-3.9	-1.0	-1.0	-1.0	FOXRED2	Homo sapiens FAD-dependent oxidoreductase domain containing 2 (FOXRED2), transcript variant 1, mRNA [NM_024955]	80020
A_24_P230570	1.5	1.2	1.0	6.3	FOXS1	Homo sapiens forkhead box S1 (FOXS1), mRNA [NM_004118]	2307
A_23_P9465	1.5	2.1	1.6	1.4	FPGS	Homo sapiens folylpolyglutamate synthase (FPGS), nuclear gene encoding mitochondrial protein, transcript variant 1, mRNA [NM_004957]	2356

A_23_P200030	1.2	-2.3	1.2	1.1	FPGT	Homo sapiens fucose-1-phosphate guanylyltransferase (FPGT), transcript variant 1, mRNA [NM_003838]	8790
A_32_P107876	1.0	2.3	1.1	2.5	FRAS1	Homo sapiens Fraser syndrome 1 (FRAS1), transcript variant 1, mRNA [NM_025074]	80144
A_23_P81717	2.1	1.9	1.2	1.9	FRMD1	Homo sapiens FERM domain containing 1 (FRMD1), transcript variant 1, mRNA [NM_024919]	79981
A_24_P374741	2.0	1.1	1.0	1.7	FRMD4A	FERM domain containing 4A [Source:HGNC Symbol;Acc:25491] [ENST00000492155]	55691

A_32_P709988	2.1	2.0	-1.0	1.9	FRMD4A	FERM domain containing 4A [Source:HGNC Symbol;Acc:25491] [ENST00000493380]	55691
A_24_P942321	1.0	3.1	-1.1	-1.1	FRMD4A	Homo sapiens FERM domain containing 4A (FRMD4A), mRNA [NM_018027]	55691
A_24_P43959	-2.1	1.7	1.6	1.4	FRMD4A	Homo sapiens FERM domain containing 4A (FRMD4A), mRNA [NM_018027]	55691
A_23_P169460	-7.0	1.4	1.2	-1.2	FRMPD1	Homo sapiens FERM and PDZ domain containing 1 (FRMPD1), mRNA [NM_014907]	22844

A_23_P86710	1.7	1.7	-2.2	1.6	FRMPD2	Homo sapiens FERM and PDZ domain containing 2 (FRMPD2), transcript variant 3, mRNA [NM_001018071]	143162
A_32_P129669	2.3	1.6	2.6	1.4	FRMPD3	PREDICTED: Homo sapiens FERM and PDZ domain containing 3 (FRMPD3), mRNA [XM_042978]	84443
A_24_P940275	-3.0	1.0	-1.0	-1.4	FRMPD4	Homo sapiens FERM and PDZ domain containing 4 (FRMPD4), mRNA [NM_014728]	9758

A_23_P430785	-1.1	-1.6	-2.7	-1.2	FRS2	Homo sapiens fibroblast growth factor receptor substrate 2 (FRS2), transcript variant 1, mRNA [NM_006654]	10818
A_23_P167970	1.5	1.6	-1.8	-2.2	FRS3	Homo sapiens fibroblast growth factor receptor substrate 3 (FRS3), mRNA [NM_006653]	10817
A_24_P68229	8.0	4.2	3.6	3.7	FRYL	Homo sapiens FRY-like (FRYL), mRNA [NM_015030]	285527
A_24_P308109	-1.0	-1.1	-16.6	-1.1	FRYL	Homo sapiens FRY-like (FRYL), mRNA [NM_015030]	285527
A_23_P10902	1.0	2.2	1.2	-1.0	FRZB	Homo sapiens frizzled-related protein (FRZB), mRNA [NM_001463]	2487

A_23_P168531	1.7	1.2	2.3	1.3	FSCN1	Homo sapiens fascin homolog 1, actin-bundling protein (Strongylocentrotus purpuratus) (FSCN1), mRNA [NM_003088]	6624
A_23_P84496	2.4	1.5	-1.1	1.3	FSCN2	Homo sapiens fascin homolog 2, actin-bundling protein, retinal (Strongylocentrotus purpuratus) (FSCN2), transcript variant 1, mRNA [NM_012418]	25794
A_23_P153651	-1.7	-3.4	-1.5	-1.2	FSD1	Homo sapiens fibronectin type III and SPRY domain containing 1 (FSD1), mRNA [NM_024333]	79187

A_23_P65901	2.1	1.0	1.2	1.0	FSD2	Homo sapiens mRNA; cDNA DKFZp451 H129 (from clone DKFZp451 H129). [AL833295]	123722
A_23_P353125	-1.5	-1.7	-2.9	1.2	FSIP1	Homo sapiens fibrous sheath interacting protein 1 (FSIP1), mRNA [NM_1525 97]	161835
A_23_P212696	1.7	1.1	2.1	-1.5	FSTL1	Homo sapiens follistatin-like 1 (FSTL1), mRNA [NM_0070 85]	11167
A_23_P91552	1.9	-1.8	2.6	1.2	FTCD	Homo sapiens formimino transferas e cyclodeam inase (FTCD), transcript variant A, mRNA [NM_2069 65]	10841

A_24_P58337	2.8	1.6	2.0	3.0	FTH1	Homo sapiens ferritin, heavy polypeptide 1 (FTH1), mRNA [NM_002032]	2495
A_32_P111565	-1.2	1.8	1.6	-3.3	FTH1	Homo sapiens ferritin, heavy polypeptide 1 (FTH1), mRNA [NM_002032]	2495
A_32_P155247	-1.8	-1.7	-2.2	2.1	FTL	Homo sapiens ferritin, light polypeptide (FTL), mRNA [NM_000146]	2512
A_23_P50498	1.9	2.6	-1.7	1.1	FTL	Homo sapiens ferritin, light polypeptide (FTL), mRNA [NM_000146]	2512

A_23_P367596	3.6	-1.4	1.4	5.4	FTMT	Homo sapiens ferritin mitochondrial (FTMT), nuclear gene encoding mitochondrial protein, mRNA [NM_177478]	94033
A_23_P113184	-1.1	-4.8	-1.1	-1.6	FTO	Homo sapiens fat mass and obesity associated (FTO), mRNA [NM_001080432]	79068
A_23_P22473	2.1	1.1	1.2	1.7	FTSJ1	Homo sapiens FtsJ homolog 1 (E. coli) (FTSJ1), transcript variant 1, mRNA [NM_012280]	24140
A_24_P357183	3.0	3.5	1.0	1.6	FTSJ1	Homo sapiens FtsJ homolog 1 (E. coli) (FTSJ1), transcript variant 3, mRNA [NM_177439]	24140

A_24_P363477	1.8	1.9	2.2	-2.6	FTSJ2	Homo sapiens FtsJ homolog 2 (E. coli) (FTSJ2), mRNA [NM_013393]	29960
A_23_P55091	-6.3	2.2	-1.2	1.1	FTSJ3	Homo sapiens FtsJ homolog 3 (E. coli) (FTSJ3), mRNA [NM_017647]	117246
A_24_P410378	-2.0	1.0	1.0	-1.0	FTSJD1	Homo sapiens FtsJ methyltransferase domain containing 1 (FTSJD1), transcript variant 1, mRNA [NM_018348]	55783
A_23_P45699	-2.3	-1.3	-2.4	-1.8	FUBP1	Homo sapiens far upstream element (FUSE) binding protein 1 (FUBP1), mRNA [NM_003902]	8880

A_23_P435833	1.2	-1.2	-2.4	-1.6	FUBP3	Homo sapiens far upstream element (FUSE) binding protein 3 (FUBP3), mRNA [NM_003934]	8939
A_23_P375549	-1.3	-2.0	-1.4	-1.2	FUNDC1	Homo sapiens FUN14 domain containing 1 (FUNDC1), mRNA [NM_173794]	139341
A_32_P459423	-1.6	-1.5	-3.7	-2.8	FUNDC2	Homo sapiens DC44 mRNA, complete cds. [AF267862]	65991
A_23_P360316	2.4	-1.0	-1.1	-1.0	FUT3	Homo sapiens fucosyltransferase 3 (galactoside 3(4)-L-fucosyltransferase, Lewis blood group) (FUT3), transcript variant 1, mRNA [NM_000149]	2525

A_24_P295609	1.0	1.0	-5.6	1.0	FUT4	Homo sapiens fucosyltransferase 4 (alpha (1,3) fucosyltransferase, myeloid-specific) (FUT4), mRNA [NM_002033]	2526
A_23_P101759	-1.6	-1.6	-1.4	-2.2	FUT5	Homo sapiens fucosyltransferase 5 (alpha (1,3) fucosyltransferase) (FUT5), mRNA [NM_002034]	2527
A_23_P252664	1.1	1.2	1.0	2.3	FUT6	Human alpha (1,3) fucosyltransferase (FUT6) mRNA, major transcript I, complete cds. [U27333]	2528

A_23_P5092	1.8	-1.0	2.5	1.2	FUT6	Homo sapiens fucosyltransferase 6 (alpha (1,3) fucosyltransferase) (FUT6), transcript variant 1, mRNA [NM_000150]	2528
A_23_P170534	2.2	1.7	1.0	1.2	FUT7	Homo sapiens fucosyltransferase 7 (alpha (1,3) fucosyltransferase) (FUT7), mRNA [NM_004479]	2529
A_23_P19556	2.6	1.4	2.3	2.2	FUT9	Homo sapiens fucosyltransferase 9 (alpha (1,3) fucosyltransferase) (FUT9), mRNA [NM_006581]	10690

A_24_P914362	-6.1	2.0	1.3	1.0	FUT9	fucosyltransferase 9 (alpha (1,3) fucosyltransferase) [Source:HGNC Symbol;Acc:4020] [ENST00000483933]	10690
A_24_P121891	2.0	3.6	-1.3	2.6	FUZ	Homo sapiens fuzzy homolog (Drosophila) (FUZ), transcript variant 1, mRNA [NM_025129]	80199
A_23_P4007	8.9	5.7	2.1	4.8	FXR2	Homo sapiens fragile X mental retardation, autosomal homolog 2 (FXR2), mRNA [NM_004860]	9513

A_23_P108216	1.4	-2.2	-1.3	-1.6	FXYD1	Homo sapiens FXYD domain containing ion transport regulator 1 (FXYD1), transcript variant a, mRNA [NM_005031]	5348
A_24_P196562	2.0	2.1	-1.2	-1.0	FXYD2	Homo sapiens FXYD domain containing ion transport regulator 2 (FXYD2), transcript variant b, mRNA [NM_021603]	486
A_23_P161769	-1.0	-2.7	-1.2	-1.1	FXYD2	Homo sapiens FXYD domain containing ion transport regulator 2 (FXYD2), transcript variant b, mRNA [NM_021603]	486

A_24_P293192	-1.0	3.6	4.3	3.2	FXYD3	Homo sapiens FXYD domain containing ion transport regulator 3 (FXYD3), transcript variant 1, mRNA [NM_005971]	5349
A_23_P98121	-1.6	-1.8	-1.9	-2.2	FXYD4	Homo sapiens FXYD domain containing ion transport regulator 4 (FXYD4), transcript variant 1, mRNA [NM_173160]	53828
A_24_P194081	-17.7	-2.0	-1.3	-3.4	FXYD5	Homo sapiens FXYD domain containing ion transport regulator 5 (FXYD5), transcript variant 1, mRNA [NM_144779]	53827

A_23_P130995	1.1	1.6	1.7	2.1	FXYD5	Homo sapiens FXYD domain containing ion transport regulator 5 (FXYD5), transcript variant 1, mRNA [NM_144779]	53827
A_23_P150394	2.4	1.2	-1.0	-1.4	FXYD6	Homo sapiens FXYD domain containing ion transport regulator 6 (FXYD6), transcript variant 1, mRNA [NM_022003]	53826
A_23_P122127	2.2	-1.0	2.2	-1.0	FYB	Homo sapiens FYN binding protein (FYB), transcript variant 1, mRNA [NM_001465]	2533

A_24_P393740	2.0	1.3	-1.2	-1.2	FYB	Homo sapiens FYN binding protein (FYB), transcript variant 1, mRNA [NM_001465]	2533
A_23_P80827	-3.1	1.4	1.2	-1.0	FYTTD1	Homo sapiens forty-two-three domain containing 1 (FYTTD1), transcript variant 2, mRNA [NM_001011537]	84248
A_24_P38276	-1.0	-1.0	-1.1	-33.4	FZD1	Homo sapiens frizzled family receptor 1 (FZD1), mRNA [NM_003505]	8321
A_23_P141362	1.0	1.1	1.0	2.0	FZD2	Homo sapiens frizzled family receptor 2 (FZD2), mRNA [NM_001466]	2535

A_23_P108437	-2.3	1.3	1.4	1.2	FZD5	Homo sapiens frizzled family receptor 5 (FZD5), mRNA [NM_003468]	7855
A_23_P142325	1.3	2.5	2.8	1.7	FZR1	Homo sapiens fizzy/cell division cycle 20 related 1 (Drosophila) (FZR1), transcript variant 1, mRNA [NM_001136198]	51343
A_23_P142333	2.2	1.4	-1.5	1.6	FZR1	Homo sapiens fizzy/cell division cycle 20 related 1 (Drosophila) (FZR1), transcript variant 2, mRNA [NM_016263]	51343
A_24_P941759	1.3	-2.3	-1.7	1.2	G2E3	Homo sapiens G2/M-phase specific E3 ubiquitin protein ligase (G2E3), mRNA [NM_017769]	55632

A_23_P67960	-1.4	-2.0	1.0	-2.4	G6PC2	Homo sapiens glucose-6-phosphatase, catalytic, 2 (G6PC2), transcript variant 1, mRNA [NM_021176]	57818
A_24_P98843	3.0	1.1	1.9	-1.8	GAB2	Homo sapiens GRB2-associated binding protein 2 (GAB2), transcript variant 2, mRNA [NM_012296]	9846
A_24_P4816	1.5	2.0	1.3	-1.1	GABARAPL1	Homo sapiens GABA(A) receptor-associated protein like 1 (GABARAPL1), mRNA [NM_031412]	23710

A_23_P65823	-1.6	1.2	-3.6	1.1	GABARAPL3	Homo sapiens GABA(A) receptors associated protein like 3, pseudogene (GABARAPL3), non-coding RNA [NR_028287]	23766
A_23_P93302	-1.3	-2.2	-2.4	-2.8	GABBR1	Homo sapiens gamma-aminobutyric acid (GABA) B receptor, 1 (GABBR1), transcript variant 1, mRNA [NM_001470]	2550
A_23_P112275	-3.9	1.2	1.1	-1.5	GABBR2	Homo sapiens gamma-aminobutyric acid (GABA) B receptor, 2 (GABBR2), mRNA [NM_005458]	9568

A_23_P205789	1.5	-3.1	1.0	-1.8	GABPB1	Homo sapiens GA binding protein transcription factor, beta subunit 1 (GABPB1), transcript variant gamma-1, mRNA [NM_002041]	2553
A_24_P176255	1.0	-1.0	-2.1	-1.7	GABPB1	Homo sapiens GA binding protein transcription factor, beta subunit 1 (GABPB1), transcript variant beta-1, mRNA [NM_005254]	2553
A_32_P177937	1.1	-7.2	-1.2	1.6	GABPB2	GA binding protein transcription factor, beta subunit 2 [Source:HGNC Symbol;Acc:28441] [ENST00000368918]	126626

A_23_P125067	-1.7	1.1	-2.1	1.6	GABRA1	Homo sapiens gamma-aminobutyric acid (GABA) A receptor, alpha 1 (GABRA1), transcript variant 1, mRNA [NM_000806]	2554
A_24_P226267	-1.6	-2.4	1.1	-1.4	GABRA1	Homo sapiens gamma-aminobutyric acid (GABA) A receptor, alpha 1 (GABRA1), transcript variant 1, mRNA [NM_000806]	2554
A_23_P212869	1.7	1.7	1.7	-2.7	GABRA2	Homo sapiens gamma-aminobutyric acid (GABA) A receptor, alpha 2 (GABRA2), transcript variant 1, mRNA [NM_000807]	2555

A_23_P436618	-3.5	10.7	10.6	5.9	GABRA5	Homo sapiens gamma-aminobutyric acid (GABA) A receptor, alpha 5 (GABRA5), transcript variant 1, mRNA [NM_000810]	2558
A_23_P19042	1.5	2.1	-1.5	-2.6	GABRB2	Homo sapiens gamma-aminobutyric acid (GABA) A receptor, beta 2 (GABRB2), transcript variant 2, mRNA [NM_000813]	2561
A_23_P10966	-5.0	-1.2	-2.1	1.0	GABRB3	Homo sapiens gamma-aminobutyric acid (GABA) A receptor, beta 3 (GABRB3), transcript variant 1, mRNA [NM_000814]	2562

A_32_P115840	-1.0	-6.6	-1.0	-1.8	GABRQ	gamma-aminobutyric acid (GABA) receptor, theta [Source:HGNC Symbol;Acc:14454] [ENST00000370306]	55879
A_23_P209578	-1.3	-1.8	-13.9	-1.2	GAD1	Homo sapiens glutamate decarboxylase 1 (brain, 67kDa) (GAD1), transcript variant GAD25, mRNA [NM_013445]	2571
A_23_P374689	2.3	1.8	1.4	1.3	GAD1	Homo sapiens glutamate decarboxylase 1 (brain, 67kDa) (GAD1), transcript variant GAD67, mRNA [NM_000817]	2571

A_23_P75008	-1.8	-1.5	-2.2	1.1	GAD2	Homo sapiens glutamate decarboxylase 2 (pancreatic islets and brain, 65kDa) (GAD2), transcript variant 1, mRNA [NM_000818]	2572
A_23_P23221	-2.0	-2.8	-2.5	-1.2	GADD45A	Homo sapiens growth arrest and DNA-damage-inducible, alpha (GADD45A), transcript variant 1, mRNA [NM_001924]	1647
A_23_P142506	1.8	-1.8	-2.1	-2.0	GADD45B	Homo sapiens growth arrest and DNA-damage-inducible, beta (GADD45B), mRNA [NM_015675]	4616

A_32_P39444	-1.3	2.1	-1.1	-1.1	GAFA1	Homo sapiens FGF2-associated protein GAFA1 (GAFA1) mRNA, complete cds. [AF190748]	100131227
A_24_P911025	-4.3	-1.8	-1.1	-1.0	GAFA2	Homo sapiens FGF-2 activity-associated protein 2 (GAFA2) mRNA, complete cds. [AF220234]	100130322
A_23_P384418	-15.8	-1.0	1.1	-2.1	GAGE3	Human GAGE-3 protein mRNA, complete cds. [U19144]	2575
A_24_P228796	-1.6	1.0	-2.4	1.1	GAGE7	Homo sapiens G antigen 7 (GAGE7), mRNA [NM_021123]	2579
A_24_P397150	-1.3	-1.0	-1.4	-31.6	GAK	Homo sapiens cyclin G associated kinase (GAK), mRNA [NM_005255]	2580

A_23_P326157	-2.0	1.2	-1.8	1.1	GAL3ST2	Homo sapiens galactose-3-O-sulfotransferase 2 (GAL3ST2), mRNA [NM_022134]	64090
A_24_P352608	-2.5	1.0	1.0	1.1	GALC	Homo sapiens galactosylceramidase (GALC), transcript variant 1, mRNA [NM_000153]	2581
A_23_P141126	1.9	2.5	-1.0	-1.2	GALK1	Homo sapiens galactokinase 1 (GALK1), mRNA [NM_000154]	2584
A_23_P48807	2.8	2.6	2.6	1.9	GALK2	Homo sapiens galactokinase 2 (GALK2), transcript variant 2, mRNA [NM_001001556]	2585

A_23_P306105	4.2	4.7	1.0	3.4	GALNT1	Homo sapiens UDP-N-acetyl-alpha-D-galactosamine:polypeptide N-acetylgalactosaminyltransferase 1 (GalNAc-T1) (GALNT1), mRNA [NM_020474]	2589
A_24_P910923	-6.2	1.3	-2.0	1.1	GALNT10	Homo sapiens UDP-N-acetyl-alpha-D-galactosamine:polypeptide N-acetylgalactosaminyltransferase 10 (GalNAc-T10) (GALNT10), mRNA [NM_198321]	55568

A_24_P161964	-1.2	1.2	2.3	1.5	GALNT10	Homo sapiens UDP-N-acetyl-alpha-D-galactosamine:polypeptide N-acetylgalactosaminyltransferase 10 (GalNAc-T10) (GALNT10), mRNA [NM_198321]	55568
A_23_P8412	-7.7	-1.0	-1.1	-1.1	GALNT11	Homo sapiens UDP-N-acetyl-alpha-D-galactosamine:polypeptide N-acetylgalactosaminyltransferase 11 (GalNAc-T11) (GALNT11), mRNA [NM_022087]	63917

A_32_P492524	1.0	1.0	-1.0	-16.1	GALNT13	Homo sapiens UDP-N-acetyl-alpha-D-galactosamine:polypeptide N-acetylgalactosaminyltransferase 13 (GalNAc-T13) (GALNT13), mRNA [NM_052917]	114805
A_24_P114249	-2.3	1.1	-1.1	-1.5	GALNT3	Homo sapiens UDP-N-acetyl-alpha-D-galactosamine:polypeptide N-acetylgalactosaminyltransferase 3 (GalNAc-T3) (GALNT3), mRNA [NM_004482]	2591

A_24_P942370	-2.5	1.0	-1.0	1.0	GALNT4	Homo sapiens UDP-N-acetyl-alpha-D-galactosamine:polypeptide N-acetylgalactosaminyltransferase 4 (GalNAc-T4) (GALNT4), mRNA [NM_003774]	8693
A_23_P144384	-3.6	-1.2	-1.9	-2.0	GALNT7	Homo sapiens UDP-N-acetyl-alpha-D-galactosamine:polypeptide N-acetylgalactosaminyltransferase 7 (GalNAc-T7) (GALNT7), mRNA [NM_017423]	51809

A_23_P76749	-2.7	-1.4	1.3	-1.3	GALNTL1	Homo sapiens UDP-N-acetyl-alpha-D-galactosamine:polypeptide N-acetylgalactosaminyltransferase-like 1 (GALNTL1), transcript variant 2, mRNA [NM_020692]	57452
A_23_P429601	1.0	-2.9	1.0	1.0	GALNTL5	Homo sapiens UDP-N-acetyl-alpha-D-galactosamine:polypeptide N-acetylgalactosaminyltransferase-like 5 (GALNTL5), transcript variant 1, mRNA [NM_145292]	168391

A_23_P39171	1.1	1.0	2.4	1.1	GALP	Homo sapiens galanin-like peptide (GALP), transcript variant 1, mRNA [NM_033106]	85569
A_23_P256663	1.7	1.3	1.1	2.1	GALR3	Homo sapiens galanin receptor 3 (GALR3), mRNA [NM_003614]	8484
A_23_P108143	-2.4	-1.8	1.8	-1.2	GAMT	Homo sapiens guanidinoacetate N-methyltransferase (GAMT), transcript variant 1, mRNA [NM_000156]	2593
A_24_P19228	-2.5	2.0	-3.3	-2.1	GAMT	Homo sapiens guanidinoacetate N-methyltransferase (GAMT), transcript variant 2, mRNA [NM_138924]	2593

A_23_P88873	2.9	3.0	2.8	3.1	GAN	Homo sapiens gigaxonin (GAN), mRNA [NM_022041]	8139
A_23_P399851	-3.4	-2.1	-1.2	-2.1	GANAB	Homo sapiens glucosidase, alpha; neutral AB (GANAB), transcript variant 3, mRNA [NM_198335]	23193
A_23_P67646	1.0	-1.0	-1.1	-2.9	GAPDHS	Homo sapiens glyceraldehyde-3-phosphate dehydrogenase, spermatogenic (GAPDHS), mRNA [NM_014364]	26330
A_23_P83320	1.1	1.1	1.0	-5.2	GARNL3	Homo sapiens GTPase activating Rap/RanGAP domain-like 3 (GARNL3), mRNA [NM_032293]	84253

A_24_P154948	2.0	2.0	2.0	-12.2	GARS	Homo sapiens glycyl-tRNA synthetase (GARS), mRNA [NM_002047]	2617
A_24_P179363	-1.3	-1.2	-1.4	-5.5	GAS2	Homo sapiens hypothetical LOC100137724, mRNA (cDNA clone IMAGE:3608551), partial cds. [BC005077]	2620
A_32_P24140	2.4	2.4	2.3	1.5	GAS2	Homo sapiens growth arrest-specific 2 (GAS2), transcript variant 1, mRNA [NM_005256]	2620
A_23_P502710	4.1	3.8	1.8	3.2	GAS2L1	Homo sapiens growth arrest-specific 2 like 1 (GAS2L1), transcript variant 3, mRNA [NM_152237]	10634

A_23_P396818	2.3	2.9	1.1	-2.3	GAS2L2	Homo sapiens growth arrest-specific 2 like 2 (GAS2L2), mRNA [NM_139285]	246176
A_32_P37143	-2.3	1.0	1.0	-1.0	GAS2L3	growth arrest-specific 2 like 3 [Source:HGNC Symbol;Acc:27475] [ENST00000552854]	283431
A_24_P82466	2.2	2.1	1.8	1.8	GAS7	Homo sapiens growth arrest-specific 7 (GAS7), transcript variant c, mRNA [NM_201433]	8522
A_23_P206435	-2.2	-1.6	-1.7	-1.6	GAS8	Homo sapiens growth arrest-specific 8 (GAS8), transcript variant 1, mRNA [NM_001481]	2622

A_23_P159191	-1.2	-1.7	-3.8	-2.6	GAST	Homo sapiens gastrin (GAST), mRNA [NM_000805]	2520
A_23_P75056	2.1	2.1	1.1	1.9	GATA3	Homo sapiens GATA binding protein 3 (GATA3), transcript variant 1, mRNA [NM_001002295]	2625
A_23_P384761	-2.5	1.4	-5.0	1.0	GATA4	Homo sapiens GATA binding protein 4 (GATA4), mRNA [NM_002052]	2626
A_24_P35169	1.2	1.2	1.1	-8.5	GATAD1	Homo sapiens GATA zinc finger domain containing 1 (GATAD1), mRNA [NM_021167]	57798

A_32_P227764	1.1	1.0	-2.8	-1.1	GATC	Homo sapiens glutamyl-tRNA(Gln) amidotransferase, subunit C homolog (bacterial) (GATC), transcript variant 1, mRNA [NM_176818]	283459
A_24_P402115	1.0	-10.8	-1.0	-1.4	GATC	Homo sapiens hypothetical protein LOC283459, mRNA (cDNA clone IMAGE:4822098). [BC034962]	283459
A_32_P141374	2.0	2.6	2.3	1.3	GATS	Homo sapiens GATS, stromal antigen 3 opposite strand (GATS), transcript variant 1, mRNA [NM_178831]	352954

A_24_P942538	3.3	-1.5	2.5	2.7	GATS	Homo sapiens GATS, stromal antigen 3 opposite strand (GATS), transcript variant 2, non-coding RNA [NR_028038]	352954
A_23_P327777	1.2	-1.2	-2.5	-1.6	GATS	Homo sapiens GATS, stromal antigen 3 opposite strand (GATS), transcript variant 1, mRNA [NM_178831]	352954
A_32_P464568	1.1	1.8	2.0	-1.0	GBA2	Homo sapiens glucosidase, beta (bile acid) 2 (GBA2), mRNA [NM_020944]	57704

A_32_P512061	-2.3	1.3	1.2	-1.8	GBAP1	Homo sapiens glucosidase, beta, acid pseudogene 1 (GBAP1), non-coding RNA [NR_002188]	2630
A_23_P161237	1.0	1.5	1.0	2.4	GBF1	Homo sapiens golgi brefeldin A resistant guanine nucleotide exchange factor 1 (GBF1), transcript variant 1, mRNA [NM_004193]	8729
A_23_P146576	-5.2	-1.3	-1.7	-1.6	GBGT1	Homo sapiens globoside alpha-1,3-N-acetylgalactosaminyltransferase 1 (GBGT1), mRNA [NM_021996]	26301

A_32_P107372	-12.9	-1.2	-1.4	-1.8	GBP1	Homo sapiens guanylate binding protein 1, interferon-inducible (GBP1), mRNA [NM_002053]	2633
A_24_P36898	11.9	11.7	16.3	9.1	GBP2	guanylate binding protein 2, interferon-inducible [Source:HGNC Symbol;Acc:4183] [ENST00000464839]	2634
A_23_P51487	-1.1	1.1	-2.3	-1.1	GBP3	Homo sapiens guanylate binding protein 3 (GBP3), mRNA [NM_018284]	2635
A_24_P370702	-1.7	-1.0	-2.0	-1.6	GBP3	Homo sapiens guanylate binding protein 3 (GBP3), mRNA [NM_018284]	2635

A_24_P45446	5.3	8.5	2.2	1.0	GBP4	Homo sapiens guanylate binding protein 4 (GBP4), mRNA [NM_052941]	115361
A_23_P103496	-1.6	1.0	-5.6	-2.0	GBP4	Homo sapiens guanylate binding protein 4 (GBP4), mRNA [NM_052941]	115361
A_23_P131183	1.8	1.8	-1.1	2.8	GBX2	Homo sapiens gastrulation brain homeobox 2 (GBX2), mRNA [NM_001485]	2637
A_23_P71094	1.0	-2.0	-1.1	-1.1	GCC1	Homo sapiens GRIP and coiled-coil domain containing 1 (GCC1), mRNA [NM_024523]	79571
A_23_P71091	4.1	1.2	2.9	1.6	GCC1	Homo sapiens GRIP and coiled-coil domain containing 1 (GCC1), mRNA [NM_024523]	79571

A_24_P85942	-4.7	-1.0	-1.1	-1.6	GCC2	Homo sapiens GRIP and coiled-coil domain containing 2 (GCC2), transcript variant 1, mRNA [NM_181453]	9648
A_23_P253250	-2.0	1.3	2.0	2.6	GCET2	Homo sapiens germinal center expressed transcript 2 (GCET2), transcript variant 3, mRNA [NM_001190259]	257144
A_24_P182947	-3.4	-2.4	-1.1	-1.0	GCET2	Homo sapiens germinal center expressed transcript 2 (GCET2), transcript variant 3, mRNA [NM_001190259]	257144

A_23_P211064	-1.6	-1.4	1.1	-2.1	GCFC1	Homo sapiens GC-rich sequence DNA-binding factor 1 (GCFC1), transcript variant 2, mRNA [NM_013329]	94104
A_24_P279938	1.9	1.5	-3.9	2.0	GCFC1	Homo sapiens GC-rich sequence DNA-binding factor 1 (GCFC1), transcript variant 2, mRNA [NM_013329]	94104
A_23_P15751	-1.6	-1.1	-2.8	-1.1	GCGR	Homo sapiens glucagon receptor (GCGR), mRNA [NM_000160]	2642
A_24_P167642	-1.0	-2.6	-3.3	-2.5	GCH1	Homo sapiens GTP cyclohydrolase 1 (GCH1), transcript variant 1, mRNA [NM_000161]	2643

A_23_P250206	-1.4	-2.6	-1.4	-1.4	GCK	Homo sapiens glucokinase (hexokinase 4) (GCK), transcript variant 3, mRNA [NM_033508]	2645
A_23_P119886	2.7	2.8	-1.3	1.8	GCKR	Homo sapiens glucokinase (hexokinase 4) regulator (GCKR), mRNA [NM_001486]	2646
A_32_P98732	-1.7	1.5	-2.4	-1.5	GCM1	Homo sapiens glial cells missing homolog 1 (Drosophila) (GCM1), mRNA [NM_003643]	8521
A_23_P133728	1.4	1.0	2.5	2.0	GCM1	Homo sapiens glial cells missing homolog 1 (Drosophila) (GCM1), mRNA [NM_003643]	8521

A_23_P117933	-4.7	1.0	-1.5	-1.1	GCSH	Homo sapiens glycine cleavage system protein H (aminomethyl carrier) (GCSH), nuclear gene encoding mitochondrial protein, transcript variant 1, mRNA [NM_004483]	2653
A_23_P383258	1.0	1.1	-2.3	-1.2	GDA	Homo sapiens guanine deaminase (GDA), transcript variant 2, mRNA [NM_004293]	9615
A_24_P233878	-1.4	-1.5	-1.1	2.5	GDAP2	Homo sapiens ganglioside induced differentiation associated protein 2 (GDAP2), transcript variant 1, mRNA [NM_017686]	54834

A_23_P209098	3.4	2.7	4.4	-1.0	GDF1	Homo sapiens growth differentiation factor 1 (GDF1), mRNA [NM_001492]	2657
A_23_P46755	1.1	2.3	1.0	-1.0	GDF2	Homo sapiens growth differentiation factor 2 (GDF2), mRNA [NM_016204]	2658
A_23_P259955	2.0	1.2	1.1	1.7	GDF5	Homo sapiens growth differentiation factor 5 (GDF5), mRNA [NM_000557]	8200
A_32_P140489	1.7	-3.1	1.0	-1.3	GDF6	Homo sapiens growth differentiation factor 6 (GDF6), mRNA [NM_001001557]	392255

A_24_P376451	2.1	2.5	-1.2	-1.2	GDNF	Homo sapiens glial cell derived neurotrophic factor (GDNF), transcript variant 4, mRNA [NM_001190469]	2668
A_24_P920730	1.8	-2.0	-3.1	1.0	GDPD1	Homo sapiens glycerophosphodiester phosphodiesterase domain containing 1 (GDPD1), transcript variant 1, mRNA [NM_182569]	284161
A_23_P217498	2.0	2.0	1.7	1.4	GDPD2	Homo sapiens glycerophosphodiester phosphodiesterase domain containing 2 (GDPD2), transcript variant 2, mRNA [NM_017711]	54857

A_23_P257043	1.2	-2.1	-1.0	-1.0	GEM	Homo sapiens GTP binding protein overexpressed in skeletal muscle (GEM), transcript variant 1, mRNA [NM_005261]	2669
A_23_P336513	1.1	1.1	-1.2	-2.5	GEMIN5	Homo sapiens gem (nuclear organelle) associated protein 5 (GEMIN5), transcript variant 1, mRNA [NM_015465]	25929
A_23_P56567	1.6	1.6	2.1	1.0	GEMIN6	Homo sapiens gem (nuclear organelle) associated protein 6 (GEMIN6), mRNA [NM_024775]	79833

A_23_P89910	2.3	-1.5	1.7	-1.2	GEMIN7	Homo sapiens gem (nuclear organelle) associated protein 7 (GEMIN7), transcript variant 1, mRNA [NM_024707]	79760
A_32_P197620	-5.8	1.3	1.7	-3.0	GEMIN8P4	Homo sapiens gem (nuclear organelle) associated protein 8 pseudogene 4 (GEMIN8P4), non-coding RNA [NR_002830]	492303
A_23_P66593	1.0	-2.0	-1.0	-1.0	GFAP	Homo sapiens glial fibrillary acidic protein (GFAP), transcript variant 1, mRNA [NM_002055]	2670

A_24_P59786	1.0	-1.0	-1.1	-7.1	GFAP	Homo sapiens glial fibrillary acidic protein (GFAP), transcript variant 3, mRNA [NM_001242376]	2670
A_24_P31003	-3.3	1.0	-1.0	1.1	GFER	Homo sapiens growth factor, augmenter of liver regeneration (GFER), mRNA [NM_005262]	2671
A_23_P343303	2.6	-1.0	1.0	1.1	GFI1B	Homo sapiens growth factor independent 1B transcription repressor (GFI1B), transcript variant 1, mRNA [NM_004188]	8328

A_23_P216845	1.6	1.3	2.1	1.2	GFI1B	Homo sapiens growth factor independent 1B transcription repressor (GFI1B), transcript variant 1, mRNA [NM_004188]	8328
A_24_P76489	1.0	-2.4	-1.0	-1.0	GFM1	G elongation factor, mitochondrial 1 [Source:HGNC Symbol;Acc:13780] [ENST00000312756]	85476
A_24_P137582	-1.4	1.2	-1.5	-4.1	GFM2	Homo sapiens G elongation factor, mitochondrial 2 (GFM2), nuclear gene encoding mitochondrial protein, transcript variant 3, mRNA [NM_170681]	84340

A_23_P122662	1.1	-2.5	-2.4	-2.4	GFOD1	Homo sapiens glucose-fructose oxidoreductase domain containing 1 (GFOD1), transcript variant 1, mRNA [NM_018988]	54438
A_23_P12686	-26.3	-1.1	-1.2	-1.2	GFRA1	Homo sapiens GDNF family receptor alpha 1 (GFRA1), transcript variant 2, mRNA [NM_145793]	2674
A_32_P59549	-2.5	-1.0	-1.1	-1.0	GFRA1	Homo sapiens GDNF family receptor alpha 1 (GFRA1), transcript variant 2, mRNA [NM_145793]	2674

A_32_P162507	76.8	40.7	66.0	55.1	GFRA2	Homo sapiens GDNF family receptor alpha 2 (GFRA2), transcript variant 1, mRNA [NM_001495]	2675
A_23_P41987	1.1	-4.3	1.4	2.0	GFRA3	Homo sapiens GDNF family receptor alpha 3 (GFRA3), mRNA [NM_001496]	2676
A_23_P41992	1.6	1.6	-1.8	-2.5	GFRA3	Homo sapiens GDNF family receptor alpha 3 (GFRA3), mRNA [NM_001496]	2676
A_24_P416257	-19.6	1.6	2.6	-11.4	GGA2	Homo sapiens golgi-associated, gamma adaptin ear containing, ARF binding protein 2 (GGA2), mRNA [NM_015044]	23062

A_23_P38181	-1.2	-1.2	-2.7	-2.1	GGA3	Homo sapiens golgi-associated, gamma adaptin ear containing, ARF binding protein 3 (GGA3), transcript variant long, mRNA [NM_138619]	23163
A_32_P221337	-1.6	3.7	-1.5	-1.9	GGCX	Homo sapiens gamma-glutamyl carboxylase (GGCX), transcript variant 1, mRNA [NM_000821]	2677
A_24_P933180	1.1	-4.9	-1.1	1.0	GGCX	Homo sapiens gamma-glutamyl carboxylase (GGCX), transcript variant 1, mRNA [NM_000821]	2677

A_32_P181103	1.3	1.3	-2.6	1.3	GGCX	Homo sapiens gamma-glutamyl carboxylase (GGCX), transcript variant 1, mRNA [NM_000821]	2677
A_23_P333552	-7.1	-4.6	-3.9	-4.3	GGN	Homo sapiens gametogenetin (GGN), mRNA [NM_152657]	199720
A_24_P67892	-1.5	-1.4	-1.7	-2.2	GGNBP2	Homo sapiens gametogenetin binding protein 2 (GGNBP2), mRNA [NM_024835]	79893
A_24_P67976	1.2	1.2	2.0	-2.4	GGNBP2	Homo sapiens gametogenetin binding protein 2 (GGNBP2), mRNA [NM_024835]	79893

A_23_P120809	4.5	5.4	4.3	6.7	GGT1	Homo sapiens gamma-glutamyltransferase 1 (GGT1), transcript variant 1, mRNA [NM_005265]	2678
A_23_P154986	1.5	1.4	-3.7	-1.0	GGT1	Homo sapiens gamma-glutamyltransferase 1 (GGT1), transcript variant 1, mRNA [NM_005265]	2678
A_24_P178175	6.0	1.5	1.0	-1.5	GGT1	Homo sapiens gamma-glutamyltransferase 1 (GGT1), transcript variant 1, mRNA [NM_005265]	2678
A_24_P42066	4.3	1.9	2.4	1.9	GGT3P	Homo sapiens gamma-glutamyltransferase 3 pseudogene (GGT3P), non-coding RNA [NR_003267]	2679

A_23_P66767	1.0	2.1	-1.1	-1.0	GGT6	Homo sapiens gamma-glutamyltransferase 6 (GGT6), transcript variant 2, mRNA [NM_153338]	124975
A_23_P66774	3.4	6.4	2.1	4.4	GGT6	Homo sapiens gamma-glutamyltransferase 6 (GGT6), transcript variant 2, mRNA [NM_153338]	124975
A_23_P325093	9.6	4.2	4.3	2.2	GGT7	Homo sapiens gamma-glutamyltransferase 7 (GGT7), mRNA [NM_178026]	2686
A_23_P207194	-2.2	2.9	2.5	-1.4	GH1	Homo sapiens growth hormone 1 (GH1), transcript variant 1, mRNA [NM_000515]	2688

A_24_P216014	-1.1	-2.9	-1.2	-1.0	GH2	Homo sapiens growth hormone 2 (GH2), transcript variant 3, mRNA [NM_022558]	2689
A_23_P207160	1.3	2.8	1.1	-1.0	GH2	Homo sapiens growth hormone 2 (GH2), transcript variant 3, mRNA [NM_022558]	2689
A_23_P207174	1.1	1.2	-8.6	-1.6	GH2	Homo sapiens growth hormone 2 (GH2), transcript variant 2, mRNA [NM_022557]	2689
A_24_P258800	-12.2	-1.9	-1.8	-1.5	GHDC	Homo sapiens GH3 domain containing (GHDC), transcript variant 1, mRNA [NM_032484]	84514

A_23_P104387	2.9	-1.5	-1.1	1.2	GHITM	Homo sapiens growth hormone inducible transmembrane protein (GHITM), mRNA [NM_014394]	27069
A_24_P72064	-1.3	-1.2	-1.4	-2.1	GHR	Homo sapiens growth hormone receptor (GHR), transcript variant 1, mRNA [NM_000163]	2690
A_23_P156087	-2.1	-1.1	-1.1	-1.1	GHR	Homo sapiens growth hormone receptor (GHR), transcript variant 1, mRNA [NM_000163]	2690
A_23_P8497	3.6	3.5	1.0	2.2	GHRHR	Homo sapiens growth hormone releasing hormone receptor (GHRHR), mRNA [NM_000823]	2692

A_24_P208737	1.8	1.0	-2.7	-18.3	GHRLOS	Homo sapiens ghrelin opposite strand RNA (non-protein coding) (GHRLOS), transcript variant 1, non-coding RNA [NR_004431]	100126793
A_23_P109864	2.1	2.3	2.6	-4.1	GHSR	Homo sapiens growth hormone secretagogue receptor (GHSR), transcript variant 1b, mRNA [NM_004122]	2693
A_24_P163574	1.8	2.6	-1.0	2.3	GIGYF1	Homo sapiens GRB10 interacting GYF protein 1 (GIGYF1), mRNA [NM_022574]	64599

A_23_P142950	-1.7	1.5	3.5	3.4	GIGYF2	Homo sapiens GRB10 interacting GYF protein 2 (GIGYF2), transcript variant 2, mRNA [NM_015575]	26058
A_24_P307896	1.0	1.0	1.0	2.0	GIGYF2	Homo sapiens GRB10 interacting GYF protein 2, mRNA (cDNA clone IMAGE:4472100), with apparent retained intron. [BC012484]	26058
A_24_P271985	1.1	1.0	-4.5	-2.3	GIGYF2	Homo sapiens GRB10 interacting GYF protein 2 (GIGYF2), transcript variant 2, mRNA [NM_015575]	26058

A_24_P926755	2.4	3.5	-1.5	-1.2	GIGYF2	GRB10 interacting GYF protein 2 [Source:HGNC Symbol;Acc:11960] [ENST00000464402]	26058
A_23_P31188	1.0	-1.0	-1.1	-4.8	GIMAP1	Homo sapiens GTPase, IMAP family member 1 (GIMAP1), mRNA [NM_130759]	170575
A_23_P368681	1.0	1.0	1.0	-8.8	GIMAP2	Homo sapiens GTPase, IMAP family member 2 (GIMAP2), mRNA [NM_015660]	26157
A_23_P42588	1.0	-2.4	-1.1	-1.1	GIMAP5	Homo sapiens GTPase, IMAP family member 5 (GIMAP5), mRNA [NM_018384]	55340

A_24_P274831	1.1	1.0	1.1	-2.4	GIMAP7	Homo sapiens GTPase, IMAP family member 7 (GIMAP7), mRNA [NM_153236]	168537
A_23_P168388	-8.1	-9.4	-3.6	-2.9	GIMAP8	Homo sapiens GTPase, IMAP family member 8 (GIMAP8), mRNA [NM_175571]	155038
A_24_P132383	1.4	-3.1	-1.1	-1.0	GIMAP8	Homo sapiens GTPase, IMAP family member 8 (GIMAP8), mRNA [NM_175571]	155038
A_23_P130648	-1.5	-2.2	-1.0	-2.5	GIPC1	Homo sapiens GIPC PDZ domain containing family, member 1 (GIPC1), transcript variant 1, mRNA [NM_005716]	10755

A_24_P100301	-1.0	1.1	-1.1	-2.0	GIPC2	Homo sapiens GIPC PDZ domain containing family, member 2 (GIPC2), mRNA [NM_017655]	54810
A_24_P118527	1.0	-1.0	2.2	1.2	GIPC3	Homo sapiens GIPC PDZ domain containing family, member 3 (GIPC3), mRNA [NM_133261]	126326
A_24_P373768	1.1	-2.3	-2.2	1.2	GIPR	Homo sapiens gastric inhibitory polypeptide receptor (GIPR), mRNA [NM_000164]	2696
A_23_P119395	3.4	6.2	8.7	5.4	GIPR	Homo sapiens gastric inhibitory polypeptide receptor (GIPR), mRNA [NM_000164]	2696

A_24_P347946	-1.4	-2.9	-2.8	-2.4	GIT1	Homo sapiens G protein-coupled receptor kinase interacting ArfGAP 1 (GIT1), transcript variant 2, mRNA [NM_014030]	28964
A_23_P100912	-2.0	-2.7	-2.6	-1.1	GIT1	Homo sapiens G protein-coupled receptor kinase interacting ArfGAP 1 (GIT1), transcript variant 2, mRNA [NM_014030]	28964
A_32_P205241	1.0	1.0	-1.6	-2.2	GJA3	Homo sapiens gap junction protein, alpha 3, 46kDa (GJA3), mRNA [NM_021954]	2700

A_23_P371729	-1.0	-1.0	-1.1	-2.6	GJA5	Homo sapiens gap junction protein, alpha 5, 40kDa (GJA5), transcript variant A, mRNA [NM_005266]	2702
A_24_P404840	1.1	-1.1	1.3	-2.5	GJB1	Homo sapiens gap junction protein, beta 1, 32kDa (GJB1), transcript variant 2, mRNA [NM_000166]	2705
A_23_P250444	-1.2	-19.7	-1.2	1.6	GJB1	Homo sapiens gap junction protein, beta 1, 32kDa (GJB1), transcript variant 2, mRNA [NM_000166]	2705

A_23_P51729	1.9	1.9	-2.2	1.2	GJC2	Homo sapiens gap junction protein, gamma 2, 47kDa (GJC2), mRNA [NM_020435]	57165
A_23_P144512	-1.6	-2.2	-1.1	-1.1	GK2	Homo sapiens glycerol kinase 2 (GK2), mRNA [NM_033214]	2712
A_23_P218579	-1.0	-1.8	-1.1	-3.4	GLB1L	Homo sapiens galactosidase, beta 1-like (GLB1L), mRNA [NM_024506]	79411
A_23_P336198	1.1	1.1	-3.7	1.1	GLCCI1	Homo sapiens glucocorticoid induced transcript 1 (GLCCI1), mRNA [NM_138426]	113263
A_24_P698125	7.5	6.3	5.7	5.8	GLDN	gliomedin [Source:HGNC Symbol;Acc:29514] [ENST00000464150]	342035

A_24_P49199	1.0	-3.9	-1.1	-1.1	GLDN	Homo sapiens gliomedin (GLDN), mRNA [NM_181789]	342035
A_24_P475556	-4.3	-1.5	-1.6	-2.0	GLG1	Homo sapiens golgi glycoprotein 1 (GLG1), transcript variant 3, mRNA [NM_001145667]	2734
A_23_P140916	-10.1	-1.4	1.1	-1.1	GLG1	Homo sapiens golgi glycoprotein 1 (GLG1), transcript variant 1, mRNA [NM_012201]	2734
A_23_P364024	-1.0	-12.2	-1.0	-1.2	GLIPR1	Homo sapiens GLI pathogenesis-related 1 (GLIPR1), mRNA [NM_006851]	11010

A_23_P414913	1.6	2.2	1.5	1.0	GLIPR2	Homo sapiens GLI pathogenesis-related 2 (GLIPR2), mRNA [NM_022343]	152007
A_23_P323154	4.5	7.8	-7.9	2.5	GLIS2	Homo sapiens GLIS family zinc finger 2 (GLIS2), mRNA [NM_032575]	84662
A_23_P350249	-1.9	4.4	3.6	5.2	GLIS3	Homo sapiens GLIS family zinc finger 3 (GLIS3), transcript variant 1, mRNA [NM_001042413]	169792
A_32_P39394	-9.3	1.7	5.9	1.0	GLIS3	Homo sapiens GLIS family zinc finger 3 (GLIS3), transcript variant 1, mRNA [NM_001042413]	169792

A_23_P312837	-2.9	1.0	1.4	1.1	GLIS3-AS1	Homo sapiens GLIS3 antisense RNA 1 (non-protein coding) (GLIS3-AS1), non-coding RNA [NR_026663]	84850
A_23_P160742	1.5	-1.6	-3.2	1.4	GLMN	Homo sapiens glomulin, FKBP associated protein (GLMN), mRNA [NM_053274]	11146
A_23_P214411	-63.6	-1.1	-1.2	-1.2	GLO1	Homo sapiens glyoxalase I (GLO1), mRNA [NM_006708]	2739
A_23_P78268	1.1	-3.3	1.0	1.0	GLOD4	Homo sapiens glyoxalase domain containing 4 (GLOD4), mRNA [NM_016080]	51031

A_32_P78681	1.0	-6.1	1.4	1.5	GLP2R	glucagon-like peptide 2 receptor [Source:HGNC Symbol;Acc:4325] [ENST00000262441]	9340
A_23_P156261	1.6	-1.1	-8.1	-1.2	GLRA1	Homo sapiens glycine receptor, alpha 1 (GLRA1), transcript variant 2, mRNA [NM_000171]	2741
A_23_P411903	-1.3	-1.2	-2.4	-1.5	GLRA2	Homo sapiens glycine receptor, alpha 2 (GLRA2), transcript variant 1, mRNA [NM_002063]	2742
A_32_P90113	1.2	1.2	1.1	-2.1	GLRA3	glycine receptor, alpha 3 [Source:HGNC Symbol;Acc:4328] [ENST00000274093]	8001

A_24_P381029	1.0	-1.3	-2.9	1.4	GLRX3	Homo sapiens glutaredoxin 3 (GLRX3), transcript variant 2, mRNA [NM_006541]	10539
A_23_P65370	-4.3	-10.7	1.3	1.0	GLRX5	Homo sapiens glutaredoxin 5 (GLRX5), nuclear gene encoding mitochondrial protein, mRNA [NM_016417]	51218
A_23_P308800	2.2	-2.0	1.0	3.3	GLS	glutaminase [Source:HGNC Symbol;Acc:4331] [ENST00000338435]	2744

A_24_P326739	2.4	2.0	2.0	1.7	GLS2	Homo sapiens glutaminase 2 (liver, mitochondrial) (GLS2), nuclear gene encoding mitochondrial protein, mRNA [NM_013267]	27165
A_32_P19294	1.0	-1.2	-1.7	2.3	GLT1D1	Homo sapiens glycosyltransferase 1 domain containing 1 (GLT1D1), mRNA [NM_144669]	144423
A_23_P48198	-1.0	-2.4	-1.0	-1.0	GLT8D2	Homo sapiens glycosyltransferase 8 domain containing 2 (GLT8D2), mRNA [NM_031302]	83468

A_24_P234105	1.1	4.2	1.1	-1.3	GLTPD1	Homo sapiens glycolipid transfer protein domain containing 1 (GLTPD1), mRNA [NM_001029885]	80772
A_23_P63281	2.4	2.4	3.1	1.5	GLTPD1	Homo sapiens glycolipid transfer protein domain containing 1 (GLTPD1), mRNA [NM_001029885]	80772
A_23_P39131	1.0	-2.5	-1.0	-1.0	GLTSCR2	Homo sapiens glioma tumor suppressor candidate region gene 2 (GLTSCR2), mRNA [NM_015710]	29997

A_23_P138665	1.1	1.1	-4.8	1.2	GLUD1	Homo sapiens glutamate dehydrogenase 1 (GLUD1), nuclear gene encoding mitochondrial protein, mRNA [NM_005271]	2746
A_23_P403886	-4.1	1.1	-1.7	-1.0	GLYAT	Homo sapiens glycine-N-acyltransferase (GLYAT), nuclear gene encoding mitochondrial protein, transcript variant 2, mRNA [NM_005838]	10249

A_23_P75749	3.6	3.5	4.9	2.6	GLYAT	Homo sapiens glycine-N-acyltransferase (GLYAT), nuclear gene encoding mitochondrial protein, transcript variant 1, mRNA [NM_201648]	10249
A_23_P47484	1.5	-1.3	3.9	1.5	GLYATL2	Homo sapiens glycine-N-acyltransferase-like 2 (GLYATL2), mRNA [NM_145016]	219970
A_24_P385092	2.4	1.4	-1.5	1.0	GLYCAM1	Homo sapiens glycosylation dependent cell adhesion molecule 1 (pseudogene) (GLYCAM1), non-coding RNA [NR_003039]	644076

A_23_P29621	1.0	-2.6	-4.0	1.0	GLYCTK	Homo sapiens glycerate kinase (GLYCTK), transcript variant 1, mRNA [NM_145262]	132158
A_23_P218302	5.1	4.7	4.8	6.1	GLYR1	Homo sapiens glyoxylate reductase 1 homolog (Arabidopsis) (GLYR1), mRNA [NM_032569]	84656
A_23_P144866	-3.3	-1.4	2.3	1.4	GM2A	Homo sapiens GM2 ganglioside activator (GM2A), transcript variant 1, mRNA [NM_000405]	2760
A_24_P925314	-2.5	1.0	1.3	-4.9	GM2A	Homo sapiens GM2 ganglioside activator (GM2A), transcript variant 1, mRNA [NM_000405]	2760

A_23_P144872	2.2	1.2	1.1	1.1	GM2A	Homo sapiens GM2 gangliosid e activator (GM2A), transcript variant 1, mRNA [NM_0004 05]	2760
A_23_P28375	7.6	6.7	7.0	6.9	GMCL1	Homo sapiens germ cell-less homolog 1 (Drosophil a) (GMCL1), mRNA [NM_1784 39]	64395
A_32_P84388	-1.2	-2.0	-1.5	1.1	GMCL1	Homo sapiens germ cell-less homolog 1 (Drosophil a) (GMCL1), mRNA [NM_1784 39]	64395

A_24_P621023	1.1	1.1	1.0	-23.3	GMEB1	glucocorticoid modulatory element binding protein 1 [Source:HGNC Symbol;Acc:4370] [ENST00000373816]	10691
A_23_P337033	-3.5	-1.8	-2.0	-2.7	GMEB2	Homo sapiens glucocorticoid modulatory element binding protein 2 (GMEB2), mRNA [NM_012384]	26205
A_24_P172768	-1.4	-2.6	-1.1	-3.0	GMFB	Homo sapiens glia maturation factor, beta (GMFB), mRNA [NM_004124]	2764

A_23_P67913	-2.1	-1.1	-1.5	1.5	GMPPA	Homo sapiens GDP-mannose pyrophosphorylase A (GMPPA), transcript variant 1, mRNA [NM_013335]	29926
A_24_P277657	3.2	2.8	1.5	1.6	GMPR	Homo sapiens guanosine monophosphate reductase (GMPR), mRNA [NM_006877]	2766
A_24_P56467	1.1	1.0	2.7	-3.0	GMPR2	Homo sapiens guanosine monophosphate reductase 2 (GMPR2), transcript variant 2, mRNA [NM_001002000]	51292
A_23_P21033	-5.3	1.2	-1.7	-2.8	GMPS	Homo sapiens guanine monphosphate synthetase (GMPS), mRNA [NM_003875]	8833

A_24_P927886	2.2	2.5	2.1	1.7	GNA11	Homo sapiens GTP-binding protein alpha 11 (GA11) mRNA, partial cds. [L40630]	2767
A_23_P354270	1.1	-7.1	-1.1	1.1	GNA12	Homo sapiens guanine nucleotide binding protein (G protein) alpha 12 (GNA12), mRNA [NM_007353]	2768
A_24_P98086	-1.0	-1.0	2.5	-1.0	GNA12	Homo sapiens guanine nucleotide binding protein (G protein) alpha 12 (GNA12), mRNA [NM_007353]	2768
A_24_P150874	1.4	-1.2	-2.4	1.1	GNA13	Homo sapiens guanine nucleotide binding protein (G protein), alpha 13 (GNA13), mRNA [NM_006572]	10672

A_24_P331128	2.0	1.7	3.8	-1.1	GNA15	Homo sapiens guanine nucleotide binding protein (G protein), alpha 15 (Gq class) (GNA15), mRNA [NM_002068]	2769
A_23_P122976	1.1	1.0	-1.1	-2.9	GNAI1	Homo sapiens guanine nucleotide binding protein (G protein), alpha inhibiting activity polypeptide 1 (GNAI1), mRNA [NM_002069]	2770
A_23_P144113	1.5	-2.2	1.2	-3.3	GNAI2	Homo sapiens guanine nucleotide binding protein (G protein), alpha inhibiting activity polypeptide 2 (GNAI2), transcript variant 1, mRNA [NM_002070]	2771

A_24_P339436	-4.2	-1.4	1.0	1.0	GNAL	Homo sapiens guanine nucleotide binding protein (G protein), alpha activating activity polypeptide, olfactory type (GNAL), transcript variant 2, mRNA [NM_002071]	2774
A_23_P38689	24.6	24.2	12.2	16.9	GNAL	Homo sapiens guanine nucleotide binding protein (G protein), alpha activating activity polypeptide, olfactory type (GNAL), transcript variant 2, mRNA [NM_002071]	2774

A_24_P393611	3.0	1.8	1.7	1.4	GNAO1	Homo sapiens guanine nucleotide binding protein (G protein), alpha activating activity polypeptide O (GNAO1), transcript variant 2, mRNA [NM_138736]	2775
A_24_P936145	1.1	-1.4	-1.0	-28.4	GNAO1	Homo sapiens guanine nucleotide binding protein (G protein), alpha activating activity polypeptide O (GNAO1), transcript variant 2, mRNA [NM_138736]	2775
A_24_P316413	4.1	5.8	4.4	-1.2	GNAO1	Homo sapiens cDNA clone IMAGE:4181241. [BC012202]	2775

A_23_P26582	-1.0	-2.4	-1.1	-1.1	GNAO1	Homo sapiens guanine nucleotide binding protein (G protein), alpha activating activity polypeptide O (GNAO1), transcript variant 1, mRNA [NM_020988]	2775
A_24_P168574	1.4	-1.2	1.2	-2.1	GNAS	GNAS complex locus [Source:HGNC Symbol;Acc:4392] [ENST00000481768]	2778
A_24_P418809	1.2	2.1	1.7	1.7	GNAS	Homo sapiens GNAS complex locus (GNAS), transcript variant 7, mRNA [NM_001077489]	2778

A_24_P168581	2.1	1.3	1.9	2.5	GNAS	Homo sapiens GNAS complex locus (GNAS), transcript variant 2, mRNA [NM_001077490]	2778
A_24_P320036	-1.1	-1.1	-1.4	-3.3	GNAT1	Homo sapiens guanine nucleotide binding protein (G protein), alpha transducing activity polypeptide 1 (GNAT1), transcript variant 2, mRNA [NM_000172]	2779
A_23_P218751	1.6	-1.6	2.1	1.7	GNB1L	Homo sapiens guanine nucleotide binding protein (G protein), beta polypeptide 1-like (GNB1L), mRNA [NM_053004]	54584

A_23_P76350	2.2	1.7	1.7	2.0	GNB3	Homo sapiens guanine nucleotide binding protein (G protein), beta polypeptide 3 (GNB3), mRNA [NM_002075]	2784
A_23_P216489	-1.0	-1.0	-2.2	-1.0	GNE	Homo sapiens glucosamine (UDP-N-acetyl)-2-epimerase/N-acetylmannosamine kinase (GNE), transcript variant 2, mRNA [NM_005476]	10020
A_23_P112260	3.3	3.3	3.4	2.2	GNG10	Homo sapiens guanine nucleotide binding protein (G protein), gamma 10 (GNG10), transcript variant 1, mRNA [NM_001017998]	2790

A_32_P208403	1.4	4.5	1.7	3.4	GNG2	Homo sapiens guanine nucleotide binding protein (G protein), gamma 2 (GNG2), transcript variant 1, mRNA [NM_053064]	54331
A_23_P32536	1.1	3.9	1.1	3.1	GNG2	Homo sapiens guanine nucleotide binding protein (G protein), gamma 2 (GNG2), transcript variant 1, mRNA [NM_053064]	54331
A_23_P335329	1.2	1.4	1.3	-2.2	GNG4	Homo sapiens guanine nucleotide binding protein (G protein), gamma 4 (GNG4), transcript variant 3, mRNA [NM_004485]	2786

A_23_P153897	3.0	3.8	3.1	-3.2	GNG7	Homo sapiens guanine nucleotide binding protein (G protein), gamma 7 (GNG7), mRNA [NM_052847]	2788
A_24_P79070	1.2	-46.2	1.5	-1.6	GNG8	Homo sapiens guanine nucleotide binding protein (G protein), gamma 8 (GNG8), mRNA [NM_033258]	94235
A_24_P196665	1.9	-18.0	1.9	1.0	GNGT1	Homo sapiens guanine nucleotide binding protein (G protein), gamma transducing activity polypeptide 1 (GNGT1), mRNA [NM_021955]	2792

A_23_P404965	-3.3	1.1	1.1	1.0	GNL1	Homo sapiens guanine nucleotide binding protein-like 1 (GNL1), mRNA [NM_005275]	2794
A_23_P34578	1.1	-1.0	-7.2	-1.0	GNL2	Homo sapiens guanine nucleotide binding protein-like 2 (nucleolar) (GNL2), mRNA [NM_013285]	29889
A_23_P22499	-1.6	-1.6	-1.6	-2.0	GNL3L	Homo sapiens guanine nucleotide binding protein-like 3 (nucleolar)-like (GNL3L), transcript variant 2, mRNA [NM_019067]	54552

A_24_P100351	1.7	1.8	2.2	1.4	GNL3L	Homo sapiens guanine nucleotide binding protein-like 3 (nucleolar)-like (GNL3L), transcript variant 2, mRNA [NM_019067]	54552
A_24_P930927	-1.3	-1.1	-1.2	-5.6	GNPTAB	N-acetylglucosamine-1-phosphate transferase, alpha and beta subunits [Source:HGNC Symbol;Acc:29670] [ENST00000552009]	79158
A_24_P288979	1.5	-8.3	1.0	-1.2	GNPTAB	Homo sapiens N-acetylglucosamine-1-phosphate transferase, alpha and beta subunits (GNPTAB), mRNA [NM_024312]	79158

A_23_P346265	1.2	-1.9	1.8	-2.7	GNPTAB	Homo sapiens N-acetylglucosamine-1-phosphate transferase, alpha and beta subunits (GNPTAB), mRNA [NM_024312]	79158
A_23_P14886	-1.1	3.1	2.7	-3.1	GNPTG	Homo sapiens N-acetylglucosamine-1-phosphate transferase, gamma subunit (GNPTG), mRNA [NM_032520]	84572
A_23_P254594	-1.4	-1.0	1.2	-2.2	GNRH1	Homo sapiens gonadotropin-releasing hormone 1 (luteinizing-releasing hormone) (GNRH1), transcript variant 1, mRNA [NM_000825]	2796

A_23_P155804	1.2	1.0	-3.1	1.0	GNRHR	Homo sapiens gonadotropin-releasing hormone receptor (GNRHR), transcript variant 2, mRNA [NM_001012763]	2798
A_23_P13998	-2.5	1.4	-3.8	-2.7	GOLGA2P5	Homo sapiens golgin A2 pseudogene 5 (GOLGA2P5), transcript variant 1, non-coding RNA [NR_036632]	55592
A_23_P98864	-5.4	1.1	1.0	-1.5	GOLGA3	Homo sapiens golgin A3 (GOLGA3), transcript variant 1, mRNA [NM_005895]	2802
A_24_P187706	1.0	1.0	-3.8	1.0	GOLGA4	Homo sapiens golgin A4 (GOLGA4), transcript variant 2, mRNA [NM_002078]	2803

A_24_P546375	-2.3	1.9	6.6	1.7	GOLGA6A	Homo sapiens golgin A6 family, member A (GOLGA6A), mRNA [NM_0010 38640]	342096
A_23_P106371	1.3	-3.0	-1.1	1.3	GOLGA6A	Homo sapiens golgin A6 family, member A (GOLGA6A), mRNA [NM_0010 38640]	342096
A_24_P153880	1.1	-8.6	-1.0	-1.2	GOLGA6L9	Homo sapiens golgin A6 family-like 9 (GOLGA6L 9), mRNA [NM_1981 81]	440295
A_32_P231086	-1.0	-2.8	-1.1	-1.3	GOLGA6L9	Homo sapiens golgin A6 family-like 9 (GOLGA6L 9), mRNA [NM_1981 81]	440295
A_24_P41079	2.1	5.3	1.1	1.1	GOLGA6L9	Homo sapiens golgin A6 family-like 9 (GOLGA6L 9), mRNA [NM_1981 81]	440295

A_24_P98277	-1.7	-2.4	-1.7	-1.1	GOLGA7	Homo sapiens golgin A7 (GOLGA7), transcript variant 2, mRNA [NM_001002296]	51125
A_32_P85273	1.0	-2.6	-1.0	-1.2	GOLGA8A	Homo sapiens golgin A8 family, member A (GOLGA8A), transcript variant 1, mRNA [NM_181077]	23015
A_32_P119569	1.1	1.0	-2.1	-1.0	GOLGA8E	Homo sapiens golgin A8 family, member E (GOLGA8E), non-coding RNA [NR_033350]	390535
A_32_P59116	-1.9	-1.1	-2.9	2.3	GOLGA8F	Homo sapiens golgin A8 family, member F (GOLGA8F), non-coding RNA [NR_033351]	100132565

A_23_P135787	-8.6	-1.0	-1.2	-1.2	GOLGB1	Homo sapiens golgin B1 (GOLGB1), mRNA [NM_004487]	2804
A_24_P927500	-1.0	-4.4	-1.0	-1.0	GOLM1	Homo sapiens cDNA FLJ23608 fis, clone ADKA01790. [AK074188]	51280
A_24_P394865	1.1	1.1	1.1	4.5	GOLM1	Homo sapiens golgi membrane protein 1 (GOLM1), transcript variant 1, mRNA [NM_016548]	51280
A_23_P146512	3.4	2.3	3.2	3.9	GOLM1	Homo sapiens golgi membrane protein 1 (GOLM1), transcript variant 1, mRNA [NM_016548]	51280

A_24_P321511	-14.7	-1.8	-1.4	-1.3	GOLT1B	Homo sapiens golgi transport 1B (GOLT1B), mRNA [NM_016072]	51026
A_24_P276853	5.4	6.4	4.8	3.1	GON4L	Homo sapiens gon-4-like (C. elegans) (GON4L), transcript variant 1, mRNA [NM_001037533]	54856
A_23_P336040	1.0	-1.0	-1.1	-3.0	GOPC	Homo sapiens golgi-associated PDZ and coiled-coil motif containing (GOPC), transcript variant 1, mRNA [NM_020399]	57120
A_23_P58877	-2.4	-3.1	-2.6	-5.2	GOPC	Homo sapiens golgi-associated PDZ and coiled-coil motif containing (GOPC), transcript variant 1, mRNA [NM_020399]	57120

A_23_P368145	-2.3	1.7	-1.4	-1.1	GORAB	Homo sapiens golgin, RAB6-interacting (GORAB), transcript variant 1, mRNA [NM_152281]	92344
A_24_P169462	-2.4	-2.3	-1.0	-1.1	GOSR1	Homo sapiens golgi SNAP receptor complex member 1 (GOSR1), transcript variant 1, mRNA [NM_004871]	9527
A_23_P311608	-2.1	-1.0	-1.1	-1.0	GOSR2	Homo sapiens golgi SNAP receptor complex member 2 (GOSR2), transcript variant B, mRNA [NM_054022]	9570

A_23_P207719	1.2	1.5	-2.5	1.1	GOSR2	Homo sapiens golgi SNAP receptor complex member 2 (GOSR2), transcript variant A, mRNA [NM_004287]	9570
A_24_P108242	-1.7	-3.6	-1.1	-1.1	GOSR2	Homo sapiens golgi SNAP receptor complex member 2 (GOSR2), transcript variant A, mRNA [NM_004287]	9570
A_23_P63825	-2.3	-1.7	-1.2	1.3	GOT1	Homo sapiens glutamic-oxaloacetic transaminase 1, soluble (aspartate aminotransferase 1) (GOT1), mRNA [NM_002079]	2805

A_24_P808720	-1.4	-2.5	-2.4	1.9	GOT1L1	Homo sapiens glutamic-oxaloacetic transaminase 1-like 1 (GOT1L1), mRNA [NM_152413]	137362
A_23_P106575	-4.9	1.6	1.6	1.6	GOT2	Homo sapiens glutamic-oxaloacetic transaminase 2, mitochondrial (aspartate aminotransferase 2) (GOT2), nuclear gene encoding mitochondrial protein, mRNA [NM_002080]	2806

A_24_P692198	1.3	1.1	-2.5	1.1	GOT2	Homo sapiens glutamic-oxaloacetic transaminase 2, mitochondrial (aspartate aminotransferase 2) (GOT2), nuclear gene encoding mitochondrial protein, mRNA [NM_002080]	2806
A_24_P270829	1.0	-1.8	1.3	-3.1	GP1BA	Homo sapiens glycoprotein Ib (platelet), alpha polypeptide (GP1BA), mRNA [NM_000173]	2811
A_24_P190007	-6.9	-1.5	-1.2	-1.5	GP2	Homo sapiens glycoprotein 2 (zymogen granule membrane) (GP2), transcript variant 1, mRNA [NM_001007240]	2813

A_23_P27784	4.0	4.0	4.4	2.4	GP6	Homo sapiens glycoprotein VI (platelet) (GP6), transcript variant 2, mRNA [NM_016363]	51206
A_24_P319374	-1.9	-2.0	-1.1	-1.0	GPA33	Homo sapiens glycoprotein A33 (transmembrane) (GPA33), mRNA [NM_005814]	10223
A_23_P104237	1.0	1.0	1.0	2.0	GPAM	Homo sapiens glycerol-3-phosphate acyltransferase, mitochondrial (GPAM), nuclear gene encoding mitochondrial protein, transcript variant 2, mRNA [NM_020918]	57678

A_23_P68240	-2.1	2.2	-1.7	1.8	GPAT2	Homo sapiens glycerol-3-phosphate acyltransferase 2, mitochondrial (GPAT2), nuclear gene encoding mitochondrial protein, mRNA [NM_207328]	150763
A_23_P400378	3.8	3.0	1.6	3.8	GPBAR1	Homo sapiens G protein-coupled bile acid receptor 1 (GPBAR1), transcript variant 1, mRNA [NM_001077191]	151306
A_23_P30395	1.2	-6.5	1.0	-1.2	GPBP1	Homo sapiens GC-rich promoter binding protein 1 (GPBP1), transcript variant 1, mRNA [NM_022913]	65056

A_23_P593	-3.1	-1.9	-9.2	-1.9	GPBP1L1	Homo sapiens GC-rich promoter binding protein 1-like 1 (GPBP1L1), mRNA [NM_021639]	60313
A_23_P209904	2.0	1.5	2.3	1.3	GPC1	Homo sapiens glypican 1 (GPC1), mRNA [NM_002081]	2817
A_23_P251795	1.1	1.1	1.1	-3.6	GPC2	Homo sapiens glypican 2 (GPC2), mRNA [NM_152742]	221914
A_23_P435407	-3.2	1.8	1.7	1.3	GPC4	Homo sapiens glypican 4 (GPC4), mRNA [NM_001448]	2239
A_23_P112874	-5.4	-1.7	-1.9	-94.2	GPC5	Homo sapiens glypican 5 (GPC5), mRNA [NM_004466]	2262
A_23_P371682	2.7	4.8	2.4	2.4	GPC6	Homo sapiens glypican 6 (GPC6), mRNA [NM_005708]	10082

A_32_P97169	1.7	1.7	2.4	-1.3	GPC6	Homo sapiens glypican 6 (GPC6), mRNA [NM_005708]	10082
A_23_P353704	1.0	-38.7	1.5	1.0	GPCPD1	Homo sapiens glycerophosphocholine phosphodiesterase GDE1 homolog (S. cerevisiae) (GPCPD1), mRNA [NM_019593]	56261
A_24_P31671	-5.6	1.2	1.2	1.0	GPCPD1	Homo sapiens glycerophosphocholine phosphodiesterase GDE1 homolog (S. cerevisiae) (GPCPD1), mRNA [NM_019593]	56261

A_23_P413721	2.6	3.1	-1.1	-1.7	GPD1	Homo sapiens glycerol-3-phosphate dehydrogenase 1 (soluble) (GPD1), mRNA [NM_005276]	2819
A_23_P162660	1.9	2.0	-4.0	1.7	GPD1	Homo sapiens glycerol-3-phosphate dehydrogenase 1 (soluble) (GPD1), mRNA [NM_005276]	2819
A_23_P204736	1.3	3.0	2.2	2.2	GPD1	Homo sapiens glycerol-3-phosphate dehydrogenase 1 (soluble) (GPD1), mRNA [NM_005276]	2819
A_23_P318284	2.3	1.5	-1.5	-1.2	GPD1L	Homo sapiens glycerol-3-phosphate dehydrogenase 1-like (GPD1L), mRNA [NM_015141]	23171

A_23_P47181	-2.4	-1.6	-2.3	-2.3	GPHA2	Homo sapiens glycoprotein hormone alpha 2 (GPHA2), mRNA [NM_130769]	170589
A_23_P336678	-1.4	-1.9	1.9	-4.0	GPHB5	Homo sapiens glycoprotein hormone beta 5 (GPHB5), mRNA [NM_145171]	122876
A_23_P156928	1.1	1.0	-1.9	-7.8	GPLD1	Homo sapiens glycosylphosphatidylinositol specific phospholipase D1 (GPLD1), transcript variant 1, mRNA [NM_001503]	2822
A_23_P121545	7.5	7.3	7.1	6.9	GPM6A	Homo sapiens glycoprotein M6A (GPM6A), transcript variant 2, mRNA [NM_201591]	2823

A_24_P933908	-13.0	-1.1	-1.3	-1.3	GPNMB	Homo sapiens glycoprotein (transmembrane) nmb, mRNA (cDNA clone IMAGE:3345861), complete cds. [BC011595]	10457
A_23_P134426	-2.2	1.0	1.1	1.0	GPNMB	Homo sapiens glycoprotein (transmembrane) nmb (GPNMB), transcript variant 1, mRNA [NM_001005340]	10457
A_24_P295379	-4.2	1.0	-1.2	1.1	GPR107	Homo sapiens G protein-coupled receptor 107 (GPR107), transcript variant 3, mRNA [NM_020960]	57720

A_24_P183544	1.3	-1.2	-1.2	3.2	GPR110	G protein-coupled receptor 110 [Source:HGNC Symbol;Acc:18990] [ENST00000371253]	266977
A_23_P214267	-1.0	-1.0	-11.5	-1.1	GPR110	Homo sapiens G protein-coupled receptor 110 (GPR110), transcript variant 1, mRNA [NM_153840]	266977
A_23_P167872	-5.4	-2.9	-3.0	-3.0	GPR111	Homo sapiens G protein-coupled receptor 111 (GPR111), mRNA [NM_153839]	222611
A_23_P312901	2.4	2.3	1.8	4.6	GPR112	Homo sapiens G protein-coupled receptor 112 (GPR112), mRNA [NM_153834]	139378

A_24_P85881	-2.6	-1.3	-3.7	-1.5	GPR113	Homo sapiens G protein-coupled receptor 113 (GPR113), transcript variant 3, mRNA [NM_153835]	165082
A_23_P206290	-5.7	1.0	1.4	1.8	GPR114	Homo sapiens G protein-coupled receptor 114 (GPR114), mRNA [NM_153837]	221188
A_23_P206293	1.3	-1.6	-4.4	-1.8	GPR114	Homo sapiens G protein-coupled receptor 114 (GPR114), mRNA [NM_153837]	221188
A_24_P337671	3.3	3.3	-2.4	2.9	GPR115	Homo sapiens G protein-coupled receptor 115 (GPR115), mRNA [NM_153838]	221393

A_23_P21425	1.1	1.1	-2.1	-1.1	GPR119	Homo sapiens G protein-coupled receptor 119 (GPR119), mRNA [NM_178471]	139760
A_23_P48320	3.3	5.5	3.8	1.9	GPR12	Homo sapiens G protein-coupled receptor 12 (GPR12), mRNA [NM_005288]	2835
A_23_P327605	1.5	-4.8	-1.2	-1.2	GPR123	Homo sapiens G protein-coupled receptor 123 (GPR123), mRNA [NM_001083909]	84435
A_23_P43276	5.4	3.4	3.8	3.8	GPR124	Homo sapiens G protein-coupled receptor 124 (GPR124), mRNA [NM_032777]	25960

A_24_P942945	-3.4	-5.2	1.0	-1.0	GPR126	Homo sapiens G protein-coupled receptor 126 (GPR126), transcript variant a1, mRNA [NM_020455]	57211
A_24_P201994	-1.7	1.8	-2.1	-2.2	GPR132	Homo sapiens G protein-coupled receptor 132 (GPR132), mRNA [NM_013345]	29933
A_24_P344045	9.1	5.2	4.8	5.7	GPR135	Homo sapiens G protein-coupled receptor 135 (GPR135), mRNA [NM_022571]	64582
A_23_P205575	-18.7	-3.2	-1.1	-1.3	GPR135	Homo sapiens G protein-coupled receptor 135 (GPR135), mRNA [NM_022571]	64582

A_23_P410736	5.2	5.2	4.3	4.7	GPR135	Homo sapiens G protein-coupled receptor 135 (GPR135), mRNA [NM_022571]	64582
A_23_P416686	3.4	-1.4	2.5	2.1	GPR137	Homo sapiens G protein-coupled receptor 137 (GPR137), transcript variant 3, mRNA [NM_020155]	56834
A_23_P49559	-2.1	-3.1	-2.0	-4.3	GPR142	Homo sapiens G protein-coupled receptor 142 (GPR142), mRNA [NM_181790]	350383
A_23_P45560	-1.2	2.3	1.6	-1.9	GPR143	Homo sapiens G protein-coupled receptor 143 (GPR143), mRNA [NM_000273]	4935

A_24_P74896	-1.3	-1.2	-2.1	-2.1	GPR144	Homo sapiens G protein-coupled receptor 144 (GPR144), mRNA [NM_001161808]	347088
A_23_P20035	-1.1	-2.4	-1.3	1.1	GPR146	Homo sapiens G protein-coupled receptor 146 (GPR146), mRNA [NM_138445]	115330
A_24_P332218	1.2	-2.5	-1.7	-1.1	GPR149	Homo sapiens G protein-coupled receptor 149 (GPR149), mRNA [NM_001038705]	344758
A_23_P6943	2.2	-1.0	1.2	1.3	GPR15	Homo sapiens G protein-coupled receptor 15 (GPR15), mRNA [NM_005290]	2838

A_23_P213527	1.2	1.0	-8.1	1.0	GPR150	G protein-coupled receptor 150 [Source:HGNC Symbol;Acc:23628] [ENST00000380007]	285601
A_23_P92275	-1.0	-2.5	-3.7	-1.9	GPR156	Homo sapiens G protein-coupled receptor 156 (GPR156), transcript variant 1, mRNA [NM_153002]	165829
A_24_P757255	1.6	1.5	1.5	-6.1	GPR157	G protein-coupled receptor 157 [Source:HGNC Symbol;Acc:23687] [ENST00000377411]	80045
A_24_P71280	4.7	4.1	5.3	1.6	GPR157	Homo sapiens cDNA FLJ12132 fis, clone MAMMA1000277. [AK022194]	80045

A_24_P349117	1.0	-1.0	-3.7	-1.8	GPR158	Homo sapiens G protein-coupled receptor 158 (GPR158), mRNA [NM_020752]	57512
A_23_P167005	1.2	1.2	1.2	-6.0	GPR160	Homo sapiens G protein-coupled receptor 160 (GPR160), mRNA [NM_014373]	26996
A_23_P303455	-1.0	-1.1	-2.1	-2.7	GPR161	Homo sapiens G protein-coupled receptor 161 (GPR161), transcript variant 2, mRNA [NM_153832]	23432
A_23_P103532	3.1	2.0	2.1	1.7	GPR161	Homo sapiens G protein-coupled receptor 161 (GPR161), transcript variant 2, mRNA [NM_153832]	23432

A_24_P287392	-2.7	-1.0	-1.0	-1.0	GPR162	Homo sapiens G protein-coupled receptor 162 (GPR162), transcript variant A-2, mRNA [NM_019858]	27239
A_23_P16963	-1.3	-3.6	-3.8	-1.6	GPR17	Homo sapiens G protein-coupled receptor 17 (GPR17), transcript variant 2, mRNA [NM_005291]	2840
A_23_P15692	1.1	1.5	-9.0	1.1	GPR172B	Homo sapiens G protein-coupled receptor 172B (GPR172B), transcript variant 2, mRNA [NM_017986]	55065
A_23_P346291	1.3	1.8	1.7	2.7	GPR173	Homo sapiens G protein-coupled receptor 173 (GPR173), mRNA [NM_018969]	54328

A_23_P37676	3.3	4.8	3.2	5.7	GPR176	Homo sapiens G protein-coupled receptor 176 (GPR176), mRNA [NM_007223]	11245
A_23_P14165	-1.2	-1.6	1.2	-2.3	GPR18	Homo sapiens G protein-coupled receptor 18 (GPR18), transcript variant 1, mRNA [NM_005292]	2841
A_23_P99285	-19.9	-1.2	1.2	2.0	GPR19	Homo sapiens G protein-coupled receptor 19 (GPR19), mRNA [NM_006143]	2842
A_23_P159237	-1.5	1.8	2.4	-1.1	GPR20	Homo sapiens G protein-coupled receptor 20 (GPR20), mRNA [NM_005293]	2843

A_23_P135219	-2.1	-1.7	-1.9	-1.7	GPR21	Homo sapiens G protein-coupled receptor 21 (GPR21), mRNA [NM_005294]	2844
A_23_P20172	-1.0	-1.0	-3.7	1.0	GPR22	Homo sapiens G protein-coupled receptor 22 (GPR22), mRNA [NM_005295]	2845
A_23_P35000	1.1	1.8	2.1	2.2	GPR3	Homo sapiens G protein-coupled receptor 3 (GPR3), mRNA [NM_005281]	2827
A_23_P42112	-1.2	-8.1	-1.2	-2.1	GPR31	Homo sapiens G protein-coupled receptor 31 (GPR31), mRNA [NM_005299]	2853

A_23_P154245	1.1	1.0	2.0	-2.1	GPR35	Homo sapiens G protein-coupled receptor 35 (GPR35), transcript variant 1, mRNA [NM_005301]	2859
A_23_P305550	1.6	2.2	-1.0	1.4	GPR35	Homo sapiens G protein-coupled receptor 35 (GPR35), transcript variant 3, mRNA [NM_001195382]	2859
A_24_P145633	-1.1	-2.8	1.0	-2.6	GPR37L1	Homo sapiens G protein-coupled receptor 37 like 1 (GPR37L1), mRNA [NM_004767]	9283
A_23_P206280	1.0	1.0	-4.1	1.0	GPR56	Homo sapiens G protein-coupled receptor 56 (GPR56), transcript variant 3, mRNA [NM_201525]	9289

A_23_P206284	-1.7	-1.1	1.1	-5.0	GPR56	Homo sapiens G protein-coupled receptor 56 (GPR56), transcript variant 3, mRNA [NM_201525]	9289
A_23_P156919	1.4	3.5	2.5	1.7	GPR6	Homo sapiens G protein-coupled receptor 6 (GPR6), mRNA [NM_005284]	2830
A_23_P326303	-2.6	-1.5	3.2	1.2	GPR61	Homo sapiens G protein-coupled receptor 61 (GPR61), mRNA [NM_031936]	83873
A_23_P253692	1.0	1.0	-3.1	1.0	GPR64	Homo sapiens G protein-coupled receptor 64 (GPR64), transcript variant 1, mRNA [NM_001079858]	10149

A_23_P54243	-1.4	-3.4	-1.4	1.7	GPR68	Homo sapiens G protein-coupled receptor 68 (GPR68), transcript variant 2, mRNA [NM_003485]	8111
A_24_P931443	-6.3	-1.0	1.0	1.0	GPR68	Homo sapiens G protein-coupled receptor 68 (GPR68), transcript variant 2, mRNA [NM_003485]	8111
A_23_P69652	-1.1	1.5	-1.1	-2.1	GPR78	Homo sapiens G protein-coupled receptor 78 (GPR78), transcript variant 1, mRNA [NM_080819]	27201
A_24_P280148	1.0	-1.5	-2.9	-2.2	GPR82	Homo sapiens G protein-coupled receptor 82 (GPR82), mRNA [NM_080817]	27197

A_23_P215687	1.3	-2.6	-2.1	-1.0	GPR85	Homo sapiens G protein-coupled receptor 85 (GPR85), transcript variant 2, mRNA [NM_018970]	54329
A_23_P149749	1.5	-3.0	1.5	-1.1	GPR88	Homo sapiens strg mRNA for striatum-specific G protein-coupled receptor, complete cds. [AB042410]	54112
A_23_P140760	-1.0	2.1	-1.0	-2.5	GPR97	Homo sapiens G protein-coupled receptor 97 (GPR97), mRNA [NM_170776]	222487
A_23_P36831	1.0	-1.1	-1.1	-3.6	GPRC5A	Homo sapiens G protein-coupled receptor, family C, group 5, member A (GPRC5A), mRNA [NM_003979]	9052

A_23_P346670	-3.0	-2.8	-1.5	-1.8	GPRC5C	G protein-coupled receptor, family C, group 5, member C [Source:HGNC Symbol;Acc:13309] [ENST00000392628]	55890
A_32_P109029	-1.0	-5.6	-1.1	-1.0	GPRC5C	Homo sapiens G protein-coupled receptor, family C, group 5, member C (GPRC5C), transcript variant 1, mRNA [NM_022036]	55890
A_23_P340296	2.8	2.1	2.0	1.3	GPSM1	Homo sapiens G-protein signaling modulator 1 (GPSM1), transcript variant 2, mRNA [NM_015597]	26086

A_23_P250671	-2.1	-1.7	-1.3	-1.4	GPX1	Homo sapiens glutathione peroxidase 1 (GPX1), transcript variant 2, mRNA [NM_201397]	2876
A_23_P28075	-3.0	1.0	2.1	1.0	GPX4	Homo sapiens glutathione peroxidase 4 (phospholipid hydroperoxidase) (GPX4), transcript variant 3, mRNA [NM_001039848]	2879
A_24_P109111	-2.2	-1.1	-1.2	-1.3	GPX6	Homo sapiens glutathione peroxidase 6 (olfactory) (GPX6), mRNA [NM_182701]	257202

A_24_P418816	-4.4	-4.5	-2.5	-1.9	GPX7	Homo sapiens glutathione peroxidase 7 (GPX7), mRNA [NM_015696]	2882
A_23_P73972	1.0	-2.0	-1.0	-1.1	GPX7	Homo sapiens glutathione peroxidase 7 (GPX7), mRNA [NM_015696]	2882
A_23_P56213	1.8	2.3	1.5	1.1	GRAMD1A	Homo sapiens GRAM domain containing 1A (GRAMD1A), transcript variant 1, mRNA [NM_020895]	57655
A_23_P49638	4.4	4.9	3.0	2.6	GRAP	Homo sapiens GRB2-related adaptor protein (GRAP), mRNA [NM_006613]	10750

A_24_P10884	-1.0	-1.6	-2.1	1.2	GRAP2	Homo sapiens GRB2-related adaptor protein 2 (GRAP2), mRNA [NM_004810]	9402
A_23_P143720	1.1	1.0	-6.3	-1.0	GRAP2	Homo sapiens GRB2-related adaptor protein 2 (GRAP2), mRNA [NM_004810]	9402
A_23_P122863	1.0	-2.1	1.0	1.0	GRB10	Homo sapiens growth factor receptor-bound protein 10 (GRB10), transcript variant 4, mRNA [NM_001001555]	2887
A_24_P407717	1.5	-6.6	-1.1	1.0	GRB2	Homo sapiens growth factor receptor-bound protein 2 (GRB2), transcript variant 1, mRNA [NM_002086]	2885

A_23_P163992	2.6	2.3	2.3	-1.0	GRB7	Homo sapiens growth factor receptor-bound protein 7 (GRB7), transcript variant 1, mRNA [NM_005310]	2886
A_23_P119040	-2.5	1.2	-1.2	1.0	GREB1L	Homo sapiens growth regulation by estrogen in breast cancer-like (GREB1L), mRNA [NM_001142966]	80000
A_23_P97181	1.1	1.3	1.1	-2.3	GREM2	Homo sapiens gremlin 2 (GREM2), mRNA [NM_022469]	64388
A_23_P52067	3.8	2.0	2.4	-1.4	GRHL3	Homo sapiens grainyhead-like 3 (Drosophila) (GRHL3), transcript variant 2, mRNA [NM_198173]	57822

A_23_P60225	-2.1	-8.9	-1.7	-1.3	GRHPR	Homo sapiens glyoxylate reductase/hydroxypyruvate reductase (GRHPR), mRNA [NM_012203]	9380
A_32_P177897	1.1	-2.3	1.1	1.0	GRIA3	Homo sapiens glutamate receptor, ionotrophic, AMPA 3 (GRIA3), transcript variant 2, mRNA [NM_000828]	2892
A_23_P32847	3.0	2.5	2.6	2.2	GRID2	Homo sapiens glutamate receptor, ionotropic, delta 2 (GRID2), mRNA [NM_001510]	2895
A_32_P229221	-1.5	-8.1	1.6	1.2	GRID2	Homo sapiens glutamate receptor, ionotropic, delta 2 (GRID2), mRNA [NM_001510]	2895

A_32_P115796	-6.3	1.3	-1.5	-1.3	GRIK2	Homo sapiens glutamate receptor, ionotropic, kainate 2 (GRIK2), transcript variant 1, mRNA [NM_021956]	2898
A_23_P149084	5.0	6.1	-6.5	-1.7	GRIK3	Homo sapiens glutamate receptor, ionotropic, kainate 3 (GRIK3), mRNA [NM_000831]	2899
A_24_P870799	23.5	23.8	22.5	14.6	GRIK3	Homo sapiens glutamate receptor, ionotropic, kainate 3 (GRIK3), mRNA [NM_000831]	2899
A_23_P16063	1.5	-3.1	1.2	1.3	GRIK5	Homo sapiens glutamate receptor, ionotropic, kainate 5 (GRIK5), mRNA [NM_002088]	2901

A_23_P96072	3.8	4.0	2.3	3.1	GRIN1	Homo sapiens glutamate receptor, ionotropic, N-methyl D-aspartate 1 (GRIN1), transcript variant NR1-3, mRNA [NM_007327]	2902
A_24_P381844	-5.6	-1.6	1.4	-1.5	GRIN2A	Homo sapiens glutamate receptor, ionotropic, N-methyl D-aspartate 2A (GRIN2A), transcript variant 2, mRNA [NM_000833]	2903
A_24_P358628	1.1	-2.8	1.0	1.1	GRIN2A	Homo sapiens glutamate receptor, ionotropic, N-methyl D-aspartate 2A (GRIN2A), transcript variant 2, mRNA [NM_000833]	2903

A_23_P108180	2.4	4.0	-1.4	1.9	GRIN3B	Homo sapiens glutamate receptor, ionotropic, N-methyl-D-aspartate 3B (GRIN3B), mRNA [NM_138690]	116444
A_23_P316960	2.6	3.3	-1.1	1.9	GRINA	Homo sapiens glutamate receptor, ionotropic, N-methyl D-aspartate-associated protein 1 (glutamate binding) (GRINA), transcript variant 1, mRNA [NM_000837]	2907
A_32_P380675	2.3	2.2	2.1	1.7	GRIP2	Homo sapiens glutamate receptor interacting protein 2 (GRIP2), mRNA [NM_001080423]	80852

A_23_P313542	-1.2	-1.6	-10.9	-2.4	GRK1	Homo sapiens G protein-coupled receptor kinase 1 (GRK1), mRNA [NM_002929]	6011
A_23_P257256	-3.3	1.7	1.4	1.1	GRK6	Homo sapiens G protein-coupled receptor kinase 6 (GRK6), transcript variant 2, mRNA [NM_002082]	2870
A_23_P30976	-1.0	-1.1	-1.1	-3.1	GRM1	Homo sapiens glutamate receptor, metabotropic 1 (GRM1), transcript variant 1, mRNA [NM_000838]	2911
A_23_P70588	2.1	2.7	2.4	1.0	GRM4	Homo sapiens glutamate receptor, metabotropic 4 (GRM4), mRNA [NM_000841]	2914

A_23_P22364	-1.0	-1.0	-1.1	-2.3	GRM7	Homo sapiens glutamate receptor, metabotropic 7 (GRM7), transcript variant 1, mRNA [NM_000844]	2917
A_32_P8221	1.9	1.0	-8.8	1.0	GRM8	Homo sapiens glutamate receptor, metabotropic 8 (GRM8), transcript variant 1, mRNA [NM_000845]	2918
A_24_P938303	1.5	3.3	-1.2	1.7	GRPR	Human GRP/bombesin receptor mRNA, partial cds. [U57365]	2925
A_24_P256929	1.3	1.4	-2.6	-1.0	GRRP1	Homo sapiens glycine/arginine rich protein 1 (GRRP1), mRNA [NM_024869]	79927

A_23_P46131	6.7	4.3	4.3	4.3	GRRP1	Homo sapiens glycine/arginine rich protein 1 (GRRP1), mRNA [NM_024869]	79927
A_23_P212937	1.7	-2.2	2.3	1.2	GRSF1	Homo sapiens G-rich RNA sequence binding factor 1 (GRSF1), transcript variant 1, mRNA [NM_002092]	2926
A_23_P142154	-1.1	-1.1	1.1	-3.7	GRWD1	Homo sapiens glutamate-rich WD repeat containing 1 (GRWD1), mRNA [NM_031485]	83743
A_24_P495367	-1.0	-1.0	-12.2	-1.1	GRXCR1	Homo sapiens glutaredoxin, cysteine rich 1 (GRXCR1), mRNA [NM_001080476]	389207

A_23_P109382	-2.3	-1.6	-1.9	-2.6	GSC2	Homo sapiens goosecoid homeobox 2 (GSC2), mRNA [NM_005315]	2928
A_23_P112103	-2.1	1.0	1.0	-1.0	GSDMD	Homo sapiens gasdermin D (GSDMD), transcript variant 1, mRNA [NM_024736]	79792
A_23_P368251	1.1	3.9	3.9	6.3	GSG1L	Homo sapiens GSG1-like (GSG1L), transcript variant 2, mRNA [NM_144675]	146395
A_32_P58872	-1.0	-6.5	-1.1	-1.1	GSG1L	Homo sapiens GSG1-like (GSG1L), transcript variant 1, mRNA [NM_001109763]	146395

A_24_P76521	2.2	2.1	1.0	1.8	GSG2	Homo sapiens cDNA FLJ32129 fis, clone PEBLM2000213, weakly similar to Mus musculus genes for integrin aM290, hapsin. [AK056691]	83903
A_23_P19115	-4.1	1.0	1.0	1.1	GSPT2	Homo sapiens G1 to S phase transition 2 (GSPT2), mRNA [NM_018094]	23708
A_23_P135417	-8.4	-1.1	-1.2	-1.2	GSTA1	Homo sapiens glutathione S-transferase alpha 1 (GSTA1), mRNA [NM_145740]	2938
A_24_P300394	-10.2	1.0	1.0	1.0	GSTA2	Homo sapiens glutathione S-transferase alpha 2 (GSTA2), mRNA [NM_000846]	2939

A_23_P110288	1.0	-1.0	-1.1	-2.3	GSTCD	Homo sapiens glutathione S-transferase, C-terminal domain containing (GSTCD), transcript variant 2, mRNA [NM_024751]	79807
A_23_P20107	-1.2	2.3	-1.7	-1.0	GSTK1	Homo sapiens glutathione S-transferase kappa 1 (GSTK1), nuclear gene encoding mitochondrial protein, transcript variant 1, mRNA [NM_015917]	373156
A_23_P58869	-1.1	-1.2	-4.5	-1.2	GSTM2P1	Homo sapiens glutathione S-transferase mu 2 (muscle) pseudogene 1 (GSTM2P1), non-coding RNA [NR_002932]	442245

A_24_P914434	-1.1	-1.2	-2.4	-1.7	GSTM3	Homo sapiens glutathione S-transferase mu 3 (brain) (GSTM3), transcript variant 1, mRNA [NM_000849]	2947
A_24_P319001	2.2	2.6	2.8	1.1	GSTT1	Homo sapiens glutathione S-transferase theta 1 (GSTT1), mRNA [NM_000853]	2952
A_23_P357571	-17.3	1.0	1.1	1.0	GSTT2	Homo sapiens glutathione S-transferase theta 2 (GSTT2), mRNA [NM_000854]	2953
A_23_P29163	1.3	1.3	1.2	-2.3	GSTTP1	Homo sapiens glutathione S-transferase theta pseudogene 1 (GSTTP1), non-coding RNA [NR_003081]	25774

A_23_P106204	-2.0	-1.3	-1.0	-2.7	GSTZ1	Homo sapiens glutathione transferase zeta 1 (GSTZ1), transcript variant 1, mRNA [NM_145870]	2954
A_32_P470728	2.1	2.2	1.7	-1.6	GSX1	Homo sapiens GS homeobox 1 (GSX1), mRNA [NM_145657]	219409
A_23_P153945	1.1	1.1	-5.7	-1.0	GTDC1	Homo sapiens glycosyltransferase-like domain containing 1 (GTDC1), transcript variant 1, mRNA [NM_001006636]	79712
A_23_P131170	1.2	-6.6	1.0	1.1	GTDC1	Homo sapiens clone HQ0159 PRO0159 mRNA, complete cds. [AF090899]	79712

A_24_P270525	-3.3	1.4	-2.0	-1.3	GTF2A2	Homo sapiens general transcription factor IIA, 2, 12kDa (GTF2A2), mRNA [NM_004492]	2958
A_23_P34628	3.1	2.1	1.4	3.7	GTF2B	Homo sapiens general transcription factor IIB (GTF2B), mRNA [NM_001514]	2959
A_24_P3704	1.4	-2.5	1.4	1.1	GTF2H2B	Homo sapiens general transcription factor IIH, polypeptide 2B (pseudogene) (GTF2H2B), non-coding RNA [NR_033417]	653238

A_24_P274640	2.2	-1.1	-1.4	-1.8	GTF2H2D	Homo sapiens general transcription factor IIH, polypeptide 2D (GTF2H2D), mRNA [NM_001042490]	730394
A_32_P27763	-1.4	1.2	-3.2	-1.8	GTF2H5	Homo sapiens general transcription factor IIH, polypeptide 5 (GTF2H5), mRNA [NM_207118]	404672
A_24_P803801	-1.3	-1.2	-1.4	-2.2	GTF2I	Homo sapiens general transcription factor IIi (GTF2I), transcript variant 1, mRNA [NM_032999]	2969
A_24_P765784	6.7	6.5	6.3	6.8	GTF2I	general transcription factor IIi [Source:HGNC Symbol;Acc:4659] [ENST00000473759]	2969

A_23_P152344	-1.0	-1.1	-4.9	-1.1	GTF3C1	Homo sapiens general transcription factor IIIC, polypeptide 1, alpha 220kDa (GTF3C1), mRNA [NM_001520]	2975
A_24_P316363	1.8	-2.7	-1.5	-1.0	GTF3C2	Homo sapiens general transcription factor IIIC, polypeptide 2, beta 110kDa (GTF3C2), transcript variant 1, mRNA [NM_001521]	2976
A_23_P429535	-2.6	-2.1	-2.6	-2.5	GTF3C3	Homo sapiens general transcription factor IIIC, polypeptide 3, 102kDa (GTF3C3), transcript variant 1, mRNA [NM_012086]	9330

A_24_P78153	4.6	4.9	4.3	3.6	GTF3C4	Homo sapiens general transcription factor IIIC, polypeptide 4, 90kDa (GTF3C4), mRNA [NM_012204]	9329
A_23_P112406	1.3	1.1	-2.5	-1.4	GTF3C5	Homo sapiens general transcription factor IIIC, polypeptide 5, 63kDa (GTF3C5), transcript variant 2, mRNA [NM_012087]	9328
A_23_P103120	1.2	2.0	-1.8	1.1	GTPBP1	Homo sapiens GTP binding protein 1 (GTPBP1), mRNA [NM_004286]	9567

A_23_P168656	1.7	2.7	2.7	-3.2	GTPBP10	Homo sapiens GTP-binding protein 10 (putative) (GTPBP10), transcript variant 2, mRNA [NM_033107]	85865
A_23_P379026	1.1	-1.2	-2.3	-1.8	GTPBP2	Homo sapiens GTP binding protein 2 (GTPBP2), mRNA [NM_019096]	54676
A_32_P115947	-1.0	1.2	-6.1	-1.1	GTPBP4	GTP binding protein 4 [Source:HGNC Symbol;Acc:21535] [ENST00000360803]	23560
A_24_P391229	2.9	3.5	1.1	1.6	GTPBP5	Homo sapiens GTP binding protein 5 (putative) (GTPBP5), mRNA [NM_015666]	26164

A_23_P113523	1.2	2.2	1.4	2.1	GTPBP6	Homo sapiens GTP binding protein 6 (putative) (GTPBP6), mRNA [NM_012227]	8225
A_23_P57588	2.2	3.2	1.9	1.6	GTSE1	Homo sapiens G-2 and S-phase expressed 1 (GTSE1), mRNA [NM_016426]	51512
A_23_P122414	1.0	1.0	-1.1	-2.7	GUCA1A	Homo sapiens guanylate cyclase activator 1A (retina) (GUCA1A), mRNA [NM_000409]	2978
A_23_P81825	-2.6	3.6	1.8	1.0	GUCA1B	Homo sapiens guanylate cyclase activator 1B (retina) (GUCA1B), mRNA [NM_002098]	2979

A_32_P64200	1.0	-1.0	1.8	-4.1	GUCA1B	Homo sapiens guanylate cyclase activator 1B (retina) (GUCA1B), mRNA [NM_002098]	2979
A_23_P11968	1.1	-1.7	-3.2	-2.3	GUCA2A	Homo sapiens guanylate cyclase activator 2A (guanylin) (GUCA2A), mRNA [NM_033553]	2980
A_23_P63032	4.4	4.3	5.5	3.9	GUCA2B	Homo sapiens guanylate cyclase activator 2B (uroguanylin) (GUCA2B), mRNA [NM_007102]	2981
A_23_P69573	1.3	-1.1	1.2	-3.1	GUCY1A3	Homo sapiens guanylate cyclase 1, soluble, alpha 3 (GUCY1A3), transcript variant 1, mRNA [NM_000856]	2982

A_23_P76312	-1.1	-3.7	-1.1	-1.1	GUCY2C	Homo sapiens guanylate cyclase 2C (heat stable enterotoxin receptor) (GUCY2C), mRNA [NM_004963]	2984
A_32_P4943	-2.4	-1.8	-2.2	-1.8	GUF1	Homo sapiens GUF1 GTPase homolog (S. cerevisiae) (GUF1), mRNA [NM_021927]	60558
A_23_P201097	1.6	1.7	2.4	1.0	GUK1	Homo sapiens guanylate kinase 1 (GUK1), transcript variant 2, mRNA [NM_000858]	2987
A_32_P92654	4.5	3.2	4.7	4.6	GUSBP1	Homo sapiens glucuronidase, beta pseudogene 1 (GUSBP1), transcript variant 3, non-coding RNA [NR_027028]	728411

A_32_P190294	1.1	1.0	-2.2	1.6	GUSBP1	Homo sapiens glucuronidase, beta pseudogene 1 (GUSBP1), transcript variant 1, non-coding RNA [NR_027026]	728411
A_24_P84822	1.1	-46.5	1.1	1.1	GUSBP1	Homo sapiens glucuronidase, beta pseudogene 1 (GUSBP1), transcript variant 3, non-coding RNA [NR_027028]	728411
A_24_P332739	-1.0	-1.7	-5.5	-45.1	GUSBP1	Homo sapiens glucuronidase, beta pseudogene 1 (GUSBP1), transcript variant 3, non-coding RNA [NR_027028]	728411

A_23_P75848	-1.4	1.4	-2.2	1.3	GVINP1	Homo sapiens GTPase, very large interferon inducible pseudogene 1 (GVINP1), non-coding RNA [NR_003945]	387751
A_23_P75850	2.0	2.5	2.3	1.0	GVINP1	Homo sapiens GTPase, very large interferon inducible pseudogene 1 (GVINP1), non-coding RNA [NR_003945]	387751
A_32_P62342	-1.2	-1.2	1.0	-2.1	GXYLT1	Homo sapiens glucoside xylosyltransferase 1 (GXYLT1), transcript variant 1, mRNA [NM_173601]	283464

A_23_P336796	-4.3	-6.3	-4.1	1.3	GXYLT1	Homo sapiens glucoside xylosyltransferase 1 (GXYLT1), transcript variant 1, mRNA [NM_173601]	283464
A_24_P118196	1.3	-2.1	1.5	1.1	GXYLT2	Homo sapiens glucoside xylosyltransferase 2 (GXYLT2), mRNA [NM_001080393]	727936
A_23_P384517	2.3	4.0	1.2	1.4	GYG1	Homo sapiens glycogenin 1 (GYG1), transcript variant 1, mRNA [NM_004130]	2992
A_32_P111701	2.1	2.4	1.7	1.7	GYG2	Homo sapiens glycogenin 2 (GYG2), transcript variant 2, mRNA [NM_003918]	8908

A_23_P208698	2.1	3.1	1.1	-1.0	GYS1	Homo sapiens glycogen synthase 1 (muscle) (GYS1), transcript variant 1, mRNA [NM_002103]	2997
A_23_P36621	-5.3	1.2	1.2	1.0	GYS2	Homo sapiens glycogen synthase 2 (liver) (GYS2), mRNA [NM_021957]	2998
A_23_P117602	-1.3	2.6	-2.1	1.7	GZMB	Homo sapiens granzyme B (granzyme 2, cytotoxic T-lymphocyte-associated serine esterase 1) (GZMB), mRNA [NM_004131]	3002

A_23_P156218	1.1	2.0	1.1	1.1	GZMK	Homo sapiens granzyme K (granzyme 3; tryptase II) (GZMK), mRNA [NM_002104]	3003
A_23_P29257	-1.7	-4.2	-1.7	-1.7	H1F0	Homo sapiens H1 histone family, member 0 (H1F0), mRNA [NM_005318]	3005
A_32_P202859	1.1	2.2	1.2	1.8	H1FNT	Homo sapiens H1 histone family, member N, testis-specific (H1FNT), mRNA [NM_181788]	341567
A_23_P372994	13.8	23.2	23.1	23.0	H1FOO	Homo sapiens H1 histone family, member O, oocyte-specific (H1FOO), mRNA [NM_153833]	132243

A_23_P171270	-1.3	1.7	1.0	-2.7	H2AFB2	Homo sapiens H2A histone family, member B2 (H2AFB2), mRNA [NM_001017991]	474381
A_23_P204277	2.1	1.3	-1.3	1.6	H2AFJ	Homo sapiens H2A histone family, member J (H2AFJ), transcript variant 1, mRNA [NM_177925]	55766
A_23_P432385	-1.0	-3.8	-1.2	-1.0	H2AFJ	Homo sapiens H2A histone family, member J (H2AFJ), transcript variant 1, mRNA [NM_177925]	55766
A_23_P316487	-42.8	1.3	1.3	1.0	H2AFV	Homo sapiens H2A histone family, member V (H2AFV), transcript variant 2, mRNA [NM_138635]	94239

A_32_P85591	-1.3	-2.2	-3.0	-2.8	H2BFXP	Homo sapiens H2B histone family, member X, pseudogene (H2BFXP), non-coding RNA [NR_003238]	767811
A_23_P23141	-2.3	-1.9	-2.9	-1.4	H3F3A	Homo sapiens H3 histone, family 3A (H3F3A), mRNA [NM_002107]	3020
A_24_P105298	-2.1	-1.7	-2.4	-1.2	H3F3A	Homo sapiens H3 histone, family 3A (H3F3A), mRNA [NM_002107]	3020
A_24_P323835	-1.7	-1.6	-2.2	-1.2	H3F3A	Homo sapiens H3 histone, family 3A (H3F3A), mRNA [NM_002107]	3020

A_24_P590537	-2.1	-1.8	-2.6	-1.4	H3F3A	Homo sapiens H3 histone, family 3A (H3F3A), mRNA [NM_002107]	3020
A_23_P152516	1.1	2.4	-1.3	-1.0	H3F3B	Homo sapiens H3 histone, family 3B (H3.3B) (H3F3B), mRNA [NM_005324]	3021
A_24_P626850	-1.0	-1.4	-1.0	-2.6	H6PD	Homo sapiens hexose-6-phosphate dehydrogenase (glucose 1-dehydrogenase) (H6PD), mRNA [NM_004285]	9563
A_24_P541489	-1.7	1.2	-4.2	-2.0	HAB1	H.sapiens B1 mRNA for mucin. [X83412]	55547
A_23_P1173	-4.5	-2.0	-1.1	-2.5	HABP2	Homo sapiens hyaluronan binding protein 2 (HABP2), transcript variant 1, mRNA [NM_004132]	3026

A_32_P79366	1.2	1.6	-3.7	1.2	HABP4	Homo sapiens hyaluronan binding protein 4 (HABP4), mRNA [NM_014282]	22927
A_24_P353964	1.0	-2.0	-1.0	-1.0	HADHA	Homo sapiens hydroxyacyl-CoA dehydrogenase/3-ketoacyl-CoA thiolase/enoyl-CoA hydratase (trifunctional protein), alpha subunit (HADHA), nuclear gene encoding mitochondrial protein, mRNA [NM_000182]	3030

A_24_P242688	3.1	3.1	3.0	2.3	HADHA	Homo sapiens hydroxyacyl-CoA dehydrogenase/3-ketoacyl-CoA thiolase/enoyl-CoA hydratase (trifunctional protein), alpha subunit (HADHA), nuclear gene encoding mitochondrial protein, mRNA [NM_000182]	3030
A_24_P176445	-1.2	-1.0	-2.8	-1.0	HAGH	Homo sapiens hydroxyacylglutathione hydrolase (HAGH), transcript variant 2, mRNA [NM_001040427]	3029
A_23_P165162	1.3	3.6	-1.4	1.2	HAMP	Homo sapiens hepcidin antimicrobial peptide (HAMP), mRNA [NM_021175]	57817

A_23_P373521	-1.5	-4.5	-3.7	-3.2	HAND2	Homo sapiens heart and neural crest derivatives expressed 2 (HAND2), mRNA [NM_021973]	9464
A_23_P22129	-5.7	1.1	-2.4	-5.2	HAP1	Homo sapiens huntingtin-associated protein 1 (HAP1), transcript variant 2, mRNA [NM_177977]	9001
A_23_P10721	1.0	1.2	-2.0	1.5	HAP1	Homo sapiens huntingtin-associated protein 1 (HAP1), transcript variant 2, mRNA [NM_177977]	9001
A_23_P430839	-4.4	1.6	-1.0	1.1	HAPLN4	Homo sapiens hyaluronan and proteoglycan link protein 4 (HAPLN4), mRNA [NM_023002]	404037

A_24_P378794	-2.1	1.1	-2.3	-1.8	HAPLN4	Homo sapiens hyaluronan and proteoglycan link protein 4 (HAPLN4), mRNA [NM_023002]	404037
A_32_P226498	-1.0	1.7	2.1	1.0	HAR1A	Homo sapiens highly accelerated region 1A (non-protein coding) (HAR1A), non-coding RNA [NR_003244]	768096
A_23_P135465	2.6	2.1	2.2	-1.4	HARBI1	Homo sapiens harbinger transposase derived 1 (HARBI1), mRNA [NM_173811]	283254
A_23_P27400	1.3	-1.2	-3.6	2.0	HAS1	Homo sapiens hyaluronan synthase 1 (HAS1), mRNA [NM_001523]	3036

A_23_P393034	1.1	1.1	-1.0	-4.3	HAS3	Homo sapiens hyaluronan synthase 3 (HAS3), transcript variant 1, mRNA [NM_005329]	3038
A_32_P221121	4.2	2.3	3.0	4.2	HAUS3	Homo sapiens cDNA FLJ52073 complete cds. [AK293948]	79441
A_24_P644742	-2.4	1.1	1.1	1.0	HAUS6	Homo sapiens HAUS augmin-like complex, subunit 6 (HAUS6), mRNA [NM_017645]	54801
A_32_P3290	-2.4	-1.0	-1.0	-1.0	HAUS6	Homo sapiens HAUS augmin-like complex, subunit 6 (HAUS6), mRNA [NM_017645]	54801

A_23_P416034	1.1	-1.4	1.3	-29.1	HAUS7	Homo sapiens HAUS augmin-like complex, subunit 7 (HAUS7), mRNA [NM_017518]	55559
A_23_P141965	-1.1	-2.7	-1.1	-1.1	HAUS8	Homo sapiens HAUS augmin-like complex, subunit 8 (HAUS8), transcript variant 1, mRNA [NM_033417]	93323
A_24_P411561	-1.1	-2.7	-1.0	-1.3	HAVCR2	Homo sapiens hepatitis A virus cellular receptor 2 (HAVCR2), mRNA [NM_032782]	84868
A_23_P115223	1.4	1.3	-1.6	-5.3	HAX1	Homo sapiens HCLS1 associated protein X-1 (HAX1), transcript variant 1, mRNA [NM_006118]	10456

A_23_P37856	6.4	4.2	2.5	1.9	HBA1	Homo sapiens hemoglobin, alpha 1 (HBA1), mRNA [NM_000558]	3039
A_23_P26457	5.8	1.3	1.5	1.4	HBA2	Homo sapiens hemoglobin, alpha 2 (HBA2), mRNA [NM_000517]	3040
A_23_P87346	2.8	2.8	2.6	2.1	HBD	Homo sapiens hemoglobin, delta (HBD), mRNA [NM_000519]	3045
A_24_P355568	3.0	1.4	1.5	1.6	HBE1	Homo sapiens hemoglobin, epsilon 1 (HBE1), mRNA [NM_005330]	3046
A_23_P64539	-3.7	-1.2	-2.0	-1.2	HBG1	Homo sapiens hemoglobin, gamma A (HBG1), mRNA [NM_000559]	3047

A_23_P215787	1.2	-3.0	1.3	1.2	HBP1	Homo sapiens HMG-box transcription factor 1 (HBP1), transcript variant 2, mRNA [NM_012257]	26959
A_24_P204971	-1.9	-8.8	-1.6	-5.1	HBP1	Homo sapiens HMG-box transcription factor 1 (HBP1), transcript variant 2, mRNA [NM_012257]	26959
A_23_P49254	-2.4	-2.0	-1.7	-3.9	HBQ1	Homo sapiens hemoglobin, theta 1 (HBQ1), mRNA [NM_005331]	3049
A_23_P3651	-1.1	1.4	2.2	1.9	HBZ	Homo sapiens hemoglobin, zeta (HBZ), mRNA [NM_005332]	3050

A_23_P436407	-1.1	3.0	1.0	2.5	HCCS	Homo sapiens holocytochrome c synthase (HCCS), transcript variant 1, mRNA [NM_005333]	3052
A_23_P257945	-2.1	-2.0	-1.6	-1.2	HCCS	Homo sapiens holocytochrome c synthase (HCCS), transcript variant 1, mRNA [NM_005333]	3052
A_24_P567952	1.4	2.1	2.3	-1.8	HCG18	Homo sapiens HLA complex group 18 (non-protein coding) (HCG18), non-coding RNA [NR_024052]	414777

A_24_P567944	2.6	1.4	-1.8	-1.1	HCG18	Homo sapiens HLA complex group 18 (non-protein coding) (HCG18), non-coding RNA [NR_024052]	414777
A_23_P254768	1.0	-3.4	1.1	1.1	HCG4	Homo sapiens HLA complex group 4 (non-protein coding) (HCG4), non-coding RNA [NR_002139]	54435
A_24_P144963	-13.3	2.1	2.1	1.5	HCG4B	Homo sapiens cDNA FLJ32165 fis, clone PLACE6000424. [AK056727]	80868

A_23_P251021	1.8	1.9	3.0	1.0	HCG9	Homo sapiens HLA complex group 9 (non-protein coding) (HCG9), non-coding RNA [NR_028032]	10255
A_23_P73429	1.7	2.1	-3.4	-1.5	HCLS1	Homo sapiens hematopoietic cell-specific Lyn substrate 1 (HCLS1), mRNA [NM_005335]	3059
A_24_P238609	1.0	2.1	1.1	1.7	HCP5	Homo sapiens HLA complex P5 (non-protein coding) (HCP5), non-coding RNA [NR_040662]	10866
A_23_P211673	-2.3	1.8	2.3	-1.0	HDAC10	histone deacetylase 10 [Source:HGNC Symbol;Acc:18128] [ENST00000475965]	83933

A_23_P122304	1.8	2.7	1.1	-3.6	HDAC2	Homo sapiens histone deacetylase 2 (HDAC2), transcript variant 1, mRNA [NM_001527]	3066
A_24_P359859	-2.6	1.6	-2.3	1.0	HDAC4	Homo sapiens histone deacetylase 4 (HDAC4), mRNA [NM_006037]	9759
A_24_P359856	-2.4	1.0	-1.0	-2.4	HDAC4	Homo sapiens histone deacetylase 4 (HDAC4), mRNA [NM_006037]	9759
A_24_P125283	-4.2	1.2	-2.5	1.1	HDAC5	Homo sapiens histone deacetylase 5 (HDAC5), transcript variant 3, mRNA [NM_001015053]	10014

A_23_P250455	1.7	-2.5	-2.3	-1.1	HDAC6	Homo sapiens histone deacetylase 6 (HDAC6), mRNA [NM_006044]	10013
A_32_P841049	1.0	-4.1	1.0	1.0	HDAC9	Homo sapiens histone deacetylase 9 (HDAC9), transcript variant 4, mRNA [NM_178423]	9734
A_24_P206317	1.0	-7.4	-1.0	-1.5	HDAC9	Homo sapiens histone deacetylase 9 (HDAC9), transcript variant 1, mRNA [NM_058176]	9734
A_23_P122796	-1.6	-2.6	-2.1	-1.2	HDDC2	Homo sapiens HD domain containing 2 (HDDC2), mRNA [NM_016063]	51020

A_24_P376707	1.1	1.0	-5.7	1.1	HDGF	Homo sapiens hepatoma-derived growth factor (HDGF), transcript variant 1, mRNA [NM_004494]	3068
A_23_P301484	2.3	4.2	3.2	2.1	HDGFL1	hepatoma derived growth factor-like 1 [Source:HGNC Symbol;Acc:21095] [ENST00000510882]	154150
A_23_P344451	-3.0	-1.3	-1.3	-1.2	HDGFRP3	Homo sapiens hepatoma-derived growth factor, related protein 3 (HDGFRP3), mRNA [NM_016073]	50810

A_23_P153098	-1.6	-1.4	-1.0	-2.2	HDHD2	Homo sapiens haloacid dehalogenase-like hydrolase domain containing 2 (HDHD2), mRNA [NM_032124]	84064
A_24_P187443	-11.9	1.1	1.5	1.2	HDLBP	Homo sapiens PRO2900 mRNA, complete cds. [AF116718]	3069
A_23_P131417	1.0	2.1	-1.0	1.6	HDLBP	Homo sapiens high density lipoprotein binding protein (HDLBP), transcript variant 1, mRNA [NM_005336]	3069
A_23_P386364	1.9	1.9	2.3	1.0	HDX	Homo sapiens highly divergent homeobox (HDX), transcript variant 2, mRNA [NM_144657]	139324

A_23_P259771	1.2	1.2	-1.0	-2.1	HEATR3	HEAT repeat containing 3 [Source:HGNC Symbol;Acc:26087] [ENST00000483299]	55027
A_24_P696470	-1.7	-1.6	-1.9	-2.1	HEATR4	Homo sapiens HEAT repeat containing 4 (HEATR4), transcript variant 2, mRNA [NM_203309]	399671
A_23_P408473	-3.9	-2.4	-3.1	-2.3	HEATR7A	Homo sapiens HEAT repeat containing 7A (HEATR7A), transcript variant 1, mRNA [NM_032450]	727957
A_23_P407080	1.0	-2.2	-1.0	-1.1	HEATR7B2	Homo sapiens HEAT repeat family member 7B2 (HEATR7B2), mRNA [NM_173489]	133558

A_23_P19691	-2.0	-1.6	-5.3	-2.1	HEBP2	Homo sapiens heme binding protein 2 (HEBP2), mRNA [NM_014320]	23593
A_23_P54006	2.4	-2.3	1.1	1.3	HECTD1	Homo sapiens HECT domain containing 1 (HECTD1), mRNA [NM_015382]	25831
A_23_P149221	-1.6	1.4	-2.6	1.5	HECTD3	Homo sapiens HECT domain containing 3 (HECTD3), mRNA [NM_024602]	79654
A_32_P166422	1.1	1.1	-1.5	-3.1	HECW1	HECT, C2 and WW domain containing E3 ubiquitin protein ligase 1 [Source:HGNC Symbol;Acc:22195] [ENST00000471527]	23072

A_23_P319027	1.0	1.0	-1.0	-3.4	HECW1	Homo sapiens HECT, C2 and WW domain containing E3 ubiquitin protein ligase 1 (HECW1), mRNA [NM_015052]	23072
A_23_P50974	-6.0	1.3	1.2	1.3	HECW2	Homo sapiens HECT, C2 and WW domain containing E3 ubiquitin protein ligase 2 (HECW2), mRNA [NM_020760]	57520
A_24_P85300	-2.8	-1.0	-1.1	-1.0	HEG1	Homo sapiens HEG homolog 1 (zebrafish) (HEG1), mRNA [NM_020733]	57493
A_23_P2293	-5.5	1.0	1.1	1.3	HELB	Homo sapiens helicase (DNA) B (HELB), mRNA [NM_033647]	92797

A_24_P273635	2.1	2.0	2.0	2.2	HELQ	Homo sapiens helicase, POLQ-like (HELQ), mRNA [NM_133636]	113510
A_23_P43412	-1.8	-1.6	-2.5	-1.2	HEMGN	Homo sapiens hemogen (HEMGN), transcript variant 1, mRNA [NM_018437]	55363
A_24_P232545	-1.1	-2.0	1.5	1.5	HEPACAM	Homo sapiens hepatic and glial cell adhesion molecule (HEPACAM), mRNA [NM_152722]	220296
A_24_P213944	1.0	-2.0	-12.2	-1.3	HEPACAM	hepatic and glial cell adhesion molecule [Source:HGNC Symbol;Acc:26361] [ENST00000298251]	220296

A_32_P118397	-2.3	1.1	1.0	-1.1	HEPACAM2	Homo sapiens HEPACAM family member 2 (HEPACAM2), transcript variant 1, mRNA [NM_001039372]	253012
A_24_P399980	1.0	-1.6	-1.5	-2.0	HEPH	Homo sapiens hephaestin (HEPH), transcript variant 2, mRNA [NM_014799]	9843
A_23_P33407	1.4	2.1	-1.6	-1.1	HERC2	Homo sapiens hect domain and RLD 2 (HERC2), mRNA [NM_004667]	8924
A_32_P211080	1.3	-1.5	-3.2	-2.7	HERC2P2	Homo sapiens hect domain and RLD 2 pseudogene 2 (HERC2P2), non-coding RNA [NR_002824]	400322

A_24_P222237	1.4	1.5	3.5	3.7	HERC2P2	Homo sapiens hect domain and RLD 2 pseudogene 2 (HERC2P2), non-coding RNA [NR_002824]	400322
A_32_P139738	1.6	-2.5	1.5	1.0	HERC2P4	Homo sapiens hect domain and RLD 2 pseudogene 4 (HERC2P4), non-coding RNA [NR_002827]	440362
A_24_P51201	-9.0	-1.1	-1.2	-1.2	HERC3	Homo sapiens hect domain and RLD 3, mRNA (cDNA clone IMAGE:6050308), complete cds. [BC038960]	8916

A_23_P121586	1.0	-1.0	-3.7	-1.1	HERC3	Homo sapiens hect domain and RLD 3 (HERC3), mRNA [NM_014606]	8916
A_23_P6596	2.2	-1.0	2.1	2.1	HES1	Homo sapiens hairy and enhancer of split 1, (Drosophila) (HES1), mRNA [NM_005524]	3280
A_23_P201687	-2.3	-1.2	-1.3	-1.3	HES2	hairy and enhancer of split 2 (Drosophila) [Source:HGNC Symbol;Acc:16005] [ENST00000377836]	54626
A_23_P149448	5.9	1.9	1.8	2.1	HES4	Homo sapiens hairy and enhancer of split 4 (Drosophila) (HES4), transcript variant 2, mRNA [NM_021170]	57801

A_32_P234184	2.0	4.8	1.5	2.0	HES5	Homo sapiens hairy and enhancer of split 5 (Drosophila) (HES5), mRNA [NM_001010926]	388585
A_23_P131202	-1.7	-1.3	-1.7	-4.2	HES6	Homo sapiens hairy and enhancer of split 6 (Drosophila) (HES6), transcript variant 1, mRNA [NM_018645]	55502
A_23_P121102	2.1	2.4	2.1	2.1	HESX1	Homo sapiens HESX homeobox 1 (HESX1), mRNA [NM_003865]	8820
A_23_P121106	-3.8	-1.0	-1.1	-1.1	HESX1	Homo sapiens HESX homeobox 1 (HESX1), mRNA [NM_003865]	8820

A_23_P156049	-3.0	-1.1	-2.0	-1.3	HEXB	Homo sapiens hexosaminidase B (beta polypeptide) (HEXB), mRNA [NM_000521]	3074
A_24_P354748	-1.2	1.1	2.3	1.0	HFE	Homo sapiens hemochromatosis (HFE), transcript variant 6, mRNA [NM_139006]	3077
A_24_P103448	-1.5	-2.0	-1.5	-1.5	HFE	Homo sapiens hemochromatosis (HFE), transcript variant 1, mRNA [NM_000410]	3077
A_24_P111996	-4.4	1.4	1.3	1.0	HFE	Homo sapiens hemochromatosis (HFE), transcript variant 1, mRNA [NM_000410]	3077

A_24_P186551	2.2	1.4	1.7	-1.2	HGC6.3	Homo sapiens uncharacterized LOC100128124 (HGC6.3), mRNA [NM_001129895]	100128124
A_23_P250164	-2.5	1.1	2.7	1.8	HGD	Homo sapiens homogentisate 1,2-dioxygenase (HGD), mRNA [NM_000187]	3081
A_23_P58108	3.5	3.5	1.1	2.9	HGFAC	Homo sapiens HGF activator (HGFAC), mRNA [NM_001528]	3083
A_23_P61230	3.1	4.6	3.6	-1.1	HGS	Homo sapiens hepatocyte growth factor-regulated tyrosine kinase substrate (HGS), mRNA [NM_004712]	9146

A_24_P125993	1.8	3.4	-1.1	1.9	HGS	Homo sapiens hepatocyte growth factor-regulated tyrosine kinase substrate (HGS), mRNA [NM_004712]	9146
A_24_P120827	2.5	1.2	1.5	3.3	HGSNAT	Homo sapiens heparan-alpha-glucosaminide N-acetyltransferase (HGSNAT), mRNA [NM_152419]	138050
A_23_P255298	-18.9	1.3	1.4	1.0	HHATL	Homo sapiens hedgehog acyltransferase-like (HHATL), transcript variant 1, mRNA [NM_020707]	57467
A_23_P47034	1.1	-2.8	-1.7	-2.0	HHEX	Homo sapiens hematopoietically expressed homeobox (HHEX), mRNA [NM_002729]	3087

A_24_P162254	1.8	-1.2	2.1	-1.2	HHIPL1	HHIP-like 1 [Source:HGNC Symbol;Acc:19710] [ENST00000330710]	84439
A_24_P127491	-2.2	-2.3	-1.3	-1.9	HHIPL1	Homo sapiens HHIP-like 1 (HHIPL1), transcript variant 1, mRNA [NM_001127258]	84439
A_23_P86059	-1.4	-1.5	-1.6	-2.0	HHIPL2	Homo sapiens HHIP-like 2 (HHIPL2), mRNA [NM_024746]	79802
A_23_P157556	-1.2	2.9	-1.8	-1.0	HHLA1	Homo sapiens HERV-H LTR-associating 1 (HHLA1), mRNA [NM_001145095]	10086
A_24_P388033	19.3	17.1	13.5	19.5	HIAT1	Homo sapiens hippocampus abundant transcript 1 (HIAT1), mRNA [NM_033055]	64645

A_23_P33187	-1.6	-4.2	-1.4	-1.1	HIATL1	Homo sapiens hippocampus abundant transcript-like 1 (HIATL1), mRNA [NM_032558]	84641
A_23_P399169	1.8	2.2	1.1	1.6	HIC1	Homo sapiens hypermethylated in cancer 1 (HIC1), transcript variant 1, mRNA [NM_006497]	3090
A_23_P129856	2.0	1.4	2.6	1.2	HIC1	Homo sapiens hypermethylated in cancer 1 (HIC1), transcript variant 1, mRNA [NM_006497]	3090
A_23_P383132	1.0	-1.0	-1.0	-6.8	HIC2	Homo sapiens hypermethylated in cancer 2 (HIC2), mRNA [NM_015094]	23119

A_23_P304386	-1.5	-1.0	-1.1	-2.4	HIGD2A	Homo sapiens HIG1 hypoxia inducible domain family, member 2A (HIGD2A), mRNA [NM_138820]	192286
A_24_P144784	-1.3	1.1	1.4	2.1	HILS1	Homo sapiens histone linker H1 domain, spermatid-specific 1 (HILS1), transcript variant 1, non-coding RNA [NR_024193]	373861
A_23_P13176	-2.2	1.0	-1.4	1.0	HINFP	Homo sapiens histone H4 transcription factor (HINFP), transcript variant 1, mRNA [NM_015517]	25988

A_23_P157784	-2.1	1.1	-1.2	-2.3	HINT2	Homo sapiens histidine triad nucleotide binding protein 2 (HINT2), nuclear gene encoding mitochondrial protein, mRNA [NM_032593]	84681
A_24_P916314	-1.5	-2.4	-2.9	1.2	HIP1	Homo sapiens huntingtin interacting protein 1 (HIP1), transcript variant 1, mRNA [NM_005338]	3092
A_23_P398294	1.0	-1.0	2.2	-1.0	HIP1R	Homo sapiens huntingtin interacting protein 1 related (HIP1R), mRNA [NM_003959]	9026

A_24_P385336	2.6	-5.3	-2.9	1.4	HIPK1	Homo sapiens homeodomain interacting protein kinase 1 (HIPK1), transcript variant 2, mRNA [NM_152696]	204851
A_24_P681011	-6.7	1.6	-2.0	-1.1	HIPK2	Homo sapiens homeodomain interacting protein kinase 2 (HIPK2), transcript variant 1, mRNA [NM_022740]	28996
A_23_P169756	-1.0	3.6	-1.1	-1.1	HIPK2	Homo sapiens homeodomain interacting protein kinase 2 (HIPK2), transcript variant 1, mRNA [NM_022740]	28996

A_24_P500621	-1.0	-1.0	-1.1	-3.6	HIPK2	Homo sapiens homeodomain interacting protein kinase 2 (HIPK2), transcript variant 1, mRNA [NM_022740]	28996
A_24_P364072	1.0	1.0	-2.7	-3.8	HIPK3	homeodomain interacting protein kinase 3 [Source:HGNC Symbol;Acc:4915] [ENST00000303296]	10114
A_23_P422809	3.0	2.9	2.1	2.9	HIPK3	Homo sapiens homeodomain interacting protein kinase 3 (HIPK3), transcript variant 1, mRNA [NM_005734]	10114

A_23_P329945	2.5	2.4	1.3	-1.4	HIPK4	Homo sapiens homeodomain interacting protein kinase 4 (HIPK4), mRNA [NM_144685]	147746
A_24_P142269	1.1	1.1	-2.6	1.0	HIRIP3	Homo sapiens HIRA interacting protein 3 (HIRIP3), transcript variant 1, mRNA [NM_003609]	8479
A_23_P250385	1.2	2.6	1.1	-1.2	HIST1H1B	Homo sapiens histone cluster 1, H1b (HIST1H1B), mRNA [NM_005322]	3009
A_23_P122443	-1.8	-3.4	2.0	-1.8	HIST1H1C	Homo sapiens histone cluster 1, H1c (HIST1H1C), mRNA [NM_005319]	3006

A_24_P260639	2.4	2.0	-1.3	2.4	HIST1H1D	Homo sapiens histone cluster 1, H1d (HIST1H1D), mRNA [NM_005320]	3007
A_23_P7976	1.6	3.6	2.7	2.9	HIST1H1E	Homo sapiens histone cluster 1, H1e (HIST1H1E), mRNA [NM_005321]	3008
A_23_P133842	-1.0	-6.3	-1.7	-1.0	HIST1H1T	Homo sapiens histone cluster 1, H1t (HIST1H1T), mRNA [NM_005323]	3010
A_23_P59045	1.1	1.6	-1.6	2.3	HIST1H2AE	Homo sapiens histone cluster 1, H2ae (HIST1H2AE), mRNA [NM_021052]	3012
A_24_P394510	-1.3	-1.4	-2.3	-1.4	HIST1H2AJ	Homo sapiens histone cluster 1, H2aj (HIST1H2AJ), mRNA [NM_021066]	8331

A_24_P940441	-2.2	1.4	-1.9	1.0	HIST1H2AK	Homo sapiens histone cluster 1, H2ak, mRNA (cDNA clone MGC:26490 IMAGE:4825623), complete cds. [BC034487]	8330
A_23_P363174	-2.9	-7.6	-8.2	-5.2	HIST1H2AL	Homo sapiens histone cluster 1, H2al (HIST1H2AL), mRNA [NM_003511]	8332
A_23_P111054	-2.2	-1.9	-1.3	-6.3	HIST1H2BB	Homo sapiens histone cluster 1, H2bb (HIST1H2BB), mRNA [NM_021062]	3018
A_23_P93180	-1.3	-1.9	-2.7	-1.5	HIST1H2BC	Homo sapiens histone cluster 1, H2bc (HIST1H2BC), mRNA [NM_003526]	8347

A_23_P167997	2.6	1.8	1.4	1.8	HIST1H2BG	Homo sapiens histone cluster 1, H2bg (HIST1H2BG), mRNA [NM_003518]	8339
A_23_P145238	1.3	2.2	2.2	-1.2	HIST1H2BK	Homo sapiens histone cluster 1, H2bk (HIST1H2BK), mRNA [NM_080593]	85236
A_24_P174924	-1.2	3.1	1.6	3.0	HIST1H3B	Homo sapiens histone cluster 1, H3b (HIST1H3B), mRNA [NM_003537]	8358
A_23_P70445	-2.1	-1.9	-2.7	-1.6	HIST1H3E	Homo sapiens histone cluster 1, H3e (HIST1H3E), mRNA [NM_003532]	8353

A_23_P8004	-1.9	-1.9	-2.1	-1.9	HIST1H3F	Homo sapiens histone cluster 1, H3f, mRNA (cDNA clone IMAGE:3162462), with apparent retained intron. [BC062305]	8968
A_24_P97914	-4.6	-2.4	-2.4	-2.4	HIST1H3F	Homo sapiens histone cluster 1, H3f, mRNA (cDNA clone IMAGE:3162462), with apparent retained intron. [BC062305]	8968
A_23_P30799	1.8	2.5	-1.9	-2.0	HIST1H3F	Homo sapiens histone cluster 1, H3f (HIST1H3F), mRNA [NM_021018]	8968

A_32_P794894	1.1	1.2	-1.1	-2.2	HIST1H3G	Homo sapiens histone cluster 1, H3g, mRNA (cDNA clone IMAGE:4563941). [BC012185]	8355
A_23_P333484	-1.5	-1.5	-3.4	-1.6	HIST1H3H	Homo sapiens histone cluster 1, H3h (HIST1H3H), mRNA [NM_003536]	8357
A_23_P395374	2.8	2.1	1.6	-1.1	HIST1H4D	Homo sapiens histone cluster 1, H4d (HIST1H4D), mRNA [NM_003539]	8360
A_23_P415411	9.9	5.3	5.5	9.6	HIST1H4E	Homo sapiens histone cluster 1, H4e (HIST1H4E), mRNA [NM_003545]	8367
A_23_P359540	-2.0	-1.2	-3.5	-2.7	HIST1H4F	Homo sapiens histone cluster 1, H4f (HIST1H4F), mRNA [NM_003540]	8361

A_23_P323685	-1.6	-1.2	-2.0	-1.3	HIST1H4H	Homo sapiens histone cluster 1, H4h (HIST1H4H), mRNA [NM_003543]	8365
A_23_P30805	-1.8	-1.4	-2.2	-1.6	HIST1H4J	Homo sapiens histone cluster 1, H4j (HIST1H4J), mRNA [NM_021968]	8363
A_23_P70480	1.8	1.0	-2.6	-1.3	HIST1H4L	Homo sapiens histone cluster 1, H4l (HIST1H4L), mRNA [NM_003546]	8368
A_23_P309381	-1.0	-1.0	-1.0	2.7	HIST2H2AA4	Homo sapiens histone cluster 2, H2aa4 (HIST2H2AA4), mRNA [NM_001040874]	723790
A_23_P343927	-1.1	-3.0	-2.8	-2.8	HIST2H2AB	Homo sapiens histone cluster 2, H2ab (HIST2H2AB), mRNA [NM_175065]	317772

A_23_P19619	-37.5	-1.3	-1.4	-1.4	HIVEP1	Homo sapiens human immunodeficiency virus type I enhancer binding protein 1 (HIVEP1), mRNA [NM_002114]	3096
A_23_P35330	-1.5	3.0	1.9	1.5	HIVEP3	Homo sapiens human immunodeficiency virus type I enhancer binding protein 3 (HIVEP3), transcript variant 1, mRNA [NM_024503]	59269
A_24_P876734	-1.4	-1.0	-2.5	-2.8	HIVEP3	Homo sapiens human immunodeficiency virus type I enhancer binding protein 3 (HIVEP3), transcript variant 3, non-coding RNA [NR_038260]	59269

A_23_P398460	-2.1	-1.3	1.1	1.3	HK2	Homo sapiens hexokinase 2 (HK2), mRNA [NM_000189]	3099
A_24_P37962	1.6	-2.9	-1.5	-2.0	HK3	Homo sapiens hexokinase 3 (white cell) (HK3), nuclear gene encoding mitochondrial protein, mRNA [NM_002115]	3101
A_23_P213584	-2.1	-1.1	-2.0	1.2	HK3	Homo sapiens hexokinase 3 (white cell) (HK3), nuclear gene encoding mitochondrial protein, mRNA [NM_002115]	3101
A_23_P202427	-1.1	-8.1	-1.2	-1.2	HKDC1	Homo sapiens hexokinase domain containing 1 (HKDC1), mRNA [NM_025130]	80201

A_24_P376483	2.1	-1.4	-1.2	-1.0	HLA-A	Homo sapiens major histocompatibility complex, class I, A (HLA-A), transcript variant 1, mRNA [NM_002116]	3105
A_23_P125107	3.8	4.3	1.1	-1.3	HLA-B	Homo sapiens major histocompatibility complex, class I, B (HLA-B), mRNA [NM_005514]	3106
A_23_P113716	1.6	2.0	2.2	1.1	HLA-C	major histocompatibility complex, class I, C [Source:HGNC Symbol;Acc:4933] [ENST00000552865]	3107
A_23_P95917	2.2	1.9	2.3	1.7	HLA-C	major histocompatibility complex, class I, C [Source:HGNC Symbol;Acc:4933] [ENST00000429840]	3107

A_24_P298409	-3.0	-1.2	-1.1	1.1	HLA-C	Homo sapiens major histocompatibility complex, class I, C (HLA-C), transcript variant 1, mRNA [NM_002117]	3107
A_32_P356316	-2.2	-1.0	-1.2	-1.0	HLA-DOA	Homo sapiens major histocompatibility complex, class II, DO alpha (HLA-DOA), mRNA [NM_002119]	3111
A_24_P354800	6.6	11.3	2.6	4.6	HLA-DOA	Homo sapiens major histocompatibility complex, class II, DO alpha (HLA-DOA), mRNA [NM_002119]	3111

A_23_P30927	1.0	2.0	-1.6	-1.0	HLA-DOA	Homo sapiens major histocompatibility complex, class II, DO alpha (HLA-DOA), mRNA [NM_002119]	3111
A_23_P30736	1.9	3.9	1.3	1.3	HLA-DOB	Homo sapiens major histocompatibility complex, class II, DO beta (HLA-DOB), mRNA [NM_002120]	3112
A_23_P30913	1.5	-2.3	-1.7	-2.6	HLA-DPA1	Homo sapiens major histocompatibility complex, class II, DP alpha 1 (HLA-DPA1), transcript variant 1, mRNA [NM_033554]	3113

A_24_P288836	-1.1	1.4	-2.3	-1.6	HLA-DPB2	Homo sapiens major histocompatibility complex, class II, DP beta 2 (pseudogene) (HLA-DPB2), non-coding RNA [NR_001435]	3116
A_24_P196827	1.6	2.2	-1.0	1.8	HLA-DQA1	Homo sapiens major histocompatibility complex, class II, DQ alpha 1 (HLA-DQA1), mRNA [NM_002122]	3117
A_24_P852756	-1.4	-1.3	-3.6	-1.8	HLA-DQA2	Homo sapiens major histocompatibility complex, class II, DQ alpha 2 (HLA-DQA2), mRNA [NM_020056]	3118

A_23_P42302	-1.0	-9.5	-1.0	-1.0	HLA-DQA2	Homo sapiens major histocompatibility complex, class II, DQ alpha 2 (HLA-DQA2), mRNA [NM_020056]	3118
A_23_P8108	-3.6	1.1	1.9	1.0	HLA-DQB1	Homo sapiens major histocompatibility complex, class II, DQ beta 1 (HLA-DQB1), transcript variant 3, mRNA [NM_001243962]	3119
A_24_P239676	-4.0	-1.1	1.1	-1.7	HLA-DQB1	Homo sapiens major histocompatibility complex, class II, DQ beta 1 (HLA-DQB1), transcript variant 1, mRNA [NM_002123]	3119

A_23_P19510	-7.1	1.4	-2.7	1.5	HLA-DQB2	Homo sapiens major histocompatibility complex, class II, DQ beta 2 (HLA-DQB2), mRNA [NM_001198858]	3120
A_24_P343233	1.9	2.3	1.5	1.7	HLA-DRB1	Homo sapiens major histocompatibility complex, class II, DR beta 1 (HLA-DRB1), transcript variant 1, mRNA [NM_002124]	3123
A_23_P31006	-7.9	-1.1	-1.7	-1.5	HLA-DRB5	Homo sapiens major histocompatibility complex, class II, DR beta 5 (HLA-DRB5), mRNA [NM_002125]	3127

A_24_P326082	1.5	-2.9	1.1	1.4	HLA-E	Homo sapiens major histocompatibility complex, class I, E (HLA-E), mRNA [NM_005516]	3133
A_32_P460973	2.7	4.3	2.5	2.6	HLA-E	Homo sapiens major histocompatibility complex, class I, E (HLA-E), mRNA [NM_005516]	3133
A_23_P145264	-1.2	-3.5	-5.1	-1.9	HLA-F	Homo sapiens major histocompatibility complex, class I, F (HLA-F), transcript variant 2, mRNA [NM_018950]	3134

A_32_P76811	2.5	1.9	1.4	-1.0	HLA-F-AS1	Homo sapiens HLA-F antisense RNA 1 (non-protein coding) (HLA-F-AS1), transcript variant 1, non-coding RNA [NR_026972]	285830
A_23_P356585	3.1	2.1	3.1	1.3	HLF	Homo sapiens hepatic leukemia factor (HLF), mRNA [NM_002126]	3131
A_23_P126266	-9.0	1.1	1.3	1.1	HLX	Homo sapiens H2.0-like homeobox (HLX), mRNA [NM_021958]	3142
A_23_P134684	1.5	3.4	1.8	2.2	HMBOX1	Homo sapiens homeobox containing 1 (HMBOX1), transcript variant 1, mRNA [NM_024567]	79618

A_24_P932736	4.0	3.9	1.7	2.1	HMBOX1	homeobox containing 1 [Source:HGNC Symbol;Acc:26137] [ENST00000397358]	79618
A_24_P222043	47.5	4.1	10.3	12.8	HMGA1	Homo sapiens high mobility group AT-hook 1 (HMGA1), transcript variant 2, mRNA [NM_002131]	3159
A_23_P95930	-4.8	-1.1	-1.0	-1.1	HMGA2	Homo sapiens high mobility group AT-hook 2 (HMGA2), transcript variant 1, mRNA [NM_003483]	8091
A_23_P99985	1.3	2.2	2.2	1.3	HMGB1	Homo sapiens high mobility group box 1 (HMGB1), mRNA [NM_002128]	3146

A_23_P133263	-1.1	-2.2	-1.1	-1.1	HMGCS1	Homo sapiens 3-hydroxy-3-methylglutaryl-CoA synthase 1 (soluble) (HMGCS1), transcript variant 2, mRNA [NM_002130]	3157
A_32_P22263	1.1	-2.1	-1.1	-1.5	HMGN1	Homo sapiens high mobility group nucleosome binding domain 1 (HMGN1), mRNA [NM_004965]	3150
A_32_P29507	-1.0	-1.0	-1.7	-2.1	HMGN2	Homo sapiens high mobility group nucleosomal binding domain 2 (HMGN2), mRNA [NM_005517]	3151

A_32_P1381	3.7	-1.1	1.1	-1.3	HMGN2	Human non-histone chromosomal protein HMG-17 mRNA, complete cds. [M12623]	3151
A_24_P132099	-8.0	1.0	-1.2	1.1	HMGN3	Homo sapiens high mobility group nucleosomal binding domain 3 (HMGN3), transcript variant 2, mRNA [NM_138730]	9324
A_23_P19389	2.1	-2.2	1.7	-1.0	HMGN4	Homo sapiens high mobility group nucleosomal binding domain 4 (HMGN4), mRNA [NM_006353]	10473

A_24_P397515	-1.3	-4.7	-1.5	-1.5	HMGN4	Homo sapiens high mobility group nucleosomal binding domain 4 (HMGN4), mRNA [NM_006353]	10473
A_32_P444460	-2.4	1.1	1.1	1.0	HMGN5	Homo sapiens high mobility group nucleosome binding domain 5 (HMGN5), mRNA [NM_030763]	79366
A_23_P319005	-1.9	-1.6	-1.7	-15.1	HMP19	Homo sapiens HMP19 protein (HMP19), mRNA [NM_015980]	51617
A_23_P434900	-1.1	-1.7	-1.7	-5.8	HN1L	Homo sapiens hematological and neurological expressed 1-like (HN1L), mRNA [NM_144570]	90861

A_23_P36757	1.7	2.1	-1.4	2.6	HNF1A	Homo sapiens HNF1 homeobox A (HNF1A), mRNA [NM_000545]	6927
A_24_P372913	1.0	2.1	1.5	1.8	HNF1A	Homo sapiens HNF1 homeobox A (HNF1A), mRNA [NM_000545]	6927
A_23_P207557	3.1	2.2	1.1	1.7	HNF1B	HNF1 homeobox B [Source:HGNC Symbol;Acc:11630] [ENST00000459669]	6928
A_23_P409287	-2.6	1.4	1.3	1.0	HNF1B	Homo sapiens HNF1 homeobox B (HNF1B), transcript variant 1, mRNA [NM_000458]	6928

A_24_P10751	-1.3	2.3	1.5	1.1	HNF4A	Homo sapiens hepatocyte nuclear factor 4, alpha (HNF4A), transcript variant 2, mRNA [NM_000457]	3172
A_24_P915806	1.7	-2.8	1.6	2.1	HNMT	Homo sapiens histamine N-methyltransferase (HNMT), transcript variant 2, mRNA [NM_001024074]	3176
A_24_P371670	-1.7	-3.0	-1.7	-3.1	HNRNPA0	Homo sapiens heterogeneous nuclear ribonucleoprotein A0 (HNRNPA0), mRNA [NM_006805]	10949
A_23_P252449	-1.3	4.2	-2.8	-1.3	HNRNPA0	Homo sapiens heterogeneous nuclear ribonucleoprotein A0 (HNRNPA0), mRNA [NM_006805]	10949

A_24_P454313	1.0	1.0	-2.9	1.0	HNRNPA1	Homo sapiens heterogeneous nuclear ribonucleoprotein A1 (HNRNPA1), transcript variant 2, mRNA [NM_031157]	3178
A_32_P168202	-5.1	1.0	1.0	1.0	HNRNPA1L2	Homo sapiens heterogeneous nuclear ribonucleoprotein A1-like 2 (HNRNPA1L2), transcript variant 1, mRNA [NM_001011724]	144983
A_32_P216426	1.8	2.4	2.1	1.4	HNRNPA1L2	Homo sapiens heterogeneous nuclear ribonucleoprotein A1-like 2 (HNRNPA1L2), transcript variant 1, mRNA [NM_001011724]	144983

A_24_P3973	-2.0	-1.7	-1.8	2.9	HNRNPA2B1	Homo sapiens heterogeneous nuclear ribonucleoprotein A2/B1 (HNRNPA2B1), transcript variant A2, mRNA [NM_002137]	3181
A_24_P488588	1.9	-1.6	2.7	2.0	HNRNPA3	Homo sapiens heterogeneous nuclear ribonucleoprotein A3 (HNRNPA3), mRNA [NM_194247]	220988
A_24_P706314	-3.6	-1.1	-1.2	-1.9	HNRNPA3	Homo sapiens heterogeneous nuclear ribonucleoprotein A3 (HNRNPA3), mRNA [NM_194247]	220988

A_32_P142028	1.1	1.0	-1.0	-2.2	HNRNPC	Homo sapiens heterogeneous nuclear ribonucleoprotein C (C1/C2) (HNRNPC), transcript variant 1, mRNA [NM_031314]	3183
A_24_P178423	2.4	1.1	-1.0	1.3	HNRNPC	Homo sapiens heterogeneous nuclear ribonucleoprotein C (C1/C2) (HNRNPC), transcript variant 1, mRNA [NM_031314]	3183
A_24_P767422	-1.9	-2.3	-2.0	-1.2	HNRNPCL1	Homo sapiens heterogeneous nuclear ribonucleoprotein C-like 1 (HNRNPCL1), mRNA [NM_001013631]	343069

A_24_P226037	2.7	2.0	-1.1	-1.7	HNRNPD	Homo sapiens heterogeneous nuclear ribonucleoprotein D (AU-rich element RNA binding protein 1, 37kDa) (HNRNPD), transcript variant 3, mRNA [NM_002138]	3184
A_24_P337380	-2.5	-4.4	-2.2	-1.4	HNRNPH1	Homo sapiens heterogeneous nuclear ribonucleoprotein H1 (H) (HNRNPH1), mRNA [NM_005520]	3187
A_24_P338187	2.1	1.5	1.0	-1.2	HNRNPH3	Homo sapiens heterogeneous nuclear ribonucleoprotein H3 (2H9) (HNRNPH3), transcript variant 2H9, mRNA [NM_012207]	3189

A_23_P119677	1.7	2.2	2.0	2.1	HNRNPL	Homo sapiens heterogeneous nuclear ribonucleoprotein L (HNRNPL), transcript variant 1, mRNA [NM_001533]	3191
A_32_P109572	-1.0	1.6	-3.8	-1.0	HNRNPL	Homo sapiens heterogeneous nuclear ribonucleoprotein L (HNRNPL), transcript variant 1, mRNA [NM_001533]	3191
A_24_P724984	1.1	2.3	2.2	1.8	HNRNPR	heterogeneous nuclear ribonucleoprotein R [Source:HGNC Symbol;Acc:5047] [ENST00000478691]	10236

A_23_P502612	-6.5	1.0	-1.0	1.1	HNRNPUL1	Homo sapiens heterogeneous nuclear ribonucleoprotein U-like 1 (HNRNPUL1), transcript variant 1, mRNA [NM_007040]	11100
A_24_P108351	-1.0	-1.2	-2.4	-1.2	HNRNPUL1	Homo sapiens heterogeneous nuclear ribonucleoprotein U-like 1 (HNRNPUL1), transcript variant 1, mRNA [NM_007040]	11100
A_23_P213153	2.3	1.5	2.1	1.9	HNRPDL	Homo sapiens heterogeneous nuclear ribonucleoprotein D-like (HNRPDL), transcript variant 2, mRNA [NM_031372]	9987

A_24_P9090	-2.1	1.0	1.1	-20.8	HNRPDL	Homo sapiens heterogeneous nuclear ribonucleoprotein D-like (HNRPDL), transcript variant 2, mRNA [NM_031372]	9987
A_24_P234214	9.5	1.0	1.0	1.0	HNRPLL	Homo sapiens heterogeneous nuclear ribonucleoprotein L-like (HNRPLL), transcript variant 1, mRNA [NM_138394]	92906
A_23_P364613	-1.9	-3.5	-1.1	-1.1	HOGA1	Homo sapiens 4-hydroxy-2-oxoglutarate aldolase 1 (HOGA1), nuclear gene encoding mitochondrial protein, transcript variant 1, mRNA [NM_138413]	112817

A_23_P99906	6.9	10.2	7.0	5.0	HOMER2	Homo sapiens homer homolog 2 (Drosophila) (HOMER2), transcript variant 2, mRNA [NM_199330]	9455
A_23_P39364	-1.1	-1.1	-3.5	-1.1	HOMER3	Homo sapiens homer homolog 3 (Drosophila) (HOMER3), transcript variant 2, mRNA [NM_004838]	9454
A_24_P40594	1.4	-5.8	-1.0	-1.0	HOMEZ	Homo sapiens homeobox and leucine zipper encoding (HOMEZ), mRNA [NM_020834]	57594

A_23_P114879	-1.0	-1.0	-1.1	-2.3	HOOK1	Homo sapiens hook homolog 1 (Drosophila) (HOOK1), mRNA [NM_015888]	51361
A_23_P153441	3.4	-1.2	3.0	2.2	HOOK2	Homo sapiens hook homolog 2 (Drosophila) (HOOK2), transcript variant 1, mRNA [NM_013312]	29911
A_23_P314216	-1.0	1.2	-3.9	-1.1	HOOK3	Homo sapiens hook homolog 3 (Drosophila) (HOOK3), mRNA [NM_032410]	84376
A_32_P49211	-2.7	-1.2	-1.0	-1.0	HOOK3	Homo sapiens hook homolog 3 (Drosophila) (HOOK3), mRNA [NM_032410]	84376

A_24_P913146	1.0	1.3	-3.0	1.0	HOPX	Homo sapiens HOP homeobox (HOPX), transcript variant 2, mRNA [NM_139211]	84525
A_23_P428366	-1.2	-2.4	1.3	-1.3	HORMAD2	Homo sapiens HORMA domain containing 2 (HORMAD2), mRNA [NM_152510]	150280
A_23_P145752	1.0	-3.5	-1.0	-1.1	HOXA1	Homo sapiens homeobox A1 (HOXA1), transcript variant 1, mRNA [NM_005522]	3198
A_23_P253368	-4.2	1.0	1.1	-1.2	HOXA10	Homo sapiens homeobox A10 (HOXA10), transcript variant 1, mRNA [NM_018951]	3206
A_23_P389281	-2.9	-1.9	-1.8	1.2	HOXA13	Homo sapiens homeobox A13 (HOXA13), mRNA [NM_000522]	3209

A_23_P31306	1.4	-1.1	1.2	-2.4	HOXA13	Homo sapiens homeobox A13 (HOXA13), mRNA [NM_000522]	3209
A_24_P200854	-1.7	1.8	1.9	-2.6	HOXA2	Homo sapiens homeobox A2 (HOXA2), mRNA [NM_006735]	3199
A_23_P501538	1.1	-1.1	-3.7	1.7	HOXA3	Homo sapiens homeobox A3 (HOXA3), transcript variant 2, mRNA [NM_153631]	3200
A_23_P70968	-7.4	-1.0	-1.1	-1.0	HOXA7	Homo sapiens homeobox A7 (HOXA7), mRNA [NM_006896]	3204
A_23_P4212	9.0	2.8	4.8	2.8	HOXB13	Homo sapiens homeobox B13 (HOXB13), mRNA [NM_006361]	10481

A_24_P365015	1.6	1.3	2.5	1.0	HOXB13	Homo sapiens homeobox B13 (HOXB13), mRNA [NM_006361]	10481
A_23_P316511	-5.8	-1.5	-4.2	-3.6	HOXB3	Homo sapiens homeobox B3 (HOXB3), mRNA [NM_002146]	3213
A_23_P100872	-1.9	-1.3	1.0	-2.9	HOXB3	Homo sapiens homeobox B3 (HOXB3), mRNA [NM_002146]	3213
A_24_P305067	-1.4	-2.8	-1.6	-1.6	HOXB4	Homo sapiens homeobox B4 (HOXB4), mRNA [NM_024015]	3214
A_23_P363316	-1.9	2.1	2.1	1.7	HOXB5	Homo sapiens homeobox B5 (HOXB5), mRNA [NM_002147]	3215
A_23_P49810	1.1	1.6	-2.3	-1.6	HOXB7	Homo sapiens homeobox B7 (HOXB7), mRNA [NM_004502]	3217

A_24_P218805	-3.0	1.2	1.5	-1.0	HOXC10	Homo sapiens homeobox C10 (HOXC10), mRNA [NM_017409]	3226
A_23_P36546	-1.7	1.1	-1.6	-2.3	HOXC10	Homo sapiens homeobox C10 (HOXC10), mRNA [NM_017409]	3226
A_23_P150974	-8.1	1.3	-3.9	1.0	HOXC4	Homo sapiens homeobox C4 (HOXC4), transcript variant 1, mRNA [NM_014620]	3221
A_23_P162355	-1.5	-6.0	1.1	-1.1	HOXC5	Homo sapiens homeobox C5 (HOXC5), transcript variant 1, mRNA [NM_018953]	3222
A_23_P2344	-2.5	1.4	-1.5	1.2	HOXC6	Homo sapiens homeobox C6 (HOXC6), transcript variant 2, mRNA [NM_153693]	3223

A_23_P120243	-2.6	1.3	1.3	1.1	HOXD1	Homo sapiens homeobox D1 (HOXD1), mRNA [NM_024501]	3231
A_23_P143029	1.5	1.7	-3.4	-1.4	HOXD11	Homo sapiens homeobox D11 (HOXD11), mRNA [NM_021192]	3237
A_23_P165796	1.3	-1.7	2.2	1.0	HOXD13	Homo sapiens homeobox D13 (HOXD13), mRNA [NM_000523]	3239
A_32_P211117	-10.4	3.1	2.8	2.2	HOXD3	homeobox D3 [Source:HGNC Symbol;Acc:5137] [ENST00000459979]	3232
A_23_P429383	1.9	2.0	2.3	-1.0	HOXD9	Homo sapiens homeobox D9 (HOXD9), mRNA [NM_014213]	3235

A_23_P137630	1.5	1.8	-2.4	1.2	HP1BP3	Homo sapiens heterochromatin protein 1, binding protein 3 (HP1BP3), mRNA [NM_016287]	50809
A_23_P5831	1.4	1.1	2.0	1.1	HPCAL1	Homo sapiens hippocalcin-like 1 (HPCAL1), transcript variant 2, mRNA [NM_134421]	3241
A_24_P139665	-1.1	-2.5	1.1	1.0	HPCAL4	Homo sapiens hippocalcin like 4 (HPCAL4), mRNA [NM_016257]	51440
A_23_P213050	-1.1	-1.1	-1.1	-3.1	HPGD	Homo sapiens hydroxyprostaglandin dehydrogenase 15-(NAD) (HPGD), transcript variant 1, mRNA [NM_000860]	3248

A_23_P10506	3.2	-2.1	2.9	-1.0	HPGDS	Homo sapiens hematopoietic prostaglandin D synthase (HPGDS), mRNA [NM_014485]	27306
A_23_P101806	2.8	2.1	-1.5	2.6	HPN	Homo sapiens hepsin (HPN), transcript variant 1, mRNA [NM_182983]	3249
A_24_P415327	-1.1	3.3	-1.2	1.6	HPS1	Homo sapiens Hermansky-Pudlak syndrome 1 (HPS1), transcript variant 1, mRNA [NM_000195]	3257
A_23_P40817	1.1	-2.0	1.1	1.1	HPS3	Homo sapiens Hermansky-Pudlak syndrome 3 (HPS3), mRNA [NM_032383]	84343

A_23_P98631	1.0	-1.0	-3.1	1.0	HPS5	Homo sapiens Hermansky-Pudlak syndrome 5 (HPS5), transcript variant 1, mRNA [NM_181507]	11234
A_23_P161998	-12.2	1.2	-1.4	-1.3	HPX	Homo sapiens hemopexin (HPX), mRNA [NM_000613]	3263
A_24_P911288	4.5	-1.4	2.6	2.3	HPX-2	H.sapiens HPX-2 mRNA. [X74861]	652764
A_23_P256131	-4.0	-3.0	-2.8	-1.8	HR	Homo sapiens hairless homolog (mouse) (HR), transcript variant 1, mRNA [NM_005144]	55806
A_24_P585676	1.0	-2.2	-1.5	-1.2	HR44	H.sapiens mRNA for Hr44 protein. [X91103]	27251
A_23_P57658	2.8	1.9	2.3	2.8	HRASLS	Homo sapiens HRAS-like suppressor (HRASLS), mRNA [NM_020386]	57110

A_24_P364263	1.0	1.3	-3.6	-1.0	HRASLS2	Homo sapiens HRAS-like suppressor 2 (HRASLS2), mRNA [NM_017878]	54979
A_23_P416314	-4.5	-3.3	-1.1	1.0	HRASLS5	Homo sapiens HRAS-like suppressor family, member 5 (HRASLS5), transcript variant 1, mRNA [NM_054108]	117245
A_23_P53018	1.4	-1.8	-2.9	-1.1	HRASLS5	Homo sapiens HRAS-like suppressor family, member 5 (HRASLS5), transcript variant 1, mRNA [NM_054108]	117245
A_23_P142125	2.5	1.2	2.8	2.2	HRC	Homo sapiens histidine rich calcium binding protein (HRC), mRNA [NM_002152]	3270

A_23_P7535	-3.4	1.0	-3.9	-1.0	HRH2	Homo sapiens histamine receptor H2 (HRH2), transcript variant 2, mRNA [NM_022304]	3274
A_24_P301837	-1.9	1.1	-1.9	2.7	HRH3	Homo sapiens histamine receptor H3 (HRH3), mRNA [NM_007232]	11255
A_23_P25194	1.7	1.2	-1.6	-3.3	HRK	Homo sapiens harakiri, BCL2 interacting protein (contains only BH3 domain) (HRK), mRNA [NM_003806]	8739
A_23_P134714	1.4	2.9	-1.4	2.6	HRSP12	Homo sapiens heat-responsive protein 12 (HRSP12), mRNA [NM_005836]	10247

A_24_P139943	3.0	3.8	3.8	3.7	HS1BP3	Homo sapiens HCLS1 binding protein 3 (HS1BP3), mRNA [NM_022460]	64342
A_23_P200109	1.1	-2.7	1.5	-5.2	HS2ST1	Homo sapiens heparan sulfate 2-O-sulfotransferase 1 (HS2ST1), transcript variant 1, mRNA [NM_012262]	9653
A_23_P118158	-2.4	-2.5	-2.9	-4.4	HS3ST2	Homo sapiens heparan sulfate (glucosamine) 3-O-sulfotransferase 2 (HS3ST2), mRNA [NM_006043]	9956
A_23_P77918	1.0	-1.0	2.2	-1.1	HS3ST3B1	Homo sapiens heparan sulfate (glucosamine) 3-O-sulfotransferase 3B1 (HS3ST3B1), mRNA [NM_006041]	9953

A_23_P9711	-1.0	-1.0	-7.4	-1.1	HS3ST4	Homo sapiens heparan sulfate (glucosamine) 3-O-sulfotransferase 4 (HS3ST4), mRNA [NM_006040]	9951
A_24_P58233	-1.0	-2.2	-2.4	-1.0	HS6ST2	Homo sapiens heparan sulfate 6-O-sulfotransferase 2 (HS6ST2), transcript variant L, mRNA [NM_001077188]	90161
A_32_P46981	8.1	7.8	7.2	7.2	HSBP1L1	Homo sapiens heat shock factor binding protein 1-like 1 (HSBP1L1), mRNA [NM_001136180]	440498
A_24_P683905	-1.2	-1.3	-1.4	-2.3	HSBP1L1	Homo sapiens cDNA FLJ10967 fis, clone PLACE1000798. [AK001829]	440498

A_24_P267552	-1.7	1.4	-4.1	-3.5	HSD11B1L	Homo sapiens hydroxysteroid (11-beta) dehydrogenase 1-like (HSD11B1L), transcript variant e, mRNA [NM_198533]	374875
A_23_P387374	2.2	-1.1	2.9	-1.1	HSD17B1	Homo sapiens, clone IMAGE:4915712, mRNA. [BC019592]	3292
A_23_P15542	-3.2	-1.0	-1.0	1.0	HSD17B1	Homo sapiens hydroxysteroid (17-beta) dehydrogenase 1 (HSD17B1), mRNA [NM_000413]	3292
A_23_P408271	-1.1	-1.1	-4.4	-1.2	HSD17B11	Homo sapiens hydroxysteroid (17-beta) dehydrogenase 11 (HSD17B11), mRNA [NM_016245]	51170

A_24_P392774	-1.4	-2.1	-1.0	-1.0	HSD17B12	Homo sapiens hydroxysteroid (17-beta) dehydrogenase 12 (HSD17B12), mRNA [NM_016142]	51144
A_23_P78762	-1.4	-2.1	1.7	1.4	HSD17B14	Homo sapiens hydroxysteroid (17-beta) dehydrogenase 14 (HSD17B14), mRNA [NM_016246]	51171
A_23_P97173	-5.5	-1.4	-8.7	1.0	HSD3B1	Homo sapiens hydroxy-delta-5-steroid dehydrogenase, 3 beta- and steroid delta-isomerase 1 (HSD3B1), mRNA [NM_000862]	3283

A_23_P206819	-2.7	-30.5	1.7	1.0	HSD3B7	Homo sapiens hydroxy-delta-5-steroid dehydrogenase, 3 beta- and steroid delta-isomerase 7 (HSD3B7), transcript variant 1, mRNA [NM_025193]	80270
A_23_P169197	1.5	2.7	1.5	1.6	HSDL2	Homo sapiens hydroxysteroid dehydrogenase like 2 (HSDL2), transcript variant 1, mRNA [NM_032303]	84263
A_23_P253841	1.0	-1.0	-3.8	-1.0	HSF1	Homo sapiens heat shock transcription factor 1 (HSF1), mRNA [NM_005526]	3297

A_23_P143512	1.0	-1.0	-8.5	-1.1	HSF2BP	Homo sapiens heat shock transcription factor 2 binding protein (HSF2BP), mRNA [NM_007031]	11077
A_32_P109270	3.6	2.4	2.6	2.7	HSF5	Homo sapiens heat shock transcription factor family member 5 (HSF5), mRNA [NM_001080439]	124535
A_23_P96497	1.1	1.1	2.2	1.5	HSFX1	Homo sapiens heat shock transcription factor family, X linked 1 (HSFX1), mRNA [NM_016153]	100506164

A_24_P145377	2.6	1.5	1.7	4.2	HSP90AA1	Homo sapiens heat shock protein 90kDa alpha (cytosolic), class A member 1 (HSP90AA1), transcript variant 1, mRNA [NM_001017963]	3320
A_24_P170295	-1.1	-1.1	-2.9	-1.2	HSP90AB1	Homo sapiens heat shock protein 90kDa alpha (cytosolic), class B member 1 (HSP90AB1), mRNA [NM_007355]	3326

A_24_P161525	1.4	2.6	1.3	-1.0	HSP90AB3P	Homo sapiens heat shock protein 90kDa alpha (cytosolic), class B member 3, pseudogene (HSP90AB3P), non-coding RNA [NR_036694]	3327
A_24_P531133	1.7	4.6	-1.0	-1.1	HSP90AB6P	Homo sapiens heat shock protein 90Bf (HSP90Bf) mRNA, complete cds. [AY956767]	541611
A_24_P150361	-2.5	-2.6	-2.0	-1.4	HSP90B1	Homo sapiens heat shock protein 90kDa beta (Grp94), member 1 (HSP90B1), mRNA [NM_003299]	7184

A_24_P134392	-1.0	-1.0	-1.0	-2.4	HSPA13	Homo sapiens heat shock protein 70kDa family, member 13 (HSPA13), mRNA [NM_006948]	6782
A_23_P70547	1.0	1.0	-3.1	1.0	HSPA1L	Homo sapiens heat shock 70kDa protein 1-like (HSPA1L), mRNA [NM_005527]	3305
A_24_P18190	-2.7	-2.9	-3.4	-2.7	HSPA5	Homo sapiens heat shock 70kDa protein 5 (glucose-regulated protein, 78kDa) (HSPA5), mRNA [NM_005347]	3309

A_23_P114903	8.6	6.0	8.4	3.9	HSPA6	Homo sapiens heat shock 70kDa protein 6 (HSP70B') (HSPA6), mRNA [NM_002155]	3310
A_32_P13728	1.3	1.1	1.0	-3.0	HSPA8	Homo sapiens heat shock 70kDa protein 8 (HSPA8), transcript variant 1, mRNA [NM_006597]	3312
A_24_P295745	-1.5	-2.0	-1.4	1.0	HSPA8	Homo sapiens heat shock 70kDa protein 8 (HSPA8), transcript variant 2, mRNA [NM_153201]	3312

A_24_P77676	30.0	26.1	26.6	27.6	HSPA9	Homo sapiens heat shock 70kDa protein 9 (mortalin) (HSPA9), nuclear gene encoding mitochondrial protein, mRNA [NM_004134]	3313
A_32_P76247	1.1	-2.5	-5.3	-6.0	HSPB1	Homo sapiens heat shock 27kDa protein 1 (HSPB1), mRNA [NM_001540]	3315
A_24_P86537	2.0	1.5	1.9	-1.7	HSPB1	Homo sapiens heat shock 27kDa protein 1 (HSPB1), mRNA [NM_001540]	3315

A_23_P96872	1.5	2.1	1.3	-1.1	HSPB11	Homo sapiens heat shock protein family B (small), member 11 (HSPB11), mRNA [NM_016126]	51668
A_23_P161727	-2.4	-2.4	-2.0	-2.1	HSPB2	Homo sapiens heat shock 27kDa protein 2 (HSPB2), mRNA [NM_001541]	3316
A_23_P416212	-1.7	-1.1	1.2	-2.9	HSPB9	Homo sapiens heat shock protein, alpha-crystallin-related, B9 (HSPB9), mRNA [NM_033194]	94086

A_23_P49725	-3.8	1.1	-2.1	-1.1	HSPB9	Homo sapiens heat shock protein, alpha-crystallin-related, B9 (HSPB9), mRNA [NM_033194]	94086
A_23_P10685	1.6	-3.7	1.7	1.2	HSPBP1	Homo sapiens HSPA (heat shock 70kDa) binding protein, cytoplasmic cochaperone 1 (HSPBP1), transcript variant 1, mRNA [NM_012267]	23640

A_23_P170687	-1.3	-2.0	-3.0	-1.4	HSPBP1	Homo sapiens HSPA (heat shock 70kDa) binding protein, cytoplasmic cochaperone 1 (HSPBP1), transcript variant 1, mRNA [NM_012267]	23640
A_23_P68577	-1.0	-1.0	1.0	-2.0	HSPC072	Homo sapiens uncharacterized LOC29075 (HSPC072), transcript variant 1, non-coding RNA [NR_026883]	29075
A_24_P59297	-1.8	1.5	-2.4	-1.2	HSPC081	Homo sapiens HSPC081 mRNA, partial cds. [AF161344]	100286989

A_32_P76091	-1.4	-1.5	-2.2	-1.5	HSPD1	Homo sapiens heat shock 60kDa protein 1 (chaperonin) (HSPD1), nuclear gene encoding mitochondrial protein, transcript variant 1, mRNA [NM_002156]	3329
A_23_P88119	1.0	1.0	-3.1	1.1	HSPH1	Homo sapiens heat shock 105kDa/110kDa protein 1 (HSPH1), mRNA [NM_006644]	10808

A_32_P135890	1.0	-2.3	1.0	-1.0	HTATSF1P2	Homo sapiens cDNA FLJ46534 fis, clone THYMU3037052, weakly similar to Homo sapiens HIV TAT specific factor 1 (HTATSF1). [AK128391]	401233
A_24_P354429	3.0	2.3	1.2	1.3	HTN1	Homo sapiens histatin 1 (HTN1), mRNA [NM_002159]	3346
A_24_P97687	1.0	-1.9	-5.7	-1.0	HTR1A	Homo sapiens 5-hydroxytryptamine (serotonin) receptor 1A (HTR1A), mRNA [NM_000524]	3350
A_23_P156824	1.4	4.1	3.7	2.4	HTR1B	Homo sapiens 5-hydroxytryptamine (serotonin) receptor 1B (HTR1B), mRNA [NM_000863]	3351

A_23_P166674	1.1	1.1	-4.7	1.2	HTR1F	Homo sapiens 5-hydroxytryptamine (serotonin) receptor 1F (HTR1F), mRNA [NM_000866]	3355
A_24_P355967	1.0	-2.1	-5.0	1.6	HTR2A	Homo sapiens 5-hydroxytryptamine (serotonin) receptor 2A (HTR2A), transcript variant 1, mRNA [NM_000621]	3356
A_24_P150068	1.0	1.0	1.5	2.7	HTR3A	Homo sapiens 5-hydroxytryptamine (serotonin) receptor 3A (HTR3A), transcript variant 1, mRNA [NM_213621]	3359
A_23_P87007	-1.1	-3.0	-1.0	-1.0	HTR3B	Homo sapiens 5-hydroxytryptamine (serotonin) receptor 3B (HTR3B), mRNA [NM_006028]	9177

A_23_P124402	1.8	2.3	1.5	-1.1	HTR3C	Homo sapiens 5-hydroxytryptamine (serotonin) receptor 3, family member C (HTR3C), mRNA [NM_130770]	170572
A_24_P32139	-1.0	2.2	-4.7	1.6	HTR3E	Homo sapiens 5-hydroxytryptamine (serotonin) receptor 3, family member E (HTR3E), mRNA [NM_182589]	285242
A_23_P133637	-2.5	-2.3	-2.3	-1.4	HTR4	Homo sapiens 5-hydroxytryptamine (serotonin) receptor 4 (HTR4), transcript variant d, mRNA [NM_001040172]	3360
A_24_P234768	-1.0	-1.0	-1.0	-3.2	HTR4	Homo sapiens 5-hydroxytryptamine (serotonin) receptor 4 (HTR4), transcript variant i, mRNA [NM_001040173]	3360

A_23_P42565	-12.3	1.2	1.2	-1.0	HTR5A	Homo sapiens 5-hydroxytryptamine (serotonin) receptor 5A (HTR5A), mRNA [NM_024012]	3361
A_24_P375819	1.4	2.2	2.5	1.9	HTR7P1	Homo sapiens 5-hydroxytryptamine (serotonin) receptor 7 pseudogene 1 (HTR7P1), non-coding RNA [NR_002774]	93164
A_23_P97990	-9.6	-1.0	-1.1	-1.1	HTRA1	Homo sapiens HtrA serine peptidase 1 (HTRA1), mRNA [NM_002775]	5654
A_23_P10542	-1.3	-2.2	-1.3	-1.2	HTRA3	Homo sapiens HtrA serine peptidase 3 (HTRA3), mRNA [NM_053044]	94031

A_23_P166235	1.5	2.6	1.5	-1.0	HUNK	Homo sapiens hormonally up-regulated Neu-associated kinase (HUNK), mRNA [NM_014586]	30811
A_23_P219207	2.7	-1.1	2.1	-1.7	HUWE1	Homo sapiens HECT, UBA and WWE domain containing 1 (HUWE1), mRNA [NM_031407]	10075
A_24_P310667	-1.9	-1.1	-4.9	-3.6	HUWE1	Homo sapiens HECT, UBA and WWE domain containing 1 (HUWE1), mRNA [NM_031407]	10075

A_24_P153820	1.0	-3.5	2.4	2.4	HYDIN	Homo sapiens hydrocephalus inducing homolog (mouse), mRNA (cDNA clone IMAGE:4837075), partial cds. [BC028351]	54768
A_23_P383679	2.2	3.6	1.2	4.5	HYDIN	Homo sapiens hydrocephalus inducing homolog (mouse) (HYDIN), transcript variant 1, mRNA [NM_032821]	54768
A_23_P160582	1.2	2.2	-1.3	2.9	HYI	Homo sapiens hydroxypyruvate isomerase (putative) (HYI), transcript variant 1, mRNA [NM_031207]	81888

A_23_P75647	1.4	1.3	-2.3	1.3	HYOU1	Homo sapiens hypoxia up-regulated 1 (HYOU1), transcript variant 1, mRNA [NM_006389]	10525
A_32_P436884	2.5	4.0	4.9	1.0	IBA57	Homo sapiens IBA57, iron-sulfur cluster assembly homolog (S. cerevisiae) (IBA57), mRNA [NM_001010867]	200205
A_23_P144549	-6.9	2.0	2.9	2.2	IBSP	Homo sapiens integrin-binding sialoprotein (IBSP), mRNA [NM_004967]	3381
A_23_P358995	-1.8	-2.5	-1.0	1.1	IBTK	Homo sapiens inhibitor of Bruton agammaglobulinemia tyrosine kinase (IBTK), mRNA [NM_015525]	25998

A_23_P215419	3.3	1.6	-1.1	-1.9	ICA1	Homo sapiens islet cell autoantigen 1, 69kDa (ICA1), transcript variant 2, mRNA [NM_004968]	3382
A_24_P372012	-1.3	-3.3	-4.2	-3.0	ICA1	Homo sapiens islet cell autoantigen 1, 69kDa (ICA1), transcript variant 2, mRNA [NM_004968]	3382
A_23_P430168	-31.1	1.0	-1.0	1.1	ICA1L	Homo sapiens islet cell autoantigen 1,69kDa-like (ICA1L), transcript variant 2, mRNA [NM_178231]	130026

A_23_P17224	1.3	1.5	-2.2	-3.6	ICA1L	Homo sapiens islet cell autoantigen 1,69kDa-like (ICA1L), transcript variant 1, mRNA [NM_138468]	130026
A_24_P254079	-1.0	2.2	2.3	1.0	ICAM5	Homo sapiens intercellular adhesion molecule 5, telencephalin (ICAM5), mRNA [NM_003259]	7087
A_23_P104065	-15.4	1.1	1.0	1.0	ICMT	Homo sapiens isoprenylcysteine carboxyl methyltransferase (ICMT), mRNA [NM_012405]	23463
A_23_P166280	-1.7	-1.2	-2.7	1.2	ICOSLG	inducible T-cell co-stimulator ligand [Source:HGNC Symbol;Acc:17087] [ENST00000407780]	23308

A_23_P6272	6.5	7.9	8.0	2.8	ICOSLG	Homo sapiens inducible T-cell co-stimulator ligand (ICOSLG), mRNA [NM_015259]	23308
A_32_P69368	1.7	2.4	2.5	-1.0	ID2	Homo sapiens inhibitor of DNA binding 2, dominant negative helix-loop-helix protein (ID2), mRNA [NM_002166]	3398
A_23_P137381	1.3	3.0	-1.5	-1.7	ID3	Homo sapiens inhibitor of DNA binding 3, dominant negative helix-loop-helix protein (ID3), mRNA [NM_002167]	3399

A_23_P59375	2.1	1.3	-3.3	2.5	ID4	Homo sapiens inhibitor of DNA binding 4, dominant negative helix-loop-helix protein (ID4), mRNA [NM_001546]	3400
A_23_P410600	-1.1	2.4	2.3	-1.9	IDI2	Homo sapiens isopentenyl-diphosphate delta isomerase 2 (IDI2), mRNA [NM_033261]	91734
A_23_P112026	-2.3	1.0	-1.0	-1.0	IDO1	Homo sapiens indoleamine 2,3-dioxygenase 1 (IDO1), mRNA [NM_002164]	3620
A_24_P285032	-2.3	-1.0	1.7	-1.0	IDS	Homo sapiens iduronate 2-sulfatase (IDS), transcript variant 2, mRNA [NM_006123]	3423

A_23_P42257	2.1	1.9	1.5	-1.0	IER3	Homo sapiens immediate early response 3 (IER3), mRNA [NM_003897]	8870
A_32_P126928	1.0	-4.8	1.0	-3.6	IER3IP1	Homo sapiens PRO2309 mRNA, complete cds. [AF119875]	51124
A_24_P379223	-2.1	-2.2	-1.0	-2.1	IER5	Homo sapiens immediate early response 5 (IER5), mRNA [NM_016545]	51278
A_23_P87742	1.0	1.3	-1.5	-4.6	IFFO1	Homo sapiens intermediate filament family orphan 1 (IFFO1), transcript variant 4, mRNA [NM_001039670]	25900

A_23_P418031	-2.1	-1.6	-2.0	-1.2	IFFO2	Homo sapiens intermediate filament family orphan 2 (IFFO2), mRNA [NM_001136265]	126917
A_23_P160025	1.5	-1.6	1.3	-3.4	IFI16	Homo sapiens interferon, gamma-inducible protein 16 (IFI16), transcript variant 2, mRNA [NM_005531]	3428
A_24_P261929	3.0	2.3	2.7	1.0	IFI27L1	Homo sapiens interferon, alpha-inducible protein 27-like 1 (IFI27L1), transcript variant 2, mRNA [NM_206949]	122509
A_23_P140146	3.4	4.2	2.5	6.1	IFI27L2	Homo sapiens interferon, alpha-inducible protein 27-like 2 (IFI27L2), mRNA [NM_032036]	83982

A_23_P153745	-1.9	-1.1	-7.7	-2.0	IFI30	Homo sapiens interferon, gamma-inducible protein 30 (IFI30), mRNA [NM_006332]	10437
A_23_P152782	1.3	1.5	1.4	6.4	IFI35	Homo sapiens interferon-induced protein 35 (IFI35), mRNA [NM_005533]	3430
A_23_P52266	2.5	2.1	1.4	1.1	IFIT1	Homo sapiens interferon-induced protein with tetratricopeptide repeats 1 (IFIT1), transcript variant 2, mRNA [NM_001548]	3434
A_23_P24004	1.1	-36.4	-1.0	-1.0	IFIT2	Homo sapiens interferon-induced protein with tetratricopeptide repeats 2 (IFIT2), mRNA [NM_001547]	3433

A_24_P304071	-9.5	-1.0	-1.0	1.0	IFIT2	Homo sapiens interferon-induced protein with tetratricopeptide repeats 2 (IFIT2), mRNA [NM_001547]	3433
A_23_P35412	1.3	1.3	-2.1	1.3	IFIT3	Homo sapiens interferon-induced protein with tetratricopeptide repeats 3 (IFIT3), transcript variant 1, mRNA [NM_001549]	3437
A_23_P63668	-4.6	1.2	-3.2	-1.1	IFIT5	Homo sapiens interferon-induced protein with tetratricopeptide repeats 5 (IFIT5), mRNA [NM_012420]	24138

A_24_P287043	1.7	1.5	2.3	1.0	IFITM2	Homo sapiens interferon induced transmembrane protein 2 (1-8D) (IFITM2), mRNA [NM_006435]	10581
A_23_P87545	-2.0	-2.4	-1.3	-1.4	IFITM3	Homo sapiens interferon induced transmembrane protein 3 (IFITM3), mRNA [NM_021034]	10410
A_23_P311050	-1.1	-1.4	-3.0	-1.2	IFLTD1	Homo sapiens intermediate filament tail domain containing 1 (IFLTD1), transcript variant 2, mRNA [NM_152590]	160492
A_23_P250251	2.1	1.1	-2.2	-1.4	IFNA2	Homo sapiens interferon, alpha 2 (IFNA2), mRNA [NM_000605]	3440

A_24_P386375	-1.2	-3.8	-1.4	-1.3	IFNA21	Homo sapiens interferon, alpha 21 (IFNA21), mRNA [NM_002175]	3452
A_23_P29036	-4.8	1.1	1.3	-1.2	IFNGR2	Homo sapiens interferon gamma receptor 2 (interferon gamma transducer 1) (IFNGR2), mRNA [NM_005534]	3460
A_23_P310372	-2.2	-1.0	-1.1	-1.0	IFNK	Homo sapiens interferon, kappa (IFNK), mRNA [NM_020124]	56832
A_24_P137897	-2.4	-1.3	-1.5	-1.7	IFRD1	Homo sapiens interferon-related developmental regulator 1 (IFRD1), transcript variant 2, mRNA [NM_001007245]	3475

A_23_P92132	1.1	-5.1	-1.0	-1.0	IFRD2	Homo sapiens interferon-related developmental regulator 2 (IFRD2), mRNA [NM_006764]	7866
A_23_P212447	-1.2	1.4	-3.4	1.3	IFT122	Homo sapiens intraflagellar transport 122 homolog (Chlamydomonas) (IFT122), transcript variant 3, mRNA [NM_018262]	55764
A_23_P406135	-1.0	3.0	2.0	1.2	IFT172	Homo sapiens intraflagellar transport 172 homolog (Chlamydomonas) (IFT172), mRNA [NM_015662]	26160

A_23_P6447	-6.5	1.2	1.1	1.2	IFT27	Homo sapiens intraflagellar transport 27 homolog (Chlamydomonas) (IFT27), transcript variant 2, mRNA [NM_006860]	11020
A_23_P165962	1.1	1.1	1.1	-4.1	IFT52	Homo sapiens intraflagellar transport 52 homolog (Chlamydomonas) (IFT52), mRNA [NM_016004]	51098
A_23_P121386	1.4	1.4	-4.6	1.1	IFT57	Homo sapiens intraflagellar transport 57 homolog (Chlamydomonas) (IFT57), mRNA [NM_018010]	55081

A_23_P255714	2.8	1.8	3.3	1.5	IFT74	Homo sapiens intraflagellar transport 74 homolog (Chlamydomonas) (IFT74), transcript variant 1, mRNA [NM_025103]	80173
A_23_P406013	1.0	-3.7	2.7	1.3	IFT80	Homo sapiens intraflagellar transport 80 homolog (Chlamydomonas) (IFT80), transcript variant 1, mRNA [NM_020800]	57560
A_24_P164123	-1.0	-17.4	-1.0	-1.0	IFT81	intraflagellar transport 81 homolog (Chlamydomonas) [Source:HGNC Symbol;Acc:14313] [ENST00000361948]	28981

A_23_P171249	1.1	1.0	3.3	-1.0	IGBP1	Homo sapiens immunoglobulin (CD79A) binding protein 1 (IGBP1), mRNA [NM_001551]	3476
A_24_P202025	1.6	1.8	-2.7	1.0	IGDCC4	Homo sapiens immunoglobulin superfamily, DCC subclass, member 4 (IGDCC4), mRNA [NM_020962]	57722
A_23_P417282	-3.3	-2.7	2.0	1.1	IGF1R	Homo sapiens insulin-like growth factor 1 receptor (IGF1R), mRNA [NM_000875]	3480
A_24_P936758	2.0	2.5	1.2	-1.4	IGF2	Homo sapiens cDNA FLJ90133 fis, clone HEMBB1000567. [AK074614]	3481

A_23_P343954	-1.1	-2.5	2.1	1.4	IGF2BP1	Homo sapiens insulin-like growth factor 2 mRNA binding protein 1 (IGF2BP1), transcript variant 1, mRNA [NM_006546]	10642
A_23_P250156	-2.2	-2.7	-1.5	-2.4	IGF2BP2	Homo sapiens insulin-like growth factor 2 mRNA binding protein 2 (IGF2BP2), transcript variant 1, mRNA [NM_006548]	10644
A_23_P334021	-1.1	-1.7	-11.8	1.0	IGF2R	Homo sapiens insulin-like growth factor 2 receptor (IGF2R), mRNA [NM_000876]	3482

A_23_P156953	1.1	1.1	2.5	1.3	IGF2R	Homo sapiens insulin-like growth factor 2 receptor (IGF2R), mRNA [NM_000876]	3482
A_23_P119943	2.1	1.9	2.7	2.7	IGFBP2	Homo sapiens insulin-like growth factor binding protein 2, 36kDa (IGFBP2), mRNA [NM_000597]	3485
A_24_P320699	-2.6	1.6	-2.0	-2.1	IGFBP3	Homo sapiens insulin-like growth factor binding protein 3 (IGFBP3), transcript variant 1, mRNA [NM_001013398]	3486

A_24_P382187	-1.4	-3.7	-3.7	-2.9	IGFBP4	Homo sapiens insulin-like growth factor binding protein 4 (IGFBP4), mRNA [NM_001552]	3487
A_23_P376497	2.5	3.1	-1.0	-2.1	IGFBP5	Homo sapiens insulin-like growth factor binding protein 5 (IGFBP5), mRNA [NM_000599]	3488
A_23_P383009	-1.0	-4.4	-2.7	1.0	IGFBP5	Homo sapiens insulin-like growth factor binding protein 5 (IGFBP5), mRNA [NM_000599]	3488
A_23_P154115	-1.2	-2.6	1.5	-1.1	IGFBP5	Homo sapiens insulin-like growth factor binding protein 5 (IGFBP5), mRNA [NM_000599]	3488

A_23_P353035	-1.3	1.1	-2.1	-1.0	IGFBP7	Homo sapiens insulin-like growth factor binding protein 7 (IGFBP7), mRNA [NM_001553]	3490
A_24_P186274	2.9	2.9	1.3	-1.0	IGFBPL1	Homo sapiens insulin-like growth factor binding protein-like 1 (IGFBPL1), mRNA [NM_001007563]	347252
A_23_P142424	-21.8	2.1	3.0	1.0	IGFLR1	Homo sapiens IGF-like family receptor 1 (IGFLR1), mRNA [NM_024660]	79713
A_32_P425998	1.1	1.1	1.1	-3.1	IGFN1	Homo sapiens immunoglobulin-like and fibronectin type III domain containing 1 (IGFN1), mRNA [NM_001164586]	91156

A_23_P124632	-1.5	-1.8	1.0	-5.5	IGHA1	Homo sapiens SNC73 protein (SNC73) mRNA, complete cds. [AF067420]	3493
A_24_P324838	-1.0	1.5	2.2	1.6	IGHD	Homo sapiens cDNA FLJ46727 fis, clone TRACH3019058, highly similar to Ig delta chain C region. [AK128568]	3495
A_23_P167168	1.1	-2.2	1.6	-1.1	IGJ	Homo sapiens immunoglobulin J polypeptide, linker protein for immunoglobulin alpha and mu polypeptides (IGJ), mRNA [NM_144646]	3512

A_24_P93523	1.2	1.5	2.2	1.1	IGKV1D-8	Homo sapiens clone S12G10K myosin-reactive immunoglobulin light chain variable region mRNA, partial cds. [AF035035]	28904
A_24_P104980	-1.9	-9.2	-4.5	1.4	IGLL5	Homo sapiens germline mRNA for immunoglobulin lambda-2 chain constant region, Daudi cell line. [AJ319669]	100423062
A_24_P271149	-10.4	2.1	1.1	1.6	IGLON5	Homo sapiens IgLON family member 5 (IGLON5), mRNA [NM_001101372]	402665

A_23_P125303	-2.7	1.1	1.2	1.6	IGSF1	Homo sapiens immunoglobulin superfamily, member 1 (IGSF1), transcript variant 1, mRNA [NM_001555]	3547
A_23_P398189	-1.0	-1.0	-1.1	-2.1	IGSF11	Homo sapiens immunoglobulin superfamily, member 11 (IGSF11), transcript variant 1, mRNA [NM_152538]	152404
A_32_P78101	2.6	1.1	1.1	1.3	IGSF21	Homo sapiens immunoglobin superfamily, member 21 (IGSF21), mRNA [NM_032880]	84966

A_32_P486620	-1.0	-1.0	-1.0	-2.1	IGSF22	Homo sapiens immunoglobulin superfamily, member 22 (IGSF22), mRNA [NM_173588]	283284
A_24_P750817	1.1	1.0	1.0	-5.1	IGSF23	Homo sapiens immunoglobulin superfamily, member 23 (IGSF23), mRNA [NM_001205280]	147710
A_23_P106629	1.2	1.1	2.7	2.3	IGSF6	Homo sapiens immunoglobulin superfamily, member 6 (IGSF6), mRNA [NM_005849]	10261
A_23_P137423	2.0	3.7	2.5	1.7	IGSF8	Homo sapiens immunoglobulin superfamily, member 8 (IGSF8), transcript variant 1, mRNA [NM_052868]	93185

A_23_P85441	2.3	1.5	1.7	-1.0	IGSF9	Homo sapiens immunoglobulin superfamily, member 9 (IGSF9), transcript variant 2, mRNA [NM_020789]	57549
A_32_P173481	-1.5	1.2	-2.8	-1.3	IK	Homo sapiens IK cytokine, down-regulator of HLA II (IK), mRNA [NM_006083]	3550
A_24_P132518	-11.8	1.0	1.0	1.1	IKBKB	Homo sapiens inhibitor of kappa light polypeptide gene enhancer in B-cells, kinase beta (IKBKB), transcript variant 1, mRNA [NM_001556]	3551

A_23_P887	1.0	1.0	1.0	3.0	IKBKE	Homo sapiens inhibitor of kappa light polypeptide gene enhancer in B-cells, kinase epsilon (IKBKE), transcript variant 1, mRNA [NM_014002]	9641
A_23_P159920	-1.0	1.4	2.1	-1.9	IKBKG	Homo sapiens inhibitor of kappa light polypeptide gene enhancer in B-cells, kinase gamma (IKBKG), transcript variant 3, mRNA [NM_003639]	8517
A_24_P190631	1.1	-2.1	1.7	4.1	IKZF1	Homo sapiens IKAROS family zinc finger 1 (Ikaros) (IKZF1), transcript variant 1, mRNA [NM_006060]	10320

A_32_P114284	1.0	1.5	1.3	-2.9	IKZF2	Homo sapiens IKAROS family zinc finger 2 (Helios) (IKZF2), transcript variant 2, mRNA [NM_001079526]	22807
A_32_P115475	2.7	2.5	3.1	3.2	IKZF3	IKAROS family zinc finger 3 (Aiolos) [Source:HGNC Symbol;Acc:13178] [ENST00000488188]	22806
A_23_P358904	1.1	-3.0	1.3	-2.7	IKZF4	Homo sapiens IKAROS family zinc finger 4 (Eos) (IKZF4), mRNA [NM_022465]	64375

A_23_P91943	-2.3	1.1	-1.9	-1.3	IL12A	Homo sapiens interleukin 12A (natural killer cell stimulatory factor 1, cytotoxic lymphocyte maturation factor 1, p35) (IL12A), mRNA [NM_000882]	3592
A_23_P7560	1.1	-5.9	1.0	-1.1	IL12B	Homo sapiens interleukin 12B (natural killer cell stimulatory factor 2, cytotoxic lymphocyte maturation factor 2, p40) (IL12B), mRNA [NM_002187]	3593
A_23_P101783	-3.2	1.9	1.1	-1.4	IL12RB1	Homo sapiens interleukin 12 receptor, beta 1 (IL12RB1), transcript variant 1, mRNA [NM_005535]	3594

A_23_P399156	2.2	1.1	2.0	-1.5	IL12RB1	Homo sapiens interleukin 12 receptor, beta 1 (IL12RB1), transcript variant 2, mRNA [NM_153701]	3594
A_24_P280113	1.0	-16.4	1.1	1.0	IL13RA1	Homo sapiens interleukin 13 receptor, alpha 1 (IL13RA1), mRNA [NM_001560]	3597
A_23_P85209	3.2	-1.4	-1.1	-1.1	IL13RA2	Homo sapiens interleukin 13 receptor, alpha 2 (IL13RA2), mRNA [NM_000640]	3598
A_23_P138680	-1.8	1.3	1.1	-2.8	IL15RA	Homo sapiens interleukin 15 receptor, alpha (IL15RA), transcript variant 2, mRNA [NM_172200]	3601

A_23_P61057	-1.4	-2.9	-2.8	-4.6	IL16	Homo sapiens interleukin 16 (IL16), transcript variant 1, mRNA [NM_004513]	3603
A_24_P73599	1.0	1.0	-2.7	-3.8	IL16	Homo sapiens interleukin 16 (IL16), transcript variant 2, mRNA [NM_172217]	3603
A_23_P49097	1.8	2.4	1.3	1.3	IL17C	Homo sapiens interleukin 17C (IL17C), mRNA [NM_013278]	27189
A_23_P167882	-1.2	-3.9	-1.3	-1.2	IL17F	Homo sapiens interleukin 17F (IL17F), mRNA [NM_052872]	112744
A_23_P17706	-3.0	1.2	1.1	1.2	IL17RA	Homo sapiens interleukin 17 receptor A (IL17RA), mRNA [NM_014339]	23765

A_23_P166775	2.6	3.0	1.8	2.0	IL17RC	Homo sapiens interleukin 17 receptor C (IL17RC), transcript variant 2, mRNA [NM_153461]	84818
A_23_P500206	1.1	6.5	1.8	1.0	IL17RE	Homo sapiens interleukin 17 receptor E (IL17RE), transcript variant 5, mRNA [NM_153483]	132014
A_24_P278602	1.7	1.1	1.7	-2.4	IL18BP	Homo sapiens interleukin 18 binding protein (IL18BP), transcript variant A, mRNA [NM_173042]	10068
A_23_P203629	1.5	2.2	2.7	2.0	IL18BP	Homo sapiens interleukin 18 binding protein (IL18BP), transcript variant A, mRNA [NM_173042]	10068

A_24_P208567	1.0	-2.5	1.0	-7.7	IL18R1	Homo sapiens interleukin 18 receptor 1 (IL18R1), mRNA [NM_003855]	8809
A_23_P35092	1.8	2.4	1.6	1.7	IL19	Homo sapiens interleukin 19 (IL19), transcript variant 1, mRNA [NM_153758]	29949
A_23_P501713	-2.0	1.0	-1.4	-2.2	IL1F10	Homo sapiens interleukin 1 family, member 10 (theta) (IL1F10), transcript variant 1, mRNA [NM_032556]	84639
A_24_P200023	-1.7	-2.2	-3.9	-1.9	IL1R1	Homo sapiens interleukin 1 receptor, type I (IL1R1), mRNA [NM_000877]	3554

A_23_P79398	2.5	-1.2	1.1	2.8	IL1R2	Homo sapiens interleukin 1 receptor, type II (IL1R2), transcript variant 1, mRNA [NM_004633]	7850
A_23_P336554	-1.0	-1.0	-3.0	-1.0	IL1RAP	Homo sapiens interleukin 1 receptor accessory protein (IL1RAP), transcript variant 2, mRNA [NM_134470]	3556
A_23_P170857	-1.2	-1.0	-1.0	-2.3	IL1RAP	Homo sapiens interleukin 1 receptor accessory protein (IL1RAP), transcript variant 1, mRNA [NM_002182]	3556
A_23_P56604	2.8	1.9	1.7	1.1	IL1RL2	Homo sapiens interleukin 1 receptor-like 2 (IL1RL2), mRNA [NM_003854]	8808

A_32_P38125	-1.0	-4.5	-1.1	-1.1	IL1RN	interleukin 1 receptor antagonist [Source:HGNC Symbol;Acc:6000] [ENST00000465812]	3557
A_23_P167250	1.1	2.7	1.7	3.4	IL21	Homo sapiens interleukin 21 (IL21), transcript variant 1, mRNA [NM_021803]	59067
A_24_P413479	-1.2	-1.2	-2.3	-1.3	IL22RA1	Homo sapiens interleukin 22 receptor, alpha 1 (IL22RA1), mRNA [NM_021258]	58985
A_23_P51951	-1.1	2.9	-1.1	1.1	IL24	Homo sapiens interleukin 24 (IL24), transcript variant 1, mRNA [NM_006850]	11009

A_24_P348326	2.0	4.9	3.9	3.1	IL27RA	Homo sapiens interleukin 27 receptor, alpha (IL27RA), mRNA [NM_004843]	9466
A_23_P27606	-2.0	-1.8	-2.4	-2.0	IL27RA	Homo sapiens interleukin 27 receptor, alpha (IL27RA), mRNA [NM_004843]	9466
A_23_P409438	1.3	2.2	1.2	-1.2	IL28A	Homo sapiens interleukin 28A (interferon, lambda 2) (IL28A), mRNA [NM_172138]	282616
A_23_P337800	-5.8	-1.4	-1.3	-1.3	IL29	Homo sapiens interleukin 29 (interferon, lambda 1) (IL29), mRNA [NM_172140]	282618

A_23_P127288	1.0	1.0	-2.2	1.1	IL2RA	Homo sapiens interleukin 2 receptor, alpha (IL2RA), mRNA [NM_000417]	3559
A_23_P148473	2.3	1.2	1.9	-10.5	IL2RG	Homo sapiens interleukin 2 receptor, gamma (IL2RG), mRNA [NM_000206]	3561
A_24_P937317	4.5	3.3	1.6	1.5	IL31	Homo sapiens interleukin 31 (IL31), mRNA [NM_001014336]	386653
A_24_P377283	1.4	-6.7	1.6	-1.0	IL31RA	Homo sapiens interleukin 31 receptor A (IL31RA), transcript variant 1, mRNA [NM_139017]	133396

A_23_P100386	-1.6	-13.6	-1.6	-1.6	IL34	Homo sapiens interleukin 34 (IL34), transcript variant 1, mRNA [NM_152456]	146433
A_23_P348028	-1.5	-2.5	-2.3	-2.3	IL36A	Homo sapiens interleukin 36, alpha (IL36A), mRNA [NM_014440]	27179
A_23_P90925	1.1	-5.0	-1.1	1.0	IL36B	Homo sapiens interleukin 36, beta (IL36B), transcript variant 2, mRNA [NM_173178]	27177
A_24_P68783	-2.8	-1.7	-1.0	-1.0	IL36RN	Homo sapiens interleukin 36 receptor antagonist (IL36RN), transcript variant 1, mRNA [NM_012275]	26525

A_23_P500676	-1.3	-2.4	-1.2	-1.2	IL5RA	Homo sapiens interleukin 5 receptor, alpha (IL5RA), transcript variant 3, mRNA [NM_175725]	3568
A_24_P379413	1.2	1.1	1.1	-3.9	IL6R	Homo sapiens interleukin 6 receptor (IL6R), transcript variant 1, mRNA [NM_000565]	3570
A_32_P45168	3.0	4.4	2.0	2.8	IL6ST	Homo sapiens interleukin 6 signal transducer (gp130, oncostatin M receptor) (IL6ST), transcript variant 1, mRNA [NM_002184]	3572
A_23_P8961	1.0	-2.6	-1.0	-1.0	IL7	Homo sapiens interleukin 7 (IL7), transcript variant 1, mRNA [NM_000880]	3574

A_32_P87013	-1.8	1.1	-2.1	-1.7	IL8	Homo sapiens interleukin 8 (IL8), mRNA [NM_000584]	3576
A_23_P34066	2.2	2.0	3.1	2.0	IL9R	Homo sapiens interleukin 9 receptor (IL9R), transcript variant 1, mRNA [NM_002186]	3581
A_23_P218456	1.2	1.2	1.1	-2.0	ILF3	Homo sapiens interleukin enhancer binding factor 3, 90kDa (ILF3), transcript variant 1, mRNA [NM_012218]	3609
A_24_P406870	1.6	2.0	1.1	2.1	ILK	Homo sapiens integrin-linked kinase (ILK), transcript variant 3, mRNA [NM_001014795]	3611

A_23_P165148	2.0	1.9	1.4	3.8	ILVBL	Homo sapiens ilvB (bacterial acetolactate synthase)-like (ILVBL), mRNA [NM_006844]	10994
A_32_P87872	1.1	-5.2	-1.3	-1.2	IMMP2L	Homo sapiens IMP2 inner mitochondrial membrane peptidase-like (S. cerevisiae) (IMMP2L), nuclear gene encoding mitochondrial protein, transcript variant 1, mRNA [NM_032549]	83943

A_24_P151622	-1.8	-1.0	-2.3	-1.1	IMMP2L	Homo sapiens IMP2 inner mitochondrial membrane peptidase-like (S. cerevisiae) (IMMP2L), nuclear gene encoding mitochondrial protein, transcript variant 1, mRNA [NM_032549]	83943
A_23_P131518	4.0	3.8	1.0	1.0	IMP4	IMP4, U3 small nucleolar ribonucleoprotein, homolog (yeast) [Source:HGNC Symbol;Acc:30856] [ENST00000462392]	92856
A_32_P142779	-1.2	1.3	-3.8	-1.4	IMP5	Homo sapiens intramembrane protease 5 (IMP5), mRNA [NM_175882]	162540

A_23_P168818	-2.1	1.1	-1.0	-1.0	IMPA1	Homo sapiens inositol(myo)-1(or 4)-monophosphatase 1 (IMPA1), transcript variant 1, mRNA [NM_005536]	3612
A_24_P89701	-3.2	-1.8	-3.4	-3.6	IMPDH1	Homo sapiens IMP (inosine 5'-monophosphate) dehydrogenase 1 (IMPDH1), transcript variant 1, mRNA [NM_000883]	3614
A_23_P42032	-3.0	-46.0	-1.0	-3.8	IMPG1	Homo sapiens interphotoreceptor matrix proteoglycan 1 (IMPG1), mRNA [NM_001563]	3617

A_23_P35444	-4.0	2.1	1.9	-3.4	INA	Homo sapiens internexin neuronal intermediate filament protein, alpha (INA), mRNA [NM_032727]	9118
A_23_P321034	1.2	-1.7	-3.4	-2.7	INADL	Homo sapiens InaD-like (Drosophila) (INADL), mRNA [NM_176877]	10207
A_23_P126100	-1.3	-3.2	-1.2	-2.3	INADL	Homo sapiens InaD-like (Drosophila) (INADL), mRNA [NM_176877]	10207
A_24_P942454	-1.0	-5.4	-1.1	-1.2	INADL	Homo sapiens InaD-like (Drosophila) (INADL), mRNA [NM_176877]	10207

A_24_P274072	2.7	2.4	2.3	1.7	INADL	Homo sapiens InaD-like (Drosophila) (INADL), mRNA [NM_176877]	10207
A_23_P116387	1.0	1.0	-2.3	-1.0	INCENP	Homo sapiens inner centromere protein antigens 135/155kDa (INCENP), transcript variant 1, mRNA [NM_001040694]	3619
A_23_P218131	2.4	2.1	2.1	2.2	INF2	Homo sapiens inverted formin, FH2 and WH2 domain containing (INF2), transcript variant 3, mRNA [NM_032714]	64423

A_24_P13649	1.7	3.1	-1.3	1.0	INF2	Homo sapiens inverted formin, FH2 and WH2 domain containing (INF2), transcript variant 3, mRNA [NM_032714]	64423
A_23_P99437	-2.4	1.2	1.4	-2.2	ING1	Homo sapiens inhibitor of growth family, member 1 (ING1), transcript variant 1, mRNA [NM_198219]	3621
A_23_P48070	-1.5	-3.4	-1.9	1.4	ING4	Homo sapiens inhibitor of growth family, member 4 (ING4), transcript variant 1, mRNA [NM_016162]	51147
A_24_P34545	1.3	-1.3	-2.2	-1.0	ING5	Homo sapiens inhibitor of growth family, member 5 (ING5), mRNA [NM_032329]	84289

A_23_P124202	1.2	-1.0	-4.4	-1.0	ING5	Homo sapiens inhibitor of growth family, member 5 (ING5), mRNA [NM_032329]	84289
A_23_P51039	2.1	1.7	2.1	-1.1	INHA	Homo sapiens inhibin, alpha (INHA), mRNA [NM_002191]	3623
A_23_P153964	-2.5	-1.1	-1.4	-1.1	INHBB	Homo sapiens inhibin, beta B (INHBB), mRNA [NM_002193]	3625
A_24_P155502	-4.3	1.3	-2.0	-3.7	INHBC	Homo sapiens inhibin, beta C (INHBC), mRNA [NM_005538]	3626
A_23_P76051	1.3	1.0	-1.9	-4.1	INHBC	Homo sapiens inhibin, beta C (INHBC), mRNA [NM_005538]	3626

A_24_P117954	1.5	1.2	1.3	3.4	INO80C	Homo sapiens INO80 complex subunit C (INO80C), transcript variant 2, mRNA [NM_194281]	125476
A_23_P209652	-1.1	1.1	-1.0	-3.5	INO80D	Homo sapiens INO80 complex subunit D (INO80D), mRNA [NM_017759]	54891
A_23_P142756	2.9	2.2	2.2	2.2	INO80D	Homo sapiens INO80 complex subunit D (INO80D), mRNA [NM_017759]	54891
A_32_P44453	2.5	2.5	2.6	2.5	INPP1	Homo sapiens inositol polyphosphate-1-phosphatase (INPP1), transcript variant 2, mRNA [NM_002194]	3628

A_24_P915492	-1.0	-1.0	-3.2	-1.0	INPP4B	inositol polyphosphate-4-phosphatase, type II, 105kDa [Source:HGNC Symbol;Acc:6075] [ENST00000507861]	8821
A_23_P202307	3.6	3.5	3.5	3.8	INPP5A	Homo sapiens inositol polyphosphate-5-phosphatase, 40kDa (INPP5A), mRNA [NM_005539]	3632
A_23_P94875	1.2	-2.0	1.0	1.1	INPP5E	Homo sapiens inositol polyphosphate-5-phosphatase, 72 kDa (INPP5E), mRNA [NM_019892]	56623

A_24_P4426	-1.5	1.0	-1.7	-2.1	INPP5F	Homo sapiens inositol polyphosphate-5-phosphatase F (INPP5F), transcript variant 1, mRNA [NM_014937]	22876
A_24_P279328	-2.7	1.1	-2.5	-1.0	INPP5K	Homo sapiens inositol polyphosphate-5-phosphatase K (INPP5K), transcript variant 2, mRNA [NM_130766]	51763
A_23_P55076	1.6	1.7	2.1	1.0	INPP5K	Homo sapiens inositol polyphosphate-5-phosphatase K (INPP5K), transcript variant 2, mRNA [NM_130766]	51763

A_23_P36322	1.4	-1.0	2.1	1.0	INPPL1	Homo sapiens inositol polyphosphate phosphatase-like 1 (INPPL1), mRNA [NM_001567]	3636
A_23_P102454	1.6	-1.5	-2.7	2.3	INSIG2	Homo sapiens insulin induced gene 2 (INSIG2), mRNA [NM_016133]	51141
A_23_P209006	2.4	1.2	1.1	1.5	INSL3	Homo sapiens insulin-like 3 (Leydig cell) (INSL3), mRNA [NM_005543]	3640
A_23_P309281	-5.8	-2.3	-1.9	-1.3	INSL3	Homo sapiens insulin-like 3 (Leydig cell) (INSL3), mRNA [NM_005543]	3640

A_24_P31676	-2.4	-1.8	-1.0	-6.6	INSM1	Homo sapiens insulinoma-associated 1 (INSM1), mRNA [NM_002196]	3642
A_24_P66672	4.2	2.8	3.3	2.3	INTS1	Homo sapiens integrator complex subunit 1 (INTS1), mRNA [NM_001080453]	26173
A_23_P157607	-1.1	-1.0	-1.0	2.4	INTS10	Homo sapiens integrator complex subunit 10 (INTS10), mRNA [NM_018142]	55174
A_23_P203737	-2.0	-2.5	-1.4	-2.6	INTS4	Homo sapiens integrator complex subunit 4 (INTS4), mRNA [NM_033547]	92105

A_32_P345444	1.3	1.3	-5.3	1.0	INTS4L1	Homo sapiens integrator complex subunit 4-like 1 (INTS4L1), non-coding RNA [NR_027393]	285905
A_23_P13338	1.0	-1.1	-4.0	-1.1	INTS5	Homo sapiens integrator complex subunit 5 (INTS5), mRNA [NM_030628]	80789
A_24_P913760	-14.8	1.0	-1.1	-1.0	INTS6	Homo sapiens integrator complex subunit 6 (INTS6), transcript variant 1, mRNA [NM_012141]	26512
A_24_P167614	2.5	1.3	-1.7	-2.2	INTS6	Homo sapiens integrator complex subunit 6 (INTS6), transcript variant 1, mRNA [NM_012141]	26512

A_23_P43079	1.1	-8.6	1.4	1.7	INTS8	Homo sapiens integrator complex subunit 8 (INTS8), mRNA [NM_017864]	55656
A_24_P396105	-2.6	1.6	-1.4	-3.9	IP6K1	Homo sapiens inositol hexakisphosphate kinase 1 (IP6K1), transcript variant 1, mRNA [NM_153273]	9807
A_23_P168130	-4.1	5.9	10.3	3.1	IP6K3	Homo sapiens inositol hexakisphosphate kinase 3 (IP6K3), transcript variant 1, mRNA [NM_054111]	117283
A_24_P940059	1.1	-2.4	-2.2	1.0	IPCEF1	Homo sapiens interaction protein for cytohesin exchange factors 1 (IPCEF1), transcript variant 3, mRNA [NM_015553]	26034

A_23_P380371	-1.1	-1.1	-8.6	-4.3	IPMK	Homo sapiens inositol polyphosphate multikinase (IPMK), mRNA [NM_152230]	253430
A_23_P115036	2.1	-1.0	-1.1	1.2	IPO13	Homo sapiens importin 13 (IPO13), mRNA [NM_014652]	9670
A_23_P205007	5.4	5.4	5.1	3.2	IPO5	Homo sapiens importin 5 (IPO5), mRNA [NM_002271]	3843
A_23_P97157	1.4	-2.3	-1.5	1.4	IPO9	Homo sapiens importin 9 (IPO9), mRNA [NM_018085]	55705
A_24_P855152	-2.5	1.0	-1.0	-1.0	IPP	intracisternal A particle-promoted polypeptide [Source:HGNC Symbol;Acc:6108] [ENST00000495072]	3652

A_24_P607880	1.0	-1.0	-1.0	-3.4	IPW	Homo sapiens imprinted in Prader-Willi syndrome (non-protein coding) (IPW), non-coding RNA [NR_023915]	3653
A_23_P143068	1.1	1.0	-3.9	1.0	IQCA1	Homo sapiens IQ motif containing with AAA domain 1 (IQCA1), mRNA [NM_024726]	79781
A_23_P73192	1.1	1.0	-6.0	1.1	IQCA1	Homo sapiens IQ motif containing with AAA domain 1 (IQCA1), mRNA [NM_024726]	79781
A_24_P266285	1.1	1.1	-1.0	-14.9	IQCE	Homo sapiens IQ motif containing E (IQCE), transcript variant 1, mRNA [NM_152558]	23288

A_23_P19852	-1.0	-1.1	-2.8	-1.1	IQCE	Homo sapiens IQ motif containing E (IQCE), transcript variant 1, mRNA [NM_152558]	23288
A_24_P145242	1.0	-2.5	-1.0	-1.1	IQCF1	Homo sapiens IQ motif containing F1, mRNA (cDNA clone IMAGE:5266078), complete cds. [BC034228]	132141
A_32_P121834	-38.8	1.4	1.5	1.0	IQCF1	Homo sapiens IQ motif containing F1 (IQCF1), mRNA [NM_152397]	132141
A_32_P138652	-2.7	-1.1	1.4	-1.0	IQCF4	Homo sapiens IQ motif containing F5 pseudogene (IQCF4), transcript variant 1, non-coding RNA [NR_038213]	100506840

A_32_P13756	-1.1	-6.6	1.9	1.1	IQCF6	Homo sapiens IQ motif containing F6 (IQCF6), mRNA [NM_001143833]	440956
A_23_P300076	-1.0	-1.1	-1.2	-5.7	IQCG	Homo sapiens IQ motif containing G (IQCG), transcript variant 1, mRNA [NM_032263]	84223
A_24_P275585	3.8	3.1	-2.0	2.2	IQCH	Homo sapiens IQ motif containing H (IQCH), transcript variant 1, mRNA [NM_001031715]	64799
A_24_P321919	-1.7	-2.2	1.1	1.7	IQGAP1	Homo sapiens IQ motif containing GTPase activating protein 1 (IQGAP1), mRNA [NM_003870]	8826

A_23_P329335	-2.6	1.3	-1.2	1.3	IQGAP3	Homo sapiens IQ motif containing GTPase activating protein 3 (IQGAP3), mRNA [NM_178229]	128239
A_23_P330788	-2.0	-1.2	1.8	1.3	IQSEC2	Homo sapiens IQ motif and Sec7 domain 2 (IQSEC2), transcript variant 2, mRNA [NM_015075]	23096
A_32_P195515	-1.7	-3.2	1.1	-1.8	IQSEC3	Homo sapiens IQ motif and Sec7 domain 3 (IQSEC3), transcript variant 2, mRNA [NM_015232]	440073
A_24_P203298	-9.6	-1.2	-1.1	-1.6	IQUB	Homo sapiens IQ motif and ubiquitin domain containing (IQUB), mRNA [NM_178827]	154865

A_23_P73780	1.2	-1.6	-2.0	1.2	IRAK1	Homo sapiens interleukin-1 receptor-associated kinase 1 (IRAK1), transcript variant 1, mRNA [NM_001569]	3654
A_32_P21474	1.0	-1.0	-4.2	-1.0	IRAK1BP1	Homo sapiens interleukin-1 receptor-associated kinase 1 binding protein 1 (IRAK1BP1), mRNA [NM_001010844]	134728
A_23_P80635	-1.6	1.4	2.4	-2.2	IRAK2	Homo sapiens interleukin-1 receptor-associated kinase 2 (IRAK2), mRNA [NM_001570]	3656

A_23_P162300	2.5	2.5	2.4	2.6	IRAK3	Homo sapiens interleukin-1 receptor-associated kinase 3 (IRAK3), transcript variant 1, mRNA [NM_007199]	11213
A_32_P83256	1.0	1.5	-6.8	1.0	IRAK3	Homo sapiens interleukin-1 receptor-associated kinase 3 (IRAK3), transcript variant 1, mRNA [NM_007199]	11213
A_23_P106497	1.8	2.0	1.3	2.2	IREB2	Homo sapiens iron-responsive element binding protein 2 (IREB2), mRNA [NM_004136]	3658
A_23_P41765	-1.9	-1.9	-2.2	-2.1	IRF1	Homo sapiens interferon regulatory factor 1 (IRF1), mRNA [NM_002198]	3659

A_23_P46588	1.1	3.4	1.1	1.1	IRF2BP2	Homo sapiens interferon regulatory factor 2 binding protein 2 (IRF2BP2), transcript variant 1, mRNA [NM_182972]	359948
A_24_P154214	9.5	9.0	8.4	6.3	IRF2BP2	Homo sapiens interferon regulatory factor 2 binding protein 2 (IRF2BP2), transcript variant 1, mRNA [NM_182972]	359948
A_32_P466514	-2.1	-1.7	-1.4	-2.1	IRF2BPL	Homo sapiens interferon regulatory factor 2 binding protein-like (IRF2BPL), mRNA [NM_024496]	64207

A_23_P156505	-1.6	1.2	-3.2	1.5	IRF4	Homo sapiens interferon regulatory factor 4 (IRF4), transcript variant 1, mRNA [NM_002460]	3662
A_23_P500271	3.1	2.3	1.3	2.4	IRF5	Homo sapiens interferon regulatory factor 5 (IRF5), transcript variant 3, mRNA [NM_001098627]	3663
A_24_P118892	-1.2	1.3	-3.4	-1.2	IRF7	Homo sapiens interferon regulatory factor 7 (IRF7), transcript variant b, mRNA [NM_004029]	3665
A_23_P65442	1.3	1.6	2.1	-1.5	IRF9	Homo sapiens interferon regulatory factor 9 (IRF9), mRNA [NM_006084]	10379

A_23_P67521	-1.0	-1.3	-3.4	1.6	IRGC	Homo sapiens immunity-related GTPase family, cinema (IRGC), mRNA [NM_019612]	56269
A_24_P225679	1.0	-2.4	-1.1	-1.1	IRS1	Homo sapiens insulin receptor substrate 1 (IRS1), mRNA [NM_005544]	3667
A_23_P133457	2.9	1.6	1.0	2.6	IRX1	Homo sapiens iroquois homeobox 1 (IRX1), mRNA [NM_024337]	79192
A_23_P152235	1.1	-1.0	-1.1	-2.7	IRX3	Homo sapiens iroquois homeobox 3 (IRX3), mRNA [NM_024336]	79191
A_24_P207195	2.8	2.3	2.4	1.2	IRX3	Homo sapiens iroquois homeobox 3 (IRX3), mRNA [NM_024336]	79191

A_24_P20795	-2.9	3.9	5.1	2.6	IRX4	Homo sapiens iroquois homeobox 4 (IRX4), mRNA [NM_016358]	50805
A_23_P9779	-1.1	-1.4	-4.9	-2.7	IRX5	Homo sapiens iroquois homeobox 5 (IRX5), transcript variant 1, mRNA [NM_005853]	10265
A_23_P73348	2.7	-1.7	2.0	1.5	IRX6	Homo sapiens iroquois homeobox 6 (IRX6), mRNA [NM_024335]	79190
A_23_P340158	-1.1	-4.4	-1.2	-1.0	ISCU	Homo sapiens iron-sulfur cluster scaffold homolog (E. coli) (ISCU), nuclear gene encoding mitochondrial protein, transcript variant 1, mRNA [NM_014301]	23479

A_23_P819	2.4	-1.0	3.0	2.1	ISG15	Homo sapiens ISG15 ubiquitin-like modifier (ISG15), mRNA [NM_005101]	9636
A_23_P126457	-3.5	-1.0	-1.1	-1.0	ISG20L2	Homo sapiens interferon stimulated exonuclease gene 20kDa-like 2 (ISG20L2), mRNA [NM_030980]	81875
A_23_P81529	5.9	5.5	1.7	2.7	ISL1	Homo sapiens ISL LIM homeobox 1 (ISL1), mRNA [NM_002202]	3670
A_23_P3312	-1.2	2.2	1.0	1.7	ISLR	Homo sapiens immunoglobulin superfamily containing leucine-rich repeat (ISLR), transcript variant 1, mRNA [NM_005545]	3671

A_32_P379467	-1.0	-1.1	2.6	-1.0	ISLR2	Homo sapiens immunoglobulin superfamily containing leucine-rich repeat 2 (ISLR2), transcript variant 2, mRNA [NM_020851]	57611
A_32_P186731	2.8	2.7	2.9	2.4	ISM1	Homo sapiens isthmin 1 homolog (zebrafish) (ISM1), mRNA [NM_080826]	140862
A_24_P99795	5.4	4.0	3.3	2.0	ISOC2	Homo sapiens isochorismatase domain containing 2 (ISOC2), nuclear gene encoding mitochondrial protein, transcript variant 2, mRNA [NM_024710]	79763

A_24_P339157	-1.8	-1.8	-3.2	-1.9	IST1	Homo sapiens increased sodium tolerance 1 homolog (yeast) (IST1), mRNA [NM_014761]	9798
A_32_P217140	1.0	-1.0	-5.3	-1.1	ISX	Homo sapiens intestine-specific homeobox (ISX), mRNA [NM_001008494]	91464
A_23_P155179	-1.0	-2.2	1.0	-1.2	ISY1	Homo sapiens ISY1 splicing factor homolog (S. cerevisiae) (ISY1), transcript variant 1, mRNA [NM_001199469]	57461

A_23_P124164	-1.7	-1.7	-2.2	-1.8	ISY1	Homo sapiens ISY1 splicing factor homolog (S. cerevisiae) (ISY1), transcript variant 2, mRNA [NM_020701]	57461
A_23_P5131	-1.7	-1.2	-2.4	-3.5	ISYNA1	Homo sapiens inositol-3-phosphate synthase 1 (ISYNA1), transcript variant 1, mRNA [NM_016368]	51477
A_24_P139191	1.6	1.5	-2.6	-1.1	ITCH	Homo sapiens itchy E3 ubiquitin protein ligase homolog (mouse) (ITCH), mRNA [NM_031483]	83737
A_23_P206022	-1.8	3.2	3.5	1.0	ITGA11	Homo sapiens integrin, alpha 11 (ITGA11), mRNA [NM_001004439]	22801

A_32_P208076	-3.1	1.7	1.1	1.1	ITGA2	Homo sapiens integrin, alpha 2 (CD49B, alpha 2 subunit of VLA-2 receptor) (ITGA2), mRNA [NM_002203]	3673
A_23_P77971	-1.1	-4.8	1.7	-1.1	ITGA2B	Homo sapiens integrin, alpha 2b (platelet glycoprotein IIb of IIb/IIIa complex, antigen CD41) (ITGA2B), mRNA [NM_000419]	3674
A_23_P36562	-1.0	-11.2	-1.7	-2.4	ITGA5	Homo sapiens integrin, alpha 5 (fibronectin receptor, alpha polypeptide) (ITGA5), mRNA [NM_002205]	3678

A_23_P128084	2.4	3.9	-1.1	11.7	ITGA7	Homo sapiens integrin, alpha 7 (ITGA7), transcript variant 2, mRNA [NM_002206]	3679
A_24_P607904	-1.0	-1.0	-2.8	-2.3	ITGA9	integrin, alpha 9 [Source:HGNC Symbol;Acc:6145] [ENST00000264741]	3680
A_23_P218375	1.2	2.7	2.3	2.4	ITGAE	Homo sapiens integrin, alpha E (antigen CD103, human mucosal lymphocyte antigen 1; alpha polypeptide) (ITGAE), mRNA [NM_002208]	3682

A_24_P344251	2.1	1.4	1.0	-1.0	ITGAX	Homo sapiens integrin, alpha X (complement component 3 receptor 4 subunit) (ITGAX), mRNA [NM_000887]	3687
A_23_P104199	-1.7	-2.0	-1.7	1.1	ITGB1	Homo sapiens integrin, beta 1 (fibronectin receptor, beta polypeptide, antigen CD29 includes MDF2, MSK12) (ITGB1), transcript variant 1E, mRNA [NM_133376]	3688

A_32_P95397	-1.0	-1.0	-23.2	-1.1	ITGB1	Homo sapiens integrin, beta 1 (fibronectin receptor, beta polypeptide, antigen CD29 includes MDF2, MSK12) (ITGB1), transcript variant 1A, mRNA [NM_002211]	3688
A_23_P430411	-1.0	-1.0	2.1	1.4	ITGB2	integrin, beta 2 (complement component 3 receptor 3 and 4 subunit) [Source:HGNC Symbol;Acc:6155] [ENST00000545414]	3689
A_24_P929003	-2.5	1.1	1.1	1.0	ITGB3	Homo sapiens integrin beta 3 mRNA, partial cds, alternatively spliced. [S70348]	3690

A_23_P66355	-2.1	2.1	1.3	1.5	ITGB4	Homo sapiens integrin, beta 4 (ITGB4), transcript variant 1, mRNA [NM_000213]	3691
A_23_P154217	2.3	2.3	5.4	2.5	ITGB6	Homo sapiens integrin, beta 6 (ITGB6), mRNA [NM_000888]	3694
A_23_P76529	1.1	1.6	1.5	-2.9	ITGB7	Homo sapiens integrin, beta 7 (ITGB7), mRNA [NM_000889]	3695
A_23_P123060	-1.4	1.0	-1.0	-15.5	ITGB8	Homo sapiens integrin, beta 8 (ITGB8), mRNA [NM_002214]	3696
A_23_P408363	-2.1	-1.3	1.1	-14.2	ITGBL1	integrin, beta-like 1 (with EGF-like repeat domains) [Source:HGNC Symbol;Acc:6164] [ENST00000490242]	9358

A_23_P18223	1.4	1.7	2.1	1.9	ITIH1	Homo sapiens inter-alpha-trypsin inhibitor heavy chain 1 (ITIH1), transcript variant 1, mRNA [NM_002215]	3697
A_23_P6822	1.3	-2.3	1.4	-1.2	ITIH3	Homo sapiens inter-alpha-trypsin inhibitor heavy chain 3 (ITIH3), mRNA [NM_002217]	3699
A_23_P109881	1.9	1.4	-1.1	2.2	ITIH4	Homo sapiens inter-alpha-trypsin inhibitor heavy chain family, member 4 (ITIH4), transcript variant 1, mRNA [NM_002218]	3700

A_23_P411993	-1.0	-2.3	1.2	1.3	ITIH5	Homo sapiens inter-alpha-trypsin inhibitor heavy chain family, member 5 (ITIH5), transcript variant 1, mRNA [NM_030569]	80760
A_24_P220855	1.1	2.0	2.0	2.8	ITIH5	Homo sapiens inter-alpha-trypsin inhibitor heavy chain family, member 5 (ITIH5), transcript variant 3, mRNA [NM_001001851]	80760
A_24_P292253	1.0	-1.0	-2.9	1.0	ITIH5	Homo sapiens inter-alpha-trypsin inhibitor heavy chain family, member 5 (ITIH5), transcript variant 1, mRNA [NM_030569]	80760

A_32_P44878	2.7	2.5	-1.0	-43.2	ITIH5	Homo sapiens inter-alpha-trypsin inhibitor heavy chain family, member 5 (ITIH5), transcript variant 1, mRNA [NM_030569]	80760
A_23_P354146	-4.4	1.1	1.1	1.1	ITK	Homo sapiens IL2-inducible T-cell kinase (ITK), mRNA [NM_005546]	3702
A_23_P171074	-1.0	1.1	-2.3	1.0	ITM2A	Homo sapiens integral membrane protein 2A (ITM2A), transcript variant 1, mRNA [NM_004867]	9452
A_24_P381604	-1.5	-3.0	-3.4	1.0	ITM2B	Homo sapiens integral membrane protein 2B (ITM2B), mRNA [NM_021999]	9445

A_24_P379820	-2.5	-4.5	-1.3	-2.9	ITM2C	Homo sapiens integral membrane protein 2C (ITM2C), transcript variant 1, mRNA [NM_030926]	81618
A_24_P167683	-2.0	-1.6	-2.4	-1.5	ITPK1	Homo sapiens inositol-tetrakisphosphate 1-kinase (ITPK1), transcript variant 1, mRNA [NM_014216]	3705
A_24_P119545	1.1	1.1	1.1	2.3	ITPKB	inositol-trisphosphate 3-kinase B [Source:HGNC Symbol;Acc:6179] [ENST00000366784]	3707
A_23_P208369	1.2	2.4	1.8	1.7	ITPKC	Homo sapiens inositol-trisphosphate 3-kinase C (ITPKC), mRNA [NM_025194]	80271

A_23_P340333	2.5	1.9	2.3	1.0	ITPRIP	Homo sapiens inositol 1,4,5-trisphosphate receptor interacting protein (ITPRIP), mRNA [NM_033397]	85450
A_24_P111061	1.0	-3.4	1.0	1.0	ITSN1	Homo sapiens intersectin 1 (SH3 domain protein) (ITSN1), transcript variant 2, mRNA [NM_001001132]	6453
A_23_P252962	-5.7	-1.2	-2.8	1.1	ITSN1	Homo sapiens intersectin 1 (SH3 domain protein) (ITSN1), transcript variant 2, mRNA [NM_001001132]	6453
A_24_P239988	-1.5	-1.4	-3.0	-1.4	ITSN2	Homo sapiens intersectin 2 (ITSN2), transcript variant 1, mRNA [NM_006277]	50618

A_23_P434347	1.5	2.5	-3.2	1.0	ITSN2	Homo sapiens intersectin 2 (ITSN2), transcript variant 2, mRNA [NM_147152]	50618
A_23_P353524	-1.3	-2.3	-5.5	-5.1	IVL	Homo sapiens involucrin (IVL), mRNA [NM_005547]	3713
A_23_P165574	1.3	-8.5	1.0	1.4	IWS1	Homo sapiens IWS1 homolog (S. cerevisiae) (IWS1), mRNA [NM_017969]	55677
A_24_P340247	1.1	-1.0	-1.1	-3.5	IZUMO1	Homo sapiens izumo sperm-egg fusion 1 (IZUMO1), mRNA [NM_182575]	284359
A_23_P101521	-1.1	-1.1	-1.2	-29.7	IZUMO2	Homo sapiens IZUMO family member 2 (IZUMO2), mRNA [NM_152358]	126123

A_23_P50674	2.7	1.7	5.7	1.6	IZUMO4	Homo sapiens IZUMO family member 4 (IZUMO4), transcript variant 1, mRNA [NM_001031735]	113177
A_23_P329112	1.1	1.1	-9.1	-1.0	JAK3	Homo sapiens Janus kinase 3 (JAK3), mRNA [NM_000215]	3718
A_24_P308096	5.3	9.0	1.1	1.5	JAK3	Homo sapiens Janus kinase 3 (JAK3), mRNA [NM_000215]	3718
A_23_P144274	-1.0	-1.0	-2.8	-1.4	JAKMIP1	Homo sapiens janus kinase and microtubule interacting protein 1 (JAKMIP1), transcript variant 2, mRNA [NM_144720]	152789

A_32_P310335	5.1	4.9	4.7	4.7	JAM2	junctional adhesion molecule 2 [Source:HGNC Symbol;Acc:14686] [ENST00000480456]	58494
A_23_P120667	1.9	2.7	2.1	1.1	JAM2	Homo sapiens junctional adhesion molecule 2 (JAM2), mRNA [NM_021219]	58494
A_23_P359897	-3.6	-1.0	-1.0	-1.0	JHDM1D	Homo sapiens jumonji C domain containing histone demethylase 1 homolog D (S. cerevisiae) (JHDM1D), mRNA [NM_030647]	80853

A_23_P205584	1.0	1.2	-5.4	1.0	JKAMP	Homo sapiens JNK1/MAPK8-associated membrane protein (JKAMP), transcript variant 1, mRNA [NM_016475]	51528
A_23_P300301	-2.4	1.1	-1.0	-1.0	JMJD1C	Homo sapiens jumonji domain containing 1C (JMJD1C), transcript variant 2, mRNA [NM_004241]	221037
A_23_P115523	-10.8	1.1	-1.3	-1.2	JMJD4	Homo sapiens jumonji domain containing 4 (JMJD4), transcript variant 1, mRNA [NM_023007]	65094
A_23_P146865	2.3	1.4	-1.0	1.5	JMJD5	Homo sapiens cDNA FLJ61151 complete cds. [AK298410]	79831

A_23_P311616	-5.4	-2.2	-1.9	-2.3	JMJD6	Homo sapiens jumonji domain containing 6 (JMJD6), transcript variant 2, mRNA [NM_015167]	23210
A_23_P403424	3.3	3.3	2.9	4.1	JMJD7-PLA2G	Homo sapiens JMJD7-PLA2G4B readthrough (JMJD7-PLA2G4B), transcript variant 1, mRNA [NM_005090]	8681
A_23_P15073	-1.6	-2.7	-1.0	-2.2	JMJD8	Homo sapiens jumonji domain containing 8 (JMJD8), mRNA [NM_001005920]	339123
A_32_P176550	-1.1	-2.9	-2.2	-2.2	JMY	Homo sapiens junction mediating and regulatory protein, p53 cofactor (JMY), mRNA [NM_152405]	133746

A_23_P67321	-1.7	1.2	-2.0	1.0	JOSD2	Homo sapiens Josephin domain containing 2 (JOSD2), mRNA [NM_138334]	126119
A_24_P194199	1.1	-4.1	-1.5	1.2	JPH2	Homo sapiens junctophilin 2 (JPH2), transcript variant 1, mRNA [NM_020433]	57158
A_24_P150791	1.4	13.2	9.4	9.7	JPH3	Homo sapiens junctophilin 3 (JPH3), mRNA [NM_020655]	57338
A_24_P938403	-1.4	-1.4	-19.3	-1.4	JPH3	Homo sapiens junctophilin 3, mRNA (cDNA clone IMAGE:3867947), complete cds. [BC008690]	57338

A_23_P202737	2.1	1.1	2.1	1.4	JRKL	Homo sapiens jerky homolog-like (mouse) (JRKL), mRNA [NM_003772]	8690
A_23_P4821	2.1	1.3	1.5	-1.0	JUNB	Homo sapiens jun B proto-oncogene (JUNB), mRNA [NM_002229]	3726
A_24_P143301	-3.2	-1.7	-1.1	-1.6	KAAG1	Homo sapiens kidney associated antigen 1 (KAAG1), mRNA [NM_181337]	353219

A_24_P410797	2.1	2.0	1.9	2.5	KALRN	Homo sapiens cDNA FLJ43991 fis, clone TESTI4019843, highly similar to Rattus norvegicus huntingtin-associated protein interacting protein (duo) (Hapip). [AK125979]	8997
A_23_P500130	-1.0	-5.7	-1.1	-1.0	KANK1	Homo sapiens KN motif and ankyrin repeat domains 1 (KANK1), transcript variant 2, mRNA [NM_153186]	23189
A_24_P7693	-1.0	-1.5	1.1	-2.1	KANK3	Homo sapiens KN motif and ankyrin repeat domains 3 (KANK3), mRNA [NM_198471]	256949

A_24_P67929	1.0	-1.4	-1.4	-2.8	KANSL2	Homo sapiens chromosome 12 open reading frame 41 (C12orf41), mRNA [NM_017822]	54934
A_23_P66608	1.1	1.1	-2.3	1.0	KAT2A	Homo sapiens K(lysine) acetyltransferase 2A (KAT2A), mRNA [NM_021078]	2648
A_23_P138849	-2.6	-10.2	-6.6	-5.6	KAT5	Homo sapiens K(lysine) acetyltransferase 5 (KAT5), transcript variant 2, mRNA [NM_006388]	10524
A_24_P928986	1.0	-1.0	-2.9	-1.3	KAT6A	Homo sapiens cDNA FLJ14455 fis, clone HEMBB1001908. [AK027361]	7994

A_23_P388855	-10.4	-1.0	-1.1	-1.1	KAT6B	Homo sapiens K(lysine) acetyltransferase 6B (KAT6B), mRNA [NM_012330]	23522
A_23_P388851	-3.0	1.9	1.2	1.2	KAT6B	Homo sapiens K(lysine) acetyltransferase 6B (KAT6B), mRNA [NM_012330]	23522
A_24_P927976	-1.0	-3.0	-1.4	1.0	KATNAL2	Homo sapiens mRNA; cDNA DKFZp667C165 (from clone DKFZp667C165). [AL512748]	83473
A_24_P331830	1.2	1.2	-11.1	1.2	KAZN	Homo sapiens kazrin, periplakin interacting protein (KAZN), transcript variant A, mRNA [NM_015209]	23254

A_24_P246573	1.1	1.0	-1.0	-3.1	KAZN	Homo sapiens kazrin, periplakin interacting protein (KAZN), transcript variant A, mRNA [NM_015209]	23254
A_23_P94319	-5.4	1.1	1.0	1.1	KBTBD11	Homo sapiens kelch repeat and BTB (POZ) domain containing 11 (KBTBD11), mRNA [NM_014867]	9920
A_32_P88240	5.8	1.7	1.6	1.0	KBTBD12	Homo sapiens kelch repeat and BTB (POZ) domain containing 12 (KBTBD12), mRNA [NM_207335]	166348

A_23_P70951	-1.0	4.4	-1.0	-1.0	KBTBD2	Homo sapiens kelch repeat and BTB (POZ) domain containing 2 (KBTBD2), mRNA [NM_015483]	25948
A_23_P202696	1.1	-5.2	1.1	1.0	KBTBD4	Homo sapiens kelch repeat and BTB (POZ) domain containing 4 (KBTBD4), transcript variant 2, mRNA [NM_016506]	55709
A_23_P12950	-16.9	1.1	1.1	1.1	KBTBD4	Homo sapiens kelch repeat and BTB (POZ) domain containing 4 (KBTBD4), transcript variant 2, mRNA [NM_016506]	55709

A_23_P392435	-1.7	-1.7	-2.6	-1.7	KBTBD6	Homo sapiens kelch repeat and BTB (POZ) domain containing 6 (KBTBD6), mRNA [NM_152903]	89890
A_23_P25605	-1.2	3.8	1.5	2.9	KBTBD7	Homo sapiens kelch repeat and BTB (POZ) domain containing 7 (KBTBD7), mRNA [NM_032138]	84078
A_23_P431252	-22.1	1.0	-1.9	-1.8	KBTBD8	Homo sapiens kelch repeat and BTB (POZ) domain containing 8 (KBTBD8), mRNA [NM_032505]	84541

A_23_P48229	4.9	4.7	4.2	3.1	KCNA1	Homo sapiens potassium voltage-gated channel, shaker-related subfamily, member 1 (episodic ataxia with myokymia) (KCNA1), mRNA [NM_000217]	3736
A_23_P126528	-2.0	-6.3	-5.6	-1.7	KCNA10	Homo sapiens potassium voltage-gated channel, shaker-related subfamily, member 10 (KCNA10), mRNA [NM_005549]	3744
A_23_P417173	-2.1	-2.2	-2.2	-2.2	KCNA5	Homo sapiens potassium voltage-gated channel, shaker-related subfamily, member 5 (KCNA5), mRNA [NM_002234]	3741

A_24_P87686	-1.2	-1.1	-2.6	-1.5	KCNA7	Homo sapiens potassium voltage-gated channel, shaker-related subfamily, member 7 (KCNA7), mRNA [NM_031886]	3743
A_24_P151	-1.2	-1.2	-2.1	-1.4	KCNAB2	Homo sapiens potassium voltage-gated channel, shaker-related subfamily, beta member 2 (KCNAB2), transcript variant 1, mRNA [NM_003636]	8514
A_23_P501232	1.2	2.7	1.3	-1.6	KCNAB2	Homo sapiens potassium voltage-gated channel, shaker-related subfamily, beta member 2 (KCNAB2), transcript variant 1, mRNA [NM_003636]	8514

A_23_P139841	2.9	2.9	1.5	2.2	KCNC2	Homo sapiens potassium voltage-gated channel, Shaw-related subfamily, member 2 (KCNC2), transcript variant 2, mRNA [NM_139137]	3747
A_23_P101516	1.2	2.9	1.5	2.3	KCNC3	Homo sapiens potassium voltage-gated channel, Shaw-related subfamily, member 3 (KCNC3), mRNA [NM_004977]	3748
A_32_P140268	-2.7	1.0	-1.0	-1.0	KCND3	potassium voltage-gated channel, Shal-related subfamily, member 3 [Source:HGNC Symbol;Acc:6239] [ENST00000369697]	3752

A_24_P941339	1.7	1.7	-14.6	1.0	KCNE1	Homo sapiens potassium voltage-gated channel, Isk-related family, member 1 (KCNE1), transcript variant 2, mRNA [NM_000219]	3753
A_23_P377882	3.5	1.8	4.8	2.0	KCNH2	Homo sapiens potassium voltage-gated channel, subfamily H (eag-related), member 2 (KCNH2), transcript variant 2, mRNA [NM_172056]	3757
A_24_P99145	-2.7	-1.3	1.2	-1.1	KCNH5	Homo sapiens potassium voltage-gated channel, subfamily H (eag-related), member 5 (KCNH5), transcript variant 2, mRNA [NM_172376]	27133

A_23_P83558	-1.6	-2.5	-1.5	-1.5	KCNH6	Homo sapiens potassium voltage-gated channel, subfamily H (eag-related), member 6 (KCNH6), transcript variant 2, mRNA [NM_173092]	81033
A_23_P30554	-6.9	-1.6	-4.0	-4.1	KCNIP1	Homo sapiens Kv channel interacting protein 1 (KCNIP1), transcript variant 1, mRNA [NM_001034837]	30820
A_24_P12998	-1.0	-1.1	1.4	-2.4	KCNIP2	Homo sapiens Kv channel interacting protein 2 (KCNIP2), transcript variant 7, mRNA [NM_173197]	30819

A_23_P202918	1.4	1.5	2.2	-6.6	KCNJ1	Homo sapiens potassium inwardly-rectifying channel, subfamily J, member 1 (KCNJ1), transcript variant rom-k5, mRNA [NM_153767]	3758
A_24_P387875	1.8	-2.7	1.7	1.0	KCNJ10	Homo sapiens potassium inwardly-rectifying channel, subfamily J, member 10 (KCNJ10), mRNA [NM_002241]	3766
A_24_P339429	1.1	1.1	-2.1	1.0	KCNJ12	Homo sapiens potassium inwardly-rectifying channel, subfamily J, member 12 (KCNJ12), mRNA [NM_021012]	3768

A_23_P312752	1.7	1.7	2.1	1.8	KCNJ13	Homo sapiens potassium inwardly-rectifying channel, subfamily J, member 13 (KCNJ13), transcript variant 1, mRNA [NM_002242]	3769
A_23_P130764	-1.6	2.4	2.4	1.9	KCNJ14	Homo sapiens potassium inwardly-rectifying channel, subfamily J, member 14 (KCNJ14), transcript variant 2, mRNA [NM_170720]	3770
A_23_P17655	-26.2	-1.1	-1.2	-1.1	KCNJ15	Homo sapiens potassium inwardly-rectifying channel, subfamily J, member 15 (KCNJ15), transcript variant 1, mRNA [NM_170736]	3772

A_23_P501193	1.2	2.0	-4.5	1.1	KCNJ16	Homo sapiens potassium inwardly-rectifying channel, subfamily J, member 16 (KCNJ16), transcript variant 2, mRNA [NM_170741]	3773
A_23_P329261	3.0	4.0	2.8	1.0	KCNJ2	Homo sapiens potassium inwardly-rectifying channel, subfamily J, member 2 (KCNJ2), mRNA [NM_000891]	3759
A_23_P153986	-1.6	1.5	-1.2	2.0	KCNJ3	Homo sapiens potassium inwardly-rectifying channel, subfamily J, member 3 (KCNJ3), mRNA [NM_002239]	3760

A_24_P6125	1.0	1.0	-1.0	-2.1	KCNJ4	Homo sapiens potassium inwardly-rectifying channel, subfamily J, member 4 (KCNJ4), transcript variant 1, mRNA [NM_152868]	3761
A_24_P309521	-1.0	-15.3	-1.0	1.2	KCNJ5	Homo sapiens potassium inwardly-rectifying channel, subfamily J, member 5 (KCNJ5), mRNA [NM_000890]	3762
A_23_P64879	4.0	1.3	2.4	6.1	KCNJ8	Homo sapiens potassium inwardly-rectifying channel, subfamily J, member 8 (KCNJ8), mRNA [NM_004982]	3764

A_23_P65629	1.1	-3.1	1.1	1.0	KCNK10	Homo sapiens potassium channel, subfamily K, member 10 (KCNK10), transcript variant 1, mRNA [NM_021161]	54207
A_23_P108883	2.2	2.5	2.1	3.1	KCNK12	Homo sapiens potassium channel, subfamily K, member 12 (KCNK12), mRNA [NM_022055]	56660
A_23_P210451	1.7	1.7	-2.1	1.4	KCNK15	Homo sapiens potassium channel, subfamily K, member 15 (KCNK15), mRNA [NM_022358]	60598

A_24_P112032	1.1	-1.2	-1.0	-2.7	KCNK17	Homo sapiens potassium channel, subfamily K, member 17 (KCNK17), transcript variant 1, mRNA [NM_031460]	89822
A_23_P91104	1.1	-1.3	-1.2	-3.5	KCNK3	Homo sapiens potassium channel, subfamily K, member 3 (KCNK3), mRNA [NM_002246]	3777
A_32_P805871	-3.0	1.0	1.3	-1.4	KCNK4	Homo sapiens potassium channel, subfamily K, member 4 (KCNK4), mRNA [NM_033310]	50801
A_23_P319423	1.9	2.5	2.5	2.8	KCNK5	Homo sapiens potassium channel, subfamily K, member 5 (KCNK5), mRNA [NM_003740]	8645

A_23_P50591	-1.1	-2.8	-1.2	-1.1	KCNK6	Homo sapiens potassium channel, subfamily K, member 6 (KCNK6), mRNA [NM_004823]	9424
A_23_P86874	3.0	1.8	-1.6	2.5	KCNK7	Homo sapiens potassium channel, subfamily K, member 7 (KCNK7), transcript variant A, mRNA [NM_033347]	10089
A_23_P111978	1.3	-2.2	-1.9	-1.1	KCNK9	Homo sapiens potassium channel, subfamily K, member 9 (KCNK9), mRNA [NM_016601]	51305

A_32_P181222	-10.1	-1.2	-1.3	-2.4	KCNMA1	Homo sapiens potassium large conductance calcium-activated channel, subfamily M, alpha member 1 (KCNMA1), transcript variant 2, mRNA [NM_002247]	3778
A_32_P192692	3.2	2.4	1.2	2.5	KCNMA1	Homo sapiens potassium large conductance calcium-activated channel, subfamily M, alpha member 1 (KCNMA1), transcript variant 1, mRNA [NM_001014797]	3778

A_24_P156490	1.7	2.0	1.3	1.5	KCNMA1	Homo sapiens potassium large conductance calcium-activated channel, subfamily M, alpha member 1 (KCNMA1), transcript variant 2, mRNA [NM_002247]	3778
A_24_P916196	-1.0	-37.3	-1.0	-1.1	KCNMA1	Homo sapiens maxi-K channel HSLO mRNA, partial cds. [AF349445]	3778
A_24_P61150	1.0	-3.0	1.2	-1.8	KCNMA1	Homo sapiens potassium large conductance calcium-activated channel, subfamily M, alpha member 1 (KCNMA1), transcript variant 2, mRNA [NM_002247]	3778

A_24_P206121	1.7	4.6	2.4	2.1	KCNMB1	Homo sapiens potassium large conductance calcium-activated channel, subfamily M, beta member 1 (KCNMB1), mRNA [NM_004137]	3779
A_23_P67529	1.1	2.0	1.5	1.0	KCNN4	Homo sapiens potassium intermediate/small conductance calcium-activated channel, subfamily N, member 4 (KCNN4), mRNA [NM_002250]	3783

A_23_P210400	-1.5	-1.5	-1.5	-4.8	KCNQ2	Homo sapiens potassium voltage-gated channel, KQT-like subfamily, member 2, mRNA (cDNA clone IMAGE:4154700). [BC020384]	3785
A_23_P123393	-2.3	-1.5	-3.0	1.1	KCNQ3	Homo sapiens potassium voltage-gated channel, KQT-like subfamily, member 3 (KCNQ3), transcript variant 1, mRNA [NM_004519]	3786
A_23_P34424	2.0	2.1	-1.4	-1.4	KCNQ4	Homo sapiens potassium voltage-gated channel, KQT-like subfamily, member 4 (KCNQ4), transcript variant 1, mRNA [NM_004700]	9132

A_23_P167841	-1.6	2.4	2.4	1.5	KCNQ5	Homo sapiens potassium voltage-gated channel, KQT-like subfamily, member 5 (KCNQ5), transcript variant 1, mRNA [NM_019842]	56479
A_23_P394377	2.5	1.2	2.3	1.4	KCNRG	Homo sapiens potassium channel regulator (KCNRG), transcript variant 2, mRNA [NM_199464]	283518
A_23_P321846	14.3	13.8	16.9	14.2	KCNS1	Homo sapiens potassium voltage-gated channel, delayed-rectifier, subfamily S, member 1 (KCNS1), mRNA [NM_002251]	3787

A_24_P46911	-44.2	1.1	1.0	1.0	KCNS2	Homo sapiens potassium voltage-gated channel, delayed-rectifier, subfamily S, member 2 (KCNS2), mRNA [NM_020697]	3788
A_23_P120103	-1.0	-2.2	-1.7	-1.3	KCNS3	Homo sapiens potassium voltage-gated channel, delayed-rectifier, subfamily S, member 3 (KCNS3), mRNA [NM_002252]	3790
A_24_P155169	1.3	1.9	1.6	2.0	KCNT1	Homo sapiens potassium channel, subfamily T, member 1 (KCNT1), mRNA [NM_020822]	57582

A_24_P246278	-1.5	-1.0	-2.6	-1.5	KCP	Homo sapiens kielin/chordin-like protein (KCP), transcript variant 2, mRNA [NM_199349]	375616
A_23_P130343	-2.8	-1.0	-1.1	-1.1	KCTD1	Homo sapiens potassium channel tetramerisation domain containing 1 (KCTD1), transcript variant 2, mRNA [NM_198991]	284252
A_24_P643376	2.0	-4.9	-1.1	-1.5	KCTD1	Homo sapiens potassium channel tetramerisation domain containing 1 (KCTD1), transcript variant 3, mRNA [NM_001142730]	284252

A_23_P44849	-2.4	-8.8	-2.2	-2.3	KCTD13	Homo sapiens potassium channel tetramerisation domain containing 13 (KCTD13), mRNA [NM_178863]	253980
A_23_P162165	-1.0	-2.3	-1.1	-1.1	KCTD14	Homo sapiens potassium channel tetramerisation domain containing 14 (KCTD14), mRNA [NM_023930]	65987
A_24_P22892	1.0	-11.4	-1.1	-1.1	KCTD15	Homo sapiens potassium channel tetramerisation domain containing 15 (KCTD15), transcript variant 1, mRNA [NM_024076]	79047

A_24_P307808	1.4	1.3	-2.0	1.2	KCTD16	Homo sapiens potassium channel tetramerisation domain containing 16 (KCTD16), mRNA [NM_020768]	57528
A_23_P402157	3.1	-2.2	1.5	-1.2	KCTD17	Homo sapiens potassium channel tetramerisation domain containing 17 (KCTD17), mRNA [NM_024681]	79734
A_23_P48327	-2.9	2.2	1.9	1.3	KCTD4	Homo sapiens potassium channel tetramerisation domain containing 4 (KCTD4), mRNA [NM_198404]	386618

A_23_P385246	-2.2	1.3	1.1	1.2	KCTD6	Homo sapiens potassium channel tetramerisation domain containing 6 (KCTD6), transcript variant 1, mRNA [NM_153331]	200845
A_23_P157380	3.2	8.6	2.7	4.9	KCTD7	Homo sapiens potassium channel tetramerisation domain containing 7 (KCTD7), transcript variant 1, mRNA [NM_153033]	154881
A_24_P298334	-1.0	-4.0	-1.1	-1.0	KCTD9	Homo sapiens potassium channel tetramerisation domain containing 9 (KCTD9), mRNA [NM_017634]	54793

A_23_P388200	-1.0	-2.2	-1.1	-1.1	KDELC2	Homo sapiens KDEL (Lys-Asp-Glu-Leu) containing 2 (KDELC2), mRNA [NM_153705]	143888
A_23_P4913	1.3	1.9	2.6	-8.5	KDELR1	Homo sapiens KDEL (Lys-Asp-Glu-Leu) endoplasmic reticulum protein retention receptor 1 (KDELR1), mRNA [NM_006801]	10945
A_24_P42517	-1.9	1.0	-2.1	-1.3	KDELR2	Homo sapiens KDEL (Lys-Asp-Glu-Leu) endoplasmic reticulum protein retention receptor 2 (KDELR2), transcript variant 1, mRNA [NM_006854]	11014

A_23_P211504	-5.4	-1.2	-1.8	1.2	KDELR3	Homo sapiens KDEL (Lys-Asp-Glu-Leu) endoplasmic reticulum protein retention receptor 3 (KDELR3), transcript variant 2, mRNA [NM_016657]	11015
A_23_P338401	1.6	1.6	-3.9	-1.0	KDM1B	Homo sapiens lysine (K)-specific demethylase 1B (KDM1B), mRNA [NM_153042]	221656
A_23_P83917	-2.3	-2.7	-3.7	-1.5	KDM2A	Homo sapiens lysine (K)-specific demethylase 2A (KDM2A), transcript variant 1, mRNA [NM_012308]	22992

A_23_P204406	13.2	16.3	23.4	13.8	KDM5A	Homo sapiens lysine (K)-specific demethylase 5A (KDM5A), mRNA [NM_001042603]	5927
A_23_P204408	-1.0	-1.1	-3.5	-1.1	KDM5A	Homo sapiens lysine (K)-specific demethylase 5A (KDM5A), mRNA [NM_001042603]	5927
A_32_P70724	1.1	1.1	2.0	-1.0	KDM5B	Homo sapiens lysine (K)-specific demethylase 5B (KDM5B), mRNA [NM_006618]	10765
A_23_P1038	2.9	5.4	1.5	1.7	KDM5B	Homo sapiens lysine (K)-specific demethylase 5B (KDM5B), mRNA [NM_006618]	10765

A_23_P252758	-1.2	2.0	1.9	1.5	KEL	Homo sapiens Kell blood group, metallo-endopeptidase (KEL), mRNA [NM_000420]	3792
A_24_P261750	1.6	2.5	2.3	2.4	KERA	Homo sapiens keratocan (KERA), mRNA [NM_007035]	11081
A_32_P86905	3.1	3.4	2.6	1.0	KGFLP2	Homo sapiens keratinocyte growth factor-like protein 2 (KGFLP2), non-coding RNA [NR_003670]	654466
A_23_P366453	-6.8	-1.0	-1.0	1.0	KHDRBS2	Homo sapiens KH domain containing, RNA binding, signal transduction associated 2 (KHDRBS2), mRNA [NM_152688]	202559

A_23_P257335	-1.1	5.9	2.7	2.5	KHDRBS3	Homo sapiens KH domain containing, RNA binding, signal transduction associated 3 (KHDRBS3), mRNA [NM_006558]	10656
A_24_P205268	-1.4	1.4	-2.2	-1.0	KHNYN	Homo sapiens KH and NYN domain containing (KHNYN), mRNA [NM_015299]	23351
A_23_P16157	1.8	2.2	1.1	-2.7	KHSRP	Homo sapiens KH-type splicing regulatory protein (KHSRP), mRNA [NM_003685]	8570
A_24_P134235	1.1	-4.1	1.7	1.4	KHSRP	Homo sapiens KH-type splicing regulatory protein (KHSRP), mRNA [NM_003685]	8570

A_23_P386356	1.1	1.1	-1.0	-13.9	KIF12	Homo sapiens kinesin family member 12 (KIF12), mRNA [NM_138424]	113220
A_23_P95441	-2.1	-2.9	-1.1	-1.5	KIF13B	Homo sapiens kinesin family member 13B (KIF13B), mRNA [NM_015254]	23303
A_23_P147388	2.1	2.8	-1.2	1.5	KIF13B	Homo sapiens kinesin family member 13B (KIF13B), mRNA [NM_015254]	23303
A_23_P400515	2.8	2.7	2.1	4.6	KIF17	Homo sapiens kinesin family member 17 (KIF17), transcript variant 1, mRNA [NM_020816]	57576

A_24_P233014	1.6	1.4	-3.2	1.6	KIF19	Homo sapiens kinesin family member 19 (KIF19), mRNA [NM_153209]	124602
A_32_P100830	1.6	-1.1	2.3	1.7	KIF19	Homo sapiens kinesin family member 19 (KIF19), mRNA [NM_153209]	124602
A_23_P395460	-1.2	-2.9	-1.3	-1.6	KIF1A	Homo sapiens kinesin family member 1A (KIF1A), transcript variant 2, mRNA [NM_004321]	547
A_23_P331638	-2.5	-4.2	-1.1	-1.0	KIF1B	Homo sapiens kinesin family member 1B (KIF1B), transcript variant 1, mRNA [NM_015074]	23095

A_24_P649624	-3.4	-1.1	-1.2	-2.1	KIF1B	Homo sapiens kinesin family member 1B (KIF1B), transcript variant 2, mRNA [NM_183416]	23095
A_23_P126888	2.3	-1.6	2.8	2.2	KIF21B	Homo sapiens kinesin family member 21B (KIF21B), transcript variant 2, mRNA [NM_017596]	23046
A_23_P71741	2.1	-1.5	2.6	1.4	KIF27	Homo sapiens kinesin family member 27 (KIF27), mRNA [NM_017576]	55582
A_23_P34788	1.2	1.3	2.3	1.1	KIF2C	Homo sapiens kinesin family member 2C (KIF2C), mRNA [NM_006845]	11004

A_23_P17560	1.1	-2.7	-1.0	-1.0	KIF3B	Homo sapiens kinesin family member 3B (KIF3B), mRNA [NM_004798]	9371
A_24_P227375	-2.2	-2.2	-3.6	-2.4	KIF5A	kinesin family member 5A [Source:HGNC Symbol;Acc:6323] [ENST00000455537]	3798
A_24_P203689	1.1	1.1	-2.5	-1.0	KIF7	Homo sapiens kinesin family member 7 (KIF7), mRNA [NM_198525]	374654
A_23_P54576	-1.1	3.6	1.7	1.1	KIFC3	Homo sapiens kinesin family member C3 (KIFC3), transcript variant 1, mRNA [NM_005550]	3801

A_23_P202067	2.4	-2.1	-1.4	2.7	KIN	Homo sapiens KIN, antigenic determinant of recA protein homolog (mouse) (KIN), mRNA [NM_012311]	22944
A_23_P50571	1.7	2.1	-1.1	1.3	KIR2DL2	Homo sapiens killer cell immunoglobulin-like receptor, two domains, long cytoplasmic tail, 2 (KIR2DL2), mRNA [NM_014219]	3803
A_23_P130815	-2.5	-1.1	-1.9	-1.2	KIR2DS2	Homo sapiens killer cell immunoglobulin-like receptor, two domains, short cytoplasmic tail, 2 (KIR2DS2), mRNA [NM_012312]	100132285

A_24_P87746	-3.0	-2.4	1.3	-1.2	KIR2DS4	Homo sapiens killer cell immunoglobulin-like receptor, two domains, short cytoplasmic tail, 4 (KIR2DS4), mRNA [NM_012314]	3809
A_23_P78840	-1.2	-1.2	-4.4	-1.3	KIR2DS4	Homo sapiens killer cell immunoglobulin-like receptor, two domains, short cytoplasmic tail, 4 (KIR2DS4), mRNA [NM_012314]	3809
A_24_P117147	5.8	2.9	1.7	3.0	KIR3DL1	Homo sapiens killer cell immunoglobulin-like receptor, three domains, long cytoplasmic tail, 1 (KIR3DL1), mRNA [NM_013289]	3811

A_24_P393312	1.0	-1.0	-1.1	-2.6	KIRREL	Homo sapiens cDNA FLJ33235 fis, clone ASTRO2002202. [AK090554]	55243
A_23_P113994	2.2	1.1	1.0	1.8	KIRREL	Homo sapiens kin of IRRE like (Drosophila) (KIRREL), mRNA [NM_018240]	55243
A_23_P104741	-1.0	5.3	2.4	1.4	KIRREL3	Homo sapiens kin of IRRE like 3 (Drosophila) (KIRREL3), transcript variant 1, mRNA [NM_032531]	84623
A_24_P133253	-2.6	-2.0	-1.1	-1.9	KITLG	Homo sapiens KIT ligand (KITLG), transcript variant b, mRNA [NM_000899]	4254

A_23_P2873	-2.3	2.2	2.0	2.1	KLC1	Homo sapiens kinesin light chain 1 (KLC1), transcript variant 2, mRNA [NM_182923]	3831
A_24_P922877	-2.7	-1.3	-3.2	-1.3	KLC1	Homo sapiens kinesin light chain 1 (KLC1), transcript variant 2, mRNA [NM_182923]	3831
A_24_P227211	-1.3	-1.3	-3.0	-2.8	KLC2	Homo sapiens kinesin light chain 2 (KLC2), transcript variant 1, mRNA [NM_022822]	64837
A_23_P317098	-3.5	-1.1	1.3	-1.1	KLC3	Homo sapiens kinesin light chain 3 (KLC3), mRNA [NM_177417]	147700
A_23_P4653	1.9	2.0	1.3	3.2	KLC3	kinesin light chain 3 [Source:HGNC Symbol;Acc:20717] [ENST00000494686]	147700

A_23_P133799	2.1	3.0	3.0	-1.1	KLC4	Homo sapiens kinesin light chain 4 (KLC4), transcript variant 3, mRNA [NM_201523]	89953
A_23_P397308	-2.1	-1.8	-2.2	-2.3	KLC4	Homo sapiens kinesin light chain 4 (KLC4), transcript variant 4, mRNA [NM_138343]	89953
A_23_P383377	1.2	-3.3	-1.0	2.1	KLF1	Homo sapiens Kruppel-like factor 1 (erythroid) (KLF1), mRNA [NM_006563]	10661
A_23_P319512	-1.4	-2.3	-1.5	-1.4	KLF11	Homo sapiens Kruppel-like factor 11 (KLF11), transcript variant 1, mRNA [NM_003597]	8462

A_23_P354805	-1.1	-2.0	-1.3	-1.5	KLF12	Homo sapiens Kruppel-like factor 12 (KLF12), mRNA [NM_007249]	11278
A_23_P393627	-2.1	-1.5	-2.5	-2.0	KLF14	Homo sapiens Kruppel-like factor 14 (KLF14), mRNA [NM_138693]	136259
A_24_P20327	-4.3	1.1	1.0	1.1	KLF15	Homo sapiens Kruppel-like factor 15 (KLF15), mRNA [NM_014079]	28999
A_24_P151305	1.1	-1.3	-1.6	-2.8	KLF2	Homo sapiens Kruppel-like factor 2 (lung) (KLF2), mRNA [NM_016270]	10365
A_23_P213306	1.7	-4.1	2.1	1.2	KLF3	Homo sapiens Kruppel-like factor 3 (basic) (KLF3), mRNA [NM_016531]	51274

A_23_P32233	1.1	3.3	1.1	1.2	KLF4	Homo sapiens Kruppel-like factor 4 (gut) (KLF4), mRNA [NM_004235]	9314
A_24_P932981	1.1	1.1	2.4	-1.0	KLF6	Homo sapiens Kruppel-like factor 6 (KLF6), transcript variant A, mRNA [NM_001300]	1316
A_32_P164513	1.1	1.1	-2.4	1.2	KLHDC1	Homo sapiens kelch domain containing 1 (KLHDC1), mRNA [NM_172193]	122773
A_24_P342127	-2.1	-1.0	-1.2	-1.3	KLHDC10	Homo sapiens kelch domain containing 10 (KLHDC10), mRNA [NM_014997]	23008

A_24_P299429	9.2	8.9	8.0	8.1	KLHDC10	Homo sapiens kelch domain containing 10 (KLHDC10), mRNA [NM_014997]	23008
A_32_P189680	1.0	-1.0	6.5	-1.1	KLHDC10	Homo sapiens kelch domain containing 10 (KLHDC10), mRNA [NM_014997]	23008
A_24_P116242	-1.7	-2.1	-1.5	-1.5	KLHDC2	Homo sapiens kelch domain containing 2 (KLHDC2), mRNA [NM_014315]	23588
A_23_P156568	-1.0	1.1	-1.1	-2.7	KLHDC3	Homo sapiens kelch domain containing 3 (KLHDC3), transcript variant 1, mRNA [NM_057161]	116138

A_24_P304485	-2.3	1.0	1.1	1.0	KLHDC5	Homo sapiens kelch domain containing 5 (KLHDC5), mRNA [NM_020782]	57542
A_24_P645765	-4.8	1.0	-1.0	1.0	KLHDC5	Homo sapiens kelch domain containing 5 (KLHDC5), mRNA [NM_020782]	57542
A_24_P940576	-3.0	-1.0	-1.1	-1.1	KLHDC5	Homo sapiens kelch domain containing 5 (KLHDC5), mRNA [NM_020782]	57542
A_23_P422131	-3.3	2.2	2.5	2.3	KLHDC7A	Homo sapiens kelch domain containing 7A (KLHDC7A), mRNA [NM_152375]	127707

A_24_P117410	2.7	3.9	5.2	-2.3	KLHDC7B	Homo sapiens kelch domain containing 7B (KLHDC7B), mRNA [NM_138433]	113730
A_24_P62800	-1.0	-2.1	-1.1	-1.2	KLHDC8A	Homo sapiens kelch domain containing 8A (KLHDC8A), mRNA [NM_018203]	55220
A_23_P125117	2.8	1.6	2.5	1.3	KLHDC8B	Homo sapiens kelch domain containing 8B (KLHDC8B), mRNA [NM_173546]	200942
A_24_P148836	-2.7	-1.0	-1.1	-1.0	KLHDC8B	Homo sapiens kelch domain containing 8B (KLHDC8B), mRNA [NM_173546]	200942

A_24_P242609	1.0	-2.5	-6.4	-1.0	KLHL12	Homo sapiens kelch-like 12 (Drosophila) (KLHL12), mRNA [NM_021633]	59349
A_23_P159974	1.2	1.2	-4.1	1.4	KLHL13	Homo sapiens kelch-like 13 (Drosophila) (KLHL13), transcript variant 1, mRNA [NM_033495]	90293
A_32_P75357	-3.3	1.0	1.5	1.6	KLHL14	kelch-like 14 (Drosophila) [Source:HGNC Symbol;Acc:29266] [ENST00000358095]	57565
A_32_P136967	-1.4	1.6	-2.8	2.0	KLHL15	Homo sapiens kelch-like 15 (Drosophila) (KLHL15), mRNA [NM_030624]	80311

A_23_P342910	-4.5	1.1	1.0	1.1	KLHL15	Homo sapiens kelch-like 15 (Drosophila) (KLHL15), mRNA [NM_030624]	80311
A_32_P359110	1.4	1.7	1.4	-2.1	KLHL17	Homo sapiens kelch-like 17 (Drosophila) (KLHL17), mRNA [NM_198317]	339451
A_24_P208721	-1.0	2.2	1.2	1.3	KLHL18	Homo sapiens kelch-like 18 (Drosophila) (KLHL18), mRNA [NM_025010]	23276
A_23_P201478	1.0	-1.1	-4.0	1.2	KLHL21	Homo sapiens kelch-like 21 (Drosophila) (KLHL21), mRNA [NM_014851]	9903

A_24_P117323	-1.8	2.6	-2.3	-1.3	KLHL22	Homo sapiens kelch-like 22 (Drosophila) (KLHL22), transcript variant 1, mRNA [NM_032775]	84861
A_23_P205659	3.0	2.0	2.0	-1.1	KLHL28	Homo sapiens mRNA for BTB (POZ) domain containing 5 variant, clone: COL03244. [AK225183]	54813
A_23_P431263	1.0	-1.0	-4.9	-1.1	KLHL29	Homo sapiens cDNA FLJ14106 fis, clone MAMMA1001222. [AK024168]	114818
A_23_P133543	29.3	28.7	26.9	20.4	KLHL3	Homo sapiens kelch-like 3 (Drosophila) (KLHL3), mRNA [NM_017415]	26249

A_23_P16754	1.1	1.3	-2.5	-30.8	KLHL30	Homo sapiens kelch-like 30 (Drosophila) (KLHL30), mRNA [NM_198582]	377007
A_32_P823560	2.8	1.1	1.7	1.1	KLHL33	Homo sapiens kelch-like 33 (Drosophila) (KLHL33), mRNA [NM_001109997]	123103
A_24_P192978	-3.1	-1.1	1.6	1.4	KLHL35	Homo sapiens kelch-like 35 (Drosophila) (KLHL35), mRNA [NM_001039548]	283212
A_24_P353486	-1.0	1.2	-3.2	-3.3	KLHL4	Homo sapiens kelch-like 4 (Drosophila) (KLHL4), transcript variant 2, mRNA [NM_057162]	56062

A_23_P121527	1.1	-2.6	-1.1	-1.0	KLHL5	Homo sapiens kelch-like 5 (Drosophila) (KLHL5), transcript variant 1, mRNA [NM_015990]	51088
A_24_P944253	-5.2	1.0	-1.1	-1.0	KLHL6	Homo sapiens kelch-like 6 (Drosophila) (KLHL6), mRNA [NM_130446]	89857
A_23_P212655	3.5	4.1	1.9	3.5	KLHL6	Homo sapiens kelch-like 6 (Drosophila) (KLHL6), mRNA [NM_130446]	89857
A_23_P324994	-3.1	1.0	-1.1	1.0	KLHL7	Homo sapiens kelch-like 7 (Drosophila) (KLHL7), transcript variant 2, mRNA [NM_018846]	55975

A_23_P107911	-1.1	-1.1	-2.9	-1.2	KLK10	Homo sapiens kallikrein-related peptidase 10 (KLK10), transcript variant 1, mRNA [NM_002776]	5655
A_24_P399490	1.5	1.4	-1.6	2.4	KLK10	Homo sapiens kallikrein-related peptidase 10 (KLK10), transcript variant 1, mRNA [NM_002776]	5655
A_24_P416645	1.5	-3.8	-1.0	-1.0	KLK13	Homo sapiens kallikrein-related peptidase 13 (KLK13), mRNA [NM_015596]	26085
A_23_P78717	-1.4	2.3	-1.0	-1.3	KLK14	Homo sapiens kallikrein-related peptidase 14 (KLK14), mRNA [NM_022046]	43847

A_23_P4856	-1.0	-1.1	-2.9	1.6	KLK15	Homo sapiens kallikrein-related peptidase 15 (KLK15), transcript variant 3, mRNA [NM_138564]	55554
A_24_P5750	-1.2	-1.3	1.6	-2.7	KLK2	Homo sapiens kallikrein-related peptidase 2 (KLK2), transcript variant 1, mRNA [NM_005551]	3817
A_24_P922015	-1.6	-1.8	-1.8	-3.1	KLK2	Homo sapiens prostate-specific KLK2 mRNA, complete cds, alternatively spliced. [AF336106]	3817
A_23_P153480	-1.5	-1.1	-2.0	1.8	KLK5	Homo sapiens kallikrein-related peptidase 5 (KLK5), transcript variant 1, mRNA [NM_012427]	25818

A_23_P119311	1.7	-2.4	-3.4	1.0	KLK9	Homo sapiens kallikrein-related peptidase 9 (KLK9), mRNA [NM_012315]	284366
A_23_P99275	1.1	1.0	1.3	-2.9	KLRB1	Homo sapiens killer cell lectin-like receptor subfamily B, member 1 (KLRB1), mRNA [NM_002258]	3820
A_23_P218058	1.0	-2.6	-1.0	1.0	KLRC4	Homo sapiens killer cell lectin-like receptor subfamily C, member 4 (KLRC4), mRNA [NM_013431]	8302
A_32_P158966	1.0	1.0	-1.0	4.5	KLRF1	Homo sapiens killer cell lectin-like receptor subfamily F, member 1 (KLRF1), mRNA [NM_016523]	51348

A_23_P8723	-1.0	-2.5	1.2	1.4	KLRG2	Homo sapiens killer cell lectin-like receptor subfamily G, member 2, mRNA (cDNA clone MGC:131846 IMAGE:6139360), complete cds. [BC110858]	346689
A_32_P33263	1.8	1.7	2.3	1.0	KLRG2	Homo sapiens cDNA FLJ44186 fis, clone THYMU2038797, weakly similar to B locus C type Lectin. [AK126174]	346689
A_23_P200838	1.6	1.8	2.2	-1.3	KMO	Homo sapiens kynurenine 3-monooxygenase (kynurenine 3-hydroxylase) (KMO), mRNA [NM_003679]	8564

A_24_P383050	1.1	1.3	1.6	-2.7	KNCN	Homo sapiens kinocilin (KNCN), mRNA [NM_001097611]	148930
A_24_P32574	1.3	-1.1	-3.6	-1.9	KNDC1	Homo sapiens kinase non-catalytic C-lobe domain (KIND) containing 1 (KNDC1), transcript variant 1, mRNA [NM_152643]	85442
A_23_P414312	-5.3	1.0	1.1	1.0	KNDC1	Homo sapiens kinase non-catalytic C-lobe domain (KIND) containing 1 (KNDC1), transcript variant 1, mRNA [NM_152643]	85442

A_24_P940680	1.0	-1.0	-1.1	-2.1	KNDC1	kinase non-catalytic C-lobe domain (KIND) containing 1 [Source:HGNC Symbol;Acc:29374] [ENST00000497778]	85442
A_32_P230808	3.5	2.8	1.2	1.4	KNG1	Homo sapiens kininogen 1 (KNG1), transcript variant 2, mRNA [NM_000893]	3827
A_23_P212258	1.4	-1.2	1.8	-2.2	KNG1	Homo sapiens kininogen 1 (KNG1), transcript variant 2, mRNA [NM_000893]	3827
A_23_P139950	-3.8	-1.0	-4.1	-3.1	KPNA3	Homo sapiens karyopherin alpha 3 (importin alpha 4) (KPNA3), mRNA [NM_002267]	3839

A_24_P11791	4.3	3.3	-1.5	3.7	KPNA4	Homo sapiens karyopherin alpha 4 (importin alpha 3) (KPNA4), mRNA [NM_002268]	3840
A_32_P3127	-5.6	-2.4	-1.5	-2.1	KPNA4	Homo sapiens karyopherin alpha 4 (importin alpha 3) (KPNA4), mRNA [NM_002268]	3840
A_23_P218835	-1.3	-4.4	-2.2	-2.4	KPNA4	Homo sapiens karyopherin alpha 4 (importin alpha 3) (KPNA4), mRNA [NM_002268]	3840
A_24_P128563	-1.5	1.4	2.1	2.3	KPNA6	Homo sapiens karyopherin alpha 6 (importin alpha 7) (KPNA6), mRNA [NM_012316]	23633

A_23_P118774	-4.1	-1.1	1.1	-1.1	KPNB1	Homo sapiens karyopherin (importin) beta 1 (KPNB1), mRNA [NM_002265]	3837
A_23_P67391	1.1	1.4	2.4	1.2	KPTN	Homo sapiens kaptin (actin binding protein) (KPTN), mRNA [NM_007059]	11133
A_24_P927287	-1.2	-1.2	-9.8	-1.4	KRAS	Homo sapiens v-Ki-ras2 Kirsten rat sarcoma viral oncogene homolog, mRNA (cDNA clone IMAGE:5301134). [BC029545]	3845
A_32_P38637	2.0	1.7	1.1	1.3	KRBA1	Homo sapiens KRAB-A domain containing 1 (KRBA1), mRNA [NM_032534]	84626

A_23_P80551	-2.9	-1.4	-1.4	-1.3	KRBOX1	Homo sapiens KRAB box domain containing 1 (KRBOX1), mRNA [NM_001205272]	100506243
A_23_P56759	1.3	2.4	2.3	4.0	KRCC1	Homo sapiens lysine-rich coiled-coil 1 (KRCC1), mRNA [NM_016618]	51315
A_23_P68851	1.4	1.6	1.1	2.4	KREMEN1	Homo sapiens kringle containing transmembrane protein 1 (KREMEN1), transcript variant 3, mRNA [NM_001039570]	83999
A_23_P42848	-1.0	-1.0	-1.0	3.5	KRIT1	Homo sapiens KRIT1, ankyrin repeat containing (KRIT1), transcript variant 4, mRNA [NM_194455]	889

A_23_P2216	-1.0	-2.1	-1.1	-1.1	KRR1	Homo sapiens KRR1, small subunit (SSU) processome component, homolog (yeast) (KRR1), mRNA [NM_007043]	11103
A_23_P55448	1.0	1.0	-1.1	-2.9	KRT12	Homo sapiens keratin 12 (KRT12), mRNA [NM_000223]	3859
A_24_P228149	2.1	3.6	2.3	5.1	KRT13	Homo sapiens keratin 13 (KRT13), transcript variant 2, mRNA [NM_002274]	3860
A_24_P265346	9.0	14.7	8.7	9.4	KRT14	Homo sapiens keratin 14 (KRT14), mRNA [NM_000526]	3861
A_23_P4335	-4.2	-1.6	-1.1	-1.0	KRT14	Homo sapiens keratin 14 (KRT14), mRNA [NM_000526]	3861

A_23_P38537	1.1	1.3	-4.1	1.0	KRT16	Homo sapiens keratin 16 (KRT16), mRNA [NM_005557]	3868
A_23_P96158	3.0	2.9	2.6	5.2	KRT17	Homo sapiens keratin 17 (KRT17), mRNA [NM_000422]	3872
A_23_P99320	-1.2	-3.3	-1.3	-1.1	KRT18	Homo sapiens keratin 18 (KRT18), transcript variant 1, mRNA [NM_000224]	3875
A_24_P924957	5.5	4.9	3.2	7.0	KRT18	Homo sapiens (clone XP6G6B) mRNA, partial EST. [L32537]	3875
A_23_P66798	1.6	1.5	2.7	1.5	KRT19	Homo sapiens keratin 19 (KRT19), mRNA [NM_002276]	3880
A_23_P66854	1.7	1.7	2.3	1.0	KRT20	Homo sapiens keratin 20 (KRT20), mRNA [NM_019010]	54474

A_23_P78248	1.0	-2.4	1.0	1.0	KRT23	Homo sapiens keratin 23 (histone deacetylase inducible) (KRT23), mRNA [NM_015515]	25984
A_23_P27186	-2.5	-1.0	-1.1	-2.0	KRT26	Homo sapiens keratin 26 (KRT26), mRNA [NM_181539]	353288
A_32_P489662	3.0	3.2	2.0	1.8	KRT28	Homo sapiens keratin 28 (KRT28), mRNA [NM_181535]	162605
A_23_P33573	1.8	1.7	1.4	-2.0	KRT3	Homo sapiens keratin 3 (KRT3), mRNA [NM_057088]	3850
A_23_P107465	1.2	6.1	1.4	1.8	KRT31	Homo sapiens keratin 31 (KRT31), mRNA [NM_002277]	3881
A_23_P89665	-1.0	-2.1	-1.5	-2.6	KRT33B	Homo sapiens keratin 33B (KRT33B), mRNA [NM_002279]	3884

A_23_P100991	-19.8	1.4	1.4	1.0	KRT38	Homo sapiens keratin 38 (KRT38), mRNA [NM_006771]	8687
A_23_P100990	-1.2	2.2	1.9	1.4	KRT38	Homo sapiens keratin 38 (KRT38), mRNA [NM_006771]	8687
A_24_P280333	-1.9	1.1	-5.5	-1.3	KRT40	Homo sapiens keratin 40 (KRT40), mRNA [NM_182497]	125115
A_23_P218047	2.5	-1.0	-1.0	-1.0	KRT5	Homo sapiens keratin 5 (KRT5), mRNA [NM_000424]	3852
A_23_P366936	-14.0	-1.3	-7.0	-1.4	KRT6C	Homo sapiens keratin 6C (KRT6C), mRNA [NM_173086]	286887
A_23_P99044	-2.0	-2.0	-2.7	-2.3	KRT71	Homo sapiens keratin 71 (KRT71), mRNA [NM_033448]	112802

A_23_P105453	3.7	3.6	3.3	3.4	KRT73	Homo sapiens keratin 73 (KRT73), mRNA [NM_175068]	319101
A_24_P927756	1.3	1.7	-2.7	1.8	KRT77	Homo sapiens keratin 77 (KRT77), mRNA [NM_175078]	374454
A_24_P391625	1.1	1.1	1.2	-6.4	KRT79	Homo sapiens keratin 79 (KRT79), mRNA [NM_175834]	338785
A_23_P14072	-1.8	-4.0	-2.5	-1.8	KRT8	Homo sapiens keratin 8 (KRT8), mRNA [NM_002273]	3856
A_24_P349264	-1.8	-1.2	-2.0	-2.0	KRT80	Homo sapiens keratin 80 (KRT80), transcript variant 1, mRNA [NM_182507]	144501
A_24_P410408	1.4	-2.3	-7.7	1.4	KRT83	Homo sapiens keratin 83 (KRT83), mRNA [NM_002282]	3889

A_23_P204144	-1.8	-2.9	-1.7	1.4	KRT85	Homo sapiens keratin 85 (KRT85), mRNA [NM_002283]	3891
A_23_P363769	1.5	3.5	-1.6	1.8	KRT86	Homo sapiens keratin 86 (KRT86), mRNA [NM_002284]	3892
A_24_P489690	-1.0	-1.0	-2.7	-1.2	KRT8P41	Homo sapiens keratin 8 pseudogene 41 (KRT8P41), non-coding RNA [NR_027713]	283102
A_24_P489695	4.1	1.6	3.1	1.7	KRT8P41	Homo sapiens keratin 8 pseudogene 41 (KRT8P41), non-coding RNA [NR_027713]	283102
A_24_P151005	1.7	1.7	3.0	1.0	KRTAP1-1	Homo sapiens keratin associated protein 1-1 (KRTAP1-1), mRNA [NM_030967]	81851

A_32_P481377	1.9	-1.8	1.6	-2.2	KRTAP11-1	Homo sapiens keratin associated protein 11-1 (KRTAP11-1), mRNA [NM_175858]	337880
A_24_P66312	-1.9	-1.3	-1.8	-2.8	KRTAP12-1	Homo sapiens keratin associated protein 12-1 (KRTAP12-1), mRNA [NM_181686]	353332
A_24_P142495	-2.8	-1.0	-2.3	-1.1	KRTAP1-3	Homo sapiens keratin associated protein 1-3 (KRTAP1-3), mRNA [NM_030966]	81850
A_24_P314534	2.6	2.5	-1.2	1.5	KRTAP13-2	Homo sapiens keratin associated protein 13-2 (KRTAP13-2), nuclear gene encoding mitochondrial protein, mRNA [NM_181621]	337959

A_24_P246107	1.8	3.3	2.2	1.4	KRTAP13-4	Homo sapiens keratin associated protein 13-4 (KRTAP13-4), mRNA [NM_181600]	284827
A_23_P118842	-15.2	-1.7	-1.9	-1.8	KRTAP1-5	Homo sapiens keratin associated protein 1-5 (KRTAP1-5), mRNA [NM_031957]	83895
A_24_P348845	-1.2	-1.2	-2.0	-3.1	KRTAP15-1	Homo sapiens keratin associated protein 15-1 (KRTAP15-1), mRNA [NM_181623]	254950
A_23_P66858	-1.0	-1.0	-1.0	-2.3	KRTAP17-1	Homo sapiens keratin associated protein 17-1 (KRTAP17-1), mRNA [NM_031964]	83902

A_23_P143461	-3.6	1.2	-2.8	-1.2	KRTAP19-1	keratin associated protein 19-1 [Source:HGNC Symbol;Acc:18936] [ENST00000390689]	337882
A_24_P76854	-1.2	-1.7	-3.9	-1.0	KRTAP2-1	Homo sapiens keratin associated protein 2-1 (KRTAP2-1), mRNA [NM_001123387]	81872
A_23_P164405	2.3	1.6	1.6	1.0	KRTAP2-4	Homo sapiens keratin associated protein 2-4 (KRTAP2-4), mRNA [NM_033184]	85294
A_23_P153022	3.1	1.3	1.5	-1.1	KRTAP2-4	Homo sapiens keratin associated protein 2-4 (KRTAP2-4), mRNA [NM_033184]	85294

A_24_P125977	6.5	5.1	1.0	-2.9	KRTAP2-4	Homo sapiens keratin associated protein 2-4 (KRTAP2-4), mRNA [NM_033184]	85294
A_23_P101048	-1.0	-3.7	-1.0	-1.0	KRTAP3-2	Homo sapiens keratin associated protein 3-2 (KRTAP3-2), mRNA [NM_031959]	83897
A_23_P89649	1.0	-1.1	-1.1	-2.8	KRTAP3-3	Homo sapiens keratin associated protein 3-3 (KRTAP3-3), mRNA [NM_033185]	85293
A_23_P10936	-2.3	-2.4	-1.1	1.3	KRTAP4-1	Homo sapiens keratin associated protein 4-1 (KRTAP4-1), mRNA [NM_033060]	85285

A_23_P15798	4.3	3.2	1.2	-1.2	KRTAP4-12	Homo sapiens keratin associated protein 4-12 (KRTAP4-12), mRNA [NM_031854]	83755
A_23_P66844	1.8	1.7	1.7	-3.4	KRTAP4-7	Homo sapiens keratin associated protein 4-7 (KRTAP4-7), mRNA [NM_033061]	100132476
A_24_P325146	-3.4	-1.8	-1.3	1.0	KRTAP4-8	Homo sapiens keratin associated protein 4-8 (KRTAP4-8), mRNA [NM_031960]	728224
A_23_P47086	1.0	3.4	1.3	-1.1	KRTAP5-8	Homo sapiens keratin associated protein 5-8 (KRTAP5-8), mRNA [NM_021046]	57830

A_23_P13048	-4.4	1.2	-1.7	-2.0	KRTAP5-9	Homo sapiens keratin associated protein 5-9 (KRTAP5-9), mRNA [NM_005553]	3846
A_24_P391604	3.1	6.1	1.2	3.1	KRTAP6-3	Homo sapiens keratin associated protein 6-3 (KRTAP6-3), mRNA [NM_181605]	337968
A_23_P89646	1.1	-2.6	1.1	1.0	KRTAP9-2	Homo sapiens keratin associated protein 9-2 (KRTAP9-2), mRNA [NM_031961]	83899
A_32_P66091	-2.0	-1.6	-2.1	-1.1	KSR1	Homo sapiens kinase suppressor of ras 1 (KSR1), mRNA [NM_014238]	8844
A_24_P255483	1.1	1.0	-4.8	1.8	KSR1	Homo sapiens kinase suppressor of ras 1 (KSR1), mRNA [NM_014238]	8844

A_23_P103276	2.1	1.4	-1.2	1.3	KTI12	Homo sapiens KTI12 homolog, chromatin associated (S. cerevisiae) (KTI12), mRNA [NM_138417]	112970
A_23_P106131	5.1	4.6	2.4	2.8	KTN1	Homo sapiens kinectin 1 (kinesin receptor) (KTN1), transcript variant 4, mRNA [NM_004986]	3895
A_24_P11506	1.0	-5.0	1.0	-1.0	KYNU	Homo sapiens kynureninase (KYNU), transcript variant 2, mRNA [NM_001032998]	8942
A_23_P137484	1.7	1.8	2.9	1.0	L1TD1	Homo sapiens LINE-1 type transposase domain containing 1 (L1TD1), transcript variant 2, mRNA [NM_019079]	54596

A_23_P106158	-1.7	1.4	-4.2	1.4	L2HGDH	Homo sapiens L-2-hydroxyglutarate dehydrogenase (L2HGDH), nuclear gene encoding mitochondrial protein, mRNA [NM_024884]	79944
A_23_P210445	1.1	1.1	1.1	-2.5	L3MBTL1	Homo sapiens l(3)mbt-like 1 (Drosophila) (L3MBTL1), transcript variant II, mRNA [NM_032107]	26013
A_24_P6135	1.4	2.6	-1.1	1.4	L3MBTL2	Homo sapiens l(3)mbt-like 2 (Drosophila) (L3MBTL2), mRNA [NM_031488]	83746

A_23_P80353	-3.2	-1.9	-1.7	-2.8	L3MBTL2	Homo sapiens l(3)mbt-like 2 (Drosophila) (L3MBTL2), mRNA [NM_031488]	83746
A_32_P67533	1.1	-2.4	1.1	1.1	L3MBTL3	Homo sapiens l(3)mbt-like 3 (Drosophila) (L3MBTL3), transcript variant 1, mRNA [NM_032438]	84456
A_23_P311885	1.0	-2.1	-1.0	-1.1	L3MBTL3	Homo sapiens l(3)mbt-like 3 (Drosophila) (L3MBTL3), transcript variant 1, mRNA [NM_032438]	84456

A_23_P366426	-3.6	-3.5	-4.0	-4.0	L3MBTL4	Homo sapiens l(3)mbt-like 4 (Drosophila), mRNA (cDNA clone MGC:39812 IMAGE:5298402), complete cds. [BC039316]	91133
A_23_P116942	-2.7	3.9	-2.3	-1.2	LAG3	Homo sapiens lymphocyte-activation gene 3 (LAG3), mRNA [NM_002286]	3902
A_23_P73763	1.8	3.4	2.8	3.1	LAGE3	Homo sapiens L antigen family, member 3 (LAGE3), mRNA [NM_006014]	8270

A_23_P209135	1.2	-4.8	1.1	1.6	LAIR1	Homo sapiens leukocyte-associated immunoglobulin-like receptor 1 (LAIR1), transcript variant a, mRNA [NM_002287]	3903
A_23_P209129	3.7	2.1	3.2	3.2	LAIR2	Homo sapiens leukocyte-associated immunoglobulin-like receptor 2 (LAIR2), transcript variant 1, mRNA [NM_002288]	3904
A_32_P313405	1.1	-2.3	1.1	-1.1	LAMA1	Homo sapiens laminin, alpha 1 (LAMA1), mRNA [NM_005559]	284217
A_24_P687302	1.1	-2.1	1.1	1.1	LAMA3	Homo sapiens laminin, alpha 3 (LAMA3), transcript variant 1, mRNA [NM_198129]	3909

A_23_P109269	-3.3	1.3	-3.1	-1.1	LAMA5	Homo sapiens laminin, alpha 5 (LAMA5), mRNA [NM_005560]	3911
A_23_P86012	1.0	1.7	-14.7	-1.1	LAMB3	Homo sapiens laminin, beta 3 (LAMB3), transcript variant 2, mRNA [NM_001017402]	3914
A_23_P201636	1.7	1.7	2.4	1.0	LAMC2	Homo sapiens laminin, gamma 2 (LAMC2), transcript variant 1, mRNA [NM_005562]	3918
A_23_P82979	2.6	2.5	1.8	2.4	LAMC3	Homo sapiens laminin, gamma 3 (LAMC3), mRNA [NM_006059]	10319
A_24_P304549	1.2	1.1	1.5	-2.3	LAMP1	Homo sapiens lysosomal-associated membrane protein 1 (LAMP1), mRNA [NM_005561]	3916

A_23_P2114	1.4	-1.1	-1.5	3.5	LAMTOR1	Homo sapiens late endosomal/lysosomal adaptor, MAPK and MTOR activator 1 (LAMTOR1), mRNA [NM_017907]	55004
A_24_P259922	1.1	-2.1	-1.1	1.3	LANCL1	Homo sapiens LanC lantibiotic synthetase component C-like 1 (bacterial) (LANCL1), transcript variant 1, mRNA [NM_006055]	10314
A_23_P82642	3.1	1.8	3.1	-1.5	LANCL2	Homo sapiens LanC lantibiotic synthetase component C-like 2 (bacterial) (LANCL2), mRNA [NM_018697]	55915

A_24_P180680	1.1	1.6	1.6	2.1	LAPTM4B	Homo sapiens lysosomal protein transmem brane 4 beta (LAPTM4B), mRNA [NM_0184 07]	55353
A_23_P392383	1.2	3.0	-1.3	-9.3	LARP1	Homo sapiens La ribonucleo protein domain family, member 1 (LARP1), transcript variant 1, mRNA [NM_0153 15]	23367
A_24_P78526	-2.2	-1.0	-2.0	-1.1	LARP4	Homo sapiens La ribonucleo protein domain family, member 4 (LARP4), transcript variant 2, mRNA [NM_1991 88]	113251

A_23_P36860	-1.6	-2.1	-2.9	-1.7	LARP4	Homo sapiens La ribonucleo protein domain family, member 4 (LARP4), transcript variant 2, mRNA [NM_1991 88]	113251
A_23_P84070	1.1	-4.4	-1.1	1.0	LARP7	Homo sapiens La ribonucleo protein domain family, member 7 (LARP7), transcript variant 1, mRNA [NM_0166 48]	51574
A_24_P51360	4.7	5.1	4.7	10.6	LARS	Homo sapiens leucyl-tRNA synthetase (LARS), mRNA [NM_0201 17]	51520
A_23_P218965	-8.1	-1.8	1.7	1.1	LARS	Homo sapiens leucyl-tRNA synthetase (LARS), mRNA [NM_0201 17]	51520

A_23_P212397	2.1	1.2	1.2	1.1	LARS2	Homo sapiens leucyl-tRNA synthetase 2, mitochondrial (LARS2), nuclear gene encoding mitochondrial protein, mRNA [NM_015340]	23395
A_23_P44112	-1.3	-1.3	-1.5	-2.5	LAT	Homo sapiens linker for activation of T cells (LAT), transcript variant 1, mRNA [NM_014387]	27040
A_23_P60977	1.3	1.0	-7.3	-1.7	LAT	Homo sapiens linker for activation of T cells (LAT), transcript variant 1, mRNA [NM_014387]	27040

A_24_P940327	-2.2	1.7	1.6	1.0	LAT2	linker for activation of T cells family, member 2 [Source:HGNC Symbol;Acc:12749] [ENST00000398475]	7462
A_23_P259621	-4.8	2.0	1.7	-1.1	LAT2	Homo sapiens linker for activation of T cells family, member 2 (LAT2), transcript variant 1, mRNA [NM_032464]	7462
A_24_P70002	-2.6	2.2	-2.2	1.1	LATS2	Homo sapiens LATS, large tumor suppressor, homolog 2 (Drosophila) (LATS2), mRNA [NM_014572]	26524

A_23_P438	1.0	-2.5	-1.1	-1.1	LAX1	Homo sapiens lymphocyte transmembrane adaptor 1 (LAX1), transcript variant 1, mRNA [NM_017773]	54900
A_24_P205025	1.3	3.1	1.2	-1.3	LAYN	Homo sapiens cDNA FLJ39606 fis, clone SKNSH2006234. [AK096925]	143903
A_24_P182858	-1.3	-1.9	-1.8	-2.2	LBH	Homo sapiens limb bud and heart development homolog (mouse) (LBH), mRNA [NM_030915]	81606
A_23_P143178	-2.2	1.2	-3.3	-4.4	LBP	Homo sapiens lipopolysaccharide binding protein (LBP), mRNA [NM_004139]	3929

A_23_P86489	-3.6	-1.3	-1.5	-2.9	LBX1	H.sapiens mRNA for transcription factor, Lbx1. [X90828]	10660
A_23_P79458	2.7	1.9	2.2	2.4	LBX2	Homo sapiens ladybird homeobox 2 (LBX2), mRNA [NM_001009812]	85474
A_32_P48466	1.3	1.3	-1.1	-2.1	LCA5L	Homo sapiens Leber congenital amaurosis 5-like (LCA5L), mRNA [NM_152505]	150082
A_23_P103315	2.2	1.1	1.2	3.4	LCE1A	Homo sapiens late cornified envelope 1A (LCE1A), mRNA [NM_178348]	353131
A_23_P404685	1.5	3.7	1.1	1.2	LCE1A	Homo sapiens late cornified envelope 1A (LCE1A), mRNA [NM_178348]	353131

A_23_P368862	-1.0	-15.5	-1.0	1.0	LCE1B	Homo sapiens late cornified envelope 1B (LCE1B), mRNA [NM_178349]	353132
A_23_P339691	-1.2	1.0	-2.4	-1.0	LCE1E	Homo sapiens late cornified envelope 1E (LCE1E), mRNA [NM_178353]	353135
A_23_P411321	-2.4	-1.0	1.4	-2.7	LCE1F	Homo sapiens late cornified envelope 1F (LCE1F), mRNA [NM_178354]	353137
A_23_P418006	2.7	1.6	3.1	-1.1	LCE2A	Homo sapiens late cornified envelope 2A (LCE2A), mRNA [NM_178428]	353139

A_24_P207828	-2.7	1.3	-3.7	2.6	LCE2B	Homo sapiens late cornified envelope 2B (LCE2B), mRNA [NM_014357]	26239
A_23_P74920	1.4	2.4	1.2	8.7	LCE2B	Homo sapiens late cornified envelope 2B (LCE2B), mRNA [NM_014357]	26239
A_23_P63521	-1.1	5.0	-2.7	1.1	LCE2C	Homo sapiens late cornified envelope 2C (LCE2C), mRNA [NM_178429]	353140
A_23_P369471	1.7	2.1	1.0	-1.0	LCE3A	Homo sapiens late cornified envelope 3A (LCE3A), mRNA [NM_178431]	353142

A_23_P324538	1.7	1.6	1.7	-16.7	LCE3B	Homo sapiens late cornified envelope 3B (LCE3B), mRNA [NM_178433]	353143
A_23_P405295	-1.1	-2.2	-1.3	-1.1	LCE3C	Homo sapiens late cornified envelope 3C (LCE3C), mRNA [NM_178434]	353144
A_23_P115519	3.4	3.1	2.0	3.8	LCE3D	Homo sapiens late cornified envelope 3D (LCE3D), mRNA [NM_032563]	84648
A_23_P340338	-1.3	-9.2	-1.7	1.4	LCE3E	Homo sapiens late cornified envelope 3E (LCE3E), mRNA [NM_178435]	353145

A_23_P310540	1.6	2.4	2.4	-2.8	LCE4A	Homo sapiens late cornified envelope 4A (LCE4A), mRNA [NM_178356]	199834
A_23_P304530	1.7	2.6	-1.1	1.8	LCE5A	Homo sapiens late cornified envelope 5A (LCE5A), mRNA [NM_178438]	254910
A_23_P32115	-4.6	-3.0	-3.0	-2.1	LCN12	Homo sapiens lipocalin 12 (LCN12), mRNA [NM_178536]	286256
A_23_P71778	1.5	1.2	-1.2	-2.4	LCN15	Homo sapiens lipocalin 15 (LCN15), mRNA [NM_203347]	389812
A_23_P169437	6.8	3.5	2.3	2.7	LCN2	Homo sapiens lipocalin 2 (LCN2), mRNA [NM_005564]	3934

A_24_P365906	-2.5	-1.7	1.7	-1.4	LCN8	lipocalin 8 [Source:HGNC Symbol;Acc:27038] [ENST00000482893]	138307
A_23_P429670	-1.6	2.8	-1.2	2.1	LCOR	Homo sapiens ligand dependent nuclear receptor corepressor (LCOR), transcript variant 1, mRNA [NM_032440]	84458
A_32_P169679	-2.1	1.0	1.0	1.2	LCORL	Homo sapiens mRNA; cDNA DKFZp434K087 (from clone DKFZp434K087). [AL133031]	254251
A_23_P30547	-3.3	-1.6	-1.8	-1.8	LCP2	Homo sapiens lymphocyte cytosolic protein 2 (SH2 domain containing leukocyte protein of 76kDa) (LCP2), mRNA [NM_005565]	3937

A_23_P79217	-1.0	1.3	-2.7	-1.0	LCT	Homo sapiens lactase (LCT), mRNA [NM_002299]	3938
A_23_P47565	-6.9	-1.1	-1.2	-1.1	LDHA	Homo sapiens lactate dehydrogenase A (LDHA), transcript variant 1, mRNA [NM_005566]	3939
A_32_P231391	1.8	-1.2	-1.0	-2.0	LDHA	Homo sapiens lactate dehydrogenase A (LDHA), transcript variant 1, mRNA [NM_005566]	3939
A_23_P53476	1.9	2.2	1.8	1.0	LDHB	Homo sapiens lactate dehydrogenase B (LDHB), transcript variant 1, mRNA [NM_002300]	3945

A_24_P913489	2.7	1.0	2.0	-1.2	LDLR	Homo sapiens low density lipoprotein receptor (FH 10 mutant causing familial hypercholesterolemia) mRNA, 3' end. [M28219]	3949
A_24_P491397	-3.6	-1.0	-1.1	-1.6	LDLRAD1	Homo sapiens low density lipoprotein receptor class A domain containing 1 (LDLRAD1), mRNA [NM_001010978]	388633
A_23_P25587	1.6	1.0	-1.0	-3.9	LECT1	Homo sapiens leukocyte cell derived chemotaxin 1 (LECT1), transcript variant 1, mRNA [NM_007015]	11061

A_24_P20630	-1.3	1.1	-2.0	-1.1	LEF1	Homo sapiens lymphoid enhancer-binding factor 1 (LEF1), transcript variant 1, mRNA [NM_016269]	51176
A_24_P305960	1.0	-2.1	-1.4	-2.2	LEMD2	Homo sapiens LEM domain containing 2 (LEMD2), transcript variant 1, mRNA [NM_181336]	221496
A_23_P208523	-5.5	1.4	1.7	1.3	LENG1	Homo sapiens leukocyte receptor cluster (LRC) member 1 (LENG1), mRNA [NM_024316]	79165
A_23_P56288	2.1	1.1	-1.3	3.8	LENG8	Homo sapiens leukocyte receptor cluster (LRC) member 8 (LENG8), mRNA [NM_052925]	114823

A_32_P493225	-2.9	1.5	-2.1	-6.9	LENG9	Homo sapiens leukocyte receptor cluster (LRC) member 9 (LENG9), mRNA [NM_198988]	94059
A_23_P314222	1.1	1.5	-1.4	3.8	LEO1	Homo sapiens Leo1, Paf1/RNA polymerase II complex component, homolog (S. cerevisiae) (LEO1), mRNA [NM_138792]	123169
A_23_P161135	2.0	-1.6	1.7	-2.1	LEPR	Homo sapiens leptin receptor (LEPR), transcript variant 1, mRNA [NM_002303]	3953

A_23_P63038	-1.5	-2.3	-2.7	-2.4	LEPRE1	Homo sapiens leucine proline-enriched proteoglycan (leprecan) 1 (LEPRE1), transcript variant 1, mRNA [NM_022356]	64175
A_23_P87752	-1.7	-1.5	-2.0	-2.3	LEPREL2	Homo sapiens leprecan-like 2 (LEPREL2), mRNA [NM_014262]	10536
A_24_P148094	1.0	1.0	3.9	-1.0	LEPROT	Homo sapiens leptin receptor overlapping transcript (LEPROT), transcript variant 1, mRNA [NM_017526]	54741

A_23_P1126	6.0	2.4	9.0	11.4	LEPROT	Homo sapiens leptin receptor overlapping transcript (LEPROT), transcript variant 1, mRNA [NM_017526]	54741
A_23_P94998	2.4	2.8	2.0	1.4	LETM1	Homo sapiens leucine zipper-EF-hand containing transmembrane protein 1 (LETM1), nuclear gene encoding mitochondrial protein, mRNA [NM_012318]	3954
A_23_P8452	-1.2	-5.6	-3.3	1.1	LFNG	Homo sapiens LFNG O-fucosylpeptide 3-beta-N-acetylglucosaminyltransferase (LFNG), transcript variant 1, mRNA [NM_001040167]	3955

A_23_P139198	-2.1	-2.1	-1.3	-2.1	LGALS12	Homo sapiens lectin, galactoside-binding, soluble, 12 (LGALS12), transcript variant 2, mRNA [NM_033101]	85329
A_23_P5031	2.8	2.8	2.7	2.9	LGALS13	Homo sapiens lectin, galactoside-binding, soluble, 13 (LGALS13), mRNA [NM_013268]	29124
A_23_P254917	-1.1	-1.8	-23.7	1.2	LGALS4	Homo sapiens lectin, galactoside-binding, soluble, 4 (LGALS4), mRNA [NM_006149]	3960
A_23_P108062	-2.3	1.2	1.1	-1.3	LGALS7B	Homo sapiens lectin, galactoside-binding, soluble, 7B (LGALS7B), mRNA [NM_001042507]	653499

A_23_P345445	-4.0	-1.6	-3.1	1.1	LGALS8-AS1	Homo sapiens LGALS8 antisense RNA 1 (non-protein coding) (LGALS8-AS1), non-coding RNA [NR_034040]	100287902
A_23_P430818	1.1	-1.1	-2.1	-1.8	LGALSL	Homo sapiens lectin, galactoside-binding-like (LGALSL), mRNA [NM_014181]	29094
A_23_P127267	2.7	2.7	2.7	2.8	LGI1	Homo sapiens leucine-rich, glioma inactivated 1 (LGI1), mRNA [NM_005097]	9211
A_24_P310256	2.5	-1.9	1.1	1.9	LGI4	Homo sapiens leucine-rich repeat LGI family, member 4 (LGI4), mRNA [NM_139284]	163175

A_32_P17765	1.1	-4.5	1.2	2.0	LGR4	Homo sapiens leucine-rich repeat containing G protein-coupled receptor 4 (LGR4), mRNA [NM_018490]	55366
A_24_P90216	5.5	5.2	4.8	5.2	LGR4	Homo sapiens leucine-rich repeat containing G protein-coupled receptor 4 (LGR4), mRNA [NM_018490]	55366
A_23_P98974	2.0	1.9	-13.8	1.6	LGR5	Homo sapiens leucine-rich repeat containing G protein-coupled receptor 5 (LGR5), mRNA [NM_003667]	8549

A_23_P23839	4.3	4.2	4.4	3.4	LGR6	Homo sapiens leucine-rich repeat containing G protein-coupled receptor 6 (LGR6), transcript variant 1, mRNA [NM_001017403]	59352
A_23_P218505	3.5	1.4	1.2	3.6	LHB	Homo sapiens luteinizing hormone beta polypeptide (LHB), mRNA [NM_000894]	3972
A_23_P102286	2.3	2.1	2.3	2.0	LHCGR	Homo sapiens luteinizing hormone/choriogonadotropin receptor (LHCGR), mRNA [NM_000233]	3973
A_23_P327499	-1.3	-1.2	-1.4	-2.3	LHFPL1	Homo sapiens lipoma HMGIC fusion partner-like 1 (LHFPL1), mRNA [NM_178175]	340596

A_23_P255104	-1.4	-1.0	-1.0	-5.2	LHFPL2	Homo sapiens lipoma HMGIC fusion partner-like 2 (LHFPL2), mRNA [NM_005779]	10184
A_23_P75299	-3.0	1.6	-1.0	-21.6	LHPP	Homo sapiens phospholysine phosphohistidine inorganic pyrophosphate phosphatase (LHPP), transcript variant 1, mRNA [NM_022126]	64077
A_24_P355493	1.2	-2.4	-1.1	-1.0	LHPP	Homo sapiens phospholysine phosphohistidine inorganic pyrophosphate phosphatase (LHPP), transcript variant 1, mRNA [NM_022126]	64077

A_23_P47967	4.1	2.6	2.4	3.0	LHX5	Homo sapiens LIM homeobox 5 (LHX5), mRNA [NM_022363]	64211
A_23_P32177	4.8	4.7	4.4	4.5	LHX6	Homo sapiens LIM homeobox 6 (LHX6), transcript variant 1, mRNA [NM_014368]	26468
A_23_P32175	1.0	1.0	-2.0	-1.0	LHX6	Homo sapiens LIM homeobox 6 (LHX6), transcript variant 1, mRNA [NM_014368]	26468
A_23_P327562	-1.6	-1.0	-1.2	-3.5	LHX9	Homo sapiens LIM homeobox 9 (LHX9), transcript variant 1, mRNA [NM_020204]	56956

A_24_P122137	6.2	4.6	1.9	3.1	LIF	Homo sapiens leukemia inhibitory factor (cholinergic differentiation factor) (LIF), mRNA [NM_002309]	3976
A_24_P233488	1.1	-1.7	-3.0	-2.2	LIF	Homo sapiens leukemia inhibitory factor (cholinergic differentiation factor) (LIF), mRNA [NM_002309]	3976
A_23_P133517	-4.0	4.0	1.1	1.1	LIFR	Homo sapiens leukemia inhibitory factor receptor alpha (LIFR), transcript variant 2, mRNA [NM_002310]	3977

A_24_P325992	2.8	4.4	4.4	2.6	LIFR	Homo sapiens leukemia inhibitory factor receptor alpha (LIFR), transcript variant 2, mRNA [NM_002310]	3977
A_24_P397386	-1.0	-1.1	-2.5	-1.1	LIFR	Homo sapiens leukemia inhibitory factor receptor alpha (LIFR), transcript variant 2, mRNA [NM_002310]	3977
A_23_P39116	2.0	1.1	1.9	1.1	LIG1	Homo sapiens ligase I, DNA, ATP-dependent (LIG1), mRNA [NM_000234]	3978

A_23_P501134	-1.6	-1.2	-2.4	1.4	LIG3	Homo sapiens ligase III, DNA, ATP-dependent (LIG3), nuclear gene encoding mitochondrial protein, transcript variant beta, mRNA [NM_002311]	3980
A_23_P25581	1.1	-1.0	-3.3	-1.1	LIG4	Homo sapiens ligase IV, DNA, ATP-dependent (LIG4), transcript variant 1, mRNA [NM_002312]	3981
A_24_P393151	2.3	1.9	4.8	-1.4	LILRA1	Homo sapiens leucocyte immunoglobulin-like receptor-6b (LIR-6) mRNA, complete cds. [AF025529]	11024

A_23_P142205	1.1	1.1	4.9	1.9	LILRA2	Homo sapiens leukocyte immunoglobulin-like receptor, subfamily A (with TM domain), member 2 (LILRA2), transcript variant 2, mRNA [NM_006866]	11027
A_23_P79094	-1.1	-2.2	-1.4	-1.6	LILRA3	Homo sapiens leukocyte immunoglobulin-like receptor, subfamily A (without TM domain), member 3 (LILRA3), transcript variant 1, mRNA [NM_006865]	11026

A_23_P90497	-4.4	-1.5	-5.6	-2.4	LILRA4	Homo sapiens leukocyte immunoglobulin-like receptor, subfamily A (with TM domain), member 4 (LILRA4), mRNA [NM_012276]	23547
A_23_P107847	-9.3	1.6	-1.8	1.6	LILRA5	Homo sapiens leukocyte immunoglobulin-like receptor, subfamily A (with TM domain), member 5 (LILRA5), transcript variant 2, mRNA [NM_181985]	353514

A_23_P343221	1.6	9.0	4.8	6.8	LILRB1	Homo sapiens leukocyte immunoglobulin-like receptor, subfamily B (with TM and ITIM domains), member 1 (LILRB1), transcript variant 1, mRNA [NM_006669]	10859
A_23_P208493	-1.0	-1.0	-1.0	-2.8	LILRB2	Homo sapiens leukocyte immunoglobulin-like receptor, subfamily B (with TM and ITIM domains), member 2 (LILRB2), transcript variant 1, mRNA [NM_005874]	10288

A_23_P164784	-1.9	-1.6	-2.5	-1.0	LILRB5	Homo sapiens leukocyte immunoglobulin-like receptor, subfamily B (with TM and ITIM domains), member 5 (LILRB5), transcript variant 2, mRNA [NM_006840]	10990
A_23_P27781	1.5	1.4	-7.9	-1.3	LILRP2	Homo sapiens leukocyte immunoglobulin-like receptor pseudogene 2 (LILRP2), non-coding RNA [NR_003061]	79166
A_24_P159635	5.9	6.7	6.7	4.3	LIMD2	Homo sapiens LIM domain containing 2 (LIMD2), mRNA [NM_030576]	80774

A_24_P168232	2.2	1.2	-1.2	-4.4	LIMD2	Homo sapiens LIM domain containing 2 (LIMD2), mRNA [NM_030576]	80774
A_23_P376088	6.3	7.9	2.0	4.4	LIME1	Homo sapiens Lck interacting transmembrane adaptor 1 (LIME1), mRNA [NM_017806]	54923
A_23_P211445	-1.0	-2.2	-1.1	-1.1	LIMK2	Homo sapiens LIM domain kinase 2 (LIMK2), transcript variant 2b, mRNA [NM_016733]	3985
A_24_P353300	2.3	3.5	1.5	1.7	LIMK2	Homo sapiens LIM domain kinase 2 (LIMK2), transcript variant 1, mRNA [NM_001031801]	3985

A_23_P142796	1.6	1.6	-2.4	1.1	LIMS2	Homo sapiens LIM and senescent cell antigen-like domains 2 (LIMS2), transcript variant 2, mRNA [NM_017980]	55679
A_24_P527716	1.0	1.7	1.5	3.2	LIMS3-LOC44	Homo sapiens LIMS3-LOC440895 readthrough (LIMS3-LOC440895), non-coding RNA [NR_027145]	100271835
A_24_P769529	1.0	-1.0	-2.0	-1.1	LIN28B	Homo sapiens lin-28 homolog B (C. elegans) (LIN28B), mRNA [NM_001004317]	389421
A_23_P165186	3.4	8.7	1.1	1.2	LIN37	Homo sapiens lin-37 homolog (C. elegans) (LIN37), mRNA [NM_019104]	55957

A_24_P867201	-4.4	1.1	1.0	1.0	LIN7A	lin-7 homolog A (C. elegans) [Source:HGNC Symbol;Acc:17787] [ENST00000552864]	8825
A_23_P164912	-1.1	1.3	-1.2	-7.5	LIN7B	Homo sapiens lin-7 homolog B (C. elegans) (LIN7B), mRNA [NM_022165]	64130
A_23_P206092	1.0	3.1	-2.5	1.9	LINGO1	Homo sapiens leucine rich repeat and Ig domain containing 1 (LINGO1), mRNA [NM_032808]	84894
A_24_P247626	-1.0	2.1	-1.1	1.7	LINGO4	Homo sapiens leucine rich repeat and Ig domain containing 4 (LINGO4), mRNA [NM_001004432]	339398

A_23_P205944	1.9	-1.1	1.0	-3.6	LINS	Homo sapiens lines homolog (Drosophila) (LINS), transcript variant 6, mRNA [NM_001040616]	55180
A_24_P198629	-1.0	-1.0	-1.1	-4.4	LINS	Homo sapiens lines homolog (Drosophila) (LINS), transcript variant 6, mRNA [NM_001040616]	55180
A_23_P97860	1.0	-1.4	-2.0	-1.9	LIPA	Homo sapiens lipase A, lysosomal acid, cholesterol esterase (LIPA), transcript variant 2, mRNA [NM_000235]	3988
A_23_P38876	-1.7	-2.9	-3.1	-18.3	LIPE	Homo sapiens lipase, hormone-sensitive (LIPE), mRNA [NM_005357]	3991

A_24_P125469	1.1	5.8	-2.9	1.0	LIPG	Homo sapiens lipase, endothelial (LIPG), mRNA [NM_006033]	9388
A_24_P799858	1.0	-1.4	-2.8	-1.5	LIPH	lipase, member H [Source:HGNC Symbol;Acc:18483] [ENST00000296252]	200879
A_23_P129738	-1.8	2.1	1.2	-1.1	LLGL2	Homo sapiens lethal giant larvae homolog 2 (Drosophila) (LLGL2), transcript variant 1, mRNA [NM_004524]	3993
A_23_P25097	-4.8	-1.2	-3.1	-1.3	LLPH	Homo sapiens LLP homolog, long-term synaptic facilitation (Aplysia) (LLPH), mRNA [NM_032338]	84298

A_24_P316127	2.2	-1.7	-3.5	-6.3	LLPH	Homo sapiens DC30 mRNA, complete cds. [AF255792]	84298
A_23_P215214	2.0	2.0	2.0	1.0	LMBR1	Homo sapiens limb region 1 homolog (mouse) (LMBR1), mRNA [NM_022458]	64327
A_23_P327698	1.0	1.0	-1.0	-2.7	LMBRD2	Homo sapiens LMBR1 domain containing 2 (LMBRD2), mRNA [NM_001007527]	92255
A_32_P105406	-1.0	-2.5	-2.2	1.0	LMBRD2	Homo sapiens LMBR1 domain containing 2 (LMBRD2), mRNA [NM_001007527]	92255

A_23_P88947	-2.3	-2.5	-5.9	-2.6	LMF1	Homo sapiens lipase maturation factor 1 (LMF1), transcript variant 1, mRNA [NM_022773]	64788
A_24_P18917	-2.1	1.3	-2.1	1.2	LMF1	Homo sapiens lipase maturation factor 1 (LMF1), transcript variant 1, mRNA [NM_022773]	64788
A_24_P367812	1.4	-2.4	-3.4	-1.0	LMF1	Homo sapiens hypothetical protein LOC650392, mRNA (cDNA clone IMAGE:5242623), partial cds. [BC028099]	64788
A_32_P527232	-1.1	1.2	-1.5	-2.2	LMF2	Homo sapiens lipase maturation factor 2 (LMF2), mRNA [NM_033200]	91289

A_23_P34835	1.1	-3.4	-1.3	-1.3	LMNA	Homo sapiens lamin A/C (LMNA), transcript variant 2, mRNA [NM_005572]	4000
A_23_P205159	2.0	2.0	2.8	1.6	LMO7	Homo sapiens LIM domain 7 (LMO7), transcript variant 1, mRNA [NM_005358]	4008
A_23_P201940	1.6	2.1	-1.2	-2.3	LMOD1	Homo sapiens leiomodin 1 (smooth muscle) (LMOD1), mRNA [NM_012134]	25802
A_24_P249253	1.4	1.4	1.3	2.1	LMOD2	Homo sapiens leiomodin 2 (cardiac) (LMOD2), mRNA [NM_207163]	442721
A_24_P76821	16.0	12.6	12.3	13.4	LMOD3	Homo sapiens leiomodin 3 (fetal) (LMOD3), mRNA [NM_198271]	56203

A_23_P340271	-1.1	3.4	-1.5	-2.5	LMX1A	Homo sapiens LIM homeobox transcription factor 1, alpha (LMX1A), transcript variant 1, mRNA [NM_177398]	4009
A_24_P141227	-2.4	-1.5	-1.8	-1.1	LMX1B	Homo sapiens LIM homeobox transcription factor 1, beta (LMX1B), transcript variant 1, mRNA [NM_002316]	4010
A_23_P257593	12.0	11.7	11.3	11.0	LOH12CR1	Homo sapiens loss of heterozygosity, 12, chromosomal region 1 (LOH12CR1), mRNA [NM_058169]	118426

A_24_P406480	-2.0	1.0	-2.3	1.7	LONRF1	Homo sapiens LON peptidase N-terminal domain and ring finger 1 (LONRF1), mRNA [NM_152271]	91694
A_32_P194032	-1.0	-1.1	-27.3	-1.1	LONRF1	Homo sapiens LON peptidase N-terminal domain and ring finger 1 (LONRF1), mRNA [NM_152271]	91694
A_24_P185909	2.1	2.2	-1.5	1.9	LONRF3	Homo sapiens LON peptidase N-terminal domain and ring finger 3 (LONRF3), transcript variant 1, mRNA [NM_001031855]	79836

A_23_P114414	6.1	1.5	2.5	4.8	LONRF3	Homo sapiens LON peptidase N-terminal domain and ring finger 3 (LONRF3), transcript variant 1, mRNA [NM_001031855]	79836
A_24_P196216	1.7	-1.1	-2.5	1.1	LOXHD1	Homo sapiens lipoxygenase homology domains 1 (LOXHD1), transcript variant 1, mRNA [NM_144612]	125336
A_24_P88763	-1.0	-2.4	-1.1	-1.1	LOXL3	Homo sapiens lysyl oxidase-like 3 (LOXL3), mRNA [NM_032603]	84695
A_23_P12755	-2.4	-3.6	-2.2	-2.3	LOXL4	Homo sapiens lysyl oxidase-like 4 (LOXL4), mRNA [NM_032211]	84171

A_24_P41801	-1.1	-3.4	-3.1	-3.3	LPA	Homo sapiens lipoprotein, Lp(a) (LPA), mRNA [NM_005577]	4018
A_23_P502879	1.1	1.1	-2.1	1.0	LPAR1	Homo sapiens lysophosphatidic acid receptor 1 (LPAR1), transcript variant 2, mRNA [NM_057159]	1902
A_23_P2705	-2.6	1.0	-4.6	-2.6	LPAR6	Homo sapiens lysophosphatidic acid receptor 6 (LPAR6), transcript variant 1, mRNA [NM_005767]	10161
A_23_P213471	-4.6	-1.8	-1.3	-1.7	LPCAT1	Homo sapiens lysophosphatidylcholine acyltransferase 1 (LPCAT1), mRNA [NM_024830]	79888

A_23_P391926	1.0	-1.7	-1.2	-3.2	LPHN1	Homo sapiens latrophilin 1 (LPHN1), transcript variant 1, mRNA [NM_001008701]	22859
A_23_P74042	1.5	1.8	1.9	4.0	LPHN2	Homo sapiens latrophilin 2 (LPHN2), mRNA [NM_012302]	23266
A_32_P52609	2.9	2.9	2.8	2.0	LPIN1	Homo sapiens lipin 1 (LPIN1), mRNA [NM_145693]	23175
A_24_P301557	-2.2	-2.9	-1.2	-1.3	LPIN2	Homo sapiens lipin 2 (LPIN2), mRNA [NM_014646]	9663
A_23_P208055	2.1	2.4	-2.3	2.2	LPIN2	Homo sapiens lipin 2 (LPIN2), mRNA [NM_014646]	9663
A_24_P100695	1.1	-1.4	1.1	-3.4	LPIN3	Homo sapiens lipin 3 (LPIN3), mRNA [NM_022896]	64900

A_24_P916515	1.9	1.8	1.8	2.1	LPP	LIM domain containing preferred translocation partner in lipoma [Source:HGNC Symbol;Acc:6679] [ENST00000483938]	4026
A_23_P259707	-6.4	1.1	1.1	1.1	LPPR1	Homo sapiens lipid phosphate phosphatase-related protein type 1 (LPPR1), transcript variant 1, mRNA [NM_207299]	54886
A_24_P151213	7.3	2.1	-3.1	-1.3	LPPR2	Homo sapiens lipid phosphate phosphatase-related protein type 2 (LPPR2), transcript variant 1, mRNA [NM_022737]	64748

A_23_P153461	1.1	1.0	1.1	-5.4	LPPR2	Homo sapiens lipid phosphate phosphatase-related protein type 2 (LPPR2), transcript variant 1, mRNA [NM_022737]	64748
A_23_P67569	-1.0	3.1	-1.9	1.8	LPPR3	Homo sapiens lipid phosphate phosphatase-related protein type 3 (LPPR3), mRNA [NM_024888]	79948
A_32_P148538	1.0	-3.4	1.4	1.0	LPPR4	Homo sapiens lipid phosphate phosphatase-related protein type 4 (LPPR4), transcript variant 1, mRNA [NM_014839]	9890

A_32_P80413	2.2	1.5	-1.1	2.7	LPPR5	Homo sapiens lipid phosphate phosphatase-related protein type 5 (LPPR5), transcript variant 2, mRNA [NM_001010861]	163404
A_23_P87150	-1.4	2.1	-1.6	2.3	LPXN	Homo sapiens leupaxin (LPXN), transcript variant 2, mRNA [NM_004811]	9404
A_23_P167182	-3.6	1.0	-1.4	-1.1	LRAT	Homo sapiens lecithin retinol acyltransferase (phosphatidylcholine--retinol O-acyltransferase) (LRAT), mRNA [NM_004744]	9227

A_24_P360078	-3.6	-3.2	-1.0	1.0	LRBA	Homo sapiens LPS-responsive vesicle trafficking, beach and anchor containing (LRBA), transcript variant 2, mRNA [NM_006726]	987
A_24_P170774	3.2	3.0	-2.5	3.1	LRCH1	Homo sapiens leucine-rich repeats and calponin homology (CH) domain containing 1 (LRCH1), transcript variant 2, mRNA [NM_015116]	23143
A_23_P355364	4.2	11.3	7.2	4.1	LRCH3	Homo sapiens leucine-rich repeats and calponin homology (CH) domain containing 3 (LRCH3), mRNA [NM_032773]	84859

A_24_P40775	-1.3	1.4	1.7	-2.1	LRFN1	Homo sapiens leucine rich repeat and fibronectin type III domain containing 1 (LRFN1), mRNA [NM_020862]	57622
A_24_P93870	1.7	2.7	1.6	2.8	LRFN1	Homo sapiens leucine rich repeat and fibronectin type III domain containing 1, mRNA (cDNA clone IMAGE:3860672), partial cds. [BC014678]	57622
A_32_P82111	-3.6	1.3	-2.7	1.5	LRFN2	Homo sapiens leucine rich repeat and fibronectin type III domain containing 2 (LRFN2), mRNA [NM_020737]	57497

A_24_P117053	1.7	2.6	2.2	1.9	LRFN3	Homo sapiens leucine rich repeat and fibronectin type III domain containing 3 (LRFN3), mRNA [NM_024509]	79414
A_23_P163195	2.3	1.9	2.1	1.1	LRFN5	Homo sapiens leucine rich repeat and fibronectin type III domain containing 5 (LRFN5), mRNA [NM_152447]	145581
A_23_P50638	1.0	-1.4	-3.2	-1.4	LRG1	Homo sapiens leucine-rich alpha-2-glycoprotein 1 (LRG1), mRNA [NM_052972]	116844

A_24_P346517	6.0	2.2	4.3	4.9	LRGUK	Homo sapiens leucine-rich repeats and guanylate kinase domain containing (LRGUK), mRNA [NM_144648]	136332
A_23_P160869	1.0	-1.0	-2.1	-1.1	LRIG2	Homo sapiens leucine-rich repeats and immunoglobulin-like domains 2 (LRIG2), mRNA [NM_014813]	9860
A_23_P47885	1.3	-1.1	-1.4	-2.2	LRIG3	Homo sapiens leucine-rich repeats and immunoglobulin-like domains 3 (LRIG3), transcript variant 2, mRNA [NM_153377]	121227

A_23_P98910	2.5	-1.9	-1.4	-1.2	LRMP	Homo sapiens lymphoid-restricted membrane protein (LRMP), transcript variant 1, mRNA [NM_006152]	4033
A_24_P926770	-1.5	1.9	-2.4	1.1	LRP10	Homo sapiens low density lipoprotein receptor-related protein 10 (LRP10), mRNA [NM_014045]	26020
A_23_P205499	1.6	-1.6	2.3	-3.1	LRP10	Homo sapiens low density lipoprotein receptor-related protein 10 (LRP10), mRNA [NM_014045]	26020

A_24_P415012	-4.6	1.0	-1.0	1.1	LRP12	Homo sapiens low density lipoprotein receptor-related protein 12 (LRP12), transcript variant 1, mRNA [NM_013437]	29967
A_24_P944741	-1.0	-6.2	-1.2	-1.1	LRP2BP	Homo sapiens LRP2 binding protein (LRP2BP), mRNA [NM_018409]	55805
A_23_P1505	2.4	1.6	2.7	3.0	LRP5	Homo sapiens low density lipoprotein receptor-related protein 5 (LRP5), mRNA [NM_002335]	4041
A_23_P392457	1.1	1.0	2.8	1.1	LRP6	Homo sapiens low density lipoprotein receptor-related protein 6 (LRP6), mRNA [NM_002336]	4040

A_23_P150827	-1.7	-2.2	-1.5	-4.8	LRP6	Homo sapiens low density lipoprotein receptor-related protein 6 (LRP6), mRNA [NM_002336]	4040
A_23_P34325	1.9	2.2	1.4	1.7	LRP8	Homo sapiens low density lipoprotein receptor-related protein 8, apolipoprotein e receptor (LRP8), transcript variant 2, mRNA [NM_033300]	7804
A_24_P196568	-1.3	-4.9	1.3	-1.0	LRPAP1	Homo sapiens low density lipoprotein receptor-related protein associated protein 1 (LRPAP1), mRNA [NM_002337]	4043

A_32_P460399	1.1	-2.9	1.1	1.1	LRPAP1	Homo sapiens low density lipoprotein receptor-related protein associated protein 1 (LRPAP1), mRNA [NM_002337]	4043
A_24_P277331	3.6	-1.3	-1.3	-1.1	LRPAP1	Homo sapiens low density lipoprotein receptor-related protein associated protein 1 (LRPAP1), mRNA [NM_002337]	4043
A_24_P26073	2.5	1.8	2.4	2.0	LRPPRC	Homo sapiens leucine-rich PPR-motif containing (LRPPRC), mRNA [NM_133259]	10128

A_24_P460419	-1.0	-1.1	-2.7	-1.1	LRRC10B	Homo sapiens leucine rich repeat containing 10B (LRRC10B), mRNA [NM_001145077]	390205
A_23_P134946	-2.0	1.1	1.0	1.0	LRRC14	Homo sapiens leucine rich repeat containing 14 (LRRC14), mRNA [NM_014665]	9684
A_23_P166584	-1.4	-1.3	-4.3	-1.3	LRRC15	Homo sapiens leucine rich repeat containing 15 (LRRC15), transcript variant 2, mRNA [NM_130830]	131578
A_24_P751327	5.1	4.7	4.5	5.0	LRRC15	Homo sapiens leucine rich repeat containing 15 (LRRC15), transcript variant 2, mRNA [NM_130830]	131578

A_24_P290079	-2.0	-2.0	-1.7	-2.1	LRRC16A	leucine rich repeat containing 16A [Source:HGNC Symbol;Acc:21581] [ENST00000476458]	55604
A_23_P19369	-1.2	1.2	1.1	-2.2	LRRC16A	Homo sapiens leucine rich repeat containing 16A (LRRC16A), transcript variant 1, mRNA [NM_017640]	55604
A_23_P35494	2.2	2.2	-2.1	-2.9	LRRC18	leucine rich repeat containing 18 [Source:HGNC Symbol;Acc:23199] [ENST00000298124]	474354

A_24_P321125	-1.2	-2.1	-2.9	-2.5	LRRC18	Homo sapiens leucine rich repeat containing 18 (LRRC18), mRNA [NM_001006939]	474354
A_23_P334798	-1.2	-1.2	-3.8	-1.1	LRRC2	Homo sapiens leucine rich repeat containing 2 (LRRC2), mRNA [NM_024512]	79442
A_23_P155463	-1.3	5.1	-2.6	-1.6	LRRC2	Homo sapiens leucine rich repeat containing 2 (LRRC2), mRNA [NM_024512]	79442
A_23_P61487	-8.8	-1.1	-1.2	-1.2	LRRC20	Homo sapiens leucine rich repeat containing 20 (LRRC20), transcript variant 3, mRNA [NM_018205]	55222

A_23_P36689	1.2	-1.9	-2.5	-2.7	LRRC23	Homo sapiens leucine rich repeat containing 23 (LRRC23), transcript variant 2, mRNA [NM_006992]	10233
A_23_P165136	1.0	1.0	1.5	3.1	LRRC25	Homo sapiens leucine rich repeat containing 25 (LRRC25), mRNA [NM_145256]	126364
A_24_P142151	-2.2	-1.8	-1.1	1.4	LRRC28	Homo sapiens leucine rich repeat containing 28 (LRRC28), mRNA [NM_144598]	123355
A_23_P333150	2.3	-4.6	-1.6	2.9	LRRC28	leucine rich repeat containing 28 [Source:HGNC Symbol;Acc:28355] [ENST00000301981]	123355

A_24_P281036	-2.2	-2.1	-2.4	-2.4	LRRC31	Homo sapiens leucine rich repeat containing 31 (LRRC31), mRNA [NM_024727]	79782
A_24_P240259	-3.0	1.0	1.0	1.0	LRRC31	Homo sapiens leucine rich repeat containing 31 (LRRC31), mRNA [NM_024727]	79782
A_24_P389916	1.8	3.5	-1.5	2.6	LRRC32	Homo sapiens leucine rich repeat containing 32 (LRRC32), transcript variant 1, mRNA [NM_005512]	2615
A_23_P41194	1.0	1.0	-1.4	-2.2	LRRC34	Homo sapiens leucine rich repeat containing 34 (LRRC34), transcript variant 3, mRNA [NM_153353]	151827

A_24_P666482	1.3	-2.3	-1.3	-1.0	LRRC37A2	Homo sapiens leucine rich repeat containing 37, member A2 (LRRC37A2), mRNA [NM_001006607]	474170
A_23_P331400	-1.0	-2.8	1.3	-2.3	LRRC37A3	Homo sapiens leucine rich repeat containing 37, member A3 (LRRC37A3), mRNA [NM_199340]	374819
A_32_P94660	2.3	2.2	2.0	2.0	LRRC37B	Homo sapiens leucine rich repeat containing 37B (LRRC37B), mRNA [NM_052888]	114659

A_24_P144983	2.3	1.3	1.3	-1.3	LRRC37BP1	Homo sapiens leucine rich repeat containing 37B pseudogene 1 (LRRC37BP1), non-coding RNA [NR_015341]	147172
A_32_P28402	1.0	-1.0	2.1	-1.0	LRRC37BP1	Homo sapiens leucine rich repeat containing 37B pseudogene 1 (LRRC37BP1), non-coding RNA [NR_015341]	147172
A_24_P307674	6.2	1.6	5.4	2.7	LRRC41	leucine rich repeat containing 41 [Source:HGNC Symbol;Acc:16917] [ENST00000254454]	10489

A_24_P276791	4.6	4.7	4.3	2.7	LRRC42	Homo sapiens leucine rich repeat containing 42 (LRRC42), mRNA [NM_052940]	115353
A_23_P381461	-3.0	4.5	1.5	-1.2	LRRC45	Homo sapiens leucine rich repeat containing 45 (LRRC45), mRNA [NM_144999]	201255
A_24_P19662	3.0	4.7	3.1	4.9	LRRC47	Homo sapiens leucine rich repeat containing 47 (LRRC47), mRNA [NM_020710]	57470
A_23_P96775	-4.4	-1.0	-1.1	-1.1	LRRC47	Homo sapiens leucine rich repeat containing 47 (LRRC47), mRNA [NM_020710]	57470

A_24_P270971	-2.4	-1.2	-1.3	-1.2	LRRC48	Homo sapiens leucine rich repeat containing 48 (LRRC48), transcript variant 2, mRNA [NM_031294]	83450
A_23_P24457	2.9	-1.3	2.5	2.5	LRRC4C	Homo sapiens leucine rich repeat containing 4C (LRRC4C), mRNA [NM_020929]	57689
A_24_P819890	-9.3	-1.0	-3.5	-1.0	LRRC55	Homo sapiens leucine rich repeat containing 55 (LRRC55), mRNA [NM_001005210]	219527
A_24_P1389	-1.0	-1.1	-2.8	-1.1	LRRC55	Homo sapiens leucine rich repeat containing 55 (LRRC55), mRNA [NM_001005210]	219527

A_24_P181585	-1.1	-2.1	-2.1	1.1	LRRC59	Homo sapiens leucine rich repeat containing 59 (LRRC59), mRNA [NM_018509]	55379
A_23_P112004	-1.5	-1.9	-1.3	-3.3	LRRC6	Homo sapiens leucine rich repeat containing 6 (LRRC6), mRNA [NM_012472]	23639
A_23_P41664	-38.6	-2.1	1.1	1.0	LRRC70	Homo sapiens leucine rich repeat containing 70 (LRRC70), mRNA [NM_181506]	100130733
A_32_P222149	3.0	1.3	1.0	-1.3	LRRC72	Homo sapiens leucine rich repeat containing 72 (LRRC72), mRNA [NM_001195280]	100506049

A_24_P376287	3.6	2.5	3.9	1.9	LRRC8A	Homo sapiens leucine rich repeat containing 8 family, member A (LRRC8A), transcript variant 2, mRNA [NM_019594]	56262
A_24_P943949	2.1	1.5	-1.1	3.7	LRRC8B	Homo sapiens leucine rich repeat containing 8 family, member B (LRRC8B), transcript variant 1, mRNA [NM_015350]	23507
A_23_P103775	1.6	3.2	3.4	3.5	LRRC8C	Homo sapiens leucine rich repeat containing 8 family, member C (LRRC8C), mRNA [NM_032270]	84230

A_23_P157527	2.2	2.2	2.1	2.2	LRRCC1	Homo sapiens leucine rich repeat and coiled-coil domain containing 1 (LRRCC1), mRNA [NM_033402]	85444
A_23_P331072	1.4	1.4	-3.4	-8.0	LRRIQ3	leucine-rich repeats and IQ motif containing 3 [Source:HGNC Symbol;Acc:28318] [ENST00000463724]	127255
A_23_P128447	1.1	1.1	2.5	1.1	LRRK2	Homo sapiens leucine-rich repeat kinase 2 (LRRK2), mRNA [NM_198578]	120892

A_23_P11843	2.7	1.2	1.5	-1.1	LRRN2	Homo sapiens leucine rich repeat neuronal 2 (LRRN2), transcript variant 2, mRNA [NM_201630]	10446
A_24_P187766	-2.2	-1.0	-1.4	-1.5	LRRN3	Homo sapiens leucine rich repeat neuronal 3 (LRRN3), transcript variant 3, mRNA [NM_018334]	54674
A_23_P120545	-1.0	-6.0	-1.0	-1.0	LRRN4	Homo sapiens cDNA FLJ23994 fis, clone HRC11286. [AK172833]	164312
A_23_P366999	-1.0	1.2	-1.0	-8.2	LRRTM3	Homo sapiens leucine rich repeat transmembrane neuronal 3 (LRRTM3), mRNA [NM_178011]	347731

A_24_P60268	-2.3	-4.1	-2.2	-2.1	LRRTM4	Homo sapiens leucine rich repeat transmembrane neuronal 4 (LRRTM4), transcript variant 2, mRNA [NM_024993]	80059
A_24_P666105	-1.0	-2.9	-1.0	1.0	LRRTM4	Homo sapiens leucine rich repeat transmembrane neuronal 4 (LRRTM4), transcript variant 1, mRNA [NM_001134745]	80059
A_23_P123916	-1.4	-2.7	-3.4	-1.6	LRSAM1	Homo sapiens leucine rich repeat and sterile alpha motif containing 1 (LRSAM1), transcript variant 1, mRNA [NM_138361]	90678

A_23_P318616	1.0	1.0	2.0	1.8	LRTM2	Homo sapiens leucine-rich repeats and transmembrane domains 2 (LRTM2), transcript variant 1, mRNA [NM_001039029]	654429
A_24_P357914	4.2	1.1	3.5	2.0	LRTOMT	Homo sapiens leucine rich transmembrane and 0-methyltransferase domain containing (LRTOMT), transcript variant 4, mRNA [NM_001145308]	220074

A_24_P184385	1.7	1.0	2.2	1.9	LRTOMT	Homo sapiens leucine rich transmembrane and 0-methyltransferase domain containing (LRTOMT), transcript variant 1, mRNA [NM_145309]	220074
A_23_P98763	2.5	-1.1	1.4	-1.3	LRTOMT	Homo sapiens leucine rich transmembrane and 0-methyltransferase domain containing (LRTOMT), transcript variant 1, mRNA [NM_145309]	220074

A_23_P360874	1.6	1.2	1.2	-2.8	LRWD1	Homo sapiens leucine-rich repeats and WD repeat domain containing 1 (LRWD1), mRNA [NM_152892]	222229
A_23_P301855	-1.2	2.0	-3.2	-1.8	LSAMP	Homo sapiens limbic system-associated membrane protein (LSAMP), mRNA [NM_002338]	4045
A_24_P148657	1.6	-2.7	1.0	-1.2	LSG1	Homo sapiens large subunit GTPase 1 homolog (S. cerevisiae) (LSG1), mRNA [NM_018385]	55341

A_23_P20384	1.5	1.4	1.3	2.0	LSM1	Homo sapiens LSM1 homolog, U6 small nuclear RNA associated (S. cerevisiae) (LSM1), transcript variant 1, mRNA [NM_014462]	27257
A_23_P307430	1.5	2.6	-1.7	1.9	LSM12	Homo sapiens LSM12 homolog (S. cerevisiae) (LSM12), mRNA [NM_152344]	124801
A_32_P95537	-6.7	-1.0	-1.5	-1.0	LSM12	Homo sapiens LSM12 homolog (S. cerevisiae) (LSM12), mRNA [NM_152344]	124801

A_24_P365327	1.4	2.5	-1.1	-1.2	LSM14A	Homo sapiens LSM14A, SCD6 homolog A (S. cerevisiae) (LSM14A), transcript variant 2, mRNA [NM_015578]	26065
A_23_P362719	1.2	-1.3	2.0	-3.1	LSM14B	Homo sapiens LSM14B, SCD6 homolog B (S. cerevisiae) (LSM14B), mRNA [NM_144703]	149986
A_23_P59153	-2.1	2.6	1.8	1.4	LSM2	Homo sapiens LSM2 homolog, U6 small nuclear RNA associated (S. cerevisiae) (LSM2), mRNA [NM_021177]	57819

A_23_P421563	-1.6	-6.4	-6.2	-2.1	LSM3	Homo sapiens LSM3 homolog, U6 small nuclear RNA associated (S. cerevisiae) (LSM3), mRNA [NM_014463]	27258
A_23_P259451	-2.0	-1.0	-1.1	-1.4	LSM6	Homo sapiens LSM6 homolog, U6 small nuclear RNA associated (S. cerevisiae) (LSM6), mRNA [NM_007080]	11157
A_23_P38206	-9.1	1.0	1.0	1.0	LSMD1	Homo sapiens LSM domain containing 1 (LSMD1), mRNA [NM_032356]	84316
A_23_P358555	-1.9	-5.7	-1.7	-5.6	LSMD1	Homo sapiens LSM domain containing 1 (LSMD1), mRNA [NM_032356]	84316

A_23_P142389	-2.0	1.3	1.2	-2.3	LSR	Homo sapiens lipolysis stimulated lipoprotein receptor (LSR), transcript variant 2, mRNA [NM_205834]	51599
A_24_P384839	3.3	3.3	-1.4	2.2	LSS	Homo sapiens lanosterol synthase (2,3-oxidosqualene-lanosterol cyclase) (LSS), transcript variant 1, mRNA [NM_002340]	4047
A_24_P94916	3.5	2.4	1.2	-2.0	LST1	Homo sapiens leukocyte specific transcript 1 (LST1), transcript variant 1, mRNA [NM_007161]	7940

A_23_P156683	-4.1	1.6	1.0	-1.4	LTA	Homo sapiens lymphotoxin alpha (TNF superfamily, member 1) (LTA), transcript variant 2, mRNA [NM_000595]	4049
A_23_P151791	-2.6	-1.3	-1.9	-3.0	LTB4R	Homo sapiens leukotriene B4 receptor (LTB4R), transcript variant 1, mRNA [NM_181657]	1241
A_23_P163173	1.2	1.0	-3.8	-2.2	LTB4R2	Homo sapiens leukotriene B4 receptor 2 (LTB4R2), transcript variant 1, mRNA [NM_019839]	56413

A_23_P405129	-2.1	1.0	-3.9	1.0	LTBP2	Homo sapiens latent transforming growth factor beta binding protein 2 (LTBP2), mRNA [NM_000428]	4053
A_24_P167668	-3.1	-5.5	-4.4	-1.4	LTBP2	Homo sapiens latent transforming growth factor beta binding protein 2 (LTBP2), mRNA [NM_000428]	4053
A_24_P176173	1.9	2.1	1.4	1.5	LTBP2	Homo sapiens latent transforming growth factor beta binding protein 2 (LTBP2), mRNA [NM_000428]	4053

A_24_P264790	-2.2	-2.6	1.4	-2.1	LTBP3	Homo sapiens latent transforming growth factor beta binding protein 3 (LTBP3), transcript variant 2, mRNA [NM_021070]	4054
A_24_P298360	-4.0	1.5	1.4	1.0	LTBP3	Homo sapiens latent transforming growth factor beta binding protein 3 (LTBP3), transcript variant 2, mRNA [NM_021070]	4054
A_23_P141946	2.3	2.9	-1.3	1.7	LTBP4	Homo sapiens latent transforming growth factor beta binding protein 4 (LTBP4), transcript variant 1, mRNA [NM_001042544]	8425

A_24_P397294	1.4	-4.5	-1.4	-1.5	LTC4S	Homo sapiens leukotriene C4 synthase (LTC4S), mRNA [NM_145867]	4056
A_23_P14853	-1.3	5.3	-1.3	-1.5	LTK	Homo sapiens leukocyte receptor tyrosine kinase (LTK), transcript variant 1, mRNA [NM_002344]	4058
A_32_P115505	-2.6	1.0	-1.0	1.1	LTN1	Homo sapiens listerin E3 ubiquitin protein ligase 1 (LTN1), mRNA [NM_015565]	26046
A_23_P106737	1.2	1.3	-2.7	-1.0	LUC7L	Homo sapiens LUC7-like (S. cerevisiae) (LUC7L), transcript variant 2, mRNA [NM_201412]	55692

A_23_P59787	1.1	1.1	-2.3	1.5	LUC7L2	Homo sapiens LUC7-like 2 (S. cerevisiae) (LUC7L2), transcript variant 1, mRNA [NM_016019]	51631
A_24_P382113	1.9	1.9	2.5	1.6	LUC7L3	Homo sapiens LUC7-like 3 (S. cerevisiae) (LUC7L3), transcript variant 2, mRNA [NM_006107]	51747
A_23_P385084	1.1	1.1	-23.6	-1.0	LUZP1	Homo sapiens mRNA for FLJ00226 protein. [AK074153]	7798
A_24_P649282	1.1	1.1	1.0	-2.0	LUZP2	Homo sapiens leucine zipper protein 2 (LUZP2), transcript variant 1, mRNA [NM_001009909]	338645

A_23_P96611	-2.3	-1.4	-1.3	-1.3	LUZP4	Homo sapiens leucine zipper protein 4 (LUZP4), mRNA [NM_016383]	51213
A_23_P6674	6.7	9.4	2.1	1.7	LXN	Homo sapiens latexin (LXN), mRNA [NM_020169]	56925
A_23_P134764	-3.3	2.2	1.4	-1.2	LY6D	Homo sapiens lymphocyte antigen 6 complex, locus D (LY6D), mRNA [NM_003695]	8581
A_32_P138348	-1.0	-1.0	-2.5	-2.1	LY6K	Homo sapiens lymphocyte antigen 6 complex, locus K (LY6K), transcript variant 1, mRNA [NM_017527]	54742

A_23_P397285	-1.0	-1.1	-3.3	2.8	LY6K	Homo sapiens lymphocyte antigen 6 complex, locus K (LY6K), transcript variant 1, mRNA [NM_017527]	54742
A_23_P70688	2.7	3.4	2.8	1.0	LY86	Homo sapiens lymphocyte antigen 86 (LY86), mRNA [NM_004271]	9450
A_24_P324674	1.0	1.6	2.6	2.7	LY9	Homo sapiens lymphocyte antigen 9 (LY9), transcript variant 1, mRNA [NM_002348]	4063
A_23_P41327	1.7	2.1	-1.5	2.7	LYAR	Homo sapiens Ly1 antibody reactive homolog (mouse) (LYAR), transcript variant 1, mRNA [NM_017816]	55646

A_23_P165707	-1.0	-1.0	-1.0	-2.6	LYG1	Homo sapiens lysozyme G-like 1 (LYG1), mRNA [NM_174898]	129530
A_23_P5281	-1.1	1.7	-2.6	-1.2	LYL1	Homo sapiens lymphoblastic leukemia derived sequence 1 (LYL1), mRNA [NM_005583]	4066
A_23_P111981	-1.2	2.2	1.5	-1.6	LYNX1	Homo sapiens Ly6/neurotoxin 1 (LYNX1), transcript variant 3, mRNA [NM_177457]	66004
A_24_P341863	1.3	1.6	-2.1	-1.1	LYNX1	Homo sapiens Ly6/neurotoxin 1 (LYNX1), transcript variant 1, mRNA [NM_023946]	66004

A_23_P257672	1.2	3.1	-1.2	1.5	LYNX1	Homo sapiens Ly6/neurotoxin 1 (LYNX1), transcript variant SLURP2, mRNA [NM_177458]	66004
A_23_P39265	1.9	1.8	2.5	2.2	LYPD3	Homo sapiens LY6/PLAUR domain containing 3 (LYPD3), mRNA [NM_014400]	27076
A_23_P311780	-2.1	1.4	-1.5	1.1	LYPD4	LY6/PLAUR domain containing 4 [Source:HGNC Symbol;Acc:28659] [ENST00000330743]	147719
A_23_P79302	-2.9	1.7	1.7	1.8	LYPD6B	Homo sapiens LY6/PLAUR domain containing 6B (LYPD6B), mRNA [NM_177964]	130576

A_23_P214091	1.2	2.1	1.1	-1.2	LYPLA1	Homo sapiens lysophospholipase I (LYPLA1), mRNA [NM_006330]	10434
A_23_P42036	2.4	-1.1	2.9	-1.3	LYRM2	Homo sapiens LYR motif containing 2 (LYRM2), transcript variant 1, mRNA [NM_020466]	57226
A_24_P312072	-4.2	-5.5	-1.4	-1.4	LYRM4	Homo sapiens LYR motif containing 4 (LYRM4), transcript variant 1, mRNA [NM_020408]	57128
A_23_P254997	3.3	3.4	6.2	2.2	LYRM4	Homo sapiens LYR motif containing 4 (LYRM4), transcript variant 1, mRNA [NM_020408]	57128

A_23_P337464	66.4	65.1	65.9	66.2	LYRM7	Homo sapiens Lyrm7 homolog (mouse) (LYRM7), mRNA [NM_181705]	90624
A_32_P120895	-1.0	-1.0	-3.0	1.0	LYSMD2	Homo sapiens LysM, putative peptidoglycan-binding, domain containing 2 (LYSMD2), transcript variant 1, mRNA [NM_153374]	256586
A_23_P354074	2.0	2.1	2.1	2.1	LYST	Homo sapiens lysosomal trafficking regulator (LYST), mRNA [NM_000081]	1130
A_23_P46740	2.2	-1.1	1.1	-1.2	LYZL1	Homo sapiens lysozyme-like 1 (LYZL1), mRNA [NM_032517]	84569

A_24_P37519	3.4	3.3	3.0	3.2	LZTFL1	Homo sapiens leucine zipper transcription factor-like 1 (LZTFL1), mRNA [NM_020347]	54585
A_23_P57401	-1.3	-1.1	-1.6	-2.3	LZTR1	Homo sapiens leucine-zipper-like transcription regulator 1 (LZTR1), mRNA [NM_006767]	8216
A_23_P149852	1.9	1.0	2.2	-1.2	LZTS2	Homo sapiens leucine zipper, putative tumor suppressor 2 (LZTS2), mRNA [NM_032429]	84445
A_23_P149847	1.3	1.7	1.5	-2.2	LZTS2	Homo sapiens leucine zipper, putative tumor suppressor 2 (LZTS2), mRNA [NM_032429]	84445

A_24_P210343	3.5	2.0	1.9	2.0	M6PR	Homo sapiens mannose-6-phosphate receptor (cation dependent) (M6PR), transcript variant 1, mRNA [NM_002355]	4074
A_23_P53884	1.9	4.3	3.3	3.4	MAB21L1	Homo sapiens mab-21-like 1 (C. elegans) (MAB21L1), mRNA [NM_005584]	4081
A_24_P148907	1.3	-1.1	-2.8	1.1	MAB21L2	Homo sapiens mab-21-like 2 (C. elegans) (MAB21L2), mRNA [NM_006439]	10586
A_23_P343366	1.1	-2.0	1.4	-2.0	MAB21L3	Homo sapiens mab-21-like 3 (C. elegans) (MAB21L3), mRNA [NM_152367]	126868

A_24_P233946	3.0	3.1	3.2	2.4	MAB21L3	Homo sapiens mab-21-like 3 (C. elegans) (MAB21L3), mRNA [NM_152367]	126868
A_32_P158272	-7.3	-3.8	-1.2	-1.0	MACC1	Homo sapiens metastasis associated in colon cancer 1 (MACC1), mRNA [NM_182762]	346389
A_24_P418637	-3.6	-3.9	-2.8	-3.0	MACF1	Homo sapiens microtubule-actin crosslinking factor 1 (MACF1), transcript variant 1, mRNA [NM_012090]	23499
A_23_P143334	2.0	-1.2	-2.8	1.1	MACROD2	MACRO domain containing 2 [Source:HGNC Symbol;Acc:16126] [ENST00000462552]	140733

A_32_P89352	-6.4	1.1	1.1	-1.1	MACROD2	Homo sapiens MACRO domain containing 2 (MACROD2), transcript variant 1, mRNA [NM_080676]	140733
A_23_P22398	-2.3	-1.0	-1.1	1.0	MACROD2	Homo sapiens MACRO domain containing 2 (MACROD2), transcript variant 1, mRNA [NM_080676]	140733
A_23_P92441	1.3	2.7	2.6	2.7	MAD2L1	Homo sapiens MAD2 mitotic arrest deficient-like 1 (yeast) (MAD2L1), mRNA [NM_002358]	4085

A_24_P43588	1.8	1.2	-2.2	1.6	MAD2L1BP	Homo sapiens MAD2L1 binding protein (MAD2L1BP), transcript variant 1, mRNA [NM_001003690]	9587
A_23_P153616	4.9	2.5	1.9	1.9	MADCAM1	Homo sapiens mucosal vascular addressin cell adhesion molecule 1 (MADCAM1), transcript variant 1, mRNA [NM_130760]	8174
A_24_P10731	2.2	1.2	2.2	2.1	MADCAM1	Homo sapiens mucosal vascular addressin cell adhesion molecule 1 (MADCAM1), transcript variant 1, mRNA [NM_130760]	8174

A_23_P202708	3.0	4.7	2.1	2.3	MADD	Homo sapiens MAP-kinase activating death domain (MADD), transcript variant 4, mRNA [NM_003682]	8567
A_23_P114934	1.1	1.0	-1.1	-7.5	MAEL	Homo sapiens maelstrom homolog (Drosophila) (MAEL), mRNA [NM_032858]	84944
A_23_P397376	1.4	-2.2	1.6	1.7	MAF	Homo sapiens v-maf musculoaponeurotic fibrosarcoma oncogene homolog (avian) (MAF), transcript variant 2, mRNA [NM_001031804]	4094

A_24_P127719	-3.0	-1.3	-2.8	-1.8	MAFA	Homo sapiens v-maf musculoaponeurotic fibrosarcoma oncogene homolog A (avian) (MAFA), mRNA [NM_201589]	389692
A_23_P17345	-1.2	-2.8	-7.1	-1.0	MAFB	Homo sapiens v-maf musculoaponeurotic fibrosarcoma oncogene homolog B (avian) (MAFB), mRNA [NM_005461]	9935
A_23_P373598	-1.0	-5.9	-1.1	-1.0	MAFK	Homo sapiens v-maf musculoaponeurotic fibrosarcoma oncogene homolog K (avian) (MAFK), mRNA [NM_002360]	7975

A_24_P279704	1.3	-3.0	1.9	1.1	MAG	Homo sapiens myelin associated glycoprotein (MAG), transcript variant 2, mRNA [NM_080600]	4099
A_23_P96291	-6.1	1.0	-1.0	1.0	MAGEA1	Homo sapiens melanoma antigen family A, 1 (directs expression of antigen MZ2-E) (MAGEA1), mRNA [NM_004988]	4100
A_23_P113553	-3.5	-3.5	-3.9	-3.9	MAGEA11	Homo sapiens melanoma antigen family A, 11 (MAGEA11), transcript variant 2, mRNA [NM_001011544]	4110

A_23_P252928	1.2	-3.3	1.4	1.1	MAGEA12	Homo sapiens melanoma antigen family A, 12 (MAGEA12), transcript variant 3, mRNA [NM_005367]	4111
A_23_P148255	8.3	11.6	11.2	12.1	MAGEA2B	Homo sapiens melanoma antigen family A, 2B (MAGEA2B), mRNA [NM_153488]	266740
A_23_P370054	-1.0	-1.1	-35.9	-1.4	MAGEB18	Homo sapiens melanoma antigen family B, 18 (MAGEB18), mRNA [NM_173699]	286514
A_23_P254831	1.6	2.2	2.0	1.4	MAGEB2	Homo sapiens melanoma antigen family B, 2 (MAGEB2), mRNA [NM_002364]	4113

A_23_P96432	-2.9	-1.1	-1.1	-1.0	MAGEB3	Homo sapiens melanoma antigen family B, 3 (MAGEB3) , mRNA [NM_0023 65]	4114
A_23_P378574	-3.0	-1.0	-1.1	-1.1	MAGEB6	Homo sapiens melanoma antigen family B, 6 (MAGEB6) , mRNA [NM_1735 23]	158809
A_23_P135634	1.6	-2.4	1.3	-1.5	MAGED1	Homo sapiens NRAGE mRNA, complete cds. [AF217963]	9500
A_24_P391526	3.3	3.2	3.2	3.2	MAGED1	Homo sapiens melanoma antigen family D, 1 (MAGED1) , transcript variant 1, mRNA [NM_0010 05333]	9500

A_24_P160263	-2.0	-2.2	-3.6	1.0	MAGED2	Homo sapiens melanoma antigen family D, 2 (MAGED2), transcript variant 3, mRNA [NM_201222]	10916
A_23_P114445	3.5	6.8	3.6	2.8	MAGEE1	Homo sapiens melanoma antigen family E, 1 (MAGEE1), mRNA [NM_020932]	57692
A_23_P21376	2.3	2.2	2.1	2.2	MAGI2	Homo sapiens membrane associated guanylate kinase, WW and PDZ domain containing 2 (MAGI2), mRNA [NM_012301]	9863

A_24_P748377	-1.4	-1.1	1.7	-3.2	MAGI2-AS3	Homo sapiens MAGI2 antisense RNA 3 (non-protein coding) (MAGI2-AS3), transcript variant 1, non-coding RNA [NR_038343]	100505881
A_24_P66105	3.2	3.0	1.0	1.7	MAGIX	MAGI family member, X-linked [Source:HGNC Symbol;Acc:30006] [ENST00000376338]	79917
A_24_P828949	-2.0	1.1	-1.1	-1.3	MAGOHB	Homo sapiens mago-nashi homolog B (Drosophila) (MAGOHB), mRNA [NM_018048]	55110

A_23_P2423	-1.1	-1.9	-3.2	-1.7	MAGOHB	Homo sapiens mago-nashi homolog B (Drosophila) (MAGOHB), mRNA [NM_018048]	55110
A_23_P148273	-2.1	1.3	-2.4	-1.0	MAGT1	Homo sapiens magnesium transporter 1 (MAGT1), mRNA [NM_032121]	84061
A_23_P96008	-13.3	-1.4	-3.8	-1.5	MALT1	Homo sapiens mucosa associated lymphoid tissue lymphoma translocation gene 1 (MALT1), transcript variant 1, mRNA [NM_006785]	10892

A_24_P116909	-2.6	-1.0	-1.3	-1.3	MALT1	Homo sapiens mucosa associated lymphoid tissue lymphoma translocation gene 1 (MALT1), transcript variant 1, mRNA [NM_006785]	10892
A_24_P117709	1.0	1.0	1.2	-2.2	MAML3	Homo sapiens mastermind-like 3 (Drosophila) (MAML3), mRNA [NM_018717]	55534
A_24_P50208	-4.4	1.5	-1.2	-1.1	MAML3	mastermind-like 3 (Drosophila) [Source:HGNC Symbol;Acc:16272] [ENST00000398940]	55534

A_24_P297537	-1.3	2.1	1.8	-1.3	MAMSTR	Homo sapiens MEF2 activating motif and SAP domain containing transcriptional regulator (MAMSTR), transcript variant 2, mRNA [NM_182574]	284358
A_32_P88603	1.6	-2.7	-2.0	2.2	MAN1A2	mannosidase, alpha, class 1A, member 2 [Source:HGNC Symbol;Acc:6822] [ENST00000356554]	10905
A_23_P157943	2.4	2.6	1.8	-1.0	MAN1B1	Homo sapiens mannosidase, alpha, class 1B, member 1 (MAN1B1), mRNA [NM_016219]	11253

A_23_P18931	-1.0	1.4	2.7	-4.4	MAN2A1	Homo sapiens mannosidase, alpha, class 2A, member 1 (MAN2A1), mRNA [NM_002372]	4124
A_24_P381838	-4.3	-1.9	-2.5	1.1	MAN2A2	Homo sapiens mannosidase, alpha, class 2A, member 2 (MAN2A2), mRNA [NM_006122]	4122
A_23_P206041	-1.0	-1.0	-3.2	-3.7	MAN2A2	Homo sapiens mannosidase, alpha, class 2A, member 2 (MAN2A2), mRNA [NM_006122]	4122
A_23_P27613	-1.2	2.7	-1.4	2.1	MAN2B1	Homo sapiens lysosomal alpha-mannosidase (manB) mRNA, complete cds. [U60266]	4125

A_23_P250380	-2.1	-1.1	1.2	-1.7	MAN2B2	Homo sapiens mannosidase, alpha, class 2B, member 2 (MAN2B2), mRNA [NM_015274]	23324
A_23_P250379	-1.5	1.1	-3.0	-1.1	MAN2B2	Homo sapiens mannosidase, alpha, class 2B, member 2 (MAN2B2), mRNA [NM_015274]	23324
A_24_P416059	1.6	3.6	-1.5	1.5	MAN2C1	Homo sapiens mannosidase, alpha, class 2C, member 1 (MAN2C1), mRNA [NM_006715]	4123
A_23_P26117	8.6	-5.8	2.1	6.8	MAN2C1	Homo sapiens mannosidase, alpha, class 2C, member 1 (MAN2C1), mRNA [NM_006715]	4123

A_23_P258698	-14.1	1.7	-1.0	1.0	MANBA	Homo sapiens mannosidase, beta A, lysosomal (MANBA), mRNA [NM_005908]	4126
A_23_P255663	-5.7	-1.1	-1.2	-1.3	MANEA	Homo sapiens mannosidase, endo-alpha (MANEA), mRNA [NM_024641]	79694
A_24_P69274	2.7	2.6	2.6	2.7	MANEA	Homo sapiens mannosidase, endo-alpha (MANEA), mRNA [NM_024641]	79694
A_23_P163455	2.3	2.5	2.8	2.5	MAP1A	Homo sapiens microtubule-associated protein 1A (MAP1A), mRNA [NM_002373]	4130

A_24_P879740	1.5	1.8	2.3	-1.5	MAP1B	Homo sapiens microtubule-associated protein 1B (MAP1B), mRNA [NM_005909]	4131
A_23_P154786	1.4	1.6	-2.0	-1.1	MAP1LC3A	Homo sapiens microtubule-associated protein 1 light chain 3 alpha (MAP1LC3A), transcript variant 1, mRNA [NM_032514]	84557
A_23_P20248	2.1	1.3	-1.2	1.7	MAP2K1	Homo sapiens mitogen-activated protein kinase kinase 1 (MAP2K1), mRNA [NM_002755]	5604

A_23_P208835	7.6	10.9	-1.1	2.9	MAP2K2	Homo sapiens mitogen-activated protein kinase kinase 2 (MAP2K2), mRNA [NM_030662]	5605
A_23_P84256	1.0	-1.0	1.0	-6.9	MAP2K5	Homo sapiens mitogen-activated protein kinase kinase 5 (MAP2K5), transcript variant 2, mRNA [NM_002757]	5607
A_24_P356130	2.1	1.7	2.3	1.6	MAP2K5	Homo sapiens mitogen-activated protein kinase kinase 5 (MAP2K5), transcript variant 2, mRNA [NM_002757]	5607

A_23_P207445	1.2	-5.4	1.1	1.1	MAP2K6	Homo sapiens mitogen-activated protein kinase kinase 6 (MAP2K6), mRNA [NM_002758]	5608
A_24_P313895	-1.4	-1.2	-1.6	-3.1	MAP2K7	mitogen-activated protein kinase kinase 7 [Source:HGNC Symbol;Acc:6847] [ENST00000475022]	5609
A_23_P78628	1.4	1.4	2.2	1.1	MAP2K7	Homo sapiens mitogen-activated protein kinase kinase 7 (MAP2K7), mRNA [NM_145185]	5609
A_23_P41796	1.2	1.1	-2.7	1.1	MAP3K1	Homo sapiens mitogen-activated protein kinase kinase kinase 1 (MAP3K1), mRNA [NM_005921]	4214

A_23_P98275	3.1	2.1	1.9	1.7	MAP3K11	Homo sapiens mitogen-activated protein kinase kinase kinase 11 (MAP3K11), mRNA [NM_002419]	4296
A_23_P105409	1.1	-3.2	-1.2	-1.6	MAP3K12	Homo sapiens mitogen-activated protein kinase kinase kinase 12 (MAP3K12), transcript variant 2, mRNA [NM_006301]	7786
A_24_P85537	-1.0	-1.0	-2.2	-1.0	MAP3K13	Homo sapiens mitogen-activated protein kinase kinase kinase 13 (MAP3K13), transcript variant 1, mRNA [NM_004721]	9175

A_23_P207319	2.9	1.8	3.1	1.8	MAP3K14	Homo sapiens mitogen-activated protein kinase kinase kinase 14 (MAP3K14), mRNA [NM_003954]	9020
A_23_P207138	2.8	2.7	2.3	-1.2	MAP3K3	Homo sapiens mitogen-activated protein kinase kinase kinase 3 (MAP3K3), transcript variant 1, mRNA [NM_203351]	4215
A_23_P42096	-3.5	-1.4	-1.0	-1.8	MAP3K4	Homo sapiens mitogen-activated protein kinase kinase kinase 4 (MAP3K4), transcript variant 1, mRNA [NM_005922]	4216

A_24_P145653	2.8	5.6	2.1	1.0	MAP3K6	Homo sapiens mitogen-activated protein kinase kinase kinase 6 (MAP3K6), mRNA [NM_004672]	9064
A_23_P500773	4.6	4.3	4.1	4.2	MAP3K7	Homo sapiens mitogen-activated protein kinase kinase kinase 7 (MAP3K7), transcript variant A, mRNA [NM_003188]	6885
A_23_P211814	1.0	1.0	2.0	1.1	MAP4	Homo sapiens microtubule-associated protein 4 (MAP4), transcript variant 1, mRNA [NM_002375]	4134

A_24_P287075	-1.1	1.1	-2.5	-4.6	MAP4K2	Homo sapiens mitogen-activated protein kinase kinase kinase kinase 2 (MAP4K2), mRNA [NM_004579]	5871
A_23_P205646	-3.4	-1.2	-2.7	-1.3	MAP4K5	Homo sapiens mitogen-activated protein kinase kinase kinase kinase 5 (MAP4K5), transcript variant 2, mRNA [NM_198794]	11183
A_23_P350491	-3.5	-1.0	-1.9	-1.0	MAP4K5	Homo sapiens mitogen-activated protein kinase kinase kinase kinase 5 (MAP4K5), transcript variant 2, mRNA [NM_198794]	11183

A_24_P16997	-1.0	-1.0	-3.4	1.1	MAP4K5	Homo sapiens mitogen-activated protein kinase kinase kinase kinase 5 (MAP4K5), transcript variant 2, mRNA [NM_198794]	11183
A_23_P47728	1.9	3.9	2.6	5.3	MAP6	Homo sapiens microtubule-associated protein 6 (MAP6), transcript variant 1, mRNA [NM_033063]	4135
A_24_P388593	3.1	1.3	1.3	-1.2	MAP6D1	Homo sapiens MAP6 domain containing 1 (MAP6D1), mRNA [NM_024871]	79929

A_32_P190416	1.4	2.1	-1.3	1.9	MAP7	Homo sapiens microtubule-associated protein 7 (MAP7), transcript variant 4, mRNA [NM_003980]	9053
A_23_P149075	1.5	3.3	1.7	2.1	MAP7D1	Homo sapiens MAP7 domain containing 1 (MAP7D1), mRNA [NM_018067]	55700
A_24_P7652	-2.4	-2.5	-4.9	-2.3	MAP7D2	Homo sapiens MAP7 domain containing 2 (MAP7D2), transcript variant 2, mRNA [NM_152780]	256714
A_23_P11160	-4.4	1.0	-1.2	-1.4	MAP7D3	Homo sapiens MAP7 domain containing 3 (MAP7D3), transcript variant 1, mRNA [NM_024597]	79649

A_23_P45025	2.2	2.1	2.0	2.0	MAPK10	Homo sapiens mitogen-activated protein kinase 10 (MAPK10), transcript variant 3, mRNA [NM_138980]	5602
A_24_P283288	1.6	2.0	2.1	1.5	MAPK14	Homo sapiens mitogen-activated protein kinase 14 (MAPK14), transcript variant 3, mRNA [NM_139013]	1432
A_24_P397566	-4.8	-1.1	-1.1	-1.0	MAPK14	Homo sapiens mitogen-activated protein kinase 14 (MAPK14), transcript variant 3, mRNA [NM_139013]	1432

A_23_P60962	1.7	2.1	1.3	-5.8	MAPK15	Homo sapiens mitogen-activated protein kinase 15 (MAPK15), mRNA [NM_139021]	225689
A_24_P89835	2.2	1.3	-1.0	-1.0	MAPK15	Homo sapiens mitogen-activated protein kinase 15 (MAPK15), mRNA [NM_139021]	225689
A_32_P7823	5.8	5.8	5.5	3.4	MAPK1IP1L	Homo sapiens mitogen-activated protein kinase 1 interacting protein 1-like (MAPK1IP1L), mRNA [NM_144578]	93487
A_23_P3204	1.4	3.1	1.8	1.6	MAPK6	Homo sapiens mitogen-activated protein kinase 6 (MAPK6), mRNA [NM_002748]	5597

A_24_P286898	1.1	-3.9	1.1	-2.0	MAPK8	mitogen-activated protein kinase 8 [Source:HGNC Symbol;Acc:6881] [ENST00000374189]	5599
A_24_P409595	-6.6	1.4	-1.5	-1.0	MAPK8IP1	Homo sapiens mitogen-activated protein kinase 8 interacting protein 1 (MAPK8IP1), mRNA [NM_005456]	9479
A_23_P502078	2.6	1.1	3.6	2.0	MAPK8IP2	Homo sapiens mitogen-activated protein kinase 8 interacting protein 2 (MAPK8IP2), transcript variant 1, mRNA [NM_012324]	23542

A_23_P329727	2.3	2.2	2.0	1.6	MAPK8IP3	Homo sapiens mitogen-activated protein kinase 8 interacting protein 3 (MAPK8IP3), transcript variant 1, mRNA [NM_015133]	23162
A_23_P167692	-5.3	-5.0	-8.7	1.2	MAPK9	Homo sapiens mitogen-activated protein kinase 9 (MAPK9), transcript variant JNK2-a2, mRNA [NM_002752]	5601
A_24_P43884	-1.6	-1.8	-1.9	-2.2	MAPKAP1	Homo sapiens mitogen-activated protein kinase associated protein 1 (MAPKAP1), transcript variant 1, mRNA [NM_001006617]	79109

A_23_P201483	3.5	1.1	1.8	1.9	MAPKAPK2	Homo sapiens mitogen-activated protein kinase-activated protein kinase 2 (MAPKAPK2), transcript variant 1, mRNA [NM_004759]	9261
A_23_P314584	-3.9	-1.9	-1.5	-1.6	MAPKAPK3	Homo sapiens mitogen-activated protein kinase-activated protein kinase 3 (MAPKAPK3), transcript variant 3, mRNA [NM_004635]	7867
A_24_P220058	-69.4	-1.0	-1.0	-1.1	MAPRE1	Homo sapiens microtubule-associated protein, RP/EB family, member 1 (MAPRE1), mRNA [NM_012325]	22919

A_23_P418015	1.0	-2.8	-1.1	-1.1	MAPRE2	Homo sapiens microtubule-associated protein, RP/EB family, member 2 (MAPRE2), transcript variant 1, mRNA [NM_014268]	10982
A_23_P131683	4.0	3.9	3.7	3.9	MAPRE3	Homo sapiens microtubule-associated protein, RP/EB family, member 3 (MAPRE3), mRNA [NM_012326]	22924
A_23_P101992	1.6	1.7	1.6	2.2	MARCO	Homo sapiens macrophage receptor with collagenous structure (MARCO), mRNA [NM_006770]	8685

A_23_P161595	1.5	2.5	1.5	1.5	MARK2	Homo sapiens MAP/microtubule affinity-regulating kinase 2 (MARK2), transcript variant 1, mRNA [NM_017490]	2011
A_23_P140111	2.1	2.1	2.0	-1.4	MARK3	Homo sapiens MAP/microtubule affinity-regulating kinase 3 (MARK3), transcript variant 3, mRNA [NM_002376]	4140
A_24_P175909	-1.6	1.3	-1.6	-2.2	MARS	Homo sapiens methionyl-tRNA synthetase (MARS), mRNA [NM_004990]	4141
A_24_P106363	-1.3	-1.4	-4.3	-2.2	MARVELD2	Homo sapiens MARVEL domain containing 2 (MARVELD2), transcript variant 1, mRNA [NM_001038603]	153562

A_23_P152428	1.0	1.0	-3.0	1.0	MARVELD3	Homo sapiens MARVEL domain containing 3 (MARVELD 3), transcript variant 2, mRNA [NM_0528 58]	91862
A_23_P168339	-1.0	-4.0	-1.0	-1.0	MAS1	Homo sapiens MAS1 oncogene (MAS1), mRNA [NM_0023 77]	4142
A_23_P214565	-1.2	-1.5	-2.6	-2.4	MAS1L	Homo sapiens MAS1 oncogene-like (MAS1L), mRNA [NM_0529 67]	116511

A_23_P212263	-1.0	-3.4	-1.0	-1.0	MASP1	Homo sapiens mannan-binding lectin serine peptidase 1 (C4/C2 activating component of Ra-reactive factor) (MASP1), transcript variant 2, mRNA [NM_139125]	5648
A_23_P85672	-1.4	-1.8	-1.7	-2.9	MASP2	Homo sapiens mannan-binding lectin serine peptidase 2 (MASP2), transcript variant 1, mRNA [NM_006610]	10747
A_23_P301971	2.9	1.1	-1.1	-1.0	MASP2	Homo sapiens mannan-binding lectin serine peptidase 2 (MASP2), transcript variant 2, mRNA [NM_139208]	10747

A_23_P126008	1.1	-8.2	1.3	1.0	MAST2	Homo sapiens microtubule associated serine/threonine kinase 2 (MAST2), mRNA [NM_015112]	23139
A_23_P426196	15.7	9.9	15.4	12.8	MAST3	Homo sapiens microtubule associated serine/threonine kinase 3 (MAST3), mRNA [NM_015016]	23031
A_23_P110571	1.4	-4.9	-1.3	2.0	MAST4	Homo sapiens microtubule associated serine/threonine kinase family member 4 (MAST4), transcript variant 3, mRNA [NM_001164664]	375449

A_24_P258051	-1.0	-1.1	-2.2	-1.0	MASTL	Homo sapiens microtubule associated serine/threonine kinase-like (MASTL), transcript variant 2, mRNA [NM_032844]	84930
A_23_P23996	-1.9	-2.2	-2.2	-4.2	MAT1A	Homo sapiens methionine adenosyltransferase I, alpha (MAT1A), mRNA [NM_000429]	4143
A_32_P902988	1.7	2.0	2.2	1.1	MATN1	Homo sapiens matrilin 1, cartilage matrix protein (MATN1), mRNA [NM_002379]	4146
A_23_P97677	1.3	-3.1	-1.4	1.0	MATN1	Homo sapiens matrilin 1, cartilage matrix protein (MATN1), mRNA [NM_002379]	4146

A_23_P70047	-2.6	-2.0	-1.1	-2.7	MATR3	Homo sapiens matrin 3 (MATR3), transcript variant 1, mRNA [NM_199189]	9782
A_32_P8361	1.3	1.2	1.1	-5.3	MAU2	Homo sapiens MAU2 chromatid cohesion factor homolog (C. elegans) (MAU2), mRNA [NM_015329]	23383
A_32_P8365	-1.1	2.0	1.6	-1.1	MAU2	Homo sapiens MAU2 chromatid cohesion factor homolog (C. elegans) (MAU2), mRNA [NM_015329]	23383

A_23_P68539	1.1	2.2	1.2	1.0	MAVS	Homo sapiens mitochondrial antiviral signaling protein (MAVS), nuclear gene encoding mitochondrial protein, transcript variant 1, mRNA [NM_020746]	57506
A_23_P205549	3.2	4.1	3.3	1.7	MAX	Homo sapiens MYC associated factor X (MAX), transcript variant 3, mRNA [NM_145113]	4149
A_23_P436138	-4.7	1.1	1.1	1.1	MAX	Homo sapiens MYC associated factor X (MAX), transcript variant 4, mRNA [NM_145114]	4149

A_24_P917178	6.4	3.8	2.1	4.1	MB	myoglobin [Source:HGNC Symbol;Acc:6915] [ENST00000472240]	4151
A_32_P1173	-2.6	1.8	1.8	1.0	MB21D1	Homo sapiens Mab-21 domain containing 1 (MB21D1), mRNA [NM_138441]	115004
A_23_P326963	-2.5	-1.2	1.4	1.2	MB21D2	Homo sapiens Mab-21 domain containing 2 (MB21D2), mRNA [NM_178496]	151963
A_24_P185347	1.3	2.5	1.5	1.2	MBD1	Homo sapiens methyl-CpG binding domain protein 1 (MBD1), transcript variant 2, mRNA [NM_015845]	4152

A_32_P107777	-2.1	-1.3	1.1	-1.9	MBD2	Homo sapiens methyl-CpG binding domain protein 2 (MBD2), transcript variant 1, mRNA [NM_003927]	8932
A_23_P50839	-1.2	-4.1	-2.1	-2.1	MBD3	Homo sapiens methyl-CpG binding domain protein 3 (MBD3), mRNA [NM_003926]	53615
A_23_P415395	1.1	-4.1	-1.5	-1.5	MBD3L1	Homo sapiens methyl-CpG binding domain protein 3-like 1 (MBD3L1), mRNA [NM_145208]	85509

A_23_P142537	20.0	19.7	17.3	19.9	MBD5	Homo sapiens methyl-CpG binding domain protein 5, mRNA (cDNA clone IMAGE:3996924). [BC014534]	55777
A_23_P2922	-1.0	-2.9	-2.2	-1.1	MBIP	Homo sapiens MAP3K12 binding inhibitory protein 1 (MBIP), transcript variant 1, mRNA [NM_016586]	51562
A_24_P169634	1.0	-39.5	-1.1	-1.2	MBL1P	Homo sapiens mannose-binding lectin (protein A) 1, pseudogene (MBL1P), non-coding RNA [NR_002724]	8512

A_24_P221198	-1.1	-1.1	-1.1	-2.1	MBLAC1	Homo sapiens metallo-beta-lactamase domain containing 1 (MBLAC1), mRNA [NM_203397]	255374
A_23_P390876	-3.6	1.0	-5.7	-1.0	MBLAC2	metallo-beta-lactamase domain containing 2 [Source:HGNC Symbol;Acc:33711] [ENST00000514906]	153364
A_24_P128057	1.7	2.2	1.5	1.2	MBNL1	Homo sapiens muscleblind-like (Drosophila), mRNA (cDNA clone IMAGE:3935812). [BC005296]	4154

A_32_P123788	2.1	1.6	2.0	1.2	MBOAT7	Homo sapiens membrane bound O-acyltransferase domain containing 7 (MBOAT7), transcript variant 1, mRNA [NM_024298]	79143
A_23_P208085	-1.2	1.3	-2.4	-1.2	MBP	Homo sapiens myelin basic protein (MBP), transcript variant 7, mRNA [NM_001025101]	4155
A_24_P935437	-1.4	1.1	-3.6	1.1	MBP	myelin basic protein [Source:HGNC Symbol;Acc:6925] [ENST00000490319]	4155
A_32_P58937	-3.5	1.7	-1.0	-1.1	MBTD1	Homo sapiens mbt domain containing 1 (MBTD1), mRNA [NM_017643]	54799

A_23_P14946	-3.0	1.1	1.1	-1.0	MBTPS1	Homo sapiens membrane-bound transcription factor peptidase, site 1 (MBTPS1), mRNA [NM_003791]	8720
A_23_P114095	9.8	6.6	-1.0	5.8	MBTPS2	Homo sapiens membrane-bound transcription factor peptidase, site 2 (MBTPS2), mRNA [NM_015884]	51360
A_23_P66095	-3.4	-1.2	1.7	1.8	MC1R	Homo sapiens melanocortin 1 receptor (alpha melanocyte stimulating hormone receptor) (MC1R), mRNA [NM_002386]	4157

A_23_P258525	-1.0	1.0	-9.2	-1.1	MC3R	Homo sapiens melanocortin 3 receptor (MC3R), mRNA [NM_019888]	4159
A_23_P50039	1.6	2.0	3.3	1.2	MC5R	Homo sapiens melanocortin 5 receptor (MC5R), mRNA [NM_005913]	4161
A_24_P326660	6.2	6.0	5.5	5.9	MCAM	Homo sapiens melanoma cell adhesion molecule (MCAM), mRNA [NM_006500]	4162
A_23_P112512	-1.7	3.8	3.4	1.8	MCART1	Homo sapiens mitochondrial carrier triple repeat 1 (MCART1), nuclear gene encoding mitochondrial protein, transcript variant 1, mRNA [NM_033412]	92014

A_23_P132358	-1.9	1.5	2.4	1.2	MCAT	Homo sapiens malonyl CoA:ACP acyltransferase (mitochondrial) (MCAT), nuclear gene encoding mitochondrial protein, transcript variant 1, mRNA [NM_173467]	27349
A_32_P20439	2.6	2.3	1.0	1.8	MCCD1	Homo sapiens mitochondrial coiled-coil domain 1 (MCCD1), nuclear gene encoding mitochondrial protein, mRNA [NM_001011700]	401250

A_23_P133904	-2.7	-1.9	-1.4	-2.7	MCCD1	Homo sapiens mitochondrial coiled-coil domain 1 (MCCD1), nuclear gene encoding mitochondrial protein, mRNA [NM_001011700]	401250
A_23_P211543	1.0	-7.4	-1.0	1.1	MCHR1	Homo sapiens melanin-concentrating hormone receptor 1 (MCHR1), mRNA [NM_005297]	2847
A_24_P98006	-1.6	1.2	-1.2	-4.5	MCHR2	Homo sapiens melanin-concentrating hormone receptor 2 (MCHR2), transcript variant 1, mRNA [NM_001040179]	84539

A_23_P156784	-1.3	1.0	1.0	-4.9	MCHR2	Homo sapiens melanin-concentrating hormone receptor 2 (MCHR2), transcript variant 1, mRNA [NM_001040179]	84539
A_32_P103633	2.4	1.6	2.8	2.7	MCM2	Homo sapiens minichromosome maintenance complex component 2 (MCM2), mRNA [NM_004526]	4171
A_24_P117301	-1.7	1.5	2.7	-3.7	MCM3AP-AS1	Homo sapiens MCM3AP antisense RNA 1 (non-protein coding) (MCM3AP-AS1), antisense RNA [NR_002776]	114044

A_23_P256694	1.2	1.5	-2.0	-1.1	MCM3AP-AS1	Homo sapiens MCM3AP antisense RNA 1 (non-protein coding) (MCM3AP-AS1), antisense RNA [NR_002776]	114044
A_23_P132277	1.0	2.2	-2.3	-1.7	MCM5	Homo sapiens minichromosome maintenance complex component 5 (MCM5), mRNA [NM_006739]	4174
A_23_P90612	1.1	-2.7	1.0	1.0	MCM6	Homo sapiens minichromosome maintenance complex component 6 (MCM6), mRNA [NM_005915]	4175

A_23_P68547	1.0	1.0	-2.6	1.2	MCM8	Homo sapiens minichromosome maintenance complex component 8 (MCM8), transcript variant 2, mRNA [NM_182802]	84515
A_23_P12241	-1.1	-1.1	-2.1	10.4	MCOLN3	Homo sapiens mucolipin 3 (MCOLN3), mRNA [NM_018298]	55283
A_24_P170936	2.9	1.8	1.3	1.4	MCPH1	Homo sapiens microcephalin 1 (MCPH1), transcript variant 2, mRNA [NM_001172574]	79648
A_24_P212481	1.6	1.1	-2.6	-1.0	MCTP1	Homo sapiens multiple C2 domains, transmembrane 1 (MCTP1), transcript variant L, mRNA [NM_024717]	79772

A_23_P65789	-2.0	-1.9	-2.0	-1.8	MCTP2	Homo sapiens multiple C2 domains, transmem brane 2 (MCTP2), transcript variant 1, mRNA [NM_0183 49]	55784
A_23_P114282	-1.5	-1.9	-5.8	-8.2	MCTS1	Homo sapiens malignant T cell amplified sequence 1 (MCTS1), transcript variant 1, mRNA [NM_0140 60]	28985
A_24_P392280	-1.1	-1.0	-3.0	-1.1	MCTS1	Homo sapiens malignant T cell amplified sequence 1 (MCTS1), transcript variant 1, mRNA [NM_0140 60]	28985

A_24_P808100	-1.8	-1.0	-1.1	-2.7	MCTS1	Homo sapiens malignant T cell amplified sequence 1 (MCTS1), transcript variant 1, mRNA [NM_014060]	28985
A_23_P346405	2.1	2.0	1.8	1.9	MCU	Homo sapiens mitochondrial calcium uniporter (MCU), nuclear gene encoding mitochondrial protein, mRNA [NM_138357]	90550
A_23_P42168	1.4	2.2	1.9	2.0	MDFI	Homo sapiens MyoD family inhibitor (MDFI), mRNA [NM_005586]	4188

A_32_P120567	1.1	1.4	-2.8	-1.2	MDFIC	Homo sapiens MyoD family inhibitor domain containing (MDFIC), transcript variant 1, mRNA [NM_199072]	29969
A_24_P314640	-1.5	6.2	2.1	2.5	MDGA1	Homo sapiens MAM domain containing glycosylphosphatidylinositol anchor 1 (MDGA1), mRNA [NM_153487]	266727
A_24_P46808	-3.9	-2.4	-1.3	-1.3	MDH2	malate dehydrogenase 2, NAD (mitochondrial) [Source:HGNC Symbol;Acc:6971] [ENST00000315758]	4191

A_23_P31372	-2.8	1.0	-2.1	1.7	MDH2	Homo sapiens malate dehydrogenase 2, NAD (mitochondrial) (MDH2), nuclear gene encoding mitochondrial protein, mRNA [NM_005918]	4191
A_23_P309545	2.5	1.0	2.2	3.1	MDM2	Homo sapiens Mdm2 p53 binding protein homolog (mouse) (MDM2), transcript variant MDM2, mRNA [NM_002392]	4193
A_24_P778649	1.8	1.7	2.5	-1.8	MDM4	Homo sapiens Mdm4 p53 binding protein homolog (mouse) (MDM4), transcript variant 1, mRNA [NM_002393]	4194

A_23_P365060	1.1	-3.2	-1.0	1.0	MDN1	Homo sapiens MDN1, midasin homolog (yeast) (MDN1), mRNA [NM_014611]	23195
A_23_P321261	1.2	-2.0	1.0	-2.2	ME2	Homo sapiens malic enzyme 2, NAD(+)-dependent, mitochondrial (ME2), nuclear gene encoding mitochondrial protein, transcript variant 1, mRNA [NM_002396]	4200

A_23_P105227	-1.2	-2.0	-1.4	-2.7	ME3	Homo sapiens malic enzyme 3, NADP(+)-dependent, mitochondrial (ME3), nuclear gene encoding mitochondrial protein, transcript variant 2, mRNA [NM_001014811]	10873
A_23_P30745	-1.3	1.1	1.1	-3.3	MEA1	Homo sapiens male-enhanced antigen 1 (MEA1), mRNA [NM_014623]	4201
A_23_P317324	1.8	1.8	1.0	-2.0	MECOM	Homo sapiens MDS1 and EVI1 complex locus (MECOM), transcript variant 2, mRNA [NM_005241]	2122

A_24_P320103	-4.6	1.1	1.1	1.1	MECOM	Homo sapiens MDS1 and EVI1 complex locus (MECOM), transcript variant 4, mRNA [NM_004991]	2122
A_24_P153490	-2.2	1.1	1.0	1.0	MECOM	Homo sapiens MDS1 and EVI1 complex locus (MECOM), transcript variant 6, mRNA [NM_001164000]	2122
A_23_P212688	1.3	-1.1	-19.5	-1.1	MECOM	Homo sapiens MDS1 and EVI1 complex locus (MECOM), transcript variant 2, mRNA [NM_005241]	2122

A_24_P237486	-2.1	1.3	1.3	-1.4	MECP2	Homo sapiens methyl CpG binding protein 2 (Rett syndrome) (MECP2), transcript variant 1, mRNA [NM_004992]	4204
A_23_P114808	1.3	2.1	1.1	1.2	MECR	Homo sapiens mitochondrial trans-2-enoyl-CoA reductase (MECR), nuclear gene encoding mitochondrial protein, transcript variant 2, mRNA [NM_001024732]	51102
A_23_P124024	1.4	1.3	-1.3	-4.1	MED10	Homo sapiens mediator complex subunit 10 (MED10), mRNA [NM_032286]	84246

A_23_P73702	-1.4	-2.2	-1.5	-1.1	MED12	Homo sapiens mediator complex subunit 12 (MED12), mRNA [NM_005120]	9968
A_23_P27247	-2.1	-4.6	-3.9	-4.2	MED13	Homo sapiens mediator complex subunit 13 (MED13), mRNA [NM_005121]	9969
A_23_P72187	2.0	2.1	2.1	1.3	MED14	Homo sapiens mediator complex subunit 14 (MED14), mRNA [NM_004229]	9282
A_24_P941459	1.1	-4.9	-1.3	1.0	MED17	mediator complex subunit 17 [Source:HGNC Symbol;Acc:2375] [ENST00000530819]	9440

A_23_P116025	-5.2	1.0	-1.0	-1.0	MED17	Homo sapiens mediator complex subunit 17 (MED17), mRNA [NM_004268]	9440
A_23_P2262	-1.0	-1.0	-2.3	-1.0	MED21	Homo sapiens mediator complex subunit 21 (MED21), mRNA [NM_004264]	9412
A_23_P501795	4.7	8.4	9.8	6.7	MED22	Homo sapiens mediator complex subunit 22 (MED22), transcript variant b, mRNA [NM_133640]	6837
A_24_P55719	2.0	2.5	-12.2	1.1	MED22	Homo sapiens mediator complex subunit 22 (MED22), transcript variant b, mRNA [NM_133640]	6837

A_23_P153510	-2.8	1.4	1.5	1.1	MED25	Homo sapiens mediator complex subunit 25 (MED25), mRNA [NM_030973]	81857
A_23_P317854	1.3	4.2	2.7	2.6	MED26	Homo sapiens mediator complex subunit 26 (MED26), mRNA [NM_004831]	9441
A_23_P301877	1.4	1.3	-2.8	1.4	MED27	Homo sapiens mediator complex subunit 27 (MED27), mRNA [NM_004269]	9442
A_24_P538478	4.0	3.8	1.0	3.8	MED28	Homo sapiens mediator complex subunit 28 (MED28), mRNA [NM_025205]	80306

A_32_P138330	1.1	-1.0	-1.1	5.3	MED29	Homo sapiens mediator complex subunit 29 (MED29), mRNA [NM_017592]	55588
A_24_P93887	2.1	-2.8	1.6	2.2	MED29	Homo sapiens mediator complex subunit 29 (MED29), mRNA [NM_017592]	55588
A_23_P128543	-3.4	-1.0	1.2	-1.0	MED4	Homo sapiens mediator complex subunit 4 (MED4), mRNA [NM_014166]	29079
A_24_P318319	3.9	4.5	5.7	4.7	MED6	Homo sapiens mediator complex subunit 6 (MED6), mRNA [NM_005466]	10001

A_23_P46170	1.0	-1.0	2.1	1.0	MED8	Homo sapiens mediator complex subunit 8 (MED8), transcript variant 3, mRNA [NM_052877]	112950
A_24_P8267	2.8	-1.8	-1.6	-1.6	MED9	Homo sapiens cDNA clone IMAGE:4095249, with apparent retained intron. [BC010906]	55090
A_23_P66402	-1.0	-1.0	-24.9	-1.1	MED9	Homo sapiens mediator complex subunit 9 (MED9), mRNA [NM_018019]	55090
A_23_P353652	1.8	-1.3	-1.7	5.4	MEF2A	Homo sapiens myocyte enhancer factor 2A (MEF2A), transcript variant 1, mRNA [NM_005587]	4205

A_24_P1731	-1.3	-2.2	-3.4	-3.5	MEF2A	Homo sapiens myocyte enhancer factor 2A (MEF2A), transcript variant 1, mRNA [NM_005587]	4205
A_24_P914940	1.7	2.3	-5.3	1.5	MEF2BNB	Homo sapiens MEF2B neighbor (MEF2BNB), transcript variant 1, mRNA [NM_001145784]	729991
A_24_P31583	2.6	-1.9	-1.4	-1.3	MEF2BNB-ME	Homo sapiens MEF2BNB-MEF2B readthrough (MEF2BNB-MEF2B), transcript variant 1, mRNA [NM_005919]	4207
A_23_P140967	2.2	3.5	1.7	-1.8	MEFV	Homo sapiens Mediterranean fever (MEFV), transcript variant 1, mRNA [NM_000243]	4210

A_24_P463973	5.3	5.2	5.1	5.0	MEG3	Homo sapiens cDNA FLJ41884 fis, clone OCBBF2023162. [AK123878]	55384
A_32_P129894	-1.2	1.5	1.5	-2.1	MEGF9	Homo sapiens multiple EGF-like-domains 9 (MEGF9), mRNA [NM_001080497]	1955
A_23_P211561	-1.3	-1.3	-1.4	-11.4	MEI1	Homo sapiens meiosis inhibitor 1 (MEI1), mRNA [NM_152513]	150365
A_32_P136516	1.4	2.7	3.1	1.9	MEI1	Homo sapiens meiosis inhibitor 1 (MEI1), mRNA [NM_152513]	150365
A_24_P319736	-2.8	-2.0	-1.5	-1.0	MEIS1	Homo sapiens Meis homeobox 1 (MEIS1), mRNA [NM_002398]	4211

A_24_P82200	1.4	-3.3	2.5	-1.7	MEIS2	Homo sapiens Meis homeobox 2 (MEIS2), transcript variant d, mRNA [NM_170676]	4212
A_24_P207503	-4.8	-1.4	1.9	-1.5	MEIS3	Homo sapiens Meis homeobox 3 (MEIS3), transcript variant 2, mRNA [NM_001009813]	56917
A_24_P648880	26.7	25.5	23.5	23.7	MEIS3P1	Homo sapiens Meis homeobox 3 pseudogene 1 (MEIS3P1), non-coding RNA [NR_002211]	4213
A_24_P154868	1.5	2.5	1.8	1.1	MEP1A	Homo sapiens meprin A, alpha (PABA peptide hydrolase) (MEP1A), mRNA [NM_005588]	4224

A_23_P78353	1.1	-17.4	1.0	1.0	MEP1B	Homo sapiens meprin A, beta (MEP1B), mRNA [NM_005925]	4225
A_24_P312189	1.1	-3.4	-1.4	-1.5	MEPCE	Homo sapiens methylphosphate capping enzyme (MEPCE), transcript variant 1, mRNA [NM_019606]	56257
A_23_P155931	-1.9	2.1	-1.5	1.8	MEPE	Homo sapiens matrix extracellular phosphoglycoprotein (MEPE), transcript variant 2, mRNA [NM_020203]	56955
A_23_P32955	-3.4	1.0	1.0	1.0	MERTK	c-mer proto-oncogene tyrosine kinase [Source:HGNC Symbol;Acc:7027] [ENST00000421804]	10461

A_24_P279121	-2.8	-9.6	-5.1	-3.9	MESP1	Homo sapiens mesoderm posterior 1 homolog (mouse) (MESP1), mRNA [NM_018670]	55897
A_32_P83803	-2.4	-12.3	-2.3	-3.1	MESTIT1	Homo sapiens MEST intronic transcript 1, antisense RNA (non-protein coding) (MESTIT1), non-coding RNA [NR_004382]	317751
A_24_P226076	1.3	-1.1	-2.3	1.3	METAP1	Homo sapiens methionyl aminopeptidase 1 (METAP1), mRNA [NM_015143]	23173
A_23_P155907	1.1	-2.0	1.0	1.4	METAP1	Homo sapiens methionyl aminopeptidase 1 (METAP1), mRNA [NM_015143]	23173

A_23_P90790	-1.0	-1.1	-1.1	-2.1	METAP1D	Homo sapiens methionyl aminopeptidase type 1D (mitochondrial) (METAP1D), nuclear gene encoding mitochondrial protein, mRNA [NM_199227]	254042
A_24_P339201	1.4	2.1	1.8	1.7	METRN	Homo sapiens meteorin, glial cell differentiation regulator (METRN), mRNA [NM_024042]	79006
A_23_P10591	1.3	-1.1	-3.7	1.4	METRNL	Homo sapiens meteorin, glial cell differentiation regulator-like (METRNL), mRNA [NM_001004431]	284207

A_23_P47790	-3.1	-8.5	-1.2	-2.2	METTL1	Homo sapiens methyltransferase like 1 (METTL1), transcript variant 1, mRNA [NM_005371]	4234
A_23_P47788	1.0	-13.7	1.6	-1.0	METTL1	Homo sapiens methyltransferase like 1 (METTL1), transcript variant 1, mRNA [NM_005371]	4234
A_23_P205529	3.5	2.2	2.4	2.4	METTL17	Homo sapiens methyltransferase like 17 (METTL17), transcript variant 2, mRNA [NM_022734]	64745
A_23_P29985	-1.0	1.1	1.3	-2.3	METTL19	Homo sapiens cDNA FLJ12891 fis, clone NT2RP2004142. [AK022953]	152992

A_23_P18590	1.6	2.3	-1.9	1.8	METTL19	Homo sapiens methyltransferase like 19 (METTL19), transcript variant 2, mRNA [NM_152544]	152992
A_23_P156809	-1.6	1.2	-1.3	-2.3	METTL21A	Homo sapiens methyltransferase like 21A (METTL21A), transcript variant 2, mRNA [NM_001127395]	151194
A_24_P162929	-6.7	-4.8	-3.2	-1.4	METTL21A	Homo sapiens methyltransferase like 21A (METTL21A), transcript variant 1, mRNA [NM_145280]	151194
A_24_P253078	-7.0	-1.0	1.2	3.3	METTL21B	Homo sapiens methyltransferase like 21B (METTL21B), transcript variant 2, mRNA [NM_206914]	25895

A_23_P3885	1.6	1.2	-4.3	-1.4	METTL22	Homo sapiens methyltransferase like 22 (METTL22), mRNA [NM_024109]	79091
A_23_P15284	2.0	2.4	1.9	1.7	METTL22	Homo sapiens methyltransferase like 22 (METTL22), mRNA [NM_024109]	79091
A_23_P54064	4.6	7.6	2.1	3.8	METTL3	Homo sapiens methyltransferase like 3 (METTL3), mRNA [NM_019852]	56339
A_23_P142634	3.6	4.2	2.3	3.2	METTL5	Homo sapiens methyltransferase like 5 (METTL5), mRNA [NM_014168]	29081
A_23_P361469	1.5	1.0	1.0	-4.0	METTL6	Homo sapiens methyltransferase like 6 (METTL6), mRNA [NM_152396]	131965

A_24_P202139	1.5	-2.6	-3.2	-1.1	METTL9	Homo sapiens methyltransferase like 9 (METTL9), transcript variant 1, mRNA [NM_016025]	51108
A_23_P206347	2.1	1.1	-1.1	1.0	METTL9	Homo sapiens methyltransferase like 9 (METTL9), transcript variant 1, mRNA [NM_016025]	51108
A_24_P112984	-3.0	1.9	1.4	-1.3	METTL9	Homo sapiens methyltransferase like 9 (METTL9), transcript variant 1, mRNA [NM_016025]	51108
A_24_P857404	2.1	1.4	1.3	1.3	MEX3A	Homo sapiens mex-3 homolog A (C. elegans) (MEX3A), mRNA [NM_001093725]	92312

A_24_P923765	2.0	2.0	1.6	1.4	MEX3D	Homo sapiens mex-3 homolog D (C. elegans) (MEX3D), transcript variant 1, mRNA [NM_203304]	399664
A_23_P39445	-1.6	-3.9	1.7	-2.2	MEX3D	Homo sapiens mex-3 homolog D (C. elegans) (MEX3D), transcript variant 1, mRNA [NM_203304]	399664
A_23_P1029	-9.5	1.0	-1.1	-1.1	MFAP2	Homo sapiens microfibrillar-associated protein 2 (MFAP2), transcript variant 1, mRNA [NM_017459]	4237

A_23_P167711	1.1	1.0	-2.3	1.0	MFAP3	Homo sapiens microfibrillar-associated protein 3 (MFAP3), transcript variant 1, mRNA [NM_005927]	4238
A_24_P76675	1.4	-1.3	-2.7	1.0	MFAP3L	Homo sapiens microfibrillar-associated protein 3-like (MFAP3L), transcript variant 1, mRNA [NM_021647]	9848
A_24_P133584	14.8	-4.9	-3.3	1.4	MFGE8	Homo sapiens milk fat globule-EGF factor 8 protein (MFGE8), transcript variant 1, mRNA [NM_005928]	4240

A_23_P48951	-1.1	-2.1	1.6	1.6	MFGE8	Homo sapiens milk fat globule-EGF factor 8 protein (MFGE8), transcript variant 1, mRNA [NM_005928]	4240
A_24_P924484	1.0	-1.0	-3.4	-1.1	MFI2	Human melanoma-associated antigen p97 (melanotransferrin) mRNA, 3' flank. [K03200]	4241
A_23_P386320	2.1	2.1	2.1	2.2	MFI2	Homo sapiens antigen p97 (melanoma associated) identified by monoclonal antibodies 133.2 and 96.5 (MFI2), transcript variant 2, mRNA [NM_033316]	4241

A_23_P103104	-1.7	-2.7	-1.1	-1.3	MFNG	Homo sapiens MFNG O-fucosylpeptide 3-beta-N-acetylglucosaminyltransferase (MFNG), transcript variant 1, mRNA [NM_002405]	4242
A_24_P224926	-1.1	-1.1	-1.1	-8.5	MFNG	Homo sapiens MFNG O-fucosylpeptide 3-beta-N-acetylglucosaminyltransferase (MFNG), transcript variant 1, mRNA [NM_002405]	4242
A_24_P98783	2.0	3.0	1.2	1.4	MFRP	Homo sapiens membrane frizzled-related protein (MFRP), mRNA [NM_031433]	83552

A_23_P166677	-2.3	1.0	1.0	1.0	MFSD1	Homo sapiens major facilitator superfamily domain containing 1 (MFSD1), transcript variant 1, mRNA [NM_022736]	64747
A_23_P41246	2.1	-1.4	1.8	1.9	MFSD10	Homo sapiens major facilitator superfamily domain containing 10 (MFSD10), transcript variant 1, mRNA [NM_001120]	10227
A_23_P43820	-1.0	-1.5	-3.3	-1.9	MFSD2A	Homo sapiens major facilitator superfamily domain containing 2A (MFSD2A), transcript variant 2, mRNA [NM_032793]	84879

A_23_P72157	1.0	-1.0	-1.6	-2.2	MFSD7	Homo sapiens major facilitator superfamily domain containing 7 (MFSD7), mRNA [NM_032219]	84179
A_23_P154158	-1.4	-1.3	-1.2	-2.2	MFSD9	Homo sapiens major facilitator superfamily domain containing 9 (MFSD9), mRNA [NM_032718]	84804
A_23_P154155	-2.0	-1.0	1.3	1.4	MFSD9	Homo sapiens major facilitator superfamily domain containing 9 (MFSD9), mRNA [NM_032718]	84804
A_23_P37654	-1.6	-1.7	-2.3	-1.7	MGA	Homo sapiens MAX gene associated (MGA), transcript variant 1, mRNA [NM_001164273]	23269

A_23_P49052	-1.2	2.9	2.2	1.7	MGA	Homo sapiens MAX gene associated (MGA), transcript variant 1, mRNA [NM_001164273]	23269
A_23_P65558	-4.4	1.6	1.6	1.0	MGAT2	Homo sapiens mannosyl (alpha-1,6-)-glycoprotein beta-1,2-N-acetylglucosaminyltransferase (MGAT2), mRNA [NM_002408]	4247
A_23_P91742	1.0	-1.8	-3.2	2.8	MGAT3	Homo sapiens mannosyl (beta-1,4-)-glycoprotein beta-1,4-N-acetylglucosaminyltransferase (MGAT3), transcript variant 1, mRNA [NM_002409]	4248

A_23_P28507	-7.9	4.0	1.3	1.7	MGAT4A	Homo sapiens mannosyl (alpha-1,3-)-glycoprotein beta-1,4-N-acetylglucosaminyltransferase, isozyme A (MGAT4A), transcript variant 1, mRNA [NM_012214]	11320
A_23_P10062	-2.6	1.0	1.0	1.1	MGAT4C	Homo sapiens mannosyl (alpha-1,3-)-glycoprotein beta-1,4-N-acetylglucosaminyltransferase, isozyme C (putative) (MGAT4C), mRNA [NM_013244]	25834

A_24_P219474	-5.8	-1.3	-1.4	1.1	MGAT5B	Homo sapiens mannosyl (alpha-1,6-)-glycoprotein beta-1,6-N-acetyl-glucosaminyltransferase, isozyme B (MGAT5B), transcript variant 1, mRNA [NM_144677]	146664
A_23_P202170	-6.3	1.0	-1.0	1.1	MGEA5	Homo sapiens meningioma expressed antigen 5 (hyaluronidase) (MGEA5), transcript variant 1, mRNA [NM_012215]	10724
A_24_P226008	1.5	2.3	1.4	1.0	MGLL	Homo sapiens monoglyceride lipase (MGLL), transcript variant 1, mRNA [NM_007283]	11343

A_23_P104323	7.1	1.0	-1.1	1.2	MGMT	Homo sapiens O-6-methylguanine-DNA methyltransferase (MGMT), mRNA [NM_002412]	4255
A_23_P355824	-4.5	1.7	1.6	1.7	MGRN1	Homo sapiens mahogunin, ring finger 1 (MGRN1), transcript variant 1, mRNA [NM_015246]	23295
A_23_P110167	-1.5	2.4	1.1	1.6	MGST2	Homo sapiens microsomal glutathione S-transferase 2 (MGST2), transcript variant 1, mRNA [NM_002413]	4258

A_23_P51548	-1.0	-1.0	-3.0	1.1	MGST3	Homo sapiens microsomal glutathione S-transferase 3 (MGST3), mRNA [NM_004528]	4259
A_23_P205408	-1.3	4.5	4.2	4.4	MIA2	Homo sapiens melanoma inhibitory activity 2 (MIA2), mRNA [NM_054024]	117153
A_23_P117387	-2.5	-2.9	1.0	-2.9	MIA2	Homo sapiens melanoma inhibitory activity 2 (MIA2), mRNA [NM_054024]	117153

A_24_P899928	-2.3	-1.1	-1.1	-1.1	MIAT	Homo sapiens myocardial infarction associated transcript (non-protein coding) (MIAT), transcript variant 1, non-coding RNA [NR_003491]	440823
A_32_P25118	-1.0	-2.1	-1.7	-1.0	MIB1	Homo sapiens mRNA; cDNA DKFZp779F0655 (from clone DKFZp779F0655). [BX648566]	57534
A_24_P259328	2.7	3.0	3.0	-1.0	MIB2	Homo sapiens mindbomb homolog 2 (Drosophila) (MIB2), transcript variant 1, mRNA [NM_080875]	142678

A_23_P257516	-4.5	-7.1	1.1	1.1	MICA	Homo sapiens MHC class I polypeptide-related sequence A (MICA), transcript variant 1 (allele MICA*001), mRNA [NM_000247]	100507436
A_24_P283341	1.0	-3.4	-1.1	1.0	MICAL1	Homo sapiens microtubule associated monoxygenase, calponin and LIM domain containing 1 (MICAL1), transcript variant 1, mRNA [NM_022765]	64780

A_24_P366082	1.0	-5.5	-1.1	1.1	MICAL3	Homo sapiens microtubule associated monoxygenase, calponin and LIM domain containing 3 (MICAL3), transcript variant 1, mRNA [NM_015241]	57553
A_23_P387471	36.6	36.2	34.6	33.7	MICB	Homo sapiens MHC class I polypeptide-related sequence B (MICB), mRNA [NM_005931]	4277
A_32_P74983	9.8	1.3	7.6	4.7	MICB	Homo sapiens MHC class I polypeptide-related sequence B (MICB), mRNA [NM_005931]	4277

A_23_P46907	2.1	1.2	-1.1	-1.0	MICU1	Homo sapiens mitochondrial calcium uptake 1 (MICU1), nuclear gene encoding mitochondrial protein, transcript variant 1, mRNA [NM_006077]	10367
A_23_P170037	3.0	2.7	2.7	1.0	MID1	Homo sapiens midline 1 (Opitz/BBB syndrome) (MID1), transcript variant 3, mRNA [NM_033290]	4281
A_32_P128656	-1.1	1.0	-3.9	1.0	MID1	Homo sapiens midline 1 (Opitz/BBB syndrome) (MID1), transcript variant 1, mRNA [NM_000381]	4281

A_23_P125579	3.0	3.2	3.2	2.5	MID1IP1	Homo sapiens MID1 interacting protein 1 (gastrulation specific G12 homolog (zebrafish)) (MID1IP1), transcript variant 1, mRNA [NM_021242]	58526
A_23_P159956	1.4	-1.0	1.1	-3.3	MID2	Homo sapiens midline 2 (MID2), transcript variant 1, mRNA [NM_012216]	11043
A_23_P324461	1.3	2.1	1.6	1.7	MIDN	Homo sapiens midnolin (MIDN), mRNA [NM_177401]	90007

A_24_P230916	-2.4	-1.1	-3.5	1.7	MIER1	Homo sapiens mesoderm induction early response 1 homolog (Xenopus laevis) (MIER1), transcript variant 4, mRNA [NM_001077702]	57708
A_32_P128572	1.3	-1.2	-2.5	-1.1	MIER1	Homo sapiens mesoderm induction early response 1 homolog (Xenopus laevis) (MIER1), transcript variant 4, mRNA [NM_001077702]	57708
A_23_P119683	-1.5	1.5	2.1	1.0	MIER2	Homo sapiens mesoderm induction early response 1, family member 2 (MIER2), mRNA [NM_017550]	54531

A_24_P941131	-2.7	-1.0	-1.0	1.0	MIER2	Homo sapiens mRNA for KIAA1193 protein, partial cds. [AB033019]	54531
A_23_P164313	2.4	2.2	2.9	2.6	MINK1	Homo sapiens misshapen-like kinase 1 (MINK1), transcript variant 3, mRNA [NM_153827]	50488
A_23_P164307	4.0	2.8	7.8	1.7	MINK1	Homo sapiens misshapen-like kinase 1 (MINK1), transcript variant 3, mRNA [NM_153827]	50488
A_23_P117274	1.1	4.4	2.3	-6.6	MIPEP	Homo sapiens mitochondrial intermediate peptidase (MIPEP), nuclear gene encoding mitochondrial protein, mRNA [NM_005932]	4285

A_24_P886966	2.8	-9.7	2.7	2.5	MIR210HG	Homo sapiens MIR210 host gene (non-protein coding) (MIR210HG), non-coding RNA [NR_038262]	100506211
A_24_P253723	2.4	2.3	-1.3	-1.5	MIR22HG	Homo sapiens MIR22 host gene (non-protein coding) (MIR22HG), transcript variant 1, non-coding RNA [NR_028502]	84981
A_32_P226907	1.5	3.5	1.2	1.6	MIR497HG	Homo sapiens mir-497-195 cluster host gene (non-protein coding) (MIR497HG), non-coding RNA [NR_038310]	100506755

A_23_P216935	-2.8	1.1	1.1	-1.0	MIR600HG	Homo sapiens MIR600 host gene (non-protein coding) (MIR600HG), non-coding RNA [NR_026677]	81571
A_23_P353667	1.0	-2.5	-1.1	-1.1	MIR7-3HG	Homo sapiens MIR7-3 host gene (non-protein coding) (MIR7-3HG), non-coding RNA [NR_027148]	284424
A_32_P330000	1.1	-6.3	-1.0	-1.0	MIRLET7BHG	Homo sapiens MIRLET7B host gene (non-protein coding) (MIRLET7BHG), non-coding RNA [NR_027033]	400931
A_23_P106162	2.2	4.0	3.8	3.9	MIS18BP1	Homo sapiens MIS18 binding protein 1 (MIS18BP1), mRNA [NM_018353]	55320

A_24_P910310	-4.0	1.0	1.0	-1.7	MITF	Homo sapiens microphthalmia-associated transcription factor (MITF), transcript variant 2, mRNA [NM_198177]	4286
A_23_P73345	1.9	3.6	1.7	-31.1	MITF	Homo sapiens microphthalmia-associated transcription factor (MITF), transcript variant 1, mRNA [NM_198159]	4286
A_23_P11739	-3.5	1.1	-1.6	-1.2	MIXL1	Homo sapiens Mix paired-like homeobox (MIXL1), mRNA [NM_031944]	83881

A_23_P50897	5.4	7.1	7.2	7.3	MKI67IP	Homo sapiens MKI67 (FHA domain) interacting nucleolar phosphoprotein (MKI67IP), mRNA [NM_032390]	84365
A_23_P79962	-6.7	-4.1	-1.0	-1.1	MKKS	Homo sapiens McKusick-Kaufman syndrome (MKKS), transcript variant 2, mRNA [NM_170784]	8195
A_23_P200126	1.8	4.0	1.7	1.8	MKNK1	Homo sapiens MAP kinase interacting serine/threonine kinase 1 (MKNK1), transcript variant 1, mRNA [NM_003684]	8569

A_23_P142304	1.6	1.6	-3.9	-1.2	MKNK2	Homo sapiens MAP kinase interacting serine/threonine kinase 2 (MKNK2), transcript variant 1, mRNA [NM_017572]	2872
A_24_P929754	-1.5	-2.4	-1.0	-2.1	MKNK2	Homo sapiens MAP kinase interacting serine/threonine kinase 2 (MKNK2), transcript variant 2, mRNA [NM_199054]	2872
A_24_P410627	1.1	-1.1	-2.7	1.6	MKRN1	Homo sapiens makorin ring finger protein 1 (MKRN1), transcript variant 2, mRNA [NM_001145125]	23608

A_23_P59798	1.7	3.0	1.8	1.0	MKRN1	Homo sapiens makorin ring finger protein 1 (MKRN1), transcript variant 1, mRNA [NM_013446]	23608
A_32_P209624	-1.0	-2.3	-1.1	-2.7	MKRN7P	Homo sapiens makorin ring finger protein 7, pseudogene (MKRN7P), non-coding RNA [NR_026640]	7686
A_23_P75002	3.2	4.4	1.6	1.3	MKX	Homo sapiens mohawk homeobox (MKX), transcript variant 1, mRNA [NM_173576]	283078
A_23_P43350	1.0	-1.0	1.0	3.7	MLANA	Homo sapiens melan-A (MLANA), mRNA [NM_005511]	2315

A_23_P211680	-1.0	-1.0	2.8	-4.8	MLC1	Homo sapiens megalencephalic leukoencephalopathy with subcortical cysts 1 (MLC1), transcript variant 1, mRNA [NM_015166]	23209
A_24_P304449	1.9	1.0	1.9	-2.5	MLEC	Homo sapiens malectin (MLEC), mRNA [NM_014730]	9761
A_23_P13873	1.5	5.6	3.2	2.1	MLF2	Homo sapiens myeloid leukemia factor 2 (MLF2), transcript variant 1, mRNA [NM_005439]	8079

A_24_P196298	-1.3	-1.3	1.1	-4.0	MLL	Homo sapiens myeloid/lymphoid or mixed-lineage leukemia (trithorax homolog, Drosophila) (MLL), transcript variant 2, mRNA [NM_005933]	4297
A_32_P209472	1.6	-9.7	1.4	1.4	MLL3	Homo sapiens myeloid/lymphoid or mixed-lineage leukemia 3 (MLL3), mRNA [NM_170606]	58508
A_23_P142421	2.9	61.0	41.1	5.3	MLL4	Homo sapiens myeloid/lymphoid or mixed-lineage leukemia 4 (MLL4), mRNA [NM_014727]	9757

A_24_P196704	1.6	-2.1	-3.5	-1.9	MLL5	Homo sapiens myeloid/lymphoid or mixed-lineage leukemia 5 (trithorax homolog, Drosophila) (MLL5), transcript variant 1, mRNA [NM_182931]	55904
A_24_P418126	-1.4	-4.0	-3.5	-2.2	MLLT1	Homo sapiens myeloid/lymphoid or mixed-lineage leukemia (trithorax homolog, Drosophila); translocated to, 1 (MLLT1), mRNA [NM_005934]	4298

A_24_P151295	-1.1	-1.1	-1.1	-2.6	MLLT1	Homo sapiens myeloid/lymphoid or mixed-lineage leukemia (trithorax homolog, Drosophila); translocated to, 1 (MLLT1), mRNA [NM_005934]	4298
A_24_P406714	-1.0	-1.8	3.0	1.7	MLLT10	Homo sapiens myeloid/lymphoid or mixed-lineage leukemia (trithorax homolog, Drosophila); translocated to, 10 (MLLT10), transcript variant 1, mRNA [NM_004641]	8028

A_23_P311834	-1.7	-1.6	-2.1	-1.6	MLLT10	Homo sapiens myeloid/lymphoid or mixed-lineage leukemia (trithorax homolog, Drosophila); translocated to, 10 (MLLT10), transcript variant 1, mRNA [NM_004641]	8028
A_24_P84540	-1.0	-1.0	-4.3	1.0	MLLT10	Homo sapiens myeloid/lymphoid or mixed-lineage leukemia (trithorax homolog, Drosophila); translocated to, 10 (MLLT10), transcript variant 1, mRNA [NM_004641]	8028

A_23_P149912	-1.7	-4.6	-2.9	-1.7	MLLT10	Homo sapiens myeloid/lymphoid or mixed-lineage leukemia (trithorax homolog, Drosophila); translocated to, 10 (MLLT10), transcript variant 1, mRNA [NM_004641]	8028
A_23_P216693	2.0	2.4	1.5	1.2	MLLT3	Homo sapiens myeloid/lymphoid or mixed-lineage leukemia (trithorax homolog, Drosophila); translocated to, 3 (MLLT3), mRNA [NM_004529]	4300

A_23_P344694	1.1	1.0	1.0	-3.3	MLLT4	Homo sapiens myeloid/lymphoid or mixed-lineage leukemia (trithorax homolog, Drosophila); translocated to, 4, mRNA (cDNA clone IMAGE:4861456), partial cds. [BC014505]	4301
A_23_P256603	-1.6	-1.0	-2.1	-1.1	MLLT4	Homo sapiens myeloid/lymphoid or mixed-lineage leukemia (trithorax homolog, Drosophila); translocated to, 4 (MLLT4), transcript variant 1, mRNA [NM_001207008]	4301

A_32_P718498	-2.1	3.7	-1.1	3.9	MLLT6	Homo sapiens myeloid/lymphoid or mixed-lineage leukemia (trithorax homolog, Drosophila); translocated to, 6 (MLLT6), mRNA [NM_005937]	4302
A_32_P98683	2.5	3.0	2.8	-1.0	MLLT6	Homo sapiens myeloid/lymphoid or mixed-lineage leukemia (trithorax homolog, Drosophila); translocated to, 6 (MLLT6), mRNA [NM_005937]	4302
A_23_P19523	7.0	2.8	14.6	2.1	MLN	Homo sapiens motilin (MLN), transcript variant 1, mRNA [NM_002418]	4295

A_23_P19529	2.4	3.8	2.3	2.4	MLN	Homo sapiens motilin (MLN), transcript variant 2, mRNA [NM_001040109]	4295
A_23_P36905	-1.0	-2.3	-1.2	-1.5	MLNR	Homo sapiens motilin receptor (MLNR), mRNA [NM_001507]	2862
A_23_P165778	1.2	-2.4	1.2	-2.1	MLPH	Homo sapiens melanophilin (MLPH), transcript variant 1, mRNA [NM_024101]	79083
A_23_P164148	-1.3	1.2	-5.3	-6.8	MLX	Homo sapiens MAX-like protein X (MLX), transcript variant 3, mRNA [NM_170607]	6945
A_24_P415618	-1.0	-1.2	3.9	3.5	MLXIP	Homo sapiens MLX interacting protein (MLXIP), mRNA [NM_014938]	22877

A_24_P415624	3.1	4.0	5.1	1.4	MLXIP	Homo sapiens MLX interacting protein (MLXIP), mRNA [NM_014938]	22877
A_23_P2537	2.7	2.6	2.4	-1.2	MMAB	Homo sapiens methylmalonic aciduria (cobalamin deficiency) cblB type (MMAB), nuclear gene encoding mitochondrial protein, transcript variant 1, mRNA [NM_052845]	326625
A_23_P418282	2.4	2.4	2.3	2.3	MMACHC	Homo sapiens cDNA FLJ25671 fis, clone TST03755. [AK098537]	25974

A_23_P164047	1.6	1.1	2.3	1.6	MMD	Homo sapiens monocyte to macrophage differentiation-associated (MMD), mRNA [NM_012329]	23531
A_23_P212061	1.0	1.0	-1.0	-18.2	MME	Homo sapiens membrane metallo-endopeptidase (MME), transcript variant 2b, mRNA [NM_007289]	4311
A_23_P123448	-3.1	-3.1	-3.1	-3.0	MMP16	matrix metallopeptidase 16 (membrane-inserted) [Source:HGNC Symbol;Acc:7162] [ENST00000286614]	4325

A_23_P136413	-2.1	1.8	-1.2	1.9	MMP17	Homo sapiens matrix metallopeptidase 17 (membrane-inserted) (MMP17), mRNA [NM_016155]	4326
A_24_P184445	3.4	2.0	4.3	1.7	MMP19	Homo sapiens matrix metallopeptidase 19 (MMP19), transcript variant 1, mRNA [NM_002429]	4327
A_23_P203888	-1.5	-8.5	-1.2	1.3	MMP19	Homo sapiens matrix metallopeptidase 19 (MMP19), transcript variant 1, mRNA [NM_002429]	4327
A_23_P376557	1.9	1.4	1.4	-3.7	MMP25	Homo sapiens matrix metallopeptidase 25 (MMP25), mRNA [NM_022468]	64386

A_23_P2052	1.0	-2.3	-1.0	-1.0	MMP26	Homo sapiens matrix metallopeptidase 26 (MMP26), mRNA [NM_021801]	56547
A_23_P26890	-1.2	-1.9	1.2	-2.3	MMP28	Homo sapiens matrix metallopeptidase 28 (MMP28), transcript variant 1, mRNA [NM_024302]	79148
A_23_P161698	-4.6	1.0	-1.0	1.1	MMP3	Homo sapiens matrix metallopeptidase 3 (stromelysin 1, progelatinase) (MMP3), mRNA [NM_002422]	4314
A_24_P369263	-2.1	-1.6	-2.2	-2.0	MMS19	Homo sapiens MMS19 nucleotide excision repair homolog (S. cerevisiae) (MMS19), mRNA [NM_022362]	64210

A_24_P83678	1.3	2.2	1.7	-3.1	MMS22L	Homo sapiens MMS22-like, DNA repair protein (MMS22L), mRNA [NM_198468]	253714
A_32_P137926	-4.3	1.0	1.0	1.1	MMS22L	Homo sapiens MMS22-like, DNA repair protein (MMS22L), mRNA [NM_198468]	253714
A_32_P95914	-1.0	-1.1	-1.1	-3.7	MMS22L	Homo sapiens MMS22-like, DNA repair protein (MMS22L), mRNA [NM_198468]	253714
A_32_P6015	-7.9	1.5	-1.1	-1.3	MNX1	Homo sapiens motor neuron and pancreas homeobox 1 (MNX1), transcript variant 1, mRNA [NM_005515]	3110

A_23_P108922	1.6	2.0	1.8	1.8	MOB1A	MOB1, Mps One Binder kinase activator-like 1B (yeast) [Source:HGNC Symbol;Acc:16015] [ENST00000396049]	55233
A_23_P130919	1.1	-12.5	-1.9	1.8	MOB3A	Homo sapiens MOB kinase activator 3A (MOB3A), mRNA [NM_130807]	126308
A_24_P225339	3.8	3.9	4.4	3.6	MOB3C	Homo sapiens MOB kinase activator 3C (MOB3C), transcript variant 1, mRNA [NM_145279]	148932
A_23_P166	2.3	2.1	1.1	1.8	MOB3C	Homo sapiens MOB kinase activator 3C (MOB3C), transcript variant 1, mRNA [NM_145279]	148932

A_23_P210274	-1.4	-2.5	-3.4	-1.6	MOB4	Homo sapiens MOB family member 4, phocein (MOB4), transcript variant 1, mRNA [NM_015387]	25843
A_23_P67042	1.4	2.0	2.0	1.5	MOCOS	Homo sapiens molybdenum cofactor sulfurase (MOCOS), mRNA [NM_017947]	55034
A_32_P521927	1.6	2.2	2.1	-1.1	MOCS1	Homo sapiens molybdenum cofactor synthesis 1 (MOCS1), transcript variant 1, mRNA [NM_005943]	4337
A_23_P28811	-1.4	-1.7	-22.7	-1.8	MOCS3	Homo sapiens molybdenum cofactor synthesis 3 (MOCS3), mRNA [NM_014484]	27304

A_23_P317796	1.7	1.8	2.0	-4.7	MOGAT3	Homo sapiens monoacylglycerol O-acyltransferase 3 (MOGAT3), mRNA [NM_178176]	346606
A_24_P44596	-4.3	2.7	-1.2	1.7	MON1B	Homo sapiens MON1 homolog B (yeast) (MON1B), mRNA [NM_014940]	22879
A_23_P304000	-2.4	1.0	-1.0	1.0	MON2	Homo sapiens MON2 homolog (S. cerevisiae) (MON2), mRNA [NM_015026]	23041
A_24_P109244	1.0	-26.3	1.0	1.0	MON2	Homo sapiens MON2 homolog (S. cerevisiae) (MON2), mRNA [NM_015026]	23041

A_23_P83683	-1.1	2.6	-1.2	-1.0	MORC1	Homo sapiens MORC family CW-type zinc finger 1 (MORC1), mRNA [NM_014429]	27136
A_23_P155027	-2.7	-3.4	-1.1	-1.0	MORC2	Homo sapiens MORC family CW-type zinc finger 2 (MORC2), mRNA [NM_014941]	22880
A_23_P325501	1.0	-4.2	-1.0	1.1	MORC3	Homo sapiens MORC family CW-type zinc finger 3 (MORC3), mRNA [NM_015358]	23515
A_23_P171117	2.4	-2.0	-3.7	-1.1	MORC4	Homo sapiens MORC family CW-type zinc finger 4 (MORC4), transcript variant 1, mRNA [NM_024657]	79710

A_23_P37579	-2.4	1.1	1.0	1.0	MORF4L1	Homo sapiens mortality factor 4 like 1 (MORF4L1), transcript variant 2, mRNA [NM_206839]	10933
A_24_P333445	1.6	2.7	3.1	3.3	MORF4L2	Homo sapiens mortality factor 4 like 2 (MORF4L2), transcript variant 2, mRNA [NM_012286]	9643
A_32_P69465	-2.8	1.2	1.1	-1.6	MORN2	Homo sapiens MORN repeat containing 2 (MORN2), mRNA [NM_001145450]	729967
A_24_P401491	-1.0	-1.0	-1.1	-3.3	MORN5	Homo sapiens MORN repeat containing 5 (MORN5), mRNA [NM_198469]	254956

A_24_P126093	-1.7	-3.2	-1.7	-3.5	MOSPD2	Homo sapiens motile sperm domain containing 2 (MOSPD2), transcript variant 1, mRNA [NM_152581]	158747
A_23_P71598	1.0	-1.9	-3.2	-1.0	MPDZ	Homo sapiens multiple PDZ domain protein (MPDZ), mRNA [NM_003829]	8777
A_23_P13657	-1.3	-1.5	-2.4	-1.1	MPHOSPH9	Homo sapiens M-phase phosphoprotein 9 (MPHOSPH9), mRNA [NM_022782]	10198

A_23_P141346	1.0	1.0	-13.5	-1.0	MPP3	Homo sapiens membrane protein, palmitoylated 3 (MAGUK p55 subfamily member 3) (MPP3), transcript variant 1, mRNA [NM_001932]	4356
A_24_P347458	1.0	-1.0	-4.9	-1.0	MPP5	Homo sapiens membrane protein, palmitoylated 5 (MAGUK p55 subfamily member 5) (MPP5), mRNA [NM_022474]	64398
A_23_P71053	-1.2	-1.5	-2.1	-1.7	MPP6	Homo sapiens membrane protein, palmitoylated 6 (MAGUK p55 subfamily member 6) (MPP6), mRNA [NM_016447]	51678

A_23_P27285	-1.0	-3.7	-1.1	-1.0	MPPE1	Homo sapiens metallophosphoesterase 1 (MPPE1), transcript variant 1, mRNA [NM_023075]	65258
A_23_P165130	1.4	2.0	2.6	1.1	MPV17L2	Homo sapiens MPV17 mitochondrial membrane protein-like 2 (MPV17L2), nuclear gene encoding mitochondrial protein, mRNA [NM_032683]	84769
A_23_P138089	-8.9	-1.0	-1.1	-1.0	MPZ	Homo sapiens myelin protein zero (MPZ), mRNA [NM_000530]	4359

A_23_P476	-1.9	-1.0	-2.1	-1.6	MPZL1	Homo sapiens myelin protein zero-like 1 (MPZL1), transcript variant 1, mRNA [NM_003953]	9019
A_24_P242391	-1.1	-3.2	-2.1	-1.9	MPZL1	Homo sapiens myelin protein zero-like 1 (MPZL1), transcript variant 1, mRNA [NM_003953]	9019
A_23_P11874	-1.8	1.0	-1.9	-3.3	MPZL1	Homo sapiens myelin protein zero-like 1 (MPZL1), transcript variant 1, mRNA [NM_003953]	9019
A_23_P150379	1.2	1.7	-2.2	-1.0	MPZL2	Homo sapiens myelin protein zero-like 2 (MPZL2), transcript variant 2, mRNA [NM_144765]	10205

A_23_P381172	-1.0	-3.2	-1.0	-1.1	MRAP	Homo sapiens melanocortin 2 receptor accessory protein (MRAP), transcript variant 2, mRNA [NM_206898]	56246
A_23_P49627	-2.9	-1.1	-1.2	-1.1	MRC2	Homo sapiens mannose receptor, C type 2 (MRC2), mRNA [NM_006039]	9902
A_24_P312671	2.1	2.0	1.8	1.3	MRE11A	Homo sapiens MRE11 meiotic recombination 11 homolog A (S. cerevisiae) (MRE11A), transcript variant 2, mRNA [NM_005590]	4361

A_23_P150189	-1.0	-5.5	-1.0	-1.0	MRE11A	Homo sapiens MRE11 meiotic recombination 11 homolog A (S. cerevisiae) (MRE11A), transcript variant 2, mRNA [NM_005590]	4361
A_23_P108948	-1.0	-3.7	-1.0	-1.0	MREG	Homo sapiens melanoregulin (MREG), mRNA [NM_018000]	55686
A_24_P11965	-2.9	1.7	-2.2	-1.3	MRFAP1	Homo sapiens Morf4 family associated protein 1 (MRFAP1), mRNA [NM_033296]	93621
A_23_P133058	1.1	1.0	-1.0	-4.4	MRFAP1L1	Homo sapiens Morf4 family associated protein 1-like 1 (MRFAP1L1), mRNA [NM_203462]	114932

A_23_P425826	-4.8	1.6	1.1	-2.9	MRFAP1L1	Homo sapiens Morf4 family associated protein 1-like 1 (MRFAP1L1), mRNA [NM_203462]	114932
A_23_P161983	1.5	2.3	-1.9	1.0	MRGPRX4	Homo sapiens MAS-related GPR, member X4 (MRGPRX4), mRNA [NM_054032]	117196
A_24_P279797	-3.6	1.1	1.0	1.1	MRI1	Homo sapiens methylthioribose-1-phosphate isomerase homolog (S. cerevisiae) (MRI1), transcript variant 1, mRNA [NM_001031727]	84245

A_23_P15603	-2.8	1.6	1.0	-1.0	MRM1	Homo sapiens mitochondrial rRNA methyltransferase 1 homolog (S. cerevisiae) (MRM1), nuclear gene encoding mitochondrial protein, mRNA [NM_024864]	79922
A_23_P4545	-1.4	2.4	1.3	-2.1	MRO	Homo sapiens maestro (MRO), transcript variant 1, mRNA [NM_031939]	83876
A_32_P49309	1.0	-1.3	-2.3	1.0	MRO	Homo sapiens maestro (MRO), transcript variant 1, mRNA [NM_031939]	83876

A_24_P32672	-1.9	-3.1	-1.4	-3.8	MRPL11	Homo sapiens mitochondrial ribosomal protein L11 (MRPL11), nuclear gene encoding mitochondrial protein, transcript variant 1, mRNA [NM_016050]	65003
A_23_P170352	2.6	2.9	1.7	-1.0	MRPL12	Homo sapiens mitochondrial ribosomal protein L12 (MRPL12), nuclear gene encoding mitochondrial protein, mRNA [NM_002949]	6182

A_23_P47497	4.3	3.4	2.9	3.9	MRPL16	Homo sapiens mitochondrial ribosomal protein L16 (MRPL16), nuclear gene encoding mitochondrial protein, mRNA [NM_017840]	54948
A_23_P102262	-48.0	1.1	1.2	1.1	MRPL19	Homo sapiens mitochondrial ribosomal protein L19 (MRPL19), nuclear gene encoding mitochondrial protein, mRNA [NM_014763]	9801

A_24_P17909	1.8	1.8	2.3	1.0	MRPL2	Homo sapiens mitochondrial ribosomal protein L2 (MRPL2), nuclear gene encoding mitochondrial protein, mRNA [NM_015950]	51069
A_23_P115955	-1.4	-4.3	-4.0	1.4	MRPL21	Homo sapiens mitochondrial ribosomal protein L21 (MRPL21), nuclear gene encoding mitochondrial protein, transcript variant 1, mRNA [NM_181515]	219927

A_23_P137848	1.4	-2.3	2.0	2.1	MRPL24	Homo sapiens mitochondrial ribosomal protein L24 (MRPL24), nuclear gene encoding mitochondrial protein, transcript variant 1, mRNA [NM_145729]	79590
A_23_P218619	3.1	3.1	2.0	1.4	MRPL30	Homo sapiens mitochondrial ribosomal protein L30 (MRPL30), nuclear gene encoding mitochondrial protein, transcript variant 1, mRNA [NM_145212]	51263

A_23_P111753	-3.9	1.0	1.1	1.0	MRPL32	Homo sapiens mitochondrial ribosomal protein L32 (MRPL32), nuclear gene encoding mitochondrial protein, mRNA [NM_031903]	64983
A_23_P165651	1.1	1.0	1.0	-2.3	MRPL35	Homo sapiens mitochondrial ribosomal protein L35 (MRPL35), nuclear gene encoding mitochondrial protein, transcript variant 2, mRNA [NM_145644]	51318

A_24_P97221	-1.9	-16.0	-1.0	1.1	MRPL35	Homo sapiens mitochondrial ribosomal protein L35 (MRPL35), nuclear gene encoding mitochondrial protein, transcript variant 1, mRNA [NM_016622]	51318
A_32_P16489	1.9	2.0	-3.2	1.0	MRPL35	Homo sapiens mitochondrial ribosomal protein L35 (MRPL35), nuclear gene encoding mitochondrial protein, transcript variant 2, mRNA [NM_145644]	51318

A_24_P371758	1.0	-7.9	-1.0	-1.0	MRPL36	Homo sapiens mitochondrial ribosomal protein L36 (MRPL36), nuclear gene encoding mitochondrial protein, mRNA [NM_032479]	64979
A_23_P164702	1.3	1.3	1.1	2.5	MRPL4	Homo sapiens mitochondrial ribosomal protein L4 (MRPL4), nuclear gene encoding mitochondrial protein, transcript variant 1, mRNA [NM_015956]	51073

A_24_P228667	3.5	3.3	3.1	3.3	MRPL40	Homo sapiens mitochondrial ribosomal protein L40 (MRPL40), nuclear gene encoding mitochondrial protein, mRNA [NM_003776]	64976
A_32_P115130	-2.1	-2.1	-2.3	-2.2	MRPL41	Homo sapiens mitochondrial ribosomal protein L41 (MRPL41), nuclear gene encoding mitochondrial protein, mRNA [NM_032477]	64975

A_24_P352445	-1.5	-1.5	-2.5	-1.7	MRPL42	Homo sapiens mitochondrial ribosomal protein L42 (MRPL42), nuclear gene encoding mitochondrial protein, transcript variant 2, mRNA [NM_172177]	28977
A_23_P398491	3.4	3.4	1.7	3.0	MRPL42P5	Homo sapiens mitochondrial ribosomal protein L42 pseudogene 5 (MRPL42P5), non-coding RNA [NR_002208]	359821

A_23_P346384	-2.5	1.1	1.2	1.0	MRPL43	Homo sapiens mitochondrial ribosomal protein L43 (MRPL43), nuclear gene encoding mitochondrial protein, transcript variant 2, mRNA [NM_176792]	84545
A_23_P90589	1.1	1.0	-11.4	-1.0	MRPL44	Homo sapiens mitochondrial ribosomal protein L44 (MRPL44), nuclear gene encoding mitochondrial protein, mRNA [NM_022915]	65080

A_32_P9575	-1.0	-1.7	-3.1	1.0	MRPL45	Homo sapiens mitochondrial ribosomal protein L45 (MRPL45), nuclear gene encoding mitochondrial protein, mRNA [NM_032351]	84311
A_32_P209989	-4.6	-1.1	-1.1	-1.1	MRPL46	Homo sapiens mitochondrial ribosomal protein L46 (MRPL46), nuclear gene encoding mitochondrial protein, mRNA [NM_022163]	26589

A_24_P409330	1.1	1.9	-1.1	-3.1	MRPL52	Homo sapiens mitochondrial ribosomal protein L52 (MRPL52), nuclear gene encoding mitochondrial protein, transcript variant 4, mRNA [NM_181304]	122704
A_24_P371194	1.9	2.3	1.6	1.5	MRPL53	Homo sapiens mitochondrial ribosomal protein L53 (MRPL53), nuclear gene encoding mitochondrial protein, mRNA [NM_053050]	116540

A_23_P101742	1.1	-3.0	-3.4	-1.0	MRPL54	Homo sapiens mitochondrial ribosomal protein L54 (MRPL54), nuclear gene encoding mitochondrial protein, mRNA [NM_172251]	116541
A_24_P49539	-1.2	-2.4	-1.2	-3.2	MRPL55	Homo sapiens mitochondrial ribosomal protein L55 (MRPL55), nuclear gene encoding mitochondrial protein, transcript variant 4, mRNA [NM_181462]	128308

A_24_P283294	7.5	5.1	11.1	10.0	MRPS10	Homo sapiens mitochondrial ribosomal protein S10 (MRPS10), nuclear gene encoding mitochondrial protein, mRNA [NM_018141]	55173
A_24_P913666	1.1	-4.1	1.1	1.1	MRPS11	Homo sapiens mitochondrial ribosomal protein S11, mRNA (cDNA clone IMAGE:3352526), with apparent retained intron. [BC000200]	64963

A_23_P48977	3.6	2.3	4.9	-1.2	MRPS11	Homo sapiens mitochondrial ribosomal protein S11 (MRPS11), nuclear gene encoding mitochondrial protein, transcript variant 1, mRNA [NM_022839]	64963
A_24_P935318	1.7	1.5	-2.0	-2.2	MRPS11	Homo sapiens mitochondrial ribosomal protein S11 (MRPS11), nuclear gene encoding mitochondrial protein, transcript variant 1, mRNA [NM_022839]	64963

A_23_P50807	-1.6	-2.1	-3.2	-3.6	MRPS12	Homo sapiens mitochondrial ribosomal protein S12 (MRPS12), nuclear gene encoding mitochondrial protein, transcript variant 1, mRNA [NM_021107]	6183
A_23_P62731	-1.7	-1.8	-2.0	-2.8	MRPS14	Homo sapiens mitochondrial ribosomal protein S14 (MRPS14), nuclear gene encoding mitochondrial protein, transcript variant 1, mRNA [NM_022100]	63931

A_23_P114826	3.8	1.2	-1.7	-1.1	MRPS15	Homo sapiens mitochondrial ribosomal protein S15 (MRPS15), nuclear gene encoding mitochondrial protein, mRNA [NM_031280]	64960
A_23_P122387	-16.8	1.0	-7.2	1.0	MRPS18A	Homo sapiens mitochondrial ribosomal protein S18A (MRPS18A), nuclear gene encoding mitochondrial protein, transcript variant 1, mRNA [NM_018135]	55168

A_23_P135104	1.7	1.7	2.4	1.0	MRPS2	Homo sapiens mitochondrial ribosomal protein S2 (MRPS2), nuclear gene encoding mitochondrial protein, mRNA [NM_016034]	51116
A_23_P86182	-2.3	1.3	1.1	1.1	MRPS21	Homo sapiens mitochondrial ribosomal protein S21 (MRPS21), nuclear gene encoding mitochondrial protein, transcript variant 1, mRNA [NM_031901]	54460

A_24_P139816	-3.0	-2.0	-3.3	1.0	MRPS21	Homo sapiens mitochondrial ribosomal protein S21 (MRPS21), nuclear gene encoding mitochondrial protein, transcript variant 1, mRNA [NM_031901]	54460
A_23_P72138	2.4	-1.0	1.6	-2.9	MRPS22	Homo sapiens mitochondrial ribosomal protein S22 (MRPS22), nuclear gene encoding mitochondrial protein, mRNA [NM_020191]	56945

A_23_P252536	1.4	1.3	3.0	1.7	MRPS26	Homo sapiens mitochondrial ribosomal protein S26 (MRPS26), nuclear gene encoding mitochondrial protein, mRNA [NM_030811]	64949
A_24_P187859	1.1	1.1	2.1	1.1	MRPS27	Homo sapiens cDNA FLJ38645 fis, clone HHDPC2005794. [AK095964]	23107
A_23_P252362	1.0	-3.9	-1.0	-1.0	MRPS30	Homo sapiens mitochondrial ribosomal protein S30 (MRPS30), nuclear gene encoding mitochondrial protein, mRNA [NM_016640]	10884

A_23_P29575	1.0	1.0	3.0	-1.0	MRPS36	Homo sapiens mitochondrial ribosomal protein S36 (MRPS36), nuclear gene encoding mitochondrial protein, mRNA [NM_033281]	92259
A_24_P365571	-1.1	1.5	1.3	-32.5	MRPS6	Homo sapiens mitochondrial ribosomal protein S6 (MRPS6), nuclear gene encoding mitochondrial protein, mRNA [NM_032476]	64968

A_24_P175427	-1.1	1.1	-4.0	1.1	MRRF	Homo sapiens mitochondrial ribosome recycling factor (MRRF), nuclear gene encoding mitochondrial protein, transcript variant 2, mRNA [NM_199177]	92399
A_23_P111373	-1.0	-3.3	1.0	-1.0	MRS2	Homo sapiens MRS2 magnesium homeostasis factor homolog (S. cerevisiae) (MRS2), mRNA [NM_020662]	57380
A_23_P35099	3.2	-1.0	1.1	1.4	MRTO4	Homo sapiens mRNA turnover 4 homolog (S. cerevisiae) (MRTO4), mRNA [NM_016183]	51154

A_23_P396299	-1.6	-2.4	-1.9	-1.8	MRVI1-AS1	Homo sapiens uncharacterized LOC100129827 (LOC100129827), non-coding RNA [NR_034094]	100129827
A_23_P98565	-1.3	-1.2	-2.0	-2.1	MS4A14	Homo sapiens membrane-spanning 4-domains, subfamily A, member 14 (MS4A14), transcript variant 1, mRNA [NM_032597]	84689
A_24_P30389	-2.3	-8.1	-1.2	-1.2	MS4A15	Homo sapiens membrane-spanning 4-domains, subfamily A, member 15 (MS4A15), transcript variant 2, mRNA [NM_152717]	219995

A_23_P1904	-3.1	-1.0	-5.1	1.8	MS4A2	Homo sapiens membrane-spanning 4-domains, subfamily A, member 2 (Fc fragment of IgE, high affinity I, receptor for; beta polypeptide) (MS4A2), mRNA [NM_000139]	2206
A_23_P75769	1.0	-2.6	-1.0	-3.6	MS4A4A	Homo sapiens membrane-spanning 4-domains, subfamily A, member 4 (MS4A4A), transcript variant 2, mRNA [NM_024021]	51338

A_23_P416238	-2.6	-1.2	-2.1	-3.1	MS4A6E	Homo sapiens membrane-spanning 4-domains, subfamily A, member 6E (MS4A6E), mRNA [NM_139249]	245802
A_23_P139146	1.0	-24.8	1.0	-1.0	MS4A8B	Homo sapiens membrane-spanning 4-domains, subfamily A, member 8B (MS4A8B), mRNA [NM_031457]	83661
A_23_P122001	2.1	-1.1	-1.3	-1.3	MSH3	Homo sapiens mutS homolog 3 (E. coli) (MSH3), mRNA [NM_002439]	4437
A_23_P102202	1.3	-6.2	-1.0	1.1	MSH6	Homo sapiens mutS homolog 6 (E. coli) (MSH6), mRNA [NM_000179]	2956

A_23_P139795	-1.5	1.5	4.0	-1.0	MSI1	Homo sapiens musashi homolog 1 (Drosophila) (MSI1), mRNA [NM_002442]	4440
A_23_P369479	1.1	-14.8	1.2	1.1	MSI2	Homo sapiens musashi homolog 2 (Drosophila) (MSI2), transcript variant 2, mRNA [NM_170721]	124540
A_23_P44195	-1.0	-3.7	-1.0	-1.0	MSI2	Homo sapiens musashi homolog 2 (Drosophila) (MSI2), transcript variant 1, mRNA [NM_138962]	124540
A_23_P132438	-1.0	-1.8	-2.8	-1.0	MSL2	Homo sapiens male-specific lethal 2 homolog (Drosophila) (MSL2), transcript variant 1, mRNA [NM_018133]	55167

A_23_P353514	-3.9	-1.0	-1.1	-1.1	MSL3	Homo sapiens male-specific lethal 3 homolog (Drosophila) (MSL3), transcript variant 4, mRNA [NM_078628]	10943
A_32_P235159	7.3	4.6	6.5	6.5	MSL3P1	Homo sapiens male-specific lethal 3 homolog (Drosophila) pseudogene 1 (MSL3P1), non-coding RNA [NR_024322]	151507
A_23_P333934	-2.5	-1.1	-1.1	1.2	MSLNL	Homo sapiens mesothelin-like (MSLNL), mRNA [NM_001025190]	401827
A_23_P73593	-2.5	1.0	1.0	1.0	MSN	Homo sapiens moesin (MSN), mRNA [NM_002444]	4478

A_23_P73589	-1.2	-1.2	-2.9	-1.2	MSN	Homo sapiens moesin (MSN), mRNA [NM_002444]	4478
A_23_P392942	2.2	1.8	1.1	4.0	MSR1	Homo sapiens macrophage scavenger receptor 1 (MSR1), transcript variant SR-AII, mRNA [NM_002445]	4481
A_32_P138359	9.8	3.8	3.6	2.1	MSRA	Homo sapiens methionine sulfoxide reductase A (MSRA), transcript variant 1, mRNA [NM_012331]	4482

A_32_P122373	1.1	1.1	1.0	-4.0	MST1P2	Homo sapiens macrophage stimulating 1 (hepatocyte growth factor-like) pseudogene 2 (MST1P2), non-coding RNA [NR_027504]	11209
A_23_P340376	-7.1	1.2	1.2	1.2	MST1P9	Homo sapiens macrophage stimulating 1 (hepatocyte growth factor-like) pseudogene 9 (MST1P9), non-coding RNA [NR_002729]	11223
A_23_P110430	-1.0	-1.0	-1.0	-5.2	MSX1	Homo sapiens msh homeobox 1 (MSX1), mRNA [NM_002448]	4487

A_23_P37983	4.1	1.3	3.0	1.5	MT1B	Homo sapiens metallothionein 1B (MT1B), mRNA [NM_005947]	4490
A_23_P206724	1.9	-2.7	-1.2	1.5	MT1E	Homo sapiens metallothionein 1E (MT1E), mRNA [NM_175617]	4493
A_23_P60933	-2.9	-1.0	-1.3	-1.2	MT1G	Homo sapiens metallothionein 1G (MT1G), mRNA [NM_005950]	4495
A_23_P414343	-2.8	-2.8	-2.1	-1.1	MT1H	Homo sapiens metallothionein 1H (MT1H), mRNA [NM_005951]	4496
A_24_P74828	5.0	4.7	4.4	4.7	MT1JP	Homo sapiens metallothionein 1J, pseudogene (MT1JP), non-coding RNA [NR_036677]	4498

A_23_P427703	-1.1	-1.0	-4.1	-1.8	MT1L	Homo sapiens metallothionein 1L (gene/pseudogene) (MT1L), non-coding RNA [NR_001447]	4500
A_23_P206718	-1.5	-1.0	-1.5	-2.7	MT1X	metallothionein 1X [Source:HGNC Symbol;Acc:7405] [ENST00000394485]	4501
A_23_P303242	-1.8	-1.4	-2.1	1.4	MT1X	Homo sapiens metallothionein 1X (MT1X), mRNA [NM_005952]	4501
A_24_P125096	1.1	1.4	1.1	2.2	MT1X	Homo sapiens metallothionein 1X (MT1X), mRNA [NM_005952]	4501
A_23_P252413	2.2	2.1	2.0	2.0	MT2A	Homo sapiens metallothionein 2A (MT2A), mRNA [NM_005953]	4502

A_23_P152398	-1.1	-10.5	-1.2	-1.2	MT4	Homo sapiens metallothionein 4 (MT4), mRNA [NM_032935]	84560
A_23_P9513	-1.8	-1.8	-7.0	-2.0	MTA1	Homo sapiens metastasis associated 1 (MTA1), transcript variant 1, mRNA [NM_004689]	9112
A_24_P241373	1.6	3.0	-1.3	-1.0	MTA1	Homo sapiens metastasis associated 1 (MTA1), transcript variant 1, mRNA [NM_004689]	9112
A_23_P203420	-4.5	-2.7	-1.2	-6.0	MTA2	Homo sapiens metastasis associated 1 family, member 2 (MTA2), mRNA [NM_004739]	9219

A_23_P411431	2.0	1.1	1.9	2.1	MTA3	Homo sapiens cDNA FLJ45312 fis, clone BRHIP3005037, moderately similar to Metastasis-associated protein MTA1. [AK127245]	57504
A_24_P297888	2.4	1.6	2.6	1.9	MTAP	Homo sapiens methylthioadenosine phosphorylase (MTAP), mRNA [NM_002451]	4507
A_23_P21278	-1.8	-41.8	-1.0	-3.1	MTBP	Mdm2, transformed 3T3 cell double minute 2, p53 binding protein (mouse) binding protein, 104kDa [Source:HGNC Symbol;Acc:7417] [ENST00000522308]	27085

A_24_P235012	3.1	6.5	6.8	1.3	MTCH1	Homo sapiens mitochondrial carrier 1 (MTCH1), nuclear gene encoding mitochondrial protein, mRNA [NM_014341]	23787
A_24_P934563	1.9	-1.4	-1.3	-2.3	MTCH2	Homo sapiens mitochondrial carrier 2 (MTCH2), nuclear gene encoding mitochondrial protein, mRNA [NM_014342]	23788
A_23_P43071	1.1	1.1	-3.1	1.0	MTERFD1	Homo sapiens MTERF domain containing 1 (MTERFD1), nuclear gene encoding mitochondrial protein, mRNA [NM_015942]	51001

A_24_P186124	-1.0	-38.5	-1.1	-1.0	MTERFD2	Homo sapiens MTERF domain containing 2 (MTERFD2), transcript variant 1, mRNA [NM_182501]	130916
A_24_P737451	-2.0	-2.1	-2.5	-2.2	MTERFD2	Homo sapiens MTERF domain containing 2 (MTERFD2), transcript variant 2, non-coding RNA [NR_028049]	130916
A_23_P53530	1.1	1.1	-1.1	-4.1	MTERFD3	Homo sapiens MTERF domain containing 3 (MTERFD3), transcript variant 1, mRNA [NM_001033050]	80298

A_23_P46333	2.3	2.5	1.8	1.7	MTF2	Homo sapiens metal response element binding transcription factor 2 (MTF2), transcript variant 1, mRNA [NM_007358]	22823
A_23_P29204	-5.3	-3.7	-1.3	-1.2	MTFP1	Homo sapiens mitochondrial fission process 1 (MTFP1), nuclear gene encoding mitochondrial protein, transcript variant 1, mRNA [NM_016498]	51537

A_23_P216215	-2.0	2.3	2.0	1.6	MTFR1	Homo sapiens mitochondrial fission regulator 1 (MTFR1), nuclear gene encoding mitochondrial protein, transcript variant 1, mRNA [NM_014637]	9650
A_23_P117494	1.0	6.8	1.4	5.4	MTHFD1	Homo sapiens methylenetetrahydrofolate dehydrogenase (NADP+ dependent) 1, methenyltetrahydrofolate cyclohydrolase, formyltetrahydrofolate synthetase (MTHFD1), mRNA [NM_005956]	4522

A_23_P214908	-4.3	-1.2	-1.3	-1.4	MTHFD1L	Homo sapiens methylene tetrahydro folate dehydroge nase (NADP+ dependen t) 1-like (MTHFD1L), nuclear gene encoding mitochond rial protein, transcript variant 4, mRNA [NM_0012 42769]	25902
A_23_P214907	-1.0	-1.0	1.3	-2.0	MTHFD1L	Homo sapiens methylene tetrahydro folate dehydroge nase (NADP+ dependen t) 1-like (MTHFD1L), nuclear gene encoding mitochond rial protein, transcript variant 2, mRNA [NM_0154 40]	25902

A_23_P112846	1.3	-1.0	-2.4	-1.0	MTHFD2L	Homo sapiens methylene tetrahydro folate dehydroge nase (NADP+ dependen t) 2-like (MTHFD2L), mRNA [NM_0011 44978]	441024
A_24_P307314	1.4	-1.7	1.2	-6.3	MTHFD2L	methylene tetrahydro folate dehydroge nase (NADP+ dependen t) 2-like [Source:H GNC Symbol;Ac c:31865] [ENST0000 0423607]	441024
A_24_P911255	-15.3	-7.2	-1.7	-1.7	MTHFR	methylene tetrahydro folate reductase (NAD(P)H) [Source:H GNC Symbol;Ac c:7436] [ENST0000 0376585]	4524

A_23_P400081	-2.1	-1.0	-1.1	1.0	MTHFR	Homo sapiens methylenetetrahydrofolate reductase (NAD(P)H) (MTHFR), mRNA [NM_005957]	4524
A_23_P163380	-8.5	1.1	-1.0	1.0	MTHFS	Homo sapiens 5,10-methenyltetrahydrofolate synthetase (5-formyltetrahydrofolate cyclo-ligase) (MTHFS), transcript variant 1, mRNA [NM_006441]	10588
A_24_P290874	2.6	-1.3	1.5	1.2	MTMR1	Homo sapiens myotubularin related protein 1 (MTMR1), mRNA [NM_003828]	8776

A_23_P126766	1.6	2.7	-1.2	1.3	MTMR11	Homo sapiens myotubularin related protein 11 (MTMR11), transcript variant 1, mRNA [NM_001145862]	10903
A_24_P354615	-1.0	-2.9	-1.1	-1.1	MTMR12	Homo sapiens myotubularin related protein 12 (MTMR12), mRNA [NM_001040446]	54545
A_24_P406830	1.7	1.3	2.4	-1.3	MTMR2	Homo sapiens myotubularin related protein 2 (MTMR2), transcript variant 2, mRNA [NM_201278]	8898
A_23_P25515	1.4	-2.1	-1.3	1.3	MTMR6	Homo sapiens myotubularin related protein 6 (MTMR6), mRNA [NM_004685]	9107

A_24_P419211	5.1	2.9	1.2	-1.1	MTMR6	Homo sapiens myotubularin related protein 6 (MTMR6), mRNA [NM_004685]	9107
A_24_P383095	-2.5	-1.0	-1.1	-1.2	MTMR7	Homo sapiens myotubularin related protein 7 (MTMR7), mRNA [NM_004686]	9108
A_23_P84995	2.3	4.1	4.6	3.5	MTMR8	Homo sapiens myotubularin related protein 8 (MTMR8), mRNA [NM_017677]	55613
A_23_P71389	-1.7	-3.6	-1.2	-1.6	MTMR9	Homo sapiens myotubularin related protein 9 (MTMR9), mRNA [NM_015458]	66036

A_23_P200930	-1.1	-1.5	1.2	-2.3	MTR	Homo sapiens 5-methyltetrahydrofolate-homocysteine methyltransferase (MTR), mRNA [NM_000254]	4548
A_24_P67806	2.0	2.2	-1.0	1.8	MTR	Homo sapiens 5-methyltetrahydrofolate-homocysteine methyltransferase (MTR), mRNA [NM_000254]	4548
A_23_P200936	-1.1	1.0	-1.8	4.1	MTR	Homo sapiens 5-methyltetrahydrofolate-homocysteine methyltransferase (MTR), mRNA [NM_000254]	4548

A_24_P349821	-1.0	-9.6	-1.2	-1.1	MTRF1	Homo sapiens mitochondrial translational release factor 1 (MTRF1), nuclear gene encoding mitochondrial protein, mRNA [NM_004294]	9617
A_23_P37005	2.1	3.6	2.9	1.1	MTRF1	Homo sapiens mitochondrial translational release factor 1 (MTRF1), nuclear gene encoding mitochondrial protein, mRNA [NM_004294]	9617

A_32_P82560	2.1	2.3	-1.2	1.3	MTRF1L	Homo sapiens mitochondrial translational release factor 1-like (MTRF1L), nuclear gene encoding mitochondrial protein, transcript variant 1, mRNA [NM_019041]	54516
A_23_P334308	-7.2	1.9	-2.9	-3.8	MTUS2	Homo sapiens microtubule associated tumor suppressor candidate 2 (MTUS2), transcript variant 1, mRNA [NM_001033602]	23281

A_23_P120335	2.0	2.7	1.3	-1.4	MTX2	Homo sapiens metaxin 2 (MTX2), nuclear gene encoding mitochondrial protein, transcript variant 1, mRNA [NM_006554]	10651
A_23_P137856	-3.9	-1.2	-1.3	1.0	MUC1	Homo sapiens mucin 1, cell surface associated (MUC1), transcript variant 1, mRNA [NM_002456]	4582
A_24_P798028	1.1	1.5	-1.6	-2.8	MUC12	Homo sapiens mucin 12, cell surface associated (MUC12), mRNA [NM_001164462]	10071

A_24_P658413	-1.6	-4.8	-1.6	-1.6	MUC12	Homo sapiens mucin 12, cell surface associated (MUC12), mRNA [NM_001164462]	10071
A_23_P5211	-1.2	-4.0	-1.4	-1.9	MUC16	Homo sapiens mucin 16, cell surface associated (MUC16), mRNA [NM_024690]	94025
A_24_P247643	-1.1	-1.1	-10.9	-1.8	MUC17	Homo sapiens mucin 17, cell surface associated (MUC17), mRNA [NM_001040105]	140453
A_24_P169507	2.6	1.5	23.6	3.9	MUC17	Homo sapiens mucin 17, cell surface associated (MUC17), mRNA [NM_001040105]	140453

A_24_P358875	-3.8	1.0	-1.0	-1.0	MUC17	Homo sapiens mucin 17, cell surface associated (MUC17), mRNA [NM_001040105]	140453
A_23_P306610	-1.1	-1.2	-1.1	-2.3	MUC17	Homo sapiens mucin 17, cell surface associated (MUC17), mRNA [NM_001040105]	140453
A_23_P256784	-28.9	-1.0	-2.0	-1.7	MUC2	Homo sapiens mucin 2, oligomeric mucus/gel-forming (MUC2), mRNA [NM_002457]	4583
A_24_P341761	-1.7	-2.3	-3.2	-2.0	MUC2	Homo sapiens mucin 2, oligomeric mucus/gel-forming (MUC2), mRNA [NM_002457]	4583

A_23_P92222	-2.9	2.4	2.5	-1.4	MUC20	Homo sapiens mucin 20, cell surface associated (MUC20), transcript variant L, mRNA [NM_152673]	200958
A_24_P264507	-2.6	1.5	-2.6	-3.9	MUC22	Homo sapiens mucin 22 (MUC22), mRNA [NM_001198815]	100507679
A_24_P630109	1.2	-2.1	1.3	-1.4	MUC3	Homo sapiens SIB 297 intestinal mucin (MUC3) mRNA, partial cds. [AF007192]	100133790
A_24_P913828	-1.3	-1.5	-2.3	-3.1	MUC3A	Homo sapiens mucin (MUC-3) mRNA, partial cds. [M55405]	4584

A_24_P239176	2.0	2.2	1.9	-1.3	MUC4	Homo sapiens mucin 4, cell surface associated (MUC4), transcript variant 1, mRNA [NM_018406]	4585
A_24_P239183	-1.5	1.3	-2.3	-1.1	MUC4	Homo sapiens mucin 4, cell surface associated (MUC4), transcript variant 1, mRNA [NM_018406]	4585
A_24_P239177	-1.6	-2.4	-2.3	-2.1	MUC4	Homo sapiens mucin 4, cell surface associated (MUC4), transcript variant 1, mRNA [NM_018406]	4585
A_24_P208825	3.5	2.1	3.1	5.6	MUC4	Homo sapiens mucin 4, cell surface associated (MUC4), transcript variant 1, mRNA [NM_018406]	4585

A_24_P935054	-2.5	1.7	-3.3	1.7	MUC5AC	PREDICTED: Homo sapiens mucin 5AC, oligomeric mucus/gel-forming (MUC5AC), mRNA [XM_003119481]	4586
A_24_P917886	2.9	1.8	-1.0	1.6	MUC5AC	PREDICTED: Homo sapiens mucin 5AC, oligomeric mucus/gel-forming (MUC5AC), partial mRNA [XM_003403637]	4586
A_24_P548274	-1.9	4.1	4.6	1.1	MUC5AC	H.sapiens (MAR11) MUC5AC mRNA for mucin (partial). [Z34282]	4586
A_23_P305863	-3.9	-1.5	-2.7	-1.4	MUC5B	Homo sapiens mucin 5B, oligomeric mucus/gel-forming (MUC5B), mRNA [NM_002458]	727897

A_24_P102650	1.3	-3.3	-2.2	1.2	MUC5B	Homo sapiens mucin 5B, oligomeric mucus/gel-forming (MUC5B), mRNA [NM_002458]	727897
A_24_P916789	1.2	2.4	2.9	2.3	MUC5B	Homo sapiens mucin 5B, oligomeric mucus/gel-forming (MUC5B), mRNA [NM_002458]	727897
A_23_P150979	-2.3	1.0	-1.0	-1.0	MUCL1	Homo sapiens mucin-like 1 (MUCL1), mRNA [NM_058173]	118430
A_23_P37265	1.3	1.2	-2.2	-2.4	MUDENG	Homo sapiens MU-2/AP1M2 domain containing, death-inducing (MUDENG), transcript variant 1, mRNA [NM_018229]	55745

A_23_P208961	-1.0	-5.4	-1.0	-1.0	MUM1	Homo sapiens melanoma associated antigen (mutated) 1 (MUM1), transcript variant 1, mRNA [NM_032853]	84939
A_24_P194755	1.0	1.0	-3.7	1.8	MUM1	Homo sapiens melanoma associated antigen (mutated) 1 (MUM1), transcript variant 1, mRNA [NM_032853]	84939
A_23_P161628	2.4	2.4	2.4	2.5	MUS81	Homo sapiens MUS81 endonuclease homolog (S. cerevisiae) (MUS81), mRNA [NM_025128]	80198

A_24_P412238	4.1	4.1	2.8	3.3	MUS81	Homo sapiens MUS81 endonuclease homolog (S. cerevisiae) (MUS81), mRNA [NM_025128]	80198
A_23_P71649	2.4	1.5	-1.2	1.3	MUSK	Homo sapiens muscle, skeletal, receptor tyrosine kinase (MUSK), transcript variant 1, mRNA [NM_005592]	4593
A_24_P910898	-1.7	-10.6	-1.4	-6.6	MUTYH	Homo sapiens mutY homolog (E. coli) (MUTYH), transcript variant gamma3, mRNA [NM_001048173]	4595

A_23_P152125	-5.0	1.4	-1.1	-1.2	MVD	Homo sapiens mevalonate (diphospho) decarboxylase (MVD), mRNA [NM_002461]	4597
A_23_P105592	2.7	2.1	1.2	1.5	MVK	Homo sapiens mevalonate kinase (MVK), transcript variant 1, mRNA [NM_000431]	4598
A_23_P88819	1.7	5.2	4.2	3.0	MVP	Homo sapiens major vault protein (MVP), transcript variant 1, mRNA [NM_017458]	9961
A_23_P124559	2.4	2.4	2.4	2.3	MXD3	Homo sapiens MAX dimerization protein 3 (MXD3), transcript variant 1, mRNA [NM_031300]	83463

A_24_P417706	-1.3	-1.5	-3.0	1.4	MXD3	Homo sapiens MAX dimerization protein 3 (MXD3), transcript variant 2, mRNA [NM_001142935]	83463
A_23_P423695	-1.6	-3.6	1.6	-1.1	MXD4	Homo sapiens MAX dimerization protein 4 (MXD4), mRNA [NM_006454]	10608
A_24_P234732	-1.4	1.3	-1.4	-14.6	MXD4	Homo sapiens MAX dimerization protein 4 (MXD4), mRNA [NM_006454]	10608
A_23_P258136	-5.7	1.1	-1.9	1.2	MXRA5	Homo sapiens matrix-remodelling associated 5 (MXRA5), mRNA [NM_015419]	25878

A_24_P810697	3.7	2.0	2.0	2.5	MXRA7	Homo sapiens matrix-remodelling associated 7 (MXRA7), transcript variant 2, mRNA [NM_001008529]	439921
A_24_P324314	1.7	2.9	2.9	-2.0	MXRA7	Homo sapiens matrix-remodelling associated 7 (MXRA7), transcript variant 2, mRNA [NM_001008529]	439921
A_23_P32444	2.0	1.2	-1.3	1.8	MXRA8	Homo sapiens matrix-remodelling associated 8 (MXRA8), mRNA [NM_032348]	54587

A_24_P134195	-1.2	2.6	1.3	-1.3	MYADM	Homo sapiens myeloid-associated differentiation marker (MYADM), transcript variant 1, mRNA [NM_001020818]	91663
A_23_P399112	1.7	2.2	1.4	-1.1	MYADM	Homo sapiens myeloid-associated differentiation marker (MYADM), transcript variant 1, mRNA [NM_001020818]	91663
A_32_P332	-2.2	-4.9	-1.2	-1.5	MYADML	Homo sapiens myeloid-associated differentiation marker-like (MYADML), non-coding RNA [NR_003143]	151325

A_24_P110331	1.3	1.3	2.4	1.2	MYADML2	Homo sapiens myeloid-associated differentiation marker-like 2 (MYADML2), mRNA [NM_001145113]	255275
A_23_P78170	1.6	1.7	-7.5	1.3	MYBBP1A	Homo sapiens MYB binding protein (P160) 1a (MYBBP1A), transcript variant 2, mRNA [NM_014520]	10514
A_23_P43157	2.6	3.6	3.1	1.6	MYBL1	Homo sapiens v-myb myeloblastosis viral oncogene homolog (avian)-like 1 (MYBL1), transcript variant 1, mRNA [NM_001080416]	4603

A_24_P911928	1.3	1.8	-2.3	-1.6	MYBL1	Homo sapiens v-myb myeloblastosis viral oncogene homolog (avian)-like 1 (MYBL1), transcript variant 2, mRNA [NM_001144755]	4603
A_23_P143190	1.7	-6.7	1.4	1.6	MYBL2	Homo sapiens v-myb myeloblastosis viral oncogene homolog (avian)-like 2 (MYBL2), mRNA [NM_002466]	4605
A_23_P128362	1.1	1.0	-3.4	1.0	MYBPC1	Homo sapiens myosin binding protein C, slow type (MYBPC1), transcript variant 2, mRNA [NM_206819]	4604

A_23_P16262	-1.2	1.3	1.1	-2.7	MYBPC2	Homo sapiens myosin binding protein C, fast type (MYBPC2), mRNA [NM_004533]	4606
A_23_P148737	-6.8	-1.1	1.0	-1.4	MYBPH	Homo sapiens myosin binding protein H (MYBPH), mRNA [NM_004997]	4608
A_24_P178011	-4.1	1.2	1.4	-1.2	MYC	Human c-myc-P64 mRNA, initiating from promoter P0, (HLmyc3.1) partial cds. [M13930]	4609
A_24_P323815	-2.2	1.9	-3.4	-2.0	MYCBP2	Homo sapiens MYC binding protein 2 (MYCBP2), mRNA [NM_015057]	23077
A_23_P141415	1.2	1.2	1.1	-3.9	MYCBPAP	Homo sapiens MYCBP associated protein (MYCBPAP), mRNA [NM_032133]	84073

A_23_P160668	1.5	2.0	1.4	-1.1	MYCL1	Homo sapiens v-myc myelocytomatosis viral oncogene homolog 1, lung carcinoma derived (avian) (MYCL1), transcript variant 3, mRNA [NM_005376]	4610
A_24_P945059	-3.8	1.1	1.1	-1.4	MYCT1	Homo sapiens myc target 1 (MYCT1), mRNA [NM_025107]	80177
A_23_P377212	-1.0	-2.2	-5.3	-1.0	MYEOV2	Homo sapiens myeloma overexpressed 2 (MYEOV2), transcript variant 1, mRNA [NM_138336]	150678
A_23_P87860	3.7	1.0	-1.1	-1.1	MYF5	Homo sapiens myogenic factor 5 (MYF5), mRNA [NM_005593]	4617

A_23_P49657	1.1	1.1	-3.5	-2.3	MYH1	Homo sapiens myosin, heavy chain 1, skeletal muscle, adult (MYH1), mRNA [NM_005963]	4619
A_24_P256764	-1.5	-1.0	-3.2	-1.2	MYH10	Homo sapiens myosin, heavy chain 10, non-muscle (MYH10), mRNA [NM_005964]	4628
A_24_P34168	-5.7	1.2	1.1	-1.0	MYH10	Homo sapiens myosin, heavy chain 10, non-muscle (MYH10), mRNA [NM_005964]	4628
A_23_P164081	-1.0	-1.0	2.3	1.6	MYH10	Homo sapiens myosin, heavy chain 10, non-muscle (MYH10), mRNA [NM_005964]	4628

A_23_P89334	2.2	1.1	1.1	-1.5	MYH13	Homo sapiens myosin, heavy chain 13, skeletal muscle (MYH13), mRNA [NM_003802]	8735
A_23_P78734	-2.5	-2.2	-5.1	-1.1	MYH14	Homo sapiens myosin, heavy chain 14, non-muscle (MYH14), transcript variant 1, mRNA [NM_001077186]	79784
A_24_P11900	3.8	-1.3	3.3	4.1	MYH15	Homo sapiens myosin, heavy chain 15 (MYH15), mRNA [NM_014981]	22989
A_23_P38271	-1.0	-1.0	-2.0	-1.0	MYH2	Homo sapiens myosin, heavy chain 2, skeletal muscle, adult (MYH2), transcript variant 1, mRNA [NM_017534]	4620

A_24_P934145	-1.6	-1.6	-5.5	-1.2	MYH6	Homo sapiens myosin, heavy chain 6, cardiac muscle, alpha (MYH6), mRNA [NM_002471]	4624
A_23_P37164	136.2	98.8	101.4	60.3	MYH6	Homo sapiens myosin, heavy chain 6, cardiac muscle, alpha (MYH6), mRNA [NM_002471]	4624
A_24_P130621	2.0	-1.9	1.5	-1.0	MYH7B	Homo sapiens myosin, heavy chain 7B, cardiac muscle, beta (MYH7B), mRNA [NM_020884]	57644
A_23_P57497	1.1	1.9	3.0	2.4	MYH9	Homo sapiens myosin, heavy chain 9, non-muscle (MYH9), mRNA [NM_002473]	4627

A_23_P4572	-1.2	2.5	-2.1	1.2	MYL12A	Homo sapiens myosin, light chain 12A, regulatory, non-sarcomeric (MYL12A), mRNA [NM_006471]	10627
A_32_P42925	-1.3	-2.2	-2.2	-2.4	MYL12B	Homo sapiens myosin, light chain 12B, regulatory (MYL12B), transcript variant 2, mRNA [NM_033546]	103910
A_23_P162547	-1.1	-1.3	-1.7	-2.6	MYL2	Homo sapiens myosin, light chain 2, regulatory, cardiac, slow (MYL2), mRNA [NM_000432]	4633

A_23_P155638	-1.8	-1.4	-3.0	-3.6	MYL3	Homo sapiens myosin, light chain 3, alkali; ventricular, skeletal, slow (MYL3), mRNA [NM_000258]	4634
A_24_P188218	1.0	-1.0	-1.1	-9.2	MYL4	Homo sapiens myosin, light chain 4, alkali; atrial, embryonic (MYL4), transcript variant 2, mRNA [NM_002476]	4635
A_23_P2223	3.1	5.9	3.8	1.7	MYL6B	Homo sapiens myosin, light chain 6B, alkali, smooth muscle and non-muscle (MYL6B), transcript variant 2, mRNA [NM_002475]	140465

A_23_P210425	-1.2	-2.3	-1.3	-1.0	MYL9	Homo sapiens myosin, light chain 9, regulatory (MYL9), transcript variant 2, mRNA [NM_181526]	10398
A_24_P917123	1.1	1.1	-1.0	-2.7	MYLIP	Homo sapiens myosin regulatory light chain interacting protein (MYLIP), mRNA [NM_013262]	29116
A_24_P319923	1.1	1.0	-4.2	1.1	MYLK	Homo sapiens myosin light chain kinase (MYLK), transcript variant 1, mRNA [NM_053025]	4638
A_23_P143817	1.2	1.2	-7.9	5.7	MYLK	Homo sapiens myosin light chain kinase (MYLK), transcript variant 1, mRNA [NM_053025]	4638

A_23_P80008	4.6	4.7	2.0	6.8	MYLK2	Homo sapiens myosin light chain kinase 2 (MYLK2), mRNA [NM_033118]	85366
A_24_P194707	-1.6	-26.9	-1.0	1.1	MYLK3	Homo sapiens myosin light chain kinase 3 (MYLK3), mRNA [NM_182493]	91807
A_23_P60742	-1.1	1.9	2.1	-3.2	MYLPF	Homo sapiens myosin light chain, phosphorylatable, fast skeletal muscle (MYLPF), mRNA [NM_013292]	29895
A_24_P260254	1.1	1.0	-2.4	1.1	MYNN	Homo sapiens myoneurin (MYNN), transcript variant 2, mRNA [NM_001185118]	55892
A_24_P46357	1.1	-1.8	-9.8	1.0	MYO10	Homo sapiens myosin X (MYO10), mRNA [NM_012334]	4651

A_24_P335759	3.1	3.0	2.8	2.9	MYO15A	Homo sapiens myosin XVA (MYO15A), mRNA [NM_016239]	51168
A_24_P369898	1.4	2.6	1.3	1.0	MYO15B	Homo sapiens cDNA clone IMAGE:40078433. [BC128044]	80022
A_24_P195669	-1.4	-2.9	2.0	-1.6	MYO15B	Homo sapiens cDNA FLJ90199 fis, clone MAMMA1001609. [AK074680]	80022
A_23_P78122	-1.0	-1.1	-1.2	-4.0	MYO18A	Homo sapiens myosin XVIIIA (MYO18A), transcript variant 1, mRNA [NM_078471]	399687
A_23_P68846	1.0	2.0	1.0	-1.3	MYO18B	Homo sapiens myosin XVIIIB (MYO18B), mRNA [NM_032608]	84700

A_23_P100868	-3.0	-1.7	1.1	1.6	MYO19	Homo sapiens myosin XIX (MYO19), transcript variant 3, mRNA [NM_001033580]	80179
A_23_P162288	-4.4	1.6	1.0	-1.1	MYO1A	Homo sapiens myosin IA (MYO1A), mRNA [NM_005379]	4640
A_23_P361049	-8.0	1.0	-1.0	-1.0	MYO1B	Homo sapiens myosin IB (MYO1B), transcript variant 2, mRNA [NM_012223]	4430
A_24_P7143	-4.1	-1.7	-1.4	-2.5	MYO1B	Homo sapiens myosin IB (MYO1B), transcript variant 2, mRNA [NM_012223]	4430
A_23_P164022	-2.1	-1.2	-1.6	-1.3	MYO1C	Homo sapiens myosin IC (MYO1C), transcript variant 3, mRNA [NM_033375]	4641

A_23_P37503	1.7	2.0	1.3	4.0	MYO1E	Homo sapiens myosin IE (MYO1E), mRNA [NM_004998]	4643
A_23_P142447	-1.7	1.4	2.0	1.6	MYO1F	Homo sapiens myosin IF (MYO1F), mRNA [NM_012335]	4542
A_24_P323610	2.5	1.8	-1.1	1.4	MYO1F	Homo sapiens myosin IF (MYO1F), mRNA [NM_012335]	4542
A_24_P110770	-5.1	-1.5	-1.0	-1.0	MYO1G	myosin IG [Source:HGNC Symbol;Acc:13880] [ENST00000480503]	64005
A_32_P78059	-3.3	-1.5	-1.7	-1.6	MYO1H	Homo sapiens myosin IH (MYO1H), mRNA [NM_001101421]	283446
A_23_P417853	-1.0	-2.8	-1.0	1.0	MYO3B	Homo sapiens myosin IIIB (MYO3B), transcript variant 2, mRNA [NM_138995]	140469

A_23_P101193	1.0	-1.3	13.7	-1.0	MYO5B	Homo sapiens myosin VB (MYO5B), mRNA [NM_001080467]	4645
A_23_P255952	1.1	1.1	-2.9	1.0	MYO6	Homo sapiens myosin VI (MYO6), mRNA [NM_004999]	4646
A_24_P283225	3.3	1.1	4.1	1.3	MYO6	Homo sapiens myosin VI (MYO6), mRNA [NM_004999]	4646
A_23_P98786	3.4	1.8	1.7	1.4	MYO7A	Homo sapiens myosin VIIA (MYO7A), transcript variant 1, mRNA [NM_000260]	4647
A_23_P209799	-1.2	2.4	1.9	1.2	MYO7B	Homo sapiens myosin VIIB (MYO7B), mRNA [NM_001080527]	4648

A_24_P53241	1.4	1.0	-3.4	-1.0	MYOCD	Homo sapiens myocardin (MYOCD), transcript variant 2, mRNA [NM_153604]	93649
A_24_P30257	-1.8	-7.2	-5.9	-1.7	MYOD1	Homo sapiens myogenic differentiation 1 (MYOD1), mRNA [NM_002478]	4654
A_24_P282309	-2.1	2.3	1.2	1.1	MYOF	Homo sapiens myoferlin (MYOF), transcript variant 2, mRNA [NM_133337]	26509
A_23_P86682	-3.9	-1.1	-1.1	-4.5	MYOF	Homo sapiens myoferlin (MYOF), transcript variant 1, mRNA [NM_013451]	26509
A_24_P311036	1.5	1.6	-3.7	-1.6	MYOG	Homo sapiens myogenin (myogenic factor 4) (MYOG), mRNA [NM_002479]	4656

A_23_P160438	1.5	2.6	2.3	1.4	MYOG	Homo sapiens myogenin (myogenic factor 4) (MYOG), mRNA [NM_002479]	4656
A_23_P96271	-1.5	-1.3	-1.2	-2.1	MYOM1	Homo sapiens myomesin 1, 185kDa (MYOM1), transcript variant 1, mRNA [NM_003803]	8736
A_23_P258912	-3.0	-1.0	-1.0	-1.0	MYOM2	Homo sapiens myomesin (M-protein) 2, 165kDa (MYOM2), mRNA [NM_003970]	9172
A_23_P51215	3.1	1.1	-1.1	-1.7	MYOM3	Homo sapiens myomesin family, member 3 (MYOM3), mRNA [NM_152372]	127294
A_32_P88415	-1.0	-1.9	1.4	-3.1	MYOZ3	Homo sapiens myozenin 3 (MYOZ3), transcript variant 2, mRNA [NM_133371]	91977

A_23_P104438	1.3	2.5	1.8	-1.5	MYPN	Homo sapiens myopalladin (MYPN), mRNA [NM_032578]	84665
A_23_P398712	9.5	9.4	6.0	9.4	MYT1L	Homo sapiens myelin transcription factor 1-like (MYT1L), mRNA [NM_015025]	23040
A_23_P130455	2.6	-1.3	1.2	-1.1	MZF1	Homo sapiens myeloid zinc finger 1 (MZF1), transcript variant 2, mRNA [NM_198055]	7593
A_23_P432610	1.3	1.0	1.1	-2.6	N4BP1	Homo sapiens NEDD4 binding protein 1 (N4BP1), mRNA [NM_153029]	9683
A_23_P309779	-1.3	1.1	-1.1	-2.3	N4BP2	Homo sapiens NEDD4 binding protein 2 (N4BP2), mRNA [NM_018177]	55728

A_24_P222114	-1.1	-1.0	-2.3	-1.1	N4BP2	Homo sapiens NEDD4 binding protein 2 (N4BP2), mRNA [NM_018177]	55728
A_23_P65262	5.5	2.2	10.7	12.5	N4BP2L2	Homo sapiens NEDD4 binding protein 2-like 2 (N4BP2L2), transcript variant 1, mRNA [NM_033111]	10443
A_23_P80086	-3.3	-1.3	-1.7	-4.7	N6AMT1	Homo sapiens N-6 adenine-specific DNA methyltransferase 1 (putative) (N6AMT1), transcript variant 1, mRNA [NM_013240]	29104

A_23_P151368	-13.9	-5.4	-1.3	-1.7	N6AMT2	Homo sapiens N-6 adenine-specific DNA methyltransferase 2 (putative) (N6AMT2), mRNA [NM_174928]	221143
A_23_P155890	-1.4	2.3	2.8	1.1	NAA11	Homo sapiens N(alpha)-acetyltransferase 11, NatA catalytic subunit (NAA11), mRNA [NM_032693]	84779
A_23_P25868	-4.4	1.0	-1.1	-1.0	NAA30	Homo sapiens N(alpha)-acetyltransferase 30, NatC catalytic subunit (NAA30), mRNA [NM_001011713]	122830
A_24_P63969	3.3	3.1	2.9	3.1	NAA38	Homo sapiens N(alpha)-acetyltransferase 38, NatC auxiliary subunit (NAA38), mRNA [NM_016200]	51691

A_32_P72466	4.3	4.1	4.0	4.0	NAA40	Homo sapiens N(alpha)-acetyltransferase 40, NatD catalytic subunit, homolog (S. cerevisiae) (NAA40), mRNA [NM_024771]	79829
A_23_P35782	-3.3	1.3	-2.6	-1.0	NAA40	Homo sapiens N(alpha)-acetyltransferase 40, NatD catalytic subunit, homolog (S. cerevisiae) (NAA40), mRNA [NM_024771]	79829
A_23_P89113	-5.5	-1.8	-1.8	-1.0	NAA60	Homo sapiens N(alpha)-acetyltransferase 60, NatF catalytic subunit (NAA60), transcript variant 2, mRNA [NM_024845]	79903

A_24_P25520	1.1	2.1	-1.6	-2.4	NAALAD2	N-acetylated alpha-linked acidic dipeptidase 2 [Source:HGNC Symbol;Acc:14526] [ENST00000529090]	10003
A_23_P52668	-7.5	-1.3	-1.4	-1.4	NAALADL1	Homo sapiens N-acetylated alpha-linked acidic dipeptidase-like 1 (NAALADL1), mRNA [NM_005468]	10004
A_24_P191417	-3.2	-3.3	-3.4	-3.2	NAB1	Homo sapiens NGFI-A binding protein 1 (EGR1 binding protein 1) (NAB1), mRNA [NM_005966]	4664

A_23_P368187	-1.1	-2.1	-1.9	-1.8	NAB2	Homo sapiens NGFI-A binding protein 2 (EGR1 binding protein 2) (NAB2), mRNA [NM_005967]	4665
A_23_P162228	-1.3	1.1	1.0	-4.1	NACA	Homo sapiens nascent polypeptide-associated complex alpha subunit (NACA), transcript variant 1, mRNA [NM_001113203]	4666
A_24_P102043	4.7	5.3	-1.2	1.7	NACA	nascent polypeptide-associated complex alpha subunit [Source:HGNC Symbol;Acc:7629] [ENST00000454682]	4666

A_23_P93938	1.2	-9.0	1.3	-1.3	NACAD	Homo sapiens NAC alpha domain containing (NACAD), mRNA [NM_001146334]	23148
A_23_P142518	-4.7	-2.1	-1.5	-1.1	NACC1	Homo sapiens nucleus accumbens associated 1, BEN and BTB (POZ) domain containing (NACC1), mRNA [NM_052876]	112939
A_24_P271285	10.5	-1.2	2.8	1.2	NACC1	Homo sapiens nucleus accumbens associated 1, BEN and BTB (POZ) domain containing (NACC1), mRNA [NM_052876]	112939

A_23_P113825	1.5	-1.5	-2.0	2.7	NACC2	Homo sapiens NACC family member 2, BEN and BTB (POZ) domain containing (NACC2), mRNA [NM_144653]	138151
A_24_P389994	3.2	2.5	3.5	2.2	NADK	Homo sapiens NAD kinase (NADK), transcript variant 1, mRNA [NM_023018]	65220
A_23_P35848	-1.0	-1.0	-2.3	-1.1	NADSYN1	Homo sapiens NAD synthetase 1 (NADSYN1), mRNA [NM_018161]	55191
A_24_P376029	1.4	-12.7	1.2	1.7	NAG18	Homo sapiens NAG18 (NAG18) mRNA, complete cds. [AF210651]	57051

A_24_P307599	1.2	2.9	-1.8	-1.0	NAG20	PREDICTED: Homo sapiens NAG20 (NAG20), miscRNA [XR_108823]	100129703
A_24_P116571	6.4	8.2	9.3	5.5	NAGPA	Homo sapiens N-acetylglucosamine-1-phosphodiester alpha-N-acetylglucosaminidase (NAGPA), mRNA [NM_016256]	51172
A_23_P38041	1.9	-1.0	2.2	2.0	NAGPA	Homo sapiens N-acetylglucosamine-1-phosphodiester alpha-N-acetylglucosaminidase (NAGPA), mRNA [NM_016256]	51172
A_23_P405794	-1.6	1.3	-3.4	-1.1	NAGS	Homo sapiens N-acetylglutamate synthase (NAGS), mRNA [NM_153006]	162417

A_23_P406187	3.4	-1.4	1.1	1.3	NAGS	Homo sapiens N-acetylglutamate synthase (NAGS), mRNA [NM_153006]	162417
A_32_P79396	1.6	-1.3	-1.7	-64.1	NAMPT	Homo sapiens nicotinamide phosphoribosyltransferase (NAMPT), mRNA [NM_005746]	10135
A_24_P383609	1.5	1.1	-2.2	1.1	NANOS1	Homo sapiens nanos homolog 1 (Drosophila) (NANOS1), mRNA [NM_199461]	340719
A_24_P703830	3.9	3.2	5.7	1.4	NANOS3	Homo sapiens nanos homolog 3 (Drosophila) (NANOS3), mRNA [NM_001098622]	342977

A_23_P427039	-4.2	-18.4	-4.4	-4.3	NANP	Homo sapiens N-acetylneuraminic acid phosphatase (NANP), mRNA [NM_152667]	140838
A_32_P82409	-1.4	1.0	1.0	-2.1	NAP1L1	Homo sapiens nucleosome assembly protein 1-like 1 (NAP1L1), transcript variant 2, mRNA [NM_004537]	4673
A_24_P166073	1.1	1.4	2.1	-1.0	NAP1L5	Homo sapiens nucleosome assembly protein 1-like 5 (NAP1L5), mRNA [NM_153757]	266812

A_23_P55990	-1.3	2.2	1.4	1.1	NAPA	Homo sapiens N-ethylmaleimide-sensitive factor attachment protein, alpha (NAPA), transcript variant 1, mRNA [NM_003827]	8775
A_23_P109193	-1.0	-1.1	-1.1	-2.7	NAPB	Homo sapiens N-ethylmaleimide-sensitive factor attachment protein, beta (NAPB), mRNA [NM_022080]	63908
A_32_P105637	8.0	25.7	22.6	17.7	NAPEPLD	Homo sapiens N-acyl phosphatidylethanolamine phospholipase D (NAPEPLD), transcript variant 2, mRNA [NM_198990]	222236

A_23_P43238	2.3	-2.6	3.0	2.6	NAPRT1	Homo sapiens nicotinate phosphoribosyltransferase domain containing 1 (NAPRT1), mRNA [NM_145201]	93100
A_32_P107029	-2.3	-1.4	-1.4	-1.2	NAPSA	Homo sapiens napsin A aspartic peptidase (NAPSA), mRNA [NM_004851]	9476
A_23_P90125	1.2	2.8	2.1	2.6	NAPSB	Homo sapiens napsin B aspartic peptidase pseudogene (NAPSB), non-coding RNA [NR_002798]	256236
A_23_P37877	-1.2	-1.6	1.2	-21.0	NARFL	Homo sapiens nuclear prelamin A recognition factor-like (NARFL), mRNA [NM_022493]	64428

A_23_P50389	3.1	-1.2	1.0	2.9	NAT14	Homo sapiens N-acetyltransferase 14 (GCN5-related, putative) (NAT14), mRNA [NM_020378]	57106
A_23_P31798	1.2	1.2	-3.8	1.1	NAT2	Homo sapiens N-acetyltransferase 2 (arylamine N-acetyltransferase) (NAT2), mRNA [NM_000015]	10
A_23_P212400	1.4	-5.1	1.3	-1.0	NAT6	Homo sapiens N-acetyltransferase 6 (GCN5-related) (NAT6), transcript variant 1, mRNA [NM_012191]	24142
A_23_P103561	-4.0	-1.7	1.3	-1.0	NAV1	Homo sapiens neuron navigator 1 (NAV1), transcript variant 1, mRNA [NM_020443]	89796

A_24_P102880	1.0	1.5	-3.6	-1.1	NAV1	Homo sapiens neuron navigator 1 (NAV1), transcript variant 1, mRNA [NM_020443]	89796
A_24_P248147	-1.7	-2.7	1.0	-1.7	NAV2	Homo sapiens partial mRNA for steerin2 protein (STEERIN2 gene), alternative exon 1c. [AJ488204]	89797
A_23_P354791	-1.0	-1.7	-2.1	-1.8	NAV2	Homo sapiens neuron navigator 2 (NAV2), transcript variant 1, mRNA [NM_182964]	89797
A_23_P90752	1.1	-2.3	-1.0	-4.1	NBAS	Homo sapiens neuroblastoma amplified sequence (NBAS), mRNA [NM_015909]	51594

A_24_P100792	1.3	1.9	1.6	2.1	NBEAL1	neurobeachin-like 1 [Source:HGNC Symbol;Acc:20681] [ENST00000460355]	65065
A_24_P272160	1.2	1.6	2.9	1.6	NBEAL2	neurobeachin-like 2 [Source:HGNC Symbol;Acc:31928] [ENST00000296147]	23218
A_23_P409168	-1.1	-4.1	1.8	1.1	NBEAL2	Homo sapiens neurobeachin-like 2 (NBEAL2), mRNA [NM_015175]	23218
A_24_P566932	-1.0	-10.0	-1.1	-1.0	NBPF1	Homo sapiens cDNA FLJ41266 fis, clone BRAMY2035869. [AK123260]	55672

A_24_P287272	7.9	7.7	7.1	7.4	NBPF1	Homo sapiens neuroblastoma breakpoint family, member 1, mRNA (cDNA clone IMAGE:3896523), complete cds. [BC034418]	55672
A_32_P171181	-5.1	-1.8	-1.8	-1.7	NBPF10	Homo sapiens neuroblastoma breakpoint family, member 10 (NBPF10), mRNA [NM_001039703]	100132406
A_32_P27020	1.1	-3.2	-1.1	1.0	NBPF15	Homo sapiens neuroblastoma breakpoint family, member 15 (NBPF15), transcript variant 2, mRNA [NM_173638]	284565

A_23_P399251	-1.3	-2.1	-3.7	-1.2	NBPF22P	Homo sapiens neuroblastoma breakpoint family, member 22, pseudogene (NBPF22P), non-coding RNA [NR_003719]	285622
A_32_P88635	-6.8	-1.2	-1.2	-1.5	NBPF3	Homo sapiens neuroblastoma breakpoint family, member 3 (NBPF3), mRNA [NM_032264]	84224
A_24_P301454	1.2	1.4	3.0	1.2	NBR2	Homo sapiens neighbor of BRCA1 gene 2 (non-protein coding) (NBR2), non-coding RNA [NR_003108]	10230

A_23_P4160	1.0	-2.3	-2.1	1.4	NBR2	Homo sapiens neighbor of BRCA1 gene 2 (non-protein coding) (NBR2), non-coding RNA [NR_003108]	10230
A_23_P203053	-5.2	-2.9	-2.4	-3.1	NCAM1	Homo sapiens neural cell adhesion molecule 1 (NCAM1), transcript variant 3, mRNA [NM_001076682]	4684
A_23_P1740	2.4	1.2	1.5	1.7	NCAM1	Homo sapiens neural cell adhesion molecule 1 (NCAM1), transcript variant 3, mRNA [NM_001076682]	4684
A_23_P415443	2.7	3.3	2.5	2.6	NCAPH	Homo sapiens non-SMC condensin I complex, subunit H (NCAPH), mRNA [NM_015341]	23397

A_23_P369960	5.0	1.1	1.0	1.0	NCAPH2	Homo sapiens non-SMC condensin II complex, subunit H2 (NCAPH2), transcript variant 1, mRNA [NM_014551]	29781
A_23_P257104	-4.8	2.7	2.7	6.4	NCBP1	Homo sapiens nuclear cap binding protein subunit 1, 80kDa (NCBP1), mRNA [NM_002486]	4686
A_23_P42746	1.6	1.5	2.6	2.0	NCF1	Homo sapiens neutrophil cytosolic factor 1 (NCF1), mRNA [NM_000265]	653361
A_32_P116203	1.7	2.3	-1.3	2.2	NCF1	Homo sapiens neutrophil cytosolic factor 1 (NCF1), mRNA [NM_000265]	653361

A_23_P138194	2.0	2.0	2.0	2.1	NCF2	Homo sapiens neutrophil cytosolic factor 2 (NCF2), transcript variant 1, mRNA [NM_000433]	4688
A_24_P298224	8.8	8.6	5.5	8.3	NCKAP5	Homo sapiens NCK-associated protein 5 (NCKAP5), transcript variant 2, mRNA [NM_207481]	344148
A_24_P50934	-1.0	-4.6	-1.6	-2.1	NCKAP5	Homo sapiens cDNA FLJ34870 fis, clone NT2NE2014651. [AK092189]	344148
A_23_P86424	3.0	-1.1	-1.3	-1.2	NCOA4	Homo sapiens nuclear receptor coactivator 4 (NCOA4), transcript variant 5, mRNA [NM_005437]	8031

A_23_P86421	2.4	5.9	10.6	5.8	NCOA4	Homo sapiens nuclear receptor coactivator 4 (NCOA4), transcript variant 5, mRNA [NM_005437]	8031
A_23_P259135	1.0	-1.6	-2.1	-1.0	NCOA6	Homo sapiens nuclear receptor coactivator 6 (NCOA6), transcript variant 1, mRNA [NM_014071]	23054
A_23_P156957	1.9	2.6	2.3	-1.2	NCOA7	Homo sapiens nuclear receptor coactivator 7 (NCOA7), transcript variant 1, mRNA [NM_181782]	135112
A_24_P911017	1.5	2.0	-2.1	-1.6	NCOR1	nuclear receptor corepressor 1 [Source:HGNC Symbol;Acc:7672] [ENST00000436068]	9611

A_23_P251881	-1.0	-1.0	-2.8	1.2	NCR3	Homo sapiens natural cytotoxicity triggering receptor 3 (NCR3), transcript variant 1, mRNA [NM_147130]	259197
A_32_P468522	3.4	3.4	2.9	1.2	NCR3	Homo sapiens natural cytotoxicity triggering receptor 3 (NCR3), transcript variant 3, mRNA [NM_001145467]	259197
A_23_P73848	1.6	1.6	3.2	1.7	NCRNA00185	Homo sapiens non-protein coding RNA 185 (NCRNA00185), non-coding RNA [NR_001544]	55410

A_23_P331028	-1.3	1.3	-2.3	-3.6	ND1	mitochondrially encoded NADH dehydrogenase 1 [Source:HGNC Symbol;Acc:7455] [ENST00000361390]	4535
A_23_P431853	1.7	-1.1	2.1	1.4	ND2	mitochondrially encoded NADH dehydrogenase 2 [Source:HGNC Symbol;Acc:7456] [ENST00000361453]	4536
A_23_P360209	1.9	4.0	-1.1	-1.1	ND3	mitochondrially encoded NADH dehydrogenase 3 [Source:HGNC Symbol;Acc:7458] [ENST00000361227]	4537

A_23_P315252	-2.0	1.0	-1.1	-1.3	ND4	mitochondrially encoded NADH dehydrogenase 4 [Source:HGNC Symbol;Acc:7459] [ENST00000361381]	4538
A_24_P710024	-1.0	-1.0	-1.2	-2.0	ND4	mitochondrially encoded NADH dehydrogenase 4 [Source:HGNC Symbol;Acc:7459] [ENST00000361381]	4538
A_23_P406928	1.4	1.7	-3.3	-1.0	ND5	mitochondrially encoded NADH dehydrogenase 5 [Source:HGNC Symbol;Acc:7461] [ENST00000361567]	4540

A_23_P206901	1.0	-4.0	1.0	1.0	NDE1	Homo sapiens nudE nuclear distribution gene E homolog 1 (A. nidulans) (NDE1), transcript variant 2, mRNA [NM_017668]	54820
A_24_P209113	-1.7	-2.7	-1.7	-1.2	NDFIP1	Homo sapiens Nedd4 family interacting protein 1 (NDFIP1), mRNA [NM_030571]	80762
A_24_P264909	5.4	1.3	-1.1	1.5	NDFIP2	Homo sapiens Nedd4 family interacting protein 2 (NDFIP2), transcript variant 1, mRNA [NM_019080]	54602
A_23_P106405	1.3	1.3	2.0	-1.0	NDN	Homo sapiens necdin homolog (mouse) (NDN), mRNA [NM_002487]	4692

A_24_P124973	1.0	-3.0	1.0	-2.3	NDNL2	Homo sapiens necdin-like 2 (NDNL2), mRNA [NM_138704]	56160
A_24_P267748	2.0	3.2	-1.4	1.5	NDOR1	Homo sapiens NADPH dependent diflavin oxidoreductase 1 (NDOR1), transcript variant 2, mRNA [NM_014434]	27158
A_23_P73609	1.5	2.6	1.0	1.3	NDP	Homo sapiens Norrie disease (pseudoglioma) (NDP), mRNA [NM_000266]	4693
A_24_P38387	2.0	1.6	-2.1	1.6	NDRG1	Homo sapiens N-myc downstream regulated 1 (NDRG1), transcript variant 2, mRNA [NM_006096]	10397

A_23_P20494	1.2	-1.0	-1.0	-3.3	NDRG1	Homo sapiens N-myc downstream regulated 1 (NDRG1), transcript variant 2, mRNA [NM_006096]	10397
A_23_P37205	2.8	2.5	2.8	1.7	NDRG2	Homo sapiens NDRG family member 2 (NDRG2), transcript variant 1, mRNA [NM_201535]	57447
A_23_P140748	1.4	2.0	1.7	1.1	NDRG4	Homo sapiens NDRG family member 4 (NDRG4), transcript variant 3, mRNA [NM_022910]	65009
A_23_P30429	1.0	-2.2	1.3	1.2	NDST1	Homo sapiens N-deacetylase/N-sulfotransferase (heparan glucosaminyl) 1 (NDST1), mRNA [NM_001543]	3340

A_24_P85365	1.4	1.1	-4.1	-1.0	NDST2	Homo sapiens N-deacetylase/N-sulfotransferase (heparan glucosaminyl) 2 (NDST2), mRNA [NM_003635]	8509
A_24_P396889	1.0	1.0	-7.6	1.3	NDUFA10	Homo sapiens NADH dehydrogenase (ubiquinone) 1 alpha subcomplex, 10, 42kDa (NDUFA10), nuclear gene encoding mitochondrial protein, mRNA [NM_004544]	4705

A_23_P120182	2.5	-1.1	-2.6	-2.0	NDUFA10	Homo sapiens NADH dehydrogenase (ubiquinone) 1 alpha subcomplex, 10, 42kDa (NDUFA10), nuclear gene encoding mitochondrial protein, mRNA [NM_004544]	4705
A_24_P316489	-7.3	-1.6	-1.1	-1.1	NDUFA10	Homo sapiens NADH dehydrogenase (ubiquinone) 1 alpha subcomplex, 10, 42kDa (NDUFA10), nuclear gene encoding mitochondrial protein, mRNA [NM_004544]	4705

A_23_P320185	1.4	-1.1	-1.1	2.3	NDUFA11	Homo sapiens NADH dehydrogenase (ubiquinone) 1 alpha subcomplex, 11, 14.7kDa (NDUFA11), nuclear gene encoding mitochondrial protein, transcript variant 1, mRNA [NM_175614]	126328
A_23_P208540	1.3	-7.0	1.7	2.0	NDUFA3	Homo sapiens NADH dehydrogenase (ubiquinone) 1 alpha subcomplex, 3, 9kDa (NDUFA3), mRNA [NM_004542]	4696

A_32_P98313	1.0	-9.3	-6.2	-1.1	NDUFA4	Homo sapiens NADH dehydrogenase (ubiquinone) 1 alpha subcomplex, 4, 9kDa (NDUFA4), nuclear gene encoding mitochondrial protein, mRNA [NM_002489]	4697
A_23_P412041	1.1	1.1	-19.1	1.2	NDUFA5	NADH dehydrogenase (ubiquinone) 1 alpha subcomplex, 5, 13kDa [Source:HGNC Symbol;Acc:7688] [ENST00000340034]	4698

A_32_P96641	2.1	2.1	2.0	2.0	NDUFA5	Homo sapiens NADH dehydrogenase (ubiquinone) 1 alpha subcomplex, 5, 13kDa (NDUFA5), nuclear gene encoding mitochondrial protein, mRNA [NM_005000]	4698
A_23_P215751	1.0	-1.1	-1.6	4.2	NDUFA5	Homo sapiens NADH dehydrogenase (ubiquinone) 1 alpha subcomplex, 5, 13kDa (NDUFA5), nuclear gene encoding mitochondrial protein, mRNA [NM_005000]	4698

A_32_P49555	2.2	2.1	2.5	2.1	NDUFAF2	Homo sapiens NADH dehydrogenase (ubiquinone) 1 alpha subcomplex, assembly factor 2 (NDUFAF2), nuclear gene encoding mitochondrial protein, mRNA [NM_174889]	91942
A_23_P256172	-1.1	-2.6	-2.5	-1.0	NDUFAF3	Homo sapiens NADH dehydrogenase (ubiquinone) 1 alpha subcomplex, assembly factor 3 (NDUFAF3), nuclear gene encoding mitochondrial protein, transcript variant 1, mRNA [NM_199069]	25915

A_24_P46659	1.8	4.9	1.2	7.9	NDUFAF4	Homo sapiens NADH dehydrogenase (ubiquinone) 1 alpha subcomplex, assembly factor 4 (NDUFAF4), nuclear gene encoding mitochondrial protein, mRNA [NM_014165]	29078
A_23_P140423	2.8	2.3	2.0	2.0	NDUFB1	Homo sapiens NADH dehydrogenase (ubiquinone) 1 beta subcomplex, 1, 7kDa (NDUFB1), nuclear gene encoding mitochondrial protein, mRNA [NM_004545]	4707

A_23_P118105	2.2	1.1	1.1	1.1	NDUFB10	Homo sapiens NADH dehydrogenase (ubiquinone) 1 beta subcomplex, 10, 22kDa (NDUFB10), nuclear gene encoding mitochondrial protein, mRNA [NM_004548]	4716
A_23_P22765	1.8	1.5	1.3	2.8	NDUFB11	Homo sapiens NADH dehydrogenase (ubiquinone) 1 beta subcomplex, 11, 17.3kDa (NDUFB11), nuclear gene encoding mitochondrial protein, transcript variant 1, mRNA [NM_019056]	54539

A_23_P50872	1.6	1.7	2.1	1.4	NDUFB7	Homo sapiens NADH dehydrogenase (ubiquinone) 1 beta subcomplex, 7, 18kDa (NDUFB7), nuclear gene encoding mitochondrial protein, mRNA [NM_004146]	4713
A_24_P14434	1.1	1.8	-2.3	1.6	NDUFB7	Homo sapiens NADH dehydrogenase (ubiquinone) 1 beta subcomplex, 7, 18kDa (NDUFB7), nuclear gene encoding mitochondrial protein, mRNA [NM_004146]	4713

A_24_P346886	-1.0	-1.4	-1.1	-5.5	NDUFB8	Homo sapiens NADH dehydrogenase (ubiquinone) 1 beta subcomplex, 8, 19kDa (NDUFB8), nuclear gene encoding mitochondrial protein, mRNA [NM_005004]	4714
A_23_P149470	1.4	2.7	3.4	1.1	NDUFS2	Homo sapiens NADH dehydrogenase (ubiquinone) Fe-S protein 2, 49kDa (NADH-coenzyme Q reductase) (NDUFS2), nuclear gene encoding mitochondrial protein, transcript variant 1, mRNA [NM_004550]	4720

A_24_P128020	1.4	5.0	4.8	1.4	NDUFS4	Homo sapiens OK/SW-cl.89 mRNA, complete cds. [AB062482]	4724
A_23_P165086	-1.1	-6.4	-3.4	-9.6	NDUFS7	Homo sapiens NADH dehydrogenase (ubiquinone) Fe-S protein 7, 20kDa (NADH-coenzyme Q reductase) (NDUFS7), nuclear gene encoding mitochondrial protein, mRNA [NM_024407]	374291
A_23_P68628	-2.2	-2.4	1.0	-1.8	NECAB3	Homo sapiens N-terminal EF-hand calcium binding protein 3 (NECAB3), transcript variant 2, mRNA [NM_031232]	63941

A_24_P116132	1.6	1.2	-1.4	2.3	NECAP1	Homo sapiens NECAP endocytosis associated 1 (NECAP1), transcript variant 1, mRNA [NM_015509]	25977
A_23_P97296	1.0	4.8	-1.0	1.1	NECAP2	Homo sapiens NECAP endocytosis associated 2 (NECAP2), transcript variant 1, mRNA [NM_018090]	55707
A_24_P302332	-1.6	1.5	2.2	1.8	NECAP2	Homo sapiens NECAP endocytosis associated 2 (NECAP2), transcript variant 1, mRNA [NM_018090]	55707

A_23_P331379	-1.0	-2.4	-1.1	-1.1	NEDD4	Homo sapiens neural precursor cell expressed, developmentally down-regulated 4 (NEDD4), transcript variant 2, mRNA [NM_198400]	4734
A_32_P160883	2.0	2.0	1.4	2.0	NEDD4	Homo sapiens neural precursor cell expressed, developmentally down-regulated 4 (NEDD4), transcript variant 2, mRNA [NM_198400]	4734

A_24_P782308	1.0	-14.9	1.0	1.0	NEDD4L	Homo sapiens neural precursor cell expressed, developmentally down-regulated 4-like (NEDD4L), transcript variant j, mRNA [NM_001144967]	23327
A_24_P108311	1.0	-1.0	-6.7	1.0	NEDD4L	Homo sapiens neural precursor cell expressed, developmentally down-regulated 4-like (NEDD4L), transcript variant d, mRNA [NM_015277]	23327

A_23_P387856	2.0	1.9	-1.9	1.3	NEDD4L	Homo sapiens neural precursor cell expressed, developmentally down-regulated 4-like (NEDD4L), transcript variant d, mRNA [NM_015277]	23327
A_24_P159135	3.7	2.8	-1.3	1.8	NEDD8	Homo sapiens neural precursor cell expressed, developmentally down-regulated 8 (NEDD8), mRNA [NM_006156]	4738

A_24_P710090	-2.7	-1.0	-1.1	-1.1	NEDD8	neural precursor cell expressed, developmentally down-regulated 8 [Source:HGNC Symbol;Acc:7732] [ENST00000533242]	4738
A_23_P344555	1.9	1.8	-2.6	-7.5	NEDD9	Homo sapiens neural precursor cell expressed, developmentally down-regulated 9 (NEDD9), transcript variant 1, mRNA [NM_006403]	4739
A_24_P264832	1.2	2.4	1.1	1.1	NEFM	Homo sapiens neurofilament, medium polypeptide (NEFM), transcript variant 1, mRNA [NM_005382]	4741

A_23_P129157	3.2	1.8	1.6	3.0	NEIL1	Homo sapiens nei endonuclease VIII-like 1 (E. coli) (NEIL1), mRNA [NM_024608]	79661
A_23_P155711	1.4	-13.6	1.0	-2.3	NEIL3	Homo sapiens nei endonuclease VIII-like 3 (E. coli) (NEIL3), mRNA [NM_018248]	55247
A_24_P914599	1.0	-1.0	-1.1	-4.5	NEK1	H.sapiens protein-serine/threonine kinase gene, complete CDS. [Z25431]	4750
A_32_P68050	-2.5	-1.0	1.0	1.1	NEK1	Homo sapiens NIMA (never in mitosis gene a)-related kinase 1 (NEK1), transcript variant 2, mRNA [NM_012224]	4750

A_23_P124427	-4.3	1.0	1.0	1.1	NEK1	Homo sapiens NIMA (never in mitosis gene a)-related kinase 1 (NEK1), transcript variant 2, mRNA [NM_012224]	4750
A_24_P50882	1.0	-3.3	-1.1	-1.1	NEK1	NIMA (never in mitosis gene a)-related kinase 1 [Source:HGNC Symbol;Acc:7744] [ENST00000509912]	4750
A_23_P155301	-14.0	1.0	1.0	1.0	NEK11	Homo sapiens NIMA (never in mitosis gene a)-related kinase 11 (NEK11), transcript variant 2, mRNA [NM_145910]	79858

A_23_P333705	1.0	1.0	-2.3	1.0	NEK3	Homo sapiens NIMA (never in mitosis gene a)-related kinase 3 (NEK3), transcript variant 1, mRNA [NM_002498]	4752
A_24_P921966	-1.0	-2.0	-1.2	-5.5	NEK3		4752
A_24_P80338	-1.1	-1.1	-4.0	-1.5	NEK4	Homo sapiens NIMA (never in mitosis gene a)-related kinase 4 (NEK4), transcript variant 1, mRNA [NM_003157]	6787
A_23_P216920	-1.9	-6.3	-1.8	-3.1	NEK6	Homo sapiens NIMA (never in mitosis gene a)-related kinase 6 (NEK6), transcript variant 2, mRNA [NM_014397]	10783

A_23_P201619	1.2	2.4	-1.1	-1.8	NEK7	Homo sapiens NIMA (never in mitosis gene a)-related kinase 7 (NEK7), mRNA [NM_133494]	140609
A_23_P353574	3.3	2.9	1.3	1.5	NEK7	Homo sapiens NIMA (never in mitosis gene a)-related kinase 7 (NEK7), mRNA [NM_133494]	140609
A_23_P152873	4.0	6.1	4.7	-1.0	NEK8	Homo sapiens NIMA (never in mitosis gene a)-related kinase 8 (NEK8), mRNA [NM_178170]	284086
A_23_P3131	-2.8	1.6	1.7	1.5	NEK9	Homo sapiens NIMA (never in mitosis gene a)-related kinase 9 (NEK9), mRNA [NM_033116]	91754

A_23_P32064	1.3	2.3	3.0	1.8	NELF	Homo sapiens nasal embryonic LHRH factor (NELF), transcript variant 2, mRNA [NM_015537]	26012
A_23_P73000	1.2	2.4	1.1	1.0	NELF	Homo sapiens nasal embryonic LHRH factor (NELF), transcript variant 2, mRNA [NM_015537]	26012
A_24_P6808	-7.7	-2.6	-5.9	-2.5	NEMF	Homo sapiens nuclear export mediator factor (NEMF), mRNA [NM_004713]	9147
A_24_P5653	-56.0	1.1	-1.0	1.0	NETO1	Homo sapiens neuropilin (NRP) and tolloid (TLL)-like 1 (NETO1), transcript variant 1, mRNA [NM_138999]	81832

A_23_P4489	-1.0	-1.0	-7.0	-1.0	NETO1	Homo sapiens neuropilin (NRP) and tolloid (TLL)-like 1 (NETO1), transcript variant 3, mRNA [NM_138966]	81832
A_23_P90980	1.4	1.4	3.3	1.0	NEU2	Homo sapiens sialidase 2 (cytosolic sialidase) (NEU2), mRNA [NM_005383]	4759
A_24_P246891	1.3	-1.3	1.5	-2.5	NEU4	Homo sapiens sialidase 4 (NEU4), transcript variant 1, mRNA [NM_080741]	129807
A_23_P165989	-2.3	1.0	-1.0	-1.3	NEURL2	Homo sapiens neuralized homolog 2 (Drosophila) (NEURL2), mRNA [NM_080749]	140825

A_23_P49579	-2.8	1.7	-1.2	1.8	NEUROD2	Homo sapiens neurogenic differentiation 2 (NEUROD2), mRNA [NM_006160]	4761
A_23_P87625	-5.0	-1.0	-1.2	-1.0	NEUROD4	Homo sapiens neurogenic differentiation 4 (NEUROD4), mRNA [NM_021191]	58158
A_24_P269101	2.9	1.4	1.3	-1.6	NEUROG1	Homo sapiens neurogenin 1 (NEUROG1), mRNA [NM_006161]	4762
A_23_P200001	1.0	1.4	-1.3	-7.5	NEXN	Homo sapiens nexilin (F actin binding protein) (NEXN), transcript variant 1, mRNA [NM_144573]	91624

A_24_P110780	3.1	3.0	2.9	3.1	NEXN-AS1	PREDICTED: Homo sapiens chromosome 1 open reading frame 118 (C1orf118), miscRNA [XR_110470]	374987
A_23_P207596	-11.8	-2.7	-5.3	-3.8	NF1	Homo sapiens neurofibromin 1 (NF1), transcript variant 1, mRNA [NM_001042492]	4763
A_24_P1919	1.7	2.3	-1.7	2.4	NF1	Homo sapiens neurofibromin 1 (NF1), transcript variant 2, mRNA [NM_000267]	4763
A_24_P917026	3.1	1.2	2.8	1.4	NF1	Homo sapiens neurofibromin 1 (NF1), transcript variant 1, mRNA [NM_001042492]	4763

A_23_P29199	2.8	3.6	1.6	3.9	NF2	Homo sapiens neurofibromin 2 (merlin) (NF2), transcript variant 13, mRNA [NM_181831]	4771
A_23_P17911	-1.0	1.4	-2.3	1.6	NFAM1	Homo sapiens NFAT activating protein with ITAM motif 1 (NFAM1), mRNA [NM_145912]	150372
A_24_P350656	-5.3	1.1	1.0	-1.1	NFASC	Homo sapiens neurofascin (NFASC), transcript variant 5, mRNA [NM_001005389]	23114
A_23_P359647	-3.7	-1.0	-3.8	1.0	NFAT5	Homo sapiens nuclear factor of activated T-cells 5, tonicity-responsive (NFAT5), transcript variant 1, mRNA [NM_138714]	10725

A_24_P525917	3.1	2.1	4.3	1.9	NFATC2	nuclear factor of activated T-cells, cytoplasmic, calcineurin-dependent 2 [Source:HGNC Symbol;Acc:7776] [ENST00000371564]	4773
A_23_P91278	1.5	1.7	2.5	2.1	NFATC2	Homo sapiens nuclear factor of activated T-cells, cytoplasmic, calcineurin-dependent 2 (NFATC2), transcript variant 2, mRNA [NM_173091]	4773

A_24_P330625	1.3	-1.8	2.0	1.1	NFATC2IP	Homo sapiens nuclear factor of activated T-cells, cytoplasmic, calcineurin-dependent 2 interacting protein (NFATC2IP), mRNA [NM_032815]	84901
A_23_P77440	1.1	1.0	1.1	-2.3	NFATC3	Homo sapiens nuclear factor of activated T-cells, cytoplasmic, calcineurin-dependent 3 (NFATC3), transcript variant 1, mRNA [NM_173165]	4775

A_23_P140394	3.9	5.9	-1.9	-4.0	NFATC4	Homo sapiens nuclear factor of activated T-cells, cytoplasmic, calcineurin-dependent 4 (NFATC4), transcript variant 2, mRNA [NM_004554]	4776
A_24_P930276	-2.1	-1.3	-2.1	-1.3	NFE2L1	Homo sapiens NRF1 protein (NRF1) mRNA. [L24123]	4779
A_32_P163469	1.0	-2.9	1.3	1.3	NFE2L1	Homo sapiens nuclear factor (erythroid-derived 2)-like 1 (NFE2L1), mRNA [NM_003204]	4779
A_32_P155030	-1.8	-1.3	1.9	-2.0	NFIA	Homo sapiens nuclear factor I/A (NFIA), transcript variant 1, mRNA [NM_001134673]	4774

A_32_P155026	1.2	2.8	-3.5	-1.8	NFIA	Homo sapiens nuclear factor I/A (NFIA), transcript variant 1, mRNA [NM_001134673]	4774
A_24_P804992	-1.1	-1.0	-1.0	-7.5	NFIB	Homo sapiens nuclear factor I/B (NFIB), transcript variant 3, mRNA [NM_005596]	4781
A_23_P216448	2.4	1.6	1.5	2.4	NFIB	Homo sapiens nuclear factor I/B (NFIB), transcript variant 3, mRNA [NM_005596]	4781
A_23_P32253	1.1	1.6	-3.8	-1.0	NFIL3	Homo sapiens nuclear factor, interleukin 3 regulated (NFIL3), mRNA [NM_005384]	4783

A_23_P165295	-26.3	1.0	-1.0	-1.0	NFIX	Homo sapiens nuclear factor I/X (CCAAT-binding transcription factor) (NFIX), mRNA [NM_002501]	4784
A_23_P202156	1.2	2.3	-1.1	1.6	NFKB2	Homo sapiens nuclear factor of kappa light polypeptide gene enhancer in B-cells 2 (p49/p100) (NFKB2), transcript variant 3, mRNA [NM_001077493]	4791
A_23_P106002	1.1	1.3	2.4	1.1	NFKBIA	Homo sapiens nuclear factor of kappa light polypeptide gene enhancer in B-cells inhibitor, alpha (NFKBIA), mRNA [NM_020529]	4792

A_24_P161086	1.0	1.0	-2.3	-1.1	NFKBIB	Homo sapiens nuclear factor of kappa light polypeptide gene enhancer in B-cells inhibitor, beta (NFKBIB), transcript variant 3, non-coding RNA [NR_040515]	4793
A_23_P79086	-1.2	-3.0	-2.3	3.0	NFKBIB	Homo sapiens nuclear factor of kappa light polypeptide gene enhancer in B-cells inhibitor, beta (NFKBIB), transcript variant 1, mRNA [NM_002503]	4793

A_23_P383422	-3.1	-1.6	-1.9	-1.7	NFKBID	Homo sapiens nuclear factor of kappa light polypeptide gene enhancer in B-cells inhibitor, delta (NFKBID), mRNA [NM_139239]	84807
A_23_P145301	1.1	-1.5	-1.4	-3.3	NFKBIL1	Homo sapiens nuclear factor of kappa light polypeptide gene enhancer in B-cells inhibitor-like 1 (NFKBIL1), transcript variant 1, mRNA [NM_005007]	4795

A_23_P212089	-165.8	1.1	1.1	1.2	NFKBIZ	Homo sapiens nuclear factor of kappa light polypeptide gene enhancer in B-cells inhibitor, zeta (NFKBIZ), transcript variant 1, mRNA [NM_031419]	64332
A_23_P24485	1.6	4.3	1.1	3.5	NFRKB	Homo sapiens nuclear factor related to kappaB binding protein (NFRKB), transcript variant 2, mRNA [NM_006165]	4798

A_23_P147296	2.3	2.2	2.1	1.3	NFU1	Homo sapiens NFU1 iron-sulfur cluster scaffold homolog (S. cerevisiae) (NFU1), nuclear gene encoding mitochondrial protein, transcript variant 1, mRNA [NM_015700]	27247
A_23_P53668	-8.9	-2.4	1.0	1.1	NFYB	Homo sapiens nuclear transcription factor Y, beta (NFYB), mRNA [NM_006166]	4801
A_24_P49731	1.1	1.1	-2.1	1.1	NFYB	Homo sapiens nuclear transcription factor Y, beta (NFYB), mRNA [NM_006166]	4801

A_23_P22994	-1.4	-2.8	1.3	-1.1	NFYC	Homo sapiens nuclear transcription factor Y, gamma (NFYC), transcript variant 2, mRNA [NM_014223]	4802
A_24_P165346	1.0	-5.0	1.3	-1.1	NFYC	Homo sapiens nuclear transcription factor Y, gamma (NFYC), transcript variant 2, mRNA [NM_014223]	4802
A_23_P140375	2.1	2.1	1.8	1.7	NGB	Homo sapiens neuroglobin (NGB), mRNA [NM_021257]	58157
A_23_P115190	2.0	-2.1	-2.6	-7.9	NGF	Homo sapiens nerve growth factor (beta polypeptide) (NGF), mRNA [NM_002506]	4803

A_23_P218841	-1.0	2.4	-1.0	-1.0	NGLY1	Homo sapiens N-glycanase 1 (NGLY1), transcript variant 1, mRNA [NM_018297]	55768
A_24_P336474	-1.3	-1.4	-6.6	-1.0	NHLH1	Homo sapiens nescient helix loop helix 1 (NHLH1), mRNA [NM_005598]	4807
A_23_P412491	2.1	-1.2	-2.5	1.4	NHLH2	Homo sapiens nescient helix loop helix 2 (NHLH2), transcript variant 1, mRNA [NM_005599]	4808
A_24_P252780	3.1	1.9	3.7	1.2	NHLRC2	Homo sapiens NHL repeat containing 2 (NHLRC2), mRNA [NM_198514]	374354

A_32_P125496	58.7	58.0	55.1	56.1	NHLRC2	Homo sapiens NHL repeat containing 2 (NHLRC2), mRNA [NM_198514]	374354
A_23_P418934	-1.2	-1.0	11.0	-1.1	NHLRC3	Homo sapiens NHL repeat containing 3 (NHLRC3), transcript variant 2, mRNA [NM_001017370]	387921
A_23_P110005	1.8	1.8	2.1	2.1	NICN1	Homo sapiens nicolin 1 (NICN1), mRNA [NM_032316]	84276
A_24_P412512	-5.8	-1.6	-1.7	-1.2	NIN	Homo sapiens ninein (GSK3B interacting protein) (NIN), transcript variant 6, mRNA [NM_016350]	51199

A_23_P54147	-1.0	1.0	-2.4	-1.0	NIN	Homo sapiens ninein (GSK3B interacting protein) (NIN), transcript variant 6, mRNA [NM_016350]	51199
A_23_P169137	1.8	1.8	3.5	1.6	NINJ1	Homo sapiens ninjurin 1 (NINJ1), mRNA [NM_004148]	4814
A_24_P338971	2.0	5.9	1.6	5.3	NIPA2	Homo sapiens non imprinted in Prader-Willi/Angelman syndrome 2 (NIPA2), transcript variant 1, mRNA [NM_030922]	81614
A_24_P798709	-2.2	-3.8	-2.9	-2.1	NIPAL2	NIPA-like domain containing 2 [Source:HGNC Symbol;Acc:25854] [ENST00000341166]	79815

A_23_P146111	1.9	2.7	1.7	2.6	NIPAL2	Homo sapiens NIPA-like domain containing 2 (NIPAL2), mRNA [NM_024759]	79815
A_23_P46604	1.9	1.6	2.4	-2.7	NIPAL3	Homo sapiens NIPA-like domain containing 3 (NIPAL3), mRNA [NM_020448]	57185
A_24_P180383	2.1	1.4	1.8	1.5	NIPBL	Homo sapiens Nipped-B homolog (Drosophila) (NIPBL), transcript variant B, mRNA [NM_015384]	25836
A_24_P925709	-1.0	-1.0	-2.4	-1.1	NISCH	nischarin [Source:HGNC Symbol;Acc:18006] [ENST00000488380]	11188
A_23_P376661	-1.1	-1.7	-2.6	-1.5	NIT1	Homo sapiens nitrilase 1 (NIT1), transcript variant 1, mRNA [NM_005600]	4817

A_23_P51376	-1.8	-1.2	-1.7	-2.4	NKAIN1	Homo sapiens Na+/K+ transporting ATPase interacting 1 (NKAIN1), mRNA [NM_024522]	79570
A_23_P62276	2.5	-2.7	2.2	1.9	NKAP	Homo sapiens NFKB activating protein (NKAP), mRNA [NM_024528]	79576
A_23_P133854	-6.3	1.0	1.0	-1.0	NKAPL	Homo sapiens NFKB activating protein-like (NKAPL), mRNA [NM_001007531]	222698
A_23_P41804	1.1	-4.6	-7.1	1.9	NKD2	Homo sapiens naked cuticle homolog 2 (Drosophila) (NKD2), mRNA [NM_033120]	85409

A_24_P129107	-2.1	1.0	-2.0	1.1	NKD2	Homo sapiens naked cuticle homolog 2 (Drosophila) (NKD2), mRNA [NM_033120]	85409
A_23_P338519	-2.0	1.6	-1.9	-1.0	NKIRAS1	Homo sapiens NFKB inhibitor interacting Ras-like 1 (NKIRAS1), mRNA [NM_020345]	28512
A_24_P152335	-1.0	-3.2	-1.1	-1.1	NKIRAS2	Homo sapiens NFKB inhibitor interacting Ras-like 2 (NKIRAS2), transcript variant 1, mRNA [NM_001001349]	28511

A_32_P319858	1.7	3.3	1.7	1.6	NKPD1	Homo sapiens NTPase, KAP family P-loop domain containing 1 (NKPD1), mRNA [NM_198478]	284353
A_24_P184692	-1.3	1.1	-1.4	-5.2	NKX2-1	Homo sapiens NK2 homeobox 1 (NKX2-1), transcript variant 2, mRNA [NM_003317]	7080
A_24_P38702	-2.3	-3.3	-2.0	-2.5	NKX2-3	Homo sapiens NK2 homeobox 3 (NKX2-3), mRNA [NM_145285]	159296
A_23_P52425	-1.1	1.1	1.5	2.5	NKX2-3	Homo sapiens NK2 homeobox 3 (NKX2-3), mRNA [NM_145285]	159296

A_23_P396135	1.1	2.0	1.2	1.5	NKX2-5	Homo sapiens NK2 homeobox 5 (NKX2-5), transcript variant 1, mRNA [NM_004387]	1482
A_24_P253293	4.6	-1.8	-2.2	-1.3	NKX2-8	Homo sapiens NK2 homeobox 8 (NKX2-8), mRNA [NM_014360]	26257
A_23_P157545	-1.1	-1.4	-3.5	-1.3	NKX3-1	Homo sapiens NK3 homeobox 1 (NKX3-1), mRNA [NM_006167]	4824
A_23_P86587	2.3	2.3	-1.3	2.5	NKX6-2	Homo sapiens NK6 homeobox 2 (NKX6-2), mRNA [NM_177400]	84504
A_24_P170365	-1.2	-3.1	-4.0	-1.6	NKX6-3	NK6 homeobox 3 [Source:HGNC Symbol;Acc:26328] [ENST00000518699]	157848

A_23_P402804	-2.5	-2.0	-2.8	-1.0	NKX6-3	Homo sapiens cDNA FLJ25169 fis, clone CBR08739. [AK057898]	157848
A_23_P141315	-1.0	-5.8	-6.7	-1.6	NLE1	Homo sapiens notchless homolog 1 (Drosophila) (NLE1), transcript variant 2, mRNA [NM_001014445]	54475
A_23_P18123	2.1	2.1	2.1	1.9	NLGN1	Homo sapiens neuroligin 1 (NLGN1), mRNA [NM_014932]	22871
A_23_P207125	1.0	2.5	1.5	-2.7	NLGN2	Homo sapiens neuroligin 2 (NLGN2), mRNA [NM_020795]	57555
A_23_P33903	-1.3	-1.3	-4.1	-1.4	NLGN4Y	Homo sapiens neuroligin 4, Y-linked (NLGN4Y), transcript variant 1, mRNA [NM_014893]	22829

A_24_P221515	-1.0	3.4	3.2	1.8	NLN	neurolysin (metallopeptidase M3 family) [Source:HGNC Symbol;Acc:16058] [ENST00000506539]	57486
A_23_P329399	1.3	1.5	-4.2	1.1	NLRC3	Homo sapiens NLR family, CARD domain containing 3 (NLRC3), mRNA [NM_178844]	197358
A_23_P402892	2.5	2.4	2.2	2.2	NLRC5	Homo sapiens NLR family, CARD domain containing 5 (NLRC5), mRNA [NM_032206]	84166
A_24_P83615	-2.1	-1.6	-1.6	-3.6	NLRP1	Homo sapiens NLR family, pyrin domain containing 1 (NLRP1), transcript variant 5, mRNA [NM_001033053]	22861

A_23_P399834	4.0	1.7	4.0	3.9	NLRP13	Homo sapiens NLR family, pyrin domain containing 13 (NLRP13), mRNA [NM_176810]	126204
A_23_P164966	-3.7	2.1	1.9	2.0	NLRP4	Homo sapiens NLR family, pyrin domain containing 4 (NLRP4), mRNA [NM_134444]	147945
A_23_P409502	1.1	-3.7	1.1	-1.4	NLRP9	Homo sapiens NLR family, pyrin domain containing 9 (NLRP9), mRNA [NM_176820]	338321
A_24_P244356	-1.0	2.4	-1.0	-1.0	NLRX1	Homo sapiens NLR family member X1 (NLRX1), transcript variant 2, mRNA [NM_170722]	79671

A_23_P88522	1.3	-20.9	-1.0	-1.0	NMB	Homo sapiens neuromed in B (NMB), transcript variant 1, mRNA [NM_0210 77]	4828
A_23_P70634	-1.0	-1.1	-2.0	-2.0	NMBR	Homo sapiens neuromed in B receptor (NMBR), mRNA [NM_0025 11]	4829
A_32_P11451	-5.9	-1.3	-1.4	-1.8	NMD3	Homo sapiens NMD3 homolog (S. cerevisiae) (NMD3), mRNA [NM_0159 38]	51068
A_32_P39324	2.4	-2.8	-1.3	-1.1	NME2P1	AGENCOU RT_13617 208 NIH_MGC _148 Homo sapiens cDNA clone IMAGE:30 333013 5', mRNA sequence [CB993898]	283458

A_23_P152115	3.1	2.6	2.2	1.6	NME3	Homo sapiens non-metastatic cells 3, protein expressed in (NME3), mRNA [NM_002513]	4832
A_24_P210829	-1.1	2.4	1.0	-1.0	NME4	Homo sapiens non-metastatic cells 4, protein expressed in (NME4), nuclear gene encoding mitochondrial protein, mRNA [NM_005009]	4833
A_23_P212672	1.5	1.9	1.7	-61.6	NME9	Homo sapiens NME gene family member 9 (NME9), mRNA [NM_178130]	347736
A_23_P304652	-1.4	-1.4	-6.0	-1.4	NME9	Homo sapiens NME gene family member 9 (NME9), mRNA [NM_178130]	347736

A_23_P201264	-1.2	-1.1	-1.2	-2.7	NMNAT1	Homo sapiens nicotinamide nucleotide adenylyltransferase 1 (NMNAT1), mRNA [NM_022787]	64802
A_23_P354908	1.0	1.0	-27.3	1.1	NMNAT2	Homo sapiens nicotinamide nucleotide adenylyltransferase 2 (NMNAT2), transcript variant 1, mRNA [NM_015039]	23057
A_23_P107373	1.9	-1.9	2.6	1.5	NMT1	Homo sapiens N-myristoyltransferase 1 (NMT1), mRNA [NM_021079]	4836
A_23_P138686	-1.7	-3.0	-1.7	-1.5	NMT2	Homo sapiens N-myristoyltransferase 2 (NMT2), mRNA [NM_004808]	9397

A_24_P382533	2.0	1.8	1.4	2.1	NNAT	Homo sapiens neuronatin (NNAT), transcript variant 1, mRNA [NM_005386]	4826
A_24_P174775	2.7	1.5	1.9	2.5	NNT	nicotinamide nucleotide transhydrogenase [Source:HGNC Symbol;Acc:7863] [ENST00000264663]	23530
A_23_P7074	-1.1	-9.1	1.6	1.0	NOA1	Homo sapiens nitric oxide associated 1 (NOA1), mRNA [NM_032313]	84273
A_24_P516215	-1.0	-2.4	-1.0	-1.0	NOB1	Homo sapiens NIN1/RPN12 binding protein 1 homolog (S. cerevisiae) (NOB1), mRNA [NM_014062]	28987

A_23_P158627	3.2	1.5	1.4	-1.4	NOC4L	Homo sapiens nucleolar complex associated 4 homolog (S. cerevisiae) (NOC4L), mRNA [NM_024078]	79050
A_23_P127322	1.5	-2.6	1.8	2.1	NODAL	Homo sapiens nodal homolog (mouse) (NODAL), mRNA [NM_018055]	4838
A_23_P55073	1.8	1.7	-2.1	1.5	NOL11	Homo sapiens nucleolar protein 11 (NOL11), mRNA [NM_015462]	25926
A_23_P416766	-9.0	-5.5	-2.6	-1.2	NOL4	Homo sapiens nucleolar protein 4 (NOL4), transcript variant 1, mRNA [NM_003787]	8715

A_24_P174903	-2.7	-1.6	-1.6	-2.1	NOL7	Homo sapiens cDNA FLJ90820 fis, clone Y79AA100 1272. [AK075301]	51406
A_24_P360601	3.6	1.6	3.2	3.3	NOL8	Homo sapiens nucleolar protein 8 (NOL8), transcript variant 1, mRNA [NM_0179 48]	55035
A_23_P144726	2.6	7.5	7.0	7.5	NOP16	NOP16 nucleolar protein homolog (yeast) [Source:H GNC Symbol;Ac c:26934] [ENST0000 0451293]	51491
A_23_P204364	2.5	1.8	-2.4	2.3	NOP2	Homo sapiens NOP2 nucleolar protein homolog (yeast) (NOP2), transcript variant 2, mRNA [NM_0010 33714]	4839

A_24_P101402	-1.3	1.0	-2.1	-6.4	NOP56	Homo sapiens NOP56 ribonucleoprotein homolog (yeast) (NOP56), transcript variant 1, mRNA [NM_006392]	10528
A_23_P154447	2.6	2.0	1.1	1.0	NOP58	Homo sapiens NOP58 ribonucleoprotein homolog (yeast) (NOP58), mRNA [NM_015934]	51602
A_24_P934355	-1.1	-1.6	1.0	-2.4	NOS1	Homo sapiens nitric oxide synthase 1 (neuronal) (NOS1), transcript variant 1, mRNA [NM_000620]	4842

A_32_P332320	-1.3	1.8	-1.5	2.4	NOS1	Homo sapiens nitric oxide synthase 1 (neuronal) (NOS1), transcript variant 2, mRNA [NM_001204218]	4842
A_24_P194017	1.4	-1.7	1.2	-12.9	NOSIP	Homo sapiens nitric oxide synthase interacting protein (NOSIP), mRNA [NM_015953]	51070
A_32_P5040	-3.3	2.8	2.6	2.7	NOTCH2NL	Homo sapiens notch 2 N-terminal like (NOTCH2NL), mRNA [NM_203458]	388677
A_24_P399606	-1.8	-2.0	-2.0	-3.2	NOTCH3	Homo sapiens notch 3 (NOTCH3), mRNA [NM_000435]	4854
A_23_P365614	-1.0	-7.9	-1.2	-1.1	NOTCH4	Homo sapiens notch 4 (NOTCH4), mRNA [NM_004557]	4855

A_23_P82929	-4.2	-2.6	-1.3	-1.5	NOV	Homo sapiens nephroblastoma overexpressed gene (NOV), mRNA [NM_002514]	4856
A_24_P29885	2.0	2.2	-4.1	-1.9	NOV	Homo sapiens nephroblastoma overexpressed gene (NOV), mRNA [NM_002514]	4856
A_23_P205435	2.5	2.4	1.0	3.4	NOVA1	Homo sapiens neuro-oncological ventral antigen 1 (NOVA1), transcript variant 1, mRNA [NM_002515]	4857
A_23_P217280	-10.2	-1.0	1.3	-1.5	NOX1	Homo sapiens NADPH oxidase 1 (NOX1), transcript variant NOH-1L, mRNA [NM_007052]	27035

A_23_P316410	-2.3	1.0	1.0	1.1	NOX1	Homo sapiens NADPH oxidase 1 (NOX1), transcript variant NOH-1L, mRNA [NM_007052]	27035
A_23_P47148	7.2	7.2	7.2	7.1	NOX4	Homo sapiens NADPH oxidase 4 (NOX4), transcript variant 1, mRNA [NM_016931]	50507
A_23_P140475	5.5	5.5	3.2	5.5	NOX5	Homo sapiens NADPH oxidase, EF-hand calcium binding domain 5 (NOX5), transcript variant 1, mRNA [NM_024505]	79400
A_23_P254353	2.8	1.1	-1.1	-1.5	NOXA1	Homo sapiens NADPH oxidase activator 1 (NOXA1), mRNA [NM_006647]	10811

A_23_P382682	-3.2	-1.6	1.6	1.8	NOXRED1	Homo sapiens NADP-dependent oxidoreductase domain containing 1 (NOXRED1), mRNA [NM_001113475]	122945
A_23_P21570	3.7	1.4	-1.8	1.4	NPAS3	Homo sapiens neuronal PAS domain protein 3 (NPAS3), transcript variant 3, mRNA [NM_173159]	64067
A_32_P478023	-1.4	-4.5	-1.4	-1.5	NPAS4	Homo sapiens neuronal PAS domain protein 4 (NPAS4), mRNA [NM_178864]	266743
A_23_P300740	-5.7	1.2	-2.8	-1.6	NPB	Homo sapiens neuropeptide B (NPB), mRNA [NM_148896]	256933

A_23_P20458	-10.9	-1.7	-1.7	-1.5	NPBWR1	Homo sapiens neuropeptides B/W receptor 1 (NPBWR1), mRNA [NM_005285]	2831
A_23_P40334	1.9	2.1	-1.5	-1.1	NPBWR2	Homo sapiens neuropeptides B/W receptor 2 (NPBWR2), mRNA [NM_005286]	2832
A_23_P107587	1.2	1.0	1.3	-5.4	NPC1	Homo sapiens Niemann-Pick disease, type C1 (NPC1), mRNA [NM_000271]	4864
A_23_P20075	-1.7	1.2	-4.4	1.2	NPC1L1	Homo sapiens NPC1 (Niemann-Pick disease, type C1, gene)-like 1 (NPC1L1), transcript variant 1, mRNA [NM_013389]	29881

A_24_P324712	13.2	13.0	5.8	2.0	NPC1L1	Homo sapiens NPC1 (Niemann-Pick disease, type C1, gene)-like 1 (NPC1L1), transcript variant 1, mRNA [NM_013389]	29881
A_23_P146572	1.2	2.4	1.3	1.4	NPDC1	Homo sapiens neural proliferation, differentiation and control, 1 (NPDC1), mRNA [NM_015392]	56654
A_23_P164331	-2.4	-1.3	1.4	1.3	NPEPPS	Homo sapiens aminopeptidase puromycin sensitive (NPEPPS), mRNA [NM_006310]	9520
A_23_P1452	5.0	1.9	-1.0	1.3	NPFFR1	Homo sapiens neuropeptide FF receptor 1 (NPFFR1), mRNA [NM_022146]	64106

A_24_P71890	1.0	1.3	-2.0	1.1	NPFFR2	Homo sapiens neuropeptide FF receptor 2 (NPFFR2), transcript variant 2, mRNA [NM_053036]	10886
A_24_P88801	2.2	2.0	1.2	1.2	NPHP1	Homo sapiens nephronophthisis 1 (juvenile) (NPHP1), transcript variant 1, mRNA [NM_000272]	4867
A_23_P393656	2.2	2.1	-2.0	1.6	NPHP3	Homo sapiens cDNA FLJ31331 fis, clone MAMGL1000056. [AK055893]	27031
A_23_P328652	1.4	-5.3	-1.2	-2.6	NPHP3	Homo sapiens nephronophthisis 3 (adolescent) (NPHP3), mRNA [NM_153240]	27031

A_23_P351734	1.2	1.1	-2.3	-1.4	NPHP4	Homo sapiens nephronophthisis 4 (NPHP4), mRNA [NM_015102]	261734
A_23_P5200	-1.6	1.0	-2.1	-1.0	NPHS1	Homo sapiens nephrosis 1, congenital, Finnish type (nephrin) (NPHS1), mRNA [NM_004646]	4868
A_24_P110564	1.2	-1.1	-2.7	3.4	NPIP	Homo sapiens nuclear pore complex interacting protein (NPIP), mRNA [NM_006985]	9284
A_32_P140823	1.5	5.9	-1.1	1.8	NPIP	Homo sapiens nuclear pore complex interacting protein (NPIP), mRNA [NM_006985]	9284

A_32_P14843	2.4	10.5	2.0	-1.0	NPIPL2	nuclear pore complex interacting protein-like 2 [Source:HGNC Symbol;Acc:34409] [ENST00000429990]	440348
A_24_P826646	3.4	3.4	4.2	3.6	NPM1	Homo sapiens nucleophosmin (nucleolar phosphoprotein B23, numatrin) (NPM1), transcript variant 3, mRNA [NM_001037738]	4869
A_23_P169007	2.5	5.8	4.6	1.8	NPM2	Homo sapiens nucleophosmin/nucleoplasmin 2 (NPM2), mRNA [NM_182795]	10361
A_23_P62752	1.3	-2.2	1.0	1.3	NPPB	Homo sapiens natriuretic peptide B (NPPB), mRNA [NM_002521]	4879

A_23_P301942	-1.1	-1.1	-2.5	-1.6	NPPC	Homo sapiens natriuretic peptide C (NPPC), mRNA [NM_024409]	4880
A_23_P123622	1.0	2.7	2.3	1.7	NPR2	Homo sapiens natriuretic peptide receptor B/guanylate cyclase B (atrionatriuretic peptide receptor B) (NPR2), mRNA [NM_003995]	4882
A_23_P327451	-1.1	-3.3	-1.2	-1.3	NPR3	Homo sapiens natriuretic peptide receptor C/guanylate cyclase C (atrionatriuretic peptide receptor C) (NPR3), transcript variant 2, mRNA [NM_000908]	4883

A_23_P18267	-1.5	3.9	3.4	2.0	NPRL2	Homo sapiens nitrogen permease regulator-like 2 (S. cerevisiae) (NPRL2), mRNA [NM_006545]	10641
A_24_P304969	-2.9	-2.5	-2.4	-2.2	NPRL3	Homo sapiens nitrogen permease regulator-like 3 (S. cerevisiae) (NPRL3), transcript variant 2, mRNA [NM_001039476]	8131
A_23_P385192	1.1	1.1	-4.4	-1.0	NPSA	Homo sapiens prostate-specific antigen mRNA, complete cds. [AF527974]	266811
A_23_P19886	1.2	-1.0	-2.8	-1.3	NPSR1	Homo sapiens neuropeptide S receptor 1 (NPSR1), transcript variant 2, mRNA [NM_207173]	387129

A_23_P256470	-1.2	1.7	2.8	1.5	NPY	Homo sapiens neuropeptide Y (NPY), mRNA [NM_000905]	4852
A_23_P69699	-30.4	1.1	-1.0	-1.1	NPY1R	Homo sapiens neuropeptide Y receptor Y1 (NPY1R), mRNA [NM_000909]	4886
A_23_P251836	1.5	1.6	1.8	2.3	NPY5R	Homo sapiens neuropeptide Y receptor Y5 (NPY5R), mRNA [NM_006174]	4889
A_23_P206661	-1.2	1.2	-3.1	-3.7	NQO1	Homo sapiens NAD(P)H dehydrogenase, quinone 1 (NQO1), transcript variant 1, mRNA [NM_000903]	1728

A_23_P160800	1.6	-1.6	2.3	1.1	NR0B2	Homo sapiens nuclear receptor subfamily 0, group B, member 2 (NR0B2), mRNA [NM_021969]	8431
A_24_P250227	1.1	1.2	-2.1	-3.8	NR1D1	Homo sapiens nuclear receptor subfamily 1, group D, member 1 (NR1D1), mRNA [NM_021724]	9572
A_23_P67332	-1.2	-1.2	-4.4	-1.9	NR1H2	Homo sapiens nuclear receptor subfamily 1, group H, member 2 (NR1H2), mRNA [NM_007121]	7376

A_23_P47135	2.3	1.5	1.1	1.7	NR1H3	Homo sapiens nuclear receptor subfamily 1, group H, member 3 (NR1H3), transcript variant 1, mRNA [NM_005693]	10062
A_23_P25396	3.7	7.0	3.5	3.4	NR1H4	Homo sapiens nuclear receptor subfamily 1, group H, member 4 (NR1H4), transcript variant 2, mRNA [NM_005123]	9971
A_23_P319572	-1.1	2.2	2.1	1.0	NR1I3	Homo sapiens nuclear receptor subfamily 1, group I, member 3 (NR1I3), transcript variant 1, mRNA [NM_001077482]	9970

A_23_P40936	1.0	-1.0	-6.1	-1.1	NR2C2	Homo sapiens nuclear receptor subfamily 2, group C, member 2 (NR2C2), mRNA [NM_003298]	7182
A_24_P126181	2.6	3.7	2.3	1.0	NR2C2AP	Homo sapiens nuclear receptor 2C2-associated protein (NR2C2AP), mRNA [NM_176880]	126382
A_32_P144710	1.8	1.7	2.6	1.7	NR2C2AP	Homo sapiens nuclear receptor 2C2-associated protein (NR2C2AP), mRNA [NM_176880]	126382
A_23_P338372	1.2	-3.2	1.2	-1.9	NR2E1	Homo sapiens nuclear receptor subfamily 2, group E, member 1 (NR2E1), mRNA [NM_003269]	7101

A_23_P205867	-2.0	1.0	-1.3	-1.2	NR2E3	Homo sapiens nuclear receptor subfamily 2, group E, member 3 (NR2E3), transcript variant 2, mRNA [NM_014249]	10002
A_23_P348737	-5.8	1.1	1.0	1.1	NR2F1	Homo sapiens nuclear receptor subfamily 2, group F, member 1 (NR2F1), mRNA [NM_005654]	7025
A_23_P108326	1.2	2.1	2.0	2.6	NR2F6	Homo sapiens nuclear receptor subfamily 2, group F, member 6 (NR2F6), mRNA [NM_005234]	2063

A_24_P787914	1.1	-2.5	1.1	1.1	NR3C1	Homo sapiens nuclear receptor subfamily 3, group C, member 1 (glucocorticoid receptor) (NR3C1), transcript variant 8, mRNA [NM_001204265]	2908
A_23_P131208	-1.4	-1.5	-1.6	-2.2	NR4A2	Homo sapiens nuclear receptor subfamily 4, group A, member 2 (NR4A2), mRNA [NM_006186]	4929
A_23_P216915	1.5	2.6	1.4	-1.0	NR5A1	Homo sapiens nuclear receptor subfamily 5, group A, member 1 (NR5A1), mRNA [NM_004959]	2516

A_23_P161004	-1.9	-2.1	-1.1	-2.3	NR5A2	Homo sapiens nuclear receptor subfamily 5, group A, member 2 (NR5A2), transcript variant 1, mRNA [NM_205860]	2494
A_23_P216900	1.6	1.1	2.5	1.0	NR6A1	nuclear receptor subfamily 6, group A, member 1 [Source:HGNC Symbol;Acc:7985] [ENST00000487099]	2649
A_24_P230703	-2.1	1.0	1.0	1.1	NRAP	Homo sapiens nebulin-related anchoring protein (NRAP), transcript variant 2, mRNA [NM_198060]	4892

A_23_P63190	1.5	1.5	-5.1	1.0	NRAS	Homo sapiens neuroblastoma RAS viral (v-ras) oncogene homolog (NRAS), mRNA [NM_002524]	4893
A_24_P242132	-3.5	-1.5	-1.1	1.0	NRBP2	Homo sapiens nuclear receptor binding protein 2 (NRBP2), mRNA [NM_178564]	340371
A_23_P23006	4.9	11.0	8.2	5.2	NRD1	Homo sapiens nardilysin (N-arginine dibasic convertase) (NRD1), transcript variant 1, mRNA [NM_002525]	4898
A_23_P136493	1.8	1.8	2.9	1.0	NRG1	Homo sapiens neuregulin 1 (NRG1), transcript variant GGF2, mRNA [NM_013962]	3084

A_23_P360777	1.6	-1.0	2.6	-2.5	NRG1	Homo sapiens neuregulin 1 (NRG1), transcript variant ndf43, mRNA [NM_013960]	3084
A_32_P163894	2.3	2.0	3.4	1.6	NRG2	Homo sapiens neuregulin 2 (NRG2), transcript variant 3, mRNA [NM_013982]	9542
A_23_P61761	2.0	-4.0	1.1	1.0	NRG3	Homo sapiens neuregulin 3 (NRG3), transcript variant 1, mRNA [NM_001010848]	10718
A_23_P116264	1.5	2.7	2.5	1.2	NRGN	Homo sapiens neurogranin (protein kinase C substrate, RC3) (NRGN), transcript variant 1, mRNA [NM_006176]	4900

A_23_P13929	1.4	1.4	1.3	2.9	NRIP2	Homo sapiens nuclear receptor interacting protein 2 (NRIP2), mRNA [NM_031474]	83714
A_24_P403799	1.8	2.4	2.6	1.9	NRIP2	Homo sapiens nuclear receptor interacting protein 2 (NRIP2), mRNA [NM_031474]	83714
A_23_P47682	1.1	4.3	-2.6	-1.9	NRIP3	Homo sapiens nuclear receptor interacting protein 3 (NRIP3), mRNA [NM_020645]	56675
A_23_P8055	3.4	1.9	3.5	-1.2	NRM	Homo sapiens nurim (nuclear envelope membrane protein) (NRM), mRNA [NM_007243]	11270
A_23_P82088	2.6	1.5	2.6	2.1	NRN1	Homo sapiens neuritin 1 (NRN1), mRNA [NM_016588]	51299

A_23_P414519	4.4	-1.1	3.3	1.7	NRN1	Homo sapiens neuritin 1 (NRN1), mRNA [NM_016588]	51299
A_24_P59327	1.8	1.8	1.7	2.9	NRP1	neuropilin 1 [Source:HGNC Symbol;Acc:8004] [ENST00000374823]	8829
A_23_P393727	1.9	3.3	-3.8	-1.2	NRP2	Homo sapiens neuropilin 2 (NRP2), transcript variant 1, mRNA [NM_201266]	8828
A_23_P429555	13.0	-1.3	4.1	4.0	NRP2	Homo sapiens neuropilin 2 (NRP2), transcript variant 4, mRNA [NM_018534]	8828
A_23_P255616	2.6	2.6	2.6	2.7	NRSN1	Homo sapiens neurensin 1 (NRSN1), mRNA [NM_080723]	140767

A_24_P416837	6.0	6.4	4.0	2.0	NRSN2	Homo sapiens neurensin 2 (NRSN2), mRNA [NM_024958]	80023
A_23_P90359	3.0	5.1	3.2	2.4	NRTN	Homo sapiens neurturin (NRTN), mRNA [NM_004558]	4902
A_23_P95453	1.7	1.7	-2.5	1.7	NRXN1	Homo sapiens neurexin 1 (NRXN1), transcript variant alpha1, mRNA [NM_004801]	9378
A_24_P261470	1.4	1.4	1.4	-2.6	NRXN2	Homo sapiens neurexin 2 (NRXN2), transcript variant alpha-2, mRNA [NM_138732]	9379

A_24_P238333	1.0	-1.0	-2.1	-1.1	NSA2	Homo sapiens NSA2 ribosome biogenesis homolog (S. cerevisiae) (NSA2), mRNA [NM_014886]	10412
A_23_P167444	-11.1	1.3	1.4	1.0	NSA2	Homo sapiens NSA2 ribosome biogenesis homolog (S. cerevisiae) (NSA2), mRNA [NM_014886]	10412
A_23_P15714	1.0	-1.0	-2.5	-1.0	NSF	Homo sapiens N-ethylmaleimide-sensitive factor (NSF), transcript variant 1, mRNA [NM_006178]	4905

A_23_P134809	-2.2	1.1	1.1	1.1	NSMAF	Homo sapiens neutral sphingomyelinase (N-SMase) activation associated factor (NSMAF), transcript variant 1, mRNA [NM_003580]	8439
A_23_P422794	-4.0	2.1	-1.1	-1.7	NSMCE2	Homo sapiens non-SMC element 2, MMS21 homolog (S. cerevisiae) (NSMCE2), mRNA [NM_173685]	286053
A_32_P213822	-3.6	1.0	1.0	1.1	NSUN3	NOP2/Sun domain family, member 3 [Source:HGNC Symbol;Acc:26208] [ENST00000314622]	63899

A_23_P21785	2.5	2.3	-1.2	1.1	NSUN3	Homo sapiens NOP2/Sun domain family, member 3 (NSUN3), mRNA [NM_022072]	63899
A_24_P130792	-1.9	-1.0	1.4	-3.0	NSUN4	Homo sapiens NOP2/Sun domain family, member 4 (NSUN4), mRNA [NM_199044]	387338
A_24_P272300	2.4	1.0	1.0	1.0	NSUN4	Homo sapiens NOP2/Sun domain family, member 4 (NSUN4), mRNA [NM_199044]	387338
A_23_P501183	-1.0	3.6	-1.1	-2.0	NSUN5P2	Homo sapiens NOP2/Sun domain family, member 5 pseudogene 2 (NSUN5P2), non-coding RNA [NR_033323]	260294

A_32_P421816	-1.0	-1.0	-17.5	-1.0	NT5C1B	Homo sapiens 5'-nucleotidase, cytosolic IB (NT5C1B), transcript variant 1, mRNA [NM_001002006]	93034
A_23_P49924	17.4	-1.0	14.8	4.7	NT5C3L	Homo sapiens 5'-nucleotidase, cytosolic III-like (NT5C3L), transcript variant 1, mRNA [NM_052935]	115024
A_23_P382043	1.0	13.9	-1.1	-1.1	NT5DC1	Homo sapiens 5'-nucleotidase domain containing 1 (NT5DC1), mRNA [NM_152729]	221294
A_23_P44836	1.5	1.4	-2.1	-1.4	NT5DC2	Homo sapiens 5'-nucleotidase domain containing 2 (NT5DC2), transcript variant 2, mRNA [NM_022908]	64943

A_32_P76720	1.1	1.0	-1.0	-7.8	NT5DC3	Homo sapiens 5'-nucleotidase domain containing 3 (NT5DC3), mRNA [NM_001031701]	51559
A_24_P530690	2.5	2.2	1.3	1.1	NTF3	neurotrophin 3 [Source:HGNC Symbol;Acc:8023] [ENST00000535299]	4908
A_23_P360797	1.6	2.9	1.5	1.0	NTF3	Homo sapiens neurotrophin 3 (NTF3), transcript variant 2, mRNA [NM_002527]	4908
A_23_P15478	1.1	-6.0	-1.4	-3.7	NTN1	Homo sapiens netrin 1 (NTN1), mRNA [NM_004822]	9423
A_23_P100408	1.4	1.7	2.4	1.0	NTN3	Homo sapiens netrin 3 (NTN3), mRNA [NM_006181]	4917

A_24_P85375	-1.0	-11.2	-1.1	-1.0	NTN5	Homo sapiens netrin 5, mRNA (cDNA clone MGC:12924 IMAGE:4137205), complete cds. [BC021210]	126147
A_24_P415195	1.1	-3.0	1.3	1.1	NTRK2	Homo sapiens mRNA; cDNA DKFZp686D1394 (from clone DKFZp686D1394). [BX649001]	4915
A_23_P205900	3.6	1.2	2.2	2.6	NTRK3	Homo sapiens neurotrophic tyrosine kinase, receptor, type 3 (NTRK3), transcript variant 1, mRNA [NM_001012338]	4916
A_23_P36882	-1.3	-1.3	-1.4	-20.3	NTS	Homo sapiens neurotensin (NTS), mRNA [NM_006183]	4922

A_24_P378806	2.7	1.9	-1.1	1.5	NTSR1	Homo sapiens neurotensin receptor 1 (high affinity) (NTSR1), mRNA [NM_002531]	4923
A_23_P315212	1.1	-3.9	-1.1	1.3	NTSR2	Homo sapiens neurotensin receptor 2 (NTSR2), mRNA [NM_012344]	23620
A_23_P348257	-1.1	-1.5	-2.2	1.1	NUAK1	Homo sapiens NUAK family, SNF1-like kinase, 1 (NUAK1), mRNA [NM_014840]	9891
A_23_P215154	1.1	3.8	3.8	1.6	NUB1	Homo sapiens negative regulator of ubiquitin-like proteins 1 (NUB1), transcript variant 2, mRNA [NM_016118]	51667

A_23_P170908	1.0	-6.4	-1.0	-1.1	NUBPL	Homo sapiens nucleotide binding protein-like (NUBPL), transcript variant 1, mRNA [NM_025152]	80224
A_23_P78771	-2.5	-1.1	-1.0	-2.7	NUCB1	Homo sapiens nucleobindin 1 (NUCB1), mRNA [NM_006184]	4924
A_23_P149724	-1.1	-5.6	-1.2	-1.2	NUCKS1	Homo sapiens nuclear casein kinase and cyclin-dependent kinase substrate 1 (NUCKS1), mRNA [NM_022731]	64710
A_32_P41471	-3.1	1.0	1.1	1.0	NUDT13	Homo sapiens nudix (nucleoside diphosphate linked moiety X)-type motif 13 (NUDT13), mRNA [NM_015901]	25961

A_32_P5976	2.8	2.8	-1.8	1.4	NUDT14	Homo sapiens nudix (nucleoside diphosphate linked moiety X)-type motif 14 (NUDT14), mRNA [NM_177533]	256281
A_24_P399065	-1.3	-1.3	-1.1	-2.2	NUDT16L1	Homo sapiens nudix (nucleoside diphosphate linked moiety X)-type motif 16-like 1 (NUDT16L1), transcript variant 1, mRNA [NM_032349]	84309

A_32_P5376	3.1	2.2	2.7	1.4	NUDT16P1	Homo sapiens nudix (nucleoside diphosphate linked moiety X)-type motif 16 pseudogene 1 (NUDT16P1), transcript variant 2, non-coding RNA [NR_002949]	152195
A_23_P112478	-2.0	-1.6	-2.9	-1.4	NUDT2	Homo sapiens nudix (nucleoside diphosphate linked moiety X)-type motif 2 (NUDT2), transcript variant 1, mRNA [NM_001161]	318
A_24_P15797	1.2	1.1	2.1	-1.0	NUDT22	Homo sapiens cDNA FLJ34477 fis, clone HLUNG2003833. [AK091796]	84304

A_23_P75441	28.4	7.8	25.1	27.7	NUDT22	Homo sapiens nudix (nucleoside diphosphate linked moiety X)-type motif 22 (NUDT22), transcript variant 1, mRNA [NM_032344]	84304
A_32_P117723	-1.5	1.1	-1.7	-3.4	NUDT4	Homo sapiens nudix (nucleoside diphosphate linked moiety X)-type motif 4 (NUDT4), transcript variant 2, mRNA [NM_199040]	11163

A_32_P40463	-5.7	1.2	1.1	1.2	NUDT9P1	Homo sapiens nudix (nucleoside diphosphate linked moiety X)-type motif 9 pseudogene 1 (NUDT9P1), non-coding RNA [NR_002779]	119369
A_23_P74349	2.2	1.5	2.0	1.6	NUF2	Homo sapiens NUF2, NDC80 kinetochore complex component, homolog (S. cerevisiae) (NUF2), transcript variant 1, mRNA [NM_145697]	83540

A_23_P36939	-2.7	-1.0	-1.7	-1.0	NUFIP1	Homo sapiens nuclear fragile X mental retardatio n protein interacting protein 1 (NUFIP1), mRNA [NM_0123 45]	26747
A_24_P270376	2.2	1.4	-1.2	-1.0	NUFIP1	Homo sapiens nuclear fragile X mental retardatio n protein interacting protein 1 (NUFIP1), mRNA [NM_0123 45]	26747
A_24_P921144	2.2	2.1	2.0	2.2	NUFIP2	Homo sapiens nuclear fragile X mental retardatio n protein interacting protein 2 (NUFIP2), mRNA [NM_0207 72]	57532

A_23_P162120	-2.5	-4.5	-1.6	1.0	NUMA1	Homo sapiens nuclear mitotic apparatus protein 1 (NUMA1), mRNA [NM_006185]	4926
A_23_P12503	-1.0	-2.6	-1.0	-1.0	NUP133	Homo sapiens nucleoporin 133kDa (NUP133), mRNA [NM_018230]	55746
A_24_P237753	3.6	2.4	1.4	5.6	NUP188	Homo sapiens nucleoporin 188kDa (NUP188), mRNA [NM_015354]	23511
A_32_P375062	1.6	-2.0	-1.2	1.2	NUP210P1	Homo sapiens nucleoporin 210kDa pseudogene 1 (NUP210P1), non-coding RNA [NR_034158]	255330
A_23_P211627	-5.4	-1.0	3.8	-1.0	NUP50	Homo sapiens nucleoporin 50kDa (NUP50), transcript variant 2, mRNA [NM_007172]	10762

A_23_P204891	1.1	-2.6	1.1	1.1	NUPL1	Homo sapiens nucleoporin like 1 (NUPL1), transcript variant 1, mRNA [NM_014089]	9818
A_23_P123039	2.5	1.0	1.1	-1.1	NUPL2	Homo sapiens nucleoporin like 2 (NUPL2), mRNA [NM_007342]	11097
A_32_P34116	-1.0	-1.0	-1.0	-2.4	NUS1	Homo sapiens nuclear undecaprenyl pyrophosphate synthase 1 homolog (S. cerevisiae) (NUS1), mRNA [NM_138459]	116150
A_23_P96990	1.5	-2.6	1.0	-1.0	NVL	Homo sapiens nuclear VCP-like (NVL), transcript variant 1, mRNA [NM_002533]	4931

A_23_P148568	-7.4	-1.1	-1.2	-1.1	NXF2	Homo sapiens nuclear RNA export factor 2 (NXF2), mRNA [NM_022053]	56001
A_23_P171336	2.1	1.7	1.6	1.8	NXF3	Homo sapiens nuclear RNA export factor 3 (NXF3), mRNA [NM_022052]	56000
A_23_P384650	2.7	2.2	2.4	1.1	NXNL1	Homo sapiens nucleoredoxin-like 1 (NXNL1), mRNA [NM_138454]	115861
A_23_P316121	3.9	1.1	1.1	1.0	NXPH2	Homo sapiens neurexophilin 2 (NXPH2), mRNA [NM_007226]	11249
A_23_P310590	-2.5	-1.0	-1.1	-1.0	OAF	Homo sapiens OAF homolog (Drosophila) (OAF), mRNA [NM_178507]	220323

A_23_P64828	1.0	-1.0	-4.6	1.0	OAS1	Homo sapiens 2'-5'-oligoadenylate synthetase 1, 40/46kDa (OAS1), transcript variant 2, mRNA [NM_002534]	4938
A_23_P204087	-1.0	2.1	-1.3	2.4	OAS2	Homo sapiens 2'-5'-oligoadenylate synthetase 2, 69/71kDa (OAS2), transcript variant 1, mRNA [NM_016817]	4939
A_24_P335305	2.4	3.3	2.4	2.4	OAS3	Homo sapiens 2'-5'-oligoadenylate synthetase 3, 100kDa (OAS3), mRNA [NM_006187]	4940

A_23_P47955	2.5	2.4	1.2	2.4	OAS3	Homo sapiens 2'-5'-oligoadenylate synthetase 3, 100kDa (OAS3), mRNA [NM_006187]	4940
A_23_P409945	-4.2	1.1	-1.0	1.1	OAZ1	Homo sapiens ornithine decarboxylase antizyme 1 (OAZ1), mRNA [NM_004152]	4946
A_23_P409951	1.7	-1.1	-9.7	1.1	OAZ1	Homo sapiens ornithine decarboxylase antizyme 1 (OAZ1), mRNA [NM_004152]	4946
A_23_P46852	-1.7	-1.7	1.4	-2.4	OBFC1	Homo sapiens oligonucleotide/oligosaccharide-binding fold containing 1 (OBFC1), mRNA [NM_024928]	79991

A_24_P759674	1.8	1.1	2.3	2.3	OBFC1	Homo sapiens oligonucleotide/oligosaccharide-binding fold containing 1 (OBFC1), mRNA [NM_024928]	79991
A_24_P229531	-1.8	-2.2	-2.1	-2.1	OBFC2A	Homo sapiens oligonucleotide/oligosaccharide-binding fold containing 2A (OBFC2A), transcript variant 1, mRNA [NM_001031716]	64859
A_23_P87482	-1.0	-2.7	-2.2	-1.2	OBFC2B	Homo sapiens oligonucleotide/oligosaccharide-binding fold containing 2B (OBFC2B), mRNA [NM_024068]	79035

A_24_P283928	2.9	4.4	4.1	2.7	OBFC2B	Homo sapiens oligonucleotide/oligosaccharide-binding fold containing 2B (OBFC2B), mRNA [NM_024068]	79035
A_23_P63128	1.1	-1.9	-1.4	-3.8	OBSCN	Homo sapiens cDNA FLJ14124 fis, clone MAMMA1002498. [AK024186]	84033
A_24_P119685	1.4	1.6	2.2	1.8	OBSCN	Homo sapiens obscurin, cytoskeletal calmodulin and titin-interacting RhoGEF (OBSCN), transcript variant 1, mRNA [NM_052843]	84033
A_23_P90523	-42.9	1.1	1.4	1.4	OCEL1	Homo sapiens occludin/ELL domain containing 1 (OCEL1), mRNA [NM_024578]	79629

A_24_P102053	1.1	1.0	-5.0	-1.0	OCLN	Homo sapiens occludin (OCLN), transcript variant 1, mRNA [NM_002538]	100506658
A_24_P196307	-1.4	1.3	-2.2	-15.9	OCR1	Homo sapiens ovarian cancer-related protein 1 (OCR1) mRNA, complete cds. [AF314543]	100128298
A_23_P170246	1.0	-1.0	-1.1	-6.8	OCRL	Homo sapiens oculocerebrorenal syndrome of Lowe (OCRL), transcript variant a, mRNA [NM_000276]	4952
A_23_P58228	-1.1	-4.0	4.9	2.7	ODAM	Homo sapiens odontogenic, ameloblast asssociated (ODAM), mRNA [NM_017855]	54959

A_23_P165840	2.8	1.9	1.2	1.5	ODC1	Homo sapiens ornithine decarboxylase 1 (ODC1), mRNA [NM_002539]	4953
A_23_P31626	-4.7	1.4	1.2	1.0	ODF1	Homo sapiens outer dense fiber of sperm tails 1 (ODF1), mRNA [NM_024410]	4956
A_32_P337508	2.8	1.1	3.3	1.9	ODF3L2	Homo sapiens outer dense fiber of sperm tails 3-like 2 (ODF3L2), mRNA [NM_182577]	284451
A_23_P421526	1.0	1.0	-2.1	1.0	ODF4	Homo sapiens outer dense fiber of sperm tails 4 (ODF4), mRNA [NM_153007]	146852

A_24_P299474	1.1	2.8	1.3	1.1	ODZ2	Homo sapiens odz, odd Oz/ten-m homolog 2 (Drosophila) (ODZ2), mRNA [NM_001122679]	57451
A_24_P418357	-1.0	-6.6	-1.1	-1.1	ODZ3	Homo sapiens odz, odd Oz/ten-m homolog 3 (Drosophila) (ODZ3), mRNA [NM_001080477]	55714
A_24_P69053	1.2	-1.0	-6.3	-1.0	ODZ3	Homo sapiens odz, odd Oz/ten-m homolog 3 (Drosophila) (ODZ3), mRNA [NM_001080477]	55714
A_23_P121722	2.6	1.8	3.0	-1.4	ODZ3	Homo sapiens odz, odd Oz/ten-m homolog 3 (Drosophila) (ODZ3), mRNA [NM_001080477]	55714

A_24_P926284	1.1	-8.4	1.1	1.0	OFCC1	Homo sapiens clone 10AP3 MRDS1 protein (MRDS1) mRNA, partial cds. [AF520801]	266553
A_24_P9454	-6.9	-1.4	-2.3	1.3	OGDH	Homo sapiens oxoglutarate (alpha-ketoglutarate) dehydrogenase (lipoamide) (OGDH), nuclear gene encoding mitochondrial protein, transcript variant 1, mRNA [NM_002541]	4967

A_23_P123133	4.1	6.1	2.8	4.5	OGDH	Homo sapiens oxoglutarate (alpha-ketoglutarate) dehydrogenase (lipoamide) (OGDH), nuclear gene encoding mitochondrial protein, transcript variant 1, mRNA [NM_002541]	4967
A_23_P3775	-2.6	1.1	-1.0	-1.0	OGFOD1	Homo sapiens 2-oxoglutarate and iron-dependent oxygenase domain containing 1 (OGFOD1), mRNA [NM_018233]	55239

A_23_P344392	1.8	2.5	-1.8	-15.4	OGG1	Homo sapiens 8-oxoguanine DNA glycosylase (OGG1), nuclear gene encoding mitochondrial protein, transcript variant 1a, mRNA [NM_002542]	4968
A_23_P82990	1.1	1.0	-2.5	1.1	OGN	Homo sapiens osteoglycin (OGN), transcript variant 1, mRNA [NM_033014]	4969
A_23_P381976	-2.6	1.0	-1.0	1.0	OGT	Homo sapiens O-linked N-acetylglucosamine (GlcNAc) transferase (UDP-N-acetylglucosamine:polypeptide-N-acetylglucosaminyl transferase) (OGT), transcript variant 1, mRNA [NM_181672]	8473

A_32_P67544	1.1	1.1	1.0	2.8	OIP5-AS1	Homo sapiens mRNA; cDNA DKFZp686E0352 (from clone DKFZp686E0352). [BX647764]	729082
A_24_P912126	-1.0	-1.0	2.4	1.2	OK/SW-CL.58	Homo sapiens mRNA for OK/SW-CL.58, complete cds. [AB064667]	100131766
A_24_P114438	-1.0	-1.6	-1.0	-3.0	OLA1	Homo sapiens Obg-like ATPase 1 (OLA1), transcript variant 1, mRNA [NM_013341]	29789
A_23_P161458	-1.0	2.1	-1.0	-1.5	OLAH	Homo sapiens oleoyl-ACP hydrolase (OLAH), transcript variant 2, mRNA [NM_001039702]	55301

A_24_P406601	1.1	-34.8	-1.0	-1.0	OLFM1	Homo sapiens olfactomedin 1 (OLFM1), transcript variant 1, mRNA [NM_014279]	10439
A_23_P219161	2.7	-1.2	-1.6	-1.8	OLFM1	Homo sapiens olfactomedin 1 (OLFM1), transcript variant 2, mRNA [NM_006334]	10439
A_23_P67151	-1.1	2.0	-1.3	-2.2	OLFM2	Homo sapiens olfactomedin 2 (OLFM2), mRNA [NM_058164]	93145
A_24_P402444	-1.0	-1.1	-1.1	-10.6	OLFM3	Homo sapiens olfactomedin 3 (OLFM3), mRNA [NM_058170]	118427
A_24_P181254	-4.7	1.0	1.0	1.1	OLFM4	Homo sapiens olfactomedin 4 (OLFM4), mRNA [NM_006418]	10562

A_24_P100856	3.2	3.2	2.6	1.4	OLFML2A	Homo sapiens olfactomedin-like 2A (OLFML2A), mRNA [NM_182487]	169611
A_32_P24832	-1.8	-1.4	-2.9	-3.2	OLFML3	Homo sapiens olfactomedin-like 3 (OLFML3), mRNA [NM_020190]	56944
A_24_P124624	3.9	3.9	3.8	3.9	OLR1	Homo sapiens oxidized low density lipoprotein (lectin-like) receptor 1 (OLR1), transcript variant 1, mRNA [NM_002543]	4973
A_23_P138139	-1.1	-1.0	-1.1	4.4	OMA1	Homo sapiens OMA1 homolog, zinc metallopeptidase (S. cerevisiae) (OMA1), mRNA [NM_145243]	115209

A_23_P116587	1.1	2.4	-2.0	1.5	OMP	Homo sapiens olfactory marker protein (OMP), mRNA [NM_006189]	4975
A_23_P340717	20.3	2.5	24.2	19.9	ONECUT1	Homo sapiens one cut homeobox 1 (ONECUT1), mRNA [NM_004498]	3175
A_32_P124708	1.1	1.1	-1.3	2.2	ONECUT2	Homo sapiens one cut homeobox 2 (ONECUT2), mRNA [NM_004852]	9480
A_32_P58606	-2.2	1.1	1.1	-1.7	ONECUT2	Homo sapiens one cut homeobox 2 (ONECUT2), mRNA [NM_004852]	9480
A_32_P14762	1.0	-4.0	1.1	-1.0	OOEP	Homo sapiens oocyte expressed protein homolog (dog) (OOEP), mRNA [NM_001080507]	441161

A_24_P710866	1.4	-2.5	1.0	-1.9	OPA3	UI-CF-FN0-afr-i-16-0-UI.s1 UI-CF-FN0 Homo sapiens cDNA clone UI-CF-FN0-afr-i-16-0-UI 3', mRNA sequence [CA944687]	80207
A_24_P14257	1.3	-1.4	-3.8	-1.2	OPA3	Homo sapiens optic atrophy 3 (autosomal recessive, with chorea and spastic paraplegia) (OPA3), nuclear gene encoding mitochondrial protein, transcript variant 2, mRNA [NM_025136]	80207

A_24_P132813	2.0	1.0	1.0	1.0	OPALIN	Homo sapiens oligodendrocytic myelin paranodal and inner loop protein (OPALIN), transcript variant 3, mRNA [NM_001040103]	93377
A_23_P72663	1.1	-3.2	-1.4	-1.3	OPCML	Homo sapiens opioid binding protein/cell adhesion molecule-like (OPCML), transcript variant 2, mRNA [NM_001012393]	4978
A_23_P417753	-1.0	-2.5	-1.1	-1.0	OPHN1	Homo sapiens oligophrenin 1 (OPHN1), mRNA [NM_002547]	4983
A_23_P170186	1.5	1.5	2.5	2.5	OPLAH	Homo sapiens 5-oxoprolinase (ATP-hydrolysing) (OPLAH), mRNA [NM_017570]	26873

A_23_P96623	1.3	-5.6	-1.1	1.4	OPN1MW	Homo sapiens opsin 1 (cone pigments), medium-wave-sensitive (OPN1MW), mRNA [NM_000513]	2652
A_23_P58934	1.1	-1.4	1.0	-2.2	OPN5	opsin 5 [Source:HGNC Symbol;Acc:19992] [ENST00000393699]	221391
A_23_P345564	1.0	1.0	2.1	1.0	OPRL1	Homo sapiens opiate receptor-like 1 (OPRL1), transcript variant 1, mRNA [NM_182647]	4987
A_23_P145480	-1.0	-3.7	-1.5	-3.5	OPRM1	Homo sapiens opioid receptor, mu 1 (OPRM1), transcript variant MOR-1, mRNA [NM_000914]	4988

A_24_P397613	-1.4	-1.9	-2.1	-1.4	OPRM1	Homo sapiens opioid receptor, mu 1 (OPRM1), transcript variant MOR-1A, mRNA [NM_001008504]	4988
A_23_P321935	1.1	1.0	1.0	-2.8	OSBP2	Homo sapiens oxysterol binding protein 2 (OSBP2), transcript variant 1, mRNA [NM_030758]	23762
A_24_P117902	2.4	1.3	-3.2	1.6	OSBPL10	Homo sapiens oxysterol binding protein-like 10 (OSBPL10), transcript variant 1, mRNA [NM_017784]	114884

A_23_P147245	3.2	3.0	2.6	-8.2	OSBPL10	Homo sapiens oxysterol binding protein-like 10 (OSBPL10), transcript variant 1, mRNA [NM_017784]	114884
A_23_P10442	1.1	-3.9	-1.0	-1.0	OSBPL1A	Homo sapiens oxysterol binding protein-like 1A (OSBPL1A), transcript variant 2, mRNA [NM_080597]	114876
A_23_P210995	-3.5	1.2	1.1	-1.3	OSBPL2	Homo sapiens oxysterol binding protein-like 2 (OSBPL2), transcript variant 2, mRNA [NM_144498]	9885

A_24_P599340	-4.2	-1.1	-1.1	-1.1	OSBPL3	Homo sapiens oxysterol binding protein-like 3 (OSBPL3), transcript variant 1, mRNA [NM_015550]	26031
A_23_P53081	-5.3	1.1	-2.9	-1.2	OSBPL5	Homo sapiens oxysterol binding protein-like 5 (OSBPL5), transcript variant 1, mRNA [NM_020896]	114879
A_32_P82671	-2.5	-3.8	1.1	-1.1	OSBPL9	Homo sapiens oxysterol binding protein-like 9 (OSBPL9), transcript variant 7, mRNA [NM_148909]	114883

A_23_P50368	2.7	1.6	1.5	1.7	OSCAR	Homo sapiens osteoclast associated, immunoglobulin-like receptor (OSCAR), transcript variant 1, mRNA [NM_206818]	126014
A_23_P50374	1.4	-1.4	-1.3	-2.1	OSCAR	Homo sapiens osteoclast associated, immunoglobulin-like receptor (OSCAR), transcript variant 3, mRNA [NM_130771]	126014
A_23_P77415	1.3	1.4	-1.3	-3.7	OSGIN1	Homo sapiens oxidative stress induced growth inhibitor 1 (OSGIN1), nuclear gene encoding mitochondrial protein, mRNA [NM_182981]	29948

A_23_P82859	-8.6	-1.0	-1.1	-1.0	OSGIN2	Homo sapiens oxidative stress induced growth inhibitor family member 2 (OSGIN2), transcript variant 2, mRNA [NM_004337]	734
A_23_P166408	1.4	2.5	1.4	1.4	OSM	Homo sapiens oncostatin M (OSM), mRNA [NM_020530]	5008
A_23_P156289	2.5	2.5	1.3	3.9	OSMR	Homo sapiens oncostatin M receptor (OSMR), transcript variant 1, mRNA [NM_003999]	9180
A_24_P145134	-1.3	1.0	1.0	-2.0	OSMR	Homo sapiens oncostatin M receptor (OSMR), transcript variant 2, mRNA [NM_001168355]	9180

A_24_P385732	1.0	2.3	-1.1	1.0	OSTalpha	Homo sapiens organic solute transporter alpha (OSTalpha), mRNA [NM_152672]	200931
A_23_P436284	-1.0	-1.1	-1.2	-2.1	OSTBETA	Homo sapiens organic solute transporter beta (OSTBETA), mRNA [NM_178859]	123264
A_23_P411814	3.5	1.2	2.4	1.5	OSTC	Homo sapiens oligosaccharyltransferase complex subunit (OSTC), mRNA [NM_021227]	58505
A_24_P317450	10.4	20.1	9.5	20.5	OSTCP1	Homo sapiens oligosaccharyltransferase complex subunit pseudogene 1 (OSTCP1), non-coding RNA [NR_028496]	202459

A_23_P60296	1.0	-2.6	-1.0	1.0	OSTF1	Homo sapiens osteoclast stimulating factor 1 (OSTF1), mRNA [NM_012383]	26578
A_23_P404785	-5.5	-17.9	1.0	1.1	OSTM1	Homo sapiens osteopetrosis associated transmembrane protein 1 (OSTM1), mRNA [NM_014028]	28962
A_23_P302634	1.6	1.6	-2.2	-1.2	OTOGL	Homo sapiens otogelin-like (OTOGL), mRNA [NM_173591]	283310
A_23_P306307	-4.7	1.0	-1.0	-1.0	OTOP1	Homo sapiens otopetrin 1 (OTOP1), mRNA [NM_177998]	133060
A_23_P365201	1.3	6.1	2.5	2.1	OTOP3	Homo sapiens otopetrin 3 (OTOP3), mRNA [NM_178233]	347741

A_23_P90997	1.4	2.0	-2.5	-1.6	OTOS	Homo sapiens otospiralin (OTOS), mRNA [NM_148961]	150677
A_23_P24375	2.2	2.7	-3.8	2.1	OTUB1	Homo sapiens OTU domain, ubiquitin aldehyde binding 1 (OTUB1), transcript variant 1, mRNA [NM_017670]	55611
A_24_P124879	1.6	2.6	1.0	2.0	OTUB2	Homo sapiens OTU domain, ubiquitin aldehyde binding 2 (OTUB2), mRNA [NM_023112]	78990
A_23_P337550	1.6	2.3	2.9	1.4	OTUD5	Homo sapiens OTU domain containing 5 (OTUD5), transcript variant 1, mRNA [NM_017602]	55593

A_24_P42389	2.7	6.2	4.0	1.5	OTUD6A	Homo sapiens OTU domain containing 6A (OTUD6A), mRNA [NM_207320]	139562
A_24_P318400	1.9	2.8	2.8	1.0	OTUD7A	Homo sapiens OTU domain containing 7A (OTUD7A), mRNA [NM_130901]	161725
A_23_P3413	1.3	1.7	2.4	-1.3	OTUD7A	Homo sapiens OTU domain containing 7A (OTUD7A), mRNA [NM_130901]	161725
A_24_P912691	1.6	2.0	-1.6	1.3	OTUD7A	OTU domain containing 7A [Source:HGNC Symbol;Acc:20718] [ENST00000307050]	161725

A_23_P138157	2.2	2.2	2.2	-2.3	OTUD7B	Homo sapiens OTU domain containing 7B (OTUD7B), mRNA [NM_020205]	56957
A_24_P359799	-2.8	-1.9	-1.2	-2.2	OTX1	Homo sapiens orthodenticle homeobox 1 (OTX1), transcript variant 1, mRNA [NM_014562]	5013
A_24_P39195	-7.2	1.0	1.0	1.0	OXGR1	Homo sapiens oxoglutarate (alpha-ketoglutarate) receptor 1 (OXGR1), mRNA [NM_080818]	27199
A_23_P418413	2.4	2.3	2.2	2.4	OXSR1	Homo sapiens oxidative-stress responsive 1 (OXSR1), mRNA [NM_005109]	9943

A_24_P337104	-2.3	1.3	-1.1	1.7	OXTR	Homo sapiens oxytocin receptor (OXTR), mRNA [NM_000916]	5021
A_23_P132619	10.2	9.8	9.4	10.5	OXTR	Homo sapiens oxytocin receptor (OXTR), mRNA [NM_000916]	5021
A_23_P372848	2.1	1.4	1.4	-1.1	P2RX1	Homo sapiens purinergic receptor P2X, ligand-gated ion channel, 1 (P2RX1), mRNA [NM_002558]	5023
A_23_P127721	-4.0	1.6	2.3	1.7	P2RX3	Homo sapiens purinergic receptor P2X, ligand-gated ion channel, 3 (P2RX3), mRNA [NM_002559]	5024

A_23_P53623	-1.8	-4.5	-1.9	-1.8	P2RX4	Homo sapiens purinergic receptor P2X, ligand-gated ion channel, 4 (P2RX4), mRNA [NM_002560]	5025
A_23_P433785	1.2	-1.0	-2.1	1.4	P2RX5	Homo sapiens purinergic receptor P2X, ligand-gated ion channel, 5 (P2RX5), transcript variant 1, mRNA [NM_002561]	5026
A_23_P346376	16.6	16.7	17.3	9.8	P2RY10	Homo sapiens purinergic receptor P2Y, G-protein coupled, 10 (P2RY10), transcript variant 1, mRNA [NM_014499]	27334

A_23_P114307	-1.1	-1.1	-3.1	1.7	P2RY4	Homo sapiens pyrimidinergic receptor P2Y, G-protein coupled, 4 (P2RY4), mRNA [NM_002565]	5030
A_23_P64611	1.9	2.3	3.7	2.2	P2RY6	Homo sapiens pyrimidinergic receptor P2Y, G-protein coupled, 6 (P2RY6), transcript variant 2, mRNA [NM_176798]	5031
A_24_P340128	-1.1	-1.0	-2.2	-1.1	P2RY8	Homo sapiens purinergic receptor P2Y, G-protein coupled, 8 (P2RY8), mRNA [NM_178129]	286530

A_23_P18966	3.7	-1.0	1.1	-1.9	P4HA2	Homo sapiens prolyl 4-hydroxylase, alpha polypeptide II (P4HA2), transcript variant 2, mRNA [NM_001017973]	8974
A_24_P213684	82.3	80.4	74.6	85.5	P4HB	Homo sapiens mRNA for OK/SW-CL.24, complete cds. [AB062434]	5034
A_23_P107412	-2.1	1.0	1.1	1.0	P4HB	Homo sapiens prolyl 4-hydroxylase, beta polypeptide (P4HB), mRNA [NM_000918]	5034

A_23_P113311	-14.5	1.1	1.3	-1.1	P4HTM	Homo sapiens prolyl 4-hydroxylase, transmembrane (endoplasmic reticulum) (P4HTM), transcript variant 3, mRNA [NM_177938]	54681
A_32_P11181	-1.9	2.1	-1.4	1.1	PA2G4	Homo sapiens proliferation-associated 2G4, 38kDa (PA2G4), mRNA [NM_006191]	5036
A_24_P221123	-1.8	-1.6	-2.2	-1.0	PA2G4	Homo sapiens proliferation-associated 2G4, 38kDa (PA2G4), mRNA [NM_006191]	5036

A_23_P139339	-1.0	-1.1	-2.6	-1.2	PAAF1	Homo sapiens proteasomal ATPase-associated factor 1 (PAAF1), mRNA [NM_025155]	80227
A_23_P436484	1.0	1.1	-2.2	-1.0	PABPC1L	poly(A) binding protein, cytoplasmic 1-like [Source:HGNC Symbol;Acc:15797] [ENST00000372821]	80336
A_23_P309599	1.1	1.7	-4.4	1.1	PABPC1L	poly(A) binding protein, cytoplasmic 1-like [Source:HGNC Symbol;Acc:15797] [ENST00000372826]	80336
A_24_P92183	-2.0	-7.2	-3.0	-4.7	PABPC1L	Homo sapiens poly(A) binding protein, cytoplasmic 1-like (PABPC1L), mRNA [NM_001124756]	80336

A_24_P162454	1.5	1.0	2.3	-1.7	PABPC1L2B	Homo sapiens poly(A) binding protein, cytoplasmic 1-like 2B (PABPC1L2B), mRNA [NM_001042506]	645974
A_23_P48307	-1.1	-1.3	-3.8	-1.7	PABPC3	Homo sapiens poly(A) binding protein, cytoplasmic 3 (PABPC3), mRNA [NM_030979]	5042
A_23_P500282	1.1	2.8	2.0	1.4	PACS1	Homo sapiens phosphofurin acidic cluster sorting protein 1 (PACS1), mRNA [NM_018026]	55690
A_24_P255628	1.0	-1.0	-1.1	-2.7	PACS2	Homo sapiens phosphofurin acidic cluster sorting protein 2 (PACS2), transcript variant 2, mRNA [NM_015197]	23241

A_23_P80377	-1.2	-2.8	1.2	1.5	PACSIN2	Homo sapiens protein kinase C and casein kinase substrate in neurons 2 (PACSIN2), transcript variant 2, mRNA [NM_007229]	11252
A_23_P138262	6.3	6.3	5.7	5.8	PADI4	Homo sapiens peptidyl arginine deiminase, type IV (PADI4), mRNA [NM_012387]	23569
A_23_P369502	1.6	2.0	3.1	1.8	PADI4	Homo sapiens peptidyl arginine deiminase, type IV (PADI4), mRNA [NM_012387]	23569

A_24_P216087	-1.5	-11.1	1.0	1.3	PAF1	Homo sapiens Paf1, RNA polymerase II associated factor, homolog (S. cerevisiae) (PAF1), mRNA [NM_019088]	54623
A_23_P207811	1.3	3.2	-1.8	1.2	PAIP1	Homo sapiens poly(A) binding protein interacting protein 1 (PAIP1), transcript variant 1, mRNA [NM_006451]	10605
A_23_P301053	-1.8	1.6	-1.6	-5.8	PAIP2B	Homo sapiens poly(A) binding protein interacting protein 2B (PAIP2B), mRNA [NM_020459]	400961

A_23_P327307	21.0	12.5	13.6	8.0	PAK2	Homo sapiens p21 protein (Cdc42/Rac)-activated kinase 2 (PAK2), mRNA [NM_002577]	5062
A_32_P104075	-2.5	-1.5	-1.0	-1.4	PAK2	Homo sapiens p21 protein (Cdc42/Rac)-activated kinase 2 (PAK2), mRNA [NM_002577]	5062
A_23_P346813	-2.2	1.1	-1.1	-1.0	PAK3	Homo sapiens p21 protein (Cdc42/Rac)-activated kinase 3 (PAK3), transcript variant 2, mRNA [NM_002578]	5063

A_24_P313822	-7.4	1.1	-1.0	4.0	PAK4	Homo sapiens p21 protein (Cdc42/Rac)-activated kinase 4 (PAK4), transcript variant 1, mRNA [NM_005884]	10298
A_23_P208991	-2.2	-24.8	-1.3	-1.1	PALM	Homo sapiens paralemmin (PALM), transcript variant 1, mRNA [NM_002579]	5064
A_32_P198177	1.5	-2.1	1.4	-1.0	PALM3	Homo sapiens paralemmin 3 (PALM3), mRNA [NM_001145028]	342979
A_23_P23669	2.1	-5.3	1.4	-3.1	PALMD	Homo sapiens palmdelphin (PALMD), mRNA [NM_017734]	54873

A_23_P385938	-3.2	1.2	1.2	1.5	PAN3	Homo sapiens PAN3 poly(A) specific ribonuclease subunit homolog (S. cerevisiae) (PAN3), mRNA [NM_175854]	255967
A_24_P921032	2.4	1.4	1.3	-1.2	PANK3	pantothenate kinase 3 [Source:HGNC Symbol;Acc:19365] [ENST00000239231]	79646
A_23_P47155	1.9	-3.4	1.5	1.6	PANX1	Homo sapiens pannexin 1 (PANX1), mRNA [NM_015368]	24145
A_23_P52634	-1.1	-15.4	-4.1	-1.4	PANX1	Homo sapiens pannexin 1 (PANX1), mRNA [NM_015368]	24145

A_23_P155162	2.2	1.2	1.1	-1.0	PANX2	Homo sapiens pannexin 2 (PANX2), transcript variant 2, mRNA [NM_001160300]	56666
A_23_P87072	-6.9	-4.5	-3.9	-2.3	PANX3	Homo sapiens pannexin 3 (PANX3), mRNA [NM_052959]	116337
A_24_P940197	4.5	5.6	4.2	3.5	PAPD5	Homo sapiens PAP associated domain containing 5 (PAPD5), transcript variant 1, mRNA [NM_001040284]	64282
A_23_P18824	1.0	-1.5	-2.5	1.0	PAPD7	Homo sapiens PAP associated domain containing 7 (PAPD7), transcript variant 1, mRNA [NM_006999]	11044

A_24_P53406	1.3	1.0	-1.0	-10.0	PAPL	Homo sapiens iron/zinc purple acid phosphatase-like protein (PAPL), mRNA [NM_001004318]	390928
A_23_P333640	-1.8	-2.7	-2.5	-22.6	PAPLN	Homo sapiens papilin, proteoglycan-like sulfated glycoprotein (PAPLN), mRNA [NM_173462]	89932
A_23_P140347	2.0	1.5	-1.1	-3.9	PAPLN	Homo sapiens papilin, proteoglycan-like sulfated glycoprotein (PAPLN), mRNA [NM_173462]	89932
A_23_P99559	-1.0	-1.5	-6.1	-1.6	PAPOLA	Homo sapiens poly(A) polymerase alpha (PAPOLA), transcript variant 1, mRNA [NM_032632]	10914

A_23_P419051	1.1	-6.8	-1.3	1.6	PAPOLA	Homo sapiens poly(A) polymerase alpha (PAPOLA), transcript variant 1, mRNA [NM_032632]	10914
A_24_P141995	-1.5	1.4	-1.2	2.0	PAPOLA	Homo sapiens poly(A) polymerase alpha (PAPOLA), transcript variant 1, mRNA [NM_032632]	10914
A_24_P807883	1.1	1.1	-2.8	-1.0	PAPOLG	Homo sapiens poly(A) polymerase gamma (PAPOLG), mRNA [NM_022894]	64895
A_23_P216742	1.0	-1.3	-1.0	-2.2	PAPPA	Homo sapiens pregnancy-associated plasma protein A, pappalysin 1 (PAPPA), mRNA [NM_002581]	5069

A_24_P940166	1.0	1.0	1.0	2.0	PAPSS2	Homo sapiens 3'-phosphoadenosine 5'-phosphosulfate synthase 2 (PAPSS2), transcript variant 2, mRNA [NM_001015880]	9060
A_23_P167276	-1.8	-2.1	-2.2	-1.7	PAQR3	Homo sapiens progestin and adipoQ receptor family member III (PAQR3), mRNA [NM_001040202]	152559
A_23_P337875	-1.1	-1.1	1.4	7.5	PAQR3	Homo sapiens progestin and adipoQ receptor family member III (PAQR3), mRNA [NM_001040202]	152559

A_23_P97283	5.3	7.7	3.9	5.0	PAQR6	Homo sapiens progestin and adipoQ receptor family member VI (PAQR6), transcript variant 1, mRNA [NM_024897]	79957
A_23_P410159	1.7	-1.6	-2.2	1.2	PAQR7	Homo sapiens progestin and adipoQ receptor family member VII (PAQR7), mRNA [NM_178422]	164091
A_32_P538180	1.6	2.2	1.5	1.4	PAQR7	Homo sapiens progestin and adipoQ receptor family member VII (PAQR7), mRNA [NM_178422]	164091

A_24_P334640	2.9	1.8	2.2	1.4	PAQR8	Homo sapiens progestin and adipoQ receptor family member VIII (PAQR8), mRNA [NM_133367]	85315
A_32_P196193	1.2	1.1	1.0	7.3	PAQR9	Homo sapiens progestin and adipoQ receptor family member IX (PAQR9), mRNA [NM_198504]	344838
A_23_P201963	1.3	1.5	-1.5	2.6	PARD3	Homo sapiens par-3 partitioning defective 3 homolog (C. elegans) (PARD3), transcript variant 1, mRNA [NM_019619]	56288

A_32_P83203	-2.7	-5.2	-2.5	1.0	PARD3B	Homo sapiens par-3 partitioning defective 3 homolog B (C. elegans) (PARD3B), mRNA [NM_205863]	117583
A_24_P34534	18.5	28.2	17.3	18.4	PARD3B	Homo sapiens par-3 partitioning defective 3 homolog B (C. elegans) (PARD3B), mRNA [NM_152526]	117583
A_24_P101248	-1.1	2.1	1.0	-1.0	PARD3B	Homo sapiens par-3 partitioning defective 3 homolog B (C. elegans) (PARD3B), mRNA [NM_152526]	117583

A_23_P140821	1.8	2.4	-1.5	1.1	PARD6A	Homo sapiens par-6 partitioning defective 6 homolog alpha (C. elegans) (PARD6A), transcript variant 1, mRNA [NM_016948]	50855
A_23_P147465	-1.2	1.4	-2.2	-1.6	PARK2	Homo sapiens parkinson protein 2, E3 ubiquitin protein ligase (parkin) (PARK2), transcript variant 1, mRNA [NM_004562]	5071
A_23_P74740	-3.5	-1.0	-1.1	1.0	PARK7	Homo sapiens parkinson protein 7 (PARK7), transcript variant 1, mRNA [NM_007262]	11315

A_24_P340853	1.8	1.8	2.2	1.4	PARP10	Homo sapiens poly (ADP-ribose) polymerase family, member 10 (PARP10), mRNA [NM_032789]	84875
A_24_P402779	2.8	4.9	3.7	3.6	PARP3	Homo sapiens poly (ADP-ribose) polymerase family, member 3 (PARP3), transcript variant 2, mRNA [NM_005485]	10039
A_32_P144342	-2.9	1.0	1.0	1.0	PARP4	Homo sapiens poly (ADP-ribose) polymerase family, member 4 (PARP4), mRNA [NM_006437]	143
A_23_P163258	1.5	1.3	1.3	2.2	PARP6	Homo sapiens poly (ADP-ribose) polymerase family, member 6 (PARP6), mRNA [NM_020214]	56965

A_23_P121898	3.9	3.8	1.6	2.0	PARP8	Homo sapiens poly (ADP-ribose) polymerase family, member 8 (PARP8), transcript variant 2, mRNA [NM_024615]	79668
A_23_P51291	-1.5	1.1	2.2	-2.0	PARS2	Homo sapiens prolyl-tRNA synthetase 2, mitochondrial (putative) (PARS2), nuclear gene encoding mitochondrial protein, mRNA [NM_152268]	25973

A_23_P335277	-1.0	-2.2	-1.0	-3.8	PART1	Homo sapiens prostate androgen-regulated transcript 1 (non-protein coding) (PART1), transcript variant 2, non-coding RNA [NR_028508]	25859
A_24_P124370	-1.1	-6.5	-1.1	-2.3	PARVA	Homo sapiens parvin, alpha (PARVA), mRNA [NM_018222]	55742
A_23_P47642	1.3	1.2	1.2	-2.3	PARVA	Homo sapiens parvin, alpha (PARVA), mRNA [NM_018222]	55742
A_23_P250413	2.1	1.2	1.0	-1.9	PARVG	Homo sapiens parvin, gamma (PARVG), transcript variant 1, mRNA [NM_022141]	64098

A_23_P408239	1.0	2.1	-1.1	1.4	PASK	Homo sapiens PAS domain containing serine/threonine kinase (PASK), transcript variant 5, mRNA [NM_001252124]	23178
A_23_P373079	2.9	2.8	2.2	1.9	PATL1	Homo sapiens protein associated with topoisomerase II homolog 1 (yeast) (PATL1), mRNA [NM_152716]	219988
A_23_P211459	-1.1	-2.5	-1.3	1.0	PATZ1	Homo sapiens POZ (BTB) and AT hook containing zinc finger 1 (PATZ1), transcript variant 1, mRNA [NM_014323]	23598
A_23_P6130	1.6	-2.8	-1.5	1.2	PAX1	Homo sapiens paired box 1 (PAX1), mRNA [NM_006192]	5075

A_23_P209499	-1.0	-1.0	-1.1	3.6	PAX3	Homo sapiens paired box 3 (PAX3), transcript variant PAX3D, mRNA [NM_181458]	5077
A_23_P414211	-2.6	1.8	-1.3	-1.2	PAX4	Homo sapiens paired box 4 (PAX4), mRNA [NM_006193]	5078
A_23_P59691	2.4	-1.1	1.1	1.2	PAX4	Homo sapiens paired-box transcription factor (PAX4) mRNA, complete cds. [AF043978]	5078
A_23_P1823	1.8	-1.1	-2.3	-2.4	PAX6	Homo sapiens paired box 6 (PAX6), transcript variant 1, mRNA [NM_000280]	5080

A_23_P500985	-8.9	1.2	-1.7	-1.5	PAX7	Homo sapiens paired box 7 (PAX7), transcript variant 2, mRNA [NM_013945]	5081
A_23_P126225	-1.3	1.2	-2.8	-1.7	PAX7	Homo sapiens paired box 7 (PAX7), transcript variant 2, mRNA [NM_013945]	5081
A_23_P210001	-2.6	3.0	2.4	2.9	PAX8	Homo sapiens paired box 8 (PAX8), transcript variant PAX8A, mRNA [NM_003466]	7849
A_32_P62997	1.4	1.3	1.0	3.5	PBK	Homo sapiens PDZ binding kinase (PBK), mRNA [NM_018492]	55872

A_24_P312519	-1.1	-2.3	-1.1	1.1	PBLD	Homo sapiens phenazine biosynthesis-like protein domain containing (PBLD), transcript variant 2, mRNA [NM_001033083]	64081
A_24_P112395	-3.8	1.6	1.4	-1.1	PBLD	Homo sapiens phenazine biosynthesis-like protein domain containing (PBLD), transcript variant 1, mRNA [NM_022129]	64081
A_24_P126484	-1.0	-1.0	-2.2	-1.9	PBLD	phenazine biosynthesis-like protein domain containing [Source:HGNC Symbol;Acc:23301] [ENST00000277795]	64081

A_23_P365874	1.0	-1.1	-2.2	-1.1	PBRM1	Homo sapiens polybromo 1 (PBRM1), transcript variant 2, mRNA [NM_018313]	55193
A_24_P247849	3.6	3.6	2.8	4.7	PBX1	pre-B-cell leukemia homeobox 1 [Source:HGNC Symbol;Acc:8632] [ENST00000474046]	5087
A_23_P62953	-2.5	1.6	-1.3	1.0	PBX1	Homo sapiens pre-B-cell leukemia homeobox 1 (PBX1), transcript variant 1, mRNA [NM_002585]	5087
A_23_P214658	-1.3	-4.3	-1.4	-1.4	PBX2	Homo sapiens pre-B-cell leukemia homeobox 2 (PBX2), mRNA [NM_002586]	5089

A_23_P367405	-1.3	-1.0	-1.2	-2.8	PCBD1	Homo sapiens pterin-4 alpha-carbinolamine dehydratase/dimerization cofactor of hepatocyte nuclear factor 1 alpha (PCBD1), mRNA [NM_000281]	5092
A_24_P141688	2.1	1.6	1.5	-2.3	PCBP2	Homo sapiens poly(rC) binding protein 2 (PCBP2), transcript variant 1, mRNA [NM_005016]	5094

A_23_P121051	1.0	1.0	-2.0	-1.5	PCCB	Homo sapiens propionyl CoA carboxylase, beta polypeptide (PCCB), nuclear gene encoding mitochondrial protein, transcript variant 1, mRNA [NM_000532]	5096
A_24_P302797	2.5	1.6	2.5	2.3	PCCB	Homo sapiens propionyl CoA carboxylase, beta polypeptide (PCCB), nuclear gene encoding mitochondrial protein, transcript variant 1, mRNA [NM_000532]	5096
A_23_P213359	1.9	1.3	2.3	3.3	PCDH1	Homo sapiens protocadherin 1 (PCDH1), transcript variant 1, mRNA [NM_002587]	5097

A_24_P234838	1.5	2.5	1.6	1.2	PCDH1	Homo sapiens protocadherin 1 (PCDH1), transcript variant 2, mRNA [NM_032420]	5097
A_23_P158851	-3.2	1.2	1.2	1.0	PCDH10	Homo sapiens protocadherin 10 (PCDH10), transcript variant 1, mRNA [NM_032961]	57575
A_23_P161331	1.0	-20.9	-1.0	1.1	PCDH15	Homo sapiens protocadherin-related 15 (PCDH15), transcript variant C, mRNA [NM_033056]	65217
A_24_P911906	1.3	1.3	-3.5	1.0	PCDH17	Homo sapiens protocadherin 17 (PCDH17), mRNA [NM_001040429]	27253

A_24_P419039	1.1	1.1	2.5	1.4	PCDH19	Homo sapiens protocadherin 19 (PCDH19), transcript variant 2, mRNA [NM_020766]	57526
A_24_P914638	-1.3	2.8	1.5	1.0	PCDH7	Homo sapiens protocadherin 7 (PCDH7), transcript variant d, mRNA [NM_001173523]	5099
A_23_P378364	1.4	-1.1	-1.6	-2.1	PCDH7	Homo sapiens protocadherin 7 (PCDH7), transcript variant c, mRNA [NM_032457]	5099
A_23_P420236	-1.0	1.1	-1.1	-2.2	PCDH9	Homo sapiens protocadherin 9 (PCDH9), transcript variant 1, mRNA [NM_203487]	5101

A_24_P187218	-9.1	-1.0	-1.0	-1.6	PCDH9	Homo sapiens protocadherin 9 (PCDH9), transcript variant 2, mRNA [NM_020403]	5101
A_24_P360206	2.2	-1.9	-1.4	-1.4	PCDHA11	Homo sapiens protocadherin alpha 11 (PCDHA11), transcript variant 1, mRNA [NM_018902]	56138
A_23_P325498	3.9	14.6	5.7	8.6	PCDHA8	Homo sapiens protocadherin alpha 8 (PCDHA8), transcript variant 2, mRNA [NM_031856]	56140
A_23_P110462	1.0	-1.3	2.2	1.1	PCDHB1	Homo sapiens protocadherin beta 1 (PCDHB1), mRNA [NM_013340]	29930

A_23_P7397	1.0	-1.8	-3.0	1.0	PCDHB10	Homo sapiens protocadherin beta 10 (PCDHB10), mRNA [NM_018930]	56126
A_23_P167401	1.2	1.9	3.1	2.0	PCDHB11	Homo sapiens protocadherin beta 11 (PCDHB11), mRNA [NM_018931]	56125
A_23_P156010	-1.0	1.1	-4.4	-1.0	PCDHB12	Homo sapiens protocadherin beta 12 (PCDHB12), mRNA [NM_018932]	56124
A_23_P30200	-1.0	-6.4	-1.1	1.2	PCDHB16	Homo sapiens protocadherin beta 16 (PCDHB16), mRNA [NM_020957]	57717
A_24_P243373	-1.5	-1.6	-2.6	1.4	PCDHB7	Homo sapiens protocadherin beta 7 (PCDHB7), mRNA [NM_018940]	56129

A_24_P380284	-2.4	-1.7	-6.6	-2.3	PCDHB9	Homo sapiens protocadherin beta 9 (PCDHB9), mRNA [NM_019119]	56127
A_23_P422044	-10.8	1.6	1.1	1.8	PCDHGA2	Homo sapiens protocadherin gamma subfamily A, 2 (PCDHGA2), transcript variant 2, mRNA [NM_032009]	56113
A_23_P435444	1.4	1.5	-19.6	-1.4	PCDHGA7	Homo sapiens protocadherin gamma subfamily A, 7 (PCDHGA7), transcript variant 2, mRNA [NM_032087]	56108

A_23_P364414	-2.5	-1.0	-2.1	-1.1	PCDHGA9	Homo sapiens protocadherin gamma subfamily A, 9 (PCDHGA9), transcript variant 2, mRNA [NM_032089]	56107
A_23_P395418	-2.5	1.1	-1.0	-1.0	PCDHGB3	Homo sapiens protocadherin gamma subfamily B, 3 (PCDHGB3), transcript variant 2, mRNA [NM_032097]	56102
A_24_P177531	-1.0	-1.0	-5.5	-1.1	PCDHGB8P	Homo sapiens protocadherin gamma subfamily B, 8 pseudogene (PCDHGB8P), non-coding RNA [NR_001297]	56120

A_24_P932479	1.2	1.2	1.1	-3.7	PCF11	PCF11, cleavage and polyadenylation factor subunit, homolog (S. cerevisiae) [Source:HGNC Symbol;Acc:30097] [ENST00000298281]	51585
A_23_P348323	-1.4	-2.2	-3.1	-2.3	PCGF2	Homo sapiens polycomb group ring finger 2 (PCGF2), mRNA [NM_007144]	7703
A_23_P21346	1.1	-2.1	-1.0	-1.0	PCGF3	Homo sapiens polycomb group ring finger 3 (PCGF3), mRNA [NM_006315]	10336
A_23_P210496	2.8	1.7	1.1	2.8	PCIF1	Homo sapiens PDX1 C-terminal inhibiting factor 1 (PCIF1), mRNA [NM_022104]	63935

A_23_P210623	-1.4	1.2	-10.9	-1.2	PCK1	Homo sapiens phosphoenolpyruvate carboxykinase 1 (soluble) (PCK1), mRNA [NM_002591]	5105
A_24_P23636	1.2	-4.7	-2.6	-2.7	PCLO	Homo sapiens piccolo (presynaptic cytomatrix protein) (PCLO), transcript variant 2, mRNA [NM_014510]	27445
A_23_P70983	1.1	1.3	-2.3	1.1	PCLO	Homo sapiens piccolo (presynaptic cytomatrix protein) (PCLO), transcript variant 2, mRNA [NM_014510]	27445

A_24_P247902	3.1	2.7	3.0	1.3	PCLO	Homo sapiens piccolo (presynaptic cytomatrix protein) (PCLO), transcript variant 2, mRNA [NM_014510]	27445
A_24_P283320	-2.0	-2.7	-2.2	-3.3	PCMT1	Homo sapiens protein-L-isoaspartate (D-aspartate) O-methyltransferase (PCMT1), transcript variant 1, mRNA [NM_005389]	5110
A_24_P140827	1.0	1.0	1.0	-2.3	PCMT1	Homo sapiens protein-L-isoaspartate (D-aspartate) O-methyltransferase (PCMT1), transcript variant 1, mRNA [NM_005389]	5110

A_32_P203500	-1.7	-1.2	-5.9	-3.1	PCMTD1	Homo sapiens protein-L-isoaspartate (D-aspartate) O-methyltransferase domain containing 1 (PCMTD1), mRNA [NM_052937]	115294
A_24_P110967	1.1	-1.8	-3.2	1.1	PCMTD1	Homo sapiens protein-L-isoaspartate (D-aspartate) O-methyltransferase domain containing 1 (PCMTD1), mRNA [NM_052937]	115294

A_24_P537188	8.2	3.4	2.1	5.3	PCMTD2	protein-L-isoaspartate (D-aspartate) O-methyltransferase domain containing 2 [Source:HGNC Symbol;Acc:15882] [ENST00000299468]	55251
A_24_P876772	2.3	2.6	2.9	2.1	PCMTD2	protein-L-isoaspartate (D-aspartate) O-methyltransferase domain containing 2 [Source:HGNC Symbol;Acc:15882] [ENST00000299468]	55251
A_23_P37311	2.4	2.5	2.4	1.4	PCNX	Homo sapiens pecanex homolog (Drosophila) (PCNX), mRNA [NM_014982]	22990

A_24_P225189	-2.8	-1.0	-2.5	-1.1	PCNXL2	Homo sapiens pecanex-like 2 (Drosophila) (PCNXL2), transcript variant 1, mRNA [NM_014801]	80003
A_24_P924202	1.4	3.8	1.6	-1.8	PCNXL3	Homo sapiens pecanex-like 3 (Drosophila) (PCNXL3), mRNA [NM_032223]	399909
A_24_P83961	1.0	1.3	-3.8	1.2	PCNXL3	Homo sapiens pecanex-like 3 (Drosophila) (PCNXL3), mRNA [NM_032223]	399909
A_24_P399500	2.0	2.4	1.5	2.1	PCP2	Homo sapiens Purkinje cell protein 2 (PCP2), mRNA [NM_174895]	126006

A_23_P109322	-2.7	-1.0	-1.1	-2.9	PCP4	Homo sapiens Purkinje cell protein 4 (PCP4), mRNA [NM_006198]	5121
A_32_P214665	1.7	2.2	-2.2	1.4	PCP4L1	Homo sapiens Purkinje cell protein 4 like 1 (PCP4L1), mRNA [NM_001102566]	654790
A_24_P14731	2.3	-1.7	-1.7	-1.5	PCSK1N	Homo sapiens proprotein convertase subtilisin/kexin type 1 inhibitor (PCSK1N), mRNA [NM_013271]	27344
A_23_P79968	-2.4	-1.1	-1.1	-1.0	PCSK2	Homo sapiens proprotein convertase subtilisin/kexin type 2 (PCSK2), transcript variant 1, mRNA [NM_002594]	5126

A_23_P16648	-2.3	-1.3	-1.1	-1.1	PCSK4	Homo sapiens proprotein convertase subtilisin/kexin type 4 (PCSK4), mRNA [NM_017573]	54760
A_24_P355246	-1.1	1.2	-4.9	-2.1	PCSK5	proprotein convertase subtilisin/kexin type 5 [Source:HGNC Symbol;Acc:8747] [ENST00000376752]	5125
A_32_P66974	-1.3	-1.5	-1.5	-5.3	PCSK7	Homo sapiens proprotein convertase subtilisin/kexin type 7 (PCSK7), mRNA [NM_004716]	9159

A_23_P203095	1.9	-7.1	1.3	1.0	PCSK7	Homo sapiens proprotein convertase subtilisin/kexin type 7 (PCSK7), mRNA [NM_004716]	9159
A_23_P64214	-2.0	-1.5	-2.4	-1.1	PCSK7	Homo sapiens proprotein convertase subtilisin/kexin type 7 (PCSK7), mRNA [NM_004716]	9159
A_24_P398370	-2.0	-1.8	-2.6	1.0	PCSK7	proprotein convertase subtilisin/kexin type 7 [Source:HGNC Symbol;Acc:8748] [ENST00000320934]	9159
A_23_P56640	1.0	-1.6	-4.2	-1.1	PCYOX1	Homo sapiens prenylcysteine oxidase 1 (PCYOX1), mRNA [NM_016297]	51449

A_23_P30275	2.0	1.1	1.2	1.0	PCYOX1L	Homo sapiens prenylcysteine oxidase 1 like (PCYOX1L), mRNA [NM_024028]	78991
A_23_P252681	-2.2	-1.6	-2.8	-2.2	PCYT1A	Homo sapiens phosphate cytidylyltransferase 1, choline, alpha (PCYT1A), mRNA [NM_005017]	5130
A_23_P148422	-1.8	-1.0	-3.6	-1.1	PCYT1B	Homo sapiens phosphate cytidylyltransferase 1, choline, beta (PCYT1B), transcript variant 1, mRNA [NM_004845]	9468

A_24_P404245	1.5	1.0	2.3	1.1	PCYT2	Homo sapiens phosphate cytidylyltr ansferase 2, ethanolam ine (PCYT2), transcript variant 2, mRNA [NM_0028 61]	5833
A_23_P123256	-1.0	-8.3	-1.2	-1.1	PDAP1	Homo sapiens PDGFA associated protein 1 (PDAP1), mRNA [NM_0148 91]	11333
A_23_P23090	-1.0	-7.1	-1.0	-1.0	PDC	Homo sapiens phosducin (PDC), transcript variant 1, mRNA [NM_0025 97]	5132
A_23_P136405	2.1	2.9	1.1	3.7	PDCD1	Homo sapiens programm ed cell death 1 (PDCD1), mRNA [NM_0050 18]	5133

A_23_P161257	-2.3	-2.0	-2.1	-2.3	PDCD11	programmed cell death 11 [Source:HGNC Symbol;Acc:13408] [ENST00000369797]	22984
A_23_P145146	1.1	-1.4	1.2	2.6	PDCD2	Homo sapiens programmed cell death 2 (PDCD2), transcript variant 1, mRNA [NM_002598]	5134
A_23_P27947	1.1	-2.0	-1.1	-1.0	PDCD2L	Homo sapiens programmed cell death 2-like (PDCD2L), mRNA [NM_032346]	84306
A_24_P339611	-1.3	-1.5	-4.1	-3.3	PDCD5	Homo sapiens programmed cell death 5 (PDCD5), mRNA [NM_004708]	9141

A_32_P22338	-2.4	-3.2	-1.1	-1.1	PDCD6IP	Homo sapiens programmed cell death 6 interacting protein (PDCD6IP), transcript variant 1, mRNA [NM_013374]	10015
A_32_P157531	-1.3	-2.4	-6.3	-1.2	PDCL3	Homo sapiens phosducin-like 3 (PDCL3), mRNA [NM_024065]	79031
A_23_P431543	-1.5	1.1	-1.8	-2.0	PDDC1	Homo sapiens Parkinson disease 7 domain containing 1 (PDDC1), mRNA [NM_182612]	347862
A_23_P359904	-4.5	1.8	2.5	-1.1	PDDC1	Homo sapiens Parkinson disease 7 domain containing 1 (PDDC1), mRNA [NM_182612]	347862

A_23_P394367	-1.4	1.1	-3.6	1.2	PDE11A	Homo sapiens phosphodiesterase 11A (PDE11A), transcript variant 4, mRNA [NM_016953]	50940
A_24_P43144	2.1	2.7	-1.1	1.5	PDE11A	Homo sapiens phosphodiesterase 11A (PDE11A), transcript variant 2, mRNA [NM_001077358]	50940
A_24_P230708	1.3	1.7	1.4	3.5	PDE12	Homo sapiens phosphodiesterase 12 (PDE12), mRNA [NM_177966]	201626
A_24_P102082	-1.5	-2.9	-1.7	-1.8	PDE1A	phosphodiesterase 1A, calmodulin-dependent [Source:HGNC Symbol;Acc:8774] [ENST00000409365]	5136

A_23_P139585	1.4	-2.1	-4.2	-1.7	PDE1B	Homo sapiens phosphodiesterase 1B, calmodulin-dependent (PDE1B), transcript variant 1, mRNA [NM_000924]	5153
A_23_P87379	-6.2	-1.5	-1.6	-1.5	PDE2A	Homo sapiens phosphodiesterase 2A, cGMP-stimulated (PDE2A), transcript variant 1, mRNA [NM_002599]	5138
A_23_P116114	2.4	1.2	3.1	2.4	PDE3B	Homo sapiens phosphodiesterase 3B, cGMP-inhibited (PDE3B), mRNA [NM_000922]	5140

A_23_P208334	-1.0	-1.0	-2.5	1.2	PDE4A	Homo sapiens phosphodiesterase 4A, cAMP-specific (PDE4A), transcript variant 4, mRNA [NM_006202]	5141
A_24_P322474	2.1	2.1	2.0	1.6	PDE4A	Homo sapiens phosphodiesterase 4A, cAMP-specific (PDE4A), transcript variant 4, mRNA [NM_006202]	5141
A_23_P74278	-2.2	-3.8	-1.8	-1.0	PDE4B	Homo sapiens phosphodiesterase 4B, cAMP-specific (PDE4B), transcript variant d, mRNA [NM_001037341]	5142
A_24_P921129	-1.0	-11.3	-1.1	-1.1	PDE4B	Homo sapiens phosphodiesterase 4B, cAMP-specific (PDE4B), transcript variant b, mRNA [NM_001037339]	5142

A_24_P139120	2.5	3.4	2.4	2.0	PDE4C	Homo sapiens phosphodiesterase 4C, cAMP-specific (PDE4C), transcript variant 1, mRNA [NM_000923]	5143
A_24_P419351	-3.8	1.0	-1.0	-1.0	PDE4D	Homo sapiens phosphodiesterase 4D, cAMP-specific (PDE4D), transcript variant 2, mRNA [NM_006203]	5144
A_24_P944519	-3.1	-1.9	-6.2	-1.9	PDE4D	Homo sapiens phosphodiesterase 4D, cAMP-specific (PDE4D), transcript variant 2, mRNA [NM_006203]	5144
A_23_P124456	-11.0	-1.1	-1.1	-1.1	PDE4D	Homo sapiens phosphodiesterase 4D, cAMP-specific (PDE4D), transcript variant 2, mRNA [NM_006203]	5144

A_23_P149153	-1.0	-1.0	1.1	-5.7	PDE4DIP	Homo sapiens phosphodiesterase 4D interacting protein (PDE4DIP), transcript variant 3, mRNA [NM_022359]	9659
A_32_P44840	-16.9	1.0	-1.5	-1.0	PDE4DIP	Homo sapiens mRNA, similar to rat myomegalin, complete cds. [AB042555]	9659
A_23_P22143	-2.9	1.1	1.1	1.1	PDE6B	Homo sapiens phosphodiesterase 6B, cGMP-specific, rod, beta (PDE6B), transcript variant 1, mRNA [NM_000283]	5158

A_23_P98070	-2.0	1.0	-1.0	-2.4	PDE6C	Homo sapiens phosphodiesterase 6C, cGMP-specific, cone, alpha prime (PDE6C), mRNA [NM_006204]	5146
A_23_P95788	1.0	-6.8	1.1	-1.0	PDE6G	Homo sapiens phosphodiesterase 6G, cGMP-specific, rod, gamma (PDE6G), transcript variant 1, mRNA [NM_002602]	5148
A_23_P123478	2.0	2.9	2.1	2.8	PDE7A	Homo sapiens phosphodiesterase 7A (PDE7A), transcript variant 1, mRNA [NM_002603]	5150

A_23_P394154	-5.5	-4.8	-1.0	-1.0	PDE8A	Homo sapiens phosphodiesterase 8A (PDE8A), transcript variant 1, mRNA [NM_002605]	5151
A_24_P339944	-1.8	-1.9	-4.1	-6.0	PDGFB	Homo sapiens platelet-derived growth factor beta polypeptide (PDGFB), transcript variant 1, mRNA [NM_002608]	5155
A_24_P124349	1.8	-2.6	1.7	2.0	PDGFD	Homo sapiens platelet derived growth factor D (PDGFD), transcript variant 1, mRNA [NM_025208]	80310

A_23_P300033	-8.5	3.4	3.6	1.0	PDGFRA	Homo sapiens platelet-derived growth factor receptor, alpha polypeptide (PDGFRA), mRNA [NM_006206]	5156
A_23_P81018	-4.2	1.0	1.0	1.2	PDHA2	Homo sapiens pyruvate dehydrogenase (lipoamide) alpha 2 (PDHA2), mRNA [NM_005390]	5161
A_23_P325642	1.0	-1.0	-7.3	-1.0	PDIA2	Homo sapiens protein disulfide isomerase family A, member 2 (PDIA2), mRNA [NM_006849]	64714
A_32_P63182	-8.6	-1.4	-1.4	-2.4	PDIA3	Homo sapiens protein disulfide isomerase family A, member 3 (PDIA3), mRNA [NM_005313]	2923

A_23_P42802	-5.9	1.4	-4.2	-1.8	PDIA4	Homo sapiens protein disulfide isomerase family A, member 4 (PDIA4), mRNA [NM_004911]	9601
A_23_P10614	1.5	2.1	2.1	1.6	PDK1	Homo sapiens pyruvate dehydrogenase kinase, isozyme 1 (PDK1), nuclear gene encoding mitochondrial protein, mRNA [NM_002610]	5163
A_23_P207517	-2.4	-2.3	1.4	-1.6	PDK2	Homo sapiens pyruvate dehydrogenase kinase, isozyme 2 (PDK2), nuclear gene encoding mitochondrial protein, transcript variant 1, mRNA [NM_002611]	5164

A_24_P941666	-1.3	-2.5	-1.4	-2.1	PDK3	Homo sapiens pyruvate dehydrogenase kinase, isozyme 3 (PDK3), nuclear gene encoding mitochondrial protein, transcript variant 1, mRNA [NM_001142386]	5165
A_24_P340112	-1.0	-5.5	-1.2	-2.8	PDK3	Homo sapiens pyruvate dehydrogenase kinase, isozyme 3 (PDK3), nuclear gene encoding mitochondrial protein, transcript variant 1, mRNA [NM_001142386]	5165
A_23_P149992	-2.2	1.3	1.2	-1.9	PDLIM1	Homo sapiens PDZ and LIM domain 1 (PDLIM1), mRNA [NM_020992]	9124

A_23_P20285	2.4	2.3	2.5	-1.1	PDLIM2	Homo sapiens PDZ and LIM domain 2 (mystique) (PDLIM2), transcript variant 2, mRNA [NM_021630]	64236
A_23_P144796	-1.2	1.2	-2.1	2.1	PDLIM4	Homo sapiens PDZ and LIM domain 4 (PDLIM4), transcript variant 1, mRNA [NM_003687]	8572
A_24_P24263	-1.5	2.0	1.8	-3.5	PDLIM5	Homo sapiens PDZ and LIM domain 5 (PDLIM5), transcript variant 4, mRNA [NM_001011515]	10611
A_24_P174641	-1.0	-1.0	-27.9	-1.1	PDLIM5	Homo sapiens PDZ and LIM domain 5 (PDLIM5), transcript variant 1, mRNA [NM_006457]	10611

A_24_P41882	-2.7	-1.8	-3.5	-1.8	PDLIM7	Homo sapiens PDZ and LIM domain 7 (enigma) (PDLIM7), transcript variant 4, mRNA [NM_213636]	9260
A_23_P144896	6.0	4.5	2.2	-1.7	PDLIM7	Homo sapiens PDZ and LIM domain 7 (enigma) (PDLIM7), transcript variant 1, mRNA [NM_005451]	9260
A_23_P66219	1.5	-3.1	-1.9	-1.3	PDPK1	Homo sapiens 3-phosphoinositide dependent protein kinase-1 (PDPK1), transcript variant 1, mRNA [NM_002613]	5170
A_23_P430068	-4.7	-3.0	-1.1	1.5	PDPN	Homo sapiens podoplanin (PDPN), transcript variant 1, mRNA [NM_006474]	10630

A_23_P166159	-1.1	-1.6	-1.7	-2.1	PDRG1	Homo sapiens p53 and DNA-damage regulated 1 (PDRG1), mRNA [NM_030815]	81572
A_24_P186216	1.1	-3.4	1.0	-1.6	PDS5A	Homo sapiens PDS5, regulator of cohesion maintenance, homolog A (S. cerevisiae) (PDS5A), transcript variant 3, mRNA [NM_001100400]	23244
A_24_P52004	6.6	1.8	3.5	2.4	PDS5A	Homo sapiens PDS5, regulator of cohesion maintenance, homolog A (S. cerevisiae) (PDS5A), transcript variant 1, mRNA [NM_001100399]	23244

A_23_P48387	1.6	3.6	1.4	1.3	PDS5B	Homo sapiens PDS5, regulator of cohesion maintenance, homolog B (S. cerevisiae) (PDS5B), mRNA [NM_015032]	23047
A_23_P205098	2.3	-1.0	-1.1	-1.1	PDS5B	Homo sapiens PDS5, regulator of cohesion maintenance, homolog B (S. cerevisiae) (PDS5B), mRNA [NM_015032]	23047
A_23_P161152	-4.9	-2.0	-2.1	-1.4	PDSS1	Homo sapiens prenyl (decaprenyl) diphosphate synthase, subunit 1 (PDSS1), mRNA [NM_014317]	23590

A_24_P160088	1.4	-1.6	1.3	-2.1	PDXK	Homo sapiens pyridoxal (pyridoxine, vitamin B6) kinase (PDXK), mRNA [NM_003681]	8566
A_24_P65949	4.6	5.0	3.7	1.4	PDXK	Homo sapiens cDNA FLJ37311 fis, clone BRAMY2016771. [AK094630]	8566
A_24_P280029	-3.3	1.9	1.8	1.0	PDXP	Homo sapiens pyridoxal (pyridoxine, vitamin B6) phosphatase (PDXP), mRNA [NM_020315]	57026
A_23_P7402	2.4	-1.9	1.2	1.1	PDZD2	Homo sapiens PDZ domain containing 2 (PDZD2), mRNA [NM_178140]	23037

A_23_P24577	1.3	2.0	1.7	1.1	PDZD3	PDZ domain containing 3 [Source:HGNC Symbol;Acc:19891] [ENST00000529098]	79849
A_24_P265088	1.0	-1.9	-2.0	-1.7	PDZD4	Homo sapiens PDZ domain containing 4 (PDZD4), mRNA [NM_032512]	57595
A_23_P75088	-5.5	-3.1	-1.3	1.1	PDZD7	Homo sapiens PDZ domain containing 7 (PDZD7), transcript variant 2, mRNA [NM_024895]	79955
A_24_P47211	1.0	-2.1	1.0	-1.0	PDZD8	PDZ domain containing 8 [Source:HGNC Symbol;Acc:26974] [ENST00000334464]	118987

A_23_P415154	1.1	-3.3	-1.1	-1.0	PDZRN4	Homo sapiens PDZ domain containing ring finger 4 (PDZRN4), transcript variant 2, mRNA [NM_013377]	29951
A_23_P105651	2.4	-1.8	2.1	-1.0	PDZRN4	Homo sapiens PDZ domain containing ring finger 4 (PDZRN4), transcript variant 2, mRNA [NM_013377]	29951
A_24_P410952	-1.3	-2.4	1.0	-2.0	PEA15	Homo sapiens phosphoprotein enriched in astrocytes 15 (PEA15), mRNA [NM_003768]	8682

A_24_P323901	6.9	9.5	7.8	2.6	PEAR1	Homo sapiens platelet endothelial aggregation receptor 1 (PEAR1), mRNA [NM_001080471]	375033
A_24_P385326	-1.9	1.5	-3.2	1.6	PEAR1	Homo sapiens platelet endothelial aggregation receptor 1 (PEAR1), mRNA [NM_001080471]	375033
A_23_P13604	-3.0	1.8	1.7	1.0	PEBP1	Homo sapiens phosphatidylethanolamine binding protein 1 (PEBP1), mRNA [NM_002567]	5037
A_23_P313	-2.0	-2.1	-2.0	-2.0	PEF1	Homo sapiens penta-EF-hand domain containing 1 (PEF1), transcript variant 1, mRNA [NM_012392]	553115

A_23_P104692	-1.2	1.5	-2.1	-1.0	PELI3	Homo sapiens pellino homolog 3 (Drosophila) (PELI3), transcript variant 1, mRNA [NM_145065]	246330
A_24_P66239	2.0	-1.5	1.9	1.4	PELO	Homo sapiens PRO1770 mRNA, complete cds. [AF118075]	53918
A_23_P163955	1.6	2.3	-1.2	-1.1	PEMT	Homo sapiens phosphatidylethanolamine N-methyltransferase (PEMT), nuclear gene encoding mitochondrial protein, transcript variant 2, mRNA [NM_007169]	10400

A_23_P89589	-1.1	-1.5	-2.3	-3.3	PER1	Homo sapiens period homolog 1 (Drosophila) (PER1), mRNA [NM_002616]	5187
A_23_P411162	-11.1	1.1	1.4	1.1	PER2	Homo sapiens period homolog 2 (Drosophila) (PER2), mRNA [NM_022817]	8864
A_23_P334555	1.1	-4.0	-1.4	-1.2	PES1	Homo sapiens pescadillo homolog 1, containing BRCT domain (zebrafish) (PES1), transcript variant 1, mRNA [NM_014303]	23481
A_23_P20045	-5.9	-1.9	-8.5	-2.1	PEX1	Homo sapiens peroxisomal biogenesis factor 1 (PEX1), mRNA [NM_000466]	5189

A_23_P501745	-10.5	1.4	-1.2	-1.1	PEX10	Homo sapiens peroxisomal biogenesis factor 10 (PEX10), transcript variant 1, mRNA [NM_153818]	5192
A_24_P173727	1.4	2.8	1.3	-1.2	PEX10	Homo sapiens peroxisomal biogenesis factor 10 (PEX10), transcript variant 2, mRNA [NM_002617]	5192
A_23_P321466	4.7	2.8	3.3	-2.0	PEX11G	Homo sapiens peroxisomal biogenesis factor 11 gamma (PEX11G), mRNA [NM_080662]	92960
A_23_P257131	-1.2	-1.0	-1.1	2.1	PEX13	Homo sapiens peroxisomal biogenesis factor 13 (PEX13), mRNA [NM_002618]	5194

A_23_P160188	-2.2	-1.1	-13.3	-1.2	PEX19	Homo sapiens peroxisomal biogenesis factor 19 (PEX19), transcript variant 1, mRNA [NM_002857]	5824
A_32_P222374	-2.5	1.3	1.4	1.1	PEX5L-AS2	PREDICTED: Homo sapiens hypothetical LOC100505598 (LOC100505598), miscRNA [XR_108517]	100505598
A_32_P41127	-4.8	1.3	-2.0	1.1	PFDN1	Homo sapiens prefoldin subunit 1 (PFDN1), mRNA [NM_002622]	5201
A_23_P51906	-2.2	-1.7	-2.7	-1.5	PFDN2	Homo sapiens prefoldin subunit 2 (PFDN2), mRNA [NM_012394]	5202

A_24_P203106	9.1	6.8	4.2	4.7	PFKFB1	Homo sapiens 6-phosphofructo-2-kinase/fructose-2,6-biphosphatase 1 (PFKFB1), mRNA [NM_002625]	5207
A_23_P201508	2.0	1.4	1.6	1.5	PFKFB2	Homo sapiens 6-phosphofructo-2-kinase/fructose-2,6-biphosphatase 2 (PFKFB2), transcript variant 1, mRNA [NM_006212]	5208
A_24_P111096	1.0	-1.7	-1.1	-2.0	PFKFB3	Homo sapiens 6-phosphofructo-2-kinase/fructose-2,6-biphosphatase 3 (PFKFB3), transcript variant 1, mRNA [NM_004566]	5209

A_24_P206604	-1.8	-2.4	-3.6	-2.0	PFKFB3	Homo sapiens 6-phosphofructo-2-kinase/fructose-2,6-biphosphatase 3 (PFKFB3), transcript variant 1, mRNA [NM_004566]	5209
A_23_P29081	-1.8	1.5	1.7	2.1	PFKL	Homo sapiens phosphofructokinase, liver (PFKL), transcript variant 2, mRNA [NM_002626]	5211
A_23_P29079	1.7	1.1	1.0	3.0	PFKL	Homo sapiens phosphofructokinase, liver (PFKL), transcript variant 2, mRNA [NM_002626]	5211
A_23_P46928	1.0	2.0	-1.1	1.2	PFKP	Homo sapiens phosphofructokinase, platelet (PFKP), transcript variant 1, mRNA [NM_002627]	5214

A_23_P27096	-2.0	1.1	-2.2	1.2	PFN1	Homo sapiens profilin 1 (PFN1), mRNA [NM_005022]	5216
A_32_P234604	-2.2	-1.0	-2.4	1.2	PFN1	Homo sapiens profilin 1 (PFN1), mRNA [NM_005022]	5216
A_23_P253301	-2.2	2.4	2.3	2.5	PFN2	Homo sapiens profilin 2 (PFN2), transcript variant 1, mRNA [NM_053024]	5217
A_23_P150547	9.1	6.5	4.7	5.7	PGA3	Homo sapiens pepsinogen 3, group I (pepsinogen A) (PGA3), mRNA [NM_001079807]	643834
A_23_P150549	1.0	-2.3	2.1	-1.0	PGA3	Homo sapiens pepsinogen 3, group I (pepsinogen A) (PGA3), mRNA [NM_001079807]	643834

A_23_P93940	-2.4	1.8	-1.9	-7.4	PGAM2	Homo sapiens phosphoglycerate mutase 2 (muscle) (PGAM2), mRNA [NM_000290]	5224
A_23_P319719	-1.5	-2.2	-2.2	1.3	PGAM5	Homo sapiens phosphoglycerate mutase family member 5 (PGAM5), nuclear gene encoding mitochondrial protein, transcript variant 3, mRNA [NM_138575]	192111
A_23_P79247	-1.5	2.4	2.3	-2.7	PGAP1	Homo sapiens post-GPI attachment to proteins 1 (PGAP1), mRNA [NM_024989]	80055

A_24_P275828	-1.9	-2.2	-2.1	-2.1	PGAP3	Homo sapiens post-GPI attachment to proteins 3 (PGAP3), mRNA [NM_033419]	93210
A_23_P319565	2.1	2.0	2.1	2.0	PGBD3	Homo sapiens piggyBac transposable element derived 3 (PGBD3), mRNA [NM_170753]	267004
A_23_P7961	2.3	-1.2	1.2	-1.4	PGC	Homo sapiens progastricsin (pepsinogen C) (PGC), transcript variant 1, mRNA [NM_002630]	5225
A_23_P76992	-3.0	1.9	-1.8	-2.3	PGF	Homo sapiens placental growth factor (PGF), transcript variant 1, mRNA [NM_002632]	5228

A_24_P254532	-1.1	-4.3	-1.2	-1.2	PGK1	Homo sapiens phosphoglycerate kinase 1 (PGK1), mRNA [NM_000291]	5230
A_24_P119774	1.1	-6.6	1.0	1.1	PGK1	phosphoglycerate kinase 1 [Source:HGNC Symbol;Acc:8896] [ENST00000474281]	5230
A_32_P14212	1.0	-1.0	-1.1	-2.3	PGK1	phosphoglycerate kinase 1 [Source:HGNC Symbol;Acc:8896] [ENST00000373316]	5230
A_24_P920994	1.2	1.6	-1.3	-2.4	PGLS	Homo sapiens 6-phosphogluconolactonase, mRNA (cDNA clone IMAGE:4395142), with apparent retained intron. [BC017706]	25796

A_23_P396765	1.0	-4.2	1.0	1.0	PGM2L1	Homo sapiens phosphoglucomutase 2-like 1 (PGM2L1), mRNA [NM_173582]	283209
A_32_P122703	1.5	-3.2	1.0	1.2	PGM2L1	Homo sapiens phosphoglucomutase 2-like 1 (PGM2L1), mRNA [NM_173582]	283209
A_23_P10956	1.1	1.1	-1.0	-4.0	PGM5	Homo sapiens phosphoglucomutase 5 (PGM5), mRNA [NM_021965]	5239
A_24_P254949	3.0	2.1	3.3	1.1	PGM5	Homo sapiens phosphoglucomutase 5 (PGM5), mRNA [NM_021965]	5239
A_24_P942805	21.0	5.1	9.0	6.5	PGP	Homo sapiens phosphoglycolate phosphatase (PGP), mRNA [NM_001042371]	283871

A_23_P22854	1.3	3.3	4.3	1.1	PGPEP1	Homo sapiens pyroglutamyl-peptidase I (PGPEP1), mRNA [NM_017712]	54858
A_23_P155868	2.6	1.5	1.7	1.1	PGRMC2	Homo sapiens progesterone receptor membrane component 2 (PGRMC2), mRNA [NM_006320]	10424
A_24_P940288	-4.2	-8.5	-1.0	1.0	PGS1	Homo sapiens mRNA; cDNA DKFZp762M186 (from clone DKFZp762M186). [AL359590]	9489

A_32_P52018	1.0	-3.8	1.1	-1.6	PHACTR1	Homo sapiens phosphatase and actin regulator 1 (PHACTR1), transcript variant 1, mRNA [NM_030948]	221692
A_32_P88626	2.4	3.1	3.5	2.1	PHACTR4	Homo sapiens phosphatase and actin regulator 4 (PHACTR4), transcript variant 1, mRNA [NM_001048183]	65979
A_23_P130040	-1.1	-4.3	-1.1	1.2	PHB	Homo sapiens prohibitin (PHB), mRNA [NM_002634]	5245
A_24_P854199	2.5	1.2	2.4	4.3	PHB	Homo sapiens prohibitin (PHB), mRNA [NM_002634]	5245

A_24_P265254	2.0	2.1	1.9	2.2	PHC2	Homo sapiens polyhomeotic homolog 2 (Drosophila) (PHC2), transcript variant 1, mRNA [NM_198040]	1912
A_23_P34510	1.2	1.1	1.6	2.3	PHC2	Homo sapiens polyhomeotic homolog 2 (Drosophila) (PHC2), transcript variant 1, mRNA [NM_198040]	1912
A_24_P179175	1.3	-3.1	1.3	-1.8	PHC3	polyhomeotic homolog 3 (Drosophila) [Source:HGNC Symbol;Acc:15682] [ENST00000474275]	80012

A_23_P409988	4.9	5.5	5.5	1.0	PHC3	Homo sapiens polyhomeotic homolog 3 (Drosophila) (PHC3), mRNA [NM_024947]	80012
A_23_P503200	7.3	6.0	1.6	1.6	PHF10	Homo sapiens PHD finger protein 10 (PHF10), transcript variant 1, mRNA [NM_018288]	55274
A_32_P58425	-11.8	1.0	1.5	-2.1	PHF13	Homo sapiens PHD finger protein 13 (PHF13), mRNA [NM_153812]	148479
A_23_P156198	-17.4	1.0	-1.2	-1.2	PHF15	Homo sapiens PHD finger protein 15 (PHF15), mRNA [NM_015288]	23338

A_23_P167256	-1.4	-3.6	-1.0	-1.0	PHF17	Homo sapiens PHD finger protein 17 (PHF17), transcript variant L, mRNA [NM_199320]	79960
A_23_P410587	-1.0	-1.0	-4.2	-1.0	PHF17	Homo sapiens PHD finger protein 17 (PHF17), transcript variant S, mRNA [NM_024900]	79960
A_23_P21436	1.8	2.0	1.9	-1.1	PHF19	Homo sapiens PHD finger protein 19 (PHF19), transcript variant 1, mRNA [NM_015651]	26147
A_23_P401904	2.0	1.6	-1.4	1.6	PHF19	Homo sapiens PHD finger protein 19 (PHF19), transcript variant 2, mRNA [NM_001009936]	26147

A_23_P216038	-1.6	-1.7	-1.8	-2.7	PHF20L1	Homo sapiens PHD finger protein 20-like 1 (PHF20L1), transcript variant 2, mRNA [NM_032205]	51105
A_23_P202715	-1.2	-1.2	-3.2	-1.3	PHF21A	Homo sapiens PHD finger protein 21A (PHF21A), transcript variant 2, mRNA [NM_016621]	51317
A_24_P5550	-1.8	-1.8	-2.1	-3.1	PHF23	Homo sapiens PHD finger protein 23 (PHF23), mRNA [NM_024297]	79142
A_23_P253571	-8.2	-1.1	-1.2	-1.2	PHF5A	Homo sapiens PHD finger protein 5A (PHF5A), mRNA [NM_032758]	84844

A_23_P171385	3.0	5.0	4.0	2.3	PHF6	Homo sapiens PHD finger protein 6 (PHF6), transcript variant 3, mRNA [NM_032335]	84295
A_24_P942253	-5.6	-4.6	-5.1	-4.9	PHF8	Homo sapiens PHD finger protein 8 (PHF8), transcript variant 2, mRNA [NM_015107]	23133
A_23_P85783	2.5	2.5	2.6	-1.4	PHGDH	Homo sapiens phosphoglycerate dehydrogenase (PHGDH), mRNA [NM_006623]	26227
A_23_P145437	1.2	2.0	1.6	-22.2	PHIP	Homo sapiens pleckstrin homology domain interacting protein (PHIP), mRNA [NM_017934]	55023

A_32_P61339	-2.4	1.0	1.0	1.0	PHIP	Homo sapiens pleckstrin homology domain interacting protein, mRNA (cDNA clone IMAGE:5260603). [BC036479]	55023
A_24_P931503	-2.1	-1.7	-1.1	-1.5	PHIP	Homo sapiens pleckstrin homology domain interacting protein (PHIP), mRNA [NM_017934]	55023
A_24_P238543	-1.8	-1.9	-2.1	-2.6	PHKA1	Homo sapiens phosphorylase kinase, alpha 1 (muscle) (PHKA1), transcript variant 1, mRNA [NM_002637]	5255

A_23_P77682	2.9	1.5	-1.4	2.2	PHKG2	Homo sapiens phosphorylase kinase, gamma 2 (testis) (PHKG2), transcript variant 1, mRNA [NM_000294]	5261
A_24_P943597	3.6	3.5	3.3	3.0	PHLDA1	Homo sapiens pleckstrin homology-like domain, family A, member 1 (PHLDA1), mRNA [NM_007350]	22822
A_23_P338912	-1.0	-5.4	1.5	1.5	PHLDA1	Homo sapiens pleckstrin homology-like domain, family A, member 1 (PHLDA1), mRNA [NM_007350]	22822

A_24_P240166	-53.1	1.1	-1.0	-1.0	PHLDB2	Homo sapiens pleckstrin homology-like domain, family B, member 2 (PHLDB2), transcript variant 4, mRNA [NM_145753]	90102
A_24_P935712	2.4	2.4	2.5	2.4	PHLPP1	Homo sapiens clone FLC0964 PRO2946 mRNA, complete cds. [AF130097]	23239
A_23_P49792	1.8	1.1	2.4	-1.0	PHOSPHO1	Homo sapiens phosphatase, orphan 1 (PHOSPHO1), transcript variant 2, mRNA [NM_178500]	162466
A_23_P83192	1.1	-2.4	-1.8	-3.2	PHPT1	Homo sapiens phosphohistidine phosphatase 1 (PHPT1), transcript variant 3, mRNA [NM_014172]	29085

A_23_P201179	-2.4	-1.6	-1.6	-1.5	PHTF1	Homo sapiens mRNA for homeobox protein LSX. [AJ011863]	10745
A_32_P409919	-11.0	-1.7	-1.0	1.0	PHTF2	Homo sapiens putative homeodomain transcription factor 2 (PHTF2), transcript variant 2, mRNA [NM_020432]	57157
A_23_P72001	-1.1	-2.5	-1.1	-1.5	PHYHD1	Homo sapiens phytanoyl-CoA dioxygenase domain containing 1 (PHYHD1), transcript variant 2, mRNA [NM_174933]	254295
A_23_P71364	1.2	2.5	1.2	1.4	PI15	Homo sapiens peptidase inhibitor 15 (PI15), mRNA [NM_015886]	51050

A_24_P300379	-3.8	-1.5	2.4	1.1	PI16	Homo sapiens peptidase inhibitor 16 (PI16), transcript variant 1, mRNA [NM_153370]	221476
A_23_P127339	-1.6	-1.4	-3.2	-1.2	PI4K2A	Homo sapiens phosphatidylinositol 4-kinase type 2 alpha (PI4K2A), mRNA [NM_018425]	55361
A_23_P18598	-3.0	-1.1	-1.3	-1.2	PI4K2B	Homo sapiens phosphatidylinositol 4-kinase type 2 beta (PI4K2B), mRNA [NM_018323]	55300
A_32_P507710	1.0	1.0	-2.2	-1.2	PI4KAP2	Homo sapiens phosphatidylinositol 4-kinase, catalytic, alpha pseudogene 2 (PI4KAP2), non-coding RNA [NR_003700]	375133

A_23_P103824	-1.0	-2.0	1.1	1.0	PI4KB	Homo sapiens phosphatidylinositol 4-kinase, catalytic, beta (PI4KB), transcript variant 2, mRNA [NM_001198773]	5298
A_23_P163353	-1.9	-1.9	-2.6	-1.1	PIAS1	Homo sapiens protein inhibitor of activated STAT, 1 (PIAS1), mRNA [NM_016166]	8554
A_23_P149678	1.8	-1.0	-3.3	-3.1	PIAS3	Homo sapiens protein inhibitor of activated STAT, 3 (PIAS3), mRNA [NM_006099]	10401
A_24_P6889	-4.5	-1.3	-2.8	-4.9	PIAS3	Homo sapiens protein inhibitor of activated STAT, 3 (PIAS3), mRNA [NM_006099]	10401

A_23_P90280	-1.7	-2.0	-1.6	-3.3	PIAS4	Homo sapiens protein inhibitor of activated STAT, 4 (PIAS4), mRNA [NM_015897]	51588
A_23_P411881	-4.7	1.2	1.1	-7.7	PIBF1	Homo sapiens progesterone immunomodulatory binding factor 1 (PIBF1), mRNA [NM_006346]	10464
A_23_P203658	1.1	-2.2	-1.2	3.2	PICALM	Homo sapiens phosphatidylinositol binding clathrin assembly protein (PICALM), transcript variant 1, mRNA [NM_007166]	8301

A_23_P147995	2.8	4.2	2.8	4.4	PICALM	Homo sapiens phosphatidylinositol binding clathrin assembly protein (PICALM), transcript variant 1, mRNA [NM_007166]	8301
A_23_P91727	-3.9	1.8	2.0	1.5	PICK1	Homo sapiens protein interacting with PRKCA 1 (PICK1), transcript variant 1, mRNA [NM_012407]	9463
A_23_P140738	2.3	2.6	1.0	1.1	PIEZO1	Homo sapiens piezo-type mechanosensitive ion channel component 1 (PIEZO1), mRNA [NM_001142864]	9780

A_23_P130376	1.2	1.3	-3.3	-1.1	PIEZO2	Homo sapiens piezo-type mechanosensitive ion channel component 2 (PIEZO2), mRNA [NM_022068]	63895
A_24_P417831	-1.9	1.3	-1.1	-3.8	PIEZO2	Homo sapiens piezo-type mechanosensitive ion channel component 2 (PIEZO2), mRNA [NM_022068]	63895
A_23_P323743	3.2	-2.6	-1.7	1.3	PIF1	Homo sapiens PIF1 5'-to-3' DNA helicase homolog (S. cerevisiae) (PIF1), mRNA [NM_025049]	80119

A_23_P74852	1.6	1.4	2.6	1.6	PIGC	phosphatidylinositol glycan anchor biosynthesis, class C [Source:HGNC Symbol;Acc:8960] [ENST00000484368]	5279
A_23_P86252	-1.0	-2.1	-8.9	-2.6	PIGC	Homo sapiens phosphatidylinositol glycan anchor biosynthesis, class C (PIGC), transcript variant 2, mRNA [NM_002642]	5279
A_23_P60240	-1.0	-21.0	-1.2	-1.1	PIGO	Homo sapiens phosphatidylinositol glycan anchor biosynthesis, class O (PIGO), transcript variant 1, mRNA [NM_032634]	84720

A_23_P79842	-2.4	1.6	1.4	1.5	PIGT	Homo sapiens phosphatidylinositol glycan anchor biosynthesis, class T (PIGT), transcript variant 1, mRNA [NM_015937]	51604
A_24_P944848	2.8	2.8	2.8	2.9	PIGT	Homo sapiens PRO0433 mRNA, complete cds. [AF305815]	51604
A_23_P46378	-2.3	-2.6	-2.7	-1.3	PIGV	Homo sapiens phosphatidylinositol glycan anchor biosynthesis, class V (PIGV), transcript variant 2, mRNA [NM_017837]	55650
A_23_P143935	-2.8	1.0	1.8	1.1	PIGZ	Homo sapiens phosphatidylinositol glycan anchor biosynthesis, class Z (PIGZ), mRNA [NM_025163]	80235

A_23_P36166	-3.1	-36.7	-2.7	1.0	PIK3C2A	Homo sapiens phosphoinositide-3-kinase, class 2, alpha polypeptide (PIK3C2A), mRNA [NM_002645]	5286
A_24_P288116	1.1	1.1	1.7	3.8	PIK3C3	phosphoinositide-3-kinase, class 3 [Source:HGNC Symbol;Acc:8974] [ENST00000262039]	5289
A_24_P71244	-1.5	-11.2	-2.6	1.1	PIK3CD	Homo sapiens phosphoinositide-3-kinase, catalytic, delta polypeptide (PIK3CD), mRNA [NM_005026]	5293

A_23_P251359	-3.3	1.3	-1.8	-2.3	PIK3CG	Homo sapiens phosphoinositide-3-kinase, catalytic, gamma polypeptide (PIK3CG), mRNA [NM_002649]	5294
A_23_P142361	-15.6	-1.0	-1.5	-1.0	PIK3R2	Homo sapiens phosphoinositide-3-kinase, regulatory subunit 2 (beta) (PIK3R2), mRNA [NM_005027]	5296
A_23_P22970	-1.5	1.1	-3.3	-1.8	PIK3R3	Homo sapiens phosphoinositide-3-kinase, regulatory subunit 3 (gamma) (PIK3R3), transcript variant 1, mRNA [NM_003629]	8503

A_24_P156781	1.0	3.8	1.0	1.6	PIK3R3	Homo sapiens phosphoinositide-3-kinase, regulatory subunit 3 (gamma) (PIK3R3), transcript variant 1, mRNA [NM_003629]	8503
A_24_P527274	-1.0	-1.2	-2.1	-1.6	PIK3R3	phosphoinositide-3-kinase, regulatory subunit 3 (gamma) [Source:HGNC Symbol;Acc:8981] [ENST00000540385]	8503
A_23_P132526	3.2	1.7	-3.3	1.5	PIK3R4	Homo sapiens phosphoinositide-3-kinase, regulatory subunit 4 (PIK3R4), mRNA [NM_014602]	30849

A_23_P66543	1.8	1.2	-1.1	-4.7	PIK3R5	Homo sapiens phosphoinositide-3-kinase, regulatory subunit 5 (PIK3R5), transcript variant 2, mRNA [NM_014308]	23533
A_24_P366509	-1.5	1.3	-1.1	2.5	PIK3R6	Homo sapiens phosphoinositide-3-kinase, regulatory subunit 6 (PIK3R6), mRNA [NM_001010855]	146850
A_23_P31224	-17.0	-1.1	-1.1	-1.1	PILRA	Homo sapiens paired immunoglobin-like type 2 receptor alpha (PILRA), transcript variant 3, mRNA [NM_178273]	29992
A_23_P345118	1.6	1.8	2.0	1.4	PIM1	Homo sapiens pim-1 oncogene (PIM1), transcript variant 1, mRNA [NM_002648]	5292

A_24_P379104	1.9	2.3	1.8	1.6	PIM2	Homo sapiens pim-2 oncogene (PIM2), mRNA [NM_006875]	11040
A_23_P67162	1.1	2.2	-1.2	1.4	PIN1	Homo sapiens peptidylprolyl cis/trans isomerase, NIMA-interacting 1 (PIN1), transcript variant 1, mRNA [NM_006221]	5300
A_24_P276493	1.6	1.2	-2.0	2.1	PIN1P1	Homo sapiens peptidylprolyl cis/trans isomerase, NIMA-interacting 1 pseudogene 1 (PIN1P1), non-coding RNA [NR_023916]	5301

A_23_P266	-3.9	1.2	1.1	-1.7	PIN1P1	Homo sapiens peptidylprolyl cis/trans isomerase, NIMA-interacting 1 pseudogene 1 (PIN1P1), non-coding RNA [NR_023916]	5301
A_24_P235360	-2.6	1.6	1.2	1.6	PINX1	PIN2/TERF1 interacting, telomerase inhibitor 1 [Source:HGNC Symbol;Acc:30046] [ENST00000426190]	54984
A_23_P8702	-18.9	-1.0	-1.1	-1.0	PIP	Homo sapiens prolactin-induced protein (PIP), mRNA [NM_002652]	5304

A_23_P407115	1.2	3.1	1.6	1.5	PIP4K2B	Homo sapiens phosphatidylinositol-5-phosphate 4-kinase, type II, beta (PIP4K2B), mRNA [NM_003559]	8396
A_23_P162199	-3.2	1.8	-2.0	-2.7	PIP4K2C	Homo sapiens phosphatidylinositol-5-phosphate 4-kinase, type II, gamma (PIP4K2C), transcript variant 1, mRNA [NM_024779]	79837
A_23_P23572	1.6	1.3	-2.6	-1.5	PIP5K1A	Homo sapiens phosphatidylinositol-4-phosphate 5-kinase, type I, alpha (PIP5K1A), transcript variant 2, mRNA [NM_003557]	8394

A_23_P78952	1.6	2.6	-1.5	1.0	PIP5K1C	Homo sapiens phosphatidylinositol-4-phosphate 5-kinase, type I, gamma (PIP5K1C), transcript variant 2, mRNA [NM_012398]	23396
A_23_P330262	-14.8	1.9	-1.0	1.1	PIP5K1C	Homo sapiens phosphatidylinositol-4-phosphate 5-kinase, type I, gamma (PIP5K1C), transcript variant 2, mRNA [NM_012398]	23396
A_23_P164258	1.8	2.7	-1.5	1.9	PIPOX	Homo sapiens pipecolic acid oxidase (PIPOX), mRNA [NM_016518]	51268

A_23_P143662	1.5	1.5	3.5	3.0	PISD	Homo sapiens phosphatidylserine decarboxylase (PISD), mRNA [NM_014338]	23761
A_23_P89283	-1.3	1.1	-2.1	-1.3	PITPNA	Homo sapiens phosphatidylinositol transfer protein, alpha (PITPNA), mRNA [NM_006224]	5306
A_24_P305678	-1.0	-1.1	-1.2	-14.9	PITPNB	Homo sapiens phosphatidylinositol transfer protein, beta (PITPNB), mRNA [NM_012399]	23760
A_24_P772103	-1.0	-2.0	-1.0	-1.0	PITPNC1	Homo sapiens cDNA FLJ37405 fis, clone BRAMY2028269. [AK094724]	26207

A_23_P1519	1.1	1.5	2.2	1.2	PITPNM1	Homo sapiens phosphatidylinositol transfer protein, membrane-associated 1 (PITPNM1), transcript variant 1, mRNA [NM_004910]	9600
A_23_P403542	-1.0	-19.1	1.0	1.0	PITPNM2	Homo sapiens phosphatidylinositol transfer protein, membrane-associated 2 (PITPNM2), mRNA [NM_020845]	57605
A_23_P401361	3.8	3.6	3.4	3.5	PITPNM2	Homo sapiens phosphatidylinositol transfer protein, membrane-associated 2 (PITPNM2), mRNA [NM_020845]	57605

A_23_P66753	-1.4	-1.4	-3.6	-3.8	PITPNM3	Homo sapiens PITPNM family member 3 (PITPNM3), transcript variant 1, mRNA [NM_031220]	83394
A_23_P52402	-1.0	-7.8	-1.5	-1.1	PITRM1	Homo sapiens pitrilysin metallopeptidase 1 (PITRM1), nuclear gene encoding mitochondrial protein, transcript variant 2, mRNA [NM_014889]	10531
A_23_P167367	2.9	1.1	3.6	2.0	PITX2	Homo sapiens paired-like homeodomain 2 (PITX2), transcript variant 2, mRNA [NM_153426]	5308

A_24_P221858	-1.0	-2.2	-1.0	-1.0	PIWIL3	Homo sapiens piwi-like 3 (Drosophila) (PIWIL3), mRNA [NM_001008496]	440822
A_23_P11244	1.9	2.4	1.7	1.8	PJA1	Homo sapiens praja ring finger 1 (PJA1), transcript variant 3, mRNA [NM_022368]	64219
A_23_P133470	3.6	3.6	3.6	3.7	PJA2	Homo sapiens praja ring finger 2 (PJA2), mRNA [NM_014819]	9867
A_32_P92489	3.0	1.0	1.1	1.1	PKD1L2	Homo sapiens polycystic kidney disease 1-like 2 (PKD1L2), transcript variant 1, mRNA [NM_052892]	114780

A_23_P129332	1.0	1.0	-7.1	1.0	PKD1L2	Homo sapiens polycystic kidney disease 1-like 2 (PKD1L2), transcript variant 3, mRNA [NM_001076780]	114780
A_23_P167324	1.1	1.0	-4.6	1.1	PKD2	Homo sapiens polycystic kidney disease 2 (autosomal dominant) (PKD2), mRNA [NM_000297]	5311
A_32_P112493	-1.0	1.1	-5.0	-1.6	PKDCC	Homo sapiens protein kinase domain containing, cytoplasmic homolog (mouse) (PKDCC), mRNA [NM_138370]	91461

A_23_P392486	20.3	19.3	27.7	18.5	PKHD1L1	Homo sapiens polycystic kidney and hepatic disease 1 (autosomal recessive)-like 1 (PKHD1L1), mRNA [NM_177531]	93035
A_23_P31765	-2.5	1.0	-1.7	-1.0	PKIA	Homo sapiens protein kinase (cAMP-dependent, catalytic) inhibitor alpha (PKIA), transcript variant 1, mRNA [NM_006823]	5569
A_23_P91221	1.0	-26.8	-1.0	1.1	PKIG	Homo sapiens protein kinase (cAMP-dependent, catalytic) inhibitor gamma (PKIG), transcript variant 1, mRNA [NM_181805]	11142

A_24_P325533	8.7	12.6	5.3	4.3	PKLR	Homo sapiens pyruvate kinase, liver and RBC (PKLR), nuclear gene encoding mitochondrial protein, transcript variant 1, mRNA [NM_000298]	5313
A_23_P201022	-1.9	-7.2	-2.5	-2.3	PKLR	Homo sapiens pyruvate kinase, liver and RBC (PKLR), nuclear gene encoding mitochondrial protein, transcript variant 1, mRNA [NM_000298]	5313
A_23_P399501	-1.4	-2.5	-1.5	-1.4	PKM2	Homo sapiens pyruvate kinase, muscle (PKM2), transcript variant 2, mRNA [NM_182470]	5315

A_23_P398515	2.4	3.1	2.2	3.4	PKMYT1	Homo sapiens protein kinase, membrane associated tyrosine/threonine 1 (PKMYT1), transcript variant 1, mRNA [NM_004203]	9088
A_24_P342410	-2.4	1.1	-1.0	-1.3	PKN2	Homo sapiens PRO2042 mRNA, complete cds. [AF118089]	5586
A_23_P23227	1.8	1.1	-2.2	1.9	PKN2	Homo sapiens protein kinase N2 (PKN2), mRNA [NM_006256]	5586
A_24_P133288	-4.1	-5.0	1.8	-1.2	PKP2	H.sapiens mRNA for plakophilin 2a and b. [X97675]	5318

A_23_P162466	1.5	2.2	1.4	1.4	PKP2	Homo sapiens plakophilin 2 (PKP2), transcript variant 2b, mRNA [NM_004572]	5318
A_23_P95810	-4.9	-2.1	-1.6	-3.9	PKP3	Homo sapiens plakophilin 3 (PKP3), mRNA [NM_007183]	11187
A_24_P294408	2.1	2.0	1.8	1.9	PLA1A	Homo sapiens phospholipase A1 member A (PLA1A), transcript variant 1, mRNA [NM_015900]	51365
A_23_P88767	-1.1	-1.2	2.6	-1.4	PLA2G10	Homo sapiens phospholipase A2, group X (PLA2G10), mRNA [NM_003561]	8399
A_24_P706752	1.0	-6.0	-1.1	-8.3	PLA2G12A	Homo sapiens phospholipase A2, group XIIA (PLA2G12A), mRNA [NM_030821]	81579

A_23_P388150	1.1	2.2	1.8	1.9	PLA2G12B	Homo sapiens phospholipase A2, group XIIB (PLA2G12B), mRNA [NM_032562]	84647
A_23_P3584	2.1	2.5	2.1	1.7	PLA2G15	Homo sapiens phospholipase A2, group XV (PLA2G15), mRNA [NM_012320]	23659
A_24_P224116	8.2	3.8	3.1	2.7	PLA2G1B	Homo sapiens phospholipase A2, group IB (pancreas) (PLA2G1B), mRNA [NM_000928]	5319
A_24_P111271	-16.7	-1.3	-1.1	-1.2	PLA2G2D	Homo sapiens phospholipase A2, group IID (PLA2G2D), mRNA [NM_012400]	26279
A_23_P114857	-1.1	-2.3	-1.4	1.0	PLA2G2E	Homo sapiens phospholipase A2, group IIE (PLA2G2E), mRNA [NM_014589]	30814

A_24_P208081	6.0	8.0	5.4	5.5	PLA2G2F	Homo sapiens phospholipase A2, group IIF (PLA2G2F), mRNA [NM_022819]	64600
A_23_P17821	2.1	2.0	1.8	-1.2	PLA2G3	Homo sapiens phospholipase A2, group III (PLA2G3), mRNA [NM_015715]	50487
A_23_P17814	-1.0	1.4	-1.0	5.3	PLA2G3	Homo sapiens phospholipase A2, group III (PLA2G3), mRNA [NM_015715]	50487
A_23_P50508	2.3	-6.3	-1.5	-1.2	PLA2G4C	Homo sapiens phospholipase A2, group IVC (cytosolic, calcium-independent) (PLA2G4C), transcript variant 1, mRNA [NM_003706]	8605

A_24_P316439	-8.6	-1.3	-1.0	-2.4	PLA2G4C	Homo sapiens phospholipase A2, group IVC (cytosolic, calcium-independent) (PLA2G4C), transcript variant 1, mRNA [NM_003706]	8605
A_23_P358531	3.5	3.3	-1.1	2.1	PLA2G4D	Homo sapiens phospholipase A2, group IVD (cytosolic), mRNA (cDNA clone IMAGE:5167964), complete cds. [BC034571]	283748
A_24_P147169	-1.0	1.3	-2.5	-1.0	PLA2G4F	Homo sapiens phospholipase A2, group IVF (PLA2G4F), transcript variant 1, mRNA [NM_213600]	255189

A_23_P103465	-3.1	1.2	1.1	-3.2	PLA2G5	Homo sapiens phospholipase A2, group V (PLA2G5), mRNA [NM_000929]	5322
A_23_P145096	-20.0	1.0	1.0	1.1	PLA2G7	Homo sapiens phospholipase A2, group VII (platelet-activating factor acetylhydrolase, plasma) (PLA2G7), transcript variant 1, mRNA [NM_005084]	7941
A_23_P142830	-1.0	-1.0	-2.5	-1.8	PLA2R1	Homo sapiens phospholipase A2 receptor 1, 180kDa (PLA2R1), transcript variant 1, mRNA [NM_007366]	22925
A_24_P76558	-4.2	1.4	1.4	1.0	PLAC4	Homo sapiens placenta-specific 4 (PLAC4), mRNA [NM_182832]	191585

A_32_P216241	-1.2	-1.8	-1.6	2.0	PLAC4	Homo sapiens placenta-specific 4 (PLAC4), mRNA [NM_182832]	191585
A_24_P183128	-1.3	-1.1	-2.1	1.1	PLAC8	Homo sapiens placenta-specific 8 (PLAC8), transcript variant 2, mRNA [NM_016619]	51316
A_24_P312041	2.5	-6.1	1.9	3.3	PLAGL1	Homo sapiens pleiomorphic adenoma gene-like 1 (PLAGL1), transcript variant 2, mRNA [NM_006718]	5325
A_23_P6151	1.5	-4.8	1.6	1.1	PLAGL2	Homo sapiens pleiomorphic adenoma gene-like 2 (PLAGL2), mRNA [NM_002657]	5326

A_23_P329321	-1.1	2.1	1.9	1.0	PLB1	Homo sapiens cDNA FLJ30866 fis, clone FEBRA2004110, highly similar to PHOSPHOLIPASE ADRAB-B PRECURSOR (EC 3.1.-.-). [AK055428]	151056
A_23_P56356	1.2	2.1	1.4	1.1	PLB1	Homo sapiens phospholipase B1 (PLB1), transcript variant 1, mRNA [NM_153021]	151056
A_24_P379616	1.5	1.5	1.0	-2.8	PLB1	Homo sapiens phospholipase B1 (PLB1), transcript variant 1, mRNA [NM_153021]	151056
A_23_P87709	1.1	1.0	1.0	2.6	PLBD1	Homo sapiens phospholipase B domain containing 1 (PLBD1), mRNA [NM_024829]	79887

A_24_P287664	5.6	6.9	5.4	5.9	PLCB2	Homo sapiens phospholipase C, beta 2 (PLCB2), mRNA [NM_004573]	5330
A_23_P343719	-1.0	-1.3	1.3	-3.1	PLCB3	Homo sapiens phospholipase C, beta 3 (phosphatidylinositol-specific) (PLCB3), transcript variant 1, mRNA [NM_000932]	5331
A_23_P28898	1.0	-3.4	-1.4	2.0	PLCB4	Homo sapiens phospholipase C, beta 4 (PLCB4), transcript variant 1, mRNA [NM_000933]	5332
A_23_P80739	-2.5	-1.3	-1.8	-2.5	PLCD1	Homo sapiens phospholipase C, delta 1 (PLCD1), transcript variant 2, mRNA [NM_006225]	5333

A_23_P351757	3.0	2.6	2.1	3.9	PLCD3	Homo sapiens phospholipase C, delta 3 (PLCD3), mRNA [NM_133373]	113026
A_23_P339240	-1.7	-1.7	-9.8	-1.1	PLCH1	Homo sapiens phospholipase C, eta 1 (PLCH1), transcript variant 2, mRNA [NM_014996]	23007
A_24_P62469	2.0	2.4	-2.0	1.9	PLCH2	Homo sapiens phospholipase C, eta 2 (PLCH2), mRNA [NM_014638]	9651
A_24_P21887	-1.0	2.3	1.1	1.9	PLCH2	Homo sapiens phospholipase C, eta 2 (PLCH2), mRNA [NM_014638]	9651
A_32_P149158	3.9	3.7	5.0	3.6	PLCL1	Homo sapiens phospholipase C-like 1 (PLCL1), mRNA [NM_006226]	5334

A_23_P80594	-15.6	-1.0	-1.1	-5.9	PLCL2	Homo sapiens phospholipase C-like 2 (PLCL2), transcript variant 2, mRNA [NM_015184]	23228
A_24_P281988	-1.1	-1.7	-2.1	-1.0	PLCL2	Homo sapiens phospholipase C-like 2 (PLCL2), transcript variant 2, mRNA [NM_015184]	23228
A_23_P105524	1.0	-3.1	1.1	1.0	PLCZ1	Homo sapiens phospholipase C, zeta 1 (PLCZ1), mRNA [NM_033123]	89869
A_23_P398774	-1.3	-4.6	-1.4	-1.5	PLD1	phospholipase D1, phosphatidylcholine-specific [Source:HGNC Symbol;Acc:9067] [ENST00000471075]	5337

A_23_P27515	2.2	1.3	1.3	1.7	PLD3	Homo sapiens phospholipase D family, member 3 (PLD3), transcript variant 2, mRNA [NM_012268]	23646
A_24_P936312	-2.7	-2.3	-4.3	-2.4	PLD3	Homo sapiens mRNA; cDNA DKFZp686J11131 (from clone DKFZp686J11131). [BX648310]	23646
A_23_P88228	-2.9	-3.0	-3.9	-1.2	PLD4	Homo sapiens phospholipase D family, member 4 (PLD4), mRNA [NM_138790]	122618

A_24_P914759	-1.8	1.0	-1.2	-8.6	PLEC	Homo sapiens plectin (PLEC), nuclear gene encoding mitochondrial protein, transcript variant 8, mRNA [NM_201382]	5339
A_23_P22119	1.2	1.1	-8.6	1.1	PLEC	Homo sapiens plectin (PLEC), transcript variant 6, mRNA [NM_201380]	5339
A_23_P151506	1.4	-1.5	-1.7	-4.5	PLEK2	Homo sapiens pleckstrin 2 (PLEK2), mRNA [NM_016445]	26499
A_23_P314363	-6.4	1.1	-1.1	-1.0	PLEKHA2	Homo sapiens pleckstrin homology domain containing, family A (phosphoinositide binding specific) member 2 (PLEKHA2), mRNA [NM_021623]	59339

A_32_P209206	1.0	-1.1	-1.7	-2.5	PLEKHA3	Homo sapiens pleckstrin homology domain containing, family A (phosphoinositide binding specific) member 3, mRNA (cDNA clone IMAGE:4345190). [BC035471]	65977
A_24_P328471	-16.1	-1.0	-1.0	-1.0	PLEKHA3	Homo sapiens pleckstrin homology domain containing, family A (phosphoinositide binding specific) member 3 (PLEKHA3), mRNA [NM_019091]	65977

A_23_P67360	1.6	-4.3	-1.0	1.0	PLEKHA4	Homo sapiens pleckstrin homology domain containing, family A (phosphoinositide binding specific) member 4 (PLEKHA4), transcript variant 1, mRNA [NM_020904]	57664
A_24_P41170	-4.1	-1.0	-1.1	1.5	PLEKHA5	pleckstrin homology domain containing, family A member 5 [Source:HGNC Symbol;Acc:30036] [ENST00000510738]	54477
A_23_P431268	-2.4	-1.1	-1.2	-1.1	PLEKHA6	Homo sapiens pleckstrin homology domain containing, family A member 6 (PLEKHA6), mRNA [NM_014935]	22874

A_23_P148753	1.8	1.8	2.4	1.0	PLEKHA6	Homo sapiens pleckstrin homology domain containing, family A member 6 (PLEKHA6), mRNA [NM_014935]	22874
A_24_P56073	-3.8	1.9	1.1	-1.0	PLEKHB1	Homo sapiens pleckstrin homology domain containing, family B (evectins) member 1 (PLEKHB1), transcript variant 1, mRNA [NM_021200]	58473
A_24_P873414	1.6	-1.2	1.5	-3.4	PLEKHB2	Homo sapiens pleckstrin homology domain containing, family B (evectins) member 2 (PLEKHB2), transcript variant 3, mRNA [NM_001100623]	55041

A_23_P79134	-4.7	1.1	-3.4	-2.9	PLEKHF1	Homo sapiens pleckstrin homology domain containing, family F (with FYVE domain) member 1 (PLEKHF1), mRNA [NM_024310]	79156
A_24_P194068	5.6	4.4	6.0	2.7	PLEKHF1	Homo sapiens pleckstrin homology domain containing, family F (with FYVE domain) member 1 (PLEKHF1), mRNA [NM_024310]	79156
A_23_P20275	1.6	1.6	2.0	1.6	PLEKHF2	Homo sapiens pleckstrin homology domain containing, family F (with FYVE domain) member 2 (PLEKHF2), mRNA [NM_024613]	79666

A_23_P323563	-1.7	3.9	1.2	1.5	PLEKHG2	Homo sapiens pleckstrin homology domain containing, family G (with RhoGef domain) member 2 (PLEKHG2), mRNA [NM_022835]	64857
A_32_P129269	1.6	2.4	-1.7	-1.2	PLEKHG4B	Homo sapiens pleckstrin homology domain containing, family G (with RhoGef domain) member 4B (PLEKHG4B), mRNA [NM_052909]	153478
A_23_P81640	-1.2	3.2	2.5	-1.1	PLEKHG4B	Homo sapiens pleckstrin homology domain containing, family G (with RhoGef domain) member 4B (PLEKHG4B), mRNA [NM_052909]	153478

A_23_P149626	1.2	1.1	-3.0	-1.1	PLEKHG5	Homo sapiens pleckstrin homology domain containing, family G (with RhoGef domain) member 5 (PLEKHG5), transcript variant 2, mRNA [NM_198681]	57449
A_24_P301063	3.3	1.3	1.3	1.7	PLEKHG6	Homo sapiens pleckstrin homology domain containing, family G (with RhoGef domain) member 6 (PLEKHG6), transcript variant 1, mRNA [NM_018173]	55200

A_32_P172188	4.0	6.0	12.9	-1.1	PLEKHH3	Homo sapiens pleckstrin homology domain containing, family H (with MyTH4 domain) member 3 (PLEKHH3), mRNA [NM_024927]	79990
A_23_P164112	6.7	6.0	9.4	1.4	PLEKHM1	Homo sapiens pleckstrin homology domain containing, family M (with RUN domain) member 1 (PLEKHM1), transcript variant 1, mRNA [NM_014798]	9842

A_23_P152727	-2.9	1.0	-1.0	1.1	PLEKHM1	Homo sapiens pleckstrin homology domain containing, family M (with RUN domain) member 1 (PLEKHM1), transcript variant 1, mRNA [NM_014798]	9842
A_23_P129246	1.2	-1.7	-2.0	-1.4	PLEKHO2	Homo sapiens pleckstrin homology domain containing, family O member 2 (PLEKHO2), transcript variant 1, mRNA [NM_025201]	80301
A_32_P14178	5.5	5.3	4.9	5.4	PLG	Homo sapiens plasminogen (PLG), transcript variant 1, mRNA [NM_000301]	5340

A_24_P923011	-1.2	1.2	-4.8	-1.7	PLG	Homo sapiens plasminogen (PLG), transcript variant 1, mRNA [NM_000301]	5340
A_23_P134953	-1.8	-2.7	-1.8	-4.3	PLIN2	Homo sapiens perilipin 2 (PLIN2), transcript variant 1, mRNA [NM_001122]	123
A_24_P272222	2.5	1.6	1.5	1.5	PLIN5	Homo sapiens perilipin 5 (PLIN5), mRNA [NM_001013706]	440503
A_23_P118174	3.4	2.2	1.7	4.4	PLK1	Homo sapiens polo-like kinase 1 (PLK1), mRNA [NM_005030]	5347
A_23_P154740	-2.6	1.3	1.1	-1.0	PLK1S1	Homo sapiens polo-like kinase 1 substrate 1 (PLK1S1), transcript variant 1, mRNA [NM_018474]	55857

A_23_P155969	3.4	3.1	3.8	2.6	PLK4	Homo sapiens polo-like kinase 4 (PLK4), transcript variant 1, mRNA [NM_014264]	10733
A_23_P43763	-3.2	1.8	-1.7	-2.1	PLLP	Homo sapiens plasmolipin (PLLP), mRNA [NM_015993]	51090
A_24_P414803	323.5	204.4	265.5	39.9	PLN	Homo sapiens phospholamban (PLN), mRNA [NM_002667]	5350
A_23_P137532	1.7	1.5	2.6	1.3	PLOD1	Homo sapiens procollagen-lysine, 2-oxoglutarate 5-dioxygenase 1 (PLOD1), mRNA [NM_000302]	5351

A_23_P80508	-1.4	-4.0	-1.8	-23.2	PLSCR2	Homo sapiens phospholipid scramblase 2 (PLSCR2), transcript variant 1, mRNA [NM_001199978]	57047
A_23_P91910	1.1	1.1	-1.0	-2.5	PLSCR4	Homo sapiens phospholipid scramblase 4 (PLSCR4), transcript variant 2, mRNA [NM_020353]	57088
A_23_P5983	1.2	1.7	1.4	4.1	PLTP	Homo sapiens phospholipid transfer protein (PLTP), transcript variant 1, mRNA [NM_006227]	5360
A_23_P56328	-1.6	-3.3	-1.9	-2.3	PLVAP	Homo sapiens plasmalemma vesicle associated protein (PLVAP), mRNA [NM_031310]	83483

A_24_P245589	-1.4	2.0	-1.8	-1.0	PLVAP	Homo sapiens plasmalemma vesicle associated protein (PLVAP), mRNA [NM_031310]	83483
A_32_P208823	10.4	11.7	11.1	8.6	PLXDC1	Homo sapiens plexin domain containing 1 (PLXDC1), mRNA [NM_020405]	57125
A_24_P246262	-1.5	-2.6	-1.7	-1.9	PLXNA4	Homo sapiens plexin A4 (PLXNA4), transcript variant 1, mRNA [NM_020911]	91584
A_23_P57961	-4.6	-1.0	-1.1	-1.0	PLXNB1	Homo sapiens plexin B1 (PLXNB1), transcript variant 1, mRNA [NM_002673]	5364
A_24_P70888	-1.2	-8.0	-1.3	-2.0	PLXNB2	Homo sapiens plexin B2 (PLXNB2), mRNA [NM_012401]	23654

A_23_P137168	1.5	2.5	1.7	1.9	PLXNB3	Homo sapiens plexin B3 (PLXNB3), transcript variant 1, mRNA [NM_005393]	5365
A_23_P414958	-2.6	-1.8	-16.4	-3.1	PLXNC1	Homo sapiens plexin C1 (PLXNC1), transcript variant 1, mRNA [NM_005761]	10154
A_23_P80752	4.9	4.6	4.1	5.3	PLXND1	Homo sapiens plexin D1 (PLXND1), mRNA [NM_015103]	23129
A_24_P376391	2.3	2.3	1.3	-2.9	PLXND1	Homo sapiens plexin D1 (PLXND1), mRNA [NM_015103]	23129
A_32_P86118	1.1	1.1	1.0	-20.4	PM20D2	Homo sapiens peptidase M20 domain containing 2 (PM20D2), mRNA [NM_001010853]	135293

A_23_P321223	-1.3	-1.0	-2.2	-1.0	PMCH	Homo sapiens pro-melanin-concentrating hormone (PMCH), mRNA [NM_002674]	5367
A_23_P312851	5.5	2.9	2.8	2.9	PMEL	Homo sapiens premelanosome protein (PMEL), transcript variant 3, mRNA [NM_006928]	6490
A_23_P2233	1.2	-21.1	-1.5	1.1	PMEL	Homo sapiens premelanosome protein (PMEL), transcript variant 3, mRNA [NM_006928]	6490
A_23_P57089	1.2	-1.5	-1.0	-2.0	PMEPA1	Homo sapiens prostate transmembrane protein, androgen induced 1 (PMEPA1), transcript variant 1, mRNA [NM_020182]	56937

A_23_P635	-1.0	-5.1	1.8	-1.1	PMF1	Homo sapiens polyamine-modulated factor 1 (PMF1), transcript variant 2, mRNA [NM_007221]	11243
A_24_P207139	-3.1	1.0	-1.0	-3.1	PML	Homo sapiens promyelocytic leukemia (PML), transcript variant 1, mRNA [NM_033238]	5371
A_23_P306148	1.1	1.1	-2.4	2.0	PML	Homo sapiens promyelocytic leukemia (PML), transcript variant 6, mRNA [NM_002675]	5371
A_24_P133542	-1.0	-1.0	-1.0	4.4	PML	Homo sapiens promyelocytic leukemia (PML), transcript variant 6, mRNA [NM_002675]	5371

A_23_P334664	-1.2	-3.6	-2.7	1.1	PML	Homo sapiens promyelocytic leukemia (PML), transcript variant 8, mRNA [NM_033247]	5371
A_23_P40059	-3.0	-3.0	-3.0	-3.1	PMS1	Homo sapiens PMS1 postmeiotic segregation increased 1 (S. cerevisiae) (PMS1), transcript variant 1, mRNA [NM_000534]	5378
A_24_P410859	1.7	1.1	2.3	1.7	PMS2	Homo sapiens PMS2 postmeiotic segregation increased 2 (S. cerevisiae) (PMS2), transcript variant 1, mRNA [NM_000535]	5395

A_32_P10272	-3.2	-1.0	-1.0	-1.0	PMS2L2	Homo sapiens postmeiotic segregation increased 2-like 2 pseudogene (PMS2L2), non-coding RNA [NR_003614]	5380
A_32_P57728	-1.1	-1.1	-2.3	-8.3	PMS2P1	Homo sapiens postmeiotic segregation increased 2 pseudogene 1 (PMS2P1), non-coding RNA [NR_003613]	5379

A_32_P185507	1.0	-1.0	4.2	-2.2	PMS2P1	Homo sapiens postmeiotic segregation increased 2-like 1, mRNA (cDNA clone IMAGE:6049115), partial cds. [BC044214]	5379
A_23_P114774	-1.0	1.1	2.3	1.0	PMVK	Homo sapiens phosphomevalonate kinase (PMVK), mRNA [NM_006556]	10654
A_23_P62377	5.5	5.6	5.6	5.6	PNCK	Homo sapiens pregnancy up-regulated non-ubiquitously expressed CaM kinase (PNCK), transcript variant 1, mRNA [NM_001039582]	139728

A_23_P35484	-2.1	-1.5	-1.6	-1.4	PNLIPRP1	Homo sapiens pancreatic lipase-related protein 1 (PNLIPRP1), mRNA [NM_006229]	5407
A_23_P24083	1.0	-4.0	-1.1	-1.2	PNLIPRP2	Homo sapiens pancreatic lipase-related protein 2 (PNLIPRP2), mRNA [NM_005396]	5408
A_23_P99771	1.2	-1.8	1.5	2.9	PNMA1	Homo sapiens paraneoplastic antigen MA1 (PNMA1), mRNA [NM_006029]	9240
A_24_P153412	-2.3	1.1	-1.0	1.0	PNMA2	Homo sapiens paraneoplastic antigen MA2 (PNMA2), mRNA [NM_007257]	10687

A_23_P352950	1.5	-1.1	-2.0	-1.9	PNMA5	Homo sapiens paraneoplastic antigen like 5 (PNMA5), transcript variant 3, mRNA [NM_052926]	114824
A_23_P100642	1.0	2.4	1.0	1.0	PNMT	Homo sapiens phenylethanolamine N-methyltransferase (PNMT), mRNA [NM_002686]	5409
A_23_P99582	2.4	1.8	1.4	1.2	PNN	Homo sapiens pinin, desmosome associated protein (PNN), mRNA [NM_002687]	5411

A_32_P526255	1.3	-3.0	1.3	1.3	PNPLA1	Homo sapiens patatin-like phospholipase domain containing 1 (PNPLA1), transcript variant 3, mRNA [NM_001145717]	285848
A_23_P17914	-1.0	2.2	-1.3	1.4	PNPLA3	Homo sapiens patatin-like phospholipase domain containing 3 (PNPLA3), mRNA [NM_025225]	80339
A_23_P159797	2.8	1.2	2.4	1.1	PNPLA4	Homo sapiens patatin-like phospholipase domain containing 4 (PNPLA4), transcript variant 1, mRNA [NM_004650]	8228

A_23_P377312	3.7	-1.1	-2.0	3.7	PNPLA5	Homo sapiens patatin-like phospholipase domain containing 5 (PNPLA5), transcript variant 1, mRNA [NM_138814]	150379
A_24_P453855	-3.0	-3.1	-4.1	-11.0	PNPLA7	patatin-like phospholipase domain containing 7 [Source:HGNC Symbol;Acc:24768] [ENST00000371450]	375775
A_23_P312718	2.3	1.3	-1.2	1.1	PNPLA8	Homo sapiens patatin-like phospholipase domain containing 8 (PNPLA8), mRNA [NM_015723]	50640

A_23_P154488	4.5	3.2	4.1	2.4	PNPT1	Homo sapiens polyribonucleotide nucleotidyltransferase 1 (PNPT1), mRNA [NM_033109]	87178
A_24_P354300	-4.3	3.6	1.4	1.3	POC1A	Homo sapiens POC1 centriolar protein homolog A (Chlamydomonas) (POC1A), transcript variant 1, mRNA [NM_015426]	25886
A_23_P212284	2.1	1.4	1.4	1.3	POC1A	Homo sapiens POC1 centriolar protein homolog A (Chlamydomonas) (POC1A), transcript variant 1, mRNA [NM_015426]	25886

A_23_P368154	-1.0	-1.1	-1.1	-8.5	PODN	Homo sapiens podocan (PODN), transcript variant 1, mRNA [NM_153703]	127435
A_23_P208567	1.1	1.0	2.6	-1.0	PODNL1	Homo sapiens podocan-like 1 (PODNL1), transcript variant 1, mRNA [NM_024825]	79883
A_23_P121037	-1.0	-1.1	-1.3	-2.5	PODXL2	Homo sapiens podocalyxin-like 2 (PODXL2), mRNA [NM_015720]	50512
A_24_P151692	1.4	2.4	1.1	2.5	POF1B	Homo sapiens premature ovarian failure, 1B (POF1B), mRNA [NM_024921]	79983
A_23_P68624	-1.2	-1.3	-3.8	-1.3	POFUT1	Homo sapiens protein O-fucosyltransferase 1 (POFUT1), transcript variant 2, mRNA [NM_172236]	23509

A_23_P137715	2.0	1.2	-1.4	-1.1	POGK	Homo sapiens pogo transposable element with KRAB domain (POGK), mRNA [NM_017542]	57645
A_23_P500734	2.5	1.7	1.4	1.3	POGZ	Homo sapiens pogo transposable element with ZNF domain (POGZ), transcript variant 1, mRNA [NM_015100]	23126
A_32_P1701	2.2	2.2	2.1	3.0	POLA1	Homo sapiens polymerase (DNA directed), alpha 1, catalytic subunit (POLA1), mRNA [NM_016937]	5422

A_23_P161615	2.8	-1.0	2.2	3.8	POLA2	Homo sapiens polymerase (DNA directed), alpha 2 (70kD subunit) (POLA2), mRNA [NM_002689]	23649
A_32_P34552	-1.4	-2.0	-3.4	-2.7	POLB	Homo sapiens polymerase (DNA directed), beta (POLB), mRNA [NM_002690]	5423
A_24_P142442	1.6	-2.4	1.1	1.1	POLDIP2	Homo sapiens polymerase (DNA-directed), delta interacting protein 2 (POLDIP2), mRNA [NM_015584]	26073

A_23_P502269	-1.6	1.2	-2.1	-2.2	POLDIP3	Homo sapiens polymerase (DNA-directed), delta interacting protein 3 (POLDIP3), transcript variant 1, mRNA [NM_032311]	84271
A_24_P918387	2.0	1.0	2.1	-1.0	POLE	polymerase (DNA directed), epsilon [Source:HGNC Symbol;Acc:9177] [ENST00000539357]	5426
A_23_P159355	-1.4	-2.9	1.3	-2.2	POLE	Homo sapiens polymerase (DNA directed), epsilon (POLE), mRNA [NM_006231]	5426

A_23_P163099	1.1	-15.2	-1.1	1.0	POLE2	Homo sapiens polymerase (DNA directed), epsilon 2 (p59 subunit) (POLE2), transcript variant 1, mRNA [NM_002692]	5427
A_23_P135326	-2.5	1.5	-2.3	-1.1	POLE3	Homo sapiens polymerase (DNA directed), epsilon 3 (p17 subunit) (POLE3), mRNA [NM_017443]	54107
A_23_P383385	-3.1	-5.8	-4.0	-2.8	POLE4	Homo sapiens polymerase (DNA-directed), epsilon 4 (p12 subunit) (POLE4), mRNA [NM_019896]	56655
A_32_P96692	1.1	1.1	-1.9	-3.4	POLH	Homo sapiens polymerase (DNA directed), eta (POLH), mRNA [NM_006502]	5429

A_23_P386450	-3.0	-1.4	-1.4	-1.6	POLK	Homo sapiens polymerase (DNA directed) kappa (POLK), mRNA [NM_016218]	51426
A_23_P97889	2.3	1.4	-1.1	3.5	POLL	Homo sapiens polymerase (DNA directed), lambda (POLL), transcript variant 2, mRNA [NM_013274]	27343
A_23_P397371	1.4	2.2	-1.9	-1.0	POLM	Homo sapiens polymerase (DNA directed), mu (POLM), mRNA [NM_013284]	27434
A_23_P333998	-1.0	-2.3	-1.1	-1.1	POLQ	Homo sapiens clone HQ0327 PRO0327 mRNA, complete cds. [AF090919]	10721

A_23_P28450	1.4	-1.1	-1.9	-2.1	POLR1A	Homo sapiens polymerase (RNA) I polypeptide A, 194kDa (POLR1A), mRNA [NM_015425]	25885
A_23_P209987	1.3	1.3	-10.4	1.1	POLR1B	Homo sapiens polymerase (RNA) I polypeptide B, 128kDa (POLR1B), transcript variant 1, mRNA [NM_019014]	84172
A_24_P942112	1.0	-4.2	1.0	1.1	POLR1B	Homo sapiens cDNA FLJ10816 fis, clone NT2RP4000997, highly similar to DNA-DIRECTED RNA POLYMERASE I 135 KD POLYPEPTIDE (EC 2.7.7.6). [AK001678]	84172

A_23_P70409	-14.9	-1.0	-1.3	1.0	POLR1C	Homo sapiens polymerase (RNA) I polypeptide C, 30kDa (POLR1C), mRNA [NM_203290]	9533
A_23_P36928	1.2	1.7	-1.1	4.1	POLR1D	Homo sapiens polymerase (RNA) I polypeptide D, 16kDa (POLR1D), transcript variant 1, mRNA [NM_015972]	51082
A_23_P409541	-2.3	1.1	1.1	-1.4	POLR1D	Homo sapiens polymerase (RNA) I polypeptide D, 16kDa (POLR1D), transcript variant 2, mRNA [NM_152705]	51082
A_23_P9458	1.1	1.1	-3.2	1.1	POLR1E	Homo sapiens polymerase (RNA) I polypeptide E, 53kDa (POLR1E), mRNA [NM_022490]	64425

A_24_P308128	1.0	1.0	1.4	2.1	POLR2A	Homo sapiens polymerase (RNA) II (DNA directed) polypeptide A, 220kDa (POLR2A), mRNA [NM_000937]	5430
A_23_P144311	1.2	2.1	-1.5	-1.1	POLR2B	Homo sapiens polymerase (RNA) II (DNA directed) polypeptide B, 140kDa (POLR2B), mRNA [NM_000938]	5431
A_32_P219753	-4.1	-1.0	-1.2	1.0	POLR2D	Homo sapiens polymerase (RNA) II (DNA directed) polypeptide D (POLR2D), mRNA [NM_004805]	5433

A_23_P165280	-1.9	2.1	2.1	-4.8	POLR2E	Homo sapiens polymerase (RNA) II (DNA directed) polypeptide E, 25kDa (POLR2E), mRNA [NM_002695]	5434
A_23_P42649	4.6	4.7	1.1	2.0	POLR2J	Homo sapiens polymerase (RNA) II (DNA directed) polypeptide J, 13.3kDa (POLR2J), mRNA [NM_006234]	5439
A_24_P292020	2.4	1.6	2.0	1.9	POLR2J	Homo sapiens polymerase (RNA) II (DNA directed) polypeptide J, 13.3kDa (POLR2J), mRNA [NM_006234]	5439

A_23_P215669	1.8	5.0	3.4	5.3	POLR2J2	Homo sapiens polymerase (RNA) II (DNA directed) polypeptide J2 (POLR2J2), mRNA [NM_032959]	246721
A_23_P157452	-1.1	2.2	1.6	-2.0	POLR2K	Homo sapiens polymerase (RNA) II (DNA directed) polypeptide K, 7.0kDa (POLR2K), mRNA [NM_005034]	5440
A_23_P157449	-2.9	1.5	1.8	-1.2	POLR2K	Homo sapiens polymerase (RNA) II (DNA directed) polypeptide K, 7.0kDa (POLR2K), mRNA [NM_005034]	5440

A_24_P85283	-1.1	-3.1	1.1	-1.1	POLR3A	Homo sapiens polymerase (RNA) III (DNA directed) polypeptide A, 155kDa (POLR3A), mRNA [NM_007055]	11128
A_23_P149834	2.4	2.8	1.0	1.5	POLR3A	Homo sapiens polymerase (RNA) III (DNA directed) polypeptide A, 155kDa (POLR3A), mRNA [NM_007055]	11128
A_23_P385529	4.1	12.1	12.1	4.6	POLR3E	Homo sapiens mRNA for KIAA1452 protein, partial cds. [AB040885]	55718

A_23_P23855	2.0	2.7	2.7	2.2	POLR3GL	Homo sapiens polymerase (RNA) III (DNA directed) polypeptide G (32kD)-like (POLR3GL), mRNA [NM_032305]	84265
A_32_P201496	-1.2	-2.2	-2.1	-1.1	POLR3H	Homo sapiens polymerase (RNA) III (DNA directed) polypeptide H (22.9kD) (POLR3H), transcript variant 2, mRNA [NM_001018052]	171568
A_24_P322847	1.0	-1.0	1.5	2.5	POLR3H	Homo sapiens polymerase (RNA) III (DNA directed) polypeptide H (22.9kD) (POLR3H), transcript variant 1, mRNA [NM_138338]	171568

A_32_P98847	1.5	1.3	-1.8	-2.2	POLRMT	Homo sapiens polymerase (RNA) mitochondrial (DNA directed) (POLRMT), nuclear gene encoding mitochondrial protein, mRNA [NM_005035]	5442
A_23_P215431	-2.9	1.1	-1.0	-1.0	POM121	POM121 membrane glycoprotein [Source:HGNC Symbol;Acc:19702] [ENST00000395270]	9883
A_23_P8541	-3.0	-1.4	-1.1	-1.2	POM121	POM121 membrane glycoprotein [Source:HGNC Symbol;Acc:19702] [ENST00000446813]	9883

A_24_P266273	1.5	-2.4	1.3	1.6	POM121	Homo sapiens POM121 membrane glycoprotein (POM121), mRNA [NM_172020]	9883
A_24_P459963	1.1	1.1	-1.0	-2.8	POM121L10P	Homo sapiens mRNA; cDNA DKFZp686I1532 (from clone DKFZp686I1532). [BX648116]	646074
A_23_P6355	2.0	2.3	3.0	2.0	POM121L1P	Homo sapiens POM121 membrane glycoprotein-like 1, pseudogene (POM121L1P), non-coding RNA [NR_024591]	25812

A_32_P14692	-1.0	1.3	-1.1	-6.6	POM121L1P	Homo sapiens POM121 membrane glycoprotein-like 1, pseudogene (POM121L1P), non-coding RNA [NR_024591]	25812
A_24_P550973	-1.3	-1.1	-2.2	-2.3	POM121L4P	Homo sapiens POM121 membrane glycoprotein-like 4 pseudogene (POM121L4P), non-coding RNA [NR_024592]	266697
A_32_P107983	-4.1	1.2	1.2	1.0	POM121L8P	Homo sapiens POM121 membrane glycoprotein-like 8 pseudogene (POM121L8P), non-coding RNA [NR_024583]	29797

A_23_P91636	12.4	12.1	11.7	12.7	POM121L9P	Homo sapiens POM121 membrane glycoprotein-like 9, pseudogene (POM121L9P), non-coding RNA [NR_003714]	29774
A_23_P96812	-2.6	1.9	-1.7	-1.3	POMGNT1	Homo sapiens protein O-linked mannose beta1,2-N-acetylglucosaminyltransferase (POMGNT1), transcript variant 1, mRNA [NM_017739]	55624
A_23_P146361	1.7	3.6	3.3	1.3	POMT1	Homo sapiens protein-O-mannosyltransferase 1 (POMT1), transcript variant 1, mRNA [NM_007171]	10585

A_23_P146354	-3.0	-9.3	-3.1	-2.8	POMT1	Homo sapiens protein-O-mannosyltransferase 1 (POMT1), transcript variant 1, mRNA [NM_007171]	10585
A_23_P168584	2.1	1.2	2.1	2.1	POMZP3	Homo sapiens POM121 and ZP3 fusion (POMZP3), transcript variant 2, mRNA [NM_152992]	22932
A_23_P168576	1.6	-1.6	-2.1	-3.3	POMZP3	Homo sapiens POM121 and ZP3 fusion (POMZP3), transcript variant 2, mRNA [NM_152992]	22932
A_23_P215549	4.2	7.6	2.2	4.2	PON3	Homo sapiens paraoxonase 3 (PON3), mRNA [NM_000940]	5446

A_23_P94133	-3.0	1.2	-1.1	1.6	POP1	Homo sapiens processing of precursor 1, ribonuclease P/MRP subunit (S. cerevisiae) (POP1), transcript variant 3, mRNA [NM_015029]	10940
A_23_P134274	1.0	1.1	2.3	1.3	POP7	Homo sapiens processing of precursor 7, ribonuclease P/MRP subunit (S. cerevisiae) (POP7), mRNA [NM_005837]	10248
A_24_P257336	-2.2	-1.8	1.2	-1.5	POPDC2	Homo sapiens popeye domain containing 2 (POPDC2), mRNA [NM_022135]	64091

A_24_P29723	2.7	-1.6	-1.6	-1.2	POR	Homo sapiens P450 (cytochrome) oxidoreductase (POR), mRNA [NM_000941]	5447
A_24_P347411	-2.8	-1.3	-4.0	-2.2	POSTN	Homo sapiens periostin, osteoblast specific factor (POSTN), transcript variant 1, mRNA [NM_006475]	10631
A_32_P9455	-1.8	1.3	4.3	2.9	POTEE	Homo sapiens POTE ankyrin domain family, member E (POTEE), mRNA [NM_001083538]	445582
A_24_P825874	-1.0	-1.9	-2.3	-1.1	POTEE	Homo sapiens POTE ankyrin domain family, member E (POTEE), mRNA [NM_001083538]	445582

A_32_P16323	1.0	-1.0	-2.3	1.5	POTEG	POTE ankyrin domain family, member G [Source:HGNC Symbol;Acc:33896] [ENST00000409832]	404785
A_32_P43588	8.5	2.1	7.2	8.5	POTEG	Homo sapiens POTE ankyrin domain family, member G (POTEG), transcript variant 1, mRNA [NM_001005356]	404785
A_32_P155776	2.8	2.7	-1.4	2.5	POTEKP	Homo sapiens POTE ankyrin domain family, member K, pseudogene (POTEKP), non-coding RNA [NR_033885]	440915

A_23_P155539	-2.6	1.0	1.0	1.1	POU1F1	Homo sapiens POU class 1 homeobox 1 (POU1F1), transcript variant alpha, mRNA [NM_000306]	5449
A_24_P929909	5.9	5.8	6.0	4.6	POU1F1	Homo sapiens POU class 1 homeobox 1 (POU1F1), transcript variant beta, mRNA [NM_001122757]	5449
A_23_P312920	1.1	-15.0	1.2	1.2	POU2AF1	Homo sapiens POU class 2 associating factor 1 (POU2AF1), mRNA [NM_006235]	5450
A_24_P296792	2.2	1.9	1.0	2.6	POU2F2	Homo sapiens POU class 2 homeobox 2 (POU2F2), transcript variant 2, mRNA [NM_002698]	5452

A_24_P131293	-1.5	-1.8	-1.9	-2.5	POU3F1	Homo sapiens POU class 3 homeobox 1 (POU3F1), mRNA [NM_002699]	5453
A_23_P97034	1.1	-2.8	1.1	1.1	POU3F1	Homo sapiens POU class 3 homeobox 1 (POU3F1), mRNA [NM_002699]	5453
A_24_P34575	1.4	2.1	1.0	1.3	POU3F3	Homo sapiens POU class 3 homeobox 3 (POU3F3), mRNA [NM_006236]	5455
A_23_P114210	3.7	5.1	3.5	1.4	POU3F4	Homo sapiens POU class 3 homeobox 4 (POU3F4), mRNA [NM_000307]	5456

A_23_P302038	2.6	3.0	-44.3	2.3	POU4F2	Homo sapiens POU class 4 homeobox 2 (POU4F2), mRNA [NM_004575]	5458
A_23_P121976	1.9	1.0	-3.3	-1.0	POU4F3	Homo sapiens POU class 4 homeobox 3 (POU4F3), mRNA [NM_002700]	5459
A_24_P144601	1.7	1.2	3.4	1.2	POU5F1	Homo sapiens POU class 5 homeobox 1 (POU5F1), transcript variant 1, mRNA [NM_002701]	5460
A_23_P59138	-2.3	-1.0	-1.1	-1.1	POU5F1	Homo sapiens POU class 5 homeobox 1 (POU5F1), transcript variant 1, mRNA [NM_002701]	5460

A_24_P214841	1.1	-17.9	1.2	1.8	POU5F1	Homo sapiens POU class 5 homeobox 1 (POU5F1), transcript variant 1, mRNA [NM_002701]	5460
A_24_P383640	3.5	2.2	4.4	3.3	POU5F1P3	Homo sapiens POU class 5 homeobox 1 pseudogene 3 (POU5F1P3), non-coding RNA [NR_036440]	642559
A_24_P340659	1.3	3.5	2.6	-2.9	POU5F1P4	Homo sapiens POU class 5 homeobox 1 pseudogene 4 (POU5F1P4), non-coding RNA [NR_034180]	645682

A_23_P358564	-7.5	-1.0	-1.1	1.0	POU5F2	Homo sapiens POU domain class 5, transcription factor 2 (POU5F2), mRNA [NM_153216]	134187
A_23_P147900	-2.9	1.8	1.5	-1.5	POU6F2	Homo sapiens POU class 6 homeobox 2 (POU6F2), transcript variant 1, mRNA [NM_007252]	11281
A_23_P325572	-1.0	-1.1	-2.3	-1.0	PP2D1	Homo sapiens protein phosphatase 2C-like domain containing 1 (PP2D1), non-coding RNA [NR_027694]	151649

A_24_P419300	-2.0	1.0	1.0	1.1	PP7080	Homo sapiens uncharacterized LOC25845 (PP7080), non-coding RNA [NR_024158]	25845
A_24_P916494	-3.3	-1.4	-1.5	-1.9	PPA2	Homo sapiens pyrophosphatase (inorganic) 2, mRNA (cDNA clone IMAGE:3866266), with apparent retained intron. [BC008246]	27068
A_24_P214625	-1.5	-1.5	-1.5	4.2	PPA2	Homo sapiens pyrophosphatase (inorganic) 2 (PPA2), nuclear gene encoding mitochondrial protein, transcript variant 1, mRNA [NM_176869]	27068

A_23_P201808	3.9	2.5	4.6	2.2	PPAP2B	Homo sapiens phosphatidic acid phosphatase type 2B (PPAP2B), mRNA [NM_003713]	8613
A_23_P108294	1.6	-1.2	-2.6	1.2	PPAP2C	Homo sapiens phosphatidic acid phosphatase type 2C (PPAP2C), transcript variant 3, mRNA [NM_177543]	8612
A_24_P252130	-22.4	1.1	1.0	1.0	PPARD	Homo sapiens peroxisome proliferator-activated receptor delta (PPARD), transcript variant 1, mRNA [NM_006238]	5467

A_24_P560519	-2.0	1.4	-2.2	1.6	PPARGC1B	Homo sapiens peroxisome proliferator-activated receptor gamma, coactivator 1 beta (PPARGC1B), transcript variant 1, mRNA [NM_133263]	133522
A_23_P213959	-1.0	-1.0	-1.2	-2.5	PPARGC1B	Homo sapiens peroxisome proliferator-activated receptor gamma, coactivator 1 beta (PPARGC1B), transcript variant 1, mRNA [NM_133263]	133522
A_24_P123347	4.0	1.6	3.2	2.5	PPAT	Homo sapiens phosphoribosyl pyrophosphate amidotransferase (PPAT), mRNA [NM_002703]	5471

A_23_P163428	-2.7	1.6	1.3	-1.0	PPCDC	Homo sapiens phosphopantothenoylcysteine decarboxylase (PPCDC), mRNA [NM_021823]	60490
A_24_P327011	1.1	-3.1	-1.0	1.0	PPCDC	Homo sapiens phosphopantothenoylcysteine decarboxylase (PPCDC), mRNA [NM_021823]	60490
A_23_P120364	-1.0	6.7	1.2	-1.3	PPDPF	Homo sapiens pancreatic progenitor cell differentiation and proliferation factor homolog (zebrafish) (PPDPF), mRNA [NM_024299]	79144

A_23_P75516	1.0	1.0	1.1	-5.7	PPFIA1	Homo sapiens protein tyrosine phosphatase, receptor type, f polypeptide (PTPRF), interacting protein (liprin), alpha 1 (PPFIA1), transcript variant 2, mRNA [NM_003626]	8500
A_23_P47867	1.0	4.4	-11.5	1.0	PPFIBP1	Homo sapiens PTPRF interacting protein, binding protein 1 (liprin beta 1) (PPFIBP1), transcript variant 1, mRNA [NM_003622]	8496

A_23_P409623	-2.3	-1.0	-1.0	-1.6	PPFIBP2	Homo sapiens PTPRF interacting protein, binding protein 2 (liprin beta 2) (PPFIBP2), mRNA [NM_003621]	8495
A_24_P222454	1.2	1.1	1.0	-2.0	PPHLN1	Homo sapiens periphilin 1 (PPHLN1), transcript variant 5, mRNA [NM_201438]	51535
A_24_P772061	-1.9	-1.8	-2.6	-1.8	PPIA	Homo sapiens peptidylprolyl isomerase A (cyclophilin A) (PPIA), mRNA [NM_021130]	5478
A_32_P68459	-1.5	5.4	2.4	-1.3	PPIA	Homo sapiens peptidylprolyl isomerase A (cyclophilin A) (PPIA), mRNA [NM_021130]	5478

A_24_P340679	2.0	1.4	-1.7	1.4	PPIA	Homo sapiens peptidylprolyl isomerase A (cyclophilin A) (PPIA), mRNA [NM_021130]	5478
A_24_P264772	-2.6	-1.4	-1.5	-1.5	PPIAL4A	Homo sapiens peptidylprolyl isomerase A (cyclophilin A)-like 4A (PPIAL4A), mRNA [NM_178230]	164022
A_24_P203226	-1.0	-13.2	-1.2	-1.1	PPIAL4A	Homo sapiens peptidylprolyl isomerase A (cyclophilin A)-like 4A (PPIAL4A), mRNA [NM_178230]	164022

A_24_P144499	1.0	-3.7	-1.1	-2.8	PPIAL4A	Homo sapiens peptidylprolyl isomerase A (cyclophilin A)-like 4A (PPIAL4A), mRNA [NM_178230]	164022
A_23_P422724	-16.8	1.0	1.0	1.1	PPIC	Homo sapiens peptidylprolyl isomerase C (cyclophilin C) (PPIC), mRNA [NM_000943]	5480
A_23_P28213	2.7	2.1	2.1	2.1	PPIL3	Homo sapiens peptidylprolyl isomerase (cyclophilin)-like 3 (PPIL3), transcript variant PPIL3a, mRNA [NM_032472]	53938

A_23_P42507	1.7	-1.4	2.2	-1.7	PPIL4	Homo sapiens peptidylprolyl isomerase (cyclophilin)-like 4 (PPIL4), mRNA [NM_139126]	85313
A_24_P925389	-1.0	-1.0	-3.8	-1.0	PPIL6	Homo sapiens peptidylprolyl isomerase (cyclophilin)-like 6, mRNA (cDNA clone IMAGE:4798147), complete cds. [BC026359]	285755
A_23_P106906	1.8	1.7	1.6	2.7	PPL	Homo sapiens periplakin (PPL), mRNA [NM_002705]	5493

A_24_P688133	2.0	2.0	2.0	2.0	PPM1A	Homo sapiens protein phosphatase, Mg2+/Mn2+ dependent, 1A (PPM1A), transcript variant 1, mRNA [NM_021003]	5494
A_23_P398073	-1.2	-1.6	-2.8	1.1	PPM1B	Homo sapiens protein phosphatase, Mg2+/Mn2+ dependent, 1B (PPM1B), transcript variant 2, mRNA [NM_177968]	5495
A_32_P150030	-1.8	-1.8	-1.2	-2.0	PPM1D	Homo sapiens protein phosphatase, Mg2+/Mn2+ dependent, 1D (PPM1D), mRNA [NM_003620]	8493

A_23_P49532	31.7	30.9	15.8	22.6	PPM1E	Homo sapiens protein phosphatase, Mg2+/Mn 2+ dependent, 1E (PPM1E), mRNA [NM_014906]	22843
A_24_P214598	1.4	1.4	2.7	1.7	PPM1K	Homo sapiens protein phosphatase, Mg2+/Mn 2+ dependent, 1K (PPM1K), nuclear gene encoding mitochondrial protein, mRNA [NM_152542]	152926
A_32_P429876	1.0	-1.0	-1.0	-3.5	PPM1L	protein phosphatase, Mg2+/Mn 2+ dependent, 1L [Source:HGNC Symbol;Acc:16381] [ENST00000498165]	151742

A_23_P147563	1.0	-1.1	-1.1	-6.6	PPM1L	Homo sapiens protein phosphatase, Mg2+/Mn2+ dependent, 1L (PPM1L), mRNA [NM_139245]	151742
A_23_P258393	1.0	1.3	2.9	1.5	PPM1M	Homo sapiens protein phosphatase, Mg2+/Mn2+ dependent, 1M (PPM1M), transcript variant 1, mRNA [NM_144641]	132160
A_24_P4701	-1.0	-1.0	-54.1	-1.1	PPME1	Homo sapiens protein phosphatase methylesterase 1 (PPME1), mRNA [NM_016147]	51400

A_24_P102726	2.8	2.8	2.6	2.7	PPOX	Homo sapiens protoporphyrinogen oxidase (PPOX), nuclear gene encoding mitochondrial protein, transcript variant 1, mRNA [NM_000309]	5498
A_23_P201400	1.6	2.3	1.4	1.7	PPOX	Homo sapiens protoporphyrinogen oxidase (PPOX), nuclear gene encoding mitochondrial protein, transcript variant 1, mRNA [NM_000309]	5498

A_23_P323227	-4.7	-1.2	-3.6	1.1	PPP1CA	Homo sapiens protein phosphatase 1, catalytic subunit, alpha isozyme (PPP1CA), transcript variant 3, mRNA [NM_001008709]	5499
A_23_P434710	-1.3	-2.4	-1.3	1.1	PPP1CA	Homo sapiens protein phosphatase 1, catalytic subunit, alpha isozyme (PPP1CA), transcript variant 3, mRNA [NM_001008709]	5499
A_23_P258641	3.0	3.1	-1.8	-16.0	PPP1CB	Homo sapiens protein phosphatase 1, catalytic subunit, beta isozyme (PPP1CB), transcript variant 1, mRNA [NM_002709]	5500

A_24_P405298	-2.0	-2.0	-2.0	-2.1	PPP1CB	Homo sapiens protein phosphatase 1, catalytic subunit, beta isozyme (PPP1CB), transcript variant 1, mRNA [NM_002709]	5500
A_23_P156667	1.9	4.3	4.6	3.7	PPP1R10	Homo sapiens protein phosphatase 1, regulatory subunit 10 (PPP1R10), mRNA [NM_002714]	5514
A_24_P363315	1.7	1.9	1.1	-2.1	PPP1R11	Homo sapiens protein phosphatase 1, regulatory (inhibitor) subunit 11 (PPP1R11), mRNA [NM_021959]	6992

A_23_P19437	2.5	2.5	1.6	-1.8	PPP1R11	Homo sapiens protein phosphatase 1, regulatory (inhibitor) subunit 11 (PPP1R11), mRNA [NM_021959]	6992
A_23_P12435	-1.0	-2.0	-1.1	-1.1	PPP1R12B	Homo sapiens protein phosphatase 1, regulatory subunit 12B (PPP1R12B), transcript variant 3, mRNA [NM_032103]	4660
A_23_P205273	1.7	2.2	2.0	-1.2	PPP1R13B	Homo sapiens protein phosphatase 1, regulatory subunit 13B (PPP1R13B), mRNA [NM_015316]	23368

A_23_P119095	-1.3	-1.4	-2.5	-2.2	PPP1R13L	Homo sapiens protein phosphatase 1, regulatory subunit 13 like (PPP1R13L), transcript variant 2, mRNA [NM_006663]	10848
A_23_P45011	-4.3	-4.5	-5.0	-4.7	PPP1R14C	Homo sapiens protein phosphatase 1, regulatory (inhibitor) subunit 14C (PPP1R14C), mRNA [NM_030949]	81706
A_23_P90172	-6.2	1.1	-1.1	-1.0	PPP1R15A	Homo sapiens protein phosphatase 1, regulatory subunit 15A (PPP1R15A), mRNA [NM_014330]	23645

A_23_P160449	-1.2	3.0	1.4	3.2	PPP1R15B	Homo sapiens protein phosphatase 1, regulatory subunit 15B (PPP1R15B), mRNA [NM_032833]	84919
A_24_P9605	1.6	-1.2	1.4	3.3	PPP1R16A	Homo sapiens protein phosphatase 1, regulatory subunit 16A (PPP1R16A), mRNA [NM_032902]	84988
A_23_P157715	2.3	-1.1	-2.1	-1.1	PPP1R16A	Homo sapiens protein phosphatase 1, regulatory subunit 16A (PPP1R16A), mRNA [NM_032902]	84988

A_23_P352535	-2.4	2.2	1.3	-3.5	PPP1R16B	Homo sapiens protein phosphatase 1, regulatory subunit 16B (PPP1R16B), transcript variant 1, mRNA [NM_015568]	26051
A_24_P13285	1.6	1.0	-13.2	1.0	PPP1R1A	Homo sapiens protein phosphatase 1, regulatory (inhibitor) subunit 1A (PPP1R1A), mRNA [NM_006741]	5502
A_23_P129835	2.1	1.9	-2.1	1.6	PPP1R1B	Homo sapiens protein phosphatase 1, regulatory (inhibitor) subunit 1B (PPP1R1B), transcript variant 1, mRNA [NM_032192]	84152

A_32_P17133	1.1	1.1	-2.0	1.8	PPP1R2	Homo sapiens protein phosphatase 1, regulatory (inhibitor) subunit 2 (PPP1R2), mRNA [NM_006241]	5504
A_23_P90895	-3.6	-3.6	-4.1	-5.9	PPP1R21	Homo sapiens protein phosphatase 1, regulatory subunit 21 (PPP1R21), transcript variant 2, mRNA [NM_152994]	129285
A_23_P146497	2.0	1.2	1.9	1.1	PPP1R26	Homo sapiens protein phosphatase 1, regulatory subunit 26 (PPP1R26), mRNA [NM_014811]	9858

A_23_P98571	1.1	-4.7	-1.1	1.0	PPP1R32	Homo sapiens protein phosphatase 1, regulatory subunit 32 (PPP1R32), nuclear gene encoding mitochondrial protein, transcript variant 1, mRNA [NM_145017]	220004
A_23_P101303	-1.0	-3.0	-1.1	-1.1	PPP1R37	Homo sapiens protein phosphatase 1, regulatory subunit 37 (PPP1R37), mRNA [NM_019121]	284352
A_24_P324326	1.0	-1.0	2.3	1.0	PPP1R37	Homo sapiens protein phosphatase 1, regulatory subunit 37 (PPP1R37), mRNA [NM_019121]	284352

A_23_P216199	1.2	-3.6	1.0	1.0	PPP1R3B	Homo sapiens protein phosphatase 1, regulatory subunit 3B (PPP1R3B), transcript variant 2, mRNA [NM_024607]	79660
A_23_P428640	3.1	3.0	2.7	2.8	PPP1R3E	Homo sapiens protein phosphatase 1, regulatory subunit 3E (PPP1R3E), non-coding RNA [NR_026862]	90673
A_23_P125533	-1.1	-4.9	1.1	-2.8	PPP1R3F	Homo sapiens protein phosphatase 1, regulatory subunit 3F (PPP1R3F), transcript variant 1, mRNA [NM_033215]	89801

A_23_P125541	-1.2	1.7	2.4	2.4	PPP1R3F	Homo sapiens protein phosphatase 1, regulatory subunit 3F (PPP1R3F), transcript variant 1, mRNA [NM_033215]	89801
A_23_P257063	-9.5	-1.0	-1.1	1.0	PPP1R42	leucine rich repeat containing 67 [Source:HGNC Symbol;Acc:33732] [ENST00000421742]	286187
A_23_P154199	-1.2	-11.5	-1.4	-1.5	PPP1R7	Homo sapiens protein phosphatase 1, regulatory subunit 7 (PPP1R7), mRNA [NM_002712]	5510

A_23_P308097	1.1	1.0	-3.5	-3.1	PPP2CB	Homo sapiens protein phosphatase 2, catalytic subunit, beta isozyme (PPP2CB), mRNA [NM_001009552]	5516
A_23_P107661	3.1	3.8	1.2	2.2	PPP2R1A	Homo sapiens protein phosphatase 2, regulatory subunit A, alpha (PPP2R1A), transcript variant 1, mRNA [NM_014225]	5518
A_24_P107291	2.5	2.3	1.9	-1.8	PPP2R1B	Homo sapiens protein phosphatase 2, regulatory subunit A, beta (PPP2R1B), transcript variant 2, mRNA [NM_181699]	5519

A_23_P52793	1.0	-1.7	2.1	-2.8	PPP2R1B	Homo sapiens protein phosphatase 2, regulatory subunit A, beta (PPP2R1B), transcript variant 1, mRNA [NM_002716]	5519
A_23_P123539	-3.4	4.0	4.2	3.3	PPP2R2A	Homo sapiens protein phosphatase 2, regulatory subunit B, alpha (PPP2R2A), transcript variant 1, mRNA [NM_002717]	5520
A_24_P346644	-5.7	1.0	-1.1	1.1	PPP2R2A	Homo sapiens protein phosphatase 2, regulatory subunit B, alpha (PPP2R2A), transcript variant 1, mRNA [NM_002717]	5520

A_24_P3415	-1.3	-1.2	-3.5	-1.3	PPP2R2C	Homo sapiens protein phosphatase 2, regulatory subunit B, gamma (PPP2R2C), transcript variant 2, mRNA [NM_181876]	5522
A_23_P135778	3.2	-1.1	1.2	2.3	PPP2R2D	Homo sapiens protein phosphatase 2, regulatory subunit B, delta (PPP2R2D), transcript variant 1, mRNA [NM_018461]	55844
A_23_P10401	1.0	1.8	-1.1	-2.5	PPP2R3A	Homo sapiens protein phosphatase 2, regulatory subunit B'', alpha (PPP2R3A), transcript variant 1, mRNA [NM_002718]	5523

A_23_P45517	1.4	3.3	1.4	2.5	PPP2R3B	Homo sapiens protein phosphatase 2, regulatory subunit B'', beta (PPP2R3B), mRNA [NM_013239]	28227
A_23_P60458	1.4	3.5	3.0	1.2	PPP2R4	Homo sapiens protein phosphatase 2A activator, regulatory subunit 4 (PPP2R4), transcript variant 1, mRNA [NM_178001]	5524
A_23_P256432	1.7	1.8	1.6	2.0	PPP2R5A	Homo sapiens protein phosphatase 2, regulatory subunit B', alpha (PPP2R5A), transcript variant 1, mRNA [NM_006243]	5525

A_23_P35796	2.0	1.9	1.8	1.8	PPP2R5B	Homo sapiens protein phosphatase 2, regulatory subunit B', beta (PPP2R5B), mRNA [NM_006244]	5526
A_24_P76898	1.2	2.6	5.9	3.0	PPP2R5C	protein phosphatase 2, regulatory subunit B', gamma [Source:HGNC Symbol;Acc:9311] [ENST00000556068]	5527
A_24_P341370	3.4	2.5	1.4	1.1	PPP2R5C	protein phosphatase 2, regulatory subunit B', gamma [Source:HGNC Symbol;Acc:9311] [ENST00000556068]	5527

A_24_P5026	-3.5	1.0	-1.0	1.0	PPP2R5E	protein phosphatase 2, regulatory subunit B', epsilon isoform [Source:HGNC Symbol;Acc:9313] [ENST00000337537]	5529
A_23_P9348	1.6	3.9	1.5	1.1	PPP3R2	Homo sapiens protein phosphatase 3, regulatory subunit B, beta (PPP3R2), mRNA [NM_147180]	5535
A_24_P271014	1.9	2.7	-3.2	1.0	PPP4R1	Homo sapiens protein phosphatase 4, regulatory subunit 1 (PPP4R1), transcript variant 1, mRNA [NM_001042388]	9989

A_24_P65851	1.1	1.1	-2.6	1.1	PPP4R1L	Homo sapiens protein phosphatase 4, regulatory subunit 1-like (PPP4R1L), non-coding RNA [NR_003505]	55370
A_24_P400729	4.0	3.9	3.5	3.6	PPP4R2	Homo sapiens protein phosphatase 4, regulatory subunit 2 (PPP4R2), mRNA [NM_174907]	151987
A_32_P228775	-1.2	1.3	-1.7	-3.3	PPP4R2	Homo sapiens protein phosphatase 4, regulatory subunit 2 (PPP4R2), mRNA [NM_174907]	151987

A_24_P186174	-3.0	2.9	2.6	1.0	PPP6R2	Homo sapiens protein phosphatase 6, regulatory subunit 2 (PPP6R2), transcript variant 3, mRNA [NM_014678]	9701
A_24_P922808	-1.4	-4.0	1.1	1.7	PPPDE1	PPPDE peptidase domain containing 1 [Source:HGNC Symbol;Acc:24264] [ENST00000484738]	51029
A_23_P201445	-1.0	-1.0	1.0	-17.3	PPPDE1	Homo sapiens PPPDE peptidase domain containing 1 (PPPDE1), mRNA [NM_016076]	51029
A_23_P356330	3.7	5.2	-1.5	1.3	PPPDE2	Homo sapiens PPPDE peptidase domain containing 2 (PPPDE2), mRNA [NM_015704]	27351

A_23_P68966	1.0	-1.0	-4.1	-1.1	PPPDE2	Homo sapiens PPPDE peptidase domain containing 2 (PPPDE2), mRNA [NM_015704]	27351
A_23_P306933	-1.6	-2.3	-2.5	-3.3	PPTC7	Homo sapiens PTC7 protein phosphatase homolog (S. cerevisiae) (PPTC7), mRNA [NM_139283]	160760
A_23_P110433	1.9	2.2	1.7	1.1	PPWD1	Homo sapiens peptidylprolyl isomerase domain and WD repeat containing 1 (PPWD1), mRNA [NM_015342]	23398

A_23_P62139	1.1	1.0	-17.9	1.1	PQBP1	Homo sapiens polyglutamine binding protein 1 (PQBP1), transcript variant 8, mRNA [NM_001167989]	10084
A_23_P405239	3.2	1.4	1.6	2.2	PQLC2	Homo sapiens cDNA FLJ20320 fis, clone HEP08923. [AK000327]	54896
A_23_P131596	2.1	1.3	-1.3	2.5	PRADC1	Homo sapiens protease-associated domain containing 1 (PRADC1), mRNA [NM_032319]	84279
A_23_P96350	-1.2	1.2	1.1	-10.0	PRAF2	Homo sapiens PRA1 domain family, member 2 (PRAF2), mRNA [NM_007213]	11230

A_23_P165219	1.1	-1.6	-2.1	1.4	PRAM1	Homo sapiens PML-RARA regulated adaptor molecule 1 (PRAM1), mRNA [NM_032152]	84106
A_23_P166360	-2.5	1.0	-1.1	-1.1	PRAME	Homo sapiens preferentially expressed antigen in melanoma (PRAME), transcript variant 5, mRNA [NM_206956]	23532
A_24_P216361	-4.0	-1.0	-1.1	-1.1	PRAME	Homo sapiens preferentially expressed antigen in melanoma (PRAME), transcript variant 5, mRNA [NM_206956]	23532

A_23_P126658	-5.5	1.0	-1.1	-1.0	PRAMEF8	Homo sapiens PRAME family member 8 (PRAMEF8), mRNA [NM_001012276]	391002
A_23_P128263	4.0	1.8	1.3	3.7	PRB1	Homo sapiens proline-rich protein BstNI subfamily 1 (PRB1), transcript variant 1, mRNA [NM_005039]	5542
A_23_P139434	-1.0	-1.0	-2.3	-1.1	PRB2	Homo sapiens proline-rich protein BstNI subfamily 2 (PRB2), mRNA [NM_006248]	653247
A_23_P116890	3.4	2.9	1.5	1.5	PRB3	Homo sapiens proline-rich protein BstNI subfamily 3 (PRB3), mRNA [NM_006249]	5544

A_23_P116872	2.0	1.7	2.6	-1.3	PRB4	Homo sapiens proline-rich protein BstNI subfamily 4 (PRB4), mRNA [NM_002723]	5545
A_23_P23411	1.8	2.7	1.8	1.5	PRCC	Homo sapiens papillary renal cell carcinoma (translocation-associated) (PRCC), mRNA [NM_005973]	5546
A_32_P228699	-4.5	-1.8	-1.2	-1.2	PRDM10	Homo sapiens PR domain containing 10 (PRDM10), transcript variant 2, mRNA [NM_199437]	56980
A_24_P9833	1.6	-5.3	-2.6	-1.1	PRDM11	Homo sapiens PR domain containing 11 (PRDM11), mRNA [NM_020229]	56981

A_23_P13057	1.4	1.4	2.2	1.4	PRDM11	Homo sapiens PR domain containing 11 (PRDM11), mRNA [NM_020229]	56981
A_23_P43317	6.4	6.5	6.2	1.1	PRDM12	Homo sapiens PR domain containing 12 (PRDM12), mRNA [NM_021619]	59335
A_23_P123488	1.3	-19.3	-1.3	1.0	PRDM14	Homo sapiens PR domain containing 14 (PRDM14), mRNA [NM_024504]	63978
A_32_P225816	1.0	-2.3	-1.0	1.3	PRDM16	Homo sapiens PR domain containing 16 (PRDM16), transcript variant 1, mRNA [NM_022114]	63976

A_23_P117146	-1.0	1.4	2.2	2.1	PRDM4	Homo sapiens PR domain containing 4 (PRDM4), mRNA [NM_012406]	11108
A_23_P7612	2.1	2.8	2.3	2.3	PRDM9	Homo sapiens PR domain containing 9 (PRDM9), mRNA [NM_020227]	56979
A_24_P168416	-1.2	1.6	-2.6	1.4	PRDX2	Homo sapiens peroxiredoxin 2 (PRDX2), nuclear gene encoding mitochondrial protein, transcript variant 3, mRNA [NM_181738]	7001

A_23_P142045	-1.6	-3.0	-3.3	-5.7	PRDX2	Homo sapiens peroxiredoxin 2 (PRDX2), nuclear gene encoding mitochondrial protein, transcript variant 1, mRNA [NM_005809]	7001
A_24_P155378	21.1	21.3	20.1	11.9	PRDX5	Homo sapiens peroxiredoxin 5 (PRDX5), nuclear gene encoding mitochondrial protein, transcript variant 1, mRNA [NM_012094]	25824
A_23_P91114	-3.6	-1.0	1.6	1.7	PREB	Homo sapiens prolactin regulatory element binding (PREB), mRNA [NM_013388]	10113

A_23_P51499	1.6	-1.3	1.9	-2.4	PRELP	Homo sapiens proline/arginine-rich end leucine-rich repeat protein (PRELP), transcript variant 1, mRNA [NM_002725]	5549
A_23_P165325	2.1	1.6	1.1	-1.1	PREPL	Homo sapiens prolyl endopeptidase-like (PREPL), transcript variant 1, mRNA [NM_006036]	9581
A_23_P413641	1.1	-2.5	-2.7	1.1	PREX1	Homo sapiens phosphatidylinositol-3,4,5-trisphosphate-dependent Rac exchange factor 1 (PREX1), mRNA [NM_020820]	57580

A_23_P134887	2.6	3.3	2.0	2.3	PREX2	phosphatidylinositol-3,4,5-trisphosphate-dependent Rac exchange factor 2 [Source:HGNC Symbol;Acc:22950] [ENST00000529398]	80243
A_23_P1473	1.1	-1.8	2.2	2.1	PRF1	Homo sapiens perforin 1 (pore forming protein) (PRF1), transcript variant 1, mRNA [NM_005041]	5551
A_23_P218002	-1.3	-4.0	-1.0	-1.0	PRG2	Homo sapiens proteoglycan 2, bone marrow (natural killer cell activator, eosinophil granule major basic protein) (PRG2), transcript variant 1, mRNA [NM_002728]	5553

A_23_P160286	-4.8	2.5	-12.5	2.1	PRG4	Homo sapiens proteoglycan 4 (PRG4), transcript variant A, mRNA [NM_005807]	10216
A_24_P79199	2.4	3.1	3.9	-1.0	PRIC285	Homo sapiens peroxisomal proliferator-activated receptor A interacting complex 285 (PRIC285), transcript variant 2, mRNA [NM_033405]	85441
A_24_P106502	2.2	1.8	1.8	1.8	PRICKLE4	Homo sapiens prickle homolog 4 (Drosophila) (PRICKLE4), mRNA [NM_013397]	29964

A_23_P60811	2.4	2.7	2.3	-1.3	PRKAA2	Homo sapiens protein kinase, AMP-activated, alpha 2 catalytic subunit (PRKAA2), mRNA [NM_006252]	5563
A_23_P52109	1.0	-4.3	-1.0	1.7	PRKAA2	Homo sapiens protein kinase, AMP-activated, alpha 2 catalytic subunit (PRKAA2), mRNA [NM_006252]	5563
A_23_P204503	1.7	1.7	1.7	-3.4	PRKAB1	Homo sapiens protein kinase, AMP-activated, beta 1 non-catalytic subunit (PRKAB1), mRNA [NM_006253]	5564

A_23_P350704	1.2	-1.4	-2.3	-1.8	PRKAB2	Homo sapiens protein kinase, AMP-activated, beta 2 non-catalytic subunit (PRKAB2), mRNA [NM_005399]	5565
A_24_P399630	2.2	1.3	1.1	-1.8	PRKACA	Homo sapiens protein kinase, cAMP-dependent, catalytic, alpha (PRKACA), transcript variant 1, mRNA [NM_002730]	5566
A_24_P408206	-1.0	-1.1	1.4	-7.2	PRKACA	Homo sapiens protein kinase, cAMP-dependent, catalytic, alpha (PRKACA), transcript variant 1, mRNA [NM_002730]	5566

A_23_P371410	-2.0	1.9	2.5	3.0	PRKACB	Homo sapiens protein kinase, cAMP-dependent, catalytic, beta (PRKACB), transcript variant 3, mRNA [NM_207578]	5567
A_24_P62708	3.4	3.1	3.0	3.2	PRKACB	Homo sapiens protein kinase, cAMP-dependent, catalytic, beta (PRKACB), transcript variant 2, mRNA [NM_002731]	5567
A_23_P36513	1.3	2.2	-4.0	-1.3	PRKAG1	Homo sapiens protein kinase, AMP-activated, gamma 1 non-catalytic subunit (PRKAG1), transcript variant 4, mRNA [NM_001206710]	5571

A_24_P216501	-1.6	3.2	-2.9	3.2	PRKAR1B	Homo sapiens protein kinase, cAMP-dependent, regulatory, type I, beta (PRKAR1B), transcript variant 2, mRNA [NM_002735]	5575
A_23_P203475	2.0	2.3	1.8	2.1	PRKCDBP	Homo sapiens protein kinase C, delta binding protein (PRKCDBP), mRNA [NM_145040]	112464
A_23_P348194	-1.4	-1.3	-2.8	-1.4	PRKCE	Homo sapiens protein kinase C, epsilon (PRKCE), mRNA [NM_005400]	5581
A_23_P16189	3.8	2.8	1.3	2.4	PRKCG	Homo sapiens protein kinase C, gamma (PRKCG), mRNA [NM_002739]	5582

A_23_P205567	8.7	7.5	10.2	7.1	PRKCH	Homo sapiens protein kinase C, eta (PRKCH), mRNA [NM_006255]	5583
A_24_P937915	2.2	1.3	1.6	6.9	PRKCH	PKC- eta b=protein kinase C eta b [human, monoblastoid U937 cells, mRNA Partial, 306 nt]. [S74620]	5583
A_24_P461118	-1.6	-1.7	-3.0	-1.9	PRKCI	Homo sapiens protein kinase C, iota (PRKCI), mRNA [NM_002740]	5584
A_23_P18392	1.0	-5.4	1.0	1.0	PRKCI	Homo sapiens protein kinase C, iota (PRKCI), mRNA [NM_002740]	5584

A_24_P396197	1.1	1.1	-1.7	2.3	PRKCSH	Homo sapiens protein kinase C substrate 80K-H (PRKCSH), transcript variant 1, mRNA [NM_002743]	5589
A_24_P924462	2.8	1.4	-1.0	1.4	PRKCZ	Homo sapiens mRNA, chromosome 1 specific transcript KIAA0505. [AB007974]	5590
A_23_P78802	-1.1	-1.2	1.3	-2.3	PRKD2	Homo sapiens protein kinase D2 (PRKD2), transcript variant 1, mRNA [NM_016457]	25865
A_24_P162911	-2.6	-1.0	-1.1	-1.0	PRKD3	Homo sapiens protein kinase D3 (PRKD3), mRNA [NM_005813]	23683

A_32_P25204	-2.7	1.0	-1.2	-1.4	PRKDC	Homo sapiens protein kinase, DNA-activated, catalytic polypeptide (PRKDC), transcript variant 1, mRNA [NM_006904]	5591
A_23_P158811	-1.4	-2.9	-1.5	-1.5	PRKG1	Homo sapiens protein kinase, cGMP-dependent, type I (PRKG1), transcript variant 2, mRNA [NM_006258]	5592
A_24_P250765	-1.0	-4.8	-1.0	1.0	PRKG1	Homo sapiens protein kinase, cGMP-dependent, type I (PRKG1), transcript variant 2, mRNA [NM_006258]	5592

A_23_P69663	-2.2	1.3	-1.7	1.2	PRKG2	Homo sapiens protein kinase, cGMP-dependent, type II (PRKG2), mRNA [NM_006259]	5593
A_23_P203686	1.1	-1.3	-1.0	-6.0	PRKRIR	protein-kinase, interferon-inducible double stranded RNA dependent inhibitor, repressor of (P58 repressor) [Source:HGNC Symbol;Acc:9440] [ENST00000260045]	5612
A_23_P143006	3.2	4.2	1.9	2.9	PRLH	Homo sapiens prolactin releasing hormone (PRLH), mRNA [NM_015893]	51052
A_23_P305470	-4.0	1.3	1.4	-2.7	PRM3	Homo sapiens protamine 3 (PRM3), mRNA [NM_021247]	58531

A_24_P298420	-1.9	-2.3	-2.3	-1.3	PRMT5	Homo sapiens protein arginine methyltransferase 5 (PRMT5), transcript variant 2, mRNA [NM_001039619]	10419
A_23_P77437	1.0	-1.0	-2.4	-1.0	PRMT7	Homo sapiens protein arginine methyltransferase 7 (PRMT7), transcript variant 1, mRNA [NM_019023]	54496
A_23_P77430	4.8	5.1	4.1	-1.5	PRMT7	Homo sapiens protein arginine methyltransferase 7 (PRMT7), transcript variant 1, mRNA [NM_019023]	54496
A_23_P139740	1.0	-1.4	-2.0	-1.1	PRMT8	Homo sapiens protein arginine methyltransferase 8 (PRMT8), mRNA [NM_019854]	56341

A_24_P927041	2.2	1.0	2.0	2.0	PRND	Homo sapiens prion protein 2 (dublet) (PRND), mRNA [NM_012409]	23627
A_24_P348828	-5.4	-1.0	-1.1	-1.1	PRNT	Homo sapiens prion protein (testis specific) (PRNT), transcript variant 1, non-coding RNA [NR_024267]	149830
A_23_P68786	-7.0	-1.8	-1.4	-1.1	PRODH	Homo sapiens proline dehydrogenase (oxidase) 1 (PRODH), nuclear gene encoding mitochondrial protein, transcript variant 1, mRNA [NM_016335]	5625

A_24_P402855	1.0	-2.1	1.0	1.2	PROL1	Homo sapiens proline rich, lacrimal 1 (PROL1), mRNA [NM_021225]	58503
A_23_P154358	2.0	-1.6	-1.2	1.6	PROM2	Homo sapiens prominin 2 (PROM2), transcript variant 3, mRNA [NM_144707]	150696
A_23_P135848	2.3	1.2	1.2	1.0	PROP1	Homo sapiens PROP paired-like homeobox 1 (PROP1), mRNA [NM_006261]	5626
A_23_P73114	1.6	1.6	1.8	-2.3	PROS1	Homo sapiens protein S (alpha) (PROS1), mRNA [NM_000313]	5627
A_23_P380298	-1.2	-2.3	-1.4	-2.9	ProSAPiP1	Homo sapiens ProSAPiP1 protein (ProSAPiP1), mRNA [NM_014731]	9762

A_23_P45166	-5.4	-1.3	-1.3	-1.3	PROSC	Homo sapiens proline synthetase co-transcribed homolog (bacterial) (PROSC), mRNA [NM_007198]	11212
A_23_P140074	-3.4	1.0	-3.2	-1.0	PROZ	Homo sapiens protein Z, vitamin K-dependent plasma glycoprotein (PROZ), mRNA [NM_003891]	8858
A_32_P59811	1.6	2.2	-1.4	-1.4	PRPF18	PRP18 pre-mRNA processing factor 18 homolog (S. cerevisiae) [Source:HGNC Symbol;Acc:17351] [ENST00000320054]	8559

A_23_P142022	-2.1	-3.1	-3.3	-1.3	PRPF31	Homo sapiens PRP31 pre-mRNA processing factor 31 homolog (S. cerevisiae) (PRPF31), mRNA [NM_015629]	26121
A_23_P60537	1.4	-2.5	1.3	1.0	PRPF4	Homo sapiens PRP4 pre-mRNA processing factor 4 homolog (yeast) (PRPF4), transcript variant 1, mRNA [NM_004697]	9128
A_23_P411922	2.1	1.3	2.1	1.2	PRPF40B	Homo sapiens PRP40 pre-mRNA processing factor 40 homolog B (S. cerevisiae) (PRPF40B), transcript variant 1, mRNA [NM_001031698]	25766

A_32_P402521	-1.9	-1.8	-2.5	-1.6	PRPF8	Homo sapiens PRP8 pre-mRNA processing factor 8 homolog (S. cerevisiae) (PRPF8), mRNA [NM_006445]	10594
A_32_P192823	4.2	3.8	1.2	2.1	PRPS1L1	Homo sapiens phosphoribosyl pyrophosphate synthetase 1-like 1 (PRPS1L1), mRNA [NM_175886]	221823
A_23_P207307	-1.0	-1.0	-4.9	-4.2	PRR11	Homo sapiens cDNA FLJ11029 fis, clone PLACE1004156. [AK001891]	55771
A_23_P55948	-4.4	3.3	1.6	-1.1	PRR12	Homo sapiens proline rich 12 (PRR12), mRNA [NM_020719]	57479

A_24_P73178	-1.2	-2.9	-2.2	-1.3	PRR13	Homo sapiens proline rich 13 (PRR13), transcript variant 3, mRNA [NM_001005354]	54458
A_24_P349466	-1.1	1.0	-3.7	-1.3	PRR13	Homo sapiens proline rich 13 (PRR13), transcript variant 3, mRNA [NM_001005354]	54458
A_23_P49412	1.7	2.1	1.4	1.5	PRR14	Homo sapiens proline rich 14 (PRR14), mRNA [NM_024031]	78994
A_23_P431346	2.2	1.9	1.6	2.1	PRR15	Homo sapiens proline rich 15 (PRR15), mRNA [NM_175887]	222171
A_23_P92929	1.4	1.4	1.3	-41.7	PRR16	Homo sapiens proline rich 16 (PRR16), mRNA [NM_016644]	51334

A_24_P178300	2.0	1.5	2.0	-2.9	PRR21	Homo sapiens proline rich 21 (PRR21), mRNA [NM_001080835]	643905
A_23_P372988	-1.0	-2.9	1.0	-1.0	PRR22	Homo sapiens proline rich 22 (PRR22), mRNA [NM_001134316]	163154
A_23_P386168	-2.1	-1.1	1.9	2.2	PRR24	Homo sapiens proline rich 24 (PRR24), transcript variant 1, mRNA [NM_178511]	255783
A_24_P106454	1.3	1.2	-2.8	3.1	PRR3	Homo sapiens proline rich 3 (PRR3), transcript variant 1, mRNA [NM_025263]	80742
A_24_P10890	-1.9	-2.5	-2.6	-2.2	PRR5	Homo sapiens proline rich 5 (renal) (PRR5), transcript variant 2, mRNA [NM_015366]	55615

A_24_P20807	-3.6	-1.5	-9.9	-1.2	PRR7	Homo sapiens proline rich 7 (synaptic) (PRR7), transcript variant 1, mRNA [NM_030567]	80758
A_23_P30464	-1.3	-1.4	-3.9	-1.4	PRR7	Homo sapiens proline rich 7 (synaptic) (PRR7), transcript variant 1, mRNA [NM_030567]	80758
A_24_P641130	1.9	2.2	1.9	1.3	PRRC2A	Homo sapiens proline-rich coiled-coil 2A (PRRC2A), transcript variant 1, mRNA [NM_080686]	7916
A_23_P30870	-2.3	-3.1	-4.5	-2.6	PRRC2A	proline-rich coiled-coil 2A [Source:HGNC Symbol;Acc:13918] [ENST00000464106]	7916

A_23_P123578	1.9	1.6	2.3	2.0	PRRC2B	Homo sapiens proline-rich coiled-coil 2B (PRRC2B), mRNA [NM_013318]	84726
A_23_P67355	1.2	-3.0	1.4	1.0	PRRG2	Homo sapiens proline rich Gla (G-carboxyglutamic acid) 2 (PRRG2), mRNA [NM_000951]	5639
A_23_P387537	1.4	1.1	2.1	-1.2	PRRG3	Homo sapiens proline rich Gla (G-carboxyglutamic acid) 3 (transmembrane) (PRRG3), transcript variant 1, mRNA [NM_024082]	79057
A_23_P127663	-4.9	1.8	-2.4	-1.5	PRRG4	Homo sapiens proline rich Gla (G-carboxyglutamic acid) 4 (transmembrane) (PRRG4), mRNA [NM_024081]	79056

A_32_P192474	1.2	-1.5	-2.5	-14.3	PRRT1	Homo sapiens proline-rich transmembrane protein 1 (PRRT1), mRNA [NM_030651]	80863
A_23_P380754	-8.6	-1.5	-1.3	-2.2	PRSS1	Homo sapiens protease, serine, 1 (trypsin 1) (PRSS1), mRNA [NM_002769]	5644
A_32_P94444	1.3	2.4	1.4	2.4	PRSS2	Homo sapiens protease, serine, 2 (trypsin 2) (PRSS2), mRNA [NM_002770]	5645
A_23_P310274	1.1	1.6	2.3	1.0	PRSS2	Homo sapiens protease, serine, 2 (trypsin 2) (PRSS2), mRNA [NM_002770]	5645
A_23_P87421	1.1	1.0	1.0	3.7	PRSS23	protease, serine, 23 [Source:HGNC Symbol;Acc:14370] [ENST00000532234]	11098

A_23_P106806	-1.0	2.0	1.2	-1.0	PRSS27	Homo sapiens protease, serine 27 (PRSS27), mRNA [NM_031948]	83886
A_23_P135257	2.5	1.8	1.5	-7.4	PRSS3	Homo sapiens protease, serine, 3 (PRSS3), transcript variant 2, mRNA [NM_002771]	5646
A_23_P350719	1.2	-8.3	-2.0	-1.1	PRSS30P	Homo sapiens protease, serine, 30 homolog (mouse), pseudogene (PRSS30P), non-coding RNA [NR_026864]	124221
A_23_P42397	1.1	-9.1	-1.1	-1.0	PRSS35	Homo sapiens protease, serine, 35 (PRSS35), transcript variant 2, mRNA [NM_153362]	167681

A_24_P412734	2.1	2.1	1.1	-1.0	PRSS36	Homo sapiens protease, serine, 36 (PRSS36), mRNA [NM_173502]	146547
A_23_P93960	-2.1	1.2	-1.6	-2.9	PRSS37	Homo sapiens protease, serine, 37 (PRSS37), transcript variant 1, mRNA [NM_001008270]	136242
A_24_P408767	-1.0	10.7	10.2	12.8	PRSS42	Homo sapiens protease, serine, 42 (PRSS42), mRNA [NM_182702]	339906
A_23_P10172	-1.5	-1.5	-4.7	-1.6	PRSS50	Homo sapiens protease, serine, 50 (PRSS50), mRNA [NM_013270]	29122
A_24_P73817	1.1	-2.1	-1.9	-3.2	PRSS53	Homo sapiens protease, serine, 53 (PRSS53), mRNA [NM_001039503]	339105

A_23_P77697	1.0	1.0	1.1	-2.1	PRSS53	protease, serine, 53 [Source:HGNC Symbol;Acc:34407] [ENST00000486499]	339105
A_24_P150803	2.7	1.8	1.8	2.3	PRSS8	Homo sapiens protease, serine, 8 (PRSS8), mRNA [NM_002773]	5652
A_23_P142345	2.1	-2.5	1.7	-1.1	PRTN3	Homo sapiens proteinase 3 (PRTN3), mRNA [NM_002777]	5657
A_24_P217001	1.8	2.3	-1.8	-1.0	PRUNE	Homo sapiens prune homolog (Drosophila) (PRUNE), mRNA [NM_021222]	58497
A_23_P429959	2.0	1.1	1.6	1.1	PRX	Homo sapiens periaxin (PRX), transcript variant 1, mRNA [NM_020956]	57716

A_24_P390909	-1.4	-1.8	-2.1	2.6	PRX	Homo sapiens periaxin (PRX), transcript variant 2, mRNA [NM_181882]	57716
A_23_P12680	-17.3	-12.8	-1.8	-1.3	PSAP	Homo sapiens prosaposin (PSAP), transcript variant 2, mRNA [NM_001042465]	5660
A_23_P213020	1.1	1.1	2.2	1.1	PSAPL1	Homo sapiens prosaposin-like 1 (gene/pseudogene) (PSAPL1), mRNA [NM_001085382]	768239
A_23_P259692	-7.8	-1.0	-1.1	-1.0	PSAT1	Homo sapiens phosphoserine aminotransferase 1 (PSAT1), transcript variant 1, mRNA [NM_058179]	29968

A_24_P201021	-1.6	-2.9	-1.1	-1.0	PSCA	Homo sapiens prostate stem cell antigen (PSCA), transcript variant 1, mRNA [NM_005672]	8000
A_23_P115699	38.4	9.7	35.0	20.0	PSD	Homo sapiens pleckstrin and Sec7 domain containing (PSD), mRNA [NM_002779]	5662
A_24_P18146	-1.0	-1.0	-2.9	-1.0	PSD3	Homo sapiens pleckstrin and Sec7 domain containing 3 (PSD3), transcript variant 1, mRNA [NM_015310]	23362
A_23_P130948	3.2	2.3	2.9	3.1	PSG11	Homo sapiens pregnancy specific beta-1-glycoprotein 11 (PSG11), transcript variant 1, mRNA [NM_002785]	5680

A_23_P142335	-6.2	-1.1	1.1	-1.2	PSG2	Homo sapiens pregnancy specific beta-1-glycoprotein 2 (PSG2), mRNA [NM_031246]	5670
A_23_P5103	-1.0	2.3	-1.9	3.1	PSG4	Homo sapiens pregnancy specific beta-1-glycoprotein 4 (PSG4), transcript variant 2, mRNA [NM_213633]	5672
A_32_P219970	-14.6	-1.8	-4.7	-2.5	PSG5	pregnancy specific beta-1-glycoprotein 5 [Source:HGNC Symbol;Acc:9522] [ENST00000366175]	5673

A_23_P108170	1.1	-5.6	1.1	1.3	PSG6	Homo sapiens pregnancy specific beta-1-glycoprotein 6 (PSG6), transcript variant 1, mRNA [NM_002782]	5675
A_24_P331761	-1.4	-1.2	-1.0	-3.4	PSG7	Homo sapiens pregnancy specific beta-1-glycoprotein 7 (gene/pseudogene) (PSG7), transcript variant 1, mRNA [NM_002783]	5676
A_23_P39309	-2.0	-1.1	1.0	-3.5	PSG9	Homo sapiens pregnancy specific beta-1-glycoprotein 9 (PSG9), mRNA [NM_002784]	5678

A_23_P39304	-1.2	-1.0	-6.3	-1.1	PSG9	Homo sapiens pregnancy specific beta-1-glycoprotein 9 (PSG9), mRNA [NM_002784]	5678
A_23_P91468	2.0	-1.1	1.5	-1.1	PSMA7	Homo sapiens proteasome (prosome, macropain) subunit, alpha type, 7 (PSMA7), mRNA [NM_002792]	5688
A_24_P284893	-2.8	-3.0	-5.6	-4.7	PSMB2	Homo sapiens proteasome (prosome, macropain) subunit, beta type, 2 (PSMB2), transcript variant 1, mRNA [NM_002794]	5690

A_23_P170058	-4.6	-1.7	-3.2	-1.8	PSMB2	Homo sapiens proteasome (prosome, macropain) subunit, beta type, 2 (PSMB2), transcript variant 1, mRNA [NM_002794]	5690
A_23_P761	-1.3	-5.4	1.0	1.0	PSMB4	Homo sapiens proteasome (prosome, macropain) subunit, beta type, 4 (PSMB4), mRNA [NM_002796]	5692
A_24_P347488	1.1	-4.6	-1.1	-1.0	PSMC1	Homo sapiens proteasome (prosome, macropain) 26S subunit, ATPase, 1 (PSMC1), mRNA [NM_002802]	5700

A_32_P9700	1.3	1.7	-2.3	-1.8	PSMC1	Homo sapiens proteasome (prosome, macropain) 26S subunit, ATPase, 1 (PSMC1), mRNA [NM_002802]	5700
A_23_P104607	1.9	1.8	2.3	1.3	PSMC3	Homo sapiens proteasome (prosome, macropain) 26S subunit, ATPase, 3 (PSMC3), mRNA [NM_002804]	5702
A_23_P128930	1.1	-1.1	-2.0	-1.1	PSMC6	Homo sapiens proteasome (prosome, macropain) 26S subunit, ATPase, 6 (PSMC6), mRNA [NM_002806]	5706

A_23_P112613	1.1	-2.2	-1.0	2.1	PSMD12	proteasome (prosome, macropain) 26S subunit, non-ATPase, 12 [Source:HGNC Symbol;Acc:9557] [ENST00000356126]	5718
A_24_P42681	-2.7	1.1	-5.6	-1.2	PSMD2	Homo sapiens proteasome (prosome, macropain) 26S subunit, non-ATPase, 2 (PSMD2), mRNA [NM_002808]	5708
A_24_P289726	2.3	-1.0	-1.3	1.3	PSMD3	Homo sapiens proteasome (prosome, macropain) 26S subunit, non-ATPase, 3 (PSMD3), mRNA [NM_002809]	5709

A_23_P12168	4.1	1.4	-1.3	1.1	PSMD4	Homo sapiens proteasome (prosome, macropain) 26S subunit, non-ATPase, 4 (PSMD4), mRNA [NM_002810]	5710
A_32_P435367	1.0	-1.0	-1.1	-14.4	PSMD4	Homo sapiens proteasome (prosome, macropain) 26S subunit, non-ATPase, 4 (PSMD4), mRNA [NM_002810]	5710
A_23_P43434	1.0	-1.0	-2.0	-2.0	PSMD5	Homo sapiens proteasome (prosome, macropain) 26S subunit, non-ATPase, 5 (PSMD5), mRNA [NM_005047]	5711

A_24_P363802	1.5	1.9	-1.1	2.4	PSMD5	Homo sapiens proteasome (prosome, macropain) 26S subunit, non-ATPase, 5 (PSMD5), mRNA [NM_005047]	5711
A_23_P106741	-2.1	1.0	-1.0	1.0	PSMD7	Homo sapiens proteasome (prosome, macropain) 26S subunit, non-ATPase, 7 (PSMD7), mRNA [NM_002811]	5713
A_32_P45875	4.3	4.9	-1.3	2.3	PSMD8	Homo sapiens cDNA clone IMAGE:5441984, **** WARNING: chimeric clone ****. [BC071826]	5714

A_23_P151614	-6.3	1.0	1.0	1.0	PSME1	Homo sapiens proteasome (prosome, macropain) activator subunit 1 (PA28 alpha) (PSME1), transcript variant 1, mRNA [NM_006263]	5720
A_24_P352864	2.0	-2.4	-5.7	-1.5	PSME3	Homo sapiens proteasome (prosome, macropain) activator subunit 3 (PA28 gamma; Ki) (PSME3), transcript variant 1, mRNA [NM_005789]	10197

A_24_P122050	2.0	2.5	-1.1	1.4	PSMF1	Homo sapiens proteasome (prosome, macropain) inhibitor subunit 1 (PI31) (PSMF1), transcript variant 1, mRNA [NM_006814]	9491
A_24_P399362	2.1	2.0	2.0	-1.1	PSMG2	Homo sapiens proteasome (prosome, macropain) assembly chaperone 2 (PSMG2), transcript variant 1, mRNA [NM_020232]	56984
A_24_P166434	2.2	1.7	1.9	1.1	PSORS1C2	Homo sapiens psoriasis susceptibility 1 candidate 2 (PSORS1C2), mRNA [NM_014069]	170680

A_23_P81926	1.4	2.2	1.3	1.3	PSORS1C2	Homo sapiens psoriasis susceptibility 1 candidate 2 (PSORS1C2), mRNA [NM_014069]	170680
A_23_P76610	-1.2	-1.2	-1.2	-4.5	PSPC1	Homo sapiens paraspeckle component 1 (PSPC1), transcript variant 3, non-coding RNA [NR_044998]	55269
A_23_P48997	-1.3	-1.4	-2.3	-1.1	PSTPIP1	Homo sapiens proline-serine-threonine phosphatase interacting protein 1 (PSTPIP1), mRNA [NM_003978]	9051

A_24_P102821	1.7	1.8	2.8	-1.0	PTAFR	Homo sapiens platelet-activating factor receptor (PTAFR), transcript variant 3, mRNA [NM_000952]	5724
A_23_P51926	-1.0	-1.0	-3.2	-1.1	PTAFR	Homo sapiens platelet-activating factor receptor (PTAFR), transcript variant 3, mRNA [NM_000952]	5724
A_23_P134650	3.9	4.0	3.8	2.2	PTCD1	Homo sapiens pentatricopeptide repeat domain 1 (PTCD1), nuclear gene encoding mitochondrial protein, mRNA [NM_015545]	26024

A_24_P191847	-3.4	-4.8	-1.0	-1.0	PTCD2	Homo sapiens pentatricopeptide repeat domain 2 (PTCD2), mRNA [NM_024754]	79810
A_24_P115490	-1.5	-1.6	-4.1	-1.9	PTCH1	Homo sapiens patched 1 (PTCH1), transcript variant 1b, mRNA [NM_000264]	5727
A_24_P106910	2.1	1.7	1.2	-2.5	PTCH1	Homo sapiens patched 1 (PTCH1), transcript variant 1a, mRNA [NM_001083602]	5727
A_23_P355311	1.1	-1.1	2.6	1.7	PTCH2	Homo sapiens patched 2 (PTCH2), transcript variant 1, mRNA [NM_003738]	8643
A_32_P232411	1.7	3.9	-2.3	-1.2	PTCHD2	Homo sapiens patched domain containing 2 (PTCHD2), mRNA [NM_020780]	57540

A_23_P30755	3.2	3.4	-1.5	-2.1	PTCRA	Homo sapiens pre T-cell antigen receptor alpha (PTCRA), transcript variant 2, mRNA [NM_138296]	171558
A_32_P353798	2.2	1.0	-1.0	1.1	PTDSS2	Homo sapiens phosphatidylserine synthase 2 (PTDSS2), mRNA [NM_030783]	81490
A_24_P913115	-1.0	-4.0	1.0	-1.4	PTEN	Homo sapiens phosphatase and tensin homolog (PTEN), mRNA [NM_000314]	5728
A_23_P323774	1.0	-1.8	-3.4	-3.1	PTER	Homo sapiens phosphotriesterase related (PTER), transcript variant 1, mRNA [NM_001001484]	9317

A_23_P428329	-1.9	-6.2	-3.1	-2.7	PTF1A	Homo sapiens pancreas specific transcription factor, 1a (PTF1A), mRNA [NM_178161]	256297
A_24_P919916	-1.7	1.1	-3.7	-2.8	PTF1A	Homo sapiens bHLH transcription factor p48 mRNA, partial cds. [AF181999]	256297
A_23_P393777	1.8	-1.0	-2.3	-1.4	PTGDR	Homo sapiens prostaglandin D2 receptor (DP) (PTGDR), mRNA [NM_000953]	5729
A_23_P146554	2.6	-1.7	1.2	1.8	PTGDS	Homo sapiens prostaglandin D2 synthase 21kDa (brain) (PTGDS), mRNA [NM_000954]	5730

A_24_P945365	1.6	-1.3	-1.5	-2.1	PTGER3	Homo sapiens prostaglandin E receptor 3 (subtype EP3) (PTGER3), transcript variant 5, mRNA [NM_198715]	5733
A_23_P435394	-2.8	1.6	1.6	1.0	PTGER4	Homo sapiens prostaglandin E receptor 4 (subtype EP4) (PTGER4), mRNA [NM_000958]	5734
A_24_P403417	1.4	-1.5	-1.2	-2.0	PTGES	Homo sapiens prostaglandin E synthase (PTGES), mRNA [NM_004878]	9536
A_24_P106953	1.1	2.6	2.1	1.3	PTGES2	Homo sapiens prostaglandin E synthase 2 (PTGES2), transcript variant 1, mRNA [NM_025072]	80142

A_24_P343869	-1.7	-1.6	-2.0	-1.2	PTGES3	Homo sapiens prostaglandin E synthase 3 (cytosolic) (PTGES3), mRNA [NM_006601]	10728
A_24_P302172	1.2	1.2	1.1	-4.9	PTGFR	Homo sapiens prostaglandin F receptor (FP) (PTGFR), transcript variant 2, mRNA [NM_001039585]	5737
A_24_P322426	3.1	3.8	1.1	4.5	PTGIR	Homo sapiens prostaglandin I2 (prostacyclin) receptor (IP) (PTGIR), mRNA [NM_000960]	5739
A_23_P91262	-1.1	-2.4	-1.3	-3.0	PTGIS	Homo sapiens prostaglandin I2 (prostacyclin) synthase (PTGIS), mRNA [NM_000961]	5740

A_23_P157809	1.0	2.4	-1.2	2.5	PTGR1	Homo sapiens prostaglandin reductase 1 (PTGR1), transcript variant 2, mRNA [NM_012212]	22949
A_23_P48705	-1.4	-3.9	2.1	2.2	PTGR2	Homo sapiens prostaglandin reductase 2 (PTGR2), transcript variant 1, mRNA [NM_152444]	145482
A_24_P64167	1.0	-3.3	-1.7	-3.9	PTGS1	Homo sapiens prostaglandin-endoperoxide synthase 1 (prostaglandin G/H synthase and cyclooxygenase) (PTGS1), transcript variant 1, mRNA [NM_000962]	5742

A_23_P167030	1.8	2.8	2.3	2.2	PTH1R	Homo sapiens parathyroid hormone 1 receptor (PTH1R), transcript variant 1, mRNA [NM_000316]	5745
A_23_P357374	2.2	1.8	1.6	1.7	PTH2	Homo sapiens parathyroid hormone 2 (PTH2), mRNA [NM_178449]	113091
A_24_P923612	-1.3	-1.3	-2.2	-2.8	PTHLH	parathyroid hormone-like hormone [Source:HGNC Symbol;Acc:9607] [ENST00000354417]	5744
A_23_P22096	-2.0	-2.5	-1.2	-1.2	PTK2	Homo sapiens PTK2 protein tyrosine kinase 2 (PTK2), transcript variant 1, mRNA [NM_153831]	5747

A_23_P56978	-3.0	-21.5	-1.0	-1.0	PTK6	Homo sapiens PTK6 protein tyrosine kinase 6 (PTK6), mRNA [NM_005975]	5753
A_23_P434301	1.8	1.2	2.8	-1.0	PTMA	Homo sapiens prothymosin, alpha (PTMA), transcript variant 2, mRNA [NM_002823]	5757
A_24_P870620	1.8	-1.8	-1.2	-3.2	PTN	Homo sapiens pleiotrophin (PTN), mRNA [NM_002825]	5764
A_24_P252043	-1.7	1.5	-2.4	1.9	PTP4A1	Homo sapiens protein tyrosine phosphatase type IVA, member 1 (PTP4A1), mRNA [NM_003463]	7803

A_23_P378968	2.6	4.9	2.5	2.8	PTP4A2	Homo sapiens protein tyrosine phosphatase type IVA, member 2 (PTP4A2), transcript variant 1, mRNA [NM_080391]	8073
A_24_P764598	3.1	1.8	1.8	1.9	PTP4A2	Homo sapiens protein tyrosine phosphatase type IVA, member 2 (PTP4A2), transcript variant 4, mRNA [NM_001195101]	8073
A_24_P110062	-1.6	-1.5	3.2	-2.1	PTPMT1	Homo sapiens protein tyrosine phosphatase, mitochondrial 1 (PTPMT1), nuclear gene encoding mitochondrial protein, transcript variant 1, mRNA [NM_175732]	114971

A_23_P338890	-2.8	-1.8	-1.1	-1.0	PTPN1	Homo sapiens protein tyrosine phosphatase, non-receptor type 1 (PTPN1), mRNA [NM_002827]	5770
A_32_P61757	1.8	1.8	2.1	1.0	PTPN11	Homo sapiens protein tyrosine phosphatase, non-receptor type 11 (PTPN11), mRNA [NM_002834]	5781
A_23_P105436	1.0	1.3	-2.3	-1.6	PTPN11	Homo sapiens protein tyrosine phosphatase, non-receptor type 11 (PTPN11), mRNA [NM_002834]	5781

A_23_P8763	1.1	-1.6	-1.3	-3.9	PTPN12	Homo sapiens protein tyrosine phosphatase, non-receptor type 12 (PTPN12), transcript variant 1, mRNA [NM_002835]	5782
A_23_P18493	1.1	1.1	1.0	-4.9	PTPN13	Homo sapiens protein tyrosine phosphatase, non-receptor type 13 (APO-1/CD95 (Fas)-associated phosphatase) (PTPN13), transcript variant 4, mRNA [NM_080685]	5783
A_23_P149111	1.5	12.0	4.3	3.3	PTPN14	Homo sapiens protein tyrosine phosphatase, non-receptor type 14 (PTPN14), mRNA [NM_005401]	5784

A_23_P309701	1.0	1.7	1.0	2.4	PTPN2	Homo sapiens protein tyrosine phosphatase, non-receptor type 2 (PTPN2), transcript variant 1, mRNA [NM_002828]	5771
A_24_P328675	3.1	5.4	-1.5	1.3	PTPN23	Homo sapiens protein tyrosine phosphatase, non-receptor type 23 (PTPN23), mRNA [NM_015466]	25930
A_23_P69306	3.6	3.6	-1.3	1.3	PTPN23	Homo sapiens protein tyrosine phosphatase, non-receptor type 23 (PTPN23), mRNA [NM_015466]	25930

A_24_P928969	-1.1	-3.7	-1.0	-21.6	PTPN3	Homo sapiens protein tyrosine phosphatase, non-receptor type 3 (PTPN3), transcript variant 3, mRNA [NM_001145369]	5774
A_23_P403898	-2.3	-1.0	-1.1	1.0	PTPN3	Homo sapiens protein tyrosine phosphatase, non-receptor type 3 (PTPN3), transcript variant 1, mRNA [NM_002829]	5774
A_23_P165343	-2.0	-1.4	-2.2	-3.1	PTPN4	Homo sapiens protein tyrosine phosphatase, non-receptor type 4 (megakaryocyte) (PTPN4), mRNA [NM_002830]	5775

A_24_P409519	-3.0	1.2	-2.6	1.9	PTPN5	Homo sapiens protein tyrosine phosphatase, non-receptor type 5 (striatum-enriched) (PTPN5), transcript variant 1, mRNA [NM_006906]	84867
A_23_P201778	-1.0	-1.0	-6.1	1.0	PTPN7	Homo sapiens protein tyrosine phosphatase, non-receptor type 7 (PTPN7), transcript variant 2, mRNA [NM_080588]	5778
A_23_P53390	-2.5	1.1	1.0	-1.0	PTPRB	Homo sapiens protein tyrosine phosphatase, receptor type, B (PTPRB), transcript variant 2, mRNA [NM_002837]	5787

A_23_P98173	1.5	-2.2	-2.7	1.8	PTPRCAP	Homo sapiens protein tyrosine phosphatase, receptor type, C-associated protein (PTPRCAP), mRNA [NM_005608]	5790
A_24_P927863	8.8	8.5	7.9	6.9	PTPRE	protein tyrosine phosphatase, receptor type, E [Source:HGNC Symbol;Acc:9669] [ENST00000455661]	5791
A_24_P213494	1.3	2.1	-1.8	1.2	PTPRE	Homo sapiens protein tyrosine phosphatase, receptor type, E (PTPRE), transcript variant 1, mRNA [NM_006504]	5791

A_23_P138495	-1.3	2.1	1.8	1.0	PTPRE	Homo sapiens protein tyrosine phosphatase, receptor type, E (PTPRE), transcript variant 1, mRNA [NM_006504]	5791
A_24_P385313	1.3	2.6	2.4	-1.2	PTPRF	Homo sapiens protein tyrosine phosphatase, receptor type, F (PTPRF), transcript variant 1, mRNA [NM_002840]	5792
A_23_P405049	-3.8	-1.0	-1.1	-1.0	PTPRJ	Homo sapiens protein tyrosine phosphatase, receptor type, J (PTPRJ), transcript variant 1, mRNA [NM_002843]	5795

A_23_P90722	44.4	30.0	26.9	27.4	PTPRN	Homo sapiens protein tyrosine phosphatase, receptor type, N (PTPRN), transcript variant 1, mRNA [NM_002846]	5798
A_24_P76809	2.2	3.3	2.1	-1.2	PTPRO	Homo sapiens protein tyrosine phosphatase, receptor type, O, mRNA (cDNA clone IMAGE:4610767), complete cds. [BC035960]	5800
A_24_P290856	-3.3	-1.4	-1.5	-1.4	PTPRS	Homo sapiens protein tyrosine phosphatase, receptor type, S (PTPRS), transcript variant 3, mRNA [NM_130853]	5802

A_23_P56163	-1.4	-1.6	-4.9	-1.3	PTPRS	Homo sapiens protein tyrosine phosphatase, receptor type, S (PTPRS), transcript variant 1, mRNA [NM_002850]	5802
A_23_P149064	-18.8	-1.3	1.1	1.8	PTPRU	Homo sapiens protein tyrosine phosphatase, receptor type, U (PTPRU), transcript variant 3, mRNA [NM_005704]	10076
A_24_P8474	-1.0	-1.1	-3.4	-1.1	PTPRZ1	Homo sapiens protein tyrosine phosphatase, receptor-type, Z polypeptide 1 (PTPRZ1), transcript variant 1, mRNA [NM_002851]	5803

A_24_P235248	1.0	-2.9	1.0	-1.0	PTPRZ1	Homo sapiens protein tyrosine phosphatase, receptor-type, Z polypeptide 1 (PTPRZ1), transcript variant 1, mRNA [NM_002851]	5803
A_23_P141376	-1.5	-5.8	-1.0	-1.4	PTRF	Homo sapiens polymerase I and transcript release factor (PTRF), mRNA [NM_012232]	284119
A_24_P16036	-26.3	-2.4	-1.1	1.6	PTRHD1	Homo sapiens peptidyl-tRNA hydrolase domain containing 1 (PTRHD1), mRNA [NM_001013663]	391356

A_23_P109345	-1.6	-2.2	-1.4	-3.1	PTTG1IP	Homo sapiens pituitary tumor-transforming 1 interacting protein (PTTG1IP), mRNA [NM_004339]	754
A_23_P18579	1.7	1.3	-7.8	1.3	PTTG2	Homo sapiens pituitary tumor-transforming 2 (PTTG2), mRNA [NM_006607]	10744
A_23_P60016	-1.1	-2.0	-2.6	-2.4	PTTG3P	Homo sapiens pituitary tumor-transforming 3, pseudogene (PTTG3P), non-coding RNA [NR_002734]	26255

A_24_P149645	1.1	2.0	1.0	-1.0	PUF60	Homo sapiens poly-U binding splicing factor 60KDa (PUF60), transcript variant 2, mRNA [NM_014281]	22827
A_24_P171345	-1.2	2.1	-28.3	1.2	PUM1	Homo sapiens pumilio homolog 1 (Drosophila) (PUM1), transcript variant 1, mRNA [NM_001020658]	9698
A_23_P5550	4.1	5.5	5.4	5.0	PUM2	Homo sapiens pumilio homolog 2 (Drosophila) (PUM2), mRNA [NM_015317]	23369
A_24_P71439	-2.3	2.2	-1.2	-1.8	PUM2	Homo sapiens pumilio homolog 2 (Drosophila) (PUM2), mRNA [NM_015317]	23369

A_23_P134517	3.0	1.8	1.1	-3.4	PURB	Homo sapiens purine-rich element binding protein B (PURB), mRNA [NM_033224]	5814
A_23_P111919	4.3	4.3	4.3	4.3	PURG	Homo sapiens purine-rich element binding protein G (PURG), transcript variant A, mRNA [NM_013357]	29942
A_23_P251771	-2.1	1.1	1.1	1.1	PUS1	Homo sapiens pseudouridylate synthase 1 (PUS1), transcript variant 1, mRNA [NM_025215]	80324
A_23_P412029	-2.1	1.2	-1.0	-1.7	PUS10	Homo sapiens pseudouridylate synthase 10 (PUS10), mRNA [NM_144709]	150962

A_23_P373687	1.1	-5.2	-1.9	1.7	PUS10	Homo sapiens pseudouridylate synthase 10 (PUS10), mRNA [NM_144709]	150962
A_24_P167496	-17.7	1.0	-1.0	-1.0	PUS7L	Homo sapiens pseudouridylate synthase 7 homolog (S. cerevisiae)-like (PUS7L), transcript variant 3, mRNA [NM_031292]	83448
A_23_P51884	1.0	-2.4	-1.0	-1.3	PUSL1	Homo sapiens pseudouridylate synthase-like 1 (PUSL1), mRNA [NM_153339]	126789
A_23_P17844	-3.7	-1.2	-1.4	-1.5	PVALB	Homo sapiens parvalbumin (PVALB), mRNA [NM_002854]	5816

A_23_P8424	2.6	3.2	1.5	1.0	PVRIG	Homo sapiens poliovirus receptor related immunoglobulin domain containing (PVRIG), mRNA [NM_024070]	79037
A_23_P76034	2.1	2.8	1.4	1.7	PVRL1	Homo sapiens poliovirus receptor-related 1 (herpesvirus entry mediator C) (PVRL1), transcript variant 3, mRNA [NM_203286]	5818
A_23_P80763	1.1	-2.8	-1.0	-10.3	PVRL3	Homo sapiens poliovirus receptor-related 3 (PVRL3), transcript variant 1, mRNA [NM_015480]	25945

A_23_P401547	-1.0	-1.0	-3.7	-1.0	PVRL3	Homo sapiens poliovirus receptor-related 3 (PVRL3), transcript variant 1, mRNA [NM_015480]	25945
A_23_P105753	4.0	3.8	3.6	3.6	PWP1	PWP1 homolog (S. cerevisiae) [Source:HGNC Symbol;Acc:17015] [ENST00000412830]	11137
A_23_P374389	1.5	1.0	-3.5	-1.3	PWWP2B	Homo sapiens PWWP domain containing 2B (PWWP2B), transcript variant 1, mRNA [NM_138499]	170394
A_24_P944570	12.5	12.3	11.8	12.8	PXDN	Homo sapiens melanoma-associated antigen MG50 mRNA, partial cds. [AF200348]	7837

A_23_P257144	1.0	2.8	-1.2	1.0	PXDN	Homo sapiens peroxidasin homolog (Drosophila) (PXDN), mRNA [NM_012293]	7837
A_32_P115606	-2.0	-1.0	-2.8	-1.1	PXK	PX domain containing serine/threonine kinase [Source:HGNC Symbol;Acc:23326] [ENST00000536750]	54899
A_24_P918065	1.1	1.1	1.0	-3.2	PXMP2	Homo sapiens peroxisomal membrane protein 2, 22kDa, mRNA (cDNA clone IMAGE:4098463), complete cds. [BC009836]	5827

A_23_P124122	2.9	4.9	4.3	3.4	PXMP2	Homo sapiens peroxisomal membrane protein 2, 22kDa (PXMP2), mRNA [NM_018663]	5827
A_23_P91430	-1.5	-2.0	-2.1	-1.6	PXMP4	Homo sapiens peroxisomal membrane protein 4, 24kDa (PXMP4), transcript variant 1, mRNA [NM_007238]	11264
A_24_P184555	2.1	2.0	2.4	1.4	PXN	Homo sapiens paxillin (PXN), transcript variant 1, mRNA [NM_002859]	5829
A_24_P204358	-29.1	1.3	2.6	1.0	PYCR1	Homo sapiens pyrroline-5-carboxylate reductase 1 (PYCR1), transcript variant 2, mRNA [NM_153824]	5831

A_23_P149042	-1.6	2.2	-2.3	-1.7	PYCR2	Homo sapiens pyrroline-5-carboxylate reductase family, member 2 (PYCR2), mRNA [NM_013328]	29920
A_24_P4212	-1.7	-7.0	-1.7	-1.7	PYCRL	Homo sapiens pyrroline-5-carboxylate reductase-like (PYCRL), mRNA [NM_023078]	65263
A_23_P210849	1.0	2.0	1.1	4.4	PYGB	Homo sapiens phosphorylase, glycogen; brain (PYGB), mRNA [NM_002862]	5834
A_23_P48676	-15.8	1.7	1.9	-1.1	PYGL	Homo sapiens phosphorylase, glycogen, liver (PYGL), transcript variant 1, mRNA [NM_002863]	5836

A_23_P52657	1.8	2.1	1.3	1.0	PYGM	Homo sapiens phosphorylase, glycogen, muscle (PYGM), transcript variant 1, mRNA [NM_005609]	5837
A_23_P411953	1.3	-1.0	2.1	1.8	PYGO2	Homo sapiens pygopus homolog 2 (Drosophila) (PYGO2), mRNA [NM_138300]	90780
A_24_P221407	2.0	-2.2	1.8	1.9	PYROXD1	Homo sapiens pyridine nucleotide-disulphide oxidoreductase domain 1 (PYROXD1), mRNA [NM_024854]	79912

A_23_P24157	5.8	6.8	3.2	2.5	PYROXD2	Homo sapiens pyridine nucleotide-disulphide oxidoreductase domain 2 (PYROXD2), mRNA [NM_032709]	84795
A_23_P4112	1.2	1.2	-2.5	-1.5	PYY	Homo sapiens peptide YY (PYY), mRNA [NM_004160]	5697
A_24_P233078	2.1	4.3	1.0	2.2	PYY2	Homo sapiens peptide YY, 2 (seminalplasmin) (PYY2), non-coding RNA [NR_003064]	23615
A_23_P139682	-10.0	1.0	-2.3	1.1	PZP	Homo sapiens pregnancy-zone protein (PZP), mRNA [NM_002864]	5858

A_32_P532216	-1.1	-1.1	-1.1	-6.1	QARS	Homo sapiens glutaminyl-tRNA synthetase (QARS), mRNA [NM_005051]	5859
A_23_P81760	-2.0	1.1	-1.1	-1.0	QKI	Homo sapiens QKI, KH domain containing, RNA binding (QKI), transcript variant 1, mRNA [NM_006775]	9444
A_24_P940426	-2.1	2.4	-2.3	1.4	QKI	Homo sapiens QKI, KH domain containing, RNA binding (QKI), transcript variant 4, mRNA [NM_206855]	9444
A_24_P384954	3.7	3.6	3.4	3.0	QKI	Homo sapiens QKI, KH domain containing, RNA binding (QKI), transcript variant 1, mRNA [NM_006775]	9444

A_23_P16915	-1.1	-1.8	-3.2	-1.3	QPCT	Homo sapiens glutaminyl-peptide cyclotransferase (QPCT), mRNA [NM_012413]	25797
A_23_P34233	1.1	-1.5	-2.5	-1.6	QPRT	Homo sapiens quinolinate phosphoribosyltransferase (QPRT), mRNA [NM_014298]	23475
A_23_P92467	-1.0	-1.0	-1.0	-2.2	QRFPR	Homo sapiens pyroglutamylated RFamide peptide receptor (QRFPR), mRNA [NM_198179]	84109
A_23_P45108	3.7	3.3	3.2	1.1	QRICH1	Homo sapiens glutamine-rich 1 (QRICH1), transcript variant 1, mRNA [NM_017730]	54870

A_24_P63799	-2.7	-1.8	1.7	2.1	QRSL1	Homo sapiens glutaminyl-tRNA synthase (glutamine-hydrolyzing)-like 1 (QRSL1), mRNA [NM_018292]	55278
A_24_P132276	1.1	-2.5	1.8	-1.0	QRSL1	Homo sapiens glutaminyl-tRNA synthase (glutamine-hydrolyzing)-like 1 (QRSL1), mRNA [NM_018292]	55278
A_24_P163477	-1.0	-1.0	-7.5	1.1	QRSL1	Homo sapiens glutaminyl-tRNA synthase (glutamine-hydrolyzing)-like 1 (QRSL1), mRNA [NM_018292]	55278

A_23_P12463	-3.5	1.1	-1.8	-1.9	QSOX1	Homo sapiens quiescin Q6 sulfhydryl oxidase 1 (QSOX1), transcript variant 1, mRNA [NM_002826]	5768
A_24_P96234	2.4	2.2	1.8	2.4	QTRT1	Homo sapiens queuine tRNA-ribosyltransferase 1 (QTRT1), mRNA [NM_031209]	81890
A_23_P10374	2.6	3.1	2.6	1.6	R3HDM2	Homo sapiens R3H domain containing 2 (R3HDM2), mRNA [NM_014925]	22864
A_24_P207907	-1.0	-1.1	-8.0	-1.0	R3HDM2	Homo sapiens R3H domain containing 2 (R3HDM2), mRNA [NM_014925]	22864

A_23_P165879	1.3	4.1	1.2	-1.3	RAB10	Homo sapiens RAB10, member RAS oncogene family (RAB10), mRNA [NM_016131]	10890
A_23_P67748	-1.6	3.1	3.1	1.6	RAB11B	Homo sapiens RAB11B, member RAS oncogene family (RAB11B), mRNA [NM_004218]	9230
A_24_P363953	2.2	2.8	1.3	-1.1	RAB11FIP2	Homo sapiens RAB11 family interacting protein 2 (class I) (RAB11FIP2), mRNA [NM_014904]	22841
A_23_P415006	-1.1	2.0	1.6	3.0	RAB11FIP5	Homo sapiens RAB11 family interacting protein 5 (class I) (RAB11FIP5), mRNA [NM_015470]	26056

A_24_P53353	1.1	-2.4	-1.3	-1.0	RAB12	Homo sapiens RAB12, member RAS oncogene family (RAB12), mRNA [NM_001025300]	201475
A_23_P46369	2.3	2.2	2.2	2.1	RAB13	Homo sapiens RAB13, member RAS oncogene family (RAB13), mRNA [NM_002870]	5872
A_24_P193295	-3.2	-6.2	-5.8	-2.7	RAB15	Homo sapiens RAB15, member RAS onocogene family (RAB15), mRNA [NM_198686]	376267
A_23_P138376	2.3	2.1	1.8	2.0	RAB18	Homo sapiens RAB18, member RAS oncogene family (RAB18), mRNA [NM_021252]	22931

A_23_P382148	-2.6	-7.8	-6.9	-1.4	RAB1A	Homo sapiens RAB1A, member RAS oncogene family (RAB1A), transcript variant 1, mRNA [NM_004161]	5861
A_23_P64090	2.1	2.6	2.2	1.9	RAB1B	Homo sapiens RAB1B, member RAS oncogene family (RAB1B), mRNA [NM_030981]	81876
A_23_P70794	-1.0	-1.1	-5.4	-1.4	RAB23	Homo sapiens RAB23, member RAS oncogene family (RAB23), transcript variant 1, mRNA [NM_016277]	51715

A_23_P21072	3.2	3.2	3.1	2.2	RAB24	RAB24, member RAS oncogene family [Source:HGNC Symbol;Acc:9765] [ENST00000495458]	53917
A_23_P54709	2.1	1.9	1.1	1.5	RAB26	Homo sapiens RAB26, member RAS oncogene family (RAB26), mRNA [NM_014353]	25837
A_23_P54373	1.6	1.5	-2.5	-1.1	RAB27A	Homo sapiens RAB27A, member RAS oncogene family (RAB27A), transcript variant 1, mRNA [NM_004580]	5873
A_23_P107612	1.2	-1.0	-1.8	-3.9	RAB27B	Homo sapiens RAB27B, member RAS oncogene family (RAB27B), mRNA [NM_004163]	5874

A_32_P7204	-1.1	-1.5	-1.1	-2.9	RAB28	Homo sapiens RAB28, member RAS oncogene family (RAB28), transcript variant 2, mRNA [NM_004249]	9364
A_23_P125147	4.4	1.2	1.1	2.7	RAB28	Homo sapiens RAB28, member RAS oncogene family (RAB28), transcript variant 2, mRNA [NM_004249]	9364
A_23_P253949	-1.0	-2.7	-1.0	-1.0	RAB2A	Homo sapiens RAB2A, member RAS oncogene family (RAB2A), transcript variant 1, mRNA [NM_002865]	5862

A_24_P236799	-2.3	1.0	1.0	1.1	RAB31	Homo sapiens RAB31, member RAS oncogene family (RAB31), mRNA [NM_006868]	11031
A_23_P141688	-1.1	-4.4	-3.3	-3.1	RAB31	Homo sapiens RAB31, member RAS oncogene family (RAB31), mRNA [NM_006868]	11031
A_24_P303480	1.1	1.0	-1.1	-9.9	RAB32	Homo sapiens RAB32, member RAS oncogene family (RAB32), mRNA [NM_006834]	10981
A_23_P42375	-1.0	1.0	-1.1	-3.1	RAB32	Homo sapiens RAB32, member RAS oncogene family (RAB32), mRNA [NM_006834]	10981

A_24_P268917	-2.3	-7.6	-1.2	1.0	RAB33B	Homo sapiens RAB33B, member RAS oncogene family (RAB33B), mRNA [NM_031296]	83452
A_23_P9565	-2.4	1.1	-1.3	1.1	RAB33B	Homo sapiens RAB33B, member RAS oncogene family (RAB33B), mRNA [NM_031296]	83452
A_24_P50028	-2.4	1.1	-1.0	-1.3	RAB39	Homo sapiens RAB39, member RAS oncogene family (RAB39), mRNA [NM_017516]	54734
A_23_P108200	1.9	-6.1	1.0	1.0	RAB3A	Homo sapiens RAB3A, member RAS oncogene family (RAB3A), mRNA [NM_002866]	5864

A_32_P18668	1.9	-3.0	1.8	1.5	RAB3B	Homo sapiens RAB3B, member RAS oncogene family (RAB3B), mRNA [NM_002867]	5865
A_24_P548966	-1.0	-1.1	-1.1	-2.6	RAB3B	Homo sapiens RAB3B, member RAS oncogene family (RAB3B), mRNA [NM_002867]	5865
A_23_P75800	-8.6	-1.1	1.1	-3.0	RAB3IL1	Homo sapiens RAB3A interacting protein (rabin3)-like 1 (RAB3IL1), mRNA [NM_013401]	5866
A_23_P396062	2.7	1.9	-1.0	-1.0	RAB40C	Homo sapiens RAB40C, member RAS oncogene family (RAB40C), transcript variant 2, mRNA [NM_021168]	57799

A_24_P277295	-1.5	1.0	1.0	-3.2	RAB43	Homo sapiens RAB43, member RAS oncogene family (RAB43), transcript variant 1, mRNA [NM_198490]	339122
A_24_P122682	1.1	1.1	-1.0	-4.3	RAB4A	Homo sapiens RAB4A, member RAS oncogene family (RAB4A), mRNA [NM_004578]	5867
A_23_P16116	1.2	1.4	2.4	1.2	RAB4B	Homo sapiens RAB4B, member RAS oncogene family (RAB4B), mRNA [NM_016154]	53916
A_24_P208809	3.0	2.9	3.6	1.0	RAB5A	Homo sapiens RAB5A, member RAS oncogene family (RAB5A), mRNA [NM_004162]	5868

A_23_P410998	1.0	1.2	-1.1	-19.9	RAB5B	Homo sapiens RAB5B, member RAS oncogene family (RAB5B), transcript variant 1, mRNA [NM_002868]	5869
A_23_P107211	2.5	-3.6	1.4	-7.4	RAB5C	Homo sapiens RAB5C, member RAS oncogene family (RAB5C), transcript variant 1, mRNA [NM_201434]	5878
A_23_P203729	1.8	3.0	3.2	2.4	RAB6A	Homo sapiens RAB6A, member RAS oncogene family (RAB6A), transcript variant 1, mRNA [NM_002869]	5870

A_23_P109974	1.1	-8.5	-1.0	1.1	RAB6B	Homo sapiens RAB6B, member RAS oncogene family (RAB6B), mRNA [NM_016577]	51560
A_24_P234572	2.5	-1.1	-1.7	1.6	RAB7A	Homo sapiens RAB7A, member RAS oncogene family (RAB7A), mRNA [NM_004637]	7879
A_23_P61590	-1.0	1.0	1.0	4.3	RAB7B	Homo sapiens RAB7B, member RAS oncogene family (RAB7B), transcript variant 1, mRNA [NM_177403]	338382

A_24_P234094	-2.3	-1.0	1.2	1.0	RAB7L1	Homo sapiens RAB7, member RAS oncogene family-like 1 (RAB7L1), transcript variant 1, mRNA [NM_003929]	8934
A_23_P126939	1.6	2.5	1.9	2.5	RAB7L1	Homo sapiens RAB7, member RAS oncogene family-like 1 (RAB7L1), transcript variant 1, mRNA [NM_003929]	8934
A_24_P190424	-2.8	1.6	1.7	-1.1	RAB8A	Homo sapiens RAB8A, member RAS oncogene family (RAB8A), mRNA [NM_005370]	4218

A_23_P317465	-13.6	1.0	-1.1	-1.0	RAB8B	Homo sapiens RAB8B, member RAS oncogene family (RAB8B), mRNA [NM_016530]	51762
A_24_P126139	1.0	-4.6	-1.1	-1.1	RAB9B	Homo sapiens RAB9B, member RAS oncogene family (RAB9B), mRNA [NM_016370]	51209
A_23_P406448	1.1	-1.1	-1.1	-4.7	RAB9B	Homo sapiens RAB9B, member RAS oncogene family (RAB9B), mRNA [NM_016370]	51209
A_32_P142013	3.1	2.4	1.3	2.8	RABEP1	Homo sapiens rabaptin, RAB GTPase binding effector protein 1 (RABEP1), transcript variant 2, mRNA [NM_001083585]	9135

A_23_P78158	5.5	5.3	5.3	5.3	RABEP1	Homo sapiens rabaptin, RAB GTPase binding effector protein 1 (RABEP1), transcript variant 1, mRNA [NM_004703]	9135
A_24_P399174	1.1	-5.3	1.0	1.1	RABEP1	Homo sapiens rabaptin, RAB GTPase binding effector protein 1 (RABEP1), transcript variant 1, mRNA [NM_004703]	9135
A_23_P61100	1.6	3.1	2.4	1.8	RABEP2	Homo sapiens rabaptin, RAB GTPase binding effector protein 2 (RABEP2), mRNA [NM_024816]	79874

A_24_P386323	-2.2	-1.4	-1.4	-3.3	RABEPK	Homo sapiens Rab9 effector protein with kelch motifs (RABEPK), transcript variant 1, mRNA [NM_005833]	10244
A_24_P252575	-4.2	-1.0	-1.1	-1.1	RABGAP1	Homo sapiens RAB GTPase activating protein 1 (RABGAP1), mRNA [NM_012197]	23637
A_23_P350895	1.0	2.5	1.0	-1.0	RABGAP1	RAB GTPase activating protein 1 [Source:HGNC Symbol;Acc:17155] [ENST00000317419]	23637
A_23_P377267	1.0	2.9	2.6	2.6	RABGAP1L	Homo sapiens RAB GTPase activating protein 1-like (RABGAP1L), transcript variant 4, mRNA [NM_001243765]	9910

A_23_P200325	2.2	1.6	3.1	1.6	RABGAP1L	Homo sapiens RAB GTPase activating protein 1-like (RABGAP1L), transcript variant 1, mRNA [NM_014857]	9910
A_24_P333525	2.4	3.5	2.4	2.3	RABGAP1L	Homo sapiens RAB GTPase activating protein 1-like (RABGAP1L), transcript variant 1, mRNA [NM_014857]	9910
A_24_P337746	1.3	1.3	1.2	-3.1	RABGEF1	Homo sapiens RAB guanine nucleotide exchange factor (GEF) 1 (RABGEF1), mRNA [NM_014504]	27342

A_23_P63503	1.6	2.9	-1.7	-1.5	RABIF	Homo sapiens RAB interacting factor (RABIF), mRNA [NM_002871]	5877
A_24_P250650	3.2	1.9	2.0	1.2	RABL2A	Homo sapiens RAB, member of RAS oncogene family-like 2A (RABL2A), transcript variant 1, mRNA [NM_013412]	11159
A_24_P911094	-2.2	1.0	-1.0	-1.9	RABL2A	RAB, member of RAS oncogene family-like 2A [Source:HGNC Symbol;Acc:9799] [ENST00000376439]	11159
A_32_P221452	1.4	-2.2	1.3	-1.0	RABL3	Homo sapiens RAB, member of RAS oncogene family-like 3 (RABL3), mRNA [NM_173825]	285282

A_32_P109057	-1.2	-2.6	2.0	-1.3	RABL3	Homo sapiens RAB, member of RAS oncogene family-like 3 (RABL3), mRNA [NM_173825]	285282
A_23_P31584	-2.0	-2.4	-2.6	-1.5	RABL5	Homo sapiens RAB, member RAS oncogene family-like 5 (RABL5), transcript variant 1, mRNA [NM_022777]	64792
A_24_P286527	-2.4	-2.1	1.1	-1.0	RABL5	Homo sapiens RAB, member RAS oncogene family-like 5 (RABL5), transcript variant 1, mRNA [NM_022777]	64792

A_32_P217709	1.1	1.1	-1.4	2.1	RAC1	Homo sapiens ras-related C3 botulinum toxin substrate 1 (rho family, small GTP binding protein Rac1) (RAC1), transcript variant Rac1, mRNA [NM_006908]	5879
A_24_P194845	2.9	2.5	1.0	-1.0	RAC1	Homo sapiens ras-related C3 botulinum toxin substrate 1 (rho family, small GTP binding protein Rac1) (RAC1), transcript variant Rac1, mRNA [NM_006908]	5879

A_23_P218774	2.2	2.4	2.3	3.3	RAC2	Homo sapiens ras-related C3 botulinum toxin substrate 2 (rho family, small GTP binding protein Rac2) (RAC2), mRNA [NM_002872]	5880
A_23_P218770	2.8	3.0	-1.1	4.6	RAC2	Homo sapiens ras-related C3 botulinum toxin substrate 2 (rho family, small GTP binding protein Rac2) (RAC2), mRNA [NM_002872]	5880

A_23_P125001	-2.3	1.5	1.0	1.8	RAC3	Homo sapiens ras-related C3 botulinum toxin substrate 3 (rho family, small GTP binding protein Rac3) (RAC3), mRNA [NM_005052]	5881
A_32_P186474	1.9	2.0	2.0	1.0	RACGAP1	Homo sapiens Rac GTPase activating protein 1 (RACGAP1), transcript variant 1, mRNA [NM_013277]	29127
A_23_P65041	1.3	1.0	1.1	-3.5	RACGAP1P	Homo sapiens Rac GTPase activating protein 1 pseudogene (RACGAP1P), non-coding RNA [NR_026583]	83956

A_23_P144697	-1.1	1.3	-2.7	-1.9	RAD1	Homo sapiens RAD1 homolog (S. pombe) (RAD1), transcript variant 1, mRNA [NM_002853]	5810
A_24_P22887	1.1	-8.7	-1.3	-1.3	RAD23A	Homo sapiens RAD23 homolog A (S. cerevisiae) (RAD23A), mRNA [NM_005053]	5886
A_24_P406664	-1.3	-9.5	-1.4	-1.5	RAD23B	Homo sapiens RAD23 homolog B (S. cerevisiae) (RAD23B), transcript variant 1, mRNA [NM_002874]	5887
A_24_P226198	1.0	-1.0	-4.5	-1.0	RAD50	Homo sapiens RAD50 homolog (S. cerevisiae) (RAD50), mRNA [NM_005732]	10111

A_23_P500873	-2.3	-1.0	-3.4	1.5	RAD51D	Homo sapiens RAD51 homolog D (S. cerevisiae) (RAD51D), transcript variant 4, mRNA [NM_133629]	5892
A_24_P40827	1.5	-4.0	-1.2	1.3	RAD51D	Homo sapiens RAD51 homolog D (S. cerevisiae) (RAD51D), transcript variant 1, mRNA [NM_002878]	5892
A_23_P64990	-1.1	-1.7	-1.5	-18.3	RAD52	Homo sapiens RAD52 homolog (S. cerevisiae) (RAD52), mRNA [NM_134424]	5893
A_24_P21715	1.5	-12.5	-1.0	-1.0	RAD9A	Homo sapiens RAD9 homolog A (S. pombe) (RAD9A), transcript variant 1, mRNA [NM_004584]	5883

A_23_P6023	-2.3	-2.6	-2.0	1.2	RAE1	RAE1 RNA export 1 homolog (S. pombe) [Source:HGNC Symbol;Acc:9828] [ENST00000371242]	8480
A_23_P8253	-4.3	-1.1	-1.8	-1.1	RAET1E	Homo sapiens retinoic acid early transcript 1E (RAET1E), transcript variant 1, mRNA [NM_139165]	135250
A_23_P64525	-1.1	-2.6	-1.0	1.1	RAG2	Homo sapiens recombination activating gene 2 (RAG2), transcript variant 1, mRNA [NM_000536]	5897
A_23_P77807	-3.9	1.4	-3.0	-1.8	RAI1	Homo sapiens retinoic acid induced 1 (RAI1), mRNA [NM_030665]	10743

A_23_P254165	1.5	-1.0	-2.4	-1.1	RAI2	Homo sapiens retinoic acid induced 2 (RAI2), transcript variant 2, mRNA [NM_021785]	10742
A_24_P192262	-1.1	-14.8	-1.1	-1.1	RALA	Homo sapiens v-ral simian leukemia viral oncogene homolog A (ras related) (RALA), mRNA [NM_005402]	5898
A_23_P168479	-1.0	-1.0	-3.2	-1.0	RALA	Homo sapiens v-ral simian leukemia viral oncogene homolog A (ras related) (RALA), mRNA [NM_005402]	5898
A_23_P153084	-1.0	1.2	-2.4	3.8	RALBP1	Homo sapiens ralA binding protein 1 (RALBP1), mRNA [NM_006788]	10928

A_24_P918843	-2.3	-2.1	1.0	-1.2	RALGAPA1	Homo sapiens Ral GTPase activating protein, alpha subunit 1 (catalytic) (RALGAPA1), transcript variant 2, mRNA [NM_194301]	253959
A_23_P135184	-1.1	-1.5	-36.4	-1.5	RALGDS	Homo sapiens ral guanine nucleotide dissociation stimulator (RALGDS), transcript variant 2, mRNA [NM_001042368]	5900
A_32_P221991	1.2	1.2	-16.1	-1.2	RALGPS1	Homo sapiens Ral GEF with PH domain and SH3 binding motif 1 (RALGPS1), transcript variant 1, mRNA [NM_014636]	9649

A_23_P390148	1.3	2.3	2.0	-1.0	RALGPS1	Homo sapiens Ral GEF with PH domain and SH3 binding motif 1 (RALGPS1), transcript variant 1, mRNA [NM_014636]	9649
A_23_P109235	-14.4	1.2	-1.8	1.1	RALY	Homo sapiens RNA binding protein, autoantigenic (hnRNP-associated with lethal yellow homolog (mouse)) (RALY), transcript variant 1, mRNA [NM_016732]	22913
A_23_P128126	1.1	1.1	-3.2	1.1	RAN	RAN, member RAS oncogene family [Source:HGNC Symbol;Acc:9846] [ENST00000543796]	5901

A_23_P369733	2.5	4.0	2.8	2.4	RANBP10	Homo sapiens RAN binding protein 10 (RANBP10), mRNA [NM_020850]	57610
A_24_P350890	-8.0	1.0	-1.0	-1.0	RANBP2	Homo sapiens RAN binding protein 2 (RANBP2), mRNA [NM_006267]	5903
A_24_P241862	2.0	2.7	1.3	2.8	RANBP3	Homo sapiens RAN binding protein 3 (RANBP3), transcript variant RANBP3-b, mRNA [NM_007320]	8498
A_23_P391207	3.6	1.3	3.5	3.8	RANBP3L	Homo sapiens RAN binding protein 3-like (RANBP3L), transcript variant 2, mRNA [NM_145000]	202151

A_23_P55136	-1.0	-19.9	-1.5	-1.4	RANGRF	Homo sapiens RAN guanine nucleotide release factor (RANGRF), transcript variant 1, mRNA [NM_016492]	29098
A_23_P51754	-2.3	-3.6	-1.8	-1.8	RAP1A	Homo sapiens RAP1A, member of RAS oncogene family (RAP1A), transcript variant 1, mRNA [NM_001010935]	5906
A_24_P36890	-1.5	1.1	-1.8	-2.2	RAP1GAP	Homo sapiens RAP1 GTPase activating protein (RAP1GAP), transcript variant 3, mRNA [NM_002885]	5909

A_32_P85813	1.1	-6.2	1.5	1.1	RAP1GDS1	Homo sapiens RAP1, GTP-GDP dissociation stimulator 1 (RAP1GDS1), transcript variant 1, mRNA [NM_001100426]	5910
A_24_P374319	2.3	2.3	2.2	3.4	RAP2C	Homo sapiens RAP2C, member of RAS oncogene family (RAP2C), mRNA [NM_021183]	57826
A_23_P147826	-1.5	-5.3	-1.7	-1.3	RAP2C	Homo sapiens RAP2C, member of RAS oncogene family (RAP2C), mRNA [NM_021183]	57826

A_23_P304237	-1.9	-3.8	-1.7	-6.2	RAPGEF1	Homo sapiens Rap guanine nucleotide exchange factor (GEF) 1 (RAPGEF1), transcript variant 2, mRNA [NM_198679]	2889
A_24_P912587	24.1	5.3	23.8	24.4	RAPGEF2	Rap guanine nucleotide exchange factor (GEF) 2 [Source:HGNC Symbol;Acc:16854] [ENST00000503328]	9693
A_32_P393316	2.3	2.3	3.0	2.3	RAPGEF3	Homo sapiens Rap guanine nucleotide exchange factor (GEF) 3 (RAPGEF3), transcript variant 1, mRNA [NM_001098531]	10411

A_23_P17197	-1.1	-1.1	1.0	-2.0	RAPGEF4	Homo sapiens Rap guanine nucleotide exchange factor (GEF) 4 (RAPGEF4), transcript variant 1, mRNA [NM_007023]	11069
A_23_P152970	3.8	5.5	3.6	3.4	RAPGEFL1	Homo sapiens Rap guanine nucleotide exchange factor (GEF)-like 1 (RAPGEFL1), mRNA [NM_016339]	51195
A_23_P409966	-3.8	-1.0	1.4	1.0	RAPH1	Ras association (RalGDS/AF-6) and pleckstrin homology domains 1 [Source:HGNC Symbol;Acc:14436] [ENST00000453034]	65059

A_24_P929570	1.0	-3.3	-1.2	-1.0	RAPH1	Homo sapiens Ras association (RalGDS/AF-6) and pleckstrin homology domains 1 (RAPH1), transcript variant 1, mRNA [NM_213589]	65059
A_32_P5251	1.7	3.5	1.6	1.1	RARA	Homo sapiens retinoic acid receptor, alpha (RARA), transcript variant 2, mRNA [NM_001024809]	5914
A_23_P117125	1.2	2.4	2.2	1.1	RARG	Homo sapiens retinoic acid receptor, gamma (RARG), transcript variant 1, mRNA [NM_000966]	5916

A_24_P354257	-1.4	-7.9	-1.2	-1.0	RASA2	RAS p21 protein activator 2 [Source:HGNC Symbol;Acc:9872] [ENST00000286364]	5922
A_23_P385673	1.7	1.4	-2.6	1.8	RASA3	Homo sapiens mRNA for Ins(1,3,4,5)P4-binding protein. [X89399]	22821
A_23_P168449	1.9	1.0	2.0	2.5	RASA4	Homo sapiens RAS p21 protein activator 4 (RASA4), transcript variant 1, mRNA [NM_006989]	10156
A_23_P139600	1.7	1.0	-1.4	-2.2	RASAL1	Homo sapiens RAS protein activator like 1 (GAP1 like) (RASAL1), transcript variant 2, mRNA [NM_004658]	8437

A_23_P79069	2.5	1.4	3.0	1.5	RASAL3	Homo sapiens RAS protein activator like 3 (RASAL3), mRNA [NM_022904]	64926
A_23_P118392	1.8	1.1	2.0	1.1	RASD1	Homo sapiens RAS, dexamethasone-induced 1 (RASD1), transcript variant 1, mRNA [NM_016084]	51655
A_24_P348006	-1.5	-1.4	-1.7	-2.7	RASD1	Homo sapiens RAS, dexamethasone-induced 1 (RASD1), transcript variant 1, mRNA [NM_016084]	51655
A_32_P231568	1.0	-1.0	3.6	-1.0	RASEF	Homo sapiens RAS and EF-hand domain containing (RASEF), mRNA [NM_152573]	158158

A_23_P391344	2.6	2.4	-1.8	3.6	RASGEF1A	Homo sapiens RasGEF domain family, member 1A, mRNA (cDNA clone MGC:26821 IMAGE:4814750), complete cds. [BC022548]	221002
A_24_P930432	1.0	-6.8	-1.8	1.2	RASGEF1B	RasGEF domain family, member 1B [Source:HGNC Symbol;Acc:24881] [ENST00000395570]	153020
A_23_P64058	-1.1	-1.3	-12.3	1.2	RASGRP2	Homo sapiens RAS guanyl releasing protein 2 (calcium and DAG-regulated) (RASGRP2), transcript variant 2, mRNA [NM_153819]	10235

A_24_P54390	2.4	-5.7	1.9	1.9	RASGRP3	Homo sapiens RAS guanyl releasing protein 3 (calcium and DAG-regulated) (RASGRP3), transcript variant 2, mRNA [NM_170672]	25780
A_23_P165007	-4.0	-1.5	-1.8	-1.6	RASGRP4	Homo sapiens RAS guanyl releasing protein 4 (RASGRP4), transcript variant a, mRNA [NM_170604]	115727
A_32_P364462	1.1	1.4	-1.5	-3.9	RASIP1	Homo sapiens Ras interacting protein 1 (RASIP1), mRNA [NM_017805]	54922

A_23_P166400	-1.5	1.3	-3.9	-2.3	RASL10A	Homo sapiens RAS-like, family 10, member A (RASL10A), transcript variant 2, mRNA [NM_001007279]	10633
A_23_P69738	-1.4	-1.2	-2.3	-1.3	RASL11B	Homo sapiens RAS-like, family 11, member B (RASL11B), mRNA [NM_023940]	65997
A_23_P41066	1.9	-1.1	2.9	1.7	RASSF1	Homo sapiens Ras association (RalGDS/AF-6) domain family member 1 (RASSF1), transcript variant C, mRNA [NM_170713]	11186

A_24_P148777	-1.7	-2.3	-2.0	-1.2	RASSF1	Homo sapiens Ras associatio n (RalGDS/A F-6) domain family member 1 (RASSF1), transcript variant C, mRNA [NM_1707 13]	11186
A_23_P166087	1.3	-1.0	-1.1	-3.2	RASSF2	Homo sapiens Ras associatio n (RalGDS/A F-6) domain family member 2 (RASSF2), transcript variant 1, mRNA [NM_0147 37]	9770
A_24_P288448	6.5	6.7	12.2	7.3	RASSF2	Ras associatio n (RalGDS/A F-6) domain family member 2 [Source:H GNC Symbol;Ac c:9883] [ENST0000 0478553]	9770

A_23_P2397	2.5	2.4	-1.1	-1.1	RASSF3	Homo sapiens Ras association (RalGDS/AF-6) domain family member 3 (RASSF3), transcript variant 1, mRNA [NM_178169]	283349
A_24_P171268	1.1	-2.0	-1.0	-2.0	RASSF5	Homo sapiens Ras association (RalGDS/AF-6) domain family member 5 (RASSF5), transcript variant 1, mRNA [NM_182663]	83593

A_23_P47116	-32.3	2.2	-1.3	1.6	RASSF7	Homo sapiens Ras association (RalGDS/AF-6) domain family (N-terminal) member 7 (RASSF7), transcript variant 1, mRNA [NM_003475]	8045
A_24_P407224	1.0	-1.0	-3.7	-1.1	RASSF8	Homo sapiens Ras association (RalGDS/AF-6) domain family (N-terminal) member 8 (RASSF8), transcript variant 1, mRNA [NM_001164746]	11228

A_24_P199251	1.0	-3.4	-1.1	-1.0	RASSF9	Homo sapiens Ras associatio n (RalGDS/A F-6) domain family (N-terminal) member 9 (RASSF9), mRNA [NM_0054 47]	9182
A_24_P126557	-1.5	-5.4	-1.8	1.0	RAVER1	Homo sapiens ribonucleo protein, PTB-binding 1 (RAVER1), mRNA [NM_1334 52]	125950
A_23_P74215	-1.1	-2.0	-2.9	-1.3	RAVER2	Homo sapiens cDNA FLJ10770 fis, clone NT2RP400 0159. [AK001632]	55225
A_23_P56146	1.2	2.4	1.0	-1.1	RAX2	Homo sapiens retina and anterior neural fold homeobox 2 (RAX2), mRNA [NM_0327 53]	84839

A_23_P56150	-1.6	1.6	1.6	-2.4	RAX2	Homo sapiens retina and anterior neural fold homeobox 2 (RAX2), mRNA [NM_032753]	84839
A_24_P102636	-3.3	-4.1	1.6	1.6	RB1	Homo sapiens retinoblastoma 1 (RB1), mRNA [NM_000321]	5925
A_24_P72394	-1.9	-1.2	-4.1	-2.2	RBAK	Homo sapiens RB-associated KRAB zinc finger (RBAK), transcript variant 1, mRNA [NM_021163]	57786
A_32_P171143	-1.4	-1.6	-2.1	-1.0	RBAK-LOC389	Homo sapiens RBAK-LOC389458 readthrough (RBAK-LOC389458), mRNA [NM_001204513]	100533952

A_24_P468810	-3.5	-2.3	1.0	1.1	RBBP4	Homo sapiens retinoblastoma binding protein 4 (RBBP4), transcript variant 1, mRNA [NM_005610]	5928
A_24_P912095	-2.1	1.2	1.0	-1.6	RBBP4	Homo sapiens retinoblastoma binding protein 4 (RBBP4), transcript variant 1, mRNA [NM_005610]	5928
A_23_P326691	2.9	3.0	-1.4	1.7	RBBP4	Homo sapiens retinoblastoma binding protein 4 (RBBP4), transcript variant 1, mRNA [NM_005610]	5928
A_23_P201887	-1.0	-1.0	-1.0	-3.7	RBBP5	Homo sapiens retinoblastoma binding protein 5 (RBBP5), transcript variant 1, mRNA [NM_005057]	5929

A_23_P363206	-1.9	1.0	2.4	-5.3	RBBP6	Homo sapiens retinoblastoma binding protein 6 (RBBP6), transcript variant 1, mRNA [NM_006910]	5930
A_23_P252371	3.3	4.3	3.1	3.1	RBBP8	Homo sapiens retinoblastoma binding protein 8 (RBBP8), transcript variant 1, mRNA [NM_002894]	5932
A_23_P359504	-2.2	-2.2	-3.9	-2.4	RBBP9	Homo sapiens retinoblastoma binding protein (RBBP9) mRNA, complete cds. [AF039564]	10741

A_23_P166051	3.4	10.9	6.9	1.0	RBCK1	Homo sapiens RanBP-type and C3HC4-type zinc finger containing 1 (RBCK1), transcript variant 2, mRNA [NM_031229]	10616
A_23_P153086	-1.3	1.5	1.9	2.3	RBFA	Homo sapiens ribosome binding factor A (putative) (RBFA), nuclear gene encoding mitochondrial protein, transcript variant 1, mRNA [NM_024805]	79863
A_23_P140858	2.3	2.1	3.4	1.1	RBFOX1	Homo sapiens RNA binding protein, fox-1 homolog (C. elegans) 1 (RBFOX1), transcript variant 4, mRNA [NM_018723]	54715

A_24_P147242	50.8	50.4	49.4	3.6	RBFOX1	Homo sapiens RNA binding protein, fox-1 homolog (C. elegans) 1 (RBFOX1), transcript variant 3, mRNA [NM_145893]	54715
A_23_P103099	-1.2	1.3	1.2	2.2	RBFOX2	Homo sapiens RNA binding protein, fox-1 homolog (C. elegans) 2 (RBFOX2), transcript variant 1, mRNA [NM_001031695]	23543
A_24_P142343	-1.3	1.4	1.3	-3.5	RBFOX3	Homo sapiens RNA binding protein, fox-1 homolog (C. elegans) 3 (RBFOX3), mRNA [NM_001082575]	146713

A_23_P9523	-1.5	-1.6	-1.1	-2.1	RBKS	Homo sapiens ribokinase (RBKS), mRNA [NM_022128]	64080
A_24_P276102	-3.1	1.1	-9.5	1.1	RBL1	Homo sapiens retinoblastoma-like 1 (p107) (RBL1), transcript variant 2, mRNA [NM_183404]	5933
A_23_P28733	1.0	-2.2	-1.3	-1.1	RBL1	Homo sapiens retinoblastoma-like 1 (p107) (RBL1), transcript variant 1, mRNA [NM_002895]	5933
A_23_P26413	2.1	1.8	1.5	1.7	RBL2	Homo sapiens retinoblastoma-like 2 (p130) (RBL2), mRNA [NM_005611]	5934

A_23_P125815	13.3	13.6	2.4	4.0	RBM10	Homo sapiens RNA binding motif protein 10 (RBM10), transcript variant 1, mRNA [NM_005676]	8241
A_23_P342000	-4.5	-6.9	-1.2	1.3	RBM11	Homo sapiens RNA binding motif protein 11 (RBM11), mRNA [NM_144770]	54033
A_23_P502832	-3.1	-1.2	-4.0	-1.2	RBM12	Homo sapiens RNA binding motif protein 12 (RBM12), transcript variant 1, mRNA [NM_006047]	10137
A_24_P945181	-6.0	-1.0	-1.1	1.2	RBM15B	Homo sapiens RNA binding motif protein 15B (RBM15B), mRNA [NM_013286]	29890

A_23_P121405	3.0	1.4	2.3	2.0	RBM15B	Homo sapiens RNA binding motif protein 15B (RBM15B), mRNA [NM_013286]	29890
A_23_P35645	-1.1	1.3	-6.1	-1.1	RBM17	Homo sapiens RNA binding motif protein 17 (RBM17), transcript variant 1, mRNA [NM_032905]	84991
A_23_P20777	-4.3	1.0	-4.6	-1.8	RBM18	Homo sapiens RNA binding motif protein 18 (RBM18), transcript variant 1, mRNA [NM_033117]	92400
A_24_P226867	1.1	4.7	4.6	4.7	RBM18	Homo sapiens RNA binding motif protein 18 (RBM18), transcript variant 1, mRNA [NM_033117]	92400

A_23_P312344	-1.1	-1.3	-6.3	-1.4	RBM19	Homo sapiens RNA binding motif protein 19 (RBM19), transcript variant 2, mRNA [NM_016196]	9904
A_24_P207091	10.6	10.3	11.7	9.0	RBM23	Homo sapiens RNA binding motif protein 23 (RBM23), transcript variant 1, mRNA [NM_001077351]	55147
A_24_P926189	-1.0	-5.7	-1.0	-1.0	RBM26	RNA binding motif protein 26 [Source:HGNC Symbol;Acc:20327] [ENST00000472143]	64062

A_32_P125803	1.0	1.0	2.9	1.0	RBM26-AS1	Homo sapiens RBM26 antisense RNA 1 (non-protein coding) (RBM26-AS1), non-coding RNA [NR_038991]	100505538
A_24_P927222	-1.1	-1.3	-1.3	-3.2	RBM27	Homo sapiens RNA binding motif protein 27 (RBM27), mRNA [NM_018989]	54439
A_23_P168629	-4.7	1.9	1.9	1.0	RBM28	Homo sapiens RNA binding motif protein 28 (RBM28), transcript variant 1, mRNA [NM_018077]	55131
A_23_P215534	1.2	1.2	1.2	-14.0	RBM33	Homo sapiens RNA binding motif protein 33 (RBM33), mRNA [NM_053043]	155435

A_23_P436476	-1.8	1.0	-1.0	-3.5	RBM34	Homo sapiens RNA binding motif protein 34 (RBM34), transcript variant 1, mRNA [NM_015014]	23029
A_24_P418086	-1.0	-4.5	-1.1	-1.0	RBM34	Homo sapiens RNA binding motif protein 34 (RBM34), transcript variant 1, mRNA [NM_015014]	23029
A_24_P14485	-1.2	-3.1	-2.1	1.6	RBM38	Homo sapiens RNA binding motif protein 38 (RBM38), transcript variant 1, mRNA [NM_017495]	55544
A_23_P17430	1.0	-2.1	-1.0	1.1	RBM38	Homo sapiens RNA binding motif protein 38 (RBM38), transcript variant 1, mRNA [NM_017495]	55544

A_23_P13033	-1.5	-1.6	-2.1	-1.2	RBM4	Homo sapiens RNA binding motif protein 4 (RBM4), transcript variant 1, mRNA [NM_002896]	5936
A_24_P404807	2.8	2.2	1.8	2.4	RBM41	Homo sapiens RNA binding motif protein 41 (RBM41), transcript variant 1, mRNA [NM_018301]	55285
A_32_P135091	1.9	-3.6	2.2	2.3	RBM41	Homo sapiens RNA binding motif protein 41 (RBM41), transcript variant 1, mRNA [NM_018301]	55285
A_32_P496952	-1.4	1.4	-1.0	-3.1	RBM43	Homo sapiens RNA binding motif protein 43 (RBM43), mRNA [NM_198557]	375287

A_23_P142994	1.1	1.1	1.0	-6.4	RBM45	Homo sapiens RNA binding motif protein 45 (RBM45), mRNA [NM_152945]	129831
A_24_P226108	1.0	-2.5	1.0	1.0	RBM47	Homo sapiens RNA binding motif protein 47 (RBM47), transcript variant 2, mRNA [NM_019027]	54502
A_32_P62571	1.1	-3.2	-1.1	-1.0	RBM8A	Homo sapiens RNA binding motif protein 8A (RBM8A), mRNA [NM_005105]	9939
A_24_P79955	1.9	2.5	2.5	2.5	RBM8A	Homo sapiens RNA binding motif protein 8A (RBM8A), mRNA [NM_005105]	9939

A_23_P36496	-1.0	5.0	4.0	4.1	RBMS1	Homo sapiens RNA binding motif, single stranded interacting protein 1 (RBMS1), transcript variant 1, mRNA [NM_016836]	5937
A_23_P10995	1.8	1.4	1.0	-2.3	RBMS3	Homo sapiens RNA binding motif, single stranded interacting protein 3 (RBMS3), transcript variant 2, mRNA [NM_014483]	27303
A_23_P96420	1.1	2.3	4.1	1.9	RBMX	Homo sapiens RNA binding motif protein, X-linked (RBMX), transcript variant 1, mRNA [NM_002139]	27316

A_24_P824592	-2.9	-1.0	-3.5	1.1	RBMX	Homo sapiens RNA binding motif protein, X-linked (RBMX), transcript variant 1, mRNA [NM_002139]	27316
A_32_P193646	-1.0	-1.1	-3.2	-1.1	RBMX	Homo sapiens RNA binding motif protein, X-linked (RBMX), transcript variant 1, mRNA [NM_002139]	27316
A_24_P359545	-1.0	-1.1	-2.2	-3.1	RBMXL1	Homo sapiens RNA binding motif protein, X-linked-like 1 (RBMXL1), transcript variant 2, mRNA [NM_019610]	494115

A_23_P61967	-2.0	1.0	-1.1	-17.0	RBMXL2	Homo sapiens RNA binding motif protein, X-linked-like 2 (RBMXL2), mRNA [NM_014469]	27288
A_23_P171397	-2.9	-2.8	-2.9	-4.3	RBMY1B	Homo sapiens RNA binding motif protein, Y-linked, family 1, member B (RBMY1B), mRNA [NM_001006121]	378948
A_24_P794648	-6.7	-1.7	-2.2	-1.7	RBMY2EP	Homo sapiens RNA binding motif protein, Y-linked, family 2, member E pseudogene (RBMY2EP), non-coding RNA [NR_001574]	159125

A_23_P424689	-3.1	-9.3	1.0	-1.0	RBMY2FP	Homo sapiens RNA binding motif protein, Y-linked, family 2, member F pseudogene (RBMY2FP), non-coding RNA [NR_002193]	159162
A_24_P165423	5.3	5.2	3.6	3.7	RBP7	Homo sapiens retinol binding protein 7, cellular (RBP7), mRNA [NM_052960]	116362
A_23_P29994	-1.3	-3.7	-1.4	-1.5	RBPJ	Homo sapiens recombination signal binding protein for immunoglobulin kappa J region (RBPJ), transcript variant 4, mRNA [NM_203284]	3516

A_23_P71316	1.0	3.6	1.9	1.3	RBPMS	Homo sapiens RNA binding protein with multiple splicing (RBPMS), transcript variant 3, mRNA [NM_001008712]	11030
A_23_P375147	2.6	1.2	1.1	2.0	RC3H2	ring finger and CCCH-type domains 2 [Source:HGNC Symbol;Acc:21461] [ENST00000373670]	54542
A_32_P156851	-1.2	-1.2	-1.3	3.3	RCAN2	Homo sapiens regulator of calcineurin 2 (RCAN2), transcript variant 1, mRNA [NM_005822]	10231

A_23_P14105	-1.3	-1.1	-6.5	-1.4	RCBTB2	Homo sapiens regulator of chromosome condensation (RCC1) and BTB (POZ) domain containing protein 2 (RCBTB2), mRNA [NM_001268]	1102
A_24_P930442	-1.7	-2.7	-7.3	-1.5	RCC1	Homo sapiens regulator of chromosome condensation 1 (RCC1), transcript variant 1, mRNA [NM_001048194]	1104
A_23_P46309	-9.2	-1.0	-10.8	-1.0	RCC1	Homo sapiens regulator of chromosome condensation 1 (RCC1), transcript variant 4, mRNA [NM_001048199]	1104

A_23_P74950	4.8	4.8	4.9	4.8	RCC2	Homo sapiens regulator of chromosome condensation 2 (RCC2), transcript variant 1, mRNA [NM_018715]	55920
A_23_P13222	1.8	-1.3	2.4	1.3	RCN1	Homo sapiens reticulocalbin 1, EF-hand calcium binding domain (RCN1), mRNA [NM_002901]	5954
A_32_P175539	-1.0	-1.0	-1.1	-2.9	RCN2	Homo sapiens reticulocalbin 2, EF-hand calcium binding domain (RCN2), mRNA [NM_002902]	5955
A_23_P24397	3.0	1.6	1.8	-1.8	RCOR2	Homo sapiens REST corepressor 2 (RCOR2), mRNA [NM_173587]	283248

A_24_P849628	3.4	-1.5	-2.0	1.4	RDBP	Homo sapiens RD RNA binding protein (RDBP), mRNA [NM_002904]	7936
A_23_P50291	1.8	2.4	-1.7	-1.3	RDH8	Homo sapiens retinol dehydrogenase 8 (all-trans) (RDH8), mRNA [NM_015725]	50700
A_23_P141447	-4.2	1.0	-1.0	1.0	RDM1	Homo sapiens RAD52 motif 1 (RDM1), transcript variant 2, mRNA [NM_001034836]	201299
A_24_P344087	-2.1	1.4	1.5	1.0	REC8	Homo sapiens REC8 homolog (yeast) (REC8), transcript variant 2, mRNA [NM_001048205]	9985

A_24_P415208	-8.9	2.2	-1.2	2.2	RECK	reversion-inducing-cysteine-rich protein with kazal motifs [Source:HGNC Symbol;Acc:11345] [ENST00000475774]	8434
A_23_P83028	1.1	1.0	1.0	2.7	RECK	Homo sapiens reversion-inducing-cysteine-rich protein with kazal motifs (RECK), mRNA [NM_021111]	8434
A_23_P71558	-2.0	-1.3	-3.1	1.0	RECQL4	Homo sapiens RecQ protein-like 4 (RECQL4), mRNA [NM_004260]	9401

A_23_P96285	1.5	-1.3	1.9	-2.2	REEP1	Homo sapiens receptor accessory protein 1 (REEP1), nuclear gene encoding mitochondrial protein, transcript variant 2, mRNA [NM_022912]	65055
A_23_P19182	-1.6	1.0	-4.3	-1.6	REEP2	Homo sapiens receptor accessory protein 2 (REEP2), mRNA [NM_016606]	51308
A_24_P286845	1.6	1.7	4.0	1.9	REEP3	Homo sapiens receptor accessory protein 3 (REEP3), mRNA [NM_001001330]	221035
A_23_P75028	3.5	2.6	5.8	-2.5	REEP3	Homo sapiens receptor accessory protein 3 (REEP3), mRNA [NM_001001330]	221035

A_23_P90510	-12.0	1.1	-2.5	-1.8	REEP6	Homo sapiens receptor accessory protein 6 (REEP6), mRNA [NM_138393]	92840
A_23_P90743	1.5	1.6	2.4	1.0	REG1A	Homo sapiens regenerating islet-derived 1 alpha (REG1A), mRNA [NM_002909]	5967
A_24_P58673	1.9	-1.8	-2.4	-1.3	REG4	Homo sapiens regenerating islet-derived family, member 4 (REG4), transcript variant 2, mRNA [NM_032044]	83998
A_23_P56938	-1.3	-1.8	-2.1	-1.3	REL	Homo sapiens v-rel reticuloendotheliosis viral oncogene homolog (avian) (REL), mRNA [NM_002908]	5966

A_24_P937256	-1.2	1.7	8.4	-1.2	RELA	Homo sapiens v-rel reticuloendotheliosis viral oncogene homolog A (avian) (RELA), transcript variant 4, mRNA [NM_001243985]	5970
A_24_P38951	1.8	2.5	2.5	2.2	RELT	Homo sapiens RELT tumor necrosis factor receptor (RELT), transcript variant 1, mRNA [NM_032871]	84957
A_23_P151624	2.4	-1.0	-3.0	-7.3	REM2	Homo sapiens RAS (RAD and GEM)-like GTP binding 2 (REM2), mRNA [NM_173527]	161253
A_23_P34637	4.8	-1.4	2.7	3.4	REN	Homo sapiens renin (REN), mRNA [NM_000537]	5972

A_23_P137157	-2.1	-1.1	1.6	-1.7	RENBP	Homo sapiens renin binding protein (RENBP), mRNA [NM_002910]	5973
A_24_P166645	5.5	2.4	2.1	1.7	REPIN1	Homo sapiens replication initiator 1 (REPIN1), transcript variant 2, mRNA [NM_014374]	29803
A_23_P70748	3.0	1.4	-1.6	-2.3	REPS1	Homo sapiens RALBP1 associated Eps domain containing 1 (REPS1), transcript variant 1, mRNA [NM_031922]	85021

A_24_P79591	1.1	-16.0	1.2	1.1	RER1	Homo sapiens RER1 retention in endoplasmic reticulum 1 homolog (S. cerevisiae) (RER1), mRNA [NM_007033]	11079
A_24_P342591	1.2	1.6	-1.0	-2.6	RERE	Homo sapiens arginine-glutamic acid dipeptide (RE) repeats (RERE), transcript variant 1, mRNA [NM_012102]	473
A_24_P321525	-1.1	1.1	1.4	-2.5	RERG	Homo sapiens RAS-like, estrogen-regulated, growth inhibitor (RERG), transcript variant 1, mRNA [NM_032918]	85004

A_24_P59236	-6.2	1.1	1.2	1.1	RESP18	Homo sapiens regulated endocrine-specific protein 18 homolog (rat) (RESP18), mRNA [NM_001007089]	389075
A_24_P246787	8.9	3.0	3.5	-1.2	REST	Homo sapiens RE1-silencing transcription factor (REST), transcript variant 1, mRNA [NM_005612]	5978
A_24_P299507	2.4	-1.1	-1.1	-1.2	REST	Human neural-restrictive silencer factor, splice variant mRNA, partial cds. [U13877]	5978
A_23_P202245	-1.3	-1.0	-1.1	-2.9	RET	Homo sapiens ret proto-oncogene (RET), transcript variant 2, mRNA [NM_020975]	5979

A_23_P92196	1.4	-2.1	1.7	1.4	RETNLB	Homo sapiens resistin like beta (RETNLB), mRNA [NM_032579]	84666
A_24_P291426	-19.8	1.0	-1.1	1.0	REV1	Homo sapiens REV1 homolog (S. cerevisiae) (REV1), transcript variant 1, mRNA [NM_016316]	51455
A_24_P256325	-1.1	1.7	2.0	1.1	REV1	Homo sapiens REV1 homolog (S. cerevisiae) (REV1), transcript variant 2, mRNA [NM_001037872]	51455
A_32_P390781	1.4	-2.5	-2.7	1.2	REXO1	Homo sapiens REX1, RNA exonuclease 1 homolog (S. cerevisiae) (REXO1), mRNA [NM_020695]	57455

A_23_P428373	1.2	1.2	2.4	-1.1	REXO1L1	Homo sapiens REX1, RNA exonuclease 1 homolog (S. cerevisiae)-like 1 (REXO1L1), mRNA [NM_172239]	254958
A_23_P93823	-1.0	-1.1	-1.1	-6.7	RFC2	Homo sapiens replication factor C (activator 1) 2, 40kDa (RFC2), transcript variant 1, mRNA [NM_181471]	5982
A_23_P14193	-5.5	-2.4	-2.3	-2.4	RFC3	Homo sapiens replication factor C (activator 1) 3, 38kDa (RFC3), transcript variant 1, mRNA [NM_002915]	5983

A_32_P197016	3.1	3.1	6.7	1.9	RFC3	Homo sapiens replication factor C (activator 1) 3, 38kDa (RFC3), transcript variant 2, mRNA [NM_181558]	5983
A_23_P18196	-1.0	-1.5	-3.2	-1.1	RFC4	Homo sapiens replication factor C (activator 1) 4, 37kDa (RFC4), transcript variant 1, mRNA [NM_002916]	5984
A_23_P95302	6.4	6.4	4.3	6.8	RFC5	Homo sapiens replication factor C (activator 1) 5, 36.5kDa (RFC5), transcript variant 2, mRNA [NM_181578]	5985

A_23_P251647	1.1	1.1	-3.5	-1.4	RFESD	Homo sapiens Rieske (Fe-S) domain containing (RFESD), transcript variant 2, mRNA [NM_173362]	317671
A_32_P218883	1.1	1.0	-1.0	-11.3	RFK	Homo sapiens riboflavin kinase (RFK), mRNA [NM_018339]	55312
A_24_P138912	-1.7	-1.3	-2.4	1.4	RFNG	Homo sapiens RFNG O-fucosylpeptide 3-beta-N-acetylglucosaminyltransferase (RFNG), mRNA [NM_002917]	5986
A_23_P254434	1.0	1.2	-7.4	-2.8	RFPL2	Homo sapiens ret finger protein-like 2 (RFPL2), transcript variant 1, mRNA [NM_006605]	10739

A_24_P169048	3.8	3.7	3.7	3.7	RFPL3-AS1	Homo sapiens RFPL3 antisense RNA 1 (non-protein coding) (RFPL3-AS1), antisense RNA [NR_001450]	10737
A_23_P155441	1.7	-1.0	-12.4	-1.1	RFT1	Homo sapiens RFT1 homolog (S. cerevisiae) (RFT1), mRNA [NM_052859]	91869
A_24_P408715	1.9	1.3	2.0	1.0	RFT1	Homo sapiens RFT1 homolog (S. cerevisiae) (RFT1), mRNA [NM_052859]	91869
A_23_P315571	1.7	1.7	2.2	1.0	RFTN1	Homo sapiens raftlin, lipid raft linker 1 (RFTN1), mRNA [NM_015150]	23180

A_23_P363878	1.0	-1.1	-1.3	-2.6	RFTN2	Homo sapiens raftlin family member 2 (RFTN2), mRNA [NM_144629]	130132
A_24_P685241	-2.5	1.0	1.0	1.1	RFX3	regulatory factor X, 3 (influences HLA class II expression) [Source:HGNC Symbol;Acc:9984] [ENST00000382004]	5991
A_23_P9289	1.1	-8.5	-1.0	1.5	RFX3	Homo sapiens regulatory factor X, 3 (influences HLA class II expression) (RFX3), transcript variant 2, mRNA [NM_134428]	5991
A_23_P26107	-1.0	1.4	-1.8	3.2	RFX7	Homo sapiens regulatory factor X, 7 (RFX7), mRNA [NM_022841]	64864

A_23_P165180	2.5	1.8	1.1	3.0	RFXANK	Homo sapiens regulatory factor X-associated ankyrin-containing protein (RFXANK), transcript variant 1, mRNA [NM_003721]	8625
A_23_P400459	-2.7	1.1	1.2	1.1	RG9MTD3	Homo sapiens RNA (guanine-9-) methyltransferase domain containing 3 (RG9MTD3), mRNA [NM_144964]	158234
A_24_P289954	-1.4	-14.9	-1.6	-1.5	RGAG4	Homo sapiens retrotransposon gag domain containing 4 (RGAG4), mRNA [NM_001024455]	340526

A_23_P93383	-2.5	1.2	-2.6	-1.5	RGL2	Homo sapiens ral guanine nucleotide dissociation stimulator-like 2 (RGL2), transcript variant 1, mRNA [NM_004761]	5863
A_24_P253454	1.1	-2.0	1.4	-1.0	RGMA	Homo sapiens RGM domain family, member A (RGMA), transcript variant 4, mRNA [NM_020211]	56963
A_23_P114423	-5.5	1.1	-1.0	1.1	RGN	Homo sapiens regucalcin (senescence marker protein-30) (RGN), transcript variant 2, mRNA [NM_152869]	9104

A_24_P177932	-2.9	1.1	1.8	1.2	RGNEF	Homo sapiens 190 kDa guanine nucleotide exchange factor (RGNEF), transcript variant 2, mRNA [NM_001177693]	64283
A_23_P135015	1.1	1.0	-1.0	-23.6	RGP1	Homo sapiens RGP1 retrograde golgi transport homolog (S. cerevisiae) (RGP1), mRNA [NM_001080496]	9827
A_32_P108544	2.4	1.0	-1.4	-2.2	RGPD2	Homo sapiens RANBP2-like and GRIP domain containing 2 (RGPD2), mRNA [NM_001078170]	729857

A_23_P218637	2.0	2.0	2.0	1.0	RGPD5	Homo sapiens RANBP2-like and GRIP domain containing 5 (RGPD5), transcript variant 1, mRNA [NM_005054]	84220
A_23_P357985	1.0	-1.0	-1.0	2.0	RGPD6	Homo sapiens RANBP2-like and GRIP domain containing 6 (RGPD6), transcript variant 2, mRNA [NM_001037866]	729540
A_24_P289273	1.4	1.0	1.2	-2.9	RGR	retinal G protein coupled receptor [Source:HGNC Symbol;Acc:9990] [ENST00000372092]	5995

A_24_P325118	2.2	2.7	-22.9	-2.7	RGS11	regulator of G-protein signaling 11 [Source:HGNC Symbol;Acc:9993] [ENST00000397768]	8786
A_23_P118122	2.1	3.0	5.9	1.9	RGS11	Homo sapiens regulator of G-protein signaling 11 (RGS11), transcript variant 2, mRNA [NM_003834]	8786
A_23_P69513	-1.3	-3.3	-3.1	-1.1	RGS12	Homo sapiens regulator of G-protein signaling 12 (RGS12), transcript variant 1, mRNA [NM_198229]	6002

A_23_P352435	-1.0	1.7	-1.1	-2.9	RGS12	Homo sapiens regulator of G-protein signaling 12 (RGS12), transcript variant 2, mRNA [NM_002926]	6002
A_24_P33705	-1.0	1.1	1.2	-5.1	RGS12	Homo sapiens regulator of G-protein signaling 12 (RGS12), transcript variant 1, mRNA [NM_198229]	6002
A_24_P220786	1.1	-1.6	-3.6	-1.3	RGS12	Homo sapiens regulator of G-protein signaling 12 (RGS12), transcript variant 1, mRNA [NM_198229]	6002

A_23_P61140	1.2	1.3	-2.1	-1.5	RGS14	Homo sapiens regulator of G-protein signaling 14 (RGS14), mRNA [NM_006480]	10636
A_23_P124927	-4.2	-16.6	1.5	1.1	RGS14	Homo sapiens regulator of G-protein signaling 14 (RGS14), mRNA [NM_006480]	10636
A_23_P156861	1.4	1.4	1.3	-24.6	RGS17	Homo sapiens regulator of G-protein signaling 17 (RGS17), mRNA [NM_012419]	26575
A_23_P257014	4.1	4.1	2.2	3.1	RGS19	Homo sapiens regulator of G-protein signaling 19 (RGS19), transcript variant 1, mRNA [NM_005873]	10287

A_23_P73097	-22.8	1.1	-2.2	1.1	RGS20	Homo sapiens regulator of G-protein signaling 20 (RGS20), transcript variant 1, mRNA [NM_170587]	8601
A_23_P219197	-8.7	1.1	1.4	-1.4	RGS3	Homo sapiens regulator of G-protein signaling 3 (RGS3), transcript variant 4, mRNA [NM_134427]	5998
A_23_P200737	1.1	-2.5	-1.3	-1.1	RGS4	Homo sapiens regulator of G-protein signaling 4 (RGS4), transcript variant 2, mRNA [NM_005613]	5999

A_23_P51518	2.4	1.8	2.4	1.1	RGS5	Homo sapiens regulator of G-protein signaling 5 (RGS5), transcript variant 1, mRNA [NM_003617]	8490
A_23_P46045	-1.3	-2.4	-2.0	1.0	RGS5	Homo sapiens regulator of G-protein signaling 5 (RGS5), transcript variant 1, mRNA [NM_003617]	8490
A_23_P205666	1.0	-1.6	5.9	1.5	RGS6	Homo sapiens regulator of G-protein signaling 6 (RGS6), transcript variant 2, mRNA [NM_004296]	9628
A_23_P81233	3.3	-1.3	3.2	3.5	RGS7BP	Homo sapiens regulator of G-protein signaling 7 binding protein (RGS7BP), mRNA [NM_001029875]	401190

A_23_P103424	10.5	3.0	4.4	5.7	RGS8	Homo sapiens regulator of G-protein signaling 8 (RGS8), transcript variant 1, mRNA [NM_033345]	85397
A_23_P66881	-1.4	-1.4	-1.6	-2.3	RGS9	Homo sapiens regulator of G-protein signaling 9 (RGS9), transcript variant 1, mRNA [NM_003835]	8787
A_32_P355943	-1.1	-1.0	-2.5	-1.0	RGSL1	Homo sapiens regulator of G-protein signaling like 1 (RGSL1), mRNA [NM_001137669]	353299
A_23_P155009	1.0	2.1	1.3	1.3	RHBDD3	Homo sapiens rhomboid domain containing 3 (RHBDD3), mRNA [NM_012265]	25807

A_23_P163682	-1.0	2.1	-5.3	1.4	RHBDF1	Homo sapiens rhomboid 5 homolog 1 (Drosophila) (RHBDF1), mRNA [NM_022450]	64285
A_32_P46571	1.9	1.9	2.6	1.2	RHBDL2	Homo sapiens rhomboid, veinlet-like 2 (Drosophila) (RHBDL2), mRNA [NM_017821]	54933
A_23_P51690	2.4	3.2	2.1	1.4	RHBG	Homo sapiens Rh family, B glycoprotein (gene/pseudogene) (RHBG), transcript variant 1, mRNA [NM_020407]	57127

A_23_P62634	2.8	2.8	1.6	2.8	RHCE	Homo sapiens Rh blood group, CcEe antigens (RHCE), transcript variant 1, mRNA [NM_020485]	6006
A_23_P151975	2.1	-1.1	3.1	1.0	RHCG	Homo sapiens Rh family, C glycoprotein (RHCG), mRNA [NM_016321]	51458
A_24_P135856	1.0	1.0	-2.1	1.1	RHD	Homo sapiens Rh blood group, D antigen (RHD), transcript variant 1, mRNA [NM_016124]	6007
A_32_P124746	-1.1	1.1	-4.1	1.0	RHEB	Homo sapiens Ras homolog enriched in brain, mRNA (cDNA clone IMAGE:3892637), partial cds. [BC009638]	6009

A_23_P134247	-2.3	-1.2	-1.0	-1.4	RHEB	Homo sapiens Ras homolog enriched in brain (RHEB), mRNA [NM_005614]	6009
A_23_P2307	3.6	-1.4	3.1	3.8	RHEBL1	Homo sapiens Ras homolog enriched in brain like 1 (RHEBL1), mRNA [NM_144593]	121268
A_24_P26181	-1.8	-3.2	-8.9	-2.5	RHO	Homo sapiens rhodopsin (RHO), mRNA [NM_000539]	6010
A_23_P407472	-3.1	1.0	-1.1	-1.6	RHO	Homo sapiens rhodopsin (RHO), mRNA [NM_000539]	6010
A_24_P174550	2.7	1.7	-2.0	1.2	RHOA	Homo sapiens ras homolog gene family, member A (RHOA), mRNA [NM_001664]	387

A_23_P51136	1.3	2.8	1.7	-1.2	RHOB	Homo sapiens ras homolog gene family, member B (RHOB), mRNA [NM_004040]	388
A_23_P1523	1.7	2.2	-2.6	1.7	RHOD	Homo sapiens ras homolog gene family, member D (RHOD), mRNA [NM_014578]	29984
A_24_P104115	1.0	1.7	-2.7	-1.7	RHOF	Homo sapiens ras homolog gene family, member F (in filopodia) (RHOF), mRNA [NM_019034]	54509
A_24_P104119	1.6	1.6	2.7	1.6	RHOF	Homo sapiens ras homolog gene family, member F (in filopodia) (RHOF), mRNA [NM_019034]	54509

A_32_P83915	-1.0	-1.0	-2.1	-1.0	RHOQ	Homo sapiens ras homolog gene family, member Q (RHOQ), mRNA [NM_012249]	23433
A_23_P117912	-2.0	-1.3	-1.5	-1.3	RHOV	Homo sapiens ras homolog gene family, member V (RHOV), mRNA [NM_133639]	171177
A_23_P85082	-5.4	-1.2	-1.3	-1.6	RHOXF1	Homo sapiens Rhox homeobox family, member 1 (RHOXF1), mRNA [NM_139282]	158800
A_23_P352494	-1.2	-1.2	-3.0	-1.2	RHOXF2	Homo sapiens Rhox homeobox family, member 2 (RHOXF2), mRNA [NM_032498]	84528

A_23_P87435	2.4	2.1	2.0	2.7	RHPN1	Homo sapiens rhophilin, Rho GTPase binding protein 1 (RHPN1), mRNA [NM_052924]	114822
A_23_P87432	-1.2	-1.6	1.3	-3.5	RHPN1	Homo sapiens rhophilin, Rho GTPase binding protein 1 (RHPN1), mRNA [NM_052924]	114822
A_23_P73667	2.7	2.1	-1.2	-1.3	RIBC1	Homo sapiens RIB43A domain with coiled-coils 1 (RIBC1), transcript variant 1, mRNA [NM_001031745]	158787

A_24_P115971	-8.6	-2.4	-8.8	-1.8	RIC8A	Homo sapiens resistance to inhibitors of cholinesterase 8 homolog A (C. elegans) (RIC8A), mRNA [NM_021932]	60626
A_23_P64489	1.2	1.4	2.9	2.4	RIC8A	Homo sapiens resistance to inhibitors of cholinesterase 8 homolog A (C. elegans) (RIC8A), mRNA [NM_021932]	60626
A_23_P64932	2.1	2.1	-1.3	2.0	RIC8B	Homo sapiens resistance to inhibitors of cholinesterase 8 homolog B (C. elegans) (RIC8B), mRNA [NM_018157]	55188

A_24_P244644	1.1	-30.5	-1.3	1.0	RIC8B	Homo sapiens resistance to inhibitors of cholinesterase 8 homolog B (C. elegans) (RIC8B), mRNA [NM_018157]	55188
A_24_P34476	-1.2	1.3	-4.3	-1.1	RIF1	Homo sapiens RAP1 interacting factor homolog (yeast) (RIF1), transcript variant 1, mRNA [NM_018151]	55183
A_32_P212471	1.5	1.0	2.1	1.1	RIIAD1	Homo sapiens regulatory subunit of type II PKA R-subunit (RIIa) domain containing 1 (RIIAD1), mRNA [NM_001144956]	284485

A_32_P334325	-1.0	-2.7	1.1	-1.3	RIMBP2	Homo sapiens RIMS binding protein 2 (RIMBP2), mRNA [NM_015347]	23504
A_24_P162226	1.2	1.2	-1.8	-4.6	RIMBP2	Homo sapiens RIMS binding protein 2 (RIMBP2), mRNA [NM_015347]	23504
A_24_P67784	-2.7	1.0	1.1	1.1	RIMBP3	Homo sapiens RIMS binding protein 3 (RIMBP3), mRNA [NM_015672]	85376
A_24_P7157	4.4	4.3	4.4	4.6	RIMKLB	Homo sapiens ribosomal modification protein rimK-like family member B (RIMKLB), mRNA [NM_020734]	57494

A_23_P361841	1.0	1.2	-2.6	-1.1	RIMKLB	Homo sapiens ribosomal modification protein rimK-like family member B (RIMKLB), mRNA [NM_020734]	57494
A_23_P147786	1.3	-2.1	-1.4	1.4	RIMS2	Homo sapiens regulating synaptic membrane exocytosis 2 (RIMS2), transcript variant 2, mRNA [NM_014677]	9699
A_24_P374532	-1.0	1.7	2.0	-1.0	RIMS4	Homo sapiens regulating synaptic membrane exocytosis 4 (RIMS4), transcript variant 2, mRNA [NM_182970]	140730

A_32_P38645	-3.2	-1.6	-1.6	-1.6	RIMS4	Homo sapiens regulating synaptic membrane exocytosis 4 (RIMS4), transcript variant 2, mRNA [NM_182970]	140730
A_23_P120572	-2.8	1.3	2.1	-1.4	RIN2	Homo sapiens Ras and Rab interactor 2 (RIN2), transcript variant 2, mRNA [NM_018993]	54453
A_23_P163200	-1.0	1.7	-1.1	-2.4	RIN3	Homo sapiens cDNA FLJ11700 fis, clone HEMBA1005050. [AK021762]	79890
A_24_P200694	1.8	1.6	1.2	-2.2	RING1	Homo sapiens ring finger protein 1 (RING1), mRNA [NM_002931]	6015

A_23_P55578	2.3	2.3	2.1	2.1	RIOK3	Homo sapiens RIO kinase 3 (yeast) (RIOK3), mRNA [NM_003831]	8780
A_23_P370005	-1.1	-1.1	3.6	-1.2	RIPK1	Homo sapiens receptor (TNFRSF)-interacting serine-threonine kinase 1 (RIPK1), mRNA [NM_003804]	8737
A_23_P252106	1.8	2.1	2.4	1.1	RIPK2	Homo sapiens receptor-interacting serine-threonine kinase 2 (RIPK2), mRNA [NM_003821]	8767
A_23_P211267	3.7	7.3	6.9	1.4	RIPK4	Homo sapiens receptor-interacting serine-threonine kinase 4 (RIPK4), mRNA [NM_020639]	54101

A_32_P199901	1.3	1.5	1.2	-3.2	RIPPLY1	Homo sapiens ripply1 homolog (zebrafish) (RIPPLY1), transcript variant 1, mRNA [NM_138382]	92129
A_23_P316601	1.0	-1.0	2.1	-1.0	RIT1	Homo sapiens Ras-like without CAAX 1 (RIT1), mRNA [NM_006912]	6016
A_23_P163361	1.2	-1.0	-1.1	-5.6	RLBP1	Homo sapiens retinaldehyde binding protein 1 (RLBP1), mRNA [NM_000326]	6017
A_23_P39008	1.2	1.8	1.6	-2.1	RLN3	Homo sapiens relaxin 3 (RLN3), mRNA [NM_080864]	117579
A_24_P374863	2.1	2.8	2.1	1.3	RLTPR	Homo sapiens mRNA for FLJ00330 protein. [AK090421]	146206

A_23_P353717	-9.8	1.1	1.3	-2.5	RMI2	Homo sapiens RMI2, RecQ mediated genome instability 2, homolog (S. cerevisiae) (RMI2), mRNA [NM_152308]	116028
A_24_P114339	4.7	7.0	4.6	6.6	RMND5A	Homo sapiens required for meiotic nuclear division 5 homolog A (S. cerevisiae) (RMND5A), mRNA [NM_022780]	64795
A_23_P401580	2.6	2.3	1.6	1.5	RMND5B	Homo sapiens required for meiotic nuclear division 5 homolog B (S. cerevisiae) (RMND5B), mRNA [NM_022762]	64777

A_23_P257372	1.0	-1.0	-5.1	1.0	RMND5B	required for meiotic nuclear division 5 homolog B (S. cerevisiae) [Source:HGNC Symbol;Acc:26181] [ENST00000507575]	64777
A_23_P48596	-5.0	-9.4	-2.6	-5.6	RNASE1	Homo sapiens ribonuclease, RNase A family, 1 (pancreatic) (RNASE1), transcript variant 3, mRNA [NM_198232]	6035
A_24_P403959	-1.7	-1.1	-2.2	-1.7	RNASE1	Homo sapiens ribonuclease, RNase A family, 1 (pancreatic) (RNASE1), transcript variant 3, mRNA [NM_198232]	6035

A_32_P1712	1.1	-1.6	-2.1	1.0	RNASE2	Homo sapiens ribonuclease, RNase A family, 2 (liver, eosinophil-derived neurotoxin) (RNASE2), mRNA [NM_002934]	6036
A_23_P151637	1.6	-1.5	-2.5	1.4	RNASE2	Homo sapiens ribonuclease, RNase A family, 2 (liver, eosinophil-derived neurotoxin) (RNASE2), mRNA [NM_002934]	6036
A_23_P163025	3.2	3.1	2.8	2.1	RNASE3	Homo sapiens ribonuclease, RNase A family, 3 (RNASE3), mRNA [NM_002935]	6037

A_23_P205531	-1.1	-1.1	-2.2	-1.3	RNASE4	Homo sapiens ribonuclease, RNase A family, 4 (RNASE4), transcript variant 2, mRNA [NM_002937]	6038
A_23_P106080	-12.3	-2.2	-1.1	1.1	RNASE7	Homo sapiens ribonuclease, RNase A family, 7 (RNASE7), mRNA [NM_032572]	84659
A_23_P27167	1.7	1.4	-1.1	2.0	RNASEH1	Homo sapiens ribonuclease H1 (RNASEH1), mRNA [NM_002936]	246243
A_23_P164826	3.1	1.1	-1.2	-1.1	RNASEH2A	Homo sapiens ribonuclease H2, subunit A (RNASEH2A), mRNA [NM_006397]	10535

A_23_P140088	1.1	1.0	-2.6	-1.0	RNASEH2B	Homo sapiens ribonuclease H2, subunit B (RNASEH2B), transcript variant 1, mRNA [NM_024570]	79621
A_23_P434807	-1.2	-1.3	4.1	4.5	RND2	Homo sapiens Rho family GTPase 2 (RND2), mRNA [NM_005440]	8153
A_23_P56709	1.4	2.7	3.2	1.5	RNF103	Homo sapiens ring finger protein 103 (RNF103), transcript variant 1, mRNA [NM_005667]	7844
A_23_P107116	-1.1	-2.3	-1.2	-1.2	RNF112	Homo sapiens ring finger protein 112 (RNF112), mRNA [NM_007148]	7732

A_23_P258071	2.5	1.3	1.9	1.6	RNF113A	Homo sapiens ring finger protein 113A (RNF113A), mRNA [NM_006978]	7737
A_24_P560909	-8.5	1.0	1.0	1.1	RNF115	ring finger protein 115 [Source:HGNC Symbol;Acc:18154] [ENST00000369291]	27246
A_23_P75973	-7.5	-2.0	-5.9	-1.4	RNF121	Homo sapiens ring finger protein 121 (RNF121), transcript variant 1, mRNA [NM_018320]	55298
A_23_P250054	1.8	1.6	1.1	2.2	RNF123	Homo sapiens ring finger protein 123 (RNF123), mRNA [NM_022064]	63891
A_23_P101141	-1.0	-1.1	-2.9	-1.1	RNF125	Homo sapiens ring finger protein 125 (RNF125), mRNA [NM_017831]	54941

A_23_P314086	2.3	-1.5	-1.2	-2.5	RNF126	Homo sapiens ring finger protein 126 (RNF126), mRNA [NM_194460]	55658
A_23_P42963	-4.9	1.1	-1.0	-1.2	RNF133	Homo sapiens ring finger protein 133 (RNF133), mRNA [NM_139175]	168433
A_23_P252283	3.6	3.5	1.5	1.6	RNF135	Homo sapiens ring finger protein 135 (RNF135), transcript variant 1, mRNA [NM_032322]	84282
A_24_P202512	-4.5	-2.7	-1.9	-1.8	RNF138	Homo sapiens ring finger protein 138 (RNF138), transcript variant 1, mRNA [NM_016271]	51444

A_23_P89755	1.0	2.6	-1.1	-1.1	RNF138	Homo sapiens ring finger protein 138 (RNF138), transcript variant 1, mRNA [NM_016271]	51444
A_24_P69150	-9.5	1.0	1.0	1.1	RNF14	Homo sapiens ring finger protein 14 (RNF14), transcript variant 1, mRNA [NM_004290]	9604
A_24_P132039	1.9	1.8	2.2	-2.5	RNF14	Homo sapiens ring finger protein 14 (RNF14), transcript variant 1, mRNA [NM_004290]	9604
A_24_P372625	-1.9	-1.9	-3.7	-1.9	RNF141	Homo sapiens ring finger protein 141 (RNF141), mRNA [NM_016422]	50862

A_24_P37264	-4.7	1.1	-1.0	-1.0	RNF144A	Homo sapiens ring finger protein 144A (RNF144A), mRNA [NM_014746]	9781
A_23_P167559	-2.3	-1.5	-3.4	-10.6	RNF145	Homo sapiens ring finger protein 145 (RNF145), transcript variant 2, mRNA [NM_144726]	153830
A_24_P170234	1.1	1.1	-3.9	1.0	RNF148	Homo sapiens ring finger protein 148 (RNF148), mRNA [NM_198085]	378925
A_32_P4199	-1.2	-1.2	-7.7	-1.2	RNF152	Homo sapiens ring finger protein 152 (RNF152), mRNA [NM_173557]	220441
A_32_P57810	-1.0	1.6	-1.1	-2.1	RNF157	Homo sapiens ring finger protein 157 (RNF157), mRNA [NM_052916]	114804

A_23_P416242	1.2	-2.3	-2.2	1.2	RNF166	Homo sapiens ring finger protein 166 (RNF166), transcript variant 1, mRNA [NM_178841]	115992
A_32_P195291	-1.5	-19.2	-1.4	-1.5	RNF168	Homo sapiens ring finger protein 168 (RNF168), mRNA [NM_152617]	165918
A_24_P99066	2.0	2.3	1.2	2.1	RNF17	Homo sapiens ring finger protein 17 (RNF17), transcript variant 1, mRNA [NM_031277]	56163
A_23_P99373	-1.0	2.4	1.0	-1.0	RNF17	Homo sapiens ring finger protein 17 (RNF17), transcript variant 1, mRNA [NM_031277]	56163

A_23_P146209	3.6	2.1	1.6	1.9	RNF170	Homo sapiens ring finger protein 170 (RNF170), transcript variant 2, mRNA [NM_030954]	81790
A_23_P340263	1.1	1.0	1.0	-3.1	RNF175	Homo sapiens ring finger protein 175 (RNF175), mRNA [NM_173662]	285533
A_23_P103062	-1.0	1.2	1.0	-3.9	RNF185	Homo sapiens ring finger protein 185 (RNF185), transcript variant 1, mRNA [NM_152267]	91445
A_32_P15421	-1.3	1.4	-1.8	-4.4	RNF185	Homo sapiens ring finger protein 185 (RNF185), transcript variant 1, mRNA [NM_152267]	91445

A_24_P411899	-1.1	2.4	1.5	-1.2	RNF19A	Homo sapiens ring finger protein 19A (RNF19A), transcript variant 1, mRNA [NM_183419]	25897
A_23_P215897	1.7	2.9	1.7	1.0	RNF19A	Homo sapiens ring finger protein 19A (RNF19A), transcript variant 1, mRNA [NM_183419]	25897
A_23_P336000	-8.6	-1.1	-2.5	-2.5	RNF207	Homo sapiens ring finger protein 207 (RNF207), mRNA [NM_207396]	388591
A_23_P42724	-1.0	-2.9	-1.2	-1.1	RNF216	Homo sapiens ring finger protein 216 (RNF216), transcript variant 1, mRNA [NM_207111]	54476

A_24_P235131	-2.8	1.7	1.3	1.0	RNF216	Homo sapiens ring finger protein 216 (RNF216), transcript variant 1, mRNA [NM_207111]	54476
A_24_P469641	-1.5	1.0	-2.4	-2.4	RNF216	Homo sapiens ring finger protein 216 (RNF216), transcript variant 1, mRNA [NM_207111]	54476
A_24_P643041	4.9	4.9	5.1	4.8	RNF216P1	Homo sapiens ring finger protein 216 pseudogene 1 (RNF216P1), transcript variant 3, non-coding RNA [NR_023385]	441191
A_24_P333019	2.6	2.0	-1.1	-1.5	RNF24	Homo sapiens ring finger protein 24 (RNF24), transcript variant 1, mRNA [NM_007219]	11237

A_23_P39656	-3.4	1.1	1.7	-1.1	RNF25	Homo sapiens ring finger protein 25 (RNF25), mRNA [NM_022453]	64320
A_23_P64630	-2.5	-1.3	-3.3	-2.2	RNF26	Homo sapiens ring finger protein 26 (RNF26), mRNA [NM_032015]	79102
A_23_P19816	-1.9	-1.0	-1.1	-3.5	RNF32	Homo sapiens ring finger protein 32 (RNF32), transcript variant 3, mRNA [NM_030936]	140545
A_24_P4334	2.4	2.3	-1.1	-1.1	RNF38	Homo sapiens ring finger protein 38 (RNF38), transcript variant 2, mRNA [NM_194328]	152006
A_23_P255851	2.0	1.0	-2.1	-1.0	RNF38	Homo sapiens ring finger protein 38 (RNF38), transcript variant 2, mRNA [NM_194328]	152006

A_23_P167308	1.2	1.2	-3.1	-1.0	RNF4	Homo sapiens ring finger protein 4 (RNF4), transcript variant 2, mRNA [NM_002938]	6047
A_24_P630875	-6.3	-1.3	-1.3	-1.2	RNF41	Homo sapiens ring finger protein 41 (RNF41), transcript variant 2, mRNA [NM_194358]	10193
A_24_P330886	3.5	1.5	1.9	3.0	RNFT1	Homo sapiens ring finger protein, transmembrane 1 (RNFT1), mRNA [NM_016125]	51136
A_24_P13390	1.2	3.5	1.5	1.5	RNFT2	Homo sapiens ring finger protein, transmembrane 2 (RNFT2), transcript variant 2, mRNA [NM_032814]	84900

A_23_P349203	-1.9	-2.0	-2.1	-2.5	RNH1	ribonuclease/angiogenin inhibitor 1 [Source:HGNC Symbol;Acc:10074] [ENST00000533592]	6050
A_24_P415601	-1.3	-3.6	-1.5	-2.4	RNH1	Homo sapiens ribonuclease/angiogenin inhibitor 1 (RNH1), transcript variant 1, mRNA [NM_002939]	6050
A_23_P202672	1.1	-5.1	1.0	1.0	RNH1	Homo sapiens ribonuclease/angiogenin inhibitor 1 (RNH1), transcript variant 1, mRNA [NM_002939]	6050
A_23_P202501	1.0	-6.1	-1.0	-1.1	RNLS	Homo sapiens renalase, FAD-dependent amine oxidase (RNLS), transcript variant 1, mRNA [NM_001031709]	55328

A_24_P48408	-3.4	1.6	-1.3	1.0	RNMT	Homo sapiens RNA (guanine-7-) methyltransferase (RNMT), mRNA [NM_003799]	8731
A_24_P344890	1.1	2.2	2.1	2.4	RNPC3	RNA-binding region (RNP1, RRM) containing 3 [Source:HGNC Symbol;Acc:18666] [ENST00000533099]	55599
A_23_P254967	-3.4	-1.0	-1.0	-1.1	RNPC3	RNA-binding region (RNP1, RRM) containing 3 [Source:HGNC Symbol;Acc:18666] [ENST00000533099]	55599

A_23_P254970	1.9	2.3	1.6	1.6	RNPC3	RNA-binding region (RNP1, RRM) containing 3 [Source:HGNC Symbol;Acc:18666] [ENST00000533099]	55599
A_24_P68649	1.9	-2.3	-1.6	1.5	RNPEP	Homo sapiens arginyl aminopeptidase (aminopeptidase B) (RNPEP), mRNA [NM_020216]	6051
A_23_P17030	-1.9	-4.5	-1.5	1.8	RNPEPL1	Homo sapiens arginyl aminopeptidase (aminopeptidase B)-like 1 (RNPEPL1), mRNA [NM_018226]	57140

A_24_P725630	-1.1	-2.3	1.1	1.0	RNPS1	Homo sapiens RNA binding protein S1, serine-rich domain (RNPS1), transcript variant 1, mRNA [NM_006711]	10921
A_23_P80503	-3.8	1.0	-1.4	1.0	ROBO1	Homo sapiens roundabout, axon guidance receptor, homolog 1 (Drosophila) (ROBO1), transcript variant 2, mRNA [NM_133631]	6091
A_24_P376150	-1.1	-1.0	-1.1	-2.4	ROBO2	Homo sapiens cDNA FLJ90299 fis, clone NT2RP2000514, highly similar to Homo sapiens roundabout 2 (robo2) mRNA. [AK074780]	6092

A_24_P16326	-1.2	-2.2	-1.3	-3.0	ROBO2	Homo sapiens roundabout, axon guidance receptor, homolog 2 (Drosophila) (ROBO2), transcript variant 2, mRNA [NM_002942]	6092
A_23_P87082	3.5	4.7	1.2	1.3	ROBO3	Homo sapiens roundabout, axon guidance receptor, homolog 3 (Drosophila) (ROBO3), mRNA [NM_022370]	64221
A_23_P344421	1.3	-1.2	-2.1	-1.1	ROBO4	Homo sapiens roundabout homolog 4, magic roundabout (Drosophila) (ROBO4), mRNA [NM_019055]	54538

A_24_P538403	-3.1	1.0	1.1	1.1	ROCK1	Homo sapiens Rho-associated, coiled-coil containing protein kinase 1 (ROCK1), mRNA [NM_005406]	6093
A_23_P130429	2.3	2.0	-1.1	1.1	ROCK1	Homo sapiens Rho-associated, coiled-coil containing protein kinase 1 (ROCK1), mRNA [NM_005406]	6093
A_32_P203320	-1.0	-1.1	-1.2	-2.0	ROCK1P1	Homo sapiens Rho-associated, coiled-coil containing protein kinase 1 pseudogene 1 (ROCK1P1), non-coding RNA [NR_033770]	727758

A_24_P328333	-1.0	-1.1	-6.7	-7.0	ROCK2	Homo sapiens Rho-associated, coiled-coil containing protein kinase 2 (ROCK2), mRNA [NM_004850]	9475
A_23_P100499	1.3	-1.6	-1.2	-2.6	ROGDI	Homo sapiens rogdi homolog (Drosophila) (ROGDI), mRNA [NM_024589]	79641
A_32_P184464	-1.0	-69.5	-1.1	-1.1	ROPN1	Homo sapiens rhophilin associated tail protein 1 (ROPN1), mRNA [NM_017578]	54763
A_24_P417407	-1.2	3.8	1.2	1.9	ROPN1B	Homo sapiens rhophilin associated tail protein 1B (ROPN1B), mRNA [NM_001012337]	152015

A_24_P410605	2.1	2.0	1.4	1.6	ROR1	Homo sapiens receptor tyrosine kinase-like orphan receptor 1 (ROR1), transcript variant 2, mRNA [NM_001083592]	4919
A_23_P158318	-2.1	1.3	2.1	1.6	ROR2	Homo sapiens receptor tyrosine kinase-like orphan receptor 2 (ROR2), mRNA [NM_004560]	4920
A_23_P26124	2.2	2.1	2.1	2.2	RORA	Homo sapiens RAR-related orphan receptor A (RORA), transcript variant 2, mRNA [NM_134260]	6095
A_23_P71699	1.7	-5.0	-1.2	1.8	RORB	Homo sapiens RAR-related orphan receptor B (RORB), mRNA [NM_006914]	6096

A_23_P372910	2.9	5.1	1.1	3.5	RORC	Homo sapiens RAR-related orphan receptor C (RORC), transcript variant 1, mRNA [NM_005060]	6097
A_23_P70278	1.5	1.4	1.4	-5.0	ROS1	Homo sapiens c-ros oncogene 1 , receptor tyrosine kinase (ROS1), mRNA [NM_002944]	6098
A_23_P71368	-1.1	-4.3	-2.7	-1.1	RP1	Homo sapiens retinitis pigmentosa 1 (autosomal dominant) (RP1), mRNA [NM_006269]	6101
A_24_P134568	1.1	1.4	-2.7	1.1	RP2	Homo sapiens retinitis pigmentosa 2 (X-linked recessive) (RP2), mRNA [NM_006915]	6102

A_23_P255215	-1.2	-1.2	-4.3	-1.5	RP9	Homo sapiens retinitis pigmentosa 9 (autosomal dominant) (RP9), mRNA [NM_203288]	6100
A_24_P257759	1.3	1.3	1.3	2.2	RP9	Homo sapiens retinitis pigmentosa 9 (autosomal dominant) (RP9), mRNA [NM_203288]	6100
A_24_P75019	-2.0	-2.0	-2.1	-2.0	RP9P	Homo sapiens retinitis pigmentosa 9 pseudogene (RP9P), non-coding RNA [NR_003500]	441212
A_23_P66766	2.6	1.9	1.8	1.0	RPAIN	Homo sapiens RPA interacting protein (RPAIN), transcript variant 4, mRNA [NM_001160246]	84268

A_23_P3453	8.5	4.2	9.9	5.1	RPAP1	Homo sapiens RNA polymerase II associated protein 1 (RPAP1), mRNA [NM_015540]	26015
A_23_P23542	1.2	1.1	-4.5	1.1	RPAP2	Homo sapiens RNA polymerase II associated protein 2 (RPAP2), mRNA [NM_024813]	79871
A_23_P165301	2.3	1.3	2.3	1.9	RPE	Homo sapiens ribulose-5-phosphate-3-epimerase (RPE), transcript variant 2, mRNA [NM_006916]	6120
A_23_P34473	1.0	-1.0	-5.0	-1.2	RPE65	Homo sapiens retinal pigment epithelium-specific protein 65kDa (RPE65), mRNA [NM_000329]	6121

A_23_P359616	3.5	3.5	3.5	3.5	RPF1	Homo sapiens ribosome productio n factor 1 homolog (S. cerevisiae) (RPF1), mRNA [NM_0250 65]	80135
A_23_P32426	-1.3	3.0	-1.3	-1.7	RPF1	Homo sapiens ribosome productio n factor 1 homolog (S. cerevisiae) (RPF1), mRNA [NM_0250 65]	80135
A_23_P19210	-1.0	-17.5	-1.0	-1.0	RPF2	Homo sapiens ribosome productio n factor 2 homolog (S. cerevisiae) (RPF2), mRNA [NM_0321 94]	84154
A_23_P136964	1.5	2.1	-1.1	-1.4	RPGR	Homo sapiens retinitis pigmentos a GTPase regulator (RPGR), transcript variant A, mRNA [NM_0003 28]	6103

A_23_P76234	-2.5	-2.5	-6.4	-3.7	RPH3A	Homo sapiens rabphilin 3A homolog (mouse) (RPH3A), transcript variant 1, mRNA [NM_001143854]	22895
A_32_P108636	-1.3	-2.1	1.3	-2.1	RPL10	Homo sapiens ribosomal protein L10, mRNA (cDNA clone IMAGE:4499390), with apparent retained intron. [BC021806]	6134
A_23_P251593	-1.2	1.6	2.5	1.0	RPL10A	Homo sapiens ribosomal protein L10a (RPL10A), mRNA [NM_007104]	4736
A_23_P62605	3.1	4.3	2.4	2.6	RPL11	Homo sapiens ribosomal protein L11 (RPL11), transcript variant 1, mRNA [NM_000975]	6135

A_24_P375237	-4.1	-2.1	-3.1	-2.0	RPL12	Homo sapiens ribosomal protein L12 (RPL12), mRNA [NM_000976]	6136
A_32_P92295	1.0	-1.7	-1.4	-2.0	RPL13A	Homo sapiens ribosomal protein L13a (RPL13A), mRNA [NM_012423]	23521
A_24_P161197	-2.7	-1.2	-1.1	-5.8	RPL13AP17	Homo sapiens ribosomal protein L13a pseudogene 17 (RPL13AP17), non-coding RNA [NR_003680]	399670
A_24_P272873	4.0	6.1	5.0	6.1	RPL13AP3	Homo sapiens ribosomal protein L13a pseudogene 3 (RPL13AP3), non-coding RNA [NR_004844]	645683

A_23_P18294	1.6	1.6	-3.4	-2.6	RPL14	Homo sapiens ribosomal protein L14 (RPL14), transcript variant 1, mRNA [NM_001034996]	9045
A_23_P253200	-1.0	-2.4	-1.0	-1.6	RPL15	Homo sapiens ribosomal protein L15 (RPL15), mRNA [NM_002948]	6138
A_32_P158746	-1.0	2.6	2.9	2.6	RPL17	Homo sapiens ribosomal protein L17 (RPL17), transcript variant 1, mRNA [NM_000985]	6139
A_24_P356721	1.5	1.0	-1.6	4.5	RPL18	Homo sapiens ribosomal protein L18 (RPL18), mRNA [NM_000979]	6141

A_23_P77779	1.5	2.2	-1.2	1.2	RPL19	Homo sapiens ribosomal protein L19 (RPL19), mRNA [NM_000981]	6143
A_32_P16747	-2.5	-2.4	-3.7	-1.6	RPL21	ribosomal protein L21 [Source:HGNC Symbol;Acc:10313] [ENST00000483765]	6144
A_24_P280901	12.9	22.2	7.1	10.6	RPL21	Homo sapiens ribosomal protein L21 (RPL21), mRNA [NM_000982]	6144
A_24_P830667	2.0	2.6	2.4	1.9	RPL21	Homo sapiens ribosomal protein L21 (RPL21), mRNA [NM_000982]	6144
A_32_P57237	-3.3	1.6	1.5	1.6	RPL21	Homo sapiens ribosomal protein L21 (RPL21), mRNA [NM_000982]	6144

A_24_P41629	1.9	-1.2	-1.2	2.1	RPL21P44	Homo sapiens ribosomal protein L21 pseudogene 44 (RPL21P44), non-coding RNA [NR_027153]	402176
A_24_P849801	1.0	1.0	-1.0	-2.3	RPL22	Homo sapiens ribosomal protein L22 (RPL22), mRNA [NM_000983]	6146
A_24_P67699	1.5	2.0	1.4	2.0	RPL23A	Homo sapiens ribosomal protein L23a (RPL23A), mRNA [NM_000984]	6147
A_23_P108932	-1.0	-3.3	-1.0	-1.0	RPL23AP32	Homo sapiens ribosomal protein L23a pseudogene 32 (RPL23AP32), non-coding RNA [NR_002229]	56969

A_24_P379649	1.3	1.3	-2.4	1.0	RPL23AP32	Homo sapiens ribosomal protein L23a pseudogene 32 (RPL23AP32), non-coding RNA [NR_002229]	56969
A_23_P33045	-3.5	-2.3	-1.1	-4.5	RPL26	Homo sapiens ribosomal protein L26 (RPL26), mRNA [NM_000987]	6154
A_32_P205553	5.5	-1.2	-1.8	-1.3	RPL26L1	Homo sapiens ribosomal protein L26-like 1 (RPL26L1), mRNA [NM_016093]	51121
A_24_P916251	1.5	1.5	-3.9	1.5	RPL28	Homo sapiens mRNA for ribosomal protein L28 variant protein. [AB209627]	6158

A_23_P257609	2.0	-2.1	-3.7	-1.8	RPL29	Homo sapiens ribosomal protein L29 (RPL29), mRNA [NM_000992]	6159
A_32_P162004	-1.0	1.1	-1.3	-4.5	RPL29	Homo sapiens ribosomal protein L29 (RPL29), mRNA [NM_000992]	6159
A_24_P203909	1.9	1.7	-1.3	-2.3	RPL34	Homo sapiens ribosomal protein L34 (RPL34), transcript variant 2, mRNA [NM_033625]	6164
A_32_P220127	2.4	-1.4	-2.6	-1.9	RPL34	Homo sapiens ribosomal protein L34 (RPL34), transcript variant 2, mRNA [NM_033625]	6164

A_23_P112429	2.9	1.5	2.2	1.7	RPL35	Homo sapiens ribosomal protein L35 (RPL35), mRNA [NM_007209]	11224
A_24_P403403	1.9	2.2	2.8	1.3	RPL35	Homo sapiens ribosomal protein L35 (RPL35), mRNA [NM_007209]	11224
A_23_P153383	2.1	1.2	1.9	-1.1	RPL36	Homo sapiens ribosomal protein L36 (RPL36), transcript variant 2, mRNA [NM_015414]	25873
A_24_P417209	1.0	-5.9	-1.0	-1.0	RPL36A	Homo sapiens ribosomal protein L36a (RPL36A), transcript variant 1, mRNA [NM_021029]	6173

A_32_P136319	-2.3	-2.4	-5.3	-2.5	RPL36A	Homo sapiens ribosomal protein L36a (RPL36A), transcript variant 1, mRNA [NM_021029]	6173
A_24_P340491	-1.1	-1.2	-1.2	-7.5	RPL36AP40	Homo sapiens ribosomal protein L36a pseudogene, mRNA (cDNA clone IMAGE:6259908). [BC058160]	554234
A_24_P20777	2.3	2.2	1.9	1.6	RPL37	Homo sapiens ribosomal protein L37 (RPL37), mRNA [NM_000997]	6167
A_32_P235128	1.6	2.3	-1.7	-2.8	RPL37	Homo sapiens ribosomal protein L37 (RPL37), mRNA [NM_000997]	6167

A_23_P142724	-1.5	-1.2	-2.3	-2.2	RPL37A	Homo sapiens ribosomal protein L37a (RPL37A), mRNA [NM_000998]	6168
A_23_P34018	2.6	1.0	-1.1	-1.1	RPL39	Homo sapiens ribosomal protein L39 (RPL39), mRNA [NM_001000]	6170
A_23_P29594	-1.0	-2.5	-1.0	-1.0	RPL39L	Homo sapiens ribosomal protein L39-like (RPL39L), mRNA [NM_052969]	116832
A_23_P118095	-4.0	-1.2	-2.3	-1.3	RPL3L	Homo sapiens ribosomal protein L3-like (RPL3L), mRNA [NM_005061]	6123
A_23_P69431	2.0	1.6	-1.2	2.0	RPL4	Homo sapiens ribosomal protein L4 (RPL4), mRNA [NM_000968]	6124

A_23_P12140	5.0	-5.6	4.0	4.1	RPL5	Homo sapiens ribosomal protein L5 (RPL5), mRNA [NM_000969]	6125
A_23_P151321	8.4	2.3	6.4	4.6	RPL6	Homo sapiens ribosomal protein L6 (RPL6), transcript variant 1, mRNA [NM_001024662]	6128
A_24_P375813	2.6	-2.5	-3.3	-1.0	RPL7A	Homo sapiens ribosomal protein L7a (RPL7A), mRNA [NM_000972]	6130
A_24_P63262	-1.1	-2.1	-2.1	-2.0	RPN1	Homo sapiens ribophorin I (RPN1), mRNA [NM_002950]	6184
A_23_P254340	-1.2	-1.0	-3.3	-1.0	RPN1	Homo sapiens ribophorin I (RPN1), mRNA [NM_002950]	6184

A_23_P132809	1.0	-1.0	-1.0	-4.3	RPN1	Homo sapiens ribophorin I (RPN1), mRNA [NM_002950]	6184
A_23_P108994	-1.6	-2.2	-1.2	1.1	RPN2	Homo sapiens ribophorin II (RPN2), transcript variant 1, mRNA [NM_002951]	6185
A_23_P214594	-1.2	-2.7	-3.8	-2.8	RPP21	Homo sapiens ribonuclease P/MRP 21kDa subunit (RPP21), transcript variant 2, mRNA [NM_024839]	79897
A_23_P3424	1.2	1.8	2.3	1.0	RPP25	Homo sapiens ribonuclease P/MRP 25kDa subunit (RPP25), mRNA [NM_017793]	54913

A_23_P150080	9.8	12.6	19.8	21.4	RPP38	Homo sapiens ribonuclease P/MRP 38kDa subunit (RPP38), transcript variant 1, mRNA [NM_183005]	10557
A_23_P111297	-1.7	1.3	2.3	-1.9	RPP40	Homo sapiens ribonuclease P/MRP 40kDa subunit (RPP40), mRNA [NM_006638]	10799
A_24_P382253	-2.1	-5.0	-1.0	-1.0	RPRD1A	Homo sapiens regulation of nuclear pre-mRNA domain containing 1A (RPRD1A), mRNA [NM_018170]	55197
A_23_P91207	-2.1	1.1	1.0	1.0	RPRD1B	Homo sapiens regulation of nuclear pre-mRNA domain containing 1B (RPRD1B), mRNA [NM_021215]	58490

A_23_P60657	-1.2	2.1	1.6	4.5	RPRD2	Homo sapiens regulation of nuclear pre-mRNA domain containing 2 (RPRD2), mRNA [NM_015203]	23248
A_23_P356484	-1.0	-3.1	-3.0	1.4	RPS10	Homo sapiens ribosomal protein S10 (RPS10), transcript variant 2, mRNA [NM_001014]	6204
A_23_P82128	4.1	4.1	3.9	2.8	RPS12	Homo sapiens ribosomal protein S12 (RPS12), mRNA [NM_001016]	6206
A_32_P86028	1.5	-8.9	-1.2	-1.1	RPS13	Homo sapiens ribosomal protein S13 (RPS13), mRNA [NM_001017]	6207

A_24_P37939	-1.3	-2.0	1.1	-2.4	RPS14	ribosomal protein S14 [Source:HGNC Symbol;Acc:10387] [ENST00000401695]	6208
A_32_P14744	1.1	-3.5	-1.0	1.0	RPS15A	Homo sapiens ribosomal protein S15a (RPS15A), transcript variant 2, mRNA [NM_001019]	6210
A_24_P740705	-1.1	-2.6	1.9	3.0	RPS19BP1	Homo sapiens ribosomal protein S19 binding protein 1 (RPS19BP1), mRNA [NM_194326]	91582
A_32_P14544	7.8	-2.1	7.4	9.9	RPS2	Homo sapiens ribosomal protein S2 (RPS2), mRNA [NM_002952]	6187

A_24_P919460	1.6	1.3	-1.0	2.1	RPS2	ribosomal protein S2 [Source:HGNC Symbol;Acc:10404] [ENST00000526908]	6187
A_32_P84605	-1.5	-2.0	-1.5	-1.5	RPS23	Homo sapiens ribosomal protein S23 (RPS23), mRNA [NM_001025]	6228
A_24_P638294	-1.4	1.1	-1.8	-2.1	RPS26	Homo sapiens ribosomal protein S26 (RPS26), mRNA [NM_001029]	6231
A_23_P79732	2.0	1.2	1.2	1.0	RPS27A	Homo sapiens ribosomal protein S27a (RPS27A), transcript variant 1, mRNA [NM_002954]	6233
A_23_P142475	-1.2	1.5	1.0	-4.3	RPS28	Homo sapiens ribosomal protein S28 (RPS28), mRNA [NM_001031]	6234

A_23_P157196	1.4	1.0	-2.2	1.4	RPS2P32	Homo sapiens ribosomal protein S2 pseudogene 32 (RPS2P32), non-coding RNA [NR_026676]	256355
A_24_P218765	2.3	2.3	2.2	1.2	RPS3	ribosomal protein S3 [Source:HGNC Symbol;Acc:10420] [ENST00000531188]	6188
A_32_P58074	2.1	-1.3	-1.2	1.7	RPS3A	Homo sapiens ribosomal protein S3A (RPS3A), mRNA [NM_001006]	6189
A_32_P196483	-3.4	1.2	-3.9	-1.7	RPS3A	Homo sapiens ribosomal protein S3A (RPS3A), mRNA [NM_001006]	6189
A_23_P123563	2.0	-4.8	-3.0	-1.5	RPS6	Homo sapiens ribosomal protein S6 (RPS6), mRNA [NM_001010]	6194

A_23_P335920	-1.6	-15.7	-1.3	-1.1	RPS6KA2	Homo sapiens ribosomal protein S6 kinase, 90kDa, polypeptide 2 (RPS6KA2), transcript variant 1, mRNA [NM_021135]	6196
A_23_P141329	2.4	1.2	2.3	1.7	RPS6KB1	Homo sapiens ribosomal protein S6 kinase, 70kDa, polypeptide 1 (RPS6KB1), mRNA [NM_003161]	6198
A_23_P24318	1.3	-3.5	1.4	1.5	RPS6KB2	Homo sapiens ribosomal protein S6 kinase, 70kDa, polypeptide 2 (RPS6KB2), mRNA [NM_003952]	6199

A_24_P219108	-3.0	1.0	2.3	1.5	RPS6KL1	Homo sapiens ribosomal protein S6 kinase-like 1 (RPS6KL1), mRNA [NM_031464]	83694
A_32_P20367	1.6	1.4	2.1	1.4	RPS7	Homo sapiens ribosomal protein S7 (RPS7), mRNA [NM_001011]	6201
A_24_P169378	1.5	-1.4	-2.5	-1.2	RPS7	Homo sapiens ribosomal protein S7 (RPS7), mRNA [NM_001011]	6201
A_32_P22078	-1.7	-2.4	-1.7	-1.8	RPS9	Homo sapiens ribosomal protein S9 (RPS9), mRNA [NM_001013]	6203
A_32_P223456	1.1	-2.2	2.0	1.1	RPS9	Homo sapiens ribosomal protein S9, mRNA (cDNA clone IMAGE:3856008). [BC020462]	6203

A_32_P219657	1.9	-7.6	2.1	1.5	RPSA	Homo sapiens ribosomal protein SA (RPSA), transcript variant 1, mRNA [NM_002295]	3921
A_24_P349743	-1.0	-1.3	-3.6	-1.2	RPSA	Homo sapiens ribosomal protein SA (RPSA), transcript variant 1, mRNA [NM_002295]	3921
A_23_P255916	-1.6	-2.4	-1.2	-2.4	RPUSD3	Homo sapiens RNA pseudouridylate synthase domain containing 3 (RPUSD3), transcript variant 1, mRNA [NM_173659]	285367

A_24_P318073	-7.1	-1.3	-1.7	-1.4	RPUSD4	Homo sapiens RNA pseudouridylate synthase domain containing 4 (RPUSD4), transcript variant 1, mRNA [NM_032795]	84881
A_23_P318039	2.1	1.6	1.8	2.7	RQCD1	Homo sapiens RCD1 required for cell differentiation1 homolog (S. pombe) (RQCD1), mRNA [NM_005444]	9125
A_24_P262127	1.7	-2.7	1.0	-1.7	RRAD	Homo sapiens Ras-related associated with diabetes (RRAD), transcript variant 2, mRNA [NM_004165]	6236

A_23_P97623	-1.8	-2.9	-1.8	1.2	RRAGC	Homo sapiens Ras-related GTP binding C (RRAGC), mRNA [NM_022157]	64121
A_23_P39076	2.2	2.3	-1.0	1.3	RRAS	Homo sapiens related RAS viral (r-ras) oncogene homolog (RRAS), mRNA [NM_006270]	6237
A_23_P214866	-1.6	-1.6	-2.5	-1.5	RREB1	Homo sapiens ras responsive element binding protein 1 (RREB1), transcript variant 1, mRNA [NM_001003699]	6239
A_24_P234196	-1.0	-6.4	-1.0	1.0	RRM2	Homo sapiens ribonucleotide reductase M2 (RRM2), transcript variant 2, mRNA [NM_001034]	6241

A_24_P590560	1.1	1.0	-1.1	-2.1	RRN3	Homo sapiens RRN3 RNA polymerase I transcription factor homolog (S. cerevisiae) (RRN3), mRNA [NM_018427]	54700
A_24_P296347	-1.0	-3.0	1.3	-1.0	RRN3	Homo sapiens RRN3 RNA polymerase I transcription factor homolog (S. cerevisiae) (RRN3), mRNA [NM_018427]	54700
A_24_P475864	-1.2	4.6	3.7	-1.0	RRN3P2	Homo sapiens RNA polymerase I transcription factor homolog (S. cerevisiae) pseudogene 2 (RRN3P2), non-coding RNA [NR_003369]	653390

A_32_P138557	-2.8	-3.6	-3.0	-3.9	RRN3P3	Homo sapiens RNA polymerase I transcription factor homolog (S. cerevisiae) pseudogene 3 (RRN3P3), non-coding RNA [NR_027460]	100131998
A_23_P23356	-2.3	2.2	2.0	2.0	RRP15	Homo sapiens ribosomal RNA processing 15 homolog (S. cerevisiae) (RRP15), mRNA [NM_016052]	51018
A_23_P308581	-1.1	-1.5	-1.3	-3.7	RRP36	Homo sapiens ribosomal RNA processing 36 homolog (S. cerevisiae) (RRP36), mRNA [NM_033112]	88745

A_23_P420038	9.9	9.6	9.3	9.0	RRP7A	Homo sapiens ribosomal RNA processing 7 homolog A (S. cerevisiae) (RRP7A), mRNA [NM_015703]	27341
A_24_P83158	1.2	-1.0	-5.0	1.5	RRP7B	Homo sapiens ribosomal RNA processing 7 homolog B (S. cerevisiae) (RRP7B), non-coding RNA [NR_002184]	91695
A_24_P242036	3.1	1.9	-1.3	1.4	RRP7B	Homo sapiens ribosomal RNA processing 7 homolog B (S. cerevisiae) (RRP7B), non-coding RNA [NR_002184]	91695

A_23_P356565	1.1	1.2	2.1	1.1	RRP8	Homo sapiens ribosomal RNA processing 8, methyltransferase, homolog (yeast) (RRP8), mRNA [NM_015324]	23378
A_23_P146187	2.6	2.5	1.4	2.5	RRS1	Homo sapiens RRS1 ribosome biogenesis regulator homolog (S. cerevisiae) (RRS1), mRNA [NM_015169]	23212
A_23_P354855	-1.3	4.1	-1.3	-1.3	RS1	Homo sapiens retinoschisin 1 (RS1), mRNA [NM_000330]	6247
A_24_P316965	-2.4	1.8	1.4	1.7	RSAD2	Homo sapiens radical S-adenosyl methionine domain containing 2 (RSAD2), mRNA [NM_080657]	91543

A_23_P137957	6.0	2.3	2.2	1.7	RSBN1	Homo sapiens round spermatid basic protein 1 (RSBN1), mRNA [NM_018364]	54665
A_24_P925292	2.1	1.1	1.9	1.0	RSBN1L	round spermatid basic protein 1-like [Source:HGNC Symbol;Acc:24765] [ENST00000445288]	222194
A_24_P246717	-1.9	-1.4	-4.2	-1.9	RSF1	Homo sapiens remodeling and spacing factor 1 (RSF1), mRNA [NM_016578]	51773
A_23_P126499	1.0	-2.4	-1.7	1.5	RSG1	Homo sapiens REM2 and RAB-like small GTPase 1 (RSG1), mRNA [NM_030907]	79363

A_24_P222022	1.6	-1.5	-1.4	-12.2	RSL1D1	Homo sapiens ribosomal L1 domain containing 1 (RSL1D1), mRNA [NM_015659]	26156
A_23_P54597	1.1	-2.3	-1.5	-1.1	RSL1D1	Homo sapiens ribosomal L1 domain containing 1 (RSL1D1), mRNA [NM_015659]	26156
A_23_P67411	-1.5	2.1	-2.8	1.1	RSPH6A	Homo sapiens radial spoke head 6 homolog A (Chlamydomonas) (RSPH6A), mRNA [NM_030785]	81492
A_24_P293698	-1.6	1.0	-2.4	-1.9	RSPO1	Homo sapiens R-spondin 1 (RSPO1), transcript variant 1, mRNA [NM_001038633]	284654

A_23_P22013	-1.4	-2.1	-1.5	1.3	RSPO1	Homo sapiens R-spondin 1 (RSPO1), transcript variant 1, mRNA [NM_001038633]	284654
A_23_P430930	-1.3	2.0	2.1	2.0	RSPO2	Homo sapiens R-spondin 2 (RSPO2), mRNA [NM_178565]	340419
A_23_P124313	-3.3	1.5	-3.9	1.8	RSRC1	arginine/serine-rich coiled-coil 1 [Source:HGNC Symbol;Acc:24152] [ENST00000464171]	51319
A_23_P149812	-18.0	-1.3	-1.3	-1.1	RSU1	Homo sapiens Ras suppressor protein 1 (RSU1), transcript variant 1, mRNA [NM_012425]	6251

A_24_P923061	-4.7	1.3	1.3	-1.3	RSU1	Homo sapiens Ras suppressor protein 1, mRNA (cDNA clone IMAGE:3949969), with apparent retained intron. [BC008384]	6251
A_23_P130653	2.8	-9.3	1.3	1.1	RTBDN	Homo sapiens retbindin (RTBDN), transcript variant 2, mRNA [NM_031429]	83546
A_32_P213624	-1.0	-1.1	-5.3	-1.4	RTCD1	Homo sapiens RNA terminal phosphate cyclase domain 1 (RTCD1), transcript variant 1, mRNA [NM_001130841]	8634

A_23_P29153	1.5	2.2	1.7	1.1	RTDR1	Homo sapiens rhabdoid tumor deletion region gene 1 (RTDR1), mRNA [NM_014433]	27156
A_23_P378526	-1.4	-1.0	-2.1	-3.5	RTEL1	Homo sapiens regulator of telomere elongation helicase 1 (RTEL1), transcript variant 1, mRNA [NM_016434]	51750
A_23_P77321	1.5	1.5	2.2	1.0	RTF1	Homo sapiens Rtf1, Paf1/RNA polymerase II complex component, homolog (S. cerevisiae) (RTF1), mRNA [NM_015138]	23168

A_24_P390403	1.7	1.2	2.4	-1.0	RTF1	Homo sapiens Rtf1, Paf1/RNA polymerase II complex component, homolog (S. cerevisiae) (RTF1), mRNA [NM_015138]	23168
A_23_P89902	-4.9	-1.1	-2.5	-3.1	RTN2	Homo sapiens reticulon 2 (RTN2), transcript variant 1, mRNA [NM_005619]	6253
A_23_P56933	2.9	2.9	2.9	2.9	RTN4	Homo sapiens reticulon 4 (RTN4), transcript variant 1, mRNA [NM_020532]	57142
A_23_P432056	-2.0	-1.6	-2.3	-2.1	RTN4RL1	Homo sapiens reticulon 4 receptor-like 1 (RTN4RL1), mRNA [NM_178568]	146760

A_32_P528311	-2.4	1.7	-1.3	2.2	RTN4RL2	Homo sapiens reticulon 4 receptor-like 2 (RTN4RL2), mRNA [NM_178570]	349667
A_32_P528967	-3.5	-1.6	-2.5	1.1	RTP1	Homo sapiens receptor (chemosensory) transporter protein 1 (RTP1), mRNA [NM_153708]	132112
A_24_P108301	1.6	1.4	-6.2	-1.2	RTTN	Homo sapiens rotatin (RTTN), mRNA [NM_173630]	25914
A_24_P380991	1.2	7.4	7.1	7.6	RUFY2	Homo sapiens RUN and FYVE domain containing 2 (RUFY2), transcript variant 2, mRNA [NM_001042417]	55680

A_24_P1167	27.4	20.0	10.8	18.0	RUFY2	Homo sapiens RUN and FYVE domain containing 2 (RUFY2), transcript variant 1, mRNA [NM_017987]	55680
A_23_P336083	1.8	1.8	-12.3	10.7	RUFY3	Homo sapiens RUN and FYVE domain containing 3 (RUFY3), transcript variant 2, mRNA [NM_014961]	22902
A_24_P362979	2.3	2.7	3.4	1.0	RUFY3	Homo sapiens RUN and FYVE domain containing 3 (RUFY3), transcript variant 1, mRNA [NM_001037442]	22902
A_24_P221575	1.0	-1.0	-1.7	-3.5	RUFY3	Homo sapiens RUN and FYVE domain containing 3 (RUFY3), transcript variant 1, mRNA [NM_001037442]	22902

A_24_P263595	1.1	1.6	-2.8	1.2	RUNDC2C	Homo sapiens RUN domain containing 2C (RUNDC2C), non-coding RNA [NR_002939]	440352
A_24_P91094	-3.5	1.0	-1.7	-1.0	RUNDC3A	Homo sapiens RUN domain containing 3A (RUNDC3A), transcript variant 2, mRNA [NM_006695]	10900
A_23_P427278	-5.3	-1.0	1.6	-8.0	RUNX1T1	runt-related transcription factor 1; translocated to, 1 (cyclin D-related) [Source:HGNC Symbol;Acc:1535] [ENST00000521751]	862

A_24_P2948	1.1	-1.0	5.7	1.6	RUSC1	Homo sapiens RUN and SH3 domain containing 1 (RUSC1), transcript variant 4, mRNA [NM_014328]	23623
A_23_P39110	-1.5	-1.3	-1.5	3.7	RUVBL2	Homo sapiens RuvB-like 2 (E. coli) (RUVBL2), mRNA [NM_006666]	10856
A_23_P255076	-3.6	1.0	-1.0	1.1	RWDD2A	Homo sapiens RWD domain containing 2A (RWDD2A), mRNA [NM_033411]	112611
A_23_P91487	1.8	-1.1	-1.3	2.1	RWDD2B	Homo sapiens RWD domain containing 2B (RWDD2B), mRNA [NM_016940]	10069

A_23_P394276	1.0	-2.3	1.1	1.0	RWDD4	Homo sapiens RWD domain containing 4 (RWDD4), mRNA [NM_152682]	201965
A_23_P124534	-1.0	-1.0	-4.3	-1.6	RXFP1	relaxin/insulin-like family peptide receptor 1 [Source:HGNC Symbol;Acc:19718] [ENST00000448688]	59350
A_23_P363365	2.2	2.8	2.3	2.2	RXFP3	Homo sapiens relaxin/insulin-like family peptide receptor 3 (RXFP3), mRNA [NM_016568]	51289
A_24_P930985	5.0	4.7	4.4	4.5	RXRA	retinoid X receptor, alpha [Source:HGNC Symbol;Acc:10477] [ENST00000484822]	6256

A_23_P29365	1.2	1.2	1.1	-5.4	RYBP	Homo sapiens RING1 and YY1 binding protein (RYBP), mRNA [NM_012234]	23429
A_24_P229377	1.1	1.6	2.2	-1.1	RYK	Homo sapiens RYK receptor-like tyrosine kinase (RYK), transcript variant 1, mRNA [NM_001005861]	6259
A_23_P94838	-1.0	-10.8	-1.0	-1.0	RYR3	Homo sapiens ryanodine receptor 3 (RYR3), transcript variant 1, mRNA [NM_001036]	6263
A_23_P383227	2.8	2.2	-13.5	1.2	S100A1	Homo sapiens S100 calcium binding protein A1 (S100A1), mRNA [NM_006271]	6271

A_23_P137984	1.1	-2.2	-1.2	1.0	S100A10	Homo sapiens S100 calcium binding protein A10 (S100A10), mRNA [NM_002966]	6281
A_23_P145863	1.4	2.5	1.4	1.3	S100A11	Homo sapiens S100 calcium binding protein A11 (S100A11), mRNA [NM_005620]	6282
A_23_P74001	1.7	1.7	-1.9	-2.2	S100A12	Homo sapiens S100 calcium binding protein A12 (S100A12), mRNA [NM_005621]	6283
A_23_P372874	-2.3	-1.5	1.2	-1.5	S100A13	Homo sapiens S100 calcium binding protein A13 (S100A13), transcript variant 1, mRNA [NM_001024210]	6284

A_23_P115467	69.2	67.4	150.4	113.5	S100A5	Homo sapiens S100 calcium binding protein A5 (S100A5), mRNA [NM_002962]	6276
A_23_P434809	2.6	1.8	-2.1	-1.2	S100A8	Homo sapiens S100 calcium binding protein A8 (S100A8), mRNA [NM_002964]	6279
A_23_P44996	-4.4	-2.1	-4.2	-2.0	S100G	Homo sapiens S100 calcium binding protein G (S100G), mRNA [NM_004057]	795
A_23_P58266	-2.5	-1.6	-1.4	-1.5	S100P	Homo sapiens S100 calcium binding protein P (S100P), mRNA [NM_005980]	6286

A_23_P338952	-2.0	1.1	1.0	1.1	S100PBP	Homo sapiens S100P binding protein (S100PBP), transcript variant 1, mRNA [NM_022753]	64766
A_32_P827528	-1.7	-2.7	-1.8	2.1	S1PR2	Homo sapiens sphingosine-1-phosphate receptor 2 (S1PR2), mRNA [NM_004230]	9294
A_23_P112820	2.2	1.7	-1.2	-2.2	S1PR3	Homo sapiens sphingosine-1-phosphate receptor 3 (S1PR3), mRNA [NM_005226]	1903
A_23_P119502	2.9	4.2	1.6	-2.0	S1PR4	Homo sapiens sphingosine-1-phosphate receptor 4 (S1PR4), mRNA [NM_003775]	8698

A_23_P98622	-7.7	-1.0	-1.1	-1.0	SAA2	Homo sapiens cDNA clone IMAGE:4716555, with apparent retained intron. [BC058008]	6289
A_23_P321349	-1.5	-1.3	-2.2	-1.8	SAAL1	Homo sapiens serum amyloid A-like 1 (SAAL1), mRNA [NM_138421]	113174
A_23_P308073	2.2	-1.0	-1.1	-1.1	SACS	Homo sapiens spastic ataxia of Charlevoix-Saguenay (sacsin) (SACS), mRNA [NM_014363]	26278
A_24_P111009	3.1	3.0	4.2	2.7	SAFB2	Homo sapiens scaffold attachment factor B2 (SAFB2), mRNA [NM_014649]	9667

A_23_P5853	-6.9	1.6	1.5	1.0	SAG	Homo sapiens S-antigen; retina and pineal gland (arrestin) (SAG), mRNA [NM_000541]	6295
A_23_P48585	2.2	1.0	-1.0	1.5	SALL2	Homo sapiens sal-like 2 (Drosophila) (SALL2), mRNA [NM_005407]	6297
A_23_P393670	-3.6	1.0	1.0	1.0	SALL3	Homo sapiens sal-like 3 (Drosophila) (SALL3), mRNA [NM_171999]	27164
A_24_P402510	-2.7	-1.7	-2.6	-1.6	SAMD11	Homo sapiens sterile alpha motif domain containing 11 (SAMD11), mRNA [NM_152486]	148398

A_32_P47265	-6.0	-1.3	-1.9	-2.9	SAMD12	AV724325 HTB Homo sapiens cDNA clone HTBAYE05 5', mRNA sequence [AV724325]	401474
A_32_P130788	-1.0	-1.1	-1.2	-4.9	SAMD13	Homo sapiens sterile alpha motif domain containing 13 (SAMD13), transcript variant 1, mRNA [NM_001010971]	148418
A_24_P383523	1.9	-1.5	2.2	-4.7	SAMD4A	Homo sapiens sterile alpha motif domain containing 4A (SAMD4A), transcript variant 1, mRNA [NM_015589]	23034

A_24_P136758	1.9	3.1	1.7	3.2	SAMD4B	Homo sapiens sterile alpha motif domain containing 4B (SAMD4B), mRNA [NM_018028]	55095
A_24_P6449	2.4	2.4	1.0	-3.2	SAMD7	Homo sapiens sterile alpha motif domain containing 7 (SAMD7), mRNA [NM_182610]	344658
A_23_P145874	-2.3	-1.3	-1.9	1.1	SAMD9L	Homo sapiens sterile alpha motif domain containing 9-like (SAMD9L), mRNA [NM_152703]	219285

A_23_P29005	2.5	2.5	2.5	2.4	SAMSN1	Homo sapiens SAM domain, SH3 domain and nuclear localization signals 1 (SAMSN1), mRNA [NM_022136]	64092
A_23_P154188	2.1	2.0	1.8	1.9	SAP130	Homo sapiens Sin3A-associated protein, 130kDa (SAP130), transcript variant 2, mRNA [NM_024545]	79595
A_24_P247794	1.4	1.3	-3.2	1.0	SAP130	Homo sapiens Sin3A-associated protein, 130kDa (SAP130), transcript variant 1, mRNA [NM_001145928]	79595
A_23_P54953	1.2	1.2	-1.5	-4.1	SAP30BP	Homo sapiens SAP30 binding protein (SAP30BP), mRNA [NM_013260]	29115

A_23_P368101	-1.0	1.2	2.5	-1.4	SAP30L	Homo sapiens SAP30-like (SAP30L), transcript variant 1, mRNA [NM_024632]	79685
A_24_P158421	-1.0	-3.0	-1.0	1.1	SAR1A	Homo sapiens SAR1 homolog A (S. cerevisiae) (SAR1A), transcript variant 2, mRNA [NM_020150]	56681
A_24_P916845	-2.7	-1.4	-2.2	-4.0	SAR1B	Homo sapiens SAR1 homolog B (S. cerevisiae) (SAR1B), transcript variant 1, mRNA [NM_001033503]	51128
A_24_P267293	-2.5	1.6	1.5	1.6	SARM1	Homo sapiens sterile alpha and TIR motif containing 1 (SARM1), mRNA [NM_015077]	23098

A_23_P86884	1.1	1.5	-1.0	-3.1	SART1	Homo sapiens squamous cell carcinoma antigen recognized by T cells (SART1), mRNA [NM_005146]	9092
A_23_P87664	-1.1	-1.1	-2.5	-1.9	SART3	Homo sapiens squamous cell carcinoma antigen recognized by T cells 3 (SART3), mRNA [NM_014706]	9733
A_23_P93442	-3.0	1.2	1.1	-2.1	SASH1	Homo sapiens SAM and SH3 domain containing 1 (SASH1), mRNA [NM_015278]	23328
A_24_P46689	1.4	-2.4	-2.1	-2.0	SASH1	Homo sapiens SAM and SH3 domain containing 1 (SASH1), mRNA [NM_015278]	23328

A_23_P33233	2.1	3.3	1.5	1.4	SASH3	Homo sapiens SAM and SH3 domain containing 3 (SASH3), mRNA [NM_018990]	54440
A_24_P349151	-2.9	-1.1	-6.5	-4.5	SASS6	Homo sapiens spindle assembly 6 homolog (C. elegans) (SASS6), mRNA [NM_194292]	163786
A_23_P15402	2.5	2.5	2.4	2.5	SAT2	Homo sapiens spermidine/spermine N1-acetyltransferase family member 2 (SAT2), mRNA [NM_133491]	112483
A_32_P174365	-1.8	-1.1	-2.8	-2.5	SATB2	Homo sapiens SATB homeobox 2 (SATB2), transcript variant 2, mRNA [NM_015265]	23314

A_23_P65555	-3.1	-4.1	-4.8	-2.0	SAV1	Homo sapiens salvador homolog 1 (Drosophila) (SAV1), mRNA [NM_021818]	60485
A_32_P177024	1.3	-3.1	1.2	-3.0	SBDS	Homo sapiens Shwachman-Bodian-Diamond syndrome (SBDS), mRNA [NM_016038]	51119
A_24_P324630	1.0	-2.0	-1.1	-1.3	SBF2	SET binding factor 2 [Source:HGNC Symbol;Acc:2135] [ENST00000526353]	81846
A_32_P8806	-2.0	-1.1	-3.4	-1.0	SBNO1	Homo sapiens strawberry notch homolog 1 (Drosophila) (SBNO1), transcript variant 1, mRNA [NM_001167856]	55206

A_23_P98446	-3.6	1.1	1.4	1.0	SC5DL	Homo sapiens sterol-C5-desaturase (ERG3 delta-5-desaturase homolog, S. cerevisiae)-like (SC5DL), transcript variant 2, mRNA [NM_001024956]	6309
A_32_P41026	-2.5	3.3	1.4	-1.5	SC5DL	Homo sapiens sterol-C5-desaturase (ERG3 delta-5-desaturase homolog, S. cerevisiae)-like (SC5DL), transcript variant 2, mRNA [NM_001024956]	6309
A_23_P6223	-2.9	1.6	1.2	1.1	SCAF4	Homo sapiens SR-related CTD-associated factor 4 (SCAF4), transcript variant 1, mRNA [NM_020706]	57466

A_23_P325625	2.3	1.8	2.3	1.4	SCAI	Homo sapiens suppresso r of cancer cell invasion (SCAI), transcript variant 1, mRNA [NM_1736 90]	286205
A_23_P405513	1.9	1.1	-5.0	1.4	SCAI	Homo sapiens suppresso r of cancer cell invasion (SCAI), transcript variant 1, mRNA [NM_1736 90]	286205
A_32_P71113	1.7	2.3	2.3	2.2	SCAI	Homo sapiens suppresso r of cancer cell invasion (SCAI), transcript variant 1, mRNA [NM_1736 90]	286205

A_24_P333341	-2.4	1.0	1.1	1.0	SCAMP4	secretory carrier membrane protein 4 [Source:HGNC Symbol;Acc:30385] [ENST00000452128]	113178
A_23_P6196	1.6	3.2	1.9	1.4	SCAND1	Homo sapiens SCAN domain containing 1 (SCAND1), transcript variant 1, mRNA [NM_016558]	51282
A_24_P90792	-18.5	1.0	1.0	1.1	SCAND2	Homo sapiens SCAN domain containing 2 pseudogene (SCAND2), transcript variant 1, non-coding RNA [NR_004859]	54581

A_32_P453191	-4.9	1.0	-1.1	-1.1	SCAND3	Homo sapiens SCAN domain containing 3 (SCAND3), mRNA [NM_052923]	114821
A_23_P215900	1.9	-3.0	1.4	1.1	SCARA3	Homo sapiens scavenger receptor class A, member 3 (SCARA3), transcript variant 1, mRNA [NM_016240]	51435
A_23_P372386	2.0	2.4	1.2	-1.6	SCARA5	Homo sapiens scavenger receptor class A, member 5 (putative) (SCARA5), mRNA [NM_173833]	286133
A_24_P324565	2.3	4.1	3.7	2.1	SCARB1	Homo sapiens scavenger receptor class B, member 1 (SCARB1), transcript variant 1, mRNA [NM_005505]	949

A_24_P108738	-1.1	5.8	-1.0	-1.1	SCARF2	Homo sapiens scavenger receptor class F, member 2 (SCARF2), transcript variant 1, mRNA [NM_153334]	91179
A_23_P132185	1.4	-5.9	1.1	-1.5	SCARF2	Homo sapiens scavenger receptor class F, member 2 (SCARF2), transcript variant 1, mRNA [NM_153334]	91179
A_23_P62807	4.7	22.0	20.4	19.3	SCCPDH	Homo sapiens saccharopine dehydrogenase (putative) (SCCPDH), mRNA [NM_016002]	51097
A_23_P63618	1.3	2.7	-1.1	1.5	SCD	Homo sapiens stearoyl-CoA desaturase (delta-9-desaturase) (SCD), mRNA [NM_005063]	6319

A_32_P231415	1.3	-2.3	-1.4	1.8	SCD	Homo sapiens PRO1933 mRNA, complete cds. [AF132203]	6319
A_24_P385134	-3.7	-1.8	1.0	-46.6	SCD5	Homo sapiens stearoyl-CoA desaturase 5 (SCD5), transcript variant 1, mRNA [NM_001037582]	79966
A_24_P844995	-1.0	-3.9	-1.1	-1.0	SCFD1	sec1 family domain containing 1 [Source:HGNC Symbol;Acc:20726] [ENST00000556486]	23256
A_23_P88209	1.0	-2.2	-1.0	-20.2	SCFD1	Homo sapiens sec1 family domain containing 1 (SCFD1), transcript variant 1, mRNA [NM_016106]	23256

A_23_P92543	2.1	2.3	1.7	-1.1	SCFD2	Homo sapiens sec1 family domain containing 2 (SCFD2), mRNA [NM_152540]	152579
A_23_P62081	-16.2	-1.1	-1.2	-1.2	SCG5	Homo sapiens secretogranin V (7B2 protein) (SCG5), transcript variant 2, mRNA [NM_003020]	6447
A_23_P150583	-2.5	-2.9	-4.9	-4.4	SCGB1A1	Homo sapiens secretoglobin, family 1A, member 1 (uteroglobin) (SCGB1A1), mRNA [NM_003357]	7356
A_23_P127781	-2.3	1.0	1.0	-1.5	SCGB1D1	Homo sapiens secretoglobin, family 1D, member 1 (SCGB1D1), mRNA [NM_006552]	10648

A_23_P150555	2.0	1.9	2.6	1.1	SCGB1D2	Homo sapiens secretoglobin, family 1D, member 2 (SCGB1D2), mRNA [NM_006551]	10647
A_23_P312300	-1.0	-2.1	-1.4	-1.3	SCGB2A1	Homo sapiens secretoglobin, family 2A, member 1 (SCGB2A1), mRNA [NM_002407]	4246
A_32_P191860	1.3	2.1	-1.9	-1.8	SCGBL	Homo sapiens secretoglobin-like (SCGBL), mRNA [NM_001025591]	284402
A_23_P28153	1.1	-17.1	-1.1	-1.1	SCLY	selenocysteine lyase [Source:HGNC Symbol;Acc:18161] [ENST00000409736]	51540

A_24_P162319	1.0	1.0	-3.8	1.0	SCML1	Homo sapiens sex comb on midleg-like 1 (Drosophila) (SCML1), transcript variant 1, mRNA [NM_001037540]	6322
A_23_P28224	-1.2	1.6	-7.7	-1.0	SCN1A	Homo sapiens sodium channel, voltage-gated, type I, alpha subunit (SCN1A), transcript variant 2, mRNA [NM_006920]	6323
A_23_P51019	-4.0	-3.9	-2.8	-4.1	SCN2A	Homo sapiens sodium channel, voltage-gated, type II, alpha subunit (SCN2A), transcript variant 1, mRNA [NM_021007]	6326

A_24_P312790	-1.6	-2.2	-1.3	-2.0	SCN3B	Homo sapiens sodium channel, voltage-gated, type III, beta (SCN3B), transcript variant 1, mRNA [NM_018400]	55800
A_24_P270999	1.3	3.2	1.2	-1.3	SCN4A	Homo sapiens sodium channel, voltage-gated, type IV, alpha subunit (SCN4A), mRNA [NM_000334]	6329
A_23_P303833	-1.0	-1.0	-4.0	-4.0	SCN4B	Homo sapiens sodium channel, voltage-gated, type IV, beta (SCN4B), transcript variant 1, mRNA [NM_174934]	6330

A_23_P90944	-3.9	1.3	1.7	1.6	SCN7A	Homo sapiens sodium channel, voltage-gated, type VII, alpha (SCN7A), mRNA [NM_002976]	6332
A_23_P76245	1.2	1.1	-2.2	1.4	SCN8A	Homo sapiens sodium channel, voltage gated, type VIII, alpha subunit (SCN8A), transcript variant 1, mRNA [NM_014191]	6334
A_23_P34968	2.0	3.0	2.4	-1.4	SCNM1	Homo sapiens sodium channel modifier 1 (SCNM1), transcript variant 2, mRNA [NM_001204856]	79005

A_24_P259799	-1.0	-7.6	1.3	-1.7	SCNM1	Homo sapiens sodium channel modifier 1 (SCNM1), transcript variant 2, mRNA [NM_001204856]	79005
A_23_P128323	2.3	2.9	1.7	1.6	SCNN1A	Homo sapiens sodium channel, nonvoltage-gated 1 alpha (SCNN1A), transcript variant 1, mRNA [NM_001038]	6337
A_32_P83098	-1.2	-2.1	-1.3	-1.3	SCNN1B	Homo sapiens sodium channel, nonvoltage-gated 1, beta (SCNN1B), mRNA [NM_000336]	6338
A_24_P306499	1.2	-1.3	-1.4	-2.5	SCNN1B	Homo sapiens sodium channel, nonvoltage-gated 1, beta (SCNN1B), mRNA [NM_000336]	6338

A_23_P15466	1.0	-1.0	-4.5	1.0	SCO1	Homo sapiens SCO cytochrome oxidase deficient homolog 1 (yeast) (SCO1), nuclear gene encoding mitochondrial protein, mRNA [NM_004589]	6341
A_23_P132388	-2.0	-1.6	-33.5	-2.3	SCO2	Homo sapiens SCO cytochrome oxidase deficient homolog 2 (yeast) (SCO2), nuclear gene encoding mitochondrial protein, transcript variant 1, mRNA [NM_005138]	9997

A_24_P156922	1.0	-3.7	-1.1	1.7	SCP2	Homo sapiens sterol carrier protein 2 (SCP2), transcript variant 2, mRNA [NM_001007098]	6342
A_23_P126057	-2.3	1.0	1.0	1.1	SCP2	Homo sapiens sterol carrier protein 2 (SCP2), transcript variant 1, mRNA [NM_002979]	6342
A_23_P167159	8.6	8.4	8.5	8.6	SCRG1	Homo sapiens stimulator of chondrogenesis 1 (SCRG1), mRNA [NM_007281]	11341
A_23_P159039	-1.0	1.5	1.2	3.2	SCRIB	Homo sapiens scribbled homolog (Drosophila) (SCRIB), transcript variant 1, mRNA [NM_182706]	23513

A_23_P366366	-1.6	2.5	1.5	1.0	SCRN1	Homo sapiens secernin 1 (SCRN1), transcript variant 2, mRNA [NM_014766]	9805
A_24_P318593	1.4	-7.1	-1.6	-1.3	SCRN2	Homo sapiens secernin 2 (SCRN2), transcript variant 1, mRNA [NM_138355]	90507
A_23_P4323	1.5	-2.5	1.1	1.2	SCRN2	Homo sapiens secernin 2 (SCRN2), transcript variant 1, mRNA [NM_138355]	90507
A_23_P17012	1.0	-2.1	-1.1	1.0	SCRN3	Homo sapiens secernin 3 (SCRN3), transcript variant 1, mRNA [NM_024583]	79634

A_24_P367138	4.1	1.6	1.8	1.6	SCRT1	Homo sapiens scratch homolog 1, zinc finger protein (Drosophila) (SCRT1), mRNA [NM_031309]	83482
A_23_P147782	1.3	2.1	1.6	-2.8	SCRT1	Homo sapiens scratch homolog 1, zinc finger protein (Drosophila) (SCRT1), mRNA [NM_031309]	83482
A_24_P117782	1.0	1.3	2.7	1.4	SCRT2	Homo sapiens scratch homolog 2, zinc finger protein (Drosophila) (SCRT2), mRNA [NM_033129]	85508
A_23_P104594	3.0	3.1	1.5	-1.3	SCT	Homo sapiens secretin (SCT), mRNA [NM_021920]	6343

A_23_P211619	-2.1	-1.3	-5.1	-10.0	SCUBE1	Homo sapiens signal peptide, CUB domain, EGF-like 1 (SCUBE1), mRNA [NM_173050]	80274
A_24_P943894	-1.6	-1.3	-2.4	-1.4	SCUBE3	Homo sapiens signal peptide, CUB domain, EGF-like 3 (SCUBE3), mRNA [NM_152753]	222663
A_23_P75470	-15.2	-2.7	-1.9	-3.9	SCYL1	Homo sapiens SCY1-like 1 (S. cerevisiae) (SCYL1), transcript variant B, mRNA [NM_001048218]	57410
A_24_P30314	-7.2	-6.4	-6.7	-9.0	SCYL1	Homo sapiens SCY1-like 1 (S. cerevisiae) (SCYL1), transcript variant A, mRNA [NM_020680]	57410

A_23_P7056	-2.0	1.4	1.1	1.0	SDAD1	Homo sapiens SDA1 domain containing 1 (SDAD1), mRNA [NM_018115]	55153
A_24_P97129	1.2	-20.7	1.4	1.4	SDC1	Homo sapiens syndecan 1 (SDC1), transcript variant 1, mRNA [NM_001006946]	6382
A_23_P16944	2.4	1.3	2.0	1.8	SDC1	Homo sapiens syndecan 1 (SDC1), transcript variant 1, mRNA [NM_001006946]	6382
A_24_P380734	-2.4	1.7	1.7	1.8	SDC2	Homo sapiens syndecan 2 (SDC2), mRNA [NM_002998]	6383
A_23_P109034	2.3	-1.3	2.1	1.3	SDC4	Homo sapiens syndecan 4 (SDC4), mRNA [NM_002999]	6385

A_23_P168974	-5.9	1.2	1.1	-1.8	SDCBP	Homo sapiens cDNA FLJ46804 fis, clone TRACH3032570, highly similar to Homo sapiens syndecan binding protein (syntenin) (SDCBP). [AK128645]	6386
A_23_P157580	3.2	3.2	3.2	1.7	SDCBP	Homo sapiens syndecan binding protein (syntenin) (SDCBP), transcript variant 1, mRNA [NM_005625]	6386
A_23_P131899	-1.0	-1.1	-3.4	-1.1	SDCBP2	Homo sapiens syndecan binding protein (syntenin) 2 (SDCBP2), transcript variant 1, mRNA [NM_080489]	27111

A_24_P179489	2.7	-17.8	3.2	2.4	SDCCAG8	Homo sapiens serologically defined colon cancer antigen 8 (SDCCAG8), mRNA [NM_006642]	10806
A_23_P6344	1.7	1.8	1.1	2.5	SDF2L1	Homo sapiens stromal cell-derived factor 2-like 1 (SDF2L1), mRNA [NM_022044]	23753
A_23_P201338	1.0	-2.4	-1.1	-1.0	SDF4	Homo sapiens stromal cell derived factor 4 (SDF4), transcript variant 2, mRNA [NM_016176]	51150

A_23_P149649	23.4	15.6	9.0	14.0	SDHB	Homo sapiens succinate dehydrogenase complex, subunit B, iron sulfur (Ip) (SDHB), nuclear gene encoding mitochondrial protein, mRNA [NM_003000]	6390
A_32_P60185	-4.8	1.0	-1.3	-3.6	SDHD	Homo sapiens succinate dehydrogenase complex, subunit D, integral membrane protein (SDHD), nuclear gene encoding mitochondrial protein, mRNA [NM_003002]	6392

A_24_P16417	4.1	3.9	1.5	3.5	SDK1	Homo sapiens sidekick homolog 1, cell adhesion molecule (chicken) (SDK1), transcript variant 1, mRNA [NM_152744]	221935
A_24_P108262	1.0	-12.4	-1.0	-1.1	SDK2	Homo sapiens sidekick homolog 2 (chicken) (SDK2), mRNA [NM_001144952]	54549
A_23_P89691	1.2	-1.6	-3.0	-2.9	SDK2	Homo sapiens sidekick homolog 2 (chicken) (SDK2), mRNA [NM_001144952]	54549
A_23_P257457	-4.6	-1.1	-1.1	-1.1	SDR16C5	Homo sapiens short chain dehydrogenase/reductase family 16C, member 5 (SDR16C5), mRNA [NM_138969]	195814

A_23_P163161	9.6	10.3	3.7	13.7	SDR39U1	Homo sapiens short chain dehydrogenase/reductase family 39U, member 1 (SDR39U1), mRNA [NM_020195]	56948
A_23_P371758	-8.4	-1.5	-1.5	-1.5	SDR9C7	Homo sapiens short chain dehydrogenase/reductase family 9C, member 7 (SDR9C7), mRNA [NM_148897]	121214
A_23_P2645	2.5	3.2	4.4	-2.3	SDS	Homo sapiens serine dehydratase (SDS), mRNA [NM_006843]	10993
A_23_P53439	2.0	1.6	-1.4	1.6	SDSL	Homo sapiens serine dehydratase-like (SDSL), mRNA [NM_138432]	113675

A_23_P17811	1.2	-11.1	-1.1	1.5	SEC14L2	Homo sapiens SEC14-like 2 (S. cerevisiae) (SEC14L2), transcript variant 1, mRNA [NM_012429]	23541
A_32_P101779	-5.5	-2.9	-1.0	-1.6	SEC14L3	Homo sapiens SEC14-like 3 (S. cerevisiae) (SEC14L3), mRNA [NM_174975]	266629
A_23_P251303	-2.8	2.2	-1.2	-3.2	SEC16A	Homo sapiens SEC16 homolog A (S. cerevisiae) (SEC16A), mRNA [NM_014866]	9919

A_24_P153734	4.6	5.6	6.2	2.6	SEC16B	Homo sapiens SEC16 homolog B (S. cerevisiae), mRNA (cDNA clone MGC:17455 IMAGE:3448742), complete cds. [BC009106]	89866
A_32_P233019	-1.3	-2.1	-1.6	-1.1	SEC22A	Homo sapiens SEC22 vesicle trafficking protein homolog A (S. cerevisiae) (SEC22A), mRNA [NM_012430]	26984
A_23_P502641	1.0	1.1	2.0	1.1	SEC22C	Homo sapiens SEC22 vesicle trafficking protein homolog C (S. cerevisiae) (SEC22C), transcript variant 1, mRNA [NM_032970]	9117

A_24_P227017	4.6	4.4	2.0	4.3	SEC23IP	Homo sapiens SEC23 interacting protein (SEC23IP), transcript variant 1, mRNA [NM_007190]	11196
A_24_P255338	-2.5	-1.8	-1.5	-1.4	SEC24C	Homo sapiens SEC24 family, member C (S. cerevisiae) (SEC24C), transcript variant 1, mRNA [NM_004922]	9632
A_23_P170273	1.4	1.4	1.5	-4.0	SEC31A	Homo sapiens SEC31 homolog A (S. cerevisiae) (SEC31A), transcript variant 5, mRNA [NM_001077207]	22872

A_24_P415280	-3.9	-2.5	-5.1	-1.2	SEC61A2	Homo sapiens Sec61 alpha 2 subunit (S. cerevisiae) (SEC61A2), transcript variant 1, mRNA [NM_018144]	55176
A_24_P8494	3.2	2.1	1.1	-1.1	SEC61B	Homo sapiens Sec61 beta subunit (SEC61B), mRNA [NM_006808]	10952
A_23_P357860	1.3	-2.1	1.1	1.1	SEC62	Homo sapiens SEC62 homolog (S. cerevisiae) (SEC62), mRNA [NM_003262]	7095
A_24_P285880	-1.8	-2.0	-2.1	-2.0	SEC62	Homo sapiens SEC62 homolog (S. cerevisiae) (SEC62), mRNA [NM_003262]	7095

A_24_P402064	-3.1	-2.0	1.0	-1.9	SEC63	Homo sapiens SEC63 homolog (S. cerevisiae) (SEC63), mRNA [NM_007214]	11231
A_24_P123720	2.2	2.1	-1.4	1.3	SEC63	Homo sapiens SEC63 homolog (S. cerevisiae) (SEC63), mRNA [NM_007214]	11231
A_24_P119482	1.3	-2.0	-1.8	-3.6	SECISBP2	Homo sapiens SECIS binding protein 2 (SECISBP2), mRNA [NM_024077]	79048
A_24_P82135	4.7	7.0	7.0	7.2	SECISBP2L	Homo sapiens SECIS binding protein 2-like (SECISBP2L), transcript variant 2, mRNA [NM_014701]	9728

A_23_P37415	2.7	3.1	-1.1	1.1	SECISBP2L	Homo sapiens SECIS binding protein 2-like (SECISBP2L), transcript variant 2, mRNA [NM_014701]	9728
A_23_P207905	-1.2	2.1	1.1	1.6	SECTM1	Homo sapiens secreted and transmembrane 1 (SECTM1), mRNA [NM_003004]	6398
A_24_P335656	2.4	2.5	-1.0	1.1	SECTM1	Homo sapiens secreted and transmembrane 1 (SECTM1), mRNA [NM_003004]	6398
A_24_P48204	-3.3	1.2	-2.2	-1.0	SECTM1	Homo sapiens secreted and transmembrane 1 (SECTM1), mRNA [NM_003004]	6398

A_23_P74619	-1.1	-2.2	-2.3	-1.4	SELENBP1	Homo sapiens selenium binding protein 1 (SELENBP1), mRNA [NM_003944]	8991
A_23_P103522	-1.3	-1.3	-2.1	-1.5	SELL	Homo sapiens selectin L (SELL), transcript variant 1, mRNA [NM_000655]	6402
A_23_P6413	2.2	2.1	2.1	-3.0	SELM	Homo sapiens selenoprotein M (SELM), mRNA [NM_080430]	140606
A_24_P49322	1.8	1.6	-7.9	-2.0	SELO	Homo sapiens selenoprotein O (SELO), mRNA [NM_031454]	83642
A_24_P341853	-2.1	-2.1	-1.1	1.4	SELO	Homo sapiens selenoprotein O (SELO), mRNA [NM_031454]	83642

A_23_P256473	-1.0	-1.0	-1.0	-16.9	SEMA3C	Homo sapiens sema domain, immunoglobulin domain (Ig), short basic domain, secreted, (semaphorin) 3C (SEMA3C), mRNA [NM_006379]	10512
A_23_P405287	1.0	-10.1	-1.0	-1.1	SEMA3D	Homo sapiens sema domain, immunoglobulin domain (Ig), short basic domain, secreted, (semaphorin) 3D (SEMA3D), mRNA [NM_152754]	223117

A_23_P59582	-4.7	-1.0	-1.1	-1.1	SEMA3E	Homo sapiens sema domain, immunoglobulin domain (Ig), short basic domain, secreted, (semaphorin) 3E (SEMA3E), transcript variant 1, mRNA [NM_012431]	9723
A_23_P6878	1.1	-2.1	-3.2	1.0	SEMA3F	Homo sapiens sema domain, immunoglobulin domain (Ig), short basic domain, secreted, (semaphorin) 3F (SEMA3F), mRNA [NM_004186]	6405

A_23_P6818	-1.6	-2.6	1.0	1.2	SEMA3G	Homo sapiens sema domain, immunoglobulin domain (Ig), short basic domain, secreted, (semaphorin) 3G (SEMA3G), mRNA [NM_020163]	56920
A_23_P23438	1.6	-2.9	1.2	-1.0	SEMA4A	Homo sapiens sema domain, immunoglobulin domain (Ig), transmembrane domain (TM) and short cytoplasmic domain, (semaphorin) 4A (SEMA4A), transcript variant 1, mRNA [NM_022367]	64218

A_23_P95165	1.4	-5.7	-2.1	-2.8	SEMA4B	Homo sapiens sema domain, immunoglobulin domain (Ig), transmembrane domain (TM) and short cytoplasmic domain, (semaphorin) 4B (SEMA4B), transcript variant 1, mRNA [NM_020210]	10509
A_23_P9677	-1.5	1.4	-3.2	-2.2	SEMA4C	Homo sapiens sema domain, immunoglobulin domain (Ig), transmembrane domain (TM) and short cytoplasmic domain, (semaphorin) 4C (SEMA4C), mRNA [NM_017789]	54910

A_23_P121374	1.0	-1.0	-1.1	-3.5	SEMA5B	Homo sapiens sema domain, seven thrombospondin repeats (type 1 and type 1-like), transmembrane domain (TM) and short cytoplasmic domain, (semaphorin) 5B (SEMA5B), transcript variant 1, mRNA [NM_001031702]	54437
A_24_P857669	3.2	1.2	3.0	1.6	SEMA6A	sema domain, transmembrane domain (TM), and cytoplasmic domain, (semaphorin) 6A [Source:HGNC Symbol;Acc:10738] [ENST00000513137]	57556

A_23_P208900	2.2	1.5	-1.4	1.7	SEMA6B	Homo sapiens sema domain, transmembrane domain (TM), and cytoplasmic domain, (semaphorin) 6B (SEMA6B), mRNA [NM_032108]	10501
A_23_P138000	1.3	1.4	-2.4	1.2	SEMA6C	Homo sapiens sema domain, transmembrane domain (TM), and cytoplasmic domain, (semaphorin) 6C (SEMA6C), transcript variant 2, mRNA [NM_030913]	10500

A_23_P420442	-1.1	-1.1	2.0	-1.0	SEMA6D	Homo sapiens sema domain, transmembrane domain (TM), and cytoplasmic domain, (semaphorin) 6D (SEMA6D), transcript variant 4, mRNA [NM_153618]	80031
A_23_P5968	1.1	-2.6	-2.4	1.0	SEMG2	Homo sapiens semenogelin II (SEMG2), mRNA [NM_003008]	6407
A_24_P167412	-2.6	1.3	1.2	1.3	SENP1	Homo sapiens SUMO1/sentrin specific peptidase 1 (SENP1), mRNA [NM_014554]	29843
A_32_P74712	8.3	8.2	7.9	8.2	SENP2	Homo sapiens SUMO1/sentrin/SMT3 specific peptidase 2 (SENP2), mRNA [NM_021627]	59343

A_24_P106382	2.4	3.1	2.2	1.9	SENP6	Homo sapiens cDNA FLJ10544 fis, clone NT2RP2001601, highly similar to Sentrin-specific protease 6 (EC 3.4.22.-). [AK001406]	26054
A_23_P214156	1.0	1.0	-8.2	-1.0	SENP6	Homo sapiens SUMO1/sentrin specific peptidase 6 (SENP6), transcript variant 1, mRNA [NM_015571]	26054
A_23_P334263	1.9	2.9	1.7	3.8	SENP8	Homo sapiens SUMO/sentrin specific peptidase family member 8 (SENP8), transcript variant 2, mRNA [NM_145204]	123228

A_23_P370625	-1.4	-2.8	1.8	-4.7	SEPN1	Homo sapiens selenoprotein N, 1 (SEPN1), transcript variant 1, mRNA [NM_020451]	57190
A_24_P377183	-11.9	1.3	1.2	-1.1	SEPSECS	Homo sapiens Sep (O-phosphoserine) tRNA:Sec (selenocysteine) tRNA synthase (SEPSECS), mRNA [NM_016955]	51091
A_23_P167201	-4.4	-1.0	-4.3	-1.1	SEPSECS	Homo sapiens Sep (O-phosphoserine) tRNA:Sec (selenocysteine) tRNA synthase (SEPSECS), mRNA [NM_016955]	51091

A_24_P75879	-1.0	-2.8	-1.0	-1.0	SEPT7P2	Homo sapiens septin 7 pseudogene 2 (SEPT7P2), non-coding RNA [NR_024271]	641977
A_32_P187599	-1.1	-1.0	-3.1	-4.5	SERBP1	Homo sapiens SERPINE1 mRNA binding protein 1 (SERBP1), transcript variant 1, mRNA [NM_001018067]	26135
A_24_P935881	1.3	1.2	1.4	-2.0	SERF1B	Homo sapiens small EDRK-rich factor 1B (centromeric) (SERF1B), transcript variant 2, mRNA [NM_022978]	728492
A_24_P313334	-4.7	-1.0	-1.0	1.0	SERF2	Homo sapiens small EDRK-rich factor 2 (SERF2), transcript variant 4, mRNA [NM_001199877]	10169

A_24_P364087	2.4	-1.3	-1.0	-1.3	SERGEF	Homo sapiens secretion regulating guanine nucleotide exchange factor (SERGEF), mRNA [NM_012139]	26297
A_23_P120953	2.0	2.0	3.2	1.0	SERHL2	Homo sapiens serine hydrolase-like 2 (SERHL2), mRNA [NM_014509]	253190
A_24_P129232	-1.0	-3.3	-1.2	-1.9	SERINC1	Homo sapiens serine incorporator 1 (SERINC1), mRNA [NM_020755]	57515
A_23_P147326	2.1	1.7	1.8	1.6	SERINC2	Homo sapiens serine incorporator 2 (SERINC2), transcript variant 1, mRNA [NM_178865]	347735

A_24_P160586	1.4	3.2	1.5	2.6	SERINC4	serine incorporator 4 [Source:HGNC Symbol;Acc:32237] [ENST00000476490]	619189
A_32_P138977	1.4	1.4	1.0	-3.1	SERINC4	serine incorporator 4 [Source:HGNC Symbol;Acc:32237] [ENST00000319327]	619189
A_23_P423457	-1.6	2.9	-1.1	1.2	SERINC5	Homo sapiens serine incorporator 5 (SERINC5), transcript variant 2, mRNA [NM_178276]	256987
A_24_P579862	2.1	2.1	1.0	-1.2	SERP1	stress-associated endoplasmic reticulum protein 1 [Source:HGNC Symbol;Acc:10759] [ENST00000490945]	27230

A_23_P218111	-2.6	1.7	-1.2	-4.5	SERPINA1	Homo sapiens serpin peptidase inhibitor, clade A (alpha-1 antiproteinase, antitrypsin), member 1 (SERPINA1), transcript variant 2, mRNA [NM_001002236]	5265
A_23_P88177	4.0	2.9	3.0	1.9	SERPINA12	Homo sapiens serpin peptidase inhibitor, clade A (alpha-1 antiproteinase, antitrypsin), member 12 (SERPINA12), mRNA [NM_173850]	145264

A_24_P341116	-36.3	-1.0	-1.1	-1.0	SERPINA13	Homo sapiens serpin peptidase inhibitor, clade A (alpha-1 antiproteinase, antitrypsin), member 13 (pseudogene) (SERPINA13), non-coding RNA [NR_015340]	388007
A_23_P44663	2.3	-1.6	-1.3	-1.7	SERPINA2	PREDICTED: Homo sapiens serpin peptidase inhibitor, clade A (alpha-1 antiproteinase, antitrypsin), member 2 (SERPINA2), miscRNA [XR_110240]	390502

A_23_P117363	-1.0	-1.6	-2.3	-2.3	SERPINA6	Homo sapiens serpin peptidase inhibitor, clade A (alpha-1 antiproteinase, antitrypsin), member 6 (SERPINA6), mRNA [NM_001756]	866
A_23_P257307	-2.9	1.4	1.4	1.4	SERPINA7	Homo sapiens serpin peptidase inhibitor, clade A (alpha-1 antiproteinase, antitrypsin), member 7 (SERPINA7), mRNA [NM_000354]	6906

A_23_P372478	5.3	1.3	2.6	2.9	SERPINA9	Homo sapiens serpin peptidase inhibitor, clade A (alpha-1 antiproteinase, antitrypsin), member 9 (SERPINA9), transcript variant A, mRNA [NM_175739]	327657
A_24_P269315	-2.9	-1.8	-7.2	-2.6	SERPINB1	serpin peptidase inhibitor, clade B (ovalbumin), member 1 [Source:HGNC Symbol;Acc:3311] [ENST00000380739]	1992

A_23_P130411	-1.6	1.4	-2.0	1.0	SERPINB11	Homo sapiens serpin peptidase inhibitor, clade B (ovalbumin), member 11 (gene/pseudogene) (SERPINB11), mRNA [NM_080475]	89778
A_23_P107623	1.0	1.0	-6.1	1.1	SERPINB12	Homo sapiens serpin peptidase inhibitor, clade B (ovalbumin), member 12 (SERPINB12), mRNA [NM_080474]	89777
A_23_P432978	1.0	-9.7	1.0	1.3	SERPINB13	Homo sapiens serpin peptidase inhibitor, clade B (ovalbumin), member 13 (SERPINB13), mRNA [NM_012397]	5275

A_23_P119015	-2.7	-1.1	-1.2	-1.2	SERPINB13	Homo sapiens serpin peptidase inhibitor, clade B (ovalbumin), member 13 (SERPINB13), mRNA [NM_012397]	5275
A_23_P153185	-1.6	-1.6	-2.5	-2.0	SERPINB2	Homo sapiens serpin peptidase inhibitor, clade B (ovalbumin), member 2 (SERPINB2), transcript variant 1, mRNA [NM_001143818]	5055
A_24_P245379	4.6	10.8	6.3	5.5	SERPINB2	Homo sapiens serpin peptidase inhibitor, clade B (ovalbumin), member 2 (SERPINB2), transcript variant 2, mRNA [NM_002575]	5055

A_23_P208126	1.6	-2.5	1.6	1.0	SERPINB5	Homo sapiens serpin peptidase inhibitor, clade B (ovalbumin), member 5 (SERPINB5), mRNA [NM_002639]	5268
A_23_P30687	-2.3	1.4	1.0	1.0	SERPINB9	Homo sapiens serpin peptidase inhibitor, clade B (ovalbumin), member 9 (SERPINB9), mRNA [NM_004155]	5272
A_32_P38323	1.1	-4.3	-1.0	1.1	SERPINB9	Homo sapiens serpin peptidase inhibitor, clade B (ovalbumin), member 9 (SERPINB9), mRNA [NM_004155]	5272

A_23_P50919	1.1	-12.4	1.1	1.1	SERPINE2	Homo sapiens serpin peptidase inhibitor, clade E (nexin, plasminogen activator inhibitor type 1), member 2 (SERPINE2), transcript variant 1, mRNA [NM_006216]	5270
A_23_P162861	1.1	-5.7	-1.1	-1.0	SERPINE3	Homo sapiens serpin peptidase inhibitor, clade E (nexin, plasminogen activator inhibitor type 1), member 3 (SERPINE3), mRNA [NM_001101320]	647174

A_23_P89270	-1.1	-4.1	1.5	-2.3	SERPINF2	Homo sapiens serpin peptidase inhibitor, clade F (alpha-2 antiplasmin, pigment epithelium derived factor), member 2 (SERPINF2), transcript variant 1, mRNA [NM_000934]	5345
A_23_P132826	-1.1	-1.9	-2.6	-2.3	SERPINI2	Homo sapiens serpin peptidase inhibitor, clade I (pancpin), member 2 (SERPINI2), mRNA [NM_006217]	5276
A_23_P141960	1.8	-1.6	-2.5	1.0	SERTAD3	Homo sapiens SERTA domain containing 3 (SERTAD3), transcript variant 2, mRNA [NM_203344]	29946

A_23_P318904	1.0	1.0	-2.9	1.0	SERTAD4	Homo sapiens SERTA domain containing 4 (SERTAD4), mRNA [NM_019605]	56256
A_23_P35082	-2.4	1.1	-1.0	-1.0	SESN2	Homo sapiens sestrin 2 (SESN2), mRNA [NM_031459]	83667
A_24_P918706	-3.1	-1.5	-3.0	-3.0	SESN3	sestrin 3 [Source:HGNC Symbol;Acc:23060] [ENST00000536441]	143686
A_23_P367610	-2.6	2.2	2.3	1.6	SESTD1	Homo sapiens SEC14 and spectrin domains 1 (SESTD1), mRNA [NM_178123]	91404
A_23_P4551	-2.3	-4.6	-2.0	-4.7	SETBP1	Homo sapiens SET binding protein 1 (SETBP1), transcript variant 1, mRNA [NM_015559]	26040

A_23_P44760	-1.6	-1.1	1.0	2.8	SETD2	Homo sapiens SET domain containing 2 (SETD2), mRNA [NM_014159]	29072
A_23_P21219	-1.5	-1.3	-2.4	1.4	SETD2	Homo sapiens SET domain containing 2 (SETD2), mRNA [NM_014159]	29072
A_23_P25989	1.2	-7.8	-1.0	-1.0	SETD3	Homo sapiens SET domain containing 3 (SETD3), transcript variant 1, mRNA [NM_032233]	84193
A_23_P212204	-1.0	-1.0	-15.5	-1.0	SETD5	Homo sapiens mRNA; cDNA DKFZp686A20205 (from clone DKFZp686A20205). [BX648380]	55209

A_24_P944427	1.0	-1.1	-5.4	-1.0	SETD5	Homo sapiens SET domain containing 5 (SETD5), mRNA [NM_001080517]	55209
A_23_P129358	1.1	1.0	-1.0	2.1	SETD6	Homo sapiens SET domain containing 6 (SETD6), transcript variant 2, mRNA [NM_024860]	79918
A_32_P191527	-1.2	1.1	-4.1	-1.1	SETD8	Homo sapiens SET domain containing (lysine methyltransferase) 8 (SETD8), mRNA [NM_020382]	387893
A_32_P154121	-15.8	1.1	-1.0	-1.0	SETDB2	Homo sapiens SET domain, bifurcated 2 (SETDB2), transcript variant 1, mRNA [NM_031915]	83852

A_24_P917261	-1.0	-1.1	-3.2	-1.1	SETX	Homo sapiens senataxin (SETX), mRNA [NM_015046]	23064
A_23_P389569	-1.3	-2.0	1.6	-1.6	SEZ6	Homo sapiens seizure related 6 homolog (mouse) (SEZ6), transcript variant 1, mRNA [NM_178860]	124925
A_24_P116587	3.2	2.6	2.1	2.4	SEZ6L2	Homo sapiens seizure related 6 homolog (mouse)-like 2 (SEZ6L2), transcript variant 2, mRNA [NM_201575]	26470
A_23_P10194	2.5	3.7	2.2	2.0	SEZ6L2	Homo sapiens seizure related 6 homolog (mouse)-like 2 (SEZ6L2), transcript variant 2, mRNA [NM_201575]	26470

A_24_P374154	1.0	1.5	-2.7	1.1	SF3A1	Homo sapiens splicing factor 3a, subunit 1, 120kDa (SF3A1), transcript variant 1, mRNA [NM_005877]	10291
A_23_P64083	-1.1	-1.8	-2.2	-1.2	SF3B2	Homo sapiens splicing factor 3b, subunit 2, 145kDa (SF3B2), mRNA [NM_006842]	10992
A_32_P173298	-2.1	1.0	-2.1	1.0	SF3B3	Homo sapiens splicing factor 3b, subunit 3, 130kDa (SF3B3), mRNA [NM_012426]	23450
A_23_P35131	14.6	19.8	14.3	10.9	SF3B4	Homo sapiens splicing factor 3b, subunit 4, 49kDa (SF3B4), mRNA [NM_005850]	10262

A_24_P242021	3.5	1.9	-1.7	2.6	SFI1	Homo sapiens Sfi1 homolog, spindle assembly associated (yeast) (SFI1), transcript variant 1, mRNA [NM_001007467]	9814
A_24_P417460	-1.0	-2.1	-1.6	-1.2	SFMBT2	Homo sapiens Scm-like with four mbt domains 2 (SFMBT2), transcript variant 1, mRNA [NM_001029880]	57713
A_32_P98291	-1.0	-1.1	-3.7	-1.1	SFMBT2	Homo sapiens Scm-like with four mbt domains 2 (SFMBT2), transcript variant 1, mRNA [NM_001029880]	57713
A_23_P63254	2.4	-1.2	2.1	-1.0	SFN	Homo sapiens stratifin (SFN), mRNA [NM_006142]	2810

A_23_P10121	1.1	1.0	2.2	1.1	SFRP1	Homo sapiens secreted frizzled-related protein 1 (SFRP1), mRNA [NM_003012]	6422
A_24_P934546	-3.0	-1.1	-2.7	1.4	SFRP2	Homo sapiens secreted frizzled-related protein 2 (SFRP2), mRNA [NM_003013]	6423
A_24_P137501	2.5	4.0	2.4	3.4	SFRP2	Homo sapiens secreted frizzled-related protein 2 (SFRP2), mRNA [NM_003013]	6423
A_23_P215328	-1.2	-3.0	-1.3	-1.3	SFRP4	Homo sapiens secreted frizzled-related protein 4 (SFRP4), mRNA [NM_003014]	6424

A_23_P1352	1.0	-2.3	1.0	1.0	SFRP5	Homo sapiens secreted frizzled-related protein 5 (SFRP5), mRNA [NM_003015]	6425
A_32_P109704	-17.5	-1.0	1.6	2.1	SFSWAP	Homo sapiens splicing factor, suppressor of white-apricot homolog (Drosophila) (SFSWAP), mRNA [NM_004592]	6433
A_23_P321959	1.1	-2.1	1.3	-1.3	SFT2D1	Homo sapiens SFT2 domain containing 1 (SFT2D1), mRNA [NM_145169]	113402
A_24_P923749	-1.8	-2.7	-2.6	-1.3	SFT2D3	Homo sapiens SFT2 domain containing 3 (SFT2D3), mRNA [NM_032740]	84826

A_23_P5568	-1.3	-1.8	-2.0	-1.0	SFT2D3	Homo sapiens SFT2 domain containing 3 (SFT2D3), mRNA [NM_032740]	84826
A_23_P115652	-3.3	-1.3	-5.2	1.4	SFTPA1	Homo sapiens surfactant protein A1 (SFTPA1), transcript variant 1, mRNA [NM_005411]	653509
A_23_P17044	3.9	-5.4	3.2	1.6	SFTPB	Homo sapiens surfactant protein B (SFTPB), transcript variant 1, mRNA [NM_000542]	6439
A_23_P95213	1.7	1.6	1.4	6.0	SFTPC	Homo sapiens surfactant protein C (SFTPC), transcript variant 1, mRNA [NM_003018]	6440

A_24_P250666	-2.6	1.9	-1.4	-1.9	SFTPC	Homo sapiens surfactant protein C (SFTPC), transcript variant 1, mRNA [NM_003018]	6440
A_24_P394420	1.1	-2.6	-1.1	-1.0	SFXN1	Homo sapiens sideroflexin 1 (SFXN1), mRNA [NM_022754]	94081
A_23_P202138	15.6	29.5	21.7	8.0	SFXN3	Homo sapiens sideroflexin 3 (SFXN3), mRNA [NM_030971]	81855
A_23_P136254	-4.6	-2.6	1.1	-1.6	SGCD	Homo sapiens sarcoglycan, delta (35kDa dystrophin-associated glycoprotein) (SGCD), transcript variant 2, mRNA [NM_172244]	6444

A_23_P373584	1.1	-1.2	-3.2	-1.1	SGCZ	Homo sapiens sarcoglycan, zeta (SGCZ), mRNA [NM_139167]	137868
A_23_P62881	-1.5	-1.5	-1.7	-2.3	SGIP1	Homo sapiens SH3-domain GRB2-like (endophilin) interacting protein 1 (SGIP1), mRNA [NM_032291]	84251
A_24_P333857	-5.0	-1.5	-7.8	1.0	SGIP1	Homo sapiens SH3-domain GRB2-like (endophilin) interacting protein 1 (SGIP1), mRNA [NM_032291]	84251
A_23_P131801	-1.8	-1.1	-2.5	1.3	SGK2	Homo sapiens serum/glucocorticoid regulated kinase 2 (SGK2), transcript variant 1, mRNA [NM_170693]	10110

A_23_P250212	1.0	-1.3	-1.6	-2.3	SGK223	Homo sapiens homolog of rat pragma of Rnd2 (SGK223), mRNA [NM_001080826]	157285
A_24_P46953	-2.5	1.5	1.5	2.4	SGK3	Homo sapiens serum/glucocorticoid regulated kinase family, member 3 (SGK3), transcript variant 1, mRNA [NM_013257]	23678
A_23_P326204	3.4	5.5	1.7	2.6	SGMS2	Homo sapiens sphingomyelin synthase 2 (SGMS2), transcript variant 1, mRNA [NM_152621]	166929
A_23_P29723	1.0	-1.1	-3.1	-1.0	SGOL1	Homo sapiens shugoshin-like 1 (S. pombe) (SGOL1), transcript variant A2, mRNA [NM_001012410]	151648

A_24_P940815	1.0	-1.0	-3.1	-1.1	SGPL1	Homo sapiens sphingosine-1-phosphate lyase 1 (SGPL1), mRNA [NM_003901]	8879
A_23_P347048	-2.5	-1.3	-3.2	-1.1	SGPP1	Homo sapiens sphingosine-1-phosphate phosphatase 1 (SGPP1), mRNA [NM_030791]	81537
A_23_P254254	1.3	-1.1	1.5	-27.7	SGSH	Homo sapiens N-sulfoglucosamine sulfohydrolase (SGSH), mRNA [NM_000199]	6448
A_23_P166491	-5.0	2.0	1.7	1.3	SGSM3	Homo sapiens small G protein signaling modulator 3 (SGSM3), mRNA [NM_015705]	27352

A_23_P108331	2.1	2.4	2.0	-1.6	SGTA	Homo sapiens small glutamine-rich tetratricopeptide repeat (TPR)-containing, alpha (SGTA), mRNA [NM_003021]	6449
A_24_P399585	5.0	-3.3	1.0	3.1	SGTA	Homo sapiens small glutamine-rich tetratricopeptide repeat (TPR)-containing, alpha (SGTA), mRNA [NM_003021]	6449
A_23_P7376	1.0	1.0	-1.0	-5.4	SGTB	Homo sapiens small glutamine-rich tetratricopeptide repeat (TPR)-containing, beta (SGTB), mRNA [NM_019072]	54557

A_24_P185029	1.2	2.2	1.2	2.6	SH2B1	Homo sapiens SH2B adaptor protein 1 (SH2B1), transcript variant 2, mRNA [NM_015503]	25970
A_23_P59507	2.9	2.4	1.2	1.2	SH2B2	Homo sapiens SH2B adaptor protein 2 (SH2B2), mRNA [NM_020979]	10603
A_23_P13946	-6.2	1.0	1.1	-1.8	SH2B3	Homo sapiens SH2B adaptor protein 3 (SH2B3), mRNA [NM_005475]	10019
A_23_P351148	-1.7	-1.8	-2.0	-1.9	SH2D1B	Homo sapiens SH2 domain containing 1B (SH2D1B), mRNA [NM_053282]	117157

A_23_P160618	3.2	2.1	-1.1	5.0	SH2D2A	Homo sapiens SH2 domain containing 2A (SH2D2A), transcript variant 2, mRNA [NM_003975]	9047
A_24_P262728	1.4	6.5	-1.2	-1.2	SH2D3A	Homo sapiens SH2 domain containing 3A (SH2D3A), mRNA [NM_005490]	10045
A_23_P169003	2.4	1.7	1.6	1.1	SH2D4A	Homo sapiens SH2 domain containing 4A (SH2D4A), transcript variant 1, mRNA [NM_022071]	63898
A_24_P417904	1.0	1.0	-2.0	-1.0	SH2D4B	Homo sapiens SH2 domain containing 4B (SH2D4B), transcript variant 1, mRNA [NM_207372]	387694

A_24_P686965	-1.1	-2.3	-2.3	-1.2	SH2D5	Homo sapiens SH2 domain containing 5 (SH2D5), transcript variant 1, mRNA [NM_001103161]	400745
A_24_P573089	1.1	-1.0	-2.7	-4.1	SH2D5	Homo sapiens SH2 domain containing 5 (SH2D5), transcript variant 1, mRNA [NM_001103161]	400745
A_23_P91520	2.4	-1.6	1.0	-1.0	SH3BGR	Homo sapiens SH3 domain binding glutamic acid-rich protein (SH3BGR), transcript variant 1, mRNA [NM_007341]	6450

A_24_P209171	-1.1	-2.0	-5.8	-1.3	SH3BGRL2	Homo sapiens SH3 domain binding glutamic acid-rich protein like 2 (SH3BGRL2), mRNA [NM_031469]	83699
A_24_P226322	1.1	1.4	-1.0	-4.3	SH3BGRL2	Homo sapiens SH3 domain binding glutamic acid-rich protein like 2 (SH3BGRL2), mRNA [NM_031469]	83699
A_23_P126388	-1.5	-3.0	1.2	-1.3	SH3BGRL3	Homo sapiens SH3 domain binding glutamic acid-rich protein like 3 (SH3BGRL3), mRNA [NM_031286]	83442

A_23_P110116	-1.1	-1.0	-1.3	-10.7	SH3BP2	Homo sapiens SH3-domain binding protein 2 (SH3BP2), transcript variant 1, mRNA [NM_003023]	6452
A_24_P148750	1.4	1.3	1.7	-3.1	SH3BP5	Homo sapiens SH3-domain binding protein 5 (BTK-associated) (SH3BP5), transcript variant 1, mRNA [NM_004844]	9467
A_23_P363	1.1	-1.2	1.3	2.1	SH3D21	Homo sapiens SH3 domain containing 21 (SH3D21), transcript variant 2, mRNA [NM_024676]	79729

A_24_P139094	-1.0	1.0	1.6	-3.3	SH3GL1	Homo sapiens SH3-domain GRB2-like 1 (SH3GL1), transcript variant 1, mRNA [NM_003025]	6455
A_24_P386334	-1.5	-1.4	-1.6	-2.1	SH3GLB2	Homo sapiens SH3-domain GRB2-like endophilin B2 (SH3GLB2), mRNA [NM_020145]	56904
A_24_P289139	1.3	-3.6	-2.0	-1.1	SH3KBP1	Homo sapiens SH3-domain kinase binding protein 1 (SH3KBP1), transcript variant 2, mRNA [NM_001024666]	30011
A_23_P35456	1.5	1.6	1.3	2.7	SH3PXD2A	Homo sapiens SH3 and PX domains 2A (SH3PXD2A), mRNA [NM_014631]	9644

A_24_P589266	1.1	1.1	1.3	-2.3	SH3RF3	Homo sapiens SH3 domain containing ring finger 3 (SH3RF3), mRNA [NM_001099289]	344558
A_23_P7473	1.0	1.4	-1.0	-3.8	SH3TC2	Homo sapiens SH3 domain and tetratricopeptide repeats 2 (SH3TC2), mRNA [NM_024577]	79628
A_24_P922367	-1.0	-4.7	-1.1	1.0	SH3YL1	Homo sapiens SH3 domain containing, Ysc84-like 1 (S. cerevisiae), mRNA (cDNA clone IMAGE:4825726), complete cds. [BC030778]	26751

A_23_P334883	2.2	1.9	2.2	2.1	SHANK2	Homo sapiens SH3 and multiple ankyrin repeat domains 2 (SHANK2), transcript variant 1, mRNA [NM_012309]	22941
A_24_P194881	1.7	-7.4	1.9	-1.1	SHANK3	Homo sapiens SH3 and multiple ankyrin repeat domains 3 (SHANK3), mRNA [NM_001080420]	85358
A_23_P257743	1.4	2.3	-1.1	3.8	SHB	Homo sapiens Src homology 2 domain containing adaptor protein B (SHB), mRNA [NM_003028]	6461
A_24_P146575	-4.0	-1.4	-2.4	1.3	SHB	Homo sapiens Src homology 2 domain containing adaptor protein B (SHB), mRNA [NM_003028]	6461

A_23_P207089	-5.5	1.0	1.0	1.1	SHBG	Homo sapiens sex hormone-binding globulin (SHBG), transcript variant 1, mRNA [NM_001040]	6462
A_24_P68585	-2.1	1.8	1.8	1.2	SHC1	Homo sapiens SHC (Src homology 2 domain containing) transforming protein 1 (SHC1), transcript variant 1, mRNA [NM_183001]	6464
A_32_P176036	4.6	9.3	6.9	3.4	SHC3	Homo sapiens SHC (Src homology 2 domain containing) transforming protein 3 (SHC3), mRNA [NM_016848]	53358

A_24_P350223	1.8	-4.6	1.9	1.4	SHC4	Homo sapiens SHC (Src homology 2 domain containing) family, member 4 (SHC4), mRNA [NM_203349]	399694
A_23_P136571	2.5	-2.0	1.1	-1.7	SHCBP1L	Homo sapiens SHC SH2-domain binding protein 1-like (SHCBP1L), mRNA [NM_030933]	81626
A_23_P142255	2.3	2.4	-1.6	-31.5	SHD	Homo sapiens Src homology 2 domain containing transforming protein D (SHD), mRNA [NM_020209]	56961
A_23_P400716	1.0	-1.0	-2.2	-1.1	SHE	Homo sapiens Src homology 2 domain containing E (SHE), mRNA [NM_001010846]	126669

A_24_P794275	1.0	1.0	2.1	1.0	SHE	Src homology 2 domain containing E [Source:HGNC Symbol;Acc:27004] [ENST00000486773]	126669
A_32_P183904	1.0	-2.5	-1.0	1.1	SHF	Homo sapiens Src homology 2 domain containing F (SHF), mRNA [NM_138356]	90525
A_24_P101314	-1.0	-1.2	-4.2	-2.4	SHF	Homo sapiens cDNA FLJ41664 fis, clone FEBRA2027352. [AK123658]	90525
A_23_P42664	1.0	-3.6	1.1	1.0	SHFM1	Homo sapiens split hand/foot malformation (ectrodactyly) type 1 (SHFM1), mRNA [NM_006304]	7979

A_32_P55241	1.0	-1.0	-5.6	-1.1	SHISA2	Homo sapiens shisa homolog 2 (Xenopus laevis) (SHISA2), mRNA [NM_001007538]	387914
A_24_P46159	-1.8	-2.8	-3.8	-1.8	SHISA5	Homo sapiens shisa homolog 5 (Xenopus laevis) (SHISA5), mRNA [NM_016479]	51246
A_24_P161581	-1.2	-2.1	-4.0	-1.5	SHISA7	Homo sapiens shisa homolog 7 (Xenopus laevis) (SHISA7), mRNA [NM_001145176]	729956
A_23_P390443	1.1	-2.8	1.8	1.1	SHISA7	Homo sapiens shisa homolog 7 (Xenopus laevis) (SHISA7), mRNA [NM_001145176]	729956

A_23_P310492	3.6	2.6	3.5	2.3	SHISA7	Homo sapiens shisa homolog 7 (Xenopus laevis) (SHISA7), mRNA [NM_001145176]	729956
A_32_P213091	1.1	1.0	-2.5	-1.1	SHISA9	Homo sapiens shisa homolog 9 (Xenopus laevis) (SHISA9), transcript variant 2, mRNA [NM_001145205]	729993
A_24_P160868	1.0	5.2	1.7	-2.0	SHISA9	Homo sapiens shisa homolog 9 (Xenopus laevis) (SHISA9), transcript variant 2, mRNA [NM_001145205]	729993

A_32_P115515	-1.6	1.4	-4.0	-3.7	SHISA9	Homo sapiens shisa homolog 9 (Xenopus laevis) (SHISA9), transcript variant 1, mRNA [NM_001145204]	729993
A_24_P919876	1.3	2.6	2.6	2.8	SHMT1	Homo sapiens mRNA for putative 14kD protein containing SHMT homology, clone pUS1215. [Y14488]	6470
A_24_P205364	2.1	1.9	2.1	1.6	SHMT1	Homo sapiens serine hydroxymethyltransferase 1 (soluble) (SHMT1), nuclear gene encoding mitochondrial protein, transcript variant 1, mRNA [NM_004169]	6470

A_23_P169629	1.0	2.1	1.2	-1.8	SHMT2	Homo sapiens serine hydroxymethyltransferase 2 (mitochondrial) (SHMT2), nuclear gene encoding mitochondrial protein, transcript variant 1, mRNA [NM_005412]	6472
A_23_P22761	1.1	1.0	-2.1	1.0	SHOX	Homo sapiens short stature homeobox (SHOX), transcript variant 2, mRNA [NM_006883]	6473
A_23_P217698	2.6	-1.9	-2.9	1.2	SHOX	Human pseudoautosomal homeodomain-containing protein (PHOG) mRNA, complete cds. [U89331]	6473

A_23_P124384	1.2	2.0	-1.8	2.0	SHOX2	Homo sapiens short stature homeobox 2 (SHOX2), transcript variant 2, mRNA [NM_006884]	6474
A_24_P8200	6.3	6.1	6.1	6.5	SHPRH	SNF2 histone linker PHD RING helicase [Source:HGNC Symbol;Acc:19336] [ENST00000433355]	257218
A_23_P113441	5.8	1.9	1.9	1.9	SHQ1	Homo sapiens SHQ1 homolog (S. cerevisiae) (SHQ1), mRNA [NM_018130]	55164
A_23_P404902	-2.3	1.1	-1.0	-1.0	SHROOM1	Homo sapiens shroom family member 1 (SHROOM1), transcript variant 1, mRNA [NM_001172700]	134549

A_23_P156185	-5.7	-1.1	-1.6	-2.4	SHROOM1	Homo sapiens shroom family member 1 (SHROOM1), transcript variant 2, mRNA [NM_133456]	134549
A_24_P265064	-1.7	-1.9	-1.4	-4.9	SHROOM4	Homo sapiens shroom family member 4 (SHROOM4), transcript variant 1, mRNA [NM_020717]	57477
A_32_P302205	-12.8	-2.0	-1.6	-1.5	SI	Homo sapiens sucrase-isomaltase (alpha-glucosidase) (SI), mRNA [NM_001041]	6476
A_23_P106798	-3.9	2.3	-2.6	2.2	SIAH1	Homo sapiens seven in absentia homolog 1 (Drosophila) (SIAH1), transcript variant 1, mRNA [NM_003031]	6477

A_23_P69121	1.9	-1.6	3.4	1.2	SIAH2	Homo sapiens seven in absentia homolog 2 (Drosophila) (SIAH2), mRNA [NM_005067]	6478
A_23_P132515	2.2	1.1	-1.0	-1.0	SIDT1	Homo sapiens SID1 transmembrane family, member 1 (SIDT1), mRNA [NM_017699]	54847
A_23_P98402	1.1	1.1	2.6	1.0	SIDT2	Homo sapiens SID1 transmembrane family, member 2 (SIDT2), mRNA [NM_001040455]	51092

A_23_P84344	2.1	3.1	-1.1	2.2	SIGIRR	Homo sapiens single immunoglobulin and toll-interleukin 1 receptor (TIR) domain (SIGIRR), transcript variant 3, mRNA [NM_021805]	59307
A_23_P301962	2.8	1.6	1.5	1.5	SIGLEC1	Homo sapiens sialic acid binding Ig-like lectin 1, sialoadhesin (SIGLEC1), mRNA [NM_023068]	6614
A_23_P208182	-1.0	-4.2	-1.2	-1.1	SIGLEC10	Homo sapiens sialic acid binding Ig-like lectin 10 (SIGLEC10), transcript variant 1, mRNA [NM_033130]	89790

A_24_P339560	3.0	-2.5	2.8	2.6	SIGLEC11	Homo sapiens sialic acid binding Ig-like lectin 11 (SIGLEC11), transcript variant 1, mRNA [NM_052884]	114132
A_23_P50146	3.1	1.9	3.1	2.6	SIGLEC15	Homo sapiens sialic acid binding Ig-like lectin 15 (SIGLEC15), mRNA [NM_213602]	284266
A_24_P125561	1.3	1.2	-2.6	1.1	SIGLEC9	Homo sapiens sialic acid binding Ig-like lectin 9 (SIGLEC9), transcript variant 2, mRNA [NM_014441]	27180
A_24_P347067	3.8	4.0	2.0	2.0	SIK3	Homo sapiens SIK family kinase 3 (SIK3), mRNA [NM_025164]	23387

A_23_P85990	1.4	2.4	1.3	1.0	SIKE1	Homo sapiens suppressor of IKBKE 1 (SIKE1), transcript variant 2, mRNA [NM_025073]	80143
A_24_P937855	1.0	-1.0	-3.1	-2.7	SIKE1	Homo sapiens suppressor of IKBKE 1 (SIKE1), transcript variant 1, mRNA [NM_001102396]	80143
A_23_P252903	-1.1	-1.3	-1.3	-5.0	SIL1	Homo sapiens SIL1 homolog, endoplasmic reticulum chaperone (S. cerevisiae) (SIL1), transcript variant 1, mRNA [NM_001037633]	64374
A_24_P344053	1.0	-3.3	1.0	1.0	SIPA1L1	Homo sapiens signal-induced proliferation-associated 1 like 1 (SIPA1L1), mRNA [NM_015556]	26037

A_24_P24343	1.3	1.2	-2.9	1.4	SIPA1L3	Homo sapiens signal-induced proliferation-associated 1 like 3 (SIPA1L3), mRNA [NM_015073]	23094
A_24_P259083	1.5	-1.2	-2.6	-1.1	SIRPA	Homo sapiens signal-regulatory protein alpha (SIRPA), transcript variant 1, mRNA [NM_001040022]	140885
A_23_P17456	-2.7	-2.0	-5.5	-1.9	SIRPB1	Homo sapiens signal-regulatory protein beta 1 (SIRPB1), transcript variant 1, mRNA [NM_006065]	10326
A_24_P202840	2.1	2.1	3.9	-1.2	SIRPB2	Homo sapiens signal-regulatory protein beta 2 (SIRPB2), transcript variant 1, mRNA [NM_001122962]	284759

A_23_P406521	12.4	8.3	3.2	3.9	SIRPD	Homo sapiens signal-regulatory protein delta (SIRPD), mRNA [NM_178460]	128646
A_23_P142455	2.4	-2.2	1.7	-1.1	SIRT2	Homo sapiens sirtuin 2 (SIRT2), transcript variant 1, mRNA [NM_012237]	22933
A_23_P139265	-5.7	1.7	-3.9	-1.8	SIRT3	Homo sapiens sirtuin 3 (SIRT3), nuclear gene encoding mitochondrial protein, transcript variant 1, mRNA [NM_012239]	23410

A_24_P923684	-1.5	6.4	3.4	6.6	SIRT3	Homo sapiens sirtuin 3 (SIRT3), nuclear gene encoding mitochondrial protein, transcript variant 1, mRNA [NM_012239]	23410
A_23_P99226	1.3	1.2	1.1	-4.1	SIRT4	Homo sapiens sirtuin 4 (SIRT4), mRNA [NM_012240]	23409
A_23_P208847	2.3	1.9	1.7	-1.2	SIRT6	Homo sapiens sirtuin 6 (SIRT6), transcript variant 1, mRNA [NM_016539]	51548
A_23_P66813	2.6	1.6	1.9	1.5	SIRT7	Homo sapiens sirtuin 7 (SIRT7), mRNA [NM_016538]	51547
A_23_P28120	-2.4	-1.1	1.9	-1.2	SIX2	Homo sapiens SIX homeobox 2 (SIX2), mRNA [NM_016932]	10736

A_23_P326885	-2.0	-1.8	-4.0	-2.9	SIX4	Homo sapiens SIX homeobox 4 (SIX4), mRNA [NM_017420]	51804
A_32_P55860	-1.2	-1.2	-1.3	-8.9	SKA2	Homo sapiens spindle and kinetochore associated complex subunit 2 (SKA2), transcript variant 1, mRNA [NM_182620]	348235
A_23_P340909	1.1	1.2	1.1	-4.3	SKA3	Homo sapiens chromosome 13 open reading frame 3, mRNA (cDNA clone MGC:4832 IMAGE:3604003), complete cds. [BC013418]	221150

A_23_P100730	-1.1	-1.1	-3.1	-1.1	SKAP1	Homo sapiens src kinase associated phosphoprotein 1 (SKAP1), transcript variant 1, mRNA [NM_003726]	8631
A_32_P63086	1.1	1.1	1.0	-4.6	SKIL	Homo sapiens SKI-like oncogene (SKIL), transcript variant 1, mRNA [NM_005414]	6498
A_23_P168080	2.2	1.6	2.1	1.2	SKIV2L	Homo sapiens superkiller viralicidic activity 2-like (S. cerevisiae) (SKIV2L), mRNA [NM_006929]	6499
A_23_P348146	-3.3	-3.8	-1.0	-1.0	SLAIN1	Homo sapiens SLAIN motif family, member 1 (SLAIN1), transcript variant 1, mRNA [NM_001040153]	122060

A_32_P808	-1.0	-9.9	-1.0	1.3	SLAIN2	Homo sapiens SLAIN motif family, member 2 (SLAIN2), mRNA [NM_020846]	57606
A_23_P366254	-1.0	2.3	2.1	1.0	SLC10A3	Homo sapiens solute carrier family 10 (sodium/bile acid cotransporter family), member 3 (SLC10A3), transcript variant 1, mRNA [NM_019848]	8273
A_23_P39665	4.4	6.1	6.9	4.3	SLC11A1	Homo sapiens solute carrier family 11 (proton-coupled divalent metal ion transporters), member 1 (SLC11A1), mRNA [NM_000578]	6556

A_24_P76831	-1.2	-1.3	-1.3	-12.0	SLC11A1	Homo sapiens solute carrier family 11 (proton-coupled divalent metal ion transporters), member 1 (SLC11A1), mRNA [NM_000578]	6556
A_24_P913210	-4.9	-2.7	-3.2	-5.5	SLC11A2	Homo sapiens solute carrier family 11 (proton-coupled divalent metal ion transporters), member 2 (SLC11A2), transcript variant 2, mRNA [NM_001174126]	4891

A_23_P99879	-1.0	1.2	-1.1	-2.2	SLC12A1	Homo sapiens solute carrier family 12 (sodium/potassium/chloride transporters), member 1 (SLC12A1), transcript variant 1, mRNA [NM_000338]	6557
A_23_P77653	-1.0	-2.2	-1.1	1.5	SLC12A3	Homo sapiens solute carrier family 12 (sodium/chloride transporters), member 3 (SLC12A3), transcript variant 1, mRNA [NM_000339]	6559

A_23_P61688	1.3	-2.1	1.1	1.2	SLC12A7	Homo sapiens solute carrier family 12 (potassium/chloride transporters), member 7 (SLC12A7), mRNA [NM_006598]	10723
A_23_P215233	2.1	1.7	1.6	1.5	SLC12A9	Homo sapiens solute carrier family 12 (potassium/chloride transporters), member 9 (SLC12A9), mRNA [NM_020246]	56996
A_23_P71226	-7.6	-1.4	-2.1	-3.4	SLC13A1	Homo sapiens solute carrier family 13 (sodium/sulfate symporters), member 1 (SLC13A1), mRNA [NM_022444]	6561

A_24_P355057	-1.0	-9.0	-3.0	-1.1	SLC13A1	Homo sapiens solute carrier family 13 (sodium/sulfate symporters), member 1 (SLC13A1), mRNA [NM_022444]	6561
A_23_P107307	-2.7	1.1	-1.4	-3.3	SLC13A2	Homo sapiens solute carrier family 13 (sodium-dependent dicarboxylate transporter), member 2 (SLC13A2), transcript variant 2, mRNA [NM_003984]	9058
A_23_P19778	1.0	1.0	-4.4	1.7	SLC13A4	Homo sapiens solute carrier family 13 (sodium/sulfate symporters), member 4 (SLC13A4), mRNA [NM_012450]	26266

A_23_P38757	-2.7	-4.3	-4.1	-4.9	SLC14A1	Human RACH1 (RACH1) mRNA, complete cds. [U35735]	6563
A_24_P926507	1.1	-1.0	-2.1	-2.8	SLC14A1	Homo sapiens solute carrier family 14 (urea transporter), member 1 (Kidd blood group) (SLC14A1), transcript variant 4, mRNA [NM_001146037]	6563
A_24_P136471	22.3	13.9	22.6	22.1	SLC14A2	solute carrier family 14 (urea transporter), member 2 [Source:HGNC Symbol;Acc:10919] [ENST00000323329]	8170

A_23_P128609	-2.0	-1.1	-1.9	-2.4	SLC15A1	Homo sapiens solute carrier family 15 (oligopeptide transporter), member 1 (SLC15A1), mRNA [NM_005073]	6564
A_24_P8165	1.1	1.1	3.8	-1.1	SLC15A1	solute carrier family 15 (oligopeptide transporter), member 1 [Source:HGNC Symbol;Acc:10920] [ENST00000376494]	6564
A_23_P126825	1.4	3.2	1.3	-1.0	SLC16A1	Homo sapiens solute carrier family 16, member 1 (monocarboxylic acid transporter 1) (SLC16A1), transcript variant 1, mRNA [NM_003051]	6566

A_23_P363313	1.1	2.1	-1.8	-2.6	SLC16A11	Homo sapiens solute carrier family 16, member 11 (monocarboxylic acid transporter 11) (SLC16A11), mRNA [NM_153357]	162515
A_23_P118741	-6.3	1.0	-1.0	-1.0	SLC16A13	Homo sapiens solute carrier family 16, member 13 (monocarboxylic acid transporter 13) (SLC16A13), mRNA [NM_201566]	201232
A_23_P131394	-4.0	1.0	-4.7	1.0	SLC16A14	Homo sapiens solute carrier family 16, member 14 (monocarboxylic acid transporter 14) (SLC16A14), mRNA [NM_152527]	151473

A_24_P342829	-1.0	-4.1	-8.3	-1.0	SLC16A14	Homo sapiens solute carrier family 16, member 14 (monocarboxylic acid transporter 14) (SLC16A14), mRNA [NM_152527]	151473
A_23_P137097	1.1	1.1	1.2	2.1	SLC16A2	Homo sapiens solute carrier family 16, member 2 (monocarboxylic acid transporter 8) (SLC16A2), mRNA [NM_006517]	6567
A_23_P117059	-1.0	-2.8	-1.1	-1.1	SLC16A7	Homo sapiens solute carrier family 16, member 7 (monocarboxylic acid transporter 2) (SLC16A7), mRNA [NM_004731]	9194

A_32_P130630	-1.9	-1.9	-2.7	-1.3	SLC16A7	Homo sapiens solute carrier family 16, member 7 (monocarboxylic acid transporter 2) (SLC16A7), mRNA [NM_004731]	9194
A_23_P115726	1.1	1.3	1.1	-2.1	SLC16A9	Homo sapiens solute carrier family 16, member 9 (monocarboxylic acid transporter 9) (SLC16A9), mRNA [NM_194298]	220963
A_23_P357270	-1.0	-1.1	1.4	-2.4	SLC17A4	Homo sapiens solute carrier family 17 (sodium phosphate), member 4 (SLC17A4), mRNA [NM_005495]	10050

A_23_P78750	-1.6	-3.0	-3.1	-1.7	SLC17A7	Homo sapiens solute carrier family 17 (sodium-dependent inorganic phosphate cotransporter), member 7 (SLC17A7), mRNA [NM_020309]	57030
A_23_P210361	1.1	1.3	-2.3	-1.1	SLC17A9	Homo sapiens solute carrier family 17, member 9 (SLC17A9), mRNA [NM_022082]	63910
A_23_P52499	-2.1	1.2	-1.2	-1.7	SLC18A2	Homo sapiens solute carrier family 18 (vesicular monoamine), member 2 (SLC18A2), mRNA [NM_003054]	6571

A_23_P52362	-2.9	-1.6	-1.6	-1.7	SLC18A3	Homo sapiens solute carrier family 18 (vesicular acetylcholine), member 3 (SLC18A3), mRNA [NM_003055]	6572
A_23_P39871	-4.5	1.1	2.5	-1.5	SLC19A3	Homo sapiens solute carrier family 19, member 3 (SLC19A3), mRNA [NM_025243]	80704
A_24_P232252	-3.3	-1.1	-1.0	1.4	SLC1A1	Homo sapiens solute carrier family 1 (neuronal/epithelial high affinity glutamate transporter, system Xag), member 1 (SLC1A1), mRNA [NM_004170]	6505

A_24_P350759	1.1	-2.1	-1.0	-1.9	SLC1A2	Homo sapiens solute carrier family 1 (glial high affinity glutamate transporter), member 2 (SLC1A2), transcript variant 1, mRNA [NM_004171]	6506
A_23_P162068	-1.0	-1.0	-2.9	-1.0	SLC1A2	Homo sapiens solute carrier family 1 (glial high affinity glutamate transporter), member 2 (SLC1A2), transcript variant 1, mRNA [NM_004171]	6506

A_24_P286114	-2.7	-1.7	-6.1	-1.3	SLC1A3	Homo sapiens solute carrier family 1 (glial high affinity glutamate transporter), member 3 (SLC1A3), transcript variant 1, mRNA [NM_004172]	6507
A_23_P91151	1.6	1.1	2.3	1.7	SLC1A4	Homo sapiens solute carrier family 1 (glutamate/neutral amino acid transporter), member 4 (SLC1A4), transcript variant 1, mRNA [NM_003038]	6509

A_24_P191312	1.0	-1.0	9.8	-1.1	SLC1A4	Homo sapiens solute carrier family 1 (glutamate/neutral amino acid transporter), member 4 (SLC1A4), transcript variant 1, mRNA [NM_003038]	6509
A_23_P55998	1.4	1.3	2.2	1.6	SLC1A5	Homo sapiens solute carrier family 1 (neutral amino acid transporter), member 5 (SLC1A5), transcript variant 1, mRNA [NM_005628]	6510

A_23_P325562	1.2	1.2	-1.4	-2.3	SLC1A7	Homo sapiens solute carrier family 1 (glutamate transporter), member 7 (SLC1A7), mRNA [NM_006671]	6512
A_24_P103004	2.6	-1.0	-1.0	-1.0	SLC20A1	Homo sapiens solute carrier family 20 (phosphate transporter), member 1 (SLC20A1), mRNA [NM_005415]	6574
A_23_P132730	2.2	-2.6	2.4	-2.3	SLC22A14	Homo sapiens solute carrier family 22, member 14 (SLC22A14), mRNA [NM_004803]	9389

A_23_P388900	5.6	5.6	7.0	4.6	SLC22A15	Homo sapiens solute carrier family 22, member 15 (SLC22A15), mRNA [NM_018420]	55356
A_24_P919084	2.5	6.9	1.9	7.4	SLC22A16	solute carrier family 22 (organic cation/carnitine transporter), member 16 [Source:HGNC Symbol;Acc:20302] [ENST00000461487]	85413
A_24_P330385	-1.7	-1.2	-1.8	-2.7	SLC22A17	Homo sapiens solute carrier family 22, member 17 (SLC22A17), transcript variant 2, mRNA [NM_016609]	51310

A_24_P372643	1.5	1.4	1.8	3.5	SLC22A18AS	Homo sapiens solute carrier family 22 (organic cation transporter), member 18 antisense (SLC22A18AS), mRNA [NM_007105]	5003
A_24_P252223	1.3	18.3	-1.9	-1.3	SLC22A23	Homo sapiens solute carrier family 22, member 23 (SLC22A23), transcript variant 2, mRNA [NM_021945]	63027
A_24_P350228	1.8	-1.4	-2.2	1.0	SLC22A23	Homo sapiens solute carrier family 22, member 23 (SLC22A23), transcript variant 2, mRNA [NM_021945]	63027

A_23_P7882	-1.0	-2.6	2.7	-1.0	SLC22A23	Homo sapiens solute carrier family 22, member 23 (SLC22A23), transcript variant 1, mRNA [NM_015482]	63027
A_23_P19733	-1.1	1.6	-1.3	-2.2	SLC22A3	Homo sapiens solute carrier family 22 (extraneuronal monoamine transporter), member 3 (SLC22A3), mRNA [NM_021977]	6581
A_23_P156180	-2.3	-1.0	1.1	-2.3	SLC22A4	Homo sapiens solute carrier family 22 (organic cation/ergothioneine transporter), member 4 (SLC22A4), mRNA [NM_003059]	6583

A_23_P93217	1.9	-1.9	2.5	-1.2	SLC22A7	Homo sapiens solute carrier family 22 (organic anion transporter), member 7 (SLC22A7), transcript variant 2, mRNA [NM_153320]	10864
A_24_P330021	-1.9	-1.8	-2.0	-2.1	SLC22A9	Homo sapiens solute carrier family 22 (organic anion transporter), member 9 (SLC22A9), mRNA [NM_080866]	114571
A_23_P6085	1.1	2.1	1.7	1.5	SLC23A2	Homo sapiens solute carrier family 23 (nucleobase transporters), member 2 (SLC23A2), transcript variant 2, mRNA [NM_203327]	9962

A_23_P112173	1.0	-1.0	-9.0	1.0	SLC24A2	Homo sapiens solute carrier family 24 (sodium/potassium/calcium exchanger), member 2 (SLC24A2), transcript variant 1, mRNA [NM_020344]	25769
A_24_P67350	-3.2	1.0	-1.0	-3.4	SLC24A3	Homo sapiens solute carrier family 24 (sodium/potassium/calcium exchanger), member 3 (SLC24A3), mRNA [NM_020689]	57419

A_23_P120776	1.3	1.8	2.1	1.1	SLC25A1	Homo sapiens solute carrier family 25 (mitochondrial carrier; citrate transporter), member 1 (SLC25A1), nuclear gene encoding mitochondrial protein, transcript variant 1, mRNA [NM_005984]	6576

A_23_P38497	2.1	1.3	1.6	-1.0	SLC25A11	Homo sapiens solute carrier family 25 (mitochondrial carrier; oxoglutarate carrier), member 11 (SLC25A11), nuclear gene encoding mitochondrial protein, transcript variant 1, mRNA [NM_003562]	8402
A_32_P157402	-1.5	-3.4	-2.1	-1.0	SLC25A13	Homo sapiens solute carrier family 25, member 13 (citrin) (SLC25A13), nuclear gene encoding mitochondrial protein, transcript variant 2, mRNA [NM_014251]	10165

A_32_P224522	1.0	-1.0	-1.1	-2.2	SLC25A23	Homo sapiens solute carrier family 25 (mitochondrial carrier; phosphate carrier), member 23 (SLC25A23), nuclear gene encoding mitochondrial protein, mRNA [NM_024103]	79085
A_24_P368544	1.4	1.4	-2.4	1.0	SLC25A26	Homo sapiens solute carrier family 25, member 26 (SLC25A26), nuclear gene encoding mitochondrial protein, transcript variant 1, mRNA [NM_173471]	115286

A_23_P169879	1.6	2.2	1.1	-1.1	SLC25A26	Homo sapiens solute carrier family 25, member 26 (SLC25A26), nuclear gene encoding mitochondrial protein, transcript variant 1, mRNA [NM_173471]	115286
A_24_P69439	4.8	1.6	3.5	1.7	SLC25A32	Homo sapiens solute carrier family 25, member 32 (SLC25A32), nuclear gene encoding mitochondrial protein, mRNA [NM_030780]	81034

A_23_P408455	1.3	1.3	1.2	-2.5	SLC25A36	Homo sapiens solute carrier family 25, member 36 (SLC25A36), transcript variant 1, mRNA [NM_001104647]	55186
A_24_P201089	-1.0	1.0	1.0	-9.2	SLC25A37	Homo sapiens solute carrier family 25, member 37 (SLC25A37), nuclear gene encoding mitochondrial protein, mRNA [NM_016612]	51312

A_23_P71117	2.8	-1.2	2.4	-1.2	SLC25A40	Homo sapiens solute carrier family 25, member 40 (SLC25A40), nuclear gene encoding mitochondrial protein, mRNA [NM_018843]	55972
A_23_P432626	1.2	-5.4	1.2	1.3	SLC25A40	Homo sapiens solute carrier family 25, member 40 (SLC25A40), nuclear gene encoding mitochondrial protein, mRNA [NM_018843]	55972
A_23_P436526	1.5	1.8	1.7	2.5	SLC25A42	Homo sapiens solute carrier family 25, member 42 (SLC25A42), mRNA [NM_178526]	284439

A_24_P34100	3.3	3.1	3.5	3.0	SLC25A43	Homo sapiens solute carrier family 25, member 43 (SLC25A43), nuclear gene encoding mitochondrial protein, mRNA [NM_145305]	203427
A_23_P64070	2.8	-1.5	-2.0	-3.5	SLC25A45	Homo sapiens solute carrier family 25, member 45 (SLC25A45), transcript variant 1, mRNA [NM_182556]	283130
A_23_P422511	1.4	1.2	1.9	2.2	SLC25A45	Homo sapiens solute carrier family 25, member 45 (SLC25A45), transcript variant 1, mRNA [NM_182556]	283130

A_23_P133375	-1.0	-1.0	-1.0	-10.1	SLC25A46	Homo sapiens solute carrier family 25, member 46 (SLC25A46), mRNA [NM_138773]	91137
A_23_P7719	-1.2	-1.2	-1.2	-3.1	SLC25A48	solute carrier family 25, member 48 [Source:HGNC Symbol;Acc:30451] [ENST00000420621]	153328
A_23_P33752	2.4	3.6	1.9	3.4	SLC25A6	Homo sapiens solute carrier family 25 (mitochondrial carrier; adenine nucleotide translocator), member 6 (SLC25A6), nuclear gene encoding mitochondrial protein, mRNA [NM_001636]	293

A_24_P37665	5.2	4.2	3.0	1.1	SLC26A1	Homo sapiens solute carrier family 26 (sulfate transporte r), member 1 (SLC26A1), transcript variant 1, mRNA [NM_0220 42]	10861
A_23_P80954	-4.5	-9.5	-6.9	-2.6	SLC26A1	Homo sapiens solute carrier family 26 (sulfate transporte r), member 1 (SLC26A1), transcript variant 1, mRNA [NM_0220 42]	10861
A_23_P116642	-1.0	-18.9	-1.0	-1.0	SLC26A10	Homo sapiens solute carrier family 26, member 10 (SLC26A10), mRNA [NM_1334 89]	65012

A_23_P203807	-2.4	1.4	-2.5	-1.6	SLC26A10	solute carrier family 26, member 10 [Source:HGNC Symbol;Acc:14470] [ENST00000440686]	65012
A_23_P109895	-1.2	-1.1	-1.1	-2.3	SLC26A6	Homo sapiens solute carrier family 26, member 6 (SLC26A6), transcript variant 4, mRNA [NM_001040454]	65010
A_24_P7974	1.0	1.0	-2.5	-1.2	SLC26A6	Homo sapiens HSPC106 mRNA, partial cds. [AF161369]	65010
A_23_P30950	1.1	-2.9	-1.0	1.1	SLC26A8	Homo sapiens solute carrier family 26, member 8 (SLC26A8), transcript variant 1, mRNA [NM_052961]	116369

A_23_P140450	-1.3	-2.3	-3.2	-1.5	SLC27A2	Homo sapiens solute carrier family 27 (fatty acid transporter), member 2 (SLC27A2), transcript variant 1, mRNA [NM_003645]	11001
A_24_P257971	-4.2	1.5	1.3	6.0	SLC27A4	Homo sapiens solute carrier family 27 (fatty acid transporter), member 4 (SLC27A4), mRNA [NM_005094]	10999
A_23_P14667	-1.2	-1.4	1.1	-2.2	SLC28A1	Homo sapiens solute carrier family 28 (sodium-coupled nucleoside transporter), member 1 (SLC28A1), transcript variant 1, mRNA [NM_004213]	9154

A_23_P32078	-1.7	-4.3	-1.1	-1.1	SLC28A3	Homo sapiens solute carrier family 28 (sodium-coupled nucleoside transporter), member 3 (SLC28A3), transcript variant 2, mRNA [NM_022127]	64078
A_32_P494169	-2.2	-1.0	-1.0	1.1	SLC28A3	Homo sapiens solute carrier family 28 (sodium-coupled nucleoside transporter), member 3 (SLC28A3), transcript variant 1, mRNA [NM_001199633]	64078

A_23_P98294	1.8	2.6	1.1	2.5	SLC29A2	Homo sapiens solute carrier family 29 (nucleoside transporters), member 2 (SLC29A2), mRNA [NM_001532]	3177
A_24_P911787	5.7	2.7	2.1	2.8	SLC29A3	Homo sapiens solute carrier family 29 (nucleoside transporters), member 3 (SLC29A3), transcript variant 1, mRNA [NM_018344]	55315
A_23_P168551	1.6	-4.7	1.6	-1.0	SLC29A4	Homo sapiens solute carrier family 29 (nucleoside transporters), member 4 (SLC29A4), transcript variant 1, mRNA [NM_001040661]	222962

A_23_P571	2.3	1.2	-1.3	-1.1	SLC2A1	Homo sapiens solute carrier family 2 (facilitated glucose transporter), member 1 (SLC2A1), mRNA [NM_006516]	6513
A_32_P174130	1.3	-1.6	-2.7	1.1	SLC2A1	solute carrier family 2 (facilitated glucose transporter), member 1 [Source:HGNC Symbol;Acc:11005] [ENST00000460369]	6513
A_23_P210521	1.0	1.0	-1.3	-4.4	SLC2A10	Homo sapiens solute carrier family 2 (facilitated glucose transporter), member 10 (SLC2A10), mRNA [NM_030777]	81031

A_23_P10211	1.5	1.7	3.5	-1.1	SLC2A13	Homo sapiens solute carrier family 2 (facilitated glucose transporter), member 13 (SLC2A13), mRNA [NM_052885]	114134
A_23_P132569	2.0	2.0	2.0	2.6	SLC2A2	Homo sapiens solute carrier family 2 (facilitated glucose transporter), member 2 (SLC2A2), mRNA [NM_000340]	6514
A_23_P107350	-2.5	-1.3	-1.4	-11.6	SLC2A4	Homo sapiens solute carrier family 2 (facilitated glucose transporter), member 4 (SLC2A4), mRNA [NM_001042]	6517

A_23_P102575	-1.3	1.0	-2.9	-3.2	SLC2A4RG	Homo sapiens SLC2A4 regulator (SLC2A4RG), mRNA [NM_020062]	56731
A_23_P169249	1.0	-3.3	-1.2	1.1	SLC2A6	Homo sapiens solute carrier family 2 (facilitated glucose transporter), member 6 (SLC2A6), transcript variant 1, mRNA [NM_017585]	11182
A_24_P196318	-1.1	1.0	-4.7	-1.7	SLC2A9	Homo sapiens solute carrier family 2 (facilitated glucose transporter), member 9 (SLC2A9), transcript variant 2, mRNA [NM_001001290]	56606

A_24_P156748	2.9	4.3	2.7	2.4	SLC30A2	Homo sapiens solute carrier family 30 (zinc transporter), member 2 (SLC30A2), transcript variant 1, mRNA [NM_001004434]	7780
A_23_P302568	2.7	-1.4	-1.4	2.2	SLC30A3	Homo sapiens solute carrier family 30 (zinc transporter), member 3 (SLC30A3), mRNA [NM_003459]	7781
A_32_P227870	1.0	-6.4	-1.1	-1.0	SLC30A4	solute carrier family 30 (zinc transporter), member 4 [Source:HGNC Symbol;Acc:11015] [ENST00000261867]	7782

A_23_P117679	-1.6	-3.5	-1.0	-2.8	SLC30A4	Homo sapiens solute carrier family 30 (zinc transporter), member 4 (SLC30A4), mRNA [NM_013309]	7782
A_23_P160103	-1.3	-1.9	-1.9	-6.7	SLC30A5	Homo sapiens solute carrier family 30 (zinc transporter), member 5 (SLC30A5), transcript variant 1, mRNA [NM_022902]	64924
A_23_P22915	-2.5	1.0	1.1	1.0	SLC30A7	Homo sapiens solute carrier family 30 (zinc transporter), member 7 (SLC30A7), transcript variant 1, mRNA [NM_133496]	148867

A_24_P321068	-1.3	-1.9	-2.2	-1.3	SLC31A1	Homo sapiens solute carrier family 31 (copper transporters), member 1 (SLC31A1), mRNA [NM_001859]	1317
A_23_P58729	2.3	-1.8	2.8	1.5	SLC34A1	Homo sapiens solute carrier family 34 (sodium phosphate), member 1 (SLC34A1), transcript variant 1, mRNA [NM_003052]	6569
A_23_P121646	1.7	1.4	1.4	-2.7	SLC34A2	Homo sapiens solute carrier family 34 (sodium phosphate), member 2 (SLC34A2), transcript variant 1, mRNA [NM_006424]	10568

A_23_P113682	3.1	2.5	1.8	1.8	SLC34A3	Homo sapiens solute carrier family 34 (sodium phosphate), member 3 (SLC34A3), transcript variant 3, mRNA [NM_080877]	142680
A_24_P406034	5.7	1.9	5.2	5.2	SLC35A1	Homo sapiens solute carrier family 35 (CMP-sialic acid transporter), member A1 (SLC35A1), transcript variant 1, mRNA [NM_006416]	10559

A_23_P58912	2.7	-1.0	-1.1	1.6	SLC35A1	Homo sapiens solute carrier family 35 (CMP-sialic acid transporter), member A1 (SLC35A1), transcript variant 1, mRNA [NM_006416]	10559
A_24_P310616	1.1	1.0	-1.7	-3.1	SLC35A2	Homo sapiens solute carrier family 35 (UDP-galactose transporter), member A2 (SLC35A2), transcript variant 3, mRNA [NM_001042498]	7355
A_24_P367561	1.7	2.3	1.7	1.7	SLC35B4	Homo sapiens solute carrier family 35, member B4 (SLC35B4), mRNA [NM_032826]	84912

A_23_P68452	1.4	-1.3	-2.1	1.6	SLC35C2	Homo sapiens solute carrier family 35, member C2 (SLC35C2), transcript variant 1, mRNA [NM_173179]	51006
A_24_P392970	1.1	-11.0	1.1	1.1	SLC35D3	Homo sapiens solute carrier family 35, member D3 (SLC35D3), mRNA [NM_001008783]	340146
A_24_P378788	-9.1	-1.3	-4.6	-3.2	SLC35E1	Homo sapiens solute carrier family 35, member E1 (SLC35E1), mRNA [NM_024881]	79939
A_23_P339098	-2.3	-1.1	1.3	1.8	SLC35F2	Homo sapiens solute carrier family 35, member F2 (SLC35F2), mRNA [NM_017515]	54733

A_23_P59988	1.0	2.4	-1.0	1.0	SLC35G5	Homo sapiens solute carrier family 35, member G5 (SLC35G5), mRNA [NM_054028]	83650
A_23_P17695	2.7	2.6	2.5	2.5	SLC37A1	Homo sapiens solute carrier family 37 (glycerol-3-phosphate transporter), member 1 (SLC37A1), mRNA [NM_018964]	54020
A_23_P86931	1.5	2.2	1.2	1.2	SLC37A2	Homo sapiens solute carrier family 37 (glycerol-3-phosphate transporter), member 2 (SLC37A2), transcript variant 1, mRNA [NM_198277]	219855

A_23_P158449	1.0	1.0	-9.2	-1.1	SLC37A3	Homo sapiens solute carrier family 37 (glycerol-3-phosphate transporter), member 3 (SLC37A3), transcript variant 2, mRNA [NM_032295]	84255
A_24_P261734	1.1	1.1	-23.0	-3.5	SLC38A1	Homo sapiens solute carrier family 38, member 1 (SLC38A1), transcript variant 1, mRNA [NM_030674]	81539
A_23_P379630	1.8	3.1	3.2	1.3	SLC38A10	Homo sapiens solute carrier family 38, member 10 (SLC38A10), transcript variant 2, mRNA [NM_138570]	124565

A_23_P218079	1.3	2.9	3.9	2.9	SLC38A2	Homo sapiens solute carrier family 38, member 2 (SLC38A2), mRNA [NM_018976]	54407
A_23_P155487	3.3	1.2	3.0	2.4	SLC38A3	Homo sapiens solute carrier family 38, member 3 (SLC38A3), mRNA [NM_006841]	10991
A_24_P321581	1.1	-2.1	-2.2	1.1	SLC38A4	Homo sapiens solute carrier family 38, member 4 (SLC38A4), transcript variant 1, mRNA [NM_018018]	55089
A_23_P65967	-1.4	1.5	4.0	2.5	SLC38A7	Homo sapiens solute carrier family 38, member 7 (SLC38A7), mRNA [NM_018231]	55238

A_23_P24149	2.2	3.8	2.2	1.9	SLC39A12	Homo sapiens solute carrier family 39 (zinc transporter), member 12 (SLC39A12), transcript variant 2, mRNA [NM_152725]	221074
A_24_P333479	2.2	2.1	2.0	2.0	SLC39A14	Homo sapiens solute carrier family 39 (zinc transporter), member 14 (SLC39A14), transcript variant 2, mRNA [NM_015359]	23516

A_24_P160460	1.7	2.1	-2.7	1.2	SLC39A3	Homo sapiens solute carrier family 39 (zinc transporter), member 3 (SLC39A3), transcript variant 1, mRNA [NM_144564]	29985
A_24_P382467	-1.1	-2.6	-2.2	-2.2	SLC39A3	Homo sapiens solute carrier family 39 (zinc transporter), member 3 (SLC39A3), transcript variant 1, mRNA [NM_144564]	29985
A_23_P388170	2.1	1.0	1.7	-1.9	SLC39A3	Homo sapiens solute carrier family 39 (zinc transporter), member 3 (SLC39A3), transcript variant 2, mRNA [NM_213568]	29985

A_23_P20502	5.2	1.6	-1.0	1.3	SLC39A4	Homo sapiens solute carrier family 39 (zinc transporter), member 4 (SLC39A4), transcript variant 1, mRNA [NM_017767]	55630
A_24_P204954	1.7	2.2	1.3	1.3	SLC39A4	Homo sapiens cDNA FLJ32338 fis, clone PROST2005919. [AK056900]	55630
A_23_P41424	1.1	-5.7	-1.1	-1.0	SLC39A8	Homo sapiens solute carrier family 39 (zinc transporter), member 8 (SLC39A8), transcript variant 1, mRNA [NM_022154]	64116

A_24_P217234	-1.0	-1.0	-12.2	-1.0	SLC3A1	Homo sapiens solute carrier family 3 (cystine, dibasic and neutral amino acid transporters, activator of cystine, dibasic and neutral amino acid transport), member 1 (SLC3A1), mRNA [NM_000341]	6519
A_23_P87216	-2.6	-2.0	-3.3	-1.6	SLC3A2	Homo sapiens solute carrier family 3 (activators of dibasic and neutral amino acid transport), member 2 (SLC3A2), transcript variant 2, mRNA [NM_001012662]	6520

A_23_P75811	-3.5	1.6	1.0	1.0	SLC3A2	Homo sapiens solute carrier family 3 (activators of dibasic and neutral amino acid transport), member 2 (SLC3A2), transcript variant 2, mRNA [NM_001012662]	6520
A_23_P102391	-1.0	-1.0	-4.3	-1.0	SLC40A1	Homo sapiens solute carrier family 40 (iron-regulated transporter), member 1 (SLC40A1), mRNA [NM_014585]	30061
A_23_P204801	2.9	2.8	2.7	2.9	SLC41A2	Homo sapiens solute carrier family 41, member 2 (SLC41A2), mRNA [NM_032148]	84102

A_24_P78862	-3.1	-2.4	-6.5	-1.3	SLC43A2	Homo sapiens solute carrier family 43, member 2 (SLC43A2), mRNA [NM_152346]	124935
A_24_P296508	2.3	1.5	2.4	2.3	SLC43A2	Homo sapiens solute carrier family 43, member 2 (SLC43A2), mRNA [NM_152346]	124935
A_24_P406986	1.9	3.4	2.2	-1.5	SLC43A3	Homo sapiens solute carrier family 43, member 3 (SLC43A3), mRNA [NM_199329]	29015
A_23_P411806	2.9	4.5	3.7	1.8	SLC44A1	Homo sapiens solute carrier family 44, member 1 (SLC44A1), mRNA [NM_080546]	23446

A_23_P208340	-1.3	1.2	1.2	-2.0	SLC44A2	Homo sapiens solute carrier family 44, member 2 (SLC44A2), transcript variant 1, mRNA [NM_020428]	57153
A_23_P93349	-1.0	-16.0	-1.1	-1.0	SLC44A4	Homo sapiens solute carrier family 44, member 4 (SLC44A4), transcript variant 1, mRNA [NM_025257]	80736
A_24_P159548	2.1	1.8	-1.6	1.3	SLC46A1	Homo sapiens solute carrier family 46 (folate transporter), member 1 (SLC46A1), transcript variant 1, mRNA [NM_080669]	113235

A_23_P105856	-4.2	-1.1	-1.2	-1.2	SLC46A3	Homo sapiens solute carrier family 46, member 3 (SLC46A3), transcript variant 1, mRNA [NM_181785]	283537
A_23_P207221	9.6	6.0	10.1	1.1	SLC47A1	Homo sapiens solute carrier family 47, member 1 (SLC47A1), mRNA [NM_018242]	55244
A_24_P142503	3.2	3.2	3.3	1.6	SLC47A1	Homo sapiens solute carrier family 47, member 1 (SLC47A1), mRNA [NM_018242]	55244
A_23_P77908	1.0	-3.2	-18.3	-1.0	SLC47A2	Homo sapiens solute carrier family 47, member 2 (SLC47A2), transcript variant 1, mRNA [NM_152908]	146802

A_23_P204511	1.3	-1.1	2.0	1.3	SLC48A1	Homo sapiens cDNA FLJ20489 fis, clone KAT08285. [AK000496]	55652
A_24_P930111	2.6	2.5	1.3	-1.1	SLC4A10	Homo sapiens solute carrier family 4, sodium bicarbonate transporter, member 10 (SLC4A10), transcript variant 2, mRNA [NM_022058]	57282
A_23_P56810	1.2	2.2	-2.7	-1.8	SLC4A1AP	Homo sapiens solute carrier family 4 (anion exchanger), member 1, adaptor protein (SLC4A1AP), mRNA [NM_018158]	22950

A_23_P39647	-8.4	-1.7	-3.7	-1.7	SLC4A3	Homo sapiens solute carrier family 4, anion exchanger, member 3 (SLC4A3), transcript variant 1, mRNA [NM_005070]	6508
A_32_P349145	1.5	1.5	-2.4	-1.6	SLC4A4	Homo sapiens solute carrier family 4, sodium bicarbonate cotransporter, member 4 (SLC4A4), transcript variant 2, mRNA [NM_003759]	8671
A_23_P132468	1.2	1.1	-1.2	-3.4	SLC4A7	Homo sapiens solute carrier family 4, sodium bicarbonate cotransporter, member 7 (SLC4A7), mRNA [NM_003615]	9497

A_23_P72912	-2.2	-1.0	-1.1	1.0	SLC4A8	Homo sapiens solute carrier family 4, sodium bicarbonate cotransporter, member 8 (SLC4A8), transcript variant 2, mRNA [NM_004858]	9498
A_24_P88565	-2.2	-3.4	-1.5	1.1	SLC50A1	Homo sapiens solute carrier family 50 (sugar transporter), member 1 (SLC50A1), transcript variant 1, mRNA [NM_018845]	55974

A_23_P328022	-6.9	1.0	-1.0	1.1	SLC5A10	Homo sapiens solute carrier family 5 (sodium/glucose cotransporter), member 10 (SLC5A10), transcript variant 1, mRNA [NM_152351]	125206
A_23_P411277	-1.3	2.3	-2.2	-1.6	SLC5A2	Homo sapiens solute carrier family 5 (sodium/glucose cotransporter), member 2 (SLC5A2), mRNA [NM_003041]	6524
A_23_P57492	1.0	-4.0	-1.0	1.1	SLC5A4	Homo sapiens solute carrier family 5 (low affinity glucose cotransporter), member 4 (SLC5A4), mRNA [NM_014227]	6527

A_23_P101774	2.2	5.0	8.7	1.8	SLC5A5	Homo sapiens solute carrier family 5 (sodium iodide symporter), member 5 (SLC5A5), mRNA [NM_000453]	6528
A_24_P247732	-1.2	-3.7	-1.4	-2.6	SLC5A6	Homo sapiens solute carrier family 5 (sodium-dependent vitamin transporter), member 6 (SLC5A6), transcript variant 1, mRNA [NM_021095]	8884

A_24_P923483	-5.2	1.1	1.1	1.1	SLC6A13	Homo sapiens solute carrier family 6 (neurotransmitter transporter, GABA), member 13 (SLC6A13), transcript variant 3, mRNA [NM_001243392]	6540
A_24_P365721	-1.1	-1.1	-2.6	-1.2	SLC6A14	Homo sapiens solute carrier family 6 (amino acid transporter), member 14 (SLC6A14), mRNA [NM_007231]	11254

A_23_P356598	-2.8	-4.1	1.0	-2.4	SLC6A15	Homo sapiens solute carrier family 6 (neutral amino acid transporter), member 15 (SLC6A15), transcript variant 2, mRNA [NM_018057]	55117
A_23_P13725	-5.0	1.1	1.1	1.5	SLC6A15	Homo sapiens solute carrier family 6 (neutral amino acid transporter), member 15 (SLC6A15), transcript variant 1, mRNA [NM_182767]	55117

A_24_P109838	2.3	2.2	2.3	1.8	SLC6A15	Homo sapiens solute carrier family 6 (neutral amino acid transporter), member 15 (SLC6A15), transcript variant 1, mRNA [NM_182767]	55117
A_24_P212234	2.7	3.2	4.4	2.5	SLC6A18	Homo sapiens solute carrier family 6, member 18 (SLC6A18), mRNA [NM_182632]	348932
A_23_P62070	3.3	3.7	2.9	-1.1	SLC6A19	Homo sapiens solute carrier family 6 (neutral amino acid transporter), member 19 (SLC6A19), mRNA [NM_001003841]	340024

A_23_P116624	-1.4	-1.8	-1.3	-2.3	SLC6A5	Homo sapiens solute carrier family 6 (neurotransmitter transporter, glycine), member 5 (SLC6A5), mRNA [NM_004211]	9152
A_23_P69206	1.6	-1.4	-2.3	2.8	SLC6A6	Homo sapiens solute carrier family 6 (neurotransmitter transporter, taurine), member 6 (SLC6A6), transcript variant 1, mRNA [NM_003043]	6533

A_24_P46093	-1.6	1.5	-2.0	1.0	SLC6A6	Homo sapiens solute carrier family 6 (neurotransmitter transporter, taurine), member 6 (SLC6A6), transcript variant 1, mRNA [NM_003043]	6533
A_24_P359191	2.8	-1.2	3.4	3.1	SLC6A6	Homo sapiens solute carrier family 6 (neurotransmitter transporter, taurine), member 6 (SLC6A6), transcript variant 1, mRNA [NM_003043]	6533

A_24_P253251	-280.7	-1.0	-1.1	-3.6	SLC7A1	Homo sapiens solute carrier family 7 (cationic amino acid transporter, y+ system), member 1 (SLC7A1), mRNA [NM_003045]	6541
A_23_P117242	-2.0	1.0	-1.2	-1.8	SLC7A1	Homo sapiens solute carrier family 7 (cationic amino acid transporter, y+ system), member 1 (SLC7A1), mRNA [NM_003045]	6541
A_23_P3792	-1.3	3.4	3.2	-1.0	SLC7A5	Homo sapiens solute carrier family 7 (amino acid transporter light chain, L system), member 5 (SLC7A5), mRNA [NM_003486]	8140

A_24_P159312	1.0	-1.0	-2.9	1.0	SLC7A6	Homo sapiens solute carrier family 7 (amino acid transporter light chain, y+L system), member 6 (SLC7A6), transcript variant 1, mRNA [NM_001076785]	9057
A_24_P125081	-1.0	-1.0	2.3	-1.0	SLC7A6OS	solute carrier family 7, member 6 opposite strand [Source:HGNC Symbol;Acc:25807] [ENST00000263997]	84138
A_32_P147078	-1.5	-3.0	-1.0	-2.9	SLC8A1	Homo sapiens solute carrier family 8 (sodium/calcium exchanger), member 1 (SLC8A1), transcript variant A, mRNA [NM_021097]	6546

A_24_P913900	1.6	1.6	-3.2	1.0	SLC8A1	H.sapiens mRNA for Na+/Ca2+ exchanger isoform. [X91815]	6546
A_23_P336954	1.4	1.0	2.4	3.4	SLC8A2	Homo sapiens solute carrier family 8 (sodium/calcium exchanger), member 2 (SLC8A2), mRNA [NM_015063]	6543
A_24_P167654	-1.5	-2.1	1.4	1.5	SLC8A3	Homo sapiens solute carrier family 8 (sodium/calcium exchanger), member 3 (SLC8A3), transcript variant c, mRNA [NM_183002]	6547
A_24_P24770	1.2	3.6	2.8	1.5	SLC9A10	Homo sapiens solute carrier family 9, member 10 (SLC9A10), mRNA [NM_183061]	285335

A_23_P30582	1.1	1.1	-1.0	-3.2	SLC9A3	Homo sapiens solute carrier family 9 (sodium/hydrogen exchanger), member 3 (SLC9A3), mRNA [NM_004174]	6550
A_24_P364838	-1.9	-2.0	-3.6	1.1	SLC9A3R2	Homo sapiens solute carrier family 9 (sodium/hydrogen exchanger), member 3 regulator 2 (SLC9A3R2), transcript variant 2, mRNA [NM_004785]	9351
A_23_P11032	-1.0	-2.7	-2.4	-1.1	SLC9A7	Homo sapiens solute carrier family 9 (sodium/hydrogen exchanger), member 7 (SLC9A7), mRNA [NM_032591]	84679

A_24_P58054	-3.5	-1.5	-1.7	-1.6	SLC9A8	Homo sapiens solute carrier family 9 (sodium/hydrogen exchanger), member 8 (SLC9A8), mRNA [NM_015266]	23315
A_32_P46214	47.7	46.4	30.8	48.2	SLC9A9	Homo sapiens solute carrier family 9 (sodium/hydrogen exchanger), member 9 (SLC9A9), mRNA [NM_173653]	285195
A_23_P384965	-4.8	-1.2	-1.2	1.7	SLC9A9	solute carrier family 9 (sodium/hydrogen exchanger), member 9 [Source:HGNC Symbol;Acc:20653] [ENST00000498717]	285195

A_24_P657226	-1.0	-1.0	-1.1	-9.6	SLC9B1	Homo sapiens solute carrier family 9, subfamily B (cation proton antiporter 2), member 1 (SLC9B1), nuclear gene encoding mitochondrial protein, transcript variant 1, mRNA [NM_139173]	150159
A_23_P415611	-2.0	1.1	1.1	-1.2	SLC9B1	Homo sapiens solute carrier family 9, subfamily B (cation proton antiporter 2), member 1 (SLC9B1), nuclear gene encoding mitochondrial protein, transcript variant 1, mRNA [NM_139173]	150159

A_23_P81094	2.6	2.5	2.3	1.6	SLC9B2	Homo sapiens solute carrier family 9, subfamily B (cation proton antiporter 2), member 2 (SLC9B2), nuclear gene encoding mitochondrial protein, mRNA [NM_178833]	133308
A_23_P13820	1.0	-1.0	-1.1	-2.1	SLCO1A2	Homo sapiens solute carrier organic anion transporter family, member 1A2 (SLCO1A2), transcript variant 1, mRNA [NM_134431]	6579

A_23_P128254	1.0	-8.9	-1.0	1.0	SLCO1B1	Homo sapiens solute carrier organic anion transporter family, member 1B1 (SLCO1B1), mRNA [NM_006446]	10599
A_23_P105475	-2.1	1.9	1.1	1.1	SLCO1B3	Homo sapiens solute carrier organic anion transporter family, member 1B3 (SLCO1B3), mRNA [NM_019844]	28234
A_23_P116868	-1.8	-1.1	-1.0	-2.3	SLCO1C1	Homo sapiens solute carrier organic anion transporter family, member 1C1 (SLCO1C1), transcript variant 2, mRNA [NM_017435]	53919

A_24_P588897	3.6	3.5	3.2	3.3	SLCO3A1	Homo sapiens solute carrier organic anion transporter family, member 3A1 (SLCO3A1), transcript variant 1, mRNA [NM_013272]	28232
A_23_P389141	-3.7	-1.0	-1.1	1.0	SLFNL1	Homo sapiens schlafen-like 1 (SLFNL1), transcript variant 1, mRNA [NM_144990]	200172
A_23_P161386	-2.0	2.4	-3.2	-1.0	SLIT1	Homo sapiens slit homolog 1 (Drosophila) (SLIT1), mRNA [NM_003061]	6585

A_24_P910566	16.7	16.8	9.9	13.6	SLIT1	slit homolog 1 (Drosophila) [Source:HGNC Symbol;Acc:11085] [ENST00000497714]	6585
A_23_P58588	4.0	4.6	4.0	3.0	SLIT3	Homo sapiens slit homolog 3 (Drosophila) (SLIT3), mRNA [NM_003062]	6586
A_23_P37041	-3.5	1.1	1.1	1.0	SLITRK1	Homo sapiens SLIT and NTRK-like family, member 1 (SLITRK1), mRNA [NM_052910]	114798
A_32_P136351	-1.3	-1.8	-2.3	-2.4	SLITRK1	Homo sapiens SLIT and NTRK-like family, member 1 (SLITRK1), mRNA [NM_052910]	114798

A_23_P18362	3.3	1.6	-1.1	4.0	SLITRK3	Homo sapiens SLIT and NTRK-like family, member 3 (SLITRK3), mRNA [NM_014926]	22865
A_32_P131640	1.1	1.1	-2.7	-1.0	SLITRK4	Homo sapiens SLIT and NTRK-like family, member 4 (SLITRK4), transcript variant 2, mRNA [NM_173078]	139065
A_23_P202199	-2.5	-2.6	-2.9	-5.1	SLK	Homo sapiens STE20-like kinase (SLK), mRNA [NM_014720]	9748
A_24_P146670	-1.4	-3.7	1.0	1.0	SLK	Homo sapiens STE20-like kinase (SLK), mRNA [NM_014720]	9748
A_23_P91230	1.1	-2.1	1.1	-1.2	SLPI	Homo sapiens secretory leukocyte peptidase inhibitor (SLPI), mRNA [NM_003064]	6590

A_24_P190472	4.9	3.8	-1.9	3.6	SLPI	Homo sapiens secretory leukocyte peptidase inhibitor (SLPI), mRNA [NM_003064]	6590
A_24_P568190	1.0	1.0	-9.4	-1.1	SMA4	Homo sapiens glucuronidase, beta pseudogene 1, mRNA (cDNA clone IMAGE:4824349). [BC035411]	11039
A_23_P121869	1.1	-5.7	-1.5	-1.0	SMA5	Homo sapiens glucuronidase, beta pseudogene (SMA5), non-coding RNA [NR_034021]	11042
A_23_P212870	1.1	-2.1	1.0	-1.0	SMAD1	Homo sapiens SMAD family member 1 (SMAD1), transcript variant 1, mRNA [NM_005900]	4086

A_32_P12580	-7.8	1.3	1.2	1.0	SMAD2	Homo sapiens SMAD family member 2 (SMAD2), transcript variant 2, mRNA [NM_001003652]	4087
A_24_P202527	-1.3	-1.9	-3.5	-1.9	SMAD2	Homo sapiens SMAD family member 2 (SMAD2), transcript variant 2, mRNA [NM_001003652]	4087
A_23_P359091	-1.3	-2.3	2.5	1.2	SMAD3	Homo sapiens mad protein homolog (hMAD-3) mRNA, complete cds. [U68019]	4088
A_23_P399797	1.7	1.7	2.4	1.0	SMAD5-AS1	Homo sapiens SMAD5 antisense RNA 1 (non-protein coding) (SMAD5-AS1), non-coding RNA [NR_026763]	9597

A_23_P65812	1.1	-2.3	-1.0	-2.1	SMAD6	Homo sapiens SMAD family member 6 (SMAD6), transcript variant 1, mRNA [NM_005585]	4091
A_23_P55518	1.4	2.7	1.3	1.3	SMAD7	Homo sapiens SMAD family member 7 (SMAD7), transcript variant 1, mRNA [NM_005904]	4092
A_23_P2814	5.7	4.7	3.1	2.6	SMAD9	Homo sapiens SMAD family member 9 (SMAD9), transcript variant b, mRNA [NM_005905]	4093
A_24_P225325	-3.3	-4.2	-1.8	1.1	SMAP2	Homo sapiens small ArfGAP2 (SMAP2), transcript variant 1, mRNA [NM_022733]	64744

A_23_P39040	-1.6	-1.1	-2.7	-2.5	SMARCA4	Homo sapiens SWI/SNF related, matrix associated, actin dependent regulator of chromatin, subfamily a, member 4 (SMARCA4), transcript variant 3, mRNA [NM_003072]	6597
A_24_P212851	-1.7	2.1	1.9	1.5	SMARCA4	Homo sapiens SWI/SNF related, matrix associated, actin dependent regulator of chromatin, subfamily a, member 4 (SMARCA4), transcript variant 3, mRNA [NM_003072]	6597

A_23_P39034	1.3	1.1	-2.8	-1.9	SMARCA4	Homo sapiens SWI/SNF related, matrix associated, actin dependent regulator of chromatin, subfamily a, member 4 (SMARCA4), transcript variant 3, mRNA [NM_003072]	6597
A_32_P93187	1.0	1.3	-2.5	-1.3	SMARCA5	Homo sapiens SWI/SNF related, matrix associated, actin dependent regulator of chromatin, subfamily a, member 5 (SMARCA5), mRNA [NM_003601]	8467

A_23_P83453	2.5	1.3	2.7	1.7	SMARCC1	Homo sapiens SWI/SNF related, matrix associated, actin dependent regulator of chromatin, subfamily c, member 1 (SMARCC1), mRNA [NM_003074]	6599
A_32_P112104	1.8	1.8	-1.0	4.9	SMARCC1	Homo sapiens SWI/SNF related, matrix associated, actin dependent regulator of chromatin, subfamily c, member 1 (SMARCC1), mRNA [NM_003074]	6599

A_23_P116682	2.1	2.7	1.9	1.7	SMARCC2	Homo sapiens SWI/SNF related, matrix associated, actin dependent regulator of chromatin, subfamily c, member 2 (SMARCC2), transcript variant 2, mRNA [NM_139067]	6601
A_23_P204745	-14.6	-1.0	-1.1	-1.7	SMARCD1	Homo sapiens SWI/SNF related, matrix associated, actin dependent regulator of chromatin, subfamily d, member 1 (SMARCD1), transcript variant 2, mRNA [NM_139071]	6602

A_23_P122852	2.4	3.5	2.4	1.4	SMARCD3	Homo sapiens SWI/SNF related, matrix associated, actin dependent regulator of chromatin, subfamily d, member 3 (SMARCD3), transcript variant 2, mRNA [NM_003078]	6604
A_23_P217411	1.5	-1.5	-2.9	-2.3	SMC1A	Homo sapiens structural maintenance of chromosomes 1A (SMC1A), mRNA [NM_006306]	8243
A_23_P60271	-2.6	-1.3	-1.5	-1.0	SMC2	Homo sapiens structural maintenance of chromosomes 2 (SMC2), transcript variant 1, mRNA [NM_001042550]	10592

A_32_P126609	2.9	-1.1	2.6	2.5	SMC5	Homo sapiens structural maintenance of chromosomes 5 (SMC5), mRNA [NM_015110]	23137
A_23_P349615	-7.2	-2.2	-1.1	-1.1	SMC5	Homo sapiens structural maintenance of chromosomes 5 (SMC5), mRNA [NM_015110]	23137
A_24_P336596	2.1	1.3	1.9	1.5	SMCP	Homo sapiens sperm mitochondria-associated cysteine-rich protein (SMCP), nuclear gene encoding mitochondrial protein, mRNA [NM_030663]	4184

A_23_P388798	-1.0	-1.2	-1.9	-4.8	SMCR5	Homo sapiens Smith-Magenis syndrome chromosome region, candidate 5 (non-protein coding) (SMCR5), non-coding RNA [NR_024007]	140771
A_23_P501372	-5.0	-4.9	1.0	1.1	SMCR7	Homo sapiens Smith-Magenis syndrome chromosome region, candidate 7 (SMCR7), nuclear gene encoding mitochondrial protein, transcript variant 1, mRNA [NM_139162]	125170

A_24_P369691	-2.1	1.1	-3.7	-1.0	SMEK1	Homo sapiens SMEK homolog 1, suppressor of mek1 (Dictyostelium) (SMEK1), mRNA [NM_032560]	55671
A_23_P218158	2.6	1.9	1.3	8.2	SMEK1	Homo sapiens SMEK homolog 1, suppressor of mek1 (Dictyostelium) (SMEK1), mRNA [NM_032560]	55671
A_32_P203592	-3.4	1.0	1.0	1.1	SMG1	Homo sapiens smg-1 homolog, phosphatidylinositol 3-kinase-related kinase (C. elegans) (SMG1), mRNA [NM_015092]	23049

A_32_P142881	1.1	-2.2	1.0	1.1	SMG1	Homo sapiens smg-1 homolog, phosphatidylinositol 3-kinase-related kinase (C. elegans) (SMG1), mRNA [NM_015092]	23049
A_32_P116323	1.1	1.0	-2.1	1.0	SMG1	Homo sapiens smg-1 homolog, phosphatidylinositol 3-kinase-related kinase (C. elegans) (SMG1), mRNA [NM_015092]	23049
A_23_P26358	1.4	1.1	2.1	2.1	SMG1	Homo sapiens smg-1 homolog, phosphatidylinositol 3-kinase-related kinase (C. elegans) (SMG1), mRNA [NM_015092]	23049

A_23_P129871	3.5	5.0	3.0	2.2	SMG6	Homo sapiens cDNA FLJ14069 fis, clone HEMBB1001562, weakly similar to CYLICIN II. [AK024131]	23293
A_23_P201647	-1.4	-4.1	-1.6	-1.6	SMG7	Homo sapiens smg-7 homolog, nonsense mediated mRNA decay factor (C. elegans) (SMG7), transcript variant 1, mRNA [NM_173156]	9887
A_24_P402090	1.2	-1.9	-2.4	-1.2	SMG8	Homo sapiens chromosome 17 open reading frame 71, mRNA (cDNA clone IMAGE:5178217), partial cds. [BC031604]	55181

A_23_P58466	2.1	1.0	-1.0	1.0	SMN1	Homo sapiens survival of motor neuron 1, telomeric (SMN1), transcript variant d, mRNA [NM_000344]	6606
A_23_P70818	-2.1	1.1	-1.6	-5.5	SMO	Homo sapiens smoothened, frizzled family receptor (SMO), mRNA [NM_005631]	6608
A_24_P150466	-2.2	1.0	-1.0	-1.4	SMOC1	Homo sapiens SPARC related modular calcium binding 1 (SMOC1), transcript variant 1, mRNA [NM_001034852]	64093

A_23_P203488	1.7	1.9	2.0	1.9	SMPD1	Homo sapiens sphingomyelin phosphodiesterase 1, acid lysosomal (SMPD1), transcript variant 1, mRNA [NM_000543]	6609
A_23_P82162	-3.7	1.0	1.0	1.0	SMPD2	Homo sapiens sphingomyelin phosphodiesterase 2, neutral membrane (neutral sphingomyelinase) (SMPD2), mRNA [NM_003080]	6610
A_32_P223859	-1.0	-2.1	-2.5	-1.0	SMPDL3A	Homo sapiens sphingomyelin phosphodiesterase, acid-like 3A (SMPDL3A), mRNA [NM_006714]	10924

A_23_P160881	-2.0	-2.8	-1.3	-3.8	SMPDL3B	Homo sapiens sphingomyelin phosphodiesterase, acid-like 3B (SMPDL3B), transcript variant 2, mRNA [NM_001009568]	27293
A_23_P253542	-1.0	-2.0	-1.1	-1.1	SMPX	Homo sapiens small muscle protein, X-linked (SMPX), mRNA [NM_014332]	23676
A_32_P50452	3.9	1.4	4.3	1.9	SMS	Homo sapiens spermine synthase (SMS), mRNA [NM_004595]	6611
A_23_P211428	-2.0	-1.5	-2.5	-3.0	SMTN	Homo sapiens smoothelin (SMTN), transcript variant 2, mRNA [NM_134269]	6525

A_23_P353316	-1.0	-1.0	-1.1	-5.2	SMURF1	Homo sapiens SMAD specific E3 ubiquitin protein ligase 1 (SMURF1), transcript variant 1, mRNA [NM_020429]	57154
A_23_P51410	-8.0	1.0	-1.1	1.0	SMYD3	Homo sapiens SET and MYND domain containing 3 (SMYD3), transcript variant 2, mRNA [NM_022743]	64754
A_32_P103291	1.0	1.0	1.0	-2.1	SMYD3	Homo sapiens SET and MYND domain containing 3 (SMYD3), transcript variant 2, mRNA [NM_022743]	64754

A_23_P91015	-3.1	-4.5	-18.2	-3.0	SMYD5	Homo sapiens SMYD family member 5 (SMYD5), mRNA [NM_006062]	10322
A_23_P131846	-1.9	-2.0	-1.1	-2.1	SNAI1	Homo sapiens snail homolog 1 (Drosophila) (SNAI1), mRNA [NM_005985]	6615
A_24_P937139	-1.2	-6.4	-1.5	-1.7	SNAI2	Homo sapiens Slug mRNA, partial cds. [U97060]	6591
A_23_P206177	1.7	2.1	-2.6	-1.0	SNAP23	Homo sapiens synaptosomal-associated protein, 23kDa (SNAP23), transcript variant 1, mRNA [NM_003825]	8773

A_23_P214803	2.0	1.6	1.8	-2.1	SNAP91	Homo sapiens synaptosomal-associated protein, 91kDa homolog (mouse) (SNAP91), transcript variant 1, mRNA [NM_014841]	9892
A_23_P37244	2.1	2.0	1.8	1.2	SNAPC1	Homo sapiens small nuclear RNA activating complex, polypeptide 1, 43kDa (SNAPC1), mRNA [NM_003082]	6617
A_23_P383977	1.2	-2.9	1.7	1.7	SNAPC5	Homo sapiens small nuclear RNA activating complex, polypeptide 5, 19kDa (SNAPC5), mRNA [NM_006049]	10302

A_23_P21776	-2.9	1.0	1.0	1.0	SNAPC5	small nuclear RNA activating complex, polypeptide 5, 19kDa [Source:HGNC Symbol;Acc:15484] [ENST00000316634]	10302
A_23_P19020	-1.0	-1.0	-6.6	-1.5	SNCAIP	Homo sapiens synuclein, alpha interacting protein (SNCAIP), transcript variant 1, mRNA [NM_005460]	9627
A_23_P121945	1.5	2.8	2.2	-1.4	SNCB	Homo sapiens synuclein, beta (SNCB), transcript variant 1, mRNA [NM_001001502]	6620

A_24_P610618	-1.2	-1.1	2.6	-1.8	SNF8	SNF8, ESCRT-II complex subunit, homolog (S. cerevisiae) [Source:HGNC Symbol;Acc:17028] [ENST00000504000]	11267
A_23_P74581	8.0	7.8	7.5	6.3	SNHG12	Homo sapiens small nucleolar RNA host gene 12 (non-protein coding) (SNHG12), non-coding RNA [NR_024127]	85028
A_24_P902052	-2.3	-1.0	-1.1	-1.1	SNHG13	Homo sapiens small nucleolar RNA host gene 13 (non-protein coding) (SNHG13), non-coding RNA [NR_024031]	57291

A_23_P94579	-2.6	-13.2	-1.0	-1.1	SNHG7	Homo sapiens small nucleolar RNA host gene 7 (non-protein coding) (SNHG7), transcript variant 1, non-coding RNA [NR_003672]	84973
A_24_P352116	-6.7	-1.1	-1.3	-1.8	SNHG7	Homo sapiens small nucleolar RNA host gene 7 (non-protein coding) (SNHG7), transcript variant 1, non-coding RNA [NR_003672]	84973
A_23_P102706	1.9	4.8	2.0	1.2	SNPH	Homo sapiens syntaphilin (SNPH), mRNA [NM_014723]	9751

A_24_P936688	-1.8	-2.2	-2.3	-2.1	SNRK	Homo sapiens SNF related kinase (SNRK), transcript variant 1, mRNA [NM_017719]	54861
A_24_P102151	-17.1	-1.0	-1.6	-1.1	SNRK	SNF related kinase [Source:HGNC Symbol;Acc:30598] [ENST00000462810]	54861
A_23_P159101	-2.8	-1.2	-3.1	-1.2	SNRNP35	Homo sapiens small nuclear ribonucleoprotein 35kDa (U11/U12) (SNRNP35), transcript variant 2, mRNA [NM_022717]	11066

A_24_P922056	-1.0	-2.1	-1.1	-1.5	SNRNP35	small nuclear ribonucleoprotein 35kDa (U11/U12) [Source:HGNC Symbol;Acc:30852] [ENST00000527158]	11066
A_23_P45913	-1.1	-2.2	-1.4	1.0	SNRNP40	Homo sapiens small nuclear ribonucleoprotein 40kDa (U5) (SNRNP40), mRNA [NM_004814]	9410
A_23_P154675	1.2	1.1	1.3	2.7	SNRPB	Homo sapiens small nuclear ribonucleoprotein polypeptides B and B1 (SNRPB), transcript variant 1, mRNA [NM_198216]	6628

A_24_P724040	-1.3	-1.4	-2.6	-1.2	SNRPB2	Homo sapiens small nuclear ribonucleoprotein polypeptide B (SNRPB2), transcript variant 1, mRNA [NM_003092]	6629
A_24_P83922	-1.3	-4.7	-1.5	-1.3	SNRPC	Homo sapiens small nuclear ribonucleoprotein polypeptide C (SNRPC), transcript variant 1, mRNA [NM_003093]	6631
A_23_P132216	-1.0	-1.0	2.0	1.5	SNRPD3	Homo sapiens small nuclear ribonucleoprotein D3 polypeptide 18kDa (SNRPD3), mRNA [NM_004175]	6634

A_23_P117068	-1.3	-1.1	-4.7	-1.2	SNRPF	Homo sapiens small nuclear ribonucleoprotein polypeptide F (SNRPF), mRNA [NM_003095]	6636
A_23_P77066	1.5	2.3	1.4	-1.0	SNRPN	Homo sapiens small nuclear ribonucleoprotein polypeptide N (SNRPN), transcript variant 4, mRNA [NM_022807]	6638
A_23_P95029	1.0	-2.7	1.0	-1.0	SNTB1	Homo sapiens syntrophin, beta 1 (dystrophin-associated protein A1, 59kDa, basic component 1) (SNTB1), mRNA [NM_021021]	6641

A_23_P33444	1.1	-3.8	-1.0	-1.0	SNTB2	Homo sapiens syntrophin, beta 2 (dystrophin-associated protein A1, 59kDa, basic component 2) (SNTB2), mRNA [NM_006750]	6645
A_32_P187704	-1.2	-1.3	-1.3	-2.4	SNTG1	syntrophin, gamma 1 [Source:HGNC Symbol;Acc:13740] [ENST00000521574]	54212
A_24_P347624	1.8	2.0	-3.0	1.1	SNURF	Homo sapiens SNRPN upstream reading frame (SNURF), transcript variant 2, mRNA [NM_022804]	8926

A_23_P49033	-2.0	-2.0	-2.1	-2.1	SNX1	Homo sapiens sorting nexin 1 (SNX1), transcript variant 1, mRNA [NM_003099]	6642
A_23_P89343	-2.7	-1.8	-1.1	-1.5	SNX11	Homo sapiens sorting nexin 11 (SNX11), transcript variant 1, mRNA [NM_152244]	29916
A_23_P10699	-4.7	-3.2	-2.7	-1.0	SNX12	sorting nexin 12 [Source:HGNC Symbol;Acc:14976] [ENST00000374274]	29934
A_23_P256342	-1.3	-2.0	-1.0	-1.0	SNX13	Homo sapiens sorting nexin 13 (SNX13), mRNA [NM_015132]	23161
A_23_P259054	1.5	1.0	-1.7	-2.4	SNX14	Homo sapiens sorting nexin 14 (SNX14), transcript variant 1, mRNA [NM_153816]	57231

A_24_P198217	-1.5	2.8	-1.4	-1.3	SNX15	Homo sapiens sorting nexin 15 (SNX15), transcript variant B, mRNA [NM_147777]	29907
A_24_P320880	-1.1	-1.1	-1.4	-4.6	SNX16	Homo sapiens sorting nexin 16 (SNX16), transcript variant 1, mRNA [NM_022133]	64089
A_24_P81695	1.6	2.3	1.6	3.2	SNX19	Homo sapiens sorting nexin 19 (SNX19), mRNA [NM_014758]	399979
A_24_P81691	1.1	1.0	1.3	2.0	SNX19	Homo sapiens sorting nexin 19 (SNX19), mRNA [NM_014758]	399979
A_23_P352469	-7.2	-2.1	-2.9	-5.9	SNX20	Homo sapiens sorting nexin 20 (SNX20), transcript variant 2, mRNA [NM_153337]	124460

A_24_P13831	1.0	-3.7	-2.9	1.5	SNX20	Homo sapiens sorting nexin 20 (SNX20), transcript variant 1, mRNA [NM_182854]	124460
A_23_P154585	1.4	-12.9	1.6	-1.1	SNX21	Homo sapiens sorting nexin family member 21 (SNX21), transcript variant 4, mRNA [NM_001042633]	90203
A_24_P375421	1.1	-1.7	1.7	-4.5	SNX22	Homo sapiens sorting nexin 22 (SNX22), mRNA [NM_024798]	79856
A_23_P19095	13.2	11.7	12.1	13.0	SNX24	Homo sapiens sorting nexin 24 (SNX24), mRNA [NM_014035]	28966
A_23_P367816	1.1	1.1	-1.0	-6.9	SNX29	Homo sapiens sorting nexin 29 (SNX29), mRNA [NM_032167]	92017

A_32_P66020	2.0	1.2	2.2	-2.1	SNX29	Homo sapiens sorting nexin 29 (SNX29), mRNA [NM_032167]	92017
A_24_P277807	-1.1	-1.1	-1.3	2.4	SNX3	Homo sapiens sorting nexin 3 (SNX3), transcript variant 1, mRNA [NM_003795]	8724
A_23_P351800	1.4	1.3	-1.5	-3.3	SNX32	sorting nexin 32 [Source:HGNC Symbol;Acc:26423] [ENST00000530101]	254122
A_23_P254404	-1.5	-8.8	-1.6	-4.1	SNX33	Homo sapiens sorting nexin 33 (SNX33), mRNA [NM_153271]	257364
A_23_P131954	2.0	2.7	1.7	2.4	SNX5	Homo sapiens sorting nexin 5 (SNX5), transcript variant 2, mRNA [NM_014426]	27131

A_23_P35066	1.2	-1.0	2.1	-1.6	SNX7	Homo sapiens sorting nexin 7 (SNX7), transcript variant 1, mRNA [NM_015976]	51375
A_23_P414252	-1.2	-11.1	1.3	1.0	SNX8	Homo sapiens cDNA, FLJ96764, highly similar to Homo sapiens sorting nexin 8 (SNX8), mRNA. [AK315670]	29886
A_23_P214789	-11.2	-2.2	-2.1	-1.2	SNX9	Homo sapiens sorting nexin 9 (SNX9), mRNA [NM_016224]	51429
A_24_P179044	1.3	2.0	2.1	-1.3	SNX9	Homo sapiens sorting nexin 9 (SNX9), mRNA [NM_016224]	51429

A_32_P103131	-1.0	-2.4	-1.2	-1.1	SOAT1	sterol O-acyltransferase 1 [Source:HGNC Symbol;Acc:11177] [ENST00000367619]	6646
A_23_P156907	-1.7	-1.9	-2.0	-1.6	SOBP	Homo sapiens sine oculis binding protein homolog (Drosophila) (SOBP), mRNA [NM_018013]	55084
A_23_P420196	2.1	-1.1	1.2	-1.0	SOCS1	Homo sapiens suppressor of cytokine signaling 1 (SOCS1), mRNA [NM_003745]	8651
A_23_P128215	-1.0	-1.0	2.2	-1.0	SOCS2	Homo sapiens suppressor of cytokine signaling 2 (SOCS2), mRNA [NM_003877]	8835

A_23_P351069	-3.6	1.9	1.2	1.3	SOCS3	Homo sapiens suppressor of cytokine signaling 3 (SOCS3), mRNA [NM_003955]	9021
A_23_P151700	2.3	1.5	1.6	-1.6	SOCS4	Homo sapiens suppressor of cytokine signaling 4 (SOCS4), transcript variant 1, mRNA [NM_199421]	122809
A_32_P43711	-1.3	-1.5	-4.1	-4.6	SOCS7	Homo sapiens suppressor of cytokine signaling 7 (SOCS7), mRNA [NM_014598]	30837
A_24_P367211	1.7	2.4	2.2	-1.0	SOCS7	Homo sapiens suppressor of cytokine signaling 7 (SOCS7), mRNA [NM_014598]	30837

A_24_P193244	1.8	-2.0	1.2	1.1	SOHLH2	Homo sapiens spermatogenesis and oogenesis specific basic helix-loop-helix 2 (SOHLH2), mRNA [NM_017826]	54937
A_24_P928639	-3.3	-1.1	-1.2	-1.2	SOLH	Homo sapiens small optic lobes homolog (Drosophila), mRNA (cDNA clone IMAGE:5518093). [BC032648]	6650
A_23_P118113	-2.8	1.0	1.1	1.0	SOLH	Homo sapiens small optic lobes homolog (Drosophila) (SOLH), mRNA [NM_005632]	6650

A_24_P282210	2.1	-1.5	1.3	1.7	SOLH	Homo sapiens small optic lobes homolog (Drosophila) (SOLH), mRNA [NM_005632]	6650
A_32_P36217	3.2	3.8	2.1	1.2	SON	Homo sapiens SON DNA binding protein (SON), transcript variant f, mRNA [NM_138927]	6651
A_23_P402733	-1.1	2.1	1.2	2.8	SON	Homo sapiens SON DNA binding protein (SON), transcript variant b, mRNA [NM_032195]	6651
A_24_P326511	-1.0	-2.4	-1.1	-1.1	SORBS1	Homo sapiens sorbin and SH3 domain containing 1 (SORBS1), transcript variant 3, mRNA [NM_001034954]	10580

A_24_P325520	-1.6	1.4	2.5	1.0	SORT1	Homo sapiens sortilin 1 (SORT1), transcript variant 1, mRNA [NM_002959]	6272
A_23_P343808	4.9	1.0	-2.1	1.1	SOS1	Homo sapiens son of sevenless homolog 1 (Drosophila) (SOS1), mRNA [NM_005633]	6654
A_23_P117546	2.4	2.4	1.0	1.7	SOS2	Homo sapiens son of sevenless homolog 2 (Drosophila) (SOS2), mRNA [NM_006939]	6655
A_23_P145841	2.8	2.5	5.0	2.5	SOSTDC1	Homo sapiens sclerostin domain containing 1 (SOSTDC1), mRNA [NM_015464]	25928

A_24_P108784	1.1	-25.6	1.4	-1.0	SOX10	Homo sapiens SRY (sex determining region Y)-box 10 (SOX10), mRNA [NM_006941]	6663
A_23_P22378	-6.5	-3.2	-3.3	-3.2	SOX11	Homo sapiens SRY (sex determining region Y)-box 11 (SOX11), mRNA [NM_003108]	6664
A_24_P156267	1.0	-2.5	1.0	1.4	SOX12	Homo sapiens SRY (sex determining region Y)-box 12 (SOX12), mRNA [NM_006943]	6666
A_23_P85703	-1.0	-4.4	-3.0	-1.0	SOX13	Homo sapiens SRY (sex determining region Y)-box 13 (SOX13), mRNA [NM_005686]	9580

A_23_P80662	4.5	3.7	4.9	3.5	SOX14	Homo sapiens SRY (sex determining region Y)-box 14 (SOX14), mRNA [NM_004189]	8403
A_24_P46946	-1.0	1.3	-1.4	3.2	SOX17	Homo sapiens SRY (sex determining region Y)-box 17 (SOX17), mRNA [NM_022454]	64321
A_23_P255418	20.2	25.7	55.0	22.8	SOX18	Homo sapiens SRY (sex determining region Y)-box 18 (SOX18), mRNA [NM_018419]	54345
A_23_P379089	-1.4	-1.7	-2.4	-3.4	SOX21	Homo sapiens SRY (sex determining region Y)-box 21 (SOX21), mRNA [NM_007084]	11166

A_24_P756289	1.1	-2.2	-1.1	1.0	SOX2-OT	Homo sapiens SOX2 overlapping transcript (non-protein coding) (SOX2-OT), non-coding RNA [NR_004053]	347689
A_24_P113725	2.2	2.3	1.2	-1.4	SOX3	Homo sapiens SRY (sex determining region Y)-box 3 (SOX3), mRNA [NM_005634]	6658
A_24_P911676	-3.8	-1.6	-2.6	1.5	SOX4	Homo sapiens SRY (sex determining region Y)-box 4 (SOX4), mRNA [NM_003107]	6659
A_23_P82169	2.6	2.1	2.5	2.9	SOX4	Homo sapiens SRY (sex determining region Y)-box 4 (SOX4), mRNA [NM_003107]	6659

A_24_P27234	2.3	-1.6	1.3	1.9	SOX5	Homo sapiens SRY (sex determining region Y)-box 5 (SOX5), transcript variant 2, mRNA [NM_152989]	6660
A_23_P87952	1.4	2.3	-1.0	-1.1	SOX5	Homo sapiens SRY (sex determining region Y)-box 5 (SOX5), transcript variant 2, mRNA [NM_152989]	6660
A_23_P169738	1.1	-8.3	1.1	1.5	SOX7	Homo sapiens SRY (sex determining region Y)-box 7 (SOX7), mRNA [NM_031439]	83595
A_24_P226782	-9.2	1.6	-1.4	-1.2	SOX7	Homo sapiens SRY (sex determining region Y)-box 7 (SOX7), mRNA [NM_031439]	83595

A_24_P65292	3.5	2.8	1.5	3.1	SOX8	Homo sapiens SRY (sex determining region Y)-box 8 (SOX8), mRNA [NM_014587]	30812
A_23_P66137	2.2	1.3	2.1	1.6	SOX8	Homo sapiens SRY (sex determining region Y)-box 8 (SOX8), mRNA [NM_014587]	30812
A_23_P209712	-22.4	-1.0	-1.1	-1.3	SP100	Homo sapiens SP100 nuclear antigen (SP100), transcript variant 1, mRNA [NM_001080391]	6672
A_23_P349928	1.7	1.7	1.5	-14.4	SP100	Homo sapiens SP100 nuclear antigen (SP100), transcript variant 2, mRNA [NM_003113]	6672

A_23_P209726	-1.7	-2.0	1.0	-1.4	SP140	Homo sapiens SP140 nuclear body protein (SP140), transcript variant 1, mRNA [NM_007237]	11262
A_24_P328504	-1.1	-2.2	-1.2	-2.7	SP140	Homo sapiens SP140 nuclear body protein (SP140), transcript variant 1, mRNA [NM_007237]	11262
A_24_P256526	2.3	1.1	-1.1	1.0	SP2	Homo sapiens Sp2 transcription factor, mRNA (cDNA clone IMAGE:4095335), complete cds. [BC005914]	6668
A_23_P381449	-1.0	-1.6	-3.3	-1.9	SP2	Homo sapiens Sp2 transcription factor (SP2), mRNA [NM_003110]	6668

A_23_P347198	116.7	59.3	51.7	32.4	SP3	Homo sapiens Sp3 transcription factor (SP3), transcript variant 1, mRNA [NM_003111]	6670
A_23_P71064	1.1	-4.4	1.0	-1.0	SP4	Homo sapiens Sp4 transcription factor (SP4), mRNA [NM_003112]	6671
A_32_P74615	1.9	2.1	-1.7	-1.9	SP5	Homo sapiens Sp5 transcription factor (SP5), mRNA [NM_001003845]	389058
A_23_P317729	-5.2	1.1	-1.0	-1.0	SP7	Homo sapiens Sp7 transcription factor (SP7), transcript variant 2, mRNA [NM_152860]	121340

A_24_P276385	5.9	3.4	6.3	1.6	SPACA5	Homo sapiens sperm acrosome associated 5 (SPACA5), mRNA [NM_205856]	389852
A_23_P425116	1.5	1.5	-3.6	1.0	SPAG11B	Homo sapiens sperm associated antigen 11B (SPAG11B), transcript variant A, mRNA [NM_016512]	10407
A_24_P332043	1.6	-1.1	2.7	-1.6	SPAG17	Homo sapiens sperm associated antigen 17 (SPAG17), mRNA [NM_206996]	200162
A_23_P89509	2.2	2.2	1.6	1.1	SPAG5	Homo sapiens sperm associated antigen 5 (SPAG5), mRNA [NM_006461]	10615

A_24_P700052	-1.3	-1.4	-2.1	-2.1	SPAG5-AS1	Homo sapiens SPAG5 antisense RNA 1 (non-protein coding) (SPAG5-AS1), non-coding RNA [NR_040012]	100506436
A_24_P412036	1.0	-2.6	-1.1	-1.1	SPAG8	Homo sapiens sperm associated antigen 8 (SPAG8), transcript variant 2, mRNA [NM_172312]	26206
A_24_P365025	2.3	3.1	3.6	1.1	SPAG9	Homo sapiens sperm associated antigen 9 (SPAG9), transcript variant 3, mRNA [NM_003971]	9043

A_23_P111826	2.1	-2.6	-2.7	-1.1	SPAM1	Homo sapiens sperm adhesion molecule 1 (PH-20 hyaluronidase, zona pellucida binding) (SPAM1), transcript variant 2, mRNA [NM_153189]	6677
A_32_P219660	1.0	-3.0	-1.0	-1.0	SPANXA1	Homo sapiens sperm protein associated with the nucleus, X-linked, family member A1 (SPANXA1), mRNA [NM_013453]	30014
A_24_P91830	3.9	2.2	2.0	2.2	SPANXN3	Homo sapiens SPANX family, member N3 (SPANXN3), mRNA [NM_001009609]	139067

A_23_P209356	1.0	1.0	-1.0	-3.6	SPAST	Homo sapiens spastin (SPAST), transcript variant 1, mRNA [NM_014946]	6683
A_24_P220472	2.4	1.0	1.6	1.1	SPATA12	Homo sapiens spermatogenesis associated 12 (SPATA12), mRNA [NM_181727]	353324
A_23_P6722	1.4	-1.7	-2.1	-2.4	SPATA16	Homo sapiens spermatogenesis associated 16 (SPATA16), mRNA [NM_031955]	83893
A_32_P216841	-5.6	1.0	-1.1	-1.3	SPATA18	Homo sapiens spermatogenesis associated 18 homolog (rat) (SPATA18), mRNA [NM_145263]	132671

A_23_P118633	1.5	3.9	-2.8	1.8	SPATA20	Homo sapiens spermatogenesis associated 20 (SPATA20), mRNA [NM_022827]	64847
A_23_P329956	1.0	1.8	1.4	-2.2	SPATA3	Homo sapiens spermatogenesis associated 3 (SPATA3), mRNA [NM_139073]	130560
A_24_P922252	2.2	-1.1	-1.1	2.7	SPATA5L1	Homo sapiens cDNA FLJ12286 fis, clone MAMMA1001768, weakly similar to CELL DIVISION CYCLE PROTEIN 48 HOMOLOG MJ1156. [AK022348]	79029

A_32_P217390	-19.6	1.0	1.0	-1.0	SPATA8	Homo sapiens spermatogenesis associated 8 (SPATA8), mRNA [NM_173499]	145946
A_23_P390576	-33.8	-1.2	-1.3	-1.4	SPATA9	spermatogenesis associated 9 [Source:HGNC Symbol;Acc:22988] [ENST00000316087]	83890
A_23_P99303	1.0	2.3	3.8	2.3	SPATS2	Homo sapiens spermatogenesis associated, serine-rich 2 (SPATS2), mRNA [NM_023071]	65244
A_23_P131255	1.1	-4.7	1.1	1.0	SPATS2L	Homo sapiens spermatogenesis associated, serine-rich 2-like (SPATS2L), transcript variant 1, mRNA [NM_015535]	26010

A_23_P425579	1.3	1.2	-3.4	-1.2	SPDYA	Homo sapiens speedy homolog A (Xenopus laevis) (SPDYA), transcript variant 2, mRNA [NM_001008779]	245711
A_23_P425967	1.1	-1.0	-1.0	-4.0	SPDYE1	Homo sapiens speedy homolog E1 (Xenopus laevis) (SPDYE1), mRNA [NM_175064]	285955
A_24_P178396	2.1	1.3	1.3	-1.4	SPDYE2	Homo sapiens speedy homolog E2 (Xenopus laevis) (SPDYE2), mRNA [NM_001031618]	441273
A_32_P222079	1.7	1.0	-1.1	-2.5	SPDYE2	Homo sapiens speedy homolog E2 (Xenopus laevis) (SPDYE2), mRNA [NM_001031618]	441273

A_24_P100846	-3.1	-1.2	-1.9	-2.9	SPDYE3	Homo sapiens speedy homolog E3 (Xenopus laevis) (SPDYE3), mRNA [NM_001004351]	441272
A_32_P122529	1.4	2.1	1.9	-1.5	SPDYE3	Homo sapiens speedy homolog E3 (Xenopus laevis) (SPDYE3), mRNA [NM_001004351]	441272
A_32_P205323	1.1	1.0	-2.1	1.1	SPDYE3	Homo sapiens speedy homolog E3 (Xenopus laevis) (SPDYE3), mRNA [NM_001004351]	441272
A_24_P333136	-1.2	1.2	-1.8	-5.0	SPDYE3	Homo sapiens speedy homolog E3 (Xenopus laevis) (SPDYE3), mRNA [NM_001004351]	441272

A_32_P107667	-5.1	3.0	1.9	1.0	SPDYE3	Homo sapiens speedy homolog E3 (Xenopus laevis) (SPDYE3), mRNA [NM_001004351]	441272
A_24_P255986	-1.0	2.2	1.4	-1.0	SPDYE4	Homo sapiens speedy homolog E4 (Xenopus laevis) (SPDYE4), mRNA [NM_001128076]	388333
A_32_P88817	-2.1	-2.5	1.4	1.6	SPDYE7P	Homo sapiens speedy homolog E7 (Xenopus laevis), pseudogene (SPDYE7P), non-coding RNA [NR_003666]	441251

A_32_P202182	1.0	-8.7	1.0	1.0	SPDYE8P	Homo sapiens speedy homolog E8 (Xenopus laevis), pseudogene (SPDYE8P), non-coding RNA [NR_003664]	389517
A_24_P943095	-7.1	-1.3	-1.3	-1.4	SPECC1	Homo sapiens sperm antigen with calponin homology and coiled-coil domains 1 (SPECC1), transcript variant 1, mRNA [NM_001033553]	92521
A_24_P927444	-3.7	-1.4	2.0	1.0	SPECC1	Homo sapiens sperm antigen with calponin homology and coiled-coil domains 1 (SPECC1), transcript variant 3, mRNA [NM_152904]	92521

A_32_P203515	1.1	1.1	-10.2	1.1	SPECC1L	Homo sapiens sperm antigen with calponin homology and coiled-coil domains 1-like (SPECC1L), transcript variant 1, mRNA [NM_015330]	23384
A_23_P40280	3.2	1.4	-1.3	1.4	SPEF1	Homo sapiens sperm flagellar 1 (SPEF1), mRNA [NM_015417]	25876
A_32_P303449	1.0	-2.3	1.1	-1.2	SPEM1	Homo sapiens spermatid maturation 1 (SPEM1), mRNA [NM_199339]	374768
A_24_P67189	2.4	1.4	3.5	2.7	SPEM1	spermatid maturation 1 [Source:HGNC Symbol;Acc:32429] [ENST00000323383]	374768

A_23_P3627	2.4	1.7	2.1	1.6	SPG7	Homo sapiens spastic paraplegia 7 (pure and complicated autosomal recessive) (SPG7), nuclear gene encoding mitochondrial protein, transcript variant 1, mRNA [NM_003119]	6687
A_24_P205120	2.8	2.0	1.1	-1.2	SPG7	Homo sapiens spastic paraplegia 7 (pure and complicated autosomal recessive) (SPG7), nuclear gene encoding mitochondrial protein, transcript variant 2, mRNA [NM_199367]	6687

A_23_P303181	1.0	1.5	1.6	-4.8	SPHK2	Homo sapiens sphingosine kinase 2 (SPHK2), transcript variant 1, mRNA [NM_020126]	56848
A_23_P115998	3.6	3.6	2.6	3.5	SPI1	Homo sapiens spleen focus forming virus (SFFV) proviral integration oncogene spi1 (SPI1), transcript variant 2, mRNA [NM_003120]	6688
A_23_P27668	-1.7	1.1	2.4	1.3	SPIB	Homo sapiens Spi-B transcription factor (Spi-1/PU.1 related) (SPIB), transcript variant 1, mRNA [NM_003121]	6689

A_32_P148275	1.8	2.5	1.6	1.3	SPIC	Homo sapiens Spi-C transcription factor (Spi-1/PU.1 related) (SPIC), mRNA [NM_152323]	121599
A_23_P109733	-2.6	2.6	2.5	1.4	SPICE1	Homo sapiens spindle and centriole associated protein 1 (SPICE1), mRNA [NM_144718]	152185
A_23_P125553	1.8	-2.1	1.1	-1.4	SPIN2B	Homo sapiens spindlin family, member 2B (SPIN2B), transcript variant 3, mRNA [NM_001006683]	474343
A_24_P194508	1.5	-2.6	1.7	1.0	SPIN2B	Homo sapiens spindlin family, member 2B (SPIN2B), transcript variant 3, mRNA [NM_001006683]	474343

A_32_P222961	-1.0	-1.3	-1.0	-2.7	SPIN4	Homo sapiens spindlin family, member 4 (SPIN4), mRNA [NM_001012968]	139886
A_23_P71880	2.4	-2.6	2.2	1.6	SPINK4	Homo sapiens serine peptidase inhibitor, Kazal type 4 (SPINK4), mRNA [NM_014471]	27290
A_23_P356494	-11.0	-1.0	1.0	-2.2	SPINK5	Homo sapiens serine peptidase inhibitor, Kazal type 5 (SPINK5), transcript variant 2, mRNA [NM_006846]	11005
A_23_P210503	1.5	1.4	-3.4	1.0	SPINLW1-WF(	Homo sapiens SPINLW1-WFDC6 readthrough (SPINLW1-WFDC6), mRNA [NM_001198986]	100526773

A_23_P433760	1.3	-2.0	1.5	-1.7	SPN	Homo sapiens sialophorin (SPN), transcript variant 1, mRNA [NM_001030288]	6693
A_23_P433758	3.1	1.7	2.3	3.6	SPN	Homo sapiens sialophorin (SPN), transcript variant 1, mRNA [NM_001030288]	6693
A_24_P339153	1.6	2.5	1.6	2.1	SPNS1	Homo sapiens spinster homolog 1 (Drosophila) (SPNS1), transcript variant 1, mRNA [NM_032038]	83985
A_24_P230173	1.4	2.8	1.5	2.8	SPNS2	Homo sapiens spinster homolog 2 (Drosophila) (SPNS2), mRNA [NM_001124758]	124976

A_23_P100963	-1.8	-1.0	-2.7	-1.4	SPNS3	Homo sapiens spinster homolog 3 (Drosophila) (SPNS3), mRNA [NM_182538]	201305
A_23_P166027	1.7	1.7	1.7	-4.6	SPO11	Homo sapiens SPO11 meiotic protein covalently bound to DSB homolog (S. cerevisiae) (SPO11), transcript variant 1, mRNA [NM_012444]	23626
A_23_P161280	4.6	3.1	6.0	2.5	SPOCK2	Homo sapiens sparc/osteonectin, cwcv and kazal-like domains proteoglycan (testican) 2 (SPOCK2), transcript variant 2, mRNA [NM_014767]	9806

A_24_P270890	-1.0	-2.1	-3.7	-1.0	SPOP	Homo sapiens speckle-type POZ protein (SPOP), transcript variant 1, mRNA [NM_001007226]	8405
A_23_P107257	4.5	6.3	4.5	2.9	SPOP	Homo sapiens speckle-type POZ protein (SPOP), transcript variant 1, mRNA [NM_001007226]	8405
A_23_P79066	-1.1	-1.5	-2.9	-5.2	SPPL2B	Homo sapiens signal peptide peptidase-like 2B (SPPL2B), transcript variant 3, mRNA [NM_001077238]	56928
A_32_P161447	1.3	-2.8	-1.1	1.1	SPPL2B	Homo sapiens signal peptide peptidase-like 2B (SPPL2B), transcript variant 3, mRNA [NM_001077238]	56928

A_24_P136838	1.1	-2.7	-1.1	-1.0	SPPL3	Homo sapiens mRNA; cDNA DKFZp547 P1310 (from clone DKFZp547 P1310) [AL834399]	121665
A_23_P427622	1.1	1.0	2.1	-1.0	SPPL3	Homo sapiens signal peptide peptidase-like 3 (SPPL3), mRNA [NM_1390 15]	121665
A_24_P299308	5.8	5.8	1.8	4.8	SPPL3	Homo sapiens cDNA FLJ39830 fis, clone SPLEN201 2846. [AK097149]	121665
A_23_P162559	2.5	1.8	2.3	2.2	SPPL3	Homo sapiens signal peptide peptidase-like 3 (SPPL3), mRNA [NM_1390 15]	121665

A_23_P68211	1.1	3.3	1.0	1.0	SPR	Homo sapiens sepiapterin reductase (7,8-dihydrobiopterin:NADP+ oxidoreductase) (SPR), mRNA [NM_003124]	6697
A_32_P225854	1.0	-1.0	4.2	-1.0	SPRED2	Homo sapiens sprouty-related, EVH1 domain containing 2 (SPRED2), transcript variant 1, mRNA [NM_181784]	200734
A_24_P930415	2.0	2.0	2.1	1.1	SPRN	Homo sapiens shadow of prion protein homolog (zebrafish) (SPRN), mRNA [NM_001012508]	503542

A_23_P159406	3.0	3.5	1.4	1.9	SPRR1B	Homo sapiens small proline-rich protein 1B (SPRR1B), mRNA [NM_003125]	6699
A_23_P11644	-3.7	1.6	-1.1	-1.5	SPRR2D	Homo sapiens small proline-rich protein 2D (SPRR2D), mRNA [NM_006945]	6703
A_23_P148949	8.7	3.4	1.9	4.4	SPRR2E	Homo sapiens small proline-rich protein 2E (SPRR2E), mRNA [NM_001024209]	6704
A_23_P311010	-2.9	1.0	1.9	1.1	SPRY3	Homo sapiens sprouty homolog 3 (Drosophila) (SPRY3), mRNA [NM_005840]	10251

A_23_P65129	-1.5	-4.3	-7.4	1.3	SPRYD3	Homo sapiens SPRY domain containing 3 (SPRYD3), mRNA [NM_032840]	84926
A_24_P116233	-2.1	1.1	1.0	1.8	SPRYD7	Homo sapiens SPRY domain containing 7 (SPRYD7), transcript variant 1, mRNA [NM_020456]	57213
A_23_P200096	2.3	2.6	1.6	2.1	SPSB1	Homo sapiens splA/ryanodine receptor domain and SOCS box containing 1 (SPSB1), mRNA [NM_025106]	80176

A_23_P151098	-2.9	-1.1	1.3	1.6	SPSB2	Homo sapiens splA/ryanodine receptor domain and SOCS box containing 2 (SPSB2), transcript variant 1, mRNA [NM_032641]	84727
A_23_P3496	-4.2	1.0	-1.3	-1.4	SPSB3	Homo sapiens splA/ryanodine receptor domain and SOCS box containing 3 (SPSB3), mRNA [NM_080861]	90864
A_23_P330537	2.2	1.3	1.5	1.0	SPSB3	Homo sapiens splA/ryanodine receptor domain and SOCS box containing 3 (SPSB3), mRNA [NM_080861]	90864

A_23_P258381	7.5	4.4	1.1	-5.7	SPSB4	Homo sapiens splA/ryanodine receptor domain and SOCS box containing 4 (SPSB4), mRNA [NM_080862]	92369
A_32_P134968	-1.4	-3.7	1.2	-1.9	SPTB	Homo sapiens spectrin, beta, erythrocytic (SPTB), transcript variant 1, mRNA [NM_001024858]	6710
A_23_P339095	1.0	1.0	1.0	-3.3	SPTBN1	Homo sapiens spectrin, beta, non-erythrocytic 1 (SPTBN1), transcript variant 2, mRNA [NM_178313]	6711
A_23_P102523	4.5	2.5	4.1	3.6	SPTBN1	Homo sapiens spectrin, beta, non-erythrocytic 1 (SPTBN1), transcript variant 1, mRNA [NM_003128]	6711

A_23_P98282	2.3	2.2	1.0	1.1	SPTBN2	Homo sapiens spectrin, beta, non-erythrocytic 2 (SPTBN2), mRNA [NM_006946]	6712
A_24_P293114	2.5	1.5	3.0	1.7	SPTBN4	Homo sapiens spectrin, beta, non-erythrocytic 4 (SPTBN4), transcript variant sigma1, mRNA [NM_020971]	57731
A_24_P238420	1.9	1.9	2.7	1.0	SPTBN4	Homo sapiens spectrin, beta, non-erythrocytic 4 (SPTBN4), transcript variant sigma5, mRNA [NM_025213]	57731
A_23_P152082	1.7	1.2	-6.0	-1.2	SPTBN5	Homo sapiens spectrin, beta, non-erythrocytic 5 (SPTBN5), mRNA [NM_016642]	51332

A_24_P150486	-3.6	1.6	1.0	-2.0	SPTLC2	Homo sapiens serine palmitoyltransferase, long chain base subunit 2 (SPTLC2), mRNA [NM_004863]	9517
A_24_P202857	1.6	-2.6	-2.6	-1.4	SPTLC3	serine palmitoyltransferase, long chain base subunit 3 [Source:HGNC Symbol;Acc:16253] [ENST00000476791]	55304
A_24_P49260	-13.8	1.0	1.0	1.1	SPTLC3	Homo sapiens serine palmitoyltransferase, long chain base subunit 3 (SPTLC3), mRNA [NM_018327]	55304

A_24_P40721	1.3	1.3	2.1	1.6	SPTLC3	Homo sapiens serine palmitoyltransferase, long chain base subunit 3 (SPTLC3), mRNA [NM_018327]	55304
A_32_P199551	-3.7	-2.4	-3.5	1.1	SPTSSB	Homo sapiens serine palmitoyltransferase, small subunit B (SPTSSB), mRNA [NM_001040100]	165679
A_24_P20753	-2.2	1.1	-1.0	-1.2	SRA1	Homo sapiens steroid receptor RNA activator 1 (SRA1), mRNA [NM_001035235]	10011
A_23_P394917	-1.0	2.5	-1.1	-1.0	SRCAP	Homo sapiens Snf2-related CREBBP activator protein (SRCAP), mRNA [NM_006662]	10847

A_24_P248083	3.9	3.9	1.9	6.0	SRCIN1	Homo sapiens SNAP25-interacting protein, mRNA (cDNA clone IMAGE:5423326), complete cds. [BC033233]	80725
A_23_P8571	2.1	5.0	1.6	2.5	SRCRB4D	Homo sapiens scavenger receptor cysteine rich domain containing, group B (4 domains) (SRCRB4D), mRNA [NM_080744]	136853
A_23_P30223	-1.0	-1.5	-1.0	-2.1	SRD5A1	Homo sapiens steroid-5-alpha-reductase, alpha polypeptide 1 (3-oxo-5 alpha-steroid delta 4-dehydrogenase alpha 1) (SRD5A1), mRNA [NM_001047]	6715

A_23_P121783	1.2	1.1	1.1	-2.5	SRD5A3	Homo sapiens steroid 5 alpha-reductase 3 (SRD5A3), mRNA [NM_024592]	79644
A_23_P129786	1.4	1.4	2.7	1.1	SREBF1	Homo sapiens sterol regulatory element binding transcription factor 1 (SREBF1), transcript variant 1, mRNA [NM_001005291]	6720
A_23_P166502	-1.0	-1.1	-3.9	-1.1	SREBF2	sterol regulatory element binding transcription factor 2 [Source:HGNC Symbol;Acc:11290] [ENST00000361204]	6721

A_24_P944991	-2.4	1.6	1.5	1.1	SREK1IP1	Homo sapiens SREK1-interacting protein 1 (SREK1IP1), mRNA [NM_173829]	285672
A_32_P198330	4.4	4.5	4.4	-3.0	SREK1IP1	Homo sapiens SREK1-interacting protein 1 (SREK1IP1), mRNA [NM_173829]	285672
A_23_P111020	-2.1	-10.4	-1.6	-2.4	SRF	Homo sapiens serum response factor (c-fos serum response element-binding transcription factor) (SRF), mRNA [NM_003131]	6722

A_24_P346277	-1.7	-1.3	-1.4	-2.9	SRF	Homo sapiens serum response factor (c-fos serum response element-binding transcription factor) (SRF), mRNA [NM_003131]	6722
A_32_P70056	-1.0	-1.1	-2.7	-1.1	SRG7	Homo sapiens spermatogenesis-related protein 7 (SRG7), non-coding RNA [NR_034168]	642864
A_23_P162449	1.0	-10.7	-1.0	-1.1	SRGAP1	Homo sapiens SLIT-ROBO Rho GTPase activating protein 1 (SRGAP1), mRNA [NM_020762]	57522

A_23_P200829	-2.9	-1.2	1.3	-2.7	SRGAP2	Homo sapiens SLIT-ROBO Rho GTPase activating protein 2 (SRGAP2), transcript variant 1, mRNA [NM_015326]	23380
A_23_P358410	2.1	2.3	2.1	1.9	SRGAP3	Homo sapiens SLIT-ROBO Rho GTPase activating protein 3 (SRGAP3), transcript variant 1, mRNA [NM_014850]	9901
A_24_P153869	1.0	-1.1	-7.6	-2.5	SRGAP3	SLIT-ROBO Rho GTPase activating protein 3 [Source:HGNC Symbol;Acc:19744] [ENST00000544908]	9901
A_23_P59718	1.2	-1.0	-3.0	-1.0	SRI	Homo sapiens sorcin (SRI), transcript variant 1, mRNA [NM_003130]	6717

A_23_P74269	3.8	1.8	-1.5	1.4	SRM	Homo sapiens spermidine synthase (SRM), mRNA [NM_003132]	6723
A_23_P68380	1.9	-3.6	5.6	1.8	SRMS	Homo sapiens src-related kinase lacking C-terminal regulatory tyrosine and N-terminal myristylation sites (SRMS), mRNA [NM_080823]	6725
A_23_P81612	-1.7	1.0	-4.6	1.1	SRP19	Homo sapiens signal recognition particle 19kDa (SRP19), transcript variant 1, mRNA [NM_003135]	6728

A_24_P388810	1.2	1.1	1.1	-3.5	SRP19	Homo sapiens signal recognition particle 19kDa (SRP19), transcript variant 1, mRNA [NM_003135]	6728
A_32_P151594	1.0	1.0	-1.1	-3.6	SRP9	Homo sapiens signal recognition particle 9kDa (SRP9), transcript variant 2, mRNA [NM_003133]	6726
A_23_P19543	1.1	1.1	-2.5	5.2	SRPK1	Homo sapiens SRSF protein kinase 1 (SRPK1), transcript variant 1, mRNA [NM_003137]	6732
A_23_P215599	1.3	-1.0	-1.9	2.4	SRPK2	Homo sapiens SRSF protein kinase 2 (SRPK2), transcript variant 2, mRNA [NM_182691]	6733

A_23_P125772	1.3	-3.6	-5.6	-1.2	SRPK3	Homo sapiens SRSF protein kinase 3 (SRPK3), transcript variant 1, mRNA [NM_014370]	26576
A_23_P86943	-3.0	-3.0	-11.5	-3.2	SRPR	Homo sapiens signal recognition particle receptor (docking protein) (SRPR), transcript variant 1, mRNA [NM_003139]	6734
A_23_P80773	-1.4	-3.2	2.5	1.7	SRPRB	Homo sapiens signal recognition particle receptor, B subunit (SRPRB), mRNA [NM_021203]	58477
A_23_P136978	-4.3	1.0	1.0	1.3	SRPX2	Homo sapiens sushi-repeat containing protein, X-linked 2 (SRPX2), mRNA [NM_014467]	27286

A_23_P141248	1.1	1.2	-3.6	1.2	SRR	Homo sapiens serine racemase (SRR), mRNA [NM_021947]	63826
A_24_P288954	1.3	1.3	-3.8	-1.0	SRRD	Homo sapiens SRR1 domain containing (SRRD), mRNA [NM_001013694]	402055
A_24_P11131	1.0	1.0	1.0	2.6	SRRM1	Homo sapiens serine/arginine repetitive matrix 1 (SRRM1), mRNA [NM_005839]	10250
A_23_P323166	-1.2	-1.2	-3.6	1.1	SRRM2	Homo sapiens serine/arginine repetitive matrix 2 (SRRM2), mRNA [NM_016333]	23524

A_23_P111481	1.2	-2.5	1.1	-1.0	SRRT	Homo sapiens serrate RNA effector molecule homolog (Arabidopsis) (SRRT), transcript variant 1, mRNA [NM_015908]	51593
A_24_P383080	-7.1	-8.1	-2.0	1.9	SRRT	Homo sapiens serrate RNA effector molecule homolog (Arabidopsis) (SRRT), transcript variant 4, mRNA [NM_001128853]	51593
A_23_P111487	1.7	1.3	2.9	1.0	SRRT	Homo sapiens serrate RNA effector molecule homolog (Arabidopsis) (SRRT), transcript variant 4, mRNA [NM_001128853]	51593

A_23_P49521	-2.5	-3.8	-2.6	1.0	SRSF1	Homo sapiens serine/arginine-rich splicing factor 1 (SRSF1), transcript variant 2, mRNA [NM_001078166]	6426
A_23_P110903	2.9	2.9	1.3	1.2	SRSF12	Homo sapiens serine/arginine-rich splicing factor 12 (SRSF12), mRNA [NM_080743]	135295
A_23_P413761	1.5	1.8	2.1	1.5	SRSF3	Homo sapiens serine/arginine-rich splicing factor 3 (SRSF3), transcript variant 1, mRNA [NM_003017]	6428
A_23_P109017	-1.1	-1.4	1.2	4.1	SRSF6	Homo sapiens serine/arginine-rich splicing factor 6 (SRSF6), transcript variant 1, mRNA [NM_006275]	6431

A_24_P222911	-1.3	-1.1	-2.8	-1.1	SRSF7	Homo sapiens serine/arginine-rich splicing factor 7 (SRSF7), transcript variant 1, mRNA [NM_001031684]	6432
A_23_P363426	2.1	2.1	1.9	2.0	SRSF8	Homo sapiens serine/arginine-rich splicing factor 8 (SRSF8), mRNA [NM_032102]	10929
A_24_P390070	-1.9	-2.2	-2.1	-1.0	SRSF9	Homo sapiens serine/arginine-rich splicing factor 9 (SRSF9), mRNA [NM_003769]	8683
A_23_P141738	-1.0	-1.1	1.2	-16.0	SS18	Homo sapiens synovial sarcoma translocation, chromosome 18 (SS18), transcript variant 1, mRNA [NM_001007559]	6760

A_24_P384851	1.7	2.3	2.2	-1.1	SS18	Homo sapiens synovial sarcoma translocation, chromosome 18 (SS18), transcript variant 1, mRNA [NM_001007559]	6760
A_23_P423926	-4.4	-12.2	-1.0	-1.6	SS18L1	Homo sapiens synovial sarcoma translocation gene on chromosome 18-like 1 (SS18L1), mRNA [NM_198935]	26039
A_23_P154025	1.3	1.7	-3.4	-1.8	SSB	Homo sapiens Sjogren syndrome antigen B (autoantigen La) (SSB), mRNA [NM_003142]	6741

A_23_P33791	-5.8	-1.9	-2.5	-2.8	SSBP2	Homo sapiens single-stranded DNA binding protein 2 (SSBP2), mRNA [NM_012446]	23635
A_24_P375453	1.0	1.1	2.2	-2.2	SSBP3	Homo sapiens single stranded DNA binding protein 3 (SSBP3), transcript variant 3, mRNA [NM_001009955]	23648
A_23_P500333	5.4	-1.1	1.1	-1.0	SSBP3	Homo sapiens single stranded DNA binding protein 3 (SSBP3), transcript variant 3, mRNA [NM_001009955]	23648

A_24_P159916	1.5	2.5	1.3	-1.2	SSBP4	Homo sapiens single stranded DNA binding protein 4 (SSBP4), transcript variant 1, mRNA [NM_032627]	170463
A_24_P170717	2.0	1.4	2.4	1.6	SSC5D	Homo sapiens cDNA FLJ35258 fis, clone PROST2004146. [AK092577]	284297
A_24_P920823	1.9	1.3	2.2	1.6	SSFA2	Homo sapiens sperm specific antigen 2 (SSFA2), transcript variant 2, mRNA [NM_006751]	6744
A_24_P332647	-3.0	-1.0	-1.0	-2.0	SSH1	Homo sapiens slingshot homolog 1 (Drosophila) (SSH1), transcript variant 3, mRNA [NM_001161331]	54434

A_23_P395954	1.2	1.4	1.3	-3.6	SSH2	slingshot homolog 2 (Drosophila) [Source:HGNC Symbol;Acc:30580] [ENST00000394848]	85464
A_23_P118712	50.0	47.7	43.3	32.9	SSH2	slingshot homolog 2 (Drosophila) [Source:HGNC Symbol;Acc:30580] [ENST00000324677]	85464
A_23_P353048	2.4	1.7	1.2	-1.4	SSPO	Homo sapiens SCO-spondin homolog (Bos taurus) (SSPO), mRNA [NM_198455]	23145
A_32_P89755	1.1	-1.1	-3.1	-1.9	SSR1	Homo sapiens signal sequence receptor, alpha (SSR1), mRNA [NM_003144]	6745

A_23_P126474	-1.0	-1.1	-1.1	-2.3	SSR2	Homo sapiens signal sequence receptor, beta (translocon-associated protein beta) (SSR2), mRNA [NM_003145]	6746
A_23_P259172	-2.0	-1.1	-2.8	-2.1	SSR4	Homo sapiens signal sequence receptor, delta (SSR4), transcript variant 2, mRNA [NM_006280]	6748
A_23_P98261	1.7	1.6	-1.5	-3.3	SSSCA1	Homo sapiens Sjogren syndrome/scleroderma autoantigen 1 (SSSCA1), mRNA [NM_006396]	10534
A_23_P318800	-5.2	-1.9	-4.0	-2.8	SSTR2	Homo sapiens somatostatin receptor 2 (SSTR2), mRNA [NM_001050]	6752

A_23_P68910	1.2	-3.0	1.1	1.3	SSTR3	Homo sapiens somatostatin receptor 3 (SSTR3), mRNA [NM_001051]	6753
A_23_P419582	-4.3	-4.5	1.4	-1.4	SSTR4	Homo sapiens somatostatin receptor 4 (SSTR4), mRNA [NM_001052]	6754
A_23_P77545	-1.2	-35.2	-1.4	-1.4	SSTR5	Homo sapiens somatostatin receptor 5 (SSTR5), transcript variant 1, mRNA [NM_001053]	6755
A_23_P307002	2.1	-1.8	1.3	1.3	SSU72	SSU72 RNA polymerase II CTD phosphatase homolog (S. cerevisiae) [Source:HGNC Symbol;Acc:25016] [ENST00000359060]	29101

A_23_P63289	2.6	2.6	2.5	2.8	SSU72	Homo sapiens SSU72 RNA polymerase II CTD phosphatase homolog (S. cerevisiae) (SSU72), mRNA [NM_014188]	29101
A_23_P252023	1.8	1.8	-1.7	-2.5	SSX2	Homo sapiens synovial sarcoma, X breakpoint 2 (SSX2), transcript variant 2, mRNA [NM_175698]	6757
A_23_P84359	1.8	1.9	3.0	-21.3	SSX5	Homo sapiens synovial sarcoma, X breakpoint 5 (SSX5), transcript variant 1, mRNA [NM_021015]	6758

A_32_P44657	-16.1	-1.0	-1.1	1.6	ST13	Homo sapiens suppression of tumorigenicity 13 (colon carcinoma) (Hsp70 interacting protein) (ST13), mRNA [NM_003932]	6767
A_32_P167791	1.1	1.1	1.0	-2.5	ST13	Homo sapiens suppression of tumorigenicity 13 (colon carcinoma) (Hsp70 interacting protein) (ST13), mRNA [NM_003932]	6767
A_32_P50431	3.5	1.1	-1.0	1.0	ST13	Homo sapiens suppression of tumorigenicity 13 (colon carcinoma) (Hsp70 interacting protein) (ST13), mRNA [NM_003932]	6767

A_24_P127021	3.5	1.6	2.4	1.0	ST13	Homo sapiens suppression of tumorigenicity 13 (colon carcinoma) (Hsp70 interacting protein) (ST13), mRNA [NM_003932]	6767
A_23_P47282	1.1	-2.2	1.8	-1.3	ST14	Homo sapiens suppression of tumorigenicity 14 (colon carcinoma) (ST14), mRNA [NM_021978]	6768
A_23_P31858	1.0	-1.0	-5.0	-1.0	ST18	Homo sapiens suppression of tumorigenicity 18 (breast carcinoma) (zinc finger protein) (ST18), mRNA [NM_014682]	9705

A_23_P77623	-2.4	2.2	-1.1	2.7	ST3GAL2	Homo sapiens ST3 beta-galactoside alpha-2,3-sialyltransferase 2 (ST3GAL2), mRNA [NM_006927]	6483
A_24_P268123	3.8	-2.6	-1.0	-2.7	ST3GAL3	Homo sapiens ST3 beta-galactoside alpha-2,3-sialyltransferase 3 (ST3GAL3), transcript variant 1, mRNA [NM_174963]	6487
A_23_P200999	2.7	2.9	2.1	-8.4	ST3GAL3	Homo sapiens ST3 beta-galactoside alpha-2,3-sialyltransferase 3 (ST3GAL3), transcript variant 1, mRNA [NM_174963]	6487

A_23_P393856	1.0	-1.0	-1.0	-3.0	ST3GAL4	Homo sapiens cDNA FLJ11867 fis, clone HEMBA1006976, weakly similar to H.sapiens mRNA for Gal-beta(1-3/1-4)GlcNAc alpha-2.3-sialyltransferase. [AK021929]	6484
A_24_P181055	-1.3	-1.4	-3.9	-1.9	ST3GAL4	Homo sapiens ST3 beta-galactoside alpha-2,3-sialyltransferase 4 (ST3GAL4), mRNA [NM_006278]	6484
A_23_P136573	1.1	1.0	-1.0	-3.4	ST3GAL5	Homo sapiens ST3 beta-galactoside alpha-2,3-sialyltransferase 5 (ST3GAL5), transcript variant 1, mRNA [NM_003896]	8869

A_32_P126157	-10.1	1.1	1.0	1.1	ST6GAL2	Homo sapiens ST6 beta-galactosamide alpha-2,6-sialyltranferase 2 (ST6GAL2), transcript variant 1, mRNA [NM_032528]	84620
A_23_P54968	-3.2	-3.1	-1.1	-4.7	ST6GALNAC1	Homo sapiens ST6 (alpha-N-acetyl-neuraminyl-2,3-beta-galactosyl-1,3)-N-acetylgalactosaminide alpha-2,6-sialyltransferase 1 (ST6GALNAC1), mRNA [NM_018414]	55808

A_23_P49499	1.3	1.8	1.1	-2.2	ST6GALNAC2	Homo sapiens ST6 (alpha-N-acetyl-neuraminyl-2,3-beta-galactosyl-1,3)-N-acetylgalactosaminide alpha-2,6-sialyltransferase 2 (ST6GALNAC2), mRNA [NM_006456]	10610
A_23_P416711	1.3	-1.9	-3.5	-1.0	ST6GALNAC3	Homo sapiens ST6 (alpha-N-acetyl-neuraminyl-2,3-beta-galactosyl-1,3)-N-acetylgalactosaminide alpha-2,6-sialyltransferase 3 (ST6GALNAC3), transcript variant 1, mRNA [NM_152996]	256435

A_23_P33093	-46.3	1.5	1.5	1.5	ST6GALNAC5	Homo sapiens ST6 (alpha-N-acetyl-neuraminyl-2,3-beta-galactosyl-1,3)-N-acetylgalactosaminide alpha-2,6-sialyltransferase 5 (ST6GALNAC5), mRNA [NM_030965]	81849
A_24_P141019	-1.1	1.8	2.0	1.9	ST7	Homo sapiens suppression of tumorigenicity 7 (ST7), transcript variant a, mRNA [NM_018412]	7982
A_23_P310582	-6.4	1.1	-1.1	1.0	ST7L	Homo sapiens suppression of tumorigenicity 7 like (ST7L), transcript variant 4, mRNA [NM_138729]	54879

A_23_P354705	1.0	1.0	-3.3	-7.6	ST8SIA1	Homo sapiens ST8 alpha-N-acetyl-neuraminide alpha-2,8-sialyltransferase 1 (ST8SIA1), mRNA [NM_003034]	6489
A_24_P664850	1.5	1.7	1.6	2.6	ST8SIA2	Homo sapiens ST8 alpha-N-acetyl-neuraminide alpha-2,8-sialyltransferase 2 (ST8SIA2), mRNA [NM_006011]	8128
A_23_P117805	1.8	1.9	2.0	2.3	ST8SIA2	Homo sapiens ST8 alpha-N-acetyl-neuraminide alpha-2,8-sialyltransferase 2 (ST8SIA2), mRNA [NM_006011]	8128

A_32_P154021	1.8	1.9	2.1	-2.6	ST8SIA3	Homo sapiens ST8 alpha-N-acetyl-neuraminide alpha-2,8-sialyltransferase 3 (ST8SIA3), mRNA [NM_015879]	51046
A_23_P78392	-1.3	-1.9	-1.4	-5.1	ST8SIA3	Homo sapiens ST8 alpha-N-acetyl-neuraminide alpha-2,8-sialyltransferase 3 (ST8SIA3), mRNA [NM_015879]	51046
A_23_P379789	1.0	-1.0	-2.1	-1.2	ST8SIA5	Homo sapiens ST8 alpha-N-acetyl-neuraminide alpha-2,8-sialyltransferase 5 (ST8SIA5), mRNA [NM_013305]	29906
A_23_P32500	2.6	2.6	2.1	-1.5	STAB1	Homo sapiens stabilin 1 (STAB1), mRNA [NM_015136]	23166

A_23_P162607	-1.2	-1.5	-1.1	-3.0	STAB2	Homo sapiens stabilin 2 (STAB2), mRNA [NM_017564]	55576
A_23_P121061	-1.4	-1.8	-2.3	-1.8	STAC	Homo sapiens SH3 and cysteine rich domain (STAC), mRNA [NM_003149]	6769
A_32_P152195	-1.2	1.0	-1.0	3.3	STAC2	Homo sapiens SH3 and cysteine rich domain 2 (STAC2), mRNA [NM_198993]	342667
A_24_P374962	1.5	-1.1	-2.6	1.3	STAG3L2	Homo sapiens stromal antigen 3-like 2 (STAG3L2), non-coding RNA [NR_040584]	442582

A_32_P98927	-1.0	-1.0	-1.0	-3.2	STAG3L4	Homo sapiens stromal antigen 3-like 4 (STAG3L4), transcript variant 1, non-coding RNA [NR_040585]	64940
A_23_P111480	-3.8	-1.9	-2.2	-3.3	STAG3L4	Homo sapiens stromal antigen 3-like 4 (STAG3L4), transcript variant 1, non-coding RNA [NR_040585]	64940
A_23_P1343	1.0	-2.3	-1.1	-1.0	STAM	Homo sapiens signal transducing adaptor molecule (SH3 domain and ITAM motif) 1 (STAM), transcript variant 1, mRNA [NM_003473]	8027

A_23_P28559	31.5	30.8	14.1	31.2	STAMBP	Homo sapiens STAM binding protein (STAMBP), transcript variant 3, mRNA [NM_213622]	10617
A_23_P385322	2.3	1.9	2.6	3.3	STAMBPL1	Homo sapiens STAM binding protein-like 1 (STAMBPL1), mRNA [NM_020799]	57559
A_23_P382188	2.3	1.5	1.7	1.5	STAP2	Homo sapiens signal transducing adaptor family member 2 (STAP2), transcript variant 2, mRNA [NM_001013841]	55620

A_23_P36345	3.0	1.9	1.1	-1.1	STARD10	Homo sapiens StAR-related lipid transfer (START) domain containing 10 (STARD10), mRNA [NM_006645]	10809
A_23_P158880	1.0	-3.5	1.0	1.0	STARD5	Homo sapiens StAR-related lipid transfer (START) domain containing 5 (STARD5), mRNA [NM_181900]	80765
A_23_P112921	1.1	1.0	-2.2	-1.8	STARD5	Homo sapiens StAR-related lipid transfer (START) domain containing 5 (STARD5), mRNA [NM_181900]	80765

A_32_P130641	1.3	1.2	2.4	1.1	STARD9	Homo sapiens StAR-related lipid transfer (START) domain containing 9 (STARD9), mRNA [NM_020759]	57519
A_23_P76090	10.7	3.9	9.6	6.1	STAT2	Homo sapiens signal transducer and activator of transcription 2, 113kDa (STAT2), transcript variant 1, mRNA [NM_005419]	6773

A_24_P116805	-1.3	1.5	-2.5	1.2	STAT3	Homo sapiens signal transducer and activator of transcription 3 (acute-phase response factor) (STAT3), transcript variant 3, mRNA [NM_213662]	6774
A_23_P68031	1.2	1.2	-2.7	1.0	STAT4	Homo sapiens signal transducer and activator of transcription 4 (STAT4), transcript variant 1, mRNA [NM_003151]	6775
A_23_P207367	1.3	1.3	2.2	1.1	STAT5A	Homo sapiens signal transducer and activator of transcription 5A (STAT5A), mRNA [NM_003152]	6776

A_23_P100788	-1.0	-2.0	-1.0	3.2	STAT5B	Homo sapiens signal transducer and activator of transcription 5B (STAT5B), mRNA [NM_012448]	6777
A_24_P342178	2.2	-3.5	2.7	1.2	STAT5B	signal transducer and activator of transcription 5B [Source:HGNC Symbol;Acc:11367] [ENST00000468312]	6777
A_23_P57059	-2.6	-2.7	-1.2	-1.1	STAU1	Homo sapiens staufen, RNA binding protein, homolog 1 (Drosophila) (STAU1), transcript variant T3, mRNA [NM_017453]	6780

A_32_P649	-11.6	1.9	1.2	1.6	STAU2	Homo sapiens staufen, RNA binding protein, homolog 2 (Drosophila) (STAU2), transcript variant 5, mRNA [NM_014393]	27067
A_24_P914830	1.0	-3.4	-1.1	-1.4	STAU2	staufen, RNA binding protein, homolog 2 (Drosophila) [Source:HGNC Symbol;Acc:11371] [ENST00000524104]	27067
A_23_P254079	5.1	5.1	5.2	5.2	STBD1	Homo sapiens starch binding domain 1 (STBD1), mRNA [NM_003943]	8987
A_23_P314755	4.9	4.9	5.0	4.9	STC1	Homo sapiens stanniocalcin 1 (STC1), mRNA [NM_003155]	6781

A_23_P416395	1.1	-1.5	-5.8	-1.3	STC2	Homo sapiens stanniocalcin 2 (STC2), mRNA [NM_003714]	8614
A_23_P110686	-4.6	1.1	-2.3	-1.0	STC2	Homo sapiens stanniocalcin 2 (STC2), mRNA [NM_003714]	8614
A_23_P31453	1.2	2.8	2.8	2.9	STEAP1	Homo sapiens six transmembrane epithelial antigen of the prostate 1 (STEAP1), mRNA [NM_012449]	26872
A_24_P200000	5.8	5.8	2.7	2.9	STEAP3	Homo sapiens STEAP family member 3, metalloreductase (STEAP3), transcript variant 1, mRNA [NM_182915]	55240

A_32_P128980	-1.1	-2.4	1.1	1.2	STEAP4	STEAP family member 4 [Source:HGNC Symbol;Acc:21923] [ENST00000380079]	79689
A_24_P214231	2.2	2.2	1.2	2.6	STIL	Homo sapiens SCL/TAL1 interrupting locus (STIL), transcript variant 1, mRNA [NM_001048166]	6491
A_24_P180242	1.0	-4.5	1.0	1.0	STIM2	Homo sapiens stromal interaction molecule 2 (STIM2), transcript variant 2, mRNA [NM_020860]	57620
A_23_P81048	1.8	3.0	3.0	2.1	STIM2	Homo sapiens stromal interaction molecule 2 (STIM2), transcript variant 2, mRNA [NM_020860]	57620

A_24_P327815	-1.4	-2.1	-2.0	-1.9	STIP1	Homo sapiens stress-induced-phosphoprotein 1 (STIP1), mRNA [NM_006819]	10963
A_24_P46417	-1.6	-1.6	-1.3	-2.2	STK10	Homo sapiens serine/threonine kinase 10 (STK10), mRNA [NM_005990]	6793
A_24_P159866	-2.5	2.6	-1.0	2.1	STK11	Homo sapiens serine/threonine kinase 11 (STK11), mRNA [NM_000455]	6794
A_23_P16483	2.7	2.6	1.8	1.2	STK11	Homo sapiens serine/threonine kinase 11 (STK11), mRNA [NM_000455]	6794

A_24_P393322	2.5	-1.1	3.1	1.8	STK11IP	serine/threonine kinase 11 interacting protein [Source:HGNC Symbol;Acc:19184] [ENST00000475396]	114790
A_23_P108514	1.9	1.9	1.8	2.3	STK16	Homo sapiens serine/threonine kinase 16 (STK16), transcript variant 1, mRNA [NM_001008910]	8576
A_24_P337796	-3.3	1.3	1.2	1.1	STK17A	Homo sapiens serine/threonine kinase 17a (STK17A), mRNA [NM_004760]	9263
A_23_P19479	1.4	-2.2	-2.1	1.5	STK19	Homo sapiens serine/threonine kinase 19 (STK19), transcript variant 2, mRNA [NM_032454]	8859

A_23_P253177	-1.2	-2.4	-1.3	-1.2	STK24	Homo sapiens serine/threonine kinase 24 (STK24), transcript variant 2, mRNA [NM_001032296]	8428
A_24_P276983	2.3	-1.4	1.1	1.8	STK25	Homo sapiens serine/threonine kinase 25 (STK25), mRNA [NM_006374]	10494
A_23_P252653	1.8	3.3	3.9	1.0	STK25	Homo sapiens serine/threonine kinase 25 (STK25), mRNA [NM_006374]	10494
A_32_P118731	-1.6	-6.4	1.1	1.0	STK32A	serine/threonine kinase 32A [Source:HGNC Symbol;Acc:28317] [ENST00000397936]	202374

A_24_P171690	-1.3	1.0	-2.0	2.4	STK32B	Homo sapiens serine/threonine kinase 32B (STK32B), mRNA [NM_018401]	55351
A_24_P940537	1.0	-3.3	1.0	1.0	STK35	Homo sapiens serine/threonine kinase 35 (STK35), mRNA [NM_080836]	140901
A_23_P342600	3.3	3.5	1.1	1.9	STK35	Homo sapiens serine/threonine kinase 35 (STK35), mRNA [NM_080836]	140901
A_23_P335905	2.1	1.5	-1.4	-1.2	STK36	Homo sapiens serine/threonine kinase 36 (STK36), transcript variant 1, mRNA [NM_015690]	27148
A_23_P390704	-10.1	1.1	1.4	1.0	STK38	Homo sapiens serine/threonine kinase 38 (STK38), mRNA [NM_007271]	11329

A_24_P94054	-1.7	-3.4	-1.6	-3.1	STK4	Homo sapiens serine/threonine kinase 4 (STK4), mRNA [NM_006282]	6789
A_24_P342511	2.2	2.2	3.9	1.8	STMN1	Homo sapiens stathmin 1 (STMN1), transcript variant 4, mRNA [NM_001145454]	3925
A_23_P399265	2.0	1.1	1.9	-1.0	STMN2	SCG10=neuron-specific growth-associated protein/stathmin homolog [human, embryo, mRNA, 696 nt]. [S82024]	11075
A_24_P678104	2.3	1.9	2.5	1.3	STMN3	Homo sapiens stathmin-like 3 (STMN3), mRNA [NM_015894]	50861

A_23_P31739	1.6	1.5	-3.2	-1.8	STMN4	Homo sapiens stathmin-like 4 (STMN4), mRNA [NM_030795]	81551
A_23_P255263	2.4	2.7	2.4	-3.5	STOM	Homo sapiens stomatin (STOM), transcript variant 2, mRNA [NM_198194]	2040
A_24_P338145	-5.3	-1.3	-1.3	3.7	STOML2	Homo sapiens stomatin (EPB72)-like 2 (STOML2), mRNA [NM_013442]	30968
A_23_P302005	-1.0	-1.0	-3.6	1.1	STON1	Homo sapiens stonin 1 (STON1), transcript variant 2, mRNA [NM_006873]	11037
A_23_P309837	-1.8	-1.8	-1.9	-2.1	STON2	Homo sapiens stonin 2 (STON2), mRNA [NM_033104]	85439

A_32_P51119	1.2	-2.0	2.1	-2.4	STOX1	Homo sapiens storkhead box 1 (STOX1), transcript variant 1, mRNA [NM_152709]	219736
A_23_P344481	1.0	-1.1	-1.1	-6.9	STOX1	Homo sapiens storkhead box 1 (STOX1), transcript variant 1, mRNA [NM_152709]	219736
A_24_P65507	1.0	1.6	-3.1	-1.5	STRA13	Homo sapiens stimulated by retinoic acid 13 homolog (mouse) (STRA13), mRNA [NM_144998]	201254
A_23_P65779	2.1	2.5	-1.1	3.4	STRA6	Homo sapiens stimulated by retinoic acid gene 6 homolog (mouse) (STRA6), transcript variant 2, mRNA [NM_022369]	64220

A_24_P147407	-2.7	1.5	-1.8	-1.0	STRADA	Homo sapiens STE20-related kinase adaptor alpha (STRADA), transcript variant 3, mRNA [NM_153335]	92335
A_32_P177750	-9.9	1.8	1.5	-3.5	STRADB	Homo sapiens STE20-related kinase adaptor beta (STRADB), transcript variant 1, mRNA [NM_018571]	55437
A_23_P13852	-1.2	-2.0	-1.1	-1.0	STRAP	Homo sapiens serine/threonine kinase receptor associated protein (STRAP), mRNA [NM_007178]	11171

A_24_P916364	1.1	1.1	-4.0	-1.4	STRBP	Homo sapiens spermatid perinuclear RNA binding protein (STRBP), transcript variant 1, mRNA [NM_018387]	55342
A_24_P398016	1.1	-1.7	-2.9	-5.8	STRBP	Homo sapiens spermatid perinuclear RNA binding protein (STRBP), transcript variant 1, mRNA [NM_018387]	55342
A_23_P163238	-2.1	-1.6	-1.3	-2.6	STRC	Homo sapiens stereocilin (STRC), mRNA [NM_153700]	161497
A_23_P165521	-1.1	-1.1	-2.2	-1.2	STRN	Homo sapiens striatin, calmodulin binding protein (STRN), mRNA [NM_003162]	6801

A_32_P78904	-4.0	1.1	1.4	1.1	STRN	Homo sapiens striatin, calmodulin binding protein (STRN), mRNA [NM_003162]	6801
A_32_P92536	-1.0	-6.1	-1.0	-2.2	STRN	Homo sapiens striatin, calmodulin binding protein (STRN), mRNA [NM_003162]	6801
A_24_P331560	1.0	-1.0	-2.2	1.0	STS	Homo sapiens steroid sulfatase (microsomal), isozyme S (STS), mRNA [NM_000351]	412
A_23_P434473	-2.3	-1.9	-2.8	-1.9	STT3B	Homo sapiens STT3, subunit of the oligosaccharyltransferase complex, homolog B (S. cerevisiae) (STT3B), mRNA [NM_178862]	201595

A_23_P28084	-1.3	-2.5	1.3	1.6	STX10	syntaxin 10 [Source:HGNC Symbol;Acc:11428] [ENST00000343587]	8677
A_23_P156788	-1.1	4.3	3.3	1.3	STX11	Homo sapiens syntaxin 11 (STX11), mRNA [NM_003764]	8676
A_23_P385206	5.5	5.5	4.0	4.5	STX12	Homo sapiens syntaxin 12 (STX12), mRNA [NM_177424]	23673
A_24_P936122	7.4	10.9	8.4	-2.1	STX16	Homo sapiens syntaxin 16 (STX16), transcript variant 1, mRNA [NM_001001433]	8675
A_23_P168556	1.4	-1.2	-1.2	-7.1	STX1A	Homo sapiens syntaxin 1A (brain) (STX1A), transcript variant 1, mRNA [NM_004603]	6804

A_23_P256375	2.5	1.3	2.6	1.4	STX4	Homo sapiens syntaxin 4 (STX4), mRNA [NM_004604]	6810
A_23_P61646	-1.3	-2.3	-2.3	1.5	STX4	Homo sapiens syntaxin 4 (STX4), mRNA [NM_004604]	6810
A_23_P24755	1.6	2.6	-1.3	1.2	STX5	Homo sapiens syntaxin 5 (STX5), transcript variant 1, mRNA [NM_003164]	6811
A_23_P97725	1.3	-1.6	2.5	1.7	STX6	Homo sapiens syntaxin 6 (STX6), mRNA [NM_005819]	10228
A_23_P146908	-2.2	-2.8	-2.8	-2.4	STX8	Homo sapiens syntaxin 8 (STX8), transcript variant 1, mRNA [NM_004853]	9482

A_23_P130619	-2.0	1.3	1.2	-7.2	STXBP2	Homo sapiens syntaxin binding protein 2 (STXBP2), transcript variant 1, mRNA [NM_006949]	6813
A_23_P418785	1.1	-1.5	-3.0	1.1	STXBP5L	Homo sapiens syntaxin binding protein 5-like (STXBP5L), mRNA [NM_014980]	9515
A_23_P205713	-1.6	-2.3	-2.6	1.0	STXBP6	Homo sapiens syntaxin binding protein 6 (amisyn) (STXBP6), mRNA [NM_014178]	29091
A_23_P117540	-4.0	1.0	1.0	-1.2	STYX	Homo sapiens serine/threonine/tyrosine interacting protein (STYX), transcript variant 1, mRNA [NM_145251]	6815

A_24_P320328	1.4	1.4	-2.2	-2.3	SUB1	Homo sapiens SUB1 homolog (S. cerevisiae) (SUB1), mRNA [NM_006713]	10923
A_24_P181275	-1.8	-2.1	-1.5	1.2	SUCLA2	Homo sapiens succinate-CoA ligase, ADP-forming, beta subunit (SUCLA2), nuclear gene encoding mitochondrial protein, mRNA [NM_003850]	8803
A_23_P69171	-2.0	-2.0	-2.3	-2.7	SUCNR1	Homo sapiens succinate receptor 1 (SUCNR1), mRNA [NM_033050]	56670

A_23_P334990	-5.6	-1.0	-1.1	-1.0	SUDS3	Homo sapiens suppressor of defective silencing 3 homolog (S. cerevisiae) (SUDS3), mRNA [NM_022491]	64426
A_24_P149893	2.6	3.7	2.4	1.6	SUFU	Homo sapiens suppressor of fused homolog (Drosophila) (SUFU), transcript variant 1, mRNA [NM_016169]	51684
A_23_P16562	-2.9	-1.0	-1.1	-1.1	SUGP2	Homo sapiens SURP and G patch domain containing 2 (SUGP2), transcript variant 1, mRNA [NM_001017392]	10147

A_24_P280706	-1.0	-1.1	-1.2	-2.2	SUGT1	Homo sapiens SGT1, suppressor of G2 allele of SKP1 (S. cerevisiae) (SUGT1), transcript variant 1A, mRNA [NM_006704]	10910
A_23_P154605	-2.2	1.0	1.1	-1.1	SULF2	Homo sapiens sulfatase 2 (SULF2), transcript variant 1, mRNA [NM_018837]	55959
A_23_P106773	-1.8	-1.9	-6.0	-2.0	SULT1A2	Homo sapiens sulfotransferase family, cytosolic, 1A, phenol-preferring, member 2 (SULT1A2), transcript variant 2, mRNA [NM_177528]	6799

A_24_P262201	1.3	1.3	-3.0	1.7	SULT1A4	Homo sapiens sulfotransferase family, cytosolic, 1A, phenol-preferring, member 4 (SULT1A4), mRNA [NM_001017390]	445329
A_23_P302470	5.2	5.2	1.6	3.6	SULT1B1	Homo sapiens sulfotransferase family, cytosolic, 1B, member 1 (SULT1B1), mRNA [NM_014465]	27284
A_23_P155786	3.8	3.9	3.7	2.2	SULT1E1	Homo sapiens sulfotransferase family 1E, estrogen-preferring, member 1 (SULT1E1), mRNA [NM_005420]	6783

A_24_P224684	-6.5	1.2	1.1	1.1	SULT2A1	Homo sapiens sulfotransferase family, cytosolic, 2A, dehydroepiandrosterone (DHEA)-preferring, member 1 (SULT2A1), mRNA [NM_003167]	6822
A_23_P67381	1.2	-3.2	-1.1	1.1	SULT2A1	Homo sapiens sulfotransferase family, cytosolic, 2A, dehydroepiandrosterone (DHEA)-preferring, member 1 (SULT2A1), mRNA [NM_003167]	6822
A_23_P69242	1.0	-1.0	-1.1	3.2	SUMF1	Homo sapiens sulfatase modifying factor 1 (SUMF1), transcript variant 1, mRNA [NM_182760]	285362

A_23_P118435	-2.0	-1.1	-3.2	-1.9	SUMO2	Homo sapiens SMT3 suppressor of mif two 3 homolog 2 (S. cerevisiae) (SUMO2), transcript variant 1, mRNA [NM_006937]	6613
A_32_P228869	1.9	2.2	1.9	1.3	SUMO2	Homo sapiens SMT3 suppressor of mif two 3 homolog 2 (S. cerevisiae) (SUMO2), transcript variant 1, mRNA [NM_006937]	6613
A_23_P147070	1.1	1.0	-1.0	-15.2	SUN1	Sad1 and UNC84 domain containing 1 [Source:HGNC Symbol;Acc:18587] [ENST00000340926]	23353

A_24_P320645	-1.7	-2.6	1.6	-1.1	SUN1	Homo sapiens Sad1 and UNC84 domain containing 1 (SUN1), transcript variant 2, mRNA [NM_025154]	23353
A_23_P329962	1.0	-1.5	-3.5	-1.4	SUN3	Homo sapiens Sad1 and UNC84 domain containing 3 (SUN3), transcript variant 1, mRNA [NM_001030019]	256979
A_23_P109224	-3.7	2.1	-1.0	-1.0	SUN5	Sad1 and UNC84 domain containing 5 [Source:HGNC Symbol;Acc:16252] [ENST00000420875]	140732
A_23_P109229	-8.3	-1.9	-1.1	-5.2	SUN5	Homo sapiens Sad1 and UNC84 domain containing 5 (SUN5), mRNA [NM_080675]	140732

A_24_P153800	1.3	1.3	-2.1	-1.2	SUPT16H	Homo sapiens suppressor of Ty 16 homolog (S. cerevisiae) (SUPT16H), mRNA [NM_007192]	11198
A_24_P827	-30.0	1.1	1.0	1.0	SUPT3H	Homo sapiens suppressor of Ty 3 homolog (S. cerevisiae) (SUPT3H), transcript variant 1, mRNA [NM_003599]	8464
A_23_P163942	-1.0	-1.3	-1.2	-3.3	SUPT4H1	Homo sapiens suppressor of Ty 4 homolog 1 (S. cerevisiae) (SUPT4H1), mRNA [NM_003168]	6827

A_24_P1910	-1.0	-1.1	-2.1	-1.1	SUPT4H1	Homo sapiens suppressor of Ty 4 homolog 1 (S. cerevisiae) (SUPT4H1), mRNA [NM_003168]	6827
A_23_P27840	4.4	1.3	1.5	-1.0	SUPT5H	Homo sapiens suppressor of Ty 5 homolog (S. cerevisiae) (SUPT5H), transcript variant 1, mRNA [NM_003169]	6829
A_23_P141479	-3.2	2.0	-2.2	-1.6	SUPT6H	Homo sapiens suppressor of Ty 6 homolog (S. cerevisiae) (SUPT6H), mRNA [NM_003170]	6830

A_24_P126873	1.2	2.1	-1.5	1.6	SUPT6H	Homo sapiens suppressor of Ty 6 homolog (S. cerevisiae) (SUPT6H), mRNA [NM_003170]	6830
A_24_P282751	-1.0	-1.0	-1.1	-3.9	SUPT7L	Homo sapiens suppressor of Ty 7 (S. cerevisiae)-like (SUPT7L), mRNA [NM_014860]	9913
A_23_P63847	1.5	2.8	-1.3	-10.8	SUPV3L1	Homo sapiens suppressor of var1, 3-like 1 (S. cerevisiae) (SUPV3L1), mRNA [NM_003171]	6832
A_23_P20648	1.1	-11.3	-1.7	1.0	SURF1	Homo sapiens surfeit 1 (SURF1), nuclear gene encoding mitochondrial protein, mRNA [NM_003172]	6834

A_24_P89971	6.4	6.1	5.6	5.7	SURF4	Homo sapiens surfeit 4 (SURF4), mRNA [NM_033161]	6836
A_23_P32052	-4.7	-2.2	-1.4	-4.1	SURF4	Homo sapiens surfeit 4 (SURF4), mRNA [NM_033161]	6836
A_23_P422193	1.9	2.5	1.9	1.0	SUV39H1	Homo sapiens suppressor of variegation 3-9 homolog 1 (Drosophila) (SUV39H1), mRNA [NM_003173]	6839
A_24_P295709	2.9	2.8	2.5	2.6	SUV420H1	suppressor of variegation 4-20 homolog 1 (Drosophila) [Source:HGNC Symbol;Acc:24283] [ENST00000304363]	51111

A_24_P873263	-1.9	-1.8	-3.9	-2.5	SUZ12	Homo sapiens suppressor of zeste 12 homolog (Drosophila) (SUZ12), mRNA [NM_015355]	23512
A_32_P24223	-2.4	-4.5	-3.4	1.9	SUZ12	Homo sapiens suppressor of zeste 12 homolog (Drosophila) (SUZ12), mRNA [NM_015355]	23512
A_23_P23728	1.4	1.4	1.2	-2.6	SV2A	Homo sapiens synaptic vesicle glycoprotein 2A (SV2A), mRNA [NM_014849]	9900
A_24_P131236	-9.8	1.6	1.2	1.1	SV2A	Homo sapiens synaptic vesicle glycoprotein 2A (SV2A), mRNA [NM_014849]	9900

A_23_P100022	4.1	4.0	3.9	3.0	SV2B	Homo sapiens synaptic vesicle glycoprotein 2B (SV2B), transcript variant 1, mRNA [NM_014848]	9899
A_32_P199573	-2.3	-1.9	-2.0	-2.2	SV2C	synaptic vesicle glycoprotein 2C [Source:HGNC Symbol;Acc:30670] [ENST00000502798]	22987
A_23_P216596	-2.4	-1.3	-1.2	-2.6	SVEP1	Homo sapiens sushi, von Willebrand factor type A, EGF and pentraxin domain containing 1 (SVEP1), mRNA [NM_153366]	79987

A_23_P323178	2.5	2.9	1.2	2.0	SVEP1	sushi, von Willebrand factor type A, EGF and pentraxin domain containing 1 [Source:HGNC Symbol;Acc:15985] [ENST00000302728]	79987
A_24_P41276	-2.7	1.0	-5.2	1.0	SVEP1	Homo sapiens sushi, von Willebrand factor type A, EGF and pentraxin domain containing 1 (SVEP1), mRNA [NM_153366]	79987
A_23_P112982	1.4	1.4	-2.0	-1.0	SVOP	Homo sapiens SV2 related protein homolog (rat) (SVOP), mRNA [NM_018711]	55530

A_23_P307860	-1.4	2.4	1.2	-2.2	SVOPL	Homo sapiens SVOP-like (SVOPL), transcript variant 2, mRNA [NM_174959]	136306
A_32_P71551	1.2	-2.0	-2.8	1.0	SYCE1L	Homo sapiens synaptonemal complex central element protein 1-like (SYCE1L), mRNA [NM_001129979]	100130958
A_32_P203528	1.0	-9.4	-1.0	1.0	SYCE2	Homo sapiens synaptonemal complex central element protein 2 (SYCE2), mRNA [NM_001105578]	256126
A_24_P39928	-1.5	-2.1	1.4	-1.5	SYCN	Homo sapiens syncollin (SYCN), mRNA [NM_001080468]	342898

A_24_P333644	-7.1	-1.0	-1.0	-1.0	SYCP2	Homo sapiens synaptonemal complex protein 2 (SYCP2), mRNA [NM_014258]	10388
A_23_P347777	1.1	-1.7	-4.4	-1.0	SYCP2L	Homo sapiens synaptonemal complex protein 2-like (SYCP2L), mRNA [NM_001040274]	221711
A_24_P915300	-1.4	-1.3	-2.8	1.7	SYDE2	Homo sapiens synapse defective 1, Rho GTPase, homolog 2 (C. elegans) (SYDE2), mRNA [NM_032184]	84144
A_23_P33847	1.2	-1.0	-1.2	2.9	SYN1	Homo sapiens synapsin I (SYN1), transcript variant Ia, mRNA [NM_006950]	6853

A_23_P500824	3.0	1.9	1.3	1.2	SYN1	Homo sapiens synapsin I (SYN1), transcript variant Ib, mRNA [NM_133499]	6853
A_23_P80295	-1.3	-4.4	-1.1	-1.1	SYN3	Homo sapiens synapsin III (SYN3), transcript variant IIIa, mRNA [NM_003490]	8224
A_24_P940599	1.2	2.4	1.2	1.3	SYNCRIP	Homo sapiens synaptotagmin binding, cytoplasmic RNA interacting protein (SYNCRIP), transcript variant 6, mRNA [NM_001159677]	10492
A_24_P595237	-3.3	-1.0	-1.0	-1.0	SYNDIG1L	Homo sapiens synapse differentiation inducing 1-like (SYNDIG1L), mRNA [NM_001105579]	646658

A_24_P410453	1.0	1.3	-1.5	-3.5	SYNE1	Homo sapiens spectrin repeat containing, nuclear envelope 1 (SYNE1), transcript variant 2, mRNA [NM_033071]	23345
A_23_P500861	1.4	1.5	-3.0	-1.2	SYNE1	Homo sapiens spectrin repeat containing, nuclear envelope 1 (SYNE1), transcript variant 1, mRNA [NM_182961]	23345
A_23_P128887	-32.7	-3.7	1.0	-15.2	SYNE2	Homo sapiens spectrin repeat containing, nuclear envelope 2 (SYNE2), transcript variant 5, mRNA [NM_182914]	23224

A_24_P9346	1.1	1.1	2.0	1.1	SYNGAP1	Homo sapiens synaptic Ras GTPase activating protein 1 (SYNGAP1), mRNA [NM_006772]	8831
A_24_P347854	-5.6	-2.8	-4.8	-1.4	SYNGR2	Homo sapiens synaptogyrin 2 (SYNGR2), mRNA [NM_004710]	9144
A_23_P164927	2.4	1.1	2.5	1.4	SYNGR4	Homo sapiens synaptogyrin 4 (SYNGR4), mRNA [NM_012451]	23546
A_24_P201919	-1.8	-5.2	-6.9	-3.3	SYNJ2BP	Homo sapiens synaptojanin 2 binding protein (SYNJ2BP), mRNA [NM_018373]	55333
A_24_P405992	2.5	3.1	-1.1	3.7	SYNPO	Homo sapiens synaptopodin (SYNPO), transcript variant 1, mRNA [NM_007286]	11346

A_23_P344531	1.0	1.6	-1.0	-5.6	SYNPO	Homo sapiens synaptopodin (SYNPO), transcript variant 1, mRNA [NM_007286]	11346
A_23_P207541	-1.2	-2.2	-1.0	1.0	SYNRG	Homo sapiens synergin, gamma (SYNRG), transcript variant 1, mRNA [NM_007247]	11276
A_24_P141005	3.4	2.5	2.8	4.2	SYPL1	Homo sapiens synaptophysin-like 1 (SYPL1), transcript variant 2, mRNA [NM_182715]	6856
A_23_P93881	1.7	2.5	1.7	-1.1	SYPL1	Homo sapiens synaptophysin-like 1 (SYPL1), transcript variant 2, mRNA [NM_182715]	6856

A_23_P369456	1.1	1.0	-1.0	2.7	SYS1	Homo sapiens SYS1 Golgi-localized integral membrane protein homolog (S. cerevisiae) (SYS1), transcript variant 1, mRNA [NM_033542]	90196
A_23_P36795	-5.5	-1.2	-3.2	-1.4	SYT1	Homo sapiens synaptotagmin I (SYT1), transcript variant 1, mRNA [NM_005639]	6857
A_23_P161659	2.7	2.7	2.7	2.6	SYT13	Homo sapiens synaptotagmin XIII (SYT13), transcript variant 1, mRNA [NM_020826]	57586
A_24_P109069	-14.0	1.8	1.6	1.2	SYT15	Homo sapiens synaptotagmin XV (SYT15), transcript variant b, mRNA [NM_181519]	83849

A_23_P163697	2.8	2.6	2.5	3.2	SYT17	Homo sapiens synaptotagmin XVII (SYT17), mRNA [NM_016524]	51760
A_23_P104098	-2.8	-1.5	-2.2	-1.5	SYT2	Homo sapiens synaptotagmin II (SYT2), transcript variant 1, mRNA [NM_177402]	127833
A_23_P55917	1.7	1.6	-3.3	1.0	SYT3	Homo sapiens synaptotagmin III (SYT3), transcript variant 1, mRNA [NM_032298]	84258
A_24_P373877	-1.3	1.3	3.4	1.1	SYT5	Homo sapiens synaptotagmin V (SYT5), mRNA [NM_003180]	6861
A_23_P36187	-3.6	-1.5	-1.2	-1.5	SYT8	Homo sapiens synaptotagmin VIII (SYT8), mRNA [NM_138567]	90019

A_23_P46390	2.1	1.6	4.2	2.4	SYTL1	Homo sapiens synaptotagmin-like 1 (SYTL1), transcript variant 2, mRNA [NM_032872]	84958
A_23_P317082	-1.3	-1.7	-1.6	-3.8	SYTL5	Homo sapiens synaptotagmin-like 5 (SYTL5), transcript variant 1, mRNA [NM_138780]	94122
A_23_P138826	-1.2	-1.3	-1.0	-7.6	SYVN1	Homo sapiens synovial apoptosis inhibitor 1, synoviolin (SYVN1), transcript variant 2, mRNA [NM_172230]	84447
A_23_P51639	-2.3	-3.9	-1.2	-1.8	SZT2	Homo sapiens seizure threshold 2 homolog (mouse) (SZT2), mRNA [NM_015284]	23334

A_24_P63642	1.0	-1.2	-1.8	-2.1	T	Homo sapiens T, brachyury homolog (mouse) (T), mRNA [NM_003181]	6862
A_23_P8269	-1.2	1.6	1.7	-2.2	TAAR8	Homo sapiens trace amine associated receptor 8 (TAAR8), mRNA [NM_053278]	83551
A_23_P168266	1.0	1.9	1.0	-11.9	TAAR9	Homo sapiens trace amine associated receptor 9 (gene/pseudogene) (TAAR9), mRNA [NM_175057]	134860
A_23_P215283	1.0	-1.5	-2.5	1.4	TAC1	Homo sapiens tachykinin, precursor 1 (TAC1), transcript variant beta, mRNA [NM_003182]	6863

A_24_P98249	1.7	1.2	2.0	1.3	TACC1	Homo sapiens transforming, acidic coiled-coil containing protein 1 (TACC1), transcript variant 1, mRNA [NM_006283]	6867
A_23_P60047	-1.0	-1.0	-1.0	-3.1	TACC1	Homo sapiens transforming, acidic coiled-coil containing protein 1 (TACC1), transcript variant 1, mRNA [NM_006283]	6867
A_24_P462656	-1.1	1.0	-2.1	1.0	TACC2	transforming, acidic coiled-coil containing protein 2 [Source:HGNC Symbol;Acc:11523] [ENST00000492689]	10579

A_23_P73142	-1.0	1.1	-1.1	-15.2	TACO1	Homo sapiens translational activator of mitochondrially encoded cytochrome c oxidase I (TACO1), nuclear gene encoding mitochondrial protein, mRNA [NM_016360]	51204
A_24_P148590	2.0	2.2	1.1	1.2	TACR1	Homo sapiens tachykinin receptor 1 (TACR1), transcript variant short, mRNA [NM_015727]	6869
A_23_P24135	2.2	2.3	-1.1	1.2	TACR2	Homo sapiens tachykinin receptor 2 (TACR2), mRNA [NM_001057]	6865

A_23_P66664	-1.0	-1.7	-1.5	-2.1	TADA2A	Homo sapiens transcriptional adaptor 2A (TADA2A), transcript variant 2, mRNA [NM_133439]	6871
A_23_P305016	-1.2	3.0	2.4	2.1	TADA3	Homo sapiens transcriptional adaptor 3 (TADA3), transcript variant 1, mRNA [NM_006354]	10474
A_32_P192615	1.7	1.7	2.2	1.1	TAF1	Homo sapiens TAF1 RNA polymerase II, TATA box binding protein (TBP)-associated factor, 250kDa (TAF1), transcript variant 1, mRNA [NM_004606]	6872

A_23_P59202	-1.3	-2.4	-1.3	-1.3	TAF11	Homo sapiens TAF11 RNA polymerase II, TATA box binding protein (TBP)-associated factor, 28kDa (TAF11), mRNA [NM_005643]	6882
A_24_P928068	2.0	-2.4	1.7	1.7	TAF15	Homo sapiens TAF15 RNA polymerase II, TATA box binding protein (TBP)-associated factor, 68kDa, mRNA (cDNA clone IMAGE:5266186). [BC048985]	8148

A_23_P165891	4.2	1.0	2.0	4.6	TAF1B	Homo sapiens TATA box binding protein (TBP)-associated factor, RNA polymerase I, B, 63kDa (TAF1B), mRNA [NM_005680]	9014
A_24_P261383	-13.8	-1.2	-1.4	-3.6	TAF1D	Homo sapiens TATA box binding protein (TBP)-associated factor, RNA polymerase I, D, 41kDa (TAF1D), mRNA [NM_024116]	79101

A_23_P419850	2.6	2.1	4.2	2.2	TAF1L	Homo sapiens TAF1 RNA polymerase II, TATA box binding protein (TBP)-associated factor, 210kDa-like (TAF1L), mRNA [NM_153809]	138474
A_24_P385119	2.7	2.6	1.2	-1.2	TAF2	Homo sapiens TAF2 RNA polymerase II, TATA box binding protein (TBP)-associated factor, 150kDa (TAF2), mRNA [NM_003184]	6873

A_32_P153892	-5.5	-1.0	-1.0	-1.4	TAF3	Homo sapiens TAF3 RNA polymeras e II, TATA box binding protein (TBP)-associated factor, 140kDa (TAF3), mRNA [NM_0319 23]	83860
A_23_P422922	-1.7	1.4	-2.1	-1.2	TAF3	Homo sapiens TAF3 RNA polymeras e II, TATA box binding protein (TBP)-associated factor, 140kDa (TAF3), mRNA [NM_0319 23]	83860
A_23_P80062	1.2	1.1	1.1	-3.6	TAF4	Homo sapiens TAF4 RNA polymeras e II, TATA box binding protein (TBP)-associated factor, 135kDa (TAF4), mRNA [NM_0031 85]	6874

A_23_P95718	1.1	1.4	2.1	1.4	TAF4B	Homo sapiens TAF4b RNA polymerase II, TATA box binding protein (TBP)-associated factor, 105kDa (TAF4B), mRNA [NM_005640]	6875
A_23_P35309	-3.8	2.1	-1.6	-1.1	TAF5L	Homo sapiens TAF5-like RNA polymerase II, p300/CBP-associated factor (PCAF)-associated factor, 65kDa (TAF5L), transcript variant 1, mRNA [NM_014409]	27097

A_24_P376441	-1.0	-1.0	-2.7	-8.2	TAF5L	Homo sapiens TAF5-like RNA polymerase II, p300/CBP-associated factor (PCAF)-associated factor, 65kDa (TAF5L), transcript variant 2, mRNA [NM_001025247]	27097
A_23_P47541	2.4	1.9	2.6	2.1	TAF6L	Homo sapiens TAF6-like RNA polymerase II, p300/CBP-associated factor (PCAF)-associated factor, 65kDa (TAF6L), mRNA [NM_006473]	10629

A_23_P254790	-2.2	-1.4	-1.6	-3.8	TAF9B	Homo sapiens TAF9B RNA polymerase II, TATA box binding protein (TBP)-associated factor, 31kDa (TAF9B), mRNA [NM_015975]	51616
A_32_P627	15.0	2.8	11.6	3.7	TAF9B	Homo sapiens cDNA FLJ23869 fis, clone LNG09860. [AK074449]	51616
A_24_P354724	2.1	2.1	2.0	3.0	TAGAP	Homo sapiens T-cell activation RhoGTPase activating protein (TAGAP), transcript variant 2, mRNA [NM_054114]	117289

A_23_P80817	-2.8	1.1	-1.2	1.2	TAGLN3	Homo sapiens transgelin 3 (TAGLN3), transcript variant 1, mRNA [NM_013259]	29114
A_23_P63371	2.1	2.0	-1.6	1.8	TAL1	Homo sapiens T-cell acute lymphocytic leukemia 1 (TAL1), mRNA [NM_003189]	6886
A_23_P409449	-1.1	1.5	2.1	1.3	TAL2	Homo sapiens T-cell acute lymphocytic leukemia 2 (TAL2), mRNA [NM_005421]	6887
A_32_P28872	2.4	2.3	2.0	2.0	TALDO1	Homo sapiens transaldolase 1 (TALDO1), mRNA [NM_006755]	6888

A_24_P942068	1.7	2.4	1.9	1.3	TANC2	Homo sapiens tetratricopeptide repeat, ankyrin repeat and coiled-coil containing 2 (TANC2), mRNA [NM_025185]	26115
A_23_P402908	-1.0	-1.1	-1.9	-4.8	TANC2	Homo sapiens cDNA FLJ11824 fis, clone HEMBA1006492. [AK021886]	26115
A_24_P257108	2.6	2.5	2.3	2.5	TANK	Homo sapiens TRAF family member-associated NFKB activator (TANK), transcript variant 2, mRNA [NM_133484]	10010

A_24_P937445	1.0	-3.4	-3.9	1.1	TAOK1	Homo sapiens cDNA FLJ14314 fis, clone PLACE3000350, weakly similar to SERINE/THREONINE-PROTEIN KINASE SULU (EC 2.7.1.-). [AK024376]	57551
A_23_P218269	3.3	3.0	-1.7	2.6	TAOK2	Homo sapiens TAO kinase 2 (TAOK2), transcript variant 1, mRNA [NM_016151]	9344
A_23_P368067	3.8	-1.0	-1.1	-1.1	TAP2	Homo sapiens transporter 2, ATP-binding cassette, sub-family B (MDR/TAP) (TAP2), transcript variant 2, mRNA [NM_018833]	6891

A_23_P214444	1.0	-2.8	1.6	-1.1	TAP2	Homo sapiens transporter 2, ATP-binding cassette, sub-family B (MDR/TAP) (TAP2), transcript variant 1, mRNA [NM_000544]	6891
A_23_P365719	-16.4	1.6	1.4	-1.0	TAPBP	Homo sapiens TAP binding protein (tapasin) (TAPBP), transcript variant 1, mRNA [NM_003190]	6892
A_23_P259580	3.0	2.8	1.2	1.1	TAPBP	Homo sapiens TAP binding protein (tapasin) (TAPBP), transcript variant 2, mRNA [NM_172208]	6892

A_23_P360291	-7.3	-1.4	-2.2	-3.4	TAPBP	TAP binding protein (tapasin) [Source:HGNC Symbol;Acc:11566] [ENST00000480730]	6892
A_23_P315336	-2.8	1.0	-1.1	-2.2	TAPBP	Homo sapiens TAP binding protein (tapasin) (TAPBP), transcript variant 3, mRNA [NM_172209]	6892
A_23_P36700	1.3	-1.7	-2.5	-1.6	TAPBPL	Homo sapiens TAP binding protein-like (TAPBPL), mRNA [NM_018009]	55080
A_23_P301372	1.3	1.3	-2.3	-1.2	TAPT1	Homo sapiens transmembrane anterior posterior transformation 1 (TAPT1), mRNA [NM_153365]	202018

A_24_P191750	-12.1	-3.8	-1.3	-1.2	TAPT1	Homo sapiens transmembrane anterior posterior transformation 1 (TAPT1), mRNA [NM_153365]	202018
A_23_P52058	-2.1	-1.4	-1.3	-1.2	TARBP1	Homo sapiens TAR (HIV-1) RNA binding protein 1 (TARBP1), mRNA [NM_005646]	6894
A_23_P87603	-1.4	-1.4	-4.2	-1.5	TARBP2	Homo sapiens TAR (HIV-1) RNA binding protein 2 (TARBP2), transcript variant 1, mRNA [NM_134323]	6895
A_23_P129128	-3.1	1.0	-1.1	-1.0	TARSL2	Homo sapiens threonyl-tRNA synthetase-like 2 (TARSL2), mRNA [NM_152334]	123283

A_23_P160890	-2.6	1.5	-5.0	1.6	TAS1R1	Homo sapiens taste receptor, type 1, member 1 (TAS1R1), transcript variant 2, mRNA [NM_138697]	80835
A_23_P160886	1.1	1.1	1.6	-2.0	TAS1R1	Homo sapiens taste receptor, type 1, member 1 (TAS1R1), transcript variant 2, mRNA [NM_138697]	80835
A_23_P340312	-1.1	-2.3	-1.0	-1.0	TAS2R19	Homo sapiens taste receptor, type 2, member 19 (TAS2R19), mRNA [NM_176888]	259294
A_23_P382794	-3.4	1.1	-1.0	1.1	TAS2R20	Homo sapiens taste receptor, type 2, member 20 (TAS2R20), mRNA [NM_176889]	259295

A_23_P416191	1.5	3.0	1.5	-1.2	TAS2R31	Homo sapiens taste receptor, type 2, member 31 (TAS2R31), mRNA [NM_176885]	259290
A_23_P359746	1.6	1.5	1.5	-2.4	TAS2R38	Homo sapiens taste receptor, type 2, member 38 (TAS2R38), mRNA [NM_176817]	5726
A_23_P20101	1.4	-2.6	1.3	1.4	TAS2R4	Homo sapiens taste receptor, type 2, member 4 (TAS2R4), mRNA [NM_016944]	50832
A_24_P14902	1.1	1.6	1.0	2.2	TAS2R42	Homo sapiens taste receptor, type 2, member 42 (TAS2R42), mRNA [NM_181429]	353164

A_23_P371254	-1.0	-1.7	-3.1	-2.8	TAS2R43	Homo sapiens taste receptor, type 2, member 43 (TAS2R43), mRNA [NM_176884]	259289
A_23_P252100	1.2	-3.5	-1.6	-3.1	TAS2R5	Homo sapiens taste receptor, type 2, member 5 (TAS2R5), mRNA [NM_018980]	54429
A_23_P367013	-2.3	1.4	-1.4	-1.1	TAS2R60	Homo sapiens taste receptor, type 2, member 60 (TAS2R60), mRNA [NM_177437]	338398
A_23_P254978	-1.7	-1.4	-4.7	-7.8	TATDN1	Homo sapiens TatD DNase domain containing 1 (TATDN1), transcript variant 1, mRNA [NM_032026]	83940

A_23_P57819	1.0	-1.0	-1.1	-2.9	TATDN2	Homo sapiens TatD DNase domain containing 2 (TATDN2), mRNA [NM_014760]	9797
A_23_P38468	3.1	2.1	2.7	2.2	TAX1BP3	Homo sapiens Tax1 (human T-cell leukemia virus type I) binding protein 3 (TAX1BP3), transcript variant 1, mRNA [NM_014604]	30851
A_23_P62371	-1.1	1.2	-1.7	-2.3	TAZ	Homo sapiens tafazzin (TAZ), nuclear gene encoding mitochondrial protein, transcript variant 1, mRNA [NM_000116]	6901

A_23_P73023	-2.1	1.4	1.2	1.1	TBC1D1	Homo sapiens TBC1 (tre-2/USP6, BUB2, cdc16) domain family, member 1 (TBC1D1), mRNA [NM_015173]	23216
A_23_P166421	2.6	2.5	2.6	-1.0	TBC1D10A	Homo sapiens TBC1 domain family, member 10A (TBC1D10A), transcript variant 2, mRNA [NM_031937]	83874
A_23_P22382	-2.9	-6.5	-5.2	-3.2	TBC1D10B	Homo sapiens TBC1 domain family, member 10B (TBC1D10B), mRNA [NM_015527]	26000

A_24_P392201	-2.9	-1.8	-1.8	-1.2	TBC1D10C	Homo sapiens TBC1 domain family, member 10C (TBC1D10C), mRNA [NM_198517]	374403
A_24_P6494	-2.3	-1.0	-1.1	-1.0	TBC1D12	Homo sapiens TBC1 domain family, member 12 (TBC1D12), mRNA [NM_015188]	23232
A_23_P155830	-1.1	-1.1	-1.3	-2.1	TBC1D14	Homo sapiens TBC1 domain family, member 14 (TBC1D14), transcript variant 1, mRNA [NM_020773]	57533

A_24_P120352	2.2	1.1	-1.0	-1.0	TBC1D14	Homo sapiens TBC1 domain family, member 14 (TBC1D14), transcript variant 1, mRNA [NM_020773]	57533
A_23_P428326	1.8	-2.7	-2.5	-2.6	TBC1D16	Homo sapiens mRNA; cDNA DKFZp451F1311 (from clone DKFZp451F1311). [CR936670]	125058
A_23_P130731	1.2	-1.6	-1.6	-2.3	TBC1D17	Homo sapiens TBC1 domain family, member 17 (TBC1D17), transcript variant 1, mRNA [NM_024682]	79735

A_23_P136196	3.4	3.3	1.4	1.0	TBC1D19	Homo sapiens TBC1 domain family, member 19 (TBC1D19), mRNA [NM_018317]	55296
A_24_P392958	-2.2	-2.0	-3.3	-1.5	TBC1D20	Homo sapiens TBC1 domain family, member 20 (TBC1D20), mRNA [NM_144628]	128637
A_23_P354187	1.5	-1.4	1.1	-2.0	TBC1D20	TBC1 domain family, member 20 [Source:HGNC Symbol;Acc:16133] [ENST00000461304]	128637
A_23_P408206	-3.7	1.1	1.1	1.2	TBC1D21	Homo sapiens TBC1 domain family, member 21 (TBC1D21), mRNA [NM_153356]	161514

A_23_P159688	1.0	-2.7	-1.1	-1.8	TBC1D25	Homo sapiens TBC1 domain family, member 25 (TBC1D25), mRNA [NM_002536]	4943
A_24_P168994	-2.1	-1.8	-1.1	1.2	TBC1D26	Homo sapiens TBC1 domain family, member 26 (TBC1D26), mRNA [NM_178571]	353149
A_32_P24372	-2.9	1.1	1.0	1.0	TBC1D3B	Homo sapiens TBC1 domain family, member 3B (TBC1D3B), mRNA [NM_001001417]	414059
A_24_P838947	1.7	2.5	-2.0	-1.9	TBC1D3P5	Homo sapiens TBC1 domain family, member 3H pseudogene (LOC440419), non-coding RNA [NR_033892]	440419

A_23_P111273	1.4	1.9	2.0	-1.1	TBC1D7	Homo sapiens TBC1 domain family, member 7 (TBC1D7), transcript variant 1, mRNA [NM_016495]	51256
A_24_P576445	-1.1	1.0	-1.8	-3.8	TBC1D8	Homo sapiens cDNA FLJ40805 fis, clone TRACH2009060. [AK098124]	11138
A_23_P251611	1.0	1.0	-7.3	1.1	TBC1D9B	Homo sapiens TBC1 domain family, member 9B (with GRAM domain) (TBC1D9B), transcript variant 1, mRNA [NM_198868]	23061
A_23_P167698	21.5	35.8	19.2	20.5	TBCA	Homo sapiens tubulin folding cofactor A (TBCA), mRNA [NM_004607]	6902

A_23_P121345	-6.6	-1.0	-1.1	-1.0	TBCCD1	Homo sapiens TBCC domain containing 1 (TBCCD1), transcript variant 2, mRNA [NM_018138]	55171
A_23_P100602	-8.7	1.1	1.4	1.4	TBCD	Homo sapiens tubulin folding cofactor D (TBCD), mRNA [NM_005993]	6904
A_24_P275873	2.2	-1.1	-1.2	1.8	TBCD	Homo sapiens tubulin folding cofactor D (TBCD), mRNA [NM_005993]	6904
A_23_P130169	-1.9	-1.4	-2.4	-1.4	TBKBP1	Homo sapiens TBK1 binding protein 1 (TBKBP1), mRNA [NM_014726]	9755
A_24_P159515	4.0	5.0	1.6	1.2	TBKBP1	Homo sapiens TBK1 binding protein 1 (TBKBP1), mRNA [NM_014726]	9755

A_24_P417162	-2.7	-1.1	1.0	-2.3	TBL1X	Homo sapiens transducin (beta)-like 1X-linked (TBL1X), transcript variant 1, mRNA [NM_005647]	6907
A_24_P128880	-2.9	2.1	2.0	2.2	TBL1XR1	Homo sapiens transducin (beta)-like 1 X-linked receptor 1 (TBL1XR1), mRNA [NM_024665]	79718
A_23_P134395	-2.2	-2.1	-2.1	-1.5	TBL2	Homo sapiens transducin (beta)-like 2 (TBL2), mRNA [NM_012453]	26608
A_23_P106720	-1.4	-1.3	-2.0	-2.1	TBL3	Homo sapiens transducin (beta)-like 3 (TBL3), mRNA [NM_006453]	10607

A_23_P5685	1.0	-3.2	-1.0	-1.1	TBR1	Homo sapiens T-box, brain, 1 (TBR1), mRNA [NM_006593]	10716
A_23_P98463	-2.3	-1.0	-1.1	-1.1	TBRG1	Homo sapiens transforming growth factor beta regulator 1 (TBRG1), transcript variant 1, mRNA [NM_032811]	84897
A_24_P21752	2.1	2.2	2.4	1.8	TBRG1	Homo sapiens transforming growth factor beta regulator 1 (TBRG1), transcript variant 1, mRNA [NM_032811]	84897

A_23_P215658	2.9	4.2	1.6	1.6	TBRG4	Homo sapiens transforming growth factor beta regulator 4 (TBRG4), transcript variant 2, mRNA [NM_030900]	9238
A_24_P197794	-1.0	-1.1	-5.3	-2.9	TBRG4	Homo sapiens mRNA for KIAA0948 protein, partial cds. [AB023165]	9238
A_23_P211345	1.8	-4.1	-2.0	-1.1	TBX1	Homo sapiens T-box 1 (TBX1), transcript variant C, mRNA [NM_080647]	6899
A_32_P538017	1.4	1.2	1.1	2.1	TBX10	Homo sapiens T-box 10 (TBX10), mRNA [NM_005995]	347853
A_23_P412962	1.0	-6.8	-1.0	1.0	TBX19	Homo sapiens T-box 19 (TBX19), mRNA [NM_005149]	9095

A_23_P164451	1.4	1.6	-2.0	1.4	TBX2	Homo sapiens T-box 2 (TBX2), mRNA [NM_005994]	6909
A_24_P30557	-3.5	1.1	1.8	1.1	TBX5	Homo sapiens T-box 5 (TBX5), transcript variant 1, mRNA [NM_000192]	6910
A_23_P90357	2.3	1.3	1.3	1.6	TBXA2R	Homo sapiens thromboxane A2 receptor (TBXA2R), transcript variant a, mRNA [NM_001060]	6915
A_24_P274814	2.2	2.6	2.4	-1.2	TBXAS1	Homo sapiens thromboxane A synthase 1 (platelet) (TBXAS1), transcript variant 2, mRNA [NM_030984]	6916

A_32_P212886	1.0	-1.0	-1.1	-2.4	TBXAS1	Homo sapiens thromboxane A synthase 1 (platelet) (TBXAS1), transcript variant 5, mRNA [NM_001166254]	6916
A_23_P107051	-7.6	2.4	-1.1	-2.3	TCAP	Homo sapiens titin-cap (telethonin) (TCAP), mRNA [NM_003673]	8557
A_23_P132444	3.1	1.1	-1.2	-1.6	TCEA1	Homo sapiens transcription elongation factor A (SII), 1 (TCEA1), transcript variant 1, mRNA [NM_006756]	6917
A_24_P327886	-2.8	1.5	1.4	-1.2	TCEA3	Homo sapiens transcription elongation factor A (SII), 3 (TCEA3), mRNA [NM_003196]	6920

A_23_P73801	-1.1	2.3	2.1	-1.0	TCEAL1	Homo sapiens transcription elongation factor A (SII)-like 1 (TCEAL1), transcript variant 3, mRNA [NM_001006640]	9338
A_23_P434442	2.1	2.1	2.1	2.1	TCEAL3	Homo sapiens transcription elongation factor A (SII)-like 3 (TCEAL3), transcript variant 1, mRNA [NM_001006933]	85012
A_23_P259166	2.3	2.2	2.4	1.2	TCEAL4	Homo sapiens transcription elongation factor A (SII)-like 4 (TCEAL4), transcript variant 1, mRNA [NM_024863]	79921

A_24_P49106	1.2	3.7	1.5	-1.3	TCEAL7	Homo sapiens transcription elongation factor A (SII)-like 7 (TCEAL7), mRNA [NM_152278]	56849
A_24_P196469	-2.2	1.0	1.0	-1.0	TCEANC	transcription elongation factor A (SII) N-terminal and central domain containing [Source:HGNC Symbol;Acc:28277] [ENST00000467590]	170082
A_24_P93728	2.1	2.3	1.3	2.6	TCEANC2	transcription elongation factor A (SII) N-terminal and central domain containing 2 [Source:HGNC Symbol;Acc:26494] [ENST00000498272]	127428

A_24_P243881	-3.0	-2.9	-2.9	-5.4	TCEB1	Homo sapiens transcription elongation factor B (SIII), polypeptide 1 (15kDa, elongin C) (TCEB1), transcript variant 3, mRNA [NM_001204858]	6921
A_23_P312181	5.3	3.0	5.5	2.9	TCEB2	Homo sapiens transcription elongation factor B (SIII), polypeptide 2 (18kDa, elongin B) (TCEB2), transcript variant 2, mRNA [NM_207013]	6923

A_23_P74097	-1.5	-2.3	-1.1	1.0	TCEB3	Homo sapiens transcription elongation factor B (SIII), polypeptide 3 (110kDa, elongin A) (TCEB3), mRNA [NM_003198]	6924
A_23_P141706	1.1	1.0	1.5	-5.7	TCEB3B	Homo sapiens transcription elongation factor B polypeptide 3B (elongin A2) (TCEB3B), mRNA [NM_016427]	51224
A_24_P349965	1.0	1.0	2.3	1.1	TCF19	Homo sapiens transcription factor 19 (TCF19), transcript variant 1, mRNA [NM_007109]	6941

A_23_P424316	-2.4	1.0	-1.0	1.0	TCF20	Homo sapiens transcription factor 20 (AR1) (TCF20), transcript variant 1, mRNA [NM_005650]	6942
A_23_P156890	-1.0	1.0	1.0	-2.9	TCF21	Homo sapiens transcription factor 21 (TCF21), transcript variant 2, mRNA [NM_003206]	6943
A_24_P237936	-2.0	-1.8	-2.0	-1.3	TCF23	Homo sapiens transcription factor 23 (TCF23), mRNA [NM_175769]	150921
A_23_P49220	1.7	2.2	1.6	1.7	TCF25	Homo sapiens transcription factor 25 (basic helix-loop-helix) (TCF25), mRNA [NM_014972]	22980

A_24_P365365	1.4	1.4	2.3	1.4	TCF3	Homo sapiens transcription factor 3 (E2A immunoglobulin enhancer binding factors E12/E47) (TCF3), transcript variant 1, mRNA [NM_003200]	6929
A_23_P67708	2.6	1.1	-1.5	1.5	TCF3	Homo sapiens transcription factor 3 (E2A immunoglobulin enhancer binding factors E12/E47) (TCF3), transcript variant 1, mRNA [NM_003200]	6929
A_23_P27332	-8.8	1.3	1.3	-1.1	TCF4	Homo sapiens transcription factor 4 (TCF4), transcript variant 2, mRNA [NM_003199]	6925

A_23_P142872	1.1	-3.7	1.0	1.1	TCF7L1	Homo sapiens transcription factor 7-like 1 (T-cell specific, HMG-box) (TCF7L1), mRNA [NM_031283]	83439
A_23_P127013	-1.0	-6.9	-1.0	1.0	TCF7L2	Homo sapiens transcription factor 7-like 2 (T-cell specific, HMG-box) (TCF7L2), transcript variant 2, mRNA [NM_030756]	6934
A_23_P357717	2.9	1.1	1.4	1.1	TCL1A	Homo sapiens T-cell leukemia/lymphoma 1A (TCL1A), transcript variant 1, mRNA [NM_021966]	8115

A_23_P363196	1.1	2.5	1.0	1.3	TCL6	Homo sapiens TCL6a2 mRNA for T-cell leukemia/lymphoma 6 ORF141, complete cds, clone:pPLA2. [AB035333]	27004
A_23_P363203	-1.0	-9.8	-1.0	1.0	TCL6	Homo sapiens T-cell leukemia/lymphoma 6 (non-protein coding) (TCL6), non-coding RNA [NR_028288]	27004
A_23_P40611	2.3	2.3	2.1	2.3	TCN2	Homo sapiens transcobalamin II (TCN2), transcript variant 1, mRNA [NM_000355]	6948

A_24_P243396	2.1	1.7	1.3	-1.2	TCOF1	Homo sapiens Treacher Collins-Franceschetti syndrome 1 (TCOF1), transcript variant 3, mRNA [NM_001008657]	6949
A_23_P310317	1.6	1.9	3.4	-1.1	TCOF1	Homo sapiens Treacher Collins-Franceschetti syndrome 1 (TCOF1), transcript variant 4, mRNA [NM_001135243]	6949
A_32_P501400	-1.8	-4.1	-1.6	1.1	TCP1	Homo sapiens t-complex 1 (TCP1), transcript variant 1, mRNA [NM_030752]	6950
A_24_P75157	1.5	1.6	-2.2	1.0	TCP10	Homo sapiens t-complex 10 homolog (mouse) (TCP10), mRNA [NM_004610]	6953

A_24_P202905	1.1	3.7	-1.2	-1.1	TCP10L	Homo sapiens t-complex 10 (mouse)-like (TCP10L), mRNA [NM_144659]	140290
A_23_P350555	-5.8	2.4	1.8	-1.2	TCP10L	Homo sapiens t-complex 10 (mouse)-like (TCP10L), mRNA [NM_144659]	140290
A_23_P419107	1.1	-3.5	-1.1	1.1	TCP11L2	Homo sapiens t-complex 11 (mouse)-like 2 (TCP11L2), mRNA [NM_152772]	255394
A_23_P58002	-2.8	-1.6	1.1	1.2	TCTA	Homo sapiens T-cell leukemia translocation altered gene (TCTA), mRNA [NM_022171]	6988

A_24_P123166	1.3	-1.6	-1.4	2.0	TCTA	Homo sapiens T-cell leukemia translocation altered gene (TCTA), mRNA [NM_022171]	6988
A_32_P203099	1.3	-1.0	-3.2	1.0	TCTE1	Homo sapiens t-complex-associated-testis-expressed 1 (TCTE1), mRNA [NM_182539]	202500
A_32_P38286	5.5	4.6	8.4	4.6	TCTE3	Homo sapiens t-complex-associated-testis-expressed 3 (TCTE3), mRNA [NM_174910]	6991
A_23_P21162	1.0	-3.2	-1.1	-1.0	TCTEX1D2	Homo sapiens Tctex1 domain containing 2 (TCTEX1D2), mRNA [NM_152773]	255758

A_23_P76402	1.6	1.6	-4.0	1.6	TCTN1	Homo sapiens tectonic family member 1 (TCTN1), transcript variant 3, mRNA [NM_024549]	79600
A_23_P117623	-2.0	1.0	-1.1	1.4	TDP1	Homo sapiens tyrosyl-DNA phosphodiesterase 1 (TDP1), transcript variant 1, mRNA [NM_018319]	55775
A_23_P8311	1.0	-2.2	-1.1	1.0	TDP2	Homo sapiens tyrosyl-DNA phosphodiesterase 2 (TDP2), mRNA [NM_016614]	51567
A_23_P97835	-7.9	1.2	1.2	1.4	TDRD1	Homo sapiens tudor domain containing protein 1 (TDRD1) mRNA, complete cds. [AF285606]	56165

A_32_P7974	2.5	2.4	1.7	1.5	TDRD10	Homo sapiens tudor domain containing 10 (TDRD10), transcript variant 2, mRNA [NM_182499]	126668
A_32_P188825	1.2	-36.3	1.1	-1.5	TDRD12	Homo sapiens tudor domain containing 12 (TDRD12), mRNA [NM_001110822]	91646
A_32_P79763	1.1	1.0	-1.1	-5.5	TDRD12	Homo sapiens cDNA FLJ13072 fis, clone NT2RP3001844. [AK023134]	91646
A_32_P187617	1.0	2.2	-1.0	1.1	TDRD3	Homo sapiens tudor domain containing 3 (TDRD3), transcript variant 2, mRNA [NM_030794]	81550

A_23_P123672	-2.7	-1.0	-1.3	-1.2	TDRD7	Homo sapiens tudor domain containing 7 (TDRD7), mRNA [NM_014290]	23424
A_32_P208350	2.1	1.0	-1.1	-1.2	TDRD9	Homo sapiens tudor domain containing 9 (TDRD9), mRNA [NM_153046]	122402
A_23_P429340	-1.5	1.2	-3.3	1.1	TEAD1	Homo sapiens TEA domain family member 1 (SV40 transcriptional enhancer factor) (TEAD1), mRNA [NM_021961]	7003
A_23_P119344	2.5	-1.4	3.3	2.9	TEAD2	Homo sapiens TEA domain family member 2 (TEAD2), mRNA [NM_003598]	8463

A_24_P37712	-1.9	2.3	1.6	1.9	TEC	Homo sapiens tec protein tyrosine kinase (TEC), mRNA [NM_003215]	7006
A_23_P97826	-1.8	-1.2	-2.1	-3.4	TECTB	Homo sapiens tectorin beta (TECTB), mRNA [NM_058222]	6975
A_23_P411947	-14.2	-1.2	1.1	1.1	TEDDM1	Homo sapiens transmembrane epididymal protein 1 (TEDDM1), mRNA [NM_172000]	127670
A_23_P91757	2.6	2.9	3.9	2.8	TEF	Homo sapiens thyrotrophic embryonic factor (TEF), transcript variant 1, mRNA [NM_003216]	7008

A_32_P94199	-3.7	1.1	-1.1	-1.1	TEKT4P2	Homo sapiens tektin 4 pseudogene 2 (TEKT4P2), transcript variant 3, non-coding RNA [NR_038329]	100132288
A_23_P206237	1.7	1.5	-4.9	1.4	TELO2	Homo sapiens TEL2, telomere maintenance 2, homolog (S. cerevisiae) (TELO2), mRNA [NM_016111]	9894
A_23_P151297	1.0	-1.5	2.0	2.3	TENC1	Homo sapiens tensin like C1 domain containing phosphatase (tensin 2) (TENC1), transcript variant 1, mRNA [NM_015319]	23371

A_24_P75183	-1.4	1.5	-3.7	-1.4	TEPP	Homo sapiens testis, prostate and placenta expressed (TEPP), transcript variant 2, mRNA [NM_199456]	374739
A_23_P216149	4.2	4.0	2.6	3.0	TERF1	Homo sapiens telomeric repeat binding factor (NIMA-interacting) 1 (TERF1), transcript variant 1, mRNA [NM_017489]	7013
A_23_P417200	-1.9	2.0	3.3	1.0	TERF2	Homo sapiens telomeric repeat binding factor 2 (TERF2), mRNA [NM_005652]	7014
A_24_P341882	3.2	3.4	3.2	1.6	TESK1	Homo sapiens testis-specific kinase 1 (TESK1), mRNA [NM_006285]	7016

A_23_P169178	-1.1	1.2	1.2	-2.1	TESK1	Homo sapiens testis-specific kinase 1 (TESK1), mRNA [NM_006285]	7016
A_23_P322756	-6.0	1.1	1.0	1.0	TET1	Homo sapiens tet methylcytosine dioxygenase 1 (TET1), mRNA [NM_030625]	80312
A_24_P932233	2.9	2.8	2.7	2.2	TET2	Homo sapiens tet methylcytosine dioxygenase 2 (TET2), transcript variant 2, mRNA [NM_017628]	54790
A_32_P542874	-2.7	-1.0	-1.1	-1.0	TET3	Homo sapiens tet methylcytosine dioxygenase 3 (TET3), mRNA [NM_144993]	200424

A_23_P112412	-1.5	-1.0	-2.6	-1.4	TEX10	Homo sapiens testis expressed 10 (TEX10), transcript variant 1, mRNA [NM_017746]	54881
A_23_P130906	-1.7	-1.1	-1.1	-2.6	TEX101	Homo sapiens testis expressed 101 (TEX101), transcript variant 1, mRNA [NM_031451]	83639
A_23_P150362	1.1	-4.0	1.1	1.1	TEX12	Homo sapiens testis expressed 12 (TEX12), mRNA [NM_031275]	56158
A_23_P73637	-1.1	-1.1	-2.8	-1.3	TEX13B	Homo sapiens testis expressed 13B (TEX13B), mRNA [NM_031273]	56156

A_23_P54996	1.1	1.0	1.0	-7.2	TEX14	Homo sapiens testis expressed 14 (TEX14), transcript variant 1, mRNA [NM_198393]	56155
A_23_P4036	2.8	2.6	1.5	-1.9	TEX2	Homo sapiens testis expressed 2 (TEX2), mRNA [NM_018469]	55852
A_23_P251548	1.1	-3.2	-1.0	-1.0	TEX261	Homo sapiens testis expressed 261 (TEX261), mRNA [NM_144582]	113419
A_23_P41009	-1.3	-2.1	-1.5	1.1	TEX264	Homo sapiens testis expressed 264 (TEX264), transcript variant 1, mRNA [NM_015926]	51368

A_23_P257936	2.3	1.7	-2.5	-4.8	TEX28	Homo sapiens testis expressed 28 (TEX28), transcript variant 2, mRNA [NM_001586]	1527
A_23_P212500	1.1	1.1	-2.2	1.0	TF	Homo sapiens transferrin (TF), mRNA [NM_001063]	7018
A_24_P20954	-1.0	-1.1	-2.6	-1.0	TFAP2B	Homo sapiens transcription factor AP-2 beta (activating enhancer binding protein 2 beta) (TFAP2B), mRNA [NM_003221]	7021
A_23_P120472	2.1	2.1	2.0	1.3	TFAP2C	Homo sapiens transcription factor AP-2 gamma (activating enhancer binding protein 2 gamma) (TFAP2C), mRNA [NM_003222]	7022

A_23_P386973	1.4	-2.2	-7.8	-9.4	TFAP2D	Homo sapiens transcription factor AP-2 delta (activating enhancer binding protein 2 delta) (TFAP2D), mRNA [NM_172238]	83741
A_23_P347528	-1.0	-2.9	-1.2	-1.1	TFCP2	Homo sapiens transcription factor CP2 (TFCP2), transcript variant 1, mRNA [NM_005653]	7024
A_23_P169789	1.1	1.1	1.1	-2.6	TFCP2	Homo sapiens transcription factor CP2 (TFCP2), transcript variant 1, mRNA [NM_005653]	7024
A_32_P199301	1.9	-2.1	-1.1	-1.4	TFDP1	Homo sapiens transcription factor Dp-1 (TFDP1), transcript variant 1, mRNA [NM_007111]	7027

A_23_P166964	-5.8	-1.1	-1.2	-1.2	TFDP2	Homo sapiens transcription factor Dp-2 (E2F dimerization partner 2) (TFDP2), transcript variant 2, mRNA [NM_006286]	7029
A_23_P368729	-1.3	-1.5	-1.8	-3.7	TFEB	Homo sapiens transcription factor EB (TFEB), transcript variant 1, mRNA [NM_007162]	7942
A_32_P184394	-2.7	1.0	-1.1	-1.1	TFEC	Homo sapiens transcription factor EC (TFEC), transcript variant 1, mRNA [NM_012252]	22797
A_23_P68759	1.5	2.9	1.4	1.5	TFF1	Homo sapiens trefoil factor 1 (TFF1), mRNA [NM_003225]	7031

A_23_P57364	1.6	2.1	-1.0	2.0	TFF2	Homo sapiens trefoil factor 2 (TFF2), mRNA [NM_005423]	7032
A_23_P120839	1.6	-2.2	1.6	1.3	TFIP11	Homo sapiens tuftelin interacting protein 11 (TFIP11), transcript variant 1, mRNA [NM_001008697]	24144
A_23_P393620	-1.0	-6.4	-1.6	-2.7	TFPI2	Homo sapiens tissue factor pathway inhibitor 2 (TFPI2), mRNA [NM_006528]	7980
A_23_P27573	-1.0	-1.1	-2.1	-3.2	TFPT	Homo sapiens TCF3 (E2A) fusion partner (in childhood Leukemia) (TFPT), mRNA [NM_013342]	29844

A_23_P122896	6.3	6.2	7.0	2.7	TFR2	Homo sapiens transferrin receptor 2 (TFR2), transcript variant 1, mRNA [NM_003227]	7036
A_24_P373096	1.0	-8.9	-1.0	1.1	TGFB3	Homo sapiens transforming growth factor, beta 3 (TGFB3), mRNA [NM_003239]	7043
A_24_P170869	1.2	-1.9	-1.4	3.4	TGFBRAP1	transforming growth factor, beta receptor associated protein 1 [Source:HGNC Symbol;Acc:16836] [ENST00000393359]	9392

A_23_P153197	-2.7	1.9	2.2	2.3	TGIF1	Homo sapiens TGFB-induced factor homeobox 1 (TGIF1), transcript variant 1, mRNA [NM_170695]	7050
A_23_P65618	-12.6	1.0	1.5	1.0	TGM1	Homo sapiens transglutaminase 1 (K polypeptide epidermal type I, protein-glutamine-gamma-glutamyltransferase) (TGM1), mRNA [NM_000359]	7051

A_24_P923251	2.8	2.9	3.7	1.5	TGM2	Homo sapiens transglutaminase 2 (C polypeptide, protein-glutamine-gamma-glutamyltransferase) (TGM2), transcript variant 2, mRNA [NM_198951]	7052
A_23_P57118	1.5	2.2	1.4	1.4	TGM3	Homo sapiens transglutaminase 3 (E polypeptide, protein-glutamine-gamma-glutamyltransferase) (TGM3), mRNA [NM_003245]	7053
A_24_P402238	-1.4	2.9	-2.2	-1.6	TGM4	Homo sapiens transglutaminase 4 (prostate) (TGM4), mRNA [NM_003241]	7047

A_23_P60899	1.1	1.8	2.1	1.4	TGS1	Homo sapiens trimethylguanosine synthase 1 (TGS1), mRNA [NM_024831]	96764
A_24_P924602	3.2	2.5	2.4	3.1	TH	Homo sapiens tyrosine hydroxylase (TH), transcript variant 1, mRNA [NM_199292]	7054
A_24_P222126	-1.7	-2.2	-1.7	-1.1	TH1L	Homo sapiens TH1-like (Drosophila) (TH1L), transcript variant 1, mRNA [NM_198976]	51497
A_23_P106391	2.7	2.6	1.0	2.3	THAP10	Homo sapiens THAP domain containing 10 (THAP10), mRNA [NM_020147]	56906

A_24_P365954	-1.8	-3.7	1.1	-1.9	THAP11	Homo sapiens THAP domain containing 11 (THAP11), mRNA [NM_020457]	57215
A_24_P15586	2.7	2.7	2.6	2.7	THAP5	Homo sapiens THAP domain containing 5 (THAP5), transcript variant 2, mRNA [NM_182529]	168451
A_24_P23527	1.4	-1.3	-2.6	-1.6	THAP5	Homo sapiens THAP domain containing 5 (THAP5), transcript variant 2, mRNA [NM_182529]	168451
A_23_P109410	-9.6	1.3	1.6	1.5	THAP7	Homo sapiens THAP domain containing 7 (THAP7), transcript variant 1, mRNA [NM_030573]	80764

A_24_P142118	4.0	6.2	4.9	2.2	THBS1	Homo sapiens thrombospondin 1 (THBS1), mRNA [NM_003246]	7057
A_23_P131074	-2.0	-1.7	-1.6	-2.5	THEG	Homo sapiens Theg homolog (mouse) (THEG), transcript variant 1, mRNA [NM_016585]	51298
A_32_P66674	1.6	2.4	-1.8	1.4	THEM4	Homo sapiens thioesterase superfamily member 4 (THEM4), mRNA [NM_053055]	117145
A_23_P149375	-17.6	-2.2	-2.5	-1.1	THEM4	Homo sapiens thioesterase superfamily member 4 (THEM4), mRNA [NM_053055]	117145

A_24_P331711	4.4	-1.6	4.2	3.5	THEM5	thioesterase superfamily member 5 [Source:HGNC Symbol;Acc:26755] [ENST00000368817]	284486
A_23_P121956	1.9	2.6	1.7	-1.1	THG1L	Homo sapiens tRNA-histidine guanylyltransferase 1-like (S. cerevisiae) (THG1L), mRNA [NM_017872]	54974
A_23_P78372	-1.9	-3.0	-1.7	-1.1	THOC1	Homo sapiens THO complex 1 (THOC1), mRNA [NM_005131]	9984
A_24_P177353	-1.6	-1.6	-1.8	-2.2	THOC2	Homo sapiens THO complex 2 (THOC2), mRNA [NM_001081550]	57187
A_32_P41405	1.4	-1.2	2.1	-2.5	THOC3	Homo sapiens THO complex 3 (THOC3), mRNA [NM_032361]	84321

A_24_P387158	-2.2	2.1	-1.1	-1.6	THOC4	Homo sapiens THO complex 4 (THOC4), mRNA [NM_005782]	10189
A_23_P109470	-7.2	1.8	-1.8	2.1	THOC5	Homo sapiens THO complex 5 (THOC5), transcript variant 1, mRNA [NM_001002878]	8563
A_24_P262407	3.2	5.1	1.4	8.1	THRA	Homo sapiens thyroid hormone receptor, alpha (THRA), transcript variant 1, mRNA [NM_199334]	7067
A_23_P160367	1.1	1.0	-2.4	1.0	THRAP3	Homo sapiens thyroid hormone receptor associated protein 3 (THRAP3), mRNA [NM_005119]	9967

A_23_P148249	-1.2	-2.2	-1.2	-1.2	THSD4	Homo sapiens thrombospondin, type I, domain containing 4 (THSD4), mRNA [NM_024817]	79875
A_24_P5153	2.6	-6.7	1.8	-3.6	THSD4	Homo sapiens thrombospondin, type I, domain containing 4 (THSD4), mRNA [NM_024817]	79875
A_32_P350897	3.4	1.8	3.2	3.5	THSD7A	Homo sapiens thrombospondin, type I, domain containing 7A (THSD7A), mRNA [NM_015204]	221981
A_32_P11670	1.0	-1.0	-9.9	-1.0	THSD7A	Homo sapiens thrombospondin, type I, domain containing 7A (THSD7A), mRNA [NM_015204]	221981

A_24_P99371	-2.1	-1.6	-1.2	-1.2	THUMPD1	Homo sapiens THUMP domain containing 1 (THUMPD1), mRNA [NM_017736]	55623
A_24_P90878	-4.0	-1.1	-1.1	-1.1	THUMPD1	Homo sapiens THUMP domain containing 1 (THUMPD1), mRNA [NM_017736]	55623
A_24_P417474	2.0	1.5	-1.8	1.3	TIA1	Homo sapiens TIA1 cytotoxic granule-associated RNA binding protein (TIA1), transcript variant 1, mRNA [NM_022037]	7072
A_23_P90311	-2.3	-1.1	-1.1	-1.3	TICAM1	Homo sapiens toll-like receptor adaptor molecule 1 (TICAM1), mRNA [NM_182919]	148022

A_23_P120170	-1.5	-1.9	-2.2	-1.5	TIGD1	Homo sapiens tigger transposable element derived 1 (TIGD1), mRNA [NM_145702]	200765
A_32_P200787	2.0	-1.3	1.5	-1.8	TIGD1	Homo sapiens tigger transposable element derived 1 (TIGD1), mRNA [NM_145702]	200765
A_23_P20437	-1.6	-14.3	-1.6	-1.6	TIGD5	Homo sapiens tigger transposable element derived 5 (TIGD5), mRNA [NM_032862]	84948
A_23_P213966	-1.1	-2.5	-1.4	-1.1	TIGD6	Homo sapiens tigger transposable element derived 6 (TIGD6), transcript variant 1, mRNA [NM_030953]	81789

A_23_P64343	2.9	1.1	2.0	-1.0	TIMM10	Homo sapiens translocase of inner mitochondrial membrane 10 homolog (yeast) (TIMM10), nuclear gene encoding mitochondrial protein, mRNA [NM_012456]	26519
A_23_P126970	-3.0	1.4	-5.4	-3.8	TIMM17A	Homo sapiens translocase of inner mitochondrial membrane 17 homolog A (yeast) (TIMM17A), nuclear gene encoding mitochondrial protein, mRNA [NM_006335]	10440

A_23_P33886	1.5	2.1	-1.6	1.0	TIMM17B	Homo sapiens translocase of inner mitochondrial membrane 17 homolog B (yeast) (TIMM17B), nuclear gene encoding mitochondrial protein, transcript variant 2, mRNA [NM_005834]	10245
A_23_P373649	1.9	2.2	-1.7	3.2	TIMM23	Homo sapiens translocase of inner mitochondrial membrane 23 homolog (yeast) (TIMM23), nuclear gene encoding mitochondrial protein, mRNA [NM_006327]	100287932

A_23_P119214	-1.1	-1.7	-2.3	-1.3	TIMM44	Homo sapiens translocase of inner mitochondrial membrane 44 homolog (yeast) (TIMM44), nuclear gene encoding mitochondrial protein, mRNA [NM_006351]	10469
A_23_P217637	-1.4	-1.4	-2.2	-3.3	TIMM8A	Homo sapiens translocase of inner mitochondrial membrane 8 homolog A (yeast) (TIMM8A), nuclear gene encoding mitochondrial protein, transcript variant 1, mRNA [NM_004085]	1678

A_23_P62115	-10.3	1.4	-1.1	-12.3	TIMP1	Homo sapiens TIMP metallopeptidase inhibitor 1 (TIMP1), mRNA [NM_003254]	7076
A_23_P107401	-1.0	-7.9	1.0	-2.5	TIMP2	Homo sapiens TIMP metallopeptidase inhibitor 2 (TIMP2), mRNA [NM_003255]	7077
A_23_P168329	1.6	-1.9	2.1	1.2	TINAG	Homo sapiens tubulointerstitial nephritis antigen (TINAG), mRNA [NM_014464]	27283
A_23_P85585	-1.4	1.7	3.3	-3.3	TINAGL1	Homo sapiens tubulointerstitial nephritis antigen-like 1 (TINAGL1), transcript variant 1, mRNA [NM_022164]	64129

A_23_P143845	-1.3	2.8	-1.3	2.9	TIPARP	Homo sapiens TCDD-inducible poly(ADP-ribose) polymerase (TIPARP), transcript variant 2, mRNA [NM_015508]	25976
A_23_P397856	1.0	1.4	-11.6	-1.0	TIRAP	Homo sapiens toll-interleukin 1 receptor (TIR) domain containing adaptor protein (TIRAP), transcript variant 3, mRNA [NM_001039661]	114609
A_23_P205828	1.3	1.1	-2.3	1.3	TJP1	Homo sapiens tight junction protein 1 (zona occludens 1) (TJP1), transcript variant 1, mRNA [NM_003257]	7082

A_23_P9293	3.4	5.0	5.9	1.4	TJP2	Homo sapiens tight junction protein 2 (zona occludens 2) (TJP2), transcript variant 1, mRNA [NM_004817]	9414
A_23_P108157	2.4	1.4	1.8	3.5	TJP3	Homo sapiens tight junction protein 3 (zona occludens 3) (TJP3), mRNA [NM_014428]	27134
A_23_P259901	-1.3	2.4	3.5	1.2	TKTL1	Homo sapiens transketolase-like 1 (TKTL1), transcript variant 1, mRNA [NM_012253]	8277
A_23_P420417	1.4	2.0	-1.1	1.1	TLCD1	Homo sapiens TLC domain containing 1 (TLCD1), transcript variant 1, mRNA [NM_138463]	116238

A_23_P135239	-1.1	1.6	-3.8	-1.1	TLE1	Homo sapiens transducin-like enhancer of split 1 (E(sp1) homolog, Drosophila) (TLE1), mRNA [NM_005077]	7088
A_23_P342934	1.4	3.4	-5.4	-2.3	TLE3	Homo sapiens transducin-like enhancer of split 3 (E(sp1) homolog, Drosophila) (TLE3), transcript variant 1, mRNA [NM_005078]	7090
A_23_P94494	-1.1	-3.2	-1.3	-1.2	TLE4	Homo sapiens transducin-like enhancer of split 4 (E(sp1) homolog, Drosophila) (TLE4), mRNA [NM_007005]	7091

A_23_P208937	-1.0	-2.6	-5.0	-1.2	TLE6	Homo sapiens transducin-like enhancer of split 6 (E(sp1) homolog, Drosophila) (TLE6), transcript variant 2, mRNA [NM_024760]	79816
A_23_P426501	-1.5	-1.4	-2.1	1.2	TLE6	transducin-like enhancer of split 6 (E(sp1) homolog, Drosophila) [Source:HGNC Symbol;Acc:30788] [ENST00000482627]	79816
A_24_P282762	-1.6	-3.3	-3.9	-1.0	TLK1	Homo sapiens tousled-like kinase 1 (TLK1), transcript variant 1, mRNA [NM_012290]	9874

A_23_P38365	1.8	-1.3	-3.3	1.2	TLK2	Homo sapiens tousled-like kinase 2 (TLK2), transcript variant A, mRNA [NM_006852]	11011
A_24_P145163	-4.5	1.2	1.1	-1.1	TLL1	Homo sapiens tolloid-like 1 (TLL1), transcript variant 2, mRNA [NM_001204760]	7092
A_23_P427645	-1.1	-1.7	-2.0	-3.5	TLL1	Homo sapiens tolloid-like 1 (TLL1), transcript variant 1, mRNA [NM_012464]	7092
A_24_P196851	2.7	8.1	3.3	3.1	TLN1	Homo sapiens talin 1 (TLN1), mRNA [NM_006289]	7094
A_23_P10873	-4.4	-1.2	-1.4	-1.5	TLR1	Homo sapiens toll-like receptor 1 (TLR1), mRNA [NM_003263]	7096

A_23_P29922	1.1	1.2	2.3	1.3	TLR3	Homo sapiens toll-like receptor 3 (TLR3), mRNA [NM_003265]	7098
A_24_P17677	-3.3	-1.1	-3.5	-1.1	TLR6	Homo sapiens toll-like receptor 6 (TLR6), mRNA [NM_006068]	10333
A_23_P62437	-2.3	1.4	1.3	-1.0	TLR7	Homo sapiens toll-like receptor 7 (TLR7), mRNA [NM_016562]	51284
A_23_P85240	1.0	1.0	-2.6	1.0	TLR7	Homo sapiens toll-like receptor 7 (TLR7), mRNA [NM_016562]	51284
A_23_P73837	1.0	-1.0	-3.5	-1.1	TLR8	Homo sapiens toll-like receptor 8 (TLR8), mRNA [NM_138636]	51311
A_23_P132654	-2.0	-2.3	-2.3	-2.0	TLR9	Homo sapiens toll-like receptor 9 (TLR9), mRNA [NM_017442]	54106

A_24_P328446	1.3	1.2	-4.7	-1.1	TLX2	Homo sapiens T-cell leukemia homeobox 2 (TLX2), mRNA [NM_016170]	3196
A_23_P77117	1.8	2.2	-1.4	1.8	TM6SF1	Homo sapiens transmembrane 6 superfamily member 1 (TM6SF1), transcript variant 1, mRNA [NM_023003]	53346
A_23_P142403	-1.5	-2.0	4.4	-2.2	TM6SF2	Homo sapiens transmembrane 6 superfamily member 2 (TM6SF2), mRNA [NM_001001524]	53345
A_23_P127426	1.7	2.0	-2.8	1.8	TM7SF2	Homo sapiens transmembrane 7 superfamily member 2 (TM7SF2), mRNA [NM_003273]	7108

A_32_P52816	3.3	3.2	3.0	3.2	TM7SF3	Homo sapiens transmembrane 7 superfamily member 3 (TM7SF3), mRNA [NM_016551]	51768
A_23_P43107	1.2	-5.8	1.9	1.1	TM7SF4	Homo sapiens transmembrane 7 superfamily member 4 (TM7SF4), mRNA [NM_030788]	81501
A_24_P148521	4.0	1.7	-2.0	2.2	TMBIM1	Homo sapiens transmembrane BAX inhibitor motif containing 1 (TMBIM1), mRNA [NM_022152]	64114
A_23_P13701	-1.6	-2.0	-2.0	1.1	TMBIM4	Homo sapiens transmembrane BAX inhibitor motif containing 4 (TMBIM4), mRNA [NM_016056]	51643

A_23_P204702	2.0	2.5	2.1	1.0	TMBIM6	Homo sapiens transmembrane BAX inhibitor motif containing 6 (TMBIM6), transcript variant 1, mRNA [NM_003217]	7009
A_23_P305527	-1.0	-4.4	-3.8	-2.2	TMC2	Homo sapiens transmembrane channel-like 2 (TMC2), mRNA [NM_080751]	117532
A_23_P258783	2.2	2.2	2.1	2.2	TMC3	Homo sapiens TMC3 protein (TMC3) mRNA, complete cds. [AY263163]	342125
A_23_P330461	-1.2	2.0	2.1	1.4	TMC4	Homo sapiens transmembrane channel-like 4 (TMC4), transcript variant 2, mRNA [NM_144686]	147798

A_23_P15101	-2.2	1.0	-1.0	1.0	TMC5	Homo sapiens transmembrane channel-like 5 (TMC5), transcript variant 3, mRNA [NM_024780]	79838
A_23_P101013	5.4	1.8	1.0	-1.1	TMC6	Homo sapiens transmembrane channel-like 6 (TMC6), transcript variant 2, mRNA [NM_007267]	11322
A_23_P411246	1.1	-2.2	1.2	-1.9	TMC8	Homo sapiens transmembrane channel-like 8 (TMC8), mRNA [NM_152468]	147138
A_24_P400573	-1.3	-1.4	-1.1	-3.1	TMC8	Homo sapiens transmembrane channel-like 8 (TMC8), mRNA [NM_152468]	147138

A_32_P41070	-2.8	-1.7	-1.2	-2.0	TMCC1	Homo sapiens transmembrane and coiled-coil domain family 1 (TMCC1), transcript variant 1, mRNA [NM_001017395]	23023
A_23_P170986	-1.0	-3.3	-4.1	-1.3	TMCC1	Homo sapiens transmembrane and coiled-coil domain family 1 (TMCC1), transcript variant 1, mRNA [NM_001017395]	23023
A_24_P922288	-3.7	-3.5	-1.0	-1.1	TMCC1	Homo sapiens transmembrane and coiled-coil domain family 1 (TMCC1), transcript variant 1, mRNA [NM_001017395]	23023

A_24_P305933	-1.5	-1.3	-1.1	-3.3	TMCC3	Homo sapiens transmembrane and coiled-coil domain family 3 (TMCC3), mRNA [NM_020698]	57458
A_23_P355067	-1.4	-1.2	-2.1	1.2	TMCO1	Homo sapiens transmembrane and coiled-coil domains 1 (TMCO1), mRNA [NM_019026]	54499
A_32_P143124	3.6	2.9	-5.3	4.3	TMCO1	Homo sapiens transmembrane and coiled-coil domains 1 (TMCO1), mRNA [NM_019026]	54499
A_23_P65857	1.5	1.5	-4.7	1.0	TMCO5A	Homo sapiens transmembrane and coiled-coil domains 5A (TMCO5A), mRNA [NM_152453]	145942

A_24_P917833	-1.3	-1.3	-1.3	-3.3	TMED10	Homo sapiens transmembrane emp24-like trafficking protein 10 (yeast) (TMED10), mRNA [NM_006827]	10972
A_24_P800629	-2.4	-1.6	-1.4	-2.1	TMED10P1	Homo sapiens transmembrane emp24-like trafficking protein 10 (yeast) pseudogene 1 (TMED10P1), non-coding RNA [NR_002807]	286102
A_23_P26173	2.0	2.7	1.8	1.7	TMED3	Homo sapiens transmembrane emp24 protein transport domain containing 3 (TMED3), mRNA [NM_007364]	23423

A_24_P163537	-1.6	6.0	-1.0	1.3	TMED4	Homo sapiens transmembrane emp24 protein transport domain containing 4 (TMED4), mRNA [NM_182547]	222068
A_32_P135063	1.0	1.0	1.0	-2.5	TMED4	Homo sapiens transmembrane emp24 protein transport domain containing 4, mRNA (cDNA clone IMAGE:3456382). [BC001576]	222068
A_24_P297827	-1.0	-1.1	-2.5	-1.3	TMED4	transmembrane emp24 protein transport domain containing 4 [Source:HGNC Symbol;Acc:22301] [ENST00000477639]	222068

A_32_P128661	-10.9	1.0	1.4	1.0	TMED5	Homo sapiens transmembrane emp24 protein transport domain containing 5 (TMED5), transcript variant 2, mRNA [NM_001167830]	50999
A_23_P26570	-1.3	1.5	2.2	1.5	TMED6	Homo sapiens transmembrane emp24 protein transport domain containing 6 (TMED6), mRNA [NM_144676]	146456
A_23_P70127	-1.3	1.1	-1.0	-4.8	TMED9	Homo sapiens transmembrane emp24 protein transport domain containing 9 (TMED9), mRNA [NM_017510]	54732

A_24_P239364	2.0	2.1	-1.4	2.5	TMEFF2	Homo sapiens mRNA for tomoregulin, complete cds. [AB004064]	23671
A_23_P125383	2.4	6.6	6.6	6.6	TMEFF2	Homo sapiens transmembrane protein with EGF-like and two follistatin-like domains 2 (TMEFF2), mRNA [NM_016192]	23671
A_23_P15516	-2.8	1.2	1.1	-1.0	TMEM101	Homo sapiens transmembrane protein 101 (TMEM101), mRNA [NM_032376]	84336
A_32_P112380	-1.7	-1.8	-1.9	-3.0	TMEM102	Homo sapiens transmembrane protein 102 (TMEM102), mRNA [NM_178518]	284114

A_24_P284324	-1.4	-8.2	-1.6	-1.0	TMEM104	Homo sapiens transmembrane protein 104 (TMEM104), mRNA [NM_017728]	54868
A_32_P919718	1.1	1.0	-2.7	1.0	TMEM105	Homo sapiens transmembrane protein 105 (TMEM105), mRNA [NM_178520]	284186
A_32_P353072	1.5	1.5	-3.5	1.0	TMEM106B	Homo sapiens transmembrane protein 106B (TMEM106B), transcript variant 1, mRNA [NM_018374]	54664
A_24_P284353	7.5	6.4	6.3	6.8	TMEM107	Homo sapiens transmembrane protein 107 (TMEM107), transcript variant 1, mRNA [NM_032354]	84314

A_24_P51016	1.3	2.6	2.2	2.2	TMEM108	transmembrane protein 108 [Source:HGNC Symbol;Acc:28451] [ENST00000515826]	66000
A_23_P107036	-2.7	-1.5	-1.5	-1.6	TMEM11	Homo sapiens transmembrane protein 11 (TMEM11), transcript variant 1, mRNA [NM_003876]	8834
A_24_P255082	-1.2	-2.4	-1.7	1.6	TMEM110	Homo sapiens transmembrane protein 110 (TMEM110), mRNA [NM_198563]	375346
A_24_P728604	1.2	2.4	1.3	1.3	TMEM114	Homo sapiens transmembrane protein 114 (TMEM114), mRNA [NM_001146336]	283953

A_23_P6869	3.3	3.6	1.4	2.8	TMEM115	Homo sapiens transmembrane protein 115 (TMEM115), mRNA [NM_007024]	11070
A_23_P2573	-19.0	-1.2	1.5	-2.4	TMEM117	Homo sapiens transmembrane protein 117 (TMEM117), mRNA [NM_032256]	84216
A_23_P29924	-1.9	-1.8	-2.0	-2.2	TMEM128	Homo sapiens transmembrane protein 128 (TMEM128), mRNA [NM_032927]	85013
A_23_P354175	1.5	1.5	-2.1	1.0	TMEM129	Homo sapiens transmembrane protein 129 (TMEM129), transcript variant 2, mRNA [NM_138385]	92305

A_23_P349966	-2.3	1.0	1.3	-1.0	TMEM130	Homo sapiens transmembrane protein 130 (TMEM130), transcript variant 2, mRNA [NM_152913]	222865
A_23_P368558	5.0	3.5	3.4	3.5	TMEM131	Homo sapiens transmembrane protein 131 (TMEM131), mRNA [NM_015348]	23505
A_24_P66691	-3.1	-5.9	-6.0	-5.8	TMEM132B	transmembrane protein 132B [Source:HGNC Symbol;Acc:29397] [ENST00000534945]	114795
A_24_P6626	1.2	-7.0	-1.0	-1.0	TMEM132B	Homo sapiens transmembrane protein 132B (TMEM132B), mRNA [NM_052907]	114795

A_24_P372134	1.2	1.4	1.4	-2.4	TMEM140	Homo sapiens transmembrane protein 140 (TMEM140), mRNA [NM_018295]	55281
A_23_P94591	2.4	3.0	1.3	1.5	TMEM141	Homo sapiens transmembrane protein 141 (TMEM141), mRNA [NM_032928]	85014
A_24_P412976	-6.7	-1.4	-1.1	-2.7	TMEM143	Homo sapiens transmembrane protein 143 (TMEM143), mRNA [NM_018273]	55260
A_23_P67127	1.0	-1.1	1.4	-2.1	TMEM145	Homo sapiens transmembrane protein 145 (TMEM145), mRNA [NM_173633]	284339

A_24_P11640	-1.6	1.0	-5.6	-1.5	TMEM150A	Homo sapiens transmembrane protein 150A (TMEM150A), transcript variant 1, mRNA [NM_001031738]	129303
A_23_P361584	1.7	2.4	1.5	-1.0	TMEM154	Homo sapiens transmembrane protein 154 (TMEM154), mRNA [NM_152680]	201799
A_23_P107994	-1.8	-2.3	-2.1	1.2	TMEM160	Homo sapiens transmembrane protein 160 (TMEM160), mRNA [NM_017854]	54958
A_23_P165171	1.4	-3.3	-1.4	-1.5	TMEM161A	Homo sapiens transmembrane protein 161A (TMEM161A), mRNA [NM_017814]	54929

A_24_P354651	3.1	8.4	1.2	3.9	TMEM161B	Homo sapiens transmembrane protein 161B (TMEM161B), mRNA [NM_153354]	153396
A_23_P133174	-1.1	-1.1	-1.3	-4.7	TMEM165	Homo sapiens transmembrane protein 165 (TMEM165), mRNA [NM_018475]	55858
A_32_P81768	1.1	-8.0	1.1	1.1	TMEM167A	Homo sapiens transmembrane protein 167A (TMEM167A), mRNA [NM_174909]	153339
A_23_P68338	-4.2	1.0	-1.0	-1.3	TMEM169	Homo sapiens transmembrane protein 169 (TMEM169), transcript variant 3, mRNA [NM_138390]	92691

A_32_P206949	-1.0	-1.1	-18.6	-1.1	TMEM17	Homo sapiens transmembrane protein 17 (TMEM17), mRNA [NM_198276]	200728
A_24_P504050	1.1	1.1	1.0	-2.2	TMEM170A	transmembrane protein 170A [Source:HGNC Symbol;Acc:29577] [ENST00000357613]	124491
A_32_P110390	-1.2	1.1	2.1	1.7	TMEM171	Homo sapiens transmembrane protein 171 (TMEM171), transcript variant 1, mRNA [NM_173490]	134285

A_23_P61371	1.5	1.3	1.4	3.3	TMEM173	Homo sapiens transmembrane protein 173 (TMEM173), nuclear gene encoding mitochondrial protein, mRNA [NM_198282]	340061
A_23_P344594	2.2	-1.2	2.0	3.2	TMEM174	Homo sapiens transmembrane protein 174 (TMEM174), mRNA [NM_153217]	134288
A_23_P252082	1.8	3.5	2.2	3.6	TMEM176A	Homo sapiens transmembrane protein 176A (TMEM176A), mRNA [NM_018487]	55365

A_23_P142738	-1.7	-1.7	-1.8	-2.0	TMEM178	Homo sapiens transmembrane protein 178 (TMEM178), transcript variant 1, mRNA [NM_152390]	130733
A_24_P93206	1.1	2.0	2.0	1.1	TMEM179	Homo sapiens transmembrane protein 179 (TMEM179), mRNA [NM_207379]	388021
A_24_P161959	1.1	-1.0	-2.9	1.1	TMEM179B	Homo sapiens transmembrane protein 179B (TMEM179B), mRNA [NM_199337]	374395
A_24_P40519	-5.4	-2.0	1.6	2.1	TMEM185A	Homo sapiens transmembrane protein 185A (TMEM185A), transcript variant 1, mRNA [NM_032508]	84548

A_32_P135558	-2.1	-3.1	-2.0	-1.2	TMEM188	Homo sapiens transmembrane protein 188 (TMEM188), mRNA [NM_153261]	255919
A_23_P397417	-5.4	1.3	1.2	1.1	TMEM188	Homo sapiens transmembrane protein 188 (TMEM188), mRNA [NM_153261]	255919
A_23_P385742	-1.3	-1.5	-2.6	1.1	TMEM19	Homo sapiens transmembrane protein 19 (TMEM19), mRNA [NM_018279]	55266
A_23_P166336	1.3	2.1	3.5	2.1	TMEM191A	Homo sapiens transmembrane protein 191A (TMEM191A), non-coding RNA [NR_026815]	84222

A_24_P478495	2.4	-1.3	-1.2	-1.1	TMEM191B	Homo sapiens cDNA FLJ35852 fis, clone TESTI2007074. [AK093171]	728229
A_24_P787889	2.5	3.5	2.0	1.3	TMEM191B	Homo sapiens transmembrane protein 191B (TMEM191B), mRNA [NM_001242313]	728229
A_23_P53346	-1.1	-1.0	-3.0	-1.0	TMEM194A	Homo sapiens transmembrane protein 194A (TMEM194A), transcript variant 2, mRNA [NM_015257]	23306
A_24_P822704	1.3	-1.3	1.0	-2.4	TMEM198	Homo sapiens transmembrane protein 198 (TMEM198), mRNA [NM_001005209]	130612

A_23_P391906	1.0	-1.4	1.3	2.1	TMEM200A	Homo sapiens transmembrane protein 200A, mRNA (cDNA clone MGC:50847 IMAGE:5760073), complete cds. [BC044246]	114801
A_24_P933011	1.7	2.2	1.4	-1.4	TMEM200C	Homo sapiens transmembrane protein 200C (TMEM200C), mRNA [NM_001080209]	645369
A_24_P522631	-1.6	-1.1	-2.4	-1.8	TMEM201	Homo sapiens transmembrane protein 201 (TMEM201), transcript variant 1, mRNA [NM_001130924]	199953

A_23_P136238	3.9	1.5	3.4	-9.0	TMEM203	Homo sapiens transmembrane protein 203 (TMEM203), mRNA [NM_053045]	94107
A_23_P206369	-4.8	1.1	1.1	1.1	TMEM208	Homo sapiens transmembrane protein 208 (TMEM208), mRNA [NM_014187]	29100
A_23_P122805	3.1	1.5	1.2	1.3	TMEM209	Homo sapiens transmembrane protein 209 (TMEM209), mRNA [NM_032842]	84928
A_23_P143083	1.3	-4.8	-3.4	1.2	TMEM214	Homo sapiens transmembrane protein 214 (TMEM214), transcript variant 1, mRNA [NM_017727]	54867

A_23_P203389	1.9	-1.2	2.0	-4.7	TMEM216	Homo sapiens transmembrane protein 216 (TMEM216), transcript variant 1, mRNA [NM_016499]	51259
A_32_P95757	-1.1	1.0	-2.1	1.0	TMEM217	Homo sapiens transmembrane protein 217 (TMEM217), transcript variant 1, mRNA [NM_145316]	221468
A_23_P77562	6.4	5.1	2.4	5.1	TMEM219	Homo sapiens transmembrane protein 219 (TMEM219), transcript variant 2, mRNA [NM_194280]	124446

A_24_P116700	-3.5	1.3	1.3	1.4	TMEM220	Homo sapiens transmembrane protein 220 (TMEM220), mRNA [NM_001004313]	388335
A_23_P1956	1.1	2.1	-2.6	2.1	TMEM223	Homo sapiens transmembrane protein 223 (TMEM223), mRNA [NM_001080501]	79064
A_32_P47027	-2.5	1.4	-2.1	-1.3	TMEM229A	Homo sapiens transmembrane protein 229A (TMEM229A), mRNA [NM_001136002]	730130
A_23_P359497	4.1	2.4	3.8	1.7	TMEM231	Homo sapiens transmembrane protein 231 (TMEM231), transcript variant 3, mRNA [NM_001077419]	79583

A_24_P510138	-1.2	1.2	3.5	-1.8	TMEM232	transmembrane protein 232 [Source:HGNC Symbol;Acc:37270] [ENST00000507188]	642987
A_24_P482104	-2.0	1.0	1.0	1.1	TMEM232	transmembrane protein 232 [Source:HGNC Symbol;Acc:37270] [ENST00000503527]	642987
A_24_P285378	-1.0	-3.4	-1.1	-2.8	TMEM234	transmembrane protein 234 [Source:HGNC Symbol;Acc:28837] [ENST00000461402]	56063
A_23_P370097	-2.7	1.4	-1.3	1.0	TMEM237	Homo sapiens transmembrane protein 237 (TMEM237), transcript variant 1, mRNA [NM_001044385]	65062

A_24_P941831	-5.7	1.2	1.1	-1.0	TMEM237	Homo sapiens transmembrane protein 237 (TMEM237), transcript variant 1, mRNA [NM_001044385]	65062
A_23_P131909	1.3	-3.5	-1.7	-2.1	TMEM239	Homo sapiens transmembrane protein 239 (TMEM239), mRNA [NM_001167670]	100288797
A_32_P133182	2.5	-1.0	1.1	-1.0	TMEM239	Uncharacterized protein [Source:UniProtKB/TrEMBL;Acc:D6RAZ5] [ENST00000554164]	100288797
A_23_P203115	1.1	-5.7	1.0	1.1	TMEM25	Homo sapiens transmembrane protein 25 (TMEM25), transcript variant 1, mRNA [NM_032780]	84866

A_24_P133017	-1.5	-1.4	-3.4	-1.6	TMEM25	Homo sapiens transmembrane protein 25 (TMEM25), transcript variant 1, mRNA [NM_032780]	84866
A_23_P33984	1.0	-1.0	3.2	-1.1	TMEM27	Homo sapiens transmembrane protein 27 (TMEM27), mRNA [NM_020665]	57393
A_24_P616840	2.1	-1.4	-1.1	-2.3	TMEM30B	Homo sapiens transmembrane protein 30B (TMEM30B), mRNA [NM_001017970]	161291
A_32_P143377	3.8	2.1	2.7	1.5	TMEM30B	Homo sapiens transmembrane protein 30B (TMEM30B), mRNA [NM_001017970]	161291

A_24_P410639	-30.7	-1.3	-33.4	-1.5	TMEM33	Homo sapiens transmembrane protein 33 (TMEM33), mRNA [NM_018126]	55161
A_24_P932066	1.4	1.4	-3.5	-1.2	TMEM33	Homo sapiens transmembrane protein 33 (TMEM33), mRNA [NM_018126]	55161
A_23_P45324	1.0	-1.0	-2.8	-1.1	TMEM35	Homo sapiens transmembrane protein 35 (TMEM35), mRNA [NM_021637]	59353
A_24_P278156	1.0	-4.3	1.2	1.1	TMEM38B	Homo sapiens transmembrane protein 38B (TMEM38B), mRNA [NM_018112]	55151
A_24_P120303	-1.9	1.2	-2.5	1.2	TMEM39A	Homo sapiens transmembrane protein 39A (TMEM39A), mRNA [NM_018266]	55254

A_23_P29551	1.3	2.1	-1.7	1.1	TMEM40	Homo sapiens transmembrane protein 40 (TMEM40), mRNA [NM_018306]	55287
A_23_P80832	-2.1	-1.0	-2.0	-1.9	TMEM41A	Homo sapiens transmembrane protein 41A (TMEM41A), mRNA [NM_080652]	90407
A_23_P29836	-1.0	-1.1	-5.3	1.6	TMEM42	Homo sapiens transmembrane protein 42 (TMEM42), mRNA [NM_144638]	131616
A_24_P615181	-2.8	1.0	-1.0	-1.6	TMEM44	transmembrane protein 44 [Source:HGNC Symbol;Acc:25120] [ENST00000476750]	93109
A_23_P1682	-1.1	1.1	-1.1	-27.5	TMEM45B	Homo sapiens transmembrane protein 45B (TMEM45B), mRNA [NM_138788]	120224

A_24_P143171	1.0	-1.0	3.1	-1.1	TMEM47	Homo sapiens transmembrane protein 47 (TMEM47), mRNA [NM_031442]	83604
A_23_P74129	1.5	1.6	-3.8	-1.1	TMEM50A	Homo sapiens transmembrane protein 50A (TMEM50A), mRNA [NM_014313]	23585
A_24_P113824	1.7	1.4	2.8	1.6	TMEM50A	Homo sapiens transmembrane protein 50A (TMEM50A), mRNA [NM_014313]	23585
A_23_P57304	1.0	-1.8	-1.0	2.9	TMEM50B	Homo sapiens transmembrane protein 50B (TMEM50B), transcript variant 1, mRNA [NM_006134]	757

A_23_P12044	1.0	1.0	-4.2	-6.1	TMEM51	Homo sapiens transmembrane protein 51 (TMEM51), transcript variant 4, mRNA [NM_018022]	55092
A_23_P429624	-1.5	-1.8	-1.8	-2.4	TMEM52	Homo sapiens transmembrane protein 52 (TMEM52), mRNA [NM_178545]	339456
A_23_P85853	1.4	-2.0	-1.1	-1.0	TMEM53	Homo sapiens transmembrane protein 53 (TMEM53), mRNA [NM_024587]	79639
A_23_P309155	-2.7	1.4	-1.0	-2.2	TMEM59L	Homo sapiens transmembrane protein 59-like (TMEM59L), mRNA [NM_012109]	25789

A_23_P11629	-3.7	1.3	-2.2	-1.3	TMEM61	Homo sapiens transmembrane protein 61 (TMEM61), mRNA [NM_182532]	199964
A_24_P102865	1.6	-1.5	-1.0	-17.9	TMEM61	Homo sapiens transmembrane protein 61 (TMEM61), mRNA [NM_182532]	199964
A_23_P167856	1.8	2.2	1.6	1.4	TMEM63B	Homo sapiens transmembrane protein 63B (TMEM63B), mRNA [NM_018426]	55362
A_32_P18723	1.1	1.1	1.7	-2.1	TMEM64	Homo sapiens transmembrane protein 64 (TMEM64), transcript variant 1, mRNA [NM_001008495]	169200

A_23_P104151	2.2	-1.0	-1.0	1.0	TMEM69	Homo sapiens transmembrane protein 69 (TMEM69), mRNA [NM_016486]	51249
A_24_P704446	1.0	-3.5	-1.1	-1.1	TMEM72	Homo sapiens transmembrane protein 72 (TMEM72), mRNA [NM_001123376]	643236
A_24_P928888	1.0	-1.0	-5.4	-1.1	TMEM74	transmembrane protein 74 [Source:HGNC Symbol;Acc:26409] [ENST00000518838]	157753
A_24_P100673	-5.3	1.3	1.2	-2.1	TMEM85	Homo sapiens transmembrane protein 85 (TMEM85), mRNA [NM_016454]	51234
A_32_P66035	-3.6	1.7	1.3	-1.1	TMEM86A	Homo sapiens transmembrane protein 86A (TMEM86A), mRNA [NM_153347]	144110

A_24_P121956	1.1	2.3	2.2	-1.2	TMEM86B	Homo sapiens transmembrane protein 86B (TMEM86B), mRNA [NM_173804]	255043
A_24_P251411	-1.0	3.5	-1.1	-1.1	TMEM87B	Homo sapiens transmembrane protein 87B (TMEM87B), mRNA [NM_032824]	84910
A_23_P303155	8.3	7.2	7.8	7.7	TMEM87B	Homo sapiens transmembrane protein 87B (TMEM87B), mRNA [NM_032824]	84910
A_23_P77859	2.1	1.3	-1.4	-1.3	TMEM88	Homo sapiens transmembrane protein 88 (TMEM88), mRNA [NM_203411]	92162

A_23_P140907	-1.6	-3.8	-1.7	-1.9	TMEM8A	Homo sapiens transmembrane protein 8A (TMEM8A), mRNA [NM_021259]	58986
A_32_P190568	5.6	2.1	1.6	1.4	TMEM8A	transmembrane protein 8A [Source:HGNC Symbol;Acc:17205] [ENST00000476735]	58986
A_23_P114952	2.2	1.0	1.6	1.1	TMEM9	Homo sapiens transmembrane protein 9 (TMEM9), mRNA [NM_016456]	252839
A_24_P307289	2.9	-1.2	1.3	1.0	TMEM95	Homo sapiens transmembrane protein 95 (TMEM95), mRNA [NM_198154]	339168
A_24_P151920	-2.2	-2.9	-4.1	-23.6	TMEM97	Homo sapiens transmembrane protein 97 (TMEM97), mRNA [NM_014573]	27346

A_24_P190168	1.0	1.0	1.0	2.2	TMEM97	Homo sapiens transmembrane protein 97 (TMEM97), mRNA [NM_014573]	27346
A_32_P201521	-1.2	-1.2	-6.8	-1.3	TMEM97	Homo sapiens transmembrane protein 97 (TMEM97), mRNA [NM_014573]	27346
A_23_P162087	-1.2	2.2	1.3	-1.3	TMEM9B	Homo sapiens TMEM9 domain family, member B (TMEM9B), mRNA [NM_020644]	56674
A_23_P56091	2.3	1.4	2.1	1.2	TMIGD2	Homo sapiens transmembrane and immunoglobulin domain containing 2 (TMIGD2), transcript variant 1, mRNA [NM_144615]	126259

A_23_P126605	-2.1	-3.4	-1.2	1.1	TMOD4	Homo sapiens tropomodulin 4 (muscle) (TMOD4), mRNA [NM_013353]	29765
A_23_P204170	2.9	2.8	3.2	2.2	TMPO	Homo sapiens thymopoietin (TMPO), transcript variant 1, mRNA [NM_003276]	7112
A_23_P325040	1.6	1.6	4.7	1.0	TMPO	Homo sapiens thymopoietin (TMPO), transcript variant 1, mRNA [NM_003276]	7112
A_24_P109101	1.1	1.2	1.0	-3.6	TMPRSS11A	Homo sapiens transmembrane protease, serine 11A (TMPRSS11A), transcript variant 1, mRNA [NM_182606]	339967

A_23_P203150	5.7	4.3	4.3	4.6	TMPRSS13	Homo sapiens transmembrane protease, serine 13 (TMPRSS13), transcript variant 3, mRNA [NM_001206790]	84000
A_23_P435477	1.7	-2.4	1.3	-1.0	TMPRSS13	Homo sapiens transmembrane protease, serine 13 (TMPRSS13), transcript variant 1, mRNA [NM_001077263]	84000
A_23_P29067	1.3	1.1	1.6	2.4	TMPRSS2	Homo sapiens transmembrane protease, serine 2 (TMPRSS2), transcript variant 2, mRNA [NM_005656]	7113

A_23_P211493	1.1	2.4	2.2	-4.2	TMPRSS6	Homo sapiens transmembrane protease, serine 6 (TMPRSS6), mRNA [NM_153609]	164656
A_23_P96599	1.0	-6.1	-1.0	1.4	TMSB15B	Homo sapiens thymosin beta 15B (TMSB15B), mRNA [NM_194324]	286527
A_23_P48264	-5.5	1.2	-1.3	1.1	TMTC3	Homo sapiens transmembrane and tetratricopeptide repeat containing 3 (TMTC3), mRNA [NM_181783]	160418
A_24_P278853	-2.4	-2.2	-7.7	-4.9	TMTC4	Homo sapiens transmembrane and tetratricopeptide repeat containing 4 (TMTC4), transcript variant 1, mRNA [NM_032813]	84899

A_23_P31195	3.6	1.3	-1.1	-2.8	TMUB1	Homo sapiens transmembrane and ubiquitin-like domain containing 1 (TMUB1), transcript variant 1, mRNA [NM_031434]	83590
A_23_P76951	2.1	1.4	1.4	1.3	TMX1	Homo sapiens thioredoxin-related transmembrane protein 1 (TMX1), mRNA [NM_030755]	81542
A_24_P417281	1.0	-1.0	-3.7	-1.0	TMX3	Homo sapiens thioredoxin-related transmembrane protein 3 (TMX3), mRNA [NM_019022]	54495
A_24_P250535	-1.0	-1.0	-4.1	-1.0	TMX4	Homo sapiens thioredoxin-related transmembrane protein 4 (TMX4), mRNA [NM_021156]	56255

A_24_P919311	3.1	3.4	6.7	2.9	TNC	tenascin C [Source:HGNC Symbol;Acc:5318] [ENST00000481475]	3371
A_24_P157926	-2.5	-7.1	-9.5	1.6	TNFAIP3	Homo sapiens tumor necrosis factor, alpha-induced protein 3 (TNFAIP3), mRNA [NM_006290]	7128
A_32_P219520	1.9	1.6	-1.1	-3.0	TNFAIP8	Homo sapiens tumor necrosis factor, alpha-induced protein 8 (TNFAIP8), transcript variant 1, mRNA [NM_014350]	25816

A_23_P428875	1.7	-3.8	1.6	1.6	TNFAIP8L1	Homo sapiens tumor necrosis factor, alpha-induced protein 8-like 1 (TNFAIP8L1), transcript variant 2, mRNA [NM_152362]	126282
A_24_P375482	2.0	2.2	1.2	1.9	TNFAIP8L3	Homo sapiens tumor necrosis factor, alpha-induced protein 8-like 3 (TNFAIP8L3), mRNA [NM_207381]	388121
A_24_P211106	-1.3	-7.8	-1.1	-1.5	TNFRSF11A	tumor necrosis factor receptor superfamily, member 11a, NFKB activator [Source:HGNC Symbol;Acc:11908] [ENST00000382790]	8792

A_24_P192485	-2.4	-1.8	-1.1	-1.0	TNFRSF11B	Homo sapiens tumor necrosis factor receptor superfamily, member 11b (TNFRSF11B), mRNA [NM_002546]	4982
A_23_P91764	21.5	13.4	5.9	15.4	TNFRSF13C	Homo sapiens tumor necrosis factor receptor superfamily, member 13C (TNFRSF13C), mRNA [NM_052945]	115650
A_23_P126908	-1.0	-5.0	-2.8	-1.1	TNFRSF14	Homo sapiens tumor necrosis factor receptor superfamily, member 14 (TNFRSF14), mRNA [NM_003820]	8764

A_24_P56310	-3.2	-1.9	-1.5	1.4	TNFRSF19	Homo sapiens tumor necrosis factor receptor superfamily, member 19 (TNFRSF19), transcript variant 2, mRNA [NM_148957]	55504
A_23_P140057	1.0	-10.4	1.6	-1.8	TNFRSF19	Homo sapiens tumor necrosis factor receptor superfamily, member 19 (TNFRSF19), transcript variant 1, mRNA [NM_018647]	55504
A_24_P364363	6.5	42.3	29.0	33.2	TNFRSF1A	Homo sapiens tumor necrosis factor receptor superfamily, member 1A (TNFRSF1A), mRNA [NM_001065]	7132

A_23_P30666	1.6	-2.6	1.1	1.9	TNFRSF21	Homo sapiens tumor necrosis factor receptor superfamily, member 21 (TNFRSF21), mRNA [NM_014452]	27242
A_23_P218646	-2.2	1.0	-1.0	2.0	TNFRSF6B	Homo sapiens tumor necrosis factor receptor superfamily, member 6b, decoy (TNFRSF6B), mRNA [NM_003823]	8771
A_23_P500614	3.5	1.5	2.7	1.9	TNFRSF8	Homo sapiens tumor necrosis factor receptor superfamily, member 8 (TNFRSF8), transcript variant 1, mRNA [NM_001243]	943

A_23_P94754	-3.0	-1.8	-4.7	-2.5	TNFSF15	Homo sapiens tumor necrosis factor (ligand) superfamily, member 15 (TNFSF15), transcript variant 1, mRNA [NM_005118]	9966
A_24_P167012	1.6	-2.5	-2.2	1.1	TNFSF15	Homo sapiens tumor necrosis factor (ligand) superfamily, member 15 (TNFSF15), transcript variant 1, mRNA [NM_005118]	9966
A_23_P126836	1.1	1.0	-3.3	5.5	TNFSF4	Homo sapiens tumor necrosis factor (ligand) superfamily, member 4 (TNFSF4), mRNA [NM_003326]	7292

A_23_P107064	2.4	1.2	2.6	1.8	TNK1	Homo sapiens tyrosine kinase, non-receptor, 1 (TNK1), transcript variant 2, mRNA [NM_003985]	8711
A_24_P94643	1.6	-1.2	-2.1	-1.1	TNK2	Homo sapiens tyrosine kinase, non-receptor, 2 (TNK2), transcript variant 2, mRNA [NM_001010938]	10188
A_32_P144596	1.3	2.4	-1.4	-3.3	TNKS	Homo sapiens tankyrase, TRF1-interacting ankyrin-related ADP-ribose polymerase (TNKS), mRNA [NM_003747]	8658

A_23_P323252	2.8	2.9	1.1	1.6	TNKS	Homo sapiens tankyrase, TRF1-interacting ankyrin-related ADP-ribose polymerase (TNKS), mRNA [NM_003747]	8658
A_23_P418597	-3.6	-1.9	-2.6	-4.3	TNKS1BP1	Homo sapiens tankyrase 1 binding protein 1, 182kDa (TNKS1BP1), mRNA [NM_033396]	85456
A_23_P125588	2.1	1.8	1.0	-1.1	TNMD	Homo sapiens tenomodulin (TNMD), mRNA [NM_022144]	64102
A_32_P480177	1.0	-18.4	-1.1	-1.0	TNN	Homo sapiens tenascin N (TNN), mRNA [NM_022093]	63923
A_23_P166823	1.0	1.7	-2.2	1.5	TNNC1	Homo sapiens troponin C type 1 (slow) (TNNC1), mRNA [NM_003280]	7134

A_23_P131825	-1.1	-5.4	-1.2	1.7	TNNC2	Homo sapiens troponin C type 2 (fast) (TNNC2), mRNA [NM_003279]	7125
A_23_P67453	-2.9	2.0	-1.1	1.6	TNNI3	Homo sapiens troponin I type 3 (cardiac) (TNNI3), mRNA [NM_000363]	7137
A_23_P200024	1.3	-1.7	-1.4	-3.1	TNNI3K	Homo sapiens TNNI3 interacting kinase (TNNI3K), mRNA [NM_015978]	51086
A_23_P56050	1.3	-6.4	-1.6	1.2	TNNT1	Homo sapiens troponin T type 1 (skeletal, slow) (TNNT1), transcript variant 2, mRNA [NM_001126132]	7138

A_24_P257022	-2.0	-1.1	-1.2	-2.4	TNNT2	Homo sapiens troponin T type 2 (cardiac) (TNNT2), transcript variant 1, mRNA [NM_000364]	7139
A_23_P34700	2.7	-1.2	2.8	1.3	TNNT2	Homo sapiens troponin T type 2 (cardiac) (TNNT2), transcript variant 1, mRNA [NM_000364]	7139
A_23_P129382	-5.3	-2.5	1.1	2.3	TNP2	Homo sapiens transition protein 2 (during histone to protamine replacement) (TNP2), mRNA [NM_005425]	7142
A_23_P326785	-2.0	1.0	1.0	1.0	TNP2	Homo sapiens transition protein 2 (during histone to protamine replacement) (TNP2), mRNA [NM_005425]	7142

A_23_P156101	-3.1	-2.0	-1.2	-2.1	TNPO1	Homo sapiens transportin 1 (TNPO1), transcript variant 1, mRNA [NM_002270]	3842
A_24_P44891	3.4	2.1	1.3	1.9	TNPO2	Homo sapiens transportin 2 (TNPO2), transcript variant 2, mRNA [NM_013433]	30000
A_23_P45864	1.1	-1.7	1.0	-2.5	TNR	Homo sapiens tenascin R (restrictin, janusin) (TNR), mRNA [NM_003285]	7143
A_24_P400528	-1.3	-1.1	-2.0	-1.2	TNRC18	Homo sapiens trinucleotide repeat containing 18 (TNRC18), mRNA [NM_001080495]	84629

A_32_P6415	1.0	-1.0	-2.3	-1.5	TNRC6B	Homo sapiens trinucleotide repeat containing 6B (TNRC6B), transcript variant 2, mRNA [NM_015088]	23112
A_24_P786172	1.1	-1.0	-2.2	-2.5	TNRC6B	Homo sapiens trinucleotide repeat containing 6B (TNRC6B), transcript variant 2, mRNA [NM_015088]	23112
A_23_P392501	-1.1	1.5	-4.4	-1.4	TNRC6C	Homo sapiens trinucleotide repeat containing 6C (TNRC6C), transcript variant 1, mRNA [NM_001142640]	57690
A_23_P15765	-1.0	-23.5	-1.0	-1.0	TNRC6C	Homo sapiens trinucleotide repeat containing 6C (TNRC6C), transcript variant 2, mRNA [NM_018996]	57690

A_24_P718833	-3.1	1.0	1.7	-1.3	TNRC6C	trinucleotide repeat containing 6C [Source:HGNC Symbol;Acc:29318] [ENST00000455761]	57690
A_24_P136453	-1.3	1.0	-1.1	-2.1	TNS1	Homo sapiens tensin 1 (TNS1), mRNA [NM_022648]	7145
A_23_P351724	-2.8	-2.0	-1.4	-25.6	TNS1	Homo sapiens tensin 1 (TNS1), mRNA [NM_022648]	7145
A_23_P215642	1.3	2.1	1.1	-1.3	TNS3	Homo sapiens tensin 3 (TNS3), mRNA [NM_022748]	64759
A_23_P156708	1.0	1.2	-2.5	1.2	TNXB	Homo sapiens tenascin XB (TNXB), transcript variant XB-S, mRNA [NM_032470]	7148

A_24_P33217	1.9	3.2	1.6	-1.4	TNXB	Homo sapiens tenascin XB (TNXB), transcript variant XB, mRNA [NM_019105]	7148
A_24_P15834	-2.0	2.3	1.1	1.2	TNXB	Homo sapiens tenascin XB (TNXB), transcript variant XB, mRNA [NM_019105]	7148
A_23_P164179	1.9	2.4	-1.6	1.2	TOB1	Homo sapiens transducer of ERBB2, 1 (TOB1), transcript variant 1, mRNA [NM_005749]	10140
A_24_P19828	-2.2	-1.8	-1.6	-1.0	TOE1	Homo sapiens target of EGR1, member 1 (nuclear) (TOE1), mRNA [NM_025077]	114034

A_23_P75903	1.0	-5.4	-5.6	-2.6	TOLLIP	Homo sapiens toll interacting protein (TOLLIP), mRNA [NM_019009]	54472
A_23_P103084	2.0	3.3	3.4	1.3	TOM1	Homo sapiens target of myb1 (chicken) (TOM1), transcript variant 1, mRNA [NM_005488]	10043
A_23_P118493	-3.8	1.3	1.3	-3.0	TOM1L1	Homo sapiens target of myb1 (chicken)-like 1 (TOM1L1), mRNA [NM_005486]	10040
A_23_P141180	1.0	1.0	-2.8	1.4	TOM1L2	Homo sapiens target of myb1-like 2 (chicken) (TOM1L2), transcript variant 3, mRNA [NM_001082968]	146691

A_24_P216765	-2.7	-4.1	-3.2	-2.4	TOMM20	Homo sapiens translocase of outer mitochondrial membrane 20 homolog (yeast) (TOMM20), nuclear gene encoding mitochondrial protein, mRNA [NM_014765]	9804
A_24_P117942	1.7	2.7	-2.5	-1.3	TOMM20L	Homo sapiens translocase of outer mitochondrial membrane 20 homolog (yeast)-like (TOMM20L), nuclear gene encoding mitochondrial protein, mRNA [NM_207377]	387990

A_24_P199097	1.6	4.1	2.0	2.5	TOMM34	Homo sapiens translocase of outer mitochondrial membrane 34 (TOMM34), nuclear gene encoding mitochondrial protein, mRNA [NM_006809]	10953
A_24_P15754	-18.9	-1.1	-2.1	-1.0	TOMM40	Homo sapiens translocase of outer mitochondrial membrane 40 homolog (yeast) (TOMM40), nuclear gene encoding mitochondrial protein, transcript variant 3, mRNA [NM_006114]	10452

A_23_P845	-2.3	1.0	1.0	1.1	TOMM40L	Homo sapiens translocase of outer mitochondrial membrane 40 homolog (yeast)-like (TOMM40L), nuclear gene encoding mitochondrial protein, mRNA [NM_032174]	84134
A_32_P75299	1.2	1.3	-1.1	-3.3	TOMM5	Homo sapiens translocase of outer mitochondrial membrane 5 homolog (yeast) (TOMM5), nuclear gene encoding mitochondrial protein, transcript variant 3, mRNA [NM_001134484]	401505

A_23_P214474	1.6	2.3	1.9	1.5	TOMM6	Homo sapiens translocase of outer mitochondrial membrane 6 homolog (yeast) (TOMM6), nuclear gene encoding mitochondrial protein, mRNA [NM_001134493]	100188893
A_24_P1054	1.0	-3.0	-1.3	-1.0	TONSL	Homo sapiens tonsoku-like, DNA repair protein (TONSL), mRNA [NM_013432]	4796
A_24_P374382	-1.0	-1.0	-2.1	-1.6	TOP1P2	Homo sapiens topoisomerase (DNA) I pseudogene 2 (TOP1P2), non-coding RNA [NR_001283]	7152

A_23_P121095	-3.2	-2.6	-2.8	-3.0	TOP2B	Homo sapiens topoisomerase (DNA) II beta 180kDa (TOP2B), mRNA [NM_001068]	7155
A_23_P80213	1.2	-15.7	-7.9	-6.4	TOP3B	Homo sapiens topoisomerase (DNA) III beta (TOP3B), mRNA [NM_003935]	8940
A_24_P267997	1.7	-5.7	1.0	1.0	TOR1AIP1	Homo sapiens torsin A interacting protein 1 (TOR1AIP1), mRNA [NM_015602]	26092
A_24_P130962	-3.0	-1.3	-1.3	1.1	TOR3A	Homo sapiens torsin family 3, member A (TOR3A), mRNA [NM_022371]	64222

A_24_P119036	5.6	7.7	5.6	3.2	TOX3	Homo sapiens TOX high mobility group box family member 3 (TOX3), transcript variant 2, mRNA [NM_001146188]	27324
A_24_P152398	-2.5	-1.7	-1.3	-1.8	TP53AIP1	Homo sapiens tumor protein p53 regulated apoptosis inducing protein 1 (TP53AIP1), nuclear gene encoding mitochondrial protein, transcript variant 2, mRNA [NM_001195195]	63970
A_24_P160969	1.0	-1.0	-1.0	-3.2	TP53I11	Homo sapiens tumor protein p53 inducible protein 11 (TP53I11), mRNA [NM_001076787]	9537

A_24_P185205	1.3	1.4	-1.2	-5.0	TP53I13	Homo sapiens tumor protein p53 inducible protein 13 (TP53I13), mRNA [NM_138349]	90313
A_23_P145895	-1.0	-1.0	-1.1	-4.1	TP53TG1	Homo sapiens TP53 target 1 (non-protein coding) (TP53TG1), non-coding RNA [NR_015381]	11257
A_24_P274842	1.2	1.1	-2.6	-2.7	TP53TG1	Homo sapiens TP53 target 1 (non-protein coding) (TP53TG1), non-coding RNA [NR_015381]	11257
A_23_P37994	1.9	1.0	2.1	1.1	TP53TG3	Homo sapiens TP53 target 3 (TP53TG3), mRNA [NM_016212]	24150

A_24_P227971	2.9	4.4	8.6	4.0	TP53TG3	Homo sapiens TP53 target 3 (TP53TG3), mRNA [NM_016212]	24150
A_24_P413470	3.0	3.6	2.7	3.8	TP73	Homo sapiens tumor protein p73 (TP73), transcript variant 1, mRNA [NM_005427]	7161
A_24_P582889	1.1	1.1	1.5	-16.3	TPBG	trophoblast glycoprotein [Source:HGNC Symbol;Acc:12004] [ENST00000369750]	7162
A_23_P308119	3.5	2.7	1.0	1.6	TPCN1	Homo sapiens two pore segment channel 1 (TPCN1), transcript variant 2, mRNA [NM_017901]	53373

A_23_P218086	1.2	-4.6	1.0	-1.3	TPCN1	Homo sapiens two pore segment channel 1 (TPCN1), transcript variant 1, mRNA [NM_001143819]	53373
A_23_P98159	1.1	-2.3	1.2	-1.4	TPCN2	Homo sapiens two pore segment channel 2 (TPCN2), mRNA [NM_139075]	219931
A_24_P166807	1.5	-1.5	-2.7	-1.9	TPD52	Homo sapiens tumor protein D52 (TPD52), transcript variant 1, mRNA [NM_001025252]	7163
A_23_P131771	-1.5	-3.7	-1.0	-2.1	TPD52L2	Homo sapiens tumor protein D52-like 2 (TPD52L2), transcript variant 1, mRNA [NM_199360]	7165

A_23_P20544	-1.1	-1.1	-2.3	-1.1	TPD52L3	Homo sapiens tumor protein D52-like 3 (TPD52L3), transcript variant 1, mRNA [NM_033516]	89882
A_24_P309216	-1.1	-3.2	-1.6	-1.4	TPD52L3	Homo sapiens tumor protein D52-like 3 (TPD52L3), transcript variant 1, mRNA [NM_033516]	89882
A_24_P709844	1.0	-1.0	-3.0	-1.0	TPH1	tryptophan hydroxylase 1 [Source:HGNC Symbol;Acc:12008] [ENST00000250018]	7166

A_24_P358328	-3.2	2.0	1.1	1.5	TPI1P2	Homo sapiens triosephosphate isomerase 1 pseudogene 2 (TPI1P2), non-coding RNA [NR_002187]	286016
A_24_P44462	-4.5	-2.5	-7.2	-3.4	TPM1	Homo sapiens tropomyosin 1 (alpha) (TPM1), transcript variant 5, mRNA [NM_000366]	7168
A_23_P363344	-1.4	1.0	1.0	2.2	TPM1	Homo sapiens tropomyosin 1 (alpha) (TPM1), transcript variant 5, mRNA [NM_000366]	7168
A_24_P924721	1.4	1.1	-8.3	1.3	TPM1	Homo sapiens tropomyosin 1 (alpha) (TPM1), transcript variant 6, mRNA [NM_001018008]	7168

A_23_P391586	1.0	1.0	-23.6	-1.0	TPM1	Homo sapiens tropomyosin 1 (alpha) (TPM1), transcript variant 3, mRNA [NM_001018004]	7168
A_32_P119197	5.2	5.2	4.6	5.4	TPM3	Homo sapiens tropomyosin 3 (TPM3), transcript variant 3, mRNA [NM_001043352]	7170
A_32_P162338	-1.5	-2.4	-1.6	1.2	TPM3	Homo sapiens tropomyosin 3 (TPM3), transcript variant 2, mRNA [NM_153649]	7170
A_23_P141974	-1.5	-2.1	-1.2	1.4	TPM4	Homo sapiens tropomyosin 4 (TPM4), transcript variant 2, mRNA [NM_003290]	7171

A_24_P82880	1.5	2.0	1.4	1.1	TPM4	Homo sapiens tropomyosin 4 (TPM4), transcript variant 2, mRNA [NM_003290]	7171
A_24_P257224	-2.9	-1.2	-1.2	-1.2	TPO	Homo sapiens thyroid peroxidase (TPO), transcript variant 1, mRNA [NM_000547]	7173
A_23_P58671	2.1	2.1	2.1	2.1	TPPP	Homo sapiens tubulin polymerization promoting protein (TPPP), mRNA [NM_007030]	11076
A_23_P26386	-1.1	3.1	2.4	1.7	TPPP3	Homo sapiens tubulin polymerization-promoting protein family member 3 (TPPP3), mRNA [NM_016140]	51673

A_24_P586712	-1.1	-1.0	-1.0	-7.1	TPRG1	Homo sapiens tumor protein p63 regulated 1 (TPRG1), mRNA [NM_198485]	285386
A_23_P5757	1.1	1.0	-2.9	1.0	TPRKB	Homo sapiens TP53RK binding protein (TPRKB), mRNA [NM_016058]	51002
A_24_P323997	2.8	2.8	2.6	2.6	TPRX1	Homo sapiens tetra-peptide repeat homeobox 1 (TPRX1), mRNA [NM_198479]	284355
A_23_P37702	-2.4	1.0	-1.0	-1.1	TPSAB1	Homo sapiens tryptase alpha/beta 1 (TPSAB1), mRNA [NM_003294]	7177

A_23_P132226	1.0	-14.2	1.0	-1.1	TPST2	Homo sapiens tyrosylprotein sulfotransferase 2 (TPST2), transcript variant 1, mRNA [NM_001008566]	8459
A_24_P306726	-1.7	-1.2	-2.4	-8.9	TPT1	Homo sapiens tumor protein, translationally-controlled 1 (TPT1), mRNA [NM_003295]	7178
A_24_P179351	-1.8	1.2	2.6	1.3	TPT1	Homo sapiens tumor protein, translationally-controlled 1 (TPT1), mRNA [NM_003295]	7178

A_24_P118052	1.4	1.2	-2.2	-5.3	TPTE2	Homo sapiens transmembrane phosphoinositide 3-phosphatase and tensin homolog 2 (TPTE2), transcript variant 3, mRNA [NM_199254]	93492
A_32_P154835	-1.1	-2.1	-1.0	1.0	TPTE2P1	Homo sapiens transmembrane phosphoinositide 3-phosphatase and tensin homolog 2 pseudogene 1 (TPTE2P1), non-coding RNA [NR_026730]	646405
A_24_P145268	2.7	2.7	2.6	2.8	TPTE2P2	Homo sapiens TPTE pseudogene 1, mRNA (cDNA clone IMAGE:5268927). [BC041842]	644623

A_32_P48134	-2.7	1.3	1.2	1.2	TPTE2P3	Homo sapiens transmembrane phosphoinositide 3-phosphatase and tensin homolog 2 pseudogene 3 (TPTE2P3), non-coding RNA [NR_002793]	220115
A_32_P89827	-2.9	-1.1	-2.1	-2.2	TPTE2P6	Homo sapiens transmembrane phosphoinositide 3-phosphatase and tensin homolog 2 pseudogene 6 (TPTE2P6), non-coding RNA [NR_002815]	374491

A_23_P31389	-26.3	-1.7	-1.5	-43.5	TRA2A	Homo sapiens transformer 2 alpha homolog (Drosophila) (TRA2A), mRNA [NM_013293]	29896
A_24_P145911	-1.9	-1.6	-3.4	-1.3	TRA2B	transformer 2 beta homolog (Drosophila) [Source:HGNC Symbol;Acc:10781] [ENST00000342294]	6434
A_23_P166548	-2.2	-4.6	-5.3	-2.3	TRABD	Homo sapiens TraB domain containing (TRABD), mRNA [NM_025204]	80305
A_23_P54649	1.4	2.4	2.1	1.2	TRADD	Homo sapiens TNFRSF1A-associated via death domain (TRADD), mRNA [NM_003789]	8717

A_23_P5359	-1.0	-1.1	-5.5	-1.6	TRAF3IP1	Homo sapiens TNF receptor-associated factor 3 interacting protein 1 (TRAF3IP1), transcript variant 1, mRNA [NM_015650]	26146
A_23_P110879	-9.4	2.0	1.8	1.7	TRAF3IP2	Homo sapiens TRAF3 interacting protein 2 (TRAF3IP2), transcript variant 2, mRNA [NM_147686]	10758
A_23_P323761	-8.9	-1.3	-1.3	-1.9	TRAF3IP3	Homo sapiens TRAF3 interacting protein 3 (TRAF3IP3), mRNA [NM_025228]	80342

A_23_P201731	-1.0	1.3	-2.4	-6.6	TRAF5	Homo sapiens TNF receptor-associated factor 5 (TRAF5), transcript variant 1, mRNA [NM_004619]	7188
A_23_P75921	1.6	1.0	2.4	1.1	TRAF6	Homo sapiens TNF receptor-associated factor 6 (TRAF6), transcript variant 1, mRNA [NM_145803]	7189
A_23_P206474	-1.4	-1.3	-1.1	2.9	TRAF7	Homo sapiens TNF receptor-associated factor 7 (TRAF7), mRNA [NM_032271]	84231
A_23_P128541	-2.2	-1.0	-1.1	1.1	TRAFD1	Homo sapiens TRAF-type zinc finger domain containing 1 (TRAFD1), transcript variant 2, mRNA [NM_006700]	10906

A_23_P170491	-1.1	-1.1	-4.2	-1.2	TRAIP	Homo sapiens TRAF interacting protein (TRAIP), mRNA [NM_005879]	10293
A_24_P341279	3.5	2.2	-1.0	-1.3	TRAK1	Homo sapiens trafficking protein, kinesin binding 1 (TRAK1), transcript variant 2, mRNA [NM_014965]	22906
A_23_P132536	-1.0	3.4	2.1	1.3	TRAK1	Homo sapiens trafficking protein, kinesin binding 1 (TRAK1), transcript variant 1, mRNA [NM_001042646]	22906
A_23_P372974	-4.1	1.0	-1.0	-1.0	TRAM1L1	Homo sapiens translocation associated membrane protein 1-like 1 (TRAM1L1), mRNA [NM_152402]	133022

A_23_P58937	-1.8	1.0	-3.5	-1.8	TRAM2	Homo sapiens translocation associated membrane protein 2 (TRAM2), mRNA [NM_012288]	9697
A_23_P3849	1.4	2.0	1.3	-1.0	TRAP1	Homo sapiens TNF receptor-associated protein 1 (TRAP1), mRNA [NM_016292]	10131
A_23_P16032	-1.1	1.0	1.3	-2.4	TRAPPC2P1	Homo sapiens trafficking protein particle complex 2 pseudogene 1 (TRAPPC2P1), non-coding RNA [NR_002166]	10597
A_23_P115636	1.7	1.7	-1.1	2.1	TRDMT1	Homo sapiens tRNA aspartic acid methyltransferase 1 (TRDMT1), mRNA [NM_004412]	1787

A_23_P42548	-1.3	-1.3	1.0	2.0	TRDN	Homo sapiens triadin (TRDN), transcript variant 1, mRNA [NM_006073]	10345
A_23_P104819	-2.1	-3.9	1.1	-1.8	TREH	Homo sapiens trehalase (brush-border membrane glycoprotein) (TREH), mRNA [NM_007180]	11181
A_24_P12397	1.0	1.2	-2.1	-1.5	TREM2	Homo sapiens triggering receptor expressed on myeloid cells 2 (TREM2), mRNA [NM_018965]	54209
A_23_P156550	2.7	-1.2	4.8	2.1	TREML1	triggering receptor expressed on myeloid cells-like 1 [Source:HGNC Symbol;Acc:20434] [ENST00000437044]	340205

A_24_P280660	-1.7	1.1	-1.4	-2.1	TREML1	Homo sapiens triggering receptor expressed on myeloid cells-like 1 (TREML1), mRNA [NM_178174]	340205
A_24_P214858	-1.0	-1.0	2.0	-1.7	TREML2	Homo sapiens triggering receptor expressed on myeloid cells-like 2 (TREML2), mRNA [NM_024807]	79865
A_24_P170439	1.1	-3.5	-1.8	-2.2	TREML3	Homo sapiens triggering receptor expressed on myeloid cells-like 3 (TREML3), non-coding RNA [NR_027256]	340206

A_24_P655458	1.8	2.1	1.6	1.6	TREML4	Homo sapiens triggering receptor expressed on myeloid cells-like 4 (TREML4), mRNA [NM_198153]	285852
A_24_P217904	1.0	-1.0	-1.1	-2.3	TRERF1	Homo sapiens transcriptional regulating factor 1 (TRERF1), mRNA [NM_033502]	55809
A_23_P59022	-2.8	1.0	1.0	1.1	TRERF1	Homo sapiens transcriptional regulating factor 1 (TRERF1), mRNA [NM_033502]	55809
A_23_P218879	-1.7	-2.3	-3.8	-1.7	TREX1	Homo sapiens three prime repair exonuclease 1 (TREX1), transcript variant 1, mRNA [NM_016381]	11277

A_23_P219223	-6.1	-2.5	-2.5	-2.2	TREX2	Homo sapiens three prime repair exonuclease 2 (TREX2), mRNA [NM_080701]	11219
A_23_P366983	-1.0	-1.1	-1.6	-2.6	TRHDE	Homo sapiens thyrotropin-releasing hormone degrading enzyme (TRHDE), mRNA [NM_013381]	29953
A_23_P123503	-1.6	-1.6	-3.7	-1.6	TRIB1	Homo sapiens tribbles homolog 1 (Drosophila) (TRIB1), mRNA [NM_025195]	10221
A_23_P122959	9.3	9.6	9.3	5.4	TRIL	Homo sapiens TLR4 interactor with leucine-rich repeats (TRIL), mRNA [NM_014817]	9865

A_23_P214578	-1.7	-1.7	-2.5	1.0	TRIM10	Homo sapiens tripartite motif containing 10 (TRIM10), transcript variant 1, mRNA [NM_006778]	10107
A_24_P166431	-2.5	-1.5	-3.1	-1.9	TRIM10	Homo sapiens tripartite motif containing 10 (TRIM10), transcript variant 1, mRNA [NM_006778]	10107
A_23_P420831	1.8	3.8	2.4	1.7	TRIM10	Homo sapiens tripartite motif containing 10 (TRIM10), transcript variant 2, mRNA [NM_052828]	10107
A_23_P87973	-1.2	1.0	-1.2	-3.1	TRIM13	Homo sapiens tripartite motif containing 13 (TRIM13), transcript variant 3, mRNA [NM_213590]	10206

A_23_P425752	-1.7	1.0	-3.3	-1.2	TRIM14	Homo sapiens tripartite motif containing 14 (TRIM14), transcript variant 2, mRNA [NM_033219]	9830
A_23_P216655	1.2	-1.0	-7.9	-1.0	TRIM14	Homo sapiens tripartite motif containing 14 (TRIM14), transcript variant 1, mRNA [NM_014788]	9830
A_23_P214554	-13.5	-1.0	-1.1	2.0	TRIM15	Homo sapiens tripartite motif containing 15 (TRIM15), mRNA [NM_033229]	89870
A_23_P129903	-2.1	-1.9	-1.3	-2.7	TRIM16L	Homo sapiens tripartite motif containing 16-like (TRIM16L), mRNA [NM_001037330]	147166

A_24_P208909	-4.7	-1.0	-1.1	-1.1	TRIM2	Homo sapiens tripartite motif containing 2 (TRIM2), transcript variant 1, mRNA [NM_015271]	23321
A_23_P47691	1.0	1.0	2.0	1.0	TRIM21	Homo sapiens tripartite motif containing 21 (TRIM21), mRNA [NM_003141]	6737
A_24_P172481	1.0	-2.7	-1.1	-1.1	TRIM22	Homo sapiens tripartite motif containing 22 (TRIM22), transcript variant 1, mRNA [NM_006074]	10346
A_24_P898583	-8.1	1.5	1.4	1.0	TRIM26	Homo sapiens tripartite motif containing 26 (TRIM26), transcript variant 1, mRNA [NM_003449]	7726

A_24_P377394	1.2	1.5	-7.8	1.1	TRIM26	Homo sapiens tripartite motif containing 26 (TRIM26), transcript variant 1, mRNA [NM_003449]	7726
A_24_P163405	5.1	6.7	5.4	1.7	TRIM27	Homo sapiens tripartite motif containing 27 (TRIM27), mRNA [NM_006510]	5987
A_23_P89884	1.8	2.2	1.6	1.3	TRIM28	Homo sapiens tripartite motif containing 28 (TRIM28), mRNA [NM_005762]	10155
A_23_P203267	-6.4	-1.2	-1.8	-1.4	TRIM29	Homo sapiens tripartite motif containing 29 (TRIM29), mRNA [NM_012101]	23650

A_23_P340123	-4.8	1.1	-1.6	-1.3	TRIM29	Homo sapiens tripartite motif containing 29 (TRIM29), mRNA [NM_012101]	23650
A_23_P122493	-3.9	-2.1	-1.2	-5.2	TRIM31	Homo sapiens tripartite motif containing 31 (TRIM31), mRNA [NM_007028]	11074
A_24_P34344	-1.3	-1.3	-1.3	-2.1	TRIM33	Homo sapiens tripartite motif containing 33 (TRIM33), transcript variant b, mRNA [NM_033020]	51592
A_23_P23526	-2.7	-2.7	-2.8	2.2	TRIM33	Homo sapiens tripartite motif containing 33 (TRIM33), transcript variant b, mRNA [NM_033020]	51592

A_24_P398323	1.6	-2.0	-1.0	2.1	TRIM34	Homo sapiens tripartite motif containing 34 (TRIM34), transcript variant 4, mRNA [NM_001003827]	53840
A_23_P124190	-1.0	-1.0	-1.0	-3.5	TRIM34	Homo sapiens tripartite motif containing 34 (TRIM34), transcript variant 3, mRNA [NM_130390]	53840
A_24_P153853	-1.0	-24.1	1.0	-1.0	TRIM37	Homo sapiens tripartite motif containing 37 (TRIM37), transcript variant 2, mRNA [NM_001005207]	4591
A_24_P294851	1.3	-3.8	1.6	-2.4	TRIM38	Homo sapiens tripartite motif containing 38 (TRIM38), mRNA [NM_006355]	10475

A_24_P89413	1.0	-3.0	1.1	1.0	TRIM39	Homo sapiens tripartite motif containing 39 (TRIM39), transcript variant 1, mRNA [NM_021253]	56658
A_24_P894776	-3.4	1.0	1.0	1.1	TRIM40	tripartite motif containing 40 [Source:HGNC Symbol;Acc:18736] [ENST00000462556]	135644
A_23_P402778	1.0	-1.0	-2.8	-1.1	TRIM40	Homo sapiens tripartite motif containing 40 (TRIM40), mRNA [NM_138700]	135644
A_23_P167521	1.5	-14.8	2.3	1.1	TRIM41	Homo sapiens tripartite motif containing 41 (TRIM41), transcript variant 1, mRNA [NM_033549]	90933

A_24_P937656	5.5	2.0	4.1	5.0	TRIM42	Homo sapiens mRNA; cDNA DKFZp434 A029 (from clone DKFZp434 A029). [AL833848]	287015
A_23_P17173	-2.5	-3.8	-1.2	2.3	TRIM43	Homo sapiens tripartite motif containing 43 (TRIM43), mRNA [NM_1388 00]	129868
A_23_P139297	2.7	2.6	1.5	2.4	TRIM44	Homo sapiens tripartite motif containing 44 (TRIM44), mRNA [NM_0175 83]	54765
A_23_P160523	-1.3	1.1	-2.1	-2.0	TRIM45	Homo sapiens tripartite motif containing 45 (TRIM45), transcript variant 1, mRNA [NM_0251 88]	80263

A_23_P46222	-1.1	-1.2	-2.3	1.3	TRIM46	tripartite motif containing 46 [Source:HGNC Symbol;Acc:19019] [ENST00000368385]	80128
A_23_P64306	-1.1	2.6	1.7	1.4	TRIM48	Homo sapiens tripartite motif containing 48 (TRIM48), mRNA [NM_024114]	79097
A_23_P350107	-1.2	-1.2	-1.3	-27.4	TRIM56	Homo sapiens tripartite motif containing 56 (TRIM56), mRNA [NM_030961]	81844
A_23_P201901	1.2	3.5	1.4	1.1	TRIM58	Homo sapiens mRNA; cDNA DKFZp686N09198 (from clone DKFZp686N09198); complete cds. [BX537432]	25893

A_23_P314052	-1.0	-1.0	5.0	-1.9	TRIM58	Homo sapiens tripartite motif containing 58 (TRIM58), mRNA [NM_015431]	25893
A_32_P72341	-6.0	-1.0	-1.0	-1.0	TRIM59	Homo sapiens tripartite motif containing 59 (TRIM59), mRNA [NM_173084]	286827
A_24_P139867	-1.1	-2.9	-2.1	-1.7	TRIM62	Homo sapiens tripartite motif containing 62 (TRIM62), mRNA [NM_018207]	55223
A_23_P250485	-3.5	-2.5	-2.6	-2.4	TRIM62	Homo sapiens tripartite motif containing 62 (TRIM62), mRNA [NM_018207]	55223

A_24_P139861	1.8	3.9	2.2	1.7	TRIM62	Homo sapiens tripartite motif containing 62 (TRIM62), mRNA [NM_018207]	55223
A_23_P112602	2.1	4.1	2.1	1.5	TRIM62	Homo sapiens tripartite motif containing 62 (TRIM62), mRNA [NM_018207]	55223
A_32_P396186	-1.0	-1.0	-1.0	-2.3	TRIM66	Homo sapiens tripartite motif containing 66 (TRIM66), mRNA [NM_014818]	9866
A_23_P48826	1.5	3.4	-1.3	1.0	TRIM69	Homo sapiens tripartite motif containing 69 (TRIM69), transcript variant a, mRNA [NM_182985]	140691

A_24_P286190	2.9	4.1	-1.3	-1.1	TRIM7	Homo sapiens tripartite motif containing 7 (TRIM7), transcript variant 6, mRNA [NM_033342]	81786
A_24_P109375	1.8	4.0	2.8	1.4	TRIM74	Homo sapiens tripartite motif containing 74 (TRIM74), mRNA [NM_198853]	378108
A_23_P205637	4.5	-1.0	3.8	4.3	TRIM9	Homo sapiens tripartite motif containing 9 (TRIM9), transcript variant 1, mRNA [NM_015163]	114088
A_24_P42603	-5.9	-1.2	-1.3	-1.3	TRIO	Homo sapiens triple functional domain (PTPRF interacting) (TRIO), mRNA [NM_007118]	7204

A_23_P408328	6.9	2.9	4.3	8.2	TRIOBP	Homo sapiens TRIO and F-actin binding protein (TRIOBP), transcript variant 2, mRNA [NM_138632]	11078
A_23_P50349	1.3	-2.5	1.5	1.4	TRIP10	Homo sapiens thyroid hormone receptor interactor 10 (TRIP10), mRNA [NM_004240]	9322
A_32_P188178	1.2	-1.3	-1.6	-2.3	TRIP12	Homo sapiens thyroid hormone receptor interactor 12 (TRIP12), mRNA [NM_004238]	9320
A_23_P147984	52.1	27.7	42.4	53.0	TRIP12	Homo sapiens thyroid hormone receptor interactor 12 (TRIP12), mRNA [NM_004238]	9320

A_23_P16683	-2.1	-1.1	-2.3	-1.5	TRMT1	Homo sapiens TRM1 tRNA methyltransferase 1 homolog (S. cerevisiae) (TRMT1), transcript variant 1, mRNA [NM_017722]	55621
A_23_P12992	-1.1	-2.5	1.9	-1.0	TRMT112	Homo sapiens tRNA methyltransferase 11-2 homolog (S. cerevisiae) (TRMT112), mRNA [NM_016404]	51504
A_24_P190877	1.1	-2.3	-1.1	-1.0	TRMT1L	Homo sapiens TRM1 tRNA methyltransferase 1-like (TRMT1L), transcript variant 1, mRNA [NM_030934]	81627

A_24_P305662	2.0	-1.0	2.1	1.0	TRMT2A	Homo sapiens TRM2 tRNA methyltransferase 2 homolog A (S. cerevisiae) (TRMT2A), transcript variant 1, mRNA [NM_022727]	27037
A_23_P73604	-3.2	1.8	-1.2	1.3	TRMT2B	Homo sapiens TRM2 tRNA methyltransferase 2 homolog B (S. cerevisiae) (TRMT2B), transcript variant 1, mRNA [NM_024917]	79979
A_23_P321630	2.0	1.4	-1.0	-1.2	TRMT5	Homo sapiens TRM5 tRNA methyltransferase 5 homolog (S. cerevisiae) (TRMT5), mRNA [NM_020810]	57570

A_23_P210747	-1.9	-2.4	-1.1	1.3	TRMT6	Homo sapiens tRNA methyltransferase 6 homolog (S. cerevisiae) (TRMT6), mRNA [NM_015939]	51605
A_24_P916195	1.1	-1.2	-1.2	-3.3	TRMU	tRNA 5-methylaminomethyl-2-thiouridylate methyltransferase [Source:HGNC Symbol;Acc:25481] [ENST00000486620]	55687

A_23_P80389	1.3	1.2	1.1	2.0	TRMU	Homo sapiens tRNA 5-methylaminomethyl-2-thiouridylate methyltransferase (TRMU), nuclear gene encoding mitochondrial protein, transcript variant 1, mRNA [NM_018006]	55687
A_24_P393787	-3.0	-1.9	-2.6	-2.8	TRNAU1AP	Homo sapiens tRNA selenocysteine 1 associated protein 1 (TRNAU1AP), transcript variant 1, mRNA [NM_017846]	54952

A_23_P372331	-2.1	-2.5	-2.6	-2.5	TRNT1	Homo sapiens tRNA nucleotidyl transferase, CCA-adding, 1 (TRNT1), nuclear gene encoding mitochondrial protein, mRNA [NM_182916]	51095
A_24_P235338	1.6	-1.9	1.5	2.1	TRPA1	Homo sapiens transient receptor potential cation channel, subfamily A, member 1 (TRPA1), mRNA [NM_007332]	8989

A_23_P105873	1.1	-1.1	1.5	4.9	TRPC4	Homo sapiens transient receptor potential cation channel, subfamily C, member 4 (TRPC4), transcript variant alpha, mRNA [NM_016179]	7223
A_23_P154801	-1.8	-2.6	-1.8	1.6	TRPC4AP	Homo sapiens transient receptor potential cation channel, subfamily C, member 4 associated protein (TRPC4AP), transcript variant 1, mRNA [NM_015638]	26133

A_23_P129225	-8.4	-1.3	-1.4	-1.5	TRPM1	Homo sapiens transient receptor potential cation channel, subfamily M, member 1 (TRPM1), transcript variant 2, mRNA [NM_002420]	4308
A_24_P27977	2.2	-1.4	2.0	2.0	TRPM2	Homo sapiens transient receptor potential cation channel, subfamily M, member 2 (TRPM2), transcript variant 1, mRNA [NM_003307]	7226
A_23_P428973	-1.7	-1.7	-1.9	-29.0	TRPM3	transient receptor potential cation channel, subfamily M, member 3 [Source:HGNC Symbol;Acc:17992] [ENST00000354500]	80036

A_23_P136310	1.0	-1.0	-1.1	-2.7	TRPM3	Homo sapiens transient receptor potential cation channel, subfamily M, member 3 (TRPM3), transcript variant 9, mRNA [NM_001007471]	80036
A_23_P114061	-1.1	1.2	-2.3	9.4	TRPM3	Homo sapiens transient receptor potential cation channel, subfamily M, member 3 (TRPM3), transcript variant 9, mRNA [NM_001007471]	80036
A_23_P153529	2.8	-1.1	-1.0	-1.1	TRPM4	Homo sapiens transient receptor potential cation channel, subfamily M, member 4 (TRPM4), transcript variant 1, mRNA [NM_017636]	54795

A_23_P87279	1.4	-1.1	1.1	-2.0	TRPM5	Homo sapiens transient receptor potential cation channel, subfamily M, member 5 (TRPM5), mRNA [NM_014555]	29850
A_23_P134755	-3.8	-1.9	-2.4	1.5	TRPS1	Homo sapiens trichorhinophalangeal syndrome I (TRPS1), mRNA [NM_014112]	7227
A_23_P98248	7.2	4.3	3.3	-1.0	TRPT1	Homo sapiens tRNA phosphotransferase 1 (TRPT1), transcript variant 1, mRNA [NM_001033678]	83707

A_23_P207911	6.7	5.8	3.8	5.3	TRPV2	Homo sapiens transient receptor potential cation channel, subfamily V, member 2 (TRPV2), mRNA [NM_016113]	51393
A_23_P49862	-21.8	-25.7	-5.0	-1.2	TRPV3	Homo sapiens transient receptor potential cation channel, subfamily V, member 3 (TRPV3), mRNA [NM_145068]	162514
A_23_P93973	2.1	1.1	-1.3	-1.4	TRPV5	Homo sapiens transient receptor potential cation channel, subfamily V, member 5 (TRPV5), mRNA [NM_019841]	56302

A_23_P71170	3.5	1.7	1.6	2.2	TRPV6	Homo sapiens transient receptor potential cation channel, subfamily V, member 6 (TRPV6), mRNA [NM_018646]	55503
A_24_P329635	-1.0	-4.6	-1.7	-1.2	TSC1	Homo sapiens tuberous sclerosis 1 (TSC1), transcript variant 1, mRNA [NM_000368]	7248
A_23_P162739	2.3	1.6	2.5	1.0	TSC22D1	Homo sapiens TSC22 domain family, member 1 (TSC22D1), transcript variant 1, mRNA [NM_183422]	8848
A_24_P337062	-1.0	1.7	1.5	2.3	TSC22D2	Homo sapiens TSC22 domain family, member 2 (TSC22D2), mRNA [NM_014779]	9819

A_23_P92230	2.6	1.2	3.0	4.3	TSC22D2	Homo sapiens TSC22 domain family, member 2 (TSC22D2), mRNA [NM_014779]	9819
A_24_P200942	1.3	1.8	-2.7	1.2	TSC22D4	Homo sapiens TSC22 domain family, member 4 (TSC22D4), mRNA [NM_030935]	81628
A_23_P147277	-3.2	2.5	2.0	5.9	TSEN54	Homo sapiens tRNA splicing endonuclease 54 homolog (S. cerevisiae) (TSEN54), mRNA [NM_207346]	283989
A_23_P75839	2.1	-19.4	2.4	1.8	TSG101	Homo sapiens tumor susceptibility gene 101 (TSG101), mRNA [NM_006292]	7251

A_23_P17103	-1.0	-1.0	1.0	-5.2	TSGA10	Homo sapiens testis specific, 10 (TSGA10), transcript variant 1, mRNA [NM_025244]	80705
A_23_P306845	1.1	-1.7	-1.0	-2.4	TSGA10IP	Homo sapiens testis specific, 10 interacting protein (TSGA10IP), mRNA [NM_152762]	254187
A_23_P12128	1.4	1.9	2.3	1.2	TSHB	Homo sapiens thyroid stimulating hormone, beta (TSHB), mRNA [NM_000549]	7252
A_24_P42557	-1.7	-2.6	-1.7	-1.5	TSHR	Homo sapiens thyroid stimulating hormone receptor (TSHR), transcript variant 2, mRNA [NM_001018036]	7253

A_23_P27381	2.8	5.3	3.0	2.3	TSHZ1	Homo sapiens teashirt zinc finger homeobox 1 (TSHZ1), mRNA [NM_005786]	10194
A_23_P143247	3.0	1.3	1.8	1.1	TSHZ2	Homo sapiens teashirt zinc finger homeobox 2 (TSHZ2), transcript variant 1, mRNA [NM_173485]	128553
A_23_P16275	-2.8	-2.1	-2.4	-2.3	TSKS	Homo sapiens testis-specific serine kinase substrate (TSKS), mRNA [NM_021733]	60385
A_23_P28105	2.3	2.6	1.6	-1.1	TSN	Homo sapiens translin (TSN), mRNA [NM_004622]	7247

A_23_P319520	-1.6	-1.7	-10.5	-4.5	TSNARE1	Homo sapiens t-SNARE domain containing 1 (TSNARE1), mRNA [NM_145003]	203062
A_32_P161661	4.8	4.3	2.4	5.9	TSNARE1	Homo sapiens t-SNARE domain containing 1, mRNA (cDNA clone IMAGE:5768818), partial cds. [BC065226]	203062
A_24_P148151	-12.9	1.3	1.2	1.1	TSNAX	Homo sapiens translin-associated factor X (TSNAX), mRNA [NM_005999]	7257
A_23_P51572	-1.6	-2.9	-1.6	-1.0	TSNAX	Homo sapiens translin-associated factor X (TSNAX), mRNA [NM_005999]	7257

A_23_P9926	1.6	1.3	2.0	1.4	TSPAN10	Homo sapiens tetraspanin 10 (TSPAN10), mRNA [NM_031945]	83882
A_23_P76136	2.1	1.3	-1.2	1.4	TSPAN11	Homo sapiens tetraspanin 11 (TSPAN11), mRNA [NM_001080509]	441631
A_23_P168610	-2.4	-2.7	-3.4	1.4	TSPAN13	Homo sapiens tetraspanin 13 (TSPAN13), mRNA [NM_014399]	27075
A_24_P135483	-1.2	-2.2	1.2	-2.6	TSPAN14	Homo sapiens tetraspanin 14 (TSPAN14), transcript variant 1, mRNA [NM_030927]	81619
A_24_P135489	1.0	-12.9	1.8	1.2	TSPAN14	Homo sapiens tetraspanin 14 (TSPAN14), transcript variant 1, mRNA [NM_030927]	81619

A_32_P91902	2.6	2.2	-2.5	1.4	TSPAN14	Homo sapiens tetraspanin 14 (TSPAN14), transcript variant 1, mRNA [NM_030927]	81619
A_23_P425516	-1.8	-2.2	-1.0	-2.3	TSPAN17	Homo sapiens tetraspanin 17 (TSPAN17), transcript variant 1, mRNA [NM_012171]	26262
A_24_P13083	1.6	2.5	1.2	-1.7	TSPAN18	Homo sapiens tetraspanin 18 (TSPAN18), mRNA [NM_130783]	90139
A_23_P2322	2.4	2.4	2.3	2.4	TSPAN19	Homo sapiens tetraspanin 19 (TSPAN19), mRNA [NM_001100917]	144448
A_24_P62659	-1.0	-4.4	-1.1	-1.1	TSPAN2	Homo sapiens tetraspanin 2 (TSPAN2), mRNA [NM_005725]	10100

A_32_P123652	1.1	3.0	1.1	1.0	TSPAN33	Homo sapiens tetraspanin 33 (TSPAN33), mRNA [NM_178562]	340348
A_23_P60856	2.5	9.6	3.0	10.1	TSPAN4	Homo sapiens tetraspanin 4 (TSPAN4), transcript variant 1, mRNA [NM_001025237]	7106
A_24_P83262	-1.7	1.0	-1.0	-14.4	TSPAN6	Homo sapiens tetraspanin 6 (TSPAN6), mRNA [NM_003270]	7105
A_23_P394972	1.5	-1.6	-4.7	1.5	TSPEAR	Homo sapiens thrombospondin-type laminin G domain and EAR repeats (TSPEAR), mRNA [NM_144991]	54084

A_23_P408341	-1.8	-1.8	-2.1	-3.8	TSPY26P	Homo sapiens testis specific protein, Y-linked 26, pseudogene (TSPY26P), non-coding RNA [NR_002781]	128854
A_24_P179277	1.0	-1.0	2.1	-1.0	TSPYL1	Homo sapiens cDNA FLJ39563 fis, clone SKMUS2001164. [AK096882]	7259
A_23_P110882	-21.7	-2.7	1.8	1.3	TSPYL4	Homo sapiens TSPY-like 4 (TSPYL4), mRNA [NM_021648]	23270
A_23_P2026	1.3	-4.3	-1.8	-1.0	TSSC4	Homo sapiens tumor suppressing subtransferable candidate 4 (TSSC4), mRNA [NM_005706]	10078

A_23_P427136	2.5	1.2	1.9	1.7	TSSK1B	Homo sapiens testis-specific serine kinase 1B (TSSK1B), mRNA [NM_032028]	83942
A_24_P352637	1.2	-3.8	1.1	-1.0	TSSK4	Homo sapiens testis-specific serine kinase 4 (TSSK4), transcript variant 2, mRNA [NM_174944]	283629
A_23_P108221	2.9	2.8	1.7	4.0	TSSK6	Homo sapiens testis-specific serine kinase 6 (TSSK6), mRNA [NM_032037]	83983

A_23_P29248	4.6	4.7	6.0	2.8	TST	Homo sapiens thiosulfate sulfurtransferase (rhodanese) (TST), nuclear gene encoding mitochondrial protein, mRNA [NM_003312]	7263
A_23_P94301	2.2	2.3	2.5	3.2	TSTA3	Homo sapiens tissue specific transplantation antigen P35B (TSTA3), mRNA [NM_003313]	7264
A_24_P224342	3.0	2.4	-1.4	-1.2	TTBK2	Homo sapiens tau tubulin kinase 2 (TTBK2), mRNA [NM_173500]	146057

A_24_P73075	-1.1	-3.1	1.2	1.1	TTC12	Homo sapiens tetratricopeptide repeat domain 12 (TTC12), mRNA [NM_017868]	54970
A_23_P347131	-1.3	-1.5	1.0	-2.8	TTC15	tetratricopeptide repeat domain 15 [Source:HGNC Symbol;Acc:24284] [ENST00000304601]	51112
A_23_P256835	2.0	-10.5	1.3	-1.6	TTC15	Homo sapiens tetratricopeptide repeat domain 15 (TTC15), mRNA [NM_016030]	51112
A_23_P375419	2.7	1.8	2.2	2.0	TTC16	Homo sapiens tetratricopeptide repeat domain 16 (TTC16), mRNA [NM_144965]	158248

A_23_P414895	1.1	-3.4	1.6	-2.5	TTC17	Homo sapiens tetratricopeptide repeat domain 17 (TTC17), mRNA [NM_018259]	55761
A_23_P414899	1.6	-2.1	1.2	-2.9	TTC17	Homo sapiens tetratricopeptide repeat domain 17 (TTC17), mRNA [NM_018259]	55761
A_23_P64247	1.6	2.1	-2.0	1.9	TTC17	tetratricopeptide repeat domain 17 [Source:HGNC Symbol;Acc:25596] [ENST00000299240]	55761
A_24_P11436	2.5	1.3	2.0	1.3	TTC22	Homo sapiens tetratricopeptide repeat domain 22 (TTC22), transcript variant 2, mRNA [NM_017904]	55001

A_24_P673968	2.5	1.2	1.5	-1.3	TTC22	tetratricopeptide repeat domain 22 [Source:HGNC Symbol;Acc:26067] [ENST00000371276]	55001
A_23_P140562	-2.0	1.0	1.2	-1.0	TTC23	Homo sapiens tetratricopeptide repeat domain 23 (TTC23), transcript variant 1, mRNA [NM_001040655]	64927
A_23_P390726	-1.0	-1.2	-1.0	2.3	TTC23L	Homo sapiens tetratricopeptide repeat domain 23-like (TTC23L), mRNA [NM_144725]	153657

A_23_P257668	-1.0	-2.8	-1.9	-1.1	TTC26	Homo sapiens tetratricopeptide repeat domain 26 (TTC26), transcript variant 1, mRNA [NM_024926]	79989
A_23_P131227	3.0	2.9	1.1	-1.1	TTC27	Homo sapiens tetratricopeptide repeat domain 27 (TTC27), transcript variant 1, mRNA [NM_017735]	55622
A_24_P84984	2.6	4.7	3.6	3.5	TTC3	Homo sapiens tetratricopeptide repeat domain 3 (TTC3), transcript variant 1, mRNA [NM_003316]	7267

A_23_P39813	-2.2	-2.6	-1.2	-1.2	TTC31	Homo sapiens tetratricopeptide repeat domain 31 (TTC31), transcript variant 1, mRNA [NM_022492]	64427
A_23_P60002	-1.0	-2.7	-2.2	-1.0	TTC35	Homo sapiens tetratricopeptide repeat domain 35 (TTC35), mRNA [NM_014673]	9694
A_23_P160214	-2.7	-1.0	-1.1	-1.1	TTC39A	Homo sapiens tetratricopeptide repeat domain 39A (TTC39A), transcript variant 2, mRNA [NM_001080494]	22996

A_32_P155052	-1.5	2.7	2.3	2.6	TTC39C	Homo sapiens tetratricopeptide repeat domain 39C (TTC39C), transcript variant 1, mRNA [NM_001135993]	125488
A_32_P123629	1.2	2.3	1.1	1.0	TTC39C	Homo sapiens tetratricopeptide repeat domain 39C (TTC39C), transcript variant 1, mRNA [NM_001135993]	125488
A_23_P230	-2.2	-1.0	1.6	-1.9	TTC4	Homo sapiens tetratricopeptide repeat domain 4 (TTC4), mRNA [NM_004623]	7268
A_24_P940909	2.9	2.3	2.9	3.0	TTC7A	Homo sapiens tetratricopeptide repeat domain 7A (TTC7A), mRNA [NM_020458]	57217

A_24_P127292	-2.1	-1.0	1.1	-1.0	TTC7B	Homo sapiens tetratricopeptide repeat domain 7B (TTC7B), mRNA [NM_001010854]	145567
A_24_P273738	-4.6	1.0	1.1	-2.6	TTC9	Homo sapiens tetratricopeptide repeat domain 9 (TTC9), mRNA [NM_015351]	23508
A_23_P332392	-4.4	-1.3	1.4	-7.9	TTC9B	Homo sapiens cDNA FLJ30373 fis, clone BRACE2007882, weakly similar to Actin-depolymerizing protein N-WASP. [AK054935]	148014

A_32_P186741	-1.2	1.4	-2.2	-1.3	TTC9C	tetratricopeptide repeat domain 9C [Source:HGNC Symbol;Acc:28432] [ENST00000532276]	283237
A_23_P24751	1.3	1.3	-2.8	-1.5	TTC9C	Homo sapiens tetratricopeptide repeat domain 9C (TTC9C), mRNA [NM_173810]	283237
A_23_P254031	-2.2	1.0	1.0	1.1	TTF1	Homo sapiens transcription termination factor, RNA polymerase I (TTF1), transcript variant 1, mRNA [NM_007344]	7270

A_24_P316475	-2.0	1.3	1.2	-1.0	TTF2	transcription termination factor, RNA polymerase II [Source:HGNC Symbol;Acc:12398] [ENST00000464062]	8458
A_23_P97161	1.7	-1.7	1.1	-2.4	TTF2	Homo sapiens transcription termination factor, RNA polymerase II (TTF2), mRNA [NM_003594]	8458
A_24_P162273	-12.9	-1.0	-1.1	-1.0	TTL	Homo sapiens tubulin tyrosine ligase (TTL), transcript variant TTL-B2, non-coding RNA [NR_024507]	646982

A_23_P143748	1.0	-1.0	-1.1	-30.4	TTLL12	Homo sapiens tubulin tyrosine ligase-like family, member 12 (TTLL12), mRNA [NM_015140]	23170
A_24_P917492	1.1	2.4	1.5	1.6	TTLL3	tubulin tyrosine ligase-like family, member 3 [Source:HGNC Symbol;Acc:24483] [ENST00000492440]	26140
A_24_P361408	1.0	-1.0	-8.9	-21.9	TTLL6	Homo sapiens tubulin tyrosine ligase-like family, member 6 (TTLL6), transcript variant 2, mRNA [NM_173623]	284076

A_32_P204169	1.0	3.0	1.7	-1.6	TTLL7	tubulin tyrosine ligase-like family, member 7 [Source:HGNC Symbol;Acc:26242] [ENST00000477524]	79739
A_24_P165450	-27.2	1.1	1.1	1.1	TTLL7	Homo sapiens tubulin tyrosine ligase-like family, member 7 (TTLL7), mRNA [NM_024686]	79739
A_23_P85269	1.0	1.3	-2.4	1.2	TTN	Homo sapiens titin (TTN), transcript variant N2-A, mRNA [NM_133378]	7273
A_23_P253561	2.0	1.0	-1.0	1.1	TTPAL	Homo sapiens tocopherol (alpha) transfer protein-like (TTPAL), transcript variant 1, mRNA [NM_024331]	79183

A_23_P11397	1.0	-1.0	-1.1	-4.1	TTTY13	Homo sapiens testis-specific transcript, Y-linked 13 (non-protein coding) (TTTY13), non-coding RNA [NR_001537]	83868
A_23_P251232	1.1	-1.9	-2.9	1.0	TTTY14	Homo sapiens testis-specific transcript, Y-linked 14 (non-protein coding) (TTTY14), non-coding RNA [NR_001543]	83869
A_32_P413094	-2.3	-1.1	-1.3	-1.4	TTTY2	Homo sapiens testis-specific transcript, Y-linked 2 (non-protein coding) (TTTY2), non-coding RNA [NR_001536]	60439

A_24_P298723	-2.9	-1.0	-1.1	-1.1	TTTY20	Homo sapiens testis-specific transcript, Y-linked 20 (non-protein coding) (TTTY20), non-coding RNA [NR_001546]	252951
A_24_P280328	2.2	5.8	-1.9	-2.6	TTTY3	Homo sapiens testis-specific transcript, Y-linked 3 (non-protein coding) (TTTY3), non-coding RNA [NR_001524]	114760
A_23_P251868	1.0	1.0	-2.0	-1.1	TTTY9A	Homo sapiens testis-specific transcript, Y-linked 9A (non-protein coding) (TTTY9A), non-coding RNA [NR_001530]	83864

A_23_P406242	1.0	-1.0	-1.1	-2.4	TTTY9A	Homo sapiens testis-specific transcript, Y-linked 9A (non-protein coding) (TTTY9A), non-coding RNA [NR_001530]	83864
A_23_P50815	-1.9	1.1	-1.1	-3.4	TTYH1	Homo sapiens tweety homolog 1 (Drosophila) (TTYH1), transcript variant 1, mRNA [NM_020659]	57348
A_24_P99526	-1.1	-2.0	-1.4	-1.2	TTYH2	Homo sapiens tweety homolog 2 (Drosophila) (TTYH2), transcript variant 1, mRNA [NM_032646]	94015

A_23_P66432	1.1	-4.3	-1.0	-1.0	TTYH2	Homo sapiens tweety homolog 2 (Drosophila) (TTYH2), transcript variant 1, mRNA [NM_032646]	94015
A_23_P331895	-1.4	1.8	1.2	-2.5	TTYH3	Homo sapiens tweety homolog 3 (Drosophila) (TTYH3), mRNA [NM_025250]	80727
A_23_P53110	-1.5	-2.3	-1.6	-3.1	TUB	Homo sapiens tubby homolog (mouse) (TUB), transcript variant 1, mRNA [NM_003320]	7275
A_23_P128598	-1.9	-1.3	-2.3	1.8	TUBA3C	Homo sapiens tubulin, alpha 3c (TUBA3C), mRNA [NM_006001]	7278

A_23_P56736	-1.1	2.2	-1.2	-1.2	TUBA3D	Homo sapiens tubulin, alpha 3d (TUBA3D), mRNA [NM_080386]	113457
A_24_P228550	1.9	-1.3	-2.2	-1.8	TUBB1	Homo sapiens tubulin, beta 1 class VI (TUBB1), mRNA [NM_030773]	81027
A_23_P113656	2.6	1.2	2.8	-1.4	TUBB4A	Homo sapiens tubulin, beta 4A class IVa (TUBB4A), mRNA [NM_006087]	10382
A_23_P312863	-1.4	2.1	1.6	-1.0	TUBB4B	Homo sapiens tubulin, beta 4B class IVb (TUBB4B), mRNA [NM_006088]	10383
A_32_P187327	1.2	-2.8	-1.3	1.0	TUBB4B	Homo sapiens tubulin, beta 4B class IVb (TUBB4B), mRNA [NM_006088]	10383

A_32_P16315	2.8	2.7	-1.0	2.7	TUBB8	Homo sapiens tubulin, beta 8 class VIII (TUBB8), transcript variant 1, mRNA [NM_177987]	347688
A_23_P26895	-1.7	-2.1	-1.1	-1.0	TUBD1	Homo sapiens tubulin, delta 1 (TUBD1), transcript variant 1, mRNA [NM_016261]	51174
A_23_P145053	1.0	-1.9	1.0	-2.4	TUBE1	Homo sapiens tubulin, epsilon 1 (TUBE1), mRNA [NM_016262]	51175
A_32_P105712	3.5	3.0	2.0	2.4	TUBGCP3	Homo sapiens tubulin, gamma complex associated protein 3 (TUBGCP3), mRNA [NM_006322]	10426

A_24_P313096	-1.0	1.4	-1.0	-33.2	TUBGCP3	Homo sapiens tubulin, gamma complex associated protein 3 (TUBGCP3), mRNA [NM_006322]	10426
A_23_P152038	1.1	1.0	-1.1	2.1	TUBGCP5	Homo sapiens tubulin, gamma complex associated protein 5 (TUBGCP5), transcript variant 1, mRNA [NM_052903]	114791
A_23_P6546	-1.2	-6.6	-3.5	-5.2	TUBGCP6	Homo sapiens tubulin, gamma complex associated protein 6 (TUBGCP6), mRNA [NM_020461]	85378

A_24_P382724	-3.0	-3.3	-2.4	1.5	TUG1	Homo sapiens taurine upregulated 1 (non-protein coding) (TUG1), non-coding RNA [NR_002323]	55000
A_23_P382045	-2.1	1.1	1.1	1.1	TULP4	Homo sapiens tubby like protein 4 (TULP4), transcript variant 1, mRNA [NM_020245]	56995
A_32_P133884	1.4	1.4	1.3	-3.2	TUSC1	Homo sapiens tumor suppressor candidate 1 (TUSC1), mRNA [NM_001004125]	286319
A_23_P251562	2.5	1.8	1.7	1.1	TUSC2	Homo sapiens tumor suppressor candidate 2 (TUSC2), mRNA [NM_007275]	11334

A_24_P915908	1.1	1.0	2.1	1.0	TUSC3	Homo sapiens cDNA: FLJ22496 fis, clone HRC11236. [AK026149]	7991
A_23_P308058	-6.2	-1.6	-1.7	-1.7	TUSC5	Homo sapiens tumor suppressor candidate 5 (TUSC5), mRNA [NM_172367]	286753
A_24_P178273	7.0	6.8	6.5	3.7	TWF1	Homo sapiens twinfilin, actin-binding protein, homolog 1 (Drosophila) (TWF1), transcript variant 2, mRNA [NM_002822]	5756
A_23_P71073	12.3	11.8	10.5	7.9	TWIST1	Homo sapiens twist homolog 1 (Drosophila) (TWIST1), mRNA [NM_000474]	7291

A_23_P71067	1.1	1.2	2.6	1.2	TWIST1	Homo sapiens twist homolog 1 (Drosophila) (TWIST1), mRNA [NM_000474]	7291
A_23_P82474	1.0	-4.0	-1.0	-1.1	TWISTNB	Homo sapiens TWIST neighbor (TWISTNB), mRNA [NM_001002926]	221830
A_23_P386561	-1.0	-1.0	-1.1	-4.5	TWISTNB	Homo sapiens TWIST neighbor (TWISTNB), mRNA [NM_001002926]	221830
A_24_P202497	-1.1	-1.1	-1.1	-3.9	TWSG1	Homo sapiens twisted gastrulation homolog 1 (Drosophila) (TWSG1), mRNA [NM_020648]	57045

A_24_P134626	2.1	2.2	-1.9	1.2	TXLNG	Homo sapiens taxilin gamma (TXLNG), transcript variant 1, mRNA [NM_018360]	55787
A_23_P96658	2.8	2.7	2.6	2.2	TXLNG2P	Homo sapiens taxilin gamma 2, pseudogene (TXLNG2P), transcript variant 1, non-coding RNA [NR_045128]	246126
A_23_P60248	2.6	2.5	2.6	1.0	TXN	Homo sapiens thioredoxin (TXN), transcript variant 1, mRNA [NM_003329]	7295
A_23_P14939	1.5	-1.1	1.1	3.4	TXNDC11	Homo sapiens thioredoxin domain containing 11 (TXNDC11), mRNA [NM_015914]	51061

A_23_P200199	2.4	2.2	2.5	1.3	TXNDC12	Homo sapiens thioredoxin domain containing 12 (endoplasmic reticulum) (TXNDC12), mRNA [NM_015913]	51060
A_23_P398836	-2.3	-1.1	-1.0	1.0	TXNDC16	Homo sapiens thioredoxin domain containing 16 (TXNDC16), transcript variant 1, mRNA [NM_020784]	57544
A_23_P380848	-1.0	-1.0	-1.1	6.5	TXNDC17	Homo sapiens thioredoxin domain containing 17 (TXNDC17), mRNA [NM_032731]	84817

A_24_P125434	-3.3	-6.6	-6.4	-3.5	TXNDC2	Homo sapiens thioredoxin domain containing 2 (spermatozoa) (TXNDC2), transcript variant 1, mRNA [NM_032243]	84203
A_24_P362646	-1.1	1.0	-9.9	-3.0	TXNDC9	Homo sapiens thioredoxin domain containing 9 (TXNDC9), mRNA [NM_005783]	10190
A_23_P4522	-1.0	-1.0	-1.0	-2.1	TXNL1	Homo sapiens thioredoxin-like 1 (TXNL1), transcript variant 1, mRNA [NM_004786]	9352
A_24_P399083	2.3	2.2	2.1	2.2	TXNL4B	Homo sapiens thioredoxin-like 4B (TXNL4B), transcript variant 1, mRNA [NM_017853]	54957

A_23_P91802	1.1	-1.4	2.1	-1.4	TYMP	Homo sapiens thymidine phosphorylase (TYMP), transcript variant 2, mRNA [NM_001953]	1890
A_23_P35755	-1.2	-3.3	4.2	-49.5	TYR	Homo sapiens tyrosinase (oculocutaneous albinism IA) (TYR), mRNA [NM_000372]	7299
A_23_P54517	3.1	5.4	-1.0	2.8	TYRO3	Homo sapiens TYRO3 protein tyrosine kinase (TYRO3), mRNA [NM_006293]	7301
A_23_P27994	2.6	-1.0	1.0	1.0	TYROBP	Homo sapiens TYRO protein tyrosine kinase binding protein (TYROBP), transcript variant 1, mRNA [NM_003332]	7305

A_23_P423480	2.0	1.9	-1.8	1.2	TYSND1	Homo sapiens trypsin domain containing 1 (TYSND1), transcript variant 1, mRNA [NM_173555]	219743
A_23_P103180	1.1	6.6	-1.1	-1.0	TYW3	Homo sapiens tRNA-yW synthesizing protein 3 homolog (S. cerevisiae) (TYW3), transcript variant 1, mRNA [NM_138467]	127253
A_23_P103172	2.8	2.7	3.0	1.3	TYW3	Homo sapiens tRNA-yW synthesizing protein 3 homolog (S. cerevisiae) (TYW3), transcript variant 1, mRNA [NM_138467]	127253

A_24_P20139	2.2	2.0	1.7	1.7	TYW5	Homo sapiens tRNA-yW synthesizing protein 5 (TYW5), transcript variant 1, mRNA [NM_001039693]	129450
A_23_P431418	2.2	-1.8	1.4	-1.4	U2AF2	Homo sapiens U2 small nuclear RNA auxiliary factor 2 (U2AF2), transcript variant 1, mRNA [NM_007279]	11338
A_24_P943106	3.3	2.9	1.5	3.7	U2SURP	Homo sapiens U2 snRNP-associated SURP domain containing (U2SURP), mRNA [NM_001080415]	23350
A_24_P323932	-1.2	-2.8	-1.0	-1.0	U2SURP	Homo sapiens U2 snRNP-associated SURP domain containing (U2SURP), mRNA [NM_001080415]	23350

A_24_P90774	1.1	-1.0	-1.1	-2.4	UACA	Homo sapiens uveal autoantigen with coiled-coil domains and ankyrin repeats (UACA), transcript variant 2, mRNA [NM_001008224]	55075
A_23_P135164	-3.3	-12.6	-1.9	-3.6	UAP1L1	Homo sapiens UDP-N-acteylglucosamine pyrophosphorylase 1-like 1 (UAP1L1), mRNA [NM_207309]	91373
A_24_P237374	4.6	5.1	4.8	2.8	UBA1	Homo sapiens ubiquitin-like modifier activating enzyme 1 (UBA1), transcript variant 1, mRNA [NM_003334]	7317

A_24_P376229	1.7	2.6	1.4	-1.0	UBA2	Homo sapiens ubiquitin-like modifier activating enzyme 2 (UBA2), mRNA [NM_005499]	10054
A_23_P56195	1.6	-1.8	-2.4	1.6	UBA52	Homo sapiens ubiquitin A-52 residue ribosomal protein fusion product 1 (UBA52), transcript variant 1, mRNA [NM_001033930]	7311
A_23_P218905	-2.4	-1.5	-1.6	-1.7	UBA6	Homo sapiens ubiquitin-like modifier activating enzyme 6 (UBA6), mRNA [NM_018227]	55236

A_23_P139998	-1.0	-1.2	-1.1	2.7	UBAC2	Homo sapiens UBA domain containing 2 (UBAC2), transcript variant 2, mRNA [NM_177967]	337867
A_23_P123866	1.0	2.2	1.4	-1.7	UBAP1	Homo sapiens ubiquitin associated protein 1 (UBAP1), transcript variant 1, mRNA [NM_016525]	51271
A_24_P682199	-2.0	-1.7	-3.0	-3.9	UBAP1L	Homo sapiens mRNA; cDNA DKFZp686J168 (from clone DKFZp686J168). [CR749500]	390595
A_24_P481783	1.1	-1.6	-2.2	1.0	UBAP1L	Homo sapiens ubiquitin associated protein 1-like (UBAP1L), mRNA [NM_001163692]	390595

A_24_P933838	1.1	1.0	-1.0	-4.2	UBAP2L	ubiquitin associated protein 2-like [Source:HGNC Symbol;Acc:29877] [ENST00000493867]	9898
A_24_P290927	-1.3	-1.4	-2.3	-1.3	UBAP2L	Homo sapiens ubiquitin associated protein 2-like (UBAP2L), transcript variant 1, mRNA [NM_014847]	9898
A_23_P81898	1.1	1.1	-1.2	-5.7	UBD	Homo sapiens ubiquitin D (UBD), mRNA [NM_006398]	10537
A_24_P200583	-1.7	-2.5	-1.7	-1.2	UBE2B	Homo sapiens ubiquitin-conjugating enzyme E2B (UBE2B), mRNA [NM_003337]	7320

A_23_P167595	-1.0	-3.5	-2.5	-1.0	UBE2B	Homo sapiens ubiquitin-conjugating enzyme E2B (UBE2B), mRNA [NM_003337]	7320
A_24_P626931	-1.5	2.9	-1.1	-1.6	UBE2CBP	Homo sapiens ubiquitin-conjugating enzyme E2C binding protein (UBE2CBP), mRNA [NM_198920]	90025
A_23_P365327	-8.7	-3.2	1.1	-3.5	UBE2CBP	ubiquitin-conjugating enzyme E2C binding protein [Source:HGNC Symbol;Acc:21381] [ENST00000369746]	90025

A_24_P13032	7.1	1.5	3.3	2.0	UBE2D1	Homo sapiens ubiquitin-conjugating enzyme E2D 1 (UBE2D1), transcript variant 1, mRNA [NM_003338]	7321
A_24_P54847	2.5	2.0	2.1	2.6	UBE2D3	ubiquitin-conjugating enzyme E2D 3 [Source:HGNC Symbol;Acc:12476] [ENST00000453744]	7323
A_24_P926684	2.4	1.2	-1.2	1.9	UBE2D3	ubiquitin-conjugating enzyme E2D 3 [Source:HGNC Symbol;Acc:12476] [ENST00000503418]	7323

A_23_P374767	2.1	2.1	2.0	-1.4	UBE2D4	Homo sapiens ubiquitin-conjugating enzyme E2D 4 (putative) (UBE2D4), mRNA [NM_015983]	51619
A_23_P367071	-1.4	-2.0	-1.4	1.2	UBE2DNL	Homo sapiens ubiquitin-conjugating enzyme E2D N-terminal like (pseudogene) (UBE2DNL), non-coding RNA [NR_024062]	100131816
A_24_P148450	2.9	1.5	2.4	2.6	UBE2E3	Homo sapiens ubiquitin-conjugating enzyme E2E 3 (UBE2E3), transcript variant 1, mRNA [NM_006357]	10477

A_23_P27066	1.1	-2.4	1.2	1.3	UBE2G1	Homo sapiens ubiquitin-conjugating enzyme E2G 1 (UBE2G1), mRNA [NM_003342]	7326
A_23_P211179	-1.0	-1.0	1.0	-4.1	UBE2G2	Homo sapiens ubiquitin-conjugating enzyme E2G 2 (UBE2G2), transcript variant 2, mRNA [NM_182688]	7327
A_23_P145584	-3.1	1.3	-1.0	-1.5	UBE2H	Homo sapiens ubiquitin-conjugating enzyme E2H (UBE2H), transcript variant 1, mRNA [NM_003344]	7328

A_23_P152107	-1.5	-1.6	-3.8	-2.5	UBE2I	Homo sapiens ubiquitin-conjugating enzyme E2I (UBE2I), transcript variant 2, mRNA [NM_194259]	7329
A_24_P227993	1.9	-6.3	1.0	1.0	UBE2I	Homo sapiens ubiquitin-conjugating enzyme E2I (UBE2I), transcript variant 2, mRNA [NM_194259]	7329
A_24_P277456	1.1	-13.9	1.0	1.1	UBE2K	Homo sapiens ubiquitin-conjugating enzyme E2K (UBE2K), transcript variant 1, mRNA [NM_005339]	3093

A_23_P75741	-1.0	-2.5	-5.8	-2.5	UBE2L6	Homo sapiens ubiquitin-conjugating enzyme E2L 6 (UBE2L6), transcript variant 2, mRNA [NM_198183]	9246
A_23_P116829	-12.8	1.1	-1.0	-1.2	UBE2N	Homo sapiens ubiquitin-conjugating enzyme E2N (UBE2N), mRNA [NM_003348]	7334
A_32_P11499	6.0	2.9	2.3	2.7	UBE2N	Homo sapiens ubiquitin-conjugating enzyme E2N (UBE2N), mRNA [NM_003348]	7334
A_23_P97423	2.2	1.6	1.6	1.3	UBE2Q1	Homo sapiens ubiquitin-conjugating enzyme E2Q family member 1 (UBE2Q1), mRNA [NM_017582]	55585

A_24_P412617	-1.1	1.1	-2.1	-2.0	UBE2Q2	Homo sapiens ubiquitin-conjugating enzyme E2Q family member 2 (UBE2Q2), transcript variant 1, mRNA [NM_173469]	92912
A_24_P816384	1.6	1.4	5.3	3.1	UBE2Q2P1	Homo sapiens ubiquitin-conjugating enzyme E2Q family member 2 pseudogene 1 (UBE2Q2P1), non-coding RNA [NR_003661]	388165
A_32_P85131	1.0	-1.0	-1.1	-3.5	UBE2QL1	Homo sapiens ubiquitin-conjugating enzyme E2Q family-like 1 (UBE2QL1), mRNA [NM_001145161]	134111

A_32_P169003	-1.0	-1.0	-1.0	-3.1	UBE2R2	Homo sapiens cDNA clone IMAGE:4825207, **** WARNING: chimeric clone ****. [BC038356]	54926
A_32_P171328	3.0	3.8	1.7	3.4	UBE2S	Homo sapiens ubiquitin-conjugating enzyme E2S (UBE2S), mRNA [NM_014501]	27338
A_23_P379657	2.2	2.1	-1.7	1.9	UBE2U	Homo sapiens ubiquitin-conjugating enzyme E2U (putative) (UBE2U), mRNA [NM_152489]	148581

A_23_P218685	-2.0	-5.8	1.4	1.3	UBE2V1	Homo sapiens ubiquitin-conjugating enzyme E2 variant 1 (UBE2V1), transcript variant 4, mRNA [NM_001032288]	7335
A_23_P11461	1.8	-1.0	-2.7	1.1	UBE2V1	Homo sapiens ubiquitin-conjugating enzyme E2 variant 1 (UBE2V1), transcript variant 4, mRNA [NM_001032288]	7335
A_24_P406514	-6.3	1.0	1.0	1.1	UBE2V2	Homo sapiens ubiquitin-conjugating enzyme E2 variant 2 (UBE2V2), mRNA [NM_003350]	7336

A_23_P71430	-2.6	-2.7	1.1	-1.1	UBE2W	Homo sapiens ubiquitin-conjugating enzyme E2W (putative) (UBE2W), transcript variant 1, mRNA [NM_001001481]	55284
A_24_P346604	-1.5	-2.2	-1.7	-1.3	UBE2W	Homo sapiens ubiquitin-conjugating enzyme E2W (putative) (UBE2W), transcript variant 1, mRNA [NM_001001481]	55284
A_24_P378506	1.2	1.4	2.0	-1.6	UBE2Z	Homo sapiens ubiquitin-conjugating enzyme E2Z (UBE2Z), mRNA [NM_023079]	65264

A_24_P381803	-3.3	1.0	-1.1	1.0	UBE3A	Homo sapiens ubiquitin protein ligase E3A (UBE3A), transcript variant 3, mRNA [NM_130839]	7337
A_23_P203137	-2.8	1.1	1.1	1.1	UBE4A	Homo sapiens ubiquitination factor E4A (UBE4A), transcript variant 1, mRNA [NM_004788]	9354
A_24_P330668	-3.7	-2.0	-2.0	-2.0	UBFD1	Homo sapiens ubiquitin family domain containing 1 (UBFD1), mRNA [NM_019116]	56061
A_23_P62361	3.4	3.1	3.0	2.1	UBL4A	Homo sapiens ubiquitin-like 4A (UBL4A), mRNA [NM_014235]	8266

A_32_P112910	-1.5	-1.5	-2.3	1.2	UBL4B	Homo sapiens ubiquitin-like 4B (UBL4B), mRNA [NM_203412]	164153
A_23_P78563	1.7	1.5	2.8	1.1	UBL5	Homo sapiens ubiquitin-like 5 (UBL5), transcript variant 1, mRNA [NM_024292]	59286
A_32_P47566	-1.0	-5.0	-1.0	-1.0	UBLCP1	Homo sapiens ubiquitin-like domain containing CTD phosphatase 1 (UBLCP1), mRNA [NM_145049]	134510
A_23_P93994	28.1	17.2	31.0	20.4	UBN2	Homo sapiens ubinuclein 2 (UBN2), mRNA [NM_173569]	254048

A_24_P23067	2.4	1.3	-1.7	1.9	UBOX5	Homo sapiens U-box domain containing 5 (UBOX5), transcript variant 1, mRNA [NM_014948]	22888
A_24_P338121	1.0	-2.1	-1.1	-1.3	UBQLN1	Homo sapiens ubiquilin 1 (UBQLN1), transcript variant 2, mRNA [NM_053067]	29979
A_23_P250910	1.0	-2.8	-1.1	-1.0	UBQLN1	Homo sapiens ubiquilin 1 (UBQLN1), transcript variant 2, mRNA [NM_053067]	29979
A_23_P250904	1.1	-7.9	-1.1	1.0	UBQLN1	Homo sapiens ubiquilin 1 (UBQLN1), transcript variant 1, mRNA [NM_013438]	29979

A_23_P114164	1.4	5.3	6.4	2.5	UBQLN2	Homo sapiens ubiquilin 2 (UBQLN2), mRNA [NM_013444]	29978
A_23_P300770	-1.0	-4.6	1.5	-1.0	UBR1	Homo sapiens ubiquitin protein ligase E3 component n-recognin 1 (UBR1), mRNA [NM_174916]	197131
A_24_P102203	-1.6	-1.0	-3.7	-1.0	UBR1	Homo sapiens ubiquitin protein ligase E3 component n-recognin 1 (UBR1), mRNA [NM_174916]	197131
A_23_P5405	-1.0	1.1	1.5	-2.1	UBR3	Homo sapiens ubiquitin protein ligase E3 component n-recognin 3 (putative) (UBR3), mRNA [NM_172070]	130507

A_24_P285501	1.4	1.3	-3.0	1.0	UBR3	Homo sapiens ubiquitin protein ligase E3 component n-recognin 3 (putative) (UBR3), mRNA [NM_172070]	130507
A_23_P161501	2.2	1.9	-1.9	-1.1	UBTD1	Homo sapiens ubiquitin domain containing 1 (UBTD1), mRNA [NM_024954]	80019
A_23_P100779	1.1	-3.1	1.2	1.7	UBTF	Homo sapiens upstream binding transcription factor, RNA polymerase I (UBTF), transcript variant 2, mRNA [NM_001076683]	7343
A_23_P85664	2.3	1.9	1.6	-1.0	UBXN10	Homo sapiens UBX domain protein 10 (UBXN10), mRNA [NM_152376]	127733

A_24_P239811	-2.0	-2.1	-1.1	-2.0	UBXN11	Homo sapiens UBX domain protein 11 (UBXN11), transcript variant 2, mRNA [NM_183008]	91544
A_23_P16451	1.0	-8.6	-1.1	3.4	UBXN6	Homo sapiens UBX domain protein 6 (UBXN6), transcript variant 1, mRNA [NM_025241]	80700
A_32_P222515	1.0	-6.1	-4.3	-1.0	UBXN7	UBX domain protein 7 [Source:HGNC Symbol;Acc:29119] [ENST00000296328]	26043
A_23_P132956	-1.0	1.0	1.0	-3.9	UCHL1	Homo sapiens ubiquitin carboxyl-terminal esterase L1 (ubiquitin thiolesterase) (UCHL1), mRNA [NM_004181]	7345

A_24_P188325	1.4	1.7	-2.6	1.0	UCHL5	Homo sapiens ubiquitin carboxyl-terminal hydrolase L5 (UCHL5), transcript variant 1, mRNA [NM_015984]	51377
A_24_P18270	2.7	1.7	3.3	1.0	UCMA	Homo sapiens upper zone of growth plate and cartilage matrix associated (UCMA), mRNA [NM_145314]	221044
A_23_P155453	1.2	-3.1	-2.0	-1.6	UCN2	Homo sapiens urocortin 2 (UCN2), mRNA [NM_033199]	90226
A_23_P136184	1.2	-1.1	1.0	-10.6	UCN3	Homo sapiens urocortin 3 (stresscopin) (UCN3), mRNA [NM_053049]	114131

A_23_P203601	-2.4	-2.3	-2.1	-2.4	UCP3	Homo sapiens uncoupling protein 3 (mitochondrial, proton carrier) (UCP3), nuclear gene encoding mitochondrial protein, transcript variant long, mRNA [NM_003356]	7352
A_24_P292470	-1.9	2.0	-1.2	1.0	UCP3	Homo sapiens uncoupling protein 3 (mitochondrial, proton carrier) (UCP3), nuclear gene encoding mitochondrial protein, transcript variant short, mRNA [NM_022803]	7352

A_23_P166333	3.3	4.6	-2.2	4.0	UFD1L	Homo sapiens ubiquitin fusion degradation 1 like (yeast) (UFD1L), transcript variant 1, mRNA [NM_005659]	7353
A_23_P30956	-4.2	-4.4	-4.7	-4.4	UFL1	Homo sapiens UFM1-specific ligase 1 (UFL1), mRNA [NM_015323]	23376
A_24_P313109	1.1	1.0	-1.1	-5.3	UFM1	Homo sapiens ubiquitin-fold modifier 1 (UFM1), mRNA [NM_016617]	51569
A_23_P123645	-4.3	-1.5	-1.7	-1.6	UGCG	UDP-glucose ceramide glucosyltransferase [Source:HGNC Symbol;Acc:12524] [ENST00000374279]	7357

A_24_P44341	-3.2	1.0	-1.1	-1.0	UGGT2	Homo sapiens UDP-glucose glycoprotein glucosyltransferase 2 (UGGT2), mRNA [NM_020121]	55757
A_23_P204980	1.0	1.5	2.2	1.9	UGGT2	Homo sapiens UDP-glucose glycoprotein glucosyltransferase 2 (UGGT2), mRNA [NM_020121]	55757
A_24_P341909	1.7	-1.0	1.5	-3.5	UGGT2	Homo sapiens UDP-glucose glycoprotein glucosyltransferase 2 (UGGT2), mRNA [NM_020121]	55757

A_23_P253046	1.0	-3.1	-1.1	-1.1	UGP2	Homo sapiens UDP-glucose pyrophosphorylase 2 (UGP2), transcript variant 1, mRNA [NM_006759]	7360
A_23_P60599	1.2	-1.7	-2.4	-1.1	UGT1A6	Homo sapiens UDP glucuronosyltransferase 1 family, polypeptide A6 (UGT1A6), transcript variant 1, mRNA [NM_001072]	54578
A_23_P158330	2.1	1.4	2.1	2.2	UGT1A8	Homo sapiens UDP glucuronosyltransferase 1 family, polypeptide A8 (UGT1A8), mRNA [NM_019076]	54576

A_24_P334378	-4.9	-1.2	-1.7	-1.3	UGT2A3	Homo sapiens UDP glucuronosyltransferase 2 family, polypeptide A3 (UGT2A3), mRNA [NM_024743]	79799
A_24_P521559	1.9	1.9	2.8	1.0	UGT2B10	Homo sapiens UDP glucuronosyltransferase 2 family, polypeptide B10 (UGT2B10), transcript variant 1, mRNA [NM_001075]	7365
A_23_P212968	-1.0	-9.7	-1.0	-1.0	UGT2B11	Homo sapiens UDP glucuronosyltransferase 2 family, polypeptide B11 (UGT2B11), mRNA [NM_001073]	10720

A_23_P501624	-3.1	-2.3	-1.1	-1.0	UGT2B17	Homo sapiens UDP glucuronosyltransferase 2 family, polypeptide B17 (UGT2B17), mRNA [NM_001077]	7367
A_24_P17691	-1.3	-3.0	-1.4	-1.5	UGT2B17	Homo sapiens UDP glucuronosyltransferase 2 family, polypeptide B17 (UGT2B17), mRNA [NM_001077]	7367
A_24_P180243	-1.7	1.7	-2.4	-1.8	UGT2B28	Homo sapiens UDP glucuronosyltransferase 2 family, polypeptide B28 (UGT2B28), transcript variant 1, mRNA [NM_053039]	54490

A_23_P386912	-5.1	-1.0	-1.1	-1.0	UGT2B4	Homo sapiens UDP glucuronosyltransferase 2 family, polypeptide B4 (UGT2B4), mRNA [NM_021139]	7363
A_23_P72747	12.0	11.9	12.0	11.7	UGT8	Human ceramide UDPgalactosyltransferase mRNA, complete cds. [U62899]	7368
A_24_P291133	1.3	1.8	2.5	3.3	UHMK1	Homo sapiens U2AF homology motif (UHM) kinase 1 (UHMK1), transcript variant 1, mRNA [NM_175866]	127933
A_32_P963	2.1	2.1	2.2	2.2	UHMK1	Homo sapiens U2AF homology motif (UHM) kinase 1 (UHMK1), transcript variant 1, mRNA [NM_175866]	127933

A_23_P317691	3.6	3.0	2.0	2.9	UIMC1	Homo sapiens ubiquitin interaction motif containing 1 (UIMC1), transcript variant 2, mRNA [NM_016290]	51720
A_24_P73370	4.5	3.4	3.5	3.1	ULK1	Homo sapiens unc-51-like kinase 1 (C. elegans) (ULK1), mRNA [NM_003565]	8408
A_23_P206103	2.9	6.2	2.6	2.6	ULK3	Homo sapiens unc-51-like kinase 3 (C. elegans) (ULK3), mRNA [NM_001099436]	25989
A_23_P206107	1.3	1.5	1.6	-2.3	ULK3	Homo sapiens unc-51-like kinase 3 (C. elegans) (ULK3), mRNA [NM_001099436]	25989

A_23_P143926	-6.0	1.0	-1.4	1.1	ULK4	Homo sapiens unc-51-like kinase 4 (C. elegans) (ULK4), mRNA [NM_017886]	54986
A_23_P158775	1.6	1.9	1.2	-2.2	UMOD	Homo sapiens uromodulin (UMOD), transcript variant 1, mRNA [NM_003361]	7369
A_23_P88576	-1.2	-1.0	-4.2	-1.3	UNC13C	Homo sapiens unc-13 homolog C (C. elegans) (UNC13C), mRNA [NM_001080534]	440279
A_32_P221624	-1.8	-2.2	-1.9	-1.1	UNC45A	unc-45 homolog A (C. elegans) [Source:HGNC Symbol;Acc:30594] [ENST00000556319]	55898

A_24_P398950	1.9	2.5	2.2	1.3	UNC45A	Homo sapiens unc-45 homolog A (C. elegans) (UNC45A), transcript variant 3, mRNA [NM_001039675]	55898
A_23_P49677	-2.3	-2.4	-1.8	-2.4	UNC45B	Homo sapiens unc-45 homolog B (C. elegans) (UNC45B), transcript variant 1, mRNA [NM_173167]	146862
A_23_P110557	1.2	-16.6	-1.9	-1.3	UNC5A	Homo sapiens unc-5 homolog A (C. elegans) (UNC5A), mRNA [NM_133369]	90249
A_23_P69617	-2.7	1.0	-2.6	-2.3	UNC5C	Homo sapiens unc-5 homolog C (C. elegans) (UNC5C), mRNA [NM_003728]	8633

A_24_P42624	-1.1	-1.4	-2.6	-1.3	UNC5C	Homo sapiens unc-5 homolog C (C. elegans) (UNC5C), mRNA [NM_003728]	8633
A_23_P350808	3.0	1.8	1.8	1.7	UNC80	unc-80 homolog (C. elegans) [Source:HGNC Symbol;Acc:26582] [ENST00000489023]	285175
A_24_P817863	15.2	17.1	8.1	8.5	UNC80	Homo sapiens unc-80 homolog (C. elegans) (UNC80), transcript variant 1, mRNA [NM_032504]	285175
A_23_P214408	1.1	-1.0	-1.0	-3.1	UNC93A	Homo sapiens unc-93 homolog A (C. elegans) (UNC93A), transcript variant 1, mRNA [NM_018974]	54346

A_23_P106559	1.8	2.1	2.6	1.1	UNKL	Homo sapiens unkempt homolog (Drosophila)-like (UNKL), transcript variant 1, mRNA [NM_001193388]	64718
A_24_P374675	2.9	-1.2	2.5	1.7	UNKL	Homo sapiens unkempt homolog (Drosophila)-like (UNKL), transcript variant 2, mRNA [NM_001037125]	64718
A_24_P929565	1.3	-3.3	1.1	-1.1	UNQ6975	Homo sapiens putative uncharacterized protein UNQ6975/PRO21958 (UNQ6975), non-coding RNA [NR_033831]	400952
A_23_P120822	1.4	2.6	2.1	-14.5	UPB1	Homo sapiens ureidopropionase, beta (UPB1), mRNA [NM_016327]	51733

A_24_P81968	4.1	3.9	1.2	2.2	UPF3A	Homo sapiens UPF3 regulator of nonsense transcripts homolog A (yeast) (UPF3A), transcript variant 1, mRNA [NM_023011]	65110
A_24_P41690	1.0	1.1	-1.0	-4.1	UPF3A	Homo sapiens UPF3 regulator of nonsense transcripts homolog A (yeast) (UPF3A), transcript variant 1, mRNA [NM_023011]	65110
A_24_P348594	2.0	-1.1	4.0	2.1	UPK3A	Homo sapiens uroplakin 3A (UPK3A), transcript variant 1, mRNA [NM_006953]	7380

A_24_P112160	1.7	-4.2	-1.6	-1.6	UPK3B	Homo sapiens uroplakin 3B (UPK3B), transcript variant 1, mRNA [NM_030570]	80761
A_24_P185986	-2.3	-1.1	-1.2	-1.2	UPRT	Homo sapiens uracil phosphoribosyltransferase (FUR1) homolog (S. cerevisiae) (UPRT), transcript variant 1, mRNA [NM_145052]	139596
A_24_P65910	1.1	1.1	-3.0	-1.0	UQCC	Homo sapiens ubiquinol-cytochrome c reductase complex chaperone (UQCC), nuclear gene encoding mitochondrial protein, transcript variant 1, mRNA [NM_018244]	55245

A_23_P68866	1.3	1.1	2.2	1.0	UQCR10	Homo sapiens ubiquinol-cytochrome c reductase, complex III subunit X (UQCR10), nuclear gene encoding mitochondrial protein, transcript variant 2, mRNA [NM_001003684]	29796
A_23_P31671	-1.0	-1.3	-2.4	-1.3	UQCRB	Homo sapiens ubiquinol-cytochrome c reductase binding protein (UQCRB), nuclear gene encoding mitochondrial protein, transcript variant 1, mRNA [NM_006294]	7381

A_23_P153586	-2.7	1.0	1.0	-1.0	UQCRFS1	Homo sapiens ubiquinol-cytochrome c reductase, Rieske iron-sulfur polypeptide 1 (UQCRFS1), nuclear gene encoding mitochondrial protein, mRNA [NM_006003]	7386
A_23_P57293	1.0	-2.8	-1.1	-1.0	URB1	Homo sapiens URB1 ribosome biogenesis 1 homolog (S. cerevisiae) (URB1), mRNA [NM_014825]	9875
A_24_P940310	2.7	-1.6	2.6	1.8	URB1	Homo sapiens URB1 ribosome biogenesis 1 homolog (S. cerevisiae) (URB1), mRNA [NM_014825]	9875

A_23_P31489	-3.4	1.1	-1.3	-2.9	URGCP	Homo sapiens upregulator of cell proliferation (URGCP), nuclear gene encoding mitochondrial protein, transcript variant 2, mRNA [NM_001077664]	55665
A_23_P94689	-15.8	-1.1	-1.0	-1.5	URM1	Homo sapiens ubiquitin related modifier 1 (URM1), transcript variant 1, mRNA [NM_030914]	81605
A_24_P415168	2.2	1.3	2.4	2.1	URM1	Homo sapiens mRNA expressed in placenta. [D83200]	81605
A_32_P38735	-1.4	-1.5	-1.9	-2.0	UROC1	Homo sapiens urocanase domain containing 1 (UROC1), transcript variant 1, mRNA [NM_144639]	131669

A_23_P35609	1.3	1.2	1.3	-2.8	UROS	Homo sapiens uroporphyrinogen III synthase (UROS), mRNA [NM_000375]	7390
A_23_P153783	1.1	2.1	2.4	1.9	USF2	Homo sapiens upstream transcription factor 2, c-fos interacting (USF2), transcript variant 1, mRNA [NM_003367]	7392
A_23_P127818	1.1	1.1	-1.0	-5.7	USH1C	Homo sapiens cDNA: FLJ21290 fis, clone COL01954. [AK024943]	10083
A_32_P914221	-2.2	-1.9	-1.4	-2.9	USH1G	Homo sapiens Usher syndrome 1G (autosomal recessive) (USH1G), mRNA [NM_173477]	124590

A_23_P86301	-1.2	-2.2	-1.2	-1.6	USH2A	Homo sapiens Usher syndrome 2A (autosomal recessive, mild) (USH2A), transcript variant 1, mRNA [NM_007123]	7399
A_23_P29855	1.0	-9.4	-1.6	-1.9	USO1	Homo sapiens USO1 vesicle docking protein homolog (yeast) (USO1), mRNA [NM_003715]	8615
A_23_P171366	2.8	1.6	1.4	1.3	USP11	Homo sapiens ubiquitin specific peptidase 11 (USP11), mRNA [NM_004651]	8237
A_23_P217596	3.8	4.2	-1.7	1.2	USP12	Homo sapiens ubiquitin specific peptidase 12 (USP12), mRNA [NM_182488]	219333

A_24_P313744	1.7	1.1	-2.5	-1.1	USP14	Homo sapiens ubiquitin specific peptidase 14 (tRNA-guanine transglycosylase) (USP14), transcript variant 1, mRNA [NM_005151]	9097
A_23_P325802	1.7	2.4	-1.4	1.6	USP15	Homo sapiens ubiquitin specific peptidase 15 (USP15), transcript variant 3, mRNA [NM_001252079]	9958
A_23_P257911	-1.0	-1.4	-1.0	-3.2	USP16	Homo sapiens ubiquitin specific peptidase 16 (USP16), transcript variant 3, mRNA [NM_001032410]	10600

A_32_P132206	-1.2	-1.0	-1.0	-3.5	USP18	Homo sapiens ubiquitin specific peptidase 18 (USP18), mRNA [NM_017414]	11274
A_23_P113789	-3.7	-2.3	-4.1	-5.1	USP19	Homo sapiens ubiquitin specific peptidase 19 (USP19), transcript variant 4, mRNA [NM_006677]	10869
A_24_P107317	2.8	3.9	-1.1	1.0	USP2	Homo sapiens ubiquitin specific peptidase 2 (USP2), transcript variant 1, mRNA [NM_004205]	9099
A_24_P139620	-21.6	1.3	-1.1	-2.0	USP21	Homo sapiens ubiquitin specific peptidase 21 (USP21), transcript variant 3, mRNA [NM_001014443]	27005

A_23_P256676	1.1	-1.1	-3.9	-1.1	USP25	Homo sapiens ubiquitin specific peptidase 25 (USP25), mRNA [NM_013396]	29761
A_24_P87114	-7.6	1.0	1.0	-3.4	USP30	Homo sapiens ubiquitin specific peptidase 30 (USP30), mRNA [NM_032663]	84749
A_23_P206612	2.5	2.2	2.3	2.2	USP31	Homo sapiens ubiquitin specific peptidase 31 (USP31), mRNA [NM_020718]	57478
A_23_P107154	-1.2	3.0	1.4	2.1	USP32	Homo sapiens ubiquitin specific peptidase 32 (USP32), mRNA [NM_032582]	84669

A_23_P106	1.5	6.9	3.4	1.8	USP33	Homo sapiens ubiquitin specific peptidase 33 (USP33), transcript variant 1, mRNA [NM_015017]	23032
A_32_P81173	-2.1	-1.4	1.2	-1.4	USP34	Homo sapiens mRNA; cDNA DKFZp586J101 (from clone DKFZp586J101). [AL050376]	9736
A_24_P126417	1.8	-1.4	-1.0	-2.2	USP34	Homo sapiens ubiquitin specific peptidase 34 (USP34), mRNA [NM_014709]	9736
A_24_P410809	1.9	1.0	1.0	2.2	USP34	Homo sapiens ubiquitin specific peptidase 34 (USP34), mRNA [NM_014709]	9736

A_32_P101301	-2.6	1.0	-2.7	-2.8	USP39	Homo sapiens ubiquitin specific peptidase 39 (USP39), mRNA [NM_006590]	10713
A_23_P58091	-1.5	-1.8	-2.9	1.3	USP4	ubiquitin specific peptidase 4 (proto-oncogene) [Source:HGNC Symbol;Acc:12627] [ENST00000462673]	7375
A_32_P54553	-2.3	1.0	1.4	1.0	USP41	ubiquitin specific peptidase 41 [Source:HGNC Symbol;Acc:20070] [ENST00000454608]	373856
A_24_P310009	-6.8	1.1	-1.0	-1.0	USP43	Homo sapiens ubiquitin specific peptidase 43 (USP43), mRNA [NM_153210]	124739

A_23_P134008	-3.3	1.0	-1.9	-1.1	USP45	ubiquitin specific peptidase 45 [Source:HGNC Symbol;Acc:20080] [ENST00000472914]	85015
A_23_P81180	-1.4	-9.5	-1.0	1.9	USP46	Homo sapiens ubiquitin specific peptidase 46 (USP46), transcript variant 1, mRNA [NM_022832]	64854
A_23_P200551	1.0	-2.1	-2.9	-1.4	USP48	Homo sapiens ubiquitin specific peptidase 48 (USP48), transcript variant 1, mRNA [NM_032236]	84196
A_32_P128701	-1.0	2.3	1.0	-10.7	USP53	Homo sapiens ubiquitin specific peptidase 53 (USP53), mRNA [NM_019050]	54532

A_24_P914155	1.1	2.5	1.4	1.5	USP54	Homo sapiens ubiquitin specific peptidase 54 (USP54), mRNA [NM_152586]	159195
A_24_P238040	1.7	1.7	2.3	1.4	USP6	Homo sapiens ubiquitin specific peptidase 6 (Tre-2 oncogene) (USP6), mRNA [NM_004505]	9098
A_23_P128641	1.7	1.6	2.7	1.6	USPL1	Homo sapiens ubiquitin specific peptidase like 1 (USPL1), mRNA [NM_005800]	10208
A_23_P33141	-1.4	-2.2	-2.3	-1.5	UTF1	Homo sapiens undifferentiated embryonic cell transcription factor 1 (UTF1), mRNA [NM_003577]	8433

A_23_P205216	1.7	1.3	2.3	1.1	UTP14A	Homo sapiens UTP14, U3 small nucleolar ribonucleoprotein, homolog A (yeast) (UTP14A), transcript variant 1, mRNA [NM_006649]	10813
A_24_P201879	2.2	2.3	1.9	1.8	UTP14C	Homo sapiens UTP14, U3 small nucleolar ribonucleoprotein, homolog C (yeast) (UTP14C), mRNA [NM_021645]	9724
A_23_P58497	-1.0	-1.1	-2.8	-1.1	UTP15	Homo sapiens UTP15, U3 small nucleolar ribonucleoprotein, homolog (S. cerevisiae) (UTP15), mRNA [NM_032175]	84135

A_23_P162525	-3.0	-2.7	1.5	1.9	UTP20	Homo sapiens UTP20, small subunit (SSU) processome component, homolog (yeast) (UTP20), mRNA [NM_014503]	27340
A_32_P208733	-2.5	1.1	-1.0	-1.0	UTP23	Homo sapiens cDNA FLJ30717 fis, clone FCBBF2001672. [AK055279]	84294
A_23_P164237	3.2	2.7	2.4	4.5	UTP6	Homo sapiens UTP6, small subunit (SSU) processome component, homolog (yeast) (UTP6), mRNA [NM_018428]	55813
A_23_P214779	-1.7	-3.5	-2.0	-2.9	UTRN	Homo sapiens utrophin (UTRN), mRNA [NM_007124]	7402

A_23_P146885	2.5	1.0	-1.2	-1.1	UTS2R	Homo sapiens urotensin 2 receptor (UTS2R), mRNA [NM_018949]	2837
A_23_P329835	-1.8	1.1	-1.8	-7.8	UTY	Homo sapiens ubiquitously transcribed tetratricopeptide repeat gene, Y-linked (UTY), transcript variant 3, mRNA [NM_007125]	7404
A_23_P67829	1.0	1.3	-2.0	1.7	UXS1	Homo sapiens UDP-glucuronate decarboxylase 1 (UXS1), mRNA [NM_025076]	80146
A_23_P77590	-2.3	-1.3	-1.1	-1.7	VAC14	Homo sapiens Vac14 homolog (S. cerevisiae) (VAC14), mRNA [NM_018052]	55697

A_23_P77593	3.8	3.0	-1.0	3.2	VAC14	Homo sapiens Vac14 homolog (S. cerevisiae) (VAC14), mRNA [NM_018052]	55697
A_23_P105545	2.7	3.8	4.1	1.0	VAMP1	Homo sapiens vesicle-associated membrane protein 1 (synaptobrevin 1) (VAMP1), transcript variant 3, mRNA [NM_016830]	6843
A_24_P942030	1.8	1.3	-3.9	1.2	VAMP4	Homo sapiens vesicle-associated membrane protein 4 (VAMP4), transcript variant 1, mRNA [NM_003762]	8674

A_23_P39840	-2.1	1.0	-2.7	-1.1	VAMP5	Homo sapiens vesicle-associated membrane protein 5 (myobrevin) (VAMP5), mRNA [NM_006634]	10791
A_23_P22671	1.9	1.9	1.7	4.6	VAMP7	Homo sapiens vesicle-associated membrane protein 7 (VAMP7), transcript variant 1, mRNA [NM_005638]	6845
A_24_P199655	1.1	1.1	1.1	-2.3	VANGL1	Homo sapiens vang-like 1 (van gogh, Drosophila) (VANGL1), transcript variant 1, mRNA [NM_138959]	81839

A_23_P369316	-1.2	-10.2	-1.3	-1.6	VANGL1	Homo sapiens vang-like 1 (van gogh, Drosophila) (VANGL1), transcript variant 1, mRNA [NM_138959]	81839
A_23_P91293	1.8	1.9	2.0	-2.1	VAPB	Homo sapiens VAMP (vesicle-associated membrane protein)-associated protein B and C (VAPB), transcript variant 1, mRNA [NM_004738]	9217
A_23_P258714	2.9	-1.0	-1.1	1.5	VARS	Homo sapiens valyl-tRNA synthetase (VARS), nuclear gene encoding mitochondrial protein, mRNA [NM_006295]	7407

A_23_P324807	1.5	-4.3	-1.5	-1.0	VARS2	Homo sapiens valyl-tRNA synthetase 2, mitochondrial (putative) (VARS2), nuclear gene encoding mitochondrial protein, transcript variant 2, mRNA [NM_020442]	57176
A_23_P77000	1.6	3.7	-2.0	1.1	VASH1	Homo sapiens vasohibin 1 (VASH1), mRNA [NM_014909]	22846
A_24_P304629	2.2	2.2	3.1	1.5	VASH1	Homo sapiens vasohibin 1 (VASH1), mRNA [NM_014909]	22846
A_24_P85391	1.0	-2.9	-1.1	-1.4	VASH2	vasohibin 2 [Source:HGNC Symbol;Acc:25723] [ENST00000271776]	79805

A_23_P115492	-1.1	2.5	2.7	1.1	VASH2	Homo sapiens vasohibin 2 (VASH2), transcript variant 1, mRNA [NM_024749]	79805
A_24_P152527	1.3	1.3	-6.6	-2.6	VAT1	Homo sapiens vesicle amine transport protein 1 homolog (T. californica) (VAT1), mRNA [NM_006373]	10493
A_23_P26954	-1.1	2.4	-1.5	-3.0	VAT1	Homo sapiens vesicle amine transport protein 1 homolog (T. californica) (VAT1), mRNA [NM_006373]	10493

A_23_P400449	-2.3	1.0	1.0	1.0	VAT1L	Homo sapiens vesicle amine transport protein 1 homolog (T. californica)-like (VAT1L), mRNA [NM_020927]	57687
A_23_P201551	-1.3	-6.9	-1.3	1.3	VAV3	Homo sapiens vav 3 guanine nucleotide exchange factor (VAV3), transcript variant 1, mRNA [NM_006113]	10451
A_24_P7950	-1.6	-2.1	-1.7	-1.6	VAV3	Homo sapiens vav 3 guanine nucleotide exchange factor (VAV3), transcript variant 1, mRNA [NM_006113]	10451

A_23_P256223	-1.5	1.1	-3.6	1.0	VBP1	Homo sapiens von Hippel-Lindau binding protein 1 (VBP1), mRNA [NM_003372]	7411
A_23_P34345	1.4	-8.6	1.3	1.3	VCAM1	Homo sapiens vascular cell adhesion molecule 1 (VCAM1), transcript variant 1, mRNA [NM_001078]	7412
A_23_P202280	4.8	4.7	4.3	4.2	VCL	Homo sapiens vinculin (VCL), transcript variant 1, mRNA [NM_014000]	7414
A_24_P47182	1.1	2.2	1.0	1.1	VCL	Homo sapiens vinculin (VCL), transcript variant 1, mRNA [NM_014000]	7414

A_24_P230282	2.2	2.0	1.2	1.3	VCX2	Homo sapiens variable charge, X-linked 2 (VCX2), mRNA [NM_016378]	51480
A_23_P11390	2.9	1.1	1.3	-1.4	VCY	Homo sapiens variable charge, Y-linked (VCY), mRNA [NM_004679]	9084
A_32_P41292	1.5	1.0	-2.7	1.9	VDAC2	Homo sapiens voltage-dependent anion channel 2 (VDAC2), nuclear gene encoding mitochondrial protein, transcript variant 2, mRNA [NM_003375]	7417
A_23_P60069	-1.3	-1.4	-3.4	-1.8	VDAC3	Homo sapiens voltage-dependent anion channel 3 (VDAC3), transcript variant 1, mRNA [NM_005662]	7419

A_23_P70398	-1.3	1.9	-3.2	1.6	VEGFA	Homo sapiens vascular endothelial growth factor A (VEGFA), transcript variant 6, mRNA [NM_001025370]	7422
A_23_P1594	4.0	3.5	3.3	1.5	VEGFB	Homo sapiens vascular endothelial growth factor B (VEGFB), transcript variant VEGFB-186, mRNA [NM_003377]	7423
A_24_P55971	1.5	2.5	1.6	1.2	VEGFB	Homo sapiens vascular endothelial growth factor B (VEGFB), transcript variant VEGFB-186, mRNA [NM_003377]	7423

A_23_P167096	1.1	2.1	2.0	1.3	VEGFC	Homo sapiens vascular endothelial growth factor C (VEGFC), mRNA [NM_005429]	7424
A_23_P419150	6.2	25.8	24.3	23.2	VENTX	Homo sapiens VENT homeobox (VENTX), mRNA [NM_014468]	27287
A_23_P380208	-5.4	-1.8	-3.3	-1.8	VEPH1	Homo sapiens ventricular zone expressed PH domain homolog 1 (zebrafish) (VEPH1), transcript variant 1, mRNA [NM_024621]	79674
A_24_P129326	7.9	5.4	4.5	5.0	VGF	Homo sapiens VGF nerve growth factor inducible (VGF), mRNA [NM_003378]	7425

A_23_P253123	1.2	-1.2	-4.5	-3.8	VGLL1	Homo sapiens vestigial like 1 (Drosophila) (VGLL1), mRNA [NM_016267]	51442
A_32_P106376	-2.0	1.4	1.0	-1.6	VGLL2	Homo sapiens vestigial like 2 (Drosophila) (VGLL2), transcript variant 1, mRNA [NM_182645]	245806
A_32_P459849	2.2	2.7	1.1	-1.3	VGLL2	Homo sapiens vestigial like 2 (Drosophila) (VGLL2), transcript variant 2, mRNA [NM_153453]	245806
A_24_P67585	1.1	-3.4	-1.4	-1.4	VGLL3	Homo sapiens vestigial like 3 (Drosophila) (VGLL3), mRNA [NM_016206]	389136

A_23_P132595	-5.4	1.1	1.1	1.3	VGLL4	Homo sapiens vestigial like 4 (Drosophila) (VGLL4), transcript variant 2, mRNA [NM_014667]	9686
A_23_P132611	1.7	1.2	3.4	1.6	VHL	Homo sapiens von Hippel-Lindau tumor suppressor (VHL), transcript variant 1, mRNA [NM_000551]	7428
A_23_P63096	1.1	-9.9	-1.0	-2.5	VHLL	Homo sapiens von Hippel-Lindau tumor suppressor-like (VHLL), mRNA [NM_001004319]	391104
A_23_P161190	2.3	1.0	2.5	1.0	VIM	Homo sapiens vimentin (VIM), mRNA [NM_003380]	7431

A_23_P161194	-2.5	-1.3	-2.5	-1.7	VIM	Homo sapiens vimentin (VIM), mRNA [NM_003380]	7431
A_23_P145978	-1.1	-1.0	-1.0	-2.6	VIPR2	Homo sapiens vasoactive intestinal peptide receptor 2 (VIPR2), mRNA [NM_003382]	7434
A_23_P413815	-1.8	-1.8	-2.6	-3.2	VKORC1L1	Homo sapiens vitamin K epoxide reductase complex, subunit 1-like 1 (VKORC1L1), mRNA [NM_173517]	154807
A_32_P151621	2.1	1.0	1.0	-1.0	VMA21	Homo sapiens VMA21 vacuolar H+-ATPase homolog (S. cerevisiae) (VMA21), mRNA [NM_001017980]	203547

A_32_P9753	-1.5	1.4	-3.9	-3.7	VMP1	Homo sapiens transmembrane protein 49, mRNA (cDNA clone IMAGE:4295896), with apparent retained intron. [BC024020]	81671
A_23_P303238	-1.2	1.1	-2.3	1.3	VN1R5	Homo sapiens vomeronasal 1 receptor 5 (gene/pseudogene) (VN1R5), mRNA [NM_173858]	317705
A_24_P337700	1.8	-1.2	-1.0	-4.4	VNN1	Homo sapiens vanin 1 (VNN1), mRNA [NM_004666]	8876
A_23_P255345	1.0	-1.0	-21.4	-1.1	VNN1	Homo sapiens vanin 1 (VNN1), mRNA [NM_004666]	8876

A_23_P122724	-1.6	-2.7	1.2	-3.3	VNN2	Homo sapiens vanin 2 (VNN2), transcript variant 1, mRNA [NM_004665]	8875
A_24_P26170	2.1	-1.1	-1.6	-7.1	VPRBP	Homo sapiens Vpr (HIV-1) binding protein (VPRBP), transcript variant 1, mRNA [NM_014703]	9730
A_23_P166371	2.3	5.5	1.8	4.8	VPREB3	Homo sapiens pre-B lymphocyte 3 (VPREB3), mRNA [NM_013378]	29802
A_32_P232523	-4.3	1.3	-1.3	1.0	VPS13A	vacuolar protein sorting 13 homolog A (S. cerevisiae) [Source:HGNC Symbol;Acc:1908] [ENST00000471439]	23230

A_24_P98251	-2.7	1.0	-1.0	1.0	VPS13B	Homo sapiens vacuolar protein sorting 13 homolog B (yeast) (VPS13B), transcript variant 5, mRNA [NM_017890]	157680
A_23_P82975	1.0	-2.5	-1.6	-1.3	VPS13B	Homo sapiens vacuolar protein sorting 13 homolog B (yeast) (VPS13B), transcript variant 4, mRNA [NM_181661]	157680
A_23_P364837	-2.3	-1.0	-3.5	-2.2	VPS13D	Homo sapiens vacuolar protein sorting 13 homolog D (S. cerevisiae) (VPS13D), transcript variant 1, mRNA [NM_015378]	55187

A_24_P18802	2.7	1.3	-1.5	1.3	VPS18	Homo sapiens vacuolar protein sorting 18 homolog (S. cerevisiae) (VPS18), mRNA [NM_020857]	57617
A_23_P75255	-1.0	-5.6	-1.0	-1.0	VPS26A	Homo sapiens vacuolar protein sorting 26 homolog A (S. pombe) (VPS26A), transcript variant 1, mRNA [NM_004896]	9559
A_23_P52903	-1.2	2.7	1.1	-1.1	VPS26B	Homo sapiens vacuolar protein sorting 26 homolog B (S. pombe) (VPS26B), mRNA [NM_052875]	112936

A_23_P203323	5.9	4.3	4.0	-1.1	VPS26B	Homo sapiens vacuolar protein sorting 26 homolog B (S. pombe) (VPS26B), mRNA [NM_052875]	112936
A_23_P128384	-2.1	-1.0	-1.4	-1.1	VPS29	Homo sapiens vacuolar protein sorting 29 homolog (S. cerevisiae) (VPS29), transcript variant 1, mRNA [NM_016226]	51699
A_24_P175989	-2.9	-4.3	-4.0	1.0	VPS29	Homo sapiens vacuolar protein sorting 29 homolog (S. cerevisiae) (VPS29), transcript variant 1, mRNA [NM_016226]	51699

A_23_P48964	1.1	2.3	1.5	-1.5	VPS33B	Homo sapiens vacuolar protein sorting 33 homolog B (yeast) (VPS33B), mRNA [NM_018668]	26276
A_24_P941649	1.1	-2.6	-1.0	-1.0	VPS37A	Homo sapiens vacuolar protein sorting 37 homolog A (S. cerevisiae) (VPS37A), transcript variant 1, mRNA [NM_152415]	137492
A_23_P150876	1.0	-1.0	-1.1	-5.6	VPS37B	Homo sapiens vacuolar protein sorting 37 homolog B (S. cerevisiae) (VPS37B), mRNA [NM_024667]	79720

A_23_P409417	1.2	1.5	2.0	-1.5	VPS37D	Homo sapiens vacuolar protein sorting 37 homolog D (S. cerevisiae) (VPS37D), mRNA [NM_001077621]	155382
A_23_P23719	2.6	1.8	4.3	2.0	VPS45	Homo sapiens vacuolar protein sorting 45 homolog (S. cerevisiae) (VPS45), mRNA [NM_007259]	11311
A_24_P362394	-2.1	-1.8	-2.4	-1.4	VPS45	Homo sapiens vacuolar protein sorting 45 homolog (S. cerevisiae) (VPS45), mRNA [NM_007259]	11311

A_24_P147398	3.4	3.5	3.4	3.4	VPS53	Homo sapiens vacuolar protein sorting 53 homolog (S. cerevisiae) (VPS53), transcript variant 2, mRNA [NM_018289]	55275
A_24_P279489	1.0	-2.5	-1.0	1.0	VPS53	Homo sapiens vacuolar protein sorting 53 homolog (S. cerevisiae) (VPS53), transcript variant 1, mRNA [NM_001128159]	55275
A_24_P357709	-1.0	-2.6	-1.0	1.4	VPS8	Homo sapiens vacuolar protein sorting 8 homolog (S. cerevisiae) (VPS8), transcript variant 1, mRNA [NM_001009921]	23355

A_23_P431587	-1.0	-1.0	-1.1	-2.6	VPS8	Homo sapiens vacuolar protein sorting 8 homolog (S. cerevisiae) (VPS8), transcript variant 1, mRNA [NM_001009921]	23355
A_23_P107933	1.3	-12.3	-5.1	-1.1	VRK3	Homo sapiens vaccinia related kinase 3 (VRK3), transcript variant 1, mRNA [NM_016440]	51231
A_32_P163147	1.9	1.1	1.6	2.2	VSIG1	Homo sapiens V-set and immunoglobulin domain containing 1 (VSIG1), transcript variant 2, mRNA [NM_182607]	340547

A_24_P914000	4.5	-1.6	-3.6	-3.0	VSIG10	Homo sapiens hypothetical protein FLJ20674, mRNA (cDNA clone IMAGE:3138594). [BC001437]	54621
A_23_P72059	-1.0	2.5	2.4	1.9	VSIG10	Homo sapiens V-set and immunoglobulin domain containing 10 (VSIG10), mRNA [NM_019086]	54621
A_23_P101246	-2.2	-3.4	-2.9	-1.2	VSIG10L	Homo sapiens V-set and immunoglobulin domain containing 10 like (VSIG10L), mRNA [NM_001163922]	147645

A_23_P101240	3.3	3.5	3.4	2.0	VSIG10L	Homo sapiens V-set and immunoglobulin domain containing 10 like (VSIG10L), mRNA [NM_001163922]	147645
A_23_P217269	-6.1	1.6	-2.1	1.2	VSIG4	Homo sapiens V-set and immunoglobulin domain containing 4 (VSIG4), transcript variant 1, mRNA [NM_007268]	11326
A_23_P142201	2.8	4.4	2.6	2.4	VSTM2B	Homo sapiens V-set and transmembrane domain containing 2B (VSTM2B), mRNA [NM_001146339]	342865

A_23_P79803	-1.0	-1.1	-4.6	-1.1	VSTM2L	Homo sapiens V-set and transmembrane domain containing 2 like (VSTM2L), mRNA [NM_080607]	128434
A_24_P941038	10.8	10.9	10.3	7.3	VSTM4	Homo sapiens V-set and transmembrane domain containing 4 (VSTM4), transcript variant 1, mRNA [NM_001031746]	196740
A_23_P131990	2.1	1.7	1.5	-1.0	VSX1	Homo sapiens visual system homeobox 1 (VSX1), transcript variant 1, mRNA [NM_014588]	30813
A_23_P78099	-1.1	3.7	1.5	2.6	VTN	Homo sapiens vitronectin (VTN), mRNA [NM_000638]	7448

A_23_P86089	-1.7	-6.8	-2.3	-2.7	VWA1	Homo sapiens von Willebrand factor A domain containing 1 (VWA1), transcript variant 1, mRNA [NM_022834]	64856
A_24_P122746	3.9	2.2	2.1	2.3	VWA1	Homo sapiens von Willebrand factor A domain containing 1 (VWA1), transcript variant 1, mRNA [NM_022834]	64856
A_23_P390665	2.2	2.2	1.3	2.3	VWA3A	Homo sapiens von Willebrand factor A domain containing 3A (VWA3A), mRNA [NM_173615]	146177

A_32_P46603	1.3	-2.4	1.3	-1.7	VWA3B	Homo sapiens von Willebrand factor A domain containing 3B (VWA3B), mRNA [NM_144992]	200403
A_23_P98455	2.0	2.0	1.8	1.9	VWA5A	Homo sapiens von Willebrand factor A domain containing 5A (VWA5A), transcript variant 1, mRNA [NM_014622]	4013
A_24_P75460	-15.1	-1.0	-1.1	-1.1	VWC2L	Homo sapiens von Willebrand factor C domain containing protein 2-like (VWC2L), mRNA [NM_001080500]	402117

A_32_P148745	1.4	1.4	2.3	-2.2	VWDE	Homo sapiens von Willebrand factor D and EGF domains (VWDE), mRNA [NM_001135924]	221806
A_23_P381505	1.1	-3.6	1.1	1.1	VWDE	Homo sapiens von Willebrand factor D and EGF domains (VWDE), mRNA [NM_001135924]	221806
A_23_P201996	-2.4	-1.0	-3.4	-2.8	WAC	Homo sapiens WW domain containing adaptor with coiled-coil (WAC), transcript variant 1, mRNA [NM_016628]	51322
A_23_P65651	1.6	1.3	2.2	1.1	WARS	Homo sapiens tryptophanyl-tRNA synthetase (WARS), transcript variant 1, mRNA [NM_004184]	7453

A_23_P96331	1.0	1.5	3.2	1.8	WAS	Homo sapiens Wiskott-Aldrich syndrome (eczema-thrombocytopenia) (WAS), mRNA [NM_000377]	7454
A_23_P168306	1.2	1.3	1.4	-3.2	WASF1	Homo sapiens WAS protein family, member 1 (WASF1), transcript variant 1, mRNA [NM_003931]	8936
A_24_P76546	1.1	1.6	2.0	1.1	WASF2	Homo sapiens WAS protein family, member 2 (WASF2), transcript variant 1, mRNA [NM_006990]	10163
A_32_P41461	-3.6	-1.6	-3.6	-3.7	WASF2	Homo sapiens WAS protein family, member 2 (WASF2), transcript variant 1, mRNA [NM_006990]	10163

A_23_P325339	1.1	1.2	-1.3	-2.4	WASH1	Homo sapiens mRNA for FLJ00038 protein, partial cds. [AK024448]	100287171
A_24_P24831	1.7	2.0	2.3	-10.9	WASH1	Homo sapiens WAS protein family homolog 1 (WASH1), mRNA [NM_182905]	100287171
A_24_P41291	1.1	1.5	2.2	-1.5	WASH1	Homo sapiens WAS protein family homolog 1 (WASH1), mRNA [NM_182905]	100287171
A_23_P11744	-1.8	-4.6	-1.2	-2.5	WASH1	Homo sapiens WAS protein family homolog 1 (WASH1), mRNA [NM_182905]	100287171

A_23_P11742	2.5	1.3	1.3	3.4	WASH2P	Homo sapiens WAS protein family homolog 2 pseudogene (WASH2P), non-coding RNA [NR_024077]	375260
A_32_P228601	2.3	-1.5	1.4	1.5	WASH7P	Homo sapiens cDNA, FLJ17181. [AK310139]	653635
A_23_P252808	-1.8	-1.5	-1.2	-2.9	WBP1	Homo sapiens WW domain binding protein 1 (WBP1), mRNA [NM_012477]	23559
A_23_P410128	2.2	1.9	1.2	1.5	WBP2NL	Homo sapiens WBP2 N-terminal like (WBP2NL), mRNA [NM_152613]	164684

A_24_P370420	1.4	2.7	2.7	5.7	WBSCR16	Homo sapiens Williams-Beuren syndrome chromosome region 16 (WBSCR16), mRNA [NM_030798]	81554
A_23_P111517	2.1	-8.5	-1.1	1.3	WBSCR17	Homo sapiens Williams-Beuren syndrome chromosome region 17 (WBSCR17), mRNA [NM_022479]	64409
A_32_P220826	4.2	3.9	3.4	1.0	WBSCR22	Williams Beuren syndrome chromosome region 22 [Source:HGNC Symbol;Acc:16405] [ENST00000423166]	114049

A_32_P10396	1.9	2.5	2.5	2.1	WDFY3	Homo sapiens WD repeat and FYVE domain containing 3 (WDFY3), mRNA [NM_014991]	23001
A_32_P30905	2.1	-1.0	1.6	1.4	WDFY4	Homo sapiens WDFY family member 4 (WDFY4), mRNA [NM_020945]	57705
A_23_P502913	-2.3	1.8	-1.8	1.1	WDR1	Homo sapiens WD repeat domain 1 (WDR1), transcript variant 1, mRNA [NM_017491]	9948
A_23_P213000	-8.1	1.0	1.0	1.5	WDR1	Homo sapiens WD repeat domain 1 (WDR1), transcript variant 1, mRNA [NM_017491]	9948

A_24_P394361	-2.1	-9.5	-2.1	-2.1	WDR19	Homo sapiens WD repeat domain 19 (WDR19), mRNA [NM_025132]	57728
A_23_P110135	1.3	-6.9	1.2	1.0	WDR19	Homo sapiens WD repeat domain 19 (WDR19), mRNA [NM_025132]	57728
A_23_P205255	3.4	3.9	3.2	-6.3	WDR20	Homo sapiens WD repeat domain 20 (WDR20), transcript variant 7, mRNA [NM_001242417]	91833
A_23_P26476	1.3	1.3	-1.5	-14.5	WDR24	Homo sapiens WD repeat domain 24 (WDR24), mRNA [NM_032259]	84219

A_23_P159071	-1.9	-1.1	1.2	-2.0	WDR25	Homo sapiens WD repeat domain 25 (WDR25), transcript variant 1, mRNA [NM_024515]	79446
A_24_P36868	1.1	-9.5	-1.3	1.1	WDR26	Homo sapiens WD repeat domain 26 (WDR26), transcript variant 1, mRNA [NM_025160]	80232
A_32_P415151	-1.0	-1.0	-4.1	-1.1	WDR27	Homo sapiens WD repeat domain 27 (WDR27), transcript variant 1, mRNA [NM_182552]	253769
A_23_P23318	1.9	1.8	1.7	2.2	WDR3	Homo sapiens WD repeat domain 3 (WDR3), mRNA [NM_006784]	10885

A_24_P102920	-4.5	1.1	1.1	1.1	WDR33	Homo sapiens WD repeat domain 33 (WDR33), transcript variant 2, mRNA [NM_001006622]	55339
A_23_P20823	1.3	-2.8	-3.8	1.4	WDR34	Homo sapiens WD repeat domain 34 (WDR34), mRNA [NM_052844]	89891
A_23_P110598	-1.1	-1.1	-1.1	2.7	WDR36	Homo sapiens WD repeat domain 36 (WDR36), mRNA [NM_139281]	134430
A_23_P211302	1.0	1.0	1.8	5.7	WDR4	Homo sapiens WD repeat domain 4 (WDR4), transcript variant 2, mRNA [NM_033661]	10785

A_23_P23748	-2.4	1.0	-1.0	1.1	WDR47	Homo sapiens WD repeat domain 47 (WDR47), transcript variant 2, mRNA [NM_014969]	22911
A_23_P80409	-1.1	-1.2	-1.3	-2.9	WDR48	Homo sapiens WD repeat domain 48 (WDR48), mRNA [NM_020839]	57599
A_23_P32558	-1.3	-1.4	-5.8	-1.4	WDR5	Homo sapiens WD repeat domain 5 (WDR5), transcript variant 1, mRNA [NM_017588]	11091
A_23_P110090	-1.2	1.8	-1.0	2.7	WDR52	Homo sapiens WD repeat domain 52 (WDR52), transcript variant 1, mRNA [NM_001164496]	55779

A_23_P68072	-2.7	2.0	2.8	2.4	WDR54	Homo sapiens WD repeat domain 54 (WDR54), mRNA [NM_032118]	84058
A_24_P200549	3.2	2.3	2.6	2.0	WDR55	Homo sapiens WD repeat domain 55 (WDR55), mRNA [NM_017706]	54853
A_23_P339705	-1.7	3.0	-1.4	1.4	WDR62	Homo sapiens WD repeat domain 62 (WDR62), transcript variant 2, mRNA [NM_173636]	284403
A_23_P399116	-2.1	1.5	2.0	1.0	WDR64	Homo sapiens WD repeat domain 64 (WDR64), mRNA [NM_144625]	128025

A_23_P11980	-1.0	-1.0	-1.1	-2.9	WDR65	Homo sapiens WD repeat domain 65 (WDR65), transcript variant 5, mRNA [NM_001195831]	149465
A_23_P363275	1.1	-7.4	-1.1	1.2	WDR66	Homo sapiens WD repeat domain 66 (WDR66), transcript variant 1, mRNA [NM_144668]	144406
A_23_P354208	-1.1	-2.9	-2.4	-1.5	WDR81	Homo sapiens WD repeat domain 81 (WDR81), transcript variant 2, mRNA [NM_152348]	124997
A_24_P388536	1.1	-14.4	1.0	1.1	WDR82	Homo sapiens WD repeat domain 82 (WDR82), mRNA [NM_025222]	80335

A_23_P39024	2.1	1.1	1.0	1.1	WDR83	Homo sapiens WD repeat domain 83 (WDR83), transcript variant 2, mRNA [NM_032332]	84292
A_23_P406957	5.3	1.0	3.7	1.7	WDR85	Homo sapiens WD repeat domain 85 (WDR85), mRNA [NM_138778]	92715
A_23_P163047	2.1	-1.3	-2.0	1.2	WDR89	Homo sapiens WD repeat domain 89 (WDR89), transcript variant 1, mRNA [NM_001008726]	112840
A_24_P275723	1.3	-1.1	-1.0	-2.3	WDR90	WD repeat domain 90 [Source:HGNC Symbol;Acc:26960] [ENST00000546516]	197335

A_23_P411833	1.3	-2.3	-2.1	-2.4	WDR90	Homo sapiens WD repeat domain 90 (WDR90), mRNA [NM_145294]	197335
A_24_P102895	1.8	2.3	-1.9	1.5	WDTC1	Homo sapiens WD and tetratricopeptide repeats 1 (WDTC1), mRNA [NM_015023]	23038
A_23_P86054	3.7	-2.3	1.9	3.3	WDTC1	Homo sapiens WD and tetratricopeptide repeats 1 (WDTC1), mRNA [NM_015023]	23038
A_23_P127926	1.5	-2.7	1.3	-1.0	WEE1	Homo sapiens WEE1 homolog (S. pombe) (WEE1), transcript variant 1, mRNA [NM_003390]	7465

A_23_P368847	-1.0	-3.5	-1.3	-3.1	WFDC10A	Homo sapiens WAP four-disulfide core domain 10A (WFDC10A), mRNA [NM_080753]	140832
A_32_P951	4.1	1.2	-1.0	-1.0	WFDC10B	Homo sapiens WAP four-disulfide core domain 10B (WFDC10B), transcript variant 1, mRNA [NM_172006]	280664
A_23_P68436	1.6	1.1	-1.0	-5.7	WFDC12	Homo sapiens WAP four-disulfide core domain 12 (WFDC12), mRNA [NM_080869]	128488
A_24_P250507	-4.1	-5.5	-1.6	1.1	WFDC13	Homo sapiens WAP four-disulfide core domain 13 (WFDC13), mRNA [NM_172005]	164237

A_23_P218675	1.9	2.4	3.5	1.4	WFDC2	Homo sapiens WAP four-disulfide core domain 2 (WFDC2), mRNA [NM_006103]	10406
A_23_P218669	-3.2	-2.3	-2.1	-2.6	WFDC5	Homo sapiens WAP four-disulfide core domain 5 (WFDC5), mRNA [NM_145652]	149708
A_23_P102634	-1.1	2.5	-1.3	-1.5	WFDC6	Homo sapiens WAP four-disulfide core domain 6 (WFDC6), mRNA [NM_080827]	140870
A_32_P873201	-1.8	-1.1	-2.4	-1.1	WFDC8	Homo sapiens WAP four-disulfide core domain 8 (WFDC8), transcript variant a, mRNA [NM_130896]	90199

A_24_P399230	1.2	2.2	-1.3	1.2	WFIKKN2	Homo sapiens WAP, follistatin/kazal, immunoglobulin, kunitz and netrin domain containing 2 (WFIKKN2), mRNA [NM_175575]	124857
A_23_P389919	2.2	1.0	1.0	1.0	WHSC1	Homo sapiens Wolf-Hirschhorn syndrome candidate 1 (WHSC1), transcript variant 1, mRNA [NM_133330]	7468
A_23_P216132	-1.1	1.6	2.9	3.2	WHSC1L1	Homo sapiens Wolf-Hirschhorn syndrome candidate 1-like 1 (WHSC1L1), transcript variant short, mRNA [NM_017778]	54904

A_24_P83183	1.5	1.5	-2.4	1.4	WHSC2	Homo sapiens Wolf-Hirschhorn syndrome candidate 2 (WHSC2), mRNA [NM_005663]	7469
A_23_P213216	2.4	-1.4	-1.1	2.8	WHSC2	Homo sapiens Wolf-Hirschhorn syndrome candidate 2 (WHSC2), mRNA [NM_005663]	7469
A_24_P917612	-1.5	-1.0	-1.0	4.4	WIBG	Homo sapiens within bgcn homolog (Drosophila), mRNA (cDNA clone IMAGE:3897762), partial cds. [BC009627]	84305

A_23_P330616	-1.1	-2.0	1.1	-2.6	WIPF1	Homo sapiens WAS/WASL interacting protein family, member 1 (WIPF1), transcript variant 2, mRNA [NM_001077269]	7456
A_23_P330611	1.1	1.1	-12.1	1.0	WIPF1	Homo sapiens WAS/WASL interacting protein family, member 1 (WIPF1), transcript variant 2, mRNA [NM_001077269]	7456
A_23_P366455	3.7	3.5	3.3	3.5	WIPF2	Homo sapiens WAS/WASL interacting protein family, member 2 (WIPF2), mRNA [NM_133264]	147179

A_24_P838748	12.8	11.9	4.0	5.4	WIPF2	WAS/WASL interacting protein family, member 2 [Source:HGNC Symbol;Acc:30923] [ENST00000323571]	147179
A_24_P149395	1.0	-5.2	-4.6	-3.1	WIPI2	Homo sapiens WD repeat domain, phosphoinositide interacting 2 (WIPI2), transcript variant 1, mRNA [NM_015610]	26100
A_23_P81676	-24.8	-1.3	1.1	1.0	WISP3	Homo sapiens WNT1 inducible signaling pathway protein 3 (WISP3), transcript variant 3, mRNA [NM_198239]	8838

A_23_P405531	-1.5	-1.1	-2.1	-1.7	WIZ	Homo sapiens widely interspaced zinc finger motifs (WIZ), mRNA [NM_021241]	58525
A_23_P84587	1.0	-1.0	-1.1	-4.4	WNK1	Homo sapiens WNK lysine deficient protein kinase 1 (WNK1), transcript variant 1, mRNA [NM_018979]	65125
A_24_P942600	-1.0	-1.1	-2.5	-1.1	WNK2	Homo sapiens mRNA for KIAA1760 protein, partial cds. [AB051547]	65268
A_32_P99055	-2.0	1.2	-1.9	1.0	WNK3	Homo sapiens WNK lysine deficient protein kinase 3 (WNK3), transcript variant 1, mRNA [NM_020922]	65267

A_23_P102117	1.5	1.2	-1.0	2.1	WNT10A	Homo sapiens wingless-type MMTV integration site family, member 10A (WNT10A), mRNA [NM_025216]	80326
A_23_P162322	3.9	1.1	1.1	1.2	WNT10B	Homo sapiens wingless-type MMTV integration site family, member 10B (WNT10B), mRNA [NM_003394]	7480
A_24_P35643	1.2	1.0	1.3	-5.6	WNT11	Homo sapiens wingless-type MMTV integration site family, member 11 (WNT11), mRNA [NM_004626]	7481

A_23_P138352	1.1	1.0	1.1	-13.5	WNT2B	Homo sapiens wingless-type MMTV integration site family, member 2B (WNT2B), transcript variant WNT-2B1, mRNA [NM_004185]	7482
A_23_P385690	2.2	1.2	-1.6	1.1	WNT3A	Homo sapiens wingless-type MMTV integration site family, member 3A (WNT3A), mRNA [NM_033131]	89780
A_23_P211926	-2.1	1.0	-1.0	1.3	WNT5A	Homo sapiens wingless-type MMTV integration site family, member 5A (WNT5A), mRNA [NM_003392]	7474

A_23_P119916	4.0	3.5	1.9	3.1	WNT6	Homo sapiens wingless-type MMTV integration site family, member 6 (WNT6), mRNA [NM_006522]	7475
A_24_P208513	-8.4	-1.7	-7.7	-1.7	WNT6	Homo sapiens wingless-type MMTV integration site family, member 6 (WNT6), mRNA [NM_006522]	7475
A_23_P258410	-1.0	-4.7	-1.7	-1.7	WNT7A	Homo sapiens wingless-type MMTV integration site family, member 7A (WNT7A), mRNA [NM_004625]	7476

A_23_P320054	1.5	-1.4	-1.0	-2.5	WNT7B	Homo sapiens wingless-type MMTV integration site family, member 7B (WNT7B), mRNA [NM_058238]	7477
A_23_P55064	3.1	2.1	2.0	-1.1	WRAP53	Homo sapiens WD repeat containing, antisense to TP53 (WRAP53), transcript variant 1, mRNA [NM_018081]	55135
A_23_P214344	-19.9	1.1	1.3	-1.5	WRNIP1	Werner helicase interacting protein 1 [Source:HGNC Symbol;Acc:20876] [ENST00000380769]	56897

A_23_P147238	1.0	-1.0	5.1	-1.1	WSB2	Homo sapiens WD repeat and SOCS box containing 2 (WSB2), mRNA [NM_018639]	55884
A_23_P305914	-1.3	-1.0	-2.2	-1.4	WSCD2	Homo sapiens WSC domain containing 2 (WSCD2), mRNA [NM_014653]	9671
A_23_P371155	-1.2	3.3	1.0	1.9	WTAP	Homo sapiens Wilms tumor 1 associated protein (WTAP), transcript variant 1, mRNA [NM_004906]	9589
A_23_P345650	1.3	1.3	-2.1	-1.0	WWC3	Homo sapiens WWC family member 3 (WWC3), mRNA [NM_015691]	55841

A_23_P34176	1.0	1.0	-2.0	1.7	WWC3	Homo sapiens WWC family member 3 (WWC3), mRNA [NM_015691]	55841
A_23_P71972	-1.3	-5.1	-1.3	-1.3	WWOX	Homo sapiens WW domain containing oxidoreductase (WWOX), transcript variant 1, mRNA [NM_016373]	51741
A_24_P116606	-1.2	5.5	-1.2	1.9	WWOX	Homo sapiens WW domain containing oxidoreductase (WWOX), transcript variant 2, mRNA [NM_130791]	51741

A_23_P206684	1.1	-7.2	-1.6	-1.2	WWP2	Homo sapiens WW domain containing E3 ubiquitin protein ligase 2 (WWP2), transcript variant 2, mRNA [NM_199424]	11060
A_24_P944383	-2.9	-1.0	-1.1	-1.1	WWTR1	Homo sapiens WW domain containing transcription regulator 1 (WWTR1), transcript variant 1, mRNA [NM_015472]	25937
A_32_P94866	1.1	1.0	-3.5	-1.1	WWTR1	WW domain containing transcription regulator 1 [Source:HGNC Symbol;Acc:24042] [ENST00000474080]	25937

A_32_P6868	3.0	3.0	3.0	1.7	WWTR1	Homo sapiens WW domain containing transcription regulator 1 (WWTR1), transcript variant 1, mRNA [NM_015472]	25937
A_24_P2093	-1.6	2.1	2.1	1.3	XAB2	Homo sapiens XPA binding protein 2 (XAB2), mRNA [NM_020196]	56949
A_23_P142013	-2.3	-3.4	-2.3	-2.3	XAB2	Homo sapiens XPA binding protein 2 (XAB2), mRNA [NM_020196]	56949
A_24_P557479	-2.7	1.0	-1.1	1.0	XAF1	Homo sapiens XIAP associated factor 1 (XAF1), transcript variant 1, mRNA [NM_017523]	54739

A_23_P4283	6.5	6.6	3.3	2.3	XAF1	Homo sapiens XIAP associated factor 1 (XAF1), transcript variant 1, mRNA [NM_017523]	54739
A_24_P315014	1.7	1.7	1.7	3.6	XAGE-4	PREDICTED: Homo sapiens XAGE-4 protein (XAGE-4), miscRNA [XR_113307]	139629
A_23_P120845	-1.7	-2.3	-1.2	-2.0	XBP1	Homo sapiens X-box binding protein 1 (XBP1), transcript variant 1, mRNA [NM_005080]	7494
A_23_P18246	1.2	1.1	-3.5	-7.6	XCR1	Homo sapiens chemokine (C motif) receptor 1 (XCR1), transcript variant 1, mRNA [NM_005283]	2829

A_32_P345659	3.0	-1.8	1.0	-1.4	XIRP1	Homo sapiens xin actin-binding repeat containing 1 (XIRP1), transcript variant 1, mRNA [NM_194293]	165904
A_32_P134007	-2.1	-3.9	-6.6	-2.4	XKR4	Homo sapiens XK, Kell blood group complex subunit-related family, member 4 (XKR4), mRNA [NM_052898]	114786
A_32_P233877	1.2	-3.8	1.2	1.1	XKR5	Homo sapiens XK, Kell blood group complex subunit-related family, member 5 (XKR5), mRNA [NM_207411]	389610

A_23_P60283	1.6	1.8	2.2	1.6	XPA	Homo sapiens xeroderma pigmentosum, complementation group A (XPA), transcript variant 1, mRNA [NM_000380]	7507
A_23_P83659	-1.1	2.1	-1.1	-1.2	XPNPEP2	Homo sapiens X-prolyl aminopeptidase (aminopeptidase P) 2, membrane-bound (XPNPEP2), mRNA [NM_003399]	7512

A_24_P923629	1.1	1.0	-2.3	1.1	XPNPEP3	Homo sapiens X-prolyl aminopeptidase (aminopeptidase P) 3, putative (XPNPEP3), nuclear gene encoding mitochondrial protein, transcript variant 1, mRNA [NM_022098]	63929
A_23_P218793	-1.3	-1.3	-1.6	2.4	XPNPEP3	Homo sapiens X-prolyl aminopeptidase (aminopeptidase P) 3, putative (XPNPEP3), nuclear gene encoding mitochondrial protein, transcript variant 1, mRNA [NM_022098]	63929

A_24_P333716	-2.1	1.6	1.4	1.3	XPO4	Homo sapiens exportin 4 (XPO4), mRNA [NM_022459]	64328
A_23_P139983	1.7	-2.2	1.8	1.1	XPO4	Homo sapiens exportin 4 (XPO4), mRNA [NM_022459]	64328
A_23_P146237	-2.3	1.0	1.1	1.0	XPO7	Homo sapiens exportin 7 (XPO7), mRNA [NM_015024]	23039
A_32_P13719	1.0	-1.0	-1.0	-2.4	XPOT	Homo sapiens exportin, tRNA (nuclear export receptor for tRNAs) (XPOT), mRNA [NM_007235]	11260
A_24_P702813	1.9	-16.6	-1.9	1.4	XPR1	Homo sapiens xenotropic and polytropic retrovirus receptor 1 (XPR1), transcript variant 1, mRNA [NM_004736]	9213

A_24_P336417	-1.5	-5.3	-2.0	1.1	XPR1	Homo sapiens xenotropic and polytropic retrovirus receptor 1 (XPR1), transcript variant 1, mRNA [NM_004736]	9213
A_23_P63534	1.0	1.6	47.6	-1.9	XPR1	Homo sapiens xenotropic and polytropic retrovirus receptor 1 (XPR1), transcript variant 1, mRNA [NM_004736]	9213
A_32_P77343	-1.3	-5.3	-3.4	-2.2	XRCC2	X-ray repair complementing defective repair in Chinese hamster cells 2 [Source:HGNC Symbol;Acc:12829] [ENST00000495707]	7516

A_23_P48470	1.6	-3.8	1.8	-1.6	XRCC3	Homo sapiens X-ray repair complementing defective repair in Chinese hamster cells 3 (XRCC3), transcript variant 2, mRNA [NM_005432]	7517
A_23_P120947	3.4	2.2	3.3	2.1	XRCC6	Homo sapiens X-ray repair complementing defective repair in Chinese hamster cells 6 (XRCC6), mRNA [NM_001469]	2547
A_24_P927404	-3.6	1.4	1.8	1.0	XRCC6BP1	Homo sapiens XRCC6 binding protein 1, mRNA (cDNA clone IMAGE:5742519), complete cds. [BC033881]	91419

A_23_P53363	-1.0	-1.0	-5.8	-1.0	XRCC6BP1	Homo sapiens XRCC6 binding protein 1 (XRCC6BP1), mRNA [NM_033276]	91419
A_23_P132822	1.9	2.5	-1.4	1.9	XRN1	Homo sapiens 5'-3' exoribonuclease 1 (XRN1), transcript variant 1, mRNA [NM_019001]	54464
A_23_P345710	1.4	4.4	-1.8	-1.9	XXYLT1	Homo sapiens xyloside xylosyltransferase 1 (XXYLT1), mRNA [NM_152531]	152002
A_23_P212423	-1.0	-3.0	-2.0	-1.0	XYLB	Homo sapiens xylulokinase homolog (H. influenzae) (XYLB), mRNA [NM_005108]	9942
A_23_P349321	1.1	1.1	-2.5	1.1	XYLT1	Homo sapiens xylosyltransferase I (XYLT1), mRNA [NM_022166]	64131

A_23_P15582	-3.0	-1.0	-1.1	-1.1	XYLT2	Homo sapiens xylosyltransferase II (XYLT2), mRNA [NM_022167]	64132
A_23_P139825	1.0	-1.0	-1.1	-3.3	YAF2	Homo sapiens YY1 associated factor 2 (YAF2), transcript variant 1, mRNA [NM_001190979]	10138
A_23_P379550	-5.4	-5.8	-9.8	-1.8	YARS	Homo sapiens tyrosyl-tRNA synthetase (YARS), mRNA [NM_003680]	8565
A_23_P151093	1.6	1.4	1.1	2.3	YARS2	Homo sapiens tyrosyl-tRNA synthetase 2, mitochondrial (YARS2), nuclear gene encoding mitochondrial protein, mRNA [NM_001040436]	51067

A_32_P18547	-1.0	2.1	-1.0	-1.0	YBEY	Homo sapiens ybeY metallopeptidase (putative) (YBEY), transcript variant 1, mRNA [NM_058181]	54059
A_32_P169754	-3.0	-3.1	-4.7	-2.1	YBX1	Homo sapiens Y box binding protein 1 (YBX1), mRNA [NM_004559]	4904
A_23_P49865	3.9	2.0	5.9	4.6	YBX2	Homo sapiens Y box binding protein 2 (YBX2), mRNA [NM_015982]	51087
A_24_P376159	-2.1	-1.2	1.2	2.8	YEATS2	Homo sapiens cDNA FLJ33401 fis, clone BRACE2009886. [AK090720]	55689

A_23_P164507	-4.9	-1.1	-1.2	-1.4	YES1	Homo sapiens v-yes-1 Yamaguchi sarcoma viral oncogene homolog 1 (YES1), mRNA [NM_005433]	7525
A_24_P48403	1.1	2.2	1.1	1.0	YES1	Homo sapiens v-yes-1 Yamaguchi sarcoma viral oncogene homolog 1 (YES1), mRNA [NM_005433]	7525
A_23_P142239	1.0	-1.1	-2.2	1.0	YIF1B	Homo sapiens Yip1 interacting factor homolog B (S. cerevisiae) (YIF1B), transcript variant 2, mRNA [NM_033557]	90522

A_24_P307248	2.2	1.8	1.9	-1.0	YIF1B	Homo sapiens Yip1 interacting factor homolog B (S. cerevisiae) (YIF1B), transcript variant 2, mRNA [NM_033557]	90522
A_23_P154006	-1.4	-2.4	-2.2	-3.7	YIPF4	Yip1 domain family, member 4 [Source:HGNC Symbol;Acc:28145] [ENST00000238831]	84272
A_32_P488818	-2.4	-1.3	-1.3	-1.3	YIPF7	Homo sapiens Yip1 domain family, member 7 (YIPF7), mRNA [NM_182592]	285525
A_24_P119374	-1.0	-2.5	-1.4	1.0	YLPM1	Homo sapiens YLP motif containing 1 (YLPM1), mRNA [NM_019589]	56252

A_32_P69803	-10.5	-1.5	-1.2	-1.2	YOD1	Homo sapiens YOD1 OTU deubiquinating enzyme 1 homolog (S. cerevisiae) (YOD1), mRNA [NM_018566]	55432
A_32_P88120	4.2	3.1	1.8	6.1	YPEL1	Homo sapiens yippee-like 1 (Drosophila) (YPEL1), mRNA [NM_013313]	29799
A_24_P787947	-1.7	-5.2	-2.3	-1.8	YPEL2	Homo sapiens yippee-like 2 (Drosophila) (YPEL2), mRNA [NM_001005404]	388403
A_23_P15108	1.1	1.3	1.4	-16.1	YPEL3	Homo sapiens yippee-like 3 (Drosophila) (YPEL3), transcript variant 1, mRNA [NM_031477]	83719

A_23_P300220	-1.5	-1.4	2.1	1.0	YPEL4	Homo sapiens yippee-like 4 (Drosophila) (YPEL4), mRNA [NM_145008]	219539
A_24_P932875	-4.8	-1.0	-3.8	-1.2	YPLR6490	PREDICTED: Homo sapiens hypothetical LOC389102 (YPLR6490), miscRNA [XR_110047]	389102
A_23_P500251	-1.3	-1.1	-1.1	-8.0	YWHAB	Homo sapiens tyrosine 3-monooxygenase/tryptophan 5-monooxygenase activation protein, beta polypeptide (YWHAB), transcript variant 1, mRNA [NM_003404]	7529

A_24_P33444	1.0	-1.0	3.6	-4.2	YWHAE	Homo sapiens tyrosine 3-monooxygenase/tryptophan 5-monooxygenase activation protein, epsilon polypeptide (YWHAE), transcript variant 1, mRNA [NM_006761]	7531
A_24_P123012	-1.1	1.4	-2.5	2.5	YWHAE	Homo sapiens tyrosine 3-monooxygenase/tryptophan 5-monooxygenase activation protein, epsilon polypeptide (YWHAE), transcript variant 1, mRNA [NM_006761]	7531

A_24_P209571	-1.7	-1.6	-1.2	-2.9	YWHAZ	Homo sapiens tyrosine 3-monooxygenase/tryptophan 5-monooxygenase activation protein, zeta polypeptide (YWHAZ), transcript variant 2, mRNA [NM_145690]	7534
A_32_P226149	1.7	3.6	3.4	1.1	YWHAZ	Homo sapiens tyrosine 3-monooxygenase/tryptophan 5-monooxygenase activation protein, zeta polypeptide (YWHAZ), transcript variant 2, mRNA [NM_145690]	7534

A_24_P492562	1.2	1.0	-1.7	-5.6	YWHAZ	Homo sapiens tyrosine 3-monooxygenase/tryptophan 5-monooxygenase activation protein, zeta polypeptide (YWHAZ), transcript variant 2, mRNA [NM_145690]	7534
A_23_P349398	-1.4	-2.3	1.4	-1.8	ZAR1	Homo sapiens zygote arrest 1 (ZAR1), mRNA [NM_175619]	326340
A_23_P61031	-2.3	1.2	1.1	-1.3	ZBBX	Homo sapiens zinc finger, B-box domain containing (ZBBX), transcript variant 2, mRNA [NM_024687]	79740

A_32_P86623	-10.3	-1.0	-2.8	-1.1	ZBED1	Homo sapiens zinc finger, BED-type containing 1 (ZBED1), transcript variant 2, mRNA [NM_004729]	9189
A_23_P93032	1.3	-1.0	1.0	-2.6	ZBED3	Homo sapiens zinc finger, BED-type containing 3 (ZBED3), mRNA [NM_032367]	84327
A_23_P98483	1.4	2.5	-1.2	2.2	ZBED5	Homo sapiens zinc finger, BED-type containing 5 (ZBED5), transcript variant 1, mRNA [NM_021211]	58486
A_24_P920319	3.7	3.6	3.7	3.6	ZBED5	Homo sapiens transposon-derived Buster1 transposase-like protein mRNA, complete cds. [AF205600]	58486

A_24_P395966	2.9	1.7	3.0	1.6	ZBP1	Homo sapiens Z-DNA binding protein 1 (ZBP1), transcript variant 1, mRNA [NM_030776]	81030
A_24_P64918	-1.0	-15.2	1.0	-1.0	ZBTB1	Homo sapiens zinc finger and BTB domain containing 1 (ZBTB1), transcript variant 2, mRNA [NM_014950]	22890
A_24_P64071	1.1	-2.1	-1.1	-1.1	ZBTB10	Homo sapiens zinc finger and BTB domain containing 10 (ZBTB10), transcript variant 2, mRNA [NM_023929]	65986

A_23_P385114	2.1	2.1	2.2	1.0	ZBTB10	Homo sapiens zinc finger and BTB domain containing 10 (ZBTB10), transcript variant 2, mRNA [NM_023929]	65986
A_23_P109774	1.0	-2.1	-1.9	-1.1	ZBTB11	Homo sapiens zinc finger and BTB domain containing 11 (ZBTB11), mRNA [NM_014415]	27107
A_23_P104804	1.1	1.0	1.7	-2.0	ZBTB16	Homo sapiens zinc finger and BTB domain containing 16 (ZBTB16), transcript variant 1, mRNA [NM_006006]	7704

A_23_P654	1.5	1.2	1.3	2.3	ZBTB17	Homo sapiens zinc finger and BTB domain containing 17 (ZBTB17), transcript variant 2, mRNA [NM_003443]	7709
A_32_P179526	-4.3	1.7	1.6	1.3	ZBTB20	zinc finger and BTB domain containing 20 [Source:HGNC Symbol;Acc:13503] [ENST00000488663]	26137
A_24_P728215	-1.3	-31.3	-1.4	-1.6	ZBTB20-AS1	Homo sapiens ZBTB20 antisense RNA 1 (non-protein coding) (ZBTB20-AS1), non-coding RNA [NR_038993]	100131117

A_23_P111188	1.8	-5.3	1.4	1.0	ZBTB22	Homo sapiens zinc finger and BTB domain containing 22 (ZBTB22), transcript variant 1, mRNA [NM_005453]	9278
A_23_P134147	-2.5	-1.8	-1.6	-2.0	ZBTB24	Homo sapiens zinc finger and BTB domain containing 24 (ZBTB24), transcript variant 1, mRNA [NM_014797]	9841
A_23_P96483	2.4	3.9	4.0	-1.2	ZBTB33	Homo sapiens zinc finger and BTB domain containing 33 (ZBTB33), transcript variant 2, mRNA [NM_006777]	10009

A_23_P212025	4.7	4.9	4.0	2.7	ZBTB38	Homo sapiens zinc finger and BTB domain containing 38 (ZBTB38), mRNA [NM_001080412]	253461
A_24_P221323	-1.2	-1.2	-1.5	-2.1	ZBTB38	Homo sapiens zinc finger and BTB domain containing 38 (ZBTB38), mRNA [NM_001080412]	253461
A_23_P13683	1.2	-3.8	-1.8	1.1	ZBTB39	Homo sapiens zinc finger and BTB domain containing 39 (ZBTB39), mRNA [NM_014830]	9880
A_23_P160136	3.7	2.4	3.5	3.8	ZBTB40	Homo sapiens zinc finger and BTB domain containing 40 (ZBTB40), transcript variant 2, mRNA [NM_014870]	9923

A_23_P113328	-1.2	1.3	-1.6	2.1	ZBTB41	Homo sapiens zinc finger and BTB domain containing 41 (ZBTB41), mRNA [NM_194314]	360023
A_23_P135315	-2.0	-1.1	-1.6	3.2	ZBTB43	Homo sapiens zinc finger and BTB domain containing 43 (ZBTB43), transcript variant 1, mRNA [NM_014007]	23099
A_23_P385826	1.1	1.0	-1.0	-2.5	ZBTB44	zinc finger and BTB domain containing 44 [Source:HGNC Symbol;Acc:25001] [ENST00000529348]	29068
A_23_P202945	-2.2	1.1	2.8	3.2	ZBTB44	Homo sapiens zinc finger and BTB domain containing 44 (ZBTB44), mRNA [NM_014155]	29068

A_32_P175183	-1.0	2.6	2.7	1.7	ZBTB44	Homo sapiens zinc finger and BTB domain containing 44 (ZBTB44), mRNA [NM_014155]	29068
A_23_P421638	-1.1	-2.7	-1.2	-1.3	ZBTB47	Homo sapiens zinc finger and BTB domain containing 47 (ZBTB47), mRNA [NM_145166]	92999
A_23_P216476	4.1	2.6	2.2	-1.0	ZBTB5	Homo sapiens zinc finger and BTB domain containing 5 (ZBTB5), mRNA [NM_014872]	9925
A_23_P27827	-1.3	-1.1	-3.0	1.2	ZBTB7A	Homo sapiens zinc finger and BTB domain containing 7A (ZBTB7A), mRNA [NM_015898]	51341

A_24_P19884	-1.2	2.0	1.8	1.2	ZBTB7B	Homo sapiens zinc finger and BTB domain containing 7B (ZBTB7B), mRNA [NM_015872]	51043
A_23_P11729	-3.7	-3.9	-4.1	-3.9	ZBTB7B	Homo sapiens zinc finger and BTB domain containing 7B (ZBTB7B), mRNA [NM_015872]	51043
A_23_P345973	2.7	1.7	1.7	-6.4	ZBTB7C	Homo sapiens zinc finger and BTB domain containing 7C (ZBTB7C), mRNA [NM_001039360]	201501
A_23_P139455	1.1	-4.5	-2.0	-1.7	ZC3H10	Homo sapiens zinc finger CCCH-type containing 10 (ZC3H10), mRNA [NM_032786]	84872

A_23_P255194	1.4	-1.9	-2.0	1.4	ZC3H11A	Homo sapiens zinc finger CCCH-type containing 11A (ZC3H11A), mRNA [NM_014827]	9877
A_24_P19993	1.5	-2.0	-2.2	-2.2	ZC3H11A	Homo sapiens zinc finger CCCH-type containing 11A (ZC3H11A), mRNA [NM_014827]	9877
A_32_P221076	1.3	-1.1	-2.1	1.0	ZC3H11A	Homo sapiens zinc finger CCCH-type containing 11A (ZC3H11A), mRNA [NM_014827]	9877
A_23_P388993	-1.0	-1.0	-1.0	-5.0	ZC3H12C	Homo sapiens zinc finger CCCH-type containing 12C (ZC3H12C), mRNA [NM_033390]	85463

A_32_P65533	-1.2	-1.1	-8.9	-1.2	ZC3H12D	Homo sapiens zinc finger CCCH-type containing 12D (ZC3H12D), mRNA [NM_207360]	340152
A_24_P109176	-7.8	1.1	1.0	1.1	ZC3H12D	Homo sapiens zinc finger CCCH-type containing 12D (ZC3H12D), mRNA [NM_207360]	340152
A_24_P187826	1.0	-1.0	-2.2	-1.1	ZC3H12D	Homo sapiens zinc finger CCCH-type containing 12D (ZC3H12D), mRNA [NM_207360]	340152
A_24_P232763	1.5	-1.9	-3.4	1.0	ZC3H13	zinc finger CCCH-type containing 13 [Source:HGNC Symbol;Acc:20368] [ENST00000242848]	23091

A_24_P241330	-1.6	-1.7	-1.5	-3.2	ZC3H14	Homo sapiens zinc finger CCCH-type containing 14 (ZC3H14), transcript variant 1, mRNA [NM_024824]	79882
A_23_P3502	1.4	2.2	1.6	1.5	ZC3H18	Homo sapiens zinc finger CCCH-type containing 18 (ZC3H18), mRNA [NM_144604]	124245
A_23_P9036	-2.1	1.6	-1.4	-2.4	ZC3H3	Homo sapiens zinc finger CCCH-type containing 3 (ZC3H3), mRNA [NM_015117]	23144
A_24_P220618	-2.2	-3.9	-2.1	-1.2	ZC3H4	Homo sapiens zinc finger CCCH-type containing 4 (ZC3H4), mRNA [NM_015168]	23211

A_24_P826348	-7.4	-2.0	-1.0	1.0	ZC3H6	Homo sapiens zinc finger CCCH-type containing 6 (ZC3H6), mRNA [NM_198581]	376940
A_23_P26336	1.0	1.0	1.1	-38.1	ZC3H7A	Homo sapiens zinc finger CCCH-type containing 7A (ZC3H7A), mRNA [NM_014153]	29066
A_24_P119283	4.3	2.9	4.3	2.0	ZC3H7A	Homo sapiens zinc finger CCCH-type containing 7A (ZC3H7A), mRNA [NM_014153]	29066
A_23_P218807	-2.6	1.0	-2.9	1.1	ZC3H7B	Homo sapiens zinc finger CCCH-type containing 7B (ZC3H7B), mRNA [NM_017590]	23264

A_24_P797678	1.0	-2.2	-2.1	-2.9	ZC3H7B	Homo sapiens zinc finger CCCH-type containing 7B, mRNA (cDNA clone IMAGE:3690478), partial cds. [BC004857]	23264
A_23_P20122	1.8	4.7	1.8	1.7	ZC3HAV1	Homo sapiens zinc finger CCCH-type, antiviral 1 (ZC3HAV1), transcript variant 2, mRNA [NM_024625]	56829
A_24_P923142	-8.6	-3.7	-3.6	-3.9	ZC3HAV1L	Homo sapiens cDNA FLJ40837 fis, clone TRACH2013495. [AK098156]	92092
A_23_P31532	-1.1	2.5	-1.1	1.8	ZC3HAV1L	Homo sapiens zinc finger CCCH-type, antiviral 1-like (ZC3HAV1L), mRNA [NM_080660]	92092

A_23_P215088	-2.5	1.0	1.0	1.1	ZC3HC1	Homo sapiens zinc finger, C3HC-type containing 1 (ZC3HC1), mRNA [NM_016478]	51530
A_32_P11096	-2.4	-2.0	-1.1	-2.1	ZCCHC13	Homo sapiens zinc finger, CCHC domain containing 13 (ZCCHC13), mRNA [NM_203303]	389874
A_23_P100420	1.1	-2.5	-4.2	1.1	ZCCHC14	Homo sapiens zinc finger, CCHC domain containing 14 (ZCCHC14), mRNA [NM_015144]	23174
A_24_P176484	1.6	1.2	-2.3	1.9	ZCCHC14	Homo sapiens zinc finger, CCHC domain containing 14 (ZCCHC14), mRNA [NM_015144]	23174

A_24_P88505	9.0	8.9	8.6	8.8	ZCCHC17	Homo sapiens zinc finger, CCHC domain containing 17 (ZCCHC17), mRNA [NM_016505]	51538
A_32_P1509	1.0	2.4	-1.1	-1.1	ZCCHC17	Homo sapiens zinc finger, CCHC domain containing 17 (ZCCHC17), mRNA [NM_016505]	51538
A_23_P335452	-3.2	1.2	2.5	1.0	ZCCHC24	Homo sapiens zinc finger, CCHC domain containing 24 (ZCCHC24), mRNA [NM_153367]	219654
A_24_P399694	3.0	2.9	2.9	2.2	ZCCHC3	Homo sapiens zinc finger, CCHC domain containing 3 (ZCCHC3), mRNA [NM_033089]	85364

A_24_P248863	1.0	-9.3	-1.1	-1.0	ZCCHC4	Homo sapiens zinc finger, CCHC domain containing 4 (ZCCHC4), mRNA [NM_024936]	29063
A_23_P155807	-2.5	1.0	-1.0	-1.0	ZCCHC4	Homo sapiens zinc finger, CCHC domain containing 4 (ZCCHC4), mRNA [NM_024936]	29063
A_23_P411353	4.9	2.3	2.3	3.1	ZCCHC5	Homo sapiens zinc finger, CCHC domain containing 5 (ZCCHC5), mRNA [NM_152694]	203430

A_24_P68275	-3.1	3.8	2.0	2.5	ZCCHC6	Homo sapiens zinc finger, CCHC domain containing 6 (ZCCHC6), transcript variant 1, mRNA [NM_024617]	79670
A_23_P123727	1.0	-2.2	-1.0	-1.1	ZCCHC6	Homo sapiens zinc finger, CCHC domain containing 6 (ZCCHC6), transcript variant 1, mRNA [NM_024617]	79670
A_24_P85026	-8.2	1.0	1.0	-1.0	ZCCHC7	Homo sapiens zinc finger, CCHC domain containing 7 (ZCCHC7), mRNA [NM_032226]	84186

A_23_P64669	-1.0	-1.1	-1.1	-2.3	ZCCHC8	Homo sapiens zinc finger, CCHC domain containing 8 (ZCCHC8), mRNA [NM_017612]	55596
A_23_P70897	-1.0	-2.1	-1.1	-1.0	ZCWPW1	Homo sapiens zinc finger, CW type with PWWP domain 1 (ZCWPW1), mRNA [NM_017984]	55063
A_23_P66038	-2.5	-2.9	-1.3	-1.5	ZDHHC1	Homo sapiens zinc finger, DHHC-type containing 1 (ZDHHC1), mRNA [NM_013304]	29800
A_23_P158007	1.5	1.2	-1.1	2.2	ZDHHC12	Homo sapiens zinc finger, DHHC-type containing 12 (ZDHHC12), mRNA [NM_032799]	84885

A_24_P269129	-2.1	-1.8	-1.8	-1.7	ZDHHC14	Homo sapiens zinc finger, DHHC-type containing 14 (ZDHHC14), transcript variant 2, mRNA [NM_1537 46]	79683
A_24_P934235	1.0	-1.0	-3.0	-1.1	ZDHHC15	Homo sapiens zinc finger, DHHC-type containing 15 (ZDHHC15), transcript variant 3, mRNA [NM_0011 46257]	158866
A_23_P150009	-1.7	-1.6	-6.8	-3.0	ZDHHC16	Homo sapiens zinc finger, DHHC-type containing 16 (ZDHHC16), transcript variant 5, mRNA [NM_1980 46]	84287

A_32_P95949	-1.7	-1.0	-1.0	3.1	ZDHHC17	Homo sapiens zinc finger, DHHC-type containing 17 (ZDHHC17), mRNA [NM_015336]	23390
A_23_P314789	1.2	-1.3	-4.6	-1.2	ZDHHC19	Homo sapiens zinc finger, DHHC-type containing 19 (ZDHHC19), mRNA [NM_001039617]	131540
A_24_P155603	-2.7	1.0	1.1	1.0	ZDHHC20	Homo sapiens zinc finger, DHHC-type containing 20 (ZDHHC20), mRNA [NM_153251]	253832
A_24_P933492	-1.3	-1.3	-8.5	-1.6	ZDHHC21	Homo sapiens zinc finger, DHHC-type containing 21 (ZDHHC21), mRNA [NM_178566]	340481

A_24_P99175	17.0	41.5	44.6	44.4	ZDHHC22	Homo sapiens zinc finger, DHHC-type containing 22 (ZDHHC22), mRNA [NM_174976]	283576
A_32_P75243	4.4	4.4	4.0	4.4	ZDHHC22	zinc finger, DHHC-type containing 22 [Source:HGNC Symbol;Acc:20106] [ENST00000555327]	283576
A_23_P355447	1.5	1.2	-3.4	1.4	ZDHHC22	Homo sapiens zinc finger, DHHC-type containing 22 (ZDHHC22), mRNA [NM_174976]	283576

A_24_P259276	-1.8	-2.5	-1.1	-1.3	ZDHHC24	Homo sapiens zinc finger, DHHC-type containing 24 (ZDHHC24), mRNA [NM_207340]	254359
A_24_P342880	1.2	-1.4	-2.1	-1.2	ZDHHC3	Homo sapiens zinc finger, DHHC-type containing 3 (ZDHHC3), transcript variant 2, mRNA [NM_016598]	51304
A_24_P351420	1.2	1.1	-11.3	1.0	ZDHHC3	Homo sapiens zinc finger, DHHC-type containing 3 (ZDHHC3), transcript variant 2, mRNA [NM_016598]	51304

A_23_P157147	3.6	3.5	3.1	3.1	ZDHHC4	Homo sapiens zinc finger, DHHC-type containing 4 (ZDHHC4), transcript variant 3, mRNA [NM_018106]	55146
A_23_P98532	-1.4	-5.6	1.9	1.0	ZDHHC5	Homo sapiens zinc finger, DHHC-type containing 5 (ZDHHC5), mRNA [NM_015457]	25921
A_23_P142560	-1.5	-1.3	-2.1	-1.1	ZEB2	Homo sapiens zinc finger E-box binding homeobox 2 (ZEB2), transcript variant 1, mRNA [NM_014795]	9839

A_23_P407203	-5.0	1.7	-1.5	1.6	ZEB2	Homo sapiens zinc finger E-box binding homeobox 2 (ZEB2), transcript variant 3, non-coding RNA [NR_033258]	9839
A_24_P170826	-2.2	-1.7	-1.8	-1.7	ZER1	Homo sapiens zer-1 homolog (C. elegans) (ZER1), mRNA [NM_006336]	10444
A_23_P122375	2.3	-1.1	1.4	-1.3	ZFAND3	Homo sapiens zinc finger, AN1-type domain 3 (ZFAND3), mRNA [NM_021943]	60685
A_23_P1170	-2.4	-1.8	-5.2	-2.2	ZFAND4	Homo sapiens AN1, ubiquitin-like, homolog (Xenopus laevis) (ANUBL1), transcript variant 1, mRNA [NM_174890]	93550

A_24_P278172	-1.5	-2.2	-2.0	-1.3	ZFAND5	Homo sapiens zinc finger, AN1-type domain 5 (ZFAND5), transcript variant c, mRNA [NM_006007]	7763
A_32_P109817	2.9	4.8	2.0	2.5	ZFAND5	Homo sapiens zinc finger, AN1-type domain 5 (ZFAND5), transcript variant c, mRNA [NM_006007]	7763
A_32_P109922	-12.5	1.1	-1.0	1.0	ZFAT	Homo sapiens zinc finger and AT hook domain containing (ZFAT), transcript variant 1, mRNA [NM_020863]	57623
A_23_P332908	-2.3	-1.1	-1.1	-3.7	ZFC3H1	Homo sapiens zinc finger, C3H1-type containing (ZFC3H1), mRNA [NM_144982]	196441

A_23_P14369	1.6	1.5	1.5	-2.2	ZFHX2	zinc finger homeobox 2 [Source:HGNC Symbol;Acc:20152] [ENST00000382785]	85446
A_32_P226205	1.2	2.7	-1.2	2.0	ZFHX2	Homo sapiens zinc finger homeobox 2 (ZFHX2), mRNA [NM_033400]	85446
A_23_P77310	1.4	1.4	1.8	2.6	ZFP106	Homo sapiens zinc finger protein 106 homolog (mouse) (ZFP106), mRNA [NM_022473]	64397
A_24_P151106	-3.2	1.0	-1.1	1.0	ZFP28	Homo sapiens zinc finger protein 28 homolog (mouse) (ZFP28), mRNA [NM_020828]	140612

A_23_P107673	-2.9	-1.6	-3.4	-4.6	ZFP28	Homo sapiens zinc finger protein 28 homolog (mouse) (ZFP28), mRNA [NM_020828]	140612
A_23_P119732	-2.3	1.1	1.1	-1.9	ZFP30	Homo sapiens zinc finger protein 30 homolog (mouse) (ZFP30), mRNA [NM_014898]	22835
A_23_P39237	-1.1	1.4	-2.3	-6.0	ZFP36	Homo sapiens zinc finger protein 36, C3H type, homolog (mouse) (ZFP36), mRNA [NM_003407]	7538
A_23_P406071	-1.1	1.7	1.5	-2.2	ZFP36L1	zinc finger protein 36, C3H type-like 1 [Source:HGNC Symbol;Acc:1107] [ENST00000408913]	677

A_24_P99152	2.4	-1.0	-1.9	-1.1	ZFP36L1	Homo sapiens zinc finger protein 36, C3H type-like 1 (ZFP36L1), transcript variant 1, mRNA [NM_004926]	677
A_23_P346357	3.2	2.5	4.4	3.9	ZFP41	Homo sapiens zinc finger protein 41 homolog (mouse) (ZFP41), mRNA [NM_173832]	286128
A_23_P69877	-1.8	1.0	1.0	-4.7	ZFP62	Homo sapiens zinc finger protein 62 homolog (mouse) (ZFP62), transcript variant 1, mRNA [NM_152283]	643836
A_24_P176409	-1.0	-5.2	-1.9	-1.2	ZFP90	Homo sapiens zinc finger protein 90 homolog (mouse) (ZFP90), mRNA [NM_133458]	146198

A_23_P168909	-1.1	-1.1	-6.2	-1.2	ZFPM2	Homo sapiens zinc finger protein, multitype 2 (ZFPM2), mRNA [NM_012082]	23414
A_24_P273857	1.1	1.1	1.0	-4.0	ZFPM2	Homo sapiens zinc finger protein, multitype 2 (ZFPM2), mRNA [NM_012082]	23414
A_23_P41818	3.9	3.9	4.0	4.7	ZFR	Homo sapiens zinc finger RNA binding protein (ZFR), mRNA [NM_016107]	51663
A_32_P55462	6.2	6.0	2.0	2.5	ZFR2	Homo sapiens zinc finger RNA binding protein 2 (ZFR2), transcript variant 2, mRNA [NM_001145640]	23217

A_23_P125639	4.9	3.6	1.4	2.3	ZFX	Homo sapiens zinc finger protein, X-linked (ZFX), transcript variant 1, mRNA [NM_003410]	7543
A_24_P290527	1.0	2.6	-1.3	1.5	ZFX	Homo sapiens zinc finger protein, X-linked (ZFX), transcript variant 1, mRNA [NM_003410]	7543
A_23_P65930	-1.5	-1.4	-1.7	-79.9	ZFYVE19	Homo sapiens zinc finger, FYVE domain containing 19 (ZFYVE19), mRNA [NM_001077268]	84936
A_23_P315872	1.6	2.2	1.9	1.9	ZFYVE20	Homo sapiens zinc finger, FYVE domain containing 20 (ZFYVE20), mRNA [NM_022340]	64145

A_24_P85322	1.7	-9.1	1.7	1.1	ZFYVE26	Homo sapiens zinc finger, FYVE domain containing 26, mRNA (cDNA clone IMAGE:3836316), partial cds. [BC008927]	23503
A_32_P104746	2.6	2.6	2.3	2.4	ZFYVE28	Homo sapiens zinc finger, FYVE domain containing 28 (ZFYVE28), transcript variant 2, mRNA [NM_020972]	57732
A_23_P302914	1.1	1.0	-1.0	-5.4	ZFYVE28	Homo sapiens zinc finger, FYVE domain containing 28 (ZFYVE28), transcript variant 2, mRNA [NM_020972]	57732

A_23_P49145	1.6	-1.2	2.0	1.1	ZG16	Homo sapiens zymogen granule protein 16 homolog (rat) (ZG16), mRNA [NM_152338]	653808
A_23_P218654	3.1	2.6	2.1	2.0	ZGPAT	Homo sapiens zinc finger, CCCH-type with G patch domain (ZGPAT), transcript variant 1, mRNA [NM_032527]	84619
A_24_P57700	14.8	12.1	2.7	12.0	ZHX3	Homo sapiens zinc fingers and homeoboxes 3 (ZHX3), mRNA [NM_015035]	23051
A_24_P217306	-3.0	-1.2	-1.6	-1.7	ZIC4	Homo sapiens Zic family member 4 (ZIC4), transcript variant 3, mRNA [NM_032153]	84107

A_24_P270345	-2.2	1.1	2.0	1.1	ZIC5	Homo sapiens Zic family member 5 (ZIC5), mRNA [NM_033132]	85416
A_32_P219942	1.0	1.0	2.2	1.0	ZIC5	Homo sapiens Zic family member 5 (ZIC5), mRNA [NM_033132]	85416
A_23_P344281	1.2	1.2	3.8	1.0	ZIK1	Homo sapiens zinc finger protein interacting with K protein 1 homolog (mouse) (ZIK1), mRNA [NM_001010879]	284307
A_23_P94063	-1.0	-4.6	2.2	-1.0	ZKSCAN1	zinc finger with KRAB and SCAN domains 1 [Source:HGNC Symbol;Acc:13101] [ENST00000482979]	7586

A_23_P168019	1.0	1.1	-11.9	-3.9	ZKSCAN3	Homo sapiens zinc finger with KRAB and SCAN domains 3 (ZKSCAN3), transcript variant 2, mRNA [NM_024493]	80317
A_24_P930314	1.4	-1.0	-2.5	-1.1	ZKSCAN4	Homo sapiens cDNA FLJ32136 fis, clone PEBLM2000395, moderately similar to ZINC FINGER PROTEIN 165. [AK056698]	387032
A_32_P164593	1.0	1.0	-3.9	-1.0	ZMAT4	Homo sapiens zinc finger, matrin-type 4 (ZMAT4), transcript variant 1, mRNA [NM_024645]	79698

A_23_P314276	-2.3	-1.5	1.7	1.1	ZMIZ1	zinc finger, MIZ-type containing 1 [Source:HGNC Symbol;Acc:16493] [ENST00000478357]	57178
A_23_P99405	-1.2	1.8	-2.4	1.6	ZMYM2	Homo sapiens zinc finger, MYM-type 2 (ZMYM2), transcript variant 1, mRNA [NM_003453]	7750
A_23_P137073	1.3	1.2	-2.9	1.1	ZMYM3	Homo sapiens zinc finger, MYM-type 3 (ZMYM3), transcript variant 2, mRNA [NM_005096]	9203
A_24_P16815	-4.1	-6.9	-4.9	-4.5	ZMYM3	Homo sapiens zinc finger, MYM-type 3 (ZMYM3), transcript variant 2, mRNA [NM_005096]	9203

A_32_P141257	2.8	2.7	2.4	1.9	ZMYM5	Homo sapiens zinc finger, MYM-type 5 (ZMYM5), transcript variant 3, mRNA [NM_001142684]	9205
A_24_P321634	-3.7	-1.2	3.8	-1.2	ZMYM5	Homo sapiens zinc finger, MYM-type 5 (ZMYM5), transcript variant 1, mRNA [NM_001039650]	9205
A_24_P110213	-1.0	-8.1	-2.3	-1.0	ZMYM5	Homo sapiens zinc finger, MYM-type 5 (ZMYM5), transcript variant 3, mRNA [NM_001142684]	9205
A_23_P29663	1.4	1.2	-4.8	-1.7	ZMYND10	Homo sapiens zinc finger, MYND-type containing 10 (ZMYND10), mRNA [NM_015896]	51364

A_24_P201323	1.2	3.1	1.6	-1.2	ZMYND11	Homo sapiens zinc finger, MYND-type containing 11 (ZMYND11), transcript variant 1, mRNA [NM_006624]	10771
A_24_P158385	3.3	1.7	3.3	2.7	ZMYND19	Homo sapiens zinc finger, MYND-type containing 19 (ZMYND19), mRNA [NM_138462]	116225
A_24_P926432	-1.0	-1.0	-2.8	1.6	ZMYND8	zinc finger, MYND-type containing 8 [Source:HGNC Symbol;Acc:9397] [ENST00000311275]	23613

A_24_P151325	2.2	2.0	1.3	12.0	ZNF100	Homo sapiens zinc finger protein 100 (ZNF100), mRNA [NM_173531]	163227
A_23_P215806	-1.8	-55.6	-4.2	-6.1	ZNF117	Homo sapiens zinc finger protein 117 (ZNF117), mRNA [NM_015852]	51351
A_23_P31335	-1.7	-1.4	-2.2	1.1	ZNF12	Homo sapiens zinc finger protein 12 (ZNF12), transcript variant 1, mRNA [NM_016265]	7559
A_24_P337774	1.1	-2.1	-1.1	-1.1	ZNF12	Homo sapiens zinc finger protein 12 (ZNF12), transcript variant 1, mRNA [NM_016265]	7559

A_32_P875758	1.2	1.2	1.2	-2.9	ZNF124	Homo sapiens zinc finger protein 124 (ZNF124), transcript variant 2, mRNA [NM_001243740]	7678
A_23_P325661	-1.1	-1.1	-3.3	-1.2	ZNF134	Homo sapiens zinc finger protein 134 (ZNF134), mRNA [NM_003435]	7693
A_23_P147121	-1.2	-1.2	-1.3	-4.6	ZNF136	Homo sapiens zinc finger protein 136 (ZNF136), mRNA [NM_003437]	7695
A_23_P67312	1.4	-4.3	1.4	5.6	ZNF136	Homo sapiens zinc finger protein 136 (ZNF136), mRNA [NM_003437]	7695
A_23_P388867	2.2	-2.8	1.6	2.2	ZNF141	Human zinc finger protein (ZNF141) mRNA, complete cds. [L15309]	7700

A_24_P402836	38.8	63.4	10.5	35.3	ZNF141	Homo sapiens zinc finger protein 141 (ZNF141), mRNA [NM_003441]	7700
A_23_P51051	1.1	-1.7	-2.3	-1.0	ZNF142	Homo sapiens zinc finger protein 142 (ZNF142), mRNA [NM_001105537]	7701
A_24_P206736	1.5	-8.1	1.8	-1.5	ZNF143	Homo sapiens zinc finger protein 143 (ZNF143), mRNA [NM_003442]	7702
A_24_P159845	5.2	3.7	2.0	3.5	ZNF146	Homo sapiens zinc finger protein 146 (ZNF146), transcript variant 1, mRNA [NM_007145]	7705
A_32_P131050	5.8	1.8	6.6	3.5	ZNF148	Homo sapiens zinc finger protein 148 (ZNF148), mRNA [NM_021964]	7707

A_23_P84910	2.3	8.9	6.5	3.6	ZNF157	Homo sapiens zinc finger protein 157 (ZNF157), mRNA [NM_003446]	7712
A_23_P168864	-1.6	1.2	3.7	2.4	ZNF16	Homo sapiens zinc finger protein 16 (ZNF16), transcript variant 2, mRNA [NM_001029976]	7564
A_24_P401241	-2.6	1.1	1.3	1.6	ZNF160	Homo sapiens zinc finger protein 160 (ZNF160), transcript variant 2, mRNA [NM_198893]	90338
A_24_P16950	2.8	2.9	18.7	-1.7	ZNF160	Homo sapiens zinc finger protein 160 (ZNF160), transcript variant 2, mRNA [NM_198893]	90338

A_24_P409451	2.9	2.2	2.1	-4.3	ZNF167	Homo sapiens zinc finger protein 167 (ZNF167), transcript variant 2, mRNA [NM_025169]	55888
A_23_P164706	1.9	-1.5	-3.0	1.0	ZNF177	Homo sapiens zinc finger protein 177 (ZNF177), transcript variant 3, mRNA [NM_003451]	7730
A_23_P89921	3.5	3.3	3.1	1.1	ZNF180	Homo sapiens zinc finger protein 180 (ZNF180), mRNA [NM_013256]	7733
A_23_P50735	-1.2	-1.1	-1.3	-5.3	ZNF181	Homo sapiens zinc finger protein 181 (ZNF181), transcript variant 1, mRNA [NM_001029997]	339318

A_23_P11025	1.5	1.6	3.8	1.1	ZNF185	Homo sapiens zinc finger protein 185 (LIM domain) (ZNF185), transcript variant 1, mRNA [NM_001178106]	7739
A_23_P145244	-4.4	-1.1	-1.2	-1.1	ZNF187	Homo sapiens zinc finger protein 187 (ZNF187), transcript variant a, mRNA [NM_001023560]	7741
A_23_P500905	-2.7	1.1	1.2	-1.4	ZNF19	Homo sapiens zinc finger protein 19 (ZNF19), mRNA [NM_006961]	7567
A_23_P214529	-1.6	-1.0	-2.1	1.1	ZNF192	Homo sapiens zinc finger protein 192 (ZNF192), mRNA [NM_006298]	7745

A_23_P122464	2.6	2.7	1.6	2.2	ZNF193	Homo sapiens zinc finger protein 193 (ZNF193), transcript variant 2, mRNA [NM_006299]	7746
A_24_P385148	-3.3	-1.5	-35.1	-1.7	ZNF197	Homo sapiens zinc finger protein 197 (ZNF197), transcript variant 1, mRNA [NM_006991]	10168
A_24_P28845	-1.5	-9.6	-1.6	1.1	ZNF2	Homo sapiens zinc finger protein 2 (ZNF2), transcript variant 1, mRNA [NM_021088]	7549
A_23_P152356	2.6	2.5	2.4	2.0	ZNF200	Homo sapiens zinc finger protein 200 (ZNF200), transcript variant 1, mRNA [NM_003454]	7752

A_24_P56689	2.8	-2.1	2.3	1.4	ZNF205	Homo sapiens zinc finger protein 205 (ZNF205), transcript variant 1, mRNA [NM_003456]	7755
A_24_P116766	-2.3	-1.0	-1.1	-1.0	ZNF207	zinc finger protein 207 [Source:HGNC Symbol;Acc:12998] [ENST00000394670]	7756
A_24_P296765	-3.2	1.8	-24.9	-1.3	ZNF211	Homo sapiens zinc finger protein 211 (ZNF211), transcript variant 2, mRNA [NM_198855]	10520
A_24_P364845	1.7	-1.3	-3.0	1.4	ZNF213	Homo sapiens zinc finger protein 213 (ZNF213), transcript variant 1, mRNA [NM_004220]	7760

A_23_P127840	-1.2	-3.2	-2.9	-2.6	ZNF214	Homo sapiens zinc finger protein 214 (ZNF214), mRNA [NM_013249]	7761
A_24_P318002	1.2	-1.4	-2.7	-1.0	ZNF215	Homo sapiens zinc finger protein 215 (ZNF215), mRNA [NM_013250]	7762
A_23_P384512	50.2	88.7	23.4	47.3	ZNF22	Homo sapiens zinc finger protein 22 (KOX 15) (ZNF22), mRNA [NM_006963]	7570
A_23_P90320	-1.2	-1.5	1.0	-5.0	ZNF221	Homo sapiens zinc finger protein 221 (ZNF221), mRNA [NM_013359]	7638
A_23_P125042	-1.4	-1.5	-1.0	-3.4	ZNF222	Homo sapiens zinc finger protein 222 (ZNF222), transcript variant 2, mRNA [NM_013360]	7673

A_23_P218517	-6.1	-1.8	-1.8	-1.5	ZNF222	Homo sapiens zinc finger protein 222 (ZNF222), transcript variant 2, mRNA [NM_013360]	7673
A_23_P10885	-2.5	-1.1	-1.2	-1.2	ZNF224	zinc finger protein 224 [Source:HGNC Symbol;Acc:13017] [ENST00000336976]	7767
A_23_P395555	-1.0	-1.0	-3.1	-1.0	ZNF226	Homo sapiens zinc finger protein 226 (ZNF226), transcript variant 1, mRNA [NM_001032372]	7769
A_24_P930347	1.0	-1.0	-1.1	-11.2	ZNF226	Homo sapiens zinc finger protein 226 (ZNF226), transcript variant 1, mRNA [NM_001032372]	7769

A_24_P113287	-1.4	1.0	-2.9	1.0	ZNF229	Homo sapiens zinc finger protein 229 (ZNF229), mRNA [NM_014518]	7772
A_23_P89073	1.0	1.0	-1.5	-3.1	ZNF23	Homo sapiens zinc finger protein 23 (KOX 16) (ZNF23), mRNA [NM_145911]	7571
A_24_P935125	2.3	1.4	1.0	1.0	ZNF23	Homo sapiens clone L3-15 zinc finger protein mRNA, partial cds. [AF024709]	7571
A_24_P147252	-2.7	-1.1	-1.9	-1.7	ZNF23	Homo sapiens zinc finger protein 23 (KOX 16) (ZNF23), mRNA [NM_145911]	7571
A_23_P4294	1.1	-1.1	-1.3	-2.2	ZNF232	Homo sapiens zinc finger protein 232 (ZNF232), mRNA [NM_014519]	7775

A_24_P151148	-1.0	-18.6	-1.1	-1.1	ZNF235	Homo sapiens zinc finger protein 235, mRNA (cDNA clone IMAGE:3605615). [BC002663]	9310
A_23_P208325	-1.6	1.0	-1.6	-2.1	ZNF235	Homo sapiens zinc finger protein 235 (ZNF235), mRNA [NM_004234]	9310
A_24_P110719	-1.4	-2.7	1.8	-4.1	ZNF236	Homo sapiens zinc finger protein 236 (ZNF236), mRNA [NM_007345]	7776
A_23_P35343	1.7	1.7	2.0	1.0	ZNF248	Homo sapiens zinc finger protein 248 (ZNF248), mRNA [NM_021045]	57209

A_24_P205019	2.5	2.4	2.3	2.7	ZNF250	Homo sapiens zinc finger protein 250 (ZNF250), transcript variant 1, mRNA [NM_021061]	58500
A_23_P412186	1.1	-8.5	-1.0	-1.1	ZNF252	Homo sapiens zinc finger protein 252 (ZNF252), non-coding RNA [NR_023392]	286101
A_24_P22981	1.9	1.4	1.0	2.0	ZNF253	Homo sapiens zinc finger protein 253 (ZNF253), mRNA [NM_021047]	56242
A_32_P144908	1.5	-1.3	-2.1	-2.3	ZNF254	Homo sapiens zinc finger protein 254 (ZNF254), mRNA [NM_203282]	9534

A_24_P298545	2.1	2.1	-1.4	2.0	ZNF257	Homo sapiens zinc finger protein 257 (ZNF257), mRNA [NM_033468]	113835
A_23_P16632	-1.8	-7.4	-1.0	-1.0	ZNF257	Homo sapiens zinc finger protein 257 (ZNF257), mRNA [NM_033468]	113835
A_23_P128060	3.3	2.1	2.0	2.1	ZNF26	Homo sapiens zinc finger protein 26 (ZNF26), mRNA [NM_019591]	7574
A_23_P140978	-2.0	-2.4	-2.5	-2.5	ZNF263	Homo sapiens zinc finger protein 263 (ZNF263), mRNA [NM_005741]	10127
A_24_P335901	1.7	-1.1	-2.1	1.0	ZNF266	Homo sapiens zinc finger protein 266 (ZNF266), mRNA [NM_006631]	10781

A_23_P107763	-6.2	1.1	-1.0	1.1	ZNF266	Homo sapiens zinc finger protein 266 (ZNF266), mRNA [NM_006631]	10781
A_23_P66260	-1.1	-1.2	-4.7	-1.3	ZNF267	Homo sapiens zinc finger protein 267 (ZNF267), transcript variant 498723, mRNA [NM_003414]	10308
A_32_P42780	-12.0	1.1	-1.6	-1.6	ZNF271	Homo sapiens zinc finger protein 271 (ZNF271), transcript variant 1, non-coding RNA [NR_024565]	10778
A_24_P58549	-1.2	-9.9	-1.0	1.0	ZNF273	Homo sapiens zinc finger protein 273 (ZNF273), transcript variant 1, mRNA [NM_021148]	10793

A_24_P218001	-6.0	1.0	1.0	1.0	ZNF273	Homo sapiens zinc finger protein 273 (ZNF273), transcript variant 1, mRNA [NM_021148]	10793
A_23_P153251	1.1	-16.9	1.0	1.1	ZNF274	Homo sapiens zinc finger protein 274 (ZNF274), transcript variant ZNF274c, mRNA [NM_133502]	10782
A_24_P194748	1.9	1.8	1.9	3.5	ZNF275	Homo sapiens zinc finger protein 275 (ZNF275), mRNA [NM_001080485]	10838
A_24_P133671	1.1	1.1	1.0	5.2	ZNF276	Homo sapiens zinc finger protein 276 (ZNF276), transcript variant b, mRNA [NM_152287]	92822

A_24_P243776	3.0	2.9	2.6	1.3	ZNF277	Homo sapiens zinc finger protein 277 (ZNF277), mRNA [NM_021994]	11179
A_24_P282043	-15.3	-1.0	-1.1	-1.0	ZNF28	Homo sapiens zinc finger protein 28 (ZNF28), transcript variant 1, mRNA [NM_006969]	7576
A_23_P402134	2.4	1.5	-1.7	-1.2	ZNF280B	Homo sapiens zinc finger protein 280B (ZNF280B), mRNA [NM_080764]	140883
A_23_P311087	2.5	3.7	1.8	2.1	ZNF281	Homo sapiens zinc finger protein 281 (ZNF281), mRNA [NM_012482]	23528
A_23_P428468	1.1	-5.8	1.0	1.0	ZNF292	Homo sapiens zinc finger protein 292 (ZNF292), mRNA [NM_015021]	23036

A_32_P15958	1.0	-1.0	-3.8	1.0	ZNF295-AS1	Homo sapiens ZNF295 antisense RNA 1 (non-protein coding) (ZNF295-AS1), non-coding RNA [NR_027273]	150142
A_23_P16058	-1.9	-1.0	-4.3	1.5	ZNF296	Homo sapiens zinc finger protein 296 (ZNF296), mRNA [NM_145288]	162979
A_23_P110699	-2.7	-2.5	-8.4	-9.9	ZNF300	Homo sapiens zinc finger protein 300 (ZNF300), transcript variant 2, mRNA [NM_052860]	91975
A_24_P159837	-1.5	1.0	-2.2	-1.5	ZNF302	Homo sapiens mRNA for PREDICTED: similar to regulator of sex-limitation candidate 11 variant protein. [AB209226]	55900

A_24_P228266	-1.1	1.1	-2.4	1.1	ZNF304	Homo sapiens zinc finger protein 304 (ZNF304), mRNA [NM_020657]	57343
A_23_P208288	-2.2	-1.0	-1.1	-1.0	ZNF304	Homo sapiens zinc finger protein 304 (ZNF304), mRNA [NM_020657]	57343
A_24_P152911	1.0	-1.0	-1.0	-8.8	ZNF311	Homo sapiens zinc finger protein 311 (ZNF311), mRNA [NM_001010877]	282890
A_23_P38813	-1.1	-1.1	-2.6	-1.1	ZNF320	Homo sapiens zinc finger protein 320 (ZNF320), mRNA [NM_207333]	162967
A_24_P83437	1.1	-3.5	-1.6	1.0	ZNF326	Homo sapiens zinc finger protein 326 (ZNF326), transcript variant 3, mRNA [NM_182975]	284695

A_23_P433690	1.0	-1.0	-6.0	-1.1	ZNF331	Homo sapiens zinc finger protein 331 (ZNF331), transcript variant 1, mRNA [NM_018555]	55422
A_23_P5221	3.0	2.4	-1.3	2.6	ZNF333	Homo sapiens zinc finger protein 333 (ZNF333), mRNA [NM_032433]	84449
A_23_P91250	-7.8	-1.4	-1.1	-1.1	ZNF334	Homo sapiens zinc finger protein 334 (ZNF334), transcript variant 2, mRNA [NM_199441]	55713
A_23_P17382	4.0	3.8	3.7	3.7	ZNF335	Homo sapiens zinc finger protein 335 (ZNF335), mRNA [NM_022095]	63925

A_23_P154758	1.4	1.7	-3.0	-1.1	ZNF337	Homo sapiens zinc finger protein 337 (ZNF337), mRNA [NM_015655]	26152
A_23_P430852	1.0	-1.0	-1.4	-4.8	ZNF33A	zinc finger protein 33A [Source:HGNC Symbol;Acc:13096] [ENST00000277672]	7581
A_24_P212127	2.8	2.7	2.5	2.7	ZNF33A	Homo sapiens zinc finger protein 33A (ZNF33A), transcript variant 1, mRNA [NM_006954]	7581
A_32_P61657	1.0	-1.0	-3.8	1.0	ZNF33B	Homo sapiens zinc finger protein 33B (ZNF33B), mRNA [NM_006955]	7582
A_23_P17570	-6.0	-1.1	-1.2	-1.5	ZNF341	Homo sapiens zinc finger protein 341 (ZNF341), mRNA [NM_032819]	84905

A_23_P17562	-23.7	-2.5	-2.3	-2.8	ZNF341	Homo sapiens zinc finger protein 341 (ZNF341), mRNA [NM_032819]	84905
A_23_P357248	-1.8	-9.5	-1.9	-1.7	ZNF343	Homo sapiens zinc finger protein 343 (ZNF343), mRNA [NM_024325]	79175
A_23_P39154	6.1	1.9	-2.5	1.7	ZNF345	Homo sapiens zinc finger protein 345 (ZNF345), transcript variant 1, mRNA [NM_003419]	25850
A_23_P400887	-1.0	-1.7	-3.2	1.1	ZNF346	Homo sapiens zinc finger protein 346 (ZNF346), mRNA [NM_012279]	23567
A_23_P400884	-1.3	2.4	-2.6	1.4	ZNF346	Homo sapiens zinc finger protein 346 (ZNF346), mRNA [NM_012279]	23567

A_23_P78458	2.2	2.3	2.3	2.3	ZNF350	Homo sapiens zinc finger protein 350 (ZNF350), mRNA [NM_021632]	59348
A_24_P19175	-3.3	1.2	1.4	1.1	ZNF358	Homo sapiens zinc finger protein 358 (ZNF358), mRNA [NM_018083]	140467
A_23_P137543	1.3	2.0	3.0	-1.3	ZNF362	Homo sapiens zinc finger protein 362 (ZNF362), mRNA [NM_152493]	149076
A_32_P183218	-1.3	2.0	1.9	2.0	ZNF367	Homo sapiens zinc finger protein 367 (ZNF367), mRNA [NM_153695]	195828
A_23_P410625	-9.5	-1.1	-1.2	-1.1	ZNF367	Homo sapiens zinc finger protein 367 (ZNF367), mRNA [NM_153695]	195828

A_24_P239668	-2.8	-1.0	-1.0	-1.1	ZNF37A	Homo sapiens zinc finger protein 37A (ZNF37A), transcript variant 1, mRNA [NM_001007094]	7587
A_23_P319013	-1.2	-1.2	-4.0	-1.4	ZNF383	Homo sapiens zinc finger protein 383 (ZNF383), mRNA [NM_152604]	163087
A_24_P403734	-2.7	1.2	-1.5	-1.0	ZNF385A	Homo sapiens zinc finger protein 385A (ZNF385A), transcript variant 3, mRNA [NM_015481]	25946
A_23_P334857	-1.0	-2.6	-1.0	-1.0	ZNF385B	Homo sapiens zinc finger protein 385B (ZNF385B), transcript variant 1, mRNA [NM_152520]	151126

A_24_P917306	-1.1	-29.7	-1.1	-1.1	ZNF385D	zinc finger protein 385D [Source:HGNC Symbol;Acc:26191] [ENST00000494108]	79750
A_23_P157416	1.1	1.5	-2.2	1.1	ZNF394	Homo sapiens zinc finger protein 394 (ZNF394), mRNA [NM_032164]	84124
A_24_P153680	1.0	-6.9	-1.9	-1.3	ZNF395	Homo sapiens zinc finger protein 395 (ZNF395), mRNA [NM_018660]	55893
A_24_P67918	-1.1	-1.1	-2.1	-1.3	ZNF396	Homo sapiens zinc finger protein 396 (ZNF396), mRNA [NM_145756]	252884
A_24_P393565	-2.2	1.1	-1.1	-1.8	ZNF396	zinc finger protein 396 [Source:HGNC Symbol;Acc:18824] [ENST00000399057]	252884

A_23_P90333	-2.2	-1.0	-1.0	-2.0	ZNF404	Homo sapiens zinc finger protein 404 (ZNF404), mRNA [NM_001033719]	342908
A_23_P380954	2.3	2.2	-1.0	1.3	ZNF407	Homo sapiens zinc finger protein 407 (ZNF407), transcript variant 2, mRNA [NM_001146189]	55628
A_23_P32722	-1.3	-1.1	-2.7	-1.3	ZNF408	Homo sapiens zinc finger protein 408 (ZNF408), transcript variant 1, mRNA [NM_024741]	79797
A_23_P45234	28.5	32.3	26.0	25.9	ZNF41	Homo sapiens zinc finger protein 41 (ZNF41), transcript variant 2, mRNA [NM_153380]	7592

A_23_P27424	1.2	1.6	-2.1	-1.0	ZNF418	Homo sapiens zinc finger protein 418 (ZNF418), mRNA [NM_133460]	147686
A_24_P941166	-1.1	-17.1	1.1	-1.2	ZNF425	Homo sapiens zinc finger protein 425 (ZNF425), mRNA [NM_001001661]	155054
A_32_P106944	-1.3	-2.0	-2.2	-4.2	ZNF429	Homo sapiens zinc finger protein 429 (ZNF429), mRNA [NM_001001415]	353088
A_23_P5234	-1.3	-1.3	-2.4	-1.3	ZNF43	Homo sapiens zinc finger protein 43 (ZNF43), mRNA [NM_003423]	7594
A_23_P56298	1.1	-19.6	1.1	1.0	ZNF430	Homo sapiens zinc finger protein 430 (ZNF430), transcript variant 1, mRNA [NM_025189]	80264

A_23_P39441	-2.0	1.4	-1.7	1.4	ZNF431	Homo sapiens zinc finger protein 431 (ZNF431), mRNA [NM_133473]	170959
A_23_P27649	1.3	1.2	-2.1	1.2	ZNF433	Homo sapiens zinc finger protein 433 (ZNF433), mRNA [NM_001080411]	163059
A_32_P728662	1.1	1.0	1.0	-9.8	ZNF44	Homo sapiens zinc finger protein 44, mRNA (cDNA clone IMAGE:5202234). [BC032246]	51710
A_24_P914240	-1.2	1.1	-8.4	1.1	ZNF44	zinc finger protein 44 [Source:HGNC Symbol;Acc:13110] [ENST00000483826]	51710
A_23_P67278	2.3	-1.4	2.3	2.3	ZNF443	Homo sapiens zinc finger protein 443 (ZNF443), mRNA [NM_005815]	10224

A_23_P27767	2.0	3.0	1.8	1.4	ZNF444	Homo sapiens zinc finger protein 444 (ZNF444), mRNA [NM_018337]	55311
A_24_P126587	-1.8	-2.8	-1.9	1.4	ZNF445	Homo sapiens zinc finger protein 445 (ZNF445), mRNA [NM_181489]	353274
A_23_P101272	2.7	1.1	1.9	1.3	ZNF446	Homo sapiens zinc finger protein 446 (ZNF446), mRNA [NM_017908]	55663
A_24_P349676	1.6	2.7	-2.4	1.8	ZNF446	Homo sapiens zinc finger protein 446 (ZNF446), mRNA [NM_017908]	55663
A_23_P309865	-1.0	-3.0	-2.0	1.0	ZNF449	Homo sapiens zinc finger protein 449 (ZNF449), mRNA [NM_152695]	203523

A_24_P68415	1.0	1.0	1.7	-2.2	ZNF45	Homo sapiens zinc finger protein 45 (ZNF45), mRNA [NM_003425]	7596
A_24_P320572	5.0	-1.0	-2.1	-1.0	ZNF451	Homo sapiens zinc finger protein 451 (ZNF451), transcript variant 2, mRNA [NM_015555]	26036
A_23_P67424	-1.0	-1.1	4.7	-1.0	ZNF461	Homo sapiens zinc finger protein 461 (ZNF461), mRNA [NM_153257]	92283
A_23_P60499	-2.9	-7.1	-1.0	1.4	ZNF462	Homo sapiens zinc finger protein 462 (ZNF462), mRNA [NM_021224]	58499
A_24_P218074	-6.8	1.3	-1.0	1.1	ZNF467	Homo sapiens zinc finger protein 467 (ZNF467), mRNA [NM_207336]	168544

A_24_P916718	1.8	1.5	-1.5	-3.2	ZNF467	Homo sapiens zinc finger protein 467 (ZNF467), mRNA [NM_207336]	168544
A_32_P116556	-1.7	-2.1	-2.9	-1.2	ZNF469	Homo sapiens zinc finger protein 469 (ZNF469), mRNA [NM_001127464]	84627
A_23_P417684	1.0	-1.0	-7.3	-1.1	ZNF471	Homo sapiens mRNA for KIAA1396 protein, partial cds. [AB037817]	57573
A_23_P107942	1.1	-9.6	-1.9	1.9	ZNF473	Homo sapiens zinc finger protein 473 (ZNF473), transcript variant 1, mRNA [NM_015428]	25888
A_24_P16249	1.1	-3.2	-1.0	1.0	ZNF479	Homo sapiens zinc finger protein 479 (ZNF479), mRNA [NM_033273]	90827

A_24_P102362	2.7	1.4	1.8	1.6	ZNF48	Homo sapiens zinc finger protein 48 (ZNF48), transcript variant 3, mRNA [NM_001214909]	197407
A_32_P226157	-1.0	-1.0	-1.0	-2.7	ZNF484	Homo sapiens cDNA, FLJ99658. [AK309617]	83744
A_23_P341700	1.0	-1.0	-1.1	-11.3	ZNF484	Homo sapiens zinc finger protein 484 (ZNF484), transcript variant 2, mRNA [NM_001007101]	83744
A_24_P15062	7.0	6.7	6.3	6.6	ZNF490	Homo sapiens zinc finger protein 490 (ZNF490), mRNA [NM_020714]	57474
A_24_P194971	11.7	11.6	7.7	11.4	ZNF492	Homo sapiens zinc finger protein 492 (ZNF492), mRNA [NM_020855]	57615

A_23_P153855	-1.0	-2.3	-1.1	-1.0	ZNF492	Homo sapiens zinc finger protein 492 (ZNF492), mRNA [NM_020855]	57615
A_23_P354942	-2.1	-1.0	-1.0	1.3	ZNF493	Homo sapiens zinc finger protein 493 (ZNF493), transcript variant 3, mRNA [NM_001076678]	284443
A_23_P319050	1.0	1.0	-16.8	-1.0	ZNF493	Homo sapiens zinc finger protein 493 (ZNF493), transcript variant 2, mRNA [NM_145326]	284443
A_24_P282261	1.0	1.0	1.0	-3.3	ZNF493	Homo sapiens zinc finger protein 493, mRNA (cDNA clone IMAGE:4617832), with apparent retained intron. [BC022394]	284443

A_24_P376787	3.4	5.1	2.1	3.0	ZNF496	Homo sapiens zinc finger protein 496 (ZNF496), mRNA [NM_032752]	84838
A_23_P309246	1.3	-3.2	-1.2	-2.5	ZNF498	Homo sapiens zinc finger protein 498 (ZNF498), mRNA [NM_145115]	221785
A_32_P486443	-2.1	1.0	-1.3	-1.2	ZNF500	Homo sapiens mRNA for KIAA0557 protein, partial cds. [AB011129]	26048
A_23_P202484	2.1	1.6	2.1	-1.5	ZNF503	Homo sapiens zinc finger protein 503 (ZNF503), mRNA [NM_032772]	84858
A_23_P16609	1.8	2.1	-1.4	2.3	ZNF506	Homo sapiens mRNA; cDNA DKFZp761G18121 (from clone DKFZp761G18121). [AL136548]	440515

A_23_P16607	-2.0	-3.2	-7.1	-2.4	ZNF506	Homo sapiens mRNA; cDNA DKFZp761G18121 (from clone DKFZp761G18121). [AL136548]	440515
A_23_P374944	-2.5	1.0	-3.2	1.1	ZNF507	Homo sapiens mRNA for KIAA1084 protein, partial cds. [AB029007]	22847
A_23_P28730	1.0	1.0	-2.5	-1.0	ZNF512B	Homo sapiens zinc finger protein 512B (ZNF512B), mRNA [NM_020713]	57473
A_23_P55606	-2.9	1.3	1.2	-1.0	ZNF516	Homo sapiens zinc finger protein 516 (ZNF516), mRNA [NM_014643]	9658
A_24_P944320	1.1	1.9	2.0	-2.0	ZNF517	Homo sapiens zinc finger protein 517 (ZNF517), mRNA [NM_213605]	340385

A_23_P328766	1.0	-1.0	1.1	-3.5	ZNF519	Homo sapiens zinc finger protein 519 (ZNF519), transcript variant 1, mRNA [NM_145287]	162655
A_32_P98348	-9.2	-1.8	1.0	1.1	ZNF525	Homo sapiens zinc finger protein 525 (ZNF525), non-coding RNA [NR_003699]	170958
A_23_P435944	-1.9	-3.0	-2.0	-1.9	ZNF530	Homo sapiens mRNA for KIAA1508 protein, partial cds. [AB040941]	348327
A_23_P130811	1.0	-1.0	-1.1	-3.3	ZNF536	Homo sapiens zinc finger protein 536 (ZNF536), mRNA [NM_014717]	9745

A_23_P130876	2.5	2.5	1.6	3.0	ZNF546	Homo sapiens zinc finger protein 546 (ZNF546), mRNA [NM_178544]	339327
A_23_P4640	23.2	22.6	21.5	23.2	ZNF548	Homo sapiens zinc finger protein 548 (ZNF548), transcript variant 2, mRNA [NM_152909]	147694
A_24_P68019	1.0	-4.6	1.0	-1.0	ZNF551	Homo sapiens zinc finger protein 551 (ZNF551), mRNA [NM_138347]	90233
A_24_P693448	-1.5	-1.2	-1.2	2.8	ZNF552	Homo sapiens cDNA FLJ13707 fis, clone PLACE2000347. [AK023769]	79818

A_32_P177097	-1.0	-2.5	-1.0	-1.0	ZNF557	Homo sapiens zinc finger protein 557 (ZNF557), transcript variant 1, mRNA [NM_024341]	79230
A_23_P28015	-2.4	-1.1	1.0	-1.5	ZNF558	Homo sapiens zinc finger protein 558 (ZNF558), mRNA [NM_144693]	148156
A_24_P284584	-1.7	-2.2	-1.9	-1.9	ZNF559	Homo sapiens zinc finger protein 559 (ZNF559), transcript variant 2, mRNA [NM_032497]	84527
A_23_P368773	1.3	-1.2	-2.8	1.1	ZNF560	Homo sapiens zinc finger protein 560 (ZNF560), mRNA [NM_152476]	147741

A_23_P95923	2.6	12.2	4.9	5.5	ZNF562	Homo sapiens zinc finger protein 562 (ZNF562), transcript variant 3, mRNA [NM_017656]	54811
A_24_P348208	-1.1	-2.1	-2.1	-2.0	ZNF562	Homo sapiens zinc finger protein 562 (ZNF562), transcript variant 3, mRNA [NM_017656]	54811
A_23_P101615	2.2	2.2	2.2	2.2	ZNF565	Homo sapiens zinc finger protein 565 (ZNF565), transcript variant 1, mRNA [NM_001042474]	147929
A_23_P208752	-5.9	1.1	-2.2	1.1	ZNF566	Homo sapiens zinc finger protein 566 (ZNF566), transcript variant 1, mRNA [NM_001145345]	84924

A_24_P915049	1.0	-2.4	-2.8	-1.5	ZNF568	Homo sapiens zinc finger protein 568, mRNA (cDNA clone IMAGE:4052822), partial cds. [BC016334]	374900
A_24_P101304	-1.1	-1.0	-2.7	-1.0	ZNF568	Homo sapiens zinc finger protein 568 (ZNF568), transcript variant 1, mRNA [NM_198539]	374900
A_23_P131128	-1.0	1.0	2.6	1.5	ZNF569	Homo sapiens zinc finger protein 569 (ZNF569), mRNA [NM_152484]	148266
A_23_P79145	-1.3	-1.7	-3.6	-1.5	ZNF570	Homo sapiens zinc finger protein 570 (ZNF570), mRNA [NM_144694]	148268

A_23_P401084	1.0	-1.0	-1.1	-3.2	ZNF575	Homo sapiens zinc finger protein 575 (ZNF575), mRNA [NM_174945]	284346
A_23_P339601	-3.3	-1.0	-1.1	-1.1	ZNF578	Homo sapiens zinc finger protein 578 (ZNF578), mRNA [NM_001099694]	147660
A_23_P164797	-1.4	1.3	-2.4	-1.3	ZNF580	Homo sapiens zinc finger protein 580 (ZNF580), transcript variant 1, mRNA [NM_016202]	51157
A_23_P4798	1.3	-1.7	-2.1	-2.6	ZNF581	Homo sapiens zinc finger protein 581 (ZNF581), mRNA [NM_016535]	51545
A_23_P395464	1.0	-4.2	-1.0	-1.0	ZNF582	Homo sapiens zinc finger protein 582 (ZNF582), mRNA [NM_144690]	147948

A_24_P849467	4.8	4.6	3.2	4.8	ZNF585A	Homo sapiens cDNA FLJ41302 fis, clone BRAMY2042122. [AK123296]	199704
A_24_P25470	2.6	2.6	2.6	2.6	ZNF586	zinc finger protein 586 [Source:HGNC Symbol;Acc:25949] [ENST00000430084]	54807
A_24_P83944	-1.0	-1.0	-3.7	-1.5	ZNF586	Homo sapiens zinc finger protein 586 (ZNF586), transcript variant 1, mRNA [NM_017652]	54807
A_24_P373726	-2.0	1.7	1.6	1.0	ZNF587	Homo sapiens zinc finger protein 587 (ZNF587), transcript variant 1, mRNA [NM_032828]	84914

A_24_P313804	-6.2	-1.8	-6.1	-2.1	ZNF587	Homo sapiens zinc finger protein 587 (ZNF587), transcript variant 1, mRNA [NM_032828]	84914
A_23_P34741	-8.2	-1.2	-1.3	-1.3	ZNF593	Homo sapiens zinc finger protein 593 (ZNF593), mRNA [NM_015871]	51042
A_23_P155939	-1.2	-1.2	-2.0	-2.6	ZNF595	Homo sapiens zinc finger protein 595 (ZNF595), mRNA [NM_182524]	152687
A_32_P187663	-4.1	1.1	-2.9	1.1	ZNF596	Homo sapiens zinc finger protein 596 (ZNF596), transcript variant 1, mRNA [NM_001042416]	169270

A_24_P378402	-2.7	-1.2	1.0	-1.8	ZNF597	Homo sapiens zinc finger protein 597 (ZNF597), mRNA [NM_152457]	146434
A_23_P3753	2.6	2.6	2.7	1.5	ZNF597	Homo sapiens zinc finger protein 597 (ZNF597), mRNA [NM_152457]	146434
A_23_P16006	2.5	2.3	2.3	2.1	ZNF600	Homo sapiens zinc finger protein 600 (ZNF600), mRNA [NM_198457]	162966
A_23_P208172	1.1	2.2	-1.5	1.8	ZNF613	Homo sapiens cDNA FLJ13590 fis, clone PLACE1009398. [AK023652]	79898
A_24_P173234	-2.1	-1.0	-1.3	-1.0	ZNF613	Homo sapiens zinc finger protein 613 (ZNF613), transcript variant 2, mRNA [NM_024840]	79898

A_23_P101258	2.0	-1.1	-1.1	-1.0	ZNF614	Homo sapiens zinc finger protein 614 (ZNF614), mRNA [NM_025040]	80110
A_24_P190345	1.0	1.0	-1.0	-2.1	ZNF615	Homo sapiens zinc finger protein 615 (ZNF615), transcript variant 2, mRNA [NM_198480]	284370
A_32_P186921	1.0	1.0	-3.6	-2.3	ZNF616	Homo sapiens zinc finger protein 616, mRNA (cDNA clone IMAGE:4186857). [BC032805]	90317
A_24_P743802	1.1	-3.0	-1.0	1.0	ZNF618	Homo sapiens zinc finger protein 618 (ZNF618), mRNA [NM_133374]	114991

A_23_P405216	-1.1	-1.1	-3.9	1.1	ZNF619	Homo sapiens zinc finger protein 619 (ZNF619), transcript variant 5, mRNA [NM_001145083]	285267
A_24_P344537	1.9	-1.1	-2.4	-2.1	ZNF625	Homo sapiens zinc finger protein 625 (ZNF625), transcript variant 1, mRNA [NM_145233]	90589
A_32_P102778	-2.7	-2.0	1.4	-2.2	ZNF626	Homo sapiens zinc finger protein 626 (ZNF626), transcript variant 1, mRNA [NM_001076675]	199777
A_23_P390384	-2.8	-1.8	1.2	-1.7	ZNF630	Homo sapiens zinc finger protein 630 (ZNF630), transcript variant 1, mRNA [NM_001037735]	57232

A_32_P199767	-1.6	-1.2	-2.0	-1.2	ZNF630	Homo sapiens zinc finger protein 630 (ZNF630), transcript variant 1, mRNA [NM_001037735]	57232
A_24_P419177	-10.7	-2.4	-3.1	-1.6	ZNF638	Homo sapiens zinc finger protein 638 (ZNF638), transcript variant 1, mRNA [NM_014497]	27332
A_32_P24709	-1.8	-1.8	-3.7	-2.0	ZNF642	Homo sapiens zinc finger protein 642 (ZNF642), mRNA [NM_198494]	339559
A_24_P238402	1.6	3.8	2.5	2.5	ZNF644	Homo sapiens zinc finger protein 644 (ZNF644), transcript variant 1, mRNA [NM_201269]	84146

A_24_P256337	-1.0	-4.3	-1.7	5.2	ZNF644	Homo sapiens zinc finger protein 644 (ZNF644), transcript variant 1, mRNA [NM_201269]	84146
A_24_P31875	1.2	1.3	1.5	-4.5	ZNF645	Homo sapiens zinc finger protein 645 (ZNF645), mRNA [NM_152577]	158506
A_24_P181506	1.0	-1.0	-26.0	-1.1	ZNF646	Homo sapiens zinc finger protein 646 (ZNF646), mRNA [NM_014699]	9726
A_23_P55256	-2.7	2.0	1.8	1.4	ZNF652	Homo sapiens zinc finger protein 652 (ZNF652), transcript variant 2, mRNA [NM_014897]	22834

A_23_P325676	1.2	3.9	-1.1	1.7	ZNF653	Homo sapiens zinc finger protein 653 (ZNF653), mRNA [NM_138783]	115950
A_23_P215819	1.0	-1.1	-1.2	-5.5	ZNF655	Homo sapiens zinc finger protein 655 (ZNF655), transcript variant 2, mRNA [NM_024061]	79027
A_23_P345081	1.0	-1.2	-1.1	-3.7	ZNF655	Homo sapiens zinc finger protein 655 (ZNF655), transcript variant 7, mRNA [NM_001083956]	79027
A_32_P220739	-3.3	-1.2	-1.6	-1.2	ZNF658	Homo sapiens zinc finger protein 658 (ZNF658), mRNA [NM_033160]	26149

A_23_P31938	-15.3	-1.1	-4.4	-1.3	ZNF658	Homo sapiens zinc finger protein 658 (ZNF658), mRNA [NM_033160]	26149
A_32_P375286	-1.0	-1.1	-17.9	-1.5	ZNF660	Homo sapiens zinc finger protein 660 (ZNF660), mRNA [NM_173658]	285349
A_24_P341292	2.6	3.5	1.1	2.6	ZNF665	Homo sapiens zinc finger protein 665 (ZNF665), mRNA [NM_024733]	79788
A_23_P66289	1.0	-8.4	2.0	2.1	ZNF668	Homo sapiens zinc finger protein 668 (ZNF668), transcript variant 2, mRNA [NM_024706]	79759
A_23_P50217	-12.5	1.2	-1.1	-1.0	ZNF671	Homo sapiens zinc finger protein 671 (ZNF671), mRNA [NM_024833]	79891

A_24_P92558	1.3	-1.0	-2.8	-1.0	ZNF674	Homo sapiens zinc finger protein 674 (ZNF674), transcript variant 1, mRNA [NM_001039891]	641339
A_23_P339687	2.5	1.0	-2.6	-1.5	ZNF675	Homo sapiens zinc finger protein 675 (ZNF675), mRNA [NM_138330]	171392
A_24_P205036	-1.6	-1.9	-1.8	-2.6	ZNF676	Homo sapiens zinc finger protein 676 (ZNF676), mRNA [NM_001001411]	163223
A_32_P189845	-1.1	-1.1	-3.3	-1.6	ZNF680	Homo sapiens zinc finger protein 680 (ZNF680), transcript variant 1, mRNA [NM_178558]	340252

A_23_P305210	2.6	2.5	1.1	2.5	ZNF680	Homo sapiens zinc finger protein 680 (ZNF680), transcript variant 1, mRNA [NM_178558]	340252
A_23_P337923	1.6	1.7	3.4	1.0	ZNF680	Homo sapiens zinc finger protein 680 (ZNF680), transcript variant 1, mRNA [NM_178558]	340252
A_23_P375517	2.0	-1.6	-1.3	2.0	ZNF681	Homo sapiens zinc finger protein 681 (ZNF681), mRNA [NM_138286]	148213
A_23_P141044	-4.7	1.1	-1.1	-2.0	ZNF688	Homo sapiens zinc finger protein 688 (ZNF688), transcript variant 1, mRNA [NM_145271]	146542

A_23_P35316	2.3	1.2	2.0	1.3	ZNF695	Homo sapiens zinc finger protein 695 (ZNF695), transcript variant 1, mRNA [NM_020394]	57116
A_23_P322076	1.1	-2.4	-1.1	1.0	ZNF697	Homo sapiens zinc finger protein 697 (ZNF697), mRNA [NM_001080470]	90874
A_32_P19716	-1.1	1.0	-3.7	1.1	ZNF697	Homo sapiens zinc finger protein 697 (ZNF697), mRNA [NM_001080470]	90874
A_23_P255805	1.7	2.4	1.7	1.4	ZNF7	Homo sapiens zinc finger protein 7 (ZNF7), mRNA [NM_003416]	7553
A_24_P941625	1.3	2.0	1.1	-4.0	ZNF70	Homo sapiens zinc finger protein 70 (ZNF70), mRNA [NM_021916]	7621

A_23_P27638	-1.0	2.1	-1.4	1.7	ZNF700	Homo sapiens zinc finger protein 700 (ZNF700), mRNA [NM_144566]	90592
A_23_P55666	1.7	1.8	2.0	1.0	ZNF702P	Homo sapiens cDNA FLJ12985 fis, clone NT2RP3000050. [AK023047]	79986
A_24_P274928	4.1	2.1	2.9	1.1	ZNF703	Homo sapiens zinc finger protein 703 (ZNF703), mRNA [NM_025069]	80139
A_32_P229493	-2.6	-1.3	-1.1	-3.3	ZNF704	Homo sapiens zinc finger protein 704 (ZNF704), mRNA [NM_001033723]	619279
A_24_P230074	-1.9	-1.5	-1.3	-2.1	ZNF704	Homo sapiens zinc finger protein 704 (ZNF704), mRNA [NM_001033723]	619279

A_24_P235305	-4.1	1.4	-1.1	-1.7	ZNF706	Homo sapiens zinc finger protein 706 (ZNF706), transcript variant 1, mRNA [NM_0010 42510]	51123
A_32_P46154	-1.5	1.4	-2.5	1.0	ZNF708	Homo sapiens zinc finger protein 708 (ZNF708), mRNA [NM_0212 69]	7562
A_24_P680908	1.2	1.2	-3.4	1.0	ZNF708	Homo sapiens zinc finger protein 708 (ZNF708), mRNA [NM_0212 69]	7562
A_32_P46155	1.1	1.1	-3.4	-1.0	ZNF708	Homo sapiens zinc finger protein 708 (ZNF708), mRNA [NM_0212 69]	7562
A_23_P340148	1.1	-9.2	1.0	-1.0	ZNF711	Homo sapiens zinc finger protein 711 (ZNF711), mRNA [NM_0219 98]	7552

A_23_P74114	5.4	3.4	4.0	2.0	ZNF713	Homo sapiens zinc finger protein 713 (ZNF713), mRNA [NM_182633]	349075
A_23_P50825	1.6	5.2	1.2	5.8	ZNF714	Homo sapiens zinc finger protein 714 (ZNF714), mRNA [NM_182515]	148206
A_24_P204639	-1.8	1.6	-3.0	-1.6	ZNF716	Homo sapiens zinc finger protein 716 (ZNF716), mRNA [NM_001159279]	441234
A_23_P124438	-6.1	-7.0	-2.0	-3.9	ZNF718	Homo sapiens zinc finger protein 718 (ZNF718), mRNA [NM_001039127]	255403
A_24_P358868	-6.6	1.0	1.3	-1.0	ZNF728	PREDICTED: Homo sapiens zinc finger protein 728 (ZNF728), mRNA [XM_001720936]	388523

A_24_P161696	1.5	1.6	1.2	2.1	ZNF729	Homo sapiens zinc finger protein 729 (ZNF729), mRNA [NM_001242680]	100287226
A_24_P126491	1.1	-1.5	2.4	1.5	ZNF729	Homo sapiens zinc finger protein 729 (ZNF729), mRNA [NM_001242680]	100287226
A_32_P181513	1.0	-2.8	-2.9	-1.9	ZNF730	zinc finger protein 730 [Source:HGNC Symbol;Acc:32470] [ENST00000327867]	100129543
A_24_P281801	1.2	-2.5	-1.7	1.0	ZNF732	Homo sapiens zinc finger protein 732 (ZNF732), mRNA [NM_001137608]	654254
A_24_P897062	-4.2	-1.0	-1.1	-1.1	ZNF736	Homo sapiens zinc finger protein 736 (ZNF736), mRNA [NM_001170905]	728927

A_23_P28042	2.1	2.7	1.9	2.0	ZNF737	Homo sapiens zinc finger protein 737 (ZNF737), mRNA [NM_001159293]	100129842
A_32_P68148	-1.7	-5.4	-3.5	-3.3	ZNF738	Homo sapiens zinc finger protein 738 (ZNF738), non-coding RNA [NR_027130]	148203
A_32_P3705	1.8	2.1	1.6	-1.1	ZNF740	Homo sapiens zinc finger protein 740 (ZNF740), mRNA [NM_001004304]	283337
A_24_P206344	-1.5	1.4	-1.1	-2.1	ZNF746	Homo sapiens zinc finger protein 746 (ZNF746), transcript variant 2, mRNA [NM_152557]	155061

A_23_P3819	5.0	4.6	2.5	5.0	ZNF747	Homo sapiens zinc finger protein 747 (ZNF747), mRNA [NM_023931]	65988
A_23_P73708	-1.0	-1.0	-2.4	-1.0	ZNF75D	Homo sapiens zinc finger protein 75D (ZNF75D), transcript variant 1, mRNA [NM_007131]	7626
A_24_P941487	2.2	1.2	1.1	-1.0	ZNF761	Homo sapiens zinc finger protein 761 (ZNF761), mRNA [NM_001008401]	388561
A_23_P206792	1.8	-2.3	1.6	1.3	ZNF764	Homo sapiens zinc finger protein 764 (ZNF764), transcript variant 1, mRNA [NM_033410]	92595

A_32_P84772	2.1	-1.0	-1.1	2.1	ZNF765	Homo sapiens zinc finger protein 765 (ZNF765), mRNA [NM_001040185]	91661
A_24_P202637	2.2	2.1	2.1	-1.2	ZNF765	zinc finger protein 765 [Source:HGNC Symbol;Acc:25092] [ENST00000504235]	91661
A_24_P935276	1.3	-9.2	1.1	1.6	ZNF766	Homo sapiens zinc finger protein 766 (ZNF766), mRNA [NM_001010851]	90321
A_23_P323143	-1.3	-1.2	-5.0	-1.9	ZNF767	Homo sapiens zinc finger family member 767 (ZNF767), transcript variant 1, non-coding RNA [NR_027788]	79970

A_23_P15218	2.0	2.1	1.6	1.3	ZNF768	Homo sapiens zinc finger protein 768 (ZNF768), mRNA [NM_024671]	79724
A_23_P65733	-3.0	-2.8	-5.7	-2.6	ZNF770	Homo sapiens zinc finger protein 770 (ZNF770), mRNA [NM_014106]	54989
A_24_P186324	-1.0	-1.0	-1.1	-4.9	ZNF770	Homo sapiens zinc finger protein 770 (ZNF770), mRNA [NM_014106]	54989
A_23_P67089	1.4	-1.8	-3.6	-1.1	ZNF772	Homo sapiens zinc finger protein 772 (ZNF772), transcript variant 1, mRNA [NM_001024596]	400720
A_24_P384099	-2.3	-1.1	-3.7	-1.4	ZNF775	Homo sapiens zinc finger protein 775 (ZNF775), mRNA [NM_173680]	285971

A_23_P351232	1.0	2.2	-1.2	-1.0	ZNF775	Homo sapiens zinc finger protein 775 (ZNF775), mRNA [NM_173680]	285971
A_23_P378499	-1.1	1.0	1.0	2.9	ZNF776	Homo sapiens cDNA FLJ38288 fis, clone FCBBF3008382, weakly similar to Homo sapiens C2H2 (Kruppel-type) zinc finger protein mRNA. [AK095607]	284309
A_23_P309803	-1.2	1.6	-3.8	-1.4	ZNF777	Homo sapiens zinc finger protein 777 (ZNF777), mRNA [NM_015694]	27153
A_32_P84846	-1.1	-1.0	-1.1	-3.1	ZNF780B	Homo sapiens zinc finger protein 780B (ZNF780B), mRNA [NM_001005851]	163131

A_24_P944063	6.1	6.4	5.8	5.2	ZNF785	Homo sapiens zinc finger protein 785 (ZNF785), mRNA [NM_152458]	146540
A_24_P919279	-2.5	-1.0	-1.1	-1.0	ZNF790	Homo sapiens zinc finger protein 790 (ZNF790), transcript variant 1, mRNA [NM_206894]	388536
A_24_P382401	1.0	-2.0	-1.1	-1.0	ZNF791	Homo sapiens zinc finger protein 791 (ZNF791), mRNA [NM_153358]	163049
A_23_P50418	3.4	5.0	3.2	3.2	ZNF791	Homo sapiens zinc finger protein 791 (ZNF791), mRNA [NM_153358]	163049
A_23_P130466	-1.2	4.0	1.1	1.0	ZNF8	Homo sapiens zinc finger protein 8 (ZNF8), mRNA [NM_021089]	7554

A_23_P419192	1.6	-3.8	1.6	1.3	ZNF800	Homo sapiens zinc finger protein 800 (ZNF800), mRNA [NM_176814]	168850
A_24_P374461	1.3	2.2	1.3	-1.0	ZNF804B	Homo sapiens zinc finger protein 804B (ZNF804B), mRNA [NM_181646]	219578
A_24_P450870	1.1	1.3	-2.1	1.1	ZNF813	zinc finger protein 813 [Source:HGNC Symbol;Acc:33257] [ENST00000396403]	126017
A_32_P96435	-2.8	1.0	1.0	1.0	ZNF829	Homo sapiens zinc finger protein 829 (ZNF829), transcript variant 2, mRNA [NM_001037232]	374899
A_23_P387071	-3.3	1.2	1.2	-1.5	ZNF830	Homo sapiens zinc finger protein 830 (ZNF830), mRNA [NM_052857]	91603

A_32_P206479	2.2	1.6	1.5	1.0	ZNF831	Homo sapiens zinc finger protein 831 (ZNF831), mRNA [NM_178457]	128611
A_23_P330929	2.0	-3.0	-3.2	1.2	ZNF835	Homo sapiens zinc finger protein 835 (ZNF835), mRNA [NM_001005850]	90485
A_23_P27404	-3.2	-1.2	-1.5	-2.1	ZNF841	Homo sapiens zinc finger protein 841 (ZNF841), mRNA [NM_001136499]	284371
A_24_P161144	-1.1	-1.1	-2.1	-1.6	ZNF843	Homo sapiens zinc finger protein 843 (ZNF843), mRNA [NM_001136509]	283933
A_24_P110743	-2.2	-1.1	-1.2	-1.2	ZNF844	zinc finger protein 844 [Source:HGNC Symbol;Acc:25932] [ENST00000439326]	284391

A_23_P67702	1.3	-3.6	-1.8	1.0	ZNF85	Homo sapiens zinc finger protein 85 (ZNF85), transcript variant 1, mRNA [NM_003429]	7639
A_24_P450596	-1.1	-1.1	-8.1	-1.4	ZNF850	Homo sapiens zinc finger protein 850 (ZNF850), mRNA [NM_001193552]	342892
A_24_P153207	-2.4	1.5	1.8	1.7	ZNF850	Homo sapiens zinc finger protein 850 (ZNF850), mRNA [NM_001193552]	342892
A_23_P8408	1.4	2.0	-2.2	2.5	ZNF862	Homo sapiens zinc finger protein 862 (ZNF862), mRNA [NM_001099220]	643641

A_32_P930685	-1.3	-1.2	-4.0	-3.6	ZNF876P	Homo sapiens zinc finger protein 876, pseudogene (ZNF876P), non-coding RNA [NR_027481]	642280
A_24_P791669	1.0	-1.0	-1.1	-4.5	ZNF879	Homo sapiens zinc finger protein 879 (ZNF879), mRNA [NM_001136116]	345462
A_24_P926783	-1.2	-1.2	-3.7	-1.3	ZNF880	Homo sapiens zinc finger protein 880 (ZNF880), mRNA [NM_001145434]	400713
A_32_P790361	1.0	-1.0	-2.9	1.0	ZNF90	Homo sapiens zinc finger protein 90 (ZNF90), mRNA [NM_007138]	7643
A_23_P209146	1.0	1.2	-2.7	-1.1	ZNF91	Homo sapiens zinc finger protein 91 (ZNF91), mRNA [NM_003430]	7644

A_24_P192209	2.4	-2.0	1.1	2.2	ZNF92	Homo sapiens zinc finger protein 92 (ZNF92), transcript variant 1, mRNA [NM_007139]	168374
A_23_P124585	1.0	-1.5	-3.6	-2.0	ZNF93	Homo sapiens zinc finger protein 93 (ZNF93), mRNA [NM_031218]	81931
A_24_P23034	2.1	1.1	2.9	2.2	ZNFX1	Homo sapiens zinc finger, NFX1-type containing 1 (ZNFX1), mRNA [NM_021035]	57169
A_32_P157385	2.1	2.1	2.6	3.5	ZNFX1-AS1	Homo sapiens cDNA FLJ42181 fis, clone THYMU2031368. [AK124175]	441951
A_23_P111843	1.0	2.1	-1.1	-4.3	ZNHIT1	Homo sapiens zinc finger, HIT-type containing 1 (ZNHIT1), mRNA [NM_006349]	10467

A_24_P193624	5.9	9.3	5.5	5.3	ZNRF1	Homo sapiens zinc and ring finger 1 (ZNRF1), mRNA [NM_032268]	84937
A_24_P16610	-2.6	-1.4	-1.7	-3.2	ZNRF1	Homo sapiens zinc and ring finger 1 (ZNRF1), mRNA [NM_032268]	84937
A_24_P262221	-1.9	2.9	-3.0	-2.4	ZNRF1	Homo sapiens zinc and ring finger 1 (ZNRF1), mRNA [NM_032268]	84937
A_23_P325017	-1.9	-3.2	-1.2	1.2	ZNRF2	Homo sapiens zinc and ring finger 2 (ZNRF2), mRNA [NM_147128]	223082
A_24_P162373	1.2	3.5	3.3	3.5	ZNRF3	Homo sapiens zinc and ring finger 3 (ZNRF3), transcript variant 1, mRNA [NM_001206998]	84133

A_23_P374559	-2.2	-3.3	-5.9	-7.3	ZNRF4	Homo sapiens zinc and ring finger 4 (ZNRF4), mRNA [NM_181710]	148066
A_23_P1912	-1.1	1.3	-3.0	-10.1	ZP1	Homo sapiens zona pellucida glycoprotein 1 (sperm receptor) (ZP1), mRNA [NM_207341]	22917
A_23_P89132	-1.5	-32.1	-1.5	-1.1	ZP2	Homo sapiens zona pellucida glycoprotein 2 (sperm receptor) (ZP2), mRNA [NM_003460]	7783
A_23_P85521	1.9	-1.8	2.0	2.1	ZRANB2	Homo sapiens zinc finger, RAN-binding domain containing 2 (ZRANB2), transcript variant 1, mRNA [NM_203350]	9406

A_23_P412059	-9.5	-1.0	-1.1	1.1	ZRANB3	zinc finger, RAN-binding domain containing 3 [Source:HGNC Symbol;Acc:25249] [ENST00000403017]	84083
A_24_P222997	1.7	1.3	-5.0	1.5	ZRANB3	Homo sapiens zinc finger, RAN-binding domain containing 3 (ZRANB3), mRNA [NM_032143]	84083
A_24_P32394	1.3	1.7	1.2	-19.5	ZRANB3	Homo sapiens zinc finger, RAN-binding domain containing 3 (ZRANB3), mRNA [NM_032143]	84083

A_23_P322395	2.8	3.2	5.1	2.0	ZSCAN1	Homo sapiens zinc finger and SCAN domain containing 1 (ZSCAN1), mRNA [NM_182572]	284312
A_23_P15135	1.6	3.2	-1.4	1.3	ZSCAN10	Homo sapiens zinc finger and SCAN domain containing 10 (ZSCAN10), mRNA [NM_032805]	84891
A_23_P323898	1.1	2.1	1.6	-1.5	ZSCAN12	Homo sapiens zinc finger and SCAN domain containing 12 (ZSCAN12), transcript variant 2, non-coding RNA [NR_028077]	9753

A_32_P360518	-2.1	5.2	-1.6	-1.6	ZSCAN12P1	Homo sapiens zinc finger and SCAN domain containing 12 pseudogene 1 (ZSCAN12P1), non-coding RNA [NR_024063]	221584
A_23_P259098	1.1	-5.2	1.1	-2.1	ZSCAN16	Homo sapiens zinc finger and SCAN domain containing 16 (ZSCAN16), mRNA [NM_025231]	80345
A_24_P920880	-1.1	1.1	-2.3	-2.1	ZSCAN18	Homo sapiens truncated zinc finger protein 447 isoform (ZNF447) mRNA, complete cds. [AY279352]	65982

A_24_P39919	1.7	-1.6	-1.6	-3.4	ZSCAN18	Homo sapiens zinc finger and SCAN domain containing 18 (ZSCAN18), transcript variant 3, mRNA [NM_023926]	65982
A_23_P417372	1.3	-1.8	1.1	-6.3	ZSCAN2	Homo sapiens zinc finger and SCAN domain containing 2 (ZSCAN2), transcript variant 2, mRNA [NM_017894]	54993
A_24_P194730	-2.0	-2.1	-2.1	-2.2	ZSCAN22	Homo sapiens zinc finger and SCAN domain containing 22 (ZSCAN22), mRNA [NM_181846]	342945

A_24_P942730	-2.1	1.0	1.0	1.0	ZSCAN29	Homo sapiens zinc finger and SCAN domain containing 29 (ZSCAN29), mRNA [NM_152455]	146050
A_23_P100092	1.3	1.0	-1.7	-2.4	ZSCAN29	Homo sapiens zinc finger and SCAN domain containing 29 (ZSCAN29), mRNA [NM_152455]	146050
A_23_P78429	-2.1	-2.2	-2.3	-2.4	ZSCAN30	Homo sapiens zinc finger and SCAN domain containing 30 (ZSCAN30), mRNA [NM_001166012]	100101467
A_23_P90233	-1.9	-2.9	-11.3	-1.8	ZSCAN5A	Homo sapiens zinc finger and SCAN domain containing 5A (ZSCAN5A), mRNA [NM_024303]	79149

A_23_P165984	-4.3	1.6	-1.1	-1.1	ZSWIM3	Homo sapiens zinc finger, SWIM-type containing 3 (ZSWIM3), transcript variant 1, mRNA [NM_080752]	140831
A_23_P383118	-6.3	1.1	1.2	1.1	ZSWIM5	Homo sapiens zinc finger, SWIM-type containing 5 (ZSWIM5), mRNA [NM_020883]	57643
A_32_P109522	12.1	12.0	17.2	13.3	ZUFSP	Homo sapiens zinc finger with UFM1-specific peptidase domain (ZUFSP), mRNA [NM_145062]	221302

A_24_P925940	1.0	1.0	-4.2	-1.4	ZXDC	Homo sapiens clone DNA108719 SERH2790 (UNQ2790) mRNA, complete cds. [AY358102]	79364
A_32_P132589	-1.0	-1.0	-1.1	-13.6	ZYG11A	Homo sapiens zyg-11 homolog A (C. elegans) (ZYG11A), mRNA [NM_001004339]	440590
A_23_P426140	9.5	9.7	8.6	8.9	ZYG11B	Homo sapiens zyg-11 homolog B (C. elegans) (ZYG11B), mRNA [NM_024646]	79699
A_23_P130107	3.6	2.2	1.3	2.7	ZZEF1	Homo sapiens cDNA FLJ10821 fis, clone NT2RP4001057. [AK001683]	23140

A_24_P282108	-1.1	3.2	-1.5	-2.2	ZZEF1	Homo sapiens zinc finger, ZZ-type with EF-hand domain 1 (ZZEF1), mRNA [NM_015113]	23140
